# Proteomic and morphologic characterization of ovine macrophage differentiation and polarization

**DOI:** 10.1038/s41598-025-30269-x

**Published:** 2025-12-01

**Authors:** A. Elkhamary, C. Gerner, A. Bileck, I. Gerner, F. Jenner

**Affiliations:** 1 Department for Companion Animals and Horses, Centre for Equine Health and Research, Veterm, Vetmeduni Vienna, Vienna, Austria; 2 Department for Surgery, Faculty of Veterinary Medicine, Damanhour University, Damanhour, Egypt; 3 Department of Livestock Tissue Metabolism, Institute of Animal Science, University of Hohenheim, Stuttgart, Germany; 4 Department of Analytical Chemistry, Faculty of Chemistry, University of Vienna, Vienna, Austria; 5 Austrian Cluster for Tissue Regeneration, Vienna, Austria

**Keywords:** Preclinical research, Animal disease models

## Abstract

Macrophages play crucial roles in inflammation and tissue homeostasis, exhibiting phenotypic and functional plasticity that enables them to initiate, sustain, or resolve inflammation. To support the evidence-based selection of biomedically relevant preclinical models, this study comprehensively characterized ovine macrophage differentiation and polarization by integrating morphological assessments with in-depth proteomic profiling of cellular lysates and secretomes. Monocytes isolated from peripheral blood were differentiated into macrophages using GM-CSF (GMØ) or M-CSF (MMØ), then polarized into M1 and M2 phenotypes. Mass spectrometry identified 4804 proteins in cell lysates and 901 in secretomes, including 42 CD antigens, enabling the establishment of CD marker profiles for monocytes and the distinct macrophage differentiation and polarization states. Proteomic analyses revealed significant upregulation of inflammatory markers in M1 and elevated tissue repair markers in M2 macrophages. Enrichment analysis confirmed activation of antimicrobial and matrix-degrading pathways in M1 macrophages, and regenerative, proteolysis-inhibiting functions in M2 macrophages. In conclusion, this study addresses the limitations of currently available immunological tools by providing an antibody-independent alternative for the classification of ovine macrophages, facilitating more precise phenotypic characterization and functional insight in ovine immunology research.

**Supplementary Information:**

The online version contains supplementary material available at 10.1038/s41598-025-30269-x.

## Introduction

Macrophages play essential roles across the continuum of inflammatory responses, from initiation to resolution, and are pivotal in modulating the outcomes of inflammation^1–10^. Their functional and phenotypic plasticity in response to environmental cues enables macrophages to either initiate, amplify and sustain inflammation or promote its resolution, tissue repair and homeostasis^1–19^.

While macrophages exhibit a continuum of phenotypes with broad expression profiles, ranging across the entire polarization spectrum, their functional states are often simplified into two extremes: the pro-inflammatory (M1) and the anti-inflammatory/pro-resolving (M2) phenotypes^12,14^. M1 macrophages are characterized by the secretion of pro-inflammatory cytokines, chemokines, and proteases and are able to start and sustain the inflammatory response. In contrast, M2 macrophages promote tissue repair, the resolution of inflammation and homeostasis, by secreting anti-inflammatory mediators, phagocytosing apoptotic bodies and promoting extracellular matrix deposition^12,14^. The dynamic interplay between these M1 and M2 states is essential for ensuring an appropriate balance between acute inflammation and its resolution. However, dysregulated macrophage polarization can result in maladaptive responses. An excessive M1 response can lead to tissue damage, chronic inflammatory diseases, neoplastic transformation, and altered glucose metabolism through insulin resistance, while an overactive M2 response can promote fibrosis, exacerbate allergic reactions, and support tumor progression^12,19^.

Understanding the molecular mechanisms that govern macrophage polarization and transitions between these states is vital for developing targeted therapeutic strategies to modulate macrophage behavior in pathological contexts. While conventional methods such as flow cytometry and immunofluorescence, have provided key insights, they often lack the resolution needed to fully capture the complexity of macrophage functions or subtle changes in protein expression that regulate their phenotypes. Although transcriptomic analyses are valuable for identifying polarization-specific surface markers and expression profiles, they do not always correlate with the actual protein abundance in macrophage secretomes due to post-transcriptional processing, mRNA stability, and post-translational modifications^20–25^. Considering that macrophage polarization can be distinguished by their secretion profiles and surface molecular markers, comprehensive proteomics, which directly measures protein abundance, offers a powerful approach to identify the molecular signatures associated with monocyte-to-macrophage differentiation and polarization. Additionally, given the established relationship between macrophage morphology and functional states^26–29^, morphologic analysis can serve as a complementary, cost-effective tool for assessing macrophage activation and polarization.

Sheep, due to their anatomical and physiological similarities to humans, including comparable immune organ structures and immune signaling pathways^30–41^, represent an excellent animal model for studying immune and inflammatory responses. However, ovine peripheral blood monocyte-derived macrophage subtypes have not been phenotypically and functionally characterized, due to a paucity of immunological tools and the limited availability of ovine-specific antibodies^32,38,42^. Therefore, this study aims to characterize macrophage differentiation and polarization by integrating detailed morphological analyses with comprehensive proteomic profiling of both cellular lysates and secreted proteins.

## Materials and methods

### Sample collection and ethics approval

The study was conducted with ethical approval from the institutional ethics and animal welfare committee of the University of Veterinary Medicine Vienna and the national authority, the Federal Ministry of Science and Research (license BMWF-68.205/0116-V/3b/2018). All procedures were performed in full compliance with the approved protocols and all relevant institutional and national guidelines and regulations. The experiments are reported in accordance with the ARRIVE guidelines.

A total of 100 mL of blood was aseptically collected from the jugular vein of four adult Merino ewes owned by the University of Veterinary Medicine Vienna (aged 3–4 years), confirmed to be healthy by physical examinations and complete blood count (CBC). Blood was drawn using a 23-gauge butterfly catheter and a heparinized 100-mL syringe (Gilvasan, 5000 IU/mL, 1 mL Heparin/10 mL blood), and immediately transported to the laboratory for further processing. All proteomic experiments and analyses were conducted using four biological replicates (from four ovine donors), each with two technical replicates, except for morphological analysis, which was performed with three biological replicates, each including three technical replicates for every experimental group.

### Ovine monocyte isolation

Peripheral blood mononuclear cells (PBMCs) were isolated by density gradient centrifugation using Lymphoprep^®^ (1.077 g/ml, STEMCELL Technologies, Germany), as previously described^43^. The isolated PBMCs (*n* = 4 biological replicates (4 ovine donors), 3 technical replicates/donor/experimental group) were resuspended at a concentration of 3 × 10^6 cells/mL in RPMI 1640 medium (Gibco, Life Technologies, Austria) supplemented with 10% fetal calf serum (FCS) (Gibco, Life Technologies, Austria), 1% Penicillin, 1% Streptomycin, and 1% Amphotericin (Sigma-Aldrich, Germany) (referred to as complete medium). Subsequently, 3 × 10^6 PBMCs/ml complete medium were plated in T-25 flasks for proteomic analysis, and in 6-well plates for morphological evaluations (2 ml/well), and incubated under standard conditions (37 °C, 5% CO2) for three hours to allow for adequate monocyte adherence^44,45^. Non-adherent cells (e.g., lymphocytes) and FCS-containing medium were then gently removed through two successive washes with PBS without calcium and magnesium (PBS−/−) (Gibco, Life Technologies, Austria). Monocyte (Mo) enrichment by plastic adherence was performed based on the distinct adhesion properties of monocytes^45–48^ and the lack of suitable ovine-specific antibodies for magnetic separation techniques^32,42^. The purity of the Mo population was assessed by mass spectrometry (MS)-based proteomic analysis showing the presence of specific monocyte markers (CD14^+^ CD16^+^) and the absence (below the limit of detection based on specific peptide counts) of lymphocyte-specific CD antigens (CD3^+^CD4^+^ T cells, CD3^+^CD8^+^ T cells, CD19^+^ CD20^+^B cells).

Thereafter (time point T0), the adherent cells were cultured for an additional 3 h in serum-free RPMI 1640 medium supplemented with 1% penicillin, streptomycin, and amphotericin (serum-free medium) and the conditioned medium was collected (at time point T3h) in a 15 mL Falcon tube, centrifuged at 2000×g for 10 min at 4 °C, and filtered through a 0.2 μm filter (Sigma-Aldrich, Germany) to remove residual cells and debris. The filtered secretome was precipitated with ice-cold 99.6% ethanol (AustrAlco, Austria) and stored at − 20 °C for subsequent proteomic analysis and isolation of secreted proteins.

The adherent cells in the T-25 flasks were washed 3 times with PBS−/−, and then 200 µl of Sodium deoxycholate lysis buffer (SDC) (4% sodium deoxycholate, 100 mM Tris HCl pH 8.5) (Sigma-Aldrich, Germany) was added. The cells were gently detached using a cell scraper and transferred to a 15 ml falcon tube. Subsequently, the cell suspension was heated to 95 °C for 5 min in a water bath to ensure complete cell lysis. The lysate was then stored at − 80 °C until further processing.

### Monocyte-derived macrophage differentiation

To generate monocyte-derived macrophages (MØ), adherent monocytes were cultured with complete culture medium without supplements (cMØ) or supplemented with 50 ng/mL of either recombinant human GM-CSF (Granulocyte-macrophage colony-stimulating factor, ImmunoTools GmbH, Germany) for (GMØ) differentiation or M-CSF (macrophage colony-stimulating factor, ImmunoTools GmbH, Germany) for (MMØ) differentiation. The culture was maintained under standard conditions (37 °C, 5% CO2) for a duration of seven days, with a medium change every 3 days.

Morphological evaluations were conducted every 48 h using phase-contrast microscopy to monitor the characteristic changes associated with macrophage differentiation, such as increased cell size, enhanced cytoplasmic granularity, and the emergence of pseudopodia. Upon completion of the seven-day differentiation period, the resultant MØ were evaluated both morphologically, using fluorescence microscopy, and phenotypically, by Mass spectrometry-based proteomics analysis of the secretome and whole-cell lysate and compared to Mo. For proteomic analysis, the cell washed twice with PBS−/− and the culture medium was replaced with serum-free RPMI 1640 medium supplemented with 1% penicillin, streptomycin, and amphotericin, and cells were incubated for 3 h at 37 °C in a humidified 5% CO₂ incubator. The secretome and whole-cell lysates were then collected as described in Sect. 2.2.

### Macrophage polarization

On day 6 of the differentiation process (T6d), the medium of GMØ was changed to complete culture medium supplemented with 1 ng/mL lipopolysaccharide (LPS, ImmunoTools GmbH, Germany) and 20 ng/mL interferon-gamma (IFN-γ, ImmunoTools GmbH, Germany) to induce M1 polarization. The medium of MMØ was changed to complete culture medium supplemented with 20 ng/mL interleukin-4 (IL-4, ImmunoTools GmbH, Germany) to induce M2 polarization. The LPS concentration (1 ng/mL) was chosen given the wide variability reported for M1 polarization (1 pg/mL–1 µg/mL)^49–54^, the cytotoxicity of higher doses^53,55^, evidence of effectiveness in human macrophages^54,56–61^, and confirmation in our ovine pilot studies where 1 ng LPS/ml induced the expression of classical M1 markers.

Twenty-four hours after stimulation (at time point T7d), the cell washed twice with PBS−/− and the culture medium was replaced with serum-free RPMI 1640 supplemented with 1% penicillin, streptomycin, and amphotericin, and cells were incubated for 3 h at 37 °C in a humidified 5% CO₂ incubator. The secretome and whole-cell lysates were then collected as described in Sect. 2.2. GMØ, MMØ, and cMØ served as baseline to which the effects of polarization stimuli on macrophage function and protein expression were compared.

### Morphologic phenotypic characterization of different cell types

To assess the morphological characteristics of different cell types, PBMCs were cultured in 6-well plates as described in Sect. 2.2 to 2.4 to derive monocytes, cMØ, GMØ, MMØ, M1 and M2 macrophages. Following the defined culture period at the specific conditions for each cell type, cells were fixed with 4% paraformaldehyde (Sigma-Aldrich, Germany) for 10 min, washed twice with PBS−/− (Gibco, Life Technologies, Austria) (5 min per wash) and then permeabilized by 0.2% Triton-X100 (Sigma-Aldrich, Germany) for 20 min at room temperature. After 2 washes with PBS−/−, non-specific binding was blocked with 5% goat serum (Sigma-Aldrich, Germany) for 30 min. This was followed by 2 washes with PBS−/− and F-actin staining of the cytoskeleton with 5 µg/ml Alexa Fluor^®^ 488 Phalloidin (Cell Signaling Technology, USA) in 1% goat serum and 0.1% sodium azide (Sigma-Aldrich, Germany) for 30 min. Cells were then washed 3 times with PBS, stained with 250 ng/ml DAPI (4′,6-Diamidino-2-Phenylindole) (Invitrogen) in PBS for 5 min, washed 3 times with PBS, then covered with anti-fade medium (Sigma-Aldrich, Germany, and finally imaged using a florescent microscope (EVOS FL Auto imaging system, Life Technologies, Thermo Fisher, USA).

#### Image analysis for phenotype identification and classification

Using Fiji software (version 2.14), images of F-actin and DAPI staining were merged. Noise reduction was achieved by applying the Anisotropic Diffuse algorithm, a class of filters designed to minimize noise while preserving sharp edges^62,63^. Then, background subtraction was performed, and the images were separated into two distinct channels. The Huang thresholding method was employed to identify the regions of interest (ROI)^64,65^. Subsequently, the primary detection of the cell nucleus (DAPI channel) was optimized, followed by the secondary detection of the cell body (F-actin channel). For each of the six conditions, 63 single cells were analyzed, distributed across three independent biological replicates. For each cell, two ROIs—nucleus and cytoplasm—were segmented, yielding 126 ROIs per condition (63 nuclear, 63 cytoplasmic) and 756 ROIs in total from 108 images. Each ROI underwent morphometric and intensity analysis comprising 36 features, including area, perimeter, Feret’s diameter, circularity, mean gray value, integrated density, and corresponding cytoplasm-to-nucleus ratios of these parameters.

### Statistical analysis

Statistical analyses were conducted utilizing GraphPad Prism software (version 8.4.3). Continuous variables were reported as mean ± standard deviation (SD), and categorical variables were expressed in percentages. After verifying the normal distribution of the data using the Shapiro–Wilk test^66^, parametric tests were employed for the analyses. Morphological differences and differences in CD marker expression across macrophage subtypes were analyzed using ANOVA with Tukey’s correction for multiple comparisons. To account for the hierarchical data structure and to avoid pseudo-replication, analyses of single-cell morphological features were conducted at the biological-replicate level using a nested one-way ANOVA (replicates nested within conditions, with cells treated as observational units within replicates).

### Shotgun proteomics by LC–MS/MS

For detailed phenotypic and functional profiling, each macrophage subtype underwent quantitative LC-MS/MS analysis of both cell lysates and secretomes.

#### Cell viability assessment and secretome integrity validation

Cell viability was determined using the Trypan Blue exclusion assay (A13262, Invitrogen, Thermo Fisher Scientific, USA) and quantified with a Countess^®^ II FL Automated Cell Counter (Life Technologies, USA)^67^. Viability was defined as the proportion of live cells relative to the total cell count, with a minimum threshold of 95% required for both monocytes and their differentiated macrophage subtypes to ensure suitability for downstream applications. Since experimental samples were immediately processed for lysate and secretome collection (Sect. 2.2), viability was assessed in parallel cultures maintained under identical conditions.

To evaluate secretome integrity and monitor potential contamination from intracellular leakage, representative intracellular proteins—TUBA1B (cytoskeletal), CANX and CKAP4 (ER-associated), VDAC2 (mitochondrial), and ATP1A1 and SLC3A2 (plasma membrane)—were quantified by mass spectrometry–based ratiometric analysis^68,69^. Secretome-to-lysate (S/L) ratios were calculated from label-free quantification values (S/L = mean LFQ intensity in conditioned medium ÷ mean LFQ intensity in lysate), with ratios threshold < 0.05 indicating minimal contamination.

For downstream proteomic analysis, only preparations with cell viability greater than 95% and S/L ratios of intracellular marker proteins below 0.05 were included, ensuring functional cell activity and secretome integrity at the time of collection.

#### Sample Preparation

Proteomic samples were prepared and processed as described previously^43^. Cell pellets were thawed and further lysed using the S220 Focused-ultrasonicator (Covaris, LLC., Woburn, MA, USA). Precipitated secretome proteins were centrifuged at 5000×*g* for 30 min at 4 °C and the resulting protein pellet was solubilized in SDC buffer. Protein concentrations were determined via bicinchoninic acid assay (BCA)-assay. All samples yielded more than 20 mg of protein, indicating that the number of cells collected per condition was sufficient to achieve meaningful and comparable results across replicates.

Protein samples (20 µg/sample) were topped-up to 90 µL with SDC lysis buffer. Subsequently, reduction/alkylation buffer (10 µL) was added and incubated on a thermoshaker (1400 rpm, 45 °C). The reduction/alkylation buffer contained TCEP (200 mM) and 2-chloracetamide (800 µM) and the pH was adjusted to 7.5–8 using NaOH (5 M). After reaching RT, trypsin/Lys-C (1 µL, 0.2 µg∙µL-1, enzyme-to-substrate ratio of 1:100) was added to each sample and incubated for 17 h on a thermoshaker (1400 rpm, 30 °C). Then, the samples were nearly dried in a vacuum concentrator (40 min, 40 °C). The samples were desalted using SDB-RPS StageTips. The StageTips were prepared by stacking two disks of a polystyrenedivinylbenzene-reversed phase sulfonate material (Empore 2241 SDB-RPS, 12 μm particle size, 47 mm; CDS Analytical LLC) into a pipet tip. A 100 µL volume of SDB-RPS loading buffer (99% IPA, 1% TFA) was added to each sample and was quantitatively transferred to the corresponding StageTip. The tips were then centrifuged (1500 g, 8 min) to allow the whole solution to pass through the RPS material. Then, loading buffer/wash buffer 1 (100 µL, 99% IPA, 1% TFA) and SDB-RPS wash buffer 2 (100 µL, 94.8% water, 5% ACN, 0.2% TFA) were sequentially added and centrifuged. The waste tube was then exchanged with a clean storage tube containing a glass LC-MS inlet. The peptides were directly eluted into the inlet using SDB-RPS elution buffer (60 µL, 39.8% water, 59.7% ACN, 0.5% NH4OH) followed by centrifugation (1500 g, 5 min). The samples were finally dried in the vacuum concentrator (40 °C) and stored at − 20 °C until analysis.

#### Liquid chromatography–mass spectrometry

Liquid chromatography–mass spectrometry was performed employing a timsTOF Pro mass spectrometer (Bruker Daltonics, Bremen, Germany) hyphenated with a Dionex UltiMateTM 3000 RSLCnano system (Thermo Scientific, Bremen, Germany). Samples were analyzed in data-dependent acquisition mode. The injection volume was 2 µl in case of cell lysates and 5 µl in case of secretomes. Samples were loaded on an AcclaimTMPepMapTM C18 HPLC pre-column (2 cm × 100 μm, 100 Å, Thermo Fisher Scientific™, Vienna, Austria) at a flow rate of 10 µl min-1 MS loading buffer. After trapping, peptides were eluted at a flow rate of 300 nl min-1 and separated on an Aurora series CSI UHPLC emitter column (25 cm × 75 μm, 1.6 μm C18, Ionopticks, Fitzroy, Australia) applying a gradient of 8–40% mobile phase B (79.9% ACN, 20% H2O, 0.1% FA) in mobile phase A (99.9% H2O, 0.1% FA) over 85 min.

#### Proteomics data analyses

Protein identification was performed via MaxQuant^70^ (version 1.6.17.0) employing the Andromeda search engine against the UniProt Ovis aries Database^71^ (version 11/2021, 20′ 375 entries). A minimum of two peptides identified per protein, a maximum of two missed cleavages per peptide, a mass tolerance of 20 ppm for MS spectra and 40 ppm for MS/MS spectra, and a false discovery rate of 0.01 at peptide and protein level were applied. Match-between-runs were enabled with a matching time window of 0.7 min and an alignment time window of 20 min. Oxidation of methionine and N-terminal protein acetylation were set as variable modifications. Carbamidomethylation of cysteine was set as fixed modification. For comparative proteome analysis, normalized label-free quantification (LFQ) intensities were derived using the MaxLFQ algorithm implemented in the MaxQuant software.

Protein tables generated by MaxQuant were imported into Perseus (version 1.6.14.0)^72^ for downstream analysis. Proteins identified only by site, common contaminants, and reverse-sequences matches were filtered out. To ensure data quality, only proteins identified in at least 70% of a sample group were further processed. LFQ-values of remaining entries were log2-transformed. Entries with missing values were then imputed with values from a normal distribution (downshift: 1.8, width: 0.3). Protein annotation was performed using UniProt ID mapping with the Ovis aries reference database to assign predicted secretion status and subcellular localization. Proteins without ID mapping were classified according to their human orthologs.

### Bioinformatic and statistical analyses of proteomics data

#### Differentially abundant proteins

Variations in protein abundance across macrophage subtypes were assessed using a two-sided Student’s t-test for pairwise group comparisons and one-way ANOVA with Tukey’s HSD post-hoc tests for multi-group comparisons. Multiple testing was controlled using a permutation-based false discovery rate (FDR) with 250 randomizations. Proteins achieving an FDR of ≤ 0.05, which served as the q-value threshold, and demonstrating a fold change (FC) of |≥2|, were deemed statistically significant and classified as differentially abundant proteins (DAPs).

#### Functional and pathway enrichment analysis

EnrichmentMap analysis was performed to visualize protein-set enrichment across macrophage subtypes. Briefly, DAPs identified from both cellular lysates and secretomes for each subtype were integrated into a unified, non-redundant dataset by mapping protein identifiers to UniProt accession numbers and consolidating duplicate entries—proteins detected in both compartments—to ensure unique representation. The merged dataset was subjected to functional enrichment analysis using the StringApp (STRING database) within Cytoscape (version 3.10.3)^73^, targeting Gene Ontology (GO) biological processes (BP) and canonical pathways from the KEGG and Reactome databases^74–78^. A false discovery rate (FDR) threshold of < 0.05 was applied to identify statistically significant enrichment terms. Enriched GO terms were then visualized using the EnrichmentMap plugin^79^. An enrichment network was constructed based on Jaccard coefficient (cutoff 0.4), with nodes representing GO terms, edges representing protein overlap, node size indicating protein count, and node color representing enrichment p-value (node cutoff q-value 0.05, edge cutoff 0.4). Clusters within the network were automatically annotated using the AutoAnnotate plugin^80^, which utilizes ClusterMaker2 for cluster identification and WordCloud for visual summaries. Protein-sets were then organized into a weighted similarity network where nodes represent protein-sets (e.g. a GO term) and edges denote protein overlap (e.g. GO cross-talk), enhancing interpretability of the network and facilitating detailed analysis of biological processes and pathways.

#### Protein-protein-interaction network construction and module analysis

Potential M1 and M2 macrophage marker proteins were first identified using Venn diagram analysis to determine subtype-specific proteins. Protein-protein interaction (PPI) networks were then constructed using STRING (experimentally validated interactions, score ≥ 0.4) and visualized in Cytoscape^81^. Network topology was analyzed with the CytoHubba plugin using five algorithms: Degree, Maximal Clique Centrality (MCC), Edge Percolated Component (EPC), Closeness, and Radiality. For each subtype, the top 30% of proteins ranked by each algorithm were extracted. To enhance robustness and minimize algorithm-specific bias, only proteins consistently present in the intersection across all five algorithms were retained as hub proteins, in line with multi-algorithm consensus strategies for PPI network analysis^81–84^. These consensus hub proteins were considered putative biomarkers of the respective macrophage subtypes.

## Results

### Ovine monocyte isolation

**Fig. 1:**
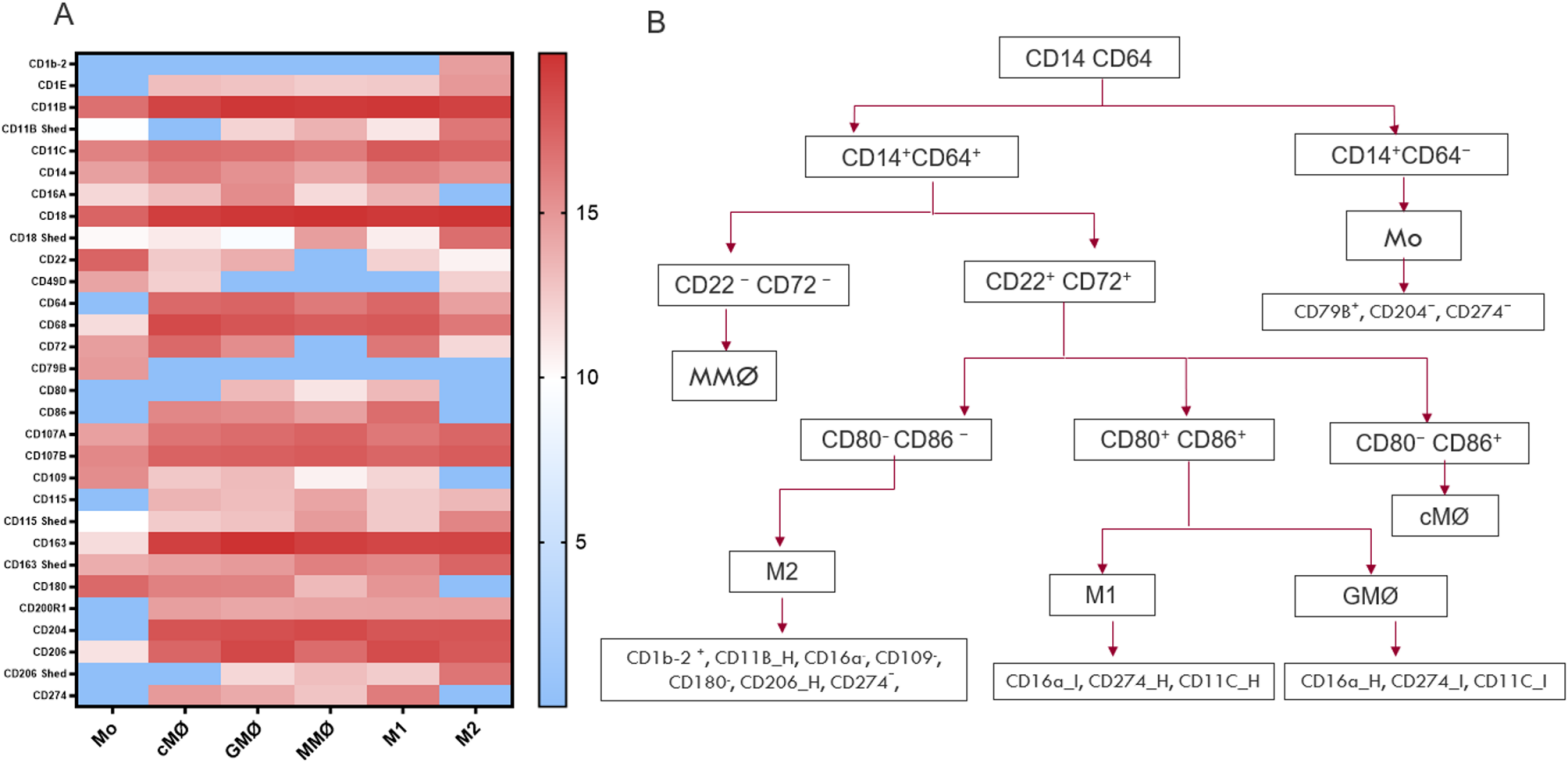
(**A**) Heatmap showing proteomic abundance patterns of CD marker panels across the monocyte–macrophage spectrum. The heatmap values represent Log₂ normalized LFQ intensities, as indicated by the color bar scale. Red indicates higher relative protein abundance, blue indicates lower abundance, and white represents intermediate levels. “Shed” markers (e.g., CD11B Shed) denote proteins detected in the secretome, whereas standard counterparts (e.g., CD11B, CD18) were measured in cell lysates. (**B**) Classification strategy of monocyte and macrophage subtypes based on abundance profiling of distinct CD markers. Marker notation is as follows: “+” = expressed, “−” = absent (below the limit of detection), “H” = high abundance, “I” = intermediate abundance. Mo, Monocytes at 3 h; cMØ, monocyte-derived macrophages differentiated without the addition of exogenous growth factors; GMØ, monocyte-derived macrophages differentiated with GM-CSF (Granulocyte-Macrophage Colony-Stimulating Factor); MMØ, monocyte-derived macrophages differentiated with M-CSF (Macrophage Colony-Stimulating Factor); M1, pro-inflammatory macrophages activated with GM-CSF/ LPS/ INF-γ; M2, anti-inflammatory macrophages activated with M-CSF/ IL-4.

Mass spectrometry-based proteomic analysis identified 42 CD surface proteins and confirmed successful monocyte isolation. Monocyte-specific markers were consistently detected (CD14: average normalized LFQ intensity 14.52 ± 0.39; CD16A: 11.82 ± 0.88, *n* = 4), whereas lymphocyte-specific markers (CD3/CD4 and CD3/CD8 for T cells, CD19/CD20 for B cells) were consistently below the limit of detection (Fig. 1). Additionally, proteomic analysis identified nine key CD markers that uniquely characterize monocytes and facilitate their differentiation from other macrophage phenotypes (Table 1, Suppl. Table 1). Among these, eight CD markers, including CD64 and CD68, were either lacking (below the limit of detection) or expressed at low levels in monocytes, but were significantly upregulated during their differentiation and polarization into different macrophage phenotypes. In contrast, CD79B was highly expressed in monocytes and subsequently showed significant lack of expression (below the limit of detection) in other macrophage subtypes (Table 1; Fig. 1).

**Table 1:** Comparative analysis of differentially abundant cluster of differentiation (CD) markers across monocyte and macrophage subtypes (adjusted p-value ≤ 0.05).

CD antigen	Protein ID	Name	Mo	cMØ	GMØ	MMØ	M1	M2
CD1b-2	Q29422	T-cell surface glycoprotein CD1b-2						U/H
CD1E	W5PBM2	T-cell surface glycoprotein CD1e, membrane-associated	U/ -					
CD11B	W5PGV0	Integrin alpha-M (ITGAM)						U/H
CD16A	W5PK31	Low affinity immunoglobulin gamma Fc region receptor III-A ( FCGR3A)			D/H			U/ -
CD22	W5P3Y9	B-cell receptor CD22				U/ -		
CD64	W5QHL1	High affinity immunoglobulin gamma Fc receptor I ( FCGR1A)	U/ -	S/H	S/H	S/H	S/H	S/H
CD68	W5PZB2	Macrosialin	U/ L	S/H	S/H	S/H	S/H	S/H
CD72	W5PQU2	B-cell differentiation antigen CD72				U/ -		
CD79B	W5PX57	B-cell antigen receptor complex-associated protein beta chain	U/ H	U/ -	U/ -	U/ -	U/ -	U/ -
CD109	W5P8E9	CD109 antigen						U/ -
CD115	W5P8R4	Macrophage colony-stimulating factor 1 receptor (CSF1R)	U/ -					
CD163	W5NY01	Scavenger receptor cysteine-rich type 1 protein M130	U/ L					
CD180	W5P7C7	CD180 antigen						U/ -
CD200R1	W5QDJ5	Cell surface glycoprotein CD200 receptor 1	U/ -					
CD204	W5PIQ6	Macrophage scavenger receptor types I and II (MSR1)	U/ -					
CD206	W5PRI6	Macrophage mannose receptor 1 (MRC1)	U/ L					U/H
CD274	W5PVJ5	Programmed cell death 1 ligand 1 ( PD1L1)	U/ -				U/H	U/ -

Unique markers (U) are defined as CD markers exhibiting statistically significant differences in abundance in one macrophage subtype compared with all other subtypes (adjusted p-value ≤ 0.05). Distinctive markers (D) are defined as CD markers that show statistically significant differences in abundance relative to the majority of subtypes, with the exception of a single subtype, thereby indicating subtype-preferential but not exclusive abundance. Shared markers (D/S) represent CD markers with no statistically significant differences in abundance across subtypes (adjusted p-value > 0.05). CD, Cluster of Differentiation, U/L, uniquely low abundance; U/H, uniquely high abundance; U/-, uniquely absent abundance (below the limit of detection); S/H, shared high abundance; D/H, distinctive high abundance. Mo, Monocytes at 3 h; cMØ, monocyte-derived macrophages differentiated without the addition of exogenous growth factors; GMØ, monocyte-derived macrophages differentiated with GM-CSF (Granulocyte-Macrophage Colony-Stimulating Factor); MMØ, monocyte-derived macrophages differentiated with M-CSF (Macrophage Colony-Stimulating Factor); M1, pro-inflammatory macrophages activated with GM-CSF/ LPS/ INF-γ; M2, anti-inflammatory macrophages activated with M-CSF/ IL-4.

**Fig. 2:**
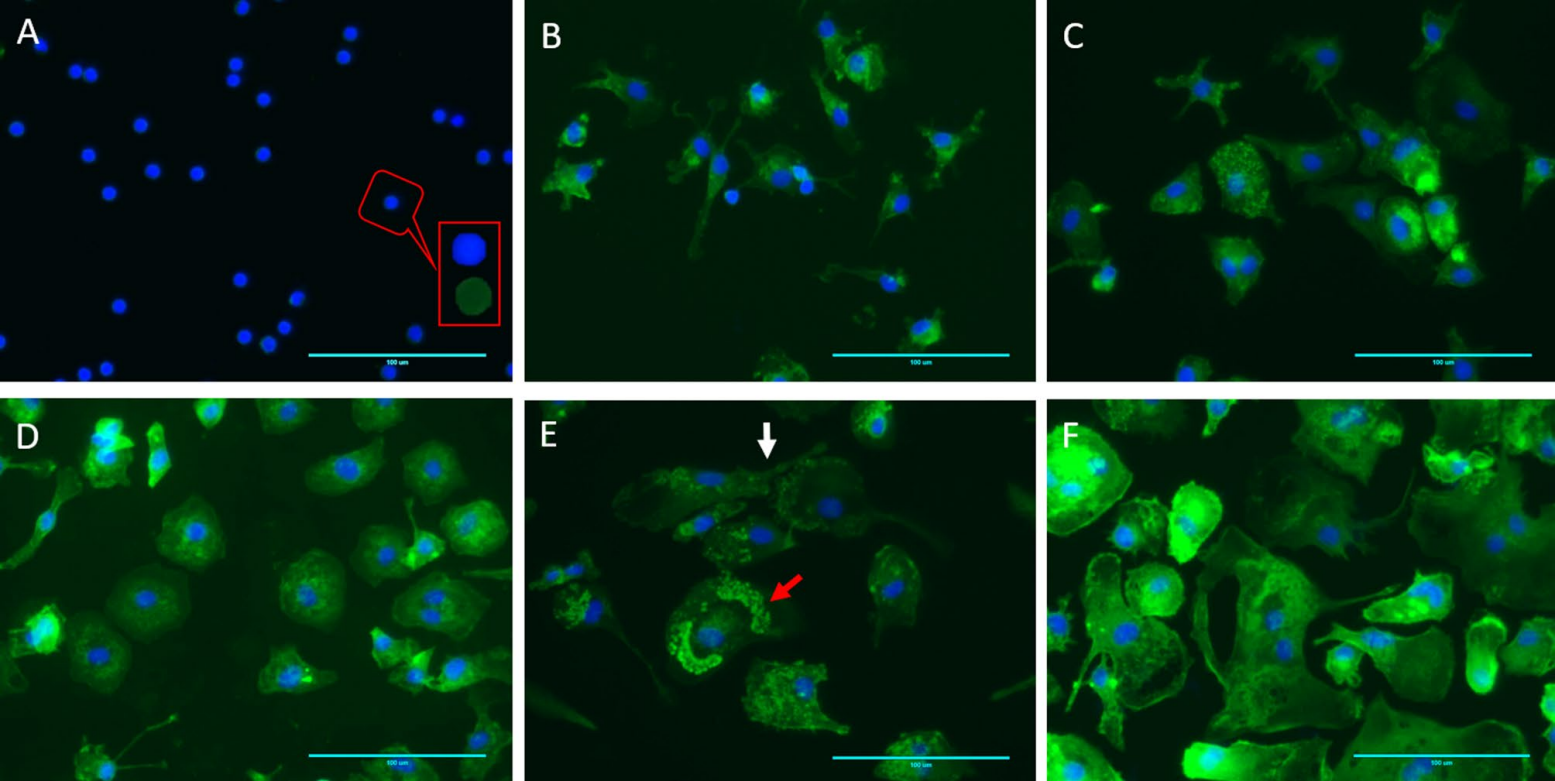
Morphological differentiation of monocytes and macrophage subtypes visualized via immunofluorescent staining. F-actin and nuclei were stained with Phalloidin Alexa Fluor 488 (green) and DAPI (blue), respectively, showcasing (**A**) Mo; monocytes at 3 h with small area, rounded morphology, and high nuclear-to-cytoplasmic ratio, showing a less prominent F-actin signal mainly localized as a cortical actin ring. Inset: one zoomed-in cell with F-actin and nucleus in separate channels, highlighting this feature, (**B**) cMØ; monocyte-derived macrophages differentiated without the addition of exogenous growth factors, (**C**), GMØ; monocyte-derived macrophages differentiated with GM-CSF (**D**) MMØ; monocyte-derived macrophages differentiated with M-CSF, (**E**) M1; pro-inflammatory macrophages activated with GM-CSF/ LPS/ INF-γ and (**F**) M2; anti-inflammatory macrophages activated with M-CSF/ IL-4. W The white arrow indicates filopodia, which are finger-like membrane protrusions, and the red arrow indicates podosomes, which are actin-rich adhesive structures. Scale bar = 100 μm.

Imaging-based morphological analysis also identified characteristic features of monocytes in their quiescent state in comparison to other macrophage subtypes (Fig. 2A, Suppl. Table S2, Suppl. Figure S1). Monocytes demonstrated a minimal cellular area and a highly circular form, indicative of an almost perfect spherical shape. The nucleus was notably large, comprising a significant proportion of the cell, resulting in a high nuclear-to-cytoplasmic ratio. The cytoplasm showed slight granularity, highlighted by a minimal Integrated Density value, suggesting subdued immune functionalities. The cell membranes were smooth, with the lowest perimeter measurements, indicating an absence of pseudopodial extensions (Fig. 2A, Suppl. Table 2, Suppl. Figure 1). This configuration aligns with their non-migratory and non-phagocytic behavior in the resting state.

### Macrophage differentiation

#### Morphologic characterization of macrophage differentiation

cMØ, GMØ, and MMØ macrophages exhibited significant morphological changes compared to their monocyte precursors (Fig. 2B and D, Suppl. Table 2, Suppl. Figure 1). These changes included a marked increase in cell size and perimeter, along with significant decreases in circularity and roundness, and increases in cell Feret’s diameter, and nuclear elongation (*p* ≤ 0.05). Concurrently, there was a notable reduction in the nuclear-to-cytoplasmic ratio due to cytoplasmic expansion, accompanied by an increase in cytoplasmic granularity, as indicated by a marked increase in integrated density measurements (Suppl. Table 2, Suppl. Figure 1). Macrophage cell membranes also developed extensive ruffles and pseudopodia (Fig. 2b and D, Suppl. Figure 1).

In the comparative analysis of shape descriptors across cMØ, GMØ, and MMØ cells, no significant differences were observed in overall shape descriptors. However, disparities were observed in specific cytoplasmic and nuclear characteristics (Suppl. Table 2, Suppl. Figure 1). Notably, MMØ cells exhibited statistically significant variances in cytoplasmic mean gray values when compared to GMØ (*p* = 0.002) and displayed a non-significant trend when compared to cMØ (*p* = 0.08). Additionally, MMØ cells showed significantly higher maximum cytoplasmic gray values compared to GMØ (*p* = 0.0001) and cMØ (*p* = 0.002). In terms of nuclear features, cells had significantly higher mean gray values compared to GMØ (*p* = 0.0002) and cMØ (*p* = 0.009), along with greater integrated nuclear density relative to both GMØ (*p* = 0.006) and cMØ (*p* = 0.008). Furthermore, nuclear circularity was the only feature where cMØ differed significantly when compared to both GMØ and MMØ (*p* = 0.01), underscoring distinct nuclear morphologies characteristic of these macrophage subtypes (Suppl. Table 2, Suppl. Figure 1).

#### Proteomic phenotypic characterization of macrophage differentiation: CD marker expression

To confirm the viability and functional activity of the cells at the time of harvest, both viability and contamination assessments were conducted. Trypan Blue exclusion assays revealed that all samples met the minimum viability threshold of 95%, with an average viability of 96.14 ± 0.82%. In parallel, mass spectrometry-based ratiometric analysis was used to monitor the presence of representative intracellular proteins in the secretome—specifically TUBA1B (cytoskeletal), CANX and CKAP4 (ER-associated), VDAC2 (mitochondrial), and ATP1A1 and SLC3A2 (plasma membrane). These markers exhibited Secretome-to-Lysate (S/L) intensity ratios ≤ 0.00005, indicating minimal contamination from intracellular leakage and confirming the high integrity of the secretome preparations (Suppl. Table 3).

Compared to monocytes, cMØ, GMØ, and MMØ (MØ) macrophages displayed significant variations in CD marker profiles, with 18, 18, and 23 significant differences, respectively (Suppl. Table 1). These macrophage subsets (MØ) demonstrated significantly higher abundance of CD68, CD64, CD86, CD1E, CD200R1, CD163, CD274, and CD206 (Suppl. Table 1). In addition, MØ uniquely lacked expression of CD79B proteins (below the limit of detection), which were positively expressed in monocytes (Table 1; Fig. 1).

Within seven days of in vitro culture, monocytes spontaneously differentiated into cMØ macrophages without the addition of exogenous growth factors such as M-CSF or GM-CSF. cMØ macrophages exhibited specific expression profiles, differing in 18 markers from Mo, 7 from GMØ, 12 from MMØ, 4 from M1, and 13 from M2 (*p* ≤ 0.05; Suppl. Table 1). Notably, cMØ displayed upregulation of pan-macrophage differentiation markers CD68 (Table 1), alongside an inverse expression pattern of CD80 and CD49D compared to GMØ and MMØ, characterized by the absence of CD80 (below the limit of detection) and the exclusive expression of CD49D (Fig. 1, Suppl. Table 1).

Furthermore, GMØ macrophages demonstrated distinct expression differences relative to other macrophage subtypes—specifically, 7 markers differed from MMØ, 7 from cMØ, 3 from M1, and 15 from M2 (*p* ≤ 0.05; Suppl. Table 1). Notably, CD16A emerged as a distinctive marker for GMØ, showing significantly higher expression compared to monocytes, M1, and M2 (*p* ≤ 0.05) and a trend toward higher expression relative to MMØ (*p* = 0.06; mean difference = 3.8; Table 1; Fig. 1).

Similarly, MMØ macrophages displayed clear differences in CD marker expression compared to other subtypes—7 markers distinct from GMØ, 12 from cMØ, 9 from M1, and 19 from M2 (*p* ≤ 0.05)– resulting in two unique lacking CD markers for MMØ, CD22 and CD72 (below the limit of detection) (Table 1; Fig. 1).

#### Proteomic profiling and functional characterization of macrophage differentiation

Mass spectrometry-based proteomic analysis successfully identified 4804 proteins in the whole cell lysates and 901 proteins in the secretomes of various macrophage subtypes. The comparison of monocytes with cMØ identified a total of 826 DAPs (670 in cell lysate, 133 in secretome, 23 in both) (Table 2, Suppl. Table 4, Suppl. Figure 2 A), with GMØ 1270 DAPs (862 in cell lysate, 338 in secretome, 70 in both) (Table 2, Suppl. Table 5, Suppl. Figure 2B), and with MMØ 1056 DAPs (827 in cell lysate, 133 in secretome, 96 in both) (Table 2, Suppl. Table 6, Suppl. Figure 2 C).

**Table 2:** Comparative analysis of differentially abundant proteins (DAPs) across monocyte and macrophage subtypes: expression levels and cellular compartmentalization.

Cell types	Cell compartment	Expression	Mo	cMØ	GMØ	MMØ	M1	M2
Mo	Cell lysate	Up		139	374	328	119	372
		Down		554	558	595	682	667
	Secretome	Up		138	375	132	125	99
		Down		18	33	97	44	135
cMØ	Cell lysate	Up	554		49	250	58	345
		Down	139		119	577	172	812
	Secretome	Up	18		21	4	6	4
		Down	138		56	383	69	498
GMØ	Cell lysate	Up	558	119		211	60	373
		Down	374	49		210	65	513
	Secretome	Up	33	56		8	4	3
		Down	375	21		268	27	466
MMØ	Cell lysate	Up	595	577	210		251	55
		Down	328	250	211		248	90
	Secretome	Up	97	383	268		333	4
		Down	132	4	8		21	58
M1	Cell lysate	Up	493	172	65	248		364
		Down	342	58	60	251		476
	Secretome	Up	36	69	27	21		19
		Down	308	6	4	333		443
M2	Cell lysate	Up	667	812	513	90	476	
		Down	372	345	373	55	364	
	Secretome	Up	135	498	466	58	443	
		Down	99	4	3	4	19	

Up, indicates significantly upregulated proteins; Down, indicates significantly downregulated proteins; Mo, Monocytes at 3 h; cMØ, monocyte-derived macrophages differentiated without the addition of exogenous growth factors; GMØ, monocyte-derived macrophages differentiated with GM-CSF (Granulocyte-Macrophage Colony-Stimulating Factor); MMØ, monocyte-derived macrophages differentiated with M-CSF (Macrophage Colony-Stimulating Factor); M1, pro-inflammatory macrophages activated with GM-CSF/ LPS/ INF-γ; M2, anti-inflammatory macrophages activated with M-CSF/ IL-4. Differentially abundant proteins (DAPs) were identified using a threshold of false discovery rate (FDR, q-value) ≤ 0.05 and absolute fold change ≥ 2.

MMØ and GMØ shared 41.9% (687 proteins) of DAPs relative to monocytes. In contrast, 35.6% (583 proteins) DAPs relative to monocytes were exclusive to GMØ, and 22.5% (369 proteins) were unique to MMØ (Suppl. Figure 3A, Suppl. Tables 7,8). A comparison of protein abundance between MMØ and GMØ macrophages, yielded 684 DAPs (408 in whole cell lysate, 263 in the secretome, and 13 in both) (Table 2, Suppl. Table 9, Suppl. Figure 3B). Comparative analysis of DAPs identified three proteins that uniquely differentiate GMØ from Mo, cMØ, MMØ, M1, and M2 and three proteins that exclusively differentiate MMØ relative to Mo, cMØ, GMØ, M1, and M2. Specifically, GMØ-exclusive proteins were DSN1, RFC1, and HSPG2, while MMØ-exclusive proteins were TGFB1, EMILIN2, and CHURC1 (Suppl. Figure 4 A-B, Suppl. Table 10).

The proteome of cMØ and GMØ macrophages aligns more closely with the M1 proteome, whereas MMØ macrophages demonstrated greater alignment with the M2 proteome. Specifically, 49% of DAPs in cMØ (578 DAPs) were linked to M1 macrophages, in contrast to 29% that aligned with M2 macrophages (449 DAPs). For GMØ, 41.5% of DAPs (646 DAPs) corresponded to M1, while 35.1% (634 DAPs) were associated with M2. Conversely, MMØ showed a pronounced overlap with the M2 proteome, with 57.5% of proteins (824 DAPs) linked to M2 and 37.4% (541 DAPs) to M1(Suppl. Figure 5 A-C).

Comparative functional enrichment analysis of GMØ and MMØ relative to monocytes revealed a substantial overlap in biological processes, with 54.1% (340) of enriched BP terms shared between the two macrophage subtypes (FDR < 0.05). However, GMØ exhibited distinct enrichment in 26.2% (165) of BP terms, while MMØ displayed unique enrichment in 19.7% (124) of BP terms (Suppl. Table 11).

Enrichment map analysis of DAPS in cMØ relative to monocytes revealed significant enrichment in 340 biological processes terms, systematically categorized into 22 distinct functional clusters. The most prominent clusters included receptor intracellular endocytosis, regulation of response to stimulus, and cell adhesion leukocyte (Suppl. Table 12). Similarly, enrichment map analysis of DAPS of GMØ relative to monocytes demonstrated significant enrichment in 505 biological processes terms, organized into 32 distinct functional clusters. The most notable clusters included chemical stimulus signal, hydrolase activity catalytic, dependent endocytosis receptor, and regulation leukocyte proliferation (Suppl. Table 13). Conversely, enrichment map analysis of DAPs of MMØ relative to monocytes revealed significant enrichment in 464 biological processes terms, organized into 32 functional clusters. Key enriched clusters encompassed processes associated with stress communication stimulus, cellular component biogenesis, endocytosis localization establishment, positive regulation activity, and monovalent cation homeostasis (Suppl. Table 14).

Pathway enrichment analysis conducted on DAPs of cMØ compared to monocytes revealed 13 key clusters, highlighting significant pathways such as glycosphingolipid biosynthesis series, LDL clearance signaling, and phosphatidylinositol signaling system (Suppl. Table 12). For GMØ relative to monocytes, the analysis identified 17 key clusters related to cellular responses stress, long term potentiation, and glycolysis gluconeogenesis glucose (Suppl. Table 13). The analysis for DAPs of MMØ relative to monocytes revealed 16 distinct clusters. Key among these were pathways involved in pyrimidine nucleotides salvage, fc mediated phagocytosis, globo isoglobo series, and integrin cell surface (Suppl. Table 14).

### Macrophage polarization

#### Morphologic characterization of macrophage polarization

During polarization, M1 and M2 macrophages exhibited marked morphological changes that distinguished them from their progenitor states across several shape descriptors, such as area, perimeter, cytoplasmic Feret’s diameter, roundness, and integrated density (*P* ≤ 0.05) (Fig. 2E-F, Suppl. Table 2, Suppl. Figure 1). M1 macrophages particularly demonstrated significant elongation and irregularity compared to GMØ, characterized by increased perimeter and cytoplasmic Feret’s diameter, alongside a decrease in circularity (*p* ≤ 0.05, Suppl. Table 2, Suppl. Figure 1). These cells also showed a notable increase in aspect ratio, inversely related to roundness, especially when compared to M2 macrophages. The cytoplasm of M1 macrophages became denser with a tightly packed, dotted actin texture, indicated by an increase in integrated density, although it remained lower than that observed in M2 macrophages (Fig. 2E-F). The nuclei of M1 macrophages tended to elongate, evidenced by increases in nuclear Feret’s diameter and decreases in nuclear circularity. Additionally, M1 macrophages developed extensive pseudopodia and membrane ruffles, further supported by significant changes in perimeter and aspect ratio (Fig. 2E, Suppl. Table 2, Suppl. Figure 1).

M2 macrophages typically presented a wider, more circular, and rounder morphology, including the presence of giant multinucleated cells, compared to both their M1 counterparts and MMØ macrophages. The cytoplasm of M2 macrophages displayed a high density with more uniformly distributed actin, devoid of intense local spots, achieving the highest integrated density among the studied cell types, including MMØ and M1 (Fig. 2F). The nuclei of M2 macrophages were rounder and more well-defined, underscored by a significant increase in nuclear circularity and nuclear Feret’s diameter relative to M1 macrophages (Fig. 2F, Suppl. Table 2, Suppl. Figure 1).

#### Proteomic phenotypic characterization of macrophage polarization: CD marker expression

M1 macrophages exhibited 20 significant CD markers compared to M2, 19 relatives to monocytes, 3 to GMØ, 9 to MMØ, and 4 to cMØ (*p* ≤ 0.05, Suppl. Table 1). Specifically, CD274 had the highest significant differential abundance in M1 relative to Mo, GMØ, MMØ, and M2 (*p* ≤ 0.05), establishing it as a unique M1 marker (Table 1; Fig. 1). Additionally, M1 macrophages displayed significantly higher abundance of CD37, CD80, CD86, CD180, and CD369 compared to M2 (*p* ≤ 0.05) (Fig. 1, Suppl. Table 1).

M2 macrophages displayed 20 significant CD markers that differentiated them from M1, 21 from monocytes, 15 from GMØ, 19 from MMØ, and 13 from cMØ (*p* ≤ 0.05) (Suppl. Table 1). Differential abundance analysis identified six key CD markers that uniquely characterize M2, namely CD1B2+, CD11B+, CD206+, CD16A-, CD109- and CD180- (Table 1; Fig. 1), with CD1B2 being exclusively expressed in M2. Compared to M1 macrophages, M2 macrophages in addition to their 6 unique markers also showed significantly higher abundance of CD18, CD115 (CSF1R), CD1E, and CD107A, CD107B (*p* ≤ 0.05), and lacked (below the limit of detection) M1-associated CD markers such as CD16A, CD80, CD86, CD109, CD180, and CD274 (Fig. 1, Suppl. Table 1). Notably, CD11B, CD18, CD115, CD163 and CD206 were identified in both cellular and secretome fractions across macrophage subtypes, with the M2 subtype showing the highest Secretome-to-Lysate (S/L) intensity ratios, ranging from 0.86 to1.18 (Fig. 1, Suppl. Table 3). Collectively, using a classification strategy based on CD64 and CD14 expression, unique CD marker profiles for monocytes and macrophage subtypes could be established, as detailed in Table 1and Fig. 1B.

#### Proteomic profiling and functional characterization of macrophage polarization

The comparison of M1 macrophages with GMØ identified 153 DAPs (122 in whole cell lysate, 28 in the secretome, and 3 in both) and with monocytes 932 DAPs (763 in whole cell lysate, 131 in the secretome, and 38 in both, Table 2, Suppl. Tables 15–16, Suppl. Figure 6 A-B). The comparison of M2 macrophages with MMØ identified 200 DAPs (138 in whole cell lysate, 55 in the secretome, and 7 in both) and with monocytes 1169 DAPs (935 in whole cell lysate, 130 in the secretome, and 104 in both) (Table 2, Suppl. Tables 17–18, Suppl. Figure 7 A-B). M1 and M2 macrophages shared 33.5% (527 proteins) DAPs compared to monocytes, while 25.7.0% (405 proteins) DAPs were specific to M1, and 40.8% (642 proteins) were exclusive to M2 (Suppl. Table 19, Suppl. Figure 8 A).

A comparison of protein abundance between M1 and M2 macrophages, yielded 1262 DAPs (800 in whole cell lysate, 422 in the secretome, and 40 in both) (Table 2, Suppl. Table 20, Suppl. Figure 8B). Subsequent analysis of proteins that were differentially abundant between M1 and, separately, M2 and each of the other cell types (Mo, GMØ, MMØ, and M1/M2), revealed 20 M1-specific markers and 57 M2-specific markers (Suppl. Table 21). The hub analysis of DAPs utilizing four CytoHubba algorithms identified 4 hub proteins for M1— IL1B, MMP3, CCL3, and CD274 and 16 hub proteins for M2 macrophages, including FN1, A2M, PDGFA, MMP12, ITGAM, and IL1RN (Table 3, Suppl. Table 22).

**Table 3:** Differential abundant proteins (DAPs) markers of M1 and M2 macrophages.

Protein	Accession IDs	Nname		M1_Mo		M1_M2		M1_GMØ		M1_MMØ	
				Secreted	Cell lysate	Secreted	Cell lysate	Secreted	Cell lysate	Secreted	Cell lysate
MMP3	W5P4V3	Stromelysin-1 (EC 3.4.24.17) (Matrix metalloproteinase-3)	Fold Change	4.25		4.62		5.19		4.22	
			p-value	0.0348		0.0175		0.0128		0.0248	
IL1B	P21621	Interleukin-1 beta	Fold Change		2.66		2.39		2.98		2.54
			p-value		0.0184		0.0262		0.0115		0.0189
	M4WG34		Fold Change		5.54		6.18		10.27		5.42
			p-value		0.0313		0.0208		0.0026		0.0347
CCL3	W5P2M5	C-X-C motif chemokine 3	Fold Change	4.41		3.40		4.02		3.30	
			p-value	0.0022		0.0006		0.0003		0.0012	
CD274	W5PVJ5	Programmed cell death 1 ligand 1	Fold Change		4.80		4.64		2.83		4.03
			p-value		0.0000		0.0001		0.0133		0.0006

				M2_Mo		M2_M1		M2_GMØ		M2_MMØ	
				Secreted	Cell lysate	Secreted	Cell lysate	Secreted	Cell lysate	Secreted	Cell lysate
MMP12	W5P4C9	Matrix metalloproteinase-12	Fold Change	7.55	5.26	3.83		7.46		6.85	
			p-value	0.0006	0.0510	0.0372		0.0001		0.0004	
IL1RN	W5QI35	Interleukin-1 receptor antagonist protein	Fold Change	5.07		4.56		6.94		4.85	4.39
			p-value	0.0039		0.0035		0.0003		0.0000	0.0002
FN1	W5QDG8	Fibronectin	Fold Change	4.74		4.27	3.73	3.51	-3.73	4.05	
			p-value	0.0001		0.0000	0.0013	0.0000	0.0002	0.0000	
A2M	W5NRI1	Alpha-2-macroglobulin bait region domain-containing protein	Fold Change	4.84	-2.34	5.91		6.90		5.09	
			p-value	0.0056	0.0017	0.0000		0.0000		0.0000	
PDGFA	W5NTX3	Platelet-derived growth factor (PDGF) family profile domain-containing protein	Fold Change	6.42		7.70		7.73		6.72	
			p-value	0.0003		0.0000		0.0000		0.0000	
ITGAM (Shed)	W5PGV0	Integrin subunit alpha M	Fold Change	6.75		5.37		4.98		2.85	
			p-value	0.0010		0.0001		0.0008		0.0061	
CSF1	W5QDI7	Macrophage colony-stimulating factor 1 (MCSF)	Fold Change	6.24		6.51		7.52		5.57	
			p-value	0.0002		0.0000		0.0002		0.0000	
ITGAX (Shed)	W5PH85	VWFA domain-containing protein (Integrin alpha-X)	Fold Change	4.10		5.06		5.73		3.22	
			p-value	0.0224		0.0011		0.0005		0.0006	
MRC1 (Shed)	W5PRI6	Mannose receptor C-type 1	Fold Change	6.47		4.20		5.30		3.56	
			p-value	0.0029		0.0029		0.0016		0.0003	
MMP19	W5PMR2	Matrix metalloproteinase-19	Fold Change	5.29		6.39		5.62		3.17	
			p-value	0.0042		0.0001		0.0002		0.06	
PLAU	W5PF73	Urokinase-type plasminogen activator	Fold Change	4.48	2.80	5.95	2.23	6.14	2.17	2.27	1.61
			p-value	0.0029	0.0021	0.0000	0.0076	0.0000	0.0026	0.0005	0.0005
NRP1	W5Q7S8	Neuropilin-1	Fold Change	3.08		3.92		5.01		3.34	
			p-value	0.0127		0.0067		0.0016		0.0005	
CD1E	W5PBM2	T-cell surface glycoprotein CD1e, membrane-associated	Fold Change		3.80		2.41		3.00		2.49
			p-value		0.0014		0.0006		0.0004		0.0016
SEMA7A	W5P0W4	Semaphorin-7 A	Fold Change	4.60		4.57		5.16		3.74	
			p-value	0.0026		0.0003		0.0004		0.0002	
CFP	W5PTL2	Properdin	Fold Change	5.06		6.91		6.20		3.83	
			p-value	0.0115		0.0001		0.0002		0.0367	
ZEB2	W5PJH6	Zinc finger E-box-binding homeobox 2	Fold Change		2.65		2.73		2.56		1.67
			p-value		0.0148		0.0010		0.0004		0.0030

Mo, Monocytes at 3 h; cMØ, monocyte-derived macrophages differentiated without the addition of exogenous growth factors; GMØ, monocyte-derived macrophages differentiated with GM-CSF (Granulocyte-Macrophage Colony-Stimulating Factor); MMØ, monocyte-derived macrophages differentiated with M-CSF (Macrophage Colony-Stimulating Factor); M1, pro-inflammatory macrophages activated with GM-CSF/ LPS/ INF-γ; M2, anti-inflammatory macrophages activated with M-CSF/ IL-4. Shed markers denote proteins detected in the secretome, whereas standard counterparts were measured in cell lysates. All marker proteins shown were differentially abundant with a false discovery rate (FDR, q-value) < 0.001 and absolute fold change ≥ 2.

Comparative functional enrichment analysis of M1 and M2 relative to monocytes revealed that 46.41% (254 terms) of the enriched BP terms were shared between the two macrophage subtypes (FDR < 0.05). However, 26.1% (143 terms) and 27.6% (151 terms) were uniquely enriched in M1 and M2 macrophages, respectively (Suppl. Table 23). A parallel analysis comparing M1 and M2 to their respective MØ revealed minimal overlap, with only 3.7% (3 terms) of BP terms commonly enriched. M1 displayed unique enrichment in 16.0% (13 terms), while M2 showed distinct enrichment in 80.2% (65 terms) (FDR < 0.05) (Suppl. Table 24).

Enrichment map analysis of M1 relative to GMØ identified significant enrichment in 16 BP terms, systematically categorized into 5 distinct functional clusters. These clusters included defense response external, collagen process catabolic, molecular function catalytic, negative regulation immune, and regulation transport localization (Suppl. Table 25). Conversely, enrichment map analysis of M2 relative to MMØ revealed significant enrichment in 68 BP terms, organized into 11 functional clusters. Key enriched clusters encompassed processes associated with protein process metabolic, regulation of immune system process, organization actin cytoskeleton, negative activity proteolysis, cellular component biogenesis, and positive cell adhesion, and positive cell migration (Suppl. Table 26).

Pathway enrichment analysis further demonstrated that M1 macrophages were associated with seven significantly enriched canonical pathways, including the IL-17 signaling pathway, TNF signaling pathway, rheumatoid arthritis, peroxisome, ferroptosis, C-type lectin receptor signaling pathway, and general metabolic pathways (Suppl. Table 25). In contrast, M2 macrophages revealed enrichment in 22 canonical pathways, prominently including extracellular matrix organization, regulation of insulin-like growth factor (IGF) transport and uptake, cell surface interactions at the vascular wall, lysosome function, the complement and coagulation cascades, and regulation of the actin cytoskeleton (Suppl. Table 26).

**Fig. 3:**
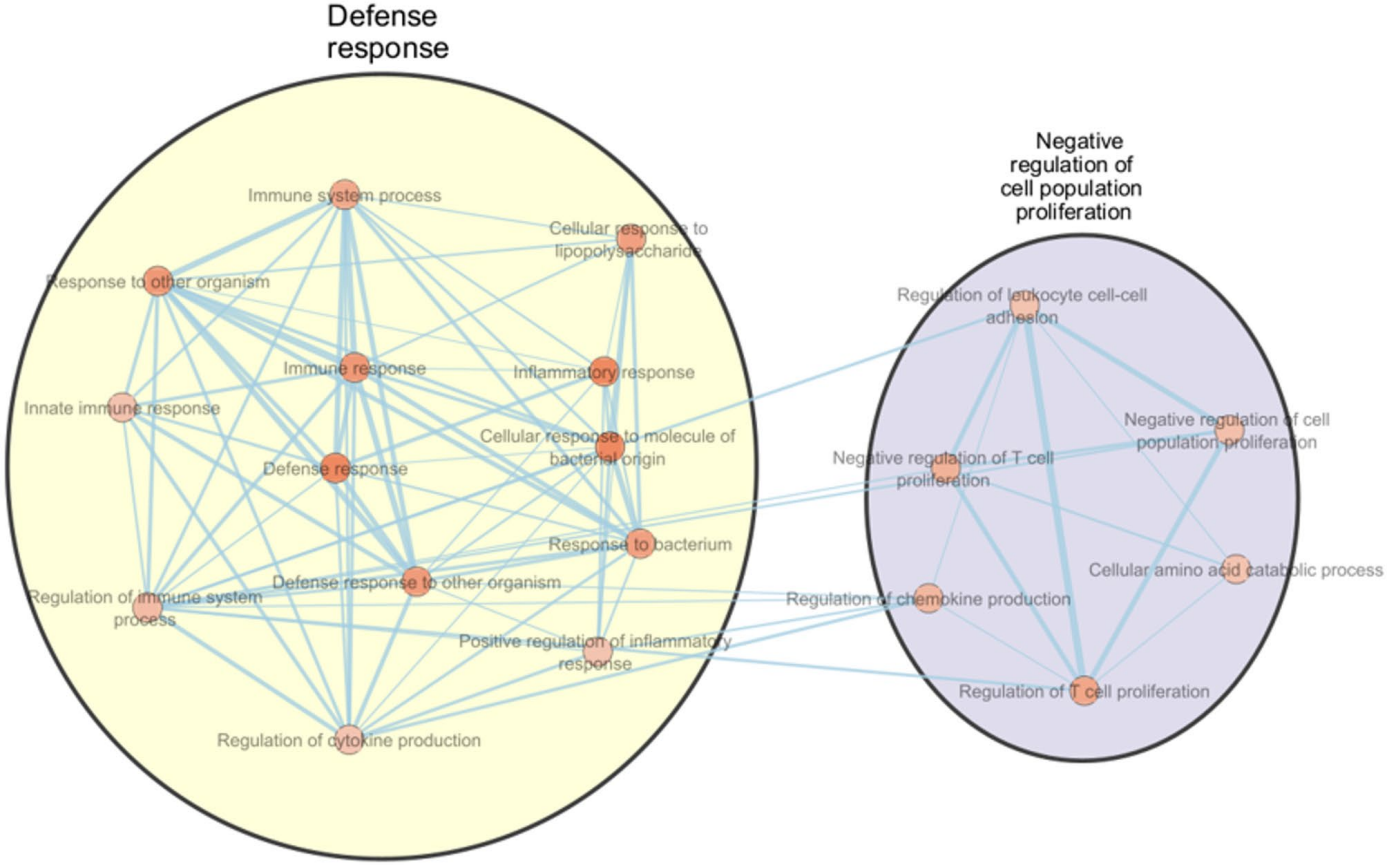
Functional enrichment mapping of M1-specific differentially abundant proteins (DAPs) relative to monocyte and macrophage subtypes (GMØ, MMØ, and M2) using String enrichment analysis. The significant biological processes were clustered and visualized in Cytoscape using EnrichmentMap and AutoAnnotate. Nodes represent individual GO terms, sized according to the number of genes within each term, and colored based on the normalized enrichment scores (NES). Edges, the lines connecting the nodes, are weighted by the thickness, which reflects the richness of shared genes between the connected nodes, illustrating the interconnectedness of biological pathways involved in M1 macrophage functionality.

**Fig. 4:**
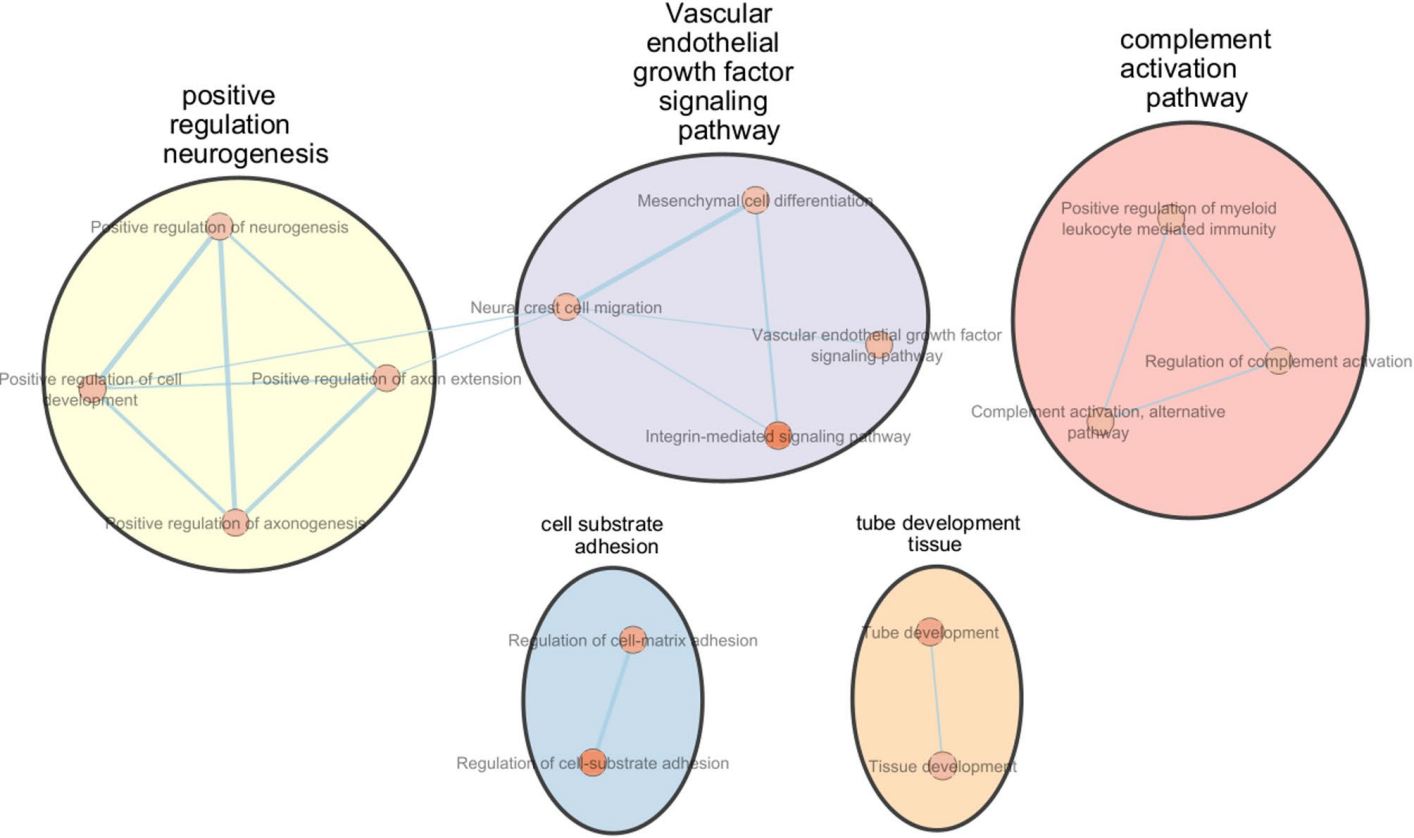
Functional enrichment mapping of M2-specific differentially abundant proteins (DAPs) relative to monocyte and macrophage subtypes (GMØ, MMØ, and M1) using String enrichment analysis. The significant biological processes were clustered and visualized in Cytoscape using EnrichmentMap and AutoAnnotate. Nodes represent individual GO terms, sized according to the number of genes within each term, and colored based on based on the normalized enrichment scores (NES). Edges, the lines connecting the nodes, are weighted by the thickness, which reflects the richness of shared genes between the connected nodes, illustrating the interconnectedness of biological pathways involved in M2 macrophage functionality.

Additionally, functional enrichment analysis of M1-specific markers (20 DAPs) revealed 20 significant enriched BP terms, systematically organized into two distinct functional clusters, namely defense responses and negative regulation of cell proliferation (Table 4; Fig. 3, Suppl. Table 27). Analysis of M2-specific markers (57 DAPs) demonstrated significant enrichment across 15 BP terms, grouped into five functional clusters. These clusters primarily involve processes associated with Vascular endothelial growth factor signaling pathway, positive regulation of neurogenesis, complement activation pathways, cell-substrate adhesion, and tube development in tissue (Table 4; Fig. 4, Suppl. Table 28).

**Table 4:** The gene ontology of biological process terms of M1 and M2 macrophage markers.

#term ID	Term description	Observed gene count	Strength	Signal	False discovery rate
M1 macrophages					
GO:0006952	Defense response	9	0.98	0.79	1.2E-03
GO:0006954	Inflammatory response	6	1.29	1.01	1.2E-03
GO:0071219	Cellular response to molecule of bacterial origin	5	1.62	1.18	1.2E-03
GO:0006955	Immune response	8	0.93	0.69	3.1E-03
GO:0051707	Response to other organism	8	0.92	0.69	3.1E-03
GO:0098542	Defense response to other organism	7	1	0.72	3.7E-03
GO:0009617	Response to bacterium	6	1.11	0.76	5.0E-03
GO:0071222	Cellular response to lipopolysaccharide	4	1.55	0.94	5.0E-03
GO:0042129	Regulation of T cell proliferation	4	1.48	0.86	7.3E-03
GO:0002376	Immune system process	9	0.74	0.51	8.5E-03
GO:0042130	Negative regulation of T cell proliferation	3	1.74	0.79	1.5E-02
GO:0032642	Regulation of chemokine production	3	1.67	0.73	2.1E-02
GO:0002682	Regulation of immune system process	7	0.81	0.47	2.6E-02
GO:2000377	Regulation of reactive oxygen species metabolic process	3	1.56	0.63	3.4E-02
GO:0008285	Negative regulation of cell population proliferation	5	1.03	0.52	3.5E-02
GO:0045087	Innate immune response	5	1.01	0.49	4.1E-02
GO:0009063	Cellular amino acid catabolic process	3	1.51	0.59	4.3E-02
GO:0050729	Positive regulation of inflammatory response	3	1.51	0.58	4.3E-02
GO:1903037	Regulation of leukocyte cell-cell adhesion	4	1.18	0.53	4.3E-02
GO:0001817	Regulation of cytokine production	5	0.98	0.47	4.5E-02
M2 macrophages					
GO:0007229	Integrin-mediated signaling pathway	6	1.44	0.88	1.4E-03
GO:0010810	Regulation of cell-substrate adhesion	7	1.19	0.76	2.3E-03
GO:0035295	Tube development	11	0.76	0.49	1.0E-02
GO:0001952	Regulation of cell-matrix adhesion	5	1.35	0.62	1.1E-02
GO:0001755	Neural crest cell migration	4	1.46	0.48	3.4E-02
GO:0006957	Complement activation, alternative pathway	3	1.84	0.5	3.4E-02
GO:0009888	Tissue development	14	0.57	0.35	3.4E-02
GO:0010720	Positive regulation of cell development	6	1.01	0.43	3.4E-02
GO:0038084	Vascular endothelial growth factor signaling pathway	3	1.79	0.49	3.4E-02
GO:0045773	Positive regulation of axon extension	3	1.79	0.49	3.4E-02
GO:0050772	Positive regulation of axonogenesis	4	1.43	0.48	3.4E-02
GO:0002888	Positive regulation of myeloid leukocyte mediated immunity	3	1.68	0.47	4.0E-02
GO:0048762	Mesenchymal cell differentiation	5	1.12	0.43	4.0E-02
GO:0030449	Regulation of complement activation	3	1.63	0.45	4.4E-02
GO:0050769	Positive regulation of neurogenesis	5	1.07	0.4	4.9E-02

M1, pro-inflammatory macrophages activated with GM-CSF/ LPS/ INF-γ; M2, anti-inflammatory macrophages activated with M-CSF/ IL-4.

Pathway enrichment analysis further showed that M1-specific markers were significantly associated with six canonical pathways, most notably the Toll-like receptor signaling pathway, rheumatoid arthritis, and tuberculosis (Suppl. Table 27). Conversely, M2-specific markers were enriched in nine canonical pathways, including regulation of insulin-like growth factor (IGF) transport and uptake, extracellular matrix organization, and modulation of the complement cascade (Suppl. Table 28).

## Discussion

This study characterized the transitions from monocytes to macrophages and their polarization into M1 and M2 phenotypes through integrated morphological and proteomic analyses, which identified subtype-specific morphological signatures, CD marker profiles, key differentially abundant proteins, and associated biological pathways.

The differentiated cMØ, GMØ and MMØ macrophages exhibited significant morphological changes compared to their monocyte precursors, including increased cell size, reduced circularity, and enhanced cytoplasmic granularity, indicative of functional maturation^85–87^. These alterations were accompanied by extensive cytoplasmic expansion, providing the structural capacity for a greater number of active organelles, including lysosomes and phagosomes, thereby enhancing cytoplasmic granularity, a hallmark of macrophage functionality^86–90^. Additionally, the formation of complex cell membrane structures, such as ruffles and pseudopodia, supported cellular motility and phagocytic efficiency, underscoring the dynamic nature of macrophage adaptation^91–95^.

Despite the lack of significant differences in overall shape descriptors between cMØ, GMØ and MMØ cells, the comparative analysis highlighted that M1 and M2 macrophages exhibited distinct morphological features, correlating with their specialized functional roles within the immune system^26,28,29,96–100^. M1 macrophages exhibited elongated, irregular shapes with increased perimeter, higher aspect ratio, and reduced circularity along with a tightly packed, dotted actin texture compared to M2 macrophages. These changes are in alignment with previous studies and suggest significant cytoskeletal reorganization, enhancing migratory and pro-inflammatory capabilities of M1 macrophages^101–103^. The altered nuclear shape in M1 macrophages, with increased Feret’s diameter and decreased circularity, may indicate changes in nuclear organization that could influence chromatin condensation and histone modification, potentially impacting genetic programs associated with the macrophage phenotype^104,105^. In contrast, M2 macrophages demonstrated broader and more circular morphologies, coupled with the highest cytoplasmic integrated density among the evaluated cell types. This elevated integrated density indicated a substantial presence of organelles, including mitochondria, lysosomes, and granules, which were crucial for their roles in tissue repair and resolution of inflammation^85,88,106–109^. Additionally, the observed cellular expansion in M2 macrophages is likely linked to actin cytoskeleton rearrangements, as evidenced by uniform actin staining across the cytoplasm. Such structural reorganization supports the macrophages’ necessary adaptations for their roles in immune modulation and tissue repair, facilitating effective response and healing processes^110^. These distinct morphological features highlighted the divergent functional roles and adaptive responses of M1 and M2 macrophages during polarization^86,88,109,111,112^.

Although our morphological findings align with several human studies^26,29,48,96,98,113^, the literature shows no consensus—either within humans^28,114–116^ or across other species such as mouse^27,28^ and canine^117^—regarding the direction of M1–M2 shape differences. This variability is particularly evident ex vivo, where tissue microenvironments modulate cell shape and can attenuate or reverse in-vitro trends^101,118–123^. While these discrepancies could stem from variations in polarization protocols reported in the literature, they highlight that reliance solely on qualitative morphology is insufficient. Instead, quantitative cytoprofiling—integrating shape descriptors and intensity metrics^29,97,114,118^—provides a precise, robust framework for discriminating macrophage subtypes and resolving subtle morphological differences, as supported by previous studies^113,124–127^. Future expansion of the dataset will enable the application of machine learning approaches, facilitating more advanced and refined cytoprofiling, enhancing subtype classification, and enabling the detection of subtle phenotypic patterns that may not be captured through conventional quantitative methods.

Proteomic analysis revealed that monocytes differentiate into macrophages not only upon GM-CSF^128,129^ or M-CSF^11,130^ induction, but also spontaneously in the absence of exogenous growth factors^131–133^. Spontaneous differentiation yielded a heterogeneous macrophage population with predominantly inflammatory but also reparative phenotypic and functional characteristics. cMØ macrophages demonstrated an enrichment in processes associated with receptor intracellular endocytosis, regulation of response to stimulus, and cell adhesion leukocyte. Similarly, as expected, GMØ macrophages were enriched in pathways related to chemical stimulus signaling and hydrolase activity, which align with the classical pro-inflammatory characteristics of M1 macrophages. Conversely, MMØ macrophages showed enrichment in processes indicative of M2 macrophages, including the negative regulation of responses to stimuli, cellular component biogenesis, and endocytosis, reflecting their anti-inflammatory and reparative functions. Collectively, these data highlight the molecular specialization of macrophage subtypes in response to specific environmental cues, and underscore the functional and molecular divergence that underpins their distinct roles within the immune system^130,134–138^.

Upon polarization, proteomic profiling revealed distinct CD marker expression patterns critical for identifying M1 and M2 macrophage subtypes. M1 macrophages were characterized by markers associated with pro-inflammatory functions, including CD86^139,140^, CD68^141–144^, CD64^141–144^, CD274^145–149^, and CD61^150,151^, all of which are linked to the classical activation pathway. CD274 was notably upregulated in M1 macrophages, serving as a key marker to distinguish them from monocytes and other macrophage subtypes. This finding supports prior research, which demonstrated a strong correlation between CD274 expression and M1 polarization especially in inflammatory and cancerous contexts^149^, and upregulation of CD274 in LPS-induced M1 macrophages via the JAK-STAT3 signaling pathway^152^, further highlighting its utility as a biomarker for M1 macrophages.

Conversely, M2 macrophages were characterized by markers such as CD206^153,154^, CD11b^115,155–158^, CD18^158–161^, CD1b-2^156,162^, and CD163^163,164^, which are associated with anti-inflammatory and reparative functions. The absence (below the limit of detection) of classical M1 markers, such as CD86, CD180, CD16A, CD274, CD80 and CD109, in M2 macrophages further supports their polarization towards an anti-inflammatory phenotype. Notably, CD206, the macrophage mannose receptor, is a scavenger receptor predominantly expressed by M2 macrophages^153,154^. It plays crucial roles in immune modulation by mediating the scavenging of endogenous glycoproteins, aiding pathogen recognition, and facilitating antigen presentation, thereby promoting immune homeostasis and tissue repair^165,166^.

Transmembrane CD markers (CD11B, CD18, CD115, CD163, and CD206) displayed the highest Secretome-to-Lysate (S/L) ratios in M2 macrophages relative to other subtypes; ranging from 0.86 to 1.18. In line with UniProt annotations designating them as cell surface proteins, their occurrence in conditioned medium is more plausibly attributable to regulated shedding rather than active secretion. Such release is governed by complex transcriptional regulation and post-translational modifications^68,167,168^, including proteolytic cleavage^168^, TACE-mediated shedding^169^ and extracellular vesicle (EV)-associated release^170^ that enhance the shedding of key proteins and enable macrophages to adapt to diverse environmental stimuli, modulate inflammatory responses and promote tissue repair^171–175^.

The selective abundance of CD1B2 in M2 macrophages, and the corroboration of its absence (below the limit of detection) in GM-CSF and M-CSF-differentiated Mo macrophages with previous research^156^, strongly indicate CD1B2 as marker for M2-polarized macrophages. While CD1b2 has been traditionally characterized as a lipid-presenting protein in ovine and human dendritic cells^176–178^, recent evidence revealed its upregulation in human M2 macrophages^156,162^, expanding its known functional roles. However, further research is required to determine if this abundance pattern is species-specific or influenced by microenvironmental factors and to elucidate its functional significance in macrophage-mediated immune modulation and inflammation resolution.

Proteomic profiling of macrophage polarization revealed a clear distinction between M1 and M2 macrophages. M1 macrophages exhibited 20 unique DAPs with network analysis identifying IL1B^108,179,180^, MMP3^181,182^, CCL3^52,183^, and CD274^145–149^, all of which are well-established mediators of pro-inflammatory pathways, as potential hub proteins, consistent with their role in pathogen defense, immune cell recruitment, and inflammation^108,179,180^. Functional enrichment analysis of the 20 unique differentially abundant proteins in M1 macrophages confirmed their involvement in cytokine production regulation, cellular response to lipopolysaccharide, and inflammatory response, supporting the established pro-inflammatory role of M1 macrophages^5,10,25,184,185^.

M2 macrophages exhibited 57 unique DAPs with network analysis identifying 16 hub proteins, many of which have well established immunomodulating and pro-regenerative functions, including fibronectin 1 (FN1)^186–188^, alpha-2‐macroglobulin (A2M)^189,190^, Platelet-Derived Growth Factor Subunit A (PDGFA)^191–195^, MMP12^196–199^, ITGAM^115,155–158^ and Interleukin-1 Receptor Antagonist Protein (IL1RN)^10,200–205^. Functional enrichment analysis of the 57 unique differentially abundant proteins in M2 macrophages revealed their involvement in tissue regeneration and anti-inflammatory pathways, specifically VEGF signaling, mesenchymal cell differentiation, positive regulation of neurogenesis, complement activation, and angiogenesis, highlighting the multifaceted role of M2 macrophages in promoting tissue repair and resolving inflammation^7,206–208^.

The distinct hub proteins for each cell type highlight key molecular regulators of macrophage activation and polarization, suggesting potential biomarkers and therapeutic targets for immune-mediated diseases. Further investigation into these regulatory mechanisms is warranted to explore their translational potential in macrophage-targeted therapies.

This study was constrained by the limited availability of sheep-specific immunological reagents, including antibodies and recombinant cytokines^32,38,42^. Consequently, we adopted adherence-based monocyte isolation rather than more selective methods (e.g., CD marker sorting). Adherence-based approaches are widely used^45–48^, and when applied under short three-hour protocols—as opposed to prolonged 24-hour incubations—they induce only minimal activation^209^ and yield higher numbers of monocyte-derived macrophages than immunomagnetic column methods, as reported in the literature^45^. Nevertheless, they can still influence monocyte phenotype and functional attributes^210–212^. Future studies should investigate the molecular mechanisms underlying flask adherence to better define its implications for macrophage biology.

A further limitation of this study is the reliance on heterologous cytokines (recombinant human cytokines) in place of species-matched ovine cytokines. Although differences from endogenous ovine cytokines cannot be excluded, this approach is supported by the high degree of sequence homology between ovine and human CSF family cytokines, sharing over 80% amino acid identity, and by the more moderate conservation of IL-4 and IFN-γ at 68.9% and 62.4%, respectively, as shown by published reports^213^ and UniProt BLAST. This conservation, together with the successful application of human GM-CSF/IL-4 or M-CSF to drive myeloid differentiation in other species, including porcine^214,215^, feline^216,217^, rabbit^218,219^, and canine models^117,220^, is consistent with our observation of robust differentiation and polarization of ovine monocytes, as evidenced by macrophage-like morphology, adherence, and polarization marker expression. Future studies employing species-matched cytokines will be important to refine and further validate these conclusions^214,216,218,219,221–226^.

In conclusion, this study highlights the complexity of macrophage classification in sheep, particularly given the limitations of available immunological tools. We advance beyond traditional qualitative morphology by integrating high-resolution mass spectrometry–based proteomics, quantitative morphological analysis, and bioinformatics to generate comprehensive “cytoprofiles” that enable detailed macrophage characterization and exploration of functional heterogeneity. Through this in-depth approach, this study contributes to the evidence-based selection of biomedically relevant preclinical models, thereby enhancing scientific rigor, supporting the reduction of animal use, and enabling future comparative studies between human and ovine systems to inform the development of novel macrophage-targeted immunotherapies for immune-mediated diseases.

## Supplementary Information

Below is the link to the electronic supplementary material.

Supplementary Material 1

## Data Availability

The datasets generated and analyzed during the current study are included in this published article (and its Supplementary Information files) or available from the corresponding author on reasonable request. The mass spectrometry proteomics data were deposited to the ProteomeXchange Consortium via the PRIDE partner repository with the dataset identifier PXD069059 (cell lysates) and PXD068541 (secretomes).
