# Supplementary material for: Proteomic and morphologic characterization of ovine macrophage differentiation and polarization
Source: Sci Rep. 2025 Dec 1;16:693. doi: 10.1038/s41598-025-30269-x (PMC12780204; doi:10.1038/s41598-025-30269-x)
Supplement: Supplementary file 1 — Supplementary Material 1 [file 41598_2025_30269_MOESM1_ESM.pdf]

Supplementary Figures

Supplementary Figure 1: Quantitative morphometric analysis of monocytes (Mo) and macrophage subsets (cMØ, GMØ, MMØ, M1, M2). Parameters measured include: (A) Cell area, defined as the selection area in square pixels (PX); (B) Cell perimeter, the length of the outer boundary of the cell; (C) Aspect ratio, ratio of the major to minor axis, serving as an indicator of cell elongation; (D) Roundness, calculated as $4 \times area / (\pi \times major\ axis\ length^2)$, inversely related to aspect ratio; (E) Integrated intensity, product of area and mean gray value; and (F) Feret's diameter, the maximum distance between any two points along the cell boundary. Boxplots represent the median (line), interquartile range (box), and minimum/maximum values (whiskers). Letters above boxplots denote statistically significant differences ($p < 0.05$), calculated with ANOVA on Ranks tests followed by Tukey's post-hoc pairwise comparisons: (a) vs. Mo, (b) vs. cMØ, (c) vs. GMØ, (d) vs. MMØ, (e) vs. M1, (f) vs. M2. Mo, Monocytes at 3 hours; cMØ, monocyte-derived macrophages differentiated without the addition of exogenous growth factors; GMØ, monocyte-derived macrophages differentiated with GM-CSF (Granulocyte-Macrophage Colony-Stimulating Factor); MMØ, monocyte-derived macrophages differentiated with M-CSF (Macrophage Colony-Stimulating Factor); M1, pro-inflammatory macrophages activated with GM-CSF/ LPS/ INF-γ; M2, anti-inflammatory macrophages activated with M-CSF/ IL-4.

Supplementary Figure 2: Venn diagrams illustrating compartment-specific expression profiles and regulatory patterns associated with macrophage differentiation (cMØ; monocyte-derived macrophages differentiated without exogenous cytokines), GM-CSF stimulation (GMØ; GM-CSF–differentiated macrophages), or M-CSF stimulation (MMØ; M-CSF–differentiated macrophages). Panels depict differentially abundant proteins (DAPs) in (A) cMØ vs. Mo (monocytes at 3 hours), (B) GMØ vs. Mo, and (C) MMØ vs. Mo. Proteins significantly upregulated or downregulated in the cell lysate are denoted as UPc and DWc, respectively, while those in the secretome are indicated as UPs (upregulated) and DWs (downregulated). DAPs were identified using a false discovery rate (FDR, q-value) ≤ 0.05 and an absolute fold change ≥ 2.

Supplementary Figure 3: (A) Venn diagram illustrating the overlap and subtype-specific distribution of differentially abundant proteins (DAPs) in GMØ (GM-CSF–differentiated macrophages) and MMØ (M-CSF–differentiated macrophages), relative to Mo (monocytes at 3 hours). (B) Four-way Venn diagram illustrating compartment-specific expression patterns and regulatory differences between GMØ and MMØ. Proteins significantly upregulated or downregulated in the cell lysate are denoted as UPc and DWc, respectively, while those in the secretome are designated as UPs (upregulated) and DWs (downregulated). Proteins were

considered differentially abundant based on a false discovery rate (FDR, q-value) ≤ 0.05 and an absolute fold change ≥ 2.

Supplementary Figure 4: Venn diagrams illustrating subtype-specific and shared differentially abundant proteins (DAPs) distinguishing GMØ (GM-CSF–differentiated macrophages) (A) and MMØ (M-CSF–differentiated macrophages) (B) from other macrophage subtypes, including Mo (monocytes at 3 hours), cMØ (macrophages differentiated without exogenous growth factors), MMØ or GMØ (respectively), M1 (pro-inflammatory), and M2 (anti-inflammatory). Proteins were considered differentially abundant based on a false discovery rate (FDR, q-value) ≤ 0.05 and an absolute fold change ≥ 2.

Supplementary Figure 5: Venn diagrams depicting the overlap and distinction of differentially abundant proteins (DAPs) between each macrophage differentiation state—(A) cMØ (macrophages differentiated without exogenous cytokines), (B) GMØ (GM-CSF–differentiated macrophages), and (C) MMØ (M-CSF–differentiated macrophages)—and polarized macrophage subsets M1 (pro-inflammatory) or M2 (anti-inflammatory), each relative to monocytes (Mo; 3-hour time point). Shared DAPs indicate proteomic alignment with M1 or M2 phenotypes, whereas unique DAPs reflect subtype-specific molecular signatures. DAPs were identified based on a false discovery rate (FDR, q-value) ≤ 0.05 and an absolute fold change ≥ 2.

Supplementary Figure 6: Venn diagrams illustrating compartment-specific expression profiles and regulatory patterns associated with M1 macrophage polarization relative to (A) GMØ (GM-CSF–differentiated macrophages) and (B) Mo (monocytes at 3-hour time point). Differentially abundant proteins (DAPs) are categorized based on subcellular localization and direction of regulation: UPc (upregulated in cell lysate), DWc (downregulated in cell lysate), UPs (upregulated in secretome), and DWs (downregulated in secretome). Differential abundance was defined by a false discovery rate (FDR, q-value) ≤ 0.05 and an absolute fold change ≥ 2.

Supplementary Figure 7: Venn diagrams illustrating compartment-specific expression profiles and regulatory patterns associated with M2 macrophage polarization relative to (A) MMØ (M-CSF–differentiated macrophages) and (B) Mo (monocytes at 3-hour time point). Differentially abundant proteins (DAPs) are categorized based on subcellular localization and direction of regulation: UPc (upregulated in cell lysate), DWc (downregulated in cell lysate), UPs (upregulated in secretome), and DWs (downregulated in secretome). Differential abundance was defined by a false discovery rate (FDR, q-value) ≤ 0.05 and an absolute fold change ≥ 2.

Supplementary Figure 8: (A) Venn diagram illustrating the overlap and subtype-specific distribution of differentially abundant proteins (DAPs) in M1 (pro-inflammatory macrophages activated with GM-CSF/LPS/IFN-γ) and M2 (anti-inflammatory macrophages activated with M-

CSF/IL-4), relative to Mo (monocytes at 3 hours). (B) Four-way Venn diagram illustrating compartment-specific expression patterns and regulatory differences between M1 and M2. Proteins significantly upregulated or downregulated in the cell lysate are denoted as UPc and DWc, respectively, while those in the secretome are designated as UPs (upregulated) and DWs (downregulated). Differential abundance was defined by a false discovery rate (FDR, q-value) ≤ 0.05 and an absolute fold change ≥ 2.

Supplementary Figures

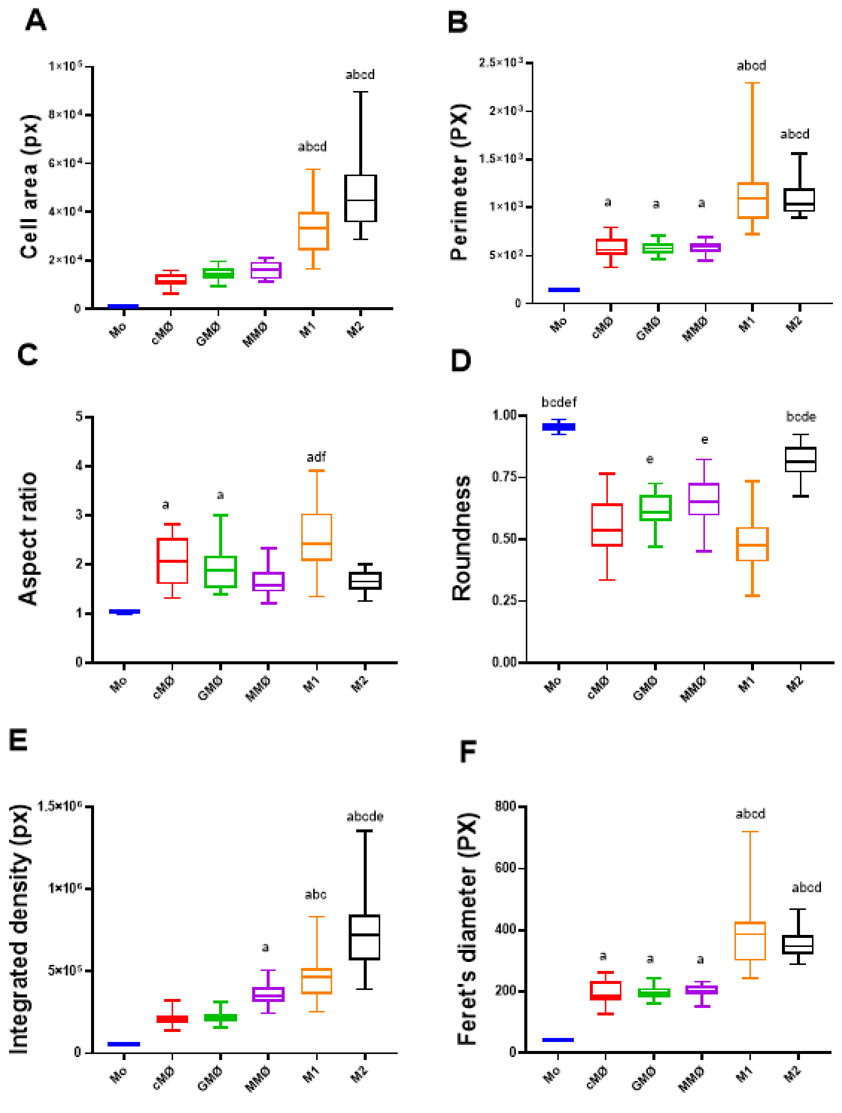

Supplementary figure 1: Quantitative morphometric analysis of monocytes (Mo) and macrophage subsets (cMØ, GMØ, MMØ, M1, M2). Parameters measured include: (A) Cell area, defined as the selection area in square pixels (PX); (B) Cell perimeter, the length of the outer boundary of the cell; (C) Aspect ratio, ratio of the major to minor axis, serving as an indicator of cell elongation; (D) Roundness, calculated as 4 × area / (π × major axis length²), inversely related to aspect ratio; (E) Integrated intensity, product of area and mean gray value; and (F) Feret's diameter, the maximum distance between any two points along the cell boundary. Boxplots represent the median (line), interquartile range (box), and minimum/maximum values (whiskers). Letters above boxplots denote statistically significant differences ($p < 0.05$), calculated with ANOVA on Ranks tests followed by Tukey's post-hoc pairwise comparisons: (a) vs. Mo, (b) vs. cMØ, (c) vs. GMØ, (d) vs. MMØ, (e) vs. M1, (f) vs. M2. Mo, Monocytes at 3 hours; cMØ, monocyte-derived macrophages differentiated without the addition of exogenous growth factors; GMØ, monocyte-derived macrophages differentiated with GM-CSF (Granulocyte-Macrophage Colony-Stimulating Factor); MMØ, monocyte-derived macrophages differentiated with M-CSF (Macrophage Colony-Stimulating Factor); M1, pro-inflammatory macrophages activated with GM-CSF/ LPS/ INF-γ; M2, anti-inflammatory macrophages activated with M-CSF/ IL-4.

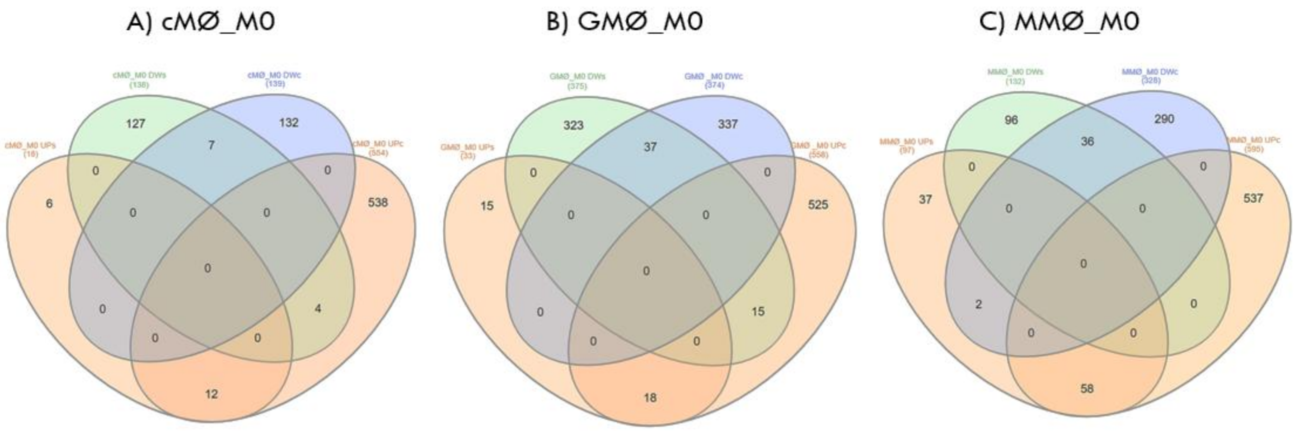

Supplementary figure 2: Venn diagrams illustrating compartment-specific expression profiles and regulatory patterns associated with macrophage differentiation (cMØ; monocyte-derived macrophages differentiated without exogenous cytokines), GM-CSF stimulation (GMØ; GM-CSF–differentiated macrophages), or M-CSF stimulation (MMØ; M-CSF–differentiated macrophages). Panels depict differentially abundant proteins (DAPs) in (A) cMØ vs. Mo (monocytes at 3 hours), (B) GMØ vs. Mo, and (C) MMØ vs. Mo. Proteins significantly upregulated or downregulated in the cell lysate are denoted as UPc and DWc, respectively, while those in the secretome are indicated as UPs (upregulated) and DWs (downregulated). DAPs were identified using a false discovery rate (FDR, q-value) ≤ 0.05 and an absolute fold change ≥ 2.

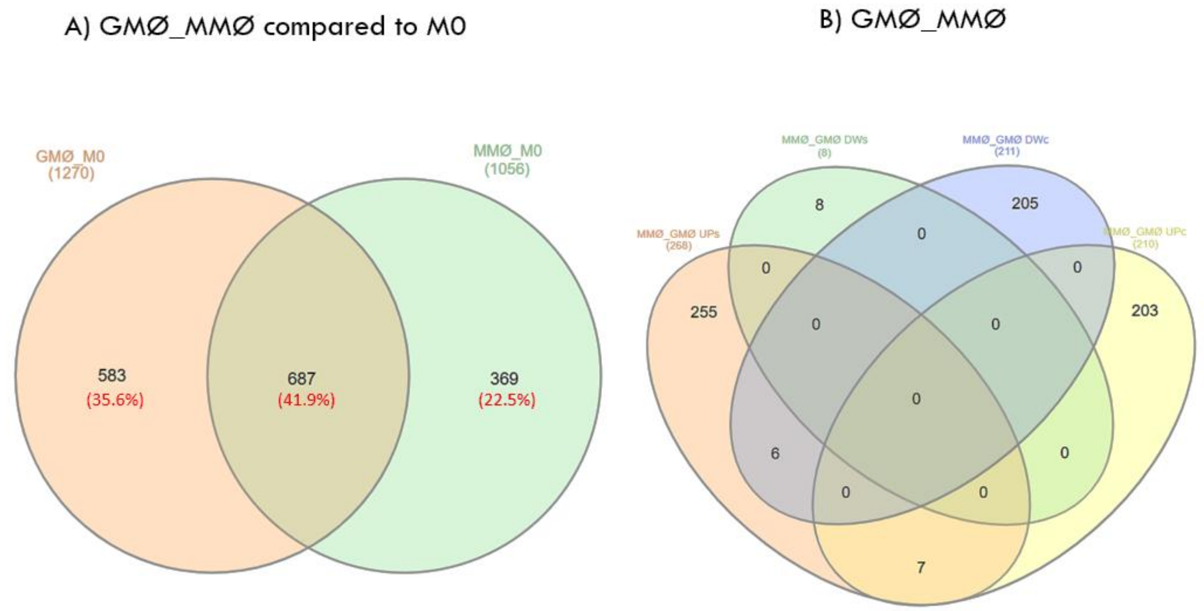

Supplementary figure 3: (A) Venn diagram illustrating the overlap and subtype-specific distribution of differentially abundant proteins (DAPs) in GMØ (GM-CSF–differentiated macrophages) and MMØ (M-CSF–differentiated macrophages), relative to Mo (monocytes at 3 hours). (B) Four-way Venn diagram illustrating compartment-specific expression patterns and regulatory differences between GMØ and MMØ. Proteins significantly upregulated or downregulated in the cell lysate are denoted as UPc and DWc, respectively, while those in the secretome are designated as UPs (upregulated) and DWs (downregulated). Proteins were considered differentially abundant based on a false discovery rate (FDR, q-value) ≤ 0.05 and an absolute fold change ≥ 2.

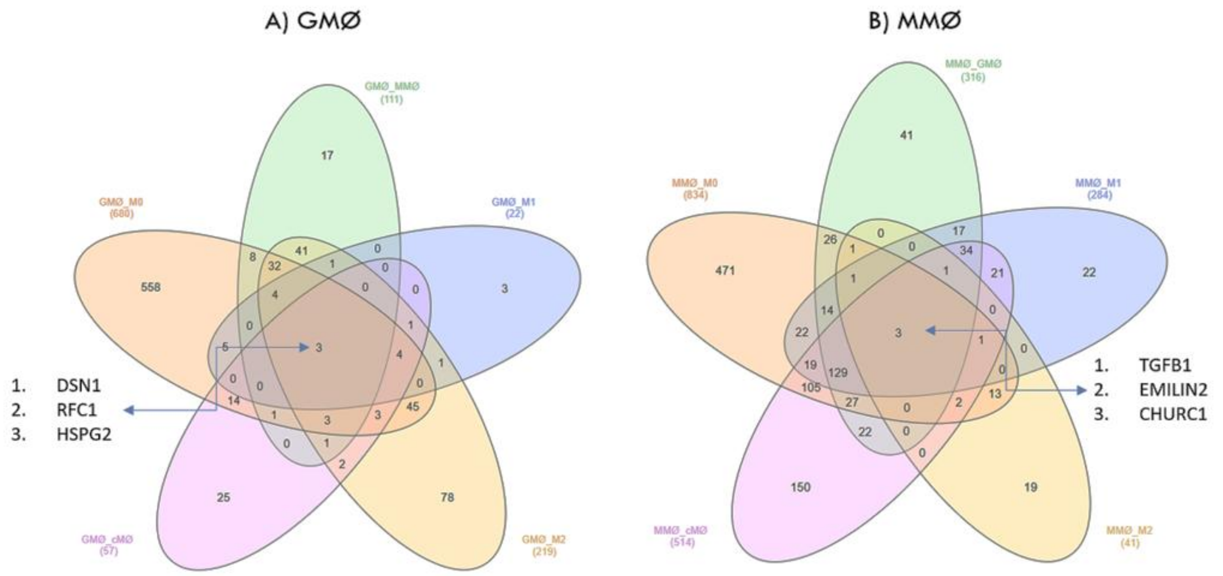

Supplementary figure 4: Venn diagrams illustrating subtype-specific and shared differentially abundant proteins (DAPs) distinguishing GMØ (GM-CSF–differentiated macrophages) (A) and MMØ (M-CSF–differentiated macrophages) (B) from other macrophage subtypes, including Mo (monocytes at 3 hours), cMØ (macrophages differentiated without exogenous growth factors), MMØ or GMØ (respectively), M1 (pro-inflammatory), and M2 (anti-inflammatory). Proteins were considered differentially abundant based on a false discovery rate (FDR, q-value) ≤ 0.05 and an absolute fold change ≥ 2.

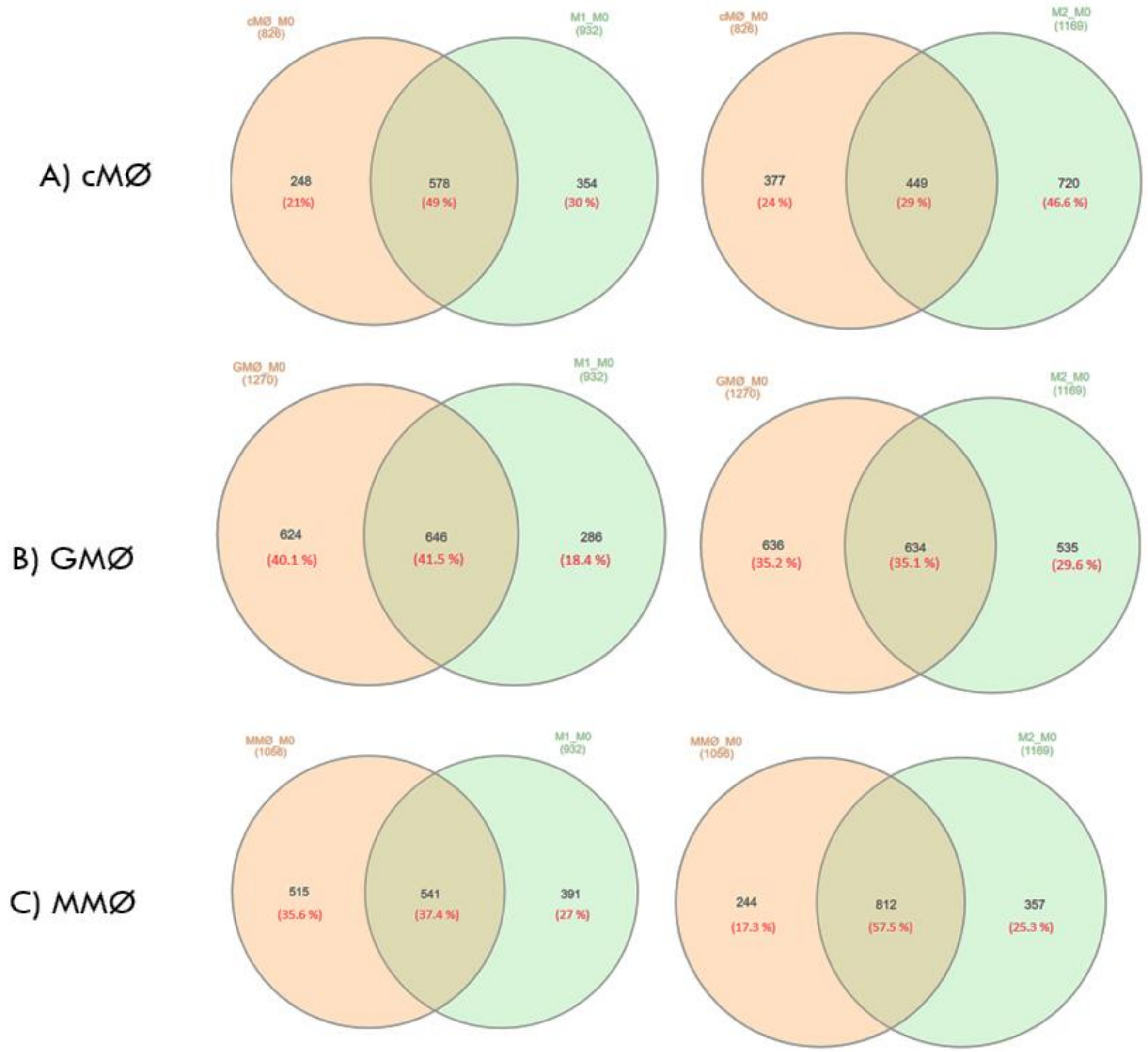

Supplementary figure 5: Venn diagrams depicting the overlap and distinction of differentially abundant proteins (DAPs) between each macrophage differentiation state—(A) cMØ (macrophages differentiated without exogenous cytokines), (B) GMØ (GM-CSF–differentiated macrophages), and (C) MMØ (M-CSF–differentiated macrophages)—and polarized macrophage subsets M1 (pro-inflammatory) or M2 (anti-inflammatory), each relative to monocytes (Mo; 3-hour time point). Shared DAPs indicate proteomic alignment with M1 or M2 phenotypes, whereas unique DAPs reflect subtype-specific molecular signatures. DAPs were identified based on a false discovery rate (FDR, q-value) ≤ 0.05 and an absolute fold change ≥ 2.

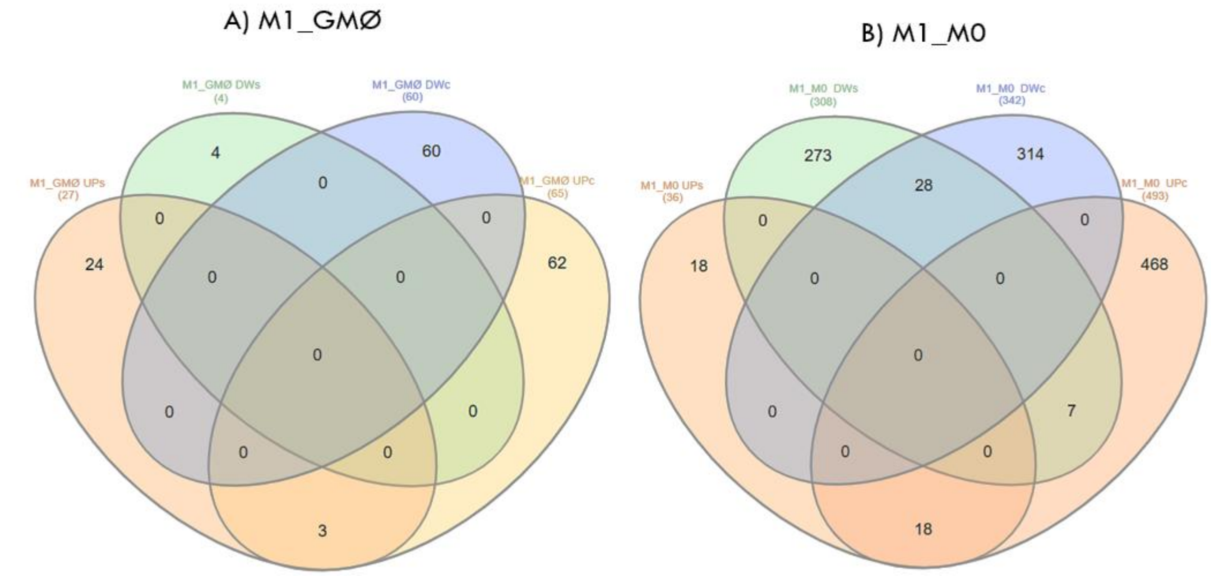

Supplementary figure 6: Venn diagrams illustrating compartment-specific expression profiles and regulatory patterns associated with M1 macrophage polarization relative to (A) GMØ (GM-CSF–differentiated macrophages) and (B) Mo (monocytes at 3-hour time point). Differentially abundant proteins (DAPs) are categorized based on subcellular localization and direction of regulation: UPc (upregulated in cell lysate), DWc (downregulated in cell lysate), UPs (upregulated in secretome), and DWs (downregulated in secretome). Differential abundance was defined by a false discovery rate (FDR, q-value) ≤ 0.05 and an absolute fold change ≥ 2.

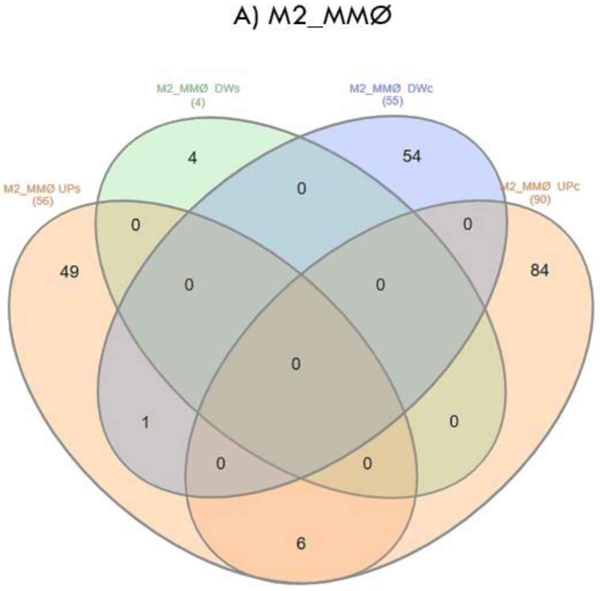

Supplementary figure 7: Venn diagrams illustrating compartment-specific expression profiles and regulatory patterns associated with M2 macrophage polarization relative to (A) MMØ (M-CSF–differentiated macrophages) and (B) Mo (monocytes at 3-hour time point). Differentially abundant proteins (DAPs) are categorized based on subcellular localization and direction of regulation: UPc (upregulated in cell lysate), DWc (downregulated in cell lysate), UPs (upregulated in secretome), and DWs (downregulated in secretome). Differential abundance was defined by a false discovery rate (FDR, q-value) ≤ 0.05 and an absolute fold change ≥ 2.

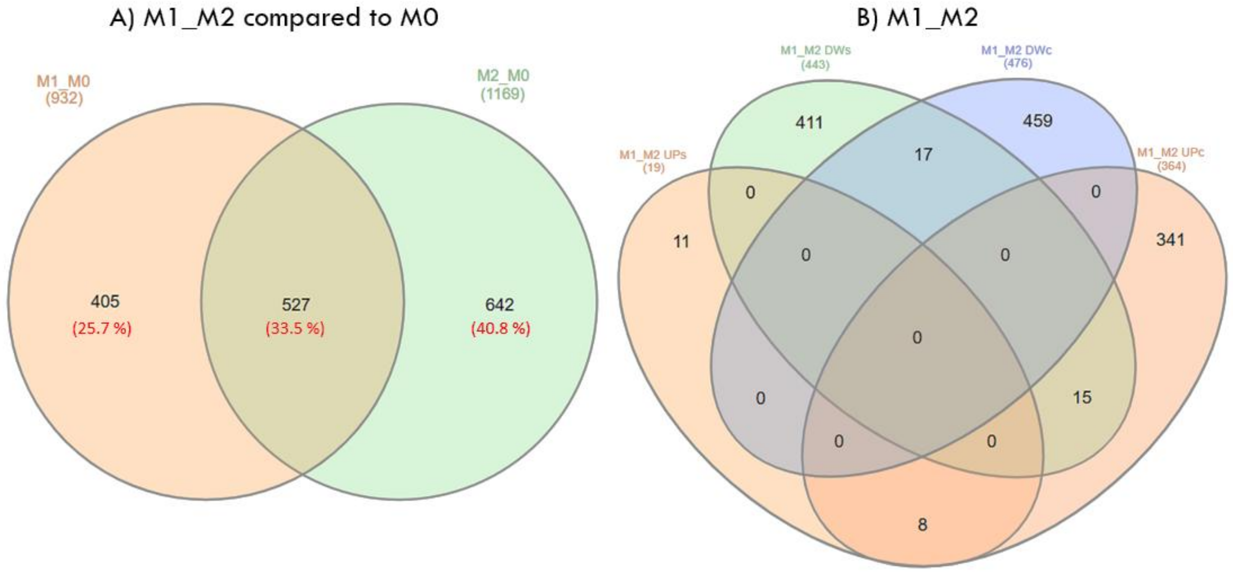

Supplementary figure 8: (A) Venn diagram illustrating the overlap and subtype-specific distribution of differentially abundant proteins (DAPs) in M1 (pro-inflammatory macrophages activated with GM-CSF/LPS/IFN-γ) and M2 (anti-inflammatory macrophages activated with M-CSF/IL-4), relative to Mo (monocytes at 3 hours). (B) Four-way Venn diagram illustrating compartment-specific expression patterns and regulatory differences between M1 and M2. Proteins significantly upregulated or downregulated in the cell lysate are denoted as UPc and DWc, respectively, while those in the secretome are designated as UPs (upregulated) and DWs (downregulated). Differential abundance was defined by a false discovery rate (FDR, q-value) ≤ 0.05 and an absolute fold change ≥ 2.

Supplementary Tables

Supplementary Table 1: Comparative proteomic analysis of 42 identified Cluster of Differentiation (CD) antigens across monocytes and macrophage subtypes. Mo, Monocytes at 3 hours; cMØ, monocyte-derived macrophages differentiated without the addition of exogenous growth factors; GMØ, monocyte-derived macrophages differentiated with GM-CSF (Granulocyte-Macrophage Colony-Stimulating Factor); MMØ, monocyte-derived macrophages differentiated with M-CSF (Macrophage Colony-Stimulating Factor); M1, pro-inflammatory macrophages activated with GM-CSF/ LPS/ INF-γ; M2, anti-inflammatory macrophages activated with M-CSF/ IL-4. Asterisks indicate levels of statistical significance for CD marker expression: * adjusted P-value < 0.05; ** adjusted P-value < 0.01; *** adjusted P-value < 0.001; **** adjusted P-value < 0.0001; ns, not significant.

Supplementary Table 2: Imaging-based comparative morphological analysis of monocytes and macrophage subtypes. "CYTO" indicates cytoplasmic morphological features, while "NC" refers to nuclear compartment features. Mo, Monocytes at 3 hours; cMØ, monocyte-derived macrophages differentiated without the addition of exogenous growth factors; GMØ, monocyte-derived macrophages differentiated with GM-CSF (Granulocyte-Macrophage Colony-Stimulating Factor); MMØ, monocyte-derived macrophages differentiated with M-CSF (Macrophage Colony-Stimulating Factor); M1, pro-inflammatory macrophages activated with GM-CSF/ LPS/ INF-γ; M2, anti-inflammatory macrophages activated with M-CSF/ IL-4. Asterisks indicate levels of statistical significance for CD marker expression: * adjusted P-value < 0.05; ** adjusted P-value < 0.01; *** adjusted P-value < 0.001; **** adjusted P-value < 0.0001; ns, not significant.

Supplementary Table 3: Ratiometric analysis of protein abundance between secretome and lysate fractions to evaluate potential intracellular leakage or protein shedding. Secretome-to-lysate (S/L) ratios were calculated from label-free quantification (LFQ) intensities, where S/L = mean LFQ intensity in conditioned medium ÷ mean LFQ intensity in lysate. Mo, Monocytes at 3 hours; cMØ, monocyte-derived macrophages differentiated without the addition of exogenous growth factors; GMØ, monocyte-derived macrophages differentiated with GM-CSF (Granulocyte-Macrophage Colony-Stimulating Factor); MMØ, monocyte-derived macrophages differentiated with M-CSF (Macrophage Colony-Stimulating Factor); M1, pro-inflammatory macrophages activated with GM-CSF/ LPS/ INF-γ; M2, anti-inflammatory macrophages activated with M-CSF/ IL-4.

Supplementary Table 4: Differentially abundant proteins (DAPs) of cMØ relative to Mo and their cellular location. cMØ, monocyte-derived macrophages differentiated without the addition

of exogenous growth factors such as M-CSF or GM-CSF and Mo, Monocytes at 3 hours. Differentially abundant proteins (DAPs) were identified using a threshold of false discovery rate (FDR, q-value) ≤ 0.05 and absolute fold change ≥ 2. Red-highlighted cells indicate shared differentially abundant proteins (DAPs) between the cell lysate (CYTO) and secretome (SEC) compartments in cMØ relative to monocytes, whereas non-highlighted cells represent compartment-specific DAPs unique to either the whole cell lysate or secretome.

Supplementary Table 5: Differentially abundant proteins (DAPs) of GMØ relative to Mo and their cellular location. GMØ, monocyte-derived macrophages differentiated with GM-CSF (Granulocyte-Macrophage Colony-Stimulating Factor) and Mo, Monocytes at 3 hours. Differentially abundant proteins (DAPs) were identified using a threshold of false discovery rate (FDR, q-value) ≤ 0.05 and absolute fold change ≥ 2. Red-highlighted cells indicate shared differentially abundant proteins (DAPs) between the cell lysate (CYTO) and secretome (SEC) compartments in GMØ relative to monocytes, whereas non-highlighted cells represent compartment-specific DAPs unique to either the whole cell lysate or secretome.

Supplementary Table 6: Differentially abundant proteins (DAPs) of MMØ relative to Mo and their cellular location. MMØ, monocyte-derived macrophages differentiated with M-CSF (Macrophage Colony-Stimulating Factor) and Mo, Monocytes at 3 hours. Differentially abundant proteins (DAPs) were identified using a threshold of false discovery rate (FDR, q-value) ≤ 0.05 and absolute fold change ≥ 2. Red-highlighted cells indicate shared differentially abundant proteins (DAPs) between the cell lysate (CYTO) and secretome (SEC) compartments in MMØ relative to monocytes, whereas non-highlighted cells represent compartment-specific DAPs unique to either the whole cell lysate or secretome.

Supplementary Table 7: Shared and unique differentially abundant proteins (DAPs) between MMØ and GMØ relative to Mo and their cellular location. GMØ, monocyte-derived macrophages differentiated with GM-CSF (Granulocyte-Macrophage Colony-Stimulating Factor), MMØ, monocyte-derived macrophages differentiated with M-CSF (Macrophage Colony-Stimulating Factor) and Mo, Monocytes at 3 hours. Differentially abundant proteins (DAPs) were identified using a threshold of false discovery rate (FDR, q-value) ≤ 0.05 and absolute fold change ≥ 2. Red-highlighted cells indicate shared differentially abundant proteins (DAPs) between GMØ and MMØ relative to Mo, whereas non-highlighted cells denote DAPs that are unique to either the GMØ or MMØ subtype. “CYTO” denotes proteins identified in whole-cell lysates, and “SEC” refers to those detected in the secretome.

Supplementary Table 8: Integrated comparative profiling of differentially abundant proteins (DAPs) across monocytes and macrophage subtypes. Analyses were conducted for Mo monocytes (3-hour time point) relative to GMØ (GM-CSF–differentiated macrophages), MMØ (M-CSF–differentiated macrophages), M1 (pro-inflammatory macrophages activated with GM-

CSF, LPS, and IFN-γ), and M2 (anti-inflammatory macrophages activated with M-CSF and IL-4); as well as for M1 and M2 relative to all other subtypes. The table delineates shared and uniquely regulated DAPs between comparisons, with subcellular localization indicated as either cell lysate or secretome. Differential regulation is denoted as “U” for significantly upregulated and “D” for significantly downregulated proteins (false discovery rate [FDR] ≤ 0.05 and absolute fold change ≥ 2).

Supplementary Table 9: Differentially abundant proteins (DAPs) of MMØ relative to GMØ and their cellular location. GMØ, monocyte-derived macrophages differentiated with GM-CSF (Granulocyte-Macrophage Colony-Stimulating Factor) and MMØ, monocyte-derived macrophages differentiated with M-CSF (Macrophage Colony-Stimulating Factor). Differentially abundant proteins (DAPs) were identified using a threshold of false discovery rate (FDR, q-value) ≤ 0.05 and absolute fold change ≥ 2. Red-highlighted cells indicate shared differentially abundant proteins (DAPs) between the cell lysate (CYTO) and secretome (SEC) compartments in MMØ relative to GMØ, whereas non-highlighted cells represent compartment-specific DAPs unique to either the whole cell lysate or secretome.

Supplementary Table 10: Comparative analysis of differentially abundant proteins (DAPs) uniquely distinguishing GMØ from Mo, cMØ, MMØ, M1, and M2, and those exclusively differentiating MMØ relative to Mo, cMØ, GMØ, M1, and M2. Mo, Monocytes at 3 hours; cMØ, monocyte-derived macrophages differentiated without the addition of exogenous growth factors such as M-CSF or GM-CSF.; GMØ, monocyte-derived macrophages differentiated with GM-CSF (Granulocyte-Macrophage Colony-Stimulating Factor); MMØ, monocyte-derived macrophages differentiated with M-CSF (Macrophage Colony-Stimulating Factor); M1, pro-inflammatory macrophages activated with GM-CSF/ LPS/ INF-γ; M2, anti-inflammatory macrophages activated with M-CSF/ IL-4. All marker proteins shown were differentially abundant with a false discovery rate (FDR, q-value) < 0.001.

Supplementary Table 11: Comparative functional enrichment analysis of GMØ (monocyte-derived macrophages differentiated with GM-CSF) and MMØ (monocyte-derived macrophages differentiated with M-CSF) relative to Mo (monocytes at 3 hours). Functional enrichment analysis was performed using the StringApp (STRING database) within Cytoscape (version 3.10.3)[1], focusing on Gene Ontology (GO) Biological Process (BP) terms[2,3]. Red-highlighted cells indicate Gene Ontology Biological Process terms shared between GMØ and MMØ compared to Mo, whereas non-highlighted cells represent subtype-specific enrichment.

Supplementary Table 12: Enrichment map analysis of differentially abundant proteins (DAPs) in cMØ compared to Mo (monocytes at 3 hours), presenting significantly enriched Gene Ontology biological process (GO-BP) terms and canonical pathways. Functional enrichment analysis was performed using the StringApp (STRING database) within Cytoscape (version

3.10.3)[1], focusing on Gene Ontology (GO) Biological Process (BP) terms and canonical pathways from the KEGG and Reactome databases[2–6]. Enriched GO terms were clustered as functionally coherent groups using the EnrichmentMap plugin[7]. Statistical significance was determined based on a false discovery rate (FDR) threshold of < 0.05. cMØ, monocyte-derived macrophages differentiated without the addition of exogenous growth factors such as M-CSF or GM-CSF and Mo, Monocytes at 3 hours.

Supplementary Table 13: Enrichment map analysis of differentially abundant proteins (DAPs) in GMØ compared to Mo (monocytes at 3 hours), presenting significantly enriched Gene Ontology biological process (GO-BP) terms and canonical pathways. Functional enrichment analysis was performed using the StringApp (STRING database) within Cytoscape (version 3.10.3) [1], focusing on Gene Ontology (GO) Biological Process (BP) terms and canonical pathways from the KEGG and Reactome databases [2–6]. Enriched GO terms were clustered as functionally coherent groups using the EnrichmentMap plugin [7]. Statistical significance was determined based on a false discovery rate (FDR) threshold of < 0.05. GMØ, monocyte-derived macrophages differentiated with GM-CSF (Granulocyte-Macrophage Colony-Stimulating Factor) and Mo, Monocytes at 3 hours.

Supplementary Table 14: Enrichment map analysis of differentially abundant proteins (DAPs) in MMØ compared to Mo (monocytes at 3 hours), presenting significantly enriched Gene Ontology biological process (GO-BP) terms and canonical pathways. Functional enrichment analysis was performed using the StringApp (STRING database) within Cytoscape (version 3.10.3) [1], focusing on Gene Ontology (GO) Biological Process (BP) terms and canonical pathways from the KEGG and Reactome databases [2–6]. Enriched GO terms were clustered as functionally coherent groups using the EnrichmentMap plugin [7]. Statistical significance was determined based on a false discovery rate (FDR) threshold of < 0.05. MMØ, monocyte-derived macrophages differentiated with M-CSF (Macrophage Colony-Stimulating Factor) and Mo, Monocytes at 3 hours.

Supplementary Table 15: Differentially abundant proteins (DAPs) of M1 relative to GMØ and their cellular location. M1, pro-inflammatory macrophages activated with GM-CSF/ LPS/ INF-γ and GMØ, monocyte-derived macrophages differentiated with GM-CSF (Granulocyte-Macrophage Colony-Stimulating Factor). Differentially abundant proteins (DAPs) were identified using a threshold of false discovery rate (FDR, q-value) ≤ 0.05 and absolute fold change ≥ 2. Red-highlighted cells indicate shared differentially abundant proteins (DAPs) between the cell lysate (CYTO) and secretome (SEC) compartments in M1 relative to GMØ, whereas non-highlighted cells represent compartment-specific DAPs unique to either the whole cell lysate or secretome.

Supplementary Table 16: Differentially abundant proteins (DAPs) of M1 relative to Mo and their cellular location. M1, pro-inflammatory macrophages activated with GM-CSF/ LPS/ INF-γ and Mo, Monocytes at 3 hours. Differentially abundant proteins (DAPs) were identified using a threshold of false discovery rate (FDR, q-value) ≤ 0.05 and absolute fold change ≥ 2. Red-highlighted cells indicate shared differentially abundant proteins (DAPs) between the cell lysate (CYTO) and secretome (SEC) compartments in M1 relative to Mo, whereas non-highlighted cells represent compartment-specific DAPs unique to either the whole cell lysate or secretome.

Supplementary Table 17: Differentially abundant proteins (DAPs) of M2 relative to MMØ and their cellular location. M2, anti-inflammatory macrophages activated with M-CSF/ IL-4 and MMØ, monocyte-derived macrophages differentiated with M-CSF (Macrophage Colony-Stimulating Factor). Differentially abundant proteins (DAPs) were identified using a threshold of false discovery rate (FDR, q-value) ≤ 0.05 and absolute fold change ≥ 2. Red-highlighted cells indicate shared differentially abundant proteins (DAPs) between the cell lysate (CYTO) and secretome (SEC) compartments in M2 relative to MMØ, whereas non-highlighted cells represent compartment-specific DAPs unique to either the whole cell lysate or secretome.

Supplementary Table 18: Differentially abundant proteins (DAPs) of M2 relative to Mo and their cellular location. M2, anti-inflammatory macrophages activated with M-CSF/ IL-4 and Mo, Monocytes at 3 hours. Differentially abundant proteins (DAPs) were identified using a threshold of false discovery rate (FDR, q-value) ≤ 0.05 and absolute fold change ≥ 2. Red-highlighted cells indicate shared differentially abundant proteins (DAPs) between the cell lysate and secretome compartments in M2 relative to Mo, whereas non-highlighted cells represent compartment-specific DAPs unique to either the whole cell lysate (CYTO) or secretome (SEC).

Supplementary Table 19: Shared and unique differentially abundant proteins (DAPs) between M1 and M2 relative to Mo and their cellular location. M1, pro-inflammatory macrophages activated with GM-CSF/ LPS/ INF-γ; M2, anti-inflammatory macrophages activated with M-CSF/ IL-4 and Mo, Monocytes at 3 hours. Differentially abundant proteins (DAPs) were identified using a threshold of false discovery rate (FDR, q-value) ≤ 0.05 and absolute fold change ≥ 2. Red-highlighted cells indicate shared differentially abundant proteins (DAPs) between M1 and M2 relative to Mo, whereas non-highlighted cells denote DAPs that are unique to either the M1 or M2 subtype. “CYTO” denotes proteins identified in whole-cell lysates, and “SEC” refers to those detected in the secretome.

Supplementary Table 20: Differentially abundant proteins (DAPs) of M1 relative to M2 and their cellular location. M1, pro-inflammatory macrophages activated with GM-CSF/ LPS/ INF-γ and M2, anti-inflammatory macrophages activated with M-CSF/ IL-4. Differentially abundant proteins (DAPs) were identified using a threshold of false discovery rate (FDR, q-value) ≤ 0.05 and absolute fold change ≥ 2. Red-highlighted cells indicate shared differentially abundant

proteins (DAPs) between the cell lysate (CYTO) and secretome (SEC)compartments in M1 relative to M2, whereas non-highlighted cells represent compartment-specific DAPs unique to either the whole cell lysate or secretome.

Supplementary Table 21: List of 20 M1-specific and 57 M2-specific differentially abundant proteins (DAPs) that uniquely distinguish M1 and M2 macrophages, respectively, from Mo (monocytes at 3 hours), GMØ (macrophages differentiated with GM-CSF), MMØ (macrophages differentiated with M-CSF), and the opposing polarized subtype. M1, pro-inflammatory macrophages activated with GM-CSF/ LPS/ INF-γ; M2, anti-inflammatory macrophages activated with M-CSF/ IL-4. All proteins listed were identified as significantly differentially abundant with a false discovery rate (FDR, q-value) < 0.001.

Supplementary Table 22: Scores of potential hub protein analysis for M1- and M2-specific differentially abundant proteins (DAPs) generated using CytoHubba in Cytoscape[8]. Hub proteins were ranked based on five topological scoring algorithms: Maximal Clique Centrality (MCC), Degree of Connectivity, Edge Percolated Component (EPC), Closeness, and Radiality. M1, pro-inflammatory macrophages activated with GM-CSF/ LPS/ INF-γ; M2, anti-inflammatory macrophages activated with M-CSF/ IL-4.

Supplementary Table 23: Comparative functional enrichment analysis of M1 and M2 relative to monocytes. Functional enrichment analysis was performed using the StringApp (STRING database) within Cytoscape (version 3.10.3)[1], focusing on Gene Ontology (GO) Biological Process (BP) terms[2,3]. Red-highlighted cells indicate Gene Ontology Biological Process terms shared between M1 and M2 compared to Mo, whereas non-highlighted cells represent subtype-specific enrichment. M1, pro-inflammatory macrophages activated with GM-CSF/ LPS/ INF-γ; M2, anti-inflammatory macrophages activated with M-CSF/ IL-4, and Mo, Monocytes at 3 hours.

Supplementary Table 24: Comparative functional enrichment analysis of M1 and M2 relative to respective MØ (monocyte derived macrophage). Functional enrichment analysis was performed using the StringApp (STRING database) within Cytoscape (version 3.10.3)[1], focusing on Gene Ontology (GO) Biological Process (BP) terms[2,3]. Red-highlighted cells indicate Gene Ontology Biological Process terms shared between M1 and M2 compared to MØ, whereas non-highlighted cells represent subtype-specific enrichment. M1, pro-inflammatory macrophages activated with GM-CSF/ LPS/ INF-γ and M2, anti-inflammatory macrophages activated with M-CSF/ IL-4.

Supplementary Table 25: Enrichment map analysis of differentially abundant proteins (DAPs) in M1 compared to GMØ, presenting significantly enriched Gene Ontology biological process (GO-BP) terms and canonical pathways. Functional enrichment analysis was performed

using the StringApp (STRING database) within Cytoscape (version 3.10.3) [1], focusing on Gene Ontology (GO) Biological Process (BP) terms and canonical pathways from the KEGG and Reactome databases [2–6]. Enriched GO terms were clustered as functionally coherent groups using the EnrichmentMap plugin [7]. Statistical significance was determined based on a false discovery rate (FDR) threshold of < 0.05. M1, pro-inflammatory macrophages activated with GM-CSF/ LPS/ INF-γ and GMØ, monocyte-derived macrophages differentiated with GM-CSF (Granulocyte-Macrophage Colony-Stimulating Factor).

Supplementary Table 26: Enrichment map analysis of differentially abundant proteins (DAPs) in M2 compared to MMØ, presenting significantly enriched Gene Ontology biological process (GO-BP) terms and canonical pathways. Functional enrichment analysis was performed using the StringApp (STRING database) within Cytoscape (version 3.10.3) [1], focusing on Gene Ontology (GO) Biological Process (BP) terms and canonical pathways from the KEGG and Reactome databases [2–6]. Enriched GO terms were clustered as functionally coherent groups using the EnrichmentMap plugin [7]. Statistical significance was determined based on a false discovery rate (FDR) threshold of < 0.05. M2, anti-inflammatory macrophages activated with M-CSF/ IL-4 and MMØ, monocyte-derived macrophages differentiated with M-CSF (Macrophage Colony-Stimulating Factor).

Supplementary Table 27: Enrichment map analysis of M1-specific differentially abundant proteins (DAPs; n = 20), highlighting significantly enriched Gene Ontology Biological Process (GO-BP) terms and canonical pathways. Functional enrichment analysis was performed using the StringApp (STRING database) within Cytoscape (version 3.10.3) [1], focusing on Gene Ontology (GO) Biological Process (BP) terms and canonical pathways from the KEGG and Reactome databases [2–6]. Enriched GO terms were clustered as functionally coherent groups using the EnrichmentMap plugin [7]. Statistical significance was assessed using a false discovery rate (FDR) threshold of < 0.05. M1 refers to pro-inflammatory macrophages stimulated with GM-CSF, LPS, and IFN-γ.

Supplementary Table 28: Enrichment map analysis of M2-specific differentially abundant proteins (DAPs; n = 57), highlighting significantly enriched Gene Ontology Biological Process (GO-BP) terms and canonical pathways. Functional enrichment analysis was performed using the StringApp (STRING database) within Cytoscape (version 3.10.3) [1], focusing on Gene Ontology (GO) Biological Process (BP) terms and canonical pathways from the KEGG and Reactome databases [2–6]. Enriched GO terms were clustered as functionally coherent groups using the EnrichmentMap plugin [7]. Statistical significance was evaluated using a false discovery rate (FDR) threshold of < 0.05. M2 denotes anti-inflammatory macrophages stimulated with M-CSF and IL-4.

References

1. Shannon, P. *et al.* Cytoscape: A Software Environment for Integrated Models of Biomolecular Interaction Networks. *Genome Res.* **13,** 2498–2504 (2003).

2. Szklarczyk, D. *et al.* STRING v11: protein–protein association networks with increased coverage, supporting functional discovery in genome-wide experimental datasets. *Nucleic Acids Res.* **47,** gky1131 (2018).

3. Consortium, T. G. O. *et al.* The Gene Ontology resource: enriching a GOld mine. *Nucleic Acids Res.* **49,** D325–D334 (2020).

4. Kanehisa, M., Furumichi, M., Sato, Y., Matsuura, Y. & Ishiguro-Watanabe, M. KEGG: biological systems database as a model of the real world. *Nucleic Acids Res.* **53,** D672–D677 (2024).

5. Milacic, M. *et al.* The Reactome Pathway Knowledgebase 2024. *Nucleic Acids Res.* **52,** D672–D678 (2023).

6. Kanehisa, M. Toward understanding the origin and evolution of cellular organisms. *Protein Sci.* **28,** 1947–1951 (2019).

7. Merico, D., Isserlin, R., Stueker, O., Emili, A. & Bader, G. D. Enrichment Map: A Network-Based Method for Gene-Set Enrichment Visualization and Interpretation. *PLoS ONE* **5,** e13984 (2010).

8. Chin, C.-H. *et al.* cytoHubba: identifying hub objects and sub-networks from complex interactome. *BMC Syst. Biol.* **8,** S11 (2014).

Supplementary Table 1: Comparative proteomic analysis of 42 identified Cluster of Differentiation (CD) antigens across monocytes and macrophage subtypes.

CD markers	Comparison	Mo_cMØ	Mo_GMØ	Mo_M1	Mo_MMØ	Mo_M2	cMØ_GMØ	cMØ_M1	cMØ_MMØ	cMØ_M2	GMØ_M1	GMØ_MMØ	GMØ_M2	M1_MMØ	M1_M2	MMØ_M2
CD1b-2	Adjusted P Value					<0.0001				<0.0001			<0.0001		<0.0001	<0.0001
	Mean Difference	0	0	0	0	-14.59	0	0	0	-14.59	0	0	-14.59	0	-14.59	-14.59
	Summary					****				****			****		****	****
CD1E	Adjusted P Value	0.0025	0.0003	<0.0001	<0.0001	<0.0001	>0.9999	0.962	0.8908	0.2623	0.9799	0.9644	0.191	0.9953	0.0136	0.001
	Mean Difference	-13.04	-12.82	-12.51	-12.39	-14.87	0.226	0.5353	0.6572	-1.83	0.3093	0.4312	-2.056	0.1219	-2.365	-2.487
	Summary	**	***	****	****	****	ns	ns	ns	ns	ns	ns	ns	ns	*	***
CD9	Adjusted P Value	0.8866	>0.9999	0.9914	0.9934	0.4846	0.3209	0.4738	0.0152	0.019	0.9962	0.9624	0.224	0.9992	0.3957	0.0402
	Mean Difference	-0.7574	0.08095	0.219	0.3633	1.777	0.8383	0.9764	1.121	2.534	0.138	0.2824	1.696	0.1443	1.558	1.414
	Summary	ns	ns	ns	ns	ns	ns	ns	*	*	ns	ns	ns	ns	ns	*
CD11A	Adjusted P Value	0.2595	0.8147	0.9979	0.007	0.05	0.9666	0.913	0.0213	0.566	0.7597	0.0289	0.3226	0.0748	0.3383	0.0438
	Mean Difference	-0.6329	-0.3581	-0.1447	-1.901	-1.054	0.2748	0.4882	-1.268	-0.4207	0.2134	-1.543	-0.6955	-1.756	-0.9089	0.8471
	Summary	ns	ns	ns	**	*	ns	ns	*	ns	ns	*	ns	ns	ns	*
CD11B	Adjusted P Value	0.5021	0.4104	0.4018	0.4856	0.5733	0.4246	0.3205	0.4788	0.9854	>0.9999	0.9744	0.1417	0.9736	0.2213	0.0448
	Mean Difference	-2.098	-2.74	-2.743	-2.615	-2.237	-0.6428	-0.6453	-0.5178	-0.1399	-0.002475	0.125	0.5029	0.1275	0.5054	0.3779
	Summary	ns	ns	ns	ns	ns	ns	ns	ns	ns	ns	ns	ns	ns	ns	*
CD11B Shed	Adjusted P Value	<0.0001	0.0017	0.1022	0.0187	0.0041	<0.0001	0.0002	0.0006	0.0003	0.1845	0.2823	0.0224	0.2029	0.0295	0.0008
	Mean Difference	9.732	-2.313	-1.364	-3.888	-6.735	-12.05	-11.1	-13.62	-16.47	0.9496	-1.575	-4.422	-2.524	-5.372	-2.847
	Summary	****	**	ns	*	**	****	***	***	***	ns	ns	*	ns	*	***
CD11C	Adjusted P Value	0.447	0.6605	0.1129	0.423	0.0249	0.9987	0.6546	0.7863	0.9149	0.0206	0.884	0.7011	0.161	0.7415	0.0937
	Mean Difference	-1.026	-0.8423	-1.934	-0.2675	-1.429	0.1832	-0.9082	0.758	-0.404	-1.091	0.5748	-0.5872	1.666	0.5043	-1.162
	Summary	ns	ns	ns	ns	*	ns	ns	ns	ns	*	ns	ns	ns	ns	ns
CD14	Adjusted P Value	0.0148	0.2375	0.1057	0.9985	0.7707	0.1057	0.8762	0.3582	0.6704	0.5441	0.8569	>0.9999	0.5027	0.9269	0.9135
	Mean Difference	-1.645	-0.7113	-1.42	0.3246	-0.717	0.9341	0.2252	1.97	0.9283	-0.7089	1.036	-0.00575	1.745	0.7031	-1.042
	Summary	*	ns	ns	ns	ns	ns	ns	ns	ns	ns	ns	ns	ns	ns	ns
CD16A	Adjusted P Value	0.2617	0.0239	0.3065	>0.9999	0.0007	0.0339	0.8189	0.7062	0.0004	0.0328	0.0658	<0.0001	0.3429	<0.0001	0.0037
	Mean Difference	-1.216	-3.648	-1.704	0.1383	11.82	-2.432	-0.4881	1.354	13.04	1.944	3.786	15.47	1.843	13.52	11.68
	Summary	ns	*	ns	ns	***	*	ns	ns	***	*	ns	****	ns	****	**
CD18	Adjusted P Value	0.1325	0.1934	0.2624	0.1791	0.1796	0.5506	0.8839	0.3697	0.414	0.9511	0.5158	0.0201	0.4673	0.6893	0.9459
	Mean Difference	-1.802	-2.179	-2.078	-2.332	-2.274	-0.3774	-0.2759	-0.53	-0.4716	0.1015	-0.1526	-0.0942	-0.2541	-0.1957	0.0584
	Summary	ns	ns	ns	ns	ns	ns	ns	ns	ns	ns	ns	*	ns	ns	ns
CD18 Shed	Adjusted P Value	0.9702	0.9474	0.9869	0.1053	0.0657	0.1671	0.0828	<0.0001	0.0024	0.2135	0.0041	0.002	<0.0001	0.0027	0.0276
	Mean Difference	-0.6889	0.8494	-0.5498	-4.368	-6.693	1.538	0.1391	-3.679	-6.004	-1.399	-5.218	-7.542	-3.819	-6.143	-2.325
	Summary	ns	ns	ns	ns	ns	ns	ns	****	**	ns	**	**	****	**	*
CD22	Adjusted P Value	0.0028	0.0046	0.1622	<0.0001	0.0161	0.3453	0.9996	0.0014	0.3339	0.8542	<0.0001	0.2203	0.0215	0.9275	0.0142
	Mean Difference	4.88	3.582	5.282	17.4	6.875	-1.298	0.4016	12.52	1.995	1.7	13.82	3.293	12.12	1.593	-10.52
	Summary	**	**	ns	****	*	ns	ns	**	ns	ns	****	ns	*	ns	*
CD29	Adjusted P Value	0.4816	0.8232	0.8828	0.9372	0.7935	0.0233	0.1237	0.7564	0.9995	0.9996	>0.9999	0.7186	>0.9999	0.545	0.2038
	Mean Difference	-1.365	-0.8543	-0.8227	-0.8714	-1.268	0.5109	0.5425	0.4938	0.09695	0.03163	-0.01713	-0.414	-0.04875	-0.4456	-0.3968
	Summary	ns	ns	ns	ns	ns	*	ns	ns	ns	ns	ns	ns	ns	ns	ns
CD32	Adjusted P Value	0.9863	0.1536	0.1683	0.9047	0.9725	0.1012	0.0905	0.9996	>0.9999	0.8128	0.2326	0.0927	0.267	0.1132	0.3814
	Mean Difference	-0.9158	-3.821	-3.763	-1.276	-0.7767	-2.906	-2.847	-0.3602	0.139	0.0588	2.546	3.045	2.487	2.986	0.4992

Supplementary Table 1: Comparative proteomic analysis of 42 identified Cluster of Differentiation (CD) antigens across monocytes and macrophage subtypes.

CD markers	Comparison	Mo_cMØ	Mo_GMØ	Mo_M1	Mo_MMØ	Mo_M2	cMØ_GMØ	cMØ_M1	cMØ_MMØ	cMØ_M2	GMØ_M1	GMØ_MMØ	GMØ_M2	M1_MMØ	M1_M2	MMØ_M2
	Summary	ns	ns	ns	ns	ns	ns	ns	ns	ns	ns	ns	ns	ns	ns	ns
CD37	Adjusted P Value	0.0938	0.9817	0.3719	0.001	0.001	0.1282	0.997	0.0008	0.0008	0.2742	0.0005	0.0005	<0.0001	<0.0001	
	Mean Difference	-1.37	0.2687	-1.186	14.64	14.64	1.639	0.1839	16.01	16.01	-1.455	14.37	14.37	15.83	15.83	0
	Summary	ns	ns	ns	***	***	ns	ns	***	***	ns	***	***	****	****	
CD47	Adjusted P Value	0.8226	0.4594	0.2999	0.8496	>0.9999	0.2135	0.1707	0.96	0.9953	>0.9999	0.3775	0.322	0.3679	0.4147	0.874
	Mean Difference	0.248	-0.7064	-0.7182	0.8628	0.09243	-0.9544	-0.9662	0.6148	-0.1556	-0.01183	1.569	0.7988	1.581	0.8107	-0.7704
	Summary	ns	ns	ns	ns	ns	ns	ns	ns	ns	ns	ns	ns	ns	ns	ns
CD49B	Adjusted P Value	0.5061	0.9997	>0.9999	0.5001	0.9902	0.3239	0.3373	0.9984	0.3884	0.4134	0.2282	0.1494	0.2456	0.2258	0.3717
	Mean Difference	3.852	-0.2891	0.003963	4.804	0.6745	-4.141	-3.848	0.952	-3.178	0.293	5.093	0.9636	4.8	0.6706	-4.13
	Summary	ns	ns	ns	ns	ns	ns	ns	ns	ns	ns	ns	ns	ns	ns	ns
CD49D	Adjusted P Value	0.1633	<0.0001	<0.0001	<0.0001	0.7565	0.0013	0.0013	0.0013	>0.9999			0.0221		0.0221	0.0221
	Mean Difference	2.124	14.33	14.33	14.33	2.178	12.21	12.21	12.21	0.05402	0	0	-12.15	0	-12.15	-12.15
	Summary	ns	****	****	****	ns	**	**	**	ns			*		*	*
CD49E	Adjusted P Value	0.2342	0.9808	0.6972	0.0113	0.9184	0.9237	0.1198	0.9963	0.7335	0.762	0.98	>0.9999	0.1055	0.7797	0.3866
	Mean Difference	-0.9718	-0.3892	0.4303	-0.8334	-0.2641	0.5826	1.402	0.1384	0.7076	0.8195	-0.4442	0.1251	-1.264	-0.6944	0.5692
	Summary	ns	ns	ns	*	ns	ns	ns	ns	ns	ns	ns	ns	ns	ns	ns
CD51	Adjusted P Value	0.0512	0.0679	0.0159	0.0078	0.0093	0.5935	>0.9999	>0.9999	0.2653	0.4771	0.1175	0.1381	0.9995	0.5138	0.2869
	Mean Difference	-2.619	-1.745	-2.682	-2.613	-3.126	0.8746	-0.06273	0.006625	-0.5072	-0.9374	-0.868	-1.382	0.06935	-0.4444	-0.5138
	Summary	ns	ns	*	**	**	ns	ns	ns	ns	ns	ns	ns	ns	ns	ns
CD55	Adjusted P Value	0.0017	0.187	0.0267	0.0965	0.2542	0.4289	0.4007	0.8308	0.9906	0.5795	0.4456	0.7655	0.4672	0.8341	0.9678
	Mean Difference	2.343	1.324	1.636	3.157	2.811	-1.019	-0.7063	0.8149	0.4688	0.3128	1.834	1.488	1.521	1.175	-0.3461
	Summary	**	ns	*	ns	ns	ns	ns	ns	ns	ns	ns	ns	ns	ns	ns
CD58	Adjusted P Value	0.9745	0.9689	0.9358	0.993	0.972	0.9857	0.4542	0.0452	0.0655	0.2196	0.0244	0.0428	0.0077	0.0219	0.2934
	Mean Difference	-0.866	-0.9446	-1.146	0.7011	1.068	-0.07862	-0.2802	1.567	1.934	-0.2016	1.646	2.012	1.847	2.214	0.3664
	Summary	ns	ns	ns	ns	ns	ns	ns	*	ns	ns	*	*	**	*	ns
CD61	Adjusted P Value	0.9199	0.8487	0.6594	0.4332	0.1152	0.9988	0.9996	0.3876	0.4285	0.9947	0.6738	0.4947	0.4094	0.2293	0.9225
	Mean Difference	1.181	1.371	1.001	4.182	5.979	0.1898	-0.1804	3.001	4.798	-0.3703	2.811	4.608	3.182	4.979	1.797
	Summary	ns	ns	ns	ns	ns	ns	ns	ns	ns	ns	ns	ns	ns	ns	ns
CD64	Adjusted P Value	<0.0001	<0.0001	<0.0001	<0.0001	0.0018	0.862	0.9999	0.3093	0.2075	0.9676	0.3042	0.2115	0.6802	0.3498	0.1746
	Mean Difference	-17.17	-17.4	-17.24	-16.36	-14.48	-0.2309	-0.0666	0.8154	2.697	0.1643	1.046	2.928	0.882	2.763	1.881
	Summary	****	****	****	****	**	ns	ns	ns	ns	ns	ns	ns	ns	ns	ns
CD68	Adjusted P Value	0.0129	0.0019	0.0034	0.0012	0.0118	0.8343	0.8052	0.7481	0.3352	0.9192	0.5755	0.3175	0.8832	0.5051	0.4518
	Mean Difference	-7.059	-6.544	-6.365	-6.185	-4.872	0.5147	0.6937	0.874	2.186	0.1789	0.3592	1.672	0.1803	1.493	1.313
	Summary	*	**	**	**	*	ns	ns	ns	ns	ns	ns	ns	ns	ns	ns
CD72	Adjusted P Value	0.3333	0.9452	0.2656	0.0007	0.8354	<0.0001	0.7803	0.0021	0.524	0.4885	0.0027	0.7775	0.0003	0.4955	0.0448
	Mean Difference	-2.553	-0.8201	-1.867	14.62	2.839	1.733	0.6864	17.17	5.392	-1.047	15.44	3.659	16.48	4.706	-11.78
	Summary	ns	ns	ns	***	ns	****	ns	**	ns	ns	**	ns	***	ns	*
CD79B	Adjusted P Value	0.0036	0.0036	0.0036	0.0036	0.0036										
	Mean Difference	14.77	14.77	14.77	14.77	14.77	0	0	0	0	0	0	0	0	0	0
	Summary	**	**	**	**	**										
CD80	Adjusted P Value		<0.0001	<0.0001	0.0113		<0.0001	<0.0001	0.0113		>0.9999	0.6118	<0.0001	0.5549	<0.0001	0.0113

Supplementary Table 1: Comparative proteomic analysis of 42 identified Cluster of Differentiation (CD) antigens across monocytes and macrophage subtypes.

CD markers	Comparison	Mo_cMØ	Mo_GMØ	Mo_M1	Mo_MMØ	Mo_M2	cMØ_GMØ	cMØ_M1	cMØ_MMØ	cMØ_M2	GMØ_M1	GMØ_MMØ	GMØ_M2	M1_MMØ	M1_M2	MMØ_M2
	Mean Difference	0	-13.29	-13.28	-11.23	0	-13.29	-13.28	-11.23	0	0.00098	2.055	13.29	2.055	13.28	11.23
	Summary		****	****	*		****	****	*		ns	ns	****	ns	****	*
CD84	Adjusted P Value	0.3001	0.3222	0.2112	0.6955	0.4169	0.9915	0.4401	0.8316	>0.9999	0.5485	0.8851	0.9997	0.1367	0.7236	0.6255
	Mean Difference	-2.666	-2.561	-3.544	-1.882	-2.656	0.1057	-0.8775	0.7839	0.0101	-0.9832	0.6782	-0.0956	1.661	0.8876	-0.7738
	Summary	ns	ns	ns	ns	ns	ns	ns	ns	ns	ns	ns	ns	ns	ns	ns
CD86	Adjusted P Value	<0.0001	<0.0001	0.0004	0.0009		0.3544	0.405	0.4022	<0.0001	0.391	0.4291	<0.0001	0.3866	0.0004	0.0009
	Mean Difference	-15.7	-15.47	-16.95	-14.51	0	0.2374	-1.245	1.192	15.7	-1.482	0.955	15.47	2.437	16.95	14.51
	Summary	****	****	***	***		ns	ns	ns	****	ns	ns	****	ns	***	***
CD97	Adjusted P Value	>0.9999	0.024	0.0027	0.0273	0.0057	0.6586	0.6764	0.4407	0.4316	0.9922	0.1126	0.0851	0.1568	0.0218	0.8133
	Mean Difference	-0.000183	-1.031	-1.096	-1.796	-1.627	-1.03	-1.096	-1.796	-1.627	-0.06585	-0.7651	-0.5963	-0.6993	-0.5304	0.1688
	Summary	ns	*	**	*	**	ns	ns	ns	ns	ns	ns	ns	ns	*	ns
CD99	Adjusted P Value	0.0499	0.3633	0.0404	0.913	0.4696	0.988	0.2879	0.1168	0.1368	0.7302	0.1131	0.4569	0.0304	0.0321	0.3645
	Mean Difference	-3.763	-3.217	-4.346	-1.079	-2.014	0.546	-0.5833	2.684	1.749	-1.129	2.138	1.203	3.267	2.332	-0.9349
	Summary	*	ns	*	ns	ns	ns	ns	ns	ns	ns	ns	ns	*	*	ns
CD107A	Adjusted P Value	0.139	0.1795	0.2135	0.0762	0.0824	0.9303	0.9843	0.2318	0.4996	0.2289	0.6116	0.879	0.0012	0.0161	0.7441
	Mean Difference	-2.145	-2.562	-1.963	-2.965	-2.827	-0.4169	0.1817	-0.8202	-0.682	0.5986	-0.4032	-0.265	-1.002	-0.8637	0.1382
	Summary	ns	ns	ns	ns	ns	ns	ns	ns	ns	ns	ns	ns	**	*	ns
CD107B	Adjusted P Value	0.0259	0.0636	0.0864	0.0247	0.0306	0.9283	0.9154	0.0305	0.3617	0.1317	0.5656	0.8115	0.0566	0.0472	0.9961
	Mean Difference	-1.777	-1.941	-1.635	-2.185	-2.133	-0.1635	0.1425	-0.4073	-0.3555	0.306	-0.2438	-0.192	-0.5498	-0.498	0.05183
	Summary	*	ns	ns	*	*	ns	ns	*	ns	ns	ns	ns	ns	*	ns
CD109	Adjusted P Value	0.2141	0.3283	0.0114	0.1176	0.0011	0.9636	0.8792	0.7095	0.0024	0.5794	0.0804	<0.0001	0.802	0.0013	0.0032
	Mean Difference	2.847	2.235	3.467	4.822	15.42	-0.6116	0.6207	1.975	12.57	1.232	2.587	13.18	1.354	11.95	10.6
	Summary	ns	ns	*	ns	**	ns	ns	ns	**	ns	ns	****	ns	**	**
CD115	Adjusted P Value	0.0012	0.0002	<0.0001	<0.0001	<0.0001	0.9936	0.8498	0.3883	0.9998	0.8354	0.6042	0.983	0.2293	0.2676	0.5984
	Mean Difference	-13.52	-13.08	-12.52	-14.29	-13.34	0.4427	0.9952	-0.7676	0.1781	0.5525	-1.21	-0.2647	-1.763	-0.8172	0.9457
	Summary	**	***	****	****	****	ns	ns	ns	ns	ns	ns	ns	ns	ns	ns
CD115 Shed	Adjusted P Value	0.1585	0.075	0.2164	0.0464	0.0108	0.9535	>0.9999	0.1622	0.0788	0.9891	0.0268	0.0047	0.2621	0.0275	0.3565
	Mean Difference	-2.509	-2.931	-2.619	-4.808	-5.871	-0.4225	-0.1102	-2.3	-3.362	0.3122	-1.877	-2.94	-2.189	-3.252	-1.063
	Summary	ns	ns	ns	*	*	ns	ns	ns	ns	ns	*	**	ns	*	ns
CD163	Adjusted P Value	0.0076	0.003	0.0158	0.0077	0.0088	0.2767	0.9402	>0.9999	0.3038	0.2143	0.3615	0.163	0.9617	>0.9999	0.0159
	Mean Difference	-7.587	-8.272	-7.258	-7.596	-7.331	-0.6859	0.3287	-0.009125	0.2558	1.015	0.6768	0.9417	-0.3378	-0.07293	0.2649
	Summary	**	**	*	**	**	ns	ns	ns	ns	ns	ns	ns	ns	ns	*
CD163 Shed	Adjusted P Value	0.992	0.9896	0.7115	0.53	0.1417	0.9962	0.1307	0.2067	0.0126	0.9203	0.7221	0.3246	0.9844	0.0939	0.2761
	Mean Difference	-0.5896	-0.9017	-1.802	-2.194	-3.472	-0.3122	-1.212	-1.605	-2.882	-0.9002	-1.293	-2.57	-0.3924	-1.67	-1.277
	Summary	ns	ns	ns	ns	ns	ns	ns	ns	*	ns	ns	ns	ns	ns	ns
CD180	Adjusted P Value	0.9205	0.9511	0.7053	0.4087	0.0053	>0.9999	0.6676	0.2099	<0.0001	0.6207	0.1269	<0.0001	0.0452	<0.0001	0.0011
	Mean Difference	1.154	1.218	2.111	3.882	17.14	0.06395	0.9563	2.728	15.99	0.8923	2.664	15.92	1.772	15.03	13.26
	Summary	ns	ns	ns	ns	**	ns	ns	ns	****	ns	ns	****	*	****	**
CD200R1	Adjusted P Value	<0.0001	0.0001	<0.0001	0.0013	<0.0001	0.7859	0.9557	0.9998	0.9998	0.9936	0.9998	0.9965	>0.9999	>0.9999	>0.9999
	Mean Difference	-14.56	-14.15	-14.38	-14.38	-14.44	0.4092	0.1772	0.1819	0.1181	-0.232	-0.2273	-0.2911	0.004672	-0.05916	-0.06383
	Summary	****	***	****	**	****	ns	ns	ns	ns	ns	ns	ns	ns	ns	ns

Supplementary Table 1: Comparative proteomic analysis of 42 identified Cluster of Differentiation (CD) antigens across monocytes and macrophage subtypes.

CD markers	Comparison	Mo_cMØ	Mo_GMØ	Mo_M1	Mo_MMØ	Mo_M2	cMØ_GMØ	cMØ_M1	cMØ_MMØ	cMØ_M2	GMØ_M1	GMØ_MMØ	GMØ_M2	M1_MMØ	M1_M2	MMØ_M2
CD204	Adjusted P Value	<0.0001	<0.0001	<0.0001	<0.0001	<0.0001	0.9284	0.9935	0.1438	0.9991	0.1569	0.5558	0.8268	0.1346	0.9999	0.023
	Mean Difference	-18.2	-18.35	-18.11	-18.65	-18.14	-0.1586	0.089	-0.451	0.05818	0.2477	-0.2924	0.2168	-0.54	-0.03083	0.5092
	Summary	****	****	****	****	****	ns	ns	ns	ns	ns	ns	ns	ns	ns	*
CD206	Adjusted P Value	0.0015	0.0011	<0.0001	0.003	0.0018	0.0027	0.0422	0.9662	0.3767	0.8998	0.1602	0.3647	0.0837	0.4411	0.1241
	Mean Difference	-6.021	-7.479	-7.267	-5.71	-6.644	-1.458	-1.246	0.3115	-0.6227	0.2121	1.769	0.8352	1.557	0.6231	-0.9341
	Summary	**	**	****	**	**	**	*	ns	ns	ns	ns	ns	ns	ns	ns
CD206 Shed	Adjusted P Value		0.0011	0.0015	0.0033	0.0006	0.0011	0.0015	0.0033	0.0006	0.8256	0.4578	0.0038	0.9768	0.0531	0.0087
	Mean Difference	0	-11.86	-12.38	-13.03	-16.58	-11.86	-12.38	-13.03	-16.58	-0.5201	-1.167	-4.723	-0.6466	-4.202	-3.556
	Summary		**	**	**	***	**	**	**	***	ns	ns	**	ns	*	**
CD274	Adjusted P Value	0.0003	0.0002	<0.0001	0.0008		0.3049	0.2322	0.1506	0.0003	0.0194	0.2003	0.0002	0.0222	<0.0001	0.0008
	Mean Difference	-14.77	-14.04	-16.24	-12.76	0	0.7292	-1.467	2.012	14.77	-2.196	1.282	14.04	3.479	16.24	12.76
	Summary	***	***	****	***		ns	ns	ns	***	*	ns	***	*	****	***
CD302	Adjusted P Value	0.2735	0.0868	0.1264	0.1623	0.119	0.3409	0.2307	0.1996	0.8791	0.6004	0.5993	0.2447	0.9827	0.8292	0.7825
	Mean Difference	-3.263	-4.467	-3.866	-3.988	-3.633	-1.204	-0.6035	-0.7258	-0.3701	0.6007	0.4784	0.8341	-0.1223	0.2334	0.3557
	Summary	ns	ns	ns	ns	ns	ns	ns	ns	ns	ns	ns	ns	ns	ns	ns
CD369	Adjusted P Value	0.0008	0.0012	0.0024	0.0053		0.999	0.1278	0.9717	0.0008	0.3185	0.9895	0.0012	0.1782	0.0024	0.0053
	Mean Difference	-11.68	-11.92	-15.69	-12.59	0	-0.2434	-4.008	-0.916	11.68	-3.765	-0.6726	11.92	3.092	15.69	12.59
	Summary	***	**	**	**		ns	ns	ns	***	ns	ns	**	ns	**	**

Mo, Monocytes at 3 hours; cMØ, monocyte-derived macrophages differentiated without the addition of exogenous growth factors such as M-CSF or GM-CSF.; GMØ, monocyte-derived macrophages differentiated with GM-CSF (Granulocyte-Macrophage Colony-Stimulating Factor); MMØ, monocyte-derived macrophages differentiated with M-CSF (Macrophage Colony-Stimulating Factor); M1, pro-inflammatory macrophages activated with GM-CSF/ LPS/ INF-γ; M2, anti-inflammatory macrophages activated with M-CSF/ IL-4. Asterisks indicate levels of statistical significance for CD marker expression: * adjusted P-value ≤ 0.05; ** adjusted P-value ≤ 0.01; *** adjusted P-value ≤ 0.001; ns, not significant.

Supplementary Table 2: Imaging-based comparative morphological analysis of monocytes and macrophage subtypes.

Morophological Features		GMØ_M0	MMØ_M0	M1_M0	M2_M0	cMØ_M0	MMØ_GMØ	M1_GMØ	M2_GMØ	cMØ_GMØ	M1_MMØ	M2_MMØ	cMØ_MMØ	M2_M1	cMØ_M1	cMØ_M2
CYTO_Area	Adjusted P Value	0.209	0.124	0.000	<0.0001	0.352	0.999	0.009	0.000	0.999	0.015	0.001	0.977	0.383	0.005	0.000
	Mean Difference	13003	14790	36241	46923	11035	1787	23239	33920	-1967	21452	32133	-3755	10681	-25206	-35888
	Summary	ns	ns	***	****	ns	ns	**	***	ns	*	***	ns	ns	**	***
CYTO_Perimeter	Adjusted P Value	0.0025	0.0023	<0.0001	<0.0001	0.0022	>0.9999	0.0003	0.0005	>0.9999	0.0003	0.0006	>0.9999	0.9967	0.0003	0.0006
	Mean Difference	435.2	438.8	995.3	956.4	442.1	3.623	560.1	521.2	6.855	556.5	517.5	3.231	-38.95	-553.3	-514.3
	Summary	**	**	****	****	**	ns	***	***	ns	***	***	ns	ns	***	***
CYTO_Feret's diameter	Adjusted P Value	0.00	0.00	<0.0001	<0.0001	0.00	>0.9999	<0.0001	0.00	>0.9999	<0.0001	0.00	>0.9999	0.57	<0.0001	0.00
	Mean Difference	153.7	156	352.7	309.5	153.3	2.294	199	155.7	-0.4133	196.7	153.4	-2.707	-43.24	-199.4	-156.2
	Summary	***	***	****	****	***	ns	****	***	ns	****	***	ns	ns	****	***
CYTO_Circularity	Adjusted P Value	<0.0001	<0.0001	<0.0001	<0.0001	<0.0001	0.8881	0.0179	0.5598	0.5496	0.0031	0.1345	0.1308	0.2757	0.2827	>0.9999
	Mean Difference	-0.3213	-0.282	-0.4684	-0.3841	-0.3848	0.03929	-0.1471	-0.06279	-0.06346	-0.1863	-0.1021	-0.1027	0.08427	0.0836	-0.000667
	Summary	****	****	****	****	****	ns	*	ns	ns	**	ns	ns	ns	ns	ns
CYTO_Mean Gray value	Adjusted P Value	<0.0001	<0.0001	<0.0001	<0.0001	<0.0001	0.00	0.91	>0.9999	0.32	0.00	0.00	0.08	0.82	0.07	0.43
	Mean Difference	-22.6	-14.74	-24.09	-22.26	-19.35	7.862	-1.492	0.3332	3.246	-9.354	-7.529	-4.616	1.825	4.738	2.913
	Summary	****	****	****	****	****	**	ns	ns	ns	***	**	ns	ns	ns	ns
CYTO_Inegrated density	Adjusted P Value	0.1657	0.0039	0.0001	<0.0001	0.1812	0.2797	0.0079	<0.0001	>0.9999	0.309	0.0006	0.2575	0.0197	0.0072	<0.0001
	Mean Difference	165942	309286	448033	695873	162221	143344	282091	529931	-3721	138747	386587	-147065	247840	-285812	-533652
	Summary	ns	**	***	****	ns	ns	**	****	ns	ns	***	ns	*	**	****
CYTO_Soliditiy	Adjusted P Value	0.01	0.07	0.00	0.00	0.00	0.93	0.19	0.92	0.51	0.04	0.45	0.14	0.63	0.97	0.96
	Mean Difference	-0.1139	-0.08808	-0.1846	-0.1409	-0.1638	0.02586	-0.07068	-0.02692	-0.04987	-0.09654	-0.05278	-0.07573	0.04376	0.02081	-0.02295
	Summary	*	ns	***	**	***	ns	ns	ns	ns	*	ns	ns	ns	ns	ns
CYTO_Roundness	Adjusted P Value	<0.0001	<0.0001	<0.0001	0.0215	<0.0001	0.6558	0.0399	0.0005	0.7271	0.0031	0.0058	0.1022	<0.0001	0.3509	<0.0001
	Mean Difference	-0.3447	-0.2922	-0.4648	-0.1327	-0.3928	0.05248	-0.1201	0.212	-0.04811	-0.1726	0.1595	-0.1006	0.3321	0.072	-0.2601
	Summary	****	****	****	*	****	ns	*	***	ns	**	**	ns	****	ns	****
CYTO_Aspect Ratio (AR)	Adjusted P Value	0.01	0.08	<0.0001	0.06	0.00	0.76	0.08	0.85	0.82	0.01	>0.9999	0.18	0.01	0.45	0.24
	Mean Difference	0.8764	0.6085	1.498	0.6457	1.119	-0.2679	0.6221	-0.2307	0.2431	0.89	0.03721	0.511	-0.8528	-0.3791	0.4737
	Summary	**	ns	****	ns	**	ns	ns	ns	ns	**	ns	ns	*	ns	ns
CYTO_Max_Gray value	Adjusted P Value	<0.0001	0.9927	<0.0001	0.0003	0.0001	0.0001	0.27	0.8737	0.9977	<0.0001	0.0006	0.0002	0.0479	0.1425	0.9823
	Mean Difference	-19.59	-1.381	-25.35	-16.83	-18.51	18.21	-5.762	2.762	1.079	-23.97	-15.44	-17.13	8.524	6.841	-1.683
	Summary	****	ns	****	***	***	***	ns	ns	ns	****	***	***	*	ns	ns
CYTO_Min_Gray value	Adjusted P Value	0.13	0.68	0.13	0.92	0.52	0.01	>0.9999	0.50	0.90	0.01	0.21	0.06	0.50	0.90	0.96
	Mean Difference	-1.952	1.048	-1.952	-0.6825	-1.238	3	0	1.27	0.7143	-3	-1.73	-2.286	1.27	0.7143	-0.5556
	Summary	ns	ns	ns	ns	ns	*	ns	ns	ns	*	ns	ns	ns	ns	ns
CYTO_Coeff. Of variation	Adjusted P Value	>0.9999	>0.9999	>0.9999	<0.0001	>0.9999	>0.9999	>0.9999	<0.0001	>0.9999	>0.9999	<0.0001	>0.9999	<0.0001	>0.9999	<0.0001
	Mean Difference	0.1126	0.03723	-0.05338	48271	0.04515	-0.07534	-0.166	48271	-0.06743	-0.09061	48271	0.007918	48271	0.09853	-48271
	Summary	ns	ns	ns	****	ns	ns	ns	****	ns	ns	****	ns	****	ns	****
CYTO_Centroid	Adjusted P Value	0.90	0.71	0.88	0.98	0.99	1.00	>0.9999	1.00	1.00	1.00	0.97	0.96	1.00	1.00	>0.9999
	Mean Difference	45.48	64.14	48.21	29.52	26.97	18.66	2.731	-15.96	-18.51	-15.93	-34.63	-37.17	-18.69	-21.24	-2.545
	Summary	ns	ns	ns	ns	ns	ns	ns	ns	ns	ns	ns	ns	ns	ns	ns
NC_Area	Adjusted P Value	<0.0001	<0.0001	<0.0001	<0.0001	<0.0001	0.998	0.2032	>0.9999	0.9994	0.3632	0.9997	0.9735	0.2459	0.1246	0.9971
	Mean Difference	1917	2015	2503	1948	1842	98.38	586.3	30.81	-74.92	487.9	-67.57	-173.3	-555.5	-661.2	-105.7
	Summary	****	****	****	****	****	ns	ns	ns	ns	ns	ns	ns	ns	ns	ns
NC_Perimeter	Adjusted P Value	<0.0001	<0.0001	<0.0001	<0.0001	<0.0001	1.00	0.14	>0.9999	>0.9999	0.21	1.00	1.00	0.13	0.14	>0.9999
	Mean Difference	89.83	92.27	113.6	89.58	89.9	2.439	23.75	-0.2544	0.06687	21.31	-2.693	-2.372	-24.01	-23.69	0.3213
	Summary	****	****	****	****	****	ns	ns	ns	ns	ns	ns	ns	ns	ns	ns
NC_Feret's diameter	Adjusted P Value	<0.0001	<0.0001	<0.0001	<0.0001	<0.0001	>0.9999	0.0254	0.9999	0.9956	0.0188	>0.9999	0.9995	0.0171	0.0111	0.9998
	Mean Difference	32.05	31.61	41.52	31.47	30.82	-0.4475	9.468	-0.5871	-1.238	9.916	-0.1396	-0.79	-10.06	-10.71	-0.6504
	Summary	****	****	****	****	****	ns	*	ns	ns	*	ns	ns	*	*	ns

Supplementary Table 2: Imaging-based comparative morphological analysis of monocytes and macrophage subtypes.

Morophological Features		GMØ_M0	MMØ_M0	M1_M0	M2_M0	cMØ_M0	MMØ_GMØ	M1_GMØ	M2_GMØ	cMØ_GMØ	M1_MMØ	M2_MMØ	cMØ_MMØ	M2_M1	cMØ_M1	cMØ_M2
NC_Circularity	Adjusted P Value	0.00	0.00	<0.0001	0.00	<0.0001	>0.9999	0.00	0.96	0.01	0.00	0.98	0.01	<0.0001	0.11	0.00
	Mean Difference	-0.03397	-0.03329	-0.07441	-0.02919	-0.05779	0.0006825	-0.04044	0.004778	-0.02383	-0.04113	0.004095	-0.02451	0.04522	0.01662	-0.0286
	Summary	***	**	****	**	****	ns	***	ns	*	***	ns	*	****	ns	**
NC_ Mean Gray value	Adjusted P Value	<0.0001	0.0058	<0.0001	<0.0001	<0.0001	0.0002	0.9211	0.5199	0.9009	<0.0001	0.0029	0.0009	0.1401	0.4017	0.9726
	Mean Difference	-19.34	-7.829	-20.97	-16.37	-17.62	11.52	-1.624	2.974	1.727	-13.14	-8.542	-9.79	4.597	3.35	-1.247
	Summary	****	**	****	****	****	***	ns	ns	ns	****	**	***	ns	ns	ns
NC_Inegrated density	Adjusted P Value	0.01	<0.0001	0.00	0.00	0.00	0.01	0.77	0.75	1.00	0.05	0.05	0.01	>0.9999	0.88	0.86
	Mean Difference	36532	72800	46853	47106	38369	36268	10321	10575	1837	-25947	-25693	-34431	253.6	-8484	-8738
	Summary	**	****	***	***	**	**	ns	ns	ns	ns	ns	**	ns	ns	ns
Area Ratio	Adjusted P Value	0.7434	0.6075	0.0286	0.0004	0.8705	0.9999	0.2578	0.0029	0.9998	0.3577	0.0042	0.9949	0.1337	0.1745	0.0019
	Mean Difference	3.543	4.167	9.548	16.68	2.858	0.6238	6.005	13.14	-0.6853	5.381	12.51	-1.309	7.134	-6.69	-13.82
	Summary	ns	ns	*	***	ns	ns	ns	**	ns	ns	**	ns	ns	ns	**
Perimeter Ratio	Adjusted P Value	0.03	0.03	<0.0001	<0.0001	0.02	>0.9999	0.00	0.00	>0.9999	0.00	0.00	>0.9999	0.87	0.00	0.00
	Mean Difference	1.605	1.596	3.818	4.294	1.634	-0.008992	2.212	2.689	0.02878	2.221	2.698	0.03777	0.4762	-2.184	-2.66
	Summary	*	*	****	****	*	ns	**	***	ns	**	***	ns	ns	**	***
Feret's diameter Ratio	Adjusted P Value	0.0126	0.0101	<0.0001	<0.0001	0.0107	>0.9999	0.0012	0.0007	>0.9999	0.0015	0.0008	>0.9999	0.9979	0.0014	0.0008
	Mean Difference	1.675	1.727	3.924	4.091	1.713	0.05194	2.249	2.416	0.03781	2.197	2.365	-0.01413	0.1673	-2.211	-2.379
	Summary	*	*	****	****	*	ns	**	***	ns	**	***	ns	ns	**	***
Mean Gray value Ratio	Adjusted P Value	<0.0001	<0.0001	<0.0001	<0.0001	0.00	0.75	0.98	0.74	0.13	0.39	0.14	0.72	0.98	0.04	0.01
	Mean Difference	-0.2532	-0.2158	-0.2718	-0.2912	-0.1768	0.03739	-0.01855	-0.03796	0.07643	-0.05594	-0.07535	0.03904	-0.01941	0.09498	0.1144
	Summary	****	****	****	****	***	ns	ns	ns	ns	ns	ns	ns	ns	*	*
Inegrated density Ratio	Adjusted P Value	0.7491	0.4659	0.0198	0.0002	0.7905	0.995	0.184	0.0013	>0.9999	0.3732	0.0029	0.9903	0.0859	0.163	0.0011
	Mean Difference	1.532	2.1	4.408	7.826	1.442	0.5685	2.876	6.294	-0.0898	2.307	5.726	-0.6583	3.419	-2.965	-6.384
	Summary	ns	ns	*	***	ns	ns	ns	**	ns	ns	**	ns	ns	ns	**
Circularity Ratio	Adjusted P Value	0.00	0.00	<0.0001	<0.0001	<0.0001	0.92	0.08	0.60	0.85	0.02	0.17	0.34	0.69	0.42	1.00
	Mean Difference	-0.3309	-0.286	-0.4738	-0.4056	-0.3839	0.04499	-0.1428	-0.07469	-0.05294	-0.1878	-0.1197	-0.09793	0.06814	0.08989	0.02175
	Summary	***	***	****	****	****	ns	ns	ns	ns	*	ns	ns	ns	ns	ns

"CYTO" indicates cytoplasmic morphological features, while "NC" refers to nuclear compartment features. Mo, Monocytes at 3 hours; cMØ, monocyte-derived macrophages differentiated without the addition of exogenous growth factors such as M-CSF or GM-CSF.; GMØ, monocyte-derived macrophages differentiated with GM-CSF (Granulocyte-Macrophage Colony-Stimulating Factor); MMØ, monocyte-derived macrophages differentiated with M-CSF (Macrophage Colony-Stimulating Factor); M1, pro-inflammatory macrophages activated with GM-CSF/ LPS/ INF-γ; M2, anti-inflammatory macrophages activated with M-CSF/ IL-4. Asterisks indicate levels of statistical significance for CD marker expression: * adjusted P-value ≤ 0.05; ** adjusted P-value ≤ 0.01; *** adjusted P-value ≤ 0.001; ns, not significant.

Supplementary Table 3: Ratiometric analysis of protein abundance between secretome and lysate fractions to evaluate potential intracellular leakage or protein shedding.

Protein	Accession ID	Protein name	Subcellular Location based on Uniprot Database	Mo	cMØ	GMØ	MMØ	M1	M2
TUBA1B	W5QC37	Tubulin alpha-1B chain	Cytoskeleton	5.47E-05	5.40E-05	5.54E-05	5.50E-05	5.32E-05	5.65E-05
CKAP4	W5Q7E0	Cytoskeleton-associated protein 4	Endoplasmic reticulum membrane	6.19E-05	6.16E-05	5.94E-05	5.89E-05	5.89E-05	5.85E-05
CANX	W5NYZ5	Calnexin	Endoplasmic reticulum membrane	5.42E-05	5.16E-05	5.09E-05	5.09E-05	5.11E-05	5.05E-05
VDAC2	W5PG36	Non-selective voltage-gated ion channel VDAC2	Mitochondrion outer membrane	5.55E-05	5.22E-05	5.30E-05	5.44E-05	5.29E-05	5.53E-05
ATP1A1	P04074	Sodium/potassium-transporting ATPase subunit alpha-1	Cell membrane	6.17E-05	5.15E-05	5.21E-05	5.20E-05	5.14E-05	5.23E-05
SLC3A2	W5Q8K4	Amino acid transporter heavy chain SLC3A2	Cell membrane	7.07E-05	5.84E-05	5.86E-05	5.71E-05	5.69E-05	5.75E-05
CD11B	W5PGV0	Integrin alpha-M (ITGAM)	Cell membrane	5.78E-01	0.00	0.62	0.70	0.57	0.86
CD18	Q6ECI6	Integrin beta-2 (ITGB2)	Cell membrane	5.86E-01	0.57	0.48	0.74	0.55	0.86
CD115	W5P8R4	Macrophage colony-stimulating factor 1 receptor (CSF1R)	Cell membrane	not defined (secretome only)	0.92	0.98	1.03	1.00	1.18
CD163	W5NY01	Scavenger receptor cysteine-rich type 1 protein M130	Cell membrane / secreted	1.20E+00	0.75	0.74	0.84	0.83	0.92
CD206	W5PRI6	Macrophage mannose receptor 1 (MRC1)	Cell membrane/ Endosome membrane	8.85E-05	0.00	0.63	0.77	0.67	0.92

Secretome-to-lysate (S/L) ratios were calculated from label-free quantification (LFQ) intensities, where S/L = mean LFQ intensity in conditioned medium ÷ mean LFQ intensity in lysate. Mo, Monocytes at 3 hours; cMØ, monocyte-derived macrophages differentiated without the addition of exogenous growth factors such as M-CSF or GM-CSF.; GMØ, monocyte-derived macrophages differentiated with GM-CSF (Granulocyte-Macrophage Colony-Stimulating Factor); MMØ, monocyte-derived macrophages differentiated with M-CSF (Macrophage Colony-Stimulating Factor); M1, pro-inflammatory macrophages activated with GM-CSF/ LPS/ INF-γ; M2, anti-inflammatory macrophages activated with M-CSF/ IL-4.

Supplementary Table 4: Differentially abundant proteins (DAPs) of cMØ relative to M0 and their cellular location.

FoldChange	Location	Protein IDs	Gene Names	P-value	FDR
10	CYTO	W5PEB0	FABP7	1.65E-09	0.00E+00
9	CYTO	W5PCH3	SCIN	2.15E-04	0.00E+00
9	CYTO	W5NU86	GLA	7.06E-05	0.00E+00
8	CYTO	W5PF33	GM2A	1.54E-06	0.00E+00
8	CYTO	W5PAM4	CTSA	2.58E-07	0.00E+00
8	CYTO	W5PT76	GPNMB	1.86E-05	0.00E+00
8	CYTO	W5NY01		1.42E-05	0.00E+00
7	CYTO	W5PKQ7		3.89E-07	6.96E-03
7	CYTO	W5P9G8	PLD3	1.86E-03	0.00E+00
7	CYTO	W5P9J8	BLVRB	1.14E-04	0.00E+00
7	CYTO	W5PZB2	CD68	7.38E-05	0.00E+00
7	CYTO	W5NRB6	MIDEAS	6.99E-06	2.18E-04
7	CYTO	W5PII2	ATG4C	1.15E-05	0.00E+00
7	CYTO	A9YUY8	FABP4	5.05E-04	0.00E+00
7	CYTO	P51977	ALDH1A1	9.55E-04	0.00E+00
7	CYTO	W5Q6N3	LOC101115115	4.54E-06	0.00E+00
7	CYTO	W5PIQ6	MSR1	1.34E-06	0.00E+00
7	CYTO	Q9MZS8	CTSD	4.73E-05	0.00E+00
7	CYTO	W5PJS4	EMILIN2	5.85E-06	0.00E+00
6	CYTO	W5P093	NQO1	9.65E-07	0.00E+00
6	CYTO	W5Q233	VCAN	1.10E-04	0.00E+00
6	CYTO	W5PBM9	SCPEP1	1.26E-04	0.00E+00
6	CYTO	W5PQR0	NIBAN2	7.46E-08	0.00E+00
6	CYTO	W5PKY1	HNMT	5.04E-05	0.00E+00
6	CYTO	G3M9U4	ACP5	3.91E-06	0.00E+00
6	CYTO	W5P640	LMNA	2.29E-03	0.00E+00
6	CYTO	W5PI56	DAB2	1.06E-04	0.00E+00
6	CYTO	W5PGC5	GALM	7.59E-05	0.00E+00
6	CYTO	W5PFY5	ASAH1	3.29E-04	0.00E+00
6	CYTO	W5P1A5	GBA1	7.92E-06	0.00E+00
6	CYTO	W5PZ47	CTSH	7.19E-03	0.00E+00
6	CYTO	W5QHL1	FCGR1A	2.03E-06	0.00E+00
6	CYTO	W5PCE0	PLBD2	2.57E-04	0.00E+00
6	CYTO	W5QGG0	TFRC	4.02E-06	0.00E+00
6	CYTO	W5PNP1	MFGE8	1.23E-04	0.00E+00
6	CYTO	W5P2F1	FOLR2	4.35E-06	0.00E+00
6	CYTO	W5QIW1	LGALS3	2.86E-04	0.00E+00
6	CYTO	W5PRI6	MRC1	5.53E-06	0.00E+00
6	CYTO	W5P530	LOC101104705	5.12E-03	0.00E+00
6	CYTO	W5PV43	LRPAP1	4.57E-06	0.00E+00
6	CYTO	W5PUH5	LGMN	6.29E-04	0.00E+00
6	CYTO	W5NVW7	NAGLU	2.15E-04	0.00E+00
6	CYTO	W5QCD6	IDH1	5.04E-06	0.00E+00
6	CYTO	W5PYW0	TCIRG1	4.02E-05	0.00E+00
6	CYTO	W5PXR1	ENPP1	2.31E-03	0.00E+00
6	CYTO	W5QC89	HEXA	4.72E-06	0.00E+00
6	CYTO	W5Q5W2	LOC101110539	7.10E-07	0.00E+00
6	CYTO	W5QI70	CTSS	1.03E-04	0.00E+00
6	CYTO	W5PA90	AGA	1.81E-06	0.00E+00
5	CYTO	P35623	SHMT1	3.53E-05	0.00E+00
5	CYTO	W5P1H0	CTSC	5.29E-04	0.00E+00
5	CYTO	W5PU22	MCUR1	1.68E-02	0.00E+00
5	CYTO	W5Q612	TRPV2	4.55E-07	0.00E+00
5	CYTO	W5QBZ7	NAGA	2.83E-04	0.00E+00
5	CYTO	W5QHG1	EPS8	3.42E-05	0.00E+00
5	CYTO	W5P8H9	SGPL1	7.06E-05	0.00E+00
5	CYTO	W5Q0U0	P2RX4	3.52E-05	0.00E+00
5	CYTO	W5PGH4	TNKS1BP1	4.08E-02	0.00E+00
5	CYTO	W5PD43	HTRA1	2.17E-02	0.00E+00
5	CYTO	W5PUP6	SPATA13	4.75E-02	2.82E-03
5	CYTO	W5QH35	CAPG	2.77E-05	0.00E+00
5	CYTO	W5QI00	LACTB	7.68E-06	0.00E+00
5	CYTO	W5NSH8	NPC2	5.69E-07	0.00E+00

Supplementary Table 4: Differentially abundant proteins (DAPs) of cMØ relative to M0 and their cellular location.

FoldChange	Location	Protein IDs	Gene Names	P-value	FDR
5	CYTO	W5NPP2	CPM	2.38E-06	0.00E+00
5	CYTO	W5NYL0	MAOA	5.56E-05	0.00E+00
5	CYTO	W5PRG9	HAL	2.88E-06	0.00E+00
5	CYTO	W5Q940	SHTN1	1.46E-03	0.00E+00
5	CYTO	W5NTZ3	RENBP	2.64E-05	0.00E+00
5	CYTO	W5P4C9	MMP12	1.67E-03	0.00E+00
5	CYTO	W5PGF4	PLAUR	3.63E-03	0.00E+00
5	CYTO	W5PBS4	LRP1	8.48E-05	0.00E+00
5	CYTO	W5QI78	CTSK	4.90E-03	0.00E+00
5	CYTO	Q29524	LPL	9.37E-04	0.00E+00
5	CYTO	W5QCG9	SRXN1	6.78E-04	0.00E+00
5	CYTO	W5NYU9	MPP1	1.18E-06	0.00E+00
5	CYTO	W5PDH4	MMP9	1.03E-04	0.00E+00
5	CYTO	W5PE67		4.72E-04	0.00E+00
5	CYTO	W5QI12	TLN2	9.93E-05	5.17E-03
5	CYTO	W5P8M9		1.16E-04	0.00E+00
5	CYTO	W5NW80	GAA	2.52E-04	0.00E+00
5	CYTO	W5QBM4	ALCAM	1.05E-05	0.00E+00
5	CYTO	W5PTG5	ATP6V0D2	5.78E-03	0.00E+00
5	CYTO	W5PKU3		2.34E-05	0.00E+00
5	CYTO	W5NUI6	SGSH	1.18E-04	0.00E+00
5	CYTO	W5NRS0		4.11E-05	0.00E+00
5	CYTO	W5PNY4	PDXK	4.75E-04	0.00E+00
5	CYTO	W5PAQ4	FUCA1	3.18E-04	0.00E+00
5	CYTO	W5PBC0		7.41E-04	0.00E+00
5	CYTO	W5QCL8	NPL	4.34E-04	0.00E+00
5	CYTO	W5PUL5	FCGRT	2.47E-04	0.00E+00
5	CYTO	W5PTU7	CA2	4.69E-04	0.00E+00
5	CYTO	W5Q0A3	TLR2	6.07E-05	0.00E+00
5	CYTO	P83205	CTSB	4.94E-04	0.00E+00
5	CYTO	W5NYE0	ATP6V0D1	4.30E-03	0.00E+00
5	CYTO	W5QFU8	CD86	8.89E-06	0.00E+00
5	CYTO	W5NZ62	GNS	4.22E-04	0.00E+00
5	CYTO	W5P703	WFS1	1.39E-03	0.00E+00
5	CYTO	W5PED4		1.83E-02	0.00E+00
5	CYTO	W5P5W6	NDRG1	2.08E-03	0.00E+00
5	CYTO	W5QDF4	GSTM3	1.04E-03	0.00E+00
4	CYTO	W5QAU1		2.00E-02	0.00E+00
4	CYTO	W5Q284	HAPLN1	6.40E-03	0.00E+00
4	CYTO	W5NUC8	ARMCX3	1.02E-04	0.00E+00
4	CYTO	W5PDH7	NPC1	1.36E-04	0.00E+00
4	CYTO	W5PNC8	PADI2	1.93E-03	0.00E+00
4	CYTO	C5IJ93	RAB9A	7.04E-06	0.00E+00
4	CYTO	W5PWX3	CRYL1	4.79E-05	0.00E+00
4	CYTO	P82197	PDXK	1.47E-03	0.00E+00
4	CYTO	W5NZK6	PLA2G15	2.49E-05	0.00E+00
4	CYTO	W5PRG8	CREG1	5.26E-04	0.00E+00
4	CYTO	W5P0L3	AOAH	2.03E-02	0.00E+00
4	CYTO	W5QAH8		1.83E-04	0.00E+00
4	CYTO	W5P2V3	PEPD	5.78E-03	0.00E+00
4	CYTO	W5P026	STAB1	5.56E-05	0.00E+00
4	CYTO	W5Q678	SEC24D	1.67E-03	0.00E+00
4	CYTO	W5PIS6	NHLRC3	6.01E-04	0.00E+00
4	CYTO	W5NU23	FUCA2	1.53E-05	0.00E+00
4	CYTO	Q9MZA9	VIM	2.98E-02	0.00E+00
4	CYTO	W5PGS4	FABP5	1.16E-04	0.00E+00
4	CYTO	W5Q2B3	SUSD5	3.01E-03	8.55E-03
4	CYTO	W5NTJ2	TPX2	3.63E-02	5.72E-03
4	CYTO	W5PLB8	EPB41L3	1.41E-03	0.00E+00
4	CYTO	W5P960	SLC29A3	5.37E-04	0.00E+00
4	CYTO	W5Q8J3	RRBP1	4.38E-03	0.00E+00
4	CYTO	W5NVC8	NLRP2	3.23E-02	9.05E-03
4	CYTO	W5Q3U3	LOC101102156	1.76E-03	0.00E+00

Supplementary Table 4: Differentially abundant proteins (DAPs) of cMØ relative to M0 and their cellular location.

FoldChange	Location	Protein IDs	Gene Names	P-value	FDR
4	CYTO	W5PEE9	LAMP1	5.86E-03	0.00E+00
4	CYTO	W5PZG5	OCRL	7.66E-04	0.00E+00
4	CYTO	W5P3Q3	LOC100101238	3.09E-05	0.00E+00
4	CYTO	W5Q7I8	TAX1BP3	2.12E-02	0.00E+00
4	CYTO	P05028	ATP1B1	1.75E-03	0.00E+00
4	CYTO	W5PE92	GRN	4.44E-05	0.00E+00
4	CYTO	W5PCK8	LOC100135455	1.56E-02	0.00E+00
4	CYTO	W5QDW4	USF3	1.04E-02	9.68E-04
4	CYTO	W5PGA9	NCSTN	7.49E-05	0.00E+00
4	CYTO	W5P895	CRABP2	1.53E-04	0.00E+00
4	CYTO	W5NY29		3.62E-02	0.00E+00
4	CYTO	W5Q263	ICAM1	1.25E-04	0.00E+00
4	CYTO	W5QBP6	GLUL	2.14E-03	0.00E+00
4	CYTO	W5PQ75	HSPH1	4.27E-03	0.00E+00
4	CYTO	W5P7Y8	PALD1	9.15E-04	0.00E+00
4	CYTO	W5PB61		1.69E-04	2.16E-04
4	CYTO	W5Q3N1	CTSZ	5.81E-06	0.00E+00
4	CYTO	W5PBG1		7.81E-04	0.00E+00
4	CYTO	W5PG10	PAPSS1	2.34E-03	0.00E+00
4	CYTO	W5PAC2	LOC101105044	8.88E-03	0.00E+00
4	CYTO	W5NT19	SPAG1	1.84E-02	0.00E+00
4	CYTO	W5Q0F1	LIPA	5.84E-03	0.00E+00
4	CYTO	W5Q2R5	SDF2L1	1.32E-02	0.00E+00
4	CYTO	W5PVE3	LOC101115252	1.86E-02	0.00E+00
4	CYTO	W5NUE5	SLC38A7	2.12E-04	0.00E+00
4	CYTO	W5PFB1	TOR1B	3.69E-04	0.00E+00
4	CYTO	W5PJT1	PLAC9	4.61E-03	0.00E+00
4	CYTO	W5Q8G9	SPG21	3.22E-05	0.00E+00
4	CYTO	W5Q2Y1	PLXNC1	1.27E-02	0.00E+00
4	CYTO	W5Q0G8	IMPA1	7.29E-04	0.00E+00
4	CYTO	W5Q6C5	GGA2	1.12E-02	0.00E+00
4	CYTO	W5Q7X3	GNPNAT1	9.07E-03	0.00E+00
4	CYTO	W5PG74	IRGQ	7.03E-03	0.00E+00
4	CYTO	W5NX56	SPP1	1.09E-03	0.00E+00
4	CYTO	W5NUG3	GNPDA1	2.47E-02	0.00E+00
4	CYTO	W5Q2U7	PLEC	1.45E-03	0.00E+00
4	CYTO	W5Q5C8	SOAT1	2.86E-05	0.00E+00
4	CYTO	W5QHR5	PLEKHO2	7.11E-05	0.00E+00
4	CYTO	W5NZ70	LGALS3BP	2.00E-02	0.00E+00
4	CYTO	W5NUE3	PRDX1	7.78E-04	0.00E+00
4	CYTO	W5QB71	AMDHD2	3.25E-03	0.00E+00
4	CYTO	W5PHG1	CDA	3.20E-03	0.00E+00
4	CYTO	W5QHF2	LOC114117536	1.26E-02	0.00E+00
4	CYTO	W5PP04	GNG12	7.08E-04	0.00E+00
4	CYTO	W5PBR7	P4HA1	8.97E-04	0.00E+00
4	CYTO	W5PCE8	ATP2B3	5.23E-02	5.18E-03
4	CYTO	Q6XXL8	DYNLT3	6.82E-03	0.00E+00
4	CYTO	W5PL89	GSR	4.76E-04	0.00E+00
4	CYTO	W5QFU4		4.06E-03	0.00E+00
4	CYTO	W5QCT2		2.34E-02	0.00E+00
4	CYTO	W5Q7R3	LOC101118736	3.84E-03	0.00E+00
4	CYTO	W5PMX5	C5AR1	1.87E-03	0.00E+00
4	CYTO	W5P3S0		3.84E-06	0.00E+00
4	CYTO	W5PC32	PGM3	1.90E-03	0.00E+00
4	CYTO	W5NUJ5		1.49E-03	0.00E+00
4	CYTO	W5PFV1	CTSL	1.64E-04	0.00E+00
4	CYTO	W5Q1W2	SDCBP	3.71E-02	0.00E+00
4	CYTO	W5QFD9		1.12E-02	0.00E+00
4	CYTO	W5NYK9	CALU	3.71E-02	0.00E+00
4	CYTO	W5QDJ5	LOC101122319	3.16E-04	0.00E+00
4	CYTO	P29330	FDX1	7.75E-05	0.00E+00
4	CYTO	W5PVC8	ERGIC3	7.25E-05	0.00E+00
4	CYTO	W5Q777	HS1BP3	4.88E-06	0.00E+00

Supplementary Table 4: Differentially abundant proteins (DAPs) of cMØ relative to M0 and their cellular location.

FoldChange	Location	Protein IDs	Gene Names	P-value	FDR
4	CYTO	W5P3N6	LOC101112162	8.66E-04	0.00E+00
4	CYTO	W5PPX2	SENP8	1.04E-02	3.01E-03
4	CYTO	W5NZX4	KATNAL2	1.53E-02	3.56E-03
4	CYTO	W5P1F5	CHMP2A	2.52E-02	0.00E+00
4	CYTO	W5Q4B1	MYCBP2	2.52E-02	0.00E+00
4	CYTO	W5NYU6	NT5DC2	8.91E-04	0.00E+00
4	CYTO	W5Q4U5	CPT1A	3.03E-03	0.00E+00
4	CYTO	W5PNK3	ACAN	2.90E-03	0.00E+00
4	CYTO	W5P369	AP2A2	3.81E-02	0.00E+00
4	CYTO	W5NTW0	CADM1	2.85E-03	0.00E+00
3	CYTO	W5PPM6	PQBP1	1.93E-03	2.03E-04
3	CYTO	W5NVV1		2.05E-02	0.00E+00
3	CYTO	W5Q5N6	BST-2B	1.31E-04	0.00E+00
3	CYTO	W5PC06	SIRPA	1.31E-02	0.00E+00
3	CYTO	W5PIE4	CLPTM1	4.82E-03	0.00E+00
3	CYTO	W5PF85	PEA15	8.99E-05	0.00E+00
3	CYTO	W5P3L5	RNF13	3.71E-04	0.00E+00
3	CYTO	W5Q6S3	WASHC4	1.08E-03	0.00E+00
3	CYTO	W5QEE5	TRAPPC3	2.01E-02	0.00E+00
3	CYTO	W5PY08		3.30E-03	0.00E+00
3	CYTO	W5NZJ7	FGR	1.01E-02	0.00E+00
3	CYTO	W5PMA0	AP2S1	1.27E-03	0.00E+00
3	CYTO	W5PUW2	IFI30	3.61E-02	0.00E+00
3	CYTO	W5P6H9	ACP2	1.22E-02	0.00E+00
3	CYTO	W5PHQ8	TSTD1	3.31E-03	0.00E+00
3	CYTO	W5Q700	APPL2	6.09E-06	0.00E+00
3	CYTO	W5PYH9		1.66E-02	0.00E+00
3	CYTO	W5PGN2	WDFY2	3.22E-02	1.15E-03
3	CYTO	W5QI36	HEBP1	4.44E-03	0.00E+00
3	CYTO	W5P1I7	UBE2M	2.33E-02	0.00E+00
3	CYTO	W5Q1W7	PALLD	4.33E-04	0.00E+00
3	CYTO	W5P1U9	OXSM	3.07E-04	0.00E+00
3	CYTO	W5QBR5	BMP2K	1.05E-02	0.00E+00
3	CYTO	W5NXT4		1.48E-02	0.00E+00
3	CYTO	W5NTW3	ITIH1	9.94E-03	0.00E+00
3	CYTO	W5PG41	H6PD	4.37E-04	0.00E+00
3	CYTO	Q10994	CSTB	2.62E-06	0.00E+00
3	CYTO	W5PVJ5	CD274	4.46E-04	0.00E+00
3	CYTO	W5P3H8	IGF2R	2.08E-02	0.00E+00
3	CYTO	W5P3I5	CNDP2	1.02E-04	0.00E+00
3	CYTO	W5NVV6	DNAJC3	5.76E-03	0.00E+00
3	CYTO	W5PJN7	HOMER3	8.95E-05	0.00E+00
3	CYTO	W5Q9Q5	PANK4	2.24E-04	2.18E-04
3	CYTO	W5PXS1	RAB3IL1	1.82E-03	0.00E+00
3	CYTO	W5QG92	OSBPL11	1.19E-04	0.00E+00
3	CYTO	W5P3C6	LOC101111906	3.19E-02	0.00E+00
3	CYTO	W5Q420		1.50E-03	0.00E+00
3	CYTO	W5QEH0	TWF1	9.63E-04	0.00E+00
3	CYTO	W5QBE4	FGL2	3.74E-02	0.00E+00
3	CYTO	W5NSS6		4.75E-03	0.00E+00
3	CYTO	W5PAX1	GCLC	7.90E-03	0.00E+00
3	CYTO	W5P164	LAMTOR1	6.93E-03	0.00E+00
3	CYTO	W5NYV5	SPAG9	8.40E-03	0.00E+00
3	CYTO	W5P536	TRIP10	8.44E-04	0.00E+00
3	CYTO	W5PEP1	ATP1A2	5.31E-03	2.08E-04
3	CYTO	W5NU94	PSTPIP1	2.80E-03	0.00E+00
3	CYTO	W5PQQ6	NECAP2	5.43E-02	0.00E+00
3	CYTO	W5P0U9	ANGPTL4	2.85E-03	2.16E-04
3	CYTO	P04074	ATP1A1	2.88E-03	0.00E+00
3	CYTO	W5PJR5	LOC101121216	2.22E-05	2.04E-04
3	CYTO	W5PAJ2	PSAP	6.48E-03	0.00E+00
3	CYTO	W5QEH8	CTTNBP2NL	2.37E-03	0.00E+00
3	CYTO	W5NX08	WASHC2C	3.39E-02	0.00E+00

Supplementary Table 4: Differentially abundant proteins (DAPs) of cMØ relative to M0 and their cellular location.

FoldChange	Location	Protein IDs	Gene Names	P-value	FDR
3	CYTO	W5QDH3	COL2A1	2.30E-03	1.72E-03
3	CYTO	W5NVR9	C21H11orf54	4.41E-03	0.00E+00
3	CYTO	W5Q318	CCNH	4.27E-04	4.29E-03
3	CYTO	W5P363	VAT1	2.04E-03	0.00E+00
3	CYTO	W5P3H9	PICALM	5.31E-02	0.00E+00
3	CYTO	W5PKV1	DNASE2	4.43E-03	0.00E+00
3	CYTO	W5NQS7	IFNGR1	1.44E-03	0.00E+00
3	CYTO	W5PVN6		3.10E-02	5.90E-03
3	CYTO	W5P2W1	NEU1	5.26E-03	0.00E+00
3	CYTO	W5P2F6	FAF1	7.66E-05	0.00E+00
3	CYTO	P68253	YWHAG	1.33E-02	0.00E+00
3	CYTO	W5Q024	GPD1L	2.84E-02	0.00E+00
3	CYTO	W5Q686	TPP1	2.20E-03	0.00E+00
3	CYTO	W5P0K8	CLIC2	5.16E-02	0.00E+00
3	CYTO	W5QFD0	RRAGC	8.88E-03	0.00E+00
3	CYTO	W5Q175	GNPTAB	5.64E-04	0.00E+00
3	CYTO	W5P4H0	DNASE1L1	2.55E-02	0.00E+00
3	CYTO	W5P1Q0	AP1B1	1.01E-04	0.00E+00
3	CYTO	W5Q5P5	GGH	2.63E-03	0.00E+00
3	CYTO	W5P8A0		2.52E-02	0.00E+00
3	CYTO	W5P726	PRCP	9.41E-03	0.00E+00
3	CYTO	W5QG63	ZMPSTE24	1.12E-04	0.00E+00
3	CYTO	W5QHU8	FNDC3B	3.89E-05	0.00E+00
3	CYTO	W5P4A8	RNASET2	8.85E-03	0.00E+00
3	CYTO	W5PF22	KMO	1.81E-02	0.00E+00
3	CYTO	W5PQT3	LOC101105107	2.33E-04	0.00E+00
3	CYTO	W5PQV2	LMBRD1	1.14E-02	0.00E+00
3	CYTO	W5Q3V0	IL18	4.50E-04	0.00E+00
3	CYTO	W5PUL4	MTMR6	8.89E-03	0.00E+00
3	CYTO	W5Q1M0	GLB1	1.26E-03	0.00E+00
3	CYTO	W5QA16		3.02E-04	0.00E+00
3	CYTO	W5PMC5	LOC101110546	5.60E-04	0.00E+00
3	CYTO	W5QAP3	TOM1	5.51E-03	0.00E+00
3	CYTO	W5NQ23	WIPF1	1.52E-02	0.00E+00
3	CYTO	W5QDN8	SLC48A1	1.53E-04	2.16E-04
3	CYTO	E7ECV8	NUDT9	5.29E-02	0.00E+00
3	CYTO	W5Q8K4	SLC3A2	3.04E-02	0.00E+00
3	CYTO	W5PMX7		2.01E-02	0.00E+00
3	CYTO	W5PCD7	ABHD12	1.25E-02	0.00E+00
3	CYTO	W5PYE8	MERTK	2.59E-04	0.00E+00
3	CYTO	W5PT36	RBM47	7.39E-04	0.00E+00
3	CYTO	W5P6U2		1.13E-03	0.00E+00
3	CYTO	W5QHA0	AGFG1	3.75E-02	0.00E+00
3	CYTO	W5P800	GAS7	1.84E-02	0.00E+00
3	CYTO	W5QIC7	YBX3	3.28E-06	0.00E+00
3	CYTO	W5PFH4	MAPKAPK3	1.64E-02	0.00E+00
3	CYTO	W5P4X6	LOC101104287	8.05E-03	0.00E+00
3	CYTO	W5P8L0	ALDH18A1	3.39E-02	0.00E+00
3	CYTO	W5NWX4		1.17E-02	0.00E+00
3	CYTO	W5P539	TYROBP	4.26E-02	0.00E+00
3	CYTO	W5NWX7	CLEC4A	5.03E-03	0.00E+00
3	CYTO	Q6XUZ5	IDH1	1.68E-02	0.00E+00
3	CYTO	W5PCD0	FUBP3	9.43E-03	0.00E+00
3	CYTO	W5PZ94	ACO1	3.19E-02	0.00E+00
3	CYTO	W5NTM7	DENND10	2.33E-02	0.00E+00
3	CYTO	W5PWG8	LOC101115420	8.12E-03	0.00E+00
3	CYTO	W5QI40	MYO1E	2.48E-02	0.00E+00
3	CYTO	W5NW08	LOC101123656	2.58E-02	0.00E+00
3	CYTO	W5PQW3	MIEN1	1.12E-02	0.00E+00
3	CYTO	W5QGR5	WDFY1	2.18E-02	0.00E+00
3	CYTO	W5P407		1.40E-02	0.00E+00
3	CYTO	W5PR73	CORO1B	4.22E-02	0.00E+00
3	CYTO	W5PW33		4.82E-04	0.00E+00

Supplementary Table 4: Differentially abundant proteins (DAPs) of cMØ relative to M0 and their cellular location.

FoldChange	Location	Protein IDs	Gene Names	P-value	FDR
3	CYTO	W5P3W6	OSBPL9	3.33E-03	0.00E+00
3	CYTO	W5NSN4		2.07E-02	2.22E-04
3	CYTO	W5PES0	STX4	3.52E-04	0.00E+00
3	CYTO	W5NWR6	LXN	1.49E-02	0.00E+00
3	CYTO	W5PJ40	EIF2AK2	5.31E-02	0.00E+00
3	CYTO	W5PY53	FTH1	4.88E-02	0.00E+00
3	CYTO	W5P743	GLMP	1.03E-03	0.00E+00
3	CYTO	W5Q2V0	YKT6	1.04E-04	0.00E+00
3	CYTO	W5PDJ0		5.26E-03	0.00E+00
3	CYTO	W5PSD4	GYS1	2.71E-02	0.00E+00
3	CYTO	W5PP47		3.35E-02	0.00E+00
3	CYTO	W5NRD1	ERLIN2	9.58E-05	0.00E+00
3	CYTO	W5PGG5	CD84	2.40E-02	0.00E+00
3	CYTO	W5QBV7	CD44	9.02E-03	0.00E+00
3	CYTO	W5P366	FAM50A	7.32E-03	0.00E+00
3	CYTO	W5QH13	VPS39	7.84E-04	0.00E+00
3	CYTO	W5PDZ1		8.39E-03	0.00E+00
3	CYTO	W5P8F3	AGPAT5	1.23E-03	0.00E+00
3	CYTO	W5NQC1	PSEN1	7.90E-03	0.00E+00
3	CYTO	W5P2U4	LOC101105644	3.75E-02	0.00E+00
3	CYTO	W5QGH9	WDR3	3.48E-02	2.09E-04
3	CYTO	W5NX16		3.16E-02	0.00E+00
3	CYTO	W5PZJ8	ASRGL1	5.97E-03	0.00E+00
3	CYTO	W5Q8I7	LOC101122123	7.20E-03	0.00E+00
3	CYTO	W5PAA0	RBMS1	1.60E-04	2.20E-04
3	CYTO	W5PLZ0	ATP6V1B2	3.13E-03	0.00E+00
3	CYTO	W5NSZ7	RAB31	2.78E-02	0.00E+00
3	CYTO	W5PGL9		4.00E-03	0.00E+00
3	CYTO	W5P3B0	INPPL1	8.81E-03	0.00E+00
3	CYTO	W5NWG5	LOC101118849	8.47E-03	0.00E+00
3	CYTO	W5PUJ2	LAMP2	2.89E-02	0.00E+00
3	CYTO	W5PDK4	AIF1	7.29E-03	0.00E+00
3	CYTO	W5NV06	ATP6V0A1	3.67E-02	0.00E+00
3	CYTO	W5QEL6	PCYOX1	3.41E-02	0.00E+00
3	CYTO	W5PJY6	ADAM28	2.32E-02	0.00E+00
3	CYTO	W5QCU8	NOP9	5.34E-02	1.34E-03
3	CYTO	W5Q553	ITGAV	4.37E-04	0.00E+00
3	CYTO	W5PPS6		1.52E-03	0.00E+00
3	CYTO	W5PPT8	GLIPR2	6.05E-04	0.00E+00
3	CYTO	W5PBL5	SLC44A1	6.64E-03	0.00E+00
3	CYTO	W5QEU6	ANXA4	1.66E-02	0.00E+00
3	CYTO	W5QA17	ATXN2	1.90E-02	0.00E+00
3	CYTO	W5Q8A7	RALA	4.17E-02	0.00E+00
3	CYTO	W5NVK6	DAGLB	3.22E-03	0.00E+00
3	CYTO	W5P9K4	NUFIP2	2.10E-02	0.00E+00
3	CYTO	W5P7L9	KLC1	7.86E-03	0.00E+00
3	CYTO	W5P261	PPM1H	1.90E-02	0.00E+00
3	CYTO	W5P1T4	PPP4C	2.00E-03	0.00E+00
3	CYTO	W5Q5T7	ATP6V1C1	5.59E-02	0.00E+00
3	CYTO	W5PBX0	ATP11A	2.82E-02	0.00E+00
3	CYTO	W5Q5K0		1.50E-03	0.00E+00
3	CYTO	W5Q0Y4	ZFAND6	5.48E-02	0.00E+00
3	CYTO	W5P340	SOD2	5.36E-02	0.00E+00
3	CYTO	W5P1U3	LACTB2	6.26E-03	0.00E+00
3	CYTO	W5PCI1	NLRP3	1.33E-02	0.00E+00
3	CYTO	W5PZD7		3.85E-02	0.00E+00
3	CYTO	W5PXY4	CLEC5A	3.27E-02	0.00E+00
2	CYTO	W5PFP5	PLIN3	4.00E-02	0.00E+00
2	CYTO	W5QDQ8	MMP14	2.18E-02	0.00E+00
2	CYTO	Q9MZD1	SLC17A5	4.12E-02	0.00E+00
2	CYTO	W5QAQ4	NRP2	5.85E-03	0.00E+00
2	CYTO	W5PB38		1.85E-03	0.00E+00
2	CYTO	W5PAX4	TEX264	1.53E-02	0.00E+00

Supplementary Table 4: Differentially abundant proteins (DAPs) of cMØ relative to M0 and their cellular location.

FoldChange	Location	Protein IDs	Gene Names	P-value	FDR
2	CYTO	W5QDY5	ATP6V1A	1.72E-02	0.00E+00
2	CYTO	W5NV37	COMMD10	4.90E-02	0.00E+00
2	CYTO	W5Q4N4	TOR3A	5.34E-03	0.00E+00
2	CYTO	W5PRQ0	RBM3	3.05E-02	0.00E+00
2	CYTO	W5Q6K0	HEATR3	2.82E-02	2.46E-03
2	CYTO	W5PC89	POLR1C	4.74E-02	0.00E+00
2	CYTO	W5PVC1	SYNJ1	7.06E-03	0.00E+00
2	CYTO	W5PH35	LOC101119706	2.18E-03	0.00E+00
2	CYTO	W5NVC9	RAC1	1.33E-02	0.00E+00
2	CYTO	W5Q928	PCYT2	1.48E-02	0.00E+00
2	CYTO	W5Q0N7	IFIT2	6.95E-03	0.00E+00
2	CYTO	W5QIJ6	SPPL2A	3.76E-02	0.00E+00
2	CYTO	W5Q3G3	SRP19	6.07E-03	2.20E-04
2	CYTO	W5QJA2	CD14	2.33E-02	0.00E+00
2	CYTO	W5PEZ1		1.17E-04	0.00E+00
2	CYTO	W5Q0B4	KYAT3	1.01E-03	2.01E-04
2	CYTO	W5QFZ2	DTX3L	5.01E-03	0.00E+00
2	CYTO	W5PTS6	CD83	1.95E-03	8.72E-03
2	CYTO	W5P7B1	SIRT2	4.95E-02	0.00E+00
2	CYTO	W5P9E4	LSM12	1.53E-02	0.00E+00
2	CYTO	W5P6Y8	SLC9A9	3.40E-02	0.00E+00
2	CYTO	W5PED7		8.00E-03	2.06E-04
2	CYTO	W5PCS4		1.00E-03	0.00E+00
2	CYTO	W5Q7X8	CRYBG3	1.65E-02	0.00E+00
2	CYTO	W5QGJ2	CHP1	1.02E-02	0.00E+00
2	CYTO	W5Q149	OXR1	5.02E-02	0.00E+00
2	CYTO	W5NQZ9	GSDMD	5.56E-03	0.00E+00
2	CYTO	W5PPY5	CD2AP	7.80E-04	0.00E+00
2	CYTO	W5PDX8	FAM120A	4.93E-04	0.00E+00
2	CYTO	W5PN31		1.92E-02	7.77E-04
2	CYTO	W5NTM0		1.18E-03	2.19E-04
2	CYTO	W5PEN2	TPD52	1.81E-03	0.00E+00
2	CYTO	W5QCF3	SLC35F6	2.09E-03	0.00E+00
2	CYTO	W5PCC0	LACC1	4.34E-02	0.00E+00
2	CYTO	W5Q867	SLC12A2	8.01E-03	3.98E-04
2	CYTO	W5PSX7	FES	4.30E-02	0.00E+00
2	CYTO	W5P434	NAGPA	9.30E-03	0.00E+00
2	CYTO	W5PBE7	ELOA	2.42E-02	2.11E-04
2	CYTO	W5QG24	PPT1	2.36E-02	0.00E+00
2	CYTO	W5P1S3	SLC25A13	4.87E-02	0.00E+00
2	CYTO	W5Q3Y3	SFXN3	9.15E-03	0.00E+00
2	CYTO	W5PRK6	STAU1	5.67E-03	0.00E+00
2	CYTO	W5Q6V7	SIPA1	5.85E-03	0.00E+00
2	CYTO	W5Q6N7		3.90E-02	0.00E+00
2	CYTO	W5Q2H2	SLC12A9	1.18E-03	0.00E+00
2	CYTO	W5PFE6	ACOX1	3.15E-02	0.00E+00
2	CYTO	W5PWA8	HSPB1	7.30E-03	0.00E+00
2	CYTO	W5PWF2	ATP6V1H	2.74E-03	0.00E+00
2	CYTO	W5NYA8	SCAMP2	3.34E-02	0.00E+00
2	CYTO	W5PHY4	TYMS	4.35E-02	0.00E+00
2	CYTO	W5Q761	UBE2D2	4.40E-02	0.00E+00
2	CYTO	W5NSS1	PPP4R1	1.58E-02	0.00E+00
2	CYTO	W5PVM8	UBR7	1.85E-02	0.00E+00
2	CYTO	W5Q0Q5	ATP5PF	3.71E-02	0.00E+00
2	CYTO	W5PKK1	MX2	5.58E-02	0.00E+00
2	CYTO	W5PXC6		7.47E-04	0.00E+00
2	CYTO	W5QA59	HGS	3.72E-04	0.00E+00
2	CYTO	W5QH66		5.37E-04	2.16E-04
2	CYTO	W5PZG8	PECAM1	6.54E-03	0.00E+00
2	CYTO	W5Q560	OGFOD3	1.42E-02	0.00E+00
2	CYTO	W5Q2C9		1.16E-02	0.00E+00
2	CYTO	W5PFE7	ACOX1	6.61E-03	0.00E+00
2	CYTO	W5P958	IDH3G	5.25E-02	0.00E+00

Supplementary Table 4: Differentially abundant proteins (DAPs) of cMØ relative to M0 and their cellular location.

FoldChange	Location	Protein IDs	Gene Names	P-value	FDR
2	CYTO	W5P5I4	LAMTOR2	1.83E-03	0.00E+00
2	CYTO	W5Q850	AKAP10	1.88E-02	0.00E+00
2	CYTO	W5PG72	GOLM1	1.50E-02	2.18E-04
2	CYTO	W5QAS2	STX6	3.95E-02	0.00E+00
2	CYTO	W5Q430	IDUA	2.06E-03	2.10E-04
2	CYTO	W5QDJ4	GIGYF2	6.81E-04	0.00E+00
2	CYTO	W5QCM8	EPS15L1	2.88E-02	0.00E+00
2	CYTO	W5PFQ8	RRAGA	9.30E-03	0.00E+00
2	CYTO	W5Q4P9	CYRIA	1.23E-02	0.00E+00
2	CYTO	W5Q0M7	RAP2B	2.23E-02	0.00E+00
2	CYTO	W5PTG9	GC	2.64E-02	0.00E+00
2	CYTO	W5Q045	TMED3	5.87E-03	0.00E+00
2	CYTO	W5PSG0		3.11E-02	0.00E+00
2	CYTO	W5PES6		1.49E-02	0.00E+00
2	CYTO	W5PJY4	RAVER2	4.90E-03	2.18E-04
2	CYTO	W5P0C5	LGALS8	1.34E-02	0.00E+00
2	CYTO	W5PF18	LOC101102454	2.15E-02	0.00E+00
2	CYTO	W5Q0E7	LIN7C	5.60E-04	5.90E-03
2	CYTO	W5NUI3	TREM2	2.89E-02	0.00E+00
2	CYTO	W5P333		2.83E-02	0.00E+00
2	CYTO	W5P1M1	ETFDH	5.44E-02	0.00E+00
2	CYTO	W5P2N4	AHCYL2	4.90E-03	0.00E+00
2	CYTO	W5P9M6	LOC101116755	2.08E-02	0.00E+00
2	CYTO	W5NUQ8	GCC1	1.34E-02	0.00E+00
2	CYTO	W5PMB1	SNX3	2.88E-03	0.00E+00
2	CYTO	W5PNN3	SMAP1	1.72E-03	3.96E-04
2	CYTO	W5PI02	TBC1D13	8.66E-03	0.00E+00
2	CYTO	W5QHP5	GNB4	1.39E-02	0.00E+00
2	CYTO	W5QBQ8	RAB5C	3.99E-02	0.00E+00
2	CYTO	W5PGC1	CHMP4B	1.43E-03	0.00E+00
2	CYTO	W5NXP7	GMDS	3.97E-02	0.00E+00
2	CYTO	W5NRR6	MGAT2	5.29E-03	0.00E+00
2	CYTO	W5NUZ1	DYNC1I2	1.68E-02	0.00E+00
2	CYTO	W5QFL4	CSDE1	1.98E-04	0.00E+00
2	CYTO	W5PFR7	ETHE1	2.84E-03	0.00E+00
2	CYTO	W5PVT3	GALNS	5.52E-02	0.00E+00
2	CYTO	W5PSD7	RAP2C	1.28E-02	0.00E+00
2	CYTO	W5QG47	CMAS	1.93E-02	0.00E+00
2	CYTO	W5NZA6	RNF121	3.54E-02	0.00E+00
2	CYTO	W5P4K6	FCHO2	5.36E-02	0.00E+00
2	CYTO	W5NXL5	STX8	4.96E-02	0.00E+00
2	CYTO	W5NY14		6.46E-04	0.00E+00
2	CYTO	W5QHV6	SNX1	1.68E-02	0.00E+00
2	CYTO	W5PYA8	ANAPC1	8.58E-03	0.00E+00
2	CYTO	W5Q9I6	OCIAD1	5.57E-03	0.00E+00
2	CYTO	W5NQG1	SETD3	3.12E-02	0.00E+00
2	CYTO	W5PCN2	ANXA7	1.01E-03	0.00E+00
2	CYTO	W5Q0L4	SAT2	1.89E-02	0.00E+00
2	CYTO	W5Q418	NELFCD	3.60E-03	0.00E+00
2	CYTO	W5PZ62	ZFYVE16	1.01E-03	0.00E+00
2	CYTO	W5P7L2	ATP6V1G1	2.74E-03	0.00E+00
2	CYTO	W5PUW5	GOLGA5	1.78E-02	0.00E+00
2	CYTO	W5QD46	CYTH4	1.64E-02	0.00E+00
2	CYTO	W5Q0P4	MTMR9	5.51E-03	0.00E+00
2	CYTO	W5NPP9	PRKCD	1.28E-02	0.00E+00
2	CYTO	Q9XT28	ATOX1	2.18E-02	0.00E+00
2	CYTO	W5NZZ3	ATP6V1F	9.37E-03	0.00E+00
2	CYTO	Q6ECI6	ITGB2	3.66E-02	0.00E+00
2	CYTO	W5P6L1	RASGRP4	4.96E-02	0.00E+00
2	CYTO	W5PAZ0	RGCC	1.11E-03	2.05E-04
2	CYTO	W5PXH4	PTPRE	2.39E-02	0.00E+00
2	CYTO	W5NSQ8	LOC101103383	2.05E-03	6.24E-03
2	CYTO	W5NS94		4.27E-02	0.00E+00

Supplementary Table 4: Differentially abundant proteins (DAPs) of cMØ relative to M0 and their cellular location.

FoldChange	Location	Protein IDs	Gene Names	P-value	FDR
2	CYTO	W5PNW7	VIM	1.30E-04	0.00E+00
2	CYTO	W5QIX6	EXOC5	2.24E-02	0.00E+00
2	CYTO	W5NYG8	TSSC4	4.55E-02	0.00E+00
2	CYTO	W5PNS2	TLR7	4.42E-02	0.00E+00
2	CYTO	W5NU80	C13H20orf27	2.89E-02	0.00E+00
2	CYTO	W5Q8I6	POLDIP2	2.35E-02	0.00E+00
2	CYTO	W5PA59	LOC101117015	1.09E-03	0.00E+00
2	CYTO	W5PNC4	JPT1	7.50E-03	0.00E+00
2	CYTO	W5PBX4	FAR1	1.46E-02	3.92E-04
2	CYTO	W5PNX6	PREP	3.17E-02	0.00E+00
2	CYTO	W5PWZ2		1.45E-02	0.00E+00
2	CYTO	W5P6D3	SEMA4A	5.59E-02	0.00E+00
2	CYTO	W5PVQ4	TMEM120A	4.07E-02	0.00E+00
2	CYTO	W5Q6F3	USP30	1.87E-02	2.21E-04
2	CYTO	W5P5A7	VPS16	1.31E-03	0.00E+00
2	CYTO	W5P7L0	SUN1	1.34E-02	2.17E-04
2	CYTO	W5PF26	JAML	4.62E-03	0.00E+00
2	CYTO	W5PUH0	OTULINL	5.29E-03	2.08E-04
2	CYTO	W5NUX5		1.11E-02	0.00E+00
2	CYTO	W5NXR5	EDEM3	1.28E-02	2.07E-04
2	CYTO	W5NS73		1.68E-02	3.19E-03
2	CYTO	W5Q6A9	OGA	4.02E-02	0.00E+00
2	CYTO	W5PGR4	LOC101112287	5.09E-02	0.00E+00
2	CYTO	W5QGB6	GCHFR	2.23E-02	0.00E+00
2	CYTO	W5QDU7	RBKS	1.31E-02	0.00E+00
2	CYTO	W5QCA2	STXBP3	2.62E-02	0.00E+00
2	CYTO	W5Q740	ABCD3	2.34E-02	0.00E+00
2	CYTO	W5NXH3	LOC101106542	2.78E-02	0.00E+00
2	CYTO	W5QI21	TARS2	1.34E-02	0.00E+00
2	CYTO	W5NVG6	PTPN9	4.51E-02	0.00E+00
2	CYTO	W5PSE3	SH3TC1	3.25E-04	2.21E-04
2	CYTO	W5P8S0	LY96	1.00E-02	0.00E+00
2	CYTO	W5NZJ1	LOC101114075	4.19E-02	0.00E+00
2	CYTO	A2SW69	ANXA2	3.52E-03	0.00E+00
2	CYTO	W5PIL6	UCHL5	5.12E-02	0.00E+00
2	CYTO	W5Q9H8	SNX5	2.10E-02	0.00E+00
2	CYTO	W5Q532	PRDX4	2.11E-02	0.00E+00
2	CYTO	W5PZ05		1.15E-02	0.00E+00
2	CYTO	W5NZ42	SLC2A6	1.03E-02	0.00E+00
2	CYTO	W5PZI9	DHFR	2.80E-05	0.00E+00
2	CYTO	W5PDE3	SPTLC1	1.06E-02	0.00E+00
2	CYTO	W5PK12	OAT	2.70E-02	0.00E+00
2	CYTO	W5PF04	MAN1A1	4.28E-02	2.02E-04
2	CYTO	W5P3X8	KIF15	1.28E-04	0.00E+00
2	CYTO	W5PCM4	LUZP1	3.02E-02	0.00E+00
2	CYTO	W5PP02	MAP7D3	2.82E-02	6.95E-03
2	CYTO	W5PJG7	CALR	1.24E-02	0.00E+00
2	CYTO	W5PSV6	RABGGTB	1.38E-02	0.00E+00
2	CYTO	W5P0K3	MBNL1	4.87E-02	0.00E+00
2	CYTO	W5NZ02	INPP5F	1.95E-02	3.94E-04
-2	CYTO	C8BKC5	PRDX2	3.26E-02	0.00E+00
-2	CYTO	W5PKT0	NAA25	4.20E-02	1.90E-03
-2	CYTO	W5P8W5		5.15E-02	0.00E+00
-2	CYTO	W5NXN8	MTOR	4.15E-02	0.00E+00
-2	CYTO	C5IWU4	ARL3	1.83E-02	0.00E+00
-2	CYTO	W5PQ88	RPS6KB2	1.07E-02	0.00E+00
-2	CYTO	W5QB99	TSC22D3	3.41E-02	0.00E+00
-2	CYTO	W5NUX2		2.90E-02	0.00E+00
-2	CYTO	P80190		7.31E-03	0.00E+00
-2	CYTO	W5QFB8	SF3A3	3.39E-02	0.00E+00
-2	CYTO	W5Q768	CDS2	4.93E-02	0.00E+00
-2	CYTO	W5PE90	ITPR1	3.05E-02	0.00E+00
-2	CYTO	W5QB24	ANP32A	4.97E-02	0.00E+00

Supplementary Table 4: Differentially abundant proteins (DAPs) of cMØ relative to M0 and their cellular location.

FoldChange	Location	Protein IDs	Gene Names	P-value	FDR
-2	CYTO	W5QCZ0	MPST	3.02E-03	0.00E+00
-2	CYTO	W5PV48	USP11	4.89E-02	0.00E+00
-2	CYTO	W5PL90	MMS19	5.27E-02	0.00E+00
-2	CYTO	W5P3W2	PPP2R5D	3.83E-02	2.04E-04
-2	CYTO	W5NXF6	TUBG1	5.55E-02	0.00E+00
-2	CYTO	W5Q7Q6	EPHX1	2.20E-02	0.00E+00
-2	CYTO	W5Q7H5	HVCN1	1.42E-02	0.00E+00
-2	CYTO	W5PRY7	PXK	3.33E-02	0.00E+00
-2	CYTO	W5QIY4	ARID4A	4.36E-02	0.00E+00
-2	CYTO	W5NSK3		1.35E-02	0.00E+00
-2	CYTO	W5PQK6	TLN1	2.05E-02	0.00E+00
-2	CYTO	W5Q804	SPECC1	2.79E-02	0.00E+00
-2	CYTO	W5P762		1.20E-02	0.00E+00
-2	CYTO	W5PXS7	DAPP1	5.37E-02	0.00E+00
-2	CYTO	W5PCV5	RALGAPA1	1.92E-02	8.37E-03
-2	CYTO	W5PK62	CDC40	1.04E-02	0.00E+00
-2	CYTO	W5PI51	NDUFAF7	1.16E-02	0.00E+00
-2	CYTO	W5PKD2	GCDH	3.76E-02	0.00E+00
-2	CYTO	W5NQ72	PFAS	2.76E-03	0.00E+00
-2	CYTO	W5Q7D2	RBM45	3.18E-02	0.00E+00
-2	CYTO	W5PCZ2	PPP3CB	2.65E-02	0.00E+00
-2	CYTO	W5Q8W8	H3F3A	1.07E-02	0.00E+00
-2	CYTO	W5PHL8	NUDT16	3.02E-02	0.00E+00
-2	CYTO	W5QEQ3	APEX1	4.90E-02	0.00E+00
-2	CYTO	W5P7A6	ATE1	4.52E-02	0.00E+00
-2	CYTO	W5NTA4	CGGBP1	1.56E-02	0.00E+00
-2	CYTO	W5PJJ5	PGM2L1	2.26E-02	2.06E-04
-2	CYTO	W5P7P9	ERBIN	4.90E-02	0.00E+00
-2	CYTO	W5PZC3	STAMBPL1	5.09E-02	0.00E+00
-2	CYTO	W5PMI8	CUL5	6.34E-04	0.00E+00
-2	CYTO	W5QCC1	STAT5B	2.10E-02	0.00E+00
-2	CYTO	W5Q8P3	USE1	3.85E-03	0.00E+00
-2	CYTO	W5PSA9	VPS13A	4.49E-02	0.00E+00
-2	CYTO	W5PZ59	LOC101104574	4.01E-02	0.00E+00
-2	CYTO	W5Q088	CBL	2.39E-02	0.00E+00
-2	CYTO	W5PTN4	TMPO	1.89E-02	0.00E+00
-2	CYTO	W5P023		3.07E-02	0.00E+00
-2	CYTO	W5P0R7	DHODH	3.38E-02	2.17E-04
-2	CYTO	W5PTZ9	LOC101120877	4.79E-02	0.00E+00
-2	CYTO	W5PNT2	EXOSC7	4.77E-02	0.00E+00
-2	CYTO	W5Q9R2	SARM1	3.99E-02	0.00E+00
-2	CYTO	W5PTQ0	RNGTT	1.52E-02	2.05E-04
-2	CYTO	W5P2N8	PIK3C2A	4.70E-02	2.19E-04
-2	CYTO	W5PUI4	RAB3A	4.62E-02	2.15E-04
-2	CYTO	W5PAV5	RFX1	2.40E-02	0.00E+00
-2	CYTO	W5PUR1	CNOT11	7.17E-03	0.00E+00
-2	CYTO	W5PVX3	PRKG1	3.88E-02	0.00E+00
-2	CYTO	W5QAK2	IK	1.77E-02	0.00E+00
-3	CYTO	W5QFT3		3.60E-02	0.00E+00
-3	CYTO	W5PG02	RASA3	5.36E-03	0.00E+00
-3	CYTO	M4WG34	IL1b	3.74E-02	0.00E+00
-3	CYTO	W5P7B8	MTMR3	4.36E-02	0.00E+00
-3	CYTO	C5IS96	LCAT	1.72E-02	2.06E-04
-3	CYTO	W5NUE6	PLEKHA2	1.64E-02	0.00E+00
-3	CYTO	W5PGX7	SSRP1	1.73E-02	0.00E+00
-3	CYTO	W5Q9B6	INTS14	4.85E-02	9.70E-04
-3	CYTO	W5Q383	ARIH2	7.51E-03	0.00E+00
-3	CYTO	W5Q9R1	RTCA	8.14E-03	0.00E+00
-3	CYTO	W5PEA0	FGD3	4.25E-04	0.00E+00
-3	CYTO	W5Q7L1	CNN3	2.04E-02	0.00E+00
-3	CYTO	W5NTQ3	H3-4	4.80E-02	2.02E-04
-3	CYTO	W5PJL3		5.19E-02	0.00E+00
-3	CYTO	W5PI65	MAP4K2	4.72E-02	0.00E+00

Supplementary Table 4: Differentially abundant proteins (DAPs) of cMØ relative to M0 and their cellular location.

FoldChange	Location	Protein IDs	Gene Names	P-value	FDR
-3	CYTO	W5NXV1	PACS1	2.47E-02	0.00E+00
-3	CYTO	W5P795	LRBA	5.49E-02	0.00E+00
-3	CYTO	W5Q4B7	RAP1GDS1	9.67E-03	0.00E+00
-3	CYTO	W5PTS4	LOC101114275	2.93E-03	0.00E+00
-3	CYTO	W5NRR9	STAT5A	3.18E-02	0.00E+00
-3	CYTO	W5PPX0	LYPLAL1	4.85E-02	0.00E+00
-3	CYTO	W5QCX7	MYBBP1A	4.45E-02	0.00E+00
-3	CYTO	W5PWZ8	SNRNP200	4.28E-02	0.00E+00
-3	CYTO	W5PGU9	PLCG2	1.02E-02	0.00E+00
-3	CYTO	W5PP85	TBC1D10C	2.92E-02	0.00E+00
-3	CYTO	W5P6T0		2.86E-02	1.16E-03
-3	CYTO	W5NV79	NUP210	4.19E-02	0.00E+00
-3	CYTO	W5PAV0	STAP1	9.56E-03	0.00E+00
-3	CYTO	W5P9M9	LOC101103862	3.75E-02	0.00E+00
-3	CYTO	W5P691	CNTRL	2.92E-02	0.00E+00
-3	CYTO	W5PTQ7	TRAF3IP3	3.85E-02	0.00E+00
-3	CYTO	W5Q5E7	PYCR3	3.50E-02	0.00E+00
-3	CYTO	W5PYK8	VNN2	4.24E-03	0.00E+00
-3	CYTO	W5Q3J8		1.50E-02	0.00E+00
-3	CYTO	W5QH43	EIF4A2	1.36E-02	0.00E+00
-3	CYTO	W5NZT7	NMRAL1	3.29E-02	0.00E+00
-3	CYTO	W5PE21	PELP1	9.67E-03	0.00E+00
-3	CYTO	W5NRG0	PTGR2	5.45E-02	0.00E+00
-3	CYTO	W5P221	GLYR1	4.51E-02	0.00E+00
-3	CYTO	W5PBE0	CD74	2.47E-02	0.00E+00
-3	CYTO	W5Q550	LOC101109747	3.31E-02	0.00E+00
-3	CYTO	W5PZG0	ADD1	9.66E-03	0.00E+00
-3	CYTO	W5PKA9	F5	3.79E-04	0.00E+00
-3	CYTO	W5NT67	TUBGCP2	1.86E-02	2.06E-04
-3	CYTO	W5PP80	FRY	4.34E-02	1.16E-03
-4	CYTO	W5Q9H1	ZYX	2.27E-02	0.00E+00
-4	CYTO	W5Q6F0	LOC101106791	3.67E-02	0.00E+00
-4	CYTO	W5Q3I7	TUBB1	1.59E-02	0.00E+00
-4	CYTO	Q09YJ2	TES	4.73E-03	0.00E+00
-4	CYTO	W5PAF7	RSL1D1	1.24E-02	0.00E+00
-4	CYTO	W5QHL5	CAB39	8.84E-04	0.00E+00
-4	CYTO	W5P815	SEPTIN1	2.97E-02	0.00E+00
-4	CYTO	W5PHI5	PALM	2.58E-03	0.00E+00
-4	CYTO	W5QFR4	LOC101112856	3.27E-02	0.00E+00
-4	CYTO	W5Q059	SASH3	2.32E-02	0.00E+00
-4	CYTO	W5PA61		2.75E-02	0.00E+00
-4	CYTO	W5QDM7	ZNF512	3.26E-02	0.00E+00
-4	CYTO	W5P1J4	NCAPG	4.59E-02	2.18E-04
-4	CYTO	W5PU80	MS4A1	1.99E-02	0.00E+00
-4	CYTO	W5PEC3	ANXA6	1.60E-02	0.00E+00
-4	CYTO	W5PFJ0	VCL	1.67E-02	0.00E+00
-4	CYTO	W5PX46	LOC101122591	1.49E-02	0.00E+00
-4	CYTO	W5PLZ3	PACSIN1	1.61E-02	0.00E+00
-4	CYTO	W5QEL7	NDRG2	1.03E-02	7.84E-03
-4	CYTO	W5NXW9		2.76E-02	0.00E+00
-4	CYTO	W5NU34	TREML1	4.63E-02	0.00E+00
-4	CYTO	W5P0K2	HEATR1	3.32E-02	0.00E+00
-5	CYTO	W5NYC7	DQA	1.61E-02	0.00E+00
-5	CYTO	W5Q9S4	ING3	2.38E-02	1.16E-03
-5	CYTO	W5Q2S8	MYL9	2.97E-03	0.00E+00
-5	CYTO	W5P733	PDLIM1	1.96E-02	0.00E+00
-5	CYTO	W5NT35	LOC443162	5.07E-02	0.00E+00
-5	CYTO	W5Q0K9	ABLIM1	3.37E-02	0.00E+00
-5	CYTO	W5PF87	ALOX15	4.42E-02	0.00E+00
-5	CYTO	W5PJ75	SPTAN1	7.31E-04	0.00E+00
-5	CYTO	W5PYD8	PARP4	2.03E-05	9.21E-03
-6	CYTO	W5QIK8	SELENBP1	1.00E-02	0.00E+00
-8	CYTO	W5NZX9	SPTBN1	1.28E-03	0.00E+00

Supplementary Table 4: Differentially abundant proteins (DAPs) of cMØ relative to M0 and their cellular location.

FoldChange	Location	Protein IDs	Gene Names	P-value	FDR
-7	SEC	W5Q0D6	MSRA	3.456E-06	0
7	SEC	W5PDH4	MMP9	3.316E-05	0
-2	SEC	W5PWF2	ATP6V1H	6.671E-05	0
-5	SEC	W5PYT7		0.0001344	0
-3	SEC	W5NZ70	LGALS3BP	0.0002286	0
-7	SEC	W5P375	TCP1	0.0003361	0
-6	SEC	W5QAB1	HPX	0.0003761	0
-2	SEC	W5NYV5	SPAG9	0.0003907	0.0016038
-5	SEC	W5P887	CST3	0.0005378	0
-6	SEC	W5NUI0		0.0005746	0
-4	SEC	W5QAP8	RPRD1B	0.0006734	0.0008045
6	SEC	W5P3Q3	LOC100101238	0.0007534	0
-5	SEC	W5P4P3	PTPN6	0.0007858	0
-4	SEC	W5PJB6	PGM1	0.0009133	0
-4	SEC	W5P538	CD93	0.0011331	0
-4	SEC	W5P880	PRG4	0.0012021	0
-3	SEC	W5P8E9	CD109	0.0012785	0
-5	SEC	W5PG63	VWF	0.00154	0
-4	SEC	W5Q517	PCOLCE	0.0016444	0
7	SEC	P83205	CTSB	0.0017898	0
-3	SEC	W5P5M7	VPS26A	0.0021865	0
-5	SEC	W5P0Y1	RYR2	0.0022072	0.0038551
4	SEC	W5QHL1	FCGR1A	0.0024468	0
-4	SEC	W5QFH5	RAB1A	0.00292	0
5	SEC	W5PHJ9	LOC101119572	0.0035245	0
-4	SEC	W5Q3A5	PAFAH1B1 LIS1	0.004291	0
-3	SEC	W5NRL8	EIF3A EIF3S10	0.0057121	0
-8	SEC	W5PFJ0	VCL	0.006426	0
-5	SEC	W5P707	ACTN4	0.0064615	0
-7	SEC	W5QBD7	YWHAZ	0.0081056	0
-4	SEC	W5PZ86	SEPTIN8	0.008276	0
-5	SEC	W5PE22	GDI2	0.008307	0
5	SEC	Q9MZS8	CTSD	0.0102524	0
6	SEC	W5P1H0	CTSC	0.0103348	0
-3	SEC	W5PEL2	OGN	0.0103646	0.0044643
-5	SEC	W5Q6H1	PLS3	0.0103872	0
-7	SEC	W5P409	FERMT3	0.0106257	0
-5	SEC	W5P359	STIP1	0.0109009	0
-3	SEC	W5PVX8	PPP3CA	0.011495	0
-8	SEC	W5P5A0	FLNA	0.0115547	0
-4	SEC	W5PN69	LOC101109111	0.011612	0
-5	SEC	W5PVJ0		0.0119244	0
-3	SEC	W5PTZ7	PPP6C	0.0125678	0
-4	SEC	W5PGW8	XPNPEP1	0.0130355	0
-5	SEC	W5PVT6	UBA1	0.0138163	0
-6	SEC	C5ISA2	TUBA4A	0.0138851	0
-4	SEC	W5QD96	PARVB	0.0141673	0
-4	SEC	W5PJ98		0.0145626	0
-5	SEC	W5QHQ7	NCL	0.014696	0
-7	SEC	W5QAR2	F13A1	0.0147441	0
-4	SEC	W5Q9M6		0.0154763	0
-5	SEC	C5IJA0	RAN	0.0160291	0
3	SEC	W5QIW1	LGALS3	0.0165207	0
-4	SEC	W5PUT6	CLTC	0.0171512	0
-5	SEC	W5Q5H8	FGA	0.017343	0
4	SEC	W5QI70	CTSS	0.0175956	0
-3	SEC	W5PYQ9	CPNE1	0.0181381	0
-4	SEC	W5PJC2	PSMB3	0.0181787	0
-4	SEC	W5QD49	PSME1	0.0184395	0
-4	SEC	W5PZ15		0.0184926	0
-3	SEC	W5P6M2	PSMD5	0.0196242	0
2	SEC	W5NYP8	DYNC1H1	0.0200018	0
-5	SEC	W5PPT6	TUBB	0.0202155	0

Supplementary Table 4: Differentially abundant proteins (DAPs) of cMØ relative to M0 and their cellular location.

FoldChange	Location	Protein IDs	Gene Names	P-value	FDR
-5	SEC	W5PY17	STX7	0.0202817	0
-5	SEC	W5PK95	HNRNPA2B1	0.0207336	0
-4	SEC	W5NS17	ENOPH1	0.0209064	0
-3	SEC	W5NSA6	LOC101122940	0.0210845	0
3	SEC	W5NSH8	NPC2	0.0216167	0
-7	SEC	W5PE01	TAGLN2	0.0221685	0
-4	SEC	W5PSM0	OSTF1	0.022203	0
-4	SEC	W5NUT8	PIP4K2A	0.0225781	0
-3	SEC	W5PNV0	RPL12	0.0227652	0
-3	SEC	W5QB88	COL11A1	0.0232727	0.0044843
-4	SEC	W5NQK6	LIMS1	0.0233018	0
-2	SEC	W5Q1R8	MAPK1	0.0234022	0.0044412
-6	SEC	W5PQA8	PRDX6	0.0242484	0
-5	SEC	W5P2V0	EZR	0.0247059	0
-6	SEC	W5P098	CORO1A	0.0248764	0
-4	SEC	W5NVE5	WARS1	0.0249227	0
4	SEC	W5NY01		0.0263331	0
-4	SEC	W5P6Z6	MAPRE2	0.0269221	0
-5	SEC	W5PE27	ESD	0.0275785	0
-6	SEC	W5QG16	CAP1	0.0278407	0
3	SEC	W5NWF5	RARRES1	0.027947	0
-4	SEC	W5NY22	PCMT1	0.0280289	0
-4	SEC	W5PPG3	ALDH9A1	0.029273	0
-3	SEC	W5NPQ6	CAPZA2	0.0294687	0
-7	SEC	W5Q3I7	TUBB1	0.0298891	0
-5	SEC	W5Q8S1	SEPTIN2	0.0299094	0
-3	SEC	W5QEU6	ANXA4	0.0302041	0
-5	SEC	W5QEK8	EHD3	0.0303941	0
-5	SEC	W5P4C7	SEPTIN7	0.0303955	0
-3	SEC	W5QAA3	LOC101114226	0.0307147	0.0045593
-3	SEC	W5QHE9	STRAP	0.0307485	0
-5	SEC	Q28554	GAPDH G3PDH GA	0.0311249	0
2	SEC	Q29524	LPL	0.0313585	0
-3	SEC	W5NZ74	HK3	0.0317066	0
3	SEC	W5Q6V2	KRT7	0.0320716	0
-5	SEC	W5PX84	CCDC171	0.0324646	0.0045662
-5	SEC	W5Q4L0		0.0328727	0
-3	SEC	W5PIM8	GALK1	0.0329465	0
-4	SEC	W5P733	PDLIM1	0.033222	0
-3	SEC	W5PNY5	ARRB1	0.0336723	0
-2	SEC	W5Q038	AHNAK	0.0338951	0
-7	SEC	W5Q9H1	ZYX	0.0343394	0
-4	SEC	W5PIN6	LDHA	0.0346136	0
-4	SEC	W5PQK3	PFKL	0.034741	0
-4	SEC	W5PRR5	LTA4H	0.0350759	0
-4	SEC	W5NZ57	PSMB10	0.0366266	0
-6	SEC	W5PFI7	VCL	0.0381159	0
3	SEC	W5PSL9	HPD	0.0381293	0.0044477
-3	SEC	W5P6F9	DPP3	0.038131	0
4	SEC	W5PEB0	FABP7	0.0381551	0
-7	SEC	W5PQK6	TLN1	0.0385757	0
2	SEC	W5PF65	TF	0.0393293	0
-5	SEC	W5PG95	HSPA1A	0.0397301	0
-3	SEC	W5PUG1	MST1	0.0404942	0
-4	SEC	W5PE11	CDC42	0.0408133	0
-5	SEC	W5QE14	PCBP1	0.0412291	0
-3	SEC	W5PC25	LOC101116273	0.0420724	0
-4	SEC	W5PLD5	CNN2	0.0421143	0
-3	SEC	W5Q701	LOC101112491	0.0427012	0
-5	SEC	W5PXX7	CCT5	0.0430579	0
-5	SEC	W5PL19	LOC101105123	0.0432685	0
-3	SEC	W5Q5R8	TXNRD1	0.0446028	0
-4	SEC	C5IWU0	ARF1 LOC1011231	0.0447242	0

Supplementary Table 4: Differentially abundant proteins (DAPs) of cMØ relative to M0 and their cellular location.

FoldChange	Location	Protein IDs	Gene Names	P-value	FDR
-6	SEC	W5PD82	CALD1	0.0448012	0
-6	SEC	W5P627	GSN	0.0456974	0
-6	SEC	W5QIK8	SELENBP1	0.0459353	0
-4	SEC	W5P3N0	MAPRE1	0.0462064	0
-2	SEC	W5NX31	AHSA1	0.0463359	0
-5	SEC	W5PQF0	CCT7	0.0463968	0
-5	SEC	W5PNU4	LOC101111106	0.0465106	0
-4	SEC	W5PK66	PARK7	0.0474479	0
-3	SEC	W5P5F6	TBCB	0.047831	0.0045627
-3	SEC	B7TJ15	MAPK14	0.0485214	0
-5	SEC	W5PEX2	GPATCH8	0.048958	0
-3	SEC	W5PSM6	HABP2	0.0493334	0
-5	SEC	W5Q2S8	MYL9	0.0495095	0
-3	SEC	W5PTA9	DCPS	0.050086	0
-4	SEC	P00349	PGD	0.0509381	0
-4	SEC	W5P500	PSMB1	0.0513823	0
-4	SEC	W5P323	GPI	0.05185	0
-3	SEC	W5PLS7	GRB2	0.0523322	0
-5	SEC	W5QBV3	PGK1	0.052475	0
-3	SEC	W5NU76	FMOD	0.0531053	0
-6	SEC	W5NYA7	LOC101114319	0.0531338	0
-5	SEC	W5PRJ4	VCP	0.0536838	0
-5	SEC	W5NXA3	HELQ	0.0538108	0.0098814
-4	SEC	W5PK85	EML2	0.0546586	0
-2	SEC	W5PI22	CNPY2	0.0546659	0
-4	SEC	W5PYQ7	RPS12	0.05472	0
-5	SEC	W5PH15	RSU1	0.0550189	0
-2	SEC	W5PMH1	GSS	0.0550832	0
-4	SEC	W5Q6U0	FASN	0.0558969	0
-4	SEC	W5Q3M9	SEPTIN6	0.0559616	0

cMØ, monocyte-derived macrophages differentiated without the addition of exogenous growth factors such as M-CSF or GM-CSF and M0, Monocytes at 3 hours. Differentially abundant proteins (DAPs) were identified using a threshold of false discovery rate (FDR, q-value) ≤ 0.05 and absolute fold change ≥ 2. Red-highlighted cells indicate shared differentially abundant proteins (DAPs) between the cell lysate (CYTO) and secretome (SEC) compartments in cMØ relative to monocytes, whereas non-highlighted cells represent compartment-specific DAPs unique to either the whole cell lysate or secretome.

Supplementary Table 5: Differentially abundant proteins (DAPs) of GMØ relative to M0 and their cellular location.

FoldChange	Location	Protein IDs	Gene Names	P-value	FDR
11	CYTO	W5PEB0	FABP7	2.16E-06	0.00E+00
9	CYTO	W5NU86	GLA	9.34E-04	0.00E+00
9	CYTO	W5PCH3	SCIN	1.19E-04	0.00E+00
8	CYTO	W5NY01		5.10E-04	0.00E+00
8	CYTO	W5PAM4	CTSA	8.29E-05	0.00E+00
8	CYTO	W5PEL7	HSPG2	1.73E-05	0.00E+00
8	CYTO	W5PF33	GM2A	7.42E-06	0.00E+00
8	CYTO	W5PT76	GPNMB	1.60E-04	0.00E+00
8	CYTO	P51977	ALDH1A1 ALDH1	1.55E-03	0.00E+00
8	CYTO	A9YUY8	FABP4	5.78E-04	0.00E+00
8	CYTO	W5PRI6	MRC1	1.15E-04	0.00E+00
8	CYTO	W5PFV1	CTSL	4.03E-04	0.00E+00
7	CYTO	W5QHG1	EPS8	3.89E-05	0.00E+00
7	CYTO	W5QBA2	ATRX	1.18E-04	0.00E+00
7	CYTO	W5P9J8	BLVRB	2.18E-04	0.00E+00
7	CYTO	W5PKQ7		6.16E-05	6.96E-03
7	CYTO	W5PBM9	SCPEP1	1.95E-04	0.00E+00
7	CYTO	W5PE67		6.85E-05	0.00E+00
7	CYTO	W5PIQ6	MSR1	6.63E-05	0.00E+00
7	CYTO	W5PFY5	ASAH1	1.46E-03	0.00E+00
7	CYTO	W5PUP6	SPATA13	5.44E-02	2.82E-03
7	CYTO	W5PVR2	LOC101108019	6.26E-05	7.84E-03
7	CYTO	W5PJS4	EMILIN2	2.03E-04	0.00E+00
7	CYTO	W5QHL1	FCGR1A	1.33E-04	0.00E+00
6	CYTO	W5P2F1	FOLR2	1.68E-05	0.00E+00
6	CYTO	W5P9G8	PLD3	1.79E-02	0.00E+00
6	CYTO	W5PZB2	CD68	2.07E-04	0.00E+00
6	CYTO	W5PQR0	NIBAN2	7.63E-06	0.00E+00
6	CYTO	W5PI56	DAB2	1.53E-03	0.00E+00
6	CYTO	W5P1A5	GBA1	2.78E-04	0.00E+00
6	CYTO	W5PGC5	GALM	1.08E-03	0.00E+00
6	CYTO	W5Q233	VCAN	1.36E-04	0.00E+00
6	CYTO	P35623	SHMT1	1.54E-04	0.00E+00
6	CYTO	Q9MZS8	CTSD	1.26E-03	0.00E+00
6	CYTO	W5PCE0	PLBD2	4.97E-03	0.00E+00
6	CYTO	W5QC89	HEXA	1.92E-04	0.00E+00
6	CYTO	W5P9M8	UCK1	2.80E-05	2.22E-04
6	CYTO	W5NVW7	NAGLU	2.94E-03	0.00E+00
6	CYTO	W5Q3S9	DSN1	1.59E-03	9.70E-04
6	CYTO	W5PE73	SMPDL3A	1.01E-05	0.00E+00
6	CYTO	W5PUL5	FCGRT	5.02E-04	0.00E+00
6	CYTO	P79365	SLC2A1 GLUT1	1.82E-04	5.18E-03
6	CYTO	W5PV43	LRPAP1	3.00E-04	0.00E+00
6	CYTO	W5PAQ4	FUCA1	3.63E-04	0.00E+00
6	CYTO	G3M9U4	ACP5	3.04E-04	0.00E+00
6	CYTO	W5NTJ2	TPX2	4.04E-05	5.72E-03
6	CYTO	W5Q2Y1	PLXNC1	7.56E-03	0.00E+00
6	CYTO	W5PKY1	HNMT	8.05E-04	0.00E+00
6	CYTO	W5PXR1	ENPP1	6.24E-04	0.00E+00
6	CYTO	W5Q940	SHTN1	3.35E-03	0.00E+00
6	CYTO	W5P093	NQO1	4.89E-06	0.00E+00
6	CYTO	W5PBS4	LRP1	3.79E-04	0.00E+00
5	CYTO	W5P0K8	CLIC2	2.22E-02	0.00E+00
5	CYTO	W5Q6N3	LOC101115115	3.12E-04	0.00E+00
5	CYTO	W5QCD6	IDH1	4.42E-05	0.00E+00
5	CYTO	W5Q612	TRPV2	4.37E-05	0.00E+00
5	CYTO	W5Q0A3	TLR2	8.55E-04	0.00E+00
5	CYTO	W5NYU9	MPP1	1.21E-04	0.00E+00
5	CYTO	W5NTZ3	RENBP	6.63E-04	0.00E+00
5	CYTO	W5NUI6	SGSH	3.56E-04	0.00E+00
5	CYTO	W5Q8D1	WDR74	2.34E-04	0.00E+00
5	CYTO	W5PNP1	MFGE8	5.07E-03	0.00E+00
5	CYTO	W5QFA5		5.82E-04	2.03E-04

Supplementary Table 5: Differentially abundant proteins (DAPs) of GMØ relative to M0 and their cellular location.

FoldChange	Location	Protein IDs	Gene Names	P-value	FDR
5	CYTO	W5QI12	TLN2	3.36E-04	5.17E-03
5	CYTO	W5QGH9	WDR3	4.25E-03	2.09E-04
5	CYTO	W5NRS0		2.55E-04	0.00E+00
5	CYTO	W5PA90	AGA	1.05E-04	0.00E+00
5	CYTO	W5P1H0	CTSC	2.07E-06	0.00E+00
5	CYTO	W5NSH8	NPC2	1.25E-04	0.00E+00
5	CYTO	W5QDW4	USF3	7.93E-03	9.68E-04
5	CYTO	W5NVC8	NLRP2	7.70E-04	9.05E-03
5	CYTO	W5P640	LMNA	3.32E-03	0.00E+00
5	CYTO	W5PWX3	CRYL1	4.62E-04	0.00E+00
5	CYTO	W5PPX2	SENP8	2.45E-04	3.01E-03
5	CYTO	W5QIW1	LGALS3	6.10E-03	0.00E+00
5	CYTO	W5PKU3		8.59E-04	0.00E+00
5	CYTO	W5PZ47	CTSH	5.50E-02	0.00E+00
5	CYTO	W5NYL0	MAOA	1.80E-03	0.00E+00
5	CYTO	W5QI00	LACTB	4.15E-04	0.00E+00
5	CYTO	W5NRB6	MIDEAS	1.73E-03	2.18E-04
5	CYTO	W5QBZ7	NAGA	3.91E-03	0.00E+00
5	CYTO	W5PQA6	CYFIP2	1.70E-05	0.00E+00
5	CYTO	W5PAC2	LOC101105044	8.15E-03	0.00E+00
5	CYTO	W5PNZ7	RFC1	3.86E-04	0.00E+00
5	CYTO	W5QI70	CTSS	1.69E-05	0.00E+00
5	CYTO	W5PUH5	LGMN	9.61E-03	0.00E+00
5	CYTO	W5Q2B3	SUSD5	1.09E-03	8.55E-03
5	CYTO	W5P3H8	IGF2R	5.60E-04	0.00E+00
5	CYTO	W5P026	STAB1	3.36E-04	0.00E+00
5	CYTO	W5NUC8	ARMCX3	2.69E-04	0.00E+00
5	CYTO	W5PD62	CPB2	9.40E-04	9.07E-03
5	CYTO	W5PG74	IRGQ	3.40E-04	0.00E+00
5	CYTO	W5PYW0	TCIRG1	6.60E-05	0.00E+00
5	CYTO	W5PXS1	RAB3IL1	1.37E-03	0.00E+00
5	CYTO	W5PDH7	NPC1	2.77E-04	0.00E+00
5	CYTO	P83205	CTSB	2.37E-03	0.00E+00
5	CYTO	W5Q284	HAPLN1	4.64E-04	0.00E+00
5	CYTO	W5P8H9	SGPL1	1.55E-03	0.00E+00
5	CYTO	W5NSN4		1.79E-03	2.22E-04
5	CYTO	W5P530	LOC101104705	3.11E-02	0.00E+00
5	CYTO	W5NX16		3.63E-04	0.00E+00
5	CYTO	W5PUW3		1.25E-03	0.00E+00
5	CYTO	W5Q4B1	MYCBP2	3.55E-04	0.00E+00
5	CYTO	W5QGG0	TFRC	7.17E-05	0.00E+00
5	CYTO	W5Q5W2	LOC101110539	6.12E-05	0.00E+00
5	CYTO	W5Q4U5	CPT1A	1.08E-03	0.00E+00
5	CYTO	W5PDH4	MMP9	2.29E-03	0.00E+00
5	CYTO	W5Q263	ICAM1	4.27E-04	0.00E+00
5	CYTO	W5P949	FILIP1	4.46E-04	3.93E-03
5	CYTO	W5PK56	FCGR2B	3.76E-02	0.00E+00
5	CYTO	W5NZ40	SGF29	3.89E-02	2.15E-04
5	CYTO	W5NQS7	IFNGR1	6.38E-04	0.00E+00
5	CYTO	W5PZG5	OCRL	1.25E-02	0.00E+00
5	CYTO	W5NTW3	ITIH1	8.51E-04	0.00E+00
5	CYTO	W5P8M9		2.60E-03	0.00E+00
5	CYTO	W5PE92	GRN	7.34E-04	0.00E+00
5	CYTO	W5PVN6		4.36E-04	5.90E-03
5	CYTO	W5Q1W7	PALLD	4.63E-04	0.00E+00
5	CYTO	W5QBM4	ALCAM	1.09E-03	0.00E+00
5	CYTO	W5NX56	SPP1	4.99E-04	0.00E+00
5	CYTO	W5NZ62	GNS	5.54E-03	0.00E+00
4	CYTO	W5P3S0		8.97E-06	0.00E+00
4	CYTO	W5NU23	FUCA2	4.09E-04	0.00E+00
4	CYTO	W5Q7I8	TAX1BP3	4.50E-02	0.00E+00
4	CYTO	W5Q3N1	CTSZ	3.22E-05	0.00E+00
4	CYTO	W5NZK6	PLA2G15	3.02E-03	0.00E+00

Supplementary Table 5: Differentially abundant proteins (DAPs) of GMØ relative to M0 and their cellular location.

FoldChange	Location	Protein IDs	Gene Names	P-value	FDR
4	CYTO	W5P407		1.20E-02	0.00E+00
4	CYTO	W5NZ47	RBP4	1.01E-02	0.00E+00
4	CYTO	W5Q678	SEC24D	8.44E-03	0.00E+00
4	CYTO	W5PSE5	LOC101110434	2.93E-03	2.05E-04
4	CYTO	W5QBE4	FGL2	2.00E-03	0.00E+00
4	CYTO	W5PJT1	PLAC9	2.87E-03	0.00E+00
4	CYTO	W5PRG8	CREG1	1.93E-03	0.00E+00
4	CYTO	W5PNY4	PDXK	1.67E-02	0.00E+00
4	CYTO	W5QH68	LRRC57	3.11E-04	2.05E-04
4	CYTO	P05028	ATP1B1	5.99E-03	0.00E+00
4	CYTO	W5P5W6	NDRG1	2.55E-02	0.00E+00
4	CYTO	W5QH35	CAPG	4.88E-04	0.00E+00
4	CYTO	W5QAB1	HPX	3.14E-03	0.00E+00
4	CYTO	W5QEM8	LOC101111528	1.37E-03	0.00E+00
4	CYTO	W5QBG8	PPFIA1	2.96E-03	0.00E+00
4	CYTO	W5Q3U3	LOC101102156	8.12E-03	0.00E+00
4	CYTO	W5QCL8	NPL	2.47E-03	0.00E+00
4	CYTO	W5PLB8	EPB41L3	4.44E-04	0.00E+00
4	CYTO	W5PP04	GNG12	7.65E-04	0.00E+00
4	CYTO	W5NYE0	ATP6V0D1	4.08E-02	0.00E+00
4	CYTO	Q29524	LPL	7.86E-03	0.00E+00
4	CYTO	W5PBC0		5.75E-03	0.00E+00
4	CYTO	W5PGS4	FABP5	1.12E-03	0.00E+00
4	CYTO	W5QI78	CTSK	3.24E-02	0.00E+00
4	CYTO	W5PY08		1.11E-02	0.00E+00
4	CYTO	W5Q700	APPL2	2.64E-04	0.00E+00
4	CYTO	W5PIS6	NHLRC3	1.94E-03	0.00E+00
4	CYTO	W5PYI8	WWC1	1.34E-02	0.00E+00
4	CYTO	W5PEE9	LAMP1	1.34E-02	0.00E+00
4	CYTO	W5P7G4	TNC	8.62E-03	0.00E+00
4	CYTO	W5PMA0	AP2S1	4.96E-03	0.00E+00
4	CYTO	W5PII2	ATG4C	6.05E-04	0.00E+00
4	CYTO	W5P3N6	LOC101112162	1.35E-03	0.00E+00
4	CYTO	W5QEH8	CTTNBP2NL	6.67E-05	0.00E+00
4	CYTO	W5P150		2.16E-03	2.21E-04
4	CYTO	W5Q8J3	RRBP1	8.72E-03	0.00E+00
4	CYTO	W5PTU7	CA2	1.03E-03	0.00E+00
4	CYTO	W5NW80	GAA	3.15E-03	0.00E+00
4	CYTO	W5Q318	CCNH	2.56E-04	4.29E-03
4	CYTO	W5NWX7	CLEC4A	3.76E-03	0.00E+00
4	CYTO	W5P3L5	RNF13	2.00E-05	0.00E+00
4	CYTO	W5Q0U0	P2RX4	1.83E-03	0.00E+00
4	CYTO	W5QA16		7.75E-04	0.00E+00
4	CYTO	Q9MZD1	SLC17A5	1.04E-03	0.00E+00
4	CYTO	W5PD43	HTRA1	2.69E-02	0.00E+00
4	CYTO	W5P2V3	PEPD	5.51E-03	0.00E+00
4	CYTO	W5QHU8	FNDC3B	7.39E-05	0.00E+00
4	CYTO	W5NVR9	C21H11orf54	1.19E-04	0.00E+00
4	CYTO	O18882	ATP6V0C ATP6C AT	4.30E-03	0.00E+00
4	CYTO	W5QI36	HEBP1	1.78E-02	0.00E+00
4	CYTO	W5Q5C8	SOAT1	6.87E-04	0.00E+00
4	CYTO	W5P6H9	ACP2	4.54E-02	0.00E+00
4	CYTO	C5IJ93	RAB9A	1.95E-03	0.00E+00
4	CYTO	W5Q5N6	BST-2B	2.81E-03	0.00E+00
4	CYTO	W5P5L4	COG5	4.69E-04	0.00E+00
4	CYTO	W5Q6Q3	TCP11L1	6.62E-04	8.73E-03
4	CYTO	W5PG10	PAPSS1	3.19E-02	0.00E+00
4	CYTO	W5PBR7	P4HA1	7.68E-03	0.00E+00
4	CYTO	W5Q9M9	GK	2.64E-02	0.00E+00
4	CYTO	W5PR99	DHPS	2.23E-04	3.98E-04
4	CYTO	W5Q3V0	IL18	5.04E-04	0.00E+00
4	CYTO	W5PN31		7.34E-05	7.77E-04
4	CYTO	W5NWX4		1.26E-02	0.00E+00

Supplementary Table 5: Differentially abundant proteins (DAPs) of GMØ relative to M0 and their cellular location.

FoldChange	Location	Protein IDs	Gene Names	P-value	FDR
4	CYTO	W5QI40	MYO1E	1.62E-02	0.00E+00
4	CYTO	W5NZV3	HMOX2	3.98E-02	0.00E+00
4	CYTO	W5PEY2	CTSV	3.00E-04	0.00E+00
4	CYTO	W5QB71	AMDHD2	2.58E-03	0.00E+00
4	CYTO	W5QDF4	GSTM3	2.24E-03	0.00E+00
4	CYTO	W5PG41	H6PD	2.21E-03	0.00E+00
4	CYTO	W5P703	WFS1	7.11E-03	0.00E+00
4	CYTO	P12303	TTR	2.19E-02	0.00E+00
4	CYTO	W5P6L1	RASGRP4	1.91E-03	0.00E+00
4	CYTO	W5PRG9	HAL	4.00E-02	0.00E+00
4	CYTO	W5QFU4		2.18E-02	0.00E+00
4	CYTO	W5PB38		2.12E-03	0.00E+00
4	CYTO	W5Q723	BHMT	2.22E-02	0.00E+00
4	CYTO	W5PH35	LOC101119706	1.03E-03	0.00E+00
4	CYTO	W5Q0F1	LIPA	1.58E-03	0.00E+00
4	CYTO	P82197	PDXK PKH	1.19E-02	0.00E+00
4	CYTO	W5PCE8	ATP2B3	1.38E-02	5.18E-03
4	CYTO	W5P168	SH3PXD2B	4.09E-03	0.00E+00
4	CYTO	W5P8N6	GAB2	6.08E-04	0.00E+00
4	CYTO	W5PVC8	ERGIC3	2.85E-03	0.00E+00
4	CYTO	W5QEH0	TWF1	5.53E-03	0.00E+00
4	CYTO	W5NPP2	CPM	2.86E-02	0.00E+00
4	CYTO	W5QBP6	GLUL	2.33E-02	0.00E+00
4	CYTO	W5PZ94	ACO1	3.12E-02	0.00E+00
4	CYTO	W5PHJ3	AHR	2.54E-03	0.00E+00
4	CYTO	W5Q6K0	HEATR3	8.44E-04	2.46E-03
4	CYTO	W5Q3H9	UPP1	3.46E-02	0.00E+00
3	CYTO	W5Q1K4	DICER1	4.75E-02	0.00E+00
3	CYTO	W5QHR5	PLEKHO2	7.08E-05	0.00E+00
3	CYTO	W5Q6S3	WASHC4	2.10E-03	0.00E+00
3	CYTO	W5PVM8	UBR7	4.48E-03	0.00E+00
3	CYTO	W5PCI1	NLRP3	1.20E-03	0.00E+00
3	CYTO	W5NS94		3.82E-05	0.00E+00
3	CYTO	W5PMX5	C5AR1	9.17E-03	0.00E+00
3	CYTO	W5PCD5	ARMC8	1.46E-02	0.00E+00
3	CYTO	W5P0C5	LGALS8	1.10E-04	0.00E+00
3	CYTO	W5PJN7	HOMER3	6.91E-04	0.00E+00
3	CYTO	W5PB61		3.01E-03	2.16E-04
3	CYTO	W5PMU8	GAB1	5.31E-03	0.00E+00
3	CYTO	W5Q2R5	SDF2L1	4.09E-02	0.00E+00
3	CYTO	W5QA17	ATXN2	3.29E-02	0.00E+00
3	CYTO	W5PE91	LOC101105400	1.07E-03	2.05E-04
3	CYTO	W5NUE5	SLC38A7	1.05E-03	0.00E+00
3	CYTO	W5PCA0	ALDOB	2.81E-03	0.00E+00
3	CYTO	W5NT90	BAG4	2.68E-02	0.00E+00
3	CYTO	W5P4A8	RNASET2	1.27E-03	0.00E+00
3	CYTO	W5Q6C5	GGA2	1.04E-02	0.00E+00
3	CYTO	W5P3W6	OSBPL9	1.42E-04	0.00E+00
3	CYTO	W5NRW1	FBXO6	4.19E-02	0.00E+00
3	CYTO	W5PS40		1.14E-03	2.13E-04
3	CYTO	W5P4X6	LOC101104287	1.08E-02	0.00E+00
3	CYTO	W5P366	FAM50A	2.50E-02	0.00E+00
3	CYTO	W5QDH3	COL2A1	3.29E-03	1.72E-03
3	CYTO	W5PGF4	PLAUR	1.28E-02	0.00E+00
3	CYTO	W5PBL5	SLC44A1	2.74E-04	0.00E+00
3	CYTO	W5Q8I7	LOC101122123	4.42E-03	0.00E+00
3	CYTO	W5Q777	HS1BP3	9.75E-04	0.00E+00
3	CYTO	W5PPM6	PQBP1	5.41E-03	2.03E-04
3	CYTO	W5QCG9	SRXN1	2.96E-02	0.00E+00
3	CYTO	W5Q2U7	PLEC	1.24E-05	0.00E+00
3	CYTO	W5NRL0	PLCD1	5.12E-04	0.00E+00
3	CYTO	W5Q5P5	GGH	1.42E-02	0.00E+00
3	CYTO	W5QHZ0	CA12	5.02E-03	0.00E+00

Supplementary Table 5: Differentially abundant proteins (DAPs) of GMØ relative to M0 and their cellular location.

FoldChange	Location	Protein IDs	Gene Names	P-value	FDR
3	CYTO	W5P2N4	AHCYL2	5.96E-03	0.00E+00
3	CYTO	W5PT36	RBM47	1.50E-03	0.00E+00
3	CYTO	W5PXX3	F13B	3.91E-02	7.13E-03
3	CYTO	W5PP47		2.25E-04	0.00E+00
3	CYTO	W5NR40	FLT4	1.46E-03	0.00E+00
3	CYTO	W5Q1M0	GLB1	3.25E-04	0.00E+00
3	CYTO	W5QHX1	EIF5A2	4.05E-03	0.00E+00
3	CYTO	W5P6V4	GLG1	5.59E-02	0.00E+00
3	CYTO	Q10994	CSTB CST6	1.93E-04	0.00E+00
3	CYTO	W5PKA1	LOC101116157	3.77E-02	0.00E+00
3	CYTO	W5PK31	FCGR3A	3.74E-04	0.00E+00
3	CYTO	W5Q0G8	IMPA1	4.63E-03	0.00E+00
3	CYTO	W5NXH3	LOC101106542	6.12E-04	0.00E+00
3	CYTO	W5PYB9	ABCA6	1.41E-03	0.00E+00
3	CYTO	W5Q430	IDUA	2.39E-03	2.10E-04
3	CYTO	W5PNK3	ACAN	1.41E-03	0.00E+00
3	CYTO	W5Q3C2	EPPK1	6.73E-03	0.00E+00
3	CYTO	W5PWI4	RGN	6.88E-03	0.00E+00
3	CYTO	W5NSA6	LOC101122940	1.94E-02	0.00E+00
3	CYTO	W5P2W1	NEU1	4.05E-03	0.00E+00
3	CYTO	W5NUJ5		3.22E-02	0.00E+00
3	CYTO	W5P800	GAS7	5.08E-02	0.00E+00
3	CYTO	W5QH13	VPS39	1.42E-03	0.00E+00
3	CYTO	W5PDZ1		1.34E-03	0.00E+00
3	CYTO	W5PKF9	FIG4	1.63E-03	0.00E+00
3	CYTO	W5NT19	SPAG1	2.26E-03	0.00E+00
3	CYTO	W5NYL7	MTHFD1L	1.03E-02	0.00E+00
3	CYTO	W5QG92	OSBPL11	1.35E-03	0.00E+00
3	CYTO	W5Q7R3	LOC101118736	2.25E-02	0.00E+00
3	CYTO	W5PPT8	GLIPR2	7.74E-03	0.00E+00
3	CYTO	W5P3B0	INPPL1	2.88E-02	0.00E+00
3	CYTO	W5QBR5	BMP2K	8.03E-03	0.00E+00
3	CYTO	W5PEC0	PKIB	4.96E-02	0.00E+00
3	CYTO	W5P7Y8	PALD1	1.50E-03	0.00E+00
3	CYTO	W5QHF2	LOC114117536	7.47E-04	0.00E+00
3	CYTO	W5P3I5	CNDP2	4.00E-04	0.00E+00
3	CYTO	W5NSQ3	HFE	1.14E-03	0.00E+00
3	CYTO	W5Q8G9	SPG21	2.54E-03	0.00E+00
3	CYTO	W5PUW2	IFI30	1.29E-03	0.00E+00
3	CYTO	W5PNS2	TLR7	2.27E-02	0.00E+00
3	CYTO	W5PGL9		2.55E-03	0.00E+00
3	CYTO	W5Q463	INTU	5.55E-02	9.90E-03
3	CYTO	W5PIE4	CLPTM1	5.17E-02	0.00E+00
3	CYTO	W5P5Q2	MVP	4.98E-02	0.00E+00
3	CYTO	W5NZJ7	FGR	4.36E-02	0.00E+00
3	CYTO	W5PYH9		3.05E-02	0.00E+00
3	CYTO	W5Q686	TPP1	2.46E-03	0.00E+00
3	CYTO	W5P4K6	FCHO2	1.75E-03	0.00E+00
3	CYTO	W5PLL2	F9	4.38E-03	0.00E+00
3	CYTO	W5PDK4	AIF1	2.92E-02	0.00E+00
3	CYTO	W5Q560	OGFOD3	2.34E-02	0.00E+00
3	CYTO	W5P536	TRIP10	1.86E-03	0.00E+00
3	CYTO	W5Q7X8	CRYBG3	9.04E-03	0.00E+00
3	CYTO	W5NUE3	PRDX1	6.16E-04	0.00E+00
3	CYTO	W5PQV2	LMBRD1	8.34E-04	0.00E+00
3	CYTO	W5PZ62	ZFYVE16	4.68E-03	0.00E+00
3	CYTO	W5Q0N7	IFIT2	1.27E-02	0.00E+00
3	CYTO	W5P369	AP2A2	4.05E-05	0.00E+00
3	CYTO	W5Q3B2	TYK2	1.52E-02	0.00E+00
3	CYTO	W5PL89	GSR	1.02E-02	0.00E+00
3	CYTO	W5PCD0	FUBP3	3.68E-02	0.00E+00
3	CYTO	W5PZD7		2.43E-03	0.00E+00
3	CYTO	W5PC32	PGM3	1.16E-02	0.00E+00

Supplementary Table 5: Differentially abundant proteins (DAPs) of GMØ relative to M0 and their cellular location.

FoldChange	Location	Protein IDs	Gene Names	P-value	FDR
3	CYTO	W5PFH4	MAPKAPK3	2.20E-02	0.00E+00
3	CYTO	W5Q7Z6	DIP2B	6.45E-03	0.00E+00
3	CYTO	W5NXQ8	KATNA1	6.26E-03	2.16E-04
3	CYTO	W5PFE6	ACOX1	3.90E-03	0.00E+00
3	CYTO	W5NZ70	LGALS3BP	2.17E-02	0.00E+00
3	CYTO	W5QBV7	CD44	9.79E-05	0.00E+00
3	CYTO	P29330	FDX1	1.14E-03	0.00E+00
3	CYTO	Q6XXL8	DYNLT3 TCTE1L	3.45E-03	0.00E+00
3	CYTO	W5NVV6	DNAJC3	4.00E-02	0.00E+00
3	CYTO	W5PDS4	C1QA	1.24E-02	0.00E+00
3	CYTO	W5Q0E7	LIN7C	1.43E-03	5.90E-03
3	CYTO	W5PVC1	SYNJ1	1.24E-03	0.00E+00
3	CYTO	W5PAX1	GCLC	3.58E-02	0.00E+00
3	CYTO	W5PL47	BMP1	3.45E-04	0.00E+00
3	CYTO	W5NYK1	PMVK	4.87E-03	0.00E+00
3	CYTO	W5PES0	STX4	1.22E-03	0.00E+00
3	CYTO	W5P743	GLMP	9.30E-03	0.00E+00
3	CYTO	W5NZQ6	CYP1A1	4.89E-02	0.00E+00
3	CYTO	W5PEP1	ATP1A2	4.28E-02	2.08E-04
3	CYTO	W5PCS4		3.16E-04	0.00E+00
3	CYTO	W5NZY1	MCMBP	4.62E-02	0.00E+00
3	CYTO	W5Q1W2	SDCBP	4.82E-02	0.00E+00
3	CYTO	W5QGF4	VPS18	2.57E-03	0.00E+00
3	CYTO	W5PAJ2	PSAP	1.45E-03	0.00E+00
3	CYTO	W5PPS6		2.34E-03	0.00E+00
3	CYTO	W5PF85	PEA15	4.00E-03	0.00E+00
3	CYTO	W5NYK9	CALU	2.03E-02	0.00E+00
3	CYTO	W5NQZ9	GSDMD	7.02E-03	0.00E+00
3	CYTO	W5NU94	PSTPIP1	3.21E-02	0.00E+00
3	CYTO	W5P2F6	FAF1	4.63E-02	0.00E+00
3	CYTO	W5QGT0	ATP13A3	2.29E-02	0.00E+00
3	CYTO	W5Q420		1.48E-02	0.00E+00
3	CYTO	W5PCM4	LUZP1	8.95E-04	0.00E+00
3	CYTO	W5PFU8	KAT6B	2.15E-02	5.84E-04
3	CYTO	W5PI67	IDS	8.00E-03	0.00E+00
3	CYTO	W5P1Q0	AP1B1	1.17E-03	0.00E+00
3	CYTO	W5PYS8		4.19E-02	0.00E+00
3	CYTO	W5Q3Y3	SFXN3	1.35E-02	0.00E+00
3	CYTO	W5NUJ2	PEAK1	7.45E-03	0.00E+00
3	CYTO	W5PCU1	ASS1	1.87E-03	0.00E+00
3	CYTO	W5P333		3.08E-02	0.00E+00
3	CYTO	W5P3C6	LOC101111906	1.12E-03	0.00E+00
3	CYTO	W5PGA9	NCSTN	1.08E-02	0.00E+00
2	CYTO	W5PFB1	TOR1B	2.32E-02	0.00E+00
2	CYTO	W5NQG2	TBC1D2B	1.47E-03	0.00E+00
2	CYTO	W5P8F3	AGPAT5	3.57E-03	0.00E+00
2	CYTO	W5P7L9	KLC1	1.75E-02	0.00E+00
2	CYTO	W5QDQ8	MMP14	1.02E-02	0.00E+00
2	CYTO	W5QFD0	RRAGC	4.13E-02	0.00E+00
2	CYTO	W5P6U2		1.50E-02	0.00E+00
2	CYTO	W5P481	COL1A1	7.14E-03	0.00E+00
2	CYTO	W5NSS6		5.16E-02	0.00E+00
2	CYTO	W5PHY4	TYMS	2.27E-02	0.00E+00
2	CYTO	W5PQ75	HSPH1	2.22E-02	0.00E+00
2	CYTO	W5P2M5	LOC101114535	3.51E-02	2.47E-03
2	CYTO	W5NUG3	GNPDA1	2.30E-02	0.00E+00
2	CYTO	W5PS52	MRC2	1.39E-03	0.00E+00
2	CYTO	W5PU61	SETD7	2.85E-02	0.00E+00
2	CYTO	W5Q045	TMED3	4.44E-04	0.00E+00
2	CYTO	W5Q2H2	SLC12A9	9.83E-04	0.00E+00
2	CYTO	W5NUQ8	GCC1	4.03E-02	0.00E+00
2	CYTO	W5P8L0	ALDH18A1	3.77E-02	0.00E+00
2	CYTO	W5QDN8	SLC48A1	1.60E-02	2.16E-04

Supplementary Table 5: Differentially abundant proteins (DAPs) of GMØ relative to M0 and their cellular location.

FoldChange	Location	Protein IDs	Gene Names	P-value	FDR
2	CYTO	W5QDR9	SLC7A7	1.40E-03	0.00E+00
2	CYTO	W5P434	NAGPA	6.86E-03	0.00E+00
2	CYTO	W5PZJ8	ASRGL1	1.40E-03	0.00E+00
2	CYTO	W5P726	PRCP	4.12E-03	0.00E+00
2	CYTO	P04074	ATP1A1	2.37E-03	0.00E+00
2	CYTO	W5NW41	TBC1D9B	2.63E-02	0.00E+00
2	CYTO	W5PUL4	MTMR6	3.97E-02	0.00E+00
2	CYTO	W5NV06	ATP6V0A1	5.29E-02	0.00E+00
2	CYTO	W5Q8J8	VPS41	3.82E-03	0.00E+00
2	CYTO	W5PFI1	TRAPPC6B	1.26E-02	0.00E+00
2	CYTO	W5Q6V7	SIPA1	4.92E-03	0.00E+00
2	CYTO	W5NVG2	EFEMP1	2.45E-02	0.00E+00
2	CYTO	W5P1U3	LACTB2	5.58E-02	0.00E+00
2	CYTO	W5QIJ6	SPPL2A	5.11E-02	0.00E+00
2	CYTO	W5P1U9	OXSM	2.35E-03	0.00E+00
2	CYTO	W5QCF3	SLC35F6	1.49E-02	0.00E+00
2	CYTO	W5Q758	DCTN5	1.92E-02	0.00E+00
2	CYTO	W5PAA0	RBMS1	1.17E-02	2.20E-04
2	CYTO	W5PFE7	ACOX1	3.36E-02	0.00E+00
2	CYTO	W5Q175	GNPTAB	5.32E-03	0.00E+00
2	CYTO	W5PMB1	SNX3	3.49E-04	0.00E+00
2	CYTO	W5P3H9	PICALM	1.16E-03	0.00E+00
2	CYTO	W5PER8	WDR91	5.20E-02	0.00E+00
2	CYTO	W5QBJ0	ANXA3	5.82E-03	0.00E+00
2	CYTO	W5PN65	PI4K2A	7.78E-03	0.00E+00
2	CYTO	W5Q989	PLOD1	1.47E-04	0.00E+00
2	CYTO	W5PWA8	HSPB1	9.04E-03	0.00E+00
2	CYTO	W5Q4N4	TOR3A	1.64E-02	0.00E+00
2	CYTO	W5PVT3	GALNS	1.80E-02	0.00E+00
2	CYTO	W5Q634	BLVRA	1.71E-02	0.00E+00
2	CYTO	W5QH69	RNF181	5.07E-02	0.00E+00
2	CYTO	W5Q1D8	GALNT7	3.70E-03	2.18E-04
2	CYTO	W5NRF7	MAP3K20	1.02E-02	0.00E+00
2	CYTO	W5P700	KIF1B	2.55E-02	0.00E+00
2	CYTO	W5Q850	AKAP10	2.22E-02	0.00E+00
2	CYTO	W5QGJ2	CHP1	1.38E-02	0.00E+00
2	CYTO	W5Q9Q5	PANK4	3.33E-02	2.18E-04
2	CYTO	W5Q971	DPP8	3.50E-04	0.00E+00
2	CYTO	W5PEX1	WASHC5	3.66E-02	0.00E+00
2	CYTO	W5NWV3	NECAP1	2.68E-02	2.02E-04
2	CYTO	W5PBJ4	ARHGAP10	3.87E-02	0.00E+00
2	CYTO	W5QG22	PLCB2	8.77E-04	0.00E+00
2	CYTO	W5PKV1	DNASE2	2.84E-02	0.00E+00
2	CYTO	W5Q411	HSPA13	2.42E-02	0.00E+00
2	CYTO	W5Q4Q8		5.74E-03	0.00E+00
2	CYTO	W5QII4	DMXL2	2.49E-03	0.00E+00
2	CYTO	W5P5E7		8.16E-03	0.00E+00
2	CYTO	W5PP02	MAP7D3	4.06E-03	6.95E-03
2	CYTO	W5PXC6		1.14E-02	0.00E+00
2	CYTO	W5Q9E6	TANGO2	2.59E-02	0.00E+00
2	CYTO	W5PQD5	DHX32	2.27E-02	2.46E-03
2	CYTO	W5P1U0	TXNL4A	3.86E-04	0.00E+00
2	CYTO	W5PQA2	PPARD	3.25E-02	4.63E-03
2	CYTO	W5NU07		3.85E-02	0.00E+00
2	CYTO	W5QAQ4	NRP2	4.24E-02	0.00E+00
2	CYTO	Q6XUZ5	IDH1	1.69E-04	0.00E+00
2	CYTO	W5QCV4	DHRS1	4.77E-02	0.00E+00
2	CYTO	W5PI02	TBC1D13	4.84E-02	0.00E+00
2	CYTO	W5Q6T6	SKIC3	4.49E-02	0.00E+00
2	CYTO	Q9XT28	ATOX1	2.71E-03	0.00E+00
2	CYTO	W5Q5K0		4.03E-02	0.00E+00
2	CYTO	W5QB79	GLCE	4.44E-02	2.21E-04
2	CYTO	W5Q2V0	YKT6	1.26E-03	0.00E+00

Supplementary Table 5: Differentially abundant proteins (DAPs) of GMØ relative to M0 and their cellular location.

FoldChange	Location	Protein IDs	Gene Names	P-value	FDR
2	CYTO	W5Q6T1	ARSB	1.09E-02	0.00E+00
2	CYTO	W5NUU7	CDH5	4.35E-02	0.00E+00
2	CYTO	W5PF68	EMB	7.81E-03	2.15E-04
2	CYTO	W5QAP3	TOM1	4.88E-03	0.00E+00
2	CYTO	W5Q8V2	LIMA1	2.82E-02	0.00E+00
2	CYTO	W5Q2V2	PAH	2.81E-02	0.00E+00
2	CYTO	W5PTI6	ST3GAL4	2.68E-02	0.00E+00
2	CYTO	Q009B1	ABCG2	4.72E-02	0.00E+00
2	CYTO	W5PRS4	FKBP5	4.77E-02	0.00E+00
2	CYTO	W5Q8K4	SLC3A2	1.27E-02	0.00E+00
2	CYTO	W5PC06	SIRPA	1.58E-03	0.00E+00
2	CYTO	W5QIK3	USP8	1.61E-02	0.00E+00
2	CYTO	W5NUU1	VRK2	2.73E-02	0.00E+00
2	CYTO	W5PY53	FTH1	5.43E-02	0.00E+00
2	CYTO	W5NWU5	SASH1	5.48E-02	0.00E+00
2	CYTO	W5PPY5	CD2AP	2.66E-03	0.00E+00
2	CYTO	W5QAS2	STX6	1.89E-02	0.00E+00
2	CYTO	W5P6G7	TG	2.48E-02	0.00E+00
2	CYTO	W5PH81	C7	3.91E-02	0.00E+00
2	CYTO	W5NRR6	MGAT2	1.49E-02	0.00E+00
2	CYTO	W5PMY4	GABARAP	4.37E-02	0.00E+00
2	CYTO	W5QCD3	RNASEL	5.14E-02	0.00E+00
2	CYTO	W5QB00	MCM5	5.24E-02	0.00E+00
2	CYTO	W5PVE3	LOC101115252	1.86E-04	0.00E+00
2	CYTO	W5PA83	PRR14	1.02E-03	4.45E-03
2	CYTO	W5Q3D3	K33	2.66E-02	2.65E-03
2	CYTO	W5QA87	AAGAB	6.07E-03	3.99E-04
2	CYTO	W5NYZ6	CD81	3.47E-03	0.00E+00
2	CYTO	W5Q8I6	POLDIP2	3.31E-02	0.00E+00
2	CYTO	W5Q740	ABCD3	4.59E-02	0.00E+00
2	CYTO	W5Q2K9	CYFIP1	9.15E-03	0.00E+00
2	CYTO	W5NYG8	TSSC4	3.05E-03	0.00E+00
2	CYTO	W5PDU4	NMT2	9.86E-03	0.00E+00
2	CYTO	W5QG24	PPT1	1.26E-03	0.00E+00
2	CYTO	W5Q0P4	MTMR9	3.19E-03	0.00E+00
2	CYTO	B3GS77	IGFBP5	3.13E-02	0.00E+00
2	CYTO	W5PLR4	MCM6	5.53E-02	0.00E+00
2	CYTO	W5PAM5		5.11E-03	0.00E+00
2	CYTO	W5PUJ2	LAMP2	1.05E-02	0.00E+00
2	CYTO	W5PK12	OAT	3.53E-04	0.00E+00
2	CYTO	W5PQZ5	GDE1	9.25E-03	2.07E-04
2	CYTO	W5P1S3	SLC25A13	1.48E-02	0.00E+00
2	CYTO	W5PQT3	LOC101105107	5.41E-02	0.00E+00
2	CYTO	W5P4T0	VHL	4.38E-03	2.04E-04
2	CYTO	W5P6M5	SEC11C	3.95E-03	1.91E-03
2	CYTO	W5PDX8	FAM120A	1.16E-02	0.00E+00
2	CYTO	W5Q0M7	RAP2B	8.65E-04	0.00E+00
2	CYTO	W5P8S8	ALDH1L1	3.06E-02	0.00E+00
2	CYTO	W5Q7Z7	DSP	1.14E-02	0.00E+00
2	CYTO	W5PMC5	LOC101110546	3.11E-02	0.00E+00
2	CYTO	W5Q1P6	STX2	6.81E-03	0.00E+00
2	CYTO	W5QC34	MAN2A1	1.11E-02	0.00E+00
2	CYTO	W5P164	LAMTOR1	3.49E-02	0.00E+00
2	CYTO	W5Q131		2.46E-02	2.09E-04
2	CYTO	W5PA59	LOC101117015 LO	3.53E-03	0.00E+00
2	CYTO	W5P001	RUFY1	4.16E-03	0.00E+00
2	CYTO	W5QEA3		1.24E-03	0.00E+00
2	CYTO	W5PUU2	RRAS	2.50E-02	0.00E+00
2	CYTO	W5Q928	PCYT2	2.85E-02	0.00E+00
2	CYTO	W5PUW5	GOLGA5	4.44E-02	0.00E+00
2	CYTO	W5PJW1	SDHC	3.12E-02	0.00E+00
2	CYTO	W5PLB6	SPART	1.24E-02	0.00E+00
2	CYTO	W5PEE6	ITGA2	4.47E-02	6.25E-03

Supplementary Table 5: Differentially abundant proteins (DAPs) of GMØ relative to M0 and their cellular location.

FoldChange	Location	Protein IDs	Gene Names	P-value	FDR
2	CYTO	W5P906	DPP4	1.74E-02	5.89E-03
2	CYTO	W5QH50	HRG	1.09E-03	0.00E+00
2	CYTO	W5PUR2	PRKCQ	3.14E-02	1.16E-03
2	CYTO	W5Q9H0	MMP2	1.45E-02	2.21E-04
2	CYTO	W5PNW7	VIM	6.77E-04	0.00E+00
2	CYTO	W5Q824	GSTA1	5.22E-02	0.00E+00
2	CYTO	W5PUI3	GOLGA1	4.16E-02	0.00E+00
2	CYTO	W5PCK8	LOC100135455	1.79E-02	0.00E+00
2	CYTO	W5NYV5	SPAG9	1.42E-02	0.00E+00
2	CYTO	W5Q3J3	COLGALT1	1.70E-02	0.00E+00
2	CYTO	W5QAE8	ALDH7A1	8.35E-06	0.00E+00
2	CYTO	W5PH58		5.24E-02	0.00E+00
2	CYTO	W5P8B4	TRAPPC8	1.29E-02	0.00E+00
2	CYTO	W5PRJ7	CCDC93	1.34E-02	0.00E+00
2	CYTO	W5P983	COL4A2	1.73E-02	2.09E-04
2	CYTO	W5P5A7	VPS16	2.32E-03	0.00E+00
2	CYTO	W5PB20		1.93E-02	0.00E+00
2	CYTO	W5NQG1	SETD3	6.80E-03	0.00E+00
2	CYTO	W5QHA0	AGFG1	2.90E-04	0.00E+00
2	CYTO	W5P5H5	CLPB	3.56E-02	0.00E+00
2	CYTO	W5Q9H8	SNX5	2.81E-02	0.00E+00
2	CYTO	W5Q5B5	CSAD	1.96E-03	0.00E+00
2	CYTO	W5P089	LOC101115969	1.68E-02	0.00E+00
2	CYTO	W5PPG2	DYSF	4.01E-02	0.00E+00
2	CYTO	W5PFW1	GAN	5.62E-03	0.00E+00
2	CYTO	W5PRQ0	RBM3	1.76E-02	0.00E+00
2	CYTO	W5QAL6	FMNL3	5.57E-03	2.18E-04
2	CYTO	W5PCN2	ANXA7	4.18E-03	0.00E+00
2	CYTO	W5QCL1	NCF4	1.51E-03	0.00E+00
2	CYTO	W5PJV4	ASGR2	4.73E-02	0.00E+00
2	CYTO	W5NYE8	SEL1L	5.12E-02	0.00E+00
2	CYTO	W5PXK7	RASA4B	3.63E-02	0.00E+00
2	CYTO	W5PJY6	ADAM28	3.04E-02	0.00E+00
2	CYTO	W5NY29		5.24E-03	0.00E+00
2	CYTO	W5PSD7	RAP2C	5.46E-02	0.00E+00
2	CYTO	W5PUM8	HIP1	3.59E-02	0.00E+00
2	CYTO	W5PSX7	FES	3.11E-02	0.00E+00
2	CYTO	W5PRK6	STAU1	5.50E-02	0.00E+00
2	CYTO	W5Q0F3	TGFBI	4.52E-02	0.00E+00
2	CYTO	W5PS60		4.65E-02	2.18E-04
2	CYTO	W5NUE1	NCF2	2.24E-02	0.00E+00
2	CYTO	W5P7P6	MYO18A	2.86E-03	0.00E+00
2	CYTO	W5Q7R9	LAMTOR4	1.63E-02	0.00E+00
2	CYTO	W5PSG0		3.96E-02	0.00E+00
2	CYTO	W5PMR2	MMP19	2.94E-02	0.00E+00
2	CYTO	W5PKP3	FAM114A1	1.77E-02	0.00E+00
2	CYTO	W5NUZ1	DYNC1I2	1.12E-02	0.00E+00
2	CYTO	W5PEN2	TPD52	2.39E-02	0.00E+00
2	CYTO	W5P6U5	LMAN1	7.29E-03	0.00E+00
2	CYTO	W5NYA8	SCAMP2	4.91E-02	0.00E+00
2	CYTO	W5PGV0	ITGAM	8.70E-03	0.00E+00
2	CYTO	Q6ECI6	ITGB2 CD18	1.03E-04	0.00E+00
2	CYTO	W5P2N8	PIK3C2A	3.18E-03	2.19E-04
2	CYTO	W5NY14		3.80E-04	0.00E+00
-2	CYTO	W5NW82	NAP1L4	8.58E-04	0.00E+00
-2	CYTO	W5Q1I8	PPM1F	5.32E-03	0.00E+00
-2	CYTO	W5PXS7	DAPP1	1.27E-02	0.00E+00
-2	CYTO	W5PQI0	PXN	1.66E-02	0.00E+00
-2	CYTO	W5Q732	PDK3	1.12E-02	3.96E-04
-2	CYTO	W5PCQ8	SLC25A4	1.90E-02	0.00E+00
-2	CYTO	W5PXW3	OSBPL2	1.35E-03	3.56E-03
-2	CYTO	W5PGV7	UBQLN1	1.37E-02	0.00E+00
-2	CYTO	W5QFQ8	LOC101111915	1.42E-02	0.00E+00

Supplementary Table 5: Differentially abundant proteins (DAPs) of GMØ relative to M0 and their cellular location.

FoldChange	Location	Protein IDs	Gene Names	P-value	FDR
-2	CYTO	W5Q992	MYO9B	1.72E-02	0.00E+00
-2	CYTO	W5PC19	STX18	2.31E-02	2.11E-04
-2	CYTO	W5PJA0	CUTA	1.37E-02	0.00E+00
-2	CYTO	W5QJ24	PPP2R5E	1.08E-02	0.00E+00
-2	CYTO	W5Q9P1	CTNNBL1	4.56E-02	0.00E+00
-2	CYTO	W5PXT1	MYO1G	3.10E-02	0.00E+00
-2	CYTO	W5QDM6	STRIP1	2.08E-02	0.00E+00
-2	CYTO	W5P7E2	PPA1	1.40E-02	0.00E+00
-2	CYTO	W5PTU8	MARK1	2.89E-02	8.89E-03
-2	CYTO	W5P090		1.01E-02	0.00E+00
-2	CYTO	W5PID5	TSG101	6.07E-03	0.00E+00
-2	CYTO	W5PCE9	MICOS13	1.42E-02	0.00E+00
-2	CYTO	W5Q9Z8	PHAX	4.09E-02	0.00E+00
-2	CYTO	W5PUV1	DCAF7	2.09E-03	0.00E+00
-2	CYTO	W5NX40	TAB2	1.39E-02	0.00E+00
-2	CYTO	W5PDK7	PCCB	2.86E-02	0.00E+00
-2	CYTO	W5NTH2	EIF2B3	4.65E-02	0.00E+00
-2	CYTO	W5QAP8	RPRD1B	1.23E-02	0.00E+00
-2	CYTO	W5PXJ6	ANKRD13A	5.74E-03	0.00E+00
-2	CYTO	W5P033	COG8	3.18E-03	4.45E-03
-2	CYTO	W5Q1V2	ZNRD2	7.65E-03	0.00E+00
-2	CYTO	W5P031	HDHD2	2.03E-02	0.00E+00
-2	CYTO	W5PQ88	RPS6KB2	1.44E-02	0.00E+00
-2	CYTO	W5NUT8	PIP4K2A	3.24E-02	0.00E+00
-2	CYTO	W5PLK6	SHOC2	1.25E-02	2.00E-04
-2	CYTO	W5PHE6	DDI2	1.07E-03	0.00E+00
-2	CYTO	W5PKR1		4.68E-02	0.00E+00
-2	CYTO	W5P2Q7	HIKESHI	1.80E-02	0.00E+00
-2	CYTO	W5Q2R9		3.96E-02	0.00E+00
-2	CYTO	W5PEE7	PDP1	4.19E-03	0.00E+00
-2	CYTO	W5NPP7	DRAP1	4.68E-02	3.93E-04
-2	CYTO	W5PK27		5.11E-02	0.00E+00
-2	CYTO	W5QFV9	PPIE	4.73E-02	2.11E-04
-2	CYTO	W5QIC3	PRUNE1	6.05E-03	0.00E+00
-2	CYTO	W5NPT4	LOC101116298	1.17E-02	0.00E+00
-2	CYTO	W5PVX8	PPP3CA	1.83E-02	0.00E+00
-2	CYTO	W5QF78		5.40E-04	0.00E+00
-2	CYTO	W5PMM8	GGA3	9.96E-04	0.00E+00
-2	CYTO	W5NY88	PSMD10	5.39E-03	0.00E+00
-2	CYTO	W5PTB3	INPP5K	3.47E-04	2.02E-04
-2	CYTO	W5NSV7	BET1	8.41E-03	0.00E+00
-2	CYTO	W5Q1I6	IMPDH2 IMPDH	4.34E-02	0.00E+00
-2	CYTO	W5QB23		2.39E-02	0.00E+00
-2	CYTO	C5IWU4	ARL3	2.56E-02	0.00E+00
-2	CYTO	W5QJ62	ACTN1	5.10E-02	0.00E+00
-2	CYTO	C8BKC5	PRDX2	3.89E-02	0.00E+00
-2	CYTO	W5PJP6	SMARCC2	5.60E-02	0.00E+00
-2	CYTO	W5Q3F6	ELP4	5.58E-02	2.20E-04
-2	CYTO	W5P762		2.93E-02	0.00E+00
-2	CYTO	W5PT31		2.27E-02	0.00E+00
-2	CYTO	W5NWI4		5.40E-03	0.00E+00
-2	CYTO	W5NUV2	ARF5	1.68E-02	0.00E+00
-2	CYTO	W5P4W0	TSFM	1.32E-02	0.00E+00
-2	CYTO	W5PFV0	HMGCS1	4.67E-02	0.00E+00
-2	CYTO	W5PMI8	CUL5	3.71E-03	0.00E+00
-2	CYTO	C5ISA4	COPS7B	1.89E-02	0.00E+00
-2	CYTO	W5P4C5	RBM42	2.00E-02	0.00E+00
-2	CYTO	W5QHL0	ATP11B	3.81E-02	0.00E+00
-2	CYTO	W5NV31	COPS3	5.30E-02	0.00E+00
-2	CYTO	W5P1E2	CNST	1.08E-02	0.00E+00
-2	CYTO	W5PQW2	MOB1B	9.55E-03	0.00E+00
-2	CYTO	W5PWH9	PRKACB	2.06E-03	0.00E+00
-2	CYTO	W5PEV7	DYNLL2	3.65E-02	0.00E+00

Supplementary Table 5: Differentially abundant proteins (DAPs) of GMØ relative to M0 and their cellular location.

FoldChange	Location	Protein IDs	Gene Names	P-value	FDR
-2	CYTO	W5PZB6	ETFB	9.49E-03	0.00E+00
-2	CYTO	W5PPE2	MGST3	5.58E-02	0.00E+00
-2	CYTO	W5PQK6	TLN1	3.25E-02	0.00E+00
-2	CYTO	W5PYM3	CAAP1	1.22E-02	1.15E-03
-2	CYTO	W5PUR1	CNOT11	9.07E-03	0.00E+00
-2	CYTO	W5QCZ0	MPST	2.01E-03	0.00E+00
-2	CYTO	W5Q7Q9	PFDN1	3.05E-02	3.93E-04
-2	CYTO	W5PUU8	LOC101122645	2.13E-02	0.00E+00
-2	CYTO	W5PTZ8	LOC101114959	3.98E-03	0.00E+00
-2	CYTO	W5QCI3	LOC101106419	3.47E-03	0.00E+00
-2	CYTO	W5P0R7	DHODH	1.53E-02	2.17E-04
-2	CYTO	W5QHP6	ARHGDIB	4.03E-04	0.00E+00
-2	CYTO	W5PJQ5	HEATR5B	1.58E-02	0.00E+00
-2	CYTO	W5PCV5	RALGAPA1	4.89E-02	8.37E-03
-2	CYTO	W5QGW0	PPIH	4.65E-02	0.00E+00
-2	CYTO	W5PY73	SF3A2	3.03E-02	0.00E+00
-2	CYTO	W5P7C2	SEPHS1	4.05E-02	0.00E+00
-2	CYTO	W5PHD7	WBP2	3.56E-03	2.47E-03
-2	CYTO	W5Q4J3	RAPGEF6	8.13E-03	9.23E-03
-2	CYTO	W5NS43	ALDH6A1	4.74E-03	0.00E+00
-2	CYTO	W5NZA5	DGKZ	3.66E-02	0.00E+00
-2	CYTO	W5QB24	ANP32A	2.33E-02	0.00E+00
-2	CYTO	W5Q5I7	NFYB	5.54E-02	0.00E+00
-2	CYTO	W5PL66	PPA2	7.27E-03	0.00E+00
-2	CYTO	W5QDF0	NRBP1	1.09E-02	0.00E+00
-2	CYTO	W5PME9	ARHGAP45	3.13E-02	0.00E+00
-2	CYTO	W5QCC1	STAT5B	6.97E-03	0.00E+00
-2	CYTO	W5Q5M7	SLC44A2	1.70E-02	0.00E+00
-2	CYTO	W5PXI2		5.21E-02	0.00E+00
-2	CYTO	W5Q4D5	BCO2	2.73E-02	0.00E+00
-2	CYTO	W5PMY9		3.85E-02	0.00E+00
-2	CYTO	W5Q8P3	USE1	5.00E-03	0.00E+00
-2	CYTO	W5PMC9	ARHGAP6	1.22E-02	7.13E-03
-2	CYTO	W5PYE5	NUDCD1	4.06E-02	0.00E+00
-2	CYTO	Q2TCH3	ACLY	2.35E-04	0.00E+00
-2	CYTO	W5PTS4	LOC101114275	2.83E-02	0.00E+00
-2	CYTO	W5PUB1	THUMPD1	4.54E-02	0.00E+00
-2	CYTO	W5NWE0	MECR	2.86E-02	0.00E+00
-2	CYTO	W5PN94	LUC7L	1.72E-02	0.00E+00
-2	CYTO	W5QIF1	MYO5A	1.23E-02	0.00E+00
-2	CYTO	W5PS76	UNC45A	6.30E-03	0.00E+00
-2	CYTO	W5Q2I3	YME1L1	8.60E-03	0.00E+00
-2	CYTO	W5NX76	MAP2K3	1.40E-02	0.00E+00
-2	CYTO	W5NZ21	LOC101105090	1.12E-02	0.00E+00
-2	CYTO	W5PK25	BAK1	4.75E-02	0.00E+00
-2	CYTO	W5Q7H5	HVCN1	3.00E-02	0.00E+00
-2	CYTO	W5Q1Q3	ARL14EP	5.87E-03	0.00E+00
-2	CYTO	W5PC20	VPS37B	1.20E-02	0.00E+00
-2	CYTO	W5Q8F8	CPOX	2.01E-02	0.00E+00
-2	CYTO	W5NRI1		2.02E-02	2.14E-04
-2	CYTO	W5PKK8	ESYT1	1.82E-03	0.00E+00
-2	CYTO	W5P0J5	ZNF22	1.59E-02	2.19E-04
-2	CYTO	W5PLB1	CLIC5	3.62E-02	0.00E+00
-2	CYTO	W5P0R4	CTIF	7.37E-04	3.94E-04
-2	CYTO	W5PUS0	CUL4B	4.81E-02	0.00E+00
-2	CYTO	W5PHB6	SF1	2.88E-02	0.00E+00
-2	CYTO	W5QIH2	TMOD3	5.13E-03	0.00E+00
-2	CYTO	W5Q5Q8	WDR44	3.42E-02	0.00E+00
-2	CYTO	W5Q198	QRICH1	1.10E-02	2.12E-04
-2	CYTO	W5PY18	LOC101118100	2.39E-02	0.00E+00
-2	CYTO	W5PLD5	CNN2	1.67E-03	0.00E+00
-2	CYTO	W5Q4G2		5.19E-03	0.00E+00
-2	CYTO	W5PRA0	BPNT1	1.83E-02	0.00E+00

Supplementary Table 5: Differentially abundant proteins (DAPs) of GMØ relative to M0 and their cellular location.

FoldChange	Location	Protein IDs	Gene Names	P-value	FDR
-2	CYTO	W5PZC3	STAMBPL1	4.08E-02	0.00E+00
-2	CYTO	W5Q9C5	LNPEP	1.12E-02	0.00E+00
-2	CYTO	W5Q6F2	ARMC1	1.38E-02	0.00E+00
-2	CYTO	W5NRG0	PTGR2	4.15E-02	0.00E+00
-2	CYTO	W5Q9B1	ATP2A3	1.24E-02	0.00E+00
-2	CYTO	W5PTJ0	CRLF3	6.95E-03	0.00E+00
-2	CYTO	W5Q0B6	PPP1R12A	2.24E-03	0.00E+00
-2	CYTO	W5PUU6	RPL35	3.97E-02	0.00E+00
-2	CYTO	W5P9F0	ARHGAP9	1.70E-02	0.00E+00
-2	CYTO	W5NSI2	RALBP1	2.25E-02	8.55E-03
-2	CYTO	W5NTA4	CGGBP1	1.46E-02	0.00E+00
-2	CYTO	W5Q6U0	FASN	3.93E-02	0.00E+00
-2	CYTO	W5PQ04	DEF6	3.14E-03	0.00E+00
-2	CYTO	W5PK62	CDC40	3.30E-02	0.00E+00
-2	CYTO	W5PB28	CLNS1A	2.60E-02	0.00E+00
-2	CYTO	W5QHX2	RPL22L1	4.90E-02	0.00E+00
-2	CYTO	W5PSX3	DARS2	1.21E-02	2.07E-04
-2	CYTO	W5PUU9	HDHD5	3.42E-02	0.00E+00
-2	CYTO	W5P9A0	ALDH5A1	7.59E-03	0.00E+00
-2	CYTO	W5Q595	NIF3L1	2.68E-02	0.00E+00
-2	CYTO	W5PZ59	LOC101104574	3.87E-02	0.00E+00
-2	CYTO	W5Q3M9	SEPTIN6	9.26E-03	0.00E+00
-2	CYTO	W5P948	INIP	4.45E-03	0.00E+00
-2	CYTO	W5Q4I7	LOC101123619	1.50E-02	1.16E-03
-2	CYTO	W5NU48	CRTC2	6.31E-03	1.91E-03
-2	CYTO	W5QEK8	EHD3	3.41E-02	0.00E+00
-2	CYTO	W5NWQ0	TMED8	1.99E-02	0.00E+00
-2	CYTO	W5PEA0	FGD3	5.34E-02	0.00E+00
-2	CYTO	W5PTQ0	RNGTT	1.43E-02	2.05E-04
-2	CYTO	W5QHP4	ACTL6A	1.49E-02	0.00E+00
-2	CYTO	W5PQQ5	DIDO1	4.49E-02	0.00E+00
-2	CYTO	W5PRR5	LTA4H	2.71E-04	0.00E+00
-2	CYTO	W5P4U4	GRK6	4.23E-04	0.00E+00
-2	CYTO	W5PKD2	GCDH	2.50E-02	0.00E+00
-2	CYTO	W5PR48	HPRT1	1.16E-02	0.00E+00
-2	CYTO	W5PTN4	TMPO	2.68E-03	0.00E+00
-2	CYTO	W5PTM6	CPSF2	1.09E-02	0.00E+00
-2	CYTO	W5PGD6	BZW2	8.48E-04	0.00E+00
-3	CYTO	W5Q2C6		4.90E-02	2.22E-04
-3	CYTO	W5PHL8	NUDT16	2.15E-03	0.00E+00
-3	CYTO	W5Q9J0	RNPS1	1.76E-02	0.00E+00
-3	CYTO	W5PSQ7		1.25E-02	0.00E+00
-3	CYTO	C8BKD4	CBX5	1.70E-02	0.00E+00
-3	CYTO	W5PRJ3	HYCC1	3.81E-02	0.00E+00
-3	CYTO	W5Q3J5	IWS1	4.01E-02	0.00E+00
-3	CYTO	W5PV48	USP11	1.95E-03	0.00E+00
-3	CYTO	W5P668		4.87E-02	2.18E-04
-3	CYTO	Q5MIB6	PYGB	4.78E-02	0.00E+00
-3	CYTO	W5P7A6	ATE1	3.36E-03	0.00E+00
-3	CYTO	W5Q9N1	LPCAT2	1.15E-03	3.96E-04
-3	CYTO	W5PZJ7		4.06E-03	0.00E+00
-3	CYTO	W5PJW9	SAP30BP	2.28E-02	0.00E+00
-3	CYTO	W5NXF6	TUBG1	4.15E-02	0.00E+00
-3	CYTO	W5Q9R2	SARM1	4.30E-02	0.00E+00
-3	CYTO	W5Q3Q2	ERCC3	5.66E-03	4.11E-03
-3	CYTO	W5P524	CC2D1B	2.16E-02	0.00E+00
-3	CYTO	W5PA22	ACOT8	2.79E-04	0.00E+00
-3	CYTO	W5PK38	VASP	3.18E-02	0.00E+00
-3	CYTO	W5PFJ5	SMC1A	1.60E-02	0.00E+00
-3	CYTO	W5P5F0	TMEM106A	4.55E-02	0.00E+00
-3	CYTO	W5Q5Y1	ITGA4	4.97E-06	2.23E-04
-3	CYTO	W5PL05	MICAL1	1.78E-04	0.00E+00
-3	CYTO	O78750	MT-CO2 COII COX2	1.95E-02	0.00E+00

Supplementary Table 5: Differentially abundant proteins (DAPs) of GMØ relative to M0 and their cellular location.

FoldChange	Location	Protein IDs	Gene Names	P-value	FDR
-3	CYTO	W5PDD5	STAG1	3.81E-02	0.00E+00
-3	CYTO	W5PGB0	SIRT3	2.43E-02	0.00E+00
-3	CYTO	W5PSA9	VPS13A	1.51E-02	0.00E+00
-3	CYTO	W5QHX6	ANP32E	4.56E-02	0.00E+00
-3	CYTO	W5PAF9	KIF2A	1.72E-02	0.00E+00
-3	CYTO	W5QH77	USP39	5.28E-03	0.00E+00
-3	CYTO	W5NQ72	PFAS	1.30E-03	0.00E+00
-3	CYTO	P80190		1.37E-02	0.00E+00
-3	CYTO	W5PPX0	LYPLAL1	3.89E-03	0.00E+00
-3	CYTO	W5NRP8	MTCL1	3.57E-02	7.78E-04
-3	CYTO	W5P2J9		3.10E-03	0.00E+00
-3	CYTO	W5P432	NIPSNAP1	5.12E-03	0.00E+00
-3	CYTO	W5PQT2	TPRKB	4.21E-02	0.00E+00
-3	CYTO	W5PMJ7	NADK2	1.38E-02	0.00E+00
-3	CYTO	W5NSP0		2.48E-03	0.00E+00
-3	CYTO	W5PZP8	FIS1	5.47E-03	0.00E+00
-3	CYTO	W5PXR8	POLR2J	5.96E-03	0.00E+00
-3	CYTO	W5Q804	SPECC1	1.10E-03	0.00E+00
-3	CYTO	W5Q0P0	GABPA	5.46E-02	0.00E+00
-3	CYTO	W5PIK1	LZTFL1	5.43E-02	0.00E+00
-3	CYTO	W5NZW5	WDFY3	5.27E-02	0.00E+00
-3	CYTO	W5Q7L1	CNN3	9.38E-03	0.00E+00
-3	CYTO	W5QIY4	ARID4A	7.64E-03	0.00E+00
-3	CYTO	W5PX66	TIAM1	5.49E-03	0.00E+00
-3	CYTO	W5Q366	SUN2	3.48E-02	0.00E+00
-3	CYTO	W5P7P9	ERBIN	4.42E-03	0.00E+00
-3	CYTO	W5PKA9	F5	1.27E-02	0.00E+00
-3	CYTO	W5Q600	TSC22D4	3.09E-02	0.00E+00
-3	CYTO	W5QGW8	PDE3A	5.12E-02	0.00E+00
-3	CYTO	W5P5N5		1.39E-03	2.14E-04
-3	CYTO	W5PLX5	PRKD2	1.90E-04	0.00E+00
-3	CYTO	W5P0N1	DHX38	1.49E-02	9.69E-04
-3	CYTO	W5PHA6	TP53RK	5.03E-02	2.14E-04
-3	CYTO	W5P955	NT5C3A	5.59E-02	0.00E+00
-3	CYTO	W5P225	PDCD5	4.62E-03	0.00E+00
-3	CYTO	W5NYK3	CTCF	5.07E-02	0.00E+00
-3	CYTO	W5QGB5	LOC101113369	8.49E-04	0.00E+00
-3	CYTO	W5NWU0	LOC101122262	3.88E-02	0.00E+00
-3	CYTO	W5Q4L7	AP3S1	2.33E-03	0.00E+00
-3	CYTO	W5QAK5	PIAS1	1.33E-02	2.17E-04
-3	CYTO	W5PJ14	ESYT2	2.65E-03	0.00E+00
-3	CYTO	W5Q2V1		2.49E-02	0.00E+00
-3	CYTO	W5QHL5	CAB39	2.33E-03	0.00E+00
-3	CYTO	W5PIW6	PRTN3	4.55E-02	0.00E+00
-3	CYTO	W5QFR4	LOC101112856	2.84E-02	0.00E+00
-3	CYTO	W5PW06	PNKP	1.71E-02	0.00E+00
-3	CYTO	W5PUI4	RAB3A	3.53E-04	2.15E-04
-3	CYTO	W5PHI5	PALM	1.99E-02	0.00E+00
-3	CYTO	W5QFP2	LOC101109397	1.97E-02	0.00E+00
-3	CYTO	W5Q8H0	SSH1	3.40E-02	0.00E+00
-3	CYTO	W5QCX7	MYBBP1A	4.71E-02	0.00E+00
-3	CYTO	W5QD23	LANCL1	1.20E-02	0.00E+00
-3	CYTO	W5QH43	EIF4A2	2.09E-02	0.00E+00
-3	CYTO	W5Q8M9	PYCR2	1.52E-03	0.00E+00
-3	CYTO	W5QIK2	RFX5	6.98E-03	0.00E+00
-3	CYTO	W5PVX3	PRKG1	2.01E-02	0.00E+00
-3	CYTO	W5NTN7	PNPLA6	2.06E-02	0.00E+00
-3	CYTO	W5PCZ2	PPP3CB	1.57E-04	0.00E+00
-3	CYTO	W5PP85	TBC1D10C	1.55E-02	0.00E+00
-3	CYTO	C0IZ95	RAB27A	1.29E-02	0.00E+00
-3	CYTO	W5P433	BRCA1	1.07E-03	3.74E-03
-3	CYTO	W5Q3J8		4.50E-02	0.00E+00
-3	CYTO	W5QJ56	RDH11	3.41E-02	0.00E+00

Supplementary Table 5: Differentially abundant proteins (DAPs) of GMØ relative to M0 and their cellular location.

FoldChange	Location	Protein IDs	Gene Names	P-value	FDR
-3	CYTO	W5P7P8	GCA	1.72E-02	0.00E+00
-3	CYTO	C5IS96	LCAT	2.39E-02	2.06E-04
-3	CYTO	W5Q3I7	TUBB1	4.57E-02	0.00E+00
-3	CYTO	W5QHY6	RPIA	3.79E-03	0.00E+00
-3	CYTO	W5NUS2	MED9	1.75E-02	2.21E-04
-3	CYTO	W5P691	CNTRL	3.32E-02	0.00E+00
-3	CYTO	W5NTW9	RIPK1	2.17E-02	0.00E+00
-3	CYTO	W5PAV5	RFX1	1.19E-02	0.00E+00
-3	CYTO	W5NUE6	PLEKHA2	1.10E-03	0.00E+00
-3	CYTO	W5PAV0	STAP1	1.42E-02	0.00E+00
-3	CYTO	W5NUX2		8.86E-03	0.00E+00
-3	CYTO	W5PGX7	SSRP1	1.94E-02	0.00E+00
-3	CYTO	W5Q878	PRKCB	5.44E-03	0.00E+00
-3	CYTO	W5P795	LRBA	2.45E-02	0.00E+00
-3	CYTO	W5NTQ3	H3-4	1.40E-04	2.02E-04
-3	CYTO	W5PG02	RASA3	3.83E-02	0.00E+00
-3	CYTO	W5QBV1	FER	8.61E-03	0.00E+00
-3	CYTO	W5PZ86	SEPTIN8	3.47E-02	0.00E+00
-3	CYTO	W5Q1C4	DNMT1	5.24E-03	9.69E-04
-3	CYTO	W5QFM2		3.91E-05	0.00E+00
-3	CYTO	W5QFT3		1.67E-02	0.00E+00
-3	CYTO	W5Q6S0	LBR	4.60E-03	0.00E+00
-3	CYTO	W5Q059	SASH3	4.91E-02	0.00E+00
-4	CYTO	W5NTV6		3.19E-05	0.00E+00
-4	CYTO	W5PV67	LIMD2	1.83E-02	0.00E+00
-4	CYTO	W5P8I8	PITPNM2	1.67E-03	0.00E+00
-4	CYTO	W5NRR9	STAT5A	2.84E-03	0.00E+00
-4	CYTO	W5P9M9	LOC101103862	3.10E-02	0.00E+00
-4	CYTO	W5Q6R6	SHPK	9.40E-03	0.00E+00
-4	CYTO	W5PZU8	ASAP2	2.20E-02	0.00E+00
-4	CYTO	W5Q088	CBL	1.21E-03	0.00E+00
-4	CYTO	W5PY97	SVIL	1.10E-03	0.00E+00
-4	CYTO	W5PIN7		2.65E-02	0.00E+00
-4	CYTO	W5PYK8	VNN2	2.12E-03	0.00E+00
-4	CYTO	W5P3Y9	CD22	3.56E-03	0.00E+00
-4	CYTO	W5PI65	MAP4K2	3.74E-05	0.00E+00
-4	CYTO	W5P4F9	PRKAR2B	1.66E-02	0.00E+00
-4	CYTO	W5Q2W4	RAVER1	2.47E-02	0.00E+00
-4	CYTO	W5PRM0	GMPR	5.44E-02	0.00E+00
-4	CYTO	W5PT20	ZC3H18	2.92E-02	0.00E+00
-4	CYTO	W5NXV1	PACS1	7.35E-03	0.00E+00
-4	CYTO	W5PZL2		3.57E-02	0.00E+00
-4	CYTO	W5QHL6	ITM2C	1.00E-03	0.00E+00
-4	CYTO	W5P3V8		1.73E-02	0.00E+00
-4	CYTO	W5P7B8	MTMR3	5.45E-03	0.00E+00
-4	CYTO	W5Q859	STX5	1.69E-04	0.00E+00
-4	CYTO	W5P8H4	DCTPP1	9.40E-05	0.00E+00
-4	CYTO	W5Q8J0	PIN4	2.24E-02	0.00E+00
-4	CYTO	W5PLN1		1.08E-02	0.00E+00
-4	CYTO	W5P7K2		4.97E-05	0.00E+00
-4	CYTO	W5Q9H1	ZYX	2.29E-02	0.00E+00
-4	CYTO	W5PU56	PIH1D1	9.50E-03	0.00E+00
-4	CYTO	W5PKY8	RAD21	1.36E-02	0.00E+00
-4	CYTO	W5Q2G2	IL16	1.40E-02	0.00E+00
-4	CYTO	W5NYC7	DQA	3.01E-02	0.00E+00
-4	CYTO	W5P5K9	SRGN	5.82E-03	2.02E-04
-4	CYTO	W5QI61	CDKN1B	7.88E-04	0.00E+00
-4	CYTO	W5PKS6	DEK	1.16E-02	0.00E+00
-4	CYTO	W5Q5E7	PYCR3	4.26E-03	0.00E+00
-4	CYTO	W5NXW9		3.06E-03	0.00E+00
-4	CYTO	W5PRC8	IKZF3	4.28E-02	0.00E+00
-4	CYTO	W5PAF7	RSL1D1	4.25E-03	0.00E+00
-4	CYTO	W5PBE0	CD74	3.65E-02	0.00E+00

Supplementary Table 5: Differentially abundant proteins (DAPs) of GMØ relative to M0 and their cellular location.

FoldChange	Location	Protein IDs	Gene Names	P-value	FDR
-4	CYTO	W5P0E3	BRD4	4.07E-03	0.00E+00
-4	CYTO	W5PGU9	PLCG2	2.54E-05	0.00E+00
-4	CYTO	W5NTI0	TOP1	1.69E-02	0.00E+00
-4	CYTO	W5PP80	FRY	7.70E-04	1.16E-03
-4	CYTO	W5PFJ0	VCL	2.77E-02	0.00E+00
-4	CYTO	W5PX46	LOC101122591	2.02E-02	0.00E+00
-4	CYTO	W5P815	SEPTIN1	3.77E-02	0.00E+00
-4	CYTO	W5Q6F1		2.79E-02	0.00E+00
-4	CYTO	W5NTF8	SPNS1	2.97E-02	0.00E+00
-4	CYTO	W5PZG0	ADD1	9.29E-03	0.00E+00
-4	CYTO	W5P6T0		1.91E-03	1.16E-03
-4	CYTO	W5QEC3	METTL3	3.90E-03	0.00E+00
-4	CYTO	W5NV79	NUP210	1.38E-02	0.00E+00
-4	CYTO	W5PE21	PELP1	2.68E-02	0.00E+00
-4	CYTO	W5QEL7	NDRG2	3.89E-04	7.84E-03
-4	CYTO	W5P5Y4	RIPOR2	2.38E-03	0.00E+00
-4	CYTO	W5QBV0	SEPTIN3	7.74E-04	2.11E-04
-4	CYTO	W5QCW9	TST	4.94E-02	0.00E+00
-4	CYTO	W5PU80	MS4A1	1.13E-02	0.00E+00
-4	CYTO	W5NR20	LOC101109940	3.61E-02	9.89E-03
-4	CYTO	W5Q2S8	MYL9	2.37E-02	0.00E+00
-4	CYTO	W5QCH5	WDR47	1.70E-04	2.04E-04
-4	CYTO	W5QHW2	DAPK2	5.11E-02	0.00E+00
-4	CYTO	W5P2D1	TOP2B	4.17E-02	0.00E+00
-4	CYTO	W5NTL4	EVL	2.42E-02	0.00E+00
-4	CYTO	Q09YJ2	TES	1.87E-03	0.00E+00
-5	CYTO	W5Q271	MAD2L1	1.10E-04	2.10E-04
-5	CYTO	W5PTQ7	TRAF3IP3	1.27E-02	0.00E+00
-5	CYTO	W5Q6L9	GRAP2	5.07E-02	0.00E+00
-5	CYTO	W5PFI6	RASGRP2	1.14E-02	0.00E+00
-5	CYTO	W5Q1Z2		4.24E-03	5.84E-04
-5	CYTO	W5Q3B8		4.40E-02	0.00E+00
-5	CYTO	W5PJ75	SPTAN1	2.21E-03	0.00E+00
-5	CYTO	W5PA61		4.17E-03	0.00E+00
-5	CYTO	M4WG34	IL1b IL1B	3.04E-03	0.00E+00
-5	CYTO	W5Q6U5	ARMH3	1.09E-02	0.00E+00
-6	CYTO	W5PB37	RRAS2	6.20E-04	0.00E+00
-6	CYTO	W5QDM7	ZNF512	6.06E-03	0.00E+00
-6	CYTO	W5Q9S4	ING3	4.92E-03	1.16E-03
-6	CYTO	W5PHR3	ADD3	1.54E-03	0.00E+00
-6	CYTO	W5PYD8	PARP4	2.37E-03	9.21E-03
-6	CYTO	W5PL70	PDCD4	2.76E-03	0.00E+00
-6	CYTO	W5NZX9	SPTBN1	1.26E-03	0.00E+00
-6	CYTO	W5P733	PDLIM1	4.44E-02	0.00E+00
-6	CYTO	W5NT35	LOC443162	1.44E-02	0.00E+00
-7	CYTO	W5PRW4	TRIAP1	1.10E-04	2.04E-04
-7	CYTO	W5Q0K9	ABLIM1	3.17E-03	0.00E+00
-7	CYTO	W5PF87	ALOX15	8.61E-03	0.00E+00
-7	CYTO	W5P4L3	AVIL	2.57E-02	0.00E+00
-8	CYTO	W5QIK8	SELENBP1	8.29E-03	0.00E+00
7	SEC	W5QHV3	FABP1	3.58E-04	0.00E+00
7	SEC	P83205	CTSB	1.68E-02	0.00E+00
6	SEC	W5Q8M1	PRRC1	2.89E-02	1.60E-03
6	SEC	W5P3Q3	LOC100101238	2.79E-02	0.00E+00
5	SEC	W5P1H0	CTSC	2.79E-02	0.00E+00
5	SEC	W5Q8Y5	HDLBP	1.81E-03	0.00E+00
5	SEC	W5PDH4	MMP9	1.65E-04	0.00E+00
5	SEC	W5PF33	GM2A	1.06E-03	0.00E+00
4	SEC	W5NWF5	RARRES1	1.16E-02	0.00E+00
4	SEC	W5PUH5	LGMN	1.33E-03	0.00E+00
4	SEC	W5QHL1	FCGR1A	5.33E-03	0.00E+00
4	SEC	W5PDN3	SPTBN2	1.01E-03	0.00E+00
4	SEC	W5PIQ4	LOC101120093	1.58E-03	0.00E+00

Supplementary Table 5: Differentially abundant proteins (DAPs) of GMØ relative to M0 and their cellular location.

FoldChange	Location	Protein IDs	Gene Names	P-value	FDR
4	SEC	W5Q0F3	TGFBI	3.66E-03	0.00E+00
4	SEC	W5NUU7	CDH5	4.48E-02	0.00E+00
4	SEC	W5Q233	VCAN	2.72E-02	0.00E+00
3	SEC	W5NSH8	NPC2	5.34E-02	0.00E+00
3	SEC	W5Q3K6		9.72E-03	0.00E+00
3	SEC	W5PAJ2	PSAP	1.72E-02	0.00E+00
3	SEC	W5PYS8		1.88E-02	0.00E+00
3	SEC	W5P530	LOC101104705	2.46E-03	0.00E+00
3	SEC	W5NTD9	CHI3L1	2.43E-02	0.00E+00
2	SEC	W5PGV0	ITGAM	3.11E-03	0.00E+00
2	SEC	W5P8R4	CSF1R	3.45E-02	0.00E+00
2	SEC	W5NTT7	COL1A2	1.46E-02	0.00E+00
2	SEC	P12303	TTR	6.62E-04	0.00E+00
2	SEC	W5QGP4	APOD	2.02E-03	9.33E-03
2	SEC	W5PQ07	LOXL4	1.88E-02	0.00E+00
2	SEC	W5P996	PDGFRB	5.01E-02	0.00E+00
2	SEC	W5PBM9	SCPEP1	2.28E-02	0.00E+00
2	SEC	W5P6G7	TG	3.72E-02	0.00E+00
2	SEC	W5NZ47	RBP4	3.90E-02	0.00E+00
2	SEC	W5P4M1	PHB2	1.70E-02	0.00E+00
-2	SEC	W5NZX1		5.25E-02	0.00E+00
-2	SEC	P35623	SHMT1	8.62E-04	0.00E+00
-2	SEC	W5NPP2	CPM	1.49E-02	0.00E+00
-2	SEC	W5PGT0	MYH11	3.17E-02	1.59E-03
-2	SEC	W5PT68	FLNB	3.47E-02	0.00E+00
-2	SEC	W5Q0Y4	ZFAND6	2.44E-02	0.00E+00
-2	SEC	W5NS10	TFPI2	3.11E-02	0.00E+00
-2	SEC	W5P5B6	VSIG4	2.31E-02	0.00E+00
-2	SEC	W5P7B1	SIRT2	1.91E-02	4.56E-03
-2	SEC	W5P4A8	RNASET2	1.65E-02	0.00E+00
-2	SEC	W5NTX3		4.24E-02	0.00E+00
-2	SEC	W5PLU6		3.21E-02	0.00E+00
-2	SEC	W5PSA3	TCN1	5.47E-02	0.00E+00
-2	SEC	W5PIN1	UFC1	2.51E-02	0.00E+00
-2	SEC	W5P4V3	MMP3	2.17E-02	0.00E+00
-2	SEC	W5Q285	PDCD6	8.51E-03	0.00E+00
-2	SEC	W5Q1W2	SDCBP	4.12E-02	0.00E+00
-2	SEC	W5Q5T7	ATP6V1C1	8.09E-03	0.00E+00
-2	SEC	W5NVW7	NAGLU	9.21E-03	0.00E+00
-2	SEC	W5QDI7	CSF1	1.82E-02	0.00E+00
-2	SEC	W5QAB7	RANBP1	2.43E-02	0.00E+00
-2	SEC	W5PDD2	TLL2	1.29E-03	6.82E-03
-2	SEC	W5QFK2	MACF1	3.87E-02	0.00E+00
-2	SEC	W5PF04	MAN1A1	4.98E-02	0.00E+00
-2	SEC	W5PQF0	CCT7	4.58E-02	0.00E+00
-2	SEC	W5Q9H2		3.25E-02	0.00E+00
-2	SEC	E7ECV8	NUDT9	1.34E-03	0.00E+00
-2	SEC	W5P8Y7		3.30E-02	3.83E-03
-2	SEC	W5P6X2	IGFBP7	2.78E-02	4.98E-03
-2	SEC	W5NRI1		5.00E-02	0.00E+00
-2	SEC	W5NPK5	LOC443475	1.54E-02	0.00E+00
-3	SEC	W5P316	NAMPT	2.10E-02	0.00E+00
-3	SEC	W5PAH6	COPB1	4.15E-02	0.00E+00
-3	SEC	W5Q2I7	USO1	1.19E-02	0.00E+00
-3	SEC	W5NUI6	SGSH	5.39E-02	0.00E+00
-3	SEC	W5PZM9	ANXA5	2.76E-02	0.00E+00
-3	SEC	W5PTA8	FURIN	1.92E-02	4.54E-03
-3	SEC	W5NXX1	IPO5	9.49E-03	0.00E+00
-3	SEC	W5NPI6	PABPC1	5.25E-02	0.00E+00
-3	SEC	W5Q1M0	GLB1	2.16E-02	0.00E+00
-3	SEC	W5PJN6	KYNU	2.73E-02	5.55E-03
-3	SEC	W5QH42	MAT2A	4.59E-02	0.00E+00
-3	SEC	W5QI35	IL1RN	9.73E-03	0.00E+00

Supplementary Table 5: Differentially abundant proteins (DAPs) of GMØ relative to M0 and their cellular location.

FoldChange	Location	Protein IDs	Gene Names	P-value	FDR
-3	SEC	W5PEN2	TPD52	2.77E-02	0.00E+00
-3	SEC	W5QAU1		2.67E-02	0.00E+00
-3	SEC	W5PV50	ADA2	1.50E-02	4.46E-03
-3	SEC	W5Q9X5	MAP2K1	2.31E-02	0.00E+00
-3	SEC	W5PXR1	ENPP1	2.35E-03	0.00E+00
-3	SEC	W5QCJ4	COMMD9	5.91E-03	0.00E+00
-3	SEC	W5PKR1		5.11E-02	4.44E-03
-3	SEC	W5PEY4	TCN2	7.41E-03	0.00E+00
-3	SEC	W5NYV5	SPAG9	2.31E-02	1.60E-03
-3	SEC	W5PI89	RO60	4.58E-02	0.00E+00
-3	SEC	W5QHU9	PPIB	1.42E-02	0.00E+00
-3	SEC	W5PEL1	NACA	3.30E-02	0.00E+00
-3	SEC	W5Q203		4.96E-02	0.00E+00
-3	SEC	W5Q9D0	PPP1R7	3.77E-02	0.00E+00
-3	SEC	W5PL66	PPA2	1.16E-02	0.00E+00
-3	SEC	W5Q1E7	KRT12	3.38E-03	4.33E-03
-3	SEC	W5PYG7	EIF3L EIF3EIP EIF3S	2.80E-02	0.00E+00
-3	SEC	W5PMM7	PDIA3	4.66E-02	0.00E+00
-3	SEC	W5QCL8	NPL	1.22E-02	0.00E+00
-3	SEC	W5P9K6	PSMC3	3.97E-02	0.00E+00
-3	SEC	W5PGJ7	LOC101105208	4.83E-02	0.00E+00
-3	SEC	W5NY99		5.53E-02	0.00E+00
-3	SEC	W5P8Q0	AP2B1	2.29E-03	0.00E+00
-3	SEC	W5QHL5	CAB39	1.63E-02	0.00E+00
-3	SEC	W5QH24		3.16E-02	0.00E+00
-3	SEC	W5Q635	CASP8	1.09E-02	2.37E-03
-3	SEC	W5QBQ9	MYH9	6.52E-03	0.00E+00
-3	SEC	P60713	ACTB	4.73E-04	0.00E+00
-3	SEC	W5Q563	GFUS	3.30E-02	3.10E-03
-3	SEC	W5NWT1	RGS10	9.04E-03	0.00E+00
-3	SEC	W5PI22	CNPY2	1.50E-02	0.00E+00
-3	SEC	W5P9Y1	SNX6	2.22E-02	0.00E+00
-3	SEC	W5NUG0	GOT2	3.91E-02	0.00E+00
-3	SEC	W5QC41	PKM	6.08E-03	0.00E+00
-3	SEC	W5NRJ3		1.96E-02	3.87E-03
-3	SEC	W5NRL8	EIF3A EIF3S10	3.72E-02	0.00E+00
-3	SEC	P82197	PDXK PKH	1.51E-03	3.84E-03
-3	SEC	W5Q0B6	PPP1R12A	2.60E-03	2.35E-03
-3	SEC	W5QFH1	ACTC1	2.67E-03	0.00E+00
-3	SEC	W5NRK4	GLUD1	7.95E-03	0.00E+00
-4	SEC	W5P2U9	LRRC59	1.57E-02	0.00E+00
-4	SEC	W5QHE9	STRAP	4.97E-02	0.00E+00
-4	SEC	W5PNY4	PDXK	4.86E-02	0.00E+00
-4	SEC	P50413	TXN	4.56E-02	0.00E+00
-4	SEC	W5PH25	USP14	4.74E-03	0.00E+00
-4	SEC	W5Q1T4		4.05E-02	0.00E+00
-4	SEC	W5Q4B7	RAP1GDS1	4.61E-02	4.33E-03
-4	SEC	W5PFM8	ADK	5.56E-02	0.00E+00
-4	SEC	W5Q7E2	GPS1	1.12E-02	0.00E+00
-4	SEC	W5PU89	ALDH16A1	4.48E-02	0.00E+00
-4	SEC	B7TJ15	MAPK14	2.87E-02	0.00E+00
-4	SEC	W5P375	TCP1	7.07E-03	0.00E+00
-4	SEC	W5PMA3	LOC101105094	2.67E-03	0.00E+00
-4	SEC	P09670	SOD1	1.04E-02	5.59E-03
-4	SEC	W5QBQ8	RAB5C	2.34E-02	0.00E+00
-4	SEC	W5PLQ6	ARCN1	6.05E-03	0.00E+00
-4	SEC	W5NYH2	LOC101102072	4.89E-03	0.00E+00
-4	SEC	W5PF71	KPNB1	1.12E-02	0.00E+00
-4	SEC	W5P5W9	TPI1	2.22E-02	0.00E+00
-4	SEC	W5P2J9		2.43E-04	0.00E+00
-4	SEC	Q09YJ2	TES	2.92E-03	0.00E+00
-4	SEC	W5Q0Q1	YWHAQ	3.13E-02	0.00E+00
-4	SEC	W5Q3E3	RAB2A	7.55E-03	0.00E+00

Supplementary Table 5: Differentially abundant proteins (DAPs) of GMØ relative to M0 and their cellular location.

FoldChange	Location	Protein IDs	Gene Names	P-value	FDR
-4	SEC	W5Q1I6	IMPDH2 IMPDH	1.66E-03	0.00E+00
-4	SEC	Q2TCH3	ACLY	5.41E-02	0.00E+00
-4	SEC	W5P363	VAT1	1.50E-02	0.00E+00
-4	SEC	W5PJB6	PGM1	3.69E-03	0.00E+00
-4	SEC	W5Q121		7.11E-03	0.00E+00
-4	SEC	P80190		4.01E-02	0.00E+00
-4	SEC	W5Q6V5	DNM2	1.14E-02	0.00E+00
-4	SEC	W5QI99	NEDD4	2.76E-03	0.00E+00
-4	SEC	W5PYQ9	CPNE1	5.44E-04	0.00E+00
-4	SEC	W5PQ76	SRI	2.81E-03	0.00E+00
-4	SEC	C5IWV1	FH	4.49E-02	0.00E+00
-4	SEC	W5P4J1	VAV1	2.13E-02	0.00E+00
-4	SEC	W5PR23	CSE1L	5.85E-03	4.34E-03
-4	SEC	W5PAX6	COPB2	3.37E-02	0.00E+00
-4	SEC	W5NVQ4	CAND1	1.24E-02	0.00E+00
-4	SEC	W5QHD8	PSMD2	4.69E-02	0.00E+00
-4	SEC	W5PDU8	GARS1	8.26E-04	0.00E+00
-4	SEC	W5PDC7	PSMB9	4.15E-02	0.00E+00
-4	SEC	W5QG77	CD58	3.22E-02	0.00E+00
-4	SEC	W5QGT4		3.14E-02	0.00E+00
-4	SEC	W5Q1R1	VPS35	2.54E-02	0.00E+00
-4	SEC	W5PLZ0	ATP6V1B2	1.46E-02	0.00E+00
-4	SEC	W5Q5R8	TXNRD1	7.48E-03	0.00E+00
-4	SEC	W5Q2D9	RAB5A	9.39E-03	0.00E+00
-4	SEC	W5PQK7	EIF5A	2.26E-02	0.00E+00
-4	SEC	W5P508	EIF3B EIF3S9	5.25E-02	0.00E+00
-4	SEC	W5PW97	PSMC5	1.08E-02	0.00E+00
-4	SEC	W5PJ98		5.19E-02	0.00E+00
-4	SEC	W5PE11	CDC42	4.39E-03	0.00E+00
-4	SEC	W5NVY0		5.12E-02	0.00E+00
-4	SEC	W5QJ49	ATP6V1D	5.77E-03	0.00E+00
-4	SEC	W5NU60	BTK	4.41E-02	4.59E-03
-4	SEC	W5Q1R8	MAPK1	1.94E-03	4.44E-03
-4	SEC	W5NSV5	ITGA6	1.29E-02	0.00E+00
-4	SEC	W5P538	CD93	6.81E-04	0.00E+00
-4	SEC	W5Q3X1		9.78E-03	3.84E-03
-4	SEC	W5PX22	SNRPD3	5.06E-02	0.00E+00
-4	SEC	W5PSP7	RUVBL2	4.94E-02	0.00E+00
-4	SEC	W5NX31	AHSA1	4.94E-03	0.00E+00
-4	SEC	W5PUJ4	SYNCRIP	1.80E-02	0.00E+00
-4	SEC	W5PZ86	SEPTIN8	7.92E-03	0.00E+00
-4	SEC	W5PIM8	GALK1	1.59E-02	0.00E+00
-4	SEC	W5P8K0	DCTN2	9.18E-03	0.00E+00
-4	SEC	W5P6M2	PSMD5	3.53E-02	0.00E+00
-4	SEC	W5Q701	LOC101112491	5.27E-03	0.00E+00
-4	SEC	W5P7J7	TWF2	2.97E-02	0.00E+00
-4	SEC	W5NSD9	REXO2	1.26E-02	8.68E-03
-4	SEC	W5P707	ACTN4	2.22E-02	0.00E+00
-4	SEC	W5P880	PRG4	1.37E-03	0.00E+00
-4	SEC	W5PVT6	UBA1	2.08E-02	0.00E+00
-4	SEC	W5QHS2	MGP	1.65E-02	0.00E+00
-4	SEC	W5P824	LOC101103096	5.28E-02	0.00E+00
-4	SEC	W5PDG3	GAPDH	5.52E-02	0.00E+00
-4	SEC	W5PUH7		1.41E-02	0.00E+00
-4	SEC	W5Q1I8	PPM1F	6.08E-03	0.00E+00
-4	SEC	W5QCP6	PSMC2	3.51E-02	0.00E+00
-4	SEC	W5PVL6	AP2A1	1.15E-03	0.00E+00
-4	SEC	W5PK27		3.20E-02	0.00E+00
-4	SEC	W5QCL5	CSNK2A1	1.86E-03	0.00E+00
-4	SEC	W5PSZ3	JARID2	1.33E-02	4.55E-03
-4	SEC	W5QFL1	LOC101116286	4.59E-03	0.00E+00
-4	SEC	W5NVD7	COPS4	4.66E-02	8.04E-03
-4	SEC	W5PW05	MDH2	1.80E-02	0.00E+00

Supplementary Table 5: Differentially abundant proteins (DAPs) of GMØ relative to M0 and their cellular location.

FoldChange	Location	Protein IDs	Gene Names	P-value	FDR
-4	SEC	W5QD23	LANCL1	3.34E-02	0.00E+00
-4	SEC	W5PFV5	NPEPPS	1.03E-02	0.00E+00
-5	SEC	W5PC25	LOC101116273	3.97E-04	0.00E+00
-5	SEC	W5QFM1		5.45E-02	0.00E+00
-5	SEC	W5QEU6	ANXA4	2.11E-02	0.00E+00
-5	SEC	W5P4F9	PRKAR2B	2.64E-03	5.56E-03
-5	SEC	W5Q3B7	PDE5A	3.91E-02	0.00E+00
-5	SEC	W5P2V0	EZR	9.58E-03	0.00E+00
-5	SEC	W5P689	USP5	3.97E-02	0.00E+00
-5	SEC	W5Q3N5	PRKAR2A	2.46E-04	0.00E+00
-5	SEC	W5PHU7	UNC13D	5.08E-03	0.00E+00
-5	SEC	W5P4R1	MSN	1.07E-02	0.00E+00
-5	SEC	W5P500	PSMB1	1.11E-02	0.00E+00
-5	SEC	W5NS93	PSMA4	1.01E-02	0.00E+00
-5	SEC	W5P7L2	ATP6V1G1	1.09E-03	0.00E+00
-5	SEC	W5PVP5	APRT	1.17E-02	0.00E+00
-5	SEC	W5PLS7	GRB2	1.33E-03	0.00E+00
-5	SEC	W5PE22	GDI2	1.68E-03	0.00E+00
-5	SEC	W5Q9B2	ARHGDIA	3.29E-02	0.00E+00
-5	SEC	W5P5C4	NARS1	3.87E-03	0.00E+00
-5	SEC	W5P1G7		4.70E-02	0.00E+00
-5	SEC	W5PVL5	HSPA4	1.29E-02	0.00E+00
-5	SEC	W5PI50	GLRX	5.07E-02	0.00E+00
-5	SEC	W5P4X6	LOC101104287	1.38E-03	0.00E+00
-5	SEC	W5QIY3	PSMA3	3.26E-02	0.00E+00
-5	SEC	W5P7E2	PPA1	5.41E-03	0.00E+00
-5	SEC	W5P6X5	STMN1	8.12E-04	0.00E+00
-5	SEC	W5PMP1	COTL1	8.35E-05	0.00E+00
-5	SEC	W5NSM1	ACTR1A	8.64E-04	0.00E+00
-5	SEC	P68253	YWHAG	2.91E-02	0.00E+00
-5	SEC	W5P694	RPLP2	2.87E-02	0.00E+00
-5	SEC	W5Q9M6		8.82E-03	0.00E+00
-5	SEC	W5NUN8	AKR1A1	1.77E-02	0.00E+00
-5	SEC	W5PFV7	COPA	6.14E-03	0.00E+00
-5	SEC	W5P7Z1		9.14E-03	0.00E+00
-5	SEC	W5PU66	IQGAP1	2.94E-02	0.00E+00
-5	SEC	W5P5M7	VPS26A	1.61E-02	0.00E+00
-5	SEC	W5PNZ5	FKBP4	1.40E-02	0.00E+00
-5	SEC	W5NZ57	PSMB10	9.02E-03	0.00E+00
-5	SEC	A8DR93	HSPCA HSP90AA1	2.03E-03	0.00E+00
-5	SEC	W5Q086	PRKAR1A	5.15E-03	0.00E+00
-5	SEC	W5Q5G8	TKT	9.88E-03	0.00E+00
-5	SEC	W5P583	ACP1	8.58E-04	0.00E+00
-5	SEC	W5NQK6	LIMS1	1.35E-03	0.00E+00
-5	SEC	W5PYT7		1.39E-03	0.00E+00
-5	SEC	W5P031	HDHD2	1.39E-02	0.00E+00
-5	SEC	W5NQ14	EIF3I EIF3S2	1.33E-03	0.00E+00
-5	SEC	W5PQI3	ITGB3	3.02E-03	0.00E+00
-5	SEC	W5P610	CLIC1	3.32E-02	0.00E+00
-5	SEC	W5PWG1	PFKP	3.25E-03	0.00E+00
-5	SEC	W5PR73	CORO1B	1.76E-03	0.00E+00
-5	SEC	W5NPQ6	CAPZA2	1.66E-04	0.00E+00
-5	SEC	W5PK13	CBX3	1.97E-02	0.00E+00
-5	SEC	W5NVC9	RAC1	6.27E-03	0.00E+00
-5	SEC	W5PHX1	AHCY	2.40E-02	0.00E+00
-5	SEC	Q5MIB6	PYGB	6.65E-03	0.00E+00
-5	SEC	W5PTM9	UBE2V2	1.25E-03	0.00E+00
-5	SEC	W5QBL7	ARPC1B	5.50E-02	0.00E+00
-5	SEC	W5PMQ9	SAE1	9.51E-04	0.00E+00
-5	SEC	W5QIU5	GMFB	3.65E-02	0.00E+00
-5	SEC	W5QCX9	TTLL12	5.83E-03	0.00E+00
-5	SEC	P81184	LGALS1	5.03E-02	0.00E+00
-5	SEC	W5QHQ7	NCL	2.18E-02	0.00E+00

Supplementary Table 5: Differentially abundant proteins (DAPs) of GMØ relative to M0 and their cellular location.

FoldChange	Location	Protein IDs	Gene Names	P-value	FDR
-5	SEC	W5QCS4	SARS1	4.35E-02	0.00E+00
-5	SEC	W5QCP7	PACSIN2	7.25E-04	0.00E+00
-5	SEC	W5PIG7	ENO1	3.69E-04	0.00E+00
-5	SEC	W5PWN6	PPP2CA	3.12E-02	0.00E+00
-5	SEC	W5PJC2	PSMB3	6.37E-03	0.00E+00
-5	SEC	W5Q9T3	LMNB1	1.03E-02	0.00E+00
-5	SEC	W5P5F6	TBCB	2.13E-03	4.56E-03
-5	SEC	W5QE19	PSMB2	5.16E-03	0.00E+00
-5	SEC	W5PPT6	TUBB	3.03E-03	0.00E+00
-5	SEC	W5PM33	SELP	1.63E-02	0.00E+00
-5	SEC	W5NW17	STXBP2	1.97E-02	0.00E+00
-5	SEC	W5PK04	PGAM1	3.04E-02	0.00E+00
-5	SEC	W5PQ98	ACTR3	2.35E-02	0.00E+00
-5	SEC	W5PK95	HNRNPA2B1	3.58E-02	0.00E+00
-5	SEC	W5PNY5	ARRB1	2.50E-03	0.00E+00
-5	SEC	W5NTW1		3.44E-03	0.00E+00
-5	SEC	C8BKE1	STAT1 STAT4	9.18E-05	0.00E+00
-5	SEC	Q1ZZU7	MIF	5.16E-03	0.00E+00
-5	SEC	W5PKD3	GNAI2	2.41E-02	0.00E+00
-5	SEC	W5QD41	PSME2	1.77E-02	0.00E+00
-5	SEC	W5QIL2	PSMB4	1.30E-02	0.00E+00
-6	SEC	W5PR48	HPRT1	3.04E-02	0.00E+00
-6	SEC	W5Q6U0	FASN	2.47E-03	0.00E+00
-6	SEC	W5NW82	NAP1L4	1.26E-04	0.00E+00
-6	SEC	W5Q4L0		2.25E-03	0.00E+00
-6	SEC	W5Q9P7	CFL1	4.60E-03	0.00E+00
-6	SEC	P00349	PGD	1.10E-02	0.00E+00
-6	SEC	W5PZN3	NAP1L1	1.26E-03	0.00E+00
-6	SEC	W5QFG8	ACTR2	3.15E-02	0.00E+00
-6	SEC	W5P6Z6	MAPRE2	3.59E-03	0.00E+00
-6	SEC	W5PNV2	PPP1CA	1.74E-02	0.00E+00
-6	SEC	W5PIJ6	PTPN11	9.85E-03	0.00E+00
-6	SEC	W5PUT6	CLTC	1.05E-02	0.00E+00
-6	SEC	W5NXE5	LOC101104501	4.63E-02	0.00E+00
-6	SEC	W5P8R3	HDGF	4.61E-02	0.00E+00
-6	SEC	W5PR81	TARS1	5.28E-02	0.00E+00
-6	SEC	W5PSZ5	ANXA1	2.54E-02	0.00E+00
-6	SEC	W5NSD5	RAP1B	2.15E-03	0.00E+00
-6	SEC	W5P1X9	ALDOA	1.50E-02	0.00E+00
-6	SEC	W5QD49	PSME1	8.05E-03	0.00E+00
-6	SEC	W5NZQ2	LAP3	1.49E-02	0.00E+00
-6	SEC	W5P889	SEPTIN9	2.08E-03	0.00E+00
-6	SEC	W5PE01	TAGLN2	2.10E-04	0.00E+00
-6	SEC	W5QFH5	RAB1A	4.28E-04	0.00E+00
-6	SEC	W5Q0N0	LOC101119757	1.70E-03	0.00E+00
-6	SEC	W5P8B4	TRAPPC8	4.73E-03	5.53E-03
-6	SEC	W5Q3A5	PAFAH1B1 LIS1	2.73E-03	0.00E+00
-6	SEC	W5PTZ9	LOC101120877	2.42E-03	0.00E+00
-6	SEC	W5Q731	ILK	9.28E-03	0.00E+00
-6	SEC	W5P9U4	PSMA1	1.06E-02	0.00E+00
-6	SEC	W5PK66	PARK7	1.53E-02	0.00E+00
-6	SEC	W5P323	GPI	6.51E-04	0.00E+00
-6	SEC	W5PYM5	CCT8	1.23E-02	0.00E+00
-6	SEC	W5PIN6	LDHA	7.39E-03	0.00E+00
-6	SEC	W5Q3M9	SEPTIN6	2.35E-03	0.00E+00
-6	SEC	W5P0V5	RAB11B	1.21E-04	0.00E+00
-6	SEC	W5NRD9		1.11E-03	1.59E-03
-6	SEC	W5PVX4	DBNL	1.18E-03	0.00E+00
-6	SEC	W5PRP1	SLC9A3R1	5.52E-04	0.00E+00
-6	SEC	W5PYK0	SENP3	3.66E-02	0.00E+00
-6	SEC	P62262	YWHAE	2.23E-02	0.00E+00
-6	SEC	W5PRR5	LTA4H	6.18E-03	0.00E+00
-6	SEC	W5Q0L1	EEF1G	8.45E-03	0.00E+00

Supplementary Table 5: Differentially abundant proteins (DAPs) of GMØ relative to M0 and their cellular location.

FoldChange	Location	Protein IDs	Gene Names	P-value	FDR
-6	SEC	C5IWU0	ARF1 LOC1011231	1.17E-03	0.00E+00
-6	SEC	W5PQK3	PFKL	4.93E-03	0.00E+00
-6	SEC	W5NY22	PCMT1	2.23E-03	0.00E+00
-6	SEC	B0FZL9	SRSF3	1.29E-02	0.00E+00
-6	SEC	W5P5A0	FLNA	3.19E-04	0.00E+00
-6	SEC	W5P9L9		1.88E-02	0.00E+00
-6	SEC	W5PZ65	PSMA7	6.29E-04	0.00E+00
-6	SEC	W5PRJ4	VCP	1.12E-03	0.00E+00
-6	SEC	W5PH15	RSU1	3.06E-03	0.00E+00
-6	SEC	W5Q694		1.30E-02	0.00E+00
-6	SEC	W5NU34	TREML1	1.70E-03	0.00E+00
-6	SEC	W5PJX1	CCT6A	1.45E-02	0.00E+00
-6	SEC	W5QBD7	YWHAZ	1.15E-03	0.00E+00
-6	SEC	W5NXW9		2.96E-04	0.00E+00
-6	SEC	W5NUV1	GNB1	7.44E-04	0.00E+00
-6	SEC	W5QEK8	EHD3	3.22E-04	0.00E+00
-6	SEC	W5P359	STIP1	7.01E-04	0.00E+00
-6	SEC	W5PPG3	ALDH9A1	1.13E-02	0.00E+00
-6	SEC	W5QD96	PARVB	7.39E-04	0.00E+00
-6	SEC	W5P7X3		1.13E-04	0.00E+00
-6	SEC	W5NRW4	TPM4	3.11E-03	0.00E+00
-6	SEC	W5QFN2	HCLS1	5.51E-05	0.00E+00
-6	SEC	W5PRF0	SUB1	5.87E-04	0.00E+00
-6	SEC	W5P733	PDLIM1	1.12E-02	0.00E+00
-6	SEC	W5P4L3	AVIL	1.44E-03	0.00E+00
-7	SEC	W5P765	CCT3	2.42E-03	0.00E+00
-7	SEC	Q5MIB5	PYGL	4.86E-04	0.00E+00
-7	SEC	W5PYQ7	RPS12	9.58E-03	0.00E+00
-7	SEC	W5PSM0	OSTF1	1.48E-03	0.00E+00
-7	SEC	W5Q7C8	BIN2	1.19E-03	0.00E+00
-7	SEC	W5QBV3	PGK1	4.16E-03	0.00E+00
-7	SEC	W5PXX7	CCT5	4.47E-03	0.00E+00
-7	SEC	C5IJA0	RAN	1.32E-03	0.00E+00
-7	SEC	W5PY17	STX7	1.71E-04	0.00E+00
-7	SEC	W5NPN4	HSPA8	1.12E-02	0.00E+00
-7	SEC	W5NUT8	PIP4K2A	2.02E-04	0.00E+00
-7	SEC	W5PG09	PGK2	3.04E-03	0.00E+00
-7	SEC	W5PX84	CCDC171	1.24E-04	4.57E-03
-7	SEC	W5PC09	PSMA6	1.45E-03	0.00E+00
-7	SEC	W5PLD5	CNN2	2.83E-04	0.00E+00
-7	SEC	W5P3E8	GDI1	1.15E-04	0.00E+00
-7	SEC	W5Q8I4	ST13	1.69E-03	0.00E+00
-7	SEC	W5QB61	FKBP1A	3.89E-04	0.00E+00
-7	SEC	W5P716		2.48E-03	0.00E+00
-7	SEC	W5QE14	PCBP1	1.18E-03	0.00E+00
-7	SEC	W5NYA7	LOC101114319	4.32E-03	0.00E+00
-7	SEC	W5QG16	CAP1	3.87E-05	0.00E+00
-7	SEC	W5Q951	CORO1C	1.62E-03	0.00E+00
-7	SEC	W5PK85	EML2	2.83E-03	0.00E+00
-7	SEC	W5PE27	ESD	2.95E-04	0.00E+00
-7	SEC	W5QGG6	CCT2	2.34E-03	0.00E+00
-7	SEC	W5NX91	RPS27A	2.48E-02	0.00E+00
-7	SEC	W5P4C7	SEPTIN7	3.73E-04	0.00E+00
-7	SEC	W5PP64	FHL1	3.93E-04	0.00E+00
-7	SEC	W5QGD1	LDHB	9.41E-03	0.00E+00
-8	SEC	W5PG95	HSPA1A	1.07E-03	0.00E+00
-8	SEC	W5PEX2	GPATCH8	4.24E-04	0.00E+00
-8	SEC	Q28554	GAPDH G3PDH GA	1.61E-03	0.00E+00
-8	SEC	W5Q2S8	MYL9	4.04E-04	0.00E+00
-8	SEC	W5P098	CORO1A	1.29E-02	0.00E+00
-8	SEC	W5PFJ0	VCL	1.54E-04	0.00E+00
-8	SEC	W5PQA8	PRDX6	4.34E-03	0.00E+00
-8	SEC	W5QFZ3	CCT4	6.73E-04	0.00E+00

Supplementary Table 5: Differentially abundant proteins (DAPs) of GMØ relative to M0 and their cellular location.

FoldChange	Location	Protein IDs	Gene Names	P-value	FDR
-8	SEC	W5PL19	LOC101105123	1.50E-03	0.00E+00
-9	SEC	W5PD82	CALD1	1.16E-03	0.00E+00
-9	SEC	W5PHW0	HSP90AB1	9.47E-04	0.00E+00
-9	SEC	W5PDL1	WDR1	1.44E-03	0.00E+00
-9	SEC	W5Q9H1	ZYX	8.70E-03	0.00E+00
-9	SEC	W5PK38	VASP	1.89E-04	0.00E+00
-9	SEC	W5PFI7	VCL	1.81E-04	0.00E+00
-9	SEC	W5QAR2	F13A1	6.18E-03	0.00E+00
-9	SEC	W5P409	FERMT3	9.81E-04	0.00E+00
-9	SEC	W5PYX0		5.44E-03	0.00E+00
-9	SEC	C5ISA2	TUBA4A	2.21E-02	0.00E+00
-9	SEC	W5QF71	PLEK	2.01E-04	0.00E+00
-9	SEC	W5QIK8	SELENBP1	1.73E-04	0.00E+00
-10	SEC	W5P627	GSN	5.97E-05	0.00E+00
-10	SEC	W5PQK6	TLN1	1.04E-04	0.00E+00
-10	SEC	W5Q3I7	TUBB1	1.88E-03	0.00E+00
-10	SEC	W5QJ62	ACTN1	2.11E-05	0.00E+00

GMØ, monocyte-derived macrophages differentiated with GM-CSF (Granulocyte-Macrophage Colony-Stimulating Factor) and M0. Monocytes at 3 hours. Differentially abundant proteins (DAPs) were identified using a threshold of false discovery rate (FDR, q-value) ≤ 0.05 and absolute fold change ≥ 2. Red-highlighted cells indicate shared differentially abundant proteins (DAPs) between the cell lysate (CYTO) and secretome (SEC) compartments in GMØ relative to monocytes, whereas non-highlighted cells represent compartment-specific DAPs unique to either the whole cell lysate or secretome.

Supplementary Table 6: Differentially abundant proteins (DAPs) of MMØ relative to M0 and their cellular location.

FoldChange	Location	Protein IDs	Gene Names	P-value	FDR
10	CYTO	W5PCH3	SCIN	4.35E-05	0.0000
10	CYTO	W5PE67		3.78E-05	0.0000
10	CYTO	W5NU86	GLA	5.61E-04	0.0000
9	CYTO	W5PD43	HTRA1	2.26E-03	0.0000
9	CYTO	W5PT76	GPNMB	8.82E-05	0.0000
8	CYTO	W5PAM4	CTSA	5.18E-05	0.0000
8	CYTO	W5PF33	GM2A	4.93E-05	0.0000
8	CYTO	W5QHG1	EPS8	7.34E-04	0.0000
8	CYTO	W5NY01		8.11E-04	0.0000
7	CYTO	P35623	SHMT1	1.92E-05	0.0000
7	CYTO	W5PIQ6	MSR1	7.62E-05	0.0000
7	CYTO	W5Q6N3	LOC101115115	1.06E-04	0.0000
7	CYTO	W5PXR1	ENPP1	3.46E-04	0.0000
7	CYTO	W5PTU7	CA2	6.13E-05	0.0000
7	CYTO	W5PQR0	NIBAN2	5.11E-06	0.0000
7	CYTO	P51977	ALDH1A1 ALDH1	1.93E-03	0.0000
7	CYTO	W5PBM9	SCPEP1	1.53E-04	0.0000
7	CYTO	W5P9M8	UCK1	2.71E-05	0.0002
7	CYTO	W5P9J8	BLVRB	1.24E-04	0.0000
7	CYTO	W5PFY5	ASAH1	1.60E-03	0.0000
7	CYTO	W5NX56	SPP1	9.19E-05	0.0000
7	CYTO	W5PGC5	GALM	5.94E-04	0.0000
7	CYTO	G3M9U4	ACP5	1.62E-04	0.0000
6	CYTO	W5P9G8	PLD3	2.32E-02	0.0000
6	CYTO	W5PCE0	PLBD2	4.28E-03	0.0000
6	CYTO	W5QC89	HEXA	1.10E-04	0.0000
6	CYTO	W5Q0Y4	ZFAND6	2.75E-03	0.0000
6	CYTO	A9YUY8	FABP4	8.63E-04	0.0000
6	CYTO	W5PZB2	CD68	1.49E-04	0.0000
6	CYTO	W5Q612	TRPV2	2.86E-05	0.0000
6	CYTO	W5NTZ3	RENBP	2.88E-04	0.0000
6	CYTO	W5Q2Y1	PLXNC1	6.97E-03	0.0000
6	CYTO	W5PAQ4	FUCA1	2.79E-04	0.0000
6	CYTO	W5QIW1	LGALS3	3.66E-03	0.0000
6	CYTO	W5PEB0	FABP7	1.29E-03	0.0000
6	CYTO	W5QCL8	NPL	5.43E-04	0.0000
6	CYTO	W5P026	STAB1	3.62E-05	0.0000
6	CYTO	W5PBS4	LRP1	2.88E-04	0.0000
6	CYTO	W5Q940	SHTN1	3.00E-03	0.0000
6	CYTO	W5PJS4	EMILIN2	1.67E-04	0.0000
6	CYTO	W5NUI6	SGSH	5.73E-05	0.0000
6	CYTO	W5NVW7	NAGLU	3.08E-03	0.0000
6	CYTO	Q9MZS8	CTSD	1.31E-03	0.0000
6	CYTO	W5P8M9		8.65E-04	0.0000
6	CYTO	W5P8H9	SGPL1	6.53E-04	0.0000
6	CYTO	W5QI00	LACTB	5.73E-04	0.0000
6	CYTO	W5P1A5	GBA1	5.41E-04	0.0000
6	CYTO	W5P5W6	NDRG1	9.95E-03	0.0000
6	CYTO	W5QEM8	LOC101111528	9.50E-05	0.0000
6	CYTO	W5QCD6	IDH1	2.87E-03	0.0000
6	CYTO	W5PZG5	OCRL	6.87E-04	0.0000
6	CYTO	W5PDH7	NPC1	8.96E-05	0.0000
6	CYTO	W5NZK6	PLA2G15	1.92E-04	0.0000
6	CYTO	W5QGG0	TFRC	2.76E-05	0.0000
6	CYTO	P05028	ATP1B1	2.26E-03	0.0000
5	CYTO	W5QBZ7	NAGA	2.57E-03	0.0000
5	CYTO	W5NPP2	CPM	1.71E-04	0.0000
5	CYTO	W5PI56	DAB2	2.50E-03	0.0000
5	CYTO	W5PKY1	HNMT	8.15E-04	0.0000
5	CYTO	W5PRI6	MRC1	1.45E-03	0.0000
5	CYTO	W5NX16		6.82E-05	0.0000
5	CYTO	W5P0C5	LGALS8	5.76E-05	0.0000
5	CYTO	W5NQS7	IFNGR1	1.35E-04	0.0000

Supplementary Table 6: Differentially abundant proteins (DAPs) of MMØ relative to M0 and their cellular location.

FoldChange	Location	Protein IDs	Gene Names	P-value	FDR
5	CYTO	W5PDJ0		9.23E-03	0.0000
5	CYTO	W5NYL7	MTHFD1L	1.11E-04	0.0000
5	CYTO	W5QHL1	FCGR1A	6.03E-04	0.0000
5	CYTO	W5P640	LMNA	3.40E-03	0.0000
5	CYTO	W5NRS0		9.20E-04	0.0000
5	CYTO	W5PWX3	CRYL1	4.48E-04	0.0000
5	CYTO	W5PV43	LRPAP1	3.81E-05	0.0000
5	CYTO	W5QI40	MYO1E	3.68E-03	0.0000
5	CYTO	W5NVR9	C21H11orf54	2.89E-05	0.0000
5	CYTO	W5QBM4	ALCAM	4.75E-04	0.0000
5	CYTO	W5Q678	SEC24D	6.14E-03	0.0000
5	CYTO	W5PG10	PAPSS1	1.06E-02	0.0000
5	CYTO	W5PYI8	WWC1	1.22E-03	0.0000
5	CYTO	W5PE92	GRN	3.81E-04	0.0000
5	CYTO	W5NYU9	MPP1	1.47E-05	0.0000
5	CYTO	W5PXS1	RAB3IL1	6.12E-04	0.0000
5	CYTO	W5PY08		6.80E-03	0.0000
5	CYTO	W5PPX2	SENP8	5.16E-04	0.0030
5	CYTO	W5PGS4	FABP5	5.39E-04	0.0000
5	CYTO	W5PBC0		3.92E-03	0.0000
5	CYTO	W5P7Y8	PALD1	4.71E-05	0.0000
5	CYTO	W5NZ62	GNS	3.48E-03	0.0000
5	CYTO	W5QFU4		7.64E-03	0.0000
5	CYTO	W5Q7I8	TAX1BP3	3.46E-02	0.0000
5	CYTO	W5QBG8	PPFIA1	4.74E-03	0.0000
5	CYTO	P83205	CTSB	2.50E-03	0.0000
5	CYTO	W5Q5T7	ATP6V1C1	1.03E-02	0.0000
5	CYTO	W5PKU3		7.64E-04	0.0000
5	CYTO	W5NSH8	NPC2	1.49E-05	0.0000
5	CYTO	W5QI78	CTSK	2.23E-02	0.0000
5	CYTO	Q29524	LPL	4.31E-03	0.0000
5	CYTO	W5Q3U3	LOC101102156	6.52E-03	0.0000
5	CYTO	W5P2V3	PEPD	2.18E-03	0.0000
5	CYTO	W5P2F1	FOLR2	1.14E-05	0.0000
5	CYTO	W5Q5C8	SOAT1	4.09E-04	0.0000
5	CYTO	W5Q0F1	LIPA	3.44E-04	0.0000
5	CYTO	P12303	TTR	1.10E-02	0.0000
4	CYTO	W5P1H0	CTSC	5.68E-06	0.0000
4	CYTO	W5Q5W2	LOC101110539	2.68E-04	0.0000
4	CYTO	W5P5Q2	MVP	1.16E-02	0.0000
4	CYTO	W5PP04	GNG12	8.75E-04	0.0000
4	CYTO	W5PJY6	ADAM28	2.53E-03	0.0000
4	CYTO	O18882	ATP6V0C ATP6C A	2.26E-03	0.0000
4	CYTO	W5PEE9	LAMP1	1.05E-02	0.0000
4	CYTO	W5Q0A3	TLR2	1.78E-03	0.0000
4	CYTO	W5Q8J3	RRBP1	6.57E-03	0.0000
4	CYTO	W5NYE0	ATP6V0D1	3.49E-02	0.0000
4	CYTO	W5P3H8	IGF2R	1.86E-03	0.0000
4	CYTO	W5NU23	FUCA2	2.90E-04	0.0000
4	CYTO	W5PNC8	PADI2	1.06E-02	0.0000
4	CYTO	W5PAC2	LOC101105044	9.35E-03	0.0000
4	CYTO	W5PYW0	TCIRG1	2.98E-04	0.0000
4	CYTO	W5NYL0	MAOA	5.92E-03	0.0000
4	CYTO	W5QH35	CAPG	4.17E-04	0.0000
4	CYTO	W5NUC8	ARMCX3	4.25E-03	0.0000
4	CYTO	W5PUL5	FCGRT	1.03E-03	0.0000
4	CYTO	W5Q777	HS1BP3	3.09E-04	0.0000
4	CYTO	W5PVR9	ERMP1	2.46E-02	0.0000
4	CYTO	W5Q0U0	P2RX4	3.22E-03	0.0000
4	CYTO	W5QB71	AMDHD2	1.27E-03	0.0000
4	CYTO	W5NSQ3	HFE	2.77E-04	0.0000
4	CYTO	W5QA16		1.83E-04	0.0000
4	CYTO	W5QEH8	CTTNBP2NL	1.37E-05	0.0000

Supplementary Table 6: Differentially abundant proteins (DAPs) of MMØ relative to M0 and their cellular location.

FoldChange	Location	Protein IDs	Gene Names	P-value	FDR
4	CYTO	W5PE91	LOC101105400	3.31E-04	0.0002
4	CYTO	Q9MZD1	SLC17A5	8.13E-04	0.0000
4	CYTO	W5NS94		4.20E-05	0.0000
4	CYTO	W5QJ37		3.55E-05	0.0000
4	CYTO	C5IJ93	RAB9A	2.68E-05	0.0000
4	CYTO	W5P3N6	LOC101112162	8.05E-04	0.0000
4	CYTO	W5Q700	APPL2	3.00E-05	0.0000
4	CYTO	W5NW80	GAA	2.58E-03	0.0000
4	CYTO	W5P168	SH3PXD2B	1.81E-05	0.0000
4	CYTO	W5PBG1		7.32E-03	0.0000
4	CYTO	W5PDZ1		1.29E-03	0.0000
4	CYTO	W5P530	LOC101104705	5.19E-02	0.0000
4	CYTO	W5PA90	AGA	1.20E-04	0.0000
4	CYTO	W5NUQ8	GCC1	1.26E-02	0.0000
4	CYTO	W5QEH0	TWF1	3.98E-03	0.0000
4	CYTO	W5P3L5	RNF13	2.52E-03	0.0000
4	CYTO	W5P5L4	COG5	1.02E-04	0.0000
4	CYTO	W5Q2U7	PLEC	1.21E-06	0.0000
4	CYTO	W5Q3N1	CTSZ	2.79E-05	0.0000
4	CYTO	W5PFE7	ACOX1	5.07E-04	0.0000
4	CYTO	W5P4A8	RNASET2	4.33E-04	0.0000
4	CYTO	W5Q539	ABL2	1.08E-02	0.0000
4	CYTO	W5PIS6	NHLRC3	2.84E-03	0.0000
4	CYTO	W5P6H9	ACP2	3.83E-02	0.0000
4	CYTO	W5PR96	DCK	3.01E-03	0.0004
4	CYTO	W5P3S0		4.23E-05	0.0000
4	CYTO	W5PAJ2	PSAP	7.07E-05	0.0000
4	CYTO	W5QI70	CTSS	4.65E-04	0.0000
4	CYTO	W5Q9L2	LOC101109820	6.22E-03	0.0000
4	CYTO	W5NYK1	PMVK	1.12E-03	0.0000
4	CYTO	W5Q1M0	GLB1	1.54E-04	0.0000
4	CYTO	W5QI36	HEBP1	1.97E-02	0.0000
4	CYTO	W5PRG8	CREG1	4.88E-03	0.0000
4	CYTO	W5NQZ9	GSDMD	1.31E-03	0.0000
4	CYTO	W5Q9M9	GK	2.91E-02	0.0000
4	CYTO	W5QHU8	FNDC3B	5.53E-05	0.0000
4	CYTO	W5P7F8		3.76E-02	0.0000
4	CYTO	W5Q6C5	GGA2	6.95E-03	0.0000
4	CYTO	W5P6L1	RASGRP4	8.55E-04	0.0000
4	CYTO	W5PFB1	TOR1B	3.43E-03	0.0000
4	CYTO	W5Q263	ICAM1	1.37E-02	0.0000
4	CYTO	W5PNP1	MFGE8	2.15E-02	0.0000
4	CYTO	W5PBJ4	ARHGAP10	4.12E-03	0.0000
4	CYTO	W5NUI3	TREM2	3.27E-02	0.0000
4	CYTO	W5QCM1	ARFGAP3	2.08E-02	0.0000
4	CYTO	W5NZV3	HMOX2	4.41E-02	0.0000
4	CYTO	W5Q3V0	IL18	1.05E-05	0.0000
4	CYTO	W5PZD7		2.75E-04	0.0000
4	CYTO	W5Q8V2	LIMA1	1.34E-03	0.0000
4	CYTO	W5P800	GAS7	3.09E-02	0.0000
4	CYTO	W5NZA6	RNF121	2.13E-03	0.0000
4	CYTO	W5NSS6		1.62E-02	0.0000
4	CYTO	W5NWP6	DIAPH2	1.14E-02	0.0000
4	CYTO	W5PF85	PEA15	1.03E-04	0.0000
4	CYTO	W5P4X6	LOC101104287	6.06E-03	0.0000
4	CYTO	W5PSE5	LOC101110434	2.76E-02	0.0002
4	CYTO	W5PLB8	EPB41L3	1.13E-03	0.0000
4	CYTO	W5NTE2	PSMG4	3.44E-02	0.0000
4	CYTO	W5Q6S3	WASHC4	2.74E-03	0.0000
4	CYTO	W5NY99		2.38E-03	0.0000
4	CYTO	W5P895	CRABP2	1.32E-02	0.0000
4	CYTO	W5P4K6	FCHO2	5.47E-04	0.0000
3	CYTO	W5P743	GLMP	2.49E-03	0.0000

Supplementary Table 6: Differentially abundant proteins (DAPs) of MMØ relative to M0 and their cellular location.

FoldChange	Location	Protein IDs	Gene Names	P-value	FDR
3	CYTO	W5PI67	IDS	1.71E-03	0.0000
3	CYTO	W5Q175	GNPTAB	1.44E-03	0.0000
3	CYTO	W5PC32	PGM3	4.51E-03	0.0000
3	CYTO	W5PKF9	FIG4	9.17E-04	0.0000
3	CYTO	W5QHC0	ST3GAL5	5.75E-03	0.0000
3	CYTO	W5PVH4	TMEM251	9.32E-04	0.0000
3	CYTO	W5P703	WFS1	4.08E-03	0.0000
3	CYTO	W5Q5Q7	ASPA	4.27E-02	0.0000
3	CYTO	W5P434	NAGPA	1.84E-03	0.0000
3	CYTO	W5PVM8	UBR7	1.05E-04	0.0000
3	CYTO	W5PCS4		1.31E-04	0.0000
3	CYTO	W5QG92	OSBPL11	2.43E-04	0.0000
3	CYTO	W5PN65	PI4K2A	1.60E-03	0.0000
3	CYTO	W5Q233	VCAN	1.02E-02	0.0000
3	CYTO	W5PQV2	LMBRD1	2.81E-05	0.0000
3	CYTO	W5PUM8	HIP1	2.05E-03	0.0000
3	CYTO	W5Q5K0		3.61E-03	0.0000
3	CYTO	W5QH13	VPS39	7.28E-04	0.0000
3	CYTO	W5NTW0	CADM1	1.31E-02	0.0000
3	CYTO	W5Q4U5	CPT1A	7.71E-03	0.0000
3	CYTO	W5Q2J4	DIP2C	2.74E-04	0.0000
3	CYTO	W5NRL0	PLCD1	6.49E-04	0.0000
3	CYTO	W5PS52	MRC2	5.71E-03	0.0000
3	CYTO	W5PZ94	ACO1	3.42E-02	0.0000
3	CYTO	W5PIE4	CLPTM1	3.56E-02	0.0000
3	CYTO	W5P3C6	LOC101111906	1.02E-04	0.0000
3	CYTO	W5P6V4	GLG1	4.84E-02	0.0000
3	CYTO	W5PQA6	CYFIP2	2.04E-02	0.0000
3	CYTO	W5PFE6	ACOX1	1.76E-03	0.0000
3	CYTO	W5P3I5	CNDP2	1.36E-04	0.0000
3	CYTO	Q10994	CSTB CST6	1.88E-05	0.0000
3	CYTO	W5PGX8	DNAJC13	4.02E-02	0.0000
3	CYTO	W5P093	NQO1	6.25E-03	0.0000
3	CYTO	W5PHY4	TYMS	4.41E-03	0.0000
3	CYTO	W5NPW8	STARD4	4.84E-03	0.0000
3	CYTO	W5PT36	RBM47	2.11E-03	0.0000
3	CYTO	W5PER8	WDR91	1.38E-02	0.0000
3	CYTO	W5P2U3	IVNS1ABP	5.25E-02	0.0000
3	CYTO	W5QBR5	BMP2K	6.44E-03	0.0000
3	CYTO	W5PCD7	ABHD12	3.68E-02	0.0000
3	CYTO	W5PRM9	BCAT2	3.54E-02	0.0000
3	CYTO	W5Q7X8	CRYBG3	3.88E-03	0.0000
3	CYTO	W5PTE6	CEMIP2	4.88E-03	0.0000
3	CYTO	W5NZ70	LGALS3BP	1.52E-02	0.0000
3	CYTO	P82197	PDXK PKH	1.87E-02	0.0000
3	CYTO	W5NUJ2	PEAK1	7.94E-03	0.0000
3	CYTO	W5QH68	LRRC57	3.92E-02	0.0002
3	CYTO	W5NYU6	NT5DC2	4.01E-03	0.0000
3	CYTO	W5QIC7	YBX3	5.75E-03	0.0000
3	CYTO	W5Q0C3	KIF13B	2.97E-02	0.0000
3	CYTO	W5Q728	TPCN2	8.83E-04	0.0000
3	CYTO	W5NU07		1.11E-02	0.0000
3	CYTO	W5P2N4	AHCYL2	1.90E-04	0.0000
3	CYTO	W5Q3Y3	SFXN3	5.22E-03	0.0000
3	CYTO	W5Q3A2		2.85E-02	0.0000
3	CYTO	W5NQ85	IDE	2.06E-02	0.0000
3	CYTO	W5PB38		3.56E-03	0.0000
3	CYTO	W5P536	TRIP10	2.79E-03	0.0000
3	CYTO	W5QBV7	CD44	1.21E-04	0.0000
3	CYTO	W5P2W1	NEU1	3.51E-03	0.0000
3	CYTO	W5QAQ4	NRP2	1.55E-03	0.0000
3	CYTO	W5Q2V0	YKT6	8.34E-04	0.0000
3	CYTO	W5P1Q0	AP1B1	4.33E-05	0.0000

Supplementary Table 6: Differentially abundant proteins (DAPs) of MMØ relative to M0 and their cellular location.

FoldChange	Location	Protein IDs	Gene Names	P-value	FDR
3	CYTO	W5QAT6	MTHFR	2.46E-03	0.0000
3	CYTO	W5Q928	PCYT2	3.83E-03	0.0000
3	CYTO	W5QAA7	MAPK7	6.91E-04	0.0000
3	CYTO	W5NYW8	CEP55	9.74E-03	0.0004
3	CYTO	W5QCG3	CLCC1	3.64E-03	0.0000
3	CYTO	W5NV37	COMMD10	2.05E-02	0.0000
3	CYTO	W5NRA9	ASL	1.61E-02	0.0000
3	CYTO	W5QDN8	SLC48A1	3.35E-03	0.0002
3	CYTO	W5P8A0		3.28E-02	0.0000
3	CYTO	W5P333		1.82E-02	0.0000
3	CYTO	W5Q3B4	TBCD	1.66E-03	0.0000
3	CYTO	W5PN70		1.02E-02	0.0000
3	CYTO	W5QI95	CERS2	1.46E-02	0.0000
3	CYTO	W5PVQ4	TMEM120A	1.47E-04	0.0000
3	CYTO	W5P366	FAM50A	3.55E-02	0.0000
3	CYTO	W5QHR5	PLEKHO2	9.32E-05	0.0000
3	CYTO	W5PVE3	LOC101115252	2.49E-04	0.0000
3	CYTO	W5NRQ3	TRIM68	5.51E-03	0.0015
3	CYTO	W5NWX4		3.25E-02	0.0000
3	CYTO	W5PCM4	LUZP1	4.41E-04	0.0000
3	CYTO	W5PAX1	GCLC	2.65E-02	0.0000
3	CYTO	W5QBJ2	SLC38A10	1.35E-03	0.0000
3	CYTO	W5QCP4	TRAF6	2.28E-03	0.0000
3	CYTO	W5PCC0	LACC1	7.04E-03	0.0000
3	CYTO	W5PDU4	NMT2	6.17E-03	0.0000
3	CYTO	W5PCD0	FUBP3	3.58E-02	0.0000
3	CYTO	W5PBX0	ATP11A	4.64E-02	0.0000
3	CYTO	W5PB61		2.72E-02	0.0002
3	CYTO	W5PH35	LOC101119706	3.30E-03	0.0000
3	CYTO	W5NPU0	NAPRT	1.07E-02	0.0000
3	CYTO	W5P3W6	OSBPL9	5.90E-03	0.0000
3	CYTO	W5PUI3	GOLGA1	3.92E-05	0.0000
3	CYTO	W5PPR1	UCK2	2.71E-03	0.0002
3	CYTO	W5PEX1	WASHC5	1.39E-02	0.0000
3	CYTO	W5Q2K9	CYFIP1	1.08E-03	0.0000
3	CYTO	W5PU61	SETD7	1.90E-02	0.0000
3	CYTO	W5QGT0	ATP13A3	1.78E-02	0.0000
3	CYTO	W5PPK8		8.00E-03	0.0000
3	CYTO	W5P5P8	PSMG3	5.21E-02	0.0000
3	CYTO	W5QET8	TEP1	1.69E-02	0.0000
3	CYTO	W5P1T1	ANO10	6.70E-03	0.0000
3	CYTO	W5QEA3		1.05E-03	0.0000
3	CYTO	W5NRF7	MAP3K20	1.02E-03	0.0000
3	CYTO	W5QFH0	ARSA	1.39E-03	0.0000
3	CYTO	W5PAP3	FCHSD2	4.33E-02	0.0000
3	CYTO	W5PFU8	KAT6B	8.62E-03	0.0006
3	CYTO	W5PUL4	MTMR6	2.36E-02	0.0000
3	CYTO	W5NT95	ATP6AP2	3.50E-05	0.0000
3	CYTO	W5P369	AP2A2	4.54E-06	0.0000
3	CYTO	W5NVV6	DNAJC3	3.70E-02	0.0000
3	CYTO	W5PJN7	HOMER3	4.78E-03	0.0000
3	CYTO	W5PQ75	HSPH1	2.37E-02	0.0000
3	CYTO	W5Q4Q8		4.77E-04	0.0000
3	CYTO	W5Q1W2	SDCBP	4.09E-02	0.0000
3	CYTO	W5PUW2	IFI30	1.85E-03	0.0000
3	CYTO	W5Q5N6	BST-2B	2.34E-02	0.0000
3	CYTO	W5PJ58	HPS5	4.85E-02	0.0000
3	CYTO	W5QIJ6	SPPL2A	3.02E-02	0.0000
3	CYTO	W5P3X8	KIF15	9.19E-03	0.0000
3	CYTO	W5P8R4	CSF1R	7.81E-03	0.0000
3	CYTO	W5PG41	H6PD	2.71E-02	0.0000
3	CYTO	W5Q3C2	EPPK1	9.43E-03	0.0000
3	CYTO	W5P3B0	INPPL1	4.61E-02	0.0000

Supplementary Table 6: Differentially abundant proteins (DAPs) of MMØ relative to M0 and their cellular location.

FoldChange	Location	Protein IDs	Gene Names	P-value	FDR
3	CYTO	W5PWR6	KCNAB2	2.64E-03	0.0000
3	CYTO	W5PEZ1		5.02E-02	0.0000
3	CYTO	W5Q0J1	PLA2G6	6.01E-03	0.0046
3	CYTO	W5Q9E6	TANGO2	1.39E-03	0.0000
3	CYTO	W5NWX7	CLEC4A	1.33E-02	0.0000
3	CYTO	W5Q8I7	LOC101122123	4.39E-03	0.0000
3	CYTO	W5PC06	SIRPA	5.64E-04	0.0000
3	CYTO	W5PI02	TBC1D13	2.12E-02	0.0000
3	CYTO	W5PQM4	MOSPD2	3.98E-04	0.0000
3	CYTO	W5PWA8	HSPB1	4.78E-03	0.0000
3	CYTO	W5PBR7	P4HA1	2.12E-02	0.0000
3	CYTO	W5QEL6	PCYOX1	2.53E-02	0.0000
3	CYTO	W5PAM5		1.76E-03	0.0000
3	CYTO	W5Q831		2.19E-02	0.0000
3	CYTO	W5PVH8	ATP6V1E1	1.37E-03	0.0000
3	CYTO	W5P3H1	LOC101111732	7.78E-03	0.0000
3	CYTO	W5PBB5	CTSF	1.03E-02	0.0000
3	CYTO	W5NUU1	VRK2	9.96E-03	0.0000
3	CYTO	W5PSG0		5.75E-03	0.0000
3	CYTO	W5QDQ8	MMP14	8.31E-03	0.0000
3	CYTO	Q6XXL8	DYNLT3 TCTE1L	7.71E-03	0.0000
3	CYTO	W5QCF3	SLC35F6	8.58E-03	0.0000
3	CYTO	W5P700	KIF1B	1.54E-02	0.0000
3	CYTO	W5NV06	ATP6V0A1	3.64E-02	0.0000
3	CYTO	W5Q7Z6	DIP2B	6.60E-03	0.0000
3	CYTO	W5PCX5	CC2D1A	3.64E-02	0.0002
3	CYTO	W5P1S3	SLC25A13	4.68E-03	0.0000
3	CYTO	W5NYK9	CALU	1.47E-02	0.0000
3	CYTO	W5P5E7		5.40E-03	0.0000
3	CYTO	W5QDC0	SNX17	3.27E-02	0.0000
3	CYTO	W5P9L5	RASA2	2.43E-03	0.0002
3	CYTO	W5PEN2	TPD52	4.53E-03	0.0000
3	CYTO	W5Q644	VPS29	4.59E-02	0.0000
3	CYTO	W5PKV1	DNASE2	1.51E-02	0.0000
3	CYTO	W5QH60	VAMP8	3.22E-02	0.0004
3	CYTO	W5Q8Y5	HDLBP	5.67E-03	0.0000
3	CYTO	Q9XT28	ATOX1	1.32E-03	0.0000
3	CYTO	W5PYH6	AP3D1	1.92E-02	0.0000
3	CYTO	W5P1U3	LACTB2	4.68E-02	0.0000
3	CYTO	W5Q553	ITGAV	6.35E-03	0.0000
3	CYTO	W5QDF4	GSTM3	5.75E-03	0.0000
3	CYTO	W5Q2H2	SLC12A9	8.24E-04	0.0000
3	CYTO	W5Q165	RRM2	1.51E-03	0.0000
2	CYTO	W5QFK2	MACF1	2.93E-02	0.0000
2	CYTO	W5QAL6	FMNL3	9.21E-03	0.0002
2	CYTO	W5Q420		2.66E-02	0.0000
2	CYTO	W5PER3	TM9SF3	2.87E-02	0.0000
2	CYTO	W5PDK4	AIF1	4.47E-02	0.0000
2	CYTO	W5NR48	KPNA6	4.64E-02	0.0000
2	CYTO	W5QCA6	UBE2F	3.16E-04	0.0000
2	CYTO	W5PK12	OAT	7.44E-05	0.0000
2	CYTO	W5PCU1	ASS1	4.04E-03	0.0000
2	CYTO	W5PWB8	ARHGAP12	6.98E-03	0.0000
2	CYTO	W5Q8K4	SLC3A2	5.20E-03	0.0000
2	CYTO	W5PS50	MPC2	2.26E-02	0.0000
2	CYTO	W5PN60	ABR	3.09E-02	0.0000
2	CYTO	W5QB79	GLCE	1.83E-02	0.0002
2	CYTO	W5PQZ7	PATL1	1.72E-02	0.0000
2	CYTO	W5QIU9	S100A10	1.17E-02	0.0000
2	CYTO	W5Q686	TPP1	1.14E-02	0.0000
2	CYTO	P81184	LGALS1	2.17E-04	0.0000
2	CYTO	P04074	ATP1A1	2.02E-03	0.0000
2	CYTO	Q6XUZ5	IDH1	4.98E-05	0.0000

Supplementary Table 6: Differentially abundant proteins (DAPs) of MMØ relative to M0 and their cellular location.

FoldChange	Location	Protein IDs	Gene Names	P-value	FDR
2	CYTO	W5NZ71		5.17E-02	0.0000
2	CYTO	W5PP47		2.26E-04	0.0000
2	CYTO	W5QIX6	EXOC5	3.67E-02	0.0000
2	CYTO	W5PMA0	AP2S1	2.27E-02	0.0000
2	CYTO	W5PZ62	ZFYVE16	5.84E-03	0.0000
2	CYTO	W5Q3L8	LOC101104306	2.34E-03	0.0000
2	CYTO	W5PL89	GSR	2.28E-02	0.0000
2	CYTO	W5P3A2		2.32E-03	0.0000
2	CYTO	W5P7L2	ATP6V1G1	5.71E-04	0.0000
2	CYTO	W5PSP1	ERC1	4.65E-02	0.0002
2	CYTO	W5QHV8	PLD1	3.24E-02	0.0000
2	CYTO	W5P6S8	COBLL1	1.82E-02	0.0000
2	CYTO	W5NT19	SPAG1	5.60E-02	0.0000
2	CYTO	W5PUM5	KANK1	2.99E-03	0.0000
2	CYTO	W5PPR6	EXOC6B	9.64E-03	0.0000
2	CYTO	W5QBW4	VAV3	1.52E-04	0.0000
2	CYTO	W5QIA8	YARS1	1.11E-03	0.0000
2	CYTO	W5PP17	LOC101123010	3.99E-02	0.0002
2	CYTO	W5P4T0	VHL	8.97E-04	0.0002
2	CYTO	W5PVC8	ERGIC3	4.30E-03	0.0000
2	CYTO	W5QAP3	TOM1	2.48E-03	0.0000
2	CYTO	W5NSS0	ARAP3	2.53E-03	0.0000
2	CYTO	W5QAE8	ALDH7A1	2.25E-04	0.0000
2	CYTO	W5PGL9		4.67E-02	0.0000
2	CYTO	W5Q6E0	CUL2	2.56E-02	0.0000
2	CYTO	W5Q4N4	TOR3A	1.29E-02	0.0000
2	CYTO	W5PLZ0	ATP6V1B2	8.69E-04	0.0000
2	CYTO	W5P4B3	NRDC	3.10E-02	0.0000
2	CYTO	W5PNW7	VIM	3.10E-05	0.0000
2	CYTO	W5P7L0	SUN1	8.59E-03	0.0002
2	CYTO	W5NUG3	GNPDA1	4.24E-02	0.0000
2	CYTO	W5P1M3		4.94E-02	0.0000
2	CYTO	W5Q5Z1	YTHDF3	1.66E-02	0.0000
2	CYTO	W5PRS4	FKBP5	2.72E-02	0.0000
2	CYTO	W5Q7V3		6.47E-04	0.0000
2	CYTO	W5QHX1	EIF5A2	1.23E-02	0.0000
2	CYTO	W5QIA5	ETV6	4.40E-02	0.0000
2	CYTO	W5PUJ2	LAMP2	5.82E-03	0.0000
2	CYTO	W5PVT3	GALNS	1.72E-02	0.0000
2	CYTO	W5PXJ8	CMBL	1.10E-03	0.0000
2	CYTO	W5PHS5	RGL2	5.12E-02	0.0000
2	CYTO	W5Q120	ANKRD28	3.07E-02	0.0002
2	CYTO	W5NRI6	PLPBP PROSC	1.68E-02	0.0000
2	CYTO	W5NUE3	PRDX1	8.76E-04	0.0000
2	CYTO	W5P7R2	HECTD1	1.92E-02	0.0000
2	CYTO	W5Q5B5	CSAD	1.72E-03	0.0000
2	CYTO	W5PWF2	ATP6V1H	1.05E-03	0.0000
2	CYTO	W5Q430	IDUA	1.72E-03	0.0002
2	CYTO	W5P5H4	AARS AARS1	3.16E-03	0.0000
2	CYTO	W5NSS1	PPP4R1	2.16E-03	0.0000
2	CYTO	W5Q740	ABCD3	3.15E-02	0.0000
2	CYTO	W5QEU6	ANXA4	3.26E-02	0.0000
2	CYTO	W5PMH1	GSS	6.29E-03	0.0000
2	CYTO	W5PHJ3	AHR	1.98E-02	0.0000
2	CYTO	W5QGX4	P3H1	1.24E-03	0.0000
2	CYTO	W5PEK7	FMNL2	2.02E-02	0.0000
2	CYTO	W5Q8J8	VPS41	2.05E-03	0.0000
2	CYTO	W5P985	ABHD14B	8.17E-03	0.0000
2	CYTO	W5QJ49	ATP6V1D	1.18E-03	0.0000
2	CYTO	W5QDY5	ATP6V1A	1.04E-03	0.0000
2	CYTO	W5PZS4	OSBPL8	3.32E-02	0.0000
2	CYTO	W5QAK7	SCARB1	4.55E-02	0.0000
2	CYTO	W5QIK3	USP8	1.78E-02	0.0000

Supplementary Table 6: Differentially abundant proteins (DAPs) of MMØ relative to M0 and their cellular location.

FoldChange	Location	Protein IDs	Gene Names	P-value	FDR
2	CYTO	W5P5A7	VPS16	1.08E-03	0.0000
2	CYTO	W5QC34	MAN2A1	4.82E-03	0.0000
2	CYTO	W5Q3P7	ITFG1	4.02E-02	0.0004
2	CYTO	W5PK68	FEZ2	3.32E-02	0.0010
2	CYTO	W5Q9H8	SNX5	2.01E-02	0.0000
2	CYTO	W5QDF2	ATG3	2.28E-02	0.0000
2	CYTO	W5P8F1	HEATR5A	2.63E-02	0.0000
2	CYTO	W5Q021	FAM20A	3.85E-02	0.0000
2	CYTO	W5PSD7	RAP2C	1.50E-02	0.0000
2	CYTO	W5PIS1	FAM91A1	8.43E-03	0.0000
2	CYTO	W5PA78	AVL9	3.14E-02	0.0000
2	CYTO	W5P7P6	MYO18A	2.56E-03	0.0000
2	CYTO	W5P316	NAMPT	1.02E-02	0.0000
2	CYTO	W5Q6T1	ARSB	8.89E-03	0.0000
2	CYTO	W5PB21	PLA2R1	3.55E-02	0.0000
2	CYTO	W5NUE1	NCF2	5.74E-03	0.0000
2	CYTO	W5Q8I6	POLDIP2	2.67E-02	0.0000
2	CYTO	W5PE73	SMPDL3A	1.28E-02	0.0000
2	CYTO	W5P3U4	COMMD2	7.65E-03	0.0000
2	CYTO	W5PMB1	SNX3	4.18E-04	0.0000
2	CYTO	W5PGC9	MMAA	5.47E-03	0.0000
2	CYTO	W5PXT9	DENND4C	6.52E-03	0.0000
2	CYTO	W5PTI6	ST3GAL4	2.95E-02	0.0000
2	CYTO	W5P4L4	TMX3	2.04E-02	0.0000
2	CYTO	W5PWZ2		7.01E-03	0.0000
2	CYTO	W5PSX7	FES	1.59E-02	0.0000
2	CYTO	W5Q6V7	SIPA1	1.41E-02	0.0000
2	CYTO	W5P363	VAT1	1.53E-02	0.0000
2	CYTO	W5P0F8	LOC101103804	2.12E-02	0.0002
2	CYTO	W5Q411	HSPA13	1.61E-02	0.0000
2	CYTO	W5P6C1	ADSS ADSS2	1.36E-02	0.0000
2	CYTO	W5NZZ3	ATP6V1F	8.14E-04	0.0000
2	CYTO	W5PRJ7	CCDC93	1.64E-02	0.0000
2	CYTO	W5P8Y0	ITGAL	1.20E-02	0.0000
2	CYTO	W5P3H9	PICALM	1.40E-04	0.0000
2	CYTO	W5QG24	PPT1	2.27E-03	0.0000
2	CYTO	W5PC18		2.06E-02	0.0000
2	CYTO	W5PWV5	WDR81	8.11E-03	0.0000
2	CYTO	W5PSF8	CRYZ	4.91E-02	0.0000
2	CYTO	W5PA83	PRR14	3.70E-03	0.0045
2	CYTO	W5Q0G8	IMPA1	8.63E-03	0.0000
2	CYTO	W5PWQ7	AIDA	5.54E-02	0.0000
2	CYTO	W5Q3C1	NDRG3	2.18E-03	0.0000
2	CYTO	W5NQG1	SETD3	6.85E-03	0.0000
2	CYTO	W5PRK6	STAU1	3.20E-02	0.0000
2	CYTO	W5PW16	NEK6	4.95E-02	0.0000
2	CYTO	W5PVS3	ANKRD17	2.10E-03	0.0000
2	CYTO	W5NSE1	DRG2	9.66E-03	0.0000
2	CYTO	W5PZ05		3.07E-04	0.0000
2	CYTO	W5Q9Z0	ARFIP2	1.14E-02	0.0000
2	CYTO	W5P164	LAMTOR1	3.14E-02	0.0000
2	CYTO	W5PVC1	SYNJ1	5.67E-03	0.0000
2	CYTO	W5Q8T1	CLPX	5.56E-02	0.0000
2	CYTO	W5PG07	TPCN1	2.99E-02	0.0000
2	CYTO	W5PQT3	LOC101105107	2.53E-02	0.0000
2	CYTO	W5P0L9	MAPK3	2.69E-03	0.0000
2	CYTO	W5P7B1	SIRT2	1.38E-02	0.0000
2	CYTO	W5PMY4	GABARAP	3.44E-02	0.0000
2	CYTO	W5PPJ2	VPS33A	7.39E-03	0.0000
2	CYTO	W5PEL8	PDXDC1	3.00E-02	0.0000
2	CYTO	W5QG63	ZMPSTE24	4.66E-02	0.0000
2	CYTO	W5Q540	HAGH	3.42E-02	0.0000
2	CYTO	W5P1K2	TRAPPC9	2.30E-02	0.0000

Supplementary Table 6: Differentially abundant proteins (DAPs) of MMØ relative to M0 and their cellular location.

FoldChange	Location	Protein IDs	Gene Names	P-value	FDR
2	CYTO	W5PDR6	EXOC7	2.11E-02	0.0000
2	CYTO	W5Q501	LOC101115640	6.11E-03	0.0000
2	CYTO	W5P7E8		9.06E-05	0.0002
2	CYTO	W5QI21	TARS2	4.42E-02	0.0000
2	CYTO	W5Q3D8	CLASP2	5.58E-02	0.0000
2	CYTO	W5PSE3	SH3TC1	2.43E-02	0.0002
2	CYTO	W5P180		2.53E-02	0.0000
2	CYTO	W5PVU5	PSMB7	8.26E-03	0.0000
2	CYTO	W5NQ97		2.21E-02	0.0000
2	CYTO	W5PBQ8	ODR4	3.62E-02	0.0000
2	CYTO	W5PDU8	GARS1	6.33E-04	0.0000
2	CYTO	W5Q922	LOC101105383	1.32E-03	0.0000
2	CYTO	W5QHA0	AGFG1	9.04E-04	0.0000
2	CYTO	W5PIC9		4.95E-03	0.0000
2	CYTO	W5Q2L9		4.80E-02	0.0000
2	CYTO	W5QGN5	DLG1	1.44E-04	0.0000
2	CYTO	W5PG60	SLC39A7	3.14E-02	0.0000
2	CYTO	W5P7H5	ADGRE5	4.69E-03	0.0000
2	CYTO	W5PJY0	EMC1	4.13E-02	0.0000
2	CYTO	W5PDN7	GPD2	7.75E-03	0.0000
2	CYTO	W5PQ47	RAI14	2.79E-03	0.0000
2	CYTO	W5QCD2	ADPGK	4.95E-03	0.0000
2	CYTO	W5QIM0	GALK2	3.31E-02	0.0000
2	CYTO	W5Q0N8	ADAM17	6.96E-03	0.0000
2	CYTO	W5PA89	ST6GALNAC2	1.13E-02	0.0021
2	CYTO	W5P001	RUFY1	3.89E-03	0.0000
2	CYTO	C8BKE1	STAT1 STAT4	2.56E-03	0.0000
2	CYTO	W5PYE8	MERTK	1.96E-02	0.0000
2	CYTO	W5QDT8		4.91E-02	0.0000
2	CYTO	W5P4G4	RAB7B	2.53E-03	0.0000
2	CYTO	W5Q7T6	AACS	4.92E-02	0.0000
2	CYTO	W5PSB2	TBL2	1.35E-02	0.0000
2	CYTO	W5QE35	IPO8	5.08E-02	0.0000
2	CYTO	W5Q045	TMED3	1.25E-02	0.0000
2	CYTO	W5PS05	MTHFD2	3.98E-03	0.0000
2	CYTO	W5Q1D8	GALNT7	1.25E-02	0.0002
2	CYTO	Q6ECI6	ITGB2 CD18	4.75E-04	0.0000
2	CYTO	W5NZJ1	LOC101114075	5.15E-02	0.0000
2	CYTO	W5PZM9	ANXA5	3.91E-04	0.0000
2	CYTO	W5PTB7	ATG7	5.23E-05	0.0000
2	CYTO	W5P5S5	EMC3	4.04E-02	0.0000
2	CYTO	W5Q0M7	RAP2B	2.17E-03	0.0000
2	CYTO	W5PDX8	FAM120A	1.18E-02	0.0000
2	CYTO	W5P7N7	IQGAP3	4.53E-02	0.0000
2	CYTO	W5PQ56	RUFY3	4.57E-03	0.0000
2	CYTO	W5QJ45	GPHN	2.82E-02	0.0000
2	CYTO	W5PD54	NCK1	1.09E-02	0.0000
2	CYTO	Q9GMC9	TSPO BZRP	4.01E-02	0.0002
2	CYTO	A2SW69	ANXA2 ANX2	4.69E-03	0.0000
2	CYTO	C5HK62	EYA3	3.16E-02	0.0000
2	CYTO	W5PUU2	RRAS	3.16E-02	0.0000
2	CYTO	W5PVL6	AP2A1	6.93E-03	0.0000
2	CYTO	W5P9Q1	ARHGEF11	1.68E-02	0.0000
2	CYTO	W5Q7I9	HPS6	1.04E-02	0.0000
2	CYTO	W5Q0B4	KYAT3	2.31E-02	0.0002
2	CYTO	W5PR93	SLC46A3	1.70E-02	0.0000
2	CYTO	W5Q289	ATP2B1	6.18E-03	0.0000
2	CYTO	W5QDJ5	LOC101122319	5.32E-02	0.0000
2	CYTO	W5PF94	TOR1A	6.03E-03	0.0002
2	CYTO	W5NTM7	DENND10	1.21E-03	0.0000
2	CYTO	W5PLB6	SPART	7.52E-03	0.0000
2	CYTO	W5PCI1	NLRP3	5.12E-02	0.0000
2	CYTO	Q8HY31	UROD	1.41E-02	0.0000

Supplementary Table 6: Differentially abundant proteins (DAPs) of MMØ relative to M0 and their cellular location.

FoldChange	Location	Protein IDs	Gene Names	P-value	FDR
2	CYTO	W5P2Q3	RPS6KA1	7.58E-03	0.0000
2	CYTO	W5Q4S7	SLC27A1	4.20E-02	0.0000
2	CYTO	W5QGJ2	CHP1	4.11E-02	0.0000
2	CYTO	W5Q0Q2		7.54E-04	0.0000
2	CYTO	W5NSV0	LTBP2	3.79E-02	0.0000
2	CYTO	W5P929	MARS1	7.18E-03	0.0000
2	CYTO	W5PD41	GIT1	5.04E-03	0.0000
2	CYTO	W5NYP8	DYNC1H1	3.41E-05	0.0000
2	CYTO	W5Q738	TMEM173 STING1	1.36E-03	0.0000
2	CYTO	W5P207		1.84E-03	0.0000
2	CYTO	W5PP30	ASNA1 GET3	3.39E-04	0.0000
2	CYTO	O78755	MT-ND4 MTND4 N	3.11E-02	0.0000
2	CYTO	W5PGJ4	CHN2	1.33E-02	0.0004
2	CYTO	W5P9Y1	SNX6	2.72E-02	0.0000
2	CYTO	W5P089	LOC101115969	4.87E-02	0.0000
2	CYTO	W5PVC2	CDC37L1	4.14E-03	0.0000
2	CYTO	W5PL73	CCDC22	1.81E-03	0.0000
2	CYTO	W5QBJ0	ANXA3	3.49E-02	0.0000
2	CYTO	W5Q7J2	AGPS	5.48E-02	0.0000
2	CYTO	W5PXD7	LAMTOR3	1.45E-04	0.0000
2	CYTO	W5PUF8	PITRM1	3.63E-02	0.0000
2	CYTO	W5Q353	TACC3	7.36E-03	0.0000
2	CYTO	W5QHF0	ABCF3	4.04E-02	0.0000
2	CYTO	W5PCA0	ALDOB	4.00E-02	0.0000
2	CYTO	W5QHT6	NCEH1	7.03E-03	0.0000
2	CYTO	W5P2M5	LOC101114535	1.58E-02	0.0025
2	CYTO	W5QFN9	UGP2	2.41E-03	0.0000
2	CYTO	W5PD62	CPB2	1.83E-02	0.0091
-2	CYTO	W5Q1G5		5.39E-02	0.0000
-2	CYTO	W5PVX8	PPP3CA	7.01E-03	0.0000
-2	CYTO	W5PXM3	PSPC1	5.20E-02	0.0000
-2	CYTO	W5Q198	QRICH1	2.97E-02	0.0002
-2	CYTO	W5PX94	CELF2	4.20E-02	0.0000
-2	CYTO	W5NW82	NAP1L4	6.09E-03	0.0000
-2	CYTO	W5PT49	NFKB1	3.80E-02	0.0000
-2	CYTO	W5QD48	TAGLN3	1.22E-02	0.0000
-2	CYTO	W5PAI1		1.01E-02	0.0000
-2	CYTO	W5NSI2	RALBP1	4.99E-02	0.0085
-2	CYTO	W5PJ14	ESYT2	2.54E-02	0.0000
-2	CYTO	W5P098	CORO1A	6.33E-04	0.0000
-2	CYTO	W5P440	HERC4	2.41E-02	0.0000
-2	CYTO	W5PEE3	FNBP1	8.66E-03	0.0000
-2	CYTO	W5PHB6	SF1	4.85E-02	0.0000
-2	CYTO	W5NWQ0	TMED8	4.05E-02	0.0000
-2	CYTO	W5PVI9	STK38	4.65E-02	0.0000
-2	CYTO	W5PUT2	FUBP1	5.34E-02	0.0000
-2	CYTO	W5Q2Y3	PLEKHB2	3.38E-02	0.0000
-2	CYTO	W5PVY6		3.83E-02	0.0000
-2	CYTO	W5Q8F8	CPOX	4.13E-02	0.0000
-2	CYTO	W5PYM3	CAAP1	1.18E-02	0.0012
-2	CYTO	W5P340	SOD2	2.32E-02	0.0000
-2	CYTO	W5QHP6	ARHGDIB	1.86E-05	0.0000
-2	CYTO	W5PK02	POSTN	4.83E-02	0.0000
-2	CYTO	W5QCZ0	MPST	2.49E-04	0.0000
-2	CYTO	W5QC32	CHERP	5.25E-02	0.0000
-2	CYTO	W5PL66	PPA2	1.10E-02	0.0000
-2	CYTO	W5PWH9	PRKACB	9.69E-04	0.0000
-2	CYTO	W5PML4	RCC2	8.13E-03	0.0000
-2	CYTO	W5NX11	EPB41	3.11E-02	0.0000
-2	CYTO	W5PVC3	LOC101117851	1.90E-02	0.0002
-2	CYTO	W5PXS7	DAPP1	9.84E-03	0.0000
-2	CYTO	W5QF71	PLEK	2.96E-02	0.0000
-2	CYTO	W5PIC3		4.02E-03	0.0000

Supplementary Table 6: Differentially abundant proteins (DAPs) of MMØ relative to M0 and their cellular location.

FoldChange	Location	Protein IDs	Gene Names	P-value	FDR
-2	CYTO	Q2TCH3	ACLY	5.78E-04	0.0000
-2	CYTO	W5PR48	HPRT1	2.85E-02	0.0000
-2	CYTO	W5P5N5		1.05E-02	0.0002
-2	CYTO	W5P4E2	PBLD	1.99E-02	0.0000
-2	CYTO	W5PXJ6	ANKRD13A	3.50E-03	0.0000
-2	CYTO	W5QIC3	PRUNE1	3.16E-04	0.0000
-2	CYTO	W5QI77	AK2	4.27E-03	0.0000
-2	CYTO	W5NVL4		2.60E-02	0.0000
-2	CYTO	W5P1E2	CNST	9.04E-04	0.0000
-2	CYTO	W5PHI7	LOC101116892	2.62E-02	0.0000
-2	CYTO	W5Q2W6	PPFIBP2	4.33E-02	0.0002
-2	CYTO	W5PIU9	ARMC6	3.67E-02	0.0000
-2	CYTO	W5PHE6	DDI2	5.05E-03	0.0000
-2	CYTO	W5QHL0	ATP11B	8.93E-03	0.0000
-2	CYTO	W5PDQ3	SAFB2	2.11E-02	0.0000
-2	CYTO	W5NQ72	PFAS	2.79E-03	0.0000
-2	CYTO	W5QFQ8	LOC101111915	1.32E-02	0.0000
-2	CYTO	W5P8J8		5.61E-02	0.0000
-2	CYTO	W5P5M9		6.34E-03	0.0000
-2	CYTO	W5PV48	USP11	2.51E-02	0.0000
-2	CYTO	W5Q0B6	PPP1R12A	2.90E-03	0.0000
-2	CYTO	W5PDB0	TRIM58	2.33E-02	0.0000
-2	CYTO	W5Q595	NIF3L1	3.32E-02	0.0000
-2	CYTO	W5Q3M9	SEPTIN6	1.94E-02	0.0000
-2	CYTO	W5NV08	PUM1	4.94E-02	0.0000
-2	CYTO	W5P4C5	RBM42	1.17E-02	0.0000
-2	CYTO	W5PXG3		1.09E-02	0.0015
-2	CYTO	W5NVG4	SMC4	3.44E-02	0.0000
-2	CYTO	W5P7C2	SEPHS1	3.00E-02	0.0000
-2	CYTO	W5NRJ3		1.95E-02	0.0000
-2	CYTO	W5PRJ3	HYCC1	4.12E-02	0.0000
-2	CYTO	W5PWZ9	RAMAC	5.02E-02	0.0043
-2	CYTO	C8BKD4	CBX5	2.54E-02	0.0000
-2	CYTO	W5Q366	SUN2	1.76E-02	0.0000
-2	CYTO	W5QGB5	LOC101113369	6.36E-03	0.0000
-2	CYTO	W5Q3Q2	ERCC3	1.00E-02	0.0041
-2	CYTO	W5QIH2	TMOD3	3.60E-03	0.0000
-2	CYTO	W5QB24	ANP32A	1.83E-02	0.0000
-2	CYTO	C8BKC5	PRDX2	2.32E-02	0.0000
-2	CYTO	W5P059	TERF2	3.93E-02	0.0002
-2	CYTO	W5NUT8	PIP4K2A	1.53E-02	0.0000
-2	CYTO	W5PMC9	ARHGAP6	1.85E-02	0.0071
-2	CYTO	W5PW66	LANCL2	3.24E-02	0.0000
-2	CYTO	W5PAV5	RFX1	4.60E-02	0.0000
-2	CYTO	W5NS43	ALDH6A1	1.11E-03	0.0000
-2	CYTO	W5PLK6	SHOC2	1.02E-02	0.0002
-2	CYTO	W5PUR1	CNOT11	8.67E-05	0.0000
-2	CYTO	W5P5Y0	AGAP2	4.79E-02	0.0000
-2	CYTO	W5PFJ5	SMC1A	2.61E-02	0.0000
-2	CYTO	W5NRR9	STAT5A	1.60E-02	0.0000
-2	CYTO	W5NQH2	CHMP6	4.65E-02	0.0000
-2	CYTO	W5PVD4	AK3	2.21E-03	0.0000
-2	CYTO	W5PY64	LOC101106288	2.02E-02	0.0000
-2	CYTO	W5PUS0	CUL4B	4.86E-04	0.0000
-2	CYTO	W5PQ04	DEF6	2.13E-02	0.0000
-2	CYTO	W5PTS4	LOC101114275	1.50E-02	0.0000
-2	CYTO	W5QCG0		4.68E-02	0.0000
-2	CYTO	W5PWS1	CD46	2.69E-02	0.0000
-2	CYTO	W5Q5Y1	ITGA4	2.19E-03	0.0002
-2	CYTO	W5P0J5	ZNF22	4.20E-02	0.0002
-2	CYTO	W5Q9R2	SARM1	5.23E-02	0.0000
-2	CYTO	W5P0D9		2.52E-02	0.0002
-2	CYTO	W5QIK2	RFX5	1.95E-02	0.0000

Supplementary Table 6: Differentially abundant proteins (DAPs) of MMØ relative to M0 and their cellular location.

FoldChange	Location	Protein IDs	Gene Names	P-value	FDR
-2	CYTO	W5PLD5	CNN2	2.90E-03	0.0000
-2	CYTO	W5PLX5	PRKD2	1.24E-02	0.0000
-2	CYTO	W5Q1F6	AIFM1	4.92E-02	0.0000
-2	CYTO	W5P225	PDCD5	3.25E-02	0.0000
-2	CYTO	W5QJ62	ACTN1	1.96E-02	0.0000
-2	CYTO	W5Q1B3	HMGB2	6.49E-03	0.0000
-2	CYTO	W5QHY6	RPIA	2.55E-03	0.0000
-2	CYTO	W5QFT3		4.26E-02	0.0000
-2	CYTO	W5PUQ5	CLASP1	5.61E-02	0.0000
-2	CYTO	W5PRP1	SLC9A3R1	3.06E-04	0.0000
-2	CYTO	W5Q7Q6	EPHX1	5.59E-02	0.0000
-2	CYTO	W5QIY4	ARID4A	7.55E-03	0.0000
-2	CYTO	W5NTA4	CGGBP1	2.52E-02	0.0000
-2	CYTO	W5PIG5	ABCC1	2.86E-02	0.0000
-2	CYTO	W5Q9U3	CNP	9.94E-04	0.0000
-2	CYTO	W5QEH9	THRAP3	2.95E-02	0.0000
-2	CYTO	W5PAF9	KIF2A	2.49E-02	0.0000
-2	CYTO	W5P9F0	ARHGAP9	2.84E-02	0.0000
-2	CYTO	W5P2J9		1.60E-02	0.0000
-2	CYTO	W5QBV1	FER	1.01E-02	0.0000
-2	CYTO	W5P433	BRCA1	1.81E-02	0.0037
-2	CYTO	W5PEG1	CETN2	5.40E-02	0.0000
-2	CYTO	W5Q088	CBL	2.62E-03	0.0000
-2	CYTO	W5PCG2	TAP2	2.68E-02	0.0000
-2	CYTO	B2LU20	SFXN1	5.52E-02	0.0000
-2	CYTO	W5PNJ5	SARNP	2.68E-03	0.0000
-2	CYTO	W5PCD9	EML4	4.54E-03	0.0000
-2	CYTO	W5Q2C6		5.06E-02	0.0002
-3	CYTO	W5PSX3	DARS2	6.21E-03	0.0002
-3	CYTO	W5QCS1	DDX23	1.68E-02	0.0000
-3	CYTO	W5PM99		1.00E-02	0.0000
-3	CYTO	W5QEP1	POGLUT1	3.51E-02	0.0000
-3	CYTO	W5QHX6	ANP32E	3.27E-02	0.0000
-3	CYTO	W5PTQ0	RNGTT	4.58E-02	0.0002
-3	CYTO	W5PW39	LMNB2	2.05E-02	0.0000
-3	CYTO	W5P0E3	BRD4	5.31E-02	0.0000
-3	CYTO	W5QEQ3	APEX1	4.47E-02	0.0000
-3	CYTO	W5Q297	PRKCA	1.12E-02	0.0000
-3	CYTO	W5QB58	AASS	3.37E-04	0.0000
-3	CYTO	W5PNV3	AK1	2.37E-02	0.0000
-3	CYTO	W5Q824	GSTA1	4.85E-02	0.0000
-3	CYTO	W5PI11	TAPBP	4.25E-02	0.0000
-3	CYTO	W5NUX2		1.20E-02	0.0000
-3	CYTO	W5PJ11		1.58E-02	0.0002
-3	CYTO	W5PSQ7		5.14E-03	0.0000
-3	CYTO	W5Q8P3	USE1	3.58E-03	0.0000
-3	CYTO	W5P4F9	PRKAR2B	2.85E-02	0.0000
-3	CYTO	W5PTM9	UBE2V2	2.19E-04	0.0000
-3	CYTO	W5P7A6	ATE1	8.23E-03	0.0000
-3	CYTO	W5PRR5	LTA4H	1.79E-04	0.0000
-3	CYTO	W5Q4G2		7.70E-03	0.0000
-3	CYTO	W5P671	DDAH2	1.34E-02	0.0095
-3	CYTO	W5QHL6	ITM2C	1.14E-03	0.0000
-3	CYTO	W5NXE7	PPIL4	4.52E-02	0.0000
-3	CYTO	W5Q3E4	NAAA	3.70E-02	0.0000
-3	CYTO	W5PP80	FRY	3.05E-02	0.0012
-3	CYTO	W5PTM6	CPSF2	1.01E-02	0.0000
-3	CYTO	W5PMQ1	ZC3H4	3.93E-02	0.0000
-3	CYTO	W5PWV6	UBE2B	4.17E-02	0.0000
-3	CYTO	W5QET9	PLA1A	3.76E-03	0.0000
-3	CYTO	O78750	MT-CO2 COII COX2	1.19E-02	0.0000
-3	CYTO	W5QD23	LANCL1	1.33E-02	0.0000
-3	CYTO	W5PKD2	GCDH	3.38E-02	0.0000

Supplementary Table 6: Differentially abundant proteins (DAPs) of MMØ relative to M0 and their cellular location.

FoldChange	Location	Protein IDs	Gene Names	P-value	FDR
-3	CYTO	W5PKY9	MRCL3	4.78E-02	0.0000
-3	CYTO	C5IWU4	ARL3	3.18E-02	0.0000
-3	CYTO	W5PEQ9	SRSF1	3.26E-02	0.0000
-3	CYTO	W5PK38	VASP	2.32E-02	0.0000
-3	CYTO	W5QI35	IL1RN	4.90E-02	0.0000
-3	CYTO	W5PJF3	PPIF	3.29E-02	0.0002
-3	CYTO	W5P6V7	DDX39A	2.47E-03	0.0000
-3	CYTO	W5PF18	LOC101102454	5.04E-02	0.0000
-3	CYTO	W5NRI1		1.83E-03	0.0002
-3	CYTO	W5Q9B6	INTS14	4.93E-02	0.0010
-3	CYTO	W5PEA0	FGD3	1.25E-04	0.0000
-3	CYTO	W5P8I8	PITPNM2	2.20E-03	0.0000
-3	CYTO	W5Q0P0	GABPA	2.73E-02	0.0000
-3	CYTO	W5PI65	MAP4K2	1.38E-04	0.0000
-3	CYTO	W5PQT2	TPRKB	3.47E-02	0.0000
-3	CYTO	W5PBZ1	PCIF1	2.68E-02	0.0000
-3	CYTO	W5PAV0	STAP1	2.65E-02	0.0000
-3	CYTO	W5P0N1	DHX38	3.78E-03	0.0010
-3	CYTO	W5PIW6	PRTN3	4.61E-02	0.0000
-3	CYTO	W5PUI4	RAB3A	1.84E-03	0.0002
-3	CYTO	W5NTV6		1.86E-03	0.0000
-3	CYTO	W5PTN4	TMPO	4.22E-03	0.0000
-3	CYTO	W5Q7L1	CNN3	1.18E-02	0.0000
-3	CYTO	W5NTW9	RIPK1	2.18E-02	0.0000
-3	CYTO	W5PCJ3	LOC101112936	1.23E-02	0.0000
-3	CYTO	W5PZL2		3.97E-02	0.0000
-3	CYTO	W5PKS6	DEK	4.23E-02	0.0000
-3	CYTO	W5PAF7	RSL1D1	3.86E-02	0.0000
-3	CYTO	W5P7K2		4.22E-02	0.0000
-3	CYTO	W5Q8H0	SSH1	2.87E-02	0.0000
-3	CYTO	W5P8N5	FBL	4.32E-02	0.0000
-3	CYTO	C5ISA2	TUBA4A	1.43E-02	0.0000
-3	CYTO	W5PDE5	LOC101120001	5.15E-03	0.0000
-3	CYTO	W5P0R4	CTIF	7.24E-05	0.0004
-3	CYTO	W5PA22	ACOT8	2.03E-03	0.0000
-3	CYTO	W5PVX3	PRKG1	4.53E-02	0.0000
-3	CYTO	W5QEC3	METTL3	1.85E-02	0.0000
-3	CYTO	W5PHI5	PALM	6.56E-03	0.0000
-3	CYTO	W5PV67	LIMD2	1.73E-02	0.0000
-3	CYTO	W5NRP8	MTCL1	9.37E-04	0.0008
-3	CYTO	W5Q2W4	RAVER1	4.19E-02	0.0000
-3	CYTO	W5PZU8	ASAP2	2.06E-02	0.0000
-3	CYTO	W5QGS4	PHGDH	2.20E-02	0.0000
-3	CYTO	W5PI68	TBCK	3.58E-02	0.0000
-3	CYTO	W5NQH6	S100A9	4.06E-02	0.0000
-3	CYTO	W5P7P8	GCA	5.91E-03	0.0000
-3	CYTO	W5PQD8	C1QTNF3	1.92E-03	0.0000
-3	CYTO	W5PP85	TBC1D10C	1.57E-02	0.0000
-3	CYTO	W5PTB3	INPP5K	1.11E-03	0.0002
-3	CYTO	W5P7E0	SARS2	3.56E-02	0.0000
-3	CYTO	W5NTQ3	H3-4	7.53E-05	0.0002
-3	CYTO	W5Q7P6	SMARCA4	4.62E-02	0.0000
-3	CYTO	W5P5K9	SRGN	1.84E-02	0.0002
-3	CYTO	W5PKK4	CCAR2	4.83E-02	0.0000
-3	CYTO	W5P795	LRBA	1.14E-02	0.0000
-3	CYTO	W5PG95	HSPA1A	5.03E-02	0.0000
-3	CYTO	W5P6Q7	MPIG6B	4.02E-02	0.0000
-3	CYTO	W5QIR2	C7H15orf48	4.26E-02	0.0000
-3	CYTO	W5PXR8	POLR2J	1.11E-02	0.0000
-3	CYTO	W5P7B8	MTMR3	1.57E-03	0.0000
-4	CYTO	W5PKA9	F5	5.50E-03	0.0000
-4	CYTO	W5PMJ7	NADK2	6.89E-03	0.0000
-4	CYTO	W5P8H4	DCTPP1	7.03E-04	0.0000

Supplementary Table 6: Differentially abundant proteins (DAPs) of MMØ relative to M0 and their cellular location.

FoldChange	Location	Protein IDs	Gene Names	P-value	FDR
-4	CYTO	W5PDG9	LOC101109746	4.05E-02	0.0000
-4	CYTO	W5NWU0	LOC101122262	1.62E-02	0.0000
-4	CYTO	W5PG02	RASA3	6.30E-03	0.0000
-4	CYTO	W5PJP5	RGS18	4.49E-02	0.0000
-4	CYTO	W5P3V8		2.47E-02	0.0000
-4	CYTO	W5QDM7	ZNF512	3.76E-02	0.0000
-4	CYTO	W5Q1Z2		1.35E-02	0.0006
-4	CYTO	W5Q9H1	ZYX	2.57E-02	0.0000
-4	CYTO	W5QFM2		9.24E-05	0.0000
-4	CYTO	W5P9M9	LOC101103862	1.96E-02	0.0000
-4	CYTO	W5P6T0		2.23E-03	0.0012
-4	CYTO	P80190		4.36E-03	0.0000
-4	CYTO	W5P432	NIPSNAP1	5.99E-03	0.0000
-4	CYTO	W5Q5M7	SLC44A2	6.54E-03	0.0000
-4	CYTO	W5NQJ0	LOC101103771	2.42E-02	0.0000
-4	CYTO	W5PGU9	PLCG2	1.54E-04	0.0000
-4	CYTO	W5Q6S0	LBR	3.64E-03	0.0000
-4	CYTO	W5PBE0	CD74	3.70E-02	0.0000
-4	CYTO	W5QFP0	THBS1	2.48E-02	0.0000
-4	CYTO	W5QEK8	EHD3	6.91E-03	0.0000
-4	CYTO	W5PXT1	MYO1G	7.06E-03	0.0000
-4	CYTO	W5PX46	LOC101122591	1.72E-02	0.0000
-4	CYTO	W5Q550	LOC101109747	4.39E-02	0.0000
-4	CYTO	W5NV79	NUP210	1.87E-02	0.0000
-4	CYTO	W5Q2G2	IL16	4.83E-03	0.0000
-4	CYTO	W5NVA8	GP6	1.47E-02	0.0000
-4	CYTO	W5Q059	SASH3	6.30E-03	0.0000
-4	CYTO	W5PEC3	ANXA6	3.86E-02	0.0000
-4	CYTO	W5Q6F1		2.74E-02	0.0000
-4	CYTO	W5PKR2	MX1	1.09E-02	0.0000
-4	CYTO	W5QCH5	WDR47	8.38E-05	0.0002
-4	CYTO	W5PKQ2	PRXL2A	4.60E-02	0.0000
-4	CYTO	W5PC20	VPS37B	7.32E-03	0.0000
-4	CYTO	Q09YJ2	TES	3.08E-03	0.0000
-4	CYTO	W5QI61	CDKN1B	5.53E-04	0.0000
-4	CYTO	C0IZ95	RAB27A	4.23E-03	0.0000
-4	CYTO	W5P5Y4	RIPOR2	5.65E-03	0.0000
-4	CYTO	W5Q1G8	LOC101120875	2.73E-02	0.0000
-4	CYTO	W5P4U4	GRK6	4.39E-04	0.0000
-4	CYTO	W5Q2V1		2.47E-03	0.0000
-4	CYTO	W5PRY2	ACAP1	3.08E-02	0.0000
-4	CYTO	W5PA61		9.05E-03	0.0000
-4	CYTO	W5QEL7	NDRG2	3.28E-04	0.0078
-4	CYTO	W5P815	SEPTIN1	2.24E-02	0.0000
-4	CYTO	W5Q813	NOP58	5.45E-02	0.0000
-4	CYTO	W5NTL4	EVL	2.03E-02	0.0000
-5	CYTO	W5NYC7	DQA	8.59E-03	0.0000
-5	CYTO	P54230	CATHL1A BAC1A D	3.90E-02	0.0000
-5	CYTO	W5QHW2	DAPK2	3.28E-02	0.0000
-5	CYTO	W5QH43	EIF4A2	1.38E-02	0.0000
-5	CYTO	W5NR20	LOC101109940	2.70E-02	0.0099
-5	CYTO	W5PHM1	ALOX12	4.20E-02	0.0000
-5	CYTO	W5PFJ0	VCL	1.69E-02	0.0000
-5	CYTO	W5PU80	MS4A1	6.78E-03	0.0000
-5	CYTO	W5QGQ3	LOC443320	3.78E-02	0.0002
-5	CYTO	W5NSC5		2.33E-03	0.0000
-5	CYTO	W5PDP2	CD55	1.03E-03	0.0000
-5	CYTO	W5QFP2	LOC101109397	7.34E-03	0.0000
-5	CYTO	W5P733	PDLIM1	5.44E-02	0.0000
-5	CYTO	W5PIN7		8.33E-03	0.0000
-5	CYTO	W5NUN1	STX11	3.18E-02	0.0000
-5	CYTO	W5P3Y9	CD22	5.83E-03	0.0000
-5	CYTO	W5PTQ7	TRAF3IP3	1.73E-03	0.0000

Supplementary Table 6: Differentially abundant proteins (DAPs) of MMØ relative to M0 and their cellular location.

FoldChange	Location	Protein IDs	Gene Names	P-value	FDR
-5	CYTO	P79362	CATHL2 BAC5	2.89E-02	0.0000
-5	CYTO	W5PV80	BANK1	1.90E-02	0.0000
-5	CYTO	W5PHR3	ADD3	2.65E-03	0.0000
-5	CYTO	W5PZG0	ADD1	2.45E-03	0.0000
-5	CYTO	W5P7W5	LOC101113516	1.23E-02	0.0000
-5	CYTO	W5PBW1	SDS	2.73E-03	0.0000
-5	CYTO	P47843	SLC2A3 GLUT3	1.15E-02	0.0000
-6	CYTO	W5P060	SYNE1	2.70E-02	0.0000
-6	CYTO	W5PD82	CALD1	5.26E-02	0.0000
-6	CYTO	W5NUE6	PLEKHA2	1.40E-03	0.0000
-6	CYTO	W5PFI6	RASGRP2	3.28E-03	0.0000
-6	CYTO	W5PFI7	VCL	2.68E-02	0.0000
-6	CYTO	W5PP64	FHL1	1.52E-02	0.0000
-6	CYTO	W5Q9B1	ATP2A3	1.77E-03	0.0000
-6	CYTO	W5PM33	SELP	3.11E-02	0.0000
-6	CYTO	W5PRW4	TRIAP1	2.82E-06	0.0002
-6	CYTO	W5P0K2	HEATR1	3.10E-04	0.0000
-6	CYTO	W5PL70	PDCD4	2.92E-03	0.0000
-6	CYTO	W5PYD8	PARP4	3.65E-04	0.0092
-6	CYTO	W5Q3J8		6.74E-04	0.0000
-6	CYTO	W5P4L3	AVIL	2.52E-02	0.0000
-6	CYTO	W5PE90	ITPR1	4.88E-04	0.0000
-6	CYTO	W5Q3B8		1.05E-02	0.0000
-6	CYTO	W5Q9S4	ING3	3.20E-03	0.0012
-6	CYTO	W5NU34	TREML1	2.44E-02	0.0000
-7	CYTO	W5PXV3	CCN2	1.33E-02	0.0000
-7	CYTO	W5PF87	ALOX15	6.10E-03	0.0000
-7	CYTO	W5NQ46	FGB	5.19E-02	0.0000
-7	CYTO	W5Q0K9	ABLIM1	2.47E-03	0.0000
-7	CYTO	W5PQI3	ITGB3	8.56E-03	0.0000
-7	CYTO	W5PJ75	SPTAN1	1.34E-02	0.0000
-7	CYTO	W5NZX9	SPTBN1	1.35E-03	0.0000
-7	CYTO	W5Q3I7	TUBB1	1.08E-02	0.0000
-8	CYTO	W5NXW9		2.30E-04	0.0000
-8	CYTO	W5NT35	LOC443162	7.05E-03	0.0000
-8	CYTO	W5Q5H8	FGA	3.20E-02	0.0000
-8	CYTO	W5QIK8	SELENBP1	4.33E-03	0.0000
-8	CYTO	W5PHQ0	MPO	2.67E-02	0.0000
-8	CYTO	W5Q2S8	MYL9	2.39E-03	0.0000
-9	CYTO	W5Q878	PRKCB	1.32E-03	0.0000
-9	CYTO	W5PEX2	GPATCH8	7.33E-03	0.0000
9	SEC	P83205	CTSB	4.58E-03	0.0000
8	SEC	W5PF33	GM2A	4.09E-04	0.0000
7	SEC	Q9MZS8	CTSD	8.61E-03	0.0000
7	SEC	W5PAJ2	PSAP	1.07E-03	0.0000
7	SEC	W5Q0F3	TGFBI	2.78E-04	0.0000
7	SEC	W5PE92	GRN	4.86E-04	0.0000
7	SEC	W5PBM9	SCPEP1	5.23E-04	0.0000
7	SEC	W5QHV3	FABP1	1.94E-03	0.0000
7	SEC	W5P1H0	CTSC	1.33E-02	0.0000
7	SEC	W5NSH8	NPC2	3.38E-03	0.0000
7	SEC	W5PDH4	MMP9	6.14E-04	0.0000
6	SEC	W5QI78	CTSK	4.16E-04	0.0000
6	SEC	W5PUH5	LGMN	2.31E-04	0.0000
6	SEC	W5NWF5	RARRES1	2.67E-03	0.0000
6	SEC	Q29524	LPL	5.01E-03	0.0000
6	SEC	W5QI70	CTSS	5.17E-03	0.0000
6	SEC	W5PBS4	LRP1	5.86E-03	0.0000
6	SEC	W5PCH3	SCIN	2.29E-03	0.0000
6	SEC	W5NX56	SPP1	5.26E-06	0.0000
6	SEC	W5NTD9	CHI3L1	1.39E-04	0.0000
6	SEC	W5Q8M1	PRRC1	3.06E-02	0.0016
6	SEC	W5Q9H0	MMP2	5.20E-05	0.0000

Supplementary Table 6: Differentially abundant proteins (DAPs) of MMØ relative to M0 and their cellular location.

FoldChange	Location	Protein IDs	Gene Names	P-value	FDR
6	SEC	W5QIW1	LGALS3	2.63E-03	0.0000
6	SEC	W5Q2U7	PLEC	2.18E-02	0.0000
6	SEC	W5PGS4	FABP5	1.79E-02	0.0000
6	SEC	W5Q754	TTN	4.70E-02	0.0024
5	SEC	W5PJS4	EMILIN2	8.07E-04	0.0000
5	SEC	W5P2V3	PEPD	9.40E-03	0.0000
5	SEC	W5QGG0	TFRC	4.25E-03	0.0000
5	SEC	W5P026	STAB1	1.63E-03	0.0000
5	SEC	W5NUI3	TREM2	2.17E-03	0.0000
5	SEC	W5Q0F1	LIPA	9.15E-03	0.0000
5	SEC	W5PS94	NUCB1	6.58E-03	0.0000
5	SEC	W5PT76	GPNMB	8.19E-04	0.0000
5	SEC	W5QC89	HEXA	2.14E-02	0.0000
5	SEC	W5PIQ4	LOC101120093	5.16E-05	0.0000
5	SEC	W5PAM4	CTSA	6.53E-03	0.0000
5	SEC	W5QHL1	FCGR1A	4.35E-03	0.0000
5	SEC	W5NT95	ATP6AP2	9.81E-04	0.0000
5	SEC	W5QH35	CAPG	4.71E-02	0.0000
5	SEC	W5NSK4		2.98E-03	0.0000
5	SEC	W5P3N6	LOC101112162	4.78E-02	0.0000
4	SEC	W5NU86	GLA	4.03E-04	0.0000
4	SEC	Q6ECI6	ITGB2 CD18	3.00E-02	0.0000
4	SEC	W5NTG6	TINAGL1	7.56E-04	0.0000
4	SEC	W5QAQ4	NRP2	5.41E-04	0.0000
4	SEC	W5NY01		4.48E-02	0.0000
4	SEC	W5QJA2	CD14	3.71E-04	0.0000
4	SEC	W5P8R4	CSF1R	1.04E-02	0.0000
4	SEC	W5PPC2		4.69E-02	0.0000
4	SEC	W5NPM4	TEX15	6.42E-03	0.0039
4	SEC	W5PIC9		6.23E-04	0.0000
4	SEC	W5NTZ3	RENBP	3.05E-02	0.0000
4	SEC	W5PGV0	ITGAM	6.34E-03	0.0000
4	SEC	W5Q689	TPP1	1.31E-03	0.0000
4	SEC	W5Q8Y5	HDLBP	2.19E-02	0.0000
4	SEC	W5Q3T9	PDCD6IP	1.85E-02	0.0000
4	SEC	W5Q3U3	LOC101102156	1.52E-02	0.0000
4	SEC	W5P9G8	PLD3	3.10E-02	0.0000
4	SEC	W5PTS4	LOC101114275	4.41E-03	0.0000
4	SEC	W5PEY4	TCN2	1.39E-02	0.0000
4	SEC	W5QBZ7	NAGA	2.40E-02	0.0000
4	SEC	W5PZB0	APLP2	3.33E-03	0.0000
4	SEC	W5PCE0	PLBD2	4.71E-02	0.0000
3	SEC	W5PKY1	HNMT	6.10E-03	0.0000
3	SEC	W5QFU8	CD86	5.56E-02	0.0000
3	SEC	W5P5W6	NDRG1	5.07E-02	0.0000
3	SEC	W5PXR1	ENPP1	4.14E-03	0.0000
3	SEC	W5PKU3		7.71E-03	0.0000
3	SEC	W5Q1W2	SDCBP	5.40E-02	0.0000
3	SEC	W5NUI6	SGSH	2.70E-02	0.0000
3	SEC	W5Q3K6		6.09E-03	0.0000
3	SEC	W5Q799	GALNT6	7.65E-03	0.0099
3	SEC	W5NZ76	TIMP2	2.08E-02	0.0000
3	SEC	W5NPP2	CPM	2.38E-03	0.0000
3	SEC	W5P4A8	RNASET2	3.36E-03	0.0000
3	SEC	W5P3C6	LOC101111906	1.66E-02	0.0000
3	SEC	W5P887	CST3	3.59E-02	0.0000
3	SEC	W5PGC5	GALM	2.29E-02	0.0000
3	SEC	W5PB21	PLA2R1	4.11E-02	0.0000
3	SEC	W5PCC7	LOC101115509	1.08E-02	0.0000
3	SEC	W5PUJ5	LAMP2	1.23E-02	0.0000
3	SEC	W5NRJ3		4.71E-02	0.0039
3	SEC	W5PYS8		3.04E-02	0.0000
3	SEC	W5P3Q3	LOC100101238	2.13E-02	0.0000

Supplementary Table 6: Differentially abundant proteins (DAPs) of MMØ relative to M0 and their cellular location.

FoldChange	Location	Protein IDs	Gene Names	P-value	FDR
3	SEC	W5QI36	HEBP1	2.57E-02	0.0000
2	SEC	Q10994	CSTB CST6	4.96E-02	0.0000
2	SEC	W5PF73	PLAU	3.50E-02	0.0000
2	SEC	W5PCS7	CXCL16	2.39E-02	0.0045
2	SEC	W5NVW7	NAGLU	5.25E-02	0.0000
2	SEC	W5Q1M0	GLB1	3.75E-02	0.0000
2	SEC	W5P2K9	LMAN2	3.87E-02	0.0000
2	SEC	W5PE53	C8B	9.07E-03	0.0000
2	SEC	W5P1W2	FOLR3	4.82E-02	0.0093
2	SEC	W5PZI1	LOC101113728	1.04E-03	0.0000
2	SEC	W5QC34	MAN2A1	5.27E-03	0.0000
2	SEC	W5NTT7	COL1A2	3.21E-02	0.0000
-2	SEC	P60713	ACTB	2.79E-02	0.0000
-2	SEC	W5PVE5	PIN1	3.67E-03	0.0000
-2	SEC	W5PSA3	TCN1	7.34E-03	0.0000
-2	SEC	W5QHL5	CAB39	3.07E-02	0.0000
-2	SEC	W5QFH1	ACTC1	3.52E-02	0.0000
-2	SEC	W5Q0B6	PPP1R12A	1.91E-02	0.0024
-2	SEC	Q09YJ2	TES	3.68E-03	0.0000
-2	SEC	W5Q1I8	PPM1F	1.71E-02	0.0000
-2	SEC	W5PR23	CSE1L	3.84E-02	0.0043
-2	SEC	W5Q3E3	RAB2A	1.67E-02	0.0000
-2	SEC	W5PZ86	SEPTIN8	5.27E-02	0.0000
-2	SEC	W5NVQ4	CAND1	7.52E-03	0.0000
-2	SEC	W5Q7E2	GPS1	2.44E-02	0.0000
-2	SEC	W5P375	TCP1	1.78E-02	0.0000
-2	SEC	W5PFV5	NPEPPS	2.32E-02	0.0000
-2	SEC	W5QGD1	LDHB	3.34E-02	0.0000
-2	SEC	W5PI22	CNPY2	3.52E-02	0.0000
-2	SEC	W5NSM1	ACTR1A	1.70E-02	0.0000
-2	SEC	W5Q9X5	MAP2K1	9.37E-03	0.0000
-3	SEC	W5NX31	AHSA1	2.00E-02	0.0000
-3	SEC	W5QCL5	CSNK2A1	4.18E-02	0.0000
-3	SEC	W5P2J9		2.45E-02	0.0000
-3	SEC	W5PTM9	UBE2V2	3.39E-02	0.0000
-3	SEC	W5PQ76	SRI	5.90E-03	0.0000
-3	SEC	W5P880	PRG4	7.59E-03	0.0000
-3	SEC	W5Q121		1.27E-02	0.0000
-3	SEC	P09670	SOD1	2.63E-03	0.0056
-3	SEC	W5PSZ3	JARID2	4.96E-02	0.0046
-3	SEC	W5P6X5	STMN1	2.96E-02	0.0000
-3	SEC	W5P583	ACP1	5.20E-02	0.0000
-3	SEC	W5PS88	GOT1	2.22E-02	0.0000
-3	SEC	W5PLS7	GRB2	1.30E-02	0.0000
-3	SEC	W5PQA8	PRDX6	2.51E-02	0.0000
-3	SEC	W5PMP1	COTL1	2.93E-02	0.0000
-3	SEC	W5Q7C8	BIN2	3.47E-02	0.0000
-3	SEC	W5P8K0	DCTN2	2.87E-02	0.0000
-3	SEC	W5Q3M9	SEPTIN6	4.09E-02	0.0000
-3	SEC	W5P359	STIP1	8.09E-03	0.0000
-3	SEC	W5NSV5	ITGA6	2.06E-02	0.0000
-3	SEC	W5NSD5	RAP1B	8.97E-03	0.0000
-3	SEC	W5PWI4	RGN	2.87E-02	0.0000
-3	SEC	W5QG77	CD58	1.80E-02	0.0000
-3	SEC	W5PRJ4	VCP	3.43E-02	0.0000
-3	SEC	W5P889	SEPTIN9	6.08E-03	0.0000
-3	SEC	W5P5A0	FLNA	2.79E-02	0.0000
-3	SEC	W5PDL1	WDR1	1.66E-02	0.0000
-3	SEC	W5PNZ5	FKBP4	4.11E-02	0.0000
-3	SEC	W5Q6U0	FASN	1.10E-02	0.0000
-4	SEC	W5PK66	PARK7	4.21E-02	0.0000
-4	SEC	W5P5C4	NARS1	1.69E-02	0.0000
-4	SEC	W5P538	CD93	7.27E-04	0.0000

Supplementary Table 6: Differentially abundant proteins (DAPs) of MMØ relative to M0 and their cellular location.

FoldChange	Location	Protein IDs	Gene Names	P-value	FDR
-4	SEC	Q1ZZU7	MIF	4.19E-02	0.0000
-4	SEC	W5NW17	STXBP2	2.05E-02	0.0000
-4	SEC	W5P7Z1		3.26E-02	0.0000
-4	SEC	W5NVC9	RAC1	2.46E-02	0.0000
-4	SEC	W5PQI3	ITGB3	2.65E-02	0.0000
-4	SEC	W5PE01	TAGLN2	7.65E-03	0.0000
-4	SEC	W5QFQ8	LOC101111915	3.55E-02	0.0000
-4	SEC	W5P4F9	PRKAR2B	5.22E-03	0.0056
-4	SEC	W5QI99	NEDD4	1.69E-03	0.0000
-4	SEC	W5QBQ9	MYH9	1.15E-02	0.0000
-4	SEC	W5PYM5	CCT8	3.69E-02	0.0000
-4	SEC	W5PHU7	UNC13D	6.54E-03	0.0000
-4	SEC	Q2TCH3	ACLY	5.55E-02	0.0000
-4	SEC	W5Q5A6	FGG	3.29E-02	0.0000
-4	SEC	W5QGT4		3.43E-02	0.0000
-4	SEC	W5NU34	TREML1	5.05E-02	0.0000
-4	SEC	W5NW82	NAP1L4	8.92E-04	0.0000
-4	SEC	W5P0V5	RAB11B	3.36E-04	0.0000
-4	SEC	C5ISA2	TUBA4A	9.24E-03	0.0000
-4	SEC	Q5MIB6	PYGB	1.53E-02	0.0000
-4	SEC	W5Q9T3	LMNB1	3.68E-02	0.0000
-4	SEC	W5P8B4	TRAPPC8	2.34E-02	0.0055
-4	SEC	W5PVT6	UBA1	2.35E-02	0.0000
-4	SEC	W5P7E2	PPA1	1.00E-02	0.0000
-4	SEC	W5Q6H1	PLS3	2.31E-03	0.0000
-4	SEC	W5P765	CCT3	4.89E-02	0.0000
-4	SEC	W5PMQ9	SAE1	2.84E-05	0.0000
-4	SEC	W5PQF0	CCT7	5.39E-02	0.0000
-4	SEC	W5PWG1	PFKP	7.52E-03	0.0000
-4	SEC	W5QHS2	MGP	2.70E-04	0.0000
-4	SEC	W5P409	FERMT3	1.85E-02	0.0000
-4	SEC	W5PM33	SELP	3.23E-02	0.0000
-5	SEC	W5QJ62	ACTN1	1.43E-03	0.0000
-5	SEC	W5QFH5	RAB1A	2.64E-03	0.0000
-5	SEC	W5NQK6	LIMS1	2.87E-03	0.0000
-5	SEC	W5PRR5	LTA4H	1.78E-03	0.0000
-5	SEC	W5PK85	EML2	1.13E-02	0.0000
-5	SEC	W5PTZ9	LOC101120877	3.37E-03	0.0000
-5	SEC	W5Q5H8	FGA	3.19E-02	0.0000
-5	SEC	W5PY17	STX7	7.88E-03	0.0000
-5	SEC	W5Q3B7	PDE5A	3.55E-02	0.0000
-5	SEC	W5P733	PDLIM1	3.36E-02	0.0000
-5	SEC	W5PPG3	ALDH9A1	2.31E-02	0.0000
-5	SEC	W5QD96	PARVB	1.41E-03	0.0000
-5	SEC	W5P6Z6	MAPRE2	5.32E-03	0.0000
-5	SEC	W5QBD7	YWHAZ	5.75E-03	0.0000
-5	SEC	W5PL19	LOC101105123	2.94E-02	0.0000
-5	SEC	W5PLD5	CNN2	1.52E-02	0.0000
-5	SEC	W5PE27	ESD	2.04E-02	0.0000
-5	SEC	W5PRF0	SUB1	3.39E-04	0.0000
-5	SEC	W5QEK8	EHD3	1.14E-03	0.0000
-5	SEC	P62262	YWHAE	3.53E-02	0.0000
-5	SEC	W5PRP1	SLC9A3R1	6.51E-06	0.0000
-5	SEC	W5NRW4	TPM4	1.25E-02	0.0000
-5	SEC	W5NRD9		5.06E-03	0.0016
-5	SEC	W5Q731	ILK	1.43E-02	0.0000
-5	SEC	W5P4C7	SEPTIN7	5.23E-03	0.0000
-6	SEC	W5QB24	ANP32A	3.48E-02	0.0000
-6	SEC	W5QFZ3	CCT4	4.18E-02	0.0000
-6	SEC	W5PIJ6	PTPN11	2.27E-02	0.0000
-6	SEC	W5PX84	CCDC171	4.61E-04	0.0046
-6	SEC	W5NXW9		3.29E-04	0.0000
-6	SEC	W5QFM1		3.11E-02	0.0000

Supplementary Table 6: Differentially abundant proteins (DAPs) of MMØ relative to M0 and their cellular location.

FoldChange	Location	Protein IDs	Gene Names	P-value	FDR
-6	SEC	W5NUT8	PIP4K2A	1.00E-03	0.0000
-6	SEC	W5PEX2	GPATCH8	5.53E-04	0.0000
-6	SEC	W5P627	GSN	3.68E-03	0.0000
-6	SEC	W5PH15	RSU1	2.34E-03	0.0000
-7	SEC	W5Q9H1	ZYX	2.56E-02	0.0000
-7	SEC	W5Q2S8	MYL9	1.70E-03	0.0000
-7	SEC	W5PQK6	TLN1	2.34E-02	0.0000
-7	SEC	W5QF71	PLEK	6.54E-04	0.0000
-7	SEC	W5P4L3	AVIL	1.76E-03	0.0000
-7	SEC	W5PG95	HSPA1A	9.28E-04	0.0000
-7	SEC	W5PP64	FHL1	9.01E-04	0.0000
-8	SEC	W5PD82	CALD1	1.89E-03	0.0000
-8	SEC	W5PFI7	VCL	5.22E-04	0.0000
-8	SEC	W5QIK8	SELENBP1	8.81E-04	0.0000
-8	SEC	W5PK38	VASP	3.03E-03	0.0000
-8	SEC	W5Q3I7	TUBB1	1.93E-03	0.0000
-9	SEC	W5PYX0		7.91E-03	0.0000
-9	SEC	W5PFJ0	VCL	2.67E-03	0.0000

MMØ, monocyte-derived macrophages differentiated with M-CSF (Macrophage Colony-Stimulating Factor) and M0, Monocytes at 3 hours. Differentially abundant proteins (DAPs) were identified using a threshold of false discovery rate (FDR, q-value) ≤ 0.05 and absolute fold change ≥ 2. Red-highlighted cells indicate shared differentially abundant proteins (DAPs) between the cell lysate (CYTO) and secretome (SEC) compartments in MMØ relative to monocytes, whereas non-highlighted cells represent compartment-specific DAPs unique to either the whole cell lysate or secretome.

Supplementary Table 7: Shared and unique differentially abundant proteins (DAPs) between MMØ and GMØ relative to M0 and their cellular location.

FoldChange	Location	Protein IDs	Gene Names	P-value	FDR	Cell type
10	CYTO	W5PCH3	SCIN	4.35E-05	0.0000	MM0_Mo
10	CYTO	W5PE67		3.78E-05	0.0000	MM0_Mo
10	CYTO	W5NU86	GLA	5.61E-04	0.0000	MM0_Mo
9	CYTO	W5PD43	HTRA1	2.26E-03	0.0000	MM0_Mo
9	CYTO	W5PT76	GPNMB	8.82E-05	0.0000	MM0_Mo
8	CYTO	W5PAM4	CTSA	5.18E-05	0.0000	MM0_Mo
8	CYTO	W5PF33	GM2A	4.93E-05	0.0000	MM0_Mo
8	CYTO	W5QHG1	EPS8	7.34E-04	0.0000	MM0_Mo
8	CYTO	W5NY01		8.11E-04	0.0000	MM0_Mo
7	CYTO	P35623	SHMT1	1.92E-05	0.0000	MM0_Mo
7	CYTO	W5PIQ6	MSR1	7.62E-05	0.0000	MM0_Mo
7	CYTO	W5Q6N3	LOC101115115	1.06E-04	0.0000	MM0_Mo
7	CYTO	W5PXR1	ENPP1	3.46E-04	0.0000	MM0_Mo
7	CYTO	W5PTU7	CA2	6.13E-05	0.0000	MM0_Mo
7	CYTO	W5PQR0	NIBAN2	5.11E-06	0.0000	MM0_Mo
7	CYTO	P51977	ALDH1	1.93E-03	0.0000	MM0_Mo
7	CYTO	W5PBM9	SCPEP1	1.53E-04	0.0000	MM0_Mo
7	CYTO	W5P9M8	UCK1	2.71E-05	0.0002	MM0_Mo
7	CYTO	W5P9J8	BLVRB	1.24E-04	0.0000	MM0_Mo
7	CYTO	W5PFY5	ASAH1	1.60E-03	0.0000	MM0_Mo
7	CYTO	W5NX56	SPP1	9.19E-05	0.0000	MM0_Mo
7	CYTO	W5PGC5	GALM	5.94E-04	0.0000	MM0_Mo
7	CYTO	G3M9U4	ACP5	1.62E-04	0.0000	MM0_Mo
6	CYTO	W5P9G8	PLD3	2.32E-02	0.0000	MM0_Mo
6	CYTO	W5PCE0	PLBD2	4.28E-03	0.0000	MM0_Mo
6	CYTO	W5QC89	HEXA	1.10E-04	0.0000	MM0_Mo
6	CYTO	W5Q0Y4	ZFAND6	2.75E-03	0.0000	MM0_Mo
6	CYTO	A9YUY8	FABP4	8.63E-04	0.0000	MM0_Mo
6	CYTO	W5PZB2	CD68	1.49E-04	0.0000	MM0_Mo
6	CYTO	W5Q612	TRPV2	2.86E-05	0.0000	MM0_Mo
6	CYTO	W5NTZ3	RENBP	2.88E-04	0.0000	MM0_Mo
6	CYTO	W5Q2Y1	PLXNC1	6.97E-03	0.0000	MM0_Mo
6	CYTO	W5PAQ4	FUCA1	2.79E-04	0.0000	MM0_Mo
6	CYTO	W5QIW1	LGALS3	3.66E-03	0.0000	MM0_Mo
6	CYTO	W5PEB0	FABP7	1.29E-03	0.0000	MM0_Mo
6	CYTO	W5QCL8	NPL	5.43E-04	0.0000	MM0_Mo
6	CYTO	W5P026	STAB1	3.62E-05	0.0000	MM0_Mo
6	CYTO	W5PBS4	LRP1	2.88E-04	0.0000	MM0_Mo
6	CYTO	W5Q940	SHTN1	3.00E-03	0.0000	MM0_Mo
6	CYTO	W5PJS4	EMILIN2	1.67E-04	0.0000	MM0_Mo
6	CYTO	W5NUI6	SGSH	5.73E-05	0.0000	MM0_Mo
6	CYTO	W5NVW7	NAGLU	3.08E-03	0.0000	MM0_Mo
6	CYTO	Q9MZS8	CTSD	1.31E-03	0.0000	MM0_Mo
6	CYTO	W5P8M9		8.65E-04	0.0000	MM0_Mo
6	CYTO	W5P8H9	SGPL1	6.53E-04	0.0000	MM0_Mo
6	CYTO	W5QI00	LACTB	5.73E-04	0.0000	MM0_Mo
6	CYTO	W5P1A5	GBA1	5.41E-04	0.0000	MM0_Mo
6	CYTO	W5P5W6	NDRG1	9.95E-03	0.0000	MM0_Mo
6	CYTO	W5QEM8	LOC101111528	9.50E-05	0.0000	MM0_Mo
6	CYTO	W5QCD6	IDH1	2.87E-03	0.0000	MM0_Mo
6	CYTO	W5PZG5	OCRL	6.87E-04	0.0000	MM0_Mo
6	CYTO	W5PDH7	NPC1	8.96E-05	0.0000	MM0_Mo
6	CYTO	W5NZK6	PLA2G15	1.92E-04	0.0000	MM0_Mo
6	CYTO	W5QGG0	TFRC	2.76E-05	0.0000	MM0_Mo
6	CYTO	P05028	ATP1B1	2.26E-03	0.0000	MM0_Mo
5	CYTO	W5QBZ7	NAGA	2.57E-03	0.0000	MM0_Mo
5	CYTO	W5NPP2	CPM	1.71E-04	0.0000	MM0_Mo
5	CYTO	W5PI56	DAB2	2.50E-03	0.0000	MM0_Mo
5	CYTO	W5PKY1	HNMT	8.15E-04	0.0000	MM0_Mo
5	CYTO	W5PRI6	MRC1	1.45E-03	0.0000	MM0_Mo
5	CYTO	W5NX16		6.82E-05	0.0000	MM0_Mo
5	CYTO	W5P0C5	LGALS8	5.76E-05	0.0000	MM0_Mo
5	CYTO	W5NQS7	IFNGR1	1.35E-04	0.0000	MM0_Mo

Supplementary Table 7: Shared and unique differentially abundant proteins (DAPs) between MMØ and GMØ relative to M0 and their cellular location.

FoldChange	Location	Protein IDs	Gene Names	P-value	FDR	Cell type
5	CYTO	W5PDJ0		9.23E-03	0.0000	MM0_Mo
5	CYTO	W5NYL7	MTHFD1L	1.11E-04	0.0000	MM0_Mo
5	CYTO	W5QHL1	FCGR1A	6.03E-04	0.0000	MM0_Mo
5	CYTO	W5P640	LMNA	3.40E-03	0.0000	MM0_Mo
5	CYTO	W5NRS0		9.20E-04	0.0000	MM0_Mo
5	CYTO	W5PWX3	CRYL1	4.48E-04	0.0000	MM0_Mo
5	CYTO	W5PV43	LRPAP1	3.81E-05	0.0000	MM0_Mo
5	CYTO	W5QI40	MYO1E	3.68E-03	0.0000	MM0_Mo
5	CYTO	W5NVR9	C21H11orf54	2.89E-05	0.0000	MM0_Mo
5	CYTO	W5QBM4	ALCAM	4.75E-04	0.0000	MM0_Mo
5	CYTO	W5Q678	SEC24D	6.14E-03	0.0000	MM0_Mo
5	CYTO	W5PG10	PAPSS1	1.06E-02	0.0000	MM0_Mo
5	CYTO	W5PYI8	WWC1	1.22E-03	0.0000	MM0_Mo
5	CYTO	W5PE92	GRN	3.81E-04	0.0000	MM0_Mo
5	CYTO	W5NYU9	MPP1	1.47E-05	0.0000	MM0_Mo
5	CYTO	W5PXS1	RAB3IL1	6.12E-04	0.0000	MM0_Mo
5	CYTO	W5PY08		6.80E-03	0.0000	MM0_Mo
5	CYTO	W5PPX2	SENP8	5.16E-04	0.0030	MM0_Mo
5	CYTO	W5PGS4	FABP5	5.39E-04	0.0000	MM0_Mo
5	CYTO	W5PBC0		3.92E-03	0.0000	MM0_Mo
5	CYTO	W5P7Y8	PALD1	4.71E-05	0.0000	MM0_Mo
5	CYTO	W5NZ62	GNS	3.48E-03	0.0000	MM0_Mo
5	CYTO	W5QFU4		7.64E-03	0.0000	MM0_Mo
5	CYTO	W5Q7I8	TAX1BP3	3.46E-02	0.0000	MM0_Mo
5	CYTO	W5QBG8	PPFIA1	4.74E-03	0.0000	MM0_Mo
5	CYTO	P83205	CTSB	2.50E-03	0.0000	MM0_Mo
5	CYTO	W5Q5T7	ATP6V1C1	1.03E-02	0.0000	MM0_Mo
5	CYTO	W5PKU3		7.64E-04	0.0000	MM0_Mo
5	CYTO	W5NSH8	NPC2	1.49E-05	0.0000	MM0_Mo
5	CYTO	W5QI78	CTSK	2.23E-02	0.0000	MM0_Mo
5	CYTO	Q29524	LPL	4.31E-03	0.0000	MM0_Mo
5	CYTO	W5Q3U3	LOC101102156	6.52E-03	0.0000	MM0_Mo
5	CYTO	W5P2V3	PEPD	2.18E-03	0.0000	MM0_Mo
5	CYTO	W5P2F1	FOLR2	1.14E-05	0.0000	MM0_Mo
5	CYTO	W5Q5C8	SOAT1	4.09E-04	0.0000	MM0_Mo
5	CYTO	W5Q0F1	LIPA	3.44E-04	0.0000	MM0_Mo
5	CYTO	P12303	TTR	1.10E-02	0.0000	MM0_Mo
4	CYTO	W5P1H0	CTSC	5.68E-06	0.0000	MM0_Mo
4	CYTO	W5Q5W2	LOC101110539	2.68E-04	0.0000	MM0_Mo
4	CYTO	W5P5Q2	MVP	1.16E-02	0.0000	MM0_Mo
4	CYTO	W5PP04	GNG12	8.75E-04	0.0000	MM0_Mo
4	CYTO	W5PJY6	ADAM28	2.53E-03	0.0000	MM0_Mo
4	CYTO	O18882	ATP6V0C ATP6C ATP	2.26E-03	0.0000	MM0_Mo
4	CYTO	W5PEE9	LAMP1	1.05E-02	0.0000	MM0_Mo
4	CYTO	W5Q0A3	TLR2	1.78E-03	0.0000	MM0_Mo
4	CYTO	W5Q8J3	RRBP1	6.57E-03	0.0000	MM0_Mo
4	CYTO	W5NYE0	ATP6V0D1	3.49E-02	0.0000	MM0_Mo
4	CYTO	W5P3H8	IGF2R	1.86E-03	0.0000	MM0_Mo
4	CYTO	W5NU23	FUCA2	2.90E-04	0.0000	MM0_Mo
4	CYTO	W5PNC8	PADI2	1.06E-02	0.0000	MM0_Mo
4	CYTO	W5PAC2	LOC101105044	9.35E-03	0.0000	MM0_Mo
4	CYTO	W5PYW0	TCIRG1	2.98E-04	0.0000	MM0_Mo
4	CYTO	W5NYL0	MAOA	5.92E-03	0.0000	MM0_Mo
4	CYTO	W5QH35	CAPG	4.17E-04	0.0000	MM0_Mo
4	CYTO	W5NUC8	ARMCX3	4.25E-03	0.0000	MM0_Mo
4	CYTO	W5PUL5	FCGRT	1.03E-03	0.0000	MM0_Mo
4	CYTO	W5Q777	HS1BP3	3.09E-04	0.0000	MM0_Mo
4	CYTO	W5PVR9	ERMP1	2.46E-02	0.0000	MM0_Mo
4	CYTO	W5Q0U0	P2RX4	3.22E-03	0.0000	MM0_Mo
4	CYTO	W5QB71	AMDHD2	1.27E-03	0.0000	MM0_Mo
4	CYTO	W5NSQ3	HFE	2.77E-04	0.0000	MM0_Mo
4	CYTO	W5QA16		1.83E-04	0.0000	MM0_Mo

Supplementary Table 7: Shared and unique differentially abundant proteins (DAPs) between MMØ and GMØ relative to M0 and their cellular location.

FoldChange	Location	Protein IDs	Gene Names	P-value	FDR	Cell type
4	CYTO	W5QEH8	CTTNBP2NL	1.37E-05	0.0000	MM0_Mo
4	CYTO	W5PE91	LOC101105400	3.31E-04	0.0002	MM0_Mo
4	CYTO	Q9MZD1	SLC17A5	8.13E-04	0.0000	MM0_Mo
4	CYTO	W5NS94		4.20E-05	0.0000	MM0_Mo
4	CYTO	W5QJ37		3.55E-05	0.0000	MM0_Mo
4	CYTO	C5IJ93	RAB9A	2.68E-05	0.0000	MM0_Mo
4	CYTO	W5P3N6	LOC101112162	8.05E-04	0.0000	MM0_Mo
4	CYTO	W5Q700	APPL2	3.00E-05	0.0000	MM0_Mo
4	CYTO	W5NW80	GAA	2.58E-03	0.0000	MM0_Mo
4	CYTO	W5P168	SH3PXD2B	1.81E-05	0.0000	MM0_Mo
4	CYTO	W5PBG1		7.32E-03	0.0000	MM0_Mo
4	CYTO	W5PDZ1		1.29E-03	0.0000	MM0_Mo
4	CYTO	W5P530	LOC101104705	5.19E-02	0.0000	MM0_Mo
4	CYTO	W5PA90	AGA	1.20E-04	0.0000	MM0_Mo
4	CYTO	W5NUQ8	GCC1	1.26E-02	0.0000	MM0_Mo
4	CYTO	W5QEH0	TWF1	3.98E-03	0.0000	MM0_Mo
4	CYTO	W5P3L5	RNF13	2.52E-03	0.0000	MM0_Mo
4	CYTO	W5P5L4	COG5	1.02E-04	0.0000	MM0_Mo
4	CYTO	W5Q2U7	PLEC	1.21E-06	0.0000	MM0_Mo
4	CYTO	W5Q3N1	CTSZ	2.79E-05	0.0000	MM0_Mo
4	CYTO	W5PFE7	ACOX1	5.07E-04	0.0000	MM0_Mo
4	CYTO	W5P4A8	RNASET2	4.33E-04	0.0000	MM0_Mo
4	CYTO	W5Q539	ABL2	1.08E-02	0.0000	MM0_Mo
4	CYTO	W5PIS6	NHLRC3	2.84E-03	0.0000	MM0_Mo
4	CYTO	W5P6H9	ACP2	3.83E-02	0.0000	MM0_Mo
4	CYTO	W5PR96	DCK	3.01E-03	0.0004	MM0_Mo
4	CYTO	W5P3S0		4.23E-05	0.0000	MM0_Mo
4	CYTO	W5PAJ2	PSAP	7.07E-05	0.0000	MM0_Mo
4	CYTO	W5QI70	CTSS	4.65E-04	0.0000	MM0_Mo
4	CYTO	W5Q9L2	LOC101109820	6.22E-03	0.0000	MM0_Mo
4	CYTO	W5NYK1	PMVK	1.12E-03	0.0000	MM0_Mo
4	CYTO	W5Q1M0	GLB1	1.54E-04	0.0000	MM0_Mo
4	CYTO	W5QI36	HEBP1	1.97E-02	0.0000	MM0_Mo
4	CYTO	W5PRG8	CREG1	4.88E-03	0.0000	MM0_Mo
4	CYTO	W5NQZ9	GSDMD	1.31E-03	0.0000	MM0_Mo
4	CYTO	W5Q9M9	GK	2.91E-02	0.0000	MM0_Mo
4	CYTO	W5QHU8	FNDC3B	5.53E-05	0.0000	MM0_Mo
4	CYTO	W5P7F8		3.76E-02	0.0000	MM0_Mo
4	CYTO	W5Q6C5	GGA2	6.95E-03	0.0000	MM0_Mo
4	CYTO	W5P6L1	RASGRP4	8.55E-04	0.0000	MM0_Mo
4	CYTO	W5PFB1	TOR1B	3.43E-03	0.0000	MM0_Mo
4	CYTO	W5Q263	ICAM1	1.37E-02	0.0000	MM0_Mo
4	CYTO	W5PNP1	MFGE8	2.15E-02	0.0000	MM0_Mo
4	CYTO	W5PBJ4	ARHGAP10	4.12E-03	0.0000	MM0_Mo
4	CYTO	W5NUI3	TREM2	3.27E-02	0.0000	MM0_Mo
4	CYTO	W5QCM1	ARFGAP3	2.08E-02	0.0000	MM0_Mo
4	CYTO	W5NZV3	HMOX2	4.41E-02	0.0000	MM0_Mo
4	CYTO	W5Q3V0	IL18	1.05E-05	0.0000	MM0_Mo
4	CYTO	W5PZD7		2.75E-04	0.0000	MM0_Mo
4	CYTO	W5Q8V2	LIMA1	1.34E-03	0.0000	MM0_Mo
4	CYTO	W5P800	GAS7	3.09E-02	0.0000	MM0_Mo
4	CYTO	W5NZA6	RNF121	2.13E-03	0.0000	MM0_Mo
4	CYTO	W5NSS6		1.62E-02	0.0000	MM0_Mo
4	CYTO	W5NWP6	DIAPH2	1.14E-02	0.0000	MM0_Mo
4	CYTO	W5PF85	PEA15	1.03E-04	0.0000	MM0_Mo
4	CYTO	W5P4X6	LOC101104287	6.06E-03	0.0000	MM0_Mo
4	CYTO	W5PSE5	LOC101110434	2.76E-02	0.0002	MM0_Mo
4	CYTO	W5PLB8	EPB41L3	1.13E-03	0.0000	MM0_Mo
4	CYTO	W5NTE2	PSMG4	3.44E-02	0.0000	MM0_Mo
4	CYTO	W5Q6S3	WASHC4	2.74E-03	0.0000	MM0_Mo
4	CYTO	W5NY99		2.38E-03	0.0000	MM0_Mo
4	CYTO	W5P895	CRABP2	1.32E-02	0.0000	MM0_Mo

Supplementary Table 7: Shared and unique differentially abundant proteins (DAPs) between MMØ and GMØ relative to M0 and their cellular location.

FoldChange	Location	Protein IDs	Gene Names	P-value	FDR	Cell type
4	CYTO	W5P4K6	FCHO2	5.47E-04	0.0000	MM0_Mo
3	CYTO	W5P743	GLMP	2.49E-03	0.0000	MM0_Mo
3	CYTO	W5PI67	IDS	1.71E-03	0.0000	MM0_Mo
3	CYTO	W5Q175	GNPTAB	1.44E-03	0.0000	MM0_Mo
3	CYTO	W5PC32	PGM3	4.51E-03	0.0000	MM0_Mo
3	CYTO	W5PKF9	FIG4	9.17E-04	0.0000	MM0_Mo
3	CYTO	W5QHC0	ST3GAL5	5.75E-03	0.0000	MM0_Mo
3	CYTO	W5PVH4	TMEM251	9.32E-04	0.0000	MM0_Mo
3	CYTO	W5P703	WFS1	4.08E-03	0.0000	MM0_Mo
3	CYTO	W5Q5Q7	ASPA	4.27E-02	0.0000	MM0_Mo
3	CYTO	W5P434	NAGPA	1.84E-03	0.0000	MM0_Mo
3	CYTO	W5PVM8	UBR7	1.05E-04	0.0000	MM0_Mo
3	CYTO	W5PCS4		1.31E-04	0.0000	MM0_Mo
3	CYTO	W5QG92	OSBPL11	2.43E-04	0.0000	MM0_Mo
3	CYTO	W5PN65	PI4K2A	1.60E-03	0.0000	MM0_Mo
3	CYTO	W5Q233	VCAN	1.02E-02	0.0000	MM0_Mo
3	CYTO	W5PQV2	LMBRD1	2.81E-05	0.0000	MM0_Mo
3	CYTO	W5PUM8	HIP1	2.05E-03	0.0000	MM0_Mo
3	CYTO	W5Q5K0		3.61E-03	0.0000	MM0_Mo
3	CYTO	W5QH13	VPS39	7.28E-04	0.0000	MM0_Mo
3	CYTO	W5NTW0	CADM1	1.31E-02	0.0000	MM0_Mo
3	CYTO	W5Q4U5	CPT1A	7.71E-03	0.0000	MM0_Mo
3	CYTO	W5Q2J4	DIP2C	2.74E-04	0.0000	MM0_Mo
3	CYTO	W5NRL0	PLCD1	6.49E-04	0.0000	MM0_Mo
3	CYTO	W5PS52	MRC2	5.71E-03	0.0000	MM0_Mo
3	CYTO	W5PZ94	ACO1	3.42E-02	0.0000	MM0_Mo
3	CYTO	W5PIE4	CLPTM1	3.56E-02	0.0000	MM0_Mo
3	CYTO	W5P3C6	LOC101111906	1.02E-04	0.0000	MM0_Mo
3	CYTO	W5P6V4	GLG1	4.84E-02	0.0000	MM0_Mo
3	CYTO	W5PQA6	CYFIP2	2.04E-02	0.0000	MM0_Mo
3	CYTO	W5PFE6	ACOX1	1.76E-03	0.0000	MM0_Mo
3	CYTO	W5P3I5	CNDP2	1.36E-04	0.0000	MM0_Mo
3	CYTO	Q10994	CSTB CST6	1.88E-05	0.0000	MM0_Mo
3	CYTO	W5PGX8	DNAJC13	4.02E-02	0.0000	MM0_Mo
3	CYTO	W5P093	NQO1	6.25E-03	0.0000	MM0_Mo
3	CYTO	W5PHY4	TYMS	4.41E-03	0.0000	MM0_Mo
3	CYTO	W5NPW8	STARD4	4.84E-03	0.0000	MM0_Mo
3	CYTO	W5PT36	RBM47	2.11E-03	0.0000	MM0_Mo
3	CYTO	W5PER8	WDR91	1.38E-02	0.0000	MM0_Mo
3	CYTO	W5P2U3	IVNS1ABP	5.25E-02	0.0000	MM0_Mo
3	CYTO	W5QBR5	BMP2K	6.44E-03	0.0000	MM0_Mo
3	CYTO	W5PCD7	ABHD12	3.68E-02	0.0000	MM0_Mo
3	CYTO	W5PRM9	BCAT2	3.54E-02	0.0000	MM0_Mo
3	CYTO	W5Q7X8	CRYBG3	3.88E-03	0.0000	MM0_Mo
3	CYTO	W5PTE6	CEMIP2	4.88E-03	0.0000	MM0_Mo
3	CYTO	W5NZ70	LGALS3BP	1.52E-02	0.0000	MM0_Mo
3	CYTO	P82197	PDXK PKH	1.87E-02	0.0000	MM0_Mo
3	CYTO	W5NUJ2	PEAK1	7.94E-03	0.0000	MM0_Mo
3	CYTO	W5QH68	LRRC57	3.92E-02	0.0002	MM0_Mo
3	CYTO	W5NYU6	NT5DC2	4.01E-03	0.0000	MM0_Mo
3	CYTO	W5QIC7	YBX3	5.75E-03	0.0000	MM0_Mo
3	CYTO	W5Q0C3	KIF13B	2.97E-02	0.0000	MM0_Mo
3	CYTO	W5Q728	TPCN2	8.83E-04	0.0000	MM0_Mo
3	CYTO	W5NU07		1.11E-02	0.0000	MM0_Mo
3	CYTO	W5P2N4	AHCYL2	1.90E-04	0.0000	MM0_Mo
3	CYTO	W5Q3Y3	SFXN3	5.22E-03	0.0000	MM0_Mo
3	CYTO	W5Q3A2		2.85E-02	0.0000	MM0_Mo
3	CYTO	W5NQ85	IDE	2.06E-02	0.0000	MM0_Mo
3	CYTO	W5PB38		3.56E-03	0.0000	MM0_Mo
3	CYTO	W5P536	TRIP10	2.79E-03	0.0000	MM0_Mo
3	CYTO	W5QBV7	CD44	1.21E-04	0.0000	MM0_Mo
3	CYTO	W5P2W1	NEU1	3.51E-03	0.0000	MM0_Mo

Supplementary Table 7: Shared and unique differentially abundant proteins (DAPs) between MMØ and GMØ relative to M0 and their cellular location.

FoldChange	Location	Protein IDs	Gene Names	P-value	FDR	Cell type
3	CYTO	W5QAQ4	NRP2	1.55E-03	0.0000	MM0_Mo
3	CYTO	W5Q2V0	YKT6	8.34E-04	0.0000	MM0_Mo
3	CYTO	W5P1Q0	AP1B1	4.33E-05	0.0000	MM0_Mo
3	CYTO	W5QAT6	MTHFR	2.46E-03	0.0000	MM0_Mo
3	CYTO	W5Q928	PCYT2	3.83E-03	0.0000	MM0_Mo
3	CYTO	W5QAA7	MAPK7	6.91E-04	0.0000	MM0_Mo
3	CYTO	W5NYW8	CEP55	9.74E-03	0.0004	MM0_Mo
3	CYTO	W5QCG3	CLCC1	3.64E-03	0.0000	MM0_Mo
3	CYTO	W5NV37	COMMD10	2.05E-02	0.0000	MM0_Mo
3	CYTO	W5NRA9	ASL	1.61E-02	0.0000	MM0_Mo
3	CYTO	W5QDN8	SLC48A1	3.35E-03	0.0002	MM0_Mo
3	CYTO	W5P8A0		3.28E-02	0.0000	MM0_Mo
3	CYTO	W5P333		1.82E-02	0.0000	MM0_Mo
3	CYTO	W5Q3B4	TBCD	1.66E-03	0.0000	MM0_Mo
3	CYTO	W5PN70		1.02E-02	0.0000	MM0_Mo
3	CYTO	W5QI95	CERS2	1.46E-02	0.0000	MM0_Mo
3	CYTO	W5PVQ4	TMEM120A	1.47E-04	0.0000	MM0_Mo
3	CYTO	W5P366	FAM50A	3.55E-02	0.0000	MM0_Mo
3	CYTO	W5QHR5	PLEKHO2	9.32E-05	0.0000	MM0_Mo
3	CYTO	W5PVE3	LOC101115252	2.49E-04	0.0000	MM0_Mo
3	CYTO	W5NRQ3	TRIM68	5.51E-03	0.0015	MM0_Mo
3	CYTO	W5NWX4		3.25E-02	0.0000	MM0_Mo
3	CYTO	W5PCM4	LUZP1	4.41E-04	0.0000	MM0_Mo
3	CYTO	W5PAX1	GCLC	2.65E-02	0.0000	MM0_Mo
3	CYTO	W5QBJ2	SLC38A10	1.35E-03	0.0000	MM0_Mo
3	CYTO	W5QCP4	TRAF6	2.28E-03	0.0000	MM0_Mo
3	CYTO	W5PCC0	LACC1	7.04E-03	0.0000	MM0_Mo
3	CYTO	W5PDU4	NMT2	6.17E-03	0.0000	MM0_Mo
3	CYTO	W5PCD0	FUBP3	3.58E-02	0.0000	MM0_Mo
3	CYTO	W5PBX0	ATP11A	4.64E-02	0.0000	MM0_Mo
3	CYTO	W5PB61		2.72E-02	0.0002	MM0_Mo
3	CYTO	W5PH35	LOC101119706	3.30E-03	0.0000	MM0_Mo
3	CYTO	W5NPU0	NAPRT	1.07E-02	0.0000	MM0_Mo
3	CYTO	W5P3W6	OSBPL9	5.90E-03	0.0000	MM0_Mo
3	CYTO	W5PUI3	GOLGA1	3.92E-05	0.0000	MM0_Mo
3	CYTO	W5PPR1	UCK2	2.71E-03	0.0002	MM0_Mo
3	CYTO	W5PEX1	WASHC5	1.39E-02	0.0000	MM0_Mo
3	CYTO	W5Q2K9	CYFIP1	1.08E-03	0.0000	MM0_Mo
3	CYTO	W5PU61	SETD7	1.90E-02	0.0000	MM0_Mo
3	CYTO	W5QGT0	ATP13A3	1.78E-02	0.0000	MM0_Mo
3	CYTO	W5PPK8		8.00E-03	0.0000	MM0_Mo
3	CYTO	W5P5P8	PSMG3	5.21E-02	0.0000	MM0_Mo
3	CYTO	W5QET8	TEP1	1.69E-02	0.0000	MM0_Mo
3	CYTO	W5P1T1	ANO10	6.70E-03	0.0000	MM0_Mo
3	CYTO	W5QEA3		1.05E-03	0.0000	MM0_Mo
3	CYTO	W5NRF7	MAP3K20	1.02E-03	0.0000	MM0_Mo
3	CYTO	W5QFH0	ARSA	1.39E-03	0.0000	MM0_Mo
3	CYTO	W5PAP3	FCHSD2	4.33E-02	0.0000	MM0_Mo
3	CYTO	W5PFU8	KAT6B	8.62E-03	0.0006	MM0_Mo
3	CYTO	W5PUL4	MTMR6	2.36E-02	0.0000	MM0_Mo
3	CYTO	W5NT95	ATP6AP2	3.50E-05	0.0000	MM0_Mo
3	CYTO	W5P369	AP2A2	4.54E-06	0.0000	MM0_Mo
3	CYTO	W5NVV6	DNAJC3	3.70E-02	0.0000	MM0_Mo
3	CYTO	W5PJN7	HOMER3	4.78E-03	0.0000	MM0_Mo
3	CYTO	W5PQ75	HSPH1	2.37E-02	0.0000	MM0_Mo
3	CYTO	W5Q4Q8		4.77E-04	0.0000	MM0_Mo
3	CYTO	W5Q1W2	SDCBP	4.09E-02	0.0000	MM0_Mo
3	CYTO	W5PUW2	IFI30	1.85E-03	0.0000	MM0_Mo
3	CYTO	W5Q5N6	BST-2B	2.34E-02	0.0000	MM0_Mo
3	CYTO	W5PJ58	HPS5	4.85E-02	0.0000	MM0_Mo
3	CYTO	W5QIJ6	SPPL2A	3.02E-02	0.0000	MM0_Mo
3	CYTO	W5P3X8	KIF15	9.19E-03	0.0000	MM0_Mo

Supplementary Table 7: Shared and unique differentially abundant proteins (DAPs) between MMØ and GMØ relative to M0 and their cellular location.

FoldChange	Location	Protein IDs	Gene Names	P-value	FDR	Cell type
3	CYTO	W5P8R4	CSF1R	7.81E-03	0.0000	MM0_Mo
3	CYTO	W5PG41	H6PD	2.71E-02	0.0000	MM0_Mo
3	CYTO	W5Q3C2	EPPK1	9.43E-03	0.0000	MM0_Mo
3	CYTO	W5P3B0	INPPL1	4.61E-02	0.0000	MM0_Mo
3	CYTO	W5PWR6	KCNAB2	2.64E-03	0.0000	MM0_Mo
3	CYTO	W5PEZ1		5.02E-02	0.0000	MM0_Mo
3	CYTO	W5Q0J1	PLA2G6	6.01E-03	0.0046	MM0_Mo
3	CYTO	W5Q9E6	TANGO2	1.39E-03	0.0000	MM0_Mo
3	CYTO	W5NWX7	CLEC4A	1.33E-02	0.0000	MM0_Mo
3	CYTO	W5Q8I7	LOC101122123	4.39E-03	0.0000	MM0_Mo
3	CYTO	W5PC06	SIRPA	5.64E-04	0.0000	MM0_Mo
3	CYTO	W5PI02	TBC1D13	2.12E-02	0.0000	MM0_Mo
3	CYTO	W5PQM4	MOSPD2	3.98E-04	0.0000	MM0_Mo
3	CYTO	W5PWA8	HSPB1	4.78E-03	0.0000	MM0_Mo
3	CYTO	W5PBR7	P4HA1	2.12E-02	0.0000	MM0_Mo
3	CYTO	W5QEL6	PCYOX1	2.53E-02	0.0000	MM0_Mo
3	CYTO	W5PAM5		1.76E-03	0.0000	MM0_Mo
3	CYTO	W5Q831		2.19E-02	0.0000	MM0_Mo
3	CYTO	W5PVH8	ATP6V1E1	1.37E-03	0.0000	MM0_Mo
3	CYTO	W5P3H1	LOC101111732	7.78E-03	0.0000	MM0_Mo
3	CYTO	W5PBB5	CTSF	1.03E-02	0.0000	MM0_Mo
3	CYTO	W5NUU1	VRK2	9.96E-03	0.0000	MM0_Mo
3	CYTO	W5PSG0		5.75E-03	0.0000	MM0_Mo
3	CYTO	W5QDQ8	MMP14	8.31E-03	0.0000	MM0_Mo
3	CYTO	Q6XXL8	DYNLT3 TCTE1L	7.71E-03	0.0000	MM0_Mo
3	CYTO	W5QCF3	SLC35F6	8.58E-03	0.0000	MM0_Mo
3	CYTO	W5P700	KIF1B	1.54E-02	0.0000	MM0_Mo
3	CYTO	W5NV06	ATP6V0A1	3.64E-02	0.0000	MM0_Mo
3	CYTO	W5Q7Z6	DIP2B	6.60E-03	0.0000	MM0_Mo
3	CYTO	W5PCX5	CC2D1A	3.64E-02	0.0002	MM0_Mo
3	CYTO	W5P1S3	SLC25A13	4.68E-03	0.0000	MM0_Mo
3	CYTO	W5NYK9	CALU	1.47E-02	0.0000	MM0_Mo
3	CYTO	W5P5E7		5.40E-03	0.0000	MM0_Mo
3	CYTO	W5QDC0	SNX17	3.27E-02	0.0000	MM0_Mo
3	CYTO	W5P9L5	RASA2	2.43E-03	0.0002	MM0_Mo
3	CYTO	W5PEN2	TPD52	4.53E-03	0.0000	MM0_Mo
3	CYTO	W5Q644	VPS29	4.59E-02	0.0000	MM0_Mo
3	CYTO	W5PKV1	DNASE2	1.51E-02	0.0000	MM0_Mo
3	CYTO	W5QH60	VAMP8	3.22E-02	0.0004	MM0_Mo
3	CYTO	W5Q8Y5	HDLBP	5.67E-03	0.0000	MM0_Mo
3	CYTO	Q9XT28	ATOX1	1.32E-03	0.0000	MM0_Mo
3	CYTO	W5PYH6	AP3D1	1.92E-02	0.0000	MM0_Mo
3	CYTO	W5P1U3	LACTB2	4.68E-02	0.0000	MM0_Mo
3	CYTO	W5Q553	ITGAV	6.35E-03	0.0000	MM0_Mo
3	CYTO	W5QDF4	GSTM3	5.75E-03	0.0000	MM0_Mo
3	CYTO	W5Q2H2	SLC12A9	8.24E-04	0.0000	MM0_Mo
3	CYTO	W5Q165	RRM2	1.51E-03	0.0000	MM0_Mo
2	CYTO	W5QFK2	MACF1	2.93E-02	0.0000	MM0_Mo
2	CYTO	W5QAL6	FMNL3	9.21E-03	0.0002	MM0_Mo
2	CYTO	W5Q420		2.66E-02	0.0000	MM0_Mo
2	CYTO	W5PER3	TM9SF3	2.87E-02	0.0000	MM0_Mo
2	CYTO	W5PDK4	AIF1	4.47E-02	0.0000	MM0_Mo
2	CYTO	W5NR48	KPNA6	4.64E-02	0.0000	MM0_Mo
2	CYTO	W5QCA6	UBE2F	3.16E-04	0.0000	MM0_Mo
2	CYTO	W5PK12	OAT	7.44E-05	0.0000	MM0_Mo
2	CYTO	W5PCU1	ASS1	4.04E-03	0.0000	MM0_Mo
2	CYTO	W5PWB8	ARHGAP12	6.98E-03	0.0000	MM0_Mo
2	CYTO	W5Q8K4	SLC3A2	5.20E-03	0.0000	MM0_Mo
2	CYTO	W5PS50	MPC2	2.26E-02	0.0000	MM0_Mo
2	CYTO	W5PN60	ABR	3.09E-02	0.0000	MM0_Mo
2	CYTO	W5QB79	GLCE	1.83E-02	0.0002	MM0_Mo
2	CYTO	W5PQZ7	PATL1	1.72E-02	0.0000	MM0_Mo

Supplementary Table 7: Shared and unique differentially abundant proteins (DAPs) between MMØ and GMØ relative to M0 and their cellular location.

FoldChange	Location	Protein IDs	Gene Names	P-value	FDR	Cell type
2	CYTO	W5QIU9	S100A10	1.17E-02	0.0000	MM0_Mo
2	CYTO	W5Q686	TPP1	1.14E-02	0.0000	MM0_Mo
2	CYTO	P81184	LGALS1	2.17E-04	0.0000	MM0_Mo
2	CYTO	P04074	ATP1A1	2.02E-03	0.0000	MM0_Mo
2	CYTO	Q6XUZ5	IDH1	4.98E-05	0.0000	MM0_Mo
2	CYTO	W5NZ71		5.17E-02	0.0000	MM0_Mo
2	CYTO	W5PP47		2.26E-04	0.0000	MM0_Mo
2	CYTO	W5QIX6	EXOC5	3.67E-02	0.0000	MM0_Mo
2	CYTO	W5PMA0	AP2S1	2.27E-02	0.0000	MM0_Mo
2	CYTO	W5PZ62	ZFYVE16	5.84E-03	0.0000	MM0_Mo
2	CYTO	W5Q3L8	LOC101104306	2.34E-03	0.0000	MM0_Mo
2	CYTO	W5PL89	GSR	2.28E-02	0.0000	MM0_Mo
2	CYTO	W5P3A2		2.32E-03	0.0000	MM0_Mo
2	CYTO	W5P7L2	ATP6V1G1	5.71E-04	0.0000	MM0_Mo
2	CYTO	W5PSP1	ERC1	4.65E-02	0.0002	MM0_Mo
2	CYTO	W5QHV8	PLD1	3.24E-02	0.0000	MM0_Mo
2	CYTO	W5P6S8	COBLL1	1.82E-02	0.0000	MM0_Mo
2	CYTO	W5NT19	SPAG1	5.60E-02	0.0000	MM0_Mo
2	CYTO	W5PUM5	KANK1	2.99E-03	0.0000	MM0_Mo
2	CYTO	W5PPR6	EXOC6B	9.64E-03	0.0000	MM0_Mo
2	CYTO	W5QBW4	VAV3	1.52E-04	0.0000	MM0_Mo
2	CYTO	W5QIA8	YARS1	1.11E-03	0.0000	MM0_Mo
2	CYTO	W5PP17	LOC101123010	3.99E-02	0.0002	MM0_Mo
2	CYTO	W5P4T0	VHL	8.97E-04	0.0002	MM0_Mo
2	CYTO	W5PVC8	ERGIC3	4.30E-03	0.0000	MM0_Mo
2	CYTO	W5QAP3	TOM1	2.48E-03	0.0000	MM0_Mo
2	CYTO	W5NSS0	ARAP3	2.53E-03	0.0000	MM0_Mo
2	CYTO	W5QAE8	ALDH7A1	2.25E-04	0.0000	MM0_Mo
2	CYTO	W5PGL9		4.67E-02	0.0000	MM0_Mo
2	CYTO	W5Q6E0	CUL2	2.56E-02	0.0000	MM0_Mo
2	CYTO	W5Q4N4	TOR3A	1.29E-02	0.0000	MM0_Mo
2	CYTO	W5PLZ0	ATP6V1B2	8.69E-04	0.0000	MM0_Mo
2	CYTO	W5P4B3	NRDC	3.10E-02	0.0000	MM0_Mo
2	CYTO	W5PNW7	VIM	3.10E-05	0.0000	MM0_Mo
2	CYTO	W5P7L0	SUN1	8.59E-03	0.0002	MM0_Mo
2	CYTO	W5NUG3	GNPDA1	4.24E-02	0.0000	MM0_Mo
2	CYTO	W5P1M3		4.94E-02	0.0000	MM0_Mo
2	CYTO	W5Q5Z1	YTHDF3	1.66E-02	0.0000	MM0_Mo
2	CYTO	W5PRS4	FKBP5	2.72E-02	0.0000	MM0_Mo
2	CYTO	W5Q7V3		6.47E-04	0.0000	MM0_Mo
2	CYTO	W5QHX1	EIF5A2	1.23E-02	0.0000	MM0_Mo
2	CYTO	W5QIA5	ETV6	4.40E-02	0.0000	MM0_Mo
2	CYTO	W5PUJ2	LAMP2	5.82E-03	0.0000	MM0_Mo
2	CYTO	W5PVT3	GALNS	1.72E-02	0.0000	MM0_Mo
2	CYTO	W5PXJ8	CMBL	1.10E-03	0.0000	MM0_Mo
2	CYTO	W5PHS5	RGL2	5.12E-02	0.0000	MM0_Mo
2	CYTO	W5Q120	ANKRD28	3.07E-02	0.0002	MM0_Mo
2	CYTO	W5NRI6	PLPBP PROSC	1.68E-02	0.0000	MM0_Mo
2	CYTO	W5NUE3	PRDX1	8.76E-04	0.0000	MM0_Mo
2	CYTO	W5P7R2	HECTD1	1.92E-02	0.0000	MM0_Mo
2	CYTO	W5Q5B5	CSAD	1.72E-03	0.0000	MM0_Mo
2	CYTO	W5PWF2	ATP6V1H	1.05E-03	0.0000	MM0_Mo
2	CYTO	W5Q430	IDUA	1.72E-03	0.0002	MM0_Mo
2	CYTO	W5P5H4	AARS AARS1	3.16E-03	0.0000	MM0_Mo
2	CYTO	W5NSS1	PPP4R1	2.16E-03	0.0000	MM0_Mo
2	CYTO	W5Q740	ABCD3	3.15E-02	0.0000	MM0_Mo
2	CYTO	W5QEU6	ANXA4	3.26E-02	0.0000	MM0_Mo
2	CYTO	W5PMH1	GSS	6.29E-03	0.0000	MM0_Mo
2	CYTO	W5PHJ3	AHR	1.98E-02	0.0000	MM0_Mo
2	CYTO	W5QGX4	P3H1	1.24E-03	0.0000	MM0_Mo
2	CYTO	W5PEK7	FMNL2	2.02E-02	0.0000	MM0_Mo
2	CYTO	W5Q8J8	VPS41	2.05E-03	0.0000	MM0_Mo

Supplementary Table 7: Shared and unique differentially abundant proteins (DAPs) between MMØ and GMØ relative to M0 and their cellular location.

FoldChange	Location	Protein IDs	Gene Names	P-value	FDR	Cell type
2	CYTO	W5P985	ABHD14B	8.17E-03	0.0000	MM0_Mo
2	CYTO	W5QJ49	ATP6V1D	1.18E-03	0.0000	MM0_Mo
2	CYTO	W5QDY5	ATP6V1A	1.04E-03	0.0000	MM0_Mo
2	CYTO	W5PZS4	OSBPL8	3.32E-02	0.0000	MM0_Mo
2	CYTO	W5QAK7	SCARB1	4.55E-02	0.0000	MM0_Mo
2	CYTO	W5QIK3	USP8	1.78E-02	0.0000	MM0_Mo
2	CYTO	W5P5A7	VPS16	1.08E-03	0.0000	MM0_Mo
2	CYTO	W5QC34	MAN2A1	4.82E-03	0.0000	MM0_Mo
2	CYTO	W5Q3P7	ITFG1	4.02E-02	0.0004	MM0_Mo
2	CYTO	W5PK68	FEZ2	3.32E-02	0.0010	MM0_Mo
2	CYTO	W5Q9H8	SNX5	2.01E-02	0.0000	MM0_Mo
2	CYTO	W5QDF2	ATG3	2.28E-02	0.0000	MM0_Mo
2	CYTO	W5P8F1	HEATR5A	2.63E-02	0.0000	MM0_Mo
2	CYTO	W5Q021	FAM20A	3.85E-02	0.0000	MM0_Mo
2	CYTO	W5PSD7	RAP2C	1.50E-02	0.0000	MM0_Mo
2	CYTO	W5PIS1	FAM91A1	8.43E-03	0.0000	MM0_Mo
2	CYTO	W5PA78	AVL9	3.14E-02	0.0000	MM0_Mo
2	CYTO	W5P7P6	MYO18A	2.56E-03	0.0000	MM0_Mo
2	CYTO	W5P316	NAMPT	1.02E-02	0.0000	MM0_Mo
2	CYTO	W5Q6T1	ARSB	8.89E-03	0.0000	MM0_Mo
2	CYTO	W5PB21	PLA2R1	3.55E-02	0.0000	MM0_Mo
2	CYTO	W5NUE1	NCF2	5.74E-03	0.0000	MM0_Mo
2	CYTO	W5Q8I6	POLDIP2	2.67E-02	0.0000	MM0_Mo
2	CYTO	W5PE73	SMPDL3A	1.28E-02	0.0000	MM0_Mo
2	CYTO	W5P3U4	COMMD2	7.65E-03	0.0000	MM0_Mo
2	CYTO	W5PMB1	SNX3	4.18E-04	0.0000	MM0_Mo
2	CYTO	W5PGC9	MMAA	5.47E-03	0.0000	MM0_Mo
2	CYTO	W5PXT9	DENND4C	6.52E-03	0.0000	MM0_Mo
2	CYTO	W5PTI6	ST3GAL4	2.95E-02	0.0000	MM0_Mo
2	CYTO	W5P4L4	TMX3	2.04E-02	0.0000	MM0_Mo
2	CYTO	W5PWZ2		7.01E-03	0.0000	MM0_Mo
2	CYTO	W5PSX7	FES	1.59E-02	0.0000	MM0_Mo
2	CYTO	W5Q6V7	SIPA1	1.41E-02	0.0000	MM0_Mo
2	CYTO	W5P363	VAT1	1.53E-02	0.0000	MM0_Mo
2	CYTO	W5P0F8	LOC101103804	2.12E-02	0.0002	MM0_Mo
2	CYTO	W5Q411	HSPA13	1.61E-02	0.0000	MM0_Mo
2	CYTO	W5P6C1	ADSS ADSS2	1.36E-02	0.0000	MM0_Mo
2	CYTO	W5NZZ3	ATP6V1F	8.14E-04	0.0000	MM0_Mo
2	CYTO	W5PRJ7	CCDC93	1.64E-02	0.0000	MM0_Mo
2	CYTO	W5P8Y0	ITGAL	1.20E-02	0.0000	MM0_Mo
2	CYTO	W5P3H9	PICALM	1.40E-04	0.0000	MM0_Mo
2	CYTO	W5QG24	PPT1	2.27E-03	0.0000	MM0_Mo
2	CYTO	W5PC18		2.06E-02	0.0000	MM0_Mo
2	CYTO	W5PWV5	WDR81	8.11E-03	0.0000	MM0_Mo
2	CYTO	W5PSF8	CRYZ	4.91E-02	0.0000	MM0_Mo
2	CYTO	W5PA83	PRR14	3.70E-03	0.0045	MM0_Mo
2	CYTO	W5Q0G8	IMPA1	8.63E-03	0.0000	MM0_Mo
2	CYTO	W5PWQ7	AIDA	5.54E-02	0.0000	MM0_Mo
2	CYTO	W5Q3C1	NDRG3	2.18E-03	0.0000	MM0_Mo
2	CYTO	W5NQG1	SETD3	6.85E-03	0.0000	MM0_Mo
2	CYTO	W5PRK6	STAU1	3.20E-02	0.0000	MM0_Mo
2	CYTO	W5PW16	NEK6	4.95E-02	0.0000	MM0_Mo
2	CYTO	W5PVS3	ANKRD17	2.10E-03	0.0000	MM0_Mo
2	CYTO	W5NSE1	DRG2	9.66E-03	0.0000	MM0_Mo
2	CYTO	W5PZ05		3.07E-04	0.0000	MM0_Mo
2	CYTO	W5Q9Z0	ARFIP2	1.14E-02	0.0000	MM0_Mo
2	CYTO	W5P164	LAMTOR1	3.14E-02	0.0000	MM0_Mo
2	CYTO	W5PVC1	SYNJ1	5.67E-03	0.0000	MM0_Mo
2	CYTO	W5Q8T1	CLPX	5.56E-02	0.0000	MM0_Mo
2	CYTO	W5PG07	TPCN1	2.99E-02	0.0000	MM0_Mo
2	CYTO	W5PQT3	LOC101105107	2.53E-02	0.0000	MM0_Mo
2	CYTO	W5P0L9	MAPK3	2.69E-03	0.0000	MM0_Mo

Supplementary Table 7: Shared and unique differentially abundant proteins (DAPs) between MMØ and GMØ relative to M0 and their cellular location.

FoldChange	Location	Protein IDs	Gene Names	P-value	FDR	Cell type
2	CYTO	W5P7B1	SIRT2	1.38E-02	0.0000	MM0_Mo
2	CYTO	W5PMY4	GABARAP	3.44E-02	0.0000	MM0_Mo
2	CYTO	W5PPJ2	VPS33A	7.39E-03	0.0000	MM0_Mo
2	CYTO	W5PEL8	PDXDC1	3.00E-02	0.0000	MM0_Mo
2	CYTO	W5QG63	ZMPSTE24	4.66E-02	0.0000	MM0_Mo
2	CYTO	W5Q540	HAGH	3.42E-02	0.0000	MM0_Mo
2	CYTO	W5P1K2	TRAPPC9	2.30E-02	0.0000	MM0_Mo
2	CYTO	W5PDR6	EXOC7	2.11E-02	0.0000	MM0_Mo
2	CYTO	W5Q501	LOC101115640	6.11E-03	0.0000	MM0_Mo
2	CYTO	W5P7E8		9.06E-05	0.0002	MM0_Mo
2	CYTO	W5QI21	TARS2	4.42E-02	0.0000	MM0_Mo
2	CYTO	W5Q3D8	CLASP2	5.58E-02	0.0000	MM0_Mo
2	CYTO	W5PSE3	SH3TC1	2.43E-02	0.0002	MM0_Mo
2	CYTO	W5P180		2.53E-02	0.0000	MM0_Mo
2	CYTO	W5PVU5	PSMB7	8.26E-03	0.0000	MM0_Mo
2	CYTO	W5NQ97		2.21E-02	0.0000	MM0_Mo
2	CYTO	W5PBQ8	ODR4	3.62E-02	0.0000	MM0_Mo
2	CYTO	W5PDU8	GARS1	6.33E-04	0.0000	MM0_Mo
2	CYTO	W5Q922	LOC101105383	1.32E-03	0.0000	MM0_Mo
2	CYTO	W5QHA0	AGFG1	9.04E-04	0.0000	MM0_Mo
2	CYTO	W5PIC9		4.95E-03	0.0000	MM0_Mo
2	CYTO	W5Q2L9		4.80E-02	0.0000	MM0_Mo
2	CYTO	W5QGN5	DLG1	1.44E-04	0.0000	MM0_Mo
2	CYTO	W5PG60	SLC39A7	3.14E-02	0.0000	MM0_Mo
2	CYTO	W5P7H5	ADGRE5	4.69E-03	0.0000	MM0_Mo
2	CYTO	W5PJY0	EMC1	4.13E-02	0.0000	MM0_Mo
2	CYTO	W5PDN7	GPD2	7.75E-03	0.0000	MM0_Mo
2	CYTO	W5PQ47	RAI14	2.79E-03	0.0000	MM0_Mo
2	CYTO	W5QCD2	ADPGK	4.95E-03	0.0000	MM0_Mo
2	CYTO	W5QIM0	GALK2	3.31E-02	0.0000	MM0_Mo
2	CYTO	W5Q0N8	ADAM17	6.96E-03	0.0000	MM0_Mo
2	CYTO	W5PA89	ST6GALNAC2	1.13E-02	0.0021	MM0_Mo
2	CYTO	W5P001	RUFY1	3.89E-03	0.0000	MM0_Mo
2	CYTO	C8BKE1	STAT1 STAT4	2.56E-03	0.0000	MM0_Mo
2	CYTO	W5PYE8	MERTK	1.96E-02	0.0000	MM0_Mo
2	CYTO	W5QDT8		4.91E-02	0.0000	MM0_Mo
2	CYTO	W5P4G4	RAB7B	2.53E-03	0.0000	MM0_Mo
2	CYTO	W5Q7T6	AACS	4.92E-02	0.0000	MM0_Mo
2	CYTO	W5PSB2	TBL2	1.35E-02	0.0000	MM0_Mo
2	CYTO	W5QE35	IPO8	5.08E-02	0.0000	MM0_Mo
2	CYTO	W5Q045	TMED3	1.25E-02	0.0000	MM0_Mo
2	CYTO	W5PS05	MTHFD2	3.98E-03	0.0000	MM0_Mo
2	CYTO	W5Q1D8	GALNT7	1.25E-02	0.0002	MM0_Mo
2	CYTO	Q6ECI6	ITGB2 CD18	4.75E-04	0.0000	MM0_Mo
2	CYTO	W5NZJ1	LOC101114075	5.15E-02	0.0000	MM0_Mo
2	CYTO	W5PZM9	ANXA5	3.91E-04	0.0000	MM0_Mo
2	CYTO	W5PTB7	ATG7	5.23E-05	0.0000	MM0_Mo
2	CYTO	W5P5S5	EMC3	4.04E-02	0.0000	MM0_Mo
2	CYTO	W5Q0M7	RAP2B	2.17E-03	0.0000	MM0_Mo
2	CYTO	W5PDX8	FAM120A	1.18E-02	0.0000	MM0_Mo
2	CYTO	W5P7N7	IQGAP3	4.53E-02	0.0000	MM0_Mo
2	CYTO	W5PQ56	RUFY3	4.57E-03	0.0000	MM0_Mo
2	CYTO	W5QJ45	GPHN	2.82E-02	0.0000	MM0_Mo
2	CYTO	W5PD54	NCK1	1.09E-02	0.0000	MM0_Mo
2	CYTO	Q9GMC9	TSPO BZRP	4.01E-02	0.0002	MM0_Mo
2	CYTO	A2SW69	ANXA2 ANX2	4.69E-03	0.0000	MM0_Mo
2	CYTO	C5HK62	EYA3	3.16E-02	0.0000	MM0_Mo
2	CYTO	W5PUU2	RRAS	3.16E-02	0.0000	MM0_Mo
2	CYTO	W5PVL6	AP2A1	6.93E-03	0.0000	MM0_Mo
2	CYTO	W5P9Q1	ARHGEF11	1.68E-02	0.0000	MM0_Mo
2	CYTO	W5Q7I9	HPS6	1.04E-02	0.0000	MM0_Mo
2	CYTO	W5Q0B4	KYAT3	2.31E-02	0.0002	MM0_Mo

Supplementary Table 7: Shared and unique differentially abundant proteins (DAPs) between MMØ and GMØ relative to M0 and their cellular location.

FoldChange	Location	Protein IDs	Gene Names	P-value	FDR	Cell type
2	CYTO	W5PR93	SLC46A3	1.70E-02	0.0000	MM0_Mo
2	CYTO	W5Q289	ATP2B1	6.18E-03	0.0000	MM0_Mo
2	CYTO	W5QDJ5	LOC101122319	5.32E-02	0.0000	MM0_Mo
2	CYTO	W5PF94	TOR1A	6.03E-03	0.0002	MM0_Mo
2	CYTO	W5NTM7	DENND10	1.21E-03	0.0000	MM0_Mo
2	CYTO	W5PLB6	SPART	7.52E-03	0.0000	MM0_Mo
2	CYTO	W5PCI1	NLRP3	5.12E-02	0.0000	MM0_Mo
2	CYTO	Q8HY31	UROD	1.41E-02	0.0000	MM0_Mo
2	CYTO	W5P2Q3	RPS6KA1	7.58E-03	0.0000	MM0_Mo
2	CYTO	W5Q4S7	SLC27A1	4.20E-02	0.0000	MM0_Mo
2	CYTO	W5QGJ2	CHP1	4.11E-02	0.0000	MM0_Mo
2	CYTO	W5Q0Q2		7.54E-04	0.0000	MM0_Mo
2	CYTO	W5NSV0	LTBP2	3.79E-02	0.0000	MM0_Mo
2	CYTO	W5P929	MARS1	7.18E-03	0.0000	MM0_Mo
2	CYTO	W5PD41	GIT1	5.04E-03	0.0000	MM0_Mo
2	CYTO	W5NYP8	DYNC1H1	3.41E-05	0.0000	MM0_Mo
2	CYTO	W5Q738	TMEM173 STING1	1.36E-03	0.0000	MM0_Mo
2	CYTO	W5P207		1.84E-03	0.0000	MM0_Mo
2	CYTO	W5PP30	ASNA1 GET3	3.39E-04	0.0000	MM0_Mo
2	CYTO	O78755	MT-ND4 MTND4 NAI	3.11E-02	0.0000	MM0_Mo
2	CYTO	W5PGJ4	CHN2	1.33E-02	0.0004	MM0_Mo
2	CYTO	W5P9Y1	SNX6	2.72E-02	0.0000	MM0_Mo
2	CYTO	W5P089	LOC101115969	4.87E-02	0.0000	MM0_Mo
2	CYTO	W5PVC2	CDC37L1	4.14E-03	0.0000	MM0_Mo
2	CYTO	W5PL73	CCDC22	1.81E-03	0.0000	MM0_Mo
2	CYTO	W5QBJ0	ANXA3	3.49E-02	0.0000	MM0_Mo
2	CYTO	W5Q7J2	AGPS	5.48E-02	0.0000	MM0_Mo
2	CYTO	W5PXD7	LAMTOR3	1.45E-04	0.0000	MM0_Mo
2	CYTO	W5PUF8	PITRM1	3.63E-02	0.0000	MM0_Mo
2	CYTO	W5Q353	TACC3	7.36E-03	0.0000	MM0_Mo
2	CYTO	W5QHF0	ABCF3	4.04E-02	0.0000	MM0_Mo
2	CYTO	W5PCA0	ALDOB	4.00E-02	0.0000	MM0_Mo
2	CYTO	W5QHT6	NCEH1	7.03E-03	0.0000	MM0_Mo
2	CYTO	W5P2M5	LOC101114535	1.58E-02	0.0025	MM0_Mo
2	CYTO	W5QFN9	UGP2	2.41E-03	0.0000	MM0_Mo
2	CYTO	W5PD62	CPB2	1.83E-02	0.0091	MM0_Mo
-2	CYTO	W5Q1G5		5.39E-02	0.0000	MM0_Mo
-2	CYTO	W5PVX8	PPP3CA	7.01E-03	0.0000	MM0_Mo
-2	CYTO	W5PXM3	PSPC1	5.20E-02	0.0000	MM0_Mo
-2	CYTO	W5Q198	QRICH1	2.97E-02	0.0002	MM0_Mo
-2	CYTO	W5PX94	CELF2	4.20E-02	0.0000	MM0_Mo
-2	CYTO	W5NW82	NAP1L4	6.09E-03	0.0000	MM0_Mo
-2	CYTO	W5PT49	NFKB1	3.80E-02	0.0000	MM0_Mo
-2	CYTO	W5QD48	TAGLN3	1.22E-02	0.0000	MM0_Mo
-2	CYTO	W5PAI1		1.01E-02	0.0000	MM0_Mo
-2	CYTO	W5NSI2	RALBP1	4.99E-02	0.0085	MM0_Mo
-2	CYTO	W5PJ14	ESYT2	2.54E-02	0.0000	MM0_Mo
-2	CYTO	W5P098	CORO1A	6.33E-04	0.0000	MM0_Mo
-2	CYTO	W5P440	HERC4	2.41E-02	0.0000	MM0_Mo
-2	CYTO	W5PEE3	FNBP1	8.66E-03	0.0000	MM0_Mo
-2	CYTO	W5PHB6	SF1	4.85E-02	0.0000	MM0_Mo
-2	CYTO	W5NWQ0	TMED8	4.05E-02	0.0000	MM0_Mo
-2	CYTO	W5PVI9	STK38	4.65E-02	0.0000	MM0_Mo
-2	CYTO	W5PUT2	FUBP1	5.34E-02	0.0000	MM0_Mo
-2	CYTO	W5Q2Y3	PLEKHB2	3.38E-02	0.0000	MM0_Mo
-2	CYTO	W5PVY6		3.83E-02	0.0000	MM0_Mo
-2	CYTO	W5Q8F8	CPOX	4.13E-02	0.0000	MM0_Mo
-2	CYTO	W5PYM3	CAAP1	1.18E-02	0.0012	MM0_Mo
-2	CYTO	W5P340	SOD2	2.32E-02	0.0000	MM0_Mo
-2	CYTO	W5QHP6	ARHGDIB	1.86E-05	0.0000	MM0_Mo
-2	CYTO	W5PK02	POSTN	4.83E-02	0.0000	MM0_Mo
-2	CYTO	W5QCZ0	MPST	2.49E-04	0.0000	MM0_Mo

Supplementary Table 7: Shared and unique differentially abundant proteins (DAPs) between MMØ and GMØ relative to M0 and their cellular location.

FoldChange	Location	Protein IDs	Gene Names	P-value	FDR	Cell type
-2	CYTO	W5QC32	CHERP	5.25E-02	0.0000	MM0_Mo
-2	CYTO	W5PL66	PPA2	1.10E-02	0.0000	MM0_Mo
-2	CYTO	W5PWH9	PRKACB	9.69E-04	0.0000	MM0_Mo
-2	CYTO	W5PML4	RCC2	8.13E-03	0.0000	MM0_Mo
-2	CYTO	W5NX11	EPB41	3.11E-02	0.0000	MM0_Mo
-2	CYTO	W5PVC3	LOC101117851	1.90E-02	0.0002	MM0_Mo
-2	CYTO	W5PXS7	DAPP1	9.84E-03	0.0000	MM0_Mo
-2	CYTO	W5QF71	PLEK	2.96E-02	0.0000	MM0_Mo
-2	CYTO	W5PIC3		4.02E-03	0.0000	MM0_Mo
-2	CYTO	Q2TCH3	ACLY	5.78E-04	0.0000	MM0_Mo
-2	CYTO	W5PR48	HPRT1	2.85E-02	0.0000	MM0_Mo
-2	CYTO	W5P5N5		1.05E-02	0.0002	MM0_Mo
-2	CYTO	W5P4E2	PBLD	1.99E-02	0.0000	MM0_Mo
-2	CYTO	W5PXJ6	ANKRD13A	3.50E-03	0.0000	MM0_Mo
-2	CYTO	W5QIC3	PRUNE1	3.16E-04	0.0000	MM0_Mo
-2	CYTO	W5QI77	AK2	4.27E-03	0.0000	MM0_Mo
-2	CYTO	W5NVL4		2.60E-02	0.0000	MM0_Mo
-2	CYTO	W5P1E2	CNST	9.04E-04	0.0000	MM0_Mo
-2	CYTO	W5PHI7	LOC101116892	2.62E-02	0.0000	MM0_Mo
-2	CYTO	W5Q2W6	PPFIBP2	4.33E-02	0.0002	MM0_Mo
-2	CYTO	W5PIU9	ARMC6	3.67E-02	0.0000	MM0_Mo
-2	CYTO	W5PHE6	DDI2	5.05E-03	0.0000	MM0_Mo
-2	CYTO	W5QHL0	ATP11B	8.93E-03	0.0000	MM0_Mo
-2	CYTO	W5PDQ3	SAFB2	2.11E-02	0.0000	MM0_Mo
-2	CYTO	W5NQ72	PFAS	2.79E-03	0.0000	MM0_Mo
-2	CYTO	W5QFQ8	LOC101111915	1.32E-02	0.0000	MM0_Mo
-2	CYTO	W5P8J8		5.61E-02	0.0000	MM0_Mo
-2	CYTO	W5P5M9		6.34E-03	0.0000	MM0_Mo
-2	CYTO	W5PV48	USP11	2.51E-02	0.0000	MM0_Mo
-2	CYTO	W5Q0B6	PPP1R12A	2.90E-03	0.0000	MM0_Mo
-2	CYTO	W5PDB0	TRIM58	2.33E-02	0.0000	MM0_Mo
-2	CYTO	W5Q595	NIF3L1	3.32E-02	0.0000	MM0_Mo
-2	CYTO	W5Q3M9	SEPTIN6	1.94E-02	0.0000	MM0_Mo
-2	CYTO	W5NV08	PUM1	4.94E-02	0.0000	MM0_Mo
-2	CYTO	W5P4C5	RBM42	1.17E-02	0.0000	MM0_Mo
-2	CYTO	W5PXG3		1.09E-02	0.0015	MM0_Mo
-2	CYTO	W5NVG4	SMC4	3.44E-02	0.0000	MM0_Mo
-2	CYTO	W5P7C2	SEPHS1	3.00E-02	0.0000	MM0_Mo
-2	CYTO	W5NRJ3		1.95E-02	0.0000	MM0_Mo
-2	CYTO	W5PRJ3	HYCC1	4.12E-02	0.0000	MM0_Mo
-2	CYTO	W5PWZ9	RAMAC	5.02E-02	0.0043	MM0_Mo
-2	CYTO	C8BKD4	CBX5	2.54E-02	0.0000	MM0_Mo
-2	CYTO	W5Q366	SUN2	1.76E-02	0.0000	MM0_Mo
-2	CYTO	W5QGB5	LOC101113369	6.36E-03	0.0000	MM0_Mo
-2	CYTO	W5Q3Q2	ERCC3	1.00E-02	0.0041	MM0_Mo
-2	CYTO	W5QIH2	TMOD3	3.60E-03	0.0000	MM0_Mo
-2	CYTO	W5QB24	ANP32A	1.83E-02	0.0000	MM0_Mo
-2	CYTO	C8BKC5	PRDX2	2.32E-02	0.0000	MM0_Mo
-2	CYTO	W5P059	TERF2	3.93E-02	0.0002	MM0_Mo
-2	CYTO	W5NUT8	PIP4K2A	1.53E-02	0.0000	MM0_Mo
-2	CYTO	W5PMC9	ARHGAP6	1.85E-02	0.0071	MM0_Mo
-2	CYTO	W5PW66	LANCL2	3.24E-02	0.0000	MM0_Mo
-2	CYTO	W5PAV5	RFX1	4.60E-02	0.0000	MM0_Mo
-2	CYTO	W5NS43	ALDH6A1	1.11E-03	0.0000	MM0_Mo
-2	CYTO	W5PLK6	SHOC2	1.02E-02	0.0002	MM0_Mo
-2	CYTO	W5PUR1	CNOT11	8.67E-05	0.0000	MM0_Mo
-2	CYTO	W5P5Y0	AGAP2	4.79E-02	0.0000	MM0_Mo
-2	CYTO	W5PFJ5	SMC1A	2.61E-02	0.0000	MM0_Mo
-2	CYTO	W5NRR9	STAT5A	1.60E-02	0.0000	MM0_Mo
-2	CYTO	W5NQH2	CHMP6	4.65E-02	0.0000	MM0_Mo
-2	CYTO	W5PVD4	AK3	2.21E-03	0.0000	MM0_Mo
-2	CYTO	W5PY64	LOC101106288	2.02E-02	0.0000	MM0_Mo

Supplementary Table 7: Shared and unique differentially abundant proteins (DAPs) between MMØ and GMØ relative to M0 and their cellular location.

FoldChange	Location	Protein IDs	Gene Names	P-value	FDR	Cell type
-2	CYTO	W5PUS0	CUL4B	4.86E-04	0.0000	MM0_Mo
-2	CYTO	W5PQ04	DEF6	2.13E-02	0.0000	MM0_Mo
-2	CYTO	W5PTS4	LOC101114275	1.50E-02	0.0000	MM0_Mo
-2	CYTO	W5QCG0		4.68E-02	0.0000	MM0_Mo
-2	CYTO	W5PWS1	CD46	2.69E-02	0.0000	MM0_Mo
-2	CYTO	W5Q5Y1	ITGA4	2.19E-03	0.0002	MM0_Mo
-2	CYTO	W5P0J5	ZNF22	4.20E-02	0.0002	MM0_Mo
-2	CYTO	W5Q9R2	SARM1	5.23E-02	0.0000	MM0_Mo
-2	CYTO	W5P0D9		2.52E-02	0.0002	MM0_Mo
-2	CYTO	W5QIK2	RFX5	1.95E-02	0.0000	MM0_Mo
-2	CYTO	W5PLD5	CNN2	2.90E-03	0.0000	MM0_Mo
-2	CYTO	W5PLX5	PRKD2	1.24E-02	0.0000	MM0_Mo
-2	CYTO	W5Q1F6	AIFM1	4.92E-02	0.0000	MM0_Mo
-2	CYTO	W5P225	PDCD5	3.25E-02	0.0000	MM0_Mo
-2	CYTO	W5QJ62	ACTN1	1.96E-02	0.0000	MM0_Mo
-2	CYTO	W5Q1B3	HMGB2	6.49E-03	0.0000	MM0_Mo
-2	CYTO	W5QHY6	RPIA	2.55E-03	0.0000	MM0_Mo
-2	CYTO	W5QFT3		4.26E-02	0.0000	MM0_Mo
-2	CYTO	W5PUQ5	CLASP1	5.61E-02	0.0000	MM0_Mo
-2	CYTO	W5PRP1	SLC9A3R1	3.06E-04	0.0000	MM0_Mo
-2	CYTO	W5Q7Q6	EPHX1	5.59E-02	0.0000	MM0_Mo
-2	CYTO	W5QIY4	ARID4A	7.55E-03	0.0000	MM0_Mo
-2	CYTO	W5NTA4	CGGBP1	2.52E-02	0.0000	MM0_Mo
-2	CYTO	W5PIG5	ABCC1	2.86E-02	0.0000	MM0_Mo
-2	CYTO	W5Q9U3	CNP	9.94E-04	0.0000	MM0_Mo
-2	CYTO	W5QEH9	THRAP3	2.95E-02	0.0000	MM0_Mo
-2	CYTO	W5PAF9	KIF2A	2.49E-02	0.0000	MM0_Mo
-2	CYTO	W5P9F0	ARHGAP9	2.84E-02	0.0000	MM0_Mo
-2	CYTO	W5P2J9		1.60E-02	0.0000	MM0_Mo
-2	CYTO	W5QBV1	FER	1.01E-02	0.0000	MM0_Mo
-2	CYTO	W5P433	BRCA1	1.81E-02	0.0037	MM0_Mo
-2	CYTO	W5PEG1	CETN2	5.40E-02	0.0000	MM0_Mo
-2	CYTO	W5Q088	CBL	2.62E-03	0.0000	MM0_Mo
-2	CYTO	W5PCG2	TAP2	2.68E-02	0.0000	MM0_Mo
-2	CYTO	B2LU20	SFXN1	5.52E-02	0.0000	MM0_Mo
-2	CYTO	W5PNJ5	SARNP	2.68E-03	0.0000	MM0_Mo
-2	CYTO	W5PCD9	EML4	4.54E-03	0.0000	MM0_Mo
-2	CYTO	W5Q2C6		5.06E-02	0.0002	MM0_Mo
-3	CYTO	W5PSX3	DARS2	6.21E-03	0.0002	MM0_Mo
-3	CYTO	W5QCS1	DDX23	1.68E-02	0.0000	MM0_Mo
-3	CYTO	W5PM99		1.00E-02	0.0000	MM0_Mo
-3	CYTO	W5QEP1	POGLUT1	3.51E-02	0.0000	MM0_Mo
-3	CYTO	W5QHX6	ANP32E	3.27E-02	0.0000	MM0_Mo
-3	CYTO	W5PTQ0	RNGTT	4.58E-02	0.0002	MM0_Mo
-3	CYTO	W5PW39	LMNB2	2.05E-02	0.0000	MM0_Mo
-3	CYTO	W5P0E3	BRD4	5.31E-02	0.0000	MM0_Mo
-3	CYTO	W5QEQ3	APEX1	4.47E-02	0.0000	MM0_Mo
-3	CYTO	W5Q297	PRKCA	1.12E-02	0.0000	MM0_Mo
-3	CYTO	W5QB58	AASS	3.37E-04	0.0000	MM0_Mo
-3	CYTO	W5PNV3	AK1	2.37E-02	0.0000	MM0_Mo
-3	CYTO	W5Q824	GSTA1	4.85E-02	0.0000	MM0_Mo
-3	CYTO	W5PI11	TAPBP	4.25E-02	0.0000	MM0_Mo
-3	CYTO	W5NUX2		1.20E-02	0.0000	MM0_Mo
-3	CYTO	W5PJ11		1.58E-02	0.0002	MM0_Mo
-3	CYTO	W5PSQ7		5.14E-03	0.0000	MM0_Mo
-3	CYTO	W5Q8P3	USE1	3.58E-03	0.0000	MM0_Mo
-3	CYTO	W5P4F9	PRKAR2B	2.85E-02	0.0000	MM0_Mo
-3	CYTO	W5PTM9	UBE2V2	2.19E-04	0.0000	MM0_Mo
-3	CYTO	W5P7A6	ATE1	8.23E-03	0.0000	MM0_Mo
-3	CYTO	W5PRR5	LTA4H	1.79E-04	0.0000	MM0_Mo
-3	CYTO	W5Q4G2		7.70E-03	0.0000	MM0_Mo
-3	CYTO	W5P671	DDAH2	1.34E-02	0.0095	MM0_Mo

Supplementary Table 7: Shared and unique differentially abundant proteins (DAPs) between MMØ and GMØ relative to M0 and their cellular location.

FoldChange	Location	Protein IDs	Gene Names	P-value	FDR	Cell type
-3	CYTO	W5QHL6	ITM2C	1.14E-03	0.0000	MM0_Mo
-3	CYTO	W5NXE7	PPIL4	4.52E-02	0.0000	MM0_Mo
-3	CYTO	W5Q3E4	NAAA	3.70E-02	0.0000	MM0_Mo
-3	CYTO	W5PP80	FRY	3.05E-02	0.0012	MM0_Mo
-3	CYTO	W5PTM6	CPSF2	1.01E-02	0.0000	MM0_Mo
-3	CYTO	W5PMQ1	ZC3H4	3.93E-02	0.0000	MM0_Mo
-3	CYTO	W5PWV6	UBE2B	4.17E-02	0.0000	MM0_Mo
-3	CYTO	W5QET9	PLA1A	3.76E-03	0.0000	MM0_Mo
-3	CYTO	O78750	MT-CO2 COII COX2 C	1.19E-02	0.0000	MM0_Mo
-3	CYTO	W5QD23	LANCL1	1.33E-02	0.0000	MM0_Mo
-3	CYTO	W5PKD2	GCDH	3.38E-02	0.0000	MM0_Mo
-3	CYTO	W5PKY9	MRCL3	4.78E-02	0.0000	MM0_Mo
-3	CYTO	C5IWU4	ARL3	3.18E-02	0.0000	MM0_Mo
-3	CYTO	W5PEQ9	SRSF1	3.26E-02	0.0000	MM0_Mo
-3	CYTO	W5PK38	VASP	2.32E-02	0.0000	MM0_Mo
-3	CYTO	W5QI35	IL1RN	4.90E-02	0.0000	MM0_Mo
-3	CYTO	W5PJF3	PPIF	3.29E-02	0.0002	MM0_Mo
-3	CYTO	W5P6V7	DDX39A	2.47E-03	0.0000	MM0_Mo
-3	CYTO	W5PF18	LOC101102454	5.04E-02	0.0000	MM0_Mo
-3	CYTO	W5NRI1		1.83E-03	0.0002	MM0_Mo
-3	CYTO	W5Q9B6	INTS14	4.93E-02	0.0010	MM0_Mo
-3	CYTO	W5PEA0	FGD3	1.25E-04	0.0000	MM0_Mo
-3	CYTO	W5P8I8	PITPNM2	2.20E-03	0.0000	MM0_Mo
-3	CYTO	W5Q0P0	GABPA	2.73E-02	0.0000	MM0_Mo
-3	CYTO	W5PI65	MAP4K2	1.38E-04	0.0000	MM0_Mo
-3	CYTO	W5PQT2	TPRKB	3.47E-02	0.0000	MM0_Mo
-3	CYTO	W5PBZ1	PCIF1	2.68E-02	0.0000	MM0_Mo
-3	CYTO	W5PAV0	STAP1	2.65E-02	0.0000	MM0_Mo
-3	CYTO	W5P0N1	DHX38	3.78E-03	0.0010	MM0_Mo
-3	CYTO	W5PIW6	PRTN3	4.61E-02	0.0000	MM0_Mo
-3	CYTO	W5PUI4	RAB3A	1.84E-03	0.0002	MM0_Mo
-3	CYTO	W5NTV6		1.86E-03	0.0000	MM0_Mo
-3	CYTO	W5PTN4	TMPO	4.22E-03	0.0000	MM0_Mo
-3	CYTO	W5Q7L1	CNN3	1.18E-02	0.0000	MM0_Mo
-3	CYTO	W5NTW9	RIPK1	2.18E-02	0.0000	MM0_Mo
-3	CYTO	W5PCJ3	LOC101112936	1.23E-02	0.0000	MM0_Mo
-3	CYTO	W5PZL2		3.97E-02	0.0000	MM0_Mo
-3	CYTO	W5PKS6	DEK	4.23E-02	0.0000	MM0_Mo
-3	CYTO	W5PAF7	RSL1D1	3.86E-02	0.0000	MM0_Mo
-3	CYTO	W5P7K2		4.22E-02	0.0000	MM0_Mo
-3	CYTO	W5Q8H0	SSH1	2.87E-02	0.0000	MM0_Mo
-3	CYTO	W5P8N5	FBL	4.32E-02	0.0000	MM0_Mo
-3	CYTO	C5ISA2	TUBA4A	1.43E-02	0.0000	MM0_Mo
-3	CYTO	W5PDE5	LOC101120001	5.15E-03	0.0000	MM0_Mo
-3	CYTO	W5P0R4	CTIF	7.24E-05	0.0004	MM0_Mo
-3	CYTO	W5PA22	ACOT8	2.03E-03	0.0000	MM0_Mo
-3	CYTO	W5PVX3	PRKG1	4.53E-02	0.0000	MM0_Mo
-3	CYTO	W5QEC3	METTL3	1.85E-02	0.0000	MM0_Mo
-3	CYTO	W5PHI5	PALM	6.56E-03	0.0000	MM0_Mo
-3	CYTO	W5PV67	LIMD2	1.73E-02	0.0000	MM0_Mo
-3	CYTO	W5NRP8	MTCL1	9.37E-04	0.0008	MM0_Mo
-3	CYTO	W5Q2W4	RAVER1	4.19E-02	0.0000	MM0_Mo
-3	CYTO	W5PZU8	ASAP2	2.06E-02	0.0000	MM0_Mo
-3	CYTO	W5QGS4	PHGDH	2.20E-02	0.0000	MM0_Mo
-3	CYTO	W5PI68	TBCK	3.58E-02	0.0000	MM0_Mo
-3	CYTO	W5NQH6	S100A9	4.06E-02	0.0000	MM0_Mo
-3	CYTO	W5P7P8	GCA	5.91E-03	0.0000	MM0_Mo
-3	CYTO	W5PQD8	C1QTNF3	1.92E-03	0.0000	MM0_Mo
-3	CYTO	W5PP85	TBC1D10C	1.57E-02	0.0000	MM0_Mo
-3	CYTO	W5PTB3	INPP5K	1.11E-03	0.0002	MM0_Mo
-3	CYTO	W5P7E0	SARS2	3.56E-02	0.0000	MM0_Mo
-3	CYTO	W5NTQ3	H3-4	7.53E-05	0.0002	MM0_Mo

Supplementary Table 7: Shared and unique differentially abundant proteins (DAPs) between MMØ and GMØ relative to M0 and their cellular location.

FoldChange	Location	Protein IDs	Gene Names	P-value	FDR	Cell type
-3	CYTO	W5Q7P6	SMARCA4	4.62E-02	0.0000	MM0_Mo
-3	CYTO	W5P5K9	SRGN	1.84E-02	0.0002	MM0_Mo
-3	CYTO	W5PKK4	CCAR2	4.83E-02	0.0000	MM0_Mo
-3	CYTO	W5P795	LRBA	1.14E-02	0.0000	MM0_Mo
-3	CYTO	W5PG95	HSPA1A	5.03E-02	0.0000	MM0_Mo
-3	CYTO	W5P6Q7	MPIG6B	4.02E-02	0.0000	MM0_Mo
-3	CYTO	W5QIR2	C7H15orf48	4.26E-02	0.0000	MM0_Mo
-3	CYTO	W5PXR8	POLR2J	1.11E-02	0.0000	MM0_Mo
-3	CYTO	W5P7B8	MTMR3	1.57E-03	0.0000	MM0_Mo
-4	CYTO	W5PKA9	F5	5.50E-03	0.0000	MM0_Mo
-4	CYTO	W5PMJ7	NADK2	6.89E-03	0.0000	MM0_Mo
-4	CYTO	W5P8H4	DCTPP1	7.03E-04	0.0000	MM0_Mo
-4	CYTO	W5PDG9	LOC101109746	4.05E-02	0.0000	MM0_Mo
-4	CYTO	W5NWU0	LOC101122262	1.62E-02	0.0000	MM0_Mo
-4	CYTO	W5PG02	RASA3	6.30E-03	0.0000	MM0_Mo
-4	CYTO	W5PJP5	RGS18	4.49E-02	0.0000	MM0_Mo
-4	CYTO	W5P3V8		2.47E-02	0.0000	MM0_Mo
-4	CYTO	W5QDM7	ZNF512	3.76E-02	0.0000	MM0_Mo
-4	CYTO	W5Q1Z2		1.35E-02	0.0006	MM0_Mo
-4	CYTO	W5Q9H1	ZYX	2.57E-02	0.0000	MM0_Mo
-4	CYTO	W5QFM2		9.24E-05	0.0000	MM0_Mo
-4	CYTO	W5P9M9	LOC101103862	1.96E-02	0.0000	MM0_Mo
-4	CYTO	W5P6T0		2.23E-03	0.0012	MM0_Mo
-4	CYTO	P80190		4.36E-03	0.0000	MM0_Mo
-4	CYTO	W5P432	NIPSNAP1	5.99E-03	0.0000	MM0_Mo
-4	CYTO	W5Q5M7	SLC44A2	6.54E-03	0.0000	MM0_Mo
-4	CYTO	W5NQJ0	LOC101103771	2.42E-02	0.0000	MM0_Mo
-4	CYTO	W5PGU9	PLCG2	1.54E-04	0.0000	MM0_Mo
-4	CYTO	W5Q6S0	LBR	3.64E-03	0.0000	MM0_Mo
-4	CYTO	W5PBE0	CD74	3.70E-02	0.0000	MM0_Mo
-4	CYTO	W5QFP0	THBS1	2.48E-02	0.0000	MM0_Mo
-4	CYTO	W5QEK8	EHD3	6.91E-03	0.0000	MM0_Mo
-4	CYTO	W5PXT1	MYO1G	7.06E-03	0.0000	MM0_Mo
-4	CYTO	W5PX46	LOC101122591	1.72E-02	0.0000	MM0_Mo
-4	CYTO	W5Q550	LOC101109747	4.39E-02	0.0000	MM0_Mo
-4	CYTO	W5NV79	NUP210	1.87E-02	0.0000	MM0_Mo
-4	CYTO	W5Q2G2	IL16	4.83E-03	0.0000	MM0_Mo
-4	CYTO	W5NVA8	GP6	1.47E-02	0.0000	MM0_Mo
-4	CYTO	W5Q059	SASH3	6.30E-03	0.0000	MM0_Mo
-4	CYTO	W5PEC3	ANXA6	3.86E-02	0.0000	MM0_Mo
-4	CYTO	W5Q6F1		2.74E-02	0.0000	MM0_Mo
-4	CYTO	W5PKR2	MX1	1.09E-02	0.0000	MM0_Mo
-4	CYTO	W5QCH5	WDR47	8.38E-05	0.0002	MM0_Mo
-4	CYTO	W5PKQ2	PRXL2A	4.60E-02	0.0000	MM0_Mo
-4	CYTO	W5PC20	VPS37B	7.32E-03	0.0000	MM0_Mo
-4	CYTO	Q09YJ2	TES	3.08E-03	0.0000	MM0_Mo
-4	CYTO	W5QI61	CDKN1B	5.53E-04	0.0000	MM0_Mo
-4	CYTO	C0IZ95	RAB27A	4.23E-03	0.0000	MM0_Mo
-4	CYTO	W5P5Y4	RIPOR2	5.65E-03	0.0000	MM0_Mo
-4	CYTO	W5Q1G8	LOC101120875	2.73E-02	0.0000	MM0_Mo
-4	CYTO	W5P4U4	GRK6	4.39E-04	0.0000	MM0_Mo
-4	CYTO	W5Q2V1		2.47E-03	0.0000	MM0_Mo
-4	CYTO	W5PRY2	ACAP1	3.08E-02	0.0000	MM0_Mo
-4	CYTO	W5PA61		9.05E-03	0.0000	MM0_Mo
-4	CYTO	W5QEL7	NDRG2	3.28E-04	0.0078	MM0_Mo
-4	CYTO	W5P815	SEPTIN1	2.24E-02	0.0000	MM0_Mo
-4	CYTO	W5Q813	NOP58	5.45E-02	0.0000	MM0_Mo
-4	CYTO	W5NTL4	EVL	2.03E-02	0.0000	MM0_Mo
-5	CYTO	W5NYC7	DQA	8.59E-03	0.0000	MM0_Mo
-5	CYTO	P54230	CATHL1A BAC1A DOI	3.90E-02	0.0000	MM0_Mo
-5	CYTO	W5QHW2	DAPK2	3.28E-02	0.0000	MM0_Mo
-5	CYTO	W5QH43	EIF4A2	1.38E-02	0.0000	MM0_Mo

Supplementary Table 7: Shared and unique differentially abundant proteins (DAPs) between MMØ and GMØ relative to M0 and their cellular location.

FoldChange	Location	Protein IDs	Gene Names	P-value	FDR	Cell type
-5	CYTO	W5NR20	LOC101109940	2.70E-02	0.0099	MM0_Mo
-5	CYTO	W5PHM1	ALOX12	4.20E-02	0.0000	MM0_Mo
-5	CYTO	W5PFJ0	VCL	1.69E-02	0.0000	MM0_Mo
-5	CYTO	W5PU80	MS4A1	6.78E-03	0.0000	MM0_Mo
-5	CYTO	W5QGQ3	LOC443320	3.78E-02	0.0002	MM0_Mo
-5	CYTO	W5NSC5		2.33E-03	0.0000	MM0_Mo
-5	CYTO	W5PDP2	CD55	1.03E-03	0.0000	MM0_Mo
-5	CYTO	W5QFP2	LOC101109397	7.34E-03	0.0000	MM0_Mo
-5	CYTO	W5P733	PDLIM1	5.44E-02	0.0000	MM0_Mo
-5	CYTO	W5PIN7		8.33E-03	0.0000	MM0_Mo
-5	CYTO	W5NUN1	STX11	3.18E-02	0.0000	MM0_Mo
-5	CYTO	W5P3Y9	CD22	5.83E-03	0.0000	MM0_Mo
-5	CYTO	W5PTQ7	TRAF3IP3	1.73E-03	0.0000	MM0_Mo
-5	CYTO	P79362	CATHL2 BAC5	2.89E-02	0.0000	MM0_Mo
-5	CYTO	W5PV80	BANK1	1.90E-02	0.0000	MM0_Mo
-5	CYTO	W5PHR3	ADD3	2.65E-03	0.0000	MM0_Mo
-5	CYTO	W5PZG0	ADD1	2.45E-03	0.0000	MM0_Mo
-5	CYTO	W5P7W5	LOC101113516	1.23E-02	0.0000	MM0_Mo
-5	CYTO	W5PBW1	SDS	2.73E-03	0.0000	MM0_Mo
-5	CYTO	P47843	SLC2A3 GLUT3	1.15E-02	0.0000	MM0_Mo
-6	CYTO	W5P060	SYNE1	2.70E-02	0.0000	MM0_Mo
-6	CYTO	W5PD82	CALD1	5.26E-02	0.0000	MM0_Mo
-6	CYTO	W5NUE6	PLEKHA2	1.40E-03	0.0000	MM0_Mo
-6	CYTO	W5PFI6	RASGRP2	3.28E-03	0.0000	MM0_Mo
-6	CYTO	W5PFI7	VCL	2.68E-02	0.0000	MM0_Mo
-6	CYTO	W5PP64	FHL1	1.52E-02	0.0000	MM0_Mo
-6	CYTO	W5Q9B1	ATP2A3	1.77E-03	0.0000	MM0_Mo
-6	CYTO	W5PM33	SELP	3.11E-02	0.0000	MM0_Mo
-6	CYTO	W5PRW4	TRIAP1	2.82E-06	0.0002	MM0_Mo
-6	CYTO	W5P0K2	HEATR1	3.10E-04	0.0000	MM0_Mo
-6	CYTO	W5PL70	PDCD4	2.92E-03	0.0000	MM0_Mo
-6	CYTO	W5PYD8	PARP4	3.65E-04	0.0092	MM0_Mo
-6	CYTO	W5Q3J8		6.74E-04	0.0000	MM0_Mo
-6	CYTO	W5P4L3	AVIL	2.52E-02	0.0000	MM0_Mo
-6	CYTO	W5PE90	ITPR1	4.88E-04	0.0000	MM0_Mo
-6	CYTO	W5Q3B8		1.05E-02	0.0000	MM0_Mo
-6	CYTO	W5Q9S4	ING3	3.20E-03	0.0012	MM0_Mo
-6	CYTO	W5NU34	TREML1	2.44E-02	0.0000	MM0_Mo
-7	CYTO	W5PXV3	CCN2	1.33E-02	0.0000	MM0_Mo
-7	CYTO	W5PF87	ALOX15	6.10E-03	0.0000	MM0_Mo
-7	CYTO	W5NQ46	FGB	5.19E-02	0.0000	MM0_Mo
-7	CYTO	W5Q0K9	ABLIM1	2.47E-03	0.0000	MM0_Mo
-7	CYTO	W5PQI3	ITGB3	8.56E-03	0.0000	MM0_Mo
-7	CYTO	W5PJ75	SPTAN1	1.34E-02	0.0000	MM0_Mo
-7	CYTO	W5NZX9	SPTBN1	1.35E-03	0.0000	MM0_Mo
-7	CYTO	W5Q3I7	TUBB1	1.08E-02	0.0000	MM0_Mo
-8	CYTO	W5NXW9		2.30E-04	0.0000	MM0_Mo
-8	CYTO	W5NT35	LOC443162	7.05E-03	0.0000	MM0_Mo
-8	CYTO	W5Q5H8	FGA	3.20E-02	0.0000	MM0_Mo
-8	CYTO	W5QIK8	SELENBP1	4.33E-03	0.0000	MM0_Mo
-8	CYTO	W5PHQ0	MPO	2.67E-02	0.0000	MM0_Mo
-8	CYTO	W5Q2S8	MYL9	2.39E-03	0.0000	MM0_Mo
-9	CYTO	W5Q878	PRKCB	1.32E-03	0.0000	MM0_Mo
-9	CYTO	W5PEX2	GPATCH8	7.33E-03	0.0000	MM0_Mo
7	SEC	W5Q0F3	TGFBI	2.78E-04	0.0000	MM0_Mo
7	SEC	W5QHV3	FABP1	1.94E-03	0.0000	MM0_Mo
7	SEC	W5PDH4	MMP9	6.14E-04	0.0000	MM0_Mo
6	SEC	W5PUH5	LGMN	2.31E-04	0.0000	MM0_Mo
6	SEC	W5NWF5	RARRES1	2.67E-03	0.0000	MM0_Mo
6	SEC	W5NTD9	CHI3L1	1.39E-04	0.0000	MM0_Mo
6	SEC	W5Q8M1	PRRC1	3.06E-02	0.0016	MM0_Mo
6	SEC	W5Q9H0	MMP2	5.20E-05	0.0000	MM0_Mo

Supplementary Table 7: Shared and unique differentially abundant proteins (DAPs) between MMØ and GMØ relative to M0 and their cellular location.

FoldChange	Location	Protein IDs	Gene Names	P-value	FDR	Cell type
6	SEC	W5Q754	TTN	4.70E-02	0.0024	MM0_Mo
5	SEC	W5PS94	NUCB1	6.58E-03	0.0000	MM0_Mo
5	SEC	W5PIQ4	LOC101120093	5.16E-05	0.0000	MM0_Mo
5	SEC	W5NSK4		2.98E-03	0.0000	MM0_Mo
4	SEC	W5NTG6	TINAGL1	7.56E-04	0.0000	MM0_Mo
4	SEC	W5QJA2	CD14	3.71E-04	0.0000	MM0_Mo
4	SEC	W5PPC2		4.69E-02	0.0000	MM0_Mo
4	SEC	W5NPM4	TEX15	6.42E-03	0.0039	MM0_Mo
4	SEC	W5PGV0	ITGAM	6.34E-03	0.0000	MM0_Mo
4	SEC	W5Q689	TPP1	1.31E-03	0.0000	MM0_Mo
4	SEC	W5Q3T9	PDCD6IP	1.85E-02	0.0000	MM0_Mo
4	SEC	W5PEY4	TCN2	1.39E-02	0.0000	MM0_Mo
4	SEC	W5PZB0	APLP2	3.33E-03	0.0000	MM0_Mo
3	SEC	W5QFU8	CD86	5.56E-02	0.0000	MM0_Mo
3	SEC	W5Q3K6		6.09E-03	0.0000	MM0_Mo
3	SEC	W5Q799	GALNT6	7.65E-03	0.0099	MM0_Mo
3	SEC	W5NZ76	TIMP2	2.08E-02	0.0000	MM0_Mo
3	SEC	W5P887	CST3	3.59E-02	0.0000	MM0_Mo
3	SEC	W5PCC7	LOC101115509	1.08E-02	0.0000	MM0_Mo
3	SEC	W5PUJ5	LAMP2	1.23E-02	0.0000	MM0_Mo
3	SEC	W5PYS8		3.04E-02	0.0000	MM0_Mo
3	SEC	W5P3Q3	LOC100101238	2.13E-02	0.0000	MM0_Mo
2	SEC	W5PF73	PLAU	3.50E-02	0.0000	MM0_Mo
2	SEC	W5PCS7	CXCL16	2.39E-02	0.0045	MM0_Mo
2	SEC	W5P2K9	LMAN2	3.87E-02	0.0000	MM0_Mo
2	SEC	W5PE53	C8B	9.07E-03	0.0000	MM0_Mo
2	SEC	W5P1W2	FOLR3	4.82E-02	0.0093	MM0_Mo
2	SEC	W5PZI1	LOC101113728	1.04E-03	0.0000	MM0_Mo
2	SEC	W5NTT7	COL1A2	3.21E-02	0.0000	MM0_Mo
-2	SEC	P60713	ACTB	2.79E-02	0.0000	MM0_Mo
-2	SEC	W5PVE5	PIN1	3.67E-03	0.0000	MM0_Mo
-2	SEC	W5PSA3	TCN1	7.34E-03	0.0000	MM0_Mo
-2	SEC	W5QHL5	CAB39	3.07E-02	0.0000	MM0_Mo
-2	SEC	W5QFH1	ACTC1	3.52E-02	0.0000	MM0_Mo
-2	SEC	W5Q1I8	PPM1F	1.71E-02	0.0000	MM0_Mo
-2	SEC	W5PR23	CSE1L	3.84E-02	0.0043	MM0_Mo
-2	SEC	W5Q3E3	RAB2A	1.67E-02	0.0000	MM0_Mo
-2	SEC	W5PZ86	SEPTIN8	5.27E-02	0.0000	MM0_Mo
-2	SEC	W5NVQ4	CAND1	7.52E-03	0.0000	MM0_Mo
-2	SEC	W5Q7E2	GPS1	2.44E-02	0.0000	MM0_Mo
-2	SEC	W5P375	TCP1	1.78E-02	0.0000	MM0_Mo
-2	SEC	W5PFV5	NPEPPS	2.32E-02	0.0000	MM0_Mo
-2	SEC	W5QGD1	LDHB	3.34E-02	0.0000	MM0_Mo
-2	SEC	W5PI22	CNPY2	3.52E-02	0.0000	MM0_Mo
-2	SEC	W5NSM1	ACTR1A	1.70E-02	0.0000	MM0_Mo
-2	SEC	W5Q9X5	MAP2K1	9.37E-03	0.0000	MM0_Mo
-3	SEC	W5NX31	AHSA1	2.00E-02	0.0000	MM0_Mo
-3	SEC	W5QCL5	CSNK2A1	4.18E-02	0.0000	MM0_Mo
-3	SEC	W5PQ76	SRI	5.90E-03	0.0000	MM0_Mo
-3	SEC	W5P880	PRG4	7.59E-03	0.0000	MM0_Mo
-3	SEC	W5Q121		1.27E-02	0.0000	MM0_Mo
-3	SEC	P09670	SOD1	2.63E-03	0.0056	MM0_Mo
-3	SEC	W5PSZ3	JARID2	4.96E-02	0.0046	MM0_Mo
-3	SEC	W5P6X5	STMN1	2.96E-02	0.0000	MM0_Mo
-3	SEC	W5P583	ACP1	5.20E-02	0.0000	MM0_Mo
-3	SEC	W5PS88	GOT1	2.22E-02	0.0000	MM0_Mo
-3	SEC	W5PLS7	GRB2	1.30E-02	0.0000	MM0_Mo
-3	SEC	W5PQA8	PRDX6	2.51E-02	0.0000	MM0_Mo
-3	SEC	W5PMP1	COTL1	2.93E-02	0.0000	MM0_Mo
-3	SEC	W5Q7C8	BIN2	3.47E-02	0.0000	MM0_Mo
-3	SEC	W5P8K0	DCTN2	2.87E-02	0.0000	MM0_Mo
-3	SEC	W5P359	STIP1	8.09E-03	0.0000	MM0_Mo

Supplementary Table 7: Shared and unique differentially abundant proteins (DAPs) between MMØ and GMØ relative to M0 and their cellular location.

FoldChange	Location	Protein IDs	Gene Names	P-value	FDR	Cell type
-3	SEC	W5NSV5	ITGA6	2.06E-02	0.0000	MM0_Mo
-3	SEC	W5NSD5	RAP1B	8.97E-03	0.0000	MM0_Mo
-3	SEC	W5PWI4	RGN	2.87E-02	0.0000	MM0_Mo
-3	SEC	W5QG77	CD58	1.80E-02	0.0000	MM0_Mo
-3	SEC	W5PRJ4	VCP	3.43E-02	0.0000	MM0_Mo
-3	SEC	W5P889	SEPTIN9	6.08E-03	0.0000	MM0_Mo
-3	SEC	W5P5A0	FLNA	2.79E-02	0.0000	MM0_Mo
-3	SEC	W5PDL1	WDR1	1.66E-02	0.0000	MM0_Mo
-3	SEC	W5PNZ5	FKBP4	4.11E-02	0.0000	MM0_Mo
-3	SEC	W5Q6U0	FASN	1.10E-02	0.0000	MM0_Mo
-4	SEC	W5PK66	PARK7	4.21E-02	0.0000	MM0_Mo
-4	SEC	W5P5C4	NARS1	1.69E-02	0.0000	MM0_Mo
-4	SEC	W5P538	CD93	7.27E-04	0.0000	MM0_Mo
-4	SEC	Q1ZZU7	MIF	4.19E-02	0.0000	MM0_Mo
-4	SEC	W5NW17	STXBP2	2.05E-02	0.0000	MM0_Mo
-4	SEC	W5P7Z1		3.26E-02	0.0000	MM0_Mo
-4	SEC	W5NVC9	RAC1	2.46E-02	0.0000	MM0_Mo
-4	SEC	W5PE01	TAGLN2	7.65E-03	0.0000	MM0_Mo
-4	SEC	W5QI99	NEDD4	1.69E-03	0.0000	MM0_Mo
-4	SEC	W5QBQ9	MYH9	1.15E-02	0.0000	MM0_Mo
-4	SEC	W5PYM5	CCT8	3.69E-02	0.0000	MM0_Mo
-4	SEC	W5PHU7	UNC13D	6.54E-03	0.0000	MM0_Mo
-4	SEC	W5Q5A6	FGG	3.29E-02	0.0000	MM0_Mo
-4	SEC	W5QGT4		3.43E-02	0.0000	MM0_Mo
-4	SEC	W5P0V5	RAB11B	3.36E-04	0.0000	MM0_Mo
-4	SEC	Q5MIB6	PYGB	1.53E-02	0.0000	MM0_Mo
-4	SEC	W5Q9T3	LMNB1	3.68E-02	0.0000	MM0_Mo
-4	SEC	W5P8B4	TRAPPC8	2.34E-02	0.0055	MM0_Mo
-4	SEC	W5PVT6	UBA1	2.35E-02	0.0000	MM0_Mo
-4	SEC	W5P7E2	PPA1	1.00E-02	0.0000	MM0_Mo
-4	SEC	W5Q6H1	PLS3	2.31E-03	0.0000	MM0_Mo
-4	SEC	W5P765	CCT3	4.89E-02	0.0000	MM0_Mo
-4	SEC	W5PMQ9	SAE1	2.84E-05	0.0000	MM0_Mo
-4	SEC	W5PQF0	CCT7	5.39E-02	0.0000	MM0_Mo
-4	SEC	W5PWG1	PFKP	7.52E-03	0.0000	MM0_Mo
-4	SEC	W5QHS2	MGP	2.70E-04	0.0000	MM0_Mo
-4	SEC	W5P409	FERMT3	1.85E-02	0.0000	MM0_Mo
-5	SEC	W5QFH5	RAB1A	2.64E-03	0.0000	MM0_Mo
-5	SEC	W5NQK6	LIMS1	2.87E-03	0.0000	MM0_Mo
-5	SEC	W5PK85	EML2	1.13E-02	0.0000	MM0_Mo
-5	SEC	W5PTZ9	LOC101120877	3.37E-03	0.0000	MM0_Mo
-5	SEC	W5PY17	STX7	7.88E-03	0.0000	MM0_Mo
-5	SEC	W5Q3B7	PDE5A	3.55E-02	0.0000	MM0_Mo
-5	SEC	W5PPG3	ALDH9A1	2.31E-02	0.0000	MM0_Mo
-5	SEC	W5QD96	PARVB	1.41E-03	0.0000	MM0_Mo
-5	SEC	W5P6Z6	MAPRE2	5.32E-03	0.0000	MM0_Mo
-5	SEC	W5QBD7	YWHAZ	5.75E-03	0.0000	MM0_Mo
-5	SEC	W5PL19	LOC101105123	2.94E-02	0.0000	MM0_Mo
-5	SEC	W5PE27	ESD	2.04E-02	0.0000	MM0_Mo
-5	SEC	W5PRF0	SUB1	3.39E-04	0.0000	MM0_Mo
-5	SEC	P62262	YWHAE	3.53E-02	0.0000	MM0_Mo
-5	SEC	W5NRW4	TPM4	1.25E-02	0.0000	MM0_Mo
-5	SEC	W5NRD9		5.06E-03	0.0016	MM0_Mo
-5	SEC	W5Q731	ILK	1.43E-02	0.0000	MM0_Mo
-5	SEC	W5P4C7	SEPTIN7	5.23E-03	0.0000	MM0_Mo
-6	SEC	W5QFZ3	CCT4	4.18E-02	0.0000	MM0_Mo
-6	SEC	W5PIJ6	PTPN11	2.27E-02	0.0000	MM0_Mo
-6	SEC	W5PX84	CCDC171	4.61E-04	0.0046	MM0_Mo
-6	SEC	W5QFM1		3.11E-02	0.0000	MM0_Mo
-6	SEC	W5P627	GSN	3.68E-03	0.0000	MM0_Mo
-6	SEC	W5PH15	RSU1	2.34E-03	0.0000	MM0_Mo
-7	SEC	W5PQK6	TLN1	2.34E-02	0.0000	MM0_Mo

Supplementary Table 7: Shared and unique differentially abundant proteins (DAPs) between MMØ and GMØ relative to M0 and their cellular location.

FoldChange	Location	Protein IDs	Gene Names	P-value	FDR	Cell type
-9	SEC	W5PYX0		7.91E-03	0.0000	MM0_Mo
11	CYTO	W5PEB0	FABP7	2.16E-06	0.0000	GM0_Mo
9	CYTO	W5NU86	GLA	9.34E-04	0.0000	GM0_Mo
9	CYTO	W5PCH3	SCIN	1.19E-04	0.0000	GM0_Mo
8	CYTO	W5NY01		5.10E-04	0.0000	GM0_Mo
8	CYTO	W5PAM4	CTSA	8.29E-05	0.0000	GM0_Mo
8	CYTO	W5PEL7	HSPG2	1.73E-05	0.0000	GM0_Mo
8	CYTO	W5PF33	GM2A	7.42E-06	0.0000	GM0_Mo
8	CYTO	W5PT76	GPNMB	1.60E-04	0.0000	GM0_Mo
8	CYTO	P51977	ALDH1A1 ALDH1	1.55E-03	0.0000	GM0_Mo
8	CYTO	A9YUY8	FABP4	5.78E-04	0.0000	GM0_Mo
8	CYTO	W5PRI6	MRC1	1.15E-04	0.0000	GM0_Mo
8	CYTO	W5PFV1	CTSL	4.03E-04	0.0000	GM0_Mo
7	CYTO	W5QHG1	EPS8	3.89E-05	0.0000	GM0_Mo
7	CYTO	W5QBA2	ATRX	1.18E-04	0.0000	GM0_Mo
7	CYTO	W5P9J8	BLVRB	2.18E-04	0.0000	GM0_Mo
7	CYTO	W5PKQ7		6.16E-05	0.0070	GM0_Mo
7	CYTO	W5PBM9	SCPEP1	1.95E-04	0.0000	GM0_Mo
7	CYTO	W5PE67		6.85E-05	0.0000	GM0_Mo
7	CYTO	W5PIQ6	MSR1	6.63E-05	0.0000	GM0_Mo
7	CYTO	W5PFY5	ASAH1	1.46E-03	0.0000	GM0_Mo
7	CYTO	W5PUP6	SPATA13	5.44E-02	0.0028	GM0_Mo
7	CYTO	W5PVR2	LOC101108019	6.26E-05	0.0078	GM0_Mo
7	CYTO	W5PJS4	EMILIN2	2.03E-04	0.0000	GM0_Mo
7	CYTO	W5QHL1	FCGR1A	1.33E-04	0.0000	GM0_Mo
6	CYTO	W5P2F1	FOLR2	1.68E-05	0.0000	GM0_Mo
6	CYTO	W5P9G8	PLD3	1.79E-02	0.0000	GM0_Mo
6	CYTO	W5PZB2	CD68	2.07E-04	0.0000	GM0_Mo
6	CYTO	W5PQR0	NIBAN2	7.63E-06	0.0000	GM0_Mo
6	CYTO	W5PI56	DAB2	1.53E-03	0.0000	GM0_Mo
6	CYTO	W5P1A5	GBA1	2.78E-04	0.0000	GM0_Mo
6	CYTO	W5PGC5	GALM	1.08E-03	0.0000	GM0_Mo
6	CYTO	W5Q233	VCAN	1.36E-04	0.0000	GM0_Mo
6	CYTO	P35623	SHMT1	1.54E-04	0.0000	GM0_Mo
6	CYTO	Q9MZS8	CTSD	1.26E-03	0.0000	GM0_Mo
6	CYTO	W5PCE0	PLBD2	4.97E-03	0.0000	GM0_Mo
6	CYTO	W5QC89	HEXA	1.92E-04	0.0000	GM0_Mo
6	CYTO	W5P9M8	UCK1	2.80E-05	0.0002	GM0_Mo
6	CYTO	W5NVW7	NAGLU	2.94E-03	0.0000	GM0_Mo
6	CYTO	W5Q3S9	DSN1	1.59E-03	0.0010	GM0_Mo
6	CYTO	W5PE73	SMPDL3A	1.01E-05	0.0000	GM0_Mo
6	CYTO	W5PUL5	FCGRT	5.02E-04	0.0000	GM0_Mo
6	CYTO	P79365	SLC2A1 GLUT1	1.82E-04	0.0052	GM0_Mo
6	CYTO	W5PV43	LRPAP1	3.00E-04	0.0000	GM0_Mo
6	CYTO	W5PAQ4	FUCA1	3.63E-04	0.0000	GM0_Mo
6	CYTO	G3M9U4	ACP5	3.04E-04	0.0000	GM0_Mo
6	CYTO	W5NTJ2	TPX2	4.04E-05	0.0057	GM0_Mo
6	CYTO	W5Q2Y1	PLXNC1	7.56E-03	0.0000	GM0_Mo
6	CYTO	W5PKY1	HNMT	8.05E-04	0.0000	GM0_Mo
6	CYTO	W5PXR1	ENPP1	6.24E-04	0.0000	GM0_Mo
6	CYTO	W5Q940	SHTN1	3.35E-03	0.0000	GM0_Mo
6	CYTO	W5P093	NQO1	4.89E-06	0.0000	GM0_Mo
6	CYTO	W5PBS4	LRP1	3.79E-04	0.0000	GM0_Mo
5	CYTO	W5P0K8	CLIC2	2.22E-02	0.0000	GM0_Mo
5	CYTO	W5Q6N3	LOC101115115	3.12E-04	0.0000	GM0_Mo
5	CYTO	W5QCD6	IDH1	4.42E-05	0.0000	GM0_Mo
5	CYTO	W5Q612	TRPV2	4.37E-05	0.0000	GM0_Mo
5	CYTO	W5Q0A3	TLR2	8.55E-04	0.0000	GM0_Mo
5	CYTO	W5NYU9	MPP1	1.21E-04	0.0000	GM0_Mo
5	CYTO	W5NTZ3	RENBP	6.63E-04	0.0000	GM0_Mo
5	CYTO	W5NUI6	SGSH	3.56E-04	0.0000	GM0_Mo
5	CYTO	W5Q8D1	WDR74	2.34E-04	0.0000	GM0_Mo

Supplementary Table 7: Shared and unique differentially abundant proteins (DAPs) between MMØ and GMØ relative to M0 and their cellular location.

FoldChange	Location	Protein IDs	Gene Names	P-value	FDR	Cell type
5	CYTO	W5PNP1	MFGE8	5.07E-03	0.0000	GM0_Mo
5	CYTO	W5QFA5		5.82E-04	0.0002	GM0_Mo
5	CYTO	W5QI12	TLN2	3.36E-04	0.0052	GM0_Mo
5	CYTO	W5QGH9	WDR3	4.25E-03	0.0002	GM0_Mo
5	CYTO	W5NRS0		2.55E-04	0.0000	GM0_Mo
5	CYTO	W5PA90	AGA	1.05E-04	0.0000	GM0_Mo
5	CYTO	W5P1H0	CTSC	2.07E-06	0.0000	GM0_Mo
5	CYTO	W5NSH8	NPC2	1.25E-04	0.0000	GM0_Mo
5	CYTO	W5QDW4	USF3	7.93E-03	0.0010	GM0_Mo
5	CYTO	W5NVC8	NLRP2	7.70E-04	0.0091	GM0_Mo
5	CYTO	W5P640	LMNA	3.32E-03	0.0000	GM0_Mo
5	CYTO	W5PWX3	CRYL1	4.62E-04	0.0000	GM0_Mo
5	CYTO	W5PPX2	SENP8	2.45E-04	0.0030	GM0_Mo
5	CYTO	W5QIW1	LGALS3	6.10E-03	0.0000	GM0_Mo
5	CYTO	W5PKU3		8.59E-04	0.0000	GM0_Mo
5	CYTO	W5PZ47	CTSH	5.50E-02	0.0000	GM0_Mo
5	CYTO	W5NYL0	MAOA	1.80E-03	0.0000	GM0_Mo
5	CYTO	W5QI00	LACTB	4.15E-04	0.0000	GM0_Mo
5	CYTO	W5NRB6	MIDEAS	1.73E-03	0.0002	GM0_Mo
5	CYTO	W5QBZ7	NAGA	3.91E-03	0.0000	GM0_Mo
5	CYTO	W5PQA6	CYFIP2	1.70E-05	0.0000	GM0_Mo
5	CYTO	W5PAC2	LOC101105044	8.15E-03	0.0000	GM0_Mo
5	CYTO	W5PNZ7	RFC1	3.86E-04	0.0000	GM0_Mo
5	CYTO	W5QI70	CTSS	1.69E-05	0.0000	GM0_Mo
5	CYTO	W5PUH5	LGMN	9.61E-03	0.0000	GM0_Mo
5	CYTO	W5Q2B3	SUSD5	1.09E-03	0.0086	GM0_Mo
5	CYTO	W5P3H8	IGF2R	5.60E-04	0.0000	GM0_Mo
5	CYTO	W5P026	STAB1	3.36E-04	0.0000	GM0_Mo
5	CYTO	W5NUC8	ARMCX3	2.69E-04	0.0000	GM0_Mo
5	CYTO	W5PD62	CPB2	9.40E-04	0.0091	GM0_Mo
5	CYTO	W5PG74	IRGQ	3.40E-04	0.0000	GM0_Mo
5	CYTO	W5PYW0	TCIRG1	6.60E-05	0.0000	GM0_Mo
5	CYTO	W5PXS1	RAB3IL1	1.37E-03	0.0000	GM0_Mo
5	CYTO	W5PDH7	NPC1	2.77E-04	0.0000	GM0_Mo
5	CYTO	P83205	CTSB	2.37E-03	0.0000	GM0_Mo
5	CYTO	W5Q284	HAPLN1	4.64E-04	0.0000	GM0_Mo
5	CYTO	W5P8H9	SGPL1	1.55E-03	0.0000	GM0_Mo
5	CYTO	W5NSN4		1.79E-03	0.0002	GM0_Mo
5	CYTO	W5P530	LOC101104705	3.11E-02	0.0000	GM0_Mo
5	CYTO	W5NX16		3.63E-04	0.0000	GM0_Mo
5	CYTO	W5PUW3		1.25E-03	0.0000	GM0_Mo
5	CYTO	W5Q4B1	MYCBP2	3.55E-04	0.0000	GM0_Mo
5	CYTO	W5QGG0	TFRC	7.17E-05	0.0000	GM0_Mo
5	CYTO	W5Q5W2	LOC101110539	6.12E-05	0.0000	GM0_Mo
5	CYTO	W5Q4U5	CPT1A	1.08E-03	0.0000	GM0_Mo
5	CYTO	W5PDH4	MMP9	2.29E-03	0.0000	GM0_Mo
5	CYTO	W5Q263	ICAM1	4.27E-04	0.0000	GM0_Mo
5	CYTO	W5P949	FILIP1	4.46E-04	0.0039	GM0_Mo
5	CYTO	W5PK56	FCGR2B	3.76E-02	0.0000	GM0_Mo
5	CYTO	W5NZ40	SGF29	3.89E-02	0.0002	GM0_Mo
5	CYTO	W5NQS7	IFNGR1	6.38E-04	0.0000	GM0_Mo
5	CYTO	W5PZG5	OCRL	1.25E-02	0.0000	GM0_Mo
5	CYTO	W5NTW3	ITIH1	8.51E-04	0.0000	GM0_Mo
5	CYTO	W5P8M9		2.60E-03	0.0000	GM0_Mo
5	CYTO	W5PE92	GRN	7.34E-04	0.0000	GM0_Mo
5	CYTO	W5PVN6		4.36E-04	0.0059	GM0_Mo
5	CYTO	W5Q1W7	PALLD	4.63E-04	0.0000	GM0_Mo
5	CYTO	W5QBM4	ALCAM	1.09E-03	0.0000	GM0_Mo
5	CYTO	W5NX56	SPP1	4.99E-04	0.0000	GM0_Mo
5	CYTO	W5NZ62	GNS	5.54E-03	0.0000	GM0_Mo
4	CYTO	W5P3S0		8.97E-06	0.0000	GM0_Mo
4	CYTO	W5NU23	FUCA2	4.09E-04	0.0000	GM0_Mo

Supplementary Table 7: Shared and unique differentially abundant proteins (DAPs) between MMØ and GMØ relative to M0 and their cellular location.

FoldChange	Location	Protein IDs	Gene Names	P-value	FDR	Cell type
4	CYTO	W5Q7I8	TAX1BP3	4.50E-02	0.0000	GM0_Mo
4	CYTO	W5Q3N1	CTSZ	3.22E-05	0.0000	GM0_Mo
4	CYTO	W5NZK6	PLA2G15	3.02E-03	0.0000	GM0_Mo
4	CYTO	W5P407		1.20E-02	0.0000	GM0_Mo
4	CYTO	W5NZ47	RBP4	1.01E-02	0.0000	GM0_Mo
4	CYTO	W5Q678	SEC24D	8.44E-03	0.0000	GM0_Mo
4	CYTO	W5PSE5	LOC101110434	2.93E-03	0.0002	GM0_Mo
4	CYTO	W5QBE4	FGL2	2.00E-03	0.0000	GM0_Mo
4	CYTO	W5PJT1	PLAC9	2.87E-03	0.0000	GM0_Mo
4	CYTO	W5PRG8	CREG1	1.93E-03	0.0000	GM0_Mo
4	CYTO	W5PNY4	PDXK	1.67E-02	0.0000	GM0_Mo
4	CYTO	W5QH68	LRRC57	3.11E-04	0.0002	GM0_Mo
4	CYTO	P05028	ATP1B1	5.99E-03	0.0000	GM0_Mo
4	CYTO	W5P5W6	NDRG1	2.55E-02	0.0000	GM0_Mo
4	CYTO	W5QH35	CAPG	4.88E-04	0.0000	GM0_Mo
4	CYTO	W5QAB1	HPX	3.14E-03	0.0000	GM0_Mo
4	CYTO	W5QEM8	LOC101111528	1.37E-03	0.0000	GM0_Mo
4	CYTO	W5QBG8	PPFIA1	2.96E-03	0.0000	GM0_Mo
4	CYTO	W5Q3U3	LOC101102156	8.12E-03	0.0000	GM0_Mo
4	CYTO	W5QCL8	NPL	2.47E-03	0.0000	GM0_Mo
4	CYTO	W5PLB8	EPB41L3	4.44E-04	0.0000	GM0_Mo
4	CYTO	W5PP04	GNG12	7.65E-04	0.0000	GM0_Mo
4	CYTO	W5NYE0	ATP6V0D1	4.08E-02	0.0000	GM0_Mo
4	CYTO	Q29524	LPL	7.86E-03	0.0000	GM0_Mo
4	CYTO	W5PBC0		5.75E-03	0.0000	GM0_Mo
4	CYTO	W5PGS4	FABP5	1.12E-03	0.0000	GM0_Mo
4	CYTO	W5QI78	CTSK	3.24E-02	0.0000	GM0_Mo
4	CYTO	W5PY08		1.11E-02	0.0000	GM0_Mo
4	CYTO	W5Q700	APPL2	2.64E-04	0.0000	GM0_Mo
4	CYTO	W5PIS6	NHLRC3	1.94E-03	0.0000	GM0_Mo
4	CYTO	W5PYI8	WWC1	1.34E-02	0.0000	GM0_Mo
4	CYTO	W5PEE9	LAMP1	1.34E-02	0.0000	GM0_Mo
4	CYTO	W5P7G4	TNC	8.62E-03	0.0000	GM0_Mo
4	CYTO	W5PMA0	AP2S1	4.96E-03	0.0000	GM0_Mo
4	CYTO	W5PII2	ATG4C	6.05E-04	0.0000	GM0_Mo
4	CYTO	W5P3N6	LOC101112162	1.35E-03	0.0000	GM0_Mo
4	CYTO	W5QEH8	CTTNBP2NL	6.67E-05	0.0000	GM0_Mo
4	CYTO	W5P150		2.16E-03	0.0002	GM0_Mo
4	CYTO	W5Q8J3	RRBP1	8.72E-03	0.0000	GM0_Mo
4	CYTO	W5PTU7	CA2	1.03E-03	0.0000	GM0_Mo
4	CYTO	W5NW80	GAA	3.15E-03	0.0000	GM0_Mo
4	CYTO	W5Q318	CCNH	2.56E-04	0.0043	GM0_Mo
4	CYTO	W5NWX7	CLEC4A	3.76E-03	0.0000	GM0_Mo
4	CYTO	W5P3L5	RNF13	2.00E-05	0.0000	GM0_Mo
4	CYTO	W5Q0U0	P2RX4	1.83E-03	0.0000	GM0_Mo
4	CYTO	W5QA16		7.75E-04	0.0000	GM0_Mo
4	CYTO	Q9MZD1	SLC17A5	1.04E-03	0.0000	GM0_Mo
4	CYTO	W5PD43	HTRA1	2.69E-02	0.0000	GM0_Mo
4	CYTO	W5P2V3	PEPD	5.51E-03	0.0000	GM0_Mo
4	CYTO	W5QHU8	FNDC3B	7.39E-05	0.0000	GM0_Mo
4	CYTO	W5NVR9	C21H11orf54	1.19E-04	0.0000	GM0_Mo
4	CYTO	O18882	ATP6V0C ATP6C ATP	4.30E-03	0.0000	GM0_Mo
4	CYTO	W5QI36	HEBP1	1.78E-02	0.0000	GM0_Mo
4	CYTO	W5Q5C8	SOAT1	6.87E-04	0.0000	GM0_Mo
4	CYTO	W5P6H9	ACP2	4.54E-02	0.0000	GM0_Mo
4	CYTO	C5IJ93	RAB9A	1.95E-03	0.0000	GM0_Mo
4	CYTO	W5Q5N6	BST-2B	2.81E-03	0.0000	GM0_Mo
4	CYTO	W5P5L4	COG5	4.69E-04	0.0000	GM0_Mo
4	CYTO	W5Q6Q3	TCP11L1	6.62E-04	0.0087	GM0_Mo
4	CYTO	W5PG10	PAPSS1	3.19E-02	0.0000	GM0_Mo
4	CYTO	W5PBR7	P4HA1	7.68E-03	0.0000	GM0_Mo
4	CYTO	W5Q9M9	GK	2.64E-02	0.0000	GM0_Mo

Supplementary Table 7: Shared and unique differentially abundant proteins (DAPs) between MMØ and GMØ relative to M0 and their cellular location.

FoldChange	Location	Protein IDs	Gene Names	P-value	FDR	Cell type
4	CYTO	W5PR99	DHPS	2.23E-04	0.0004	GM0_Mo
4	CYTO	W5Q3V0	IL18	5.04E-04	0.0000	GM0_Mo
4	CYTO	W5PN31		7.34E-05	0.0008	GM0_Mo
4	CYTO	W5NWX4		1.26E-02	0.0000	GM0_Mo
4	CYTO	W5QI40	MYO1E	1.62E-02	0.0000	GM0_Mo
4	CYTO	W5NZV3	HMOX2	3.98E-02	0.0000	GM0_Mo
4	CYTO	W5PEY2	CTSV	3.00E-04	0.0000	GM0_Mo
4	CYTO	W5QB71	AMDHD2	2.58E-03	0.0000	GM0_Mo
4	CYTO	W5QDF4	GSTM3	2.24E-03	0.0000	GM0_Mo
4	CYTO	W5PG41	H6PD	2.21E-03	0.0000	GM0_Mo
4	CYTO	W5P703	WFS1	7.11E-03	0.0000	GM0_Mo
4	CYTO	P12303	TTR	2.19E-02	0.0000	GM0_Mo
4	CYTO	W5P6L1	RASGRP4	1.91E-03	0.0000	GM0_Mo
4	CYTO	W5PRG9	HAL	4.00E-02	0.0000	GM0_Mo
4	CYTO	W5QFU4		2.18E-02	0.0000	GM0_Mo
4	CYTO	W5PB38		2.12E-03	0.0000	GM0_Mo
4	CYTO	W5Q723	BHMT	2.22E-02	0.0000	GM0_Mo
4	CYTO	W5PH35	LOC101119706	1.03E-03	0.0000	GM0_Mo
4	CYTO	W5Q0F1	LIPA	1.58E-03	0.0000	GM0_Mo
4	CYTO	P82197	PDXK PKH	1.19E-02	0.0000	GM0_Mo
4	CYTO	W5PCE8	ATP2B3	1.38E-02	0.0052	GM0_Mo
4	CYTO	W5P168	SH3PXD2B	4.09E-03	0.0000	GM0_Mo
4	CYTO	W5P8N6	GAB2	6.08E-04	0.0000	GM0_Mo
4	CYTO	W5PVC8	ERGIC3	2.85E-03	0.0000	GM0_Mo
4	CYTO	W5QEH0	TWF1	5.53E-03	0.0000	GM0_Mo
4	CYTO	W5NPP2	CPM	2.86E-02	0.0000	GM0_Mo
4	CYTO	W5QBP6	GLUL	2.33E-02	0.0000	GM0_Mo
4	CYTO	W5PZ94	ACO1	3.12E-02	0.0000	GM0_Mo
4	CYTO	W5PHJ3	AHR	2.54E-03	0.0000	GM0_Mo
4	CYTO	W5Q6K0	HEATR3	8.44E-04	0.0025	GM0_Mo
4	CYTO	W5Q3H9	UPP1	3.46E-02	0.0000	GM0_Mo
3	CYTO	W5Q1K4	DICER1	4.75E-02	0.0000	GM0_Mo
3	CYTO	W5QHR5	PLEKHO2	7.08E-05	0.0000	GM0_Mo
3	CYTO	W5Q6S3	WASHC4	2.10E-03	0.0000	GM0_Mo
3	CYTO	W5PVM8	UBR7	4.48E-03	0.0000	GM0_Mo
3	CYTO	W5PCI1	NLRP3	1.20E-03	0.0000	GM0_Mo
3	CYTO	W5NS94		3.82E-05	0.0000	GM0_Mo
3	CYTO	W5PMX5	C5AR1	9.17E-03	0.0000	GM0_Mo
3	CYTO	W5PCD5	ARMC8	1.46E-02	0.0000	GM0_Mo
3	CYTO	W5P0C5	LGALS8	1.10E-04	0.0000	GM0_Mo
3	CYTO	W5PJN7	HOMER3	6.91E-04	0.0000	GM0_Mo
3	CYTO	W5PB61		3.01E-03	0.0002	GM0_Mo
3	CYTO	W5PMU8	GAB1	5.31E-03	0.0000	GM0_Mo
3	CYTO	W5Q2R5	SDF2L1	4.09E-02	0.0000	GM0_Mo
3	CYTO	W5QA17	ATXN2	3.29E-02	0.0000	GM0_Mo
3	CYTO	W5PE91	LOC101105400	1.07E-03	0.0002	GM0_Mo
3	CYTO	W5NUE5	SLC38A7	1.05E-03	0.0000	GM0_Mo
3	CYTO	W5PCA0	ALDOB	2.81E-03	0.0000	GM0_Mo
3	CYTO	W5NT90	BAG4	2.68E-02	0.0000	GM0_Mo
3	CYTO	W5P4A8	RNASET2	1.27E-03	0.0000	GM0_Mo
3	CYTO	W5Q6C5	GGA2	1.04E-02	0.0000	GM0_Mo
3	CYTO	W5P3W6	OSBPL9	1.42E-04	0.0000	GM0_Mo
3	CYTO	W5NRW1	FBXO6	4.19E-02	0.0000	GM0_Mo
3	CYTO	W5PS40		1.14E-03	0.0002	GM0_Mo
3	CYTO	W5P4X6	LOC101104287	1.08E-02	0.0000	GM0_Mo
3	CYTO	W5P366	FAM50A	2.50E-02	0.0000	GM0_Mo
3	CYTO	W5QDH3	COL2A1	3.29E-03	0.0017	GM0_Mo
3	CYTO	W5PGF4	PLAUR	1.28E-02	0.0000	GM0_Mo
3	CYTO	W5PBL5	SLC44A1	2.74E-04	0.0000	GM0_Mo
3	CYTO	W5Q8I7	LOC101122123	4.42E-03	0.0000	GM0_Mo
3	CYTO	W5Q777	HS1BP3	9.75E-04	0.0000	GM0_Mo
3	CYTO	W5PPM6	PQBP1	5.41E-03	0.0002	GM0_Mo

Supplementary Table 7: Shared and unique differentially abundant proteins (DAPs) between MMØ and GMØ relative to M0 and their cellular location.

FoldChange	Location	Protein IDs	Gene Names	P-value	FDR	Cell type
3	CYTO	W5QCG9	SRXN1	2.96E-02	0.0000	GM0_Mo
3	CYTO	W5Q2U7	PLEC	1.24E-05	0.0000	GM0_Mo
3	CYTO	W5NRL0	PLCD1	5.12E-04	0.0000	GM0_Mo
3	CYTO	W5Q5P5	GGH	1.42E-02	0.0000	GM0_Mo
3	CYTO	W5QHZ0	CA12	5.02E-03	0.0000	GM0_Mo
3	CYTO	W5P2N4	AHCYL2	5.96E-03	0.0000	GM0_Mo
3	CYTO	W5PT36	RBM47	1.50E-03	0.0000	GM0_Mo
3	CYTO	W5PXX3	F13B	3.91E-02	0.0071	GM0_Mo
3	CYTO	W5PP47		2.25E-04	0.0000	GM0_Mo
3	CYTO	W5NR40	FLT4	1.46E-03	0.0000	GM0_Mo
3	CYTO	W5Q1M0	GLB1	3.25E-04	0.0000	GM0_Mo
3	CYTO	W5QHX1	EIF5A2	4.05E-03	0.0000	GM0_Mo
3	CYTO	W5P6V4	GLG1	5.59E-02	0.0000	GM0_Mo
3	CYTO	Q10994	CSTB CST6	1.93E-04	0.0000	GM0_Mo
3	CYTO	W5PKA1	LOC101116157	3.77E-02	0.0000	GM0_Mo
3	CYTO	W5PK31	FCGR3A	3.74E-04	0.0000	GM0_Mo
3	CYTO	W5Q0G8	IMPA1	4.63E-03	0.0000	GM0_Mo
3	CYTO	W5NXH3	LOC101106542	6.12E-04	0.0000	GM0_Mo
3	CYTO	W5PYB9	ABCA6	1.41E-03	0.0000	GM0_Mo
3	CYTO	W5Q430	IDUA	2.39E-03	0.0002	GM0_Mo
3	CYTO	W5PNK3	ACAN	1.41E-03	0.0000	GM0_Mo
3	CYTO	W5Q3C2	EPPK1	6.73E-03	0.0000	GM0_Mo
3	CYTO	W5PWI4	RGN	6.88E-03	0.0000	GM0_Mo
3	CYTO	W5NSA6	LOC101122940	1.94E-02	0.0000	GM0_Mo
3	CYTO	W5P2W1	NEU1	4.05E-03	0.0000	GM0_Mo
3	CYTO	W5NUJ5		3.22E-02	0.0000	GM0_Mo
3	CYTO	W5P800	GAS7	5.08E-02	0.0000	GM0_Mo
3	CYTO	W5QH13	VPS39	1.42E-03	0.0000	GM0_Mo
3	CYTO	W5PDZ1		1.34E-03	0.0000	GM0_Mo
3	CYTO	W5PKF9	FIG4	1.63E-03	0.0000	GM0_Mo
3	CYTO	W5NT19	SPAG1	2.26E-03	0.0000	GM0_Mo
3	CYTO	W5NYL7	MTHFD1L	1.03E-02	0.0000	GM0_Mo
3	CYTO	W5QG92	OSBPL11	1.35E-03	0.0000	GM0_Mo
3	CYTO	W5Q7R3	LOC101118736	2.25E-02	0.0000	GM0_Mo
3	CYTO	W5PPT8	GLIPR2	7.74E-03	0.0000	GM0_Mo
3	CYTO	W5P3B0	INPPL1	2.88E-02	0.0000	GM0_Mo
3	CYTO	W5QBR5	BMP2K	8.03E-03	0.0000	GM0_Mo
3	CYTO	W5PEC0	PKIB	4.96E-02	0.0000	GM0_Mo
3	CYTO	W5P7Y8	PALD1	1.50E-03	0.0000	GM0_Mo
3	CYTO	W5QHF2	LOC114117536	7.47E-04	0.0000	GM0_Mo
3	CYTO	W5P3I5	CNDP2	4.00E-04	0.0000	GM0_Mo
3	CYTO	W5NSQ3	HFE	1.14E-03	0.0000	GM0_Mo
3	CYTO	W5Q8G9	SPG21	2.54E-03	0.0000	GM0_Mo
3	CYTO	W5PUW2	IFI30	1.29E-03	0.0000	GM0_Mo
3	CYTO	W5PNS2	TLR7	2.27E-02	0.0000	GM0_Mo
3	CYTO	W5PGL9		2.55E-03	0.0000	GM0_Mo
3	CYTO	W5Q463	INTU	5.55E-02	0.0099	GM0_Mo
3	CYTO	W5PIE4	CLPTM1	5.17E-02	0.0000	GM0_Mo
3	CYTO	W5P5Q2	MVP	4.98E-02	0.0000	GM0_Mo
3	CYTO	W5NZJ7	FGR	4.36E-02	0.0000	GM0_Mo
3	CYTO	W5PYH9		3.05E-02	0.0000	GM0_Mo
3	CYTO	W5Q686	TPP1	2.46E-03	0.0000	GM0_Mo
3	CYTO	W5P4K6	FCHO2	1.75E-03	0.0000	GM0_Mo
3	CYTO	W5PLL2	F9	4.38E-03	0.0000	GM0_Mo
3	CYTO	W5PDK4	AIF1	2.92E-02	0.0000	GM0_Mo
3	CYTO	W5Q560	OGFOD3	2.34E-02	0.0000	GM0_Mo
3	CYTO	W5P536	TRIP10	1.86E-03	0.0000	GM0_Mo
3	CYTO	W5Q7X8	CRYBG3	9.04E-03	0.0000	GM0_Mo
3	CYTO	W5NUE3	PRDX1	6.16E-04	0.0000	GM0_Mo
3	CYTO	W5PQV2	LMBRD1	8.34E-04	0.0000	GM0_Mo
3	CYTO	W5PZ62	ZFYVE16	4.68E-03	0.0000	GM0_Mo
3	CYTO	W5Q0N7	IFIT2	1.27E-02	0.0000	GM0_Mo

Supplementary Table 7: Shared and unique differentially abundant proteins (DAPs) between MMØ and GMØ relative to M0 and their cellular location.

FoldChange	Location	Protein IDs	Gene Names	P-value	FDR	Cell type
3	CYTO	W5P369	AP2A2	4.05E-05	0.0000	GM0_Mo
3	CYTO	W5Q3B2	TYK2	1.52E-02	0.0000	GM0_Mo
3	CYTO	W5PL89	GSR	1.02E-02	0.0000	GM0_Mo
3	CYTO	W5PCD0	FUBP3	3.68E-02	0.0000	GM0_Mo
3	CYTO	W5PZD7		2.43E-03	0.0000	GM0_Mo
3	CYTO	W5PC32	PGM3	1.16E-02	0.0000	GM0_Mo
3	CYTO	W5PFH4	MAPKAPK3	2.20E-02	0.0000	GM0_Mo
3	CYTO	W5Q7Z6	DIP2B	6.45E-03	0.0000	GM0_Mo
3	CYTO	W5NXQ8	KATNA1	6.26E-03	0.0002	GM0_Mo
3	CYTO	W5PFE6	ACOX1	3.90E-03	0.0000	GM0_Mo
3	CYTO	W5NZ70	LGALS3BP	2.17E-02	0.0000	GM0_Mo
3	CYTO	W5QBV7	CD44	9.79E-05	0.0000	GM0_Mo
3	CYTO	P29330	FDX1	1.14E-03	0.0000	GM0_Mo
3	CYTO	Q6XXL8	DYNLT3 TCTE1L	3.45E-03	0.0000	GM0_Mo
3	CYTO	W5NVV6	DNAJC3	4.00E-02	0.0000	GM0_Mo
3	CYTO	W5PDS4	C1QA	1.24E-02	0.0000	GM0_Mo
3	CYTO	W5Q0E7	LIN7C	1.43E-03	0.0059	GM0_Mo
3	CYTO	W5PVC1	SYNJ1	1.24E-03	0.0000	GM0_Mo
3	CYTO	W5PAX1	GCLC	3.58E-02	0.0000	GM0_Mo
3	CYTO	W5PL47	BMP1	3.45E-04	0.0000	GM0_Mo
3	CYTO	W5NYK1	PMVK	4.87E-03	0.0000	GM0_Mo
3	CYTO	W5PES0	STX4	1.22E-03	0.0000	GM0_Mo
3	CYTO	W5P743	GLMP	9.30E-03	0.0000	GM0_Mo
3	CYTO	W5NZQ6	CYP1A1	4.89E-02	0.0000	GM0_Mo
3	CYTO	W5PEP1	ATP1A2	4.28E-02	0.0002	GM0_Mo
3	CYTO	W5PCS4		3.16E-04	0.0000	GM0_Mo
3	CYTO	W5NZY1	MCMBP	4.62E-02	0.0000	GM0_Mo
3	CYTO	W5Q1W2	SDCBP	4.82E-02	0.0000	GM0_Mo
3	CYTO	W5QGF4	VPS18	2.57E-03	0.0000	GM0_Mo
3	CYTO	W5PAJ2	PSAP	1.45E-03	0.0000	GM0_Mo
3	CYTO	W5PPS6		2.34E-03	0.0000	GM0_Mo
3	CYTO	W5PF85	PEA15	4.00E-03	0.0000	GM0_Mo
3	CYTO	W5NYK9	CALU	2.03E-02	0.0000	GM0_Mo
3	CYTO	W5NQZ9	GSDMD	7.02E-03	0.0000	GM0_Mo
3	CYTO	W5NU94	PSTPIP1	3.21E-02	0.0000	GM0_Mo
3	CYTO	W5P2F6	FAF1	4.63E-02	0.0000	GM0_Mo
3	CYTO	W5QGT0	ATP13A3	2.29E-02	0.0000	GM0_Mo
3	CYTO	W5Q420		1.48E-02	0.0000	GM0_Mo
3	CYTO	W5PCM4	LUZP1	8.95E-04	0.0000	GM0_Mo
3	CYTO	W5PFU8	KAT6B	2.15E-02	0.0006	GM0_Mo
3	CYTO	W5PI67	IDS	8.00E-03	0.0000	GM0_Mo
3	CYTO	W5P1Q0	AP1B1	1.17E-03	0.0000	GM0_Mo
3	CYTO	W5PYS8		4.19E-02	0.0000	GM0_Mo
3	CYTO	W5Q3Y3	SFXN3	1.35E-02	0.0000	GM0_Mo
3	CYTO	W5NUJ2	PEAK1	7.45E-03	0.0000	GM0_Mo
3	CYTO	W5PCU1	ASS1	1.87E-03	0.0000	GM0_Mo
3	CYTO	W5P333		3.08E-02	0.0000	GM0_Mo
3	CYTO	W5P3C6	LOC101111906	1.12E-03	0.0000	GM0_Mo
3	CYTO	W5PGA9	NCSTN	1.08E-02	0.0000	GM0_Mo
2	CYTO	W5PFB1	TOR1B	2.32E-02	0.0000	GM0_Mo
2	CYTO	W5NQG2	TBC1D2B	1.47E-03	0.0000	GM0_Mo
2	CYTO	W5P8F3	AGPAT5	3.57E-03	0.0000	GM0_Mo
2	CYTO	W5P7L9	KLC1	1.75E-02	0.0000	GM0_Mo
2	CYTO	W5QDQ8	MMP14	1.02E-02	0.0000	GM0_Mo
2	CYTO	W5QFD0	RRAGC	4.13E-02	0.0000	GM0_Mo
2	CYTO	W5P6U2		1.50E-02	0.0000	GM0_Mo
2	CYTO	W5P481	COL1A1	7.14E-03	0.0000	GM0_Mo
2	CYTO	W5NSS6		5.16E-02	0.0000	GM0_Mo
2	CYTO	W5PHY4	TYMS	2.27E-02	0.0000	GM0_Mo
2	CYTO	W5PQ75	HSPH1	2.22E-02	0.0000	GM0_Mo
2	CYTO	W5P2M5	LOC101114535	3.51E-02	0.0025	GM0_Mo
2	CYTO	W5NUG3	GNPDA1	2.30E-02	0.0000	GM0_Mo

Supplementary Table 7: Shared and unique differentially abundant proteins (DAPs) between MMØ and GMØ relative to M0 and their cellular location.

FoldChange	Location	Protein IDs	Gene Names	P-value	FDR	Cell type
2	CYTO	W5PS52	MRC2	1.39E-03	0.0000	GM0_Mo
2	CYTO	W5PU61	SETD7	2.85E-02	0.0000	GM0_Mo
2	CYTO	W5Q045	TMED3	4.44E-04	0.0000	GM0_Mo
2	CYTO	W5Q2H2	SLC12A9	9.83E-04	0.0000	GM0_Mo
2	CYTO	W5NUQ8	GCC1	4.03E-02	0.0000	GM0_Mo
2	CYTO	W5P8L0	ALDH18A1	3.77E-02	0.0000	GM0_Mo
2	CYTO	W5QDN8	SLC48A1	1.60E-02	0.0002	GM0_Mo
2	CYTO	W5QDR9	SLC7A7	1.40E-03	0.0000	GM0_Mo
2	CYTO	W5P434	NAGPA	6.86E-03	0.0000	GM0_Mo
2	CYTO	W5PZJ8	ASRGL1	1.40E-03	0.0000	GM0_Mo
2	CYTO	W5P726	PRCP	4.12E-03	0.0000	GM0_Mo
2	CYTO	P04074	ATP1A1	2.37E-03	0.0000	GM0_Mo
2	CYTO	W5NW41	TBC1D9B	2.63E-02	0.0000	GM0_Mo
2	CYTO	W5PUL4	MTMR6	3.97E-02	0.0000	GM0_Mo
2	CYTO	W5NV06	ATP6V0A1	5.29E-02	0.0000	GM0_Mo
2	CYTO	W5Q8J8	VPS41	3.82E-03	0.0000	GM0_Mo
2	CYTO	W5PFI1	TRAPPC6B	1.26E-02	0.0000	GM0_Mo
2	CYTO	W5Q6V7	SIPA1	4.92E-03	0.0000	GM0_Mo
2	CYTO	W5NVG2	EFEMP1	2.45E-02	0.0000	GM0_Mo
2	CYTO	W5P1U3	LACTB2	5.58E-02	0.0000	GM0_Mo
2	CYTO	W5QIJ6	SPPL2A	5.11E-02	0.0000	GM0_Mo
2	CYTO	W5P1U9	OXSM	2.35E-03	0.0000	GM0_Mo
2	CYTO	W5QCF3	SLC35F6	1.49E-02	0.0000	GM0_Mo
2	CYTO	W5Q758	DCTN5	1.92E-02	0.0000	GM0_Mo
2	CYTO	W5PAA0	RBMS1	1.17E-02	0.0002	GM0_Mo
2	CYTO	W5PFE7	ACOX1	3.36E-02	0.0000	GM0_Mo
2	CYTO	W5Q175	GNPTAB	5.32E-03	0.0000	GM0_Mo
2	CYTO	W5PMB1	SNX3	3.49E-04	0.0000	GM0_Mo
2	CYTO	W5P3H9	PICALM	1.16E-03	0.0000	GM0_Mo
2	CYTO	W5PER8	WDR91	5.20E-02	0.0000	GM0_Mo
2	CYTO	W5QBJ0	ANXA3	5.82E-03	0.0000	GM0_Mo
2	CYTO	W5PN65	PI4K2A	7.78E-03	0.0000	GM0_Mo
2	CYTO	W5Q989	PLOD1	1.47E-04	0.0000	GM0_Mo
2	CYTO	W5PWA8	HSPB1	9.04E-03	0.0000	GM0_Mo
2	CYTO	W5Q4N4	TOR3A	1.64E-02	0.0000	GM0_Mo
2	CYTO	W5PVT3	GALNS	1.80E-02	0.0000	GM0_Mo
2	CYTO	W5Q634	BLVRA	1.71E-02	0.0000	GM0_Mo
2	CYTO	W5QH69	RNF181	5.07E-02	0.0000	GM0_Mo
2	CYTO	W5Q1D8	GALNT7	3.70E-03	0.0002	GM0_Mo
2	CYTO	W5NRF7	MAP3K20	1.02E-02	0.0000	GM0_Mo
2	CYTO	W5P700	KIF1B	2.55E-02	0.0000	GM0_Mo
2	CYTO	W5Q850	AKAP10	2.22E-02	0.0000	GM0_Mo
2	CYTO	W5QGJ2	CHP1	1.38E-02	0.0000	GM0_Mo
2	CYTO	W5Q9Q5	PANK4	3.33E-02	0.0002	GM0_Mo
2	CYTO	W5Q971	DPP8	3.50E-04	0.0000	GM0_Mo
2	CYTO	W5PEX1	WASHC5	3.66E-02	0.0000	GM0_Mo
2	CYTO	W5NWV3	NECAP1	2.68E-02	0.0002	GM0_Mo
2	CYTO	W5PBJ4	ARHGAP10	3.87E-02	0.0000	GM0_Mo
2	CYTO	W5QG22	PLCB2	8.77E-04	0.0000	GM0_Mo
2	CYTO	W5PKV1	DNASE2	2.84E-02	0.0000	GM0_Mo
2	CYTO	W5Q411	HSPA13	2.42E-02	0.0000	GM0_Mo
2	CYTO	W5Q4Q8		5.74E-03	0.0000	GM0_Mo
2	CYTO	W5QII4	DMXL2	2.49E-03	0.0000	GM0_Mo
2	CYTO	W5P5E7		8.16E-03	0.0000	GM0_Mo
2	CYTO	W5PP02	MAP7D3	4.06E-03	0.0069	GM0_Mo
2	CYTO	W5PXC6		1.14E-02	0.0000	GM0_Mo
2	CYTO	W5Q9E6	TANGO2	2.59E-02	0.0000	GM0_Mo
2	CYTO	W5PQD5	DHX32	2.27E-02	0.0025	GM0_Mo
2	CYTO	W5P1U0	TXNL4A	3.86E-04	0.0000	GM0_Mo
2	CYTO	W5PQA2	PPARD	3.25E-02	0.0046	GM0_Mo
2	CYTO	W5NU07		3.85E-02	0.0000	GM0_Mo
2	CYTO	W5QAQ4	NRP2	4.24E-02	0.0000	GM0_Mo

Supplementary Table 7: Shared and unique differentially abundant proteins (DAPs) between MMØ and GMØ relative to M0 and their cellular location.

FoldChange	Location	Protein IDs	Gene Names	P-value	FDR	Cell type
2	CYTO	Q6XUZ5	IDH1	1.69E-04	0.0000	GM0_Mo
2	CYTO	W5QCV4	DHRS1	4.77E-02	0.0000	GM0_Mo
2	CYTO	W5PI02	TBC1D13	4.84E-02	0.0000	GM0_Mo
2	CYTO	W5Q6T6	SKIC3	4.49E-02	0.0000	GM0_Mo
2	CYTO	Q9XT28	ATOX1	2.71E-03	0.0000	GM0_Mo
2	CYTO	W5Q5K0		4.03E-02	0.0000	GM0_Mo
2	CYTO	W5QB79	GLCE	4.44E-02	0.0002	GM0_Mo
2	CYTO	W5Q2V0	YKT6	1.26E-03	0.0000	GM0_Mo
2	CYTO	W5Q6T1	ARSB	1.09E-02	0.0000	GM0_Mo
2	CYTO	W5NUU7	CDH5	4.35E-02	0.0000	GM0_Mo
2	CYTO	W5PF68	EMB	7.81E-03	0.0002	GM0_Mo
2	CYTO	W5QAP3	TOM1	4.88E-03	0.0000	GM0_Mo
2	CYTO	W5Q8V2	LIMA1	2.82E-02	0.0000	GM0_Mo
2	CYTO	W5Q2V2	PAH	2.81E-02	0.0000	GM0_Mo
2	CYTO	W5PTI6	ST3GAL4	2.68E-02	0.0000	GM0_Mo
2	CYTO	Q009B1	ABCG2	4.72E-02	0.0000	GM0_Mo
2	CYTO	W5PRS4	FKBP5	4.77E-02	0.0000	GM0_Mo
2	CYTO	W5Q8K4	SLC3A2	1.27E-02	0.0000	GM0_Mo
2	CYTO	W5PC06	SIRPA	1.58E-03	0.0000	GM0_Mo
2	CYTO	W5QIK3	USP8	1.61E-02	0.0000	GM0_Mo
2	CYTO	W5NUU1	VRK2	2.73E-02	0.0000	GM0_Mo
2	CYTO	W5PY53	FTH1	5.43E-02	0.0000	GM0_Mo
2	CYTO	W5NWU5	SASH1	5.48E-02	0.0000	GM0_Mo
2	CYTO	W5PPY5	CD2AP	2.66E-03	0.0000	GM0_Mo
2	CYTO	W5QAS2	STX6	1.89E-02	0.0000	GM0_Mo
2	CYTO	W5P6G7	TG	2.48E-02	0.0000	GM0_Mo
2	CYTO	W5PH81	C7	3.91E-02	0.0000	GM0_Mo
2	CYTO	W5NRR6	MGAT2	1.49E-02	0.0000	GM0_Mo
2	CYTO	W5PMY4	GABARAP	4.37E-02	0.0000	GM0_Mo
2	CYTO	W5QCD3	RNASEL	5.14E-02	0.0000	GM0_Mo
2	CYTO	W5QB00	MCM5	5.24E-02	0.0000	GM0_Mo
2	CYTO	W5PVE3	LOC101115252	1.86E-04	0.0000	GM0_Mo
2	CYTO	W5PA83	PRR14	1.02E-03	0.0045	GM0_Mo
2	CYTO	W5Q3D3	K33	2.66E-02	0.0026	GM0_Mo
2	CYTO	W5QA87	AAGAB	6.07E-03	0.0004	GM0_Mo
2	CYTO	W5NYZ6	CD81	3.47E-03	0.0000	GM0_Mo
2	CYTO	W5Q8I6	POLDIP2	3.31E-02	0.0000	GM0_Mo
2	CYTO	W5Q740	ABCD3	4.59E-02	0.0000	GM0_Mo
2	CYTO	W5Q2K9	CYFIP1	9.15E-03	0.0000	GM0_Mo
2	CYTO	W5NYG8	TSSC4	3.05E-03	0.0000	GM0_Mo
2	CYTO	W5PDU4	NMT2	9.86E-03	0.0000	GM0_Mo
2	CYTO	W5QG24	PPT1	1.26E-03	0.0000	GM0_Mo
2	CYTO	W5Q0P4	MTMR9	3.19E-03	0.0000	GM0_Mo
2	CYTO	B3GS77	IGFBP5	3.13E-02	0.0000	GM0_Mo
2	CYTO	W5PLR4	MCM6	5.53E-02	0.0000	GM0_Mo
2	CYTO	W5PAM5		5.11E-03	0.0000	GM0_Mo
2	CYTO	W5PUJ2	LAMP2	1.05E-02	0.0000	GM0_Mo
2	CYTO	W5PK12	OAT	3.53E-04	0.0000	GM0_Mo
2	CYTO	W5PQZ5	GDE1	9.25E-03	0.0002	GM0_Mo
2	CYTO	W5P1S3	SLC25A13	1.48E-02	0.0000	GM0_Mo
2	CYTO	W5PQT3	LOC101105107	5.41E-02	0.0000	GM0_Mo
2	CYTO	W5P4T0	VHL	4.38E-03	0.0002	GM0_Mo
2	CYTO	W5P6M5	SEC11C	3.95E-03	0.0019	GM0_Mo
2	CYTO	W5PDX8	FAM120A	1.16E-02	0.0000	GM0_Mo
2	CYTO	W5Q0M7	RAP2B	8.65E-04	0.0000	GM0_Mo
2	CYTO	W5P8S8	ALDH1L1	3.06E-02	0.0000	GM0_Mo
2	CYTO	W5Q7Z7	DSP	1.14E-02	0.0000	GM0_Mo
2	CYTO	W5PMC5	LOC101110546	3.11E-02	0.0000	GM0_Mo
2	CYTO	W5Q1P6	STX2	6.81E-03	0.0000	GM0_Mo
2	CYTO	W5QC34	MAN2A1	1.11E-02	0.0000	GM0_Mo
2	CYTO	W5P164	LAMTOR1	3.49E-02	0.0000	GM0_Mo
2	CYTO	W5Q131		2.46E-02	0.0002	GM0_Mo

Supplementary Table 7: Shared and unique differentially abundant proteins (DAPs) between MMØ and GMØ relative to M0 and their cellular location.

FoldChange	Location	Protein IDs	Gene Names	P-value	FDR	Cell type
2	CYTO	W5PA59	LOC101117015 LOC1	3.53E-03	0.0000	GM0_Mo
2	CYTO	W5P001	RUFY1	4.16E-03	0.0000	GM0_Mo
2	CYTO	W5QEA3		1.24E-03	0.0000	GM0_Mo
2	CYTO	W5PUU2	RRAS	2.50E-02	0.0000	GM0_Mo
2	CYTO	W5Q928	PCYT2	2.85E-02	0.0000	GM0_Mo
2	CYTO	W5PUW5	GOLGA5	4.44E-02	0.0000	GM0_Mo
2	CYTO	W5PJW1	SDHC	3.12E-02	0.0000	GM0_Mo
2	CYTO	W5PLB6	SPART	1.24E-02	0.0000	GM0_Mo
2	CYTO	W5PEE6	ITGA2	4.47E-02	0.0062	GM0_Mo
2	CYTO	W5P906	DPP4	1.74E-02	0.0059	GM0_Mo
2	CYTO	W5QH50	HRG	1.09E-03	0.0000	GM0_Mo
2	CYTO	W5PUR2	PRKCQ	3.14E-02	0.0012	GM0_Mo
2	CYTO	W5Q9H0	MMP2	1.45E-02	0.0002	GM0_Mo
2	CYTO	W5PNW7	VIM	6.77E-04	0.0000	GM0_Mo
2	CYTO	W5Q824	GSTA1	5.22E-02	0.0000	GM0_Mo
2	CYTO	W5PUI3	GOLGA1	4.16E-02	0.0000	GM0_Mo
2	CYTO	W5PCK8	LOC100135455	1.79E-02	0.0000	GM0_Mo
2	CYTO	W5NYV5	SPAG9	1.42E-02	0.0000	GM0_Mo
2	CYTO	W5Q3J3	COLGALT1	1.70E-02	0.0000	GM0_Mo
2	CYTO	W5QAE8	ALDH7A1	8.35E-06	0.0000	GM0_Mo
2	CYTO	W5PH58		5.24E-02	0.0000	GM0_Mo
2	CYTO	W5P8B4	TRAPPC8	1.29E-02	0.0000	GM0_Mo
2	CYTO	W5PRJ7	CCDC93	1.34E-02	0.0000	GM0_Mo
2	CYTO	W5P983	COL4A2	1.73E-02	0.0002	GM0_Mo
2	CYTO	W5P5A7	VPS16	2.32E-03	0.0000	GM0_Mo
2	CYTO	W5PB20		1.93E-02	0.0000	GM0_Mo
2	CYTO	W5NQG1	SETD3	6.80E-03	0.0000	GM0_Mo
2	CYTO	W5QHA0	AGFG1	2.90E-04	0.0000	GM0_Mo
2	CYTO	W5P5H5	CLPB	3.56E-02	0.0000	GM0_Mo
2	CYTO	W5Q9H8	SNX5	2.81E-02	0.0000	GM0_Mo
2	CYTO	W5Q5B5	CSAD	1.96E-03	0.0000	GM0_Mo
2	CYTO	W5P089	LOC101115969	1.68E-02	0.0000	GM0_Mo
2	CYTO	W5PPG2	DYSF	4.01E-02	0.0000	GM0_Mo
2	CYTO	W5PFW1	GAN	5.62E-03	0.0000	GM0_Mo
2	CYTO	W5PRQ0	RBM3	1.76E-02	0.0000	GM0_Mo
2	CYTO	W5QAL6	FMNL3	5.57E-03	0.0002	GM0_Mo
2	CYTO	W5PCN2	ANXA7	4.18E-03	0.0000	GM0_Mo
2	CYTO	W5QCL1	NCF4	1.51E-03	0.0000	GM0_Mo
2	CYTO	W5PJV4	ASGR2	4.73E-02	0.0000	GM0_Mo
2	CYTO	W5NYE8	SEL1L	5.12E-02	0.0000	GM0_Mo
2	CYTO	W5PXK7	RASA4B	3.63E-02	0.0000	GM0_Mo
2	CYTO	W5PJY6	ADAM28	3.04E-02	0.0000	GM0_Mo
2	CYTO	W5NY29		5.24E-03	0.0000	GM0_Mo
2	CYTO	W5PSD7	RAP2C	5.46E-02	0.0000	GM0_Mo
2	CYTO	W5PUM8	HIP1	3.59E-02	0.0000	GM0_Mo
2	CYTO	W5PSX7	FES	3.11E-02	0.0000	GM0_Mo
2	CYTO	W5PRK6	STAU1	5.50E-02	0.0000	GM0_Mo
2	CYTO	W5Q0F3	TGFBI	4.52E-02	0.0000	GM0_Mo
2	CYTO	W5PS60		4.65E-02	0.0002	GM0_Mo
2	CYTO	W5NUE1	NCF2	2.24E-02	0.0000	GM0_Mo
2	CYTO	W5P7P6	MYO18A	2.86E-03	0.0000	GM0_Mo
2	CYTO	W5Q7R9	LAMTOR4	1.63E-02	0.0000	GM0_Mo
2	CYTO	W5PSG0		3.96E-02	0.0000	GM0_Mo
2	CYTO	W5PMR2	MMP19	2.94E-02	0.0000	GM0_Mo
2	CYTO	W5PKP3	FAM114A1	1.77E-02	0.0000	GM0_Mo
2	CYTO	W5NUZ1	DYNC1I2	1.12E-02	0.0000	GM0_Mo
2	CYTO	W5PEN2	TPD52	2.39E-02	0.0000	GM0_Mo
2	CYTO	W5P6U5	LMAN1	7.29E-03	0.0000	GM0_Mo
2	CYTO	W5NYA8	SCAMP2	4.91E-02	0.0000	GM0_Mo
2	CYTO	W5PGV0	ITGAM	8.70E-03	0.0000	GM0_Mo
2	CYTO	Q6ECI6	ITGB2 CD18	1.03E-04	0.0000	GM0_Mo
2	CYTO	W5P2N8	PIK3C2A	3.18E-03	0.0002	GM0_Mo

Supplementary Table 7: Shared and unique differentially abundant proteins (DAPs) between MMØ and GMØ relative to M0 and their cellular location.

FoldChange	Location	Protein IDs	Gene Names	P-value	FDR	Cell type
2	CYTO	W5NY14		3.80E-04	0.0000	GM0_Mo
-2	CYTO	W5NW82	NAP1L4	8.58E-04	0.0000	GM0_Mo
-2	CYTO	W5Q1I8	PPM1F	5.32E-03	0.0000	GM0_Mo
-2	CYTO	W5PXS7	DAPP1	1.27E-02	0.0000	GM0_Mo
-2	CYTO	W5PQI0	PXN	1.66E-02	0.0000	GM0_Mo
-2	CYTO	W5Q732	PDK3	1.12E-02	0.0004	GM0_Mo
-2	CYTO	W5PCQ8	SLC25A4	1.90E-02	0.0000	GM0_Mo
-2	CYTO	W5PXW3	OSBPL2	1.35E-03	0.0036	GM0_Mo
-2	CYTO	W5PGV7	UBQLN1	1.37E-02	0.0000	GM0_Mo
-2	CYTO	W5QFQ8	LOC101111915	1.42E-02	0.0000	GM0_Mo
-2	CYTO	W5Q992	MYO9B	1.72E-02	0.0000	GM0_Mo
-2	CYTO	W5PC19	STX18	2.31E-02	0.0002	GM0_Mo
-2	CYTO	W5PJA0	CUTA	1.37E-02	0.0000	GM0_Mo
-2	CYTO	W5QJ24	PPP2R5E	1.08E-02	0.0000	GM0_Mo
-2	CYTO	W5Q9P1	CTNNBL1	4.56E-02	0.0000	GM0_Mo
-2	CYTO	W5PXT1	MYO1G	3.10E-02	0.0000	GM0_Mo
-2	CYTO	W5QDM6	STRIP1	2.08E-02	0.0000	GM0_Mo
-2	CYTO	W5P7E2	PPA1	1.40E-02	0.0000	GM0_Mo
-2	CYTO	W5PTU8	MARK1	2.89E-02	0.0089	GM0_Mo
-2	CYTO	W5P090		1.01E-02	0.0000	GM0_Mo
-2	CYTO	W5PID5	TSG101	6.07E-03	0.0000	GM0_Mo
-2	CYTO	W5PCE9	MICOS13	1.42E-02	0.0000	GM0_Mo
-2	CYTO	W5Q9Z8	PHAX	4.09E-02	0.0000	GM0_Mo
-2	CYTO	W5PUV1	DCAF7	2.09E-03	0.0000	GM0_Mo
-2	CYTO	W5NX40	TAB2	1.39E-02	0.0000	GM0_Mo
-2	CYTO	W5PDK7	PCCB	2.86E-02	0.0000	GM0_Mo
-2	CYTO	W5NTH2	EIF2B3	4.65E-02	0.0000	GM0_Mo
-2	CYTO	W5QAP8	RPRD1B	1.23E-02	0.0000	GM0_Mo
-2	CYTO	W5PXJ6	ANKRD13A	5.74E-03	0.0000	GM0_Mo
-2	CYTO	W5P033	COG8	3.18E-03	0.0045	GM0_Mo
-2	CYTO	W5Q1V2	ZNRD2	7.65E-03	0.0000	GM0_Mo
-2	CYTO	W5P031	HDHD2	2.03E-02	0.0000	GM0_Mo
-2	CYTO	W5PQ88	RPS6KB2	1.44E-02	0.0000	GM0_Mo
-2	CYTO	W5NUT8	PIP4K2A	3.24E-02	0.0000	GM0_Mo
-2	CYTO	W5PLK6	SHOC2	1.25E-02	0.0002	GM0_Mo
-2	CYTO	W5PHE6	DDI2	1.07E-03	0.0000	GM0_Mo
-2	CYTO	W5PKR1		4.68E-02	0.0000	GM0_Mo
-2	CYTO	W5P2Q7	HIKESHI	1.80E-02	0.0000	GM0_Mo
-2	CYTO	W5Q2R9		3.96E-02	0.0000	GM0_Mo
-2	CYTO	W5PEE7	PDP1	4.19E-03	0.0000	GM0_Mo
-2	CYTO	W5NPP7	DRAP1	4.68E-02	0.0004	GM0_Mo
-2	CYTO	W5PK27		5.11E-02	0.0000	GM0_Mo
-2	CYTO	W5QFV9	PPIE	4.73E-02	0.0002	GM0_Mo
-2	CYTO	W5QIC3	PRUNE1	6.05E-03	0.0000	GM0_Mo
-2	CYTO	W5NPT4	LOC101116298	1.17E-02	0.0000	GM0_Mo
-2	CYTO	W5PVX8	PPP3CA	1.83E-02	0.0000	GM0_Mo
-2	CYTO	W5QF78		5.40E-04	0.0000	GM0_Mo
-2	CYTO	W5PMM8	GGA3	9.96E-04	0.0000	GM0_Mo
-2	CYTO	W5NY88	PSMD10	5.39E-03	0.0000	GM0_Mo
-2	CYTO	W5PTB3	INPP5K	3.47E-04	0.0002	GM0_Mo
-2	CYTO	W5NSV7	BET1	8.41E-03	0.0000	GM0_Mo
-2	CYTO	W5Q1I6	IMPDH2 IMPDH	4.34E-02	0.0000	GM0_Mo
-2	CYTO	W5QB23		2.39E-02	0.0000	GM0_Mo
-2	CYTO	C5IWU4	ARL3	2.56E-02	0.0000	GM0_Mo
-2	CYTO	W5QJ62	ACTN1	5.10E-02	0.0000	GM0_Mo
-2	CYTO	C8BKC5	PRDX2	3.89E-02	0.0000	GM0_Mo
-2	CYTO	W5PJP6	SMARCC2	5.60E-02	0.0000	GM0_Mo
-2	CYTO	W5Q3F6	ELP4	5.58E-02	0.0002	GM0_Mo
-2	CYTO	W5P762		2.93E-02	0.0000	GM0_Mo
-2	CYTO	W5PT31		2.27E-02	0.0000	GM0_Mo
-2	CYTO	W5NWI4		5.40E-03	0.0000	GM0_Mo
-2	CYTO	W5NUV2	ARF5	1.68E-02	0.0000	GM0_Mo

Supplementary Table 7: Shared and unique differentially abundant proteins (DAPs) between MMØ and GMØ relative to M0 and their cellular location.

FoldChange	Location	Protein IDs	Gene Names	P-value	FDR	Cell type
-2	CYTO	W5P4W0	TSFM	1.32E-02	0.0000	GM0_Mo
-2	CYTO	W5PFV0	HMGCS1	4.67E-02	0.0000	GM0_Mo
-2	CYTO	W5PMI8	CUL5	3.71E-03	0.0000	GM0_Mo
-2	CYTO	C5ISA4	COPS7B	1.89E-02	0.0000	GM0_Mo
-2	CYTO	W5P4C5	RBM42	2.00E-02	0.0000	GM0_Mo
-2	CYTO	W5QHL0	ATP11B	3.81E-02	0.0000	GM0_Mo
-2	CYTO	W5NV31	COPS3	5.30E-02	0.0000	GM0_Mo
-2	CYTO	W5P1E2	CNST	1.08E-02	0.0000	GM0_Mo
-2	CYTO	W5PQW2	MOB1B	9.55E-03	0.0000	GM0_Mo
-2	CYTO	W5PWH9	PRKACB	2.06E-03	0.0000	GM0_Mo
-2	CYTO	W5PEV7	DYNLL2	3.65E-02	0.0000	GM0_Mo
-2	CYTO	W5PZB6	ETFB	9.49E-03	0.0000	GM0_Mo
-2	CYTO	W5PPE2	MGST3	5.58E-02	0.0000	GM0_Mo
-2	CYTO	W5PQK6	TLN1	3.25E-02	0.0000	GM0_Mo
-2	CYTO	W5PYM3	CAAP1	1.22E-02	0.0012	GM0_Mo
-2	CYTO	W5PUR1	CNOT11	9.07E-03	0.0000	GM0_Mo
-2	CYTO	W5QCZ0	MPST	2.01E-03	0.0000	GM0_Mo
-2	CYTO	W5Q7Q9	PFDN1	3.05E-02	0.0004	GM0_Mo
-2	CYTO	W5PUU8	LOC101122645	2.13E-02	0.0000	GM0_Mo
-2	CYTO	W5PTZ8	LOC101114959	3.98E-03	0.0000	GM0_Mo
-2	CYTO	W5QCI3	LOC101106419	3.47E-03	0.0000	GM0_Mo
-2	CYTO	W5P0R7	DHODH	1.53E-02	0.0002	GM0_Mo
-2	CYTO	W5QHP6	ARHGDIB	4.03E-04	0.0000	GM0_Mo
-2	CYTO	W5PJQ5	HEATR5B	1.58E-02	0.0000	GM0_Mo
-2	CYTO	W5PCV5	RALGAPA1	4.89E-02	0.0084	GM0_Mo
-2	CYTO	W5QGW0	PPIH	4.65E-02	0.0000	GM0_Mo
-2	CYTO	W5PY73	SF3A2	3.03E-02	0.0000	GM0_Mo
-2	CYTO	W5P7C2	SEPHS1	4.05E-02	0.0000	GM0_Mo
-2	CYTO	W5PHD7	WBP2	3.56E-03	0.0025	GM0_Mo
-2	CYTO	W5Q4J3	RAPGEF6	8.13E-03	0.0092	GM0_Mo
-2	CYTO	W5NS43	ALDH6A1	4.74E-03	0.0000	GM0_Mo
-2	CYTO	W5NZA5	DGKZ	3.66E-02	0.0000	GM0_Mo
-2	CYTO	W5QB24	ANP32A	2.33E-02	0.0000	GM0_Mo
-2	CYTO	W5Q5I7	NFYB	5.54E-02	0.0000	GM0_Mo
-2	CYTO	W5PL66	PPA2	7.27E-03	0.0000	GM0_Mo
-2	CYTO	W5QDF0	NRBP1	1.09E-02	0.0000	GM0_Mo
-2	CYTO	W5PME9	ARHGAP45	3.13E-02	0.0000	GM0_Mo
-2	CYTO	W5QCC1	STAT5B	6.97E-03	0.0000	GM0_Mo
-2	CYTO	W5Q5M7	SLC44A2	1.70E-02	0.0000	GM0_Mo
-2	CYTO	W5PXI2		5.21E-02	0.0000	GM0_Mo
-2	CYTO	W5Q4D5	BCO2	2.73E-02	0.0000	GM0_Mo
-2	CYTO	W5PMY9		3.85E-02	0.0000	GM0_Mo
-2	CYTO	W5Q8P3	USE1	5.00E-03	0.0000	GM0_Mo
-2	CYTO	W5PMC9	ARHGAP6	1.22E-02	0.0071	GM0_Mo
-2	CYTO	W5PYE5	NUDCD1	4.06E-02	0.0000	GM0_Mo
-2	CYTO	Q2TCH3	ACLY	2.35E-04	0.0000	GM0_Mo
-2	CYTO	W5PTS4	LOC101114275	2.83E-02	0.0000	GM0_Mo
-2	CYTO	W5PUB1	THUMPD1	4.54E-02	0.0000	GM0_Mo
-2	CYTO	W5NWE0	MECR	2.86E-02	0.0000	GM0_Mo
-2	CYTO	W5PN94	LUC7L	1.72E-02	0.0000	GM0_Mo
-2	CYTO	W5QIF1	MYO5A	1.23E-02	0.0000	GM0_Mo
-2	CYTO	W5PS76	UNC45A	6.30E-03	0.0000	GM0_Mo
-2	CYTO	W5Q2I3	YME1L1	8.60E-03	0.0000	GM0_Mo
-2	CYTO	W5NX76	MAP2K3	1.40E-02	0.0000	GM0_Mo
-2	CYTO	W5NZ21	LOC101105090	1.12E-02	0.0000	GM0_Mo
-2	CYTO	W5PK25	BAK1	4.75E-02	0.0000	GM0_Mo
-2	CYTO	W5Q7H5	HVCN1	3.00E-02	0.0000	GM0_Mo
-2	CYTO	W5Q1Q3	ARL14EP	5.87E-03	0.0000	GM0_Mo
-2	CYTO	W5PC20	VPS37B	1.20E-02	0.0000	GM0_Mo
-2	CYTO	W5Q8F8	CPOX	2.01E-02	0.0000	GM0_Mo
-2	CYTO	W5NRI1		2.02E-02	0.0002	GM0_Mo
-2	CYTO	W5PKK8	ESYT1	1.82E-03	0.0000	GM0_Mo

Supplementary Table 7: Shared and unique differentially abundant proteins (DAPs) between MMØ and GMØ relative to M0 and their cellular location.

FoldChange	Location	Protein IDs	Gene Names	P-value	FDR	Cell type
-2	CYTO	W5P0J5	ZNF22	1.59E-02	0.0002	GM0_Mo
-2	CYTO	W5PLB1	CLIC5	3.62E-02	0.0000	GM0_Mo
-2	CYTO	W5P0R4	CTIF	7.37E-04	0.0004	GM0_Mo
-2	CYTO	W5PUS0	CUL4B	4.81E-02	0.0000	GM0_Mo
-2	CYTO	W5PHB6	SF1	2.88E-02	0.0000	GM0_Mo
-2	CYTO	W5QIH2	TMOD3	5.13E-03	0.0000	GM0_Mo
-2	CYTO	W5Q5Q8	WDR44	3.42E-02	0.0000	GM0_Mo
-2	CYTO	W5Q198	QRICH1	1.10E-02	0.0002	GM0_Mo
-2	CYTO	W5PY18	LOC101118100	2.39E-02	0.0000	GM0_Mo
-2	CYTO	W5PLD5	CNN2	1.67E-03	0.0000	GM0_Mo
-2	CYTO	W5Q4G2		5.19E-03	0.0000	GM0_Mo
-2	CYTO	W5PRA0	BPNT1	1.83E-02	0.0000	GM0_Mo
-2	CYTO	W5PZC3	STAMBPL1	4.08E-02	0.0000	GM0_Mo
-2	CYTO	W5Q9C5	LNPEP	1.12E-02	0.0000	GM0_Mo
-2	CYTO	W5Q6F2	ARMC1	1.38E-02	0.0000	GM0_Mo
-2	CYTO	W5NRG0	PTGR2	4.15E-02	0.0000	GM0_Mo
-2	CYTO	W5Q9B1	ATP2A3	1.24E-02	0.0000	GM0_Mo
-2	CYTO	W5PTJ0	CRLF3	6.95E-03	0.0000	GM0_Mo
-2	CYTO	W5Q0B6	PPP1R12A	2.24E-03	0.0000	GM0_Mo
-2	CYTO	W5PUU6	RPL35	3.97E-02	0.0000	GM0_Mo
-2	CYTO	W5P9F0	ARHGAP9	1.70E-02	0.0000	GM0_Mo
-2	CYTO	W5NSI2	RALBP1	2.25E-02	0.0085	GM0_Mo
-2	CYTO	W5NTA4	CGGBP1	1.46E-02	0.0000	GM0_Mo
-2	CYTO	W5Q6U0	FASN	3.93E-02	0.0000	GM0_Mo
-2	CYTO	W5PQ04	DEF6	3.14E-03	0.0000	GM0_Mo
-2	CYTO	W5PK62	CDC40	3.30E-02	0.0000	GM0_Mo
-2	CYTO	W5PB28	CLNS1A	2.60E-02	0.0000	GM0_Mo
-2	CYTO	W5QHX2	RPL22L1	4.90E-02	0.0000	GM0_Mo
-2	CYTO	W5PSX3	DARS2	1.21E-02	0.0002	GM0_Mo
-2	CYTO	W5PUU9	HDHD5	3.42E-02	0.0000	GM0_Mo
-2	CYTO	W5P9A0	ALDH5A1	7.59E-03	0.0000	GM0_Mo
-2	CYTO	W5Q595	NIF3L1	2.68E-02	0.0000	GM0_Mo
-2	CYTO	W5PZ59	LOC101104574	3.87E-02	0.0000	GM0_Mo
-2	CYTO	W5Q3M9	SEPTIN6	9.26E-03	0.0000	GM0_Mo
-2	CYTO	W5P948	INIP	4.45E-03	0.0000	GM0_Mo
-2	CYTO	W5Q4I7	LOC101123619	1.50E-02	0.0012	GM0_Mo
-2	CYTO	W5NU48	CRTC2	6.31E-03	0.0019	GM0_Mo
-2	CYTO	W5QEK8	EHD3	3.41E-02	0.0000	GM0_Mo
-2	CYTO	W5NWQ0	TMED8	1.99E-02	0.0000	GM0_Mo
-2	CYTO	W5PEA0	FGD3	5.34E-02	0.0000	GM0_Mo
-2	CYTO	W5PTQ0	RNGTT	1.43E-02	0.0002	GM0_Mo
-2	CYTO	W5QHP4	ACTL6A	1.49E-02	0.0000	GM0_Mo
-2	CYTO	W5PQQ5	DIDO1	4.49E-02	0.0000	GM0_Mo
-2	CYTO	W5PRR5	LTA4H	2.71E-04	0.0000	GM0_Mo
-2	CYTO	W5P4U4	GRK6	4.23E-04	0.0000	GM0_Mo
-2	CYTO	W5PKD2	GCDH	2.50E-02	0.0000	GM0_Mo
-2	CYTO	W5PR48	HPRT1	1.16E-02	0.0000	GM0_Mo
-2	CYTO	W5PTN4	TMPO	2.68E-03	0.0000	GM0_Mo
-2	CYTO	W5PTM6	CPSF2	1.09E-02	0.0000	GM0_Mo
-2	CYTO	W5PGD6	BZW2	8.48E-04	0.0000	GM0_Mo
-3	CYTO	W5Q2C6		4.90E-02	0.0002	GM0_Mo
-3	CYTO	W5PHL8	NUDT16	2.15E-03	0.0000	GM0_Mo
-3	CYTO	W5Q9J0	RNPS1	1.76E-02	0.0000	GM0_Mo
-3	CYTO	W5PSQ7		1.25E-02	0.0000	GM0_Mo
-3	CYTO	C8BKD4	CBX5	1.70E-02	0.0000	GM0_Mo
-3	CYTO	W5PRJ3	HYCC1	3.81E-02	0.0000	GM0_Mo
-3	CYTO	W5Q3J5	IWS1	4.01E-02	0.0000	GM0_Mo
-3	CYTO	W5PV48	USP11	1.95E-03	0.0000	GM0_Mo
-3	CYTO	W5P668		4.87E-02	0.0002	GM0_Mo
-3	CYTO	Q5MIB6	PYGB	4.78E-02	0.0000	GM0_Mo
-3	CYTO	W5P7A6	ATE1	3.36E-03	0.0000	GM0_Mo
-3	CYTO	W5Q9N1	LPCAT2	1.15E-03	0.0004	GM0_Mo

Supplementary Table 7: Shared and unique differentially abundant proteins (DAPs) between MMØ and GMØ relative to M0 and their cellular location.

FoldChange	Location	Protein IDs	Gene Names	P-value	FDR	Cell type
-3	CYTO	W5PZJ7		4.06E-03	0.0000	GM0_Mo
-3	CYTO	W5PJW9	SAP30BP	2.28E-02	0.0000	GM0_Mo
-3	CYTO	W5NXF6	TUBG1	4.15E-02	0.0000	GM0_Mo
-3	CYTO	W5Q9R2	SARM1	4.30E-02	0.0000	GM0_Mo
-3	CYTO	W5Q3Q2	ERCC3	5.66E-03	0.0041	GM0_Mo
-3	CYTO	W5P524	CC2D1B	2.16E-02	0.0000	GM0_Mo
-3	CYTO	W5PA22	ACOT8	2.79E-04	0.0000	GM0_Mo
-3	CYTO	W5PK38	VASP	3.18E-02	0.0000	GM0_Mo
-3	CYTO	W5PFJ5	SMC1A	1.60E-02	0.0000	GM0_Mo
-3	CYTO	W5P5F0	TMEM106A	4.55E-02	0.0000	GM0_Mo
-3	CYTO	W5Q5Y1	ITGA4	4.97E-06	0.0002	GM0_Mo
-3	CYTO	W5PL05	MICAL1	1.78E-04	0.0000	GM0_Mo
-3	CYTO	O78750	MT-CO2 COII COX2 C	1.95E-02	0.0000	GM0_Mo
-3	CYTO	W5PDD5	STAG1	3.81E-02	0.0000	GM0_Mo
-3	CYTO	W5PGB0	SIRT3	2.43E-02	0.0000	GM0_Mo
-3	CYTO	W5PSA9	VPS13A	1.51E-02	0.0000	GM0_Mo
-3	CYTO	W5QHX6	ANP32E	4.56E-02	0.0000	GM0_Mo
-3	CYTO	W5PAF9	KIF2A	1.72E-02	0.0000	GM0_Mo
-3	CYTO	W5QH77	USP39	5.28E-03	0.0000	GM0_Mo
-3	CYTO	W5NQ72	PFAS	1.30E-03	0.0000	GM0_Mo
-3	CYTO	P80190		1.37E-02	0.0000	GM0_Mo
-3	CYTO	W5PPX0	LYPLAL1	3.89E-03	0.0000	GM0_Mo
-3	CYTO	W5NRP8	MTCL1	3.57E-02	0.0008	GM0_Mo
-3	CYTO	W5P2J9		3.10E-03	0.0000	GM0_Mo
-3	CYTO	W5P432	NIPSNAP1	5.12E-03	0.0000	GM0_Mo
-3	CYTO	W5PQT2	TPRKB	4.21E-02	0.0000	GM0_Mo
-3	CYTO	W5PMJ7	NADK2	1.38E-02	0.0000	GM0_Mo
-3	CYTO	W5NSP0		2.48E-03	0.0000	GM0_Mo
-3	CYTO	W5PZP8	FIS1	5.47E-03	0.0000	GM0_Mo
-3	CYTO	W5PXR8	POLR2J	5.96E-03	0.0000	GM0_Mo
-3	CYTO	W5Q804	SPECC1	1.10E-03	0.0000	GM0_Mo
-3	CYTO	W5Q0P0	GABPA	5.46E-02	0.0000	GM0_Mo
-3	CYTO	W5PIK1	LZTFL1	5.43E-02	0.0000	GM0_Mo
-3	CYTO	W5NZW5	WDFY3	5.27E-02	0.0000	GM0_Mo
-3	CYTO	W5Q7L1	CNN3	9.38E-03	0.0000	GM0_Mo
-3	CYTO	W5QIY4	ARID4A	7.64E-03	0.0000	GM0_Mo
-3	CYTO	W5PX66	TIAM1	5.49E-03	0.0000	GM0_Mo
-3	CYTO	W5Q366	SUN2	3.48E-02	0.0000	GM0_Mo
-3	CYTO	W5P7P9	ERBIN	4.42E-03	0.0000	GM0_Mo
-3	CYTO	W5PKA9	F5	1.27E-02	0.0000	GM0_Mo
-3	CYTO	W5Q600	TSC22D4	3.09E-02	0.0000	GM0_Mo
-3	CYTO	W5QGW8	PDE3A	5.12E-02	0.0000	GM0_Mo
-3	CYTO	W5P5N5		1.39E-03	0.0002	GM0_Mo
-3	CYTO	W5PLX5	PRKD2	1.90E-04	0.0000	GM0_Mo
-3	CYTO	W5P0N1	DHX38	1.49E-02	0.0010	GM0_Mo
-3	CYTO	W5PHA6	TP53RK	5.03E-02	0.0002	GM0_Mo
-3	CYTO	W5P955	NT5C3A	5.59E-02	0.0000	GM0_Mo
-3	CYTO	W5P225	PDCD5	4.62E-03	0.0000	GM0_Mo
-3	CYTO	W5NYK3	CTCF	5.07E-02	0.0000	GM0_Mo
-3	CYTO	W5QGB5	LOC101113369	8.49E-04	0.0000	GM0_Mo
-3	CYTO	W5NWU0	LOC101122262	3.88E-02	0.0000	GM0_Mo
-3	CYTO	W5Q4L7	AP3S1	2.33E-03	0.0000	GM0_Mo
-3	CYTO	W5QAK5	PIAS1	1.33E-02	0.0002	GM0_Mo
-3	CYTO	W5PJ14	ESYT2	2.65E-03	0.0000	GM0_Mo
-3	CYTO	W5Q2V1		2.49E-02	0.0000	GM0_Mo
-3	CYTO	W5QHL5	CAB39	2.33E-03	0.0000	GM0_Mo
-3	CYTO	W5PIW6	PRTN3	4.55E-02	0.0000	GM0_Mo
-3	CYTO	W5QFR4	LOC101112856	2.84E-02	0.0000	GM0_Mo
-3	CYTO	W5PW06	PNKP	1.71E-02	0.0000	GM0_Mo
-3	CYTO	W5PUI4	RAB3A	3.53E-04	0.0002	GM0_Mo
-3	CYTO	W5PHI5	PALM	1.99E-02	0.0000	GM0_Mo
-3	CYTO	W5QFP2	LOC101109397	1.97E-02	0.0000	GM0_Mo

Supplementary Table 7: Shared and unique differentially abundant proteins (DAPs) between MMØ and GMØ relative to M0 and their cellular location.

FoldChange	Location	Protein IDs	Gene Names	P-value	FDR	Cell type
-3	CYTO	W5Q8H0	SSH1	3.40E-02	0.0000	GM0_Mo
-3	CYTO	W5QCX7	MYBBP1A	4.71E-02	0.0000	GM0_Mo
-3	CYTO	W5QD23	LANCL1	1.20E-02	0.0000	GM0_Mo
-3	CYTO	W5QH43	EIF4A2	2.09E-02	0.0000	GM0_Mo
-3	CYTO	W5Q8M9	PYCR2	1.52E-03	0.0000	GM0_Mo
-3	CYTO	W5QIK2	RFX5	6.98E-03	0.0000	GM0_Mo
-3	CYTO	W5PVX3	PRKG1	2.01E-02	0.0000	GM0_Mo
-3	CYTO	W5NTN7	PNPLA6	2.06E-02	0.0000	GM0_Mo
-3	CYTO	W5PCZ2	PPP3CB	1.57E-04	0.0000	GM0_Mo
-3	CYTO	W5PP85	TBC1D10C	1.55E-02	0.0000	GM0_Mo
-3	CYTO	C0IZ95	RAB27A	1.29E-02	0.0000	GM0_Mo
-3	CYTO	W5P433	BRCA1	1.07E-03	0.0037	GM0_Mo
-3	CYTO	W5Q3J8		4.50E-02	0.0000	GM0_Mo
-3	CYTO	W5QJ56	RDH11	3.41E-02	0.0000	GM0_Mo
-3	CYTO	W5P7P8	GCA	1.72E-02	0.0000	GM0_Mo
-3	CYTO	C5IS96	LCAT	2.39E-02	0.0002	GM0_Mo
-3	CYTO	W5Q3I7	TUBB1	4.57E-02	0.0000	GM0_Mo
-3	CYTO	W5QHY6	RPIA	3.79E-03	0.0000	GM0_Mo
-3	CYTO	W5NUS2	MED9	1.75E-02	0.0002	GM0_Mo
-3	CYTO	W5P691	CNTRL	3.32E-02	0.0000	GM0_Mo
-3	CYTO	W5NTW9	RIPK1	2.17E-02	0.0000	GM0_Mo
-3	CYTO	W5PAV5	RFX1	1.19E-02	0.0000	GM0_Mo
-3	CYTO	W5NUE6	PLEKHA2	1.10E-03	0.0000	GM0_Mo
-3	CYTO	W5PAV0	STAP1	1.42E-02	0.0000	GM0_Mo
-3	CYTO	W5NUX2		8.86E-03	0.0000	GM0_Mo
-3	CYTO	W5PGX7	SSRP1	1.94E-02	0.0000	GM0_Mo
-3	CYTO	W5Q878	PRKCB	5.44E-03	0.0000	GM0_Mo
-3	CYTO	W5P795	LRBA	2.45E-02	0.0000	GM0_Mo
-3	CYTO	W5NTQ3	H3-4	1.40E-04	0.0002	GM0_Mo
-3	CYTO	W5PG02	RASA3	3.83E-02	0.0000	GM0_Mo
-3	CYTO	W5QBV1	FER	8.61E-03	0.0000	GM0_Mo
-3	CYTO	W5PZ86	SEPTIN8	3.47E-02	0.0000	GM0_Mo
-3	CYTO	W5Q1C4	DNMT1	5.24E-03	0.0010	GM0_Mo
-3	CYTO	W5QFM2		3.91E-05	0.0000	GM0_Mo
-3	CYTO	W5QFT3		1.67E-02	0.0000	GM0_Mo
-3	CYTO	W5Q6S0	LBR	4.60E-03	0.0000	GM0_Mo
-3	CYTO	W5Q059	SASH3	4.91E-02	0.0000	GM0_Mo
-4	CYTO	W5NTV6		3.19E-05	0.0000	GM0_Mo
-4	CYTO	W5PV67	LIMD2	1.83E-02	0.0000	GM0_Mo
-4	CYTO	W5P8I8	PITPNM2	1.67E-03	0.0000	GM0_Mo
-4	CYTO	W5NRR9	STAT5A	2.84E-03	0.0000	GM0_Mo
-4	CYTO	W5P9M9	LOC101103862	3.10E-02	0.0000	GM0_Mo
-4	CYTO	W5Q6R6	SHPK	9.40E-03	0.0000	GM0_Mo
-4	CYTO	W5PZU8	ASAP2	2.20E-02	0.0000	GM0_Mo
-4	CYTO	W5Q088	CBL	1.21E-03	0.0000	GM0_Mo
-4	CYTO	W5PY97	SVIL	1.10E-03	0.0000	GM0_Mo
-4	CYTO	W5PIN7		2.65E-02	0.0000	GM0_Mo
-4	CYTO	W5PYK8	VNN2	2.12E-03	0.0000	GM0_Mo
-4	CYTO	W5P3Y9	CD22	3.56E-03	0.0000	GM0_Mo
-4	CYTO	W5PI65	MAP4K2	3.74E-05	0.0000	GM0_Mo
-4	CYTO	W5P4F9	PRKAR2B	1.66E-02	0.0000	GM0_Mo
-4	CYTO	W5Q2W4	RAVER1	2.47E-02	0.0000	GM0_Mo
-4	CYTO	W5PRM0	GMPR	5.44E-02	0.0000	GM0_Mo
-4	CYTO	W5PT20	ZC3H18	2.92E-02	0.0000	GM0_Mo
-4	CYTO	W5NXV1	PACS1	7.35E-03	0.0000	GM0_Mo
-4	CYTO	W5PZL2		3.57E-02	0.0000	GM0_Mo
-4	CYTO	W5QHL6	ITM2C	1.00E-03	0.0000	GM0_Mo
-4	CYTO	W5P3V8		1.73E-02	0.0000	GM0_Mo
-4	CYTO	W5P7B8	MTMR3	5.45E-03	0.0000	GM0_Mo
-4	CYTO	W5Q859	STX5	1.69E-04	0.0000	GM0_Mo
-4	CYTO	W5P8H4	DCTPP1	9.40E-05	0.0000	GM0_Mo
-4	CYTO	W5Q8J0	PIN4	2.24E-02	0.0000	GM0_Mo

Supplementary Table 7: Shared and unique differentially abundant proteins (DAPs) between MMØ and GMØ relative to M0 and their cellular location.

FoldChange	Location	Protein IDs	Gene Names	P-value	FDR	Cell type
-4	CYTO	W5PLN1		1.08E-02	0.0000	GM0_Mo
-4	CYTO	W5P7K2		4.97E-05	0.0000	GM0_Mo
-4	CYTO	W5Q9H1	ZYX	2.29E-02	0.0000	GM0_Mo
-4	CYTO	W5PU56	PIH1D1	9.50E-03	0.0000	GM0_Mo
-4	CYTO	W5PKY8	RAD21	1.36E-02	0.0000	GM0_Mo
-4	CYTO	W5Q2G2	IL16	1.40E-02	0.0000	GM0_Mo
-4	CYTO	W5NYC7	DQA	3.01E-02	0.0000	GM0_Mo
-4	CYTO	W5P5K9	SRGN	5.82E-03	0.0002	GM0_Mo
-4	CYTO	W5QI61	CDKN1B	7.88E-04	0.0000	GM0_Mo
-4	CYTO	W5PKS6	DEK	1.16E-02	0.0000	GM0_Mo
-4	CYTO	W5Q5E7	PYCR3	4.26E-03	0.0000	GM0_Mo
-4	CYTO	W5NXW9		3.06E-03	0.0000	GM0_Mo
-4	CYTO	W5PRC8	IKZF3	4.28E-02	0.0000	GM0_Mo
-4	CYTO	W5PAF7	RSL1D1	4.25E-03	0.0000	GM0_Mo
-4	CYTO	W5PBE0	CD74	3.65E-02	0.0000	GM0_Mo
-4	CYTO	W5P0E3	BRD4	4.07E-03	0.0000	GM0_Mo
-4	CYTO	W5PGU9	PLCG2	2.54E-05	0.0000	GM0_Mo
-4	CYTO	W5NTI0	TOP1	1.69E-02	0.0000	GM0_Mo
-4	CYTO	W5PP80	FRY	7.70E-04	0.0012	GM0_Mo
-4	CYTO	W5PFJ0	VCL	2.77E-02	0.0000	GM0_Mo
-4	CYTO	W5PX46	LOC101122591	2.02E-02	0.0000	GM0_Mo
-4	CYTO	W5P815	SEPTIN1	3.77E-02	0.0000	GM0_Mo
-4	CYTO	W5Q6F1		2.79E-02	0.0000	GM0_Mo
-4	CYTO	W5NTF8	SPNS1	2.97E-02	0.0000	GM0_Mo
-4	CYTO	W5PZG0	ADD1	9.29E-03	0.0000	GM0_Mo
-4	CYTO	W5P6T0		1.91E-03	0.0012	GM0_Mo
-4	CYTO	W5QEC3	METTL3	3.90E-03	0.0000	GM0_Mo
-4	CYTO	W5NV79	NUP210	1.38E-02	0.0000	GM0_Mo
-4	CYTO	W5PE21	PELP1	2.68E-02	0.0000	GM0_Mo
-4	CYTO	W5QEL7	NDRG2	3.89E-04	0.0078	GM0_Mo
-4	CYTO	W5P5Y4	RIPOR2	2.38E-03	0.0000	GM0_Mo
-4	CYTO	W5QBV0	SEPTIN3	7.74E-04	0.0002	GM0_Mo
-4	CYTO	W5QCW9	TST	4.94E-02	0.0000	GM0_Mo
-4	CYTO	W5PU80	MS4A1	1.13E-02	0.0000	GM0_Mo
-4	CYTO	W5NR20	LOC101109940	3.61E-02	0.0099	GM0_Mo
-4	CYTO	W5Q2S8	MYL9	2.37E-02	0.0000	GM0_Mo
-4	CYTO	W5QCH5	WDR47	1.70E-04	0.0002	GM0_Mo
-4	CYTO	W5QHW2	DAPK2	5.11E-02	0.0000	GM0_Mo
-4	CYTO	W5P2D1	TOP2B	4.17E-02	0.0000	GM0_Mo
-4	CYTO	W5NTL4	EVL	2.42E-02	0.0000	GM0_Mo
-4	CYTO	Q09YJ2	TES	1.87E-03	0.0000	GM0_Mo
-5	CYTO	W5Q271	MAD2L1	1.10E-04	0.0002	GM0_Mo
-5	CYTO	W5PTQ7	TRAF3IP3	1.27E-02	0.0000	GM0_Mo
-5	CYTO	W5Q6L9	GRAP2	5.07E-02	0.0000	GM0_Mo
-5	CYTO	W5PFI6	RASGRP2	1.14E-02	0.0000	GM0_Mo
-5	CYTO	W5Q1Z2		4.24E-03	0.0006	GM0_Mo
-5	CYTO	W5Q3B8		4.40E-02	0.0000	GM0_Mo
-5	CYTO	W5PJ75	SPTAN1	2.21E-03	0.0000	GM0_Mo
-5	CYTO	W5PA61		4.17E-03	0.0000	GM0_Mo
-5	CYTO	M4WG34	IL1b IL1B	3.04E-03	0.0000	GM0_Mo
-5	CYTO	W5Q6U5	ARMH3	1.09E-02	0.0000	GM0_Mo
-6	CYTO	W5PB37	RRAS2	6.20E-04	0.0000	GM0_Mo
-6	CYTO	W5QDM7	ZNF512	6.06E-03	0.0000	GM0_Mo
-6	CYTO	W5Q9S4	ING3	4.92E-03	0.0012	GM0_Mo
-6	CYTO	W5PHR3	ADD3	1.54E-03	0.0000	GM0_Mo
-6	CYTO	W5PYD8	PARP4	2.37E-03	0.0092	GM0_Mo
-6	CYTO	W5PL70	PDCD4	2.76E-03	0.0000	GM0_Mo
-6	CYTO	W5NZX9	SPTBN1	1.26E-03	0.0000	GM0_Mo
-6	CYTO	W5P733	PDLIM1	4.44E-02	0.0000	GM0_Mo
-6	CYTO	W5NT35	LOC443162	1.44E-02	0.0000	GM0_Mo
-7	CYTO	W5PRW4	TRIAP1	1.10E-04	0.0002	GM0_Mo
-7	CYTO	W5Q0K9	ABLIM1	3.17E-03	0.0000	GM0_Mo

Supplementary Table 7: Shared and unique differentially abundant proteins (DAPs) between MMØ and GMØ relative to M0 and their cellular location.

FoldChange	Location	Protein IDs	Gene Names	P-value	FDR	Cell type
-7	CYTO	W5PF87	ALOX15	8.61E-03	0.0000	GM0_Mo
-7	CYTO	W5P4L3	AVIL	2.57E-02	0.0000	GM0_Mo
-8	CYTO	W5QIK8	SELENBP1	8.29E-03	0.0000	GM0_Mo
7	SEC	W5QHV3	FABP1	3.58E-04	0.0000	GM0_Mo
6	SEC	W5Q8M1	PRRC1	2.89E-02	0.0016	GM0_Mo
6	SEC	W5P3Q3	LOC100101238	2.79E-02	0.0000	GM0_Mo
5	SEC	W5Q8Y5	HDLBP	1.81E-03	0.0000	GM0_Mo
4	SEC	W5NWF5	RARRES1	1.16E-02	0.0000	GM0_Mo
4	SEC	W5PDN3	SPTBN2	1.01E-03	0.0000	GM0_Mo
4	SEC	W5PIQ4	LOC101120093	1.58E-03	0.0000	GM0_Mo
3	SEC	W5Q3K6		9.72E-03	0.0000	GM0_Mo
3	SEC	W5NTD9	CHI3L1	2.43E-02	0.0000	GM0_Mo
2	SEC	W5P8R4	CSF1R	3.45E-02	0.0000	GM0_Mo
2	SEC	W5NTT7	COL1A2	1.46E-02	0.0000	GM0_Mo
2	SEC	W5QGP4	APOD	2.02E-03	0.0093	GM0_Mo
2	SEC	W5PQ07	LOXL4	1.88E-02	0.0000	GM0_Mo
2	SEC	W5P996	PDGFRB	5.01E-02	0.0000	GM0_Mo
2	SEC	W5P4M1	PHB2	1.70E-02	0.0000	GM0_Mo
-2	SEC	W5NZX1		5.25E-02	0.0000	GM0_Mo
-2	SEC	W5PGT0	MYH11	3.17E-02	0.0016	GM0_Mo
-2	SEC	W5PT68	FLNB	3.47E-02	0.0000	GM0_Mo
-2	SEC	W5Q0Y4	ZFAND6	2.44E-02	0.0000	GM0_Mo
-2	SEC	W5NS10	TFPI2	3.11E-02	0.0000	GM0_Mo
-2	SEC	W5P5B6	VSIG4	2.31E-02	0.0000	GM0_Mo
-2	SEC	W5P7B1	SIRT2	1.91E-02	0.0046	GM0_Mo
-2	SEC	W5NTX3		4.24E-02	0.0000	GM0_Mo
-2	SEC	W5PLU6		3.21E-02	0.0000	GM0_Mo
-2	SEC	W5PSA3	TCN1	5.47E-02	0.0000	GM0_Mo
-2	SEC	W5PIN1	UFC1	2.51E-02	0.0000	GM0_Mo
-2	SEC	W5P4V3	MMP3	2.17E-02	0.0000	GM0_Mo
-2	SEC	W5Q285	PDCD6	8.51E-03	0.0000	GM0_Mo
-2	SEC	W5Q5T7	ATP6V1C1	8.09E-03	0.0000	GM0_Mo
-2	SEC	W5QDI7	CSF1	1.82E-02	0.0000	GM0_Mo
-2	SEC	W5QAB7	RANBP1	2.43E-02	0.0000	GM0_Mo
-2	SEC	W5PDD2	TLL2	1.29E-03	0.0068	GM0_Mo
-2	SEC	W5QFK2	MACF1	3.87E-02	0.0000	GM0_Mo
-2	SEC	W5PF04	MAN1A1	4.98E-02	0.0000	GM0_Mo
-2	SEC	W5PQF0	CCT7	4.58E-02	0.0000	GM0_Mo
-2	SEC	W5Q9H2		3.25E-02	0.0000	GM0_Mo
-2	SEC	E7ECV8	NUDT9	1.34E-03	0.0000	GM0_Mo
-2	SEC	W5P8Y7		3.30E-02	0.0038	GM0_Mo
-2	SEC	W5P6X2	IGFBP7	2.78E-02	0.0050	GM0_Mo
-2	SEC	W5NPK5	LOC443475	1.54E-02	0.0000	GM0_Mo
-3	SEC	W5P316	NAMPT	2.10E-02	0.0000	GM0_Mo
-3	SEC	W5PAH6	COPB1	4.15E-02	0.0000	GM0_Mo
-3	SEC	W5Q2I7	USO1	1.19E-02	0.0000	GM0_Mo
-3	SEC	W5PZM9	ANXA5	2.76E-02	0.0000	GM0_Mo
-3	SEC	W5PTA8	FURIN	1.92E-02	0.0045	GM0_Mo
-3	SEC	W5NXX1	IPO5	9.49E-03	0.0000	GM0_Mo
-3	SEC	W5NPI6	PABPC1	5.25E-02	0.0000	GM0_Mo
-3	SEC	W5PJN6	KYNU	2.73E-02	0.0055	GM0_Mo
-3	SEC	W5QH42	MAT2A	4.59E-02	0.0000	GM0_Mo
-3	SEC	W5QI35	IL1RN	9.73E-03	0.0000	GM0_Mo
-3	SEC	W5QAU1		2.67E-02	0.0000	GM0_Mo
-3	SEC	W5PV50	ADA2	1.50E-02	0.0045	GM0_Mo
-3	SEC	W5Q9X5	MAP2K1	2.31E-02	0.0000	GM0_Mo
-3	SEC	W5QCJ4	COMMD9	5.91E-03	0.0000	GM0_Mo
-3	SEC	W5PEY4	TCN2	7.41E-03	0.0000	GM0_Mo
-3	SEC	W5PI89	RO60	4.58E-02	0.0000	GM0_Mo
-3	SEC	W5QHU9	PPIB	1.42E-02	0.0000	GM0_Mo
-3	SEC	W5PEL1	NACA	3.30E-02	0.0000	GM0_Mo
-3	SEC	W5Q203		4.96E-02	0.0000	GM0_Mo

Supplementary Table 7: Shared and unique differentially abundant proteins (DAPs) between MMØ and GMØ relative to M0 and their cellular location.

FoldChange	Location	Protein IDs	Gene Names	P-value	FDR	Cell type
-3	SEC	W5Q9D0	PPP1R7	3.77E-02	0.0000	GM0_Mo
-3	SEC	W5Q1E7	KRT12	3.38E-03	0.0043	GM0_Mo
-3	SEC	W5PYG7	EIF3L EIF3EIP EIF3S6I	2.80E-02	0.0000	GM0_Mo
-3	SEC	W5PMM7	PDIA3	4.66E-02	0.0000	GM0_Mo
-3	SEC	W5P9K6	PSMC3	3.97E-02	0.0000	GM0_Mo
-3	SEC	W5PGJ7	LOC101105208	4.83E-02	0.0000	GM0_Mo
-3	SEC	W5NY99		5.53E-02	0.0000	GM0_Mo
-3	SEC	W5P8Q0	AP2B1	2.29E-03	0.0000	GM0_Mo
-3	SEC	W5QH24		3.16E-02	0.0000	GM0_Mo
-3	SEC	W5Q635	CASP8	1.09E-02	0.0024	GM0_Mo
-3	SEC	W5QBQ9	MYH9	6.52E-03	0.0000	GM0_Mo
-3	SEC	P60713	ACTB	4.73E-04	0.0000	GM0_Mo
-3	SEC	W5Q563	GFUS	3.30E-02	0.0031	GM0_Mo
-3	SEC	W5NWT1	RGS10	9.04E-03	0.0000	GM0_Mo
-3	SEC	W5PI22	CNPY2	1.50E-02	0.0000	GM0_Mo
-3	SEC	W5P9Y1	SNX6	2.22E-02	0.0000	GM0_Mo
-3	SEC	W5NUG0	GOT2	3.91E-02	0.0000	GM0_Mo
-3	SEC	W5QC41	PKM	6.08E-03	0.0000	GM0_Mo
-3	SEC	W5NRJ3		1.96E-02	0.0039	GM0_Mo
-3	SEC	W5NRL8	EIF3A EIF3S10	3.72E-02	0.0000	GM0_Mo
-3	SEC	W5QFH1	ACTC1	2.67E-03	0.0000	GM0_Mo
-3	SEC	W5NRK4	GLUD1	7.95E-03	0.0000	GM0_Mo
-4	SEC	W5P2U9	LRRC59	1.57E-02	0.0000	GM0_Mo
-4	SEC	W5QHE9	STRAP	4.97E-02	0.0000	GM0_Mo
-4	SEC	P50413	TXN	4.56E-02	0.0000	GM0_Mo
-4	SEC	W5PH25	USP14	4.74E-03	0.0000	GM0_Mo
-4	SEC	W5Q1T4		4.05E-02	0.0000	GM0_Mo
-4	SEC	W5Q4B7	RAP1GDS1	4.61E-02	0.0043	GM0_Mo
-4	SEC	W5PFM8	ADK	5.56E-02	0.0000	GM0_Mo
-4	SEC	W5Q7E2	GPS1	1.12E-02	0.0000	GM0_Mo
-4	SEC	W5PU89	ALDH16A1	4.48E-02	0.0000	GM0_Mo
-4	SEC	B7TJ15	MAPK14	2.87E-02	0.0000	GM0_Mo
-4	SEC	W5P375	TCP1	7.07E-03	0.0000	GM0_Mo
-4	SEC	W5PMA3	LOC101105094	2.67E-03	0.0000	GM0_Mo
-4	SEC	P09670	SOD1	1.04E-02	0.0056	GM0_Mo
-4	SEC	W5QBQ8	RAB5C	2.34E-02	0.0000	GM0_Mo
-4	SEC	W5PLQ6	ARCN1	6.05E-03	0.0000	GM0_Mo
-4	SEC	W5NYH2	LOC101102072	4.89E-03	0.0000	GM0_Mo
-4	SEC	W5PF71	KPNB1	1.12E-02	0.0000	GM0_Mo
-4	SEC	W5P5W9	TPI1	2.22E-02	0.0000	GM0_Mo
-4	SEC	W5Q0Q1	YWHAQ	3.13E-02	0.0000	GM0_Mo
-4	SEC	W5Q3E3	RAB2A	7.55E-03	0.0000	GM0_Mo
-4	SEC	W5P363	VAT1	1.50E-02	0.0000	GM0_Mo
-4	SEC	W5PJB6	PGM1	3.69E-03	0.0000	GM0_Mo
-4	SEC	W5Q121		7.11E-03	0.0000	GM0_Mo
-4	SEC	W5Q6V5	DNM2	1.14E-02	0.0000	GM0_Mo
-4	SEC	W5QI99	NEDD4	2.76E-03	0.0000	GM0_Mo
-4	SEC	W5PYQ9	CPNE1	5.44E-04	0.0000	GM0_Mo
-4	SEC	W5PQ76	SRI	2.81E-03	0.0000	GM0_Mo
-4	SEC	C5IWV1	FH	4.49E-02	0.0000	GM0_Mo
-4	SEC	W5P4J1	VAV1	2.13E-02	0.0000	GM0_Mo
-4	SEC	W5PR23	CSE1L	5.85E-03	0.0043	GM0_Mo
-4	SEC	W5PAX6	COPB2	3.37E-02	0.0000	GM0_Mo
-4	SEC	W5NVQ4	CAND1	1.24E-02	0.0000	GM0_Mo
-4	SEC	W5QHD8	PSMD2	4.69E-02	0.0000	GM0_Mo
-4	SEC	W5PDU8	GARS1	8.26E-04	0.0000	GM0_Mo
-4	SEC	W5PDC7	PSMB9	4.15E-02	0.0000	GM0_Mo
-4	SEC	W5QG77	CD58	3.22E-02	0.0000	GM0_Mo
-4	SEC	W5QGT4		3.14E-02	0.0000	GM0_Mo
-4	SEC	W5Q1R1	VPS35	2.54E-02	0.0000	GM0_Mo
-4	SEC	W5PLZ0	ATP6V1B2	1.46E-02	0.0000	GM0_Mo
-4	SEC	W5Q5R8	TXNRD1	7.48E-03	0.0000	GM0_Mo

Supplementary Table 7: Shared and unique differentially abundant proteins (DAPs) between MMØ and GMØ relative to M0 and their cellular location.

FoldChange	Location	Protein IDs	Gene Names	P-value	FDR	Cell type
-4	SEC	W5Q2D9	RAB5A	9.39E-03	0.0000	GM0_Mo
-4	SEC	W5PQK7	EIF5A	2.26E-02	0.0000	GM0_Mo
-4	SEC	W5P508	EIF3B EIF3S9	5.25E-02	0.0000	GM0_Mo
-4	SEC	W5PW97	PSMC5	1.08E-02	0.0000	GM0_Mo
-4	SEC	W5PJ98		5.19E-02	0.0000	GM0_Mo
-4	SEC	W5PE11	CDC42	4.39E-03	0.0000	GM0_Mo
-4	SEC	W5NVY0		5.12E-02	0.0000	GM0_Mo
-4	SEC	W5QJ49	ATP6V1D	5.77E-03	0.0000	GM0_Mo
-4	SEC	W5NU60	BTK	4.41E-02	0.0046	GM0_Mo
-4	SEC	W5Q1R8	MAPK1	1.94E-03	0.0044	GM0_Mo
-4	SEC	W5NSV5	ITGA6	1.29E-02	0.0000	GM0_Mo
-4	SEC	W5P538	CD93	6.81E-04	0.0000	GM0_Mo
-4	SEC	W5Q3X1		9.78E-03	0.0038	GM0_Mo
-4	SEC	W5PX22	SNRPD3	5.06E-02	0.0000	GM0_Mo
-4	SEC	W5PSP7	RUVBL2	4.94E-02	0.0000	GM0_Mo
-4	SEC	W5NX31	AHSA1	4.94E-03	0.0000	GM0_Mo
-4	SEC	W5PUJ4	SYNCRIP	1.80E-02	0.0000	GM0_Mo
-4	SEC	W5PIM8	GALK1	1.59E-02	0.0000	GM0_Mo
-4	SEC	W5P8K0	DCTN2	9.18E-03	0.0000	GM0_Mo
-4	SEC	W5P6M2	PSMD5	3.53E-02	0.0000	GM0_Mo
-4	SEC	W5Q701	LOC101112491	5.27E-03	0.0000	GM0_Mo
-4	SEC	W5P7J7	TWF2	2.97E-02	0.0000	GM0_Mo
-4	SEC	W5NSD9	REXO2	1.26E-02	0.0087	GM0_Mo
-4	SEC	W5P707	ACTN4	2.22E-02	0.0000	GM0_Mo
-4	SEC	W5P880	PRG4	1.37E-03	0.0000	GM0_Mo
-4	SEC	W5PVT6	UBA1	2.08E-02	0.0000	GM0_Mo
-4	SEC	W5QHS2	MGP	1.65E-02	0.0000	GM0_Mo
-4	SEC	W5P824	LOC101103096	5.28E-02	0.0000	GM0_Mo
-4	SEC	W5PDG3	GAPDH	5.52E-02	0.0000	GM0_Mo
-4	SEC	W5PUH7		1.41E-02	0.0000	GM0_Mo
-4	SEC	W5QCP6	PSMC2	3.51E-02	0.0000	GM0_Mo
-4	SEC	W5PVL6	AP2A1	1.15E-03	0.0000	GM0_Mo
-4	SEC	W5QCL5	CSNK2A1	1.86E-03	0.0000	GM0_Mo
-4	SEC	W5PSZ3	JARID2	1.33E-02	0.0046	GM0_Mo
-4	SEC	W5QFL1	LOC101116286	4.59E-03	0.0000	GM0_Mo
-4	SEC	W5NVD7	COPS4	4.66E-02	0.0080	GM0_Mo
-4	SEC	W5PW05	MDH2	1.80E-02	0.0000	GM0_Mo
-4	SEC	W5PFV5	NPEPPS	1.03E-02	0.0000	GM0_Mo
-5	SEC	W5PC25	LOC101116273	3.97E-04	0.0000	GM0_Mo
-5	SEC	W5QFM1		5.45E-02	0.0000	GM0_Mo
-5	SEC	W5QEU6	ANXA4	2.11E-02	0.0000	GM0_Mo
-5	SEC	W5Q3B7	PDE5A	3.91E-02	0.0000	GM0_Mo
-5	SEC	W5P2V0	EZR	9.58E-03	0.0000	GM0_Mo
-5	SEC	W5P689	USP5	3.97E-02	0.0000	GM0_Mo
-5	SEC	W5Q3N5	PRKAR2A	2.46E-04	0.0000	GM0_Mo
-5	SEC	W5PHU7	UNC13D	5.08E-03	0.0000	GM0_Mo
-5	SEC	W5P4R1	MSN	1.07E-02	0.0000	GM0_Mo
-5	SEC	W5P500	PSMB1	1.11E-02	0.0000	GM0_Mo
-5	SEC	W5NS93	PSMA4	1.01E-02	0.0000	GM0_Mo
-5	SEC	W5P7L2	ATP6V1G1	1.09E-03	0.0000	GM0_Mo
-5	SEC	W5PVP5	APRT	1.17E-02	0.0000	GM0_Mo
-5	SEC	W5PLS7	GRB2	1.33E-03	0.0000	GM0_Mo
-5	SEC	W5PE22	GDI2	1.68E-03	0.0000	GM0_Mo
-5	SEC	W5Q9B2	ARHGDIA	3.29E-02	0.0000	GM0_Mo
-5	SEC	W5P5C4	NARS1	3.87E-03	0.0000	GM0_Mo
-5	SEC	W5P1G7		4.70E-02	0.0000	GM0_Mo
-5	SEC	W5PVL5	HSPA4	1.29E-02	0.0000	GM0_Mo
-5	SEC	W5PI50	GLRX	5.07E-02	0.0000	GM0_Mo
-5	SEC	W5QIY3	PSMA3	3.26E-02	0.0000	GM0_Mo
-5	SEC	W5P6X5	STMN1	8.12E-04	0.0000	GM0_Mo
-5	SEC	W5PMP1	COTL1	8.35E-05	0.0000	GM0_Mo
-5	SEC	W5NSM1	ACTR1A	8.64E-04	0.0000	GM0_Mo

Supplementary Table 7: Shared and unique differentially abundant proteins (DAPs) between MMØ and GMØ relative to M0 and their cellular location.

FoldChange	Location	Protein IDs	Gene Names	P-value	FDR	Cell type
-5	SEC	P68253	YWHAG	2.91E-02	0.0000	GM0_Mo
-5	SEC	W5P694	RPLP2	2.87E-02	0.0000	GM0_Mo
-5	SEC	W5Q9M6		8.82E-03	0.0000	GM0_Mo
-5	SEC	W5NUN8	AKR1A1	1.77E-02	0.0000	GM0_Mo
-5	SEC	W5PFV7	COPA	6.14E-03	0.0000	GM0_Mo
-5	SEC	W5P7Z1		9.14E-03	0.0000	GM0_Mo
-5	SEC	W5PU66	IQGAP1	2.94E-02	0.0000	GM0_Mo
-5	SEC	W5P5M7	VPS26A	1.61E-02	0.0000	GM0_Mo
-5	SEC	W5PNZ5	FKBP4	1.40E-02	0.0000	GM0_Mo
-5	SEC	W5NZ57	PSMB10	9.02E-03	0.0000	GM0_Mo
-5	SEC	A8DR93	HSPCA HSP90AA1	2.03E-03	0.0000	GM0_Mo
-5	SEC	W5Q086	PRKAR1A	5.15E-03	0.0000	GM0_Mo
-5	SEC	W5Q5G8	TKT	9.88E-03	0.0000	GM0_Mo
-5	SEC	W5P583	ACP1	8.58E-04	0.0000	GM0_Mo
-5	SEC	W5NQK6	LIMS1	1.35E-03	0.0000	GM0_Mo
-5	SEC	W5PYT7		1.39E-03	0.0000	GM0_Mo
-5	SEC	W5NQ14	EIF3I EIF3S2	1.33E-03	0.0000	GM0_Mo
-5	SEC	W5PQI3	ITGB3	3.02E-03	0.0000	GM0_Mo
-5	SEC	W5P610	CLIC1	3.32E-02	0.0000	GM0_Mo
-5	SEC	W5PWG1	PFKP	3.25E-03	0.0000	GM0_Mo
-5	SEC	W5PR73	CORO1B	1.76E-03	0.0000	GM0_Mo
-5	SEC	W5NPQ6	CAPZA2	1.66E-04	0.0000	GM0_Mo
-5	SEC	W5PK13	CBX3	1.97E-02	0.0000	GM0_Mo
-5	SEC	W5NVC9	RAC1	6.27E-03	0.0000	GM0_Mo
-5	SEC	W5PHX1	AHCY	2.40E-02	0.0000	GM0_Mo
-5	SEC	W5PTM9	UBE2V2	1.25E-03	0.0000	GM0_Mo
-5	SEC	W5QBL7	ARPC1B	5.50E-02	0.0000	GM0_Mo
-5	SEC	W5PMQ9	SAE1	9.51E-04	0.0000	GM0_Mo
-5	SEC	W5QIU5	GMFB	3.65E-02	0.0000	GM0_Mo
-5	SEC	W5QCX9	TTLL12	5.83E-03	0.0000	GM0_Mo
-5	SEC	P81184	LGALS1	5.03E-02	0.0000	GM0_Mo
-5	SEC	W5QHQ7	NCL	2.18E-02	0.0000	GM0_Mo
-5	SEC	W5QCS4	SARS1	4.35E-02	0.0000	GM0_Mo
-5	SEC	W5QCP7	PACSIN2	7.25E-04	0.0000	GM0_Mo
-5	SEC	W5PIG7	ENO1	3.69E-04	0.0000	GM0_Mo
-5	SEC	W5PWN6	PPP2CA	3.12E-02	0.0000	GM0_Mo
-5	SEC	W5PJC2	PSMB3	6.37E-03	0.0000	GM0_Mo
-5	SEC	W5Q9T3	LMNB1	1.03E-02	0.0000	GM0_Mo
-5	SEC	W5P5F6	TBCB	2.13E-03	0.0046	GM0_Mo
-5	SEC	W5QE19	PSMB2	5.16E-03	0.0000	GM0_Mo
-5	SEC	W5PPT6	TUBB	3.03E-03	0.0000	GM0_Mo
-5	SEC	W5PM33	SELP	1.63E-02	0.0000	GM0_Mo
-5	SEC	W5NW17	STXBP2	1.97E-02	0.0000	GM0_Mo
-5	SEC	W5PK04	PGAM1	3.04E-02	0.0000	GM0_Mo
-5	SEC	W5PQ98	ACTR3	2.35E-02	0.0000	GM0_Mo
-5	SEC	W5PK95	HNRNPA2B1	3.58E-02	0.0000	GM0_Mo
-5	SEC	W5PNY5	ARRB1	2.50E-03	0.0000	GM0_Mo
-5	SEC	W5NTW1		3.44E-03	0.0000	GM0_Mo
-5	SEC	C8BKE1	STAT1 STAT4	9.18E-05	0.0000	GM0_Mo
-5	SEC	Q1ZZU7	MIF	5.16E-03	0.0000	GM0_Mo
-5	SEC	W5PKD3	GNAI2	2.41E-02	0.0000	GM0_Mo
-5	SEC	W5QD41	PSME2	1.77E-02	0.0000	GM0_Mo
-5	SEC	W5QIL2	PSMB4	1.30E-02	0.0000	GM0_Mo
-6	SEC	W5Q4L0		2.25E-03	0.0000	GM0_Mo
-6	SEC	W5Q9P7	CFL1	4.60E-03	0.0000	GM0_Mo
-6	SEC	P00349	PGD	1.10E-02	0.0000	GM0_Mo
-6	SEC	W5PZN3	NAP1L1	1.26E-03	0.0000	GM0_Mo
-6	SEC	W5QFG8	ACTR2	3.15E-02	0.0000	GM0_Mo
-6	SEC	W5P6Z6	MAPRE2	3.59E-03	0.0000	GM0_Mo
-6	SEC	W5PNV2	PPP1CA	1.74E-02	0.0000	GM0_Mo
-6	SEC	W5PIJ6	PTPN11	9.85E-03	0.0000	GM0_Mo
-6	SEC	W5PUT6	CLTC	1.05E-02	0.0000	GM0_Mo

Supplementary Table 7: Shared and unique differentially abundant proteins (DAPs) between MMØ and GMØ relative to M0 and their cellular location.

FoldChange	Location	Protein IDs	Gene Names	P-value	FDR	Cell type
-6	SEC	W5NXE5	LOC101104501	4.63E-02	0.0000	GM0_Mo
-6	SEC	W5P8R3	HDGF	4.61E-02	0.0000	GM0_Mo
-6	SEC	W5PR81	TARS1	5.28E-02	0.0000	GM0_Mo
-6	SEC	W5PSZ5	ANXA1	2.54E-02	0.0000	GM0_Mo
-6	SEC	W5NSD5	RAP1B	2.15E-03	0.0000	GM0_Mo
-6	SEC	W5P1X9	ALDOA	1.50E-02	0.0000	GM0_Mo
-6	SEC	W5QD49	PSME1	8.05E-03	0.0000	GM0_Mo
-6	SEC	W5NZQ2	LAP3	1.49E-02	0.0000	GM0_Mo
-6	SEC	W5P889	SEPTIN9	2.08E-03	0.0000	GM0_Mo
-6	SEC	W5PE01	TAGLN2	2.10E-04	0.0000	GM0_Mo
-6	SEC	W5QFH5	RAB1A	4.28E-04	0.0000	GM0_Mo
-6	SEC	W5Q0N0	LOC101119757	1.70E-03	0.0000	GM0_Mo
-6	SEC	W5Q3A5	PAFAH1B1 LIS1	2.73E-03	0.0000	GM0_Mo
-6	SEC	W5PTZ9	LOC101120877	2.42E-03	0.0000	GM0_Mo
-6	SEC	W5Q731	ILK	9.28E-03	0.0000	GM0_Mo
-6	SEC	W5P9U4	PSMA1	1.06E-02	0.0000	GM0_Mo
-6	SEC	W5PK66	PARK7	1.53E-02	0.0000	GM0_Mo
-6	SEC	W5P323	GPI	6.51E-04	0.0000	GM0_Mo
-6	SEC	W5PYM5	CCT8	1.23E-02	0.0000	GM0_Mo
-6	SEC	W5PIN6	LDHA	7.39E-03	0.0000	GM0_Mo
-6	SEC	W5P0V5	RAB11B	1.21E-04	0.0000	GM0_Mo
-6	SEC	W5NRD9		1.11E-03	0.0016	GM0_Mo
-6	SEC	W5PVX4	DBNL	1.18E-03	0.0000	GM0_Mo
-6	SEC	W5PRP1	SLC9A3R1	5.52E-04	0.0000	GM0_Mo
-6	SEC	W5PYK0	SENP3	3.66E-02	0.0000	GM0_Mo
-6	SEC	P62262	YWHAE	2.23E-02	0.0000	GM0_Mo
-6	SEC	W5Q0L1	EEF1G	8.45E-03	0.0000	GM0_Mo
-6	SEC	C5IWU0	ARF1 LOC101123118	1.17E-03	0.0000	GM0_Mo
-6	SEC	W5PQK3	PFKL	4.93E-03	0.0000	GM0_Mo
-6	SEC	W5NY22	PCMT1	2.23E-03	0.0000	GM0_Mo
-6	SEC	B0FZL9	SRSF3	1.29E-02	0.0000	GM0_Mo
-6	SEC	W5P5A0	FLNA	3.19E-04	0.0000	GM0_Mo
-6	SEC	W5P9L9		1.88E-02	0.0000	GM0_Mo
-6	SEC	W5PZ65	PSMA7	6.29E-04	0.0000	GM0_Mo
-6	SEC	W5PRJ4	VCP	1.12E-03	0.0000	GM0_Mo
-6	SEC	W5PH15	RSU1	3.06E-03	0.0000	GM0_Mo
-6	SEC	W5Q694		1.30E-02	0.0000	GM0_Mo
-6	SEC	W5NU34	TREML1	1.70E-03	0.0000	GM0_Mo
-6	SEC	W5PJX1	CCT6A	1.45E-02	0.0000	GM0_Mo
-6	SEC	W5QBD7	YWHAZ	1.15E-03	0.0000	GM0_Mo
-6	SEC	W5NUV1	GNB1	7.44E-04	0.0000	GM0_Mo
-6	SEC	W5P359	STIP1	7.01E-04	0.0000	GM0_Mo
-6	SEC	W5PPG3	ALDH9A1	1.13E-02	0.0000	GM0_Mo
-6	SEC	W5QD96	PARVB	7.39E-04	0.0000	GM0_Mo
-6	SEC	W5P7X3		1.13E-04	0.0000	GM0_Mo
-6	SEC	W5NRW4	TPM4	3.11E-03	0.0000	GM0_Mo
-6	SEC	W5QFN2	HCLS1	5.51E-05	0.0000	GM0_Mo
-6	SEC	W5PRF0	SUB1	5.87E-04	0.0000	GM0_Mo
-7	SEC	W5P765	CCT3	2.42E-03	0.0000	GM0_Mo
-7	SEC	Q5MIB5	PYGL	4.86E-04	0.0000	GM0_Mo
-7	SEC	W5PYQ7	RPS12	9.58E-03	0.0000	GM0_Mo
-7	SEC	W5PSM0	OSTF1	1.48E-03	0.0000	GM0_Mo
-7	SEC	W5Q7C8	BIN2	1.19E-03	0.0000	GM0_Mo
-7	SEC	W5QBV3	PGK1	4.16E-03	0.0000	GM0_Mo
-7	SEC	W5PXX7	CCT5	4.47E-03	0.0000	GM0_Mo
-7	SEC	C5IJA0	RAN	1.32E-03	0.0000	GM0_Mo
-7	SEC	W5PY17	STX7	1.71E-04	0.0000	GM0_Mo
-7	SEC	W5NPN4	HSPA8	1.12E-02	0.0000	GM0_Mo
-7	SEC	W5PG09	PGK2	3.04E-03	0.0000	GM0_Mo
-7	SEC	W5PX84	CCDC171	1.24E-04	0.0046	GM0_Mo
-7	SEC	W5PC09	PSMA6	1.45E-03	0.0000	GM0_Mo
-7	SEC	W5P3E8	GDI1	1.15E-04	0.0000	GM0_Mo

Supplementary Table 7: Shared and unique differentially abundant proteins (DAPs) between MMØ and GMØ relative to M0 and their cellular location.

FoldChange	Location	Protein IDs	Gene Names	P-value	FDR	Cell type
-7	SEC	W5Q8I4	ST13	1.69E-03	0.0000	GM0_Mo
-7	SEC	W5QB61	FKBP1A	3.89E-04	0.0000	GM0_Mo
-7	SEC	W5P716		2.48E-03	0.0000	GM0_Mo
-7	SEC	W5QE14	PCBP1	1.18E-03	0.0000	GM0_Mo
-7	SEC	W5NYA7	LOC101114319	4.32E-03	0.0000	GM0_Mo
-7	SEC	W5QG16	CAP1	3.87E-05	0.0000	GM0_Mo
-7	SEC	W5Q951	CORO1C	1.62E-03	0.0000	GM0_Mo
-7	SEC	W5PK85	EML2	2.83E-03	0.0000	GM0_Mo
-7	SEC	W5PE27	ESD	2.95E-04	0.0000	GM0_Mo
-7	SEC	W5QGG6	CCT2	2.34E-03	0.0000	GM0_Mo
-7	SEC	W5NX91	RPS27A	2.48E-02	0.0000	GM0_Mo
-7	SEC	W5P4C7	SEPTIN7	3.73E-04	0.0000	GM0_Mo
-7	SEC	W5PP64	FHL1	3.93E-04	0.0000	GM0_Mo
-7	SEC	W5QGD1	LDHB	9.41E-03	0.0000	GM0_Mo
-8	SEC	W5PG95	HSPA1A	1.07E-03	0.0000	GM0_Mo
-8	SEC	W5PEX2	GPATCH8	4.24E-04	0.0000	GM0_Mo
-8	SEC	Q28554	GAPDH G3PDH GAPI	1.61E-03	0.0000	GM0_Mo
-8	SEC	W5P098	CORO1A	1.29E-02	0.0000	GM0_Mo
-8	SEC	W5PQA8	PRDX6	4.34E-03	0.0000	GM0_Mo
-8	SEC	W5QFZ3	CCT4	6.73E-04	0.0000	GM0_Mo
-8	SEC	W5PL19	LOC101105123	1.50E-03	0.0000	GM0_Mo
-9	SEC	W5PD82	CALD1	1.16E-03	0.0000	GM0_Mo
-9	SEC	W5PHW0	HSP90AB1	9.47E-04	0.0000	GM0_Mo
-9	SEC	W5PDL1	WDR1	1.44E-03	0.0000	GM0_Mo
-9	SEC	W5PFI7	VCL	1.81E-04	0.0000	GM0_Mo
-9	SEC	W5QAR2	F13A1	6.18E-03	0.0000	GM0_Mo
-9	SEC	W5P409	FERMT3	9.81E-04	0.0000	GM0_Mo
-9	SEC	W5PYX0		5.44E-03	0.0000	GM0_Mo
-9	SEC	C5ISA2	TUBA4A	2.21E-02	0.0000	GM0_Mo
-9	SEC	W5QF71	PLEK	2.01E-04	0.0000	GM0_Mo
-10	SEC	W5P627	GSN	5.97E-05	0.0000	GM0_Mo

GMØ, monocyte-derived macrophages differentiated with GM-CSF (Granulocyte-Macrophage Colony-Stimulating Factor), MMØ, monocyte-derived macrophages differentiated with M-CSF (Macrophage Colony-Stimulating Factor) and M0, Monocytes at 3 hours. Differentially abundant proteins (DAPs) were identified using a threshold of false discovery rate (FDR, q-value) ≤ 0.05 and absolute fold change ≥ 2. Red-highlighted cells indicate shared differentially abundant proteins (DAPs) between GMØ and MMØ relative to M0, whereas non-highlighted cells denote DAPs that are unique to either the GMØ or MMØ subtype. “CYTO” denotes proteins identified in whole-cell lysates, and “SEC” refers to those detected in the secretome.

Supplementary Table 8: Integrated comparative profiling of differentially abundant proteins (DAPs) across monocytes and macrophage subtypes.

								Shared					
				**GMØ_Mo**		**MMØ_Mo**		**cMØ_Mo**		**M1_Mo**		**M2_Mo**	
Cell Compartment	Group comparison	Unique Up/Down	Up/Down	U	D	U	D	U	D	U	D	U	D
Cell Lysate	GMØ_Mo	71/121	U			352	1	368	1	419	0	324	1
			D			0	193	0	101	1	93	2	180
	MMØ_Mo	44/50	U	352	0			318	0	382	0	482	0
			D	1	193			2	79	3	85	0	229
	cMØ_Mo	63/22	U	368	0	318	2			433	0	311	4
			D	1	101	0	79			1	69	4	68
	M1_Mo	106/9	U	419	1	324	3	433	1			374	4
			D	0	93	0	85	0	69			0	74
	M2_Mo	126/106	U	324	2	482	0	311	4	374	0		
			D	1	180	0	229	4	68	4	74		
Secretome	GMØ_Mo	8/170	U			22	0	7	0	16	0	18	0
			D			9	124	0	99	2	122	18	95
	MMØ_Mo	25/2	U	22	9			12	1	18	1	87	0
			D	0	124			0	50	0	59	1	86
	cMØ_Mo	3/30	U	7	0	12	0			10	0	12	3
			D	0	99	1	50			0	77	0	39
	M1_Mo	18/6	U	16	2	18	0	10	0			27	0
			D	0	122	1	59	0	77			2	46
	M2_Mo	601/320	U	18	18	87	1	12	0	27	2		
			D	0	95	0	86	3	39	0	46		

Supplementary Table 8: Integrated comparative profiling of differentially abundant proteins (DAPs) across monocytes and macrophage subtypes.

Cell Compartment	Group comparison	Unique Up/Down	Up/Down	Mo_M1		GMØ_M1		Shared MMØ_M1		cMØ_M1		M2_M1	
				U	D	U	D	U	D	U	D	U	D
Cell Lysate	Mo_M1	95/491	U			5	0	8	6	4	1	14	8
			D			6	40	20	94	7	55	29	133
	GMØ_M1	39/14	U	5	6			7	2	11	1	9	4
			D	0	40			0	30	0	31	4	32
	MMØ_M1	63/32	U	8	20	7	0			16	10	180	0
			D	6	94	2	30			4	34	1	185
	cMØ_M1	28/96	U	4	7	11	0	16	4			15	5
			D	1	55	1	31	10	34			17	38
	M2_M1	2816/109	U	14	29	9	4	180	1	15	17		
			D	8	133	4	32	0	185	5	38		
Secretome	Mo_M1	37/10	U			1	2	63	0	2	0	69	0
			D			0	11	10	13	0	32	16	10
	GMØ_M1	0/9	U	1	0			4	0	0	0	4	0
			D	2	11			4	8	0	16	8	9
	MMØ_M1	44/2	U	63	10	4	4			5	16	271	0
			D	0	13	0	8			0	13	0	14
	cMØ_M1	0/30	U	2	0	0	0	5	0			4	0
			D	0	32	0	16	16	13			23	15
	M2_M1	148/1	U	69	16	4	8	271	0	4	23		
			D	0	10	0	9	0	14	0	15		

Supplementary Table 8: Integrated comparative profiling of differentially abundant proteins (DAPs) across monocytes and macrophage subtypes.

				Shared									
				Mo_M2		GMØ_M2		MMØ_M2		cMØ_M2		M1_M2	
Cell Compartment	Group comparison	Unique Up/Down	Up/Down	U	D	U	D	U	D	U	D	U	D
Cell Lysate	Mo_M2	221/358	U			114	5	22	1	105	8	114	2
			D			13	182	3	34	12	243	13	197
	GMØ_M2	53/46	U	114	13			37	0	241	0	250	0
			D	5	182			0	57	0	407	1	334
	MMØ_M2	9/15	U	22	3	37	0			27	0	33	0
			D	1	34	0	57			0	60	0	51
	cMØ_M2	57/256	U	105	12	241	0	27	0			220	0
			D	8	243	0	407	0	60			1	360
	M1_M2	64/37	U	114	13	250	1	33	0	220	1		
			D	2	197	0	334	0	51	0	360		
Secretome	Mo_M2	97/4	U			0	21	2	1	0	22	0	20
			D			0	122	0	46	0	131	1	121
	GMØ_M2	1/38	U	0	0			1	0	0	0	1	0
			D	21	122			0	55	0	395	1	387
	MMØ_M2	1/0	U	2	0	1	0			0	1	0	0
			D	1	46	0	55			0	58	0	53
	cMØ_M2	3/61	U	0	0	0	0	0	0			1	0
			D	22	131	0	395	1	58			4	390
	M1_M2	17/20	U	0	1	1	1	0	0	1	4		
			D	20	121	0	387	0	53	0	390		

Analyses were conducted for M0 monocytes (3-hour time point) relative to GMØ (GM-CSF–differentiated macrophages), MMØ (M-CSF–differentiated macrophages), M1 (pro-inflammatory macrophages activated with GM-CSF, LPS, and IFN-γ), and M2 (anti-inflammatory macrophages activated with M-CSF and IL-4); as well as for M1 and M2 relative to all other subtypes. The table delineates shared and uniquely regulated DAPs between comparisons, with subcellular localization indicated as either cell lysate or secretome. Differential regulation is denoted as “U” for significantly upregulated and “D” for significantly downregulated proteins (false discovery rate [FDR] ≤ 0.05 and absolute fold change ≥ 2).

Supplementary Table 9: Differentially abundant proteins (DAPs) of MMØ relative to GMØ and their cellular location.

FoldChange	Location	Protein IDs	Gene Names	P-value	FDR
5	CYTO	W5NT23	LOC101119864	1.37E-04	0.007
5	CYTO	W5PD43	HTRA1	1.04E-02	0.000
5	CYTO	M4WG34	IL1b IL1B	3.70E-03	0.000
5	CYTO	W5PB37	RRAS2	9.90E-05	0.000
4	CYTO	W5NYW8	CEP55	2.98E-03	0.000
4	CYTO	W5QJ37		3.62E-03	0.000
4	CYTO	W5PDJ0		3.69E-02	0.000
4	CYTO	W5P816	NAXE AIBP APOA1BP	9.82E-03	0.000
4	CYTO	W5Q341	NDUFA12	2.74E-02	0.000
4	CYTO	W5NY99		1.24E-03	0.000
3	CYTO	W5NZA5	DGKZ	2.09E-05	0.000
3	CYTO	W5P2U3	IVNS1ABP	5.59E-02	0.000
3	CYTO	W5Q5Q7	ASPA	4.25E-02	0.000
3	CYTO	W5Q539	ABL2	6.94E-04	0.000
3	CYTO	W5PLN1		1.85E-02	0.000
3	CYTO	W5P6M0	PCTP	1.03E-02	0.000
3	CYTO	W5QBV0	SEPTIN3	4.29E-03	0.000
3	CYTO	W5PTU7	CA2	5.80E-03	0.000
3	CYTO	W5NZF3	LUC7L3	2.74E-02	0.000
3	CYTO	W5PGD6	BZW2	2.30E-04	0.000
3	CYTO	W5PE21	PELP1	3.30E-02	0.000
3	CYTO	W5Q9C5	LNPEP	1.30E-03	0.000
3	CYTO	W5PS76	UNC45A	5.24E-03	0.000
3	CYTO	W5QAJ6	GRAP	8.38E-03	0.000
3	CYTO	W5Q6R6	SHPK	2.27E-03	0.000
3	CYTO	W5Q4L7	AP3S1	3.52E-03	0.000
3	CYTO	W5PV90	EIF5B	1.24E-02	0.000
3	CYTO	W5PJY6	ADAM28	8.34E-03	0.000
3	CYTO	W5PR96	DCK	1.04E-02	0.000
3	CYTO	W5PVH4	TMEM251	2.01E-03	0.000
3	CYTO	W5PL05	MICAL1	2.40E-03	0.000
3	CYTO	W5NTI0	TOP1	7.49E-03	0.000
3	CYTO	W5PE67		1.95E-03	0.000
3	CYTO	W5PM03	RFC3	1.37E-03	0.000
3	CYTO	W5PG60	SLC39A7	1.21E-03	0.000
3	CYTO	W5Q9Z9	RPL4	8.13E-03	0.000
3	CYTO	W5QHC0	ST3GAL5	4.78E-03	0.000
3	CYTO	W5P3W2	PPP2R5D	4.50E-03	0.000
3	CYTO	W5P1M3		3.31E-02	0.000
3	CYTO	W5NXV1	PACS1	2.97E-02	0.000
3	CYTO	W5NR48	KPNA6	1.66E-02	0.000
3	CYTO	W5NYQ9	TIMM44	1.82E-02	0.000
3	CYTO	W5NTH2	EIF2B3	5.93E-03	0.000
3	CYTO	W5QET8	TEP1	2.52E-03	0.000
3	CYTO	W5Q165	RRM2	3.28E-03	0.000
3	CYTO	W5PVA4	ABCF1	8.14E-04	0.000
3	CYTO	W5PKT0	NAA25	1.19E-03	0.002
3	CYTO	W5NRQ3	TRIM68	2.02E-02	0.002
2	CYTO	W5QA91	NAT10	1.34E-02	0.000
2	CYTO	W5PBA2	BAG6	5.46E-03	0.000
2	CYTO	W5Q5T7	ATP6V1C1	1.78E-02	0.000
2	CYTO	W5PSW3	BAZ1B	1.05E-02	0.000
2	CYTO	W5NXM4	RPS9	3.64E-03	0.000
2	CYTO	W5PQ88	RPS6KB2	2.21E-03	0.000
2	CYTO	W5PPR1	UCK2	3.72E-04	0.000
2	CYTO	W5QFY4	COMMD5	5.76E-03	0.000
2	CYTO	W5PMK9	MRPS11	3.24E-02	0.000
2	CYTO	W5Q9Z8	PHAX	4.52E-04	0.000
2	CYTO	W5PF94	TOR1A	4.07E-04	0.000
2	CYTO	W5NPC7	ACBD3	9.01E-03	0.000
2	CYTO	W5PB28	CLNS1A	2.60E-02	0.000
2	CYTO	W5NX56	SPP1	1.11E-03	0.000
2	CYTO	W5Q537		3.30E-03	0.000

Supplementary Table 9: Differentially abundant proteins (DAPs) of MMØ relative to GMØ and their cellular location.

FoldChange	Location	Protein IDs	Gene Names	P-value	FDR
2	CYTO	W5PUM5	KANK1	2.03E-02	0.000
2	CYTO	W5PMJ1	PTGES2	3.35E-02	0.000
2	CYTO	W5PA78	AVL9	4.07E-04	0.000
2	CYTO	W5NQE1	MAK16	2.49E-02	0.000
2	CYTO	W5NYL7	MTHFD1L	2.69E-02	0.000
2	CYTO	W5QCI3	LOC101106419	7.63E-04	0.000
2	CYTO	W5PYE8	MERTK	1.17E-02	0.000
2	CYTO	W5QFH0	ARSA	3.30E-02	0.000
2	CYTO	W5PFP6	TRNT1	5.51E-03	0.000
2	CYTO	W5PG93	HP1BP3	4.78E-02	0.000
2	CYTO	W5Q1U3	MAP1S	1.66E-02	0.000
2	CYTO	W5PYE5	NUDCD1	5.91E-03	0.000
2	CYTO	W5PTT3	CRTC3	2.52E-02	0.000
2	CYTO	W5PUE5	GART	1.05E-02	0.000
2	CYTO	W5QGC6	FYTTD1	1.23E-02	0.000
2	CYTO	W5P994	LONP1	2.23E-02	0.000
2	CYTO	W5PCE9	MICOS13	1.50E-03	0.000
2	CYTO	W5PRB0	UFL1	2.05E-02	0.000
2	CYTO	W5PGB0	SIRT3	7.96E-03	0.000
2	CYTO	W5NQ97		2.09E-02	0.000
2	CYTO	W5NU25	DENND4B	2.31E-02	0.000
2	CYTO	W5Q0J1	PLA2G6	1.19E-02	0.005
2	CYTO	W5QG63	ZMPSTE24	3.07E-02	0.000
2	CYTO	W5QHD4	EIF4G1	2.06E-03	0.000
2	CYTO	W5P4G4	RAB7B	1.56E-04	0.000
2	CYTO	W5PNH4	ATG5	2.45E-02	0.000
2	CYTO	Q9MZS7	BCL2L1 BCLX	4.29E-02	0.000
2	CYTO	W5NTB8		7.53E-03	0.000
2	CYTO	W5NX86	EIF1B	1.19E-02	0.000
2	CYTO	W5PYH6	AP3D1	2.77E-03	0.000
2	CYTO	W5NT95	ATP6AP2	7.64E-03	0.000
2	CYTO	W5P5T6	PPP1R14B	8.80E-03	0.000
2	CYTO	W5PPR6	EXOC6B	1.05E-02	0.000
2	CYTO	W5NTT1	ARHGAP4	6.91E-03	0.000
2	CYTO	W5P1Z5	PMPCA	1.02E-03	0.000
2	CYTO	W5P180		4.74E-03	0.000
2	CYTO	W5NPW8	STARD4	9.47E-03	0.000
2	CYTO	W5PSW9	RPL8	3.74E-03	0.000
2	CYTO	W5NTF8	SPNS1	8.74E-03	0.000
2	CYTO	W5QHL5	CAB39	6.84E-04	0.000
2	CYTO	W5PUU9	HDHD5	5.35E-02	0.000
2	CYTO	W5P4L4	TMX3	5.83E-03	0.000
2	CYTO	W5P5S5	EMC3	1.21E-02	0.000
2	CYTO	W5Q3C1	NDRG3	1.12E-02	0.000
2	CYTO	W5QJ24	PPP2R5E	8.38E-03	0.000
2	CYTO	W5Q7M1	RRP8	2.54E-02	0.000
2	CYTO	W5Q6U5	ARMH3	4.53E-02	0.000
2	CYTO	W5Q3X2		2.86E-02	0.000
2	CYTO	W5NQC8	RPL27A	1.62E-03	0.000
2	CYTO	W5PVQ1	INPP4A	7.24E-03	0.000
2	CYTO	W5P6P2	LOC101110647	5.62E-02	0.000
2	CYTO	W5PZ86	SEPTIN8	1.33E-02	0.000
2	CYTO	W5P7Y8	PALD1	4.92E-03	0.000
2	CYTO	W5P0C5	LGALS8	1.08E-03	0.000
2	CYTO	W5NRR3	PARP1	8.16E-03	0.000
2	CYTO	W5PZJ7		5.18E-04	0.000
2	CYTO	W5Q6K2	QTRT1	1.93E-02	0.000
2	CYTO	W5P5P8	PSMG3	3.11E-05	0.000
2	CYTO	W5P3N2	FLOT2	1.64E-02	0.000
2	CYTO	W5PZ99	KIDINS220	2.10E-02	0.000
2	CYTO	W5QFV9	PPIE	4.87E-02	0.000
2	CYTO	W5Q3B4	TBCD	1.91E-03	0.000
2	CYTO	W5PNC8	PADI2	2.29E-02	0.000

Supplementary Table 9: Differentially abundant proteins (DAPs) of MMØ relative to GMØ and their cellular location.

FoldChange	Location	Protein IDs	Gene Names	P-value	FDR
2	CYTO	W5Q2P3		8.38E-03	0.000
2	CYTO	W5NRI6	PLPBP PROSC	3.38E-03	0.000
2	CYTO	W5P9L5	RASA2	1.39E-02	0.000
2	CYTO	W5P1H1	MTRF1L	9.87E-04	0.000
2	CYTO	W5PFZ9	XRCC1	4.80E-02	0.000
2	CYTO	W5NWE0	MECR	1.82E-02	0.000
2	CYTO	W5P4B3	NRDC	2.32E-02	0.000
2	CYTO	W5PRA0	BPNT1	1.91E-02	0.000
2	CYTO	W5NZC5	DNAJA3	3.68E-03	0.000
2	CYTO	W5QG62	UMPS	7.52E-03	0.000
2	CYTO	W5NSS0	ARAP3	2.52E-03	0.000
2	CYTO	W5PQ01	NOC4L	7.12E-03	0.000
2	CYTO	W5PW06	PNKP	1.35E-02	0.000
2	CYTO	W5QGS6	LOC101121590	7.83E-04	0.000
2	CYTO	W5QDT3	DAD1	5.91E-05	0.000
2	CYTO	W5PP17	LOC101123010	4.47E-02	0.000
2	CYTO	W5Q675	RPS2	2.16E-03	0.000
2	CYTO	W5NZD0	EXOC2	5.56E-02	0.000
2	CYTO	W5NYC5	SSU72	1.93E-02	0.000
2	CYTO	W5Q9T2	RANGAP1	2.91E-02	0.000
2	CYTO	W5PU49	SDHAF2 PGL2 SDH5	3.92E-02	0.000
2	CYTO	W5PN94	LUC7L	3.63E-02	0.000
2	CYTO	W5PC19	STX18	1.36E-02	0.000
2	CYTO	W5PVQ4	TMEM120A	3.80E-04	0.000
2	CYTO	W5PUM8	HIP1	3.36E-02	0.000
2	CYTO	W5P2K4	CLYBL	1.33E-02	0.000
2	CYTO	W5PPF1	AGPAT3	5.41E-03	0.000
2	CYTO	W5PG07	TPCN1	2.72E-02	0.000
2	CYTO	W5P929	MARS1	3.81E-03	0.000
2	CYTO	W5QFZ3	CCT4	4.77E-02	0.000
2	CYTO	W5P995	LRPPRC	9.71E-03	0.000
2	CYTO	W5P8Y0	ITGAL	1.90E-03	0.000
2	CYTO	W5NY25	EIF3C EIF3S8 LOC10111	3.22E-02	0.000
2	CYTO	W5Q9J0	RNPS1	2.10E-03	0.000
2	CYTO	W5QCC0	RALGAPB	2.41E-02	0.000
2	CYTO	W5Q6F2	ARMC1	2.26E-02	0.000
2	CYTO	W5NZY8	TBCE	2.16E-02	0.000
2	CYTO	W5PPX0	LYPLAL1	1.32E-02	0.000
2	CYTO	W5Q2N0	RASA1	9.17E-03	0.000
2	CYTO	W5Q5I5	LRCH4	1.16E-02	0.000
2	CYTO	W5PZS4	OSBPL8	2.25E-02	0.000
2	CYTO	W5NQR1		1.03E-03	0.000
2	CYTO	W5QCL8	NPL	1.72E-02	0.000
2	CYTO	W5P9A0	ALDH5A1	5.08E-02	0.000
2	CYTO	W5NXL1	CASK	1.25E-02	0.000
2	CYTO	W5NZJ8	WIPI2	3.26E-02	0.000
2	CYTO	W5QHF4	TRIP12	2.31E-02	0.000
2	CYTO	W5PXZ7	BLOC1S2	3.27E-02	0.000
2	CYTO	W5NW49	COASY	3.88E-02	0.000
2	CYTO	W5QCJ0	PIKFYVE	2.97E-02	0.000
2	CYTO	W5NWP6	DIAPH2	4.71E-03	0.000
2	CYTO	W5PNX7		8.14E-04	0.000
2	CYTO	W5PQC6	INPP4B	5.45E-03	0.007
2	CYTO	W5PZN5	KIF3A	4.56E-03	0.000
2	CYTO	W5QA63	FRAS1	9.31E-03	0.008
2	CYTO	W5Q6N3	LOC101115115	1.63E-02	0.000
2	CYTO	W5QI06	PRPF3	5.37E-02	0.000
2	CYTO	W5NZ21	LOC101105090	2.02E-02	0.000
2	CYTO	W5P5Q2	MVP	2.82E-02	0.000
2	CYTO	W5Q992	MYO9B	3.79E-03	0.000
2	CYTO	W5PXN5	CCM2	1.75E-02	0.000
2	CYTO	W5Q2I7	USO1	4.30E-02	0.000
2	CYTO	W5PS50	MPC2	1.41E-02	0.000

Supplementary Table 9: Differentially abundant proteins (DAPs) of MMØ relative to GMØ and their cellular location.

FoldChange	Location	Protein IDs	Gene Names	P-value	FDR
2	CYTO	W5PCD7	ABHD12	2.35E-02	0.000
2	CYTO	W5NRH5	HSD17B4	7.10E-04	0.000
2	CYTO	W5P480	RIT1	3.57E-02	0.000
2	CYTO	W5P958	IDH3G	1.91E-02	0.000
2	CYTO	W5PC18		4.08E-03	0.000
2	CYTO	W5Q3A2		1.68E-02	0.000
2	CYTO	W5P0V0	LOC114110488	9.45E-03	0.000
2	CYTO	W5QHG2	PTCD3	2.95E-02	0.000
2	CYTO	W5PI51	NDUFAF7	1.02E-02	0.000
2	CYTO	W5NQX0	ARHGEF18	9.93E-03	0.000
2	CYTO	W5QHT6	NCEH1	6.92E-03	0.000
2	CYTO	W5PME7	PALS2	3.69E-02	0.000
2	CYTO	W5PVZ7	MRPL57	2.28E-03	0.000
2	CYTO	W5QFN9	UGP2	4.08E-02	0.000
2	CYTO	W5PPE2	MGST3	4.23E-02	0.000
2	CYTO	W5PGS2	VPS52	3.42E-02	0.000
2	CYTO	W5Q2N7	METAP1	4.78E-02	0.000
2	CYTO	W5QAY7	RPL24	4.24E-02	0.000
2	CYTO	W5PBJ4	ARHGAP10	3.05E-02	0.000
2	CYTO	W5P2U9	LRRC59	2.45E-02	0.000
-2	CYTO	W5QEK8	EHD3	3.07E-02	0.000
-2	CYTO	W5PPA7	DDX6	5.48E-02	0.000
-2	CYTO	W5QJ02	DHRS7	1.47E-02	0.000
-2	CYTO	W5PYH9		4.22E-02	0.000
-2	CYTO	W5PFR7	ETHE1	2.80E-02	0.000
-2	CYTO	W5NUX5		6.85E-03	0.000
-2	CYTO	W5PXH4	PTPRE	3.92E-02	0.000
-2	CYTO	W5NTA0		1.70E-02	0.000
-2	CYTO	W5PTB3	INPP5K	1.31E-02	0.000
-2	CYTO	W5QCG0		4.74E-02	0.000
-2	CYTO	W5PUL5	FCGRT	1.19E-02	0.000
-2	CYTO	W5PXG3		7.86E-03	0.002
-2	CYTO	W5PTM9	UBE2V2	5.09E-02	0.000
-2	CYTO	W5PD71	LOC101115495	1.21E-02	0.001
-2	CYTO	W5PM99		1.58E-02	0.000
-2	CYTO	W5P6M5	SEC11C	3.18E-02	0.002
-2	CYTO	W5PGT0	MYH11	2.01E-02	0.000
-2	CYTO	W5PMA0	AP2S1	2.15E-02	0.000
-2	CYTO	W5PMH4	RAB39A	3.98E-02	0.000
-2	CYTO	W5PB89	BCAP31	5.30E-02	0.000
-2	CYTO	W5NX11	EPB41	6.83E-03	0.000
-2	CYTO	W5QHX9	PLBD1	5.14E-03	0.000
-2	CYTO	W5QET2	ADPRH	3.43E-02	0.000
-2	CYTO	W5Q7J0	APOB	2.69E-03	0.000
-2	CYTO	W5Q5M7	SLC44A2	3.69E-02	0.000
-2	CYTO	W5P213		6.82E-03	0.000
-2	CYTO	W5PC89	POLR1C	5.40E-02	0.000
-2	CYTO	W5QG77	CD58	1.06E-02	0.000
-2	CYTO	W5PCA0	ALDOB	5.41E-02	0.000
-2	CYTO	W5P631	KLC4	7.73E-03	0.000
-2	CYTO	W5PZJ8	ASRGL1	1.12E-02	0.000
-2	CYTO	W5P4U4	GRK6	1.66E-02	0.000
-2	CYTO	W5Q8R4	MCM7	1.61E-02	0.000
-2	CYTO	W5P2F1	FOLR2	3.94E-03	0.000
-2	CYTO	W5QGR5	WDFY1	2.14E-02	0.000
-2	CYTO	W5PGF4	PLAUR	1.02E-02	0.000
-2	CYTO	W5PCI1	NLRP3	1.45E-02	0.000
-2	CYTO	W5P5M9		8.36E-03	0.000
-2	CYTO	W5P726	PRCP	1.07E-02	0.000
-2	CYTO	W5PNK3	ACAN	1.57E-02	0.000
-2	CYTO	W5PVB9	VPS28	9.27E-04	0.000
-2	CYTO	W5QGW7	CCDC50	8.81E-03	0.000
-2	CYTO	W5P094	NID1	2.73E-02	0.000

Supplementary Table 9: Differentially abundant proteins (DAPs) of MMØ relative to GMØ and their cellular location.

FoldChange	Location	Protein IDs	Gene Names	P-value	FDR
-2	CYTO	W5Q1S6	EML3	1.55E-02	0.000
-2	CYTO	W5PMU8	GAB1	3.94E-02	0.000
-2	CYTO	W5Q2Y3	PLEKHB2	3.71E-02	0.000
-2	CYTO	W5PAV1	DUS3L	2.38E-02	0.000
-2	CYTO	W5P3T0	USF2	9.31E-03	0.000
-2	CYTO	W5P5T4	LOC101108131	4.38E-02	0.000
-2	CYTO	W5NYF8	CEP135	2.44E-02	0.006
-2	CYTO	W5Q9Q5	PANK4	4.40E-02	0.000
-2	CYTO	W5P5Q1		3.83E-02	0.000
-2	CYTO	W5PQD5	DHX32	5.94E-03	0.002
-2	CYTO	W5NVG2	EFEMP1	2.86E-02	0.000
-2	CYTO	W5PAL2	LOC101105297	5.06E-02	0.000
-2	CYTO	W5PAC1	SVEP1	1.17E-02	0.000
-2	CYTO	Q28893	IGFBP4	4.88E-02	0.000
-2	CYTO	W5PI39	ATP2C1	1.69E-02	0.000
-2	CYTO	W5PW33		4.70E-02	0.000
-2	CYTO	W5P8L0	ALDH18A1	4.68E-02	0.000
-2	CYTO	W5Q0N7	IFIT2	2.67E-03	0.000
-2	CYTO	W5QI32	ADAMTSL4	4.87E-02	0.000
-2	CYTO	W5PLL2	F9	4.46E-03	0.000
-2	CYTO	W5PRI6	MRC1	1.33E-02	0.000
-2	CYTO	W5PZB7	RIGI	3.74E-02	0.000
-2	CYTO	W5QF48	NOP10	1.55E-02	0.000
-2	CYTO	W5NR40	FLT4	1.85E-02	0.000
-2	CYTO	W5NVS5	CRNKL1	2.69E-02	0.000
-2	CYTO	W5PBE1	ISG15	2.29E-02	0.000
-2	CYTO	W5NWW4	CREBBP	1.51E-02	0.000
-2	CYTO	W5QHZ0	CA12	2.07E-02	0.000
-2	CYTO	W5NYG8	TSSC4	1.15E-02	0.000
-2	CYTO	W5NWF2	STXBP5	3.91E-02	0.000
-2	CYTO	W5PDP6	C1QC	3.13E-02	0.000
-2	CYTO	W5NVG4	SMC4	3.15E-02	0.000
-2	CYTO	W5PSM6	HABP2	3.01E-02	0.000
-2	CYTO	W5P8S8	ALDH1L1	4.23E-03	0.000
-2	CYTO	W5PUP6	SPATA13	5.53E-03	0.003
-2	CYTO	W5PI76	ANGPTL3	2.71E-02	0.000
-2	CYTO	W5NZH3		2.10E-03	0.000
-2	CYTO	W5PXT1	MYO1G	1.84E-02	0.000
-2	CYTO	W5QFB2	OLFML3	5.27E-02	0.000
-2	CYTO	W5PGC1	CHMP4B	3.47E-02	0.000
-2	CYTO	W5P6G7	TG	1.72E-02	0.000
-2	CYTO	W5PT68	FLNB	5.33E-02	0.000
-2	CYTO	W5NWC0	MCEMP1	3.91E-02	0.000
-2	CYTO	W5P481	COL1A1	2.18E-02	0.000
-2	CYTO	W5PPM6	PQBP1	1.23E-04	0.000
-2	CYTO	W5PWS1	CD46	1.10E-02	0.000
-2	CYTO	W5PKK4	CCAR2	3.24E-03	0.000
-2	CYTO	W5P093	NQO1	1.60E-02	0.000
-2	CYTO	W5Q1S8	CEMIP	2.41E-02	0.000
-2	CYTO	W5PQE5	SERPINH1	3.99E-02	0.000
-2	CYTO	W5P340	SOD2	9.45E-04	0.000
-2	CYTO	W5NUE5	SLC38A7	3.67E-03	0.000
-2	CYTO	W5Q7H9	TTC19	4.84E-03	0.004
-2	CYTO	W5PPS6		3.85E-03	0.000
-2	CYTO	W5QEH9	THRAP3	2.49E-02	0.000
-3	CYTO	W5NUE6	PLEKHA2	2.83E-02	0.000
-3	CYTO	W5PKQ2	PRXL2A	2.26E-03	0.000
-3	CYTO	W5PHN9	F11R	4.97E-03	0.000
-3	CYTO	W5PDE5	LOC101120001	1.70E-02	0.000
-3	CYTO	W5PVC3	LOC101117851	5.15E-03	0.000
-3	CYTO	W5NT90	BAG4	2.45E-02	0.000
-3	CYTO	W5PII2	ATG4C	6.11E-04	0.000
-3	CYTO	W5PDQ0		1.37E-02	0.000

Supplementary Table 9: Differentially abundant proteins (DAPs) of MMØ relative to GMØ and their cellular location.

FoldChange	Location	Protein IDs	Gene Names	P-value	FDR
-3	CYTO	W5PEU9	STAM2	2.57E-02	0.000
-3	CYTO	W5PNJ5	SARNP	3.84E-02	0.000
-3	CYTO	W5NXY7	LOC101117960	4.13E-03	0.000
-3	CYTO	W5PHM1	ALOX12	2.02E-03	0.000
-3	CYTO	W5PIJ3	STS	4.59E-02	0.000
-3	CYTO	W5Q233	VCAN	1.36E-02	0.000
-3	CYTO	W5PS40		1.81E-03	0.000
-3	CYTO	W5P7C7	CD180	3.78E-02	0.000
-3	CYTO	W5P4F5	RAB27B	4.07E-02	0.000
-3	CYTO	W5NSA6	LOC101122940	9.28E-03	0.000
-3	CYTO	W5PYS8		2.21E-02	0.000
-3	CYTO	W5PEI4	KLKB1	2.00E-02	0.000
-3	CYTO	W5PED4		2.34E-02	0.000
-3	CYTO	W5PFI7	VCL	8.37E-03	0.000
-3	CYTO	W5PQD8	C1QTNF3	7.86E-04	0.000
-3	CYTO	W5PTE9	COL6A1	3.62E-02	0.000
-3	CYTO	W5Q4T6		1.06E-02	0.000
-3	CYTO	W5PL47	BMP1	1.91E-05	0.000
-3	CYTO	P29701	AHSG FETUA	7.88E-03	0.000
-3	CYTO	W5PN31		3.97E-02	0.001
-3	CYTO	W5QCY7	SPP2	5.84E-04	0.000
-3	CYTO	W5P5V0	COLEC11	2.02E-03	0.000
-3	CYTO	W5P060	SYNE1	8.27E-03	0.000
-3	CYTO	W5PWI4	RGN	7.90E-05	0.000
-3	CYTO	W5Q4B1	MYCBP2	5.40E-03	0.000
-3	CYTO	W5Q9K1	QSOX1	3.82E-02	0.000
-3	CYTO	W5NZ40	SGF29	5.22E-02	0.000
-3	CYTO	W5PWH2	MYH14	3.92E-02	0.000
-3	CYTO	W5PK31	FCGR3A	2.05E-03	0.000
-3	CYTO	W5PBD8	LAMB1	1.58E-02	0.000
-3	CYTO	W5NUU7	CDH5	1.09E-03	0.000
-3	CYTO	W5PD82	CALD1	7.83E-03	0.000
-3	CYTO	W5PDS4	C1QA	1.37E-03	0.000
-3	CYTO	W5PUH5	LGMN	1.85E-02	0.000
-3	CYTO	W5QI29	ECM1	3.23E-02	0.000
-3	CYTO	W5P880	PRG4	1.73E-02	0.000
-3	CYTO	W5QI35	IL1RN	1.82E-02	0.000
-3	CYTO	W5PD62	CPB2	4.81E-03	0.009
-3	CYTO	W5PHG1	CDA	1.49E-02	0.000
-3	CYTO	W5PNY4	PDXK	3.16E-02	0.000
-3	CYTO	W5QGS4	PHGDH	4.78E-03	0.000
-3	CYTO	W5PEQ9	SRSF1	1.47E-02	0.000
-3	CYTO	W5PH81	C7	7.08E-05	0.000
-4	CYTO	W5NYA8	SCAMP2	5.75E-03	0.000
-4	CYTO	W5PVN6		2.94E-04	0.006
-4	CYTO	W5Q2B3	SUSD5	2.92E-03	0.009
-4	CYTO	W5P7G4	TNC	1.66E-02	0.000
-4	CYTO	W5Q9B1	ATP2A3	3.96E-03	0.000
-4	CYTO	W5NXW9		9.23E-04	0.000
-4	CYTO	W5QHV3	FABP1	3.59E-03	0.000
-4	CYTO	P54230	CATHL1A BAC1A DODE	2.90E-02	0.000
-4	CYTO	W5QFA5		2.67E-02	0.000
-4	CYTO	W5P407		2.99E-02	0.000
-4	CYTO	W5PG74	IRGQ	3.64E-03	0.000
-4	CYTO	W5PP64	FHL1	3.66E-02	0.000
-4	CYTO	W5NQH2	CHMP6	1.68E-03	0.000
-4	CYTO	W5PCE8	ATP2B3	8.10E-03	0.005
-4	CYTO	W5NSN4		3.55E-02	0.000
-4	CYTO	W5NUJ7	LOC101123419	8.05E-03	0.000
-4	CYTO	W5Q2S8	MYL9	3.45E-03	0.000
-4	CYTO	W5PJT1	PLAC9	4.80E-03	0.000
-4	CYTO	W5PE73	SMPDL3A	1.45E-03	0.000
-4	CYTO	W5PC51	TGFB1	1.02E-02	0.000

Supplementary Table 9: Differentially abundant proteins (DAPs) of MMØ relative to GMØ and their cellular location.

FoldChange	Location	Protein IDs	Gene Names	P-value	FDR
-4	CYTO	W5PV80	BANK1	1.04E-03	0.000
-4	CYTO	W5QGH9	WDR3	2.93E-02	0.000
-4	CYTO	W5NW47		1.75E-02	0.000
-4	CYTO	W5PDP2	CD55	5.00E-03	0.000
-4	CYTO	Q29400	IGFBP2	1.94E-04	0.000
-4	CYTO	W5QBE4	FGL2	2.71E-03	0.000
-4	CYTO	W5P2P7	SYPL1	1.46E-02	0.000
-4	CYTO	W5Q3E4	NAAA	5.34E-03	0.000
-4	CYTO	W5PLT8		3.73E-03	0.000
-4	CYTO	W5PNZ7	RFC1	3.91E-04	0.000
-4	CYTO	W5Q3H9	UPP1	5.02E-02	0.000
-4	CYTO	W5QI12	TLN2	5.10E-03	0.005
-4	CYTO	W5QFP0	THBS1	3.00E-03	0.000
-4	CYTO	W5Q3I7	TUBB1	2.99E-02	0.000
-4	CYTO	P47843	SLC2A3 GLUT3	5.81E-03	0.000
-4	CYTO	W5QAK3	MMRN1	2.20E-02	0.000
-4	CYTO	W5Q824	GSTA1	6.22E-03	0.000
-4	CYTO	W5PF18	LOC101102454	1.99E-02	0.000
-4	CYTO	W5Q7T8	THBS4	4.03E-04	0.000
-4	CYTO	W5NQW9	LOC101104482	2.14E-03	0.000
-4	CYTO	W5Q284	HAPLN1	6.86E-04	0.000
-5	CYTO	W5NVC8	NLRP2	1.62E-03	0.009
-5	CYTO	W5PFV1	CTSL	2.02E-02	0.000
-5	CYTO	W5Q7K5	ZBP1	4.04E-05	0.000
-5	CYTO	W5Q3S9	DSN1	4.29E-03	0.001
-5	CYTO	W5PEB0	FABP7	2.26E-03	0.000
-5	CYTO	W5QGQ3	LOC443320	2.06E-02	0.000
-5	CYTO	P79365	SLC2A1 GLUT1	8.06E-03	0.005
-5	CYTO	W5NTJ2	TPX2	2.82E-04	0.006
-6	CYTO	W5Q878	PRKCB	7.16E-03	0.000
-6	CYTO	W5PEL7	HSPG2	9.21E-03	0.000
-6	CYTO	W5PXV3	CCN2	1.56E-03	0.000
-6	CYTO	W5Q723	BHMT	1.23E-03	0.000
-6	CYTO	W5QDG8	FN1	2.00E-02	0.000
-6	CYTO	W5PK56	FCGR2B	4.06E-03	0.000
-6	CYTO	W5PQI3	ITGB3	1.07E-03	0.000
-7	CYTO	W5PG63	VWF	4.04E-04	0.000
-7	CYTO	W5P0K8	CLIC2	8.50E-03	0.000
-7	CYTO	W5Q5A6	FGG	3.34E-02	0.000
-8	CYTO	W5NRX0	PPFIA4	2.24E-04	0.001
-8	CYTO	W5NQ46	FGB	2.07E-02	0.000
-8	CYTO	W5Q5H8	FGA	1.89E-02	0.000
-9	CYTO	W5PEX2	GPATCH8	4.75E-03	0.000
9	SEC	P81184	LGALS1	5.94E-05	0.000
8	SEC	W5QI78	CTSK	2.06E-04	0.000
7	SEC	W5PCH3	SCIN	2.17E-03	0.000
7	SEC	W5Q9H0	MMP2	6.88E-05	0.000
7	SEC	W5P026	STAB1	1.40E-03	0.000
7	SEC	W5PEY4	TCN2	9.00E-04	0.000
6	SEC	W5PNY4	PDXK	3.85E-03	0.000
6	SEC	W5PZ47	CTSH	4.11E-03	0.000
6	SEC	W5Q0F1	LIPA	5.02E-03	0.000
6	SEC	Q28554	GAPDH G3PDH GAPD	6.26E-03	0.000
6	SEC	W5P2V3	PEPD	2.93E-03	0.000
6	SEC	W5PNW7	VIM	4.87E-03	0.000
6	SEC	Q6ECI6	ITGB2 CD18	2.10E-03	0.000
6	SEC	W5NT95	ATP6AP2	2.66E-04	0.000
6	SEC	W5NRJ3		1.08E-05	0.004
6	SEC	W5NPM4	TEX15	2.59E-04	0.004
6	SEC	W5NUI3	TREM2	7.11E-05	0.000
6	SEC	W5QG24	PPT1	2.40E-03	0.000
6	SEC	W5PXR1	ENPP1	1.33E-04	0.000
6	SEC	G3M9U4	ACP5	2.85E-02	0.000

Supplementary Table 9: Differentially abundant proteins (DAPs) of MMØ relative to GMØ and their cellular location.

FoldChange	Location	Protein IDs	Gene Names	P-value	FDR
6	SEC	W5NX56	SPP1	5.04E-02	0.000
6	SEC	Q29524	LPL	1.36E-02	0.000
6	SEC	W5PYQ7	RPS12	7.38E-04	0.000
6	SEC	W5QJ62	ACTN1	6.50E-04	0.000
6	SEC	W5Q3T9	PDCD6IP	1.62E-03	0.000
6	SEC	Q6XUZ5	IDH1	2.12E-02	0.000
6	SEC	W5PGS4	FABP5	7.86E-03	0.000
6	SEC	W5PIC9		9.10E-04	0.000
6	SEC	W5NUI6	SGSH	6.80E-04	0.000
6	SEC	W5QBZ7	NAGA	1.19E-02	0.000
6	SEC	W5P7L2	ATP6V1G1	8.08E-03	0.000
6	SEC	W5PT76	GPNMB	1.43E-02	0.000
6	SEC	C8BKE1	STAT1 STAT4	6.54E-03	0.000
5	SEC	W5QFU8	CD86	4.76E-04	0.000
5	SEC	W5P726	PRCP	1.35E-07	0.000
5	SEC	W5P9L9		4.29E-03	0.000
5	SEC	W5PHW0	HSP90AB1	1.39E-02	0.000
5	SEC	W5PDD0	LCP1	4.73E-02	0.000
5	SEC	W5PAM4	CTSA	1.13E-02	0.000
5	SEC	W5PDL1	WDR1	1.64E-02	0.000
5	SEC	W5QFN2	HCLS1	6.21E-03	0.000
5	SEC	W5PUT6	CLTC	5.47E-02	0.000
5	SEC	W5P3I5	CNDP2	1.80E-02	0.000
5	SEC	W5QAQ4	NRP2	4.08E-06	0.000
5	SEC	W5NTG6	TINAGL1	4.65E-04	0.000
5	SEC	W5Q1W2	SDCBP	6.08E-03	0.000
5	SEC	W5NX91	RPS27A	4.91E-03	0.000
5	SEC	W5QIW1	LGALS3	4.25E-02	0.000
5	SEC	W5QI36	HEBP1	1.87E-02	0.000
5	SEC	W5NTZ3	RENBP	1.51E-02	0.000
5	SEC	W5PCE0	PLBD2	2.54E-03	0.000
5	SEC	W5P7X3		4.21E-03	0.000
5	SEC	W5Q3U3	LOC101102156	2.35E-03	0.000
5	SEC	W5QGD1	LDHB	3.80E-02	0.000
5	SEC	W5P9J8	BLVRB	3.91E-03	0.000
5	SEC	W5PJB6	PGM1	1.31E-03	0.000
5	SEC	W5PCN2	ANXA7	2.30E-02	0.000
5	SEC	W5QGG0	TFRC	1.62E-03	0.000
5	SEC	W5PBM9	SCPEP1	9.90E-04	0.000
5	SEC	P00349	PGD	3.16E-02	0.000
5	SEC	W5P5W6	NDRG1	2.94E-02	0.000
5	SEC	W5P5M7	VPS26A	3.14E-02	0.000
5	SEC	W5QBV3	PGK1	8.80E-03	0.000
5	SEC	W5PQA8	PRDX6	2.87E-02	0.000
5	SEC	W5NPN4	HSPA8	3.67E-02	0.000
5	SEC	W5P640	LMNA	2.70E-02	0.000
5	SEC	W5Q799	GALNT6	1.99E-04	0.010
5	SEC	W5PZ65	PSMA7	2.60E-03	0.000
5	SEC	W5Q1M0	GLB1	8.81E-04	0.000
5	SEC	W5P4A8	RNASET2	4.14E-05	0.000
5	SEC	W5PUW2	IFI30	1.04E-02	0.000
5	SEC	W5PQ98	ACTR3	1.49E-02	0.000
5	SEC	W5Q2U7	PLEC	3.00E-02	0.000
5	SEC	W5PIN6	LDHA	2.00E-02	0.000
5	SEC	W5QFG8	ACTR2	3.57E-02	0.000
5	SEC	W5PJ54	DPYSL2	3.25E-02	0.000
5	SEC	W5PMB1	SNX3	3.34E-02	0.000
5	SEC	W5P409	FERMT3	3.24E-02	0.000
5	SEC	W5QEU6	ANXA4	2.01E-02	0.000
5	SEC	W5PNY5	ARRB1	8.20E-03	0.000
5	SEC	W5QJ49	ATP6V1D	3.76E-02	0.000
5	SEC	W5PKY1	HNMT	2.44E-03	0.000
5	SEC	W5QI70	CTSS	2.39E-03	0.000

Supplementary Table 9: Differentially abundant proteins (DAPs) of MMØ relative to GMØ and their cellular location.

FoldChange	Location	Protein IDs	Gene Names	P-value	FDR
5	SEC	W5PSZ5	ANXA1	5.20E-02	0.000
5	SEC	W5PV50	ADA2	1.49E-05	0.004
5	SEC	W5P3C6	LOC101111906	1.53E-03	0.000
5	SEC	W5Q689	TPP1	2.05E-03	0.000
5	SEC	W5NVW7	NAGLU	4.04E-03	0.000
5	SEC	W5P1G7		2.86E-02	0.000
5	SEC	W5PJC2	PSMB3	1.03E-02	0.000
5	SEC	W5PKU3		2.72E-04	0.000
5	SEC	W5NPP2	CPM	1.35E-04	0.000
4	SEC	W5NZ76	TIMP2	1.02E-05	0.000
4	SEC	W5Q0L1	EEF1G	1.53E-02	0.000
4	SEC	W5PVL6	AP2A1	9.10E-03	0.000
4	SEC	W5PC09	PSMA6	2.52E-02	0.000
4	SEC	W5QB61	FKBP1A	1.77E-02	0.000
4	SEC	W5Q9M6		2.33E-03	0.000
4	SEC	W5PG09	PGK2	7.29E-03	0.000
4	SEC	W5NY25	EIF3C EIF3S8 LOC10111	7.83E-03	0.000
4	SEC	W5PUJ4	SYNCRIP	2.21E-02	0.000
4	SEC	W5NQ14	EIF3I EIF3S2	1.73E-02	0.000
4	SEC	W5NU86	GLA	3.51E-03	0.000
4	SEC	W5QG16	CAP1	3.12E-02	0.000
4	SEC	W5PIQ6	MSR1	5.53E-02	0.000
4	SEC	W5PR73	CORO1B	5.96E-03	0.000
4	SEC	W5PJS4	EMILIN2	1.55E-02	0.000
4	SEC	W5PF73	PLAU	3.53E-03	0.000
4	SEC	W5PAJ2	PSAP	6.07E-04	0.000
4	SEC	W5P3N6	LOC101112162	9.03E-03	0.000
4	SEC	W5PZB0	APLP2	8.53E-06	0.000
4	SEC	W5NUG0	GOT2	3.23E-02	0.000
4	SEC	W5PS45	MAN2B1	9.41E-04	0.000
4	SEC	W5Q086	PRKAR1A	3.56E-02	0.000
4	SEC	W5QFB2	OLFML3	2.62E-02	0.000
4	SEC	W5PVP5	APRT	1.86E-02	0.000
4	SEC	W5NUG3	GNPDA1	1.93E-02	0.000
4	SEC	W5QCS4	SARS1	4.65E-02	0.000
4	SEC	W5Q4L0		3.51E-02	0.000
4	SEC	W5Q3N1	CTSZ	2.15E-02	0.000
4	SEC	W5QDZ3	ARPC2	4.98E-02	0.000
4	SEC	W5Q1T4		3.21E-02	0.000
4	SEC	W5PBS4	LRP1	1.64E-04	0.000
4	SEC	W5PBC0		1.66E-02	0.000
4	SEC	W5PW05	MDH2	1.07E-02	0.000
4	SEC	W5NZQ2	LAP3	3.74E-02	0.000
4	SEC	W5NUV1	GNB1	3.49E-02	0.000
4	SEC	W5QIL2	PSMB4	4.27E-02	0.000
4	SEC	W5P9U4	PSMA1	5.14E-03	0.000
4	SEC	W5PCC7	LOC101115509	5.46E-03	0.000
4	SEC	W5PVX4	DBNL	5.15E-03	0.000
4	SEC	W5NVT3	SND1	2.48E-02	0.000
4	SEC	W5PN69	LOC101109111	2.72E-02	0.000
4	SEC	W5Q9B2	ARHGDIA	4.77E-02	0.000
4	SEC	W5PWZ2		1.23E-03	0.000
4	SEC	W5P323	GPI	2.14E-02	0.000
4	SEC	W5NY99		2.28E-02	0.000
4	SEC	W5PGM1	OXCT1	2.66E-02	0.000
4	SEC	W5PGC5	GALM	1.17E-02	0.000
4	SEC	W5PB21	PLA2R1	7.89E-03	0.000
4	SEC	W5PEL1	NACA	5.13E-03	0.000
4	SEC	W5PIG7	ENO1	4.18E-03	0.000
4	SEC	W5P689	USP5	3.75E-02	0.000
4	SEC	W5Q5G8	TKT	1.70E-02	0.000
4	SEC	W5Q7C8	BIN2	5.38E-03	0.000
4	SEC	W5QD41	PSME2	5.00E-02	0.000

Supplementary Table 9: Differentially abundant proteins (DAPs) of MMØ relative to GMØ and their cellular location.

FoldChange	Location	Protein IDs	Gene Names	P-value	FDR
4	SEC	W5NY22	PCMT1	2.84E-02	0.000
4	SEC	W5PIH2	B4GALT1	7.08E-03	0.000
4	SEC	W5PHX1	AHCY	2.92E-02	0.000
3	SEC	W5NSH8	NPC2	6.33E-03	0.000
3	SEC	W5Q5K9	ARPC3	4.86E-02	0.000
3	SEC	W5P4R1	MSN	3.08E-02	0.000
3	SEC	W5NYA7	LOC101114319	3.83E-02	0.000
3	SEC	W5QH35	CAPG	3.06E-02	0.000
3	SEC	W5PPT6	TUBB	3.64E-02	0.000
3	SEC	W5NUN8	AKR1A1	4.01E-02	0.000
3	SEC	W5PLB9	PSMD11	4.49E-02	0.000
3	SEC	A2SW69	ANXA2 ANX2	3.79E-02	0.000
3	SEC	W5QDF4	GSTM3	3.97E-02	0.000
3	SEC	W5P627	GSN	3.09E-02	0.000
3	SEC	W5QJ99	TNFRSF1A	4.25E-02	0.000
3	SEC	W5NTD9	CHI3L1	1.33E-02	0.000
3	SEC	W5PZM9	ANXA5	1.43E-02	0.000
3	SEC	W5P500	PSMB1	4.26E-02	0.000
3	SEC	W5QC41	PKM	8.67E-03	0.000
3	SEC	W5Q9H2		1.81E-02	0.000
3	SEC	W5QC34	MAN2A1	3.13E-03	0.000
3	SEC	W5P359	STIP1	2.80E-04	0.000
3	SEC	W5QBQ8	RAB5C	1.39E-02	0.000
3	SEC	W5Q0F3	TGFBI	3.38E-04	0.000
3	SEC	W5QAA9	ACO2	2.01E-02	0.000
3	SEC	W5NUZ1	DYNC1I2	3.45E-02	0.000
3	SEC	B0FZL9	SRSF3	2.75E-02	0.000
3	SEC	W5PKD3	GNAI2	1.98E-02	0.000
3	SEC	W5PE22	GDI2	2.62E-02	0.000
3	SEC	W5PTS4	LOC101114275	2.11E-02	0.000
3	SEC	P50413	TXN	4.27E-02	0.000
3	SEC	W5PJ66	CFD	1.77E-02	0.000
3	SEC	W5PK33	EFHD2	4.53E-02	0.000
3	SEC	W5Q4L6	UBXN1	1.37E-02	0.000
3	SEC	W5PG72	GOLM1	4.51E-02	0.000
3	SEC	W5P8R3	HDGF	5.19E-02	0.000
3	SEC	W5PF33	GM2A	2.65E-03	0.000
3	SEC	W5PMM7	PDIA3	5.56E-02	0.000
3	SEC	A8DR93	HSPCA HSP90AA1	2.82E-02	0.000
3	SEC	W5PRJ4	VCP	3.65E-02	0.000
3	SEC	W5QIU5	GMFB	5.14E-02	0.000
3	SEC	W5P2V0	EZR	2.78E-02	0.000
3	SEC	W5Q3M9	SEPTIN6	5.22E-02	0.000
3	SEC	W5PD48	CYRIB	4.59E-02	0.000
3	SEC	W5P5A0	FLNA	3.01E-02	0.000
3	SEC	P83205	CTSB	5.38E-03	0.000
3	SEC	W5QF71	PLEK	1.89E-02	0.000
3	SEC	W5P659	HK1	1.97E-02	0.000
3	SEC	W5QJA2	CD14	1.17E-04	0.000
3	SEC	W5PCS7	CXCL16	4.68E-02	0.004
3	SEC	W5PF04	MAN1A1	2.37E-02	0.000
3	SEC	P82197	PDXK PKH	3.57E-02	0.004
3	SEC	W5PF12	HSD17B10	1.86E-03	0.000
3	SEC	W5PCM7	LOC101105937	4.56E-02	0.004
3	SEC	W5PS94	NUCB1	4.39E-03	0.000
3	SEC	W5PI50	GLRX	7.69E-03	0.000
2	SEC	W5NSD5	RAP1B	1.21E-02	0.000
2	SEC	W5PAX6	COPB2	5.23E-02	0.000
2	SEC	W5NYH2	LOC101102072	1.72E-02	0.000
2	SEC	W5P889	SEPTIN9	4.15E-02	0.000
2	SEC	W5NSM1	ACTR1A	1.95E-02	0.000
2	SEC	W5QFL1	LOC101116286	3.20E-02	0.000
2	SEC	W5QI35	IL1RN	2.06E-02	0.000

Supplementary Table 9: Differentially abundant proteins (DAPs) of MMØ relative to GMØ and their cellular location.

FoldChange	Location	Protein IDs	Gene Names	P-value	FDR
2	SEC	W5P1W2	FOLR3	2.03E-02	0.009
2	SEC	W5PAH6	COPB1	2.44E-02	0.000
2	SEC	W5PFC9	LOC101117129	5.16E-02	0.000
2	SEC	W5QD02		3.19E-02	0.004
2	SEC	C5IWV1	FH	3.00E-02	0.000
2	SEC	W5PUH7		2.93E-02	0.000
2	SEC	W5Q1I6	IMPDH2 IMPDH	1.55E-02	0.000
2	SEC	W5QAU1		2.74E-02	0.000
2	SEC	W5Q9H1	ZYX	5.06E-02	0.000
2	SEC	W5PTZ8	LOC101114959	3.08E-02	0.000
2	SEC	W5PUH5	LGMN	3.99E-02	0.000
2	SEC	W5PE01	TAGLN2	4.07E-02	0.000
2	SEC	W5Q1I8	PPM1F	5.35E-02	0.000
2	SEC	W5P8K5	HNRNPAB	3.94E-02	0.000
2	SEC	P07846	SORD	5.54E-02	0.000
2	SEC	W5PIM8	GALK1	2.80E-02	0.000
2	SEC	W5PFV5	NPEPPS	4.41E-02	0.000
2	SEC	W5PDN2		5.57E-02	0.000
2	SEC	W5Q6U0	FASN	8.38E-03	0.000
2	SEC	W5QDI7	CSF1	2.23E-02	0.000
2	SEC	W5P0V5	RAB11B	8.36E-04	0.000
2	SEC	W5P814	KIF5A	5.04E-02	0.000
2	SEC	W5P2U9	LRRC59	1.49E-02	0.000
2	SEC	W5P303	YWHAB	4.73E-02	0.000
2	SEC	W5NWF5	RARRES1	3.88E-02	0.000
2	SEC	W5P5W9	TPI1	4.17E-02	0.000
2	SEC	W5PBW1	SDS	9.78E-03	0.000
2	SEC	W5NRI1		8.73E-03	0.000
2	SEC	W5PGW9	TNFAIP6	4.58E-02	0.000
2	SEC	W5Q6V2	KRT7	4.54E-02	0.000
2	SEC	W5NQP9	ALDOC	3.33E-02	0.000
2	SEC	W5PZ86	SEPTIN8	1.81E-02	0.000
2	SEC	W5QD23	LANCL1	1.50E-02	0.000
2	SEC	W5Q0Q1	YWHAQ	3.27E-02	0.000
2	SEC	W5NPK5	LOC443475	5.05E-02	0.000
2	SEC	W5QCL5	CSNK2A1	4.58E-02	0.000
2	SEC	P60713	ACTB	1.12E-02	0.000
2	SEC	W5PSP7	RUVBL2	4.61E-03	0.000
2	SEC	W5P0W4	SEMA7A	3.89E-03	0.000
2	SEC	W5NS10	TFPI2	3.12E-02	0.000
2	SEC	W5PR23	CSE1L	3.46E-03	0.004
2	SEC	W5Q1E7	KRT12	5.61E-03	0.004
2	SEC	W5NX31	AHSA1	1.95E-02	0.000
2	SEC	W5NW82	NAP1L4	8.23E-03	0.000
2	SEC	W5PLS7	GRB2	4.14E-02	0.000
2	SEC	W5P4C9	MMP12	1.87E-02	0.000
2	SEC	W5QBP6	GLUL	5.62E-03	0.000
2	SEC	W5NVB9	ARPC5	2.37E-02	0.000
2	SEC	Q09YJ2	TES	5.13E-02	0.000
2	SEC	W5PVU5	PSMB7	2.58E-02	0.000
2	SEC	W5PFR8	FBP1	1.27E-02	0.000
2	SEC	W5NR04		2.87E-02	0.000
2	SEC	W5PSZ3	JARID2	1.28E-02	0.005
2	SEC	W5QCL8	NPL	7.64E-03	0.000
2	SEC	W5PDD2	TLL2	8.60E-03	0.007
2	SEC	W5P316	NAMPT	4.07E-02	0.000
2	SEC	W5P880	PRG4	3.83E-03	0.000
2	SEC	W5QDM2	PSMB5	2.49E-02	0.000
2	SEC	W5PPG3	ALDH9A1	4.56E-02	0.000
-2	SEC	W5QGP4	APOD	2.52E-02	0.009
-2	SEC	W5P4M1	PHB2	1.65E-03	0.000
-2	SEC	W5QB24	ANP32A	2.96E-02	0.000
-3	SEC	W5QG01	DNPEP	1.17E-02	0.000

Supplementary Table 9: Differentially abundant proteins (DAPs) of MMØ relative to GMØ and their cellular location.

FoldChange	Location	Protein IDs	Gene Names	P-value	FDR
-3	SEC	W5QJA1		3.06E-03	0.000
-4	SEC	W5Q6H1	PLS3	1.46E-03	0.000
-5	SEC	W5Q8S1	SEPTIN2	5.32E-02	0.000
-6	SEC	W5QB02	TGM2	2.12E-06	0.002

GMØ, monocyte-derived macrophages differentiated with GM-CSF (Granulocyte-Macrophage Colony-Stimulating Factor) and MMØ, monocyte-derived macrophages differentiated with M-CSF (Macrophage Colony-Stimulating Factor). Differentially abundant proteins (DAPs) were identified using a threshold of false discovery rate (FDR, q-value) ≤ 0.05 and absolute fold change ≥ 2. Red-highlighted cells indicate shared differentially abundant proteins (DAPs) between the cell lysate (CYTO) and secretome (SEC) compartments in MMØ relative to GMØ, whereas non-highlighted cells represent compartment-specific DAPs unique to either the whole cell lysate or secretome.

Supplementary Table 10: Comparative analysis of differentially abundant proteins (DAPs) uniquely distinguishing GMØ from M0, cMØ, MMØ, M1, and M2, and those exclusively differentiating MMØ relative to Mo, cMØ, GMØ, M1, and M2.

Protein	Accession IDs		GMØ_Mo		GMØ_MMØ		GMØ_cMØ		GMØ_M1		GMØ_M2	
			secreted	cell lysate	secreted	cell lysate	secreted	cell lysate	secreted	cell lysate	secreted	cell lysate
DSN1	W5Q3S9	Fold Change		6.01		5.05		4.15		3.17		4.44
		p-value		0.0016		0.0043		0.0087		0.0377		0.0007
RFC1	W5PNZ7	Fold Change		4.95		4.18		4.42		3.46		4.53
		p-value		0.0004		0.0004		0.0000		0.0194		0.0000
HSPG2	W5PEL7	Fold Change		8.34		6.00		7.95		5.89		7.43
		p-value		0.0000		0.0092		0.0000		0.0102		0.0000
			**MMØ_Mo**		**MMØ_GMØ**		**MMØ_cMØ**		**MMØ_M1**		**MMØ_M2**	
			secreted	cell lysate	secreted	cell lysate	secreted	cell lysate	secreted	cell lysate	secreted	cell lysate
TGFB1	W5Q0F3	Fold Change	6.96		3.12		7.94		4.07			2.19
		p-value	0.0003		0.0003		0.0006		0.0070			0.0411
EMILIN2	W5PJS4	Fold Change	5.39	5.91	4.18		7.94		5.49		-1.75	2.48
		p-value	0.0008	0.0002	0.0155		0.0006		0.0002		0.0177	0.0266
CHURC1	W5QJ37	Fold Change		4.16		3.84		3.44		3.82		1.53
		p-value		0.0000		0.0036		0.0000		0.0000		0.0113

Mo, Monocytes at 3 hours; cMØ, monocyte-derived macrophages differentiated without the addition of exogenous growth factors such as M-CSF or GM-CSF.; GMØ, monocyte-derived macrophages differentiated with GM-CSF (Granulocyte-Macrophage Colony-Stimulating Factor); MMØ, monocyte-derived macrophages differentiated with M-CSF (Macrophage Colony-Stimulating Factor); M1, pro-inflammatory macrophages activated with GM-CSF/ LPS/ INF-γ; M2, anti-inflammatory macrophages activated with M-CSF/ IL-4. All marker proteins shown were differentially abundant with a false discovery rate (FDR, q-value) < 0.001.

Supplementary Table 11: Comparative functional enrichment analysis of GMØ (monocyte-derived macrophages differentiated with GM-CSF) and MMØ (monocyte-derived macrophages differentiated with M-CSF) relative to Mo (monocytes at 3 hours). Functional enrichment analysis was performed using the StringApp (STRING database) within Cytoscape (version 3.10.3)[1], focusing on Gene Ontology (GO) Biological Process (BP) terms[2,3]. Red-highlighted cells indicate Gene Ontology Biological Process terms shared between GMØ and MMØ compared to Mo, whereas non-highlighted cells represent subtype-specific enrichment.

Functional enrichment analysis of GMØ_Mo

<table>
<tr><th># background genes</th><th># genes</th><th>category</th><th>description_GMØ_Mo</th><th>FDR value</th><th>genes</th><th>p-value</th><th>term name</th></tr>
<tr><td>1197</td><td>164</td><td>GO Biological Process</td><td>Vesicle-mediated transport</td><td>1.52E-16</td><td>HSPA8|TBC1D2B|RALBP1|BET1|ARL3|NCF2|PIP4K2A|ARF5|RAC1|STXB</td><td>1.34E-20</td><td>GO:0016192</td></tr>
<tr><td>1731</td><td>209</td><td>GO Biological Process</td><td>Catabolic process</td><td>1.66E-16</td><td>PABPC1|HSPA8|GLUD1|PLCD1|FBXO6|ALDH6A1|PSMA4|PNPLA6|RIPK1</td><td>2.93E-20</td><td>GO:0009056</td></tr>
<tr><td>15933</td><td>1090</td><td>GO Biological Process</td><td>Cellular process</td><td>3.21E-15</td><td>COX2|PABPC1|W5NPK5_SHEEP|DYNLT3|HSPA8|CPM|DRAP1|CAPZA2|S</td><td>8.5E-19</td><td>GO:0009987</td></tr>
<tr><td>2117</td><td>233</td><td>GO Biological Process</td><td>Macromolecule localization</td><td>1.25E-14</td><td>HSPA8|STAT5A|NPC2|BET1|BAG4|SPNS1|ARMCX3|ARL3|GOT2|PIP4K2</td><td>4.4E-18</td><td>GO:0033036</td></tr>
<tr><td>1497</td><td>182</td><td>GO Biological Process</td><td>Small molecule metabolic process</td><td>1.55E-14</td><td>W5NPK5_SHEEP|PFAS|PTGR2|W5NRI1_SHEEP|GLUD1|W5NRS0_SHEEP|</td><td>6.83E-18</td><td>GO:0044281</td></tr>
<tr><td>1509</td><td>180</td><td>GO Biological Process</td><td>Organic substance catabolic process</td><td>1.12E-13</td><td>PABPC1|HSPA8|GLUD1|PLCD1|FBXO6|ALDH6A1|PSMA4|PNPLA6|RIPK1</td><td>5.93E-17</td><td>GO:1901575</td></tr>
<tr><td>8199</td><td>639</td><td>GO Biological Process</td><td>Metabolic process</td><td>1.47E-12</td><td>COX2|PABPC1|W5NPK5_SHEEP|HSPA8|CPM|DRAP1|SULT1C4|EIF3I|PF</td><td>9.06E-16</td><td>GO:0008152</td></tr>
<tr><td>506</td><td>85</td><td>GO Biological Process</td><td>Actin cytoskeleton organization</td><td>3.37E-12</td><td>CAPZA2|SETD3|TPM4|EVL|PSTPIP1|RAC1|FSCN1|SPTBN1|CORO1A|W5</td><td>2.37E-15</td><td>GO:0030036</td></tr>
<tr><td>4284</td><td>380</td><td>GO Biological Process</td><td>Localization</td><td>4.08E-12</td><td>COX2|HSPA8|TBC1D2B|GSDMD|STAT5A|NPC2|RALBP1|HFE|BET1|BAG</td><td>3.24E-15</td><td>GO:0051179</td></tr>
<tr><td>2416</td><td>245</td><td>GO Biological Process</td><td>Cellular localization</td><td>4.55E-12</td><td>HSPA8|NPC2|HFE|BET1|BAG4|ARMCX3|ARL3|PIP4K2A|CDH5|ARF5|DY</td><td>4.01E-15</td><td>GO:0051641</td></tr>
<tr><td>548</td><td>88</td><td>GO Biological Process</td><td>Actin filament-based process</td><td>7.99E-12</td><td>CAPZA2|SETD3|TPM4|EVL|PSTPIP1|RAC1|FSCN1|SPTBN1|CORO1A|W5</td><td>7.75E-15</td><td>GO:0030029</td></tr>
<tr><td>1701</td><td>188</td><td>GO Biological Process</td><td>Protein localization</td><td>9.65E-12</td><td>HSPA8|BET1|BAG4|ARMCX3|ARL3|CDH5|ARF5|STXBP2|NECAP1|PACS1</td><td>1.02E-14</td><td>GO:0008104</td></tr>
<tr><td>1955</td><td>207</td><td>GO Biological Process</td><td>Regulation of cellular component organization</td><td>1.55E-11</td><td>W5NPK5_SHEEP|HSPA8|CAPZA2|W5NRI1_SHEEP|RALBP1|HFE|BAG4|T</td><td>1.92E-14</td><td>GO:0051128</td></tr>
<tr><td>2039</td><td>213</td><td>GO Biological Process</td><td>Cellular response to chemical stimulus</td><td>1.97E-11</td><td>HSPA8|IFNGR1|FLT4|STAT5A|RAP1B|HFE|BAG4|CHI3L1|COL1A2|PGF|</td><td>2.6E-14</td><td>GO:0070887</td></tr>
<tr><td>3596</td><td>323</td><td>GO Biological Process</td><td>Transport</td><td>1.75E-10</td><td>COX2|HSPA8|TBC1D2B|GSDMD|STAT5A|NPC2|RALBP1|HFE|BET1|SPN</td><td>2.47E-13</td><td>GO:0006810</td></tr>
<tr><td>3748</td><td>333</td><td>GO Biological Process</td><td>Establishment of localization</td><td>2.26E-10</td><td>COX2|HSPA8|TBC1D2B|GSDMD|STAT5A|NPC2|RALBP1|HFE|BET1|SPN</td><td>3.38E-13</td><td>GO:0051234</td></tr>
<tr><td>7705</td><td>592</td><td>GO Biological Process</td><td>Organic substance metabolic process</td><td>4.91E-10</td><td>PABPC1|W5NPK5_SHEEP|HSPA8|CPM|DRAP1|EIF3I|PFAS|SETD3|FLT4|</td><td>7.78E-13</td><td>GO:0071704</td></tr>
<tr><td>429</td><td>71</td><td>GO Biological Process</td><td>Endocytosis</td><td>5.67E-10</td><td>HSPA8|TBC1D2B|RALBP1|RAC1|NECAP1|STAB1|CD163|CD81|LGALS3B</td><td>9.49E-13</td><td>GO:0006897</td></tr>
<tr><td>315</td><td>59</td><td>GO Biological Process</td><td>Regulation of actin cytoskeleton organization</td><td>5.67E-10</td><td>CAPZA2|BAG4|EVL|RAC1|FSCN1|SPTBN1|CORO1A|AVIL|FLNA|GSN|TW</td><td>9.84E-13</td><td>GO:0032956</td></tr>
<tr><td>2016</td><td>205</td><td>GO Biological Process</td><td>Regulation of catalytic activity</td><td>5.67E-10</td><td>W5NPK5_SHEEP|TBC1D2B|FLT4|W5NRI1_SHEEP|RASA3|TFPI2|A2M|RA</td><td>9.63E-13</td><td>GO:0050790</td></tr>
<tr><td>344</td><td>62</td><td>GO Biological Process</td><td>Regulation of actin filament-based process</td><td>5.79E-10</td><td>CAPZA2|BAG4|EVL|RAC1|FSCN1|SPTBN1|CORO1A|AVIL|FLNA|GSN|TW</td><td>1.12E-12</td><td>GO:0032970</td></tr>
<tr><td>2635</td><td>250</td><td>GO Biological Process</td><td>Regulation of molecular function</td><td>9.1E-10</td><td>W5NPK5_SHEEP|TBC1D2B|FLT4|W5NRI1_SHEEP|RASA3|TFPI2|A2M|RA</td><td>1.84E-12</td><td>GO:0065009</td></tr>
<tr><td>1990</td><td>201</td><td>GO Biological Process</td><td>Response to organic substance</td><td>1.41E-09</td><td>HSPA8|IFNGR1|FLT4|STAT5A|FBXO6|RAP1B|HFE|BAG4|CHI3L1|COL1A</td><td>2.98E-12</td><td>GO:0010033</td></tr>
<tr><td>1160</td><td>135</td><td>GO Biological Process</td><td>Cytoskeleton organization</td><td>1.7E-09</td><td>CAPZA2|SETD3|MAP3K20|TPM4|W5NSS8_SHEEP|SPAG1|TPX2|EVL|PST</td><td>3.75E-12</td><td>GO:0007010</td></tr>
<tr><td>6572</td><td>515</td><td>GO Biological Process</td><td>Cellular metabolic process</td><td>2.44E-09</td><td>COX2|PABPC1|W5NPK5_SHEEP|HSPA8|CPM|DRAP1|SULT1C4|EIF3I|PF</td><td>5.59E-12</td><td>GO:0044237</td></tr>
<tr><td>1037</td><td>124</td><td>GO Biological Process</td><td>Regulation of organelle organization</td><td>2.59E-09</td><td>CAPZA2|RALBP1|BAG4|TPX2|EVL|PIP4K2A|CDH5|RAC1|LOC101104501</td><td>6.15E-12</td><td>GO:0033043</td></tr>
<tr><td>481</td><td>74</td><td>GO Biological Process</td><td>Regulation of cytoskeleton organization</td><td>2.9E-09</td><td>CAPZA2|BAG4|TPX2|EVL|CDH5|RAC1|FSCN1|SPTBN1|CORO1A|AVIL|FL</td><td>7.15E-12</td><td>GO:0051493</td></tr>
<tr><td>878</td><td>110</td><td>GO Biological Process</td><td>Regulation of hydrolase activity</td><td>3.28E-09</td><td>W5NPK5_SHEEP|TBC1D2B|W5NRI1_SHEEP|RASA3|TFPI2|A2M|RALBP1|</td><td>8.37E-12</td><td>GO:0051336</td></tr>
<tr><td>1071</td><td>126</td><td>GO Biological Process</td><td>Establishment of protein localization</td><td>4.16E-09</td><td>HSPA8|BET1|ARL3|ARF5|STXBP2|NECAP1|IPO5|SCAMP2|SEL1L|SPTBN</td><td>1.1E-11</td><td>GO:0045184</td></tr>
<tr><td>156</td><td>38</td><td>GO Biological Process</td><td>Regulation of actin polymerization or depolymerization</td><td>6.39E-09</td><td>CAPZA2|BAG4|EVL|SPTBN1|CORO1A|AVIL|GSN|TWF2|ARPC4|SCIN|WD</td><td>1.75E-11</td><td>GO:0008064</td></tr>
<tr><td>5039</td><td>411</td><td>GO Biological Process</td><td>Organonitrogen compound metabolic process</td><td>8.62E-09</td><td>HSPA8|CPM|EIF3I|PFAS|SETD3|FLT4|ELMSAN1|MAP3K20|GLUD1|EIF3</td><td>2.51E-11</td><td>GO:1901564</td></tr>
<tr><td>308</td><td>55</td><td>GO Biological Process</td><td>Regulation of cellular component size</td><td>9.76E-09</td><td>CAPZA2|BAG4|EVL|RTN4|SPTBN1|CORO1A|W5P2V0_SHEEP|PICALM|V</td><td>2.92E-11</td><td>GO:0032535</td></tr>
<tr><td>357</td><td>60</td><td>GO Biological Process</td><td>Regulation of protein-containing complex assembly</td><td>1.03E-08</td><td>CAPZA2|BAG4|EVL|CDH5|FSCN1|LCAT|SPTBN1|CORO1A|GBA|AVIL|MS</td><td>3.27E-11</td><td>GO:0043254</td></tr>
<tr><td>1237</td><td>138</td><td>GO Biological Process</td><td>Intracellular transport</td><td>1.03E-08</td><td>HSPA8|NPC2|BET1|ARMCX3|ARL3|PIP4K2A|ARF5|DYNC1I2|STXBP2|TR</td><td>3.21E-11</td><td>GO:0046907</td></tr>
<tr><td>2276</td><td>218</td><td>GO Biological Process</td><td>Regulation of protein metabolic process</td><td>1.03E-08</td><td>PABPC1|W5NPK5_SHEEP|EIF3I|FLT4|W5NRI1_SHEEP|EIF3A|TFPI2|A2M</td><td>3.18E-11</td><td>GO:0051246</td></tr>
<tr><td>5055</td><td>411</td><td>GO Biological Process</td><td>Cellular component organization</td><td>1.15E-08</td><td>W5NPK5_SHEEP|HSPA8|CAPZA2|EIF3I|SETD3|LIMS1|GSDMD|W5NRD9</td><td>3.83E-11</td><td>GO:0016043</td></tr>
<tr><td>248</td><td>48</td><td>GO Biological Process</td><td>Regulation of actin filament organization</td><td>1.42E-08</td><td>CAPZA2|BAG4|EVL|SPTBN1|CORO1A|AVIL|FLNA|GSN|TWF2|ARPC4|SC</td><td>4.88E-11</td><td>GO:0110053</td></tr>
<tr><td>993</td><td>117</td><td>GO Biological Process</td><td>Protein transport</td><td>1.67E-08</td><td>HSPA8|BET1|ARL3|ARF5|STXBP2|NECAP1|IPO5|SCAMP2|SEL1L|SPTBN</td><td>5.88E-11</td><td>GO:0015031</td></tr>
<tr><td>261</td><td>49</td><td>GO Biological Process</td><td>Actin filament organization</td><td>2.13E-08</td><td>TPM4|EVL|PSTPIP1|RAC1|FSCN1|CORO1A|W5P2V0_SHEEP|INPPL1|AVI</td><td>7.78E-11</td><td>GO:0007015</td></tr>
<tr><td>182</td><td>40</td><td>GO Biological Process</td><td>Regulation of protein polymerization</td><td>2.13E-08</td><td>CAPZA2|BAG4|EVL|CDH5|SPTBN1|CORO1A|AVIL|GSN|TWF2|ARPC4|SC</td><td>7.7E-11</td><td>GO:0032271</td></tr>
<tr><td>742</td><td>95</td><td>GO Biological Process</td><td>Regulation of cell migration</td><td>2.24E-08</td><td>FLT4|STAT5A|ITGA6|BAG4|EVL|PGF|CDH5|RAC1|SASH1|MAP2K3|RTN</td><td>8.49E-11</td><td>GO:0030334</td></tr>
<tr><td>339</td><td>57</td><td>GO Biological Process</td><td>Regulation of supramolecular fiber organization</td><td>2.59E-08</td><td>CAPZA2|BAG4|EVL|CDH5|SPTBN1|CORO1A|AVIL|FLNA|GSN|TWF2|AR</td><td>1E-10</td><td>GO:1902903</td></tr>
<tr><td>420</td><td>65</td><td>GO Biological Process</td><td>Carbohydrate metabolic process</td><td>2.81E-08</td><td>MGAT2|CHI3L1|RENBP|GUSB|FUCA2|GLA|GNPDA1|AKR1A1|GAA|RBP</td><td>1.11E-10</td><td>GO:0005975</td></tr>
<tr><td>507</td><td>73</td><td>GO Biological Process</td><td>Supramolecular fiber organization</td><td>3.68E-08</td><td>TPM4|EVL|COL1A2|RIPK1|PSTPIP1|RAC1|TUBG1|KATNA1|FSCN1|COR</td><td>1.49E-10</td><td>GO:0097435</td></tr>
<tr><td>138</td><td>34</td><td>GO Biological Process</td><td>Regulation of actin filament polymerization</td><td>3.75E-08</td><td>CAPZA2|BAG4|EVL|SPTBN1|CORO1A|AVIL|GSN|TWF2|ARPC4|SCIN|SP</td><td>1.55E-10</td><td>GO:0030833</td></tr>
<tr><td>1757</td><td>176</td><td>GO Biological Process</td><td>Phosphorus metabolic process</td><td>3.76E-08</td><td>PFAS|FLT4|MAP3K20|PLCD1|W5NS94_SHEEP|W5NSP0_SHEEP|PNPLA6|</td><td>1.59E-10</td><td>GO:0006793</td></tr>
<tr><td>768</td><td>96</td><td>GO Biological Process</td><td>Oxoacid metabolic process</td><td>4.98E-08</td><td>W5NPK5_SHEEP|PFAS|PTGR2|W5NRI1_SHEEP|GLUD1|ALDH6A1|RENBP</td><td>2.15E-10</td><td>GO:0043436</td></tr>
<tr><td>438</td><td>66</td><td>GO Biological Process</td><td>Regulation of vesicle-mediated transport</td><td>4.98E-08</td><td>W5NPK5_SHEEP|W5NRI1_SHEEP|RAP1B|HFE|FGR|RUFY1|CORO1A|RAB</td><td>2.17E-10</td><td>GO:0060627</td></tr>
<tr><td>1675</td><td>169</td><td>GO Biological Process</td><td>Regulation of localization</td><td>5.29E-08</td><td>W5NPK5_SHEEP|W5NRI1_SHEEP|GLUD1|RAP1B|HFE|BAG4|RIPK1|SPP1</td><td>2.38E-10</td><td>GO:0032879</td></tr>
<tr><td>878</td><td>105</td><td>GO Biological Process</td><td>Positive regulation of cellular component organization</td><td>6.26E-08</td><td>W5NPK5_SHEEP|W5NRI1_SHEEP|RALBP1|HFE|BAG4|EVL|EIF5A-2|PIP4</td><td>2.87E-10</td><td>GO:0051130</td></tr>
<tr><td>5301</td><td>422</td><td>GO Biological Process</td><td>Cellular component organization or biogenesis</td><td>6.28E-08</td><td>W5NPK5_SHEEP|HSPA8|CAPZA2|EIF3I|SETD3|LIMS1|GSDMD|W5NRD9</td><td>2.93E-10</td><td>GO:0071840</td></tr>
<tr><td>821</td><td>100</td><td>GO Biological Process</td><td>Regulation of locomotion</td><td>6.63E-08</td><td>LOC101109940|FLT4|STAT5A|ITGA6|BAG4|EVL|PGF|CDH5|RAC1|SASH</td><td>3.2E-10</td><td>GO:0040012</td></tr>
<tr><td>1423</td><td>149</td><td>GO Biological Process</td><td>Cellular catabolic process</td><td>6.63E-08</td><td>PABPC1|HSPA8|GLUD1|FBXO6|ALDH6A1|PSMA4|RENBP|GUSB|GLA|G</td><td>3.15E-10</td><td>GO:0044248</td></tr>
<tr><td>799</td><td>98</td><td>GO Biological Process</td><td>Organic acid metabolic process</td><td>7.04E-08</td><td>W5NPK5_SHEEP|PFAS|PTGR2|W5NRI1_SHEEP|GLUD1|ALDH6A1|RENBP</td><td>3.47E-10</td><td>GO:0006082</td></tr>
</table>

Supplementary Table 11: Comparative functional enrichment analysis of GMØ (monocyte-derived macrophages differentiated with GM-CSF) and MMØ (monocyte-derived macrophages differentiated with M-CSF) relative to Mo (monocytes at 3 hours). Functional enrichment analysis was performed using the StringApp (STRING database) within Cytoscape (version 3.10.3)[1], focusing on Gene Ontology (GO) Biological Process (BP) terms[2,3]. Red-highlighted cells indicate Gene Ontology Biological Process terms shared between GMØ and MMØ compared to Mo, whereas non-highlighted cells represent subtype-specific enrichment.

<table>
<tr><td>1580</td><td>161</td><td>GO Biological Process</td><td>Establishment of localization in cell</td><td>7.04E-08</td><td>HSPA8|NPC2|HFE|BET1|ARMCX3|ARL3|PIP4K2A|ARF5|DYNC1I2|STXBP</td><td>3.5E-10</td><td>GO:0051649</td></tr>
<tr><td>788</td><td>97</td><td>GO Biological Process</td><td>Regulation of cell motility</td><td>7.07E-08</td><td>LOC101109940|FLT4|STAT5A|ITGA6|BAG4|EVL|PGF|CDH5|RAC1|SASH</td><td>3.61E-10</td><td>GO:2000145</td></tr>
<tr><td>1003</td><td>115</td><td>GO Biological Process</td><td>Organonitrogen compound catabolic process</td><td>7.36E-08</td><td>HSPA8|GLUD1|FBXO6|ALDH6A1|PSMA4|RIPK1|RENBP|GLA|GOT2|GNP</td><td>3.83E-10</td><td>GO:1901565</td></tr>
<tr><td>1756</td><td>174</td><td>GO Biological Process</td><td>Organic substance transport</td><td>8.41E-08</td><td>HSPA8|NPC2|RALBP1|BET1|SPNS1|ARL3|SLC38A7|GOT2|PIP4K2A|ARF</td><td>4.45E-10</td><td>GO:0071702</td></tr>
<tr><td>1736</td><td>172</td><td>GO Biological Process</td><td>Phosphate-containing compound metabolic process</td><td>0.000000107</td><td>PFAS|FLT4|MAP3K20|PLCD1|W5NSP0_SHEEP|PNPLA6|W5NTV6_SHEEP</td><td>5.74E-10</td><td>GO:0006796</td></tr>
<tr><td>54</td><td>21</td><td>GO Biological Process</td><td>Regulation of actin filament depolymerization</td><td>0.000000122</td><td>CAPZA2|SPTBN1|AVIL|GSN|TWF2|SCIN|WDR1|SPTBN2|ADD3|SPTAN1|</td><td>6.68E-10</td><td>GO:0030834</td></tr>
<tr><td>1807</td><td>177</td><td>GO Biological Process</td><td>Immune system process</td><td>0.000000125</td><td>W5NPK5_SHEEP|IFNGR1|FLT4|W5NRI1_SHEEP|STAT5A|ITGA6|EIF2B3|</td><td>6.94E-10</td><td>GO:0002376</td></tr>
<tr><td>799</td><td>97</td><td>GO Biological Process</td><td>Organophosphate metabolic process</td><td>0.000000125</td><td>PFAS|PLCD1|W5NSP0_SHEEP|PNPLA6|W5NTV6_SHEEP|PIP4K2A|NME1</td><td>7E-10</td><td>GO:0019637</td></tr>
<tr><td>754</td><td>93</td><td>GO Biological Process</td><td>Carboxylic acid metabolic process</td><td>0.000000135</td><td>W5NPK5_SHEEP|PFAS|PTGR2|W5NRI1_SHEEP|GLUD1|ALDH6A1|RENBP</td><td>7.74E-10</td><td>GO:0019752</td></tr>
<tr><td>1562</td><td>158</td><td>GO Biological Process</td><td>Cellular response to organic substance</td><td>0.000000146</td><td>HSPA8|IFNGR1|FLT4|STAT5A|RAP1B|HFE|BAG4|CHI3L1|COL1A2|PGF|</td><td>8.48E-10</td><td>GO:0071310</td></tr>
<tr><td>864</td><td>102</td><td>GO Biological Process</td><td>Carbohydrate derivative metabolic process</td><td>0.000000167</td><td>PFAS|MGAT2|W5NRS0_SHEEP|FBXO6|W5NS94_SHEEP|W5NSP0_SHEEP</td><td>9.85E-10</td><td>GO:1901135</td></tr>
<tr><td>397</td><td>60</td><td>GO Biological Process</td><td>Regulation of anatomical structure size</td><td>0.000000218</td><td>CAPZA2|BAG4|EVL|RTN4|SPTBN1|CORO1A|SOD1-2|W5P2V0_SHEEP|PI</td><td>1.31E-09</td><td>GO:0090066</td></tr>
<tr><td>713</td><td>88</td><td>GO Biological Process</td><td>Regulation of anatomical structure morphogenesis</td><td>0.00000036</td><td>W5NPK5_SHEEP|LIMS1|W5NRI1_SHEEP|RALBP1|CHI3L1|PGF|CDH5|RA</td><td>2.19E-09</td><td>GO:0022603</td></tr>
<tr><td>1230</td><td>130</td><td>GO Biological Process</td><td>Positive regulation of protein metabolic process</td><td>0.000000465</td><td>PABPC1|W5NPK5_SHEEP|FLT4|W5NRI1_SHEEP|RALBP1|HFE|BAG4|CHI</td><td>2.87E-09</td><td>GO:0051247</td></tr>
<tr><td>73</td><td>23</td><td>GO Biological Process</td><td>Chaperone-mediated protein folding</td><td>0.000000478</td><td>HSPA8|TCP1|CCT3|CD74|TOR1B|HSPA1A-2|CCT6A|FKBP4|HSPH1|CCT7</td><td>2.99E-09</td><td>GO:0061077</td></tr>
<tr><td>42</td><td>18</td><td>GO Biological Process</td><td>Negative regulation of actin filament depolymerization</td><td>0.00000051</td><td>CAPZA2|SPTBN1|AVIL|GSN|TWF2|SCIN|SPTBN2|ADD3|SPTAN1|CAPZB</td><td>3.26E-09</td><td>GO:0030835</td></tr>
<tr><td>461</td><td>65</td><td>GO Biological Process</td><td>Nucleobase-containing small molecule metabolic process</td><td>0.00000051</td><td>PFAS|W5NRS0_SHEEP|ALDH6A1|W5NS94_SHEEP|W5NSP0_SHEEP|W5N</td><td>3.24E-09</td><td>GO:0055086</td></tr>
<tr><td>37</td><td>17</td><td>GO Biological Process</td><td>Actin filament capping</td><td>0.000000599</td><td>CAPZA2|SPTBN1|AVIL|GSN|TWF2|SCIN|SPTBN2|ADD3|SPTAN1|CAPZB</td><td>3.9E-09</td><td>GO:0051693</td></tr>
<tr><td>1391</td><td>142</td><td>GO Biological Process</td><td>Nitrogen compound transport</td><td>0.000000599</td><td>HSPA8|RALBP1|BET1|ARL3|SLC38A7|ARF5|ABCG2|STXBP2|NECAP1|LO</td><td>3.96E-09</td><td>GO:0071705</td></tr>
<tr><td>3085</td><td>265</td><td>GO Biological Process</td><td>Regulation of biological quality</td><td>0.000000722</td><td>PABPC1|HSPA8|CAPZA2|GLUD1|STAT5A|TFPI2|RAP1B|NPC2|HFE|BAG</td><td>4.84E-09</td><td>GO:0065008</td></tr>
<tr><td>7312</td><td>543</td><td>GO Biological Process</td><td>Primary metabolic process</td><td>0.000000766</td><td>PABPC1|W5NPK5_SHEEP|HSPA8|CPM|DRAP1|EIF3I|PFAS|SETD3|FLT4|</td><td>5.19E-09</td><td>GO:0044238</td></tr>
<tr><td>1223</td><td>128</td><td>GO Biological Process</td><td>Regulation of cell death</td><td>0.000000957</td><td>FLT4|MAP3K20|STAT5A|ITGA6|EIF5A-2|RIPK1|CDH5|W5NUX2_SHEEP|</td><td>6.57E-09</td><td>GO:0010941</td></tr>
<tr><td>133</td><td>30</td><td>GO Biological Process</td><td>Receptor-mediated endocytosis</td><td>0.00000152</td><td>CD81|W5P2V0_SHEEP|AP2A2|PICALM|FCHO2|LRP1|CTSL|MSR1|FCGR</td><td>1.06E-08</td><td>GO:0006898</td></tr>
<tr><td>20</td><td>13</td><td>GO Biological Process</td><td>Barbed-end actin filament capping</td><td>0.00000172</td><td>CAPZA2|AVIL|GSN|TWF2|SCIN|ADD3|CAPZB|SVIL|ADD1|CAPZA1-2|TW</td><td>1.21E-08</td><td>GO:0051016</td></tr>
<tr><td>417</td><td>59</td><td>GO Biological Process</td><td>Positive regulation of cell migration</td><td>0.00000217</td><td>FLT4|STAT5A|ITGA6|BAG4|PGF|CDH5|SASH1|MAP2K3|RTN4|SPAG9|F</td><td>1.55E-08</td><td>GO:0030335</td></tr>
<tr><td>3265</td><td>274</td><td>GO Biological Process</td><td>Organelle organization</td><td>0.00000269</td><td>CAPZA2|SETD3|W5NRD9_SHEEP|MAP3K20|TPM4|W5NSS8_SHEEP|BET</td><td>1.94E-08</td><td>GO:0006996</td></tr>
<tr><td>146</td><td>31</td><td>GO Biological Process</td><td>Carbohydrate derivative catabolic process</td><td>0.0000028</td><td>FBXO6|RENBP|GUSB|FUCA2|GLA|GNPDA1|SGSH|GBA|NEU1|HEXB|LO</td><td>2.04E-08</td><td>GO:1901136</td></tr>
<tr><td>316</td><td>49</td><td>GO Biological Process</td><td>Small molecule catabolic process</td><td>0.00000282</td><td>GLUD1|ALDH6A1|RENBP|GOT2|GNPDA1|NQO2|AKR1A1|TPI1|ALDH1L</td><td>2.09E-08</td><td>GO:0044282</td></tr>
<tr><td>1202</td><td>124</td><td>GO Biological Process</td><td>Regulation of immune system process</td><td>0.00000325</td><td>W5NPK5_SHEEP|W5NRI1_SHEEP|STAT5A|HFE|LOC443162|EIF2B3|PGF|</td><td>2.46E-08</td><td>GO:0002682</td></tr>
<tr><td>625</td><td>77</td><td>GO Biological Process</td><td>Regulation of cell adhesion</td><td>0.00000325</td><td>LIMS1|STAT5A|HFE|LOC443162|BAG4|PLEKHA2|PEAK1|W5NYC7_SHEE</td><td>2.44E-08</td><td>GO:0030155</td></tr>
<tr><td>435</td><td>60</td><td>GO Biological Process</td><td>Positive regulation of cell motility</td><td>0.00000347</td><td>LOC101109940|FLT4|STAT5A|ITGA6|BAG4|PGF|CDH5|SASH1|MAP2K3|</td><td>2.66E-08</td><td>GO:2000147</td></tr>
<tr><td>5423</td><td>417</td><td>GO Biological Process</td><td>Positive regulation of biological process</td><td>0.00000372</td><td>PABPC1|W5NPK5_SHEEP|HSPA8|DRAP1|SETD3|LIMS1|GSDMD|LOC101</td><td>2.88E-08</td><td>GO:0048518</td></tr>
<tr><td>1340</td><td>134</td><td>GO Biological Process</td><td>Regulation of transport</td><td>0.00000449</td><td>W5NPK5_SHEEP|W5NRI1_SHEEP|GLUD1|RAP1B|HFE|BAG4|RIPK1|SPP1</td><td>3.52E-08</td><td>GO:0051049</td></tr>
<tr><td>93</td><td>24</td><td>GO Biological Process</td><td>Carbohydrate catabolic process</td><td>0.00000451</td><td>GUSB|GAA|ALDOA|NEU1|GPI|TPI1|PYGB|ALDOB|GAPDH|GM2A|PGK2</td><td>3.58E-08</td><td>GO:0016052</td></tr>
<tr><td>450</td><td>61</td><td>GO Biological Process</td><td>Positive regulation of locomotion</td><td>0.00000451</td><td>LOC101109940|FLT4|STAT5A|ITGA6|BAG4|PGF|CDH5|SASH1|MAP2K3|</td><td>3.57E-08</td><td>GO:0040017</td></tr>
<tr><td>58</td><td>19</td><td>GO Biological Process</td><td>Actin polymerization or depolymerization</td><td>0.00000516</td><td>EVL|PSTPIP1|AVIL|GSN|TWF2|GAS7|ARPC4|SCIN|AIF1|WDR1|WASHC</td><td>4.18E-08</td><td>GO:0008154</td></tr>
<tr><td>206</td><td>37</td><td>GO Biological Process</td><td>Protein folding</td><td>0.00000531</td><td>HSPA8|DNAJC3|AHSA1|HSP90AA1|STIP1|TCP1|CCT3|CD74|TOR1B|HSP</td><td>4.38E-08</td><td>GO:0006457</td></tr>
<tr><td>623</td><td>76</td><td>GO Biological Process</td><td>Response to cytokine</td><td>0.00000531</td><td>IFNGR1|STAT5A|BAG4|CHI3L1|RIPK1|BTK|W5NWX4_SHEEP|W5NXH3_</td><td>4.35E-08</td><td>GO:0034097</td></tr>
<tr><td>1281</td><td>129</td><td>GO Biological Process</td><td>Positive regulation of molecular function</td><td>0.00000539</td><td>TBC1D2B|FLT4|RALBP1|HFE|ITGA6|CHI3L1|TPX2|RIPK1|W5NU48_SHEE</td><td>4.51E-08</td><td>GO:0044093</td></tr>
<tr><td>566</td><td>71</td><td>GO Biological Process</td><td>Cellular response to cytokine stimulus</td><td>0.0000054</td><td>IFNGR1|STAT5A|BAG4|CHI3L1|RIPK1|BTK|W5NWX4_SHEEP|W5NXH3_</td><td>4.57E-08</td><td>GO:0071345</td></tr>
<tr><td>155</td><td>31</td><td>GO Biological Process</td><td>Regulation of endocytosis</td><td>0.00000783</td><td>W5NPK5_SHEEP|W5NRI1_SHEEP|HFE|RUFY1|PICALM|CD22|W5P9L9_S</td><td>6.69E-08</td><td>GO:0030100</td></tr>
<tr><td>1127</td><td>116</td><td>GO Biological Process</td><td>Regulation of programmed cell death</td><td>0.00000931</td><td>FLT4|MAP3K20|STAT5A|ITGA6|EIF5A-2|RIPK1|CDH5|W5NUX2_SHEEP|</td><td>8.03E-08</td><td>GO:0043067</td></tr>
<tr><td>408</td><td>56</td><td>GO Biological Process</td><td>Nucleoside phosphate metabolic process</td><td>0.0000103</td><td>PFAS|W5NSP0_SHEEP|W5NTV6_SHEEP|NME1-NME2|PMVK|DHODH|SL</td><td>8.95E-08</td><td>GO:0006753</td></tr>
<tr><td>3268</td><td>270</td><td>GO Biological Process</td><td>Regulation of response to stimulus</td><td>0.0000109</td><td>W5NPK5_SHEEP|LIMS1|FLT4|MAP3K20|W5NRI1_SHEEP|STAT5A|HFE|I</td><td>9.61E-08</td><td>GO:0048583</td></tr>
<tr><td>6684</td><td>494</td><td>GO Biological Process</td><td>Nitrogen compound metabolic process</td><td>0.0000112</td><td>PABPC1|HSPA8|CPM|DRAP1|EIF3I|PFAS|SETD3|FLT4|ELMSAN1|MAP3</td><td>9.98E-08</td><td>GO:0006807</td></tr>
<tr><td>377</td><td>53</td><td>GO Biological Process</td><td>Small molecule biosynthetic process</td><td>0.0000112</td><td>W5NRS0_SHEEP|GOT2|AKR1A1|MECR|TMED8|PMVK|MTHFD1L|RBP4|</td><td>9.99E-08</td><td>GO:0044283</td></tr>
<tr><td>84</td><td>22</td><td>GO Biological Process</td><td>Regulation of protein depolymerization</td><td>0.0000116</td><td>CAPZA2|SPTBN1|AVIL|GSN|TWF2|SCIN|WDR1|SPTBN2|ADD3|SPTAN1|</td><td>0.000000106</td><td>GO:1901879</td></tr>
<tr><td>2159</td><td>192</td><td>GO Biological Process</td><td>Regulation of multicellular organismal process</td><td>0.0000125</td><td>W5NPK5_SHEEP|SETD3|GSDMD|FLT4|W5NRI1_SHEEP|STAT5A|HFE|LO</td><td>0.000000114</td><td>GO:0051239</td></tr>
<tr><td>401</td><td>55</td><td>GO Biological Process</td><td>Nucleotide metabolic process</td><td>0.0000129</td><td>PFAS|W5NSP0_SHEEP|W5NTV6_SHEEP|NME1-NME2|PMVK|DHODH|SL</td><td>0.000000119</td><td>GO:0009117</td></tr>
<tr><td>70</td><td>20</td><td>GO Biological Process</td><td>Negative regulation of protein polymerization</td><td>0.0000129</td><td>CAPZA2|CDH5|SPTBN1|AVIL|GSN|TWF2|SCIN|SPTBN2|ADD3|SPTAN1|</td><td>0.000000122</td><td>GO:0032272</td></tr>
<tr><td>4831</td><td>374</td><td>GO Biological Process</td><td>Positive regulation of cellular process</td><td>0.0000129</td><td>PABPC1|W5NPK5_SHEEP|HSPA8|DRAP1|SETD3|LIMS1|LOC101109940|</td><td>0.00000012</td><td>GO:0048522</td></tr>
<tr><td>412</td><td>56</td><td>GO Biological Process</td><td>Regulation of peptidase activity</td><td>0.0000129</td><td>W5NPK5_SHEEP|W5NRI1_SHEEP|TFPI2|A2M|ITIH1|RENBP|NLRP2|RAR</td><td>0.00000012</td><td>GO:0052547</td></tr>
<tr><td>1100</td><td>113</td><td>GO Biological Process</td><td>Regulation of apoptotic process</td><td>0.0000137</td><td>FLT4|MAP3K20|STAT5A|ITGA6|EIF5A-2|RIPK1|CDH5|W5NUX2_SHEEP|</td><td>0.000000131</td><td>GO:0042981</td></tr>
<tr><td>240</td><td>39</td><td>GO Biological Process</td><td>Regulation of cell morphogenesis</td><td>0.0000203</td><td>LIMS1|RAC1|FGR|CORO1A|ALDOA|LOC101114535|W5P2V0_SHEEP|SE</td><td>0.000000197</td><td>GO:0022604</td></tr>
<tr><td>147</td><td>29</td><td>GO Biological Process</td><td>Negative regulation of cytoskeleton organization</td><td>0.0000231</td><td>CAPZA2|CDH5|SPTBN1|CORO1A|AVIL|GSN|TWF2|SCIN|SPTBN2|ADD3|</td><td>0.000000226</td><td>GO:0051494</td></tr>
<tr><td>558</td><td>68</td><td>GO Biological Process</td><td>Negative regulation of cellular component organization</td><td>0.0000236</td><td>CAPZA2|EVL|CDH5|LOC101104501|RTN4|PSMD10|SPTBN1|CORO1A|G</td><td>0.000000233</td><td>GO:0051129</td></tr>
</table>

Supplementary Table 11: Comparative functional enrichment analysis of GMØ (monocyte-derived macrophages differentiated with GM-CSF) and MMØ (monocyte-derived macrophages differentiated with M-CSF) relative to Mo (monocytes at 3 hours). Functional enrichment analysis was performed using the StringApp (STRING database) within Cytoscape (version 3.10.3)[1], focusing on Gene Ontology (GO) Biological Process (BP) terms[2,3]. Red-highlighted cells indicate Gene Ontology Biological Process terms shared between GMØ and MMØ compared to Mo, whereas non-highlighted cells represent subtype-specific enrichment.

67	19	GO Biological Process	Negative regulation of protein depolymerization	0.0000277	CAPZA2|SPTBN1|AVIL|GSN|TWF2|SCIN|SPTBN2|ADD3|SPTAN1|CAPZB	0.000000276	GO:1901880
1190	118	GO Biological Process	Biological process involved in interspecies interaction between organi	0.0000366	W5NPK5_SHEEP|HSPA8|IFNGR1|GSDMD|W5NRI1_SHEEP|NPC2|FUCA2	0.000000368	GO:0044419
674	77	GO Biological Process	Regulation of proteolysis	0.0000386	W5NPK5_SHEEP|W5NRI1_SHEEP|TFPI2|A2M|HFE|ITIH1|RENBP|NLRP2|	0.000000391	GO:0030162
62	18	GO Biological Process	Negative regulation of actin filament polymerization	0.0000414	CAPZA2|SPTBN1|AVIL|GSN|TWF2|SCIN|SPTBN2|ADD3|SPTAN1|CAPZB	0.000000423	GO:0030837
581	69	GO Biological Process	Regulation of cytokine production	0.0000434	W5NPK5_SHEEP|GSDMD|FLT4|W5NRI1_SHEEP|STAT5A|HFE|LOC44316	0.000000448	GO:0001817
42	15	GO Biological Process	Regulation of lamellipodium organization	0.0000434	FSCN1|TWF2|CDC42|CAPZB|ACTR3|LOC101105107|CORO1B|EPHA2|W	0.000000447	GO:1902743
144	28	GO Biological Process	Response to toxic substance	0.0000446	RALBP1|PRDX1|AKR1A1|ABCG2|CYP1A1|NQO1|CLIC2|SOD1-2|LOC101	0.000000469	GO:0009636
153	29	GO Biological Process	Negative regulation of supramolecular fiber organization	0.0000446	CAPZA2|CDH5|SPTBN1|CORO1A|AVIL|GSN|TWF2|SCIN|SPTBN2|ADD3	0.000000468	GO:1902904
468	59	GO Biological Process	Positive regulation of hydrolase activity	0.0000509	TBC1D2B|RALBP1|ITGA6|NLRP2|TBC1D9B|RGS10|CTSC|PDCD5|LOC101	0.000000543	GO:0051345
119	25	GO Biological Process	Regulation of protein-containing complex disassembly	0.0000516	CAPZA2|EIF5A-2|SPTBN1|GBA|AVIL|GSN|TWF2|SCIN|WDR1|SPTBN2|A	0.000000554	GO:0043244
947	98	GO Biological Process	Positive regulation of catalytic activity	0.0000638	TBC1D2B|FLT4|RALBP1|ITGA6|CHI3L1|TPX2|NLRP2|TBC1D9B|RGS10|S	0.000000691	GO:0043085
266	40	GO Biological Process	Regulation of protein stability	0.0000748	BAG4|RTN4|CD81|HSP90AA1|TCP1|PHB2|FLNA|GSN|LMNA|WFS1|GL	0.000000817	GO:0031647
374	50	GO Biological Process	Regulation of endopeptidase activity	0.0000792	W5NPK5_SHEEP|W5NRI1_SHEEP|TFPI2|A2M|ITIH1|NLRP2|RARRES1|PD	0.000000872	GO:0052548
98	22	GO Biological Process	Cellular detoxification	0.000091	PRDX1|AKR1A1|ABCG2|NQO1|CLIC2|SOD1-2|GPX7|LOC101116273|ES	0.00000101	GO:1990748
333	46	GO Biological Process	Ribose phosphate metabolic process	0.0000953	PFAS|W5NSP0_SHEEP|W5NTV6_SHEEP|NME1-NME2|PMVK|DHODH|SL	0.00000107	GO:0019693
107	23	GO Biological Process	Detoxification	0.0000981	RALBP1|PRDX1|AKR1A1|ABCG2|NQO1|CLIC2|SOD1-2|GPX7|LOC10111	0.00000111	GO:0098754
971	99	GO Biological Process	Response to oxygen-containing compound	0.0000981	STAT5A|RAP1B|COL1A2|RIPK1|PRDX1|GOT2|AKR1A1|RGS10|TAB2|SP	0.00000111	GO:1901700
757	82	GO Biological Process	Regulation of cellular localization	0.0001	GLUD1|HFE|BAG4|IPO5|RTN4|CD81|RBP4|SPTBN1|RAB11B|CNST|PDC	0.00000116	GO:0060341
335	46	GO Biological Process	Purine-containing compound metabolic process	0.00011	PFAS|W5NRS0_SHEEP|W5NSP0_SHEEP|W5NTV6_SHEEP|NME1-NME2|F	0.00000124	GO:0072521
614	70	GO Biological Process	Intracellular protein transport	0.00012	HSPA8|ARF5|STXBP2|IPO5|SEL1L|AP1B1|HIKESHI|AP2A2|PHB2|VPS16	0.00000142	GO:0006886
4510	345	GO Biological Process	Negative regulation of biological process	0.00013	PABPC1|W5NPK5_SHEEP|HSPA8|DRAP1|CAPZA2|FLT4|ELMSAN1|MAP3	0.00000147	GO:0048519
2640	219	GO Biological Process	Response to stress	0.00014	W5NPK5_SHEEP|HSPA8|IFNGR1|GSDMD|MAP3K20|W5NRI1_SHEEP|FB	0.00000168	GO:0006950
156	28	GO Biological Process	Cellular modified amino acid metabolic process	0.00016	MTHFD1L|W5NZK6_SHEEP|CLIC2|SOD1-2|CLIC1|TG|W5P716_SHEEP|A	0.00000188	GO:0006575
157	28	GO Biological Process	Nucleoside triphosphate metabolic process	0.00017	W5NSP0_SHEEP|W5NTV6_SHEEP|NME1-NME2|SLC25A13|ALDOA|GPI|	0.0000021	GO:0009141
148	27	GO Biological Process	Phagocytosis	0.00018	NCF2|RAC1|CORO1A|VAV1|GSN|CD302|LRP1|AIF1|CDC42|ALOX15|UN	0.00000218	GO:0006909
322	44	GO Biological Process	Ribonucleotide metabolic process	0.00019	PFAS|W5NSP0_SHEEP|W5NTV6_SHEEP|NME1-NME2|PMVK|DHODH|SL	0.00000234	GO:0009259
36	13	GO Biological Process	Membrane lipid catabolic process	0.00019	GLA|GBA|NEU1|HEXB|LOC101112162|SGPL1|FUCA1|SMPDL3A|GM2A	0.00000236	GO:0046466
1758	156	GO Biological Process	Positive regulation of response to stimulus	0.00019	W5NPK5_SHEEP|LIMS1|FLT4|MAP3K20|W5NRI1_SHEEP|STAT5A|HFE|B	0.00000237	GO:0048584
30	12	GO Biological Process	Clathrin-dependent endocytosis	0.00019	AP2A2|PICALM|FCHO2|AP2S1|ARRB1|CLTA|LMBRD1|HIP1|CLTC|AP2A	0.00000245	GO:0072583
839	87	GO Biological Process	Regulation of cellular component biogenesis	0.00022	CAPZA2|BAG4|EVL|PEAK1|PIP4K2A|CDH5|FSCN1|LCAT|SPTBN1|CORO	0.00000276	GO:0044087
179	30	GO Biological Process	Leukocyte migration	0.00022	ITGA6|CORO1A|GBA|LOC101114535|LOC100101238|W5P3V8_SHEEP|V	0.00000283	GO:0050900
151	27	GO Biological Process	Monosaccharide metabolic process	0.00023	FUCA2|AKR1A1|RBP4|ALDOA|GPI|INPPL1|PGD|TPI1|FUCA1|ALDOB|G	0.00000304	GO:0005996
461	56	GO Biological Process	Positive regulation of organelle organization	0.00023	RALBP1|BAG4|EVL|PIP4K2A|LOC101104501|FSCN1|PDCD5|TCP1|MSN	0.00000293	GO:0010638
316	43	GO Biological Process	Purine nucleotide metabolic process	0.00026	PFAS|W5NSP0_SHEEP|W5NTV6_SHEEP|NME1-NME2|PMVK|SLC25A13|	0.00000335	GO:0006163
82	19	GO Biological Process	Cellular response to interferon-gamma	0.00028	IFNGR1|LOC101114535|LOC100101238|W5P3V8_SHEEP|GSN|W5PB20_	0.00000365	GO:0071346
659	72	GO Biological Process	Positive regulation of transport	0.0003	W5NPK5_SHEEP|W5NRI1_SHEEP|GLUD1|HFE|SPP1|IPO5|CD81|RBP4|F	0.00000398	GO:0051050
75	18	GO Biological Process	Membrane docking	0.00032	STXBP2|W5P2V0_SHEEP|MSN|STX4|ESYT2|ESYT1|SNX3|RAB3A|STX7|S	0.00000429	GO:0022406
674	73	GO Biological Process	Regulation of protein localization	0.00033	GLUD1|HFE|BAG4|IPO5|RTN4|CD81|RBP4|SPTBN1|RAB11B|CNST|PDC	0.00000452	GO:0032880
100	21	GO Biological Process	Response to interferon-gamma	0.00033	IFNGR1|LOC101114535|LOC100101238|W5P3V8_SHEEP|GSN|W5PB20_	0.00000439	GO:0034341
287	40	GO Biological Process	Negative regulation of transport	0.00033	RAP1B|BAG4|CORO1A|CLIC2|GDI1|PICALM|TXN|CD74|MMP9|PEA15|	0.00000436	GO:0051051
942	94	GO Biological Process	Positive regulation of gene expression	0.00034	PABPC1|W5NPK5_SHEEP|HSPA8|GSDMD|FLT4|W5NRI1_SHEEP|STAT5A	0.00000473	GO:0010628
204	32	GO Biological Process	Regulation of leukocyte proliferation	0.00034	STAT5A|LOC443162|BTK|CD81|CORO1A|CD22|VSIG4|W5P5N5_SHEEP|	0.00000461	GO:0070663
530	61	GO Biological Process	Regulation of cell activation	0.00037	STAT5A|HFE|LOC443162|BTK|IGHM|W5NYC7_SHEEP|CD81|FGR|CORO	0.00000512	GO:0050865
739	78	GO Biological Process	Cellular response to oxygen-containing compound	0.00037	STAT5A|RAP1B|COL1A2|RIPK1|PRDX1|AKR1A1|RGS10|SPP1|IPO5|NQ	0.00000507	GO:1901701
753	79	GO Biological Process	Cell migration	0.00039	ITGA6|PEAK1|CDH5|RAC1|RTN4|FSCN1|CORO1A|LGALS8|GBA|TOP2B	0.0000054	GO:0016477
16	9	GO Biological Process	Glycolipid catabolic process	0.00039	GLA|GBA|NEU1|HEXB|LOC101112162|FUCA1|GM2A|NAGA|HEXA	0.0000054	GO:0019377
33	12	GO Biological Process	Collagen catabolic process	0.00039	PEPD|MMP3|MMP9|CTSV|CTSL|MMP19|MRC2|CTSB|MMP2|MMP14	0.00000544	GO:0030574
449	54	GO Biological Process	Endomembrane system organization	0.0004	BET1|RTN4|SPTBN1|COG8|LAMTOR1|FOLR2|SOD1-2|HIKESHI|SRGN|C	0.00000571	GO:0010256
302	41	GO Biological Process	Purine ribonucleotide metabolic process	0.00041	PFAS|W5NSP0_SHEEP|W5NTV6_SHEEP|NME1-NME2|PMVK|SLC25A13|	0.0000059	GO:0009150
270	38	GO Biological Process	Alcohol metabolic process	0.00042	NPC2|AKR1A1|PMVK|RBP4|LCAT|CYP1A1|GBA|TPI1|PCBD1|TTR|HSD1	0.00000613	GO:0006066
392	49	GO Biological Process	Organic hydroxy compound metabolic process	0.00042	NPC2|AKR1A1|PMVK|MAOA|RBP4|LCAT|CYP1A1|NQO1|GBA|VHL|TPI	0.00000603	GO:1901615
694	74	GO Biological Process	Regulation of immune response	0.00044	W5NPK5_SHEEP|W5NRI1_SHEEP|HFE|EIF2B3|BTK|RTN4|CD81|FGR|NC	0.00000642	GO:0050776
168	28	GO Biological Process	Protein stabilization	0.00045	BAG4|RTN4|HSP90AA1|TCP1|PHB2|FLNA|WFS1|GLMP|CCT3|CD74|GA	0.00000658	GO:0050821
326	43	GO Biological Process	Regulation of GTPase activity	0.00046	TBC1D2B|RASA3|RALBP1|W5NSN4_SHEEP|ITGA6|TBC1D9B|RGS10|SO	0.00000676	GO:0043087
70	17	GO Biological Process	Dicarboxylic acid metabolic process	0.00046	GLUD1|GOT2|MTHFD1L|ALDH18A1|ALDH1L1|ALDH5A1|ACOT8|GCLC|	0.00000688	GO:0043648
140	25	GO Biological Process	Vacuolar transport	0.00048	HSPA8|ATP6V0D1|IGF2R|VPS16|PSAP|VPS37B|GRN|LAMP1|HSPA1A-2|	0.00000712	GO:0007034

Supplementary Table 11: Comparative functional enrichment analysis of GMØ (monocyte-derived macrophages differentiated with GM-CSF) and MMØ (monocyte-derived macrophages differentiated with M-CSF) relative to Mo (monocytes at 3 hours). Functional enrichment analysis was performed using the StringApp (STRING database) within Cytoscape (version 3.10.3)[1], focusing on Gene Ontology (GO) Biological Process (BP) terms[2,3]. Red-highlighted cells indicate Gene Ontology Biological Process terms shared between GMØ and MMØ compared to Mo, whereas non-highlighted cells represent subtype-specific enrichment.

723	76	GO Biological Process	Regulation of response to external stimulus	0.00051	W5NPK5_SHEEP|W5NRI1_SHEEP|STAT5A|PGF|RIPK1|CDH5|NLRP2|SAS	0.00000765	GO:0032101
329	43	GO Biological Process	Generation of precursor metabolites and energy	0.00055	COX2|GNPDA1|NQO2|GAA|GBA|SLC25A13|ALDOA|GPI|PGD|TPI1|TAL	0.00000829	GO:0006091
221	33	GO Biological Process	Small GTPase mediated signal transduction	0.00055	RAP1B|RALBP1|COL1A2|ARL3|RAC1|RUFY1|GDI1|VAV1|RASGRP4|RRA	0.00000831	GO:0007264
42	13	GO Biological Process	Glycolytic process	0.00062	ALDOA|GPI|TPI1|ALDOB|GAPDH|PGK2|ENO1|GALK1|W5PK04_SHEEP|	0.00000949	GO:0006096
309	41	GO Biological Process	Negative regulation of organelle organization	0.00062	CAPZA2|CDH5|LOC101104501|PSMD10|SPTBN1|CORO1A|VAT1|AVIL|V	0.00000968	GO:0010639
124	23	GO Biological Process	Negative regulation of protein-containing complex assembly	0.00062	CAPZA2|CDH5|SPTBN1|GBA|AVIL|GSN|SIRT2|TWF2|SCIN|SPTBN2|CDC	0.00000953	GO:0031333
310	41	GO Biological Process	Regulation of protein catabolic process	0.00066	PSMD10|CD81|NQO1|GBA|W5P2V0_SHEEP|MSN|VHL|FLNA|USP5|SIR	0.0000104	GO:0042176
288	39	GO Biological Process	Regulation of leukocyte cell-cell adhesion	0.00066	STAT5A|HFE|LOC443162|W5NYC7_SHEEP|CD81|CORO1A|VSIG4|W5P5	0.0000104	GO:1903037
496	57	GO Biological Process	Regulation of leukocyte activation	0.00068	STAT5A|HFE|LOC443162|BTK|IGHM|W5NYC7_SHEEP|CD81|FGR|CORO	0.0000108	GO:0002694
107	21	GO Biological Process	Lysosomal transport	0.00068	HSPA8|IGF2R|VPS16|PSAP|GRN|LAMP1|HSPA1A-2|TSG101|BIN1-2|GG	0.0000109	GO:0007041
367	46	GO Biological Process	Positive regulation of cell adhesion	0.00068	LIMS1|STAT5A|BAG4|PLEKHA2|W5NYC7_SHEEP|CD81|CORO1A|VAV1|	0.0000108	GO:0045785
135	24	GO Biological Process	Purine nucleoside triphosphate metabolic process	0.00071	W5NSP0_SHEEP|W5NTV6_SHEEP|NME1-NME2|SLC25A13|ALDOA|GPI|	0.0000115	GO:0009144
50	14	GO Biological Process	Zymogen activation	0.00071	CTSC|CTSV|CTSL|PYCARD|MSR1|BAK1|F9|CYFIP2|FURIN|LGMN|CTSH|	0.0000114	GO:0031638
117	22	GO Biological Process	Positive regulation of epithelial cell migration	0.00075	FLT4|STAT5A|SASH1|MAP2K3|RTN4|GPI|MAPRE2|MMP9|PLCG2|PRKD	0.0000123	GO:0010634
175	28	GO Biological Process	Regulation of leukocyte migration	0.00078	PGF|RAC1|MPP1|CD81|CORO1A|LOC101114535|MSN|RIPOR2|W5P6T	0.0000129	GO:0002685
24	10	GO Biological Process	Ruffle organization	0.00078	BAG4|INPPL1|CSF1R|AIF1|CORO1B|INPP5K|DBNL|TCIRG1|LIMA1|PLEK	0.0000128	GO:0031529
24	10	GO Biological Process	Amyloid-beta clearance	0.00078	W5NPK5_SHEEP|W5NRI1_SHEEP|PICALM|LRP1|MSR1|C5AR1|CLTC|LRF	0.0000128	GO:0097242
137	24	GO Biological Process	Ribonucleoside triphosphate metabolic process	0.00085	W5NSP0_SHEEP|W5NTV6_SHEEP|NME1-NME2|SLC25A13|ALDOA|GPI|	0.0000143	GO:0009199
337	43	GO Biological Process	Positive regulation of response to external stimulus	0.00085	W5NPK5_SHEEP|W5NRI1_SHEEP|STAT5A|PGF|RIPK1|SASH1|CD81|CTS	0.0000141	GO:0032103
7243	511	GO Biological Process	Response to stimulus	0.00088	W5NPK5_SHEEP|HSPA8|IFNGR1|GSDMD|FLT4|MAP3K20|W5NRI1_SHE	0.0000148	GO:0050896
1254	115	GO Biological Process	Intracellular signal transduction	0.0009	MAP3K20|PLCD1|RAP1B|RALBP1|CHI3L1|COL1A2|RIPK1|BTK|ARL3|RA	0.0000152	GO:0035556
947	92	GO Biological Process	Negative regulation of molecular function	0.00092	W5NPK5_SHEEP|W5NRI1_SHEEP|TFPI2|A2M|HFE|BAG4|ITIH1|RENBP|	0.0000157	GO:0044092
31	11	GO Biological Process	Regulation of lamellipodium assembly	0.00094	FSCN1|TWF2|CDC42|CAPZB|ACTR3|EPHA2|W5PVN6_SHEEP|CYFIP1|FE	0.0000161	GO:0010591
31	11	GO Biological Process	Sphingolipid catabolic process	0.00094	GLA|GBA|NEU1|HEXB|LOC101112162|SGPL1|SMPDL3A|GM2A|ASAH1|	0.0000161	GO:0030149
52	14	GO Biological Process	Collagen metabolic process	0.00096	COL1A2|PEPD|COL1A1|MMP3|MMP9|CTSV|CTSL|MMP19|MRC2|CTSB	0.0000165	GO:0032963
14	8	GO Biological Process	Glycosphingolipid catabolic process	0.00096	GLA|GBA|NEU1|HEXB|LOC101112162|GM2A|NAGA|HEXA	0.0000167	GO:0046479
3935	299	GO Biological Process	Negative regulation of cellular process	0.00096	PABPC1|HSPA8|DRAP1|CAPZA2|FLT4|ELMSAN1|MAP3K20|STAT5A|RA	0.0000168	GO:0048523
189	29	GO Biological Process	Regulation of mononuclear cell proliferation	0.001	STAT5A|LOC443162|BTK|CD81|CORO1A|CD22|VSIG4|W5P5N5_SHEEP|	0.0000183	GO:0032944
914	89	GO Biological Process	Regulation of protein phosphorylation	0.0011	W5NPK5_SHEEP|FLT4|W5NRI1_SHEEP|RALBP1|HFE|BAG4|CHI3L1|TPX2	0.0000199	GO:0001932
130	23	GO Biological Process	Purine ribonucleoside triphosphate metabolic process	0.0011	W5NSP0_SHEEP|W5NTV6_SHEEP|NME1-NME2|SLC25A13|ALDOA|GPI|	0.0000186	GO:0009205
2439	198	GO Biological Process	Regulation of signal transduction	0.0011	W5NPK5_SHEEP|LIMS1|FLT4|MAP3K20|W5NRI1_SHEEP|HFE|ITGA6|BA	0.0000205	GO:0009966
221	32	GO Biological Process	Organic acid biosynthetic process	0.0011	GOT2|AKR1A1|MECR|TMED8|MTHFD1L|OXSM|BRCA1|SEPHS1|ALDH1	0.0000196	GO:0016053
103	20	GO Biological Process	Positive regulation of inflammatory response	0.0011	W5NPK5_SHEEP|W5NRI1_SHEEP|STAT5A|RIPK1|CD81|CTSC|LOC10111	0.00002	GO:0050729
734	75	GO Biological Process	Positive regulation of immune system process	0.0012	W5NPK5_SHEEP|W5NRI1_SHEEP|STAT5A|EIF2B3|PGF|RIPK1|BTK|IGHM	0.0000213	GO:0002684
39	12	GO Biological Process	Positive regulation of telomere maintenance via telomerase	0.0012	LOC101104501|TCP1|CCT3|PKIB|CCT6A|CCT7|PRKCQ|PNKP|CCT5|MA	0.0000219	GO:0032212
20	9	GO Biological Process	Ceramide catabolic process	0.0012	GLA|GBA|NEU1|HEXB|LOC101112162|GM2A|ASAH1|NAGA|HEXA	0.0000215	GO:0046514
61	15	GO Biological Process	Nucleotide phosphorylation	0.0012	NME1-NME2|ALDOA|GPI|TPI1|ALDOB|GAPDH|PGK2|ENO1|GALK1|W5	0.0000208	GO:0046939
266	36	GO Biological Process	Golgi vesicle transport	0.0012	BET1|ARL3|ARF5|TRAPPC5|SPTBN1|COG8|SNX8|COG5|LMAN1|TRAPP	0.0000227	GO:0048193
86	18	GO Biological Process	Regulation of phagocytosis	0.0012	W5NPK5_SHEEP|W5NRI1_SHEEP|FGR|SOD1-2|STAP1|SIRPA|ALOX15|P	0.0000216	GO:0050764
39	12	GO Biological Process	Protein localization to lysosome	0.0012	HSPA8|RTN4|CD81|GLMP|GGA3|LMBRD1|LAMP2|ZFYVE16|GNPTAB|S	0.0000219	GO:0061462
61	15	GO Biological Process	Protein localization to vacuole	0.0012	HSPA8|RTN4|CD81|GLMP|VPS37B|GGA3|LMBRD1|VPS13A|LAMP2|ZF	0.0000208	GO:0072665
54	14	GO Biological Process	Purine ribonucleoside diphosphate metabolic process	0.0013	ALDOA|GPI|TPI1|ALDOB|GAPDH|PGK2|NUDT16|ENO1|GALK1|W5PK0	0.0000237	GO:0009179
1161	107	GO Biological Process	Homeostatic process	0.0013	STAT5A|NPC2|HFE|PRDX1|CDH5|W5NUX2_SHEEP|ATP6V0A1|GAA|RTN	0.0000252	GO:0042592
879	86	GO Biological Process	Cellular lipid metabolic process	0.0013	W5NPK5_SHEEP|PTGR2|W5NRI1_SHEEP|PLCD1|PNPLA6|GLA|PIP4K2A|	0.0000235	GO:0044255
236	33	GO Biological Process	Adaptive immune response	0.0014	W5NPK5_SHEEP|W5NRI1_SHEEP|BTK|W5NYC7_SHEEP|CD81|FGR|IL18	0.0000275	GO:0002250
194	29	GO Biological Process	Regulation of epithelial cell migration	0.0015	FLT4|STAT5A|EVL|SASH1|MAP2K3|RTN4|GPI|MAPRE2|PRCP|MMP9|P	0.0000282	GO:0010632
64	15	GO Biological Process	Ribonucleoside diphosphate metabolic process	0.0017	DHODH|ALDOA|GPI|TPI1|ALDOB|GAPDH|PGK2|NUDT16|ENO1|GALK1	0.0000337	GO:0009185
351	43	GO Biological Process	Lipid localization	0.0017	STAT5A|NPC2|SPNS1|GOT2|PIP4K2A|ABCG2|RBP4|LCAT|GBA|OSBPL9	0.0000337	GO:0010876
261	35	GO Biological Process	Lipid catabolic process	0.0017	PLCD1|PNPLA6|GLA|SPP1|W5NZK6_SHEEP|GBA|NEU1|HEXB|LOC1011	0.0000348	GO:0016042
136	23	GO Biological Process	Hexose metabolic process	0.0017	FUCA2|RBP4|ALDOA|GPI|INPPL1|TPI1|FUCA1|ALDOB|GAPDH|PGK2|H	0.0000348	GO:0019318
469	53	GO Biological Process	Monocarboxylic acid metabolic process	0.0017	W5NPK5_SHEEP|PTGR2|W5NRI1_SHEEP|AKR1A1|MECR|MTHFD1L|W5N	0.0000328	GO:0032787
186	28	GO Biological Process	Regulation of lymphocyte proliferation	0.0017	STAT5A|LOC443162|BTK|CD81|CORO1A|CD22|VSIG4|W5P5N5_SHEEP|	0.0000344	GO:0050670
386	46	GO Biological Process	Regulation of lymphocyte activation	0.0017	STAT5A|HFE|LOC443162|BTK|IGHM|W5NYC7_SHEEP|CD81|FGR|CORO	0.0000338	GO:0051249
305	39	GO Biological Process	Regulation of apoptotic signaling pathway	0.0017	ITGA6|RIPK1|PSMD10|CTSC|SOD1-2|BRCA1|VHL|LMNA|WFS1|W5P9L	0.0000333	GO:2001233
218	31	GO Biological Process	Carboxylic acid biosynthetic process	0.0018	GOT2|AKR1A1|MECR|TMED8|MTHFD1L|OXSM|BRCA1|SEPHS1|ALDH1	0.0000358	GO:0046394
657	68	GO Biological Process	Membrane organization	0.0018	NPC2|BET1|PIP4K2A|RAC1|RTN4|SPTBN1|CORO1A|FOLR2|SOD1-2|PIC	0.0000357	GO:0061024

Supplementary Table 11: Comparative functional enrichment analysis of GMØ (monocyte-derived macrophages differentiated with GM-CSF) and MMØ (monocyte-derived macrophages differentiated with M-CSF) relative to Mo (monocytes at 3 hours). Functional enrichment analysis was performed using the StringApp (STRING database) within Cytoscape (version 3.10.3)[1], focusing on Gene Ontology (GO) Biological Process (BP) terms[2,3]. Red-highlighted cells indicate Gene Ontology Biological Process terms shared between GMØ and MMØ compared to Mo, whereas non-highlighted cells represent subtype-specific enrichment.

57	14	GO Biological Process	Nucleoside diphosphate phosphorylation	0.0019	NME1-NME2|ALDOA|GPI|TPI1|ALDOB|GAPDH|PGK2|ENO1|GALK1|W5	0.0000394	GO:0006165
22	9	GO Biological Process	Hexose catabolic process	0.0019	TPI1|ALDOB|GALM|ENO1|GALK1|PFKL|PFKP|PGK1|PKM	0.0000391	GO:0019320
1298	116	GO Biological Process	Regulation of protein modification process	0.0019	W5NPK5_SHEEP|FLT4|W5NRI1_SHEEP|RALBP1|HFE|BAG4|CHI3L1|TPX2	0.0000392	GO:0031399
100	19	GO Biological Process	ATP metabolic process	0.002	SLC25A13|ALDOA|GPI|TPI1|ALDOB|GAPDH|ATP1A2|PGK2|ATP1B1|EN	0.0000415	GO:0046034
3365	258	GO Biological Process	Response to chemical	0.0021	HSPA8|IFNGR1|FLT4|STAT5A|FBXO6|RAP1B|RALBP1|HFE|BAG4|CHI3L1	0.0000437	GO:0042221
309	39	GO Biological Process	Positive regulation of proteolysis	0.0021	NLRP2|PSMD10|GBA|CTSC|PDCD5|CARD9|PICALM|GSN|USP5|SIRT2|P	0.000043	GO:0045862
66	15	GO Biological Process	Glutamine family amino acid metabolic process	0.0022	PFAS|GLUD1|GOT2|TMED8|ALDH18A1|ALDH5A1|GCLC|ASS1|OAT|HAL	0.0000457	GO:0009064
83	17	GO Biological Process	Regulation of receptor-mediated endocytosis	0.0022	W5NPK5_SHEEP|W5NRI1_SHEEP|HFE|PICALM|W5P9L9_SHEEP|PLCG2|	0.000046	GO:0048259
211	30	GO Biological Process	Positive regulation of immune effector process	0.0023	W5NPK5_SHEEP|W5NRI1_SHEEP|CD81|FGR|GPI|CARD9|VAV1|PHB2|ST	0.0000476	GO:0002699
1222	110	GO Biological Process	Positive regulation of signal transduction	0.0023	W5NPK5_SHEEP|LIMS1|FLT4|MAP3K20|W5NRI1_SHEEP|HFE|BAG4|CHI	0.0000477	GO:0009967
17	8	GO Biological Process	Regulation of protein localization to chromosome, telomeric region	0.0023	TCP1|CCT3|CCT6A|CCT7|CCT5|MACROH2A1|W5QFZ3_SHEEP|CCT2	0.0000486	GO:1904814
12	7	GO Biological Process	Positive regulation of establishment of protein localization to telomer	0.0024	TCP1|CCT3|CCT6A|CCT7|CCT5|W5QFZ3_SHEEP|CCT2	0.0000522	GO:1904851
2747	216	GO Biological Process	Regulation of cell communication	0.0025	W5NPK5_SHEEP|LIMS1|FLT4|MAP3K20|W5NRI1_SHEEP|GLUD1|RAP1B	0.000053	GO:0010646
121	21	GO Biological Process	Cytosolic transport	0.0025	PIP4K2A|SPAG9|CORO1A|FLNA|SNX8|VPS26A|SNX6|LAMP1|WDR91|H	0.000054	GO:0016482
382	45	GO Biological Process	Regulation of cell-cell adhesion	0.0025	STAT5A|HFE|LOC443162|W5NYC7_SHEEP|CD81|CORO1A|FERMT3|VSIG	0.0000525	GO:0022407
2763	217	GO Biological Process	Regulation of signaling	0.0025	W5NPK5_SHEEP|LIMS1|FLT4|MAP3K20|W5NRI1_SHEEP|GLUD1|RAP1B	0.0000544	GO:0023051
1958	162	GO Biological Process	Regulation of developmental process	0.0025	W5NPK5_SHEEP|SETD3|LIMS1|W5NRI1_SHEEP|STAT5A|RALBP1|CHI3L1	0.0000533	GO:0050793
1049	97	GO Biological Process	Response to other organism	0.0025	W5NPK5_SHEEP|IFNGR1|GSDMD|W5NRI1_SHEEP|NPC2|FUCA2|CLEC4A	0.0000541	GO:0051707
257	34	GO Biological Process	Response to wounding	0.0026	TFPI2|TREML1|SOD1-2|FERMT3|FLNA|PRCP|TNC|CPB2|ALOX15|PLCG2	0.0000569	GO:0009611
1353	119	GO Biological Process	Positive regulation of cell communication	0.0026	W5NPK5_SHEEP|LIMS1|FLT4|MAP3K20|W5NRI1_SHEEP|GLUD1|HFE|BA	0.0000584	GO:0010647
204	29	GO Biological Process	Wound healing	0.0029	TFPI2|TREML1|FERMT3|FLNA|PRCP|CPB2|ALOX15|F5|CNN2|F9|DYSF|	0.0000633	GO:0042060
163	25	GO Biological Process	Ras protein signal transduction	0.003	RAP1B|COL1A2|RAC1|GDI1|RASGRP4|RRAS2|AIF1|CDC42|KPNB1|RASC	0.0000678	GO:0007265
8	6	GO Biological Process	Glycoside catabolic process	0.003	GUSB|FUCA2|GLA|FUCA1|W5PKU3_SHEEP|NAGA	0.0000677	GO:0016139
293	37	GO Biological Process	Regulation of T cell activation	0.003	STAT5A|HFE|LOC443162|W5NYC7_SHEEP|CD81|CORO1A|VSIG4|W5P5I	0.0000662	GO:0050863
423	48	GO Biological Process	Positive regulation of cell death	0.0031	MAP3K20|ITGA6|EIF5A-2|RIPK1|CTSC|PDCD5|FAF1|SOD1-2|LOC101114	0.0000693	GO:0010942
923	87	GO Biological Process	Phosphorylation	0.0031	FLT4|MAP3K20|RIPK1|BTK|PEAK1|PIP4K2A|VRK2|EFEMP1|MAP2K3|NN	0.0000716	GO:0016310
1166	105	GO Biological Process	Regulation of phosphate metabolic process	0.0031	W5NPK5_SHEEP|FLT4|W5NRI1_SHEEP|RALBP1|HFE|ITGA6|BAG4|CHI3L	0.0000701	GO:0019220
69	15	GO Biological Process	Positive regulation of telomere maintenance	0.0031	LOC101104501|TCP1|CCT3|PKIB|CCT6A|CCT7|RUVBL2|PRKCQ|PNKP|C	0.0000708	GO:0032206
12208	805	GO Biological Process	Biological regulation	0.0031	PABPC1|W5NPK5_SHEEP|DYNLT3|HSPA8|DRAP1|CAPZA2|EIF3I|SETD3|	0.0000719	GO:0065007
1362	119	GO Biological Process	Positive regulation of signaling	0.0032	W5NPK5_SHEEP|LIMS1|FLT4|MAP3K20|W5NRI1_SHEEP|GLUD1|HFE|BA	0.0000755	GO:0023056
61	14	GO Biological Process	Regulation of interleukin-8 production	0.0032	CHI3L1|RIPK1|CD74|PYCARD|PARK7|PRKD2|TLR7|ARRB1|ANXA1|TLR2|	0.0000742	GO:0032677
78	16	GO Biological Process	Positive regulation of endocytosis	0.0032	W5NPK5_SHEEP|W5NRI1_SHEEP|HFE|W5P9L9_SHEEP|STAP1|LRP1|CDC	0.0000751	GO:0045807
1155	104	GO Biological Process	Lipid metabolic process	0.0033	W5NPK5_SHEEP|PTGR2|W5NRI1_SHEEP|PLCD1|NPC2|PNPLA6|GLA|AK	0.0000762	GO:0006629
13	7	GO Biological Process	NADPH regeneration	0.0033	PGD|TALDO1|ALDH1L1|H6PD|TKT|SHPK|RPIA	0.0000763	GO:0006740
1046	96	GO Biological Process	Regulation of phosphorylation	0.0033	W5NPK5_SHEEP|FLT4|W5NRI1_SHEEP|RALBP1|HFE|ITGA6|BAG4|CHI3L	0.0000762	GO:0042325
13	7	GO Biological Process	Glycolytic process through glucose-6-phosphate	0.0033	TPI1|ENO1|GALK1|PFKL|PFKP|PGK1|PKM	0.0000763	GO:0061620
125	21	GO Biological Process	Positive regulation of angiogenesis	0.0034	W5NPK5_SHEEP|W5NRI1_SHEEP|CHI3L1|PGF|CDH5|SASH1|BRCA1|CYP	0.0000815	GO:0045766
145	23	GO Biological Process	Vacuole organization	0.0035	NAGLU|GAA|CORO1A|LAMTOR1|GBA|WDR45B|VPS16|ACP2|TRAPPC8	0.0000833	GO:0007033
3053	235	GO Biological Process	Positive regulation of macromolecule metabolic process	0.0035	PABPC1|W5NPK5_SHEEP|HSPA8|DRAP1|SETD3|GSDMD|FLT4|W5NRI1_	0.0000835	GO:0010604
439	49	GO Biological Process	Organelle localization	0.0035	ARMCX3|STXBP2|SPAG9|RAB11B|LAMTOR1|W5P2V0_SHEEP|PICALM|F	0.0000846	GO:0051640
19	8	GO Biological Process	NAD metabolic process	0.0037	TPI1|ALDOB|ENO1|NADK2|PFKL|PFKP|PGK1|PKM	0.0000901	GO:0019674
146	23	GO Biological Process	Regulation of T cell proliferation	0.0037	STAT5A|LOC443162|CD81|CORO1A|VSIG4|W5P5N5_SHEEP|AIF1|PYCAF	0.0000913	GO:0042129
19	8	GO Biological Process	Clathrin coat assembly	0.0037	AP2A2|PICALM|FCHO2|CLTA|HIP1|CLTC|AP2A2-2|TLN2	0.0000901	GO:0048268
39	11	GO Biological Process	De novo post-translational protein folding	0.0037	HSPA8|CD74|TOR1B|HSPA1A-2|HSPH1|W5Q1T4_SHEEP|SDF2L1|HSPA1	0.0000927	GO:0051084
177	26	GO Biological Process	Regulation of cysteine-type endopeptidase activity	0.0037	NLRP2|PDCD5|CARD9|GSN|PSMB9|MMP9|PLAUR|PYCARD|BAK1|PARK	0.0000924	GO:2000116
516	55	GO Biological Process	Cell activation	0.0039	STAT5A|TREML1|BTK|PRDX1|STXBP2|CLEC4A|CD81|PSMB10|CORO1A|	0.0000978	GO:0001775
80	16	GO Biological Process	Integrin-mediated signaling pathway	0.0039	ITGA6|NME1-NME2|FGR|FERMT3|VAV1|CDC42|ITGA2|ITGAM|PTPN11	0.0000973	GO:0007229
734	72	GO Biological Process	Response to abiotic stimulus	0.004	MAP3K20|CHI3L1|PGF|DNAJC3|ATP6V0D1|HMOX2|RAB11B|HSP90AA1	0.0001	GO:0009628
178	26	GO Biological Process	Positive regulation of protein catabolic process	0.004	PSMD10|CD81|GBA|W5P2V0_SHEEP|MSN|USP5|SIRT2|PSMC3|LRP1|H	0.0001	GO:0045732
63	14	GO Biological Process	Organic hydroxy compound catabolic process	0.004	MAOA|TPI1|HSD17B6|NUDT3|BPNT1|INPP5K|ALDH16A1|SYNJ1|W5PY	0.0001	GO:1901616
158	24	GO Biological Process	Positive regulation of protein-containing complex assembly	0.0042	BAG4|EVL|CDH5|FSCN1|MSN|MMP3|ALOX15|HSPA1A-2|PYCARD|PLC	0.00011	GO:0031334
372	43	GO Biological Process	Positive regulation of cytokine production	0.0043	W5NPK5_SHEEP|GSDMD|FLT4|W5NRI1_SHEEP|STAT5A|CHI3L1|RIPK1|	0.00011	GO:0001819
81	16	GO Biological Process	Nucleoside diphosphate metabolic process	0.0043	NME1-NME2|DHODH|ALDOA|GPI|TPI1|ALDOB|GAPDH|PGK2|NUDT16|	0.00011	GO:0009132
179	26	GO Biological Process	Cellular lipid catabolic process	0.0043	GLA|W5NZK6_SHEEP|GBA|NEU1|HEXB|LOC101112162|SIRT2|SGPL1|A	0.00011	GO:0044242
190	27	GO Biological Process	Biological process involved in symbiotic interaction	0.0043	HSPA8|CD81|LOC101114535|CARD9|TCP1|IGF2R|CSF1R|DPP4|PSMC3|	0.00011	GO:0044403
14	7	GO Biological Process	Transcytosis	0.0043	PICALM|LRP1|CLTC|LRPAP1|VPS35|RAB5A|USO1	0.00011	GO:0045056

Supplementary Table 11: Comparative functional enrichment analysis of GMØ (monocyte-derived macrophages differentiated with GM-CSF) and MMØ (monocyte-derived macrophages differentiated with M-CSF) relative to Mo (monocytes at 3 hours). Functional enrichment analysis was performed using the StringApp (STRING database) within Cytoscape (version 3.10.3)[1], focusing on Gene Ontology (GO) Biological Process (BP) terms[2,3]. Red-highlighted cells indicate Gene Ontology Biological Process terms shared between GMØ and MMØ compared to Mo, whereas non-highlighted cells represent subtype-specific enrichment.

<table>
<tr><td>723</td><td>71</td><td>GO Biological Process</td><td>Response to nitrogen compound</td><td>0.0043</td><td>STAT5A|FBXO6|RAP1B|COL1A2|RGS10|IPO5|SEL1L|CD81|LAMTOR1|FC</td><td>0.00011</td><td>GO:1901698</td></tr>
<tr><td>64</td><td>14</td><td>GO Biological Process</td><td>Response to heat</td><td>0.0044</td><td>HSP90AA1|SOD1-2|HIKESHI|CLPB|GCLC|HSP90AB1|VCP|YWHAE|HSPB1</td><td>0.00012</td><td>GO:0009408</td></tr>
<tr><td>256</td><td>33</td><td>GO Biological Process</td><td>Regulation of inflammatory response</td><td>0.0044</td><td>W5NPK5_SHEEP|W5NRI1_SHEEP|STAT5A|RIPK1|CDH5|NLRP2|CD81|CT</td><td>0.00011</td><td>GO:0050727</td></tr>
<tr><td>56</td><td>13</td><td>GO Biological Process</td><td>Regulation of interleukin-2 production</td><td>0.0046</td><td>STAT5A|SPTBN1|W5P2V0_SHEEP|CARD9|VSIG4|PLCG2|W5PJN7_SHEEP</td><td>0.00012</td><td>GO:0032663</td></tr>
<tr><td>202</td><td>28</td><td>GO Biological Process</td><td>Endosomal transport</td><td>0.0047</td><td>SPAG9|RAB11B|LAMTOR1|PICALM|VPS16|SNX8|VPS26A|SNX6|SNX18|</td><td>0.00012</td><td>GO:0016197</td></tr>
<tr><td>82</td><td>16</td><td>GO Biological Process</td><td>Cellular oxidant detoxification</td><td>0.0048</td><td>PRDX1|NQO1|CLIC2|SOD1-2|GPX7|LOC101116273|GLRX|PARK7|GSR|P</td><td>0.00013</td><td>GO:0098869</td></tr>
<tr><td>130</td><td>21</td><td>GO Biological Process</td><td>Regulation of cell shape</td><td>0.005</td><td>RAC1|FGR|CORO1A|ALDOA|LOC101114535|W5P2V0_SHEEP|SEPTIN7|M</td><td>0.00013</td><td>GO:0008360</td></tr>
<tr><td>690</td><td>68</td><td>GO Biological Process</td><td>Negative regulation of programmed cell death</td><td>0.0051</td><td>FLT4|STAT5A|ITGA6|RIPK1|CDH5|DNAJC3|PSMD10|NME1-NME2|ATAD</td><td>0.00014</td><td>GO:0043069</td></tr>
<tr><td>74</td><td>15</td><td>GO Biological Process</td><td>Retrograde transport, endosome to Golgi</td><td>0.0052</td><td>SPAG9|SNX8|VPS26A|SNX6|HEATR5B|SNX3|TBC1D10C|CLTC|VPS35|YK</td><td>0.00014</td><td>GO:0042147</td></tr>
<tr><td>1300</td><td>113</td><td>GO Biological Process</td><td>Protein-containing complex organization</td><td>0.0052</td><td>HSPA8|EIF3I|GSDMD|W5NRD9_SHEEP|EIF3A|SPAG1|EVL|H3-4|COL1A2</td><td>0.00014</td><td>GO:0043933</td></tr>
<tr><td>34</td><td>10</td><td>GO Biological Process</td><td>Chaperone cofactor-dependent protein refolding</td><td>0.0054</td><td>HSPA8|CD74|TOR1B|HSPA1A-2|HSPH1|W5Q1T4_SHEEP|SDF2L1|HSPA1</td><td>0.00014</td><td>GO:0051085</td></tr>
<tr><td>500</td><td>53</td><td>GO Biological Process</td><td>Cellular homeostasis</td><td>0.0055</td><td>HFE|PRDX1|CDH5|W5NUX2_SHEEP|ATP6V0A1|GAA|RTN4|ATP6V0D1|E</td><td>0.00015</td><td>GO:0019725</td></tr>
<tr><td>1081</td><td>97</td><td>GO Biological Process</td><td>Regulation of multicellular organismal development</td><td>0.0055</td><td>W5NPK5_SHEEP|W5NRI1_SHEEP|STAT5A|CHI3L1|PGF|RIPK1|CDH5|EFE</td><td>0.00015</td><td>GO:2000026</td></tr>
<tr><td>15</td><td>7</td><td>GO Biological Process</td><td>Glutamine family amino acid biosynthetic process</td><td>0.0056</td><td>TMED8|ALDH18A1|ASS1|OAT|PYCR3|PYCR2|GLUL</td><td>0.00015</td><td>GO:0009084</td></tr>
<tr><td>906</td><td>84</td><td>GO Biological Process</td><td>Macromolecule catabolic process</td><td>0.0057</td><td>PABPC1|HSPA8|FBXO6|PSMA4|RIPK1|SGSH|W5NUX2_SHEEP|DNAJC3|</td><td>0.00016</td><td>GO:0009057</td></tr>
<tr><td>3328</td><td>251</td><td>GO Biological Process</td><td>Positive regulation of metabolic process</td><td>0.0057</td><td>PABPC1|W5NPK5_SHEEP|HSPA8|DRAP1|SETD3|GSDMD|FLT4|W5NRI1_</td><td>0.00016</td><td>GO:0009893</td></tr>
<tr><td>21</td><td>8</td><td>GO Biological Process</td><td>Glycoside metabolic process</td><td>0.0057</td><td>GUSB|FUCA2|GLA|AKR1A1|FUCA1|W5PKU3_SHEEP|LOC101110434|NA</td><td>0.00016</td><td>GO:0016137</td></tr>
<tr><td>58</td><td>13</td><td>GO Biological Process</td><td>Glutathione metabolic process</td><td>0.0059</td><td>CLIC2|SOD1-2|CLIC1|GCLC|LOC101116273|PARK7|GSR|CLIC5|W5PUH4</td><td>0.00016</td><td>GO:0006749</td></tr>
<tr><td>355</td><td>41</td><td>GO Biological Process</td><td>Response to growth factor</td><td>0.0059</td><td>FLT4|HFE|BAG4|COL1A2|PGF|RIPK1|CDH5|MAP2K3|CORO1A|PDCD5|</td><td>0.00016</td><td>GO:0070848</td></tr>
<tr><td>320</td><td>38</td><td>GO Biological Process</td><td>Response to inorganic substance</td><td>0.006</td><td>HFE|RIPK1|PRDX1|LOC101104501|NQO1|SLC25A13|SOD1-2|TXN|MMP</td><td>0.00017</td><td>GO:0010035</td></tr>
<tr><td>761</td><td>73</td><td>GO Biological Process</td><td>Negative regulation of cell death</td><td>0.006</td><td>FLT4|STAT5A|ITGA6|RIPK1|CDH5|DNAJC3|PSMD10|NME1-NME2|ATAD</td><td>0.00017</td><td>GO:0060548</td></tr>
<tr><td>153</td><td>23</td><td>GO Biological Process</td><td>Positive regulation of peptidase activity</td><td>0.0061</td><td>NLRP2|CTSC|PDCD5|CARD9|PICALM|GSN|GRN|NCSTN|PYCARD|BAK1|</td><td>0.00017</td><td>GO:0010952</td></tr>
<tr><td>35</td><td>10</td><td>GO Biological Process</td><td>Negative regulation of smooth muscle cell proliferation</td><td>0.0063</td><td>AIF1|SF1|PDCD4|RNASE4|PRKG1|HMOX1|IGFBP5|NDRG2|APOD|CDKN</td><td>0.00018</td><td>GO:0048662</td></tr>
<tr><td>67</td><td>14</td><td>GO Biological Process</td><td>Organelle localization by membrane tethering</td><td>0.0063</td><td>STXBP2|STX4|ESYT2|ESYT1|RAB3A|STX7|STX2|USO1|YKT6|SNAP29|STX</td><td>0.00018</td><td>GO:0140056</td></tr>
<tr><td>59</td><td>13</td><td>GO Biological Process</td><td>Cellular aldehyde metabolic process</td><td>0.0067</td><td>AKR1A1|TPI1|ALDOB|ESD|CYP1B1|PARK7|PDXK|ALDH9A1|W5PY08_SH</td><td>0.00019</td><td>GO:0006081</td></tr>
<tr><td>1103</td><td>98</td><td>GO Biological Process</td><td>Response to biotic stimulus</td><td>0.0068</td><td>W5NPK5_SHEEP|IFNGR1|GSDMD|W5NRI1_SHEEP|NPC2|FUCA2|CLEC4A</td><td>0.00019</td><td>GO:0009607</td></tr>
<tr><td>114</td><td>19</td><td>GO Biological Process</td><td>Positive regulation of leukocyte proliferation</td><td>0.0068</td><td>STAT5A|CD81|CORO1A|CSF1R|CD74|AIF1|PYCARD|DHPS|ANXA1|PRKC</td><td>0.00019</td><td>GO:0070665</td></tr>
<tr><td>95</td><td>17</td><td>GO Biological Process</td><td>Glucose metabolic process</td><td>0.0069</td><td>RBP4|GPI|INPPL1|TPI1|GAPDH|PGK2|H6PD|GALM|FABP5|ENO1|PGM</td><td>0.0002</td><td>GO:0006006</td></tr>
<tr><td>2176</td><td>173</td><td>GO Biological Process</td><td>Cellular component assembly</td><td>0.007</td><td>EIF3I|GSDMD|W5NRD9_SHEEP|EIF3A|SPAG1|BAG4|TPX2|EVL|H3-4|CC</td><td>0.0002</td><td>GO:0022607</td></tr>
<tr><td>300</td><td>36</td><td>GO Biological Process</td><td>Regulation of immune effector process</td><td>0.0071</td><td>W5NPK5_SHEEP|W5NRI1_SHEEP|HFE|CD81|FGR|GPI|CARD9|VAV1|PHI</td><td>0.00021</td><td>GO:0002697</td></tr>
<tr><td>77</td><td>15</td><td>GO Biological Process</td><td>Positive regulation of T cell proliferation</td><td>0.0071</td><td>STAT5A|CD81|CORO1A|AIF1|PYCARD|DHPS|ANXA1|PRKCQ|PPP3CA|SA</td><td>0.0002</td><td>GO:0042102</td></tr>
<tr><td>6</td><td>5</td><td>GO Biological Process</td><td>Amyloid-beta clearance by transcytosis</td><td>0.0071</td><td>PICALM|LRP1|CLTC|LRPAP1|RAB5A</td><td>0.0002</td><td>GO:0150093</td></tr>
<tr><td>6</td><td>5</td><td>GO Biological Process</td><td>Negative regulation of cysteine-type endopeptidase activity involved</td><td>0.0071</td><td>MMP9|PLAUR|PARK7|PIH1D1|CAAP1</td><td>0.0002</td><td>GO:2001268</td></tr>
<tr><td>901</td><td>83</td><td>GO Biological Process</td><td>Cell motility</td><td>0.0072</td><td>ITGA6|PEAK1|CDH5|RAC1|RTN4|FSCN1|CORO1A|LGALS8|GBA|TOP2B|</td><td>0.00021</td><td>GO:0048870</td></tr>
<tr><td>5846</td><td>412</td><td>GO Biological Process</td><td>Cellular response to stimulus</td><td>0.0072</td><td>W5NPK5_SHEEP|HSPA8|IFNGR1|GSDMD|FLT4|MAP3K20|W5NRI1_SHEE</td><td>0.00021</td><td>GO:0051716</td></tr>
<tr><td>60</td><td>13</td><td>GO Biological Process</td><td>Alpha-amino acid biosynthetic process</td><td>0.0075</td><td>GOT2|TMED8|SEPHS1|ALDH18A1|PCBD1|ASS1|OAT|PAH|PYCR3|BHMT</td><td>0.00022</td><td>GO:1901607</td></tr>
<tr><td>69</td><td>14</td><td>GO Biological Process</td><td>Cellular amino acid biosynthetic process</td><td>0.0078</td><td>GOT2|TMED8|SEPHS1|ALDH18A1|PCBD1|ASS1|OAT|ALDH1A1|PAH|PY</td><td>0.00023</td><td>GO:0008652</td></tr>
<tr><td>931</td><td>85</td><td>GO Biological Process</td><td>Response to endogenous stimulus</td><td>0.0078</td><td>STAT5A|RAP1B|HFE|BAG4|COL1A2|CDH5|IPO5|CD81|CORO1A|LAMTO</td><td>0.00023</td><td>GO:0009719</td></tr>
<tr><td>136</td><td>21</td><td>GO Biological Process</td><td>Maintenance of location</td><td>0.0078</td><td>STAT5A|GAA|PSMD10|GBA|FLNA|GSN|TWF2|TXN|SCIN|GM2A|PARK7</td><td>0.00023</td><td>GO:0051235</td></tr>
<tr><td>87</td><td>16</td><td>GO Biological Process</td><td>Biological process involved in interaction with symbiont</td><td>0.0078</td><td>HSPA8|LOC101114535|CARD9|IGF2R|CSF1R|PSMC3|GAPDH|CDC42|SA</td><td>0.00023</td><td>GO:0051702</td></tr>
<tr><td>178</td><td>25</td><td>GO Biological Process</td><td>Alpha-amino acid metabolic process</td><td>0.0078</td><td>PFAS|GLUD1|ALDH6A1|GOT2|TMED8|SEPHS1|ALDH18A1|PCBD1|ALDH</td><td>0.00023</td><td>GO:1901605</td></tr>
<tr><td>106</td><td>18</td><td>GO Biological Process</td><td>Positive regulation of chromosome organization</td><td>0.0078</td><td>LOC101104501|TCP1|CCT3|PKIB|CCT6A|RAD21|CCT7|RUVBL2|PRKCQ|</td><td>0.00023</td><td>GO:2001252</td></tr>
<tr><td>2718</td><td>209</td><td>GO Biological Process</td><td>Positive regulation of nitrogen compound metabolic process</td><td>0.0079</td><td>PABPC1|W5NPK5_SHEEP|HSPA8|DRAP1|SETD3|FLT4|W5NRI1_SHEEP|S</td><td>0.00024</td><td>GO:0051173</td></tr>
<tr><td>11</td><td>6</td><td>GO Biological Process</td><td>Pentose-phosphate shunt</td><td>0.0081</td><td>PGD|TALDO1|H6PD|TKT|SHPK|RPIA</td><td>0.00024</td><td>GO:0006098</td></tr>
<tr><td>11</td><td>6</td><td>GO Biological Process</td><td>Actin filament depolymerization</td><td>0.0081</td><td>GSN|TWF2|WDR1|MICAL1|CFL1|TWF1</td><td>0.00024</td><td>GO:0030042</td></tr>
<tr><td>11</td><td>6</td><td>GO Biological Process</td><td>Regulation of cysteine-type endopeptidase activity involved in apopto</td><td>0.0081</td><td>GSN|MMP9|PLAUR|PARK7|PIH1D1|CAAP1</td><td>0.00024</td><td>GO:2001267</td></tr>
<tr><td>223</td><td>29</td><td>GO Biological Process</td><td>Regulation of angiogenesis</td><td>0.0083</td><td>W5NPK5_SHEEP|W5NRI1_SHEEP|CHI3L1|PGF|CDH5|SASH1|STAB1|W5I</td><td>0.00025</td><td>GO:0045765</td></tr>
<tr><td>70</td><td>14</td><td>GO Biological Process</td><td>Modulation by host of symbiont process</td><td>0.0086</td><td>HSPA8|LOC101114535|CARD9|IGF2R|CSF1R|PSMC3|CDC42|SAP30BP|I</td><td>0.00026</td><td>GO:0051851</td></tr>
<tr><td>45</td><td>11</td><td>GO Biological Process</td><td>Alcohol catabolic process</td><td>0.0089</td><td>TPI1|NUDT3|BPNT1|INPP5K|ALDH16A1|SYNJ1|W5PY08_SHEEP|OCRL|II</td><td>0.00027</td><td>GO:0046164</td></tr>
<tr><td>247</td><td>31</td><td>GO Biological Process</td><td>Response to oxidative stress</td><td>0.009</td><td>RIPK1|PRDX1|VRK2|HMOX2|NQO1|SOD1-2|GPX7|SIRT2|GCLC|MMP9|</td><td>0.00027</td><td>GO:0006979</td></tr>
<tr><td>643</td><td>63</td><td>GO Biological Process</td><td>Response to organonitrogen compound</td><td>0.009</td><td>STAT5A|FBXO6|RAP1B|COL1A2|IPO5|SEL1L|CD81|LAMTOR1|FOLR2|FA</td><td>0.00027</td><td>GO:0010243</td></tr>
<tr><td>191</td><td>26</td><td>GO Biological Process</td><td>Gliogenesis</td><td>0.009</td><td>EIF2B3|RTN4|GBA|SOD1-2|LOC101114535|NDRG1|SIRT2|CSF1R|RRAS2</td><td>0.00027</td><td>GO:0042063</td></tr>
<tr><td>669</td><td>65</td><td>GO Biological Process</td><td>Negative regulation of apoptotic process</td><td>0.009</td><td>FLT4|STAT5A|ITGA6|RIPK1|CDH5|DNAJC3|PSMD10|NME1-NME2|ATAD</td><td>0.00027</td><td>GO:0043066</td></tr>
<tr><td>62</td><td>13</td><td>GO Biological Process</td><td>Actin cytoskeleton reorganization</td><td>0.0094</td><td>W5P2V0_SHEEP|FLNA|GSN|GRB2|GAB1|ANXA1|W5PVN6_SHEEP|PLEC|</td><td>0.00029</td><td>GO:0031532</td></tr>
<tr><td>149</td><td>22</td><td>GO Biological Process</td><td>Cellular carbohydrate metabolic process</td><td>0.0094</td><td>GUSB|GAA|PGD|TPI1|PYGB|GALK1|W5PKU3_SHEEP|PPP1CA|PFKL|BPN</td><td>0.00029</td><td>GO:0044262</td></tr>
<tr><td>62</td><td>13</td><td>GO Biological Process</td><td>Positive regulation of DNA biosynthetic process</td><td>0.0094</td><td>LOC101104501|TCP1|CCT3|PKIB|CCT6A|PRKD2|CCT7|PRKCQ|PNKP|CC</td><td>0.00029</td><td>GO:2000573</td></tr>
</table>

Supplementary Table 11: Comparative functional enrichment analysis of GMØ (monocyte-derived macrophages differentiated with GM-CSF) and MMØ (monocyte-derived macrophages differentiated with M-CSF) relative to Mo (monocytes at 3 hours). Functional enrichment analysis was performed using the StringApp (STRING database) within Cytoscape (version 3.10.3)[1], focusing on Gene Ontology (GO) Biological Process (BP) terms[2,3]. Red-highlighted cells indicate Gene Ontology Biological Process terms shared between GMØ and MMØ compared to Mo, whereas non-highlighted cells represent subtype-specific enrichment.

<table>
<tr><td>71</td><td>14</td><td>GO Biological Process</td><td>Glycosyl compound metabolic process</td><td>0.0095</td><td>W5NRS0_SHEEP|GUSB|FUCA2|GLA|AKR1A1|FUCA1|ADK|W5PKU3_SHE</td><td>0.0003</td><td>GO:1901657</td></tr>
<tr><td>237</td><td>30</td><td>GO Biological Process</td><td>Negative regulation of peptidase activity</td><td>0.0096</td><td>W5NPK5_SHEEP|W5NRI1_SHEEP|TFPI2|A2M|ITIH1|RARRES1|PICALM|S</td><td>0.0003</td><td>GO:0010466</td></tr>
<tr><td>160</td><td>23</td><td>GO Biological Process</td><td>Regulation of cysteine-type endopeptidase activity involved in apopto</td><td>0.0097</td><td>NLRP2|PDCD5|CARD9|GSN|MMP9|PLAUR|PYCARD|BAK1|PARK7|MICA</td><td>0.0003</td><td>GO:0043281</td></tr>
<tr><td>129</td><td>20</td><td>GO Biological Process</td><td>Membrane fusion</td><td>0.0098</td><td>BET1|PIP4K2A|CORO1A|FOLR2|GCA|STX18|STX4|CTSL|DYSF|STX7|STX</td><td>0.00031</td><td>GO:0061025</td></tr>
<tr><td>204</td><td>27</td><td>GO Biological Process</td><td>Positive regulation of leukocyte cell-cell adhesion</td><td>0.01</td><td>STAT5A|W5NYC7_SHEEP|CD81|CORO1A|DPP4|CD74|SIRPA|AIF1|PYCA</td><td>0.00031</td><td>GO:1903039</td></tr>
<tr><td>46</td><td>11</td><td>GO Biological Process</td><td>Positive regulation of interleukin-8 production</td><td>0.0101</td><td>CHI3L1|RIPK1|CD74|PYCARD|PARK7|PRKD2|TLR7|TLR2|RAB1A|CD58|I</td><td>0.00032</td><td>GO:0032757</td></tr>
<tr><td>700</td><td>67</td><td>GO Biological Process</td><td>Protein catabolic process</td><td>0.0102</td><td>HSPA8|FBXO6|PSMA4|RIPK1|SGSH|DNAJC3|RPS27A|SEL1L|PSMB10|G</td><td>0.00032</td><td>GO:0030163</td></tr>
<tr><td>24</td><td>8</td><td>GO Biological Process</td><td>NADH metabolic process</td><td>0.0104</td><td>TPI1|ALDOB|ENO1|PFKL|VCP|PFKP|PGK1|PKM</td><td>0.00033</td><td>GO:0006734</td></tr>
<tr><td>31</td><td>9</td><td>GO Biological Process</td><td>Antigen processing and presentation of exogenous peptide antigen</td><td>0.0105</td><td>CLEC4A|W5NYC7_SHEEP|CD74|IFI30|W5Q2C4_SHEEP|W5Q2V1_SHEEP|</td><td>0.00033</td><td>GO:0002478</td></tr>
<tr><td>7</td><td>5</td><td>GO Biological Process</td><td>Ganglioside catabolic process</td><td>0.0105</td><td>NEU1|HEXB|LOC101112162|GM2A|HEXA</td><td>0.00033</td><td>GO:0006689</td></tr>
<tr><td>381</td><td>42</td><td>GO Biological Process</td><td>Positive regulation of apoptotic process</td><td>0.0105</td><td>MAP3K20|ITGA6|EIF5A-2|RIPK1|CTSC|PDCD5|SOD1-2|LOC101114535|</td><td>0.00033</td><td>GO:0043065</td></tr>
<tr><td>183</td><td>25</td><td>GO Biological Process</td><td>Negative regulation of apoptotic signaling pathway</td><td>0.0106</td><td>ITGA6|RIPK1|PSMD10|BRCA1|LMNA|WFS1|CD74|TPT1|SLC25A4|MMP</td><td>0.00034</td><td>GO:2001234</td></tr>
<tr><td>321</td><td>37</td><td>GO Biological Process</td><td>Lipid transport</td><td>0.0107</td><td>NPC2|SPNS1|GOT2|PIP4K2A|ABCG2|RBP4|LCAT|OSBPL9|PITPNM2|PSA</td><td>0.00034</td><td>GO:0006869</td></tr>
<tr><td>456</td><td>48</td><td>GO Biological Process</td><td>Leukocyte activation</td><td>0.0107</td><td>STAT5A|BTK|PRDX1|STXBP2|CLEC4A|CD81|PSMB10|CORO1A|GBA|LOC</td><td>0.00034</td><td>GO:0045321</td></tr>
<tr><td>12</td><td>6</td><td>GO Biological Process</td><td>Canonical glycolysis</td><td>0.0107</td><td>TPI1|ENO1|PFKL|PFKP|PGK1|PKM</td><td>0.00034</td><td>GO:0061621</td></tr>
<tr><td>39</td><td>10</td><td>GO Biological Process</td><td>Protein targeting to vacuole</td><td>0.0112</td><td>HSPA8|VPS37B|GGA3|VPS13A|LAMP2|ZFYVE16|GNPTAB|SCARB2|VPS4</td><td>0.00037</td><td>GO:0006623</td></tr>
<tr><td>18</td><td>7</td><td>GO Biological Process</td><td>Vascular endothelial growth factor signaling pathway</td><td>0.0112</td><td>FLT4|PGF|PDGFRB|PRKD2|GAB1|HSPB1|NRP2</td><td>0.00037</td><td>GO:0038084</td></tr>
<tr><td>825</td><td>76</td><td>GO Biological Process</td><td>Positive regulation of protein modification process</td><td>0.0116</td><td>W5NPK5_SHEEP|FLT4|W5NRI1_SHEEP|RALBP1|HFE|BAG4|CHI3L1|TPX2</td><td>0.00038</td><td>GO:0031401</td></tr>
<tr><td>32</td><td>9</td><td>GO Biological Process</td><td>Regulation of vascular endothelial growth factor production</td><td>0.0123</td><td>W5NPK5_SHEEP|FLT4|W5NRI1_SHEEP|BRCA1|CYP1B1|C5AR1|SARS1|N</td><td>0.00041</td><td>GO:0010574</td></tr>
<tr><td>102</td><td>17</td><td>GO Biological Process</td><td>Positive regulation of mononuclear cell proliferation</td><td>0.0123</td><td>STAT5A|CD81|CORO1A|CD74|AIF1|PYCARD|DHPS|ANXA1|PRKCQ|PPP</td><td>0.00041</td><td>GO:0032946</td></tr>
<tr><td>277</td><td>33</td><td>GO Biological Process</td><td>Sulfur compound metabolic process</td><td>0.0124</td><td>SULT1C4|PMVK|CLIC2|OXSM|SOD1-2|AHCYL2|CLIC1|ACOT8|GCLC|LOC</td><td>0.00042</td><td>GO:0006790</td></tr>
<tr><td>164</td><td>23</td><td>GO Biological Process</td><td>Regulation of cell-substrate adhesion</td><td>0.0124</td><td>LIMS1|PLEKHA2|PEAK1|COL1A1|FLNA|ACTN4|CDC42|ALOX15|VCL|RSU</td><td>0.00041</td><td>GO:0010810</td></tr>
<tr><td>164</td><td>23</td><td>GO Biological Process</td><td>Protein processing</td><td>0.0124</td><td>CPM|CTSC|GLG1|CASP6|TLL2|CTSV|CTSL|NCSTN|PYCARD|DDI2|MSR1|</td><td>0.00041</td><td>GO:0016485</td></tr>
<tr><td>56</td><td>12</td><td>GO Biological Process</td><td>Receptor internalization</td><td>0.0124</td><td>CD81|W5P2V0_SHEEP|PICALM|GRB2|ARRB1|LMBRD1|ITGB2|CLTC|RA</td><td>0.00041</td><td>GO:0031623</td></tr>
<tr><td>231</td><td>29</td><td>GO Biological Process</td><td>Negative regulation of endopeptidase activity</td><td>0.0126</td><td>W5NPK5_SHEEP|W5NRI1_SHEEP|TFPI2|A2M|ITIH1|RARRES1|PICALM|S</td><td>0.00042</td><td>GO:0010951</td></tr>
<tr><td>40</td><td>10</td><td>GO Biological Process</td><td>Antigen processing and presentation of exogenous antigen</td><td>0.013</td><td>CLEC4A|W5NYC7_SHEEP|CD74|IFI30|W5Q2C4_SHEEP|W5Q2V1_SHEEP|</td><td>0.00044</td><td>GO:0019884</td></tr>
<tr><td>84</td><td>15</td><td>GO Biological Process</td><td>Positive regulation of endothelial cell migration</td><td>0.0137</td><td>FLT4|STAT5A|SASH1|MAP2K3|GPI|PRKD2|ITGB3|ANXA1|LGMN|RRAS|</td><td>0.00046</td><td>GO:0010595</td></tr>
<tr><td>1289</td><td>109</td><td>GO Biological Process</td><td>Proteolysis</td><td>0.0138</td><td>CPM|FBXO6|PSMA4|COPS3|COPS4|DNAJC3|RPS27A|SEL1L|SGF29|PSM</td><td>0.00047</td><td>GO:0006508</td></tr>
<tr><td>256</td><td>31</td><td>GO Biological Process</td><td>Cellular amino acid metabolic process</td><td>0.014</td><td>PFAS|GLUD1|ALDH6A1|GOT2|TMED8|NARS1|SEPHS1|ALDH18A1|PCBD</td><td>0.00048</td><td>GO:0006520</td></tr>
<tr><td>19</td><td>7</td><td>GO Biological Process</td><td>Vacuolar acidification</td><td>0.014</td><td>ATP6V0A1|ATP6V0D1|GRN|ATP6V1B2|TCIRG1|PPT1|DMXL2</td><td>0.00048</td><td>GO:0007035</td></tr>
<tr><td>1851</td><td>148</td><td>GO Biological Process</td><td>Response to external stimulus</td><td>0.014</td><td>W5NPK5_SHEEP|IFNGR1|GSDMD|W5NRI1_SHEEP|NPC2|HFE|CHI3L1|P</td><td>0.00048</td><td>GO:0009605</td></tr>
<tr><td>134</td><td>20</td><td>GO Biological Process</td><td>Positive regulation of endopeptidase activity</td><td>0.014</td><td>NLRP2|PDCD5|CARD9|PICALM|GSN|NCSTN|PYCARD|BAK1|CYFIP2|VCP</td><td>0.00048</td><td>GO:0010950</td></tr>
<tr><td>233</td><td>29</td><td>GO Biological Process</td><td>Vesicle organization</td><td>0.014</td><td>BET1|PIP4K2A|CORO1A|LAMTOR1|PICALM|SRGN|STX4|WASHC5|SNX3</td><td>0.00048</td><td>GO:0016050</td></tr>
<tr><td>124</td><td>19</td><td>GO Biological Process</td><td>Lymphocyte mediated immunity</td><td>0.0142</td><td>W5NPK5_SHEEP|W5NRI1_SHEEP|BTK|CD81|CORO1A|CTSC|CARD9|CD7</td><td>0.00049</td><td>GO:0002449</td></tr>
<tr><td>33</td><td>9</td><td>GO Biological Process</td><td>Cell redox homeostasis</td><td>0.0143</td><td>PRDX1|W5NUX2_SHEEP|NQO1|TXN|GCLC|GSR|PRDX2|PRDX6|TXNRD1</td><td>0.00049</td><td>GO:0045454</td></tr>
<tr><td>234</td><td>29</td><td>GO Biological Process</td><td>Autophagy</td><td>0.0148</td><td>HSPA8|PIP4K2A|GAA|WDFY3|LGALS8|GBA|WDR45B|PHB2|VPS16|TRA</td><td>0.00051</td><td>GO:0006914</td></tr>
<tr><td>85</td><td>15</td><td>GO Biological Process</td><td>Regulation of smooth muscle cell proliferation</td><td>0.0149</td><td>PDGFRB|MMP9|AIF1|SF1|PDCD4|RNASE4|PRKG1|DNMT1|IL18|MMP2</td><td>0.00052</td><td>GO:0048660</td></tr>
<tr><td>8</td><td>5</td><td>GO Biological Process</td><td>L-proline biosynthetic process</td><td>0.0149</td><td>TMED8|ALDH18A1|OAT|PYCR3|PYCR2</td><td>0.00052</td><td>GO:0055129</td></tr>
<tr><td>294</td><td>34</td><td>GO Biological Process</td><td>Regulation of lipid metabolic process</td><td>0.0159</td><td>W5NPK5_SHEEP|W5NRI1_SHEEP|STAT5A|PIP4K2A|CD81|DGKZ|FGR|LA</td><td>0.00055</td><td>GO:0019216</td></tr>
<tr><td>467</td><td>48</td><td>GO Biological Process</td><td>Regulation of plasma membrane bounded cell projection organizatior</td><td>0.0159</td><td>EVL|RTN4|FSCN1|W5P2V0_SHEEP|GDI1|AVIL|FLNA|RIPOR2|TWF2|SEP</td><td>0.00055</td><td>GO:0120035</td></tr>
<tr><td>1368</td><td>114</td><td>GO Biological Process</td><td>Regulation of intracellular signal transduction</td><td>0.0162</td><td>FLT4|MAP3K20|BAG4|CHI3L1|RIPK1|VRK2|COPS3|SASH1|TAB2|MAP2K</td><td>0.00057</td><td>GO:1902531</td></tr>
<tr><td>96</td><td>16</td><td>GO Biological Process</td><td>Regulation of mononuclear cell migration</td><td>0.0169</td><td>CORO1A|LOC101114535|MSN|RIPOR2|W5P6T0_SHEEP|CSF1R|AIF1|PY</td><td>0.0006</td><td>GO:0071675</td></tr>
<tr><td>20</td><td>7</td><td>GO Biological Process</td><td>Glucose 6-phosphate metabolic process</td><td>0.0174</td><td>GPI|PGD|TALDO1|H6PD|TKT|SHPK|RPIA</td><td>0.00062</td><td>GO:0051156</td></tr>
<tr><td>827</td><td>75</td><td>GO Biological Process</td><td>Cellular response to endogenous stimulus</td><td>0.0177</td><td>STAT5A|RAP1B|HFE|BAG4|COL1A2|CDH5|IPO5|CORO1A|LAMTOR1|PD</td><td>0.00063</td><td>GO:0071495</td></tr>
<tr><td>666</td><td>63</td><td>GO Biological Process</td><td>Negative regulation of catalytic activity</td><td>0.0179</td><td>W5NPK5_SHEEP|W5NRI1_SHEEP|TFPI2|A2M|BAG4|ITIH1|RENBP|PIP4K</td><td>0.00064</td><td>GO:0043086</td></tr>
<tr><td>27</td><td>8</td><td>GO Biological Process</td><td>Polyol catabolic process</td><td>0.0179</td><td>TPI1|NUDT3|BPNT1|INPP5K|SYNJ1|OCRL|IMPA1|GK</td><td>0.00064</td><td>GO:0046174</td></tr>
<tr><td>14</td><td>6</td><td>GO Biological Process</td><td>Negative regulation of glucose transmembrane transport</td><td>0.0182</td><td>PEA15|FABP5|INPP5K|ENPP1|APPL2|PRKCB</td><td>0.00065</td><td>GO:0010829</td></tr>
<tr><td>238</td><td>29</td><td>GO Biological Process</td><td>Positive regulation of cell-cell adhesion</td><td>0.0182</td><td>STAT5A|W5NYC7_SHEEP|CD81|CORO1A|DPP4|CD74|SIRPA|AIF1|PLAU</td><td>0.00065</td><td>GO:0022409</td></tr>
<tr><td>78</td><td>14</td><td>GO Biological Process</td><td>Cell killing</td><td>0.0189</td><td>W5NPK5_SHEEP|W5NRI1_SHEEP|STXBP2|CORO1A|CTSC|PPP3CB|GAPD</td><td>0.00068</td><td>GO:0001906</td></tr>
<tr><td>78</td><td>14</td><td>GO Biological Process</td><td>Negative regulation of transmembrane transport</td><td>0.0189</td><td>CLIC2|MMP9|PEA15|FABP5|UBQLN1|SRI|SLC9A3R1|INPP5K|ENPP1|AP</td><td>0.00068</td><td>GO:0034763</td></tr>
<tr><td>35</td><td>9</td><td>GO Biological Process</td><td>Positive regulation of interleukin-2 production</td><td>0.0194</td><td>STAT5A|SPTBN1|PLCG2|PRKD2|ANXA1|PRKCQ|SASH3|STAT5B|IL1B</td><td>0.00071</td><td>GO:0032743</td></tr>
<tr><td>347</td><td>38</td><td>GO Biological Process</td><td>Cellular response to organic cyclic compound</td><td>0.0194</td><td>RAP1B|SPP1|CYP1A1|FOLR2|LOC101114535|W5P2V0_SHEEP|BRCA1|P</td><td>0.0007</td><td>GO:0071407</td></tr>
<tr><td>43</td><td>10</td><td>GO Biological Process</td><td>Ribonucleoside monophosphate biosynthetic process</td><td>0.0195</td><td>PFAS|DHODH|W5P7K2_SHEEP|UCK1|ADK|W5PMA3_SHEEP|HPRT1|AP</td><td>0.00071</td><td>GO:0009156</td></tr>
<tr><td>43</td><td>10</td><td>GO Biological Process</td><td>Regulation of cell-substrate junction organization</td><td>0.0195</td><td>PEAK1|MAPRE2|VCL|ARHGAP6|PPM1F|CORO1C|ACTG1|MACF1|APOD</td><td>0.00071</td><td>GO:0150116</td></tr>
<tr><td>98</td><td>16</td><td>GO Biological Process</td><td>Myeloid leukocyte migration</td><td>0.0199</td><td>LOC101114535|LOC100101238|W5P3V8_SHEEP|VAV1|WDR1|CCL2|PRT</td><td>0.00073</td><td>GO:0097529</td></tr>
<tr><td>172</td><td>23</td><td>GO Biological Process</td><td>Ameboidal-type cell migration</td><td>0.0203</td><td>CDH5|CORO1A|LGALS8|VHL|SGPL1|DPP4|CDC42|CYP1B1|PTPN11|PPA</td><td>0.00075</td><td>GO:0001667</td></tr>
</table>

Supplementary Table 11: Comparative functional enrichment analysis of GMØ (monocyte-derived macrophages differentiated with GM-CSF) and MMØ (monocyte-derived macrophages differentiated with M-CSF) relative to Mo (monocytes at 3 hours). Functional enrichment analysis was performed using the StringApp (STRING database) within Cytoscape (version 3.10.3)[1], focusing on Gene Ontology (GO) Biological Process (BP) terms[2,3]. Red-highlighted cells indicate Gene Ontology Biological Process terms shared between GMØ and MMØ compared to Mo, whereas non-highlighted cells represent subtype-specific enrichment.

172	23	GO Biological Process	Leukocyte mediated immunity	0.0203	W5NPK5_SHEEP|W5NRI1_SHEEP|BTK|STXBP2|CD81|CORO1A|CTSC|LO	0.00075	GO:0002443
161	22	GO Biological Process	Macroautophagy	0.0203	PIP4K2A|GAA|WDFY3|LGALS8|GBA|WDR45B|PHB2|VPS16|TRAPPC8|N	0.00075	GO:0016236
79	14	GO Biological Process	Cellular response to reactive oxygen species	0.0204	RIPK1|PRDX1|NQO1|SOD1-2|MMP9|CYP1B1|PARK7|PRDX2|ANXA1|W	0.00076	GO:0034614
539	53	GO Biological Process	Innate immune response	0.0204	W5NPK5_SHEEP|IFNGR1|W5NRI1_SHEEP|CLEC4A|W5NY14_SHEEP|ATA	0.00076	GO:0045087
436	45	GO Biological Process	Organophosphate biosynthetic process	0.0204	PFAS|PIP4K2A|NME1-NME2|PMVK|LCAT|DGKZ|DHODH|SLC25A13|ALD	0.00075	GO:0090407
462	47	GO Biological Process	Regulation of defense response	0.0206	W5NPK5_SHEEP|W5NRI1_SHEEP|STAT5A|RIPK1|CDH5|NLRP2|CD81|FG	0.00077	GO:0031347
288	33	GO Biological Process	Positive regulation of cell activation	0.0206	STAT5A|IGHM|W5NYC7_SHEEP|CD81|FGR|CORO1A|CTSC|DPP4|STAP1	0.00077	GO:0050867
52	11	GO Biological Process	Nucleoside monophosphate biosynthetic process	0.0208	PFAS|DHODH|W5P7K2_SHEEP|UCK1|ADK|TYMS|W5PMA3_SHEEP|HPR	0.00078	GO:0009124
52	11	GO Biological Process	Vesicle docking	0.0208	STXBP2|STX4|RAB3A|STX7|STX2|USO1|YKT6|STX5|STX6|PLEK|VPS18	0.00078	GO:0048278
70	13	GO Biological Process	Lysosome organization	0.021	NAGLU|GAA|CORO1A|LAMTOR1|GBA|ACP2|GRN|WASHC5|GNPTAB|T	0.00079	GO:0007040
218	27	GO Biological Process	Positive regulation of defense response	0.021	W5NPK5_SHEEP|W5NRI1_SHEEP|STAT5A|RIPK1|CD81|CTSC|LOC101114	0.00079	GO:0031349
151	21	GO Biological Process	Cellular response to oxidative stress	0.021	RIPK1|PRDX1|VRK2|NQO1|SOD1-2|SIRT2|MMP9|AIF1|CYP1B1|PARK7|	0.00079	GO:0034599
109	17	GO Biological Process	Organelle fusion	0.021	BET1|PIP4K2A|CORO1A|VPS16|STX4|BAK1|DYSF|STX7|FIS1|STX2|USO	0.00079	GO:0048284
61	12	GO Biological Process	Positive regulation of phagocytosis	0.021	W5NPK5_SHEEP|W5NRI1_SHEEP|SOD1-2|STAP1|SIRPA|PYCARD|PLCG2	0.0008	GO:0050766
780	71	GO Biological Process	Positive regulation of intracellular signal transduction	0.021	FLT4|MAP3K20|BAG4|CHI3L1|RIPK1|SASH1|TAB2|MAP2K3|RTN4|SPA	0.00079	GO:1902533
242	29	GO Biological Process	Regulation of intracellular transport	0.0217	BAG4|IPO5|CD81|RAB11B|PDCD5|W5P2V0_SHEEP|GDI1|MSN|FLNA|T	0.00083	GO:0032386
120	18	GO Biological Process	Endoplasmic reticulum to Golgi vesicle-mediated transport	0.0221	BET1|TRAPPC5|LMAN1|TRAPPC8|COPB1|COPB2|TRAPPC6B|COPA|ARC	0.00085	GO:0006888
1143	97	GO Biological Process	Protein-containing complex assembly	0.0223	EIF3I|GSDMD|W5NRD9_SHEEP|EIF3A|SPAG1|EVL|H3-4|COL1A2|RIPK1|	0.00086	GO:0065003
15	6	GO Biological Process	Regulation of phagocytosis, engulfment	0.0225	W5NPK5_SHEEP|W5NRI1_SHEEP|STAP1|ALOX15|PLCG2|APPL2	0.00087	GO:0060099
90	15	GO Biological Process	Antigen processing and presentation	0.0226	CLEC4A|W5NYC7_SHEEP|GBA|CD74|CTSV|FCGR3A|PDIA3|IFI30|W5PX	0.00087	GO:0019882
609	58	GO Biological Process	Cell morphogenesis	0.0229	PEAK1|CDH5|RAC1|PSMB10|TOP2B|SOD1-2|PICALM|FERMT3|RIPOR2|	0.00089	GO:0000902
53	11	GO Biological Process	Regulation of myeloid leukocyte mediated immunity	0.0229	W5NPK5_SHEEP|W5NRI1_SHEEP|FGR|STAP1|ITGAM|UNC13D|ITGB2|F	0.00089	GO:0002886
743	68	GO Biological Process	Positive regulation of phosphate metabolic process	0.0229	W5NPK5_SHEEP|FLT4|W5NRI1_SHEEP|RALBP1|HFE|ITGA6|BAG4|CHI3L	0.00089	GO:0045937
2869	214	GO Biological Process	Organic cyclic compound metabolic process	0.0232	PABPC1|HSPA8|DRAP1|PFAS|W5NRS0_SHEEP|FBXO6|ALDH6A1|W5NS	0.00091	GO:1901360
771	70	GO Biological Process	Defense response to other organism	0.0233	W5NPK5_SHEEP|IFNGR1|GSDMD|W5NRI1_SHEEP|CLEC4A|STAB1|W5N	0.00091	GO:0098542
1062	91	GO Biological Process	Regulation of response to stress	0.0238	W5NPK5_SHEEP|FLT4|MAP3K20|W5NRI1_SHEEP|STAT5A|RIPK1|CDH5|	0.00093	GO:0080134
29	8	GO Biological Process	Homeostasis of number of cells within a tissue	0.0239	CORO1A|VHL|FH|PTPN11|ADD1|SASH3|LIPA|CSF1	0.00094	GO:0048873
304	34	GO Biological Process	Organic cyclic compound catabolic process	0.0239	PABPC1|ALDH6A1|W5NUX2_SHEEP|SPP1|MAOA|HMOX2|CTIF|RNASET	0.00094	GO:1901361
111	17	GO Biological Process	Response to temperature stimulus	0.0241	DNAJC3|HSP90AA1|SOD1-2|HIKESHI|CLPB|GCLC|HSP90AB1|VCP|YWHA	0.00095	GO:0009266
341	37	GO Biological Process	Cellular response to growth factor stimulus	0.0241	FLT4|HFE|BAG4|COL1A2|PGF|RIPK1|CDH5|MAP2K3|CORO1A|PDCD5|	0.00095	GO:0071363
91	15	GO Biological Process	Polyol metabolic process	0.0243	GBA|TPI1|PCBD1|ASAH1|PLCG2|GALK1|NUDT3|BPNT1|INPP5K|SYNJ1|	0.00096	GO:0019751
101	16	GO Biological Process	Positive regulation of lymphocyte proliferation	0.0243	STAT5A|CD81|CORO1A|CD74|AIF1|PYCARD|DHPS|ANXA1|PRKCQ|PPP	0.00097	GO:0050671
22	7	GO Biological Process	Regulation of histone H3-K9 methylation	0.0245	BRCA1|LMNA|JARID2|PIH1D1|SETD7|DNMT1|ATRX	0.00098	GO:0051570
37	9	GO Biological Process	Lipid storage	0.0247	STAT5A|GBA|GM2A|ENPP1|SOAT1|W5Q6N3_SHEEP|HEXA|STAT5B|IL1	0.00099	GO:0019915
37	9	GO Biological Process	Cellular response to heat	0.0247	HSP90AA1|HIKESHI|CLPB|HSP90AB1|VCP|YWHAE|PDCD6|HMOX1|W5	0.00099	GO:0034605
37	9	GO Biological Process	Cellular response to vascular endothelial growth factor stimulus	0.0247	FLT4|PGF|MAP2K3|PDGFRB|PRKD2|GAB1|ANXA1|HSPB1|NRP2	0.00099	GO:0035924
176	23	GO Biological Process	Regulation of chemotaxis	0.0247	PGF|MPP1|LOC101114535|RIPOR2|CSF1R|DPP4|STAP1|CD74|AIF1|US	0.00099	GO:0050920
63	12	GO Biological Process	Antigen processing and presentation of peptide antigen	0.0253	CLEC4A|W5NYC7_SHEEP|CD74|CTSV|PDIA3|IFI30|W5PXC6_SHEEP|W5	0.001	GO:0048002
5	4	GO Biological Process	Pentose-phosphate shunt, non-oxidative branch	0.0258	TALDO1|TKT|SHPK|RPIA	0.001	GO:0009052
5	4	GO Biological Process	Heme catabolic process	0.0258	HMOX2|BLVRB|BLVRA|HMOX1	0.001	GO:0042167
1322	109	GO Biological Process	Negative regulation of response to stimulus	0.0259	HFE|ITGA6|RIPK1|PIP4K2A|CDH5|RGS10|MAP2K3|PSMD10|PEBP1|GB	0.0011	GO:0048585
144	20	GO Biological Process	Positive regulation of supramolecular fiber organization	0.026	BAG4|EVL|GSN|SCIN|WDR1|CDC42|ALOX15|HSPA1A-2|PYCARD|VASP|	0.0011	GO:1902905
200	25	GO Biological Process	Negative regulation of cytokine production	0.0264	HFE|LOC443162|BTK|CLEC4A|GBA|W5P2V0_SHEEP|VSIG4|ERBIN|PYCA	0.0011	GO:0001818
369	39	GO Biological Process	Regulation of establishment of protein localization	0.0268	GLUD1|BAG4|IPO5|CD81|RBP4|CNST|PDCD5|W5P2V0_SHEEP|TCP1|G	0.0011	GO:0070201
10	5	GO Biological Process	Cellular detoxification of aldehyde	0.0268	AKR1A1|ESD|PARK7|ALDH1A1|RDH11	0.0011	GO:0110095
1125	95	GO Biological Process	Positive regulation of multicellular organismal process	0.0271	W5NPK5_SHEEP|GSDMD|FLT4|W5NRI1_SHEEP|STAT5A|CHI3L1|PGF|RI	0.0011	GO:0051240
30	8	GO Biological Process	Modulation by host of viral process	0.0275	HSPA8|IGF2R|CSF1R|PSMC3|CDC42|RAB5A|CFL1|PPIB	0.0011	GO:0044788
30	8	GO Biological Process	Regulation of macrophage migration	0.0275	CD81|CSF1R|STAP1|CNN2|C5AR1|MAPK1|CSF1|MMP14	0.0011	GO:1905521
236	28	GO Biological Process	Regulation of ERK1 and ERK2 cascade	0.0279	FLT4|CHI3L1|LOC101114535|W5P2V0_SHEEP|CARD9|LOC100101238|W	0.0012	GO:0070372
23	7	GO Biological Process	Protein targeting to lysosome	0.0292	HSPA8|GGA3|LAMP2|ZFYVE16|GNPTAB|SCARB2|NEDD4	0.0012	GO:0006622
740	67	GO Biological Process	Regulation of transferase activity	0.0298	FLT4|CHI3L1|TPX2|PIP4K2A|DNAJC3|SASH1|TAB2|MAP2K3|PSMD10|C	0.0012	GO:0051338
754	68	GO Biological Process	Negative regulation of multicellular organismal process	0.0301	HFE|LOC443162|EVL|BTK|EFEMP1|CLEC4A|STAB1|RTN4|W5P026_SHEE	0.0013	GO:0051241
47	10	GO Biological Process	Positive regulation of receptor-mediated endocytosis	0.0306	W5NPK5_SHEEP|W5NRI1_SHEEP|HFE|W5P9L9_SHEEP|PLCG2|DAB2|AR	0.0013	GO:0048260
94	15	GO Biological Process	Blood coagulation	0.0307	TFPI2|TREML1|FERMT3|CPB2|F5|F9|ITGB3|F13B|ANXA5|RAP2B|PROC	0.0013	GO:0007596
65	12	GO Biological Process	Regulation of cell morphogenesis involved in differentiation	0.0307	LIMS1|FLNA|ACTN4|CDC42|UNC13D|ITGB3|DBNL|CDKL3|PAFAH1B1|IL	0.0013	GO:0010769
894	78	GO Biological Process	Negative regulation of protein metabolic process	0.0319	W5NPK5_SHEEP|W5NRI1_SHEEP|TFPI2|A2M|HFE|ITIH1|DNAJC3|RARR	0.0014	GO:0051248

Supplementary Table 11: Comparative functional enrichment analysis of GMØ (monocyte-derived macrophages differentiated with GM-CSF) and MMØ (monocyte-derived macrophages differentiated with M-CSF) relative to Mo (monocytes at 3 hours). Functional enrichment analysis was performed using the StringApp (STRING database) within Cytoscape (version 3.10.3)[1], focusing on Gene Ontology (GO) Biological Process (BP) terms[2,3]. Red-highlighted cells indicate Gene Ontology Biological Process terms shared between GMØ and MMØ compared to Mo, whereas non-highlighted cells represent subtype-specific enrichment.

<table>
<tr><td>39</td><td>9</td><td>GO Biological Process</td><td>Regulation of focal adhesion assembly</td><td>0.0321</td><td>PEAK1|VCL|ARHGAP6|PPM1F|CORO1C|ACTG1|MACF1|APOD|HRG</td><td>0.0014</td><td>GO:0051893</td></tr>
<tr><td>31</td><td>8</td><td>GO Biological Process</td><td>Glycosyl compound catabolic process</td><td>0.0321</td><td>GUSB|FUCA2|GLA|FUCA1|W5PKU3_SHEEP|ADA2|UPP1|NAGA</td><td>0.0014</td><td>GO:1901658</td></tr>
<tr><td>263</td><td>30</td><td>GO Biological Process</td><td>Cellular component disassembly</td><td>0.0324</td><td>W5NPK5_SHEEP|HSPA8|W5NRI1_SHEEP|SH3PXD2B|GBA|WDR45B|PHB</td><td>0.0014</td><td>GO:0022411</td></tr>
<tr><td>148</td><td>20</td><td>GO Biological Process</td><td>Negative regulation of protein localization</td><td>0.0334</td><td>BAG4|GDI1|PICALM|RIPOR2|TXN|DAB2|PTPN11|LZTFL1|PARK7|SNX3|</td><td>0.0014</td><td>GO:1903828</td></tr>
<tr><td>17</td><td>6</td><td>GO Biological Process</td><td>Inositol phosphate catabolic process</td><td>0.0341</td><td>NUDT3|BPNT1|INPP5K|SYNJ1|OCRL|IMPA1</td><td>0.0015</td><td>GO:0071545</td></tr>
<tr><td>313</td><td>34</td><td>GO Biological Process</td><td>Negative regulation of hydrolase activity</td><td>0.0342</td><td>W5NPK5_SHEEP|W5NRI1_SHEEP|TFPI2|A2M|BAG4|ITIH1|RARRES1|PIC</td><td>0.0015</td><td>GO:0051346</td></tr>
<tr><td>24</td><td>7</td><td>GO Biological Process</td><td>Regulation of fibroblast migration</td><td>0.0346</td><td>BAG4|ITGB3|SLC9A3R1|W5PVN6_SHEEP|APPL2|CORO1C|FER</td><td>0.0015</td><td>GO:0010762</td></tr>
<tr><td>11</td><td>5</td><td>GO Biological Process</td><td>Meiotic spindle organization</td><td>0.035</td><td>TUBG1|SEPTIN1|WASHC5|ATRX|MYH9</td><td>0.0015</td><td>GO:0000212</td></tr>
<tr><td>638</td><td>59</td><td>GO Biological Process</td><td>Regulation of kinase activity</td><td>0.035</td><td>FLT4|CHI3L1|TPX2|PIP4K2A|DNAJC3|SASH1|TAB2|MAP2K3|PSMD10|C</td><td>0.0015</td><td>GO:0043549</td></tr>
<tr><td>571</td><td>54</td><td>GO Biological Process</td><td>Hematopoietic or lymphoid organ development</td><td>0.035</td><td>FLT4|STAT5A|BTK|GCC1|PIP4K2A|CD81|GBA|PICALM|VAV1|W5P5N5_</td><td>0.0015</td><td>GO:0048534</td></tr>
<tr><td>760</td><td>68</td><td>GO Biological Process</td><td>Chemical homeostasis</td><td>0.035</td><td>NPC2|HFE|CDH5|ATP6V0A1|RTN4|ATP6V0D1|RBP4|LCAT|HMOX2|BOL</td><td>0.0015</td><td>GO:0048878</td></tr>
<tr><td>11</td><td>5</td><td>GO Biological Process</td><td>Actin filament severing</td><td>0.035</td><td>AVIL|GSN|SCIN|SVIL|CAPG</td><td>0.0015</td><td>GO:0051014</td></tr>
<tr><td>206</td><td>25</td><td>GO Biological Process</td><td>Regulation of leukocyte mediated immunity</td><td>0.0358</td><td>W5NPK5_SHEEP|W5NRI1_SHEEP|HFE|CD81|FGR|VAV1|STAP1|PPP3CB|</td><td>0.0016</td><td>GO:0002703</td></tr>
<tr><td>32</td><td>8</td><td>GO Biological Process</td><td>NADP metabolic process</td><td>0.0369</td><td>PGD|TALDO1|ALDH1L1|H6PD|NADK2|TKT|SHPK|RPIA</td><td>0.0016</td><td>GO:0006739</td></tr>
<tr><td>32</td><td>8</td><td>GO Biological Process</td><td>Nucleobase metabolic process</td><td>0.0369</td><td>ALDH6A1|DHODH|ADK|HPRT1|GMPR|APRT|MAPK1|SHMT1</td><td>0.0016</td><td>GO:0009112</td></tr>
<tr><td>32</td><td>8</td><td>GO Biological Process</td><td>Regulation of actin nucleation</td><td>0.0369</td><td>CORO1A|GSN|SCIN|WASHC5|LOC101105107|CORO1B|CYFIP1|GMFB</td><td>0.0016</td><td>GO:0051125</td></tr>
<tr><td>128</td><td>18</td><td>GO Biological Process</td><td>Adaptive immune response based on somatic recombination of immu</td><td>0.037</td><td>W5NPK5_SHEEP|W5NRI1_SHEEP|BTK|CD81|IL18BP|CTSC|CARD9|CD74</td><td>0.0016</td><td>GO:0002460</td></tr>
<tr><td>6</td><td>4</td><td>GO Biological Process</td><td>Protein catabolic process in the vacuole</td><td>0.0376</td><td>VPS13A|LAMP2|TCIRG1|TPP1</td><td>0.0017</td><td>GO:0007039</td></tr>
<tr><td>1029</td><td>87</td><td>GO Biological Process</td><td>Immune response</td><td>0.0387</td><td>W5NPK5_SHEEP|IFNGR1|W5NRI1_SHEEP|BTK|STXBP2|CLEC4A|W5NY14</td><td>0.0017</td><td>GO:0006955</td></tr>
<tr><td>87</td><td>14</td><td>GO Biological Process</td><td>Cellular monovalent inorganic cation homeostasis</td><td>0.0387</td><td>ATP6V0A1|ATP6V0D1|SLC8A1|GRN|ATP1A2|ATP1B1|ATP6V1B2|CA2|T</td><td>0.0017</td><td>GO:0030004</td></tr>
<tr><td>280</td><td>31</td><td>GO Biological Process</td><td>Positive regulation of leukocyte activation</td><td>0.0398</td><td>STAT5A|IGHM|W5NYC7_SHEEP|CD81|FGR|CORO1A|CTSC|DPP4|STAP1</td><td>0.0018</td><td>GO:0002696</td></tr>
<tr><td>470</td><td>46</td><td>GO Biological Process</td><td>Response to organic cyclic compound</td><td>0.0398</td><td>RAP1B|SPP1|PMVK|CYP1A1|FOLR2|LOC101114535|W5P2V0_SHEEP|CA</td><td>0.0018</td><td>GO:0014070</td></tr>
<tr><td>470</td><td>46</td><td>GO Biological Process</td><td>Cellular response to nitrogen compound</td><td>0.0398</td><td>STAT5A|RAP1B|COL1A2|RGS10|IPO5|LAMTOR1|FOLR2|W5P2V0_SHEEP</td><td>0.0018</td><td>GO:1901699</td></tr>
<tr><td>108</td><td>16</td><td>GO Biological Process</td><td>Positive regulation of leukocyte migration</td><td>0.0401</td><td>PGF|CORO1A|LOC101114535|RIPOR2|CSF1R|CD74|AIF1|PYCARD|SELP</td><td>0.0018</td><td>GO:0002687</td></tr>
<tr><td>268</td><td>30</td><td>GO Biological Process</td><td>Glycerophospholipid metabolic process</td><td>0.0401</td><td>PLCD1|PNPLA6|PIP4K2A|LCAT|DGKZ|W5NZK6_SHEEP|PIK3C2A|INPPL1|</td><td>0.0018</td><td>GO:0006650</td></tr>
<tr><td>25</td><td>7</td><td>GO Biological Process</td><td>Antigen processing and presentation of exogenous peptide antigen vi</td><td>0.0404</td><td>W5NYC7_SHEEP|CD74|IFI30|W5Q2C4_SHEEP|W5Q2V1_SHEEP|W5Q3J8</td><td>0.0018</td><td>GO:0019886</td></tr>
<tr><td>3373</td><td>244</td><td>GO Biological Process</td><td>Cellular nitrogen compound metabolic process</td><td>0.0407</td><td>PABPC1|HSPA8|CPM|DRAP1|EIF3I|PFAS|EIF3A|W5NRS0_SHEEP|FBXO6</td><td>0.0018</td><td>GO:0034641</td></tr>
<tr><td>2442</td><td>183</td><td>GO Biological Process</td><td>Cellular component biogenesis</td><td>0.0407</td><td>EIF3I|GSDMD|W5NRD9_SHEEP|EIF3A|SPAG1|BAG4|TPX2|EVL|H3-4|CC</td><td>0.0018</td><td>GO:0044085</td></tr>
<tr><td>41</td><td>9</td><td>GO Biological Process</td><td>Monosaccharide biosynthetic process</td><td>0.0407</td><td>AKR1A1|RBP4|GPI|PGD|TPI1|PGK2|MDH2|RGN|PGK1</td><td>0.0018</td><td>GO:0046364</td></tr>
<tr><td>41</td><td>9</td><td>GO Biological Process</td><td>Protein depolymerization</td><td>0.0407</td><td>HSPA8|GSN|STMN1|TWF2|KIF2A|WDR1|MICAL1|CFL1|TWF1</td><td>0.0018</td><td>GO:0051261</td></tr>
<tr><td>98</td><td>15</td><td>GO Biological Process</td><td>Cytokinesis</td><td>0.0409</td><td>ARL3|SPTBN1|SEPTIN7|SEPTIN1|SEPTIN9|SNX18|WASHC5|ACTR3|SEPT</td><td>0.0019</td><td>GO:0000910</td></tr>
<tr><td>18</td><td>6</td><td>GO Biological Process</td><td>Response to platelet-derived growth factor</td><td>0.0409</td><td>PDGFRB|ITGB3|CORO1B|IQGAP1|W5PVN6_SHEEP|FER</td><td>0.0019</td><td>GO:0036119</td></tr>
<tr><td>33</td><td>8</td><td>GO Biological Process</td><td>intra-Golgi vesicle-mediated transport</td><td>0.0415</td><td>COG8|COG5|COPB1|COPB2|COPA|ARCN1|GOLGA5|USO1</td><td>0.0019</td><td>GO:0006891</td></tr>
<tr><td>59</td><td>11</td><td>GO Biological Process</td><td>Substrate adhesion-dependent cell spreading</td><td>0.0415</td><td>PEAK1|FERMT3|PXN|ITGB3|W5PVN6_SHEEP|ITGA4|ILK|FER|PARVB|RA</td><td>0.0019</td><td>GO:0034446</td></tr>
<tr><td>245</td><td>28</td><td>GO Biological Process</td><td>Positive regulation of GTPase activity</td><td>0.0415</td><td>TBC1D2B|RALBP1|ITGA6|TBC1D9B|RGS10|LOC101114535|PICALM|LOC</td><td>0.0019</td><td>GO:0043547</td></tr>
<tr><td>33</td><td>8</td><td>GO Biological Process</td><td>Cellular carbohydrate catabolic process</td><td>0.0415</td><td>GUSB|GAA|TPI1|PYGB|W5PKU3_SHEEP|STBD1|GK|PYGL</td><td>0.0019</td><td>GO:0044275</td></tr>
<tr><td>33</td><td>8</td><td>GO Biological Process</td><td>Phosphatidylinositol dephosphorylation</td><td>0.0415</td><td>INPPL1|MTMR3|FIG4|INPP5K|MTMR6|SYNJ1|OCRL|MTMR9</td><td>0.0019</td><td>GO:0046856</td></tr>
<tr><td>33</td><td>8</td><td>GO Biological Process</td><td>Platelet-derived growth factor receptor signaling pathway</td><td>0.0415</td><td>SGPL1|PDGFRB|PTPN11|ITGB3|IQGAP1|W5PVN6_SHEEP|FER|MYO1E</td><td>0.0019</td><td>GO:0048008</td></tr>
<tr><td>186</td><td>23</td><td>GO Biological Process</td><td>Positive regulation of T cell activation</td><td>0.0415</td><td>STAT5A|W5NYC7_SHEEP|CD81|CORO1A|DPP4|CD74|SIRPA|AIF1|PYCA</td><td>0.0019</td><td>GO:0050870</td></tr>
<tr><td>130</td><td>18</td><td>GO Biological Process</td><td>Vesicle localization</td><td>0.0415</td><td>RAB11B|PICALM|ACTN4|DCTN2|MAP4K2|TSG101|RAB3A|MYO1G|TCIR</td><td>0.0019</td><td>GO:0051648</td></tr>
<tr><td>130</td><td>18</td><td>GO Biological Process</td><td>Cellular response to tumor necrosis factor</td><td>0.0415</td><td>BAG4|CHI3L1|RIPK1|GBA|LOC101114535|LOC100101238|W5P3V8_SHE</td><td>0.0019</td><td>GO:0071356</td></tr>
<tr><td>221</td><td>26</td><td>GO Biological Process</td><td>Positive regulation of establishment of protein localization</td><td>0.0415</td><td>GLUD1|IPO5|CD81|RBP4|CNST|PDCD5|W5P2V0_SHEEP|TCP1|FLNA|CC</td><td>0.0019</td><td>GO:1904951</td></tr>
<tr><td>1817</td><td>141</td><td>GO Biological Process</td><td>Anatomical structure morphogenesis</td><td>0.0418</td><td>FLT4|MAP3K20|ITGA6|COL1A2|PGF|PEAK1|CDH5|RAC1|EFEMP1|NAGL</td><td>0.0019</td><td>GO:0009653</td></tr>
<tr><td>319</td><td>34</td><td>GO Biological Process</td><td>Negative regulation of immune system process</td><td>0.042</td><td>HFE|LOC443162|BTK|FGR|LOC101114535|W5P2V0_SHEEP|CD22|VSIG4</td><td>0.002</td><td>GO:0002683</td></tr>
<tr><td>1034</td><td>87</td><td>GO Biological Process</td><td>Defense response</td><td>0.042</td><td>W5NPK5_SHEEP|IFNGR1|GSDMD|W5NRI1_SHEEP|CHI3L1|CLEC4A|STAB</td><td>0.002</td><td>GO:0006952</td></tr>
<tr><td>606</td><td>56</td><td>GO Biological Process</td><td>Immune system development</td><td>0.0429</td><td>FLT4|STAT5A|BTK|GCC1|PIP4K2A|CD81|GBA|PICALM|VAV1|W5P5N5_</td><td>0.002</td><td>GO:0002520</td></tr>
<tr><td>12</td><td>5</td><td>GO Biological Process</td><td>Positive regulation by host of viral process</td><td>0.0439</td><td>HSPA8|IGF2R|CSF1R|CFL1|PPIB</td><td>0.0021</td><td>GO:0044794</td></tr>
<tr><td>12</td><td>5</td><td>GO Biological Process</td><td>pH reduction</td><td>0.0439</td><td>ATP6V0A1|ATP6V0D1|ATP6V1B2|TCIRG1|ATP6V1C1</td><td>0.0021</td><td>GO:0045851</td></tr>
<tr><td>223</td><td>26</td><td>GO Biological Process</td><td>Protein maturation</td><td>0.0449</td><td>CPM|BOLA2B|CTSC|GLG1|CASP6|TLL2|CTSV|CTSL|NCSTN|PYCARD|DD</td><td>0.0021</td><td>GO:0051604</td></tr>
<tr><td>110</td><td>16</td><td>GO Biological Process</td><td>Positive regulation of cysteine-type endopeptidase activity</td><td>0.045</td><td>NLRP2|PDCD5|CARD9|GSN|PYCARD|BAK1|CYFIP2|VCP|LGMN|HIP1|CT</td><td>0.0021</td><td>GO:2001056</td></tr>
<tr><td>154</td><td>20</td><td>GO Biological Process</td><td>Negative regulation of leukocyte activation</td><td>0.046</td><td>HFE|LOC443162|BTK|FGR|VSIG4|W5P5N5_SHEEP|RIPOR2|CD74|GRN|</td><td>0.0022</td><td>GO:0002695</td></tr>
<tr><td>100</td><td>15</td><td>GO Biological Process</td><td>Response to reactive oxygen species</td><td>0.0466</td><td>RIPK1|PRDX1|NQO1|SOD1-2|MMP9|CYP1B1|PARK7|PRDX2|ANXA1|W</td><td>0.0022</td><td>GO:0000302</td></tr>
<tr><td>100</td><td>15</td><td>GO Biological Process</td><td>Regulation of DNA biosynthetic process</td><td>0.0466</td><td>LOC101104501|TCP1|CCT3|PKIB|CCT6A|PRKD2|CCT7|NIBAN2|PRKCQ|</td><td>0.0022</td><td>GO:2000278</td></tr>
<tr><td>34</td><td>8</td><td>GO Biological Process</td><td>Cellular metabolic compound salvage</td><td>0.047</td><td>W5NRS0_SHEEP|UCK1|ADK|PDXK|HPRT1|APRT|UPP1|BHMT</td><td>0.0022</td><td>GO:0043094</td></tr>
<tr><td>70</td><td>12</td><td>GO Biological Process</td><td>Regulation of chemokine production</td><td>0.0471</td><td>CARD9|ERBIN|CSF1R|CD74|AIF1|PYCARD|LPL|TLR7|EPHA2|TLR2|APOD</td><td>0.0023</td><td>GO:0032642</td></tr>
<tr><td>90</td><td>14</td><td>GO Biological Process</td><td>Regulation of reactive oxygen species metabolic process</td><td>0.0475</td><td>RIPK1|SOD1-2|BRCA1|MMP3|PRCP|SIRT2|PLCG2|ITGAM|CYP1B1|PARK</td><td>0.0023</td><td>GO:2000377</td></tr>
</table>

Supplementary Table 11: Comparative functional enrichment analysis of GMØ (monocyte-derived macrophages differentiated with GM-CSF) and MMØ (monocyte-derived macrophages differentiated with M-CSF) relative to Mo (monocytes at 3 hours). Functional enrichment analysis was performed using the StringApp (STRING database) within Cytoscape (version 3.10.3)[1], focusing on Gene Ontology (GO) Biological Process (BP) terms[2,3]. Red-highlighted cells indicate Gene Ontology Biological Process terms shared between GMØ and MMØ compared to Mo, whereas non-highlighted cells represent subtype-specific enrichment.

<table>
<tr><td>189</td><td>23</td><td>GO Biological Process</td><td>Response to metal ion</td><td>0.0477</td><td>HFE|SLC25A13|MMP9|ALOX15|RASGRP2|NCSTN|PLCG2|FABP4|CUTA|</td><td>0.0023</td><td>GO:0010038</td></tr>
<tr><td>348</td><td>36</td><td>GO Biological Process</td><td>Cellular ion homeostasis</td><td>0.0485</td><td>HFE|CDH5|ATP6V0A1|ATP6V0D1|BOLA2B|SOD1-2|LOC101114535|WFS</td><td>0.0023</td><td>GO:0006873</td></tr>
<tr><td>213</td><td>25</td><td>GO Biological Process</td><td>Homeostasis of number of cells</td><td>0.049</td><td>STAT5A|CORO1A|GBA|SOD1-2|GPI|CARD9|VHL|PPP3CB|FH|NCSTN|PT</td><td>0.0024</td><td>GO:0048872</td></tr>
</table>

Functional enrichment analysis of MMØ_Mo

<table>
<tr><th># background genes</th><th># genes</th><th>category</th><th>description_MMØ_Mo</th><th>FDR value</th><th>genes</th><th>p-value</th><th>term name</th></tr>
<tr><td>1197</td><td>146</td><td>GO Biological Process</td><td>Vesicle-mediated transport</td><td>2.65E-17</td><td>4K2|BIN1-2|MSR1|FAM91A1|PRTN3|CCDC22|CNN2|GRB2|AP2S1|SNX3</td><td>2.33E-21</td><td>GO:0016192</td></tr>
<tr><td>4284</td><td>346</td><td>GO Biological Process</td><td>Localization</td><td>1.2E-16</td><td>NA|VPS16|SNX8|COG5|W5P5N5_SHEEP|EMC3|RIPOR2|GSN|LMNA|DD</td><td>2.11E-20</td><td>GO:0051179</td></tr>
<tr><td>3748</td><td>308</td><td>GO Biological Process</td><td>Establishment of localization</td><td>3.08E-15</td><td>T3|W5P7F8_SHEEP|SUN1|ATP6V1G1|SEPTIN1|PRG4|SLC17A5|CRABP2|</td><td>8.13E-19</td><td>GO:0051234</td></tr>
<tr><td>3596</td><td>294</td><td>GO Biological Process</td><td>Transport</td><td>4.29E-14</td><td>PRG4|SLC17A5|CRABP2|TRAPPC8|HEATR5A|PITPNM2|W5P8J8_SHEEP|</td><td>1.51E-17</td><td>GO:0006810</td></tr>
<tr><td>1731</td><td>172</td><td>GO Biological Process</td><td>Catabolic process</td><td>1.9E-13</td><td>PARK7|GCDH|W5PKU3_SHEEP|DNASE2|HNMT|LPL|PRDX2|ZC3H4|GABA</td><td>8.37E-17</td><td>GO:0009056</td></tr>
<tr><td>2416</td><td>216</td><td>GO Biological Process</td><td>Cellular localization</td><td>4.55E-13</td><td>1|NPC1|EXOC7|SNX18|GRN|LAMP1|CETN2|TM9SF3|WDR91|WASHC5|</td><td>2.4E-16</td><td>GO:0051641</td></tr>
<tr><td>2117</td><td>195</td><td>GO Biological Process</td><td>Macromolecule localization</td><td>1.07E-12</td><td>R1B|VCL|FABP5|PALM|FABP4|ATP1B1|TBC1D13|ABCC1|MSR1|FAM91</td><td>6.63E-16</td><td>GO:0033036</td></tr>
<tr><td>1497</td><td>151</td><td>GO Biological Process</td><td>Small molecule metabolic process</td><td>4.09E-12</td><td>FABP5|PLCG2|CYP1B1|ALOX12|MPO|FABP4|TYMS|ATP1B1|PUDP|OAT|</td><td>2.88E-15</td><td>GO:0044281</td></tr>
<tr><td>1675</td><td>159</td><td>GO Biological Process</td><td>Regulation of localization</td><td>7.18E-11</td><td>8|FHL1|SLC30A1|SRI|C1QTNF3|CCT7|ITGB3|VCP|YWHAE|SLC9A3R1|IT</td><td>5.69E-14</td><td>GO:0032879</td></tr>
<tr><td>15933</td><td>897</td><td>GO Biological Process</td><td>Cellular process</td><td>1.02E-10</td><td>ATP6V0A1|NUP210|GP6|RAC1|SMC4|W5NVL4_SHEEP|CAND1|C11orf54</td><td>8.97E-14</td><td>GO:0009987</td></tr>
<tr><td>429</td><td>65</td><td>GO Biological Process</td><td>Endocytosis</td><td>1.15E-10</td><td>|FNBP1|TPCN1|DNAJC13|DAB2|MSR1|GRB2|AP2S1|MFGE8|LMBRD1|</td><td>1.12E-13</td><td>GO:0006897</td></tr>
<tr><td>1701</td><td>158</td><td>GO Biological Process</td><td>Protein localization</td><td>3.24E-10</td><td>PS33A|EXOC6B|SRI|LMBRD1|CSE1L|FAM126A|VCP|STAU1|YWHAE|SLC</td><td>3.6E-13</td><td>GO:0008104</td></tr>
<tr><td>2635</td><td>218</td><td>GO Biological Process</td><td>Regulation of molecular function</td><td>3.24E-10</td><td>0|LRP1|ALDOB|NLRP3|GIT1|NCK1|MMP9|SNX18|GRN|FGD3|WDR91|</td><td>3.43E-13</td><td>GO:0065009</td></tr>
<tr><td>3085</td><td>245</td><td>GO Biological Process</td><td>Regulation of biological quality</td><td>3.54E-10</td><td>ASHC5|PLAU|ALOX15|TOR1A|ACOX1|H6PD|SLC39A7|FABP5|CYP1B1|D</td><td>4.68E-13</td><td>GO:0065008</td></tr>
<tr><td>438</td><td>64</td><td>GO Biological Process</td><td>Regulation of vesicle-mediated transport</td><td>5.24E-10</td><td>M|DNAJC13|UNC13D|DAB2|BIN1-2|PRTN3|CNN2|SNX3|MFGE8|ITGB3|</td><td>7.38E-13</td><td>GO:0060627</td></tr>
<tr><td>5055</td><td>356</td><td>GO Biological Process</td><td>Cellular component organization</td><td>1.51E-09</td><td>RIP10|FLNA|VPS16|SRGN|COG5|W5P5N5_SHEEP|PSMG3|EMC3|NDRG1</td><td>2.27E-12</td><td>GO:0016043</td></tr>
<tr><td>1509</td><td>140</td><td>GO Biological Process</td><td>Organic substance catabolic process</td><td>6.8E-09</td><td>NHLRC3|OAT|PARK7|GCDH|W5PKU3_SHEEP|DNASE2|HNMT|LPL|ZC3H4</td><td>1.08E-11</td><td>GO:1901575</td></tr>
<tr><td>625</td><td>75</td><td>GO Biological Process</td><td>Regulation of cell adhesion</td><td>2.63E-08</td><td>PRDX2|ARHGAP6|RCC2|ITGB3|ITGB2|FES|GPNMB|ST3GAL4|KANK1|RR</td><td>4.59E-11</td><td>GO:0030155</td></tr>
<tr><td>5301</td><td>363</td><td>GO Biological Process</td><td>Cellular component organization or biogenesis</td><td>2.63E-08</td><td>HO2|AVIL|VHL|TRIP10|FLNA|VPS16|SRGN|COG5|W5P5N5_SHEEP|PSM</td><td>4.41E-11</td><td>GO:0071840</td></tr>
<tr><td>1423</td><td>131</td><td>GO Biological Process</td><td>Cellular catabolic process</td><td>4.77E-08</td><td>YP1B1|MPO|NHLRC3|OAT|PARK7|GCDH|W5PKU3_SHEEP|DNASE2|HN</td><td>8.82E-11</td><td>GO:0044248</td></tr>
<tr><td>344</td><td>51</td><td>GO Biological Process</td><td>Regulation of actin filament-based process</td><td>5.39E-08</td><td>VASP|CNN2|GRB2|ARHGAP6|COTL1|SRI|CYFIP2|ITGB3|LOC101105107|</td><td>1.04E-10</td><td>GO:0032970</td></tr>
<tr><td>2016</td><td>169</td><td>GO Biological Process</td><td>Regulation of catalytic activity</td><td>5.72E-08</td><td>HEEP|CHN2|RSU1|PALM|W5PHI7_SHEEP|ALOX12|RGL2|FABP4|TBC1D1</td><td>1.16E-10</td><td>GO:0050790</td></tr>
<tr><td>1340</td><td>125</td><td>GO Biological Process</td><td>Regulation of transport</td><td>5.72E-08</td><td>MFGE8|FHL1|SLC30A1|SRI|C1QTNF3|ITGB3|YWHAE|SLC9A3R1|ITGB2|</td><td>1.17E-10</td><td>GO:0051049</td></tr>
<tr><td>315</td><td>48</td><td>GO Biological Process</td><td>Regulation of actin cytoskeleton organization</td><td>7.25E-08</td><td>SPTAN1|VASP|GRB2|ARHGAP6|COTL1|CYFIP2|ITGB3|LOC101105107|FE</td><td>1.63E-10</td><td>GO:0032956</td></tr>
<tr><td>657</td><td>76</td><td>GO Biological Process</td><td>Membrane organization</td><td>7.25E-08</td><td>EMC1|SNX3|MFGE8|GET3|SLC9A3R1|TRIAP1|SH3TC1|RAB3A|LMNB2|A</td><td>1.6E-10</td><td>GO:0061024</td></tr>
<tr><td>3268</td><td>244</td><td>GO Biological Process</td><td>Regulation of response to stimulus</td><td>8.52E-08</td><td>112936|GIT1|HTRA1|NCK1|CPB2|LOC101120001|LOC101109746|MMP9</td><td>2.03E-10</td><td>GO:0048583</td></tr>
<tr><td>1955</td><td>163</td><td>GO Biological Process</td><td>Regulation of cellular component organization</td><td>0.000000157</td><td>X3|ARHGAP6|RCC2|COTL1|VIM|FKBP4|FHL1|RUFY3|CYFIP2|CCT7|ITG</td><td>3.87E-10</td><td>GO:0051128</td></tr>
<tr><td>878</td><td>91</td><td>GO Biological Process</td><td>Regulation of hydrolase activity</td><td>0.000000177</td><td>RP2|W5PG02_SHEEP|CHN2|RSU1|W5PHI7_SHEEP|ALOX12|RGL2|TBC1</td><td>4.53E-10</td><td>GO:0051336</td></tr>
<tr><td>481</td><td>61</td><td>GO Biological Process</td><td>Regulation of cytoskeleton organization</td><td>0.000000177</td><td>/5PIC9_SHEEP|SPTAN1|VASP|EML2|GRB2|ARHGAP6|COTL1|FKBP4|CYF</td><td>4.58E-10</td><td>GO:0051493</td></tr>
<tr><td>1807</td><td>153</td><td>GO Biological Process</td><td>Immune system process</td><td>0.0000002</td><td>LP|PRDX2|VIM|TBC1D10C|VPS33A|MRC1|ITGB2|W5PSQ7_SHEEP|FES|J</td><td>5.47E-10</td><td>GO:0002376</td></tr>
<tr><td>1580</td><td>137</td><td>GO Biological Process</td><td>Establishment of localization in cell</td><td>0.000000442</td><td>RK7|CCDC22|CNN2|AP2S1|SNX3|SARNP|GET3|TBC1D10C|VPS33A|EXO</td><td>1.24E-09</td><td>GO:0051649</td></tr>
<tr><td>339</td><td>48</td><td>GO Biological Process</td><td>Regulation of supramolecular fiber organization</td><td>0.000000497</td><td>RHGAP6|COTL1|CYFIP2|LOC101105107|FES|INPP5K|KANK1|CCN2|ADD</td><td>1.44E-09</td><td>GO:1902903</td></tr>
<tr><td>506</td><td>61</td><td>GO Biological Process</td><td>Actin cytoskeleton organization</td><td>0.000000891</td><td>A|EPB41L3|CNN2|GRB2|ARHGAP6|TLN1|SLC9A3R1|KANK1|HIP1|ARHG</td><td>2.67E-09</td><td>GO:0030036</td></tr>
<tr><td>723</td><td>77</td><td>GO Biological Process</td><td>Regulation of response to external stimulus</td><td>0.00000117</td><td>W5PIC9_SHEEP|ABCC1|CCL2|EMILIN2|PARK7|PDCD4|PRKD2|LPL|PRDX</td><td>3.62E-09</td><td>GO:0032101</td></tr>
<tr><td>145</td><td>29</td><td>GO Biological Process</td><td>Vacuole organization</td><td>0.00000121</td><td>PC8|GRN|WASHC5|FIG4|GABARAP|VPS33A|ATG7|TCIRG1|GNPTAB|CL</td><td>3.83E-09</td><td>GO:0007033</td></tr>
<tr><td>248</td><td>39</td><td>GO Biological Process</td><td>Regulation of actin filament organization</td><td>0.00000121</td><td>RB2|ARHGAP6|COTL1|CYFIP2|LOC101105107|INPP5K|KANK1|CCN2|A</td><td>3.9E-09</td><td>GO:0110053</td></tr>
<tr><td>768</td><td>80</td><td>GO Biological Process</td><td>Oxoacid metabolic process</td><td>0.00000132</td><td>PSS1|CYP1B1|ALOX12|MPO|FABP4|OAT|PARK7|GCDH|PPA2|LPL|BCAT</td><td>4.41E-09</td><td>GO:0043436</td></tr>
<tr><td>5423</td><td>358</td><td>GO Biological Process</td><td>Positive regulation of biological process</td><td>0.00000148</td><td>C|RAB7B|AVIL|VHL|FLNA|EIPR1|W5P5N5_SHEEP|AGAP2|RIPOR2|GSN|</td><td>5.1E-09</td><td>GO:0048518</td></tr>
<tr><td>2039</td><td>163</td><td>GO Biological Process</td><td>Cellular response to chemical stimulus</td><td>0.00000182</td><td>|CCL2|POSTN|PARK7|PRXL2A|MX1|PDCD4|GSR|SPART|GRB2|PRKD2|L</td><td>6.42E-09</td><td>GO:0070887</td></tr>
<tr><td>548</td><td>63</td><td>GO Biological Process</td><td>Actin filament-based process</td><td>0.00000212</td><td>L3|CNN2|GRB2|ARHGAP6|TLN1|SLC9A3R1|KANK1|HIP1|ARHGAP12|M</td><td>7.64E-09</td><td>GO:0030029</td></tr>
<tr><td>1202</td><td>109</td><td>GO Biological Process</td><td>Regulation of immune system process</td><td>0.0000023</td><td>CCL2|PARK7|PRXL2A|DNASE2|CNN2|PRKD2|SELP|PRDX2|PADI2|TBC1D</td><td>8.5E-09</td><td>GO:0002682</td></tr>
<tr><td>337</td><td>46</td><td>GO Biological Process</td><td>Positive regulation of response to external stimulus</td><td>0.00000232</td><td>36|AIF1|LAMP1|PLCG2|FABP4|ABCC1|EMILIN2|PARK7|PDCD4|PRKD2|</td><td>8.8E-09</td><td>GO:0032103</td></tr>
<tr><td>1160</td><td>106</td><td>GO Biological Process</td><td>Cytoskeleton organization</td><td>0.00000246</td><td>EML2|MYL12A|EPB41L3|CNN2|GRB2|ARHGAP6|GABARAP|VIM|TLN1|V</td><td>9.6E-09</td><td>GO:0007010</td></tr>
<tr><td>182</td><td>32</td><td>GO Biological Process</td><td>Regulation of protein polymerization</td><td>0.00000246</td><td>-2|ADD3|SPTAN1|VASP|EML2|GRB2|COTL1|CYFIP2|FES|KANK1|ADD1|</td><td>9.54E-09</td><td>GO:0032271</td></tr>
<tr><td>1071</td><td>100</td><td>GO Biological Process</td><td>Establishment of protein localization</td><td>0.00000246</td><td>33A|EXOC6B|CSE1L|VCP|ATG7|RAB3A|LAMP2|LOC101122662|AP2A1|P</td><td>9.64E-09</td><td>GO:0045184</td></tr>
<tr><td>2439</td><td>186</td><td>GO Biological Process</td><td>Regulation of signal transduction</td><td>0.00000306</td><td>CYP1B1|PALM|RGL2|DAB2|TBCK|MAP4K2|W5PIC9_SHEEP|PTPN11|CCL</td><td>1.27E-08</td><td>GO:0009966</td></tr>
<tr><td>530</td><td>61</td><td>GO Biological Process</td><td>Regulation of cell activation</td><td>0.00000306</td><td>W5PIC9_SHEEP|CLPTM1|EMILIN2|SELP|PRDX2|TBC1D10C|ITGB2|FES|G</td><td>1.28E-08</td><td>GO:0050865</td></tr>
<tr><td>156</td><td>29</td><td>GO Biological Process</td><td>Regulation of actin polymerization or depolymerization</td><td>0.0000037</td><td>LOX15|ADD3|SPTAN1|VASP|GRB2|COTL1|CYFIP2|KANK1|ADD1|CYFIP1|</td><td>0.000000016</td><td>GO:0008064</td></tr>
<tr><td>1281</td><td>113</td><td>GO Biological Process</td><td>Positive regulation of molecular function</td><td>0.00000404</td><td>4K2|CCL2|PRTN3|PARK7|PRKD2|ARHGAP6|RCC2|ABR|TBC1D10C|CYFIP</td><td>1.78E-08</td><td>GO:0044093</td></tr>
<tr><td>4831</td><td>322</td><td>GO Biological Process</td><td>Positive regulation of cellular process</td><td>0.00000436</td><td>P5N5_SHEEP|AGAP2|RIPOR2|GSN|LMNA|DDAH2|RASGRP4|MAPRE2|P</td><td>1.96E-08</td><td>GO:0048522</td></tr>
</table>

Supplementary Table 11: Comparative functional enrichment analysis of GMØ (monocyte-derived macrophages differentiated with GM-CSF) and MMØ (monocyte-derived macrophages differentiated with M-CSF) relative to Mo (monocytes at 3 hours). Functional enrichment analysis was performed using the StringApp (STRING database) within Cytoscape (version 3.10.3)[1], focusing on Gene Ontology (GO) Biological Process (BP) terms[2,3]. Red-highlighted cells indicate Gene Ontology Biological Process terms shared between GMØ and MMØ compared to Mo, whereas non-highlighted cells represent subtype-specific enrichment.

148	28	GO Biological Process	Phagocytosis	0.00000442	ALOX15|UNC13D|MSR1|PRTN3|CNN2|MFGE8|ITGB3|ITGB2|ARHGAP12	2.03E-08	GO:0006909
2640	197	GO Biological Process	Response to stress	0.00000442	M58|MMP9|AIF1|C8B|ITPR1|ANXA6|CETN2|PLAU|ALOX15|TOR1A|SM	2.03E-08	GO:0006950
742	76	GO Biological Process	Regulation of cell migration	0.00000442	MILIN2|CNN2|PRKD2|SELP|RCC2|PADI2|ITGB3|MOSPD2|LOC101105107	2.09E-08	GO:0030334
1756	143	GO Biological Process	Organic substance transport	0.00000445	|CSE1L|VCP|SLC9A3R1|TRIAP1|MPC2|TCN1|ATG7|RAB3A|LAMP2|LOC1	2.23E-08	GO:0071702
788	79	GO Biological Process	Regulation of cell motility	0.00000496	|EMILIN2|CNN2|PRKD2|SELP|RCC2|PADI2|ITGB3|MOSPD2|LOC1011051	2.53E-08	GO:2000145
1190	106	GO Biological Process	Biological process involved in interspecies interaction between organi	0.00000626	PLCG2|MPO|UNC13D|FABP4|ATP1B1|CCL2|MX1|PDCD4|LPL|PRDX2|SN	3.25E-08	GO:0044419
133	26	GO Biological Process	Receptor-mediated endocytosis	0.00000686	SR1|GRB2|AP2S1|LMBRD1|MRC1|ITGB2|HIP1|AP2A1|SCARB2|W5Q6N	3.63E-08	GO:0006898
754	76	GO Biological Process	Carboxylic acid metabolic process	0.00000716	X1|ASAH1|CYP1B1|ALOX12|FABP4|OAT|PARK7|GCDH|LPL|BCAT2|LTA4	3.85E-08	GO:0019752
8199	499	GO Biological Process	Metabolic process	0.00000735	|RAB11B|SH3PXD2B|CTSD|GBA|CTSC|SLC25A13|LACTB2|ADA|SOD1-2|	4.07E-08	GO:0008152
1758	142	GO Biological Process	Positive regulation of response to stimulus	0.00000735	AR2|PDCD4|CCDC22|SHOC2|ATP6V0C|PRKD2|LPL|SELP|PRDX2|CYFIP2	4.02E-08	GO:0048584
357	46	GO Biological Process	Regulation of protein-containing complex assembly	0.00000748	N11|SPTAN1|VASP|PARK7|EML2|GRB2|SELP|COTL1|CYFIP2|VCP|FES|K	4.22E-08	GO:0043254
659	69	GO Biological Process	Positive regulation of transport	0.00000771	CCL2|PARK7|MFGE8|FHL1|SRI|C1QTNF3|YWHAE|ITGB2|MPC2|INPP5K	4.42E-08	GO:0051050
155	28	GO Biological Process	Regulation of endocytosis	0.00000827	DX15|TOR1A|PLCG2|DAB2|BIN1-2|ITGB3|HIP1|LRPAP1|PPP3CA|ANKRD	4.81E-08	GO:0030100
397	49	GO Biological Process	Regulation of anatomical structure size	0.00000873	SPTAN1|VASP|SPART|GRB2|COTL1|CYFIP2|SLC9A3R1|KANK1|PRKG1|A	5.15E-08	GO:0090066
3265	231	GO Biological Process	Organelle organization	0.0000101	|AIF1|WDR1|GRN|FGD3|CETN2|FMNL2|WASHC5|TOR1A|TOR1B|SMC1	6.06E-08	GO:0006996
821	80	GO Biological Process	Regulation of locomotion	0.0000103	MILIN2|CNN2|PRKD2|SELP|RCC2|PADI2|ITGB3|MOSPD2|LOC101105107	6.27E-08	GO:0040012
1757	141	GO Biological Process	Phosphorus metabolic process	0.0000105	DP|PTPN11|PARK7|GCDH|FIG4|PPA2|PRKD2|ATP6V1B2|NADK2|ABR|PI	0.000000065	GO:0006793
31	13	GO Biological Process	Antigen processing and presentation of exogenous peptide antigen	0.0000128	P2|LOC101109746|IFI30|W5Q2C4_SHEEP|W5Q2V1_SHEEP|W5Q3J8_SHE	8.03E-08	GO:0002478
204	32	GO Biological Process	Wound healing	0.000016	TOR1A|HPS5|F5|CNN2|ITGB3|RAB3A|MERTK|ANXA5|RAP2B|ADAM17|	0.000000101	GO:0042060
1237	107	GO Biological Process	Intracellular transport	0.0000171	CCDC22|AP2S1|SNX3|SARNP|GET3|TBC1D10C|VPS33A|EXOC6B|CSE1L|	0.00000011	GO:0046907
1161	102	GO Biological Process	Homeostatic process	0.0000172	6V1B2|LPL|PRDX2|VPS33A|SLC30A1|PRDX6|C1QTNF3|STAU1|SLC9A3R1	0.000000112	GO:0042592
1223	106	GO Biological Process	Regulation of cell death	0.0000175	|ASAH1|PLCG2|CYP1B1|ALOX12|RGL2|DAB2|BIN1-2|CCL2|EMILIN2|PA	0.000000116	GO:0010941
864	82	GO Biological Process	Carbohydrate derivative metabolic process	0.0000178	1102454|GM2A|PAPSS1|H6PD|TYMS|ATP1B1|IDS|PARK7|GCDH|W5PKU	0.000000119	GO:1901135
449	52	GO Biological Process	Endomembrane system organization	0.0000189	LB|DNAJC13|BIN1-2|ESYT2|EMC1|SNX3|PI4K2A|GET3|SLC9A3R1|SH3TC	0.000000128	GO:0010256
2747	199	GO Biological Process	Regulation of cell communication	0.0000198	15|CHN2|FABP5|PLCG2|CYP1B1|PALM|RGL2|DAB2|TBCK|MAP4K2|W5	0.000000136	GO:0010646
799	77	GO Biological Process	Organophosphate metabolic process	0.0000242	|PAPSS1|H6PD|FABP5|PLCG2|TYMS|ATP1B1|PUDP|PARK7|GCDH|FIG4	0.000000168	GO:0019637
757	74	GO Biological Process	Regulation of cellular localization	0.0000252	|C1QTNF3|CCT7|ITGB3|VCP|YWHAE|MPC2|SH3TC1|INPP5K|RNASE4|E	0.000000178	GO:0060341
146	26	GO Biological Process	Carbohydrate derivative catabolic process	0.0000257	C101112162|DCTPP1|FUCA1|GM2A|IDS|W5PKU3_SHEEP|ALDH1A1|CEN	0.000000184	GO:1901136
993	90	GO Biological Process	Protein transport	0.0000263	CP|ATG7|RAB3A|LAMP2|LOC101122662|AP2A1|PPP3CA|HSPB1|RAB3IL	0.00000019	GO:0015031
257	36	GO Biological Process	Response to wounding	0.0000277	|TOR1A|PLCG2|HPS5|F5|CNN2|ITGB3|RAB3A|MERTK|ANXA5|RAP2B|A	0.000000205	GO:0009611
2763	199	GO Biological Process	Regulation of signaling	0.0000277	15|CHN2|FABP5|PLCG2|CYP1B1|PALM|RGL2|DAB2|TBCK|MAP4K2|W5	0.000000202	GO:0023051
138	25	GO Biological Process	Regulation of actin filament polymerization	0.0000316	CK1|ALOX15|ADD3|SPTAN1|VASP|GRB2|COTL1|CYFIP2|KANK1|ADD1|C	0.000000237	GO:0030833
420	49	GO Biological Process	Carbohydrate metabolic process	0.0000319	BP5|W5PKU3_SHEEP|GOT1|INPP5K|ST3GAL4|SYNJ1|LANCL2|PFKP|RGN|	0.000000242	GO:0005975
1736	137	GO Biological Process	Phosphate-containing compound metabolic process	0.0000327	ARK7|GCDH|FIG4|PPA2|PRKD2|ATP6V1B2|NADK2|ABR|PI4K2A|AK1|PD	0.000000252	GO:0006796
382	46	GO Biological Process	Regulation of cell-cell adhesion	0.0000327	SELP|PRDX2|ITGB2|GPNMB|ST3GAL4|PRKG1|PPP3CA|LGALS1|SASH3|P	0.00000025	GO:0022407
308	40	GO Biological Process	Regulation of cellular component size	0.0000339	D3|SPTAN1|VASP|SPART|GRB2|COTL1|CYFIP2|SLC9A3R1|KANK1|ADD1	0.000000266	GO:0032535
461	52	GO Biological Process	Nucleobase-containing small molecule metabolic process	0.000034	ALDOB|GARS1|SMPDL3A|NUDT14|LOC101102454|PAPSS1|TYMS|ATP1E	0.00000027	GO:0055086
12208	693	GO Biological Process	Biological regulation	0.0000348	CLEC4A|EPB41|STAB1|AHSA1|SPP1|PPIL4|IGHM|FSCN1|W5NYC7_SHEEP	0.000000279	GO:0065007
238	34	GO Biological Process	Regulation of body fluid levels	0.0000365	S5|EMILIN2|F5|PRDX2|ITGB3|INPP5K|ST3GAL4|PRKG1|PPP3CA|MERTK	0.000000295	GO:0050878
1990	152	GO Biological Process	Response to organic substance	0.0000396	1|CCL2|POSTN|PARK7|MX1|PDCD4|SPART|GRB2|PRKD2|LPL|PRDX2|PA	0.000000324	GO:0010033
36	13	GO Biological Process	Membrane lipid catabolic process	0.0000396	J1|HEXB|LOC101112162|SGPL1|FUCA1|SMPDL3A|GM2A|ASAH1|CYP1B	0.000000325	GO:0046466
326	41	GO Biological Process	Regulation of GTPase activity	0.0000484	HGAP9|RASA2|SNX18|RASGRP2|W5PG02_SHEEP|CHN2|RSU1|RGL2|TBC	0.000000405	GO:0043087
1353	112	GO Biological Process	Positive regulation of cell communication	0.0000504	|CCL2|PARK7|CCAR2|PDCD4|CCDC22|SHOC2|ATP6V0C|PRKD2|SELP|PR	0.000000427	GO:0010647
496	54	GO Biological Process	Regulation of leukocyte activation	0.0000516	UNC13D|W5PIC9_SHEEP|CLPTM1|PRDX2|TBC1D10C|ITGB2|FES|GPNMB	0.000000441	GO:0002694
1003	89	GO Biological Process	Organonitrogen compound catabolic process	0.0000598	BA|GM2A|TOR1A|NPEPPS|ASAH1|DDI2|IDS|NHLRC3|OAT|HNMT|VCP|E	0.000000516	GO:1901565
1127	97	GO Biological Process	Regulation of programmed cell death	0.0000637	RN|PEA15|ASAH1|PLCG2|CYP1B1|ALOX12|RGL2|DAB2|BIN1-2|CCL2|EM	0.000000556	GO:0043067
1362	112	GO Biological Process	Positive regulation of signaling	0.000065	|CCL2|PARK7|CCAR2|PDCD4|CCDC22|SHOC2|ATP6V0C|PRKD2|SELP|PR	0.000000577	GO:0023056
739	71	GO Biological Process	Cellular response to oxygen-containing compound	0.000065	D4|GRB2|LPL|PRDX2|VIM|MRC1|VCP|SLC9A3R1|GOT1|FES|ALDH1A1|	0.000000572	GO:1901701
1222	103	GO Biological Process	Positive regulation of signal transduction	0.0000662	32|MAP4K2|PTPN11|CCL2|PARK7|CCAR2|PDCD4|CCDC22|SHOC2|ATP6V	0.0000006	GO:0009967
1037	91	GO Biological Process	Regulation of organelle organization	0.0000662	PA1A-2|ADD3|W5PIC9_SHEEP|SPTAN1|VASP|FEZ2|EML2|GRB2|ARHGA	0.000000594	GO:0033043
320	40	GO Biological Process	Response to inorganic substance	0.0000696	CYP1B1|DDI2|MPO|FABP4|PARK7|PRDX2|GSS|SLC30A1|VCP|LGMN|PPF	0.000000644	GO:0010035
1100	95	GO Biological Process	Regulation of apoptotic process	0.0000696	MP9|GRN|PEA15|CYP1B1|ALOX12|RGL2|DAB2|BIN1-2|CCL2|EMILIN2|	0.000000638	GO:0042981
107	21	GO Biological Process	Lysosomal transport	0.0000735	MP1|HSPA1A-2|BIN1-2|VPS33A|VCP|LAMP2|AP3D1|ZFYVE16|GNPTAB|	0.000000692	GO:0007041
54	15	GO Biological Process	Regulation of actin filament depolymerization	0.0000735	GSN|SCIN|WDR1|ADD3|SPTAN1|ADD1|DSTN|LIMA1|TWF1|PLEK|CAPG	0.000000686	GO:0030834
261	35	GO Biological Process	Actin filament organization	0.0000794	AIF1|WDR1|WASHC5|VASP|ARHGAP6|HIP1|ARHGAP12|MYO1G|PLEC|P	0.000000755	GO:0007015
261	35	GO Biological Process	Lipid catabolic process	0.0000794	BHD12|PLBD2|SMPDL3A|HSD17B6|GM2A|ACOX1|ASAH1|PLCG2|CYP1B	0.000000755	GO:0016042

Supplementary Table 11: Comparative functional enrichment analysis of GMØ (monocyte-derived macrophages differentiated with GM-CSF) and MMØ (monocyte-derived macrophages differentiated with M-CSF) relative to Mo (monocytes at 3 hours). Functional enrichment analysis was performed using the StringApp (STRING database) within Cytoscape (version 3.10.3)[1], focusing on Gene Ontology (GO) Biological Process (BP) terms[2,3]. Red-highlighted cells indicate Gene Ontology Biological Process terms shared between GMØ and MMØ compared to Mo, whereas non-highlighted cells represent subtype-specific enrichment.

<table>
<tr><td>80</td><td>18</td><td>GO Biological Process</td><td>Integrin-mediated signaling pathway</td><td>0.0000807</td><td>ITGAL|ITGAM|PTPN11|RCC2|ITGB3|TLN1|ITGB2|CCN2|ITFG1|ITGAV|IT</td><td>0.000000782</td><td>GO:0007229</td></tr>
<tr><td>63</td><td>16</td><td>GO Biological Process</td><td>Antigen processing and presentation of peptide antigen</td><td>0.0000832</td><td>1109746|TAPBP|W5PJ11_SHEEP|IFI30|W5Q2C4_SHEEP|W5Q2V1_SHEEP</td><td>0.000000813</td><td>GO:0048002</td></tr>
<tr><td>1391</td><td>113</td><td>GO Biological Process</td><td>Nitrogen compound transport</td><td>0.0000892</td><td>3R1|TCN1|ATG7|RAB3A|LAMP2|LOC101122662|AP2A1|PPP3CA|HSPB1</td><td>0.000000888</td><td>GO:0071705</td></tr>
<tr><td>1049</td><td>91</td><td>GO Biological Process</td><td>Response to other organism</td><td>0.0000931</td><td>58|AIF1|C8B|PLCG2|MPO|UNC13D|FABP4|ATP1B1|CCL2|MX1|PDCD4|</td><td>0.000000935</td><td>GO:0051707</td></tr>
<tr><td>161</td><td>26</td><td>GO Biological Process</td><td>Macroautophagy</td><td>0.0000952</td><td>APPC8|NPC1|GABARAP|VPS33A|VCP|ATG7|LAMP2|WDR81|TCIRG1|SN</td><td>0.000000964</td><td>GO:0016236</td></tr>
<tr><td>500</td><td>53</td><td>GO Biological Process</td><td>Cellular homeostasis</td><td>0.00011</td><td>|ATP1B1|CCDC22|GSR|ATP6V1B2|PRDX2|VPS33A|SLC30A1|PRDX6|C1C</td><td>0.00000118</td><td>GO:0019725</td></tr>
<tr><td>367</td><td>43</td><td>GO Biological Process</td><td>Positive regulation of cell adhesion</td><td>0.00011</td><td>2|PRKD2|SELP|ITGB3|ITGB2|ST3GAL4|RRAS|PPP3CA|LGALS1|SASH3|PP</td><td>0.00000118</td><td>GO:0045785</td></tr>
<tr><td>1103</td><td>94</td><td>GO Biological Process</td><td>Response to biotic stimulus</td><td>0.00012</td><td>1|C8B|SMC1A|PLCG2|MPO|UNC13D|FABP4|ATP1B1|CCL2|MX1|PDCD4</td><td>0.00000125</td><td>GO:0009607</td></tr>
<tr><td>16</td><td>9</td><td>GO Biological Process</td><td>Glycolipid catabolic process</td><td>0.00012</td><td>GLA|GBA|NEU1|HEXB|LOC101112162|FUCA1|GM2A|NAGA|HEXA</td><td>0.00000126</td><td>GO:0019377</td></tr>
<tr><td>517</td><td>54</td><td>GO Biological Process</td><td>Response to bacterium</td><td>0.00013</td><td>AP2|GIT1|PLCG2|MPO|FABP4|CCL2|PDCD4|LPL|PRDX2|SNX3|VIM|MR</td><td>0.00000141</td><td>GO:0009617</td></tr>
<tr><td>674</td><td>65</td><td>GO Biological Process</td><td>Regulation of protein localization</td><td>0.00014</td><td>T7|ITGB3|VCP|YWHAE|MPC2|SH3TC1|INPP5K|RNASE4|ERGIC3|PIN1|K</td><td>0.00000154</td><td>GO:0032880</td></tr>
<tr><td>94</td><td>19</td><td>GO Biological Process</td><td>Blood coagulation</td><td>0.00015</td><td>T3|MPIG6B|CPB2|HPS5|F5|ITGB3|MERTK|ANXA5|RAP2B|PROC|GNAS|</td><td>0.00000158</td><td>GO:0007596</td></tr>
<tr><td>157</td><td>25</td><td>GO Biological Process</td><td>Cell-substrate adhesion</td><td>0.00017</td><td>ITGAL|ITGAM|RCC2|ITGB3|ITGB2|MYO1G|CCN2|MERTK|ITGAV|FGG|F</td><td>0.00000194</td><td>GO:0031589</td></tr>
<tr><td>468</td><td>50</td><td>GO Biological Process</td><td>Positive regulation of hydrolase activity</td><td>0.00017</td><td>GRP2|CHN2|RSU1|ALOX12|RGL2|TBC1D13|TBCK|CCL2|PRTN3|PRKD2|</td><td>0.00000192</td><td>GO:0051345</td></tr>
<tr><td>753</td><td>70</td><td>GO Biological Process</td><td>Cell migration</td><td>0.00018</td><td>LA|CYP1B1|PTPN11|CCL2|PRTN3|CNN2|SELP|ITGB3|SLC9A3R1|ITGB2|R</td><td>0.00000203</td><td>GO:0016477</td></tr>
<tr><td>1562</td><td>122</td><td>GO Biological Process</td><td>Cellular response to organic substance</td><td>0.00018</td><td>CCL2|PARK7|MX1|PDCD4|SPART|GRB2|PRKD2|LPL|PADI2|VIM|ITGB3|</td><td>0.000002</td><td>GO:0071310</td></tr>
<tr><td>4510</td><td>291</td><td>GO Biological Process</td><td>Negative regulation of biological process</td><td>0.0002</td><td>6|ABI3|CSF1R|RASA2|W5P9M9_SHEEP|ACOT8|LOC101105044|CTSA|ST</td><td>0.00000224</td><td>GO:0048519</td></tr>
<tr><td>240</td><td>32</td><td>GO Biological Process</td><td>Regulation of cell morphogenesis</td><td>0.00021</td><td>1|FMNL2|PALM|UNC13D|CCL2|MYL12B|RCC2|ITGB3|LOC101105107|S</td><td>0.00000248</td><td>GO:0022604</td></tr>
<tr><td>8</td><td>7</td><td>GO Biological Process</td><td>Glycoside catabolic process</td><td>0.00022</td><td>GUSB|FUCA2|GLA|FUCA1|W5PKU3_SHEEP|NAGA|ABHD10</td><td>0.00000253</td><td>GO:0016139</td></tr>
<tr><td>878</td><td>78</td><td>GO Biological Process</td><td>Positive regulation of cellular component organization</td><td>0.00022</td><td>X18|GRN|ALOX15|TOR1A|HSPA1A-2|PLCG2|PALM|DAB2|VASP|PARK7</td><td>0.00000257</td><td>GO:0051130</td></tr>
<tr><td>70</td><td>16</td><td>GO Biological Process</td><td>Lysosome organization</td><td>0.00023</td><td>|LAMTOR1|GBA|RAB7B|ACP2|GRN|WASHC5|VPS33A|GNPTAB|CLN5|TP</td><td>0.00000266</td><td>GO:0007040</td></tr>
<tr><td>408</td><td>45</td><td>GO Biological Process</td><td>Nucleoside phosphate metabolic process</td><td>0.00024</td><td>3A|NUDT14|LOC101102454|PAPSS1|TYMS|ATP1B1|PUDP|PARK7|GCDH</td><td>0.00000293</td><td>GO:0006753</td></tr>
<tr><td>734</td><td>68</td><td>GO Biological Process</td><td>Positive regulation of immune system process</td><td>0.00025</td><td>HEEP|PARK7|PRKD2|SELP|ITGB3|MOSPD2|ITGB2|FES|MS4A1|LGMN|AN</td><td>0.00000317</td><td>GO:0002684</td></tr>
<tr><td>140</td><td>23</td><td>GO Biological Process</td><td>Vacuolar transport</td><td>0.00025</td><td>B|GRN|LAMP1|HSPA1A-2|BIN1-2|VPS33A|VCP|LAMP2|AP3D1|ZFYVE16</td><td>0.00000311</td><td>GO:0007034</td></tr>
<tr><td>31</td><td>11</td><td>GO Biological Process</td><td>Sphingolipid catabolic process</td><td>0.00025</td><td>GBA|NEU1|HEXB|LOC101112162|SGPL1|SMPDL3A|GM2A|ASAH1|NAGA</td><td>0.00000302</td><td>GO:0030149</td></tr>
<tr><td>2159</td><td>157</td><td>GO Biological Process</td><td>Regulation of multicellular organismal process</td><td>0.00025</td><td>4|SPART|PRKD2|LPL|PRDX2|SRI|C1QTNF3|ITGB3|SLC9A3R1|FES|JARID</td><td>0.00000309</td><td>GO:0051239</td></tr>
<tr><td>2276</td><td>164</td><td>GO Biological Process</td><td>Regulation of protein metabolic process</td><td>0.00025</td><td>2|CCDC22|PRKD2|MIF4GD|SNX3|SAE1|GABARAP|VIM|LOC101123010|C</td><td>0.00000296</td><td>GO:0051246</td></tr>
<tr><td>130</td><td>22</td><td>GO Biological Process</td><td>Regulation of cell shape</td><td>0.00026</td><td>CSF1R|PALM2AKAP2|WDR1|FMNL2|PALM|CCL2|MYL12B|SLC9A3R1|FE</td><td>0.00000331</td><td>GO:0008360</td></tr>
<tr><td>1368</td><td>109</td><td>GO Biological Process</td><td>Regulation of intracellular signal transduction</td><td>0.00026</td><td>7_SHEEP|PARK7|CCAR2|PDCD4|CCDC22|SHOC2|GRB2|PRKD2|SELP|PR</td><td>0.00000323</td><td>GO:1902531</td></tr>
<tr><td>1322</td><td>106</td><td>GO Biological Process</td><td>Negative regulation of response to stimulus</td><td>0.00027</td><td>P|PARK7|CCAR2|CCDC22|SPART|PRDX2|PADI2|TBC1D10C|C1QTNF3|V</td><td>0.00000344</td><td>GO:0048585</td></tr>
<tr><td>25</td><td>10</td><td>GO Biological Process</td><td>Antigen processing and presentation of exogenous peptide antigen vi</td><td>0.00028</td><td>|LOC101109746|IFI30|W5Q2C4_SHEEP|W5Q2V1_SHEEP|W5Q3J8_SHEEP</td><td>0.0000036</td><td>GO:0019886</td></tr>
<tr><td>947</td><td>82</td><td>GO Biological Process</td><td>Positive regulation of catalytic activity</td><td>0.00028</td><td>CCL2|PRTN3|PARK7|PRKD2|ARHGAP6|RCC2|ABR|TBC1D10C|CYFIP2|IT</td><td>0.00000352</td><td>GO:0043085</td></tr>
<tr><td>386</td><td>43</td><td>GO Biological Process</td><td>Regulation of lymphocyte activation</td><td>0.0003</td><td>R|W5PIC9_SHEEP|CLPTM1|PRDX2|TBC1D10C|GPNMB|BANK1|PPP3CA|</td><td>0.00000385</td><td>GO:0051249</td></tr>
<tr><td>566</td><td>56</td><td>GO Biological Process</td><td>Cellular response to cytokine stimulus</td><td>0.00031</td><td>R|CD74|CSF2RA|LOC101112936|AIF1|ALOX15|ASAH1|FABP4|CCL2|MX</td><td>0.00000408</td><td>GO:0071345</td></tr>
<tr><td>713</td><td>66</td><td>GO Biological Process</td><td>Regulation of anatomical structure morphogenesis</td><td>0.00035</td><td>2|CCL2|EMILIN2|MYL12B|SPART|PRKD2|RCC2|RUFY3|ITGB3|LOC1011</td><td>0.00000454</td><td>GO:0022603</td></tr>
<tr><td>14</td><td>8</td><td>GO Biological Process</td><td>Glycosphingolipid catabolic process</td><td>0.00035</td><td>GLA|GBA|NEU1|HEXB|LOC101112162|GM2A|NAGA|HEXA</td><td>0.00000459</td><td>GO:0046479</td></tr>
<tr><td>156</td><td>24</td><td>GO Biological Process</td><td>Cellular modified amino acid metabolic process</td><td>0.00039</td><td>2|ASS1|PARK7|GSR|GSS|ALDH9A1|MTHFD2|CTSB|SLC27A1|CPT1A|HA</td><td>0.00000524</td><td>GO:0006575</td></tr>
<tr><td>20</td><td>9</td><td>GO Biological Process</td><td>Ceramide catabolic process</td><td>0.00039</td><td>GLA|GBA|NEU1|HEXB|LOC101112162|GM2A|ASAH1|NAGA|HEXA</td><td>0.00000521</td><td>GO:0046514</td></tr>
<tr><td>103</td><td>19</td><td>GO Biological Process</td><td>Positive regulation of inflammatory response</td><td>0.00039</td><td>REM2|CTSC|LOC101114535|STAP1|PLCG2|FABP4|ABCC1|PARK7|PDCD</td><td>0.00000513</td><td>GO:0050729</td></tr>
<tr><td>202</td><td>28</td><td>GO Biological Process</td><td>Endosomal transport</td><td>0.0004</td><td>5A|SNX6|SNX18|WASHC5|CCDC22|SNX3|TBC1D10C|VCP|CCDC93|AP3D</td><td>0.00000539</td><td>GO:0016197</td></tr>
<tr><td>179</td><td>26</td><td>GO Biological Process</td><td>Cellular lipid catabolic process</td><td>0.0004</td><td>L1|ACOT8|FUCA1|ABHD12|PLBD2|SMPDL3A|GM2A|ACOX1|ASAH1|PL</td><td>0.00000548</td><td>GO:0044242</td></tr>
<tr><td>288</td><td>35</td><td>GO Biological Process</td><td>Positive regulation of cell activation</td><td>0.00041</td><td>LAMP1|ITGAM|W5PIC9_SHEEP|SELP|ITGB2|PPP3CA|LGALS1|SASH3|W</td><td>0.00000558</td><td>GO:0050867</td></tr>
<tr><td>288</td><td>35</td><td>GO Biological Process</td><td>Regulation of leukocyte cell-cell adhesion</td><td>0.00041</td><td>6|AIF1|SELP|PRDX2|ITGB2|GPNMB|ST3GAL4|PPP3CA|LGALS1|SASH3|</td><td>0.00000558</td><td>GO:1903037</td></tr>
<tr><td>749</td><td>68</td><td>GO Biological Process</td><td>Cell adhesion</td><td>0.00042</td><td>TP1B1|POSTN|SELP|RCC2|MFGE8|CYFIP2|ITGB3|TLN1|ITGB2|FES|GPN</td><td>0.00000592</td><td>GO:0007155</td></tr>
<tr><td>839</td><td>74</td><td>GO Biological Process</td><td>Regulation of cellular component biogenesis</td><td>0.00042</td><td>D3|PTPN11|SPTAN1|VASP|PARK7|FEZ2|EML2|GRB2|SELP|ARHGAP6|R</td><td>0.00000591</td><td>GO:0044087</td></tr>
<tr><td>947</td><td>81</td><td>GO Biological Process</td><td>Negative regulation of molecular function</td><td>0.00044</td><td>4|DAB2|BIN1-2|PARK7|CCAR2|CCDC22|PRDX2|RCC2|LOC101123010|CS</td><td>0.00000617</td><td>GO:0044092</td></tr>
<tr><td>58</td><td>14</td><td>GO Biological Process</td><td>Actin polymerization or depolymerization</td><td>0.00045</td><td>PH2|AVIL|GSN|GAS7|SCIN|AIF1|WDR1|WASHC5|VASP|DSTN|TWF1|DL</td><td>0.0000065</td><td>GO:0008154</td></tr>
<tr><td>42</td><td>12</td><td>GO Biological Process</td><td>Negative regulation of actin filament depolymerization</td><td>0.00048</td><td>N1|AVIL|GSN|SCIN|ADD3|SPTAN1|ADD1|LIMA1|TWF1|CAPG|EPS8|TM</td><td>0.00000698</td><td>GO:0030835</td></tr>
<tr><td>86</td><td>17</td><td>GO Biological Process</td><td>Regulation of phagocytosis</td><td>0.00049</td><td>|LMAN2|STAP1|SIRPA|ALOX15|PLCG2|PRTN3|CNN2|SNX3|MFGE8|MER</td><td>0.00000718</td><td>GO:0050764</td></tr>
<tr><td>1155</td><td>94</td><td>GO Biological Process</td><td>Lipid metabolic process</td><td>0.0005</td><td>BP4|PTPN11|GCDH|FIG4|LPL|PI4K2A|FAM126A|BCAT2|LOC101110434</td><td>0.00000732</td><td>GO:0006629</td></tr>
<tr><td>623</td><td>59</td><td>GO Biological Process</td><td>Response to cytokine</td><td>0.00053</td><td>74|CSF2RA|LOC101112936|CXCL16|AIF1|ALOX15|ASAH1|FABP4|CCL2|</td><td>0.00000778</td><td>GO:0034097</td></tr>
<tr><td>581</td><td>56</td><td>GO Biological Process</td><td>Regulation of cytokine production</td><td>0.00056</td><td>T1|AIF1|PLCG2|CYP1B1|PTPN11|W5PJN7_SHEEP|PARK7|PDCD4|PRKD2</td><td>0.00000828</td><td>GO:0001817</td></tr>
<tr><td>256</td><td>32</td><td>GO Biological Process</td><td>Regulation of inflammatory response</td><td>0.00056</td><td>C1|NLRP3|GIT1|GRN|ALOX15|PLCG2|FABP4|W5PIC9_SHEEP|ABCC1|PA</td><td>0.0000083</td><td>GO:0050727</td></tr>
<tr><td>6572</td><td>397</td><td>GO Biological Process</td><td>Cellular metabolic process</td><td>0.00058</td><td>BP|NEU1|NAMPT|SOD2|VAC14|INPPL1|HEXB|RNF13|LOC101112162|</td><td>0.00000865</td><td>GO:0044237</td></tr>
<tr><td>78</td><td>16</td><td>GO Biological Process</td><td>Positive regulation of endocytosis</td><td>0.00059</td><td>5P7F8_SHEEP|STAP1|LRP1|TOR1A|PLCG2|DAB2|HIP1|PPP3CA|LOC101</td><td>0.00000884</td><td>GO:0045807</td></tr>
<tr><td>401</td><td>43</td><td>GO Biological Process</td><td>Nucleotide metabolic process</td><td>0.0006</td><td>1|LOC101102454|PAPSS1|TYMS|ATP1B1|PUDP|PARK7|GCDH|ATP6V1B</td><td>0.00000915</td><td>GO:0009117</td></tr>
</table>

Supplementary Table 11: Comparative functional enrichment analysis of GMØ (monocyte-derived macrophages differentiated with GM-CSF) and MMØ (monocyte-derived macrophages differentiated with M-CSF) relative to Mo (monocytes at 3 hours). Functional enrichment analysis was performed using the StringApp (STRING database) within Cytoscape (version 3.10.3)[1], focusing on Gene Ontology (GO) Biological Process (BP) terms[2,3]. Red-highlighted cells indicate Gene Ontology Biological Process terms shared between GMØ and MMØ compared to Mo, whereas non-highlighted cells represent subtype-specific enrichment.

760	68	GO Biological Process	Chemical homeostasis	0.0006	6V1B2|LPL|VPS33A|SLC30A1|C1QTNF3|STAU1|SLC9A3R1|GOT1|INPP5K	0.00000921	GO:0048878
175	25	GO Biological Process	Regulation of leukocyte migration	0.00069	V5P6T0_SHEEP|CSF1R|STAP1|CD74|AIF1|W5PIC9_SHEEP|CCL2|CNN2|SE	0.0000107	GO:0002685
99	18	GO Biological Process	Cell-matrix adhesion	0.00069	3|W5P7F8_SHEEP|ITGAL|ITGAM|RCC2|ITGB3|ITGB2|CCN2|ITGAV|FGG|	0.0000107	GO:0007160
70	15	GO Biological Process	Negative regulation of protein polymerization	0.00069	GSN|SCIN|ADD3|SPTAN1|EML2|KANK1|ADD1|TBCD|SSH1|TWF1|CAPG	0.0000107	GO:0032272
351	39	GO Biological Process	Lipid localization	0.00073	FABP5|FABP4|ABCC1|MSR1|ESYT2|TRIAP1|ENPP1|OSBPL8|CLPTM1L|SL	0.0000113	GO:0010876
236	30	GO Biological Process	Adaptive immune response	0.00075	D|FES|W5PWS0_SHEEP|MYO1G|W5PYS8_SHEEP|TCIRG1|W5Q2C4_SHEE	0.0000117	GO:0002250
392	42	GO Biological Process	Organic hydroxy compound metabolic process	0.00075	Y|PDXK|GOT1|LOC101110434|ALDH1A1|INPP5K|SYNJ1|W5PVY6_SHEEP|	0.0000118	GO:1901615
90	17	GO Biological Process	Antigen processing and presentation	0.00076	101109746|TAPBP|W5PJ11_SHEEP|IFI30|W5Q2C4_SHEEP|W5Q2V1_SHE	0.0000121	GO:0019882
37	11	GO Biological Process	Actin filament capping	0.00077	PTBN1|AVIL|GSN|SCIN|ADD3|SPTAN1|ADD1|TWF1|CAPG|EPS8|TMOD	0.0000123	GO:0051693
1062	87	GO Biological Process	Regulation of response to stress	0.00081	ABP4|MAP4K2|W5PIC9_SHEEP|ABCC1|EMILIN2|PARK7|CCAR2|DEK|PDC	0.0000129	GO:0080134
144	22	GO Biological Process	Response to toxic substance	0.00087	14535|SOD2|ESD|CYP1B1|AHR|MPO|PARK7|PRXL2A|GSR|PRDX2|SLC3	0.0000141	GO:0009636
316	36	GO Biological Process	Small molecule catabolic process	0.00092	T|PARK7|GCDH|BCAT2|GOT1|INPP5K|SYNJ1|PFKP|W5PY08_SHEEP|OCR	0.000015	GO:0044282
179	25	GO Biological Process	Leukocyte migration	0.00092	238|W5P3V8_SHEEP|ITGAL|CXCL16|MMP9|WDR1|CCL2|PRTN3|CNN2|	0.000015	GO:0050900
439	45	GO Biological Process	Organelle localization	0.00098	|FAM91A1|ESYT2|VPS33A|EXOC6B|SLC9A3R1|RAB3A|CLASP1|LMNB2|	0.0000161	GO:0051640
12	7	GO Biological Process	pH reduction	0.001	ATP6V0A1|ATP6V0D1|ATP6V1B2|ATP6V1E1|TCIRG1|ATP6V1C1|ATP6V1A	0.0000167	GO:0045851
83	16	GO Biological Process	Regulation of receptor-mediated endocytosis	0.001	SHEEP|PLCG2|DAB2|ITGB3|HIP1|LRPAP1|ANKRD13A|LOC101113728|CE	0.0000174	GO:0048259
55	13	GO Biological Process	Regulation of coagulation	0.001	2|PLAU|ALOX12|EMILIN2|PRDX2|ST3GAL4|PRKG1|ANXA5|PROC|FGG|F	0.0000168	GO:0050818
64	14	GO Biological Process	Regulation of calcium-mediated signaling	0.001	R1|PLCG2|W5PIC9_SHEEP|W5PJN7_SHEEP|TBC1D10C|RGN|P2RX4|W5Q	0.0000171	GO:0050848
412	43	GO Biological Process	Regulation of peptidase activity	0.001	P9M9_SHEEP|LOC101105044|MMP9|GRN|W5PHI7_SHEEP|ALOX12|BIN1	0.0000167	GO:0052547
204	27	GO Biological Process	Regulation of leukocyte proliferation	0.001	K1|LOC101109746|AIF1|AHR|W5PIC9_SHEEP|CNN2|GPNMB|PPP3CA|SA	0.0000167	GO:0070663
7705	453	GO Biological Process	Organic substance metabolic process	0.0011	RUSC1|LOC101114535|AHCYL2|RPS6KA1|IVNS1ABP|PEPD|NEU1|NAMP	0.0000195	GO:0071704
256	31	GO Biological Process	Cellular amino acid metabolic process	0.0012	EEP|PCBD1|MARS1|GCLC|SDS|ASS1|GARS1|OAT|GCDH|BCAT2|GOT1|	0.00002	GO:0006520
458	46	GO Biological Process	Localization within membrane	0.0012	1|CCDC22|SNX3|GET3|EXOC6B|FAM126A|CCDC93|SLC9A3R1|ITGB2|IN	0.0000208	GO:0051668
1048	85	GO Biological Process	Negative regulation of signal transduction	0.0013	DC22|SPART|PRDX2|PADI2|TBC1D10C|C1QTNF3|VCP|SLC9A3R1|INPP5K	0.0000229	GO:0009968
233	29	GO Biological Process	Vesicle organization	0.0013	C5|DNAJC13|SNX3|PI4K2A|RAB3A|STX7|AP3D1|SNAP29|SEC24D|WASH	0.0000233	GO:0016050
234	29	GO Biological Process	Autophagy	0.0014	PS16|TRAPPC8|NPC1|PARK7|GABARAP|VPS33A|VCP|ATG7|LAMP2|WD	0.000025	GO:0006914
879	74	GO Biological Process	Cellular lipid metabolic process	0.0014	X1|ASAH1|FABP5|PLCG2|CYP1B1|ALOX12|FABP4|PTPN11|GCDH|FIG4|	0.0000256	GO:0044255
771	67	GO Biological Process	Defense response to other organism	0.0014	EP|CD74|CXCL16|TRIM58|AIF1|C8B|PLCG2|MPO|UNC13D|ATP1B1|CCL	0.0000251	GO:0098542
971	80	GO Biological Process	Response to oxygen-containing compound	0.0014	GRB2|LPL|PRDX2|VIM|MRC1|VCP|SLC9A3R1|GOT1|FES|ALDH1A1|NFK	0.0000242	GO:1901700
1104	88	GO Biological Process	Negative regulation of signaling	0.0016	|CCDC22|SPART|PRDX2|PADI2|TBC1D10C|C1QTNF3|VCP|SLC9A3R1|INP	0.0000296	GO:0023057
287	33	GO Biological Process	Negative regulation of transport	0.0016	NN2|SNX3|SLC30A1|SRI|ITGB3|SLC9A3R1|INPP5K|LRPAP1|ANKRD13A|	0.0000283	GO:0051051
50	12	GO Biological Process	Regulation of blood coagulation	0.0017	CPB2|PLAU|ALOX12|EMILIN2|PRDX2|ST3GAL4|PRKG1|PROC|FGG|FGA|	0.0000309	GO:0030193
153	22	GO Biological Process	Negative regulation of supramolecular fiber organization	0.0018	TAN1|EML2|ARHGAP6|LOC101105107|INPP5K|KANK1|ADD1|LOC10111	0.0000324	GO:1902904
69	14	GO Biological Process	Cellular amino acid biosynthetic process	0.0019	SEPHS1|PCBD1|SDS|ASS1|OAT|BCAT2|GOT1|ALDH1A1|MTHFR|SHMT1|	0.0000353	GO:0008652
20	8	GO Biological Process	Barbed-end actin filament capping	0.0019	AVIL|GSN|SCIN|ADD3|ADD1|TWF1|CAPG|EPS8	0.0000345	GO:0051016
780	67	GO Biological Process	Positive regulation of intracellular signal transduction	0.0019	NCK1|ALOX15|PLCG2|RGL2|MAP4K2|PTPN11|CCL2|PARK7|CCAR2|PDCD	0.000035	GO:1902533
121	19	GO Biological Process	Cytosolic transport	0.002	K8|HEATR5A|SNX6|LAMP1|WDR91|SNX3|TBC1D10C|WDR81|YKT6|CLN	0.0000381	GO:0016482
79	15	GO Biological Process	Cellular response to reactive oxygen species	0.002	D1|MAPK3|SOD1-2|SOD2|MMP9|CYP1B1|MPO|PARK7|PRDX2|MMP2|	0.0000369	GO:0034614
442	44	GO Biological Process	Ion homeostasis	0.002	22|ATP6V1B2|VPS33A|SLC30A1|SLC9A3R1|CA2|ATP6V1E1|RGN|ENPP1|	0.0000379	GO:0050801
305	34	GO Biological Process	Regulation of apoptotic signaling pathway	0.002	WFS1|CD74|NCK1|MMP9|PEA15|PARK7|CCAR2|PRDX2|SLC9A3R1|HSPB	0.0000382	GO:2001233
100	17	GO Biological Process	Response to interferon-gamma	0.0021	01238|W5P3V8_SHEEP|RAB7B|GSN|CD74|CXCL16|AIF1|CCL2|VIM|MR	0.0000399	GO:0034341
144	21	GO Biological Process	Positive regulation of supramolecular fiber organization	0.0021	|WDR1|ALOX15|HSPA1A-2|VASP|GRB2|FES|CCN2|PPM1F|CYFIP1|DSTN	0.0000404	GO:1902905
280	32	GO Biological Process	Positive regulation of leukocyte activation	0.0022	|LAMP1|ITGAM|W5PIC9_SHEEP|ITGB2|PPP3CA|LGALS1|SASH3|W5Q2C	0.0000414	GO:0002696
1099	87	GO Biological Process	Negative regulation of cell communication	0.0022	AR2|CCDC22|SPART|PRDX2|PADI2|TBC1D10C|C1QTNF3|VCP|SLC9A3R1	0.0000414	GO:0010648
293	33	GO Biological Process	Regulation of T cell activation	0.0022	C101109746|AIF1|CLPTM1|PRDX2|GPNMB|PPP3CA|LGALS1|SASH3|W5	0.0000411	GO:0050863
1230	95	GO Biological Process	Positive regulation of protein metabolic process	0.0022	M|CYFIP2|ITGB3|VCP|RAP2C|JARID2|GPNMB|UBE2V2|RNASE4|LGMN|	0.0000437	GO:0051247
430	43	GO Biological Process	Inorganic ion homeostasis	0.0022	CDC22|ATP6V1B2|VPS33A|SLC30A1|SLC9A3R1|CA2|ATP6V1E1|RGN|ENP	0.000042	GO:0098771
5039	310	GO Biological Process	Organonitrogen compound metabolic process	0.0022	WFS1|ATE1|SIRT2|MTMR3|SEPHS1|W5P7E0_SHEEP|DUSP3|W5P7K2_SHE	0.0000424	GO:1901564
321	35	GO Biological Process	Lipid transport	0.0023	FABP5|FABP4|ABCC1|MSR1|ESYT2|TRIAP1|OSBPL8|CLPTM1L|SLC27A1|	0.0000446	GO:0006869
348	37	GO Biological Process	Cellular ion homeostasis	0.0023	|ATP1B1|CCDC22|ATP6V1B2|VPS33A|SLC30A1|SLC9A3R1|CA2|RGN|EN	0.0000445	GO:0006873
21	8	GO Biological Process	Glycoside metabolic process	0.0023	USB|FUCA2|GLA|FUCA1|W5PKU3_SHEEP|LOC101110434|NAGA|ABHD1	0.0000457	GO:0016137
417	42	GO Biological Process	Positive regulation of cell migration	0.0023	AU|PLCG2|ALOX12|DAB2|PRKD2|SELP|ITGB3|MOSPD2|GPNMB|LGMN|	0.0000442	GO:0030335
218	27	GO Biological Process	Positive regulation of defense response	0.0024	114535|CARD9|STAP1|LOC101112936|LAMP1|PLCG2|FABP4|ABCC1|EN	0.0000474	GO:0031349
62	13	GO Biological Process	Negative regulation of actin filament polymerization	0.0025	|AVIL|GSN|SCIN|ADD3|SPTAN1|KANK1|ADD1|SSH1|TWF1|CAPG|EPS8|	0.0000496	GO:0030837
124	19	GO Biological Process	Negative regulation of protein-containing complex assembly	0.0025	GSN|SIRT2|SCIN|ADD3|SPTAN1|EML2|KANK1|AIDA|ADD1|TBCD|SSH1	0.0000512	GO:0031333
170	23	GO Biological Process	Positive regulation of cytoskeleton organization	0.0025	T1|NCK1|WDR1|ALOX15|HSPA1A-2|VASP|GRB2|FES|CCN2|PPM1F|CYF	0.0000507	GO:0051495

Supplementary Table 11: Comparative functional enrichment analysis of GMØ (monocyte-derived macrophages differentiated with GM-CSF) and MMØ (monocyte-derived macrophages differentiated with M-CSF) relative to Mo (monocytes at 3 hours). Functional enrichment analysis was performed using the StringApp (STRING database) within Cytoscape (version 3.10.3)[1], focusing on Gene Ontology (GO) Biological Process (BP) terms[2,3]. Red-highlighted cells indicate Gene Ontology Biological Process terms shared between GMØ and MMØ compared to Mo, whereas non-highlighted cells represent subtype-specific enrichment.

<table>
<tr><td>901</td><td>74</td><td>GO Biological Process</td><td>Cell motility</td><td>0.0026</td><td>CL2|PRTN3|CNN2|SELP|ITGB3|SLC9A3R1|ITGB2|RNASE4|KANK1|RRAS</td><td>0.0000538</td><td>GO:0048870</td></tr>
<tr><td>147</td><td>21</td><td>GO Biological Process</td><td>Negative regulation of cytoskeleton organization</td><td>0.0026</td><td>TAN1|EML2|ARHGAP6|FKBP4|LOC101105107|INPP5K|KANK1|ADD1|TB</td><td>0.0000528</td><td>GO:0051494</td></tr>
<tr><td>82</td><td>15</td><td>GO Biological Process</td><td>Cellular response to interferon-gamma</td><td>0.0026</td><td>OC100101238|W5P3V8_SHEEP|RAB7B|GSN|AIF1|CCL2|VIM|MRC1|TLR</td><td>0.0000538</td><td>GO:0071346</td></tr>
<tr><td>507</td><td>48</td><td>GO Biological Process</td><td>Supramolecular fiber organization</td><td>0.0026</td><td>IF1|WDR1|WASHC5|CYP1B1|VASP|ARHGAP6|VIM|HIP1|ARHGAP12|M</td><td>0.0000543</td><td>GO:0097435</td></tr>
<tr><td>435</td><td>43</td><td>GO Biological Process</td><td>Positive regulation of cell motility</td><td>0.0026</td><td>RN|PLAU|PLCG2|ALOX12|DAB2|PRKD2|SELP|ITGB3|MOSPD2|GPNMB|</td><td>0.0000536</td><td>GO:2000147</td></tr>
<tr><td>183</td><td>24</td><td>GO Biological Process</td><td>Negative regulation of apoptotic signaling pathway</td><td>0.0026</td><td>|WFS1|CD74|MMP9|PEA15|PARK7|CCAR2|PRDX2|HSPB1|CAAP1|ITGA</td><td>0.0000548</td><td>GO:2001234</td></tr>
<tr><td>72</td><td>14</td><td>GO Biological Process</td><td>Negative regulation of extrinsic apoptotic signaling pathway</td><td>0.0026</td><td>|RIPK1|EYA3|BRCA1|LMNA|PEA15|PARK7|PRDX2|ITGAV|FGG|FGA|MA</td><td>0.000053</td><td>GO:2001237</td></tr>
<tr><td>221</td><td>27</td><td>GO Biological Process</td><td>Organic acid biosynthetic process</td><td>0.0027</td><td>X15|ALOX12|OAT|PARK7|LPL|BCAT2|LTA4H|GOT1|ALDH1A1|RGN|CSA</td><td>0.0000585</td><td>GO:0016053</td></tr>
<tr><td>450</td><td>44</td><td>GO Biological Process</td><td>Positive regulation of locomotion</td><td>0.0027</td><td>N|PLAU|PLCG2|ALOX12|DAB2|PRKD2|SELP|ITGB3|MOSPD2|GPNMB|L</td><td>0.0000556</td><td>GO:0040017</td></tr>
<tr><td>7243</td><td>425</td><td>GO Biological Process</td><td>Response to stimulus</td><td>0.0027</td><td>5|FOLR2|SOD1-2|LOC101114535|RPS6KA1|IVNS1ABP|NAMPT|CARD9|S</td><td>0.0000565</td><td>GO:0050896</td></tr>
<tr><td>436</td><td>43</td><td>GO Biological Process</td><td>Organophosphate biosynthetic process</td><td>0.0027</td><td>454|ALOX15|PAPSS1|FABP5|PLCG2|TYMS|GCDH|FIG4|NADK2|PI4K2A|</td><td>0.0000562</td><td>GO:0090407</td></tr>
<tr><td>114</td><td>18</td><td>GO Biological Process</td><td>Regulation of response to wounding</td><td>0.0027</td><td>|ALOX12|EMILIN2|PRDX2|ST3GAL4|KANK1|PRKG1|EPPK1|PROC|FGG|</td><td>0.0000566</td><td>GO:1903034</td></tr>
<tr><td>11362</td><td>633</td><td>GO Biological Process</td><td>Regulation of biological process</td><td>0.0028</td><td>P|MAOA|EYA3|DYNC1H1|MPP1|CEP55|TIMP2|SPTBN1|RUFY1|W5P02</td><td>0.0000592</td><td>GO:0050789</td></tr>
<tr><td>73</td><td>14</td><td>GO Biological Process</td><td>Chaperone-mediated protein folding</td><td>0.0028</td><td>R1A|TOR1B|HSPA1A-2|FKBP4|HSPH1|CCT7|FKBP5|HSPB1|HSPA13|TO</td><td>0.0000605</td><td>GO:0061077</td></tr>
<tr><td>382</td><td>39</td><td>GO Biological Process</td><td>Export from cell</td><td>0.0029</td><td>|PARK7|EXOC6B|NIBAN2|RAB3A|LOC101122662|MYO1G|MERTK|GNP</td><td>0.0000628</td><td>GO:0140352</td></tr>
<tr><td>186</td><td>24</td><td>GO Biological Process</td><td>Regulation of lymphocyte proliferation</td><td>0.0032</td><td>OC101109746|AIF1|AHR|W5PIC9_SHEEP|GPNMB|PPP3CA|SASH3|W5Q</td><td>0.000069</td><td>GO:0050670</td></tr>
<tr><td>236</td><td>28</td><td>GO Biological Process</td><td>Regulation of ERK1 and ERK2 cascade</td><td>0.0032</td><td>EEP|DUSP3|CSF1R|CD74|ALOX15|PTPN11|CCL2|PRKD2|TBC1D10C|SLC</td><td>0.0000684</td><td>GO:0070372</td></tr>
<tr><td>30</td><td>9</td><td>GO Biological Process</td><td>Clathrin-dependent endocytosis</td><td>0.0033</td><td>AP2A2|PICALM|FCHO2|FCHSD2|AP2S1|LMBRD1|HIP1|AP2A1|AP2A2-2</td><td>0.0000717</td><td>GO:0072583</td></tr>
<tr><td>238</td><td>28</td><td>GO Biological Process</td><td>Positive regulation of cell-cell adhesion</td><td>0.0035</td><td>EMILIN2|SELP|ITGB2|ST3GAL4|PPP3CA|LGALS1|SASH3|W5Q2C4_SHEE</td><td>0.0000781</td><td>GO:0022409</td></tr>
<tr><td>23</td><td>8</td><td>GO Biological Process</td><td>Platelet aggregation</td><td>0.0035</td><td>FGB|FERMT3|ITGB3|RAP2B|GNAS|FGG|FGA|PLEK</td><td>0.0000772</td><td>GO:0070527</td></tr>
<tr><td>3935</td><td>248</td><td>GO Biological Process</td><td>Negative regulation of cellular process</td><td>0.0036</td><td>RA1|NCK1|MMP9|NPC1|AIF1|ITPR1|GRN|ANXA6|PEA15|KAT6B|TPCN1</td><td>0.0000809</td><td>GO:0048523</td></tr>
<tr><td>164</td><td>22</td><td>GO Biological Process</td><td>Regulation of cell-substrate adhesion</td><td>0.0037</td><td>|RSU1|UNC13D|W5PIC9_SHEEP|ARHGAP6|RCC2|ITGB3|KANK1|RRAS|</td><td>0.0000819</td><td>GO:0010810</td></tr>
<tr><td>107</td><td>17</td><td>GO Biological Process</td><td>Detoxification</td><td>0.0038</td><td>O1|SOD1-2|SOD2|ESD|MPO|PARK7|PRXL2A|GSR|PRDX2|SLC30A1|PRD</td><td>0.0000843</td><td>GO:0098754</td></tr>
<tr><td>669</td><td>58</td><td>GO Biological Process</td><td>Negative regulation of apoptotic process</td><td>0.0041</td><td>4|GCLC|CD74|ARHGAP10|MMP9|GRN|PEA15|ALOX12|RGL2|DAB2|CCL</td><td>0.0000925</td><td>GO:0043066</td></tr>
<tr><td>119</td><td>18</td><td>GO Biological Process</td><td>Regulation of protein-containing complex disassembly</td><td>0.0041</td><td>CIN|WDR1|ADD3|SPTAN1|ADD1|DSTN|LIMA1|TWF1|PLEK|ZMPSTE24|C</td><td>0.0000926</td><td>GO:0043244</td></tr>
<tr><td>761</td><td>64</td><td>GO Biological Process</td><td>Negative regulation of cell death</td><td>0.0041</td><td>ARHGAP10|NCK1|MMP9|GRN|PEA15|PLCG2|ALOX12|RGL2|DAB2|CCL2</td><td>0.0000921</td><td>GO:0060548</td></tr>
<tr><td>335</td><td>35</td><td>GO Biological Process</td><td>Cellular cation homeostasis</td><td>0.0042</td><td>|SLC39A7|ATP1B1|CCDC22|ATP6V1B2|VPS33A|SLC30A1|CA2|RGN|TCIR</td><td>0.0000971</td><td>GO:0030003</td></tr>
<tr><td>462</td><td>44</td><td>GO Biological Process</td><td>Regulation of defense response</td><td>0.0042</td><td>36|GIT1|GRN|LAMP1|ALOX15|PLCG2|FABP4|W5PIC9_SHEEP|ABCC1|E</td><td>0.0000968</td><td>GO:0031347</td></tr>
<tr><td>2176</td><td>149</td><td>GO Biological Process</td><td>Cellular component assembly</td><td>0.0043</td><td>TGB3|TLN1|PATL1|SUB1|VCP|SLC9A3R1|CRYZ|H4C14|INPP5K|ATG7|L</td><td>0.0000998</td><td>GO:0022607</td></tr>
<tr><td>377</td><td>38</td><td>GO Biological Process</td><td>Small molecule biosynthetic process</td><td>0.0043</td><td>SAH1|PLCG2|ALOX12|MPO|OAT|PARK7|LPL|PDXK|HPRT1|BCAT2|LTA4</td><td>0.000099</td><td>GO:0044283</td></tr>
<tr><td>322</td><td>34</td><td>GO Biological Process</td><td>Establishment of organelle localization</td><td>0.0043</td><td>MAP4K2|FAM91A1|EXOC6B|SLC9A3R1|RAB3A|CLASP1|LMNB2|UBE2B|</td><td>0.0001</td><td>GO:0051656</td></tr>
<tr><td>24</td><td>8</td><td>GO Biological Process</td><td>Amyloid-beta clearance</td><td>0.0043</td><td>E|W5NRI1_SHEEP|TREM2|PICALM|LRP1|MSR1|LRPAP1|W5Q6N3_SHE</td><td>0.0000986</td><td>GO:0097242</td></tr>
<tr><td>420</td><td>41</td><td>GO Biological Process</td><td>Cation homeostasis</td><td>0.0044</td><td>TP1B1|CCDC22|ATP6V1B2|VPS33A|SLC30A1|CA2|ATP6V1E1|RGN|TCIR</td><td>0.0001</td><td>GO:0055080</td></tr>
<tr><td>1851</td><td>130</td><td>GO Biological Process</td><td>Response to external stimulus</td><td>0.0045</td><td>NC13D|FABP4|ATP1B1|CCL2|POSTN|VASP|MX1|PDCD4|CNN2|LPL|PR</td><td>0.00011</td><td>GO:0009605</td></tr>
<tr><td>40</td><td>10</td><td>GO Biological Process</td><td>Platelet activation</td><td>0.0045</td><td>FGB|TREML1|FERMT3|ITGB3|MERTK|RAP2B|GNAS|FGG|FGA|PLEK</td><td>0.00011</td><td>GO:0030168</td></tr>
<tr><td>674</td><td>58</td><td>GO Biological Process</td><td>Regulation of proteolysis</td><td>0.0047</td><td>44|PITHD1|MMP9|GRN|HSPA1A-2|W5PHI7_SHEEP|ALOX12|DAB2|BIN1-</td><td>0.00011</td><td>GO:0030162</td></tr>
<tr><td>168</td><td>22</td><td>GO Biological Process</td><td>Protein stabilization</td><td>0.0047</td><td>P1|ATP1B1|PARK7|CCT7|LAMP2|HIP1|IFI30|CDC37L1|PIN1|WDR81|UB</td><td>0.00011</td><td>GO:0050821</td></tr>
<tr><td>690</td><td>59</td><td>GO Biological Process</td><td>Negative regulation of programmed cell death</td><td>0.0048</td><td>CLC|CD74|ARHGAP10|MMP9|GRN|PEA15|PLCG2|ALOX12|RGL2|DAB2|</td><td>0.00011</td><td>GO:0043069</td></tr>
<tr><td>218</td><td>26</td><td>GO Biological Process</td><td>Carboxylic acid biosynthetic process</td><td>0.0048</td><td>ALOX15|ALOX12|OAT|PARK7|LPL|BCAT2|LTA4H|GOT1|ALDH1A1|RGN|</td><td>0.00011</td><td>GO:0046394</td></tr>
<tr><td>245</td><td>28</td><td>GO Biological Process</td><td>Positive regulation of GTPase activity</td><td>0.0051</td><td>P2|RASGRP4|MAPRE2|SNX18|RASGRP2|CHN2|RSU1|RGL2|TBC1D13|TE</td><td>0.00012</td><td>GO:0043547</td></tr>
<tr><td>79</td><td>14</td><td>GO Biological Process</td><td>Regulation of pH</td><td>0.0053</td><td>APK3|LACC1|GRN|ATP6V1B2|VPS33A|CA2|ATP6V1E1|TCIRG1|ATP6V1</td><td>0.00013</td><td>GO:0006885</td></tr>
<tr><td>12</td><td>6</td><td>GO Biological Process</td><td>Removal of superoxide radicals</td><td>0.0054</td><td>PRDX1|NQO1|SOD1-2|SOD2|MPO|PRDX2</td><td>0.00013</td><td>GO:0019430</td></tr>
<tr><td>195</td><td>24</td><td>GO Biological Process</td><td>Response to molecule of bacterial origin</td><td>0.0055</td><td>T2|STAP1|TAP2|GIT1|PLCG2|CCL2|PDCD4|PRDX2|VIM|MRC1|NFKB1|T</td><td>0.00013</td><td>GO:0002237</td></tr>
<tr><td>33</td><td>9</td><td>GO Biological Process</td><td>Cell redox homeostasis</td><td>0.0055</td><td>PRDX1|W5NUX2_SHEEP|NQO1|GCLC|GIT1|GSR|PRDX2|PRDX6|APEX1</td><td>0.00013</td><td>GO:0045454</td></tr>
<tr><td>558</td><td>50</td><td>GO Biological Process</td><td>Negative regulation of cellular component organization</td><td>0.0055</td><td>RT|ARHGAP6|RCC2|VIM|FKBP4|ITGB3|LOC101105107|INPP5K|KANK1|</td><td>0.00013</td><td>GO:0051129</td></tr>
<tr><td>60</td><td>12</td><td>GO Biological Process</td><td>Alpha-amino acid biosynthetic process</td><td>0.0058</td><td>TMED8|SEPHS1|PCBD1|SDS|ASS1|OAT|GOT1|MTHFR|SHMT1|AASS|PH</td><td>0.00014</td><td>GO:1901607</td></tr>
<tr><td>7</td><td>5</td><td>GO Biological Process</td><td>Ganglioside catabolic process</td><td>0.0059</td><td>NEU1|HEXB|LOC101112162|GM2A|HEXA</td><td>0.00015</td><td>GO:0006689</td></tr>
<tr><td>209</td><td>25</td><td>GO Biological Process</td><td>Nucleotide biosynthetic process</td><td>0.0059</td><td>|UCK1|PRPSAP1|GARS1|LOC101102454|PAPSS1|TYMS|GCDH|NADK2|A</td><td>0.00015</td><td>GO:0009165</td></tr>
<tr><td>209</td><td>25</td><td>GO Biological Process</td><td>Positive regulation of secretion</td><td>0.0059</td><td>UNC13D|PTPN11|SRI|C1QTNF3|ITGB2|MPC2|RNASE4|RAB3A|PPP3CA|T</td><td>0.00015</td><td>GO:0051047</td></tr>
<tr><td>70</td><td>13</td><td>GO Biological Process</td><td>Regulation of epithelial cell apoptotic process</td><td>0.0059</td><td>AP2|GSN|WFS1|PLA2R1|CCL2|PDCD4|FGG|FGA|ITGA4|MAPK7|THBS1|</td><td>0.00015</td><td>GO:1904035</td></tr>
<tr><td>136</td><td>19</td><td>GO Biological Process</td><td>Maintenance of location</td><td>0.0061</td><td>A|FLNA|GSN|SUN1|SCIN|GM2A|PARK7|CCDC22|SRI|ENPP1|SOAT1|W5</td><td>0.00015</td><td>GO:0051235</td></tr>
<tr><td>91</td><td>15</td><td>GO Biological Process</td><td>Regulation of wound healing</td><td>0.0061</td><td>ALOX12|EMILIN2|PRDX2|ST3GAL4|KANK1|PRKG1|EPPK1|PROC|FGG|F</td><td>0.00015</td><td>GO:0061041</td></tr>
<tr><td>34</td><td>9</td><td>GO Biological Process</td><td>Superoxide metabolic process</td><td>0.0064</td><td>CF2|PRDX1|NQO1|SH3PXD2B|SOD1-2|SOD2|MPO|PRDX2|LOC1011226</td><td>0.00016</td><td>GO:0006801</td></tr>
<tr><td>125</td><td>18</td><td>GO Biological Process</td><td>Monovalent inorganic cation homeostasis</td><td>0.0064</td><td>8A1|GRN|ATP1B1|ATP6V1B2|VPS33A|CA2|ATP6V1E1|TCIRG1|SLC12A9</td><td>0.00016</td><td>GO:0055067</td></tr>
<tr><td>211</td><td>25</td><td>GO Biological Process</td><td>Positive regulation of immune effector process</td><td>0.0066</td><td>AP2|LOC101109746|LAMP1|PLCG2|ITGAM|UNC13D|ITGB2|FES|ANKRD</td><td>0.00017</td><td>GO:0002699</td></tr>
<tr><td>1034</td><td>80</td><td>GO Biological Process</td><td>Defense response</td><td>0.0066</td><td>58|AIF1|C8B|PLCG2|MPO|UNC13D|ATP1B1|CCL2|PARK7|MX1|SELP|P</td><td>0.00017</td><td>GO:0006952</td></tr>
</table>

Supplementary Table 11: Comparative functional enrichment analysis of GMØ (monocyte-derived macrophages differentiated with GM-CSF) and MMØ (monocyte-derived macrophages differentiated with M-CSF) relative to Mo (monocytes at 3 hours). Functional enrichment analysis was performed using the StringApp (STRING database) within Cytoscape (version 3.10.3)[1], focusing on Gene Ontology (GO) Biological Process (BP) terms[2,3]. Red-highlighted cells indicate Gene Ontology Biological Process terms shared between GMØ and MMØ compared to Mo, whereas non-highlighted cells represent subtype-specific enrichment.

<table>
<tr><td>114</td><td>17</td><td>GO Biological Process</td><td>Positive regulation of leukocyte proliferation</td><td>0.0066</td><td>CSF1R|CD74|NCK1|LOC101109746|AIF1|W5PIC9_SHEEP|PPP3CA|SASH3</td><td>0.00017</td><td>GO:0070665</td></tr>
<tr><td>374</td><td>37</td><td>GO Biological Process</td><td>Regulation of endopeptidase activity</td><td>0.0067</td><td>_SHEEP|LOC101105044|MMP9|W5PHI7_SHEEP|ALOX12|BIN1-2|PARK7|</td><td>0.00017</td><td>GO:0052548</td></tr>
<tr><td>82</td><td>14</td><td>GO Biological Process</td><td>Regulation of cell-matrix adhesion</td><td>0.007</td><td>U|VCL|W5PIC9_SHEEP|ARHGAP6|RCC2|ITGB3|RRAS|PPM1F|ACTG1|MA</td><td>0.00018</td><td>GO:0001952</td></tr>
<tr><td>319</td><td>33</td><td>GO Biological Process</td><td>Negative regulation of immune system process</td><td>0.007</td><td>01|CD55|GRN|ALOX15|AHR|CCL2|CNN2|PRDX2|PADI2|TBC1D10C|GPN</td><td>0.00018</td><td>GO:0002683</td></tr>
<tr><td>93</td><td>15</td><td>GO Biological Process</td><td>Carbohydrate catabolic process</td><td>0.0072</td><td>YGB|ALDOB|GPD2|GM2A|GALM|W5PKU3_SHEEP|PFKP|STBD1|GK|NA</td><td>0.00019</td><td>GO:0016052</td></tr>
<tr><td>942</td><td>74</td><td>GO Biological Process</td><td>Positive regulation of gene expression</td><td>0.0074</td><td>B1|ALOX12|FABP4|PTPN11|PARK7|CNN2|PRKD2|LPL|MIF4GD|PADI2|V</td><td>0.00019</td><td>GO:0010628</td></tr>
<tr><td>266</td><td>29</td><td>GO Biological Process</td><td>Regulation of protein stability</td><td>0.0074</td><td>DI2|ATP1B1|PARK7|CCAR2|CCT7|LAMP2|HIP1|IFI30|CDC37L1|PIN1|WD</td><td>0.00019</td><td>GO:0031647</td></tr>
<tr><td>894</td><td>71</td><td>GO Biological Process</td><td>Negative regulation of protein metabolic process</td><td>0.0074</td><td>|W5PHI7_SHEEP|FABP4|BIN1-2|PARK7|CCAR2|SNX3|LOC101123010|CS</td><td>0.00019</td><td>GO:0051248</td></tr>
<tr><td>151</td><td>20</td><td>GO Biological Process</td><td>Cellular response to oxidative stress</td><td>0.0075</td><td>|SOD1-2|SOD2|SIRT2|PLA2R1|MMP9|AIF1|CYP1B1|MPO|PARK7|GSR|</td><td>0.0002</td><td>GO:0034599</td></tr>
<tr><td>189</td><td>23</td><td>GO Biological Process</td><td>Response to metal ion</td><td>0.008</td><td>GRP2|PLCG2|FABP4|PARK7|GSS|SLC30A1|LGMN|PPP3CA|TCIRG1|ACO</td><td>0.00021</td><td>GO:0010038</td></tr>
<tr><td>63</td><td>12</td><td>GO Biological Process</td><td>Organic hydroxy compound catabolic process</td><td>0.008</td><td>2|HSD17B6|INPP5K|SYNJ1|W5PY08_SHEEP|OCRL|IMPA1|FAH|GK|SCAR</td><td>0.00021</td><td>GO:1901616</td></tr>
<tr><td>694</td><td>58</td><td>GO Biological Process</td><td>Regulation of immune response</td><td>0.0085</td><td>LOX15|PLCG2|ITGAM|AHR|UNC13D|TAPBP|PARK7|DNASE2|PRKD2|ITG</td><td>0.00023</td><td>GO:0050776</td></tr>
<tr><td>1029</td><td>79</td><td>GO Biological Process</td><td>Immune response</td><td>0.0086</td><td>|MRC1|W5PSQ7_SHEEP|FES|ANKRD17|W5PWS0_SHEEP|LGALS1|PSPC</td><td>0.00023</td><td>GO:0006955</td></tr>
<tr><td>178</td><td>22</td><td>GO Biological Process</td><td>Alpha-amino acid metabolic process</td><td>0.0088</td><td>SS2|SEPHS1|PCBD1|GCLC|SDS|ASS1|OAT|GCDH|BCAT2|GOT1|KYAT3|F</td><td>0.00024</td><td>GO:1901605</td></tr>
<tr><td>191</td><td>23</td><td>GO Biological Process</td><td>Gliogenesis</td><td>0.009</td><td>4535|NDRG1|SIRT2|SUN1|CSF1R|PTPN11|CCL2|VIM|SH3TC1|CUL4B|R</td><td>0.00024</td><td>GO:0042063</td></tr>
<tr><td>410</td><td>39</td><td>GO Biological Process</td><td>Cellular chemical homeostasis</td><td>0.009</td><td>ATP1B1|CCDC22|ATP6V1B2|VPS33A|SLC30A1|C1QTNF3|SLC9A3R1|CA2</td><td>0.00024</td><td>GO:0055082</td></tr>
<tr><td>204</td><td>24</td><td>GO Biological Process</td><td>Positive regulation of leukocyte cell-cell adhesion</td><td>0.0091</td><td>6|AIF1|SELP|ITGB2|ST3GAL4|PPP3CA|LGALS1|SASH3|W5Q2C4_SHEEP|</td><td>0.00025</td><td>GO:1903039</td></tr>
<tr><td>666</td><td>56</td><td>GO Biological Process</td><td>Negative regulation of catalytic activity</td><td>0.0092</td><td>4|NCK1|MMP9|PALM|W5PHI7_SHEEP|FABP4|BIN1-2|PARK7|CCAR2|RC</td><td>0.00025</td><td>GO:0043086</td></tr>
<tr><td>14</td><td>6</td><td>GO Biological Process</td><td>Negative regulation of glucose transmembrane transport</td><td>0.0093</td><td>PEA15|FABP5|INPP5K|ENPP1|APPL2|PRKCB</td><td>0.00025</td><td>GO:0010829</td></tr>
<tr><td>179</td><td>22</td><td>GO Biological Process</td><td>Cellular response to chemical stress</td><td>0.0093</td><td>1-2|SOD2|SIRT2|PLA2R1|MMP9|AIF1|CYP1B1|MPO|PARK7|GSR|PRDX2</td><td>0.00025</td><td>GO:0062197</td></tr>
<tr><td>155</td><td>20</td><td>GO Biological Process</td><td>Cellular response to biotic stimulus</td><td>0.0098</td><td>STAP1|GIT1|SMC1A|PLCG2|CCL2|PDCD4|VIM|MRC1|NFKB1|TLR2|IL18|</td><td>0.00027</td><td>GO:0071216</td></tr>
<tr><td>37</td><td>9</td><td>GO Biological Process</td><td>Negative regulation of coagulation</td><td>0.01</td><td>FGB|CPB2|PLAU|ALOX12|PRKG1|ANXA5|PROC|FGG|FGA</td><td>0.00028</td><td>GO:0050819</td></tr>
<tr><td>206</td><td>24</td><td>GO Biological Process</td><td>Regulation of leukocyte mediated immunity</td><td>0.0101</td><td>20001|CD55|LAMP1|PLCG2|ITGAM|AHR|UNC13D|ITGB2|FES|W5PWS0_</td><td>0.00028</td><td>GO:0002703</td></tr>
<tr><td>206</td><td>24</td><td>GO Biological Process</td><td>Protein folding</td><td>0.0101</td><td>PA1A-2|PPIF|FKBP4|HSPH1|CCT7|FKBP5|CDC37L1|HSPB1|CCT8|W5PYX</td><td>0.00028</td><td>GO:0006457</td></tr>
<tr><td>300</td><td>31</td><td>GO Biological Process</td><td>Regulation of immune effector process</td><td>0.0102</td><td>C101109746|CD55|GRN|LAMP1|PLCG2|ITGAM|AHR|UNC13D|ITGB2|F</td><td>0.00028</td><td>GO:0002697</td></tr>
<tr><td>156</td><td>20</td><td>GO Biological Process</td><td>Positive regulation of ERK1 and ERK2 cascade</td><td>0.0104</td><td>RD9|LOC100101238|W5P3V8_SHEEP|CSF1R|CD74|ALOX15|PTPN11|CCL</td><td>0.00029</td><td>GO:0070374</td></tr>
<tr><td>132</td><td>18</td><td>GO Biological Process</td><td>Cellular response to molecule of bacterial origin</td><td>0.0105</td><td>35|SIRT2|STAP1|GIT1|PLCG2|CCL2|PDCD4|VIM|MRC1|NFKB1|TLR2|IL1</td><td>0.00029</td><td>GO:0071219</td></tr>
<tr><td>734</td><td>60</td><td>GO Biological Process</td><td>Response to abiotic stimulus</td><td>0.0107</td><td>PALM|CCAR2|CNN2|GRB2|VCP|YWHAE|TBL2|NFKB1|RNASE4|LGMN|RA</td><td>0.0003</td><td>GO:0009628</td></tr>
<tr><td>109</td><td>16</td><td>GO Biological Process</td><td>Positive regulation of chemotaxis</td><td>0.0107</td><td>535|RIPOR2|CSF1R|CD74|AIF1|PRKD2|MOSPD2|LGMN|HSPB1|ADAM17</td><td>0.0003</td><td>GO:0050921</td></tr>
<tr><td>372</td><td>36</td><td>GO Biological Process</td><td>Positive regulation of cytokine production</td><td>0.0108</td><td>RCA1|RAB7B|CSF1R|PLA2R1|CD74|LACC1|AIF1|PLCG2|CYP1B1|PTPN11|</td><td>0.00031</td><td>GO:0001819</td></tr>
<tr><td>98</td><td>15</td><td>GO Biological Process</td><td>Cytokinesis</td><td>0.0109</td><td>TBN1|SEPTIN7|SEPTIN1|SEPTIN9|SNX18|WASHC5|SEPTIN8|PLEC|SEPTIN</td><td>0.00031</td><td>GO:0000910</td></tr>
<tr><td>157</td><td>20</td><td>GO Biological Process</td><td>Nucleoside triphosphate metabolic process</td><td>0.0109</td><td>|ALDOB|SMPDL3A|LOC101102454|TYMS|ATP1B1|ATP6V1B2|AK1|UCK2</td><td>0.00031</td><td>GO:0009141</td></tr>
<tr><td>87</td><td>14</td><td>GO Biological Process</td><td>Cellular monovalent inorganic cation homeostasis</td><td>0.0109</td><td>1|MAPK3|LACC1|SLC8A1|GRN|ATP1B1|ATP6V1B2|VPS33A|CA2|TCIRG1</td><td>0.00031</td><td>GO:0030004</td></tr>
<tr><td>98</td><td>15</td><td>GO Biological Process</td><td>Cellular detoxification</td><td>0.0109</td><td>O1|SOD1-2|SOD2|ESD|MPO|PARK7|PRXL2A|GSR|PRDX2|PRDX6|ALDH</td><td>0.00031</td><td>GO:1990748</td></tr>
<tr><td>461</td><td>42</td><td>GO Biological Process</td><td>Positive regulation of organelle organization</td><td>0.0114</td><td>|STAP1|SCIN|GIT1|NCK1|MMP9|WDR1|SNX18|GRN|ALOX15|HSPA1A-2</td><td>0.00033</td><td>GO:0010638</td></tr>
<tr><td>38</td><td>9</td><td>GO Biological Process</td><td>Apoptotic cell clearance</td><td>0.0114</td><td>TREM2|RAC1|ALOX15|MFGE8|ITGB3|MERTK|ITGAV|SCARB1|THBS1</td><td>0.00033</td><td>GO:0043277</td></tr>
<tr><td>15</td><td>6</td><td>GO Biological Process</td><td>Regulation of phagocytosis, engulfment</td><td>0.0118</td><td>W5NRI1_SHEEP|TREM2|STAP1|ALOX15|PLCG2|APPL2</td><td>0.00034</td><td>GO:0060099</td></tr>
<tr><td>78</td><td>13</td><td>GO Biological Process</td><td>Negative regulation of transmembrane transport</td><td>0.0127</td><td>ABP5|SLC30A1|SRI|SLC9A3R1|INPP5K|ENPP1|APPL2|PRKCB|CTTNBP2N</td><td>0.00037</td><td>GO:0034763</td></tr>
<tr><td>172</td><td>21</td><td>GO Biological Process</td><td>Leukocyte mediated immunity</td><td>0.0128</td><td>535|CARD9|CD74|TAP2|WDR1|CD55|C8B|UNC13D|W5PWS0_SHEEP|M</td><td>0.00038</td><td>GO:0002443</td></tr>
<tr><td>135</td><td>18</td><td>GO Biological Process</td><td>Organophosphate catabolic process</td><td>0.0128</td><td>D12|PLBD2|SMPDL3A|PLCG2|VCP|INPP5K|SYNJ1|ENPP1|OCRL|IMPA1|</td><td>0.00038</td><td>GO:0046434</td></tr>
<tr><td>1166</td><td>86</td><td>GO Biological Process</td><td>Regulation of phosphate metabolic process</td><td>0.0139</td><td>B3|VCP|SLC9A3R1|ITGB2|RAP2C|GPNMB|INPP5K|RNASE4|BANK1|PIN1</td><td>0.00041</td><td>GO:0019220</td></tr>
<tr><td>101</td><td>15</td><td>GO Biological Process</td><td>Positive regulation of lymphocyte proliferation</td><td>0.0139</td><td>CD74|NCK1|LOC101109746|AIF1|W5PIC9_SHEEP|PPP3CA|SASH3|IL18|V</td><td>0.00041</td><td>GO:0050671</td></tr>
<tr><td>186</td><td>22</td><td>GO Biological Process</td><td>Positive regulation of secretion by cell</td><td>0.0139</td><td>C13D|PTPN11|SRI|C1QTNF3|ITGB2|MPC2|RNASE4|RAB3A|TLR2|PLA2G</td><td>0.00041</td><td>GO:1903532</td></tr>
<tr><td>23</td><td>7</td><td>GO Biological Process</td><td>Nuclear migration</td><td>0.0142</td><td>DYNC1H1|LMNA|SUN1|SLC9A3R1|LMNB2|SUN2|LMNB1</td><td>0.00043</td><td>GO:0007097</td></tr>
<tr><td>423</td><td>39</td><td>GO Biological Process</td><td>Positive regulation of cell death</td><td>0.0142</td><td>CK1|MMP9|PEA15|CYP1B1|ALOX12|BIN1-2|CCL2|EMILIN2|PARK7|CCA</td><td>0.00043</td><td>GO:0010942</td></tr>
<tr><td>31</td><td>8</td><td>GO Biological Process</td><td>Glycosyl compound catabolic process</td><td>0.0142</td><td>GUSB|FUCA2|GLA|ADA|FUCA1|W5PKU3_SHEEP|NAGA|ABHD10</td><td>0.00043</td><td>GO:1901658</td></tr>
<tr><td>149</td><td>19</td><td>GO Biological Process</td><td>Cellular carbohydrate metabolic process</td><td>0.0143</td><td>_SHEEP|GOT1|INPP5K|SYNJ1|PFKP|OCRL|IMPA1|GNPTAB|GLB1|STBD1</td><td>0.00043</td><td>GO:0044262</td></tr>
<tr><td>240</td><td>26</td><td>GO Biological Process</td><td>Negative regulation of cell adhesion</td><td>0.0147</td><td>2|PTPN11|PRDX2|ARHGAP6|RCC2|GPNMB|KANK1|PRKG1|PPM1F|PLX</td><td>0.00045</td><td>GO:0007162</td></tr>
<tr><td>16</td><td>6</td><td>GO Biological Process</td><td>Platelet formation</td><td>0.0148</td><td>MPIG6B|WDR1|PTPN11|VPS33A|MYH9|ACTN1</td><td>0.00045</td><td>GO:0030220</td></tr>
<tr><td>59</td><td>11</td><td>GO Biological Process</td><td>Substrate adhesion-dependent cell spreading</td><td>0.0148</td><td>AK1|FERMT3|ITGB3|MERTK|ITGAV|ITGA4|ILK|FER|PARVB|RAB1A|FND</td><td>0.00045</td><td>GO:0034446</td></tr>
<tr><td>516</td><td>45</td><td>GO Biological Process</td><td>Cell activation</td><td>0.0159</td><td>GAL|CD74|NCK1|TPD52|PLCG2|UNC13D|PRDX2|TBC1D10C|ITGB3|MS4</td><td>0.00049</td><td>GO:0001775</td></tr>
<tr><td>70</td><td>12</td><td>GO Biological Process</td><td>Dicarboxylic acid metabolic process</td><td>0.016</td><td>MTHFD1L|ADSS2|ACOT8|GCLC|ASS1|OAT|GOT1|KYAT3|ACLY|SHMT1|</td><td>0.00049</td><td>GO:0043648</td></tr>
<tr><td>176</td><td>21</td><td>GO Biological Process</td><td>Regulation of chemotaxis</td><td>0.016</td><td>F1R|STAP1|CD74|AIF1|W5PIC9_SHEEP|CCL2|PRKD2|PADI2|MOSPD2|L</td><td>0.00049</td><td>GO:0050920</td></tr>
<tr><td>103</td><td>15</td><td>GO Biological Process</td><td>Regulation of protein localization to plasma membrane</td><td>0.016</td><td>ICALM|HECTD1|ABI3|LRP1|DAB2|STX7|W5Q3L8_SHEEP|ACTG1|MMP1</td><td>0.0005</td><td>GO:1903076</td></tr>
<tr><td>32</td><td>8</td><td>GO Biological Process</td><td>Regulation of actin nucleation</td><td>0.0165</td><td>CORO1A|GSN|FCHSD2|SCIN|WASHC5|LOC101105107|CYFIP1|ARFIP2</td><td>0.00051</td><td>GO:0051125</td></tr>
<tr><td>81</td><td>13</td><td>GO Biological Process</td><td>Reactive oxygen species metabolic process</td><td>0.0165</td><td>O1|SH3PXD2B|SOD1-2|SOD2|PLA2R1|ACOX1|MPO|PARK7|PRDX2|LOC</td><td>0.00051</td><td>GO:0072593</td></tr>
</table>

Supplementary Table 11: Comparative functional enrichment analysis of GMØ (monocyte-derived macrophages differentiated with GM-CSF) and MMØ (monocyte-derived macrophages differentiated with M-CSF) relative to Mo (monocytes at 3 hours). Functional enrichment analysis was performed using the StringApp (STRING database) within Cytoscape (version 3.10.3)[1], focusing on Gene Ontology (GO) Biological Process (BP) terms[2,3]. Red-highlighted cells indicate Gene Ontology Biological Process terms shared between GMØ and MMØ compared to Mo, whereas non-highlighted cells represent subtype-specific enrichment.

24	7	GO Biological Process	Ruffle organization	0.0169	INPPL1|CSF1R|AIF1|INPP5K|TCIRG1|LIMA1|PLEK	0.00053	GO:0031529
341	33	GO Biological Process	Secretion by cell	0.0171	D|PARK7|EXOC6B|NIBAN2|RAB3A|LOC101122662|MYO1G|MERTK|GNF	0.00054	GO:0032940
71	12	GO Biological Process	Positive regulation of protein polymerization	0.0175	FCHSD2|GIT1|NCK1|ALOX15|HSPA1A-2|VASP|GRB2|FES|FER|DLG1|CDI	0.00055	GO:0032273
82	13	GO Biological Process	Membrane invagination	0.0179	C1|GSN|AIF1|SNX18|MSR1|SNX3|MFGE8|ARHGAP12|BIN2|MYH9|THBS	0.00057	GO:0010324
1046	78	GO Biological Process	Regulation of phosphorylation	0.0179	K7|PRKD2|ITGB3|SLC9A3R1|ITGB2|RAP2C|GPNMB|INPP5K|RNASE4|BAN	0.00057	GO:0042325
61	11	GO Biological Process	Positive regulation of phagocytosis	0.0182	HEEP|TREM2|SOD1-2|LMAN2|STAP1|SIRPA|PLCG2|MFGE8|MERTK|APP	0.00058	GO:0050766
117	16	GO Biological Process	Positive regulation of epithelial cell migration	0.0191	PLCG2|ALOX12|PRKD2|ITGB3|LGMN|RRAS|HSPB1|ADAM17|P2RX4|PPN	0.00061	GO:0010634
33	8	GO Biological Process	Negative regulation of blood coagulation	0.0191	FGB|CPB2|PLAU|ALOX12|PRKG1|PROC|FGG|FGA	0.00061	GO:0030195
33	8	GO Biological Process	Collagen catabolic process	0.0191	PEPD|MMP9|MRC2|CTSB|MMP2|MMP14|CTSS|CTSK	0.00061	GO:0030574
33	8	GO Biological Process	Cellular carbohydrate catabolic process	0.0191	GUSB|GAA|PYGB|GPD2|W5PKU3_SHEEP|STBD1|GK|ABHD10	0.00061	GO:0044275
129	17	GO Biological Process	Membrane fusion	0.0191	|FOLR3|FOLR2|RAB7B|GCA|STX7|YKT6|SNAP29|VPS41|USE1|VAV3|VAN	0.00061	GO:0061025
94	14	GO Biological Process	Cytoskeleton-dependent cytokinesis	0.0191	|SPTBN1|SEPTIN7|SEPTIN1|SEPTIN9|SNX18|WASHC5|SEPTIN8|PLEC|SEF	0.00062	GO:0061640
25	7	GO Biological Process	Peptide antigen assembly with MHC protein complex	0.02	EP|LOC101109746|TAPBP|W5Q2C4_SHEEP|W5Q2V1_SHEEP|W5Q3J8_SH	0.00065	GO:0002501
247	26	GO Biological Process	Response to oxidative stress	0.02	D1-2|SOD2|SIRT2|GCLC|PLA2R1|MMP9|AIF1|CYP1B1|MPO|PARK7|GSR	0.00066	GO:0006979
52	10	GO Biological Process	Collagen metabolic process	0.02	COL1A2|PEPD|MMP9|MRC2|CTSB|MMP2|MMP14|P3H1|CTSS|CTSK	0.00066	GO:0032963
539	46	GO Biological Process	Innate immune response	0.02	CXCL16|TRIM58|AIF1|C8B|PLCG2|UNC13D|ATP1B1|CCL2|MX1|VIM|MR	0.00065	GO:0045087
130	17	GO Biological Process	Vesicle localization	0.02	TOR1A|MAP4K2|FAM91A1|VPS33A|EXOC6B|RAB3A|MYO1G|AP3D1|TCI	0.00066	GO:0051648
130	17	GO Biological Process	Cellular response to tumor necrosis factor	0.02	114535|LOC100101238|W5P3V8_SHEEP|BRCA1|ASAH1|FABP4|CCL2|NF	0.00066	GO:0071356
571	48	GO Biological Process	Hematopoietic or lymphoid organ development	0.0205	MC6|PRTN3|DNASE2|CNN2|PRDX2|VPS33A|JARID2|RBM47|MS4A1|RR	0.00068	GO:0048534
1254	90	GO Biological Process	Intracellular signal transduction	0.0207	MAP4K2|PTPN11|CCL2|GRB2|PRKD2|SELP|ABR|TBC1D10C|YWHAE|NU	0.00069	GO:0035556
11	5	GO Biological Process	Fibrinolysis	0.0207	FGB|CPB2|PLAU|FGG|FGA	0.00069	GO:0042730
11	5	GO Biological Process	Actin filament severing	0.0207	AVIL|GSN|SCIN|DSTN|CAPG	0.00069	GO:0051014
11	5	GO Biological Process	Protein localization to nuclear envelope	0.0207	LMNA|LMNB2|OSBPL8|TOR1AIP2|LMNB1	0.00069	GO:0090435
221	24	GO Biological Process	Small GTPase mediated signal transduction	0.0209	GAP3|ARHGEF11|AIF1|RASGRP2|RGL2|NUCB1|RAP2C|RRAS|RAP2B|CYF	0.0007	GO:0007264
333	32	GO Biological Process	Ribose phosphate metabolic process	0.0212	|ALDOB|NUDT14|LOC101102454|PAPSS1|ATP1B1|GCDH|ATP6V1B2|AK1	0.00072	GO:0019693
43	9	GO Biological Process	Regulation of cell-substrate junction organization	0.0212	PEAK1|MAPRE2|VCL|ARHGAP6|RCC2|PPM1F|ACTG1|MACF1|S100A10	0.00071	GO:0150116
119	16	GO Biological Process	Regulation of extrinsic apoptotic signaling pathway	0.0212	EYA3|BRCA1|LMNA|PEA15|PARK7|PRDX2|ITGAV|FGG|FGA|MAPK7|TH	0.00072	GO:2001236
34	8	GO Biological Process	Cellular metabolic compound salvage	0.0214	NAPRT|W5NRS0_SHEEP|ADA|UCK1|PDXK|UCK2|HPRT1|DCK	0.00072	GO:0043094
18	6	GO Biological Process	Antigen processing and presentation of peptide antigen via MHC class	0.0218	IDE|CLEC4A|TAP2|TAPBP|IFI30|FCGR1A	0.00075	GO:0002474
96	14	GO Biological Process	Regulation of leukocyte chemotaxis	0.0218	14535|RIPOR2|CSF1R|STAP1|CD74|AIF1|W5PIC9_SHEEP|CCL2|PADI2|M	0.00074	GO:0002688
277	28	GO Biological Process	Regulation of metal ion transport	0.0218	LB1|CCL2|W5PJN7_SHEEP|FHL1|SLC30A1|SRI|SLC9A3R1|INPP5K|MS4A1	0.00075	GO:0010959
263	27	GO Biological Process	Cellular component disassembly	0.0218	|GSN|STMN1|KIF2A|WDR1|DNASE2|GABARAP|VPS33A|VCP|ATG7|LAN	0.00074	GO:0022411
96	14	GO Biological Process	Regulation of mononuclear cell migration	0.0218	14535|RIPOR2|W5P6T0_SHEEP|CSF1R|AIF1|CCL2|PADI2|ITGB3|MOSPD	0.00074	GO:0071675
53	10	GO Biological Process	Regulation of cellular response to insulin stimulus	0.0218	4K2A|NCK1|PTPN11|INPP5K|KANK1|ENPP1|OSBPL8|ATP2B1|APPL2|PR	0.00075	GO:1900076
108	15	GO Biological Process	Positive regulation of leukocyte migration	0.0221	LOC101114535|RIPOR2|CSF1R|CD74|AIF1|SELP|ITGB3|MOSPD2|LGMN|	0.00077	GO:0002687
74	12	GO Biological Process	Sterol transport	0.0221	PC2|PIP4K2A|OSBPL9|NPC1|MSR1|OSBPL8|SOAT1|TPCN2|LIMA1|SCARE	0.00077	GO:0015918
74	12	GO Biological Process	Retrograde transport, endosome to Golgi	0.0221	|SNX8|HEATR5A|SNX6|SNX3|TBC1D10C|YKT6|CLN5|VPS29|SNX5|RAB9A	0.00077	GO:0042147
237	25	GO Biological Process	Negative regulation of peptidase activity	0.0231	V5P9M9_SHEEP|LOC101105044|MMP9|W5PHI7_SHEEP|BIN1-2|PARK7|L	0.00081	GO:0010466
365	34	GO Biological Process	Regulation of system process	0.0234	|ATP1B1|PTPN11|W5PJN7_SHEEP|FIG4|SRI|SLC9A3R1|INPP5K|PRKG1|	0.00082	GO:0044057
158	19	GO Biological Process	Positive regulation of protein-containing complex assembly	0.0235	CK1|ALOX15|HSPA1A-2|PLCG2|VASP|PARK7|GRB2|VCP|FES|FER|PLEK|	0.00083	GO:0031334
158	19	GO Biological Process	Regulation of protein localization to membrane	0.0235	M|GSN|HECTD1|ABI3|LRP1|DAB2|INPP5K|STX7|W5Q3L8_SHEEP|ACTG1	0.00083	GO:1905475
171	20	GO Biological Process	Negative regulation of cell activation	0.0236	SP3|CD74|GRN|ALOX12|PRDX2|TBC1D10C|GPNMB|BANK1|PRKG1|MEI	0.00083	GO:0050866
121	16	GO Biological Process	Regulation of protein localization to cell periphery	0.0238	ST|PICALM|HECTD1|ABI3|LRP1|DAB2|STX7|W5Q3L8_SHEEP|ACTG1|MN	0.00084	GO:1904375
35	8	GO Biological Process	Regulation of extrinsic apoptotic signaling pathway via death domain	0.024	FGB|BRCA1|PEA15|PARK7|FGG|FGA|THBS1|LGALS3	0.00085	GO:1902041
914	69	GO Biological Process	Regulation of protein phosphorylation	0.0242	|PRKD2|ITGB3|SLC9A3R1|ITGB2|RAP2C|GPNMB|INPP5K|RNASE4|BANK	0.00086	GO:0001932
146	18	GO Biological Process	Regulation of T cell proliferation	0.0242	CK1|LOC101109746|AIF1|GPNMB|PPP3CA|SASH3|W5Q3B8_SHEEP|LOC1	0.00086	GO:0042129
754	59	GO Biological Process	Negative regulation of multicellular organismal process	0.0257	G4|PDCD4|SPART|PRDX2|SRI|C1QTNF3|JARID2|NFKB1|GPNMB|LGMN	0.00092	GO:0051241
19	6	GO Biological Process	Vacuolar acidification	0.0263	ATP6V0A1|ATP6V0D1|GRN|ATP6V1B2|TCIRG1|PPT1	0.00094	GO:0007035
45	9	GO Biological Process	Alcohol catabolic process	0.0264	GPD2|INPP5K|SYNJ1|W5PY08_SHEEP|OCRL|IMPA1|GK|SCARB1|CYP27A	0.00095	GO:0046164
27	7	GO Biological Process	Leukocyte degranulation	0.0265	S100A12|STXBP2|CORO1A|LOC101114535|UNC13D|ANXA3|VAMP2	0.00096	GO:0043299
123	16	GO Biological Process	Establishment of vesicle localization	0.0273	N2|TOR1A|MAP4K2|FAM91A1|EXOC6B|RAB3A|MYO1G|AP3D1|TCIRG1	0.00098	GO:0051650
227	24	GO Biological Process	Negative regulation of cell motility	0.0273	|CNN2|PADI2|LOC101105107|SLC9A3R1|RAP2C|KANK1|RRAS|PIN1|PRK	0.00099	GO:2000146
312	30	GO Biological Process	Immune effector process	0.0283	74|TAP2|WDR1|CD55|C8B|PLCG2|MPO|UNC13D|PRDX2|W5PWS0_SHE	0.001	GO:0002252
66	11	GO Biological Process	Regulation of cellular pH	0.0283	A1|ATP6V0D1|MAPK3|LACC1|GRN|ATP6V1B2|VPS33A|CA2|TCIRG1|PPT	0.001	GO:0030641
77	12	GO Biological Process	Plasma membrane invagination	0.0286	RAC1|GSN|AIF1|SNX18|MSR1|MFGE8|ARHGAP12|BIN2|MYH9|THBS1|	0.001	GO:0099024
242	25	GO Biological Process	Positive regulation of lymphocyte activation	0.0288	AIF1|LAMP1|W5PIC9_SHEEP|PPP3CA|LGALS1|SASH3|W5Q2C4_SHEEP|	0.0011	GO:0051251

Supplementary Table 11: Comparative functional enrichment analysis of GMØ (monocyte-derived macrophages differentiated with GM-CSF) and MMØ (monocyte-derived macrophages differentiated with M-CSF) relative to Mo (monocytes at 3 hours). Functional enrichment analysis was performed using the StringApp (STRING database) within Cytoscape (version 3.10.3)[1], focusing on Gene Ontology (GO) Biological Process (BP) terms[2,3]. Red-highlighted cells indicate Gene Ontology Biological Process terms shared between GMØ and MMØ compared to Mo, whereas non-highlighted cells represent subtype-specific enrichment.

270	27	GO Biological Process	Alcohol metabolic process	0.0289	1|PARK7|GOT1|LOC101110434|ALDH1A1|INPP5K|SYNJ1|W5PY08_SHEE	0.0011	GO:0006066
149	18	GO Biological Process	Response to tumor necrosis factor	0.0289	535|LOC100101238|W5P3V8_SHEEP|BRCA1|CXCL16|ASAH1|FABP4|CCL	0.0011	GO:0034612
46	9	GO Biological Process	Positive regulation of interleukin-8 production	0.0294	CHI3L1|RIPK1|CD74|PARK7|PRKD2|TLR2|RAB1A|CD58|CD14	0.0011	GO:0032757
163	19	GO Biological Process	Ras protein signal transduction	0.0312	3|ARHGEF11|AIF1|RASGRP2|RGL2|RAP2C|RRAS|RAP2B|CYFIP1|TAX1BP	0.0012	GO:0007265
20	6	GO Biological Process	Peptide antigen assembly with MHC class II protein complex	0.0314	SHEEP|LOC101109746|W5Q2C4_SHEEP|W5Q2V1_SHEEP|W5Q3J8_SHEEP	0.0012	GO:0002503
37	8	GO Biological Process	Lipid storage	0.0314	STARD4|STAT5A|GBA|GM2A|ENPP1|SOAT1|W5Q6N3_SHEEP|HEXA	0.0012	GO:0019915
418	37	GO Biological Process	Secretion	0.0314	PARK7|EXOC6B|NIBAN2|SLC9A3R1|RAB3A|LOC101122662|MYO1G|MEI	0.0012	GO:0046903
113	15	GO Biological Process	Proton transmembrane transport	0.0314	25A13|ATP6V1G1|LOC101102454|ATP6V0C|ATP6V1B2|ATP6V1E1|ATP6V	0.0012	GO:1902600
20	6	GO Biological Process	Regulation of cytoplasmic transport	0.0314	DENND10|MAPK3|EIPR1|DNAJC13|DAB2|SNX3	0.0012	GO:1903649
37	8	GO Biological Process	Regulation of endothelial cell apoptotic process	0.0314	FGB|CCL2|PDCD4|FGG|FGA|ITGA4|MAPK7|THBS1	0.0012	GO:2000351
37	8	GO Biological Process	Positive regulation of reactive oxygen species metabolic process	0.0314	SOD1-2|PLCG2|ITGAM|PARK7|GRB2|ITGB2|TSPO|THBS1	0.0012	GO:2000379
217	23	GO Biological Process	Negative regulation of cell migration	0.0319	N2|CNN2|PADI2|LOC101105107|SLC9A3R1|RAP2C|KANK1|RRAS|PRKG1	0.0012	GO:0030336
90	13	GO Biological Process	Ceramide metabolic process	0.0322	HEEP|GBA|NEU1|HEXB|LOC101112162|SGPL1|GM2A|ASAH1|CERT1|NA	0.0012	GO:0006672
231	24	GO Biological Process	Negative regulation of endopeptidase activity	0.0322	5|W5P9M9_SHEEP|LOC101105044|MMP9|W5PHI7_SHEEP|BIN1-2|PARK	0.0012	GO:0010951
57	10	GO Biological Process	Cortical cytoskeleton organization	0.0322	RAC1|EPB41|WDR1|FMNL2|EPB41L3|TLN1|PLEC|FMNL3|PLEK|DLG1	0.0012	GO:0030865
164	19	GO Biological Process	Positive regulation of ion transport	0.0322	1|PLA2R1|PLCG2|ATP1B1|CCL2|FHL1|SRI|MS4A1|P2RX4|ATP2B1|TRPV2	0.0012	GO:0043270
177	20	GO Biological Process	Regulation of cysteine-type endopeptidase activity	0.0322	MMP9|ALOX12|PARK7|CYFIP2|VCP|LGMN|HIP1|CAAP1|AIFM1|PPM1F	0.0012	GO:2000116
90	13	GO Biological Process	Regulation of reactive oxygen species metabolic process	0.0322	1-2|BRCA1|SIRT2|PLCG2|ITGAM|CYP1B1|PARK7|GRB2|PRDX2|ITGB2|T	0.0012	GO:2000377
204	22	GO Biological Process	Exocytosis	0.0323	11B|LOC101114535|SCIN|EXOC7|UNC13D|EXOC6B|RAB3A|MYO1G|YKT	0.0012	GO:0006887
47	9	GO Biological Process	Regulation of lipid storage	0.0323	V5NRI1_SHEEP|TREM2|MSR1|LPL|ITGB3|OSBPL8|ITGAV|SCARB1|OSBPL	0.0012	GO:0010883
558	46	GO Biological Process	Regulation of MAPK cascade	0.0323	4|ALOX15|PLCG2|MAP4K2|PTPN11|CCL2|GRB2|PRKD2|PRDX2|TBC1D1	0.0013	GO:0043408
47	9	GO Biological Process	Positive regulation of receptor-mediated endocytosis	0.0323	RI1_SHEEP|HFE|W5P7F8_SHEEP|PLCG2|DAB2|HIP1|LOC101113728|CBL	0.0012	GO:0048260
13	5	GO Biological Process	Positive regulation of phagocytosis, engulfment	0.0323	W5NRI1_SHEEP|TREM2|STAP1|PLCG2|APPL2	0.0012	GO:0060100
68	11	GO Biological Process	Phagocytosis, engulfment	0.0328	M2|RAC1|GSN|AIF1|MSR1|MFGE8|ARHGAP12|BIN2|MYH9|THBS1|FCG	0.0013	GO:0006911
178	20	GO Biological Process	Response to lipopolysaccharide	0.0334	5|STAP1|GIT1|PLCG2|CCL2|PDCD4|PRDX2|VIM|MRC1|NFKB1|ADAM17	0.0013	GO:0032496
2442	156	GO Biological Process	Cellular component biogenesis	0.0337	RAP|ALDH9A1|ITGB3|TLN1|PATL1|SUB1|VCP|SLC9A3R1|CRYZ|H4C14|IN	0.0013	GO:0044085
91	13	GO Biological Process	Polyol metabolic process	0.0343	3D1|GPD2|ASAH1|PLCG2|GOT1|INPP5K|SYNJ1|OCRL|IMPA1|NAAA|GK|	0.0013	GO:0019751
38	8	GO Biological Process	Cortical actin cytoskeleton organization	0.0345	EPB41|FMNL2|EPB41L3|TLN1|PLEC|FMNL3|PLEK|DLG1	0.0014	GO:0030866
304	29	GO Biological Process	Organic cyclic compound catabolic process	0.0351	TPP1|BLVRB|SMPDL3A|HSD17B6|DNASE2|HNMT|ZC3H4|PATL1|VCP|CN	0.0014	GO:1901361
48	9	GO Biological Process	Nuclear envelope organization	0.0358	CHMP6|LMNA|SUN1|TOR1A|TOR1B|LMNB2|SUN2|LMNB1|ZMPSTE24	0.0014	GO:0006998
69	11	GO Biological Process	Pigmentation	0.0358	3|VHL|W5P7F8_SHEEP|DCTN2|HPS5|FIG4|VPS33A|ENPP1|AP3D1|HPS6	0.0014	GO:0043473
48	9	GO Biological Process	Negative regulation of wound healing	0.0358	FGB|CPB2|PLAU|ALOX12|PRKG1|EPPK1|PROC|FGG|FGA	0.0014	GO:0061045
128	16	GO Biological Process	Adaptive immune response based on somatic recombination of immu	0.0361	9|CD74|TAP2|CD55|C8B|UNC13D|W5PWS0_SHEEP|MYO1G|W5PYS8_SH	0.0014	GO:0002460
21	6	GO Biological Process	Negative regulation of extrinsic apoptotic signaling pathway via death	0.0364	FGB|BRCA1|PEA15|PARK7|FGG|FGA	0.0015	GO:1902042
439	38	GO Biological Process	Regulation of secretion	0.0367	PN11|SRI|C1QTNF3|SLC9A3R1|ITGB2|MPC2|FES|RNASE4|RAB3A|PPP3C	0.0015	GO:0051046
335	31	GO Biological Process	Purine-containing compound metabolic process	0.0375	PRPSAP1|ALDOB|LOC101102454|PAPSS1|ATP1B1|GCDH|ATP6V1B2|AK1	0.0015	GO:0072521
39	8	GO Biological Process	Protein targeting to vacuole	0.039	VPS37B|LAMP2|AP3D1|ZFYVE16|GNPTAB|SCARB2|VPS41|NEDD4	0.0016	GO:0006623
39	8	GO Biological Process	Endodermal cell differentiation	0.039	MMP9|GRB2|ITGB2|ITGAV|ITGA4|MMP2|MAP2K1|MMP14	0.0016	GO:0035987
39	8	GO Biological Process	Regulation of focal adhesion assembly	0.039	PEAK1|VCL|ARHGAP6|RCC2|PPM1F|ACTG1|MACF1|S100A10	0.0016	GO:0051893
39	8	GO Biological Process	Protein localization to lysosome	0.039	GLMP|LMBRD1|LAMP2|AP3D1|ZFYVE16|GNPTAB|SCARB2|NEDD4	0.0016	GO:0061462
155	18	GO Biological Process	Purine-containing compound biosynthetic process	0.0393	SS2|W5P7K2_SHEEP|PRPSAP1|LOC101102454|PAPSS1|GCDH|AK1|HPRT	0.0016	GO:0072522
381	34	GO Biological Process	Positive regulation of apoptotic process	0.0394	A1|NCK1|MMP9|PEA15|CYP1B1|ALOX12|BIN1-2|CCL2|EMILIN2|PARK7|	0.0016	GO:0043065
30	7	GO Biological Process	Cell-substrate junction assembly	0.0396	PEAK1|RCC2|ITGB3|TLN1|PLEC|TNS1|ACTN1	0.0016	GO:0007044
322	30	GO Biological Process	Ribonucleotide metabolic process	0.0396	ALDOB|LOC101102454|PAPSS1|ATP1B1|GCDH|ATP6V1B2|AK1|UCK2|H	0.0016	GO:0009259
14	5	GO Biological Process	Relaxation of muscle	0.0397	SOD1-2|GSN|ATP1B1|PRKG1|P2RX4	0.0016	GO:0090075
14	5	GO Biological Process	Regulation of early endosome to late endosome transport	0.0397	DENND10|MAPK3|DNAJC13|DAB2|SNX3	0.0016	GO:2000641
294	28	GO Biological Process	Regulation of lipid metabolic process	0.0411	|MTMR3|PSAP|CD74|ASAH1|H6PD|FABP5|PLCG2|SF1|NFKB1|PLA2G6|	0.0017	GO:0019216
82	12	GO Biological Process	Positive regulation of hormone secretion	0.0411	3|HFE|SPP1|PTPN11|SRI|C1QTNF3|MPC2|PLA2G6|FGG|FGA|AACS|VAN	0.0017	GO:0046887
82	12	GO Biological Process	Cellular oxidant detoxification	0.0411	|PRDX1|NQO1|SOD1-2|SOD2|MPO|PARK7|PRXL2A|GSR|PRDX2|PRDX6	0.0017	GO:0098869
309	29	GO Biological Process	Negative regulation of organelle organization	0.0418	03|SPTAN1|FEZ2|EML2|ARHGAP6|FKBP4|LOC101105107|INPP5K|KANK1	0.0017	GO:0010639
71	11	GO Biological Process	Positive regulation of exocytosis	0.0419	3|LAMP1|ITGAM|UNC13D|ITGB2|RAB3A|FGG|FGA|RAB9A|VAMP8|RAB	0.0017	GO:0045921
71	11	GO Biological Process	Glycosyl compound metabolic process	0.0419	|GUSB|FUCA2|GLA|ADA|FUCA1|W5PKU3_SHEEP|HPRT1|LOC101110434	0.0017	GO:1901657
50	9	GO Biological Process	Zymogen activation	0.0434	FGB|CTSC|PLAU|MSR1|CYFIP2|LGMN|FGG|FGA|MMP14	0.0018	GO:0031638
83	12	GO Biological Process	Nucleoside monophosphate metabolic process	0.0443	5|ADA|ADSS2|W5P7K2_SHEEP|UCK1|TYMS|AK1|UCK2|HPRT1|DCK|AK3	0.0019	GO:0009123
83	12	GO Biological Process	Mononuclear cell migration	0.0443	01114535|LOC100101238|W5P3V8_SHEEP|ITGAL|CXCL16|CCL2|MYO1G	0.0019	GO:0071674
8	4	GO Biological Process	N-acetylneuraminate catabolic process	0.0446	RENBP|GNPDA1|W5QB70_SHEEP|NPL	0.0019	GO:0019262

Supplementary Table 11: Comparative functional enrichment analysis of GMØ (monocyte-derived macrophages differentiated with GM-CSF) and MMØ (monocyte-derived macrophages differentiated with M-CSF) relative to Mo (monocytes at 3 hours). Functional enrichment analysis was performed using the StringApp (STRING database) within Cytoscape (version 3.10.3)[1], focusing on Gene Ontology (GO) Biological Process (BP) terms[2,3]. Red-highlighted cells indicate Gene Ontology Biological Process terms shared between GMØ and MMØ compared to Mo, whereas non-highlighted cells represent subtype-specific enrichment.

8	4	GO Biological Process	Positive regulation of neuroinflammatory response	0.0446	TREM2|CTSC|STAP1|PLCG2	0.0019	GO:0150078
107	14	GO Biological Process	Actomyosin structure organization	0.0448	MYL12A|EPB41L3|CNN2|MYL9|PLEC|CNN3|ZYX|ACTG1|MYH9|ACTC1|T	0.0019	GO:0031032
7312	413	GO Biological Process	Primary metabolic process	0.0448	4|INPPL1|HEXB|CNDP2|RNF13|LOC101112162|WDR45B|BRCA1|NAGPA	0.0019	GO:0044238
132	16	GO Biological Process	Sphingolipid metabolic process	0.0451	NEU1|HEXB|LOC101112162|SGPL1|PSAP|SMPDL3A|GM2A|ASAH1|NAAA	0.0019	GO:0006665
61	10	GO Biological Process	Regulation of interleukin-8 production	0.0451	CHI3L1|RIPK1|CD74|PARK7|PRKD2|TLR2|ANXA4|RAB1A|CD58|CD14	0.0019	GO:0032677
61	10	GO Biological Process	Protein localization to vacuole	0.0451	P|VPS37B|LMBRD1|LAMP2|AP3D1|ZFYVE16|GNPTAB|SCARB2|VPS41|N	0.0019	GO:0072665
72	11	GO Biological Process	Autophagosome organization	0.0451	DR45B|TRAPPC8|GABARAP|ATG7|TCIRG1|STBD1|STING1|ARFIP2|ATG3	0.0019	GO:1905037
145	17	GO Biological Process	Glial cell differentiation	0.0453	GBA|SOD1-2|NDRG1|SIRT2|PTPN11|VIM|SH3TC1|CUL4B|PLEC|ASPA|IL	0.0019	GO:0010001
3	3	GO Biological Process	CMP biosynthetic process	0.0454	UCK1|UCK2|DCK	0.0019	GO:0009224
3	3	GO Biological Process	Glucuronoside catabolic process	0.0454	GUSB|W5PKU3_SHEEP|ABHD10	0.0019	GO:0019391
84	12	GO Biological Process	Positive regulation of endothelial cell migration	0.0471	LOX12|PRKD2|ITGB3|LGMN|RRAS|HSPB1|ADAM17|P2RX4|PRKCA|ANX	0.002	GO:0010595
41	8	GO Biological Process	Myeloid leukocyte mediated immunity	0.0478	S100A12|STXBP2|LOC101114535|CARD9|WDR1|ANXA3|VAMP2|FCGR1A	0.0021	GO:0002444
41	8	GO Biological Process	Positive regulation of blood vessel endothelial cell migration	0.0478	STAT5A|ALOX12|PRKD2|HSPB1|ADAM17|P2RX4|PRKCA|THBS1	0.0021	GO:0043536
41	8	GO Biological Process	Negative regulation of sequestering of calcium ion	0.0478	LOC101114535|ITPR1|ANXA6|TPCN1|PLCG2|ANXA5|TPCN2|CHERP	0.0021	GO:0051283
15	5	GO Biological Process	Arginine metabolic process	0.0479	ASL|DDAH2|ASS1|OAT|FAH	0.0021	GO:0006525
15	5	GO Biological Process	Amino sugar catabolic process	0.0479	RENBP|GNPDA1|ALDH1A1|W5QB70_SHEEP|NPL	0.0021	GO:0046348
186	20	GO Biological Process	Positive regulation of T cell activation	0.048	|LOC101109746|AIF1|PPP3CA|LGALS1|SASH3|W5Q2C4_SHEEP|W5Q2V1	0.0021	GO:0050870
62	10	GO Biological Process	Negative regulation of response to wounding	0.0487	FGB|GIT1|CPB2|PLAU|ALOX12|PRKG1|EPPK1|PROC|FGG|FGA	0.0021	GO:1903035
23	6	GO Biological Process	Nucleotide salvage	0.0489	NAPRT|ADA|UCK1|UCK2|HPRT1|DCK	0.0021	GO:0043173
23	6	GO Biological Process	Positive regulation of calcium-mediated signaling	0.0489	TREM2|ADA|LOC101114535|PLCG2|P2RX4|CHERP	0.0021	GO:0050850
134	16	GO Biological Process	Plasma membrane organization	0.0497	LR2|SOD1-2|NDRG1|SNX18|ANXA6|BIN1-2|SLC9A3R1|SH3TC1|RAB3A|I	0.0022	GO:0007009
160	18	GO Biological Process	Regulation of cysteine-type endopeptidase activity involved in apopto	0.0497	9|GSN|MMP9|ALOX12|PARK7|VCP|HIP1|CAAP1|AIFM1|PPM1F|ATP2A	0.0022	GO:0043281

Red-highlighted cells indicate Gene Ontology Biological Process terms shared between GMØ and MMØ compared to M0, whereas non-highlighted cells represent subtype-specific enrichment.

Supplementary Table 12: Enrichment map analysis of differentially abundant proteins (DAPs) in cMØ compared to M0 (monocytes at 3 hours), presenting significantly enriched Gene Ontology biological process (GO-BP) terms and canonical pathways. Functional enrichment analysis was performed using the StringApp (STRING database) within Cytoscape (version 3.10.3)[1], focusing on Gene Ontology (GO) Biological Process (BP) terms and canonical pathways from the KEGG and Reactome databases[2–6]. Enriched GO terms were clustered as functionally coherent groups using the EnrichmentMap plugin[7]. . Statistical significance was determined based on a false discovery rate (FDR) threshold of < 0.05.

EnrichmentMap analysis of cMØ_M0_ Biological Process

Annatotation	Nodes	EnrichmentMap name	EnrichmentMap GS_DESCR	EnrichmentMap Genes	EnrichmentMap gs_size	EnrichmentMap -log10 (pv	EnrichmentMap: pvalue (Da	EnrichmentMap::fdr_qv	EnrichmentMap::NES (Data	EnrichmentMap::-log10 (p	EnrichmentMap::-lo	EnrichmentMap colouring	_mdcluster
		GO:0046907	Intracellular transport	PSEN1NPC2W5NSQ8_SHEEPARMC	99	10.15989391	6.92E-11	4.14E-08	0.738299966	10.15989391	10.15989391	1	1
		GO:0006886	Intracellular protein transport	LOC101118849RAB1AW5P023_SH	49	5.221125528	6.01E-06	6.50E-04	0.318708664	5.221125528	5.221125528	0.99999399	1
		GO:0051179	Localization	PSEN1TREM2PIP4K2AGAAMTORIN	267	14.8569852	1.39E-15	3.94E-12	1.140450378	14.8569852	14.8569852	1	1
		GO:0016192	Vesicle-mediated transport	PSEN1TREM2PIP4K2AINPP5FW5P0	119	18.59516628	2.54E-19	1.44E-15	1.484163751	18.59516628	18.59516628	1	1
receptor intracellular endocytosis	9	GO:0072583	Clathrin-dependent endocytosis	PICALMFCHO2INPP5FCLTAAP2A2-2	11	6.642065153	2.28E-07	4.83E-05	0.431605287	6.642065153	6.642065153	0.999999772	1
		GO:0031623	Receptor internalization	PICALMLMBRD1TFRCCLTCITGB2AR	11	4.42136079	3.79E-05	0.0026	0.258502665	4.42136079	4.42136079	0.9999621	1
		GO:0006897	Endocytosis	W5Q6N3_SHEEPTREM2INPP5FRAB	57	13.54975089	2.82E-14	5.52E-11	1.025806092	13.54975089	13.54975089	1	1
		GO:0008104	Protein localization	SEPTIN10PSEN1ARMCX3LOC10111	132	12.66958623	2.14E-13	2.49E-10	0.960380065	12.66958623	12.66958623	1	1
		GO:0006898	Receptor-mediated endocytosis	CTSLW5Q6N3_SHEEPMSR1GRB2AI	27	10.2313619	5.87E-11	4.14E-08	0.738299966	10.2313619	10.2313619	1	1
		GO:0022604	Regulation of cell morphogenesis	MYL12BCORO1BPLXNC1EPS8CD44	25	4.590066877	2.57E-05	0.0021	0.267778071	4.590066877	4.590066877	0.9999743	2
		GO:0048522	Positive regulation of cellular process	CASP6PSEN1TREM2LY96RUNX3PIP	231	3.301029996	5.00E-04	0.0196	0.170774393	3.301029996	3.301029996	0.9995	2
		GO:0022603	Regulation of anatomical structure morphoge	PSEN1MYL12BWARS1SARM1HMO)	51	4.226213555	5.94E-05	0.0036	0.24436975	4.226213555	4.226213555	0.9999406	2
Regulation of response to stimulus	8	GO:0051239	Regulation of multicellular organismal proces	PSEN1CD109TREM2LY96RTN4PLD3	132	6.402304814	3.96E-07	7.76E-05	0.411013828	6.402304814	6.402304814	0.999999604	2
		GO:0048583	Regulation of response to stimulus	CASP6PSEN1CD109C20ORF27TREN	169	3.958607315	1.10E-04	0.0061	0.221467016	3.958607315	3.958607315	0.99989	2
		GO:0001817	Regulation of cytokine production	PSEN1TREM2LY96PLD3IL18RGCCR/	54	7.634512015	2.32E-08	7.12E-06	0.514752001	7.634512015	7.634512015	0.999999977	2
		GO:0050793	Regulation of developmental process	PSEN1MYL12BCD109WDFY2TREM:	107	3.37675071	4.20E-04	0.0169	0.17721133	3.37675071	3.37675071	0.99958	2
		GO:0010628	Positive regulation of gene expression	PSEN1NCLWARS1TREM2LY96IL18D	64	4.517126416	3.04E-05	0.0023	0.263827216	4.517126416	4.517126416	0.9999696	2
		GO:0030155	Regulation of cell adhesion	PLEKHA2CD86ALOX15CYP1B1PLXN	48	4.723538196	1.89E-05	0.0016	0.279588002	4.723538196	4.723538196	0.9999811	3
		GO:0002682	Regulation of immune system process	CASP6PSEN1SARM1TREM2LY96RTI	92	8.543633967	2.86E-09	1.12E-06	0.595078198	8.543633967	8.543633967	0.999999997	3
cell adhesion leukocyte	6	GO:1903039	Positive regulation of leukocyte cell-cell adhe	CD86CD274LGALS1CD74CORO1AIL	21	3.886056648	1.30E-04	0.0069	0.216115091	3.886056648	3.886056648	0.99987	3
		GO:0070663	Regulation of leukocyte proliferation	CD86MAPK1CD274CD74CORO1AIL	21	3.886056648	1.30E-04	0.0069	0.216115091	3.886056648	3.886056648	0.99987	3
		GO:0050865	Regulation of cell activation	CD86LAMP1TREM2W5Q3J8_SHEEI	46	5.823908741	1.50E-06	2.10E-04	0.367778071	5.823908741	5.823908741	0.9999985	3
		GO:0050866	Negative regulation of cell activation	CD86CD274PRKG1TREM2CD74FGR	20	4.425968732	3.75E-05	0.0026	0.258502665	4.425968732	4.425968732	0.9999625	3
		GO:0032879	Regulation of localization	PSEN1TREM2RTN4DYNC1H1RSL1D	120	9.425968732	3.75E-10	1.80E-07	0.674472749	9.425968732	9.425968732	1	5
		GO:0030100	Regulation of endocytosis	ALOX15PLCG2ITGAVAPPL2TREM2D	23	6.581698709	2.62E-07	5.42E-05	0.426600071	6.581698709	6.581698709	0.999999738	5
regulation transport localization	5	GO:0060627	Regulation of vesicle-mediated transport	TREM2INPP5FPPT1W5P023_SHEEF	52	10.73518218	1.84E-11	1.49E-08	0.782681373	10.73518218	10.73518218	1	5
		GO:0051050	Positive regulation of transport	PSEN1TREM2DYNC1H1SPP1RGCCR	52	5.375717904	4.21E-06	4.90E-04	0.330980392	5.375717904	5.375717904	0.99999579	5
		GO:0060341	Regulation of cellular localization	PSEN1TREM2RTN4DYNC1H1MYCB	54	4.412289035	3.87E-05	0.0027	0.256863624	4.412289035	4.412289035	0.9999613	5
		GO:0065009	Regulation of molecular function	PSEN1RENBPCD109TREM2PIP4K2A	154	6.316052869	4.83E-07	9.29E-05	0.403198429	6.316052869	6.316052869	0.999999517	4
		GO:0051248	Negative regulation of protein metabolic proc	PSEN1WARS1CTSACD109NQO1TRE	56	3.193820026	6.40E-04	0.024	0.161978876	3.193820026	3.193820026	0.99936	4
regulation molecular function	5	GO:0051246	Regulation of protein metabolic process	PSEN1RENBPGLYR1CTSACD109WD	125	3.958607315	1.10E-04	0.0058	0.223657201	3.958607315	3.958607315	0.99989	4
		GO:0001932	Regulation of protein phosphorylation	PSEN1WARS1CD109WDFY2TREM2	58	3.420216403	3.80E-04	0.0158	0.180134291	3.420216403	3.420216403	0.99962	4
		GO:0044092	Negative regulation of molecular function	PSEN1RENBPWARS1CD109NQO1P	59	3.283996656	5.20E-04	0.0204	0.169036983	3.283996656	3.283996656	0.99948	4
		GO:0019637	Organophosphate metabolic process	MTMR3HINT1SARM1DHODHOXSM	55	4.096910013	8.00E-05	0.0046	0.233724217	4.096910013	4.096910013	0.99992	7
phosphorus metabolic nucleobase	4	GO:1901135	Carbohydrate derivative metabolic process	W5NRS0_SHEEPPSEN1W5NS94_SH	74	8.823908741	1.50E-09	6.37E-07	0.619586057	8.823908741	8.823908741	0.999999999	7
		GO:0006793	Phosphorus metabolic process	MTMR3CD109GNPDA1RUNX3PIP4	112	6.273272791	5.33E-07	9.76E-05	0.401055018	6.273272791	6.273272791	0.999999467	7
		GO:0055086	Nucleobase-containing small molecule metab	W5NRS0_SHEEPATP6V1B2W5NS94	39	4.793174124	1.61E-05	0.0014	0.285387196	4.793174124	4.793174124	0.9999839	7
		GO:0046466	Membrane lipid catabolic process	SGPL1NEU1ASAH1FUCA1HEXBGLA	12	6.821023053	1.51E-07	3.49E-05	0.445717457	6.821023053	6.821023053	0.999999849	6
lipid ganglioside catabolic	4	GO:0006689	Ganglioside catabolic process	NEU1HEXBLOC101112162HEXAGM	5	4.322393047	4.76E-05	0.0031	0.250863831	4.322393047	4.322393047	0.9999524	6
		GO:0044242	Cellular lipid catabolic process	ACOX1ASAH1ABCD3PLCG2GLACYP	25	6.647817482	2.25E-07	4.83E-05	0.431605287	6.647817482	6.647817482	0.999999775	6
		GO:0044255	Cellular lipid metabolic process	MTMR3ABCD3EPHX1GLAAGPAT5O	62	4.844663963	1.43E-05	0.0013	0.288605665	4.844663963	4.844663963	0.9999857	6
		GO:0019262	N-acetylneuraminate catabolic process	RENBPGNPDA1W5QB70_SHEEPNP	4	3.107905397	7.80E-04	0.0279	0.15543958	3.107905397	3.107905397	0.99922	12
udp acetylglucosamine sugar	3	GO:0006040	Amino sugar metabolic process	PGM3ALDH1A1W5NS94_SHEEPREI	10	5.188424994	6.48E-06	6.90E-04	0.316115091	5.188424994	5.188424994	0.99999352	12
		GO:0006048	UDP-N-acetylglucosamine biosynthetic proce	PGM3W5NS94_SHEEPGNPNAT1GN	4	2.958607315	0.0011	0.0365	0.143770714	2.958607315	2.958607315	0.9989	12
		GO:0070887	Cellular response to chemical stimulus	CASP6PSEN1TREM2LY96ATP6V0D1	129	7.030584088	9.32E-08	2.20E-05	0.465757732	7.030584088	7.030584088	0.999999907	13
oxygen chemical organonitrogen	3	GO:1901700	Response to oxygen-containing compound	PSEN1SARM1NQO1TREM2LY96SHI	68	5.168130226	6.79E-06	7.10E-04	0.314874165	5.168130226	5.168130226	0.99999321	13
		GO:0010243	Response to organonitrogen compound	CASP6PSEN1VIMERLIN2TREM2SHN	47	4.14691047	7.13E-05	0.0042	0.237675071	4.14691047	4.14691047	0.9999287	13
		GO:0006575	Cellular modified amino acid metabolic proce	DHFRETHE1ALDH9A1IDH1SHMT1G	22	5.991399828	1.02E-06	1.60E-04	0.379588002	5.991399828	5.991399828	0.99999898	10
modified glutathione sulfur	3	GO:0006749	Glutathione metabolic process	CLIC2PARK7LOC101116273ETHE1II	10	3.657577319	2.20E-04	0.0103	0.198716278	3.657577319	3.657577319	0.99978	10
		GO:0006790	Sulfur compound metabolic process	ENOPH1ETHE1IDH1MPSTOXSMAH	23	2.958607315	0.0011	0.0359	0.144490555	2.958607315	2.958607315	0.9989	10
		GO:0006508	Proteolysis	CASP6USP30PSEN1ERLIN2CTSACTS	77	3.585026652	2.60E-04	0.0119	0.192445304	3.585026652	3.585026652	0.99974	9
macromolecule process proteolysis	3	GO:0009057	Macromolecule catabolic process	USP30ERLIN2SGSHCD44W5NUX2_	64	5.003926346	9.91E-06	9.70E-04	0.301322827	5.003926346	5.003926346	0.99999009	9
		GO:0009056	Catabolic process	PSEN1ERLIN2RENBPFUCA2GLAGNI	154	19.94309515	1.14E-20	1.29E-16	1.588941029	19.94309515	19.94309515	1	9
		GO:0044419	Biological process involved in interspecies int	NPC2FUCA2SARM1HMOX1TREM2I	85	6.684029655	2.07E-07	4.52E-05	0.434486157	6.684029655	6.684029655	0.999999793	11
involved interspecies interaction	3	GO:0045087	Innate immune response	VIMPLCG2SARM1PQBP1LY96CCL8I	39	3.455931956	3.50E-04	0.0149	0.182681373	3.455931956	3.455931956	0.99965	11
		GO:0009617	Response to bacterium	CD86VIMFUCA2MAPKAPK3PLCG2L	39	3.795880017	1.60E-04	0.008	0.209691001	3.795880017	3.795880017	0.99984	11
		GO:0051493	Regulation of cytoskeleton organization	ALOX15CAPGTPX2HSPA1A-2CORO1	39	4.41453927	3.85E-05	0.0027	0.256863624	4.41453927	4.41453927	0.9999615	8
complex assembly size	3	GO:0032535	Regulation of cellular component size	ALOX15CAPGEPS8RTN4GRB2FAM4	27	3.721246399	1.90E-04	0.0092	0.203621217	3.721246399	3.721246399	0.99981	8
		GO:0043254	Regulation of protein-containing complex ass	W5PNU4_SHEEPALOX15CAPGWAR	36	6.030118356	9.33E-07	1.50E-04	0.382390874	6.030118356	6.030118356	0.999999067	8
regulation gene expression	2	GO:0001818	Negative regulation of cytokine production	ERBINAPPL2TREM2CD2APW5P2V0	19	3.180456064	6.60E-04	0.0246	0.160906489	3.180456064	3.180456064	0.99934	22
		GO:0010629	Negative regulation of gene expression	PSEN1W5PNU4_SHEEPTREM2SHM	47	2.823908741	0.0015	0.0457	0.13400838	2.823908741	2.823908741	0.9985	22
		GO:0019886	Antigen processing and presentation of exoge	HLA-DRAIFI30CD74CTSSW5Q3J8_S	6	3.036212173	9.20E-04	0.0321	0.149349497	3.036212173	3.036212173	0.99908	18
processing presentation antigen	2	GO:0019882	Antigen processing and presentation	CD74W5Q3J8_SHEEPCALRRAB35H	14	4.448550002	3.56E-05	0.0026	0.258502665	4.448550002	4.448550002	0.9999644	18
		GO:0007035	Vacuolar acidification	ATP6V1B2ATP6V0A1PPT1ATP6V0D	7	4.432973634	3.69E-05	0.0026	0.258502665	4.432973634	4.432973634	0.9999631	21
ph reduction vacuolar	2	GO:0045851	pH reduction	ATP6V1B2ATP6V1C1ATP6V0A1ATP(	6	4.452225295	3.53E-05	0.0026	0.258502665	4.452225295	4.452225295	0.9999647	21
monovalent cation homeostasis	2	GO:0055067	Monovalent inorganic cation homeostasis	ATP6V1B2MAPK1ATP6V1C1ATP6V(	20	6.262012674	5.47E-07	9.85E-05	0.400656377	6.262012674	6.262012674	0.999999453	17
		GO:0006873	Cellular ion homeostasis	ANXA6ATP6V1B2PSEN1ATP6V0A1H	35	5.8569852	1.39E-06	2.10E-04	0.367778071	5.8569852	5.8569852	0.99999861	17
lipid modification phosphatidylinositol	2	GO:0046856	Phosphatidylinositol dephosphorylation	OCRLMTMR3MTMR6INPP5FSYNJ1	7	3.180456064	6.60E-04	0.0246	0.160906489	3.180456064	3.180456064	0.99934	20
		GO:0030258	Lipid modification	ACOX1MTMR3MTMR6SOAT1ALOX	17	3.15490196	7.00E-04	0.0254	0.159516628	3.15490196	3.15490196	0.9993	20
		GO:0031349	Positive regulation of defense response	CASP6LAMP1EMILIN2PLCG2PQBP1	23	4.350665141	4.46E-05	0.003	0.252287875	4.350665141	4.350665141	0.9999554	14
defense positive inflammatory	2	GO:0050727	Regulation of inflammatory response	NLRP2NLRP3ALOX15PLCG2CD200F	22	3.045757491	9.00E-04	0.0314	0.150307035	3.045757491	3.045757491	0.9991	14
		GO:0009987	Cellular process	PSEN1ERLIN2RENBPGLAGNPDA1TF	700	10.21681131	6.07E-11	4.14E-08	0.738299966	10.21681131	10.21681131	1	16
cellular metabolic process	2	GO:0044237	Cellular metabolic process	PSEN1MTMR3ERLIN2RENBPGLACD	337	8.257274869	5.53E-09	2.03E-06	0.569250396	8.257274869	8.257274869	0.999999994	16
		GO:1990748	Cellular detoxification	ALDH1A1NQO1SOD2PRDX1PRDX6(	17	5.841637508	1.44E-06	2.10E-04	0.367778071	5.841637508	5.841637508	0.99999856	19
cell redox homeostasis	2	GO:0045454	Cell redox homeostasis	PRDX4APEX1TXNRD1NQO1PRDX2(	11	6.302770657	4.98E-07	9.29E-05	0.403198429	6.302770657	6.302770657	0.999999502	19
		GO:0008154	Actin polymerization or depolymerization	CAPZBCAPGGAS7GSNTWF1WIPF1P	9	3.055517328	8.80E-04	0.031	0.150863831	3.055517328	3.055517328	0.99912	15
barbed end actin	2	GO:0051016	Barbed-end actin filament capping	CAPZBCAPGADD1GSNCAPZA2TWF1	8	5.205511953	6.23E-06	6.70E-04	0.31739252	5.205511953	5.205511953	0.99999377	15

Supplementary Table 12: Enrichment map analysis of differentially abundant proteins (DAPs) in cMØ compared to M0 (monocytes at 3 hours), presenting significantly enriched Gene Ontology biological process (GO-BP) terms and canonical pathways. Functional enrichment analysis was performed using the StringApp (STRING database) within Cytoscape (version 3.10.3)[1], focusing on Gene Ontology (GO) Biological Process (BP) terms and canonical pathways from the KEGG and Reactome databases[2–6]. Enriched GO terms were clustered as functionally coherent groups using the EnrichmentMap plugin[7]. . Statistical significance was determined based on a false discovery rate (FDR) threshold of < 0.05.

EnrichmentMap analysis of cMØ_M0_ Pathway

Annatotation	Nodes	EnrichmentMap name	EnrichmentMap GS_DESCR	EnrichmentMap Genes	EnrichmentMap gs_size	EnrichmentMap -log10 (pv	EnrichmentMap: pvalue (Da	EnrichmentMap::fdr_qv	EnrichmentMap::NES (Data	EnrichmentMap::-log10 (p	EnrichmentMap::-lo	EnrichmentMap colouring	_mdcluster
		oas00600	Sphingolipid metabolism	SGPL1GBAGLANEU1GLB1ASAH1SP	8	3.537602002	2.90E-04	3.40E-03	0.246852108	3.537602002	3.537602002	0.99971	1
		MAP-2024101	CS/DS degradation	LOC101112162HEXAVCANIDUAHE	5	2.585026652	2.60E-03	4.24E-02	0.137263414	2.585026652	2.585026652	0.9974	1
glycosphingolipid biosynthesis series	5	oas00603	Glycosphingolipid biosynthesis - globo and isc	LOC101112162HEXAGLANAGAHEX	5	3.251811973	5.60E-04	5.50E-03	0.225963731	3.251811973	3.251811973	0.99944	1
		oas00511	Other glycan degradation	LOC101112162FUCA2GBAHEXANE	9	6.42945706	3.72E-07	1.37E-05	0.486327943	6.42945706	6.42945706	0.999999628	1
		MAP-1660662	Glycosphingolipid metabolism	LOC101112162GBAHEXAGLAGM2A	12	4.534617149	2.92E-05	1.50E-03	0.282390874	4.534617149	4.534617149	0.9999708	1
		MAP-9639288	Amino acids regulate mTORC1	TCIRG1ATP6V1FATP6V0D1LAMTOR	14	5.299296283	5.02E-06	3.70E-04	0.343179828	5.299296283	5.299296283	0.99999498	5
amino acids regulate	3	MAP-917937	Iron uptake and transport	FTH1TCIRG1ATP6V1FHMOX1ATP6V	17	4.879426069	1.32E-05	8.10E-04	0.309151498	4.879426069	4.879426069	0.9999868	5
		oas04721	Synaptic vesicle cycle	ATP6V1FATP6V0D1ATP6V1C1ATP6V	14	5.876148359	1.33E-06	4.41E-05	0.435556141	5.876148359	5.876148359	0.99999867	5
		MAP-437239	Recycling pathway of L1	CLTACLTCSHTN1MAPK1AP2A2-2AP	9	4.950781977	1.12E-05	7.20E-04	0.31426675	4.950781977	4.950781977	0.9999888	4
ldl clearance signaling	3	MAP-8964038	LDL clearance	NPC1CLTACLTCSOAT1LIPANPC2AP2	10	6.005243055	9.88E-07	9.37E-05	0.402826041	6.005243055	6.005243055	0.999999012	4
		MAP-187037	Signaling by NTRK1 (TRKA)	CLTARALAMAPK1PPP2R5DCLTCCSD	14	3.958607315	1.10E-04	0.0039	0.240893539	3.958607315	3.958607315	0.99989	4
		oas04070	Phosphatidylinositol signaling system	PIP4K2AMTMR3IMPA1INPP5FPLCG	12	3.721246399	1.90E-04	0.0029	0.2537602	3.721246399	3.721246399	0.99981	3
phosphatidylinositol signaling system	3	MAP-1483257	Phospholipid metabolism	PIP4K2ACPNE1INPP5FPLEKHA2PIK3	23	2.795880017	1.60E-03	2.92E-02	0.153461715	2.795880017	2.795880017	0.9984	3
		MAP-1660499	Synthesis of PIPs at the plasma membrane	PIP4K2AMTMR3SASH3MTMR6MTI	11	3.337242168	4.60E-04	0.0121	0.191721463	3.337242168	3.337242168	0.99954	3
		oas04370	VEGF signaling pathway	RAC1PLCG2MAPKAPK3MAPK14PPP	8	3.113509275	7.70E-04	6.80E-03	0.216749109	3.113509275	3.113509275	0.99923	2
vegf signaling pathway	3	oas04662	B cell receptor signaling pathway	RAC1IGHMPTPN6PLCG2INPPL1PPP	9	3.200659451	6.30E-04	0.0058	0.223657201	3.200659451	3.200659451	0.99937	2
		oas04625	C-type lectin receptor signaling pathway	PRKCDIL1BPLCG2MAPK14PPP3CBN	9	2.229147988	5.90E-03	0.032	0.149485002	2.229147988	2.229147988	0.9941	2
adaptive immune system	2	MAP-1280218	Adaptive Immune System	CTSSPSME1KIF15AP1B1W5P7L9_S	83	5.752026734	1.77E-06	1.40E-04	0.385387196	5.752026734	5.752026734	0.99999823	6
		MAP-2132295	MHC class II antigen presentation	CTSSHLA-DRAKIF15AP1B1W5P7L9_	35	11.82973828	1.48E-12	3.48E-10	0.945842076	11.82973828	11.82973828	1	6
aggrephagy macroautophagy	2	MAP-9646399	Aggrephagy	DYNC1H1VIMW5NSS8_SHEEPTUBA	8	2.958607315	1.10E-03	0.0237	0.162525165	2.958607315	2.958607315	0.9989	13
		MAP-1632852	Macroautophagy	CHMP2ALAMTOR2TUBB1DYNC1H1	20	4.627087997	2.36E-05	0.0012	0.292081875	4.627087997	4.627087997	0.9999764	13
endocrine factor regulated	2	oas04978	Mineral absorption	TFATOX1ATP1A2ATP1B1ATP1A1ATP	8	3.055517328	8.80E-04	7.50E-03	0.212493874	3.055517328	3.055517328	0.99912	11
		oas04961	Endocrine and other factor-regulated calcium	CLTCATP1A2ATP1B1ATP1A1ATP2B3	9	4.038578906	9.15E-05	0.0017	0.276955108	4.038578906	4.038578906	0.9999085	11
g2 transition phase	2	MAP-68886	M Phase	W5P023_SHEEPCHMP2APSME1NU	37	2.823908741	1.50E-03	2.83E-02	0.154821356	2.823908741	2.823908741	0.9985	8
		MAP-69275	G2/M Transition	PSMB1PSME1PAFAH1B1MAPRE2PS	21	2.958607315	1.10E-03	2.38E-02	0.162342304	2.958607315	2.958607315	0.9989	8
kaposi sarcoma herpesvirus	2	oas05170	Human immunodeficiency virus 1 infection	RAC1RPS6KB2PLCG2TLR2AP1B1GN	16	2.823908741	1.50E-03	1.05E-02	0.19788107	2.823908741	2.823908741	0.9985	12
		oas05167	Kaposi sarcoma-associated herpesvirus infect	RAC1EIF2AK2PLCG2ICAM1CD86GN	15	3	1.00E-03	7.90E-03	0.210237291	3	3	0.999	12
metabolic pathways metabolism	2	MAP-1430728	Metabolism	W5NRS0_SHEEPGLAARF1GAAGNSH	174	13.04431225	9.03E-14	2.57E-11	1.059006688	13.04431225	13.04431225	1	7
		oas01100	Metabolic pathways	W5NRS0_SHEEPGLAATP6V0A1GAA	120	17.3400838	4.57E-18	7.59E-16	1.511975822	17.3400838	17.3400838	1	7
platelet sensitization ldl	2	MAP-418346	Platelet homeostasis	PTPN6FGRPRKG1PECAM1P2RX4MA	12	3.008773924	9.80E-04	0.0217	0.166354027	3.008773924	3.008773924	0.99902	10
		MAP-432142	Platelet sensitization by LDL	FGRPTPN6PECAM1MAPK14PPP2R5	5	2.522878745	3.00E-03	0.0493	0.130715308	2.522878745	2.522878745	0.997	10
retrograde golgi traffic	2	MAP-6807878	COPI-mediated anterograde transport	SPTBN1SPTAN1TMED3YKT6TUBB1A	14	2.886056648	1.30E-03	0.0268	0.157186521	2.886056648	2.886056648	0.9987	9
		MAP-6811442	Intra-Golgi and retrograde Golgi-to-ER traffic	KIF15W5P7L9_SHEEPARF1DYNC1H	25	4.32330639	4.75E-05	0.0021	0.267778071	4.32330639	4.32330639	0.9999525	9

cMØ, monocyte-derived macrophages differentiated without the addition of exogenous growth factors such as M-CSF or GM-CSF and M0, Monocytes at 3 hours.

Supplementary Table 13: Enrichment map analysis of differentially abundant proteins (DAPs) in GMØ compared to M0 (monocytes at 3 hours), presenting significantly enriched Gene Ontology biological process (GO-BP) terms and canonical pathways. Functional enrichment analysis was performed using the StringApp (STRING database) within Cytoscape (version 3.10.3)[1], focusing on Gene Ontology (GO) Biological Process (BP) terms and canonical pathways from the KEGG and Reactome databases[2–6]. Enriched GO terms were clustered as functionally coherent groups using the EnrichmentMap plugin[7]. Statistical significance was determined based on a false discovery rate (FDR) threshold of < 0.05.

EnrichmentMap analysis of GMØ_M0_ Biological Process

Annotation	Nodes	EnrichmentMap name	EnrichmentMap GS_DESCR	EnrichmentMap Genes	hmentMap gs	EnrichmentM	EnrichmentM	EnrichmentM	EnrichmentM	Enrichmen	Enrichmen	Enrichme	_mdcluster
		GO:0071704	Organic substance metabolic process	MPSTHSPA8COL2A1PIP4K2AMETTL3	592	12.109	7.78E-13	4.91E-10	0.93089185	12.10902	12.10902	1	1
		GO:0048583	Regulation of response to stimulus	COL2A1RASGRP4CSF1W5P6T0_SHEI	270	7.017	9.61E-08	1.09E-05	0.49625735	7.017277	7.017277	1	1
		GO:0070887	Cellular response to chemical stimulus	HSPA8COL2A1CSF1IGFBP5GLG1ACTI	213	13.585	2.60E-14	1.97E-11	1.07055338	13.58503	13.58503	1	1
		GO:0009967	Positive regulation of signal transduction	RASGRP4CSF1PARK7MAPRE2ACTN4	110	4.321	4.77E-05	2.30E-03	0.26382722	4.321482	4.321482	0.99995	1
**Chemical stimulus signal**	10	GO:0048585	Negative regulation of response to stimulus	COL2A1IGFBP5PIP4K2APARK7GLG1N	109	2.959	1.10E-03	2.59E-02	0.15867002	2.958607	2.958607	0.9989	1
		GO:0009987	Cellular process	HSPA8RALBP1BET1ARL3NCF2PIP4K2	1090	18.071	8.50E-19	3.21E-15	1.4493495	18.07058	18.07058	1	1
		GO:0006950	Response to stress	HSPA8CSF1W5P6T0_SHEEPW5QEA3	219	5.775	1.68E-06	1.40E-04	0.3853872	5.774691	5.774691	1	1
		GO:0050896	Response to stimulus	HSPA8COL2A1RALBP1CSF1ARL3IGFE	511	4.830	1.48E-05	8.80E-04	0.30555173	4.829738	4.829738	0.99999	1
		GO:0048518	Positive regulation of biological process	HSPA8RALBP1CSF1IGFBP5PIP4K2AR	417	7.541	2.88E-08	3.72E-06	0.54294571	7.540608	7.540608	1	1
		GO:1902531	Regulation of intracellular signal transduction	RASGRP4CSF1DIPK2APARK7MAPRE2	114	3.244	5.70E-04	0.0162	0.1790485	3.244125	3.244125	0.99943	1
		GO:0050790	Regulation of catalytic activity	TBC1D2BRALBP1RASGRP4CSF1NCF2	205	12.016	9.63E-13	5.67E-10	0.92464169	12.01637	12.01637	1	2
		GO:0051246	Regulation of protein metabolic process	RALBP1CSF1IGFBP5DIPK2AGLG1RAC	218	10.498	3.18E-11	1.03E-08	0.79871628	10.49757	10.49757	1	2
**Hydrolase activity catalytic**	6	GO:0044093	Positive regulation of molecular function	TBC1D2BRALBP1RASGRP4CSF1DIPK2	129	7.346	4.51E-08	5.39E-06	0.52684112	7.345823	7.345823	1	2
		GO:0001932	Regulation of protein phosphorylation	BAG4GPNMBCCNHCHI3L1RALBP1CS	89	4.701	1.99E-05	0.0011	0.29586073	4.701147	4.701147	0.99998	2
		GO:0043087	Regulation of GTPase activity	PLXNC1TBC1D2BTRAPPC6BRALBP1R	43	5.170	6.76E-06	4.60E-04	0.33372422	5.170053	5.170053	0.99999	2
		GO:0051345	Positive regulation of hydrolase activity	TBC1D2BRALBP1RASGRP4RNASE4NI	59	6.265	5.43E-07	5.09E-05	0.42932822	6.2652	6.2652	1	2
		GO:0072583	Clathrin-dependent endocytosis	FCHO2AP2S1HIP1CLTCARRB1AP2A2	12	5.611	2.45E-06	1.90E-04	0.37212464	5.610834	5.610834	1	4
		GO:0016192	Vesicle-mediated transport	HSPA8TBC1D2BRALBP1BET1ARL3NC	164	19.873	1.34E-20	1.52E-16	1.58181564	19.8729	19.8729	1	4
**Dependent endocytosis receptor**	5	GO:0006898	Receptor-mediated endocytosis	SCARB2CTSLW5Q6N3_SHEEPDNM2I	30	7.975	1.06E-08	1.52E-06	0.58181564	7.974694	7.974694	1	4
		GO:0031623	Receptor internalization	CD81ITGB2NEDD4GRB2DNM2CLTCA	12	3.387	4.10E-04	0.0124	0.19065783	3.387216	3.387216	0.99959	4
		GO:0006897	Endocytosis	SCARB2HSPA8TBC1D2BRALBP1STBD	71	12.023	9.49E-13	5.67E-10	0.92464169	12.02273	12.02273	1	4
		GO:0042102	Positive regulation of T cell proliferation	TYK2ANXA1PYCARDDHPSPPP3CAIL1	15	3.699	2.00E-04	7.10E-03	0.21487417	3.69897	3.69897	0.9998	3
		GO:0002682	Regulation of immune system process	GPNMBST3GAL4CSF1W5P6T0_SHEE	124	7.609	2.46E-08	3.25E-06	0.54881166	7.609065	7.609065	1	3
**Regulation leukocyte proliferation**	5	GO:0070663	Regulation of leukocyte proliferation	GPNMBTYK2CSF1PYCARDAHRCD74I	32	5.336	4.61E-06	3.40E-04	0.34685211	5.336299	5.336299	1	3
		GO:0050865	Regulation of cell activation	GPNMBTYK2W5P6T0_SHEEPAHRW5	61	5.291	5.12E-06	3.70E-04	0.34317983	5.29073	5.29073	0.99999	3
		GO:0030155	Regulation of cell adhesion	BAG4PLXNC1GPNMBST3GAL4TYK2C	77	7.613	2.44E-08	3.25E-06	0.54881166	7.61261	7.61261	1	3
		GO:0070201	Regulation of establishment of protein localization	BAG4PARK7IL1BTSG101RNASE4SEPT	39	2.959	1.10E-03	2.68E-02	0.15718652	2.958607	2.958607	0.9989	6
**Establishment protein localization**	4	GO:0032879	Regulation of localization	FURINPARK7RNASE4CA2CTTNBP2NI	169	9.623	2.38E-10	5.29E-08	0.72765443	9.623423	9.623423	1	6
		GO:0060341	Regulation of cellular localization	BAG4PARK7IWS1RNASE4RAB9ASTX6	82	5.936	1.16E-06	1.00E-04	0.4	5.935542	5.935542	1	6
		GO:0051050	Positive regulation of transport	PARK7RNASE4CA2ATP1B1RAB9ACD8	72	5.400	3.98E-06	3.00E-04	0.35228787	5.400117	5.400117	1	6
		GO:0010628	Positive regulation of gene expression	HSPA8CHI3L1FURINTYK2CSF1WBP2I	94	5.325	4.73E-06	3.40E-04	0.34685211	5.325139	5.325139	1	5
**Multicellular organismal gene**	4	GO:0051239	Regulation of multicellular organismal process	TGGPNMBFURINCSF1ST3GAL4PARK7	192	6.943	1.14E-07	1.25E-05	0.490309	6.943095	6.943095	1	5
		GO:0001817	Regulation of cytokine production	GPNMBCHI3L1FURINTYK2PARK7NLF	69	6.349	4.48E-07	4.34E-05	0.43625103	6.348722	6.348722	1	5
		GO:0051240	Positive regulation of multicellular organismal proces	PLXNC1CHI3L1FURINST3GAL4TYK2C	95	2.959	1.10E-03	0.0271	0.15670307	2.958607	2.958607	0.9989	5
		GO:0090066	Regulation of anatomical structure size	BAG4DNM2EVLADD3TWF1SPTBN1C	60	8.883	1.31E-09	2.18E-07	0.66615435	8.882729	8.882729	1	7
**Protein complex assembly**	4	GO:0043254	Regulation of protein-containing complex assembly	BAG4EVLADD3PARK7TWF1PIH1D1P	60	10.485	3.27E-11	1.03E-08	0.79871628	10.48545	10.48545	1	7
		GO:0031334	Positive regulation of protein-containing complex ass	BAG4ALOX15VCPW5PVN6_SHEEPHS	24	3.959	1.10E-04	0.0042	0.23767507	3.958607	3.958607	0.99989	7
		GO:0032956	Regulation of actin cytoskeleton organization	BAG4EVLADD3RAC1TWF1ACTG1IQG	59	12.007	9.84E-13	5.67E-10	0.92464169	12.007	12.007	1	7
		GO:0002250	Adaptive immune response	FGRC7CTSLTYK2CTSCUNC13DCD74V	33	4.561	2.75E-05	0.0014	0.2853872	4.560667	4.560667	0.99997	13
**Adaptive immune response**	3	GO:0001906	Cell killing	W5NPK5_SHEEPCTSCUNC13DCTSHC	14	3.167	6.80E-04	1.89E-02	0.17235382	3.167491	3.167491	0.99932	13
		GO:0002449	Lymphocyte mediated immunity	W5NPK5_SHEEPC7CTSCTCIRG1UNC	19	3.310	4.90E-04	0.0142	0.18477117	3.309804	3.309804	0.99951	13
		GO:0006520	Cellular amino acid metabolic process	MPSTBHMTGCLCKYNUOATPYCR2GC	31	3.319	4.80E-04	0.014	0.1853872	3.318759	3.318759	0.99952	9
**Amino glutamine family**	3	GO:0009064	Glutamine family amino acid metabolic process	GCLCTMED8GLUD1OATPYCR2PFASA	15	4.340	4.57E-05	2.20E-03	0.26575773	4.340084	4.340084	0.99995	9
		GO:0009084	Glutamine family amino acid biosynthetic process	GLULASS1TMED8OATPYCR2ALDH18A	7	3.824	1.50E-04	0.0056	0.2251812	3.823909	3.823909	0.99985	9
		GO:1901698	Response to nitrogen compound	TYK2KYNUAHRIGFBP5COL1A2CA2LP	71	3.959	1.10E-04	4.30E-03	0.23665315	3.958607	3.958607	0.99989	8
**Endogenous compound nitrogen**	3	GO:1901700	Response to oxygen-containing compound	KYNUIGFBP5PARK7CA2LPLPRDX2RR	99	5.955	1.11E-06	9.81E-05	0.4008331	5.954677	5.954677	1	8
		GO:0009719	Response to endogenous stimulus	BAG4COL2A1TYK2WBP2AHRIGFBP5	85	3.638	2.30E-04	0.0078	0.21079054	3.638272	3.638272	0.99977	8
		GO:0071545	Inositol phosphate catabolic process	BPNT1OCRLINPP5KSYNJ1NUDT3IMP	6	2.824	1.50E-03	3.41E-02	0.14672456	2.823909	2.823909	0.9985	14
**Inositol phosphate hydroxy**	3	GO:0019751	Polyol metabolic process	GALK1GBAGKOCRLSYNJ1SHPKPCBD	15	3.018	9.60E-04	0.0243	0.16143937	3.017729	3.017729	0.99904	14
		GO:1901616	Organic hydroxy compound catabolic process	W5PY08_SHEEPGKOCRLSYNJ1NUDT	14	4.000	1.00E-04	0.004	0.239794	4	4	0.9999	14
		GO:0009057	Macromolecule catabolic process	HSPA8NHLRC3PSMB3STBD1DNASE2	84	3.796	1.60E-04	0.0057	0.22441251	3.79588	3.79588	0.99984	11
**Macromolecule catabolic proteolysis**	3	GO:0006508	Proteolysis	NHLRC3FURINPSMB3PARK7GLG1CU	109	3.328	4.70E-04	1.38E-02	0.18601209	3.327902	3.327902	0.99953	11
		GO:0009056	Catabolic process	HSPA8PIP4K2AVPS16TRAPPC8LRP1N	209	19.533	2.93E-20	1.66E-16	1.57798919	19.53313	19.53313	1	11
		GO:0009141	Nucleoside triphosphate metabolic process	GALK1ALDOAW5PK04_SHEEPGPIW5	28	5.678	2.10E-06	1.70E-04	0.37695511	5.677781	5.677781	1	15
**Nucleoside triphosphate metabolic**	3	GO:1901135	Carbohydrate derivative metabolic process	GALK1COL2A1ST3GAL4W5PK04_SHI	102	9.007	9.85E-10	1.67E-07	0.67772835	9.006564	9.006564	1	15
		GO:0055086	Nucleobase-containing small molecule metabolic pro	GALK1KYNUW5PK04_SHEEPPARK7G	65	8.489	3.24E-09	5.10E-07	0.62924298	8.489455	8.489455	1	15
		GO:0019725	Cellular homeostasis	CA2ATP1B1PRDX2PRDX1RAB11BCDI	53	3.824	1.50E-04	0.0055	0.22596373	3.823909	3.823909	0.99985	12
**Regulation biological quality**	3	GO:0042592	Homeostatic process	COL2A1CSF1PARK7DNASE2CA2LPLP	107	4.599	2.52E-05	0.0013	0.28860566	4.598599	4.598599	0.99997	12

Supplementary Table 13: Enrichment map analysis of differentially abundant proteins (DAPs) in GMØ compared to M0 (monocytes at 3 hours), presenting significantly enriched Gene Ontology biological process (GO-BP) terms and canonical pathways. Functional enrichment analysis was performed using the StringApp (STRING database) within Cytoscape (version 3.10.3)[1], focusing on Gene Ontology (GO) Biological Process (BP) terms and canonical pathways from the KEGG and Reactome databases[2–6]. Enriched GO terms were clustered as functionally coherent groups using the EnrichmentMap plugin[7]. Statistical significance was determined based on a false discovery rate (FDR) threshold of < 0.05.

		GO:0065008	Regulation of biological quality	HSPA8TGCOL2A1CSF1STXBP2TWF1P	265	8.315	4.84E-09	7.22E-07	0.61414628	8.315155	8.315155	1	12
Targeting vacuole lysosome	3	GO:0006623	Protein targeting to vacuole	SCARB2HSPA8ZFYVE16NEDD4VPS37	10	3.432	3.70E-04	0.0112	0.1950782	3.431798	3.431798	0.99963	10
		GO:0007034	Vacuolar transport	SCARB2HSPA8PSAPHSPA1A-2VPS41	25	5.148	7.12E-06	4.80E-04	0.33187588	5.14752	5.14752	0.99999	10
		GO:0061462	Protein localization to lysosome	SCARB2CD81GLMPHSPA8ZFYVE16N	12	4.660	2.19E-05	0.0012	0.29208188	4.659556	4.659556	0.99998	10
Amyloid beta clearance	2	GO:0045056	Transcytosis	LRP1VPS35CLTCRAB5ALRPAP1USO1	7	3.959	1.10E-04	0.0043	0.23665315	3.958607	3.958607	0.99989	28
		GO:0097242	Amyloid-beta clearance	W5NPK5_SHEEPW5NRI1_SHEEPC5A	10	4.893	1.28E-05	7.80E-04	0.31079054	4.89279	4.89279	0.99999	28
Anatomical structure morphogenesis	2	GO:0022603	Regulation of anatomical structure morphogenesis	PLXNC1PARVBCHI3L1RALBP1CSF1ST	88	8.660	2.19E-09	3.60E-07	0.64436975	8.659556	8.659556	1	29
		GO:0022604	Regulation of cell morphogenesis	PLXNC1FGRPARVBSTRIP1ALDOAPAL	39	6.706	1.97E-07	2.03E-05	0.4692504	6.705534	6.705534	1	29
Anatomical structure morphogenesis	2	GO:0000902	Cell morphogenesis	PLXNC1PARVBDNM2RAC1PTPN11IQ	58	3.051	8.90E-04	2.29E-02	0.16401645	3.05061	3.05061	0.99911	32
		GO:0009653	Anatomical structure morphogenesis	PARVBFURINCOL2A1CSF1GLG1RAC1	141	2.721	1.90E-03	0.0418	0.13788237	2.721246	2.721246	0.9981	32
Biological process involved	2	GO:0045087	Innate immune response	C7CSF1KYNUATP1B1W5PTZ8_SHEEF	53	3.119	7.60E-04	0.0204	0.16903698	3.119186	3.119186	0.99924	17
		GO:0044419	Biological process involved in interspecies interaction	HSPA8CSF1KYNUW5P6T0_SHEEPRN	118	6.434	3.68E-07	3.66E-05	0.44365189	6.434152	6.434152	1	17
Blood coagulation response	2	GO:0007596	Blood coagulation	F13A1FERMT3ANXA5TFPI2TREML1F	15	2.886	1.30E-03	0.0307	0.15128616	2.886057	2.886057	0.9987	26
		GO:0009611	Response to wounding	ALOX15F13A1EPPK1SARM1TFPI2HM	34	4.245	5.69E-05	2.60E-03	0.25850267	4.244888	4.244888	0.99994	26
Endomembrane system organization	2	GO:0010256	Endomembrane system organization	SUN2BET1WASHC4BIN2STX5CORO1	54	5.243	5.71E-06	4.00E-04	0.339794	5.243364	5.243364	0.99999	20
		GO:0061024	Membrane organization	SEC24DSUN2BET1PIP4K2ABIN2STX5	68	4.447	3.57E-05	1.80E-03	0.27447275	4.447332	4.447332	0.99996	20
Endosomal transport cytosolic	2	GO:0016482	Cytosolic transport	FLNASNX8PIP4K2AVPS26ACLTCSTX5	21	4.268	5.40E-05	2.50E-03	0.260206	4.267606	4.267606	0.99995	31
		GO:0016197	Endosomal transport	WASHC4STX5CORO1CSNX5RAB9AHI	28	3.921	1.20E-04	0.0047	0.23279021	3.920819	3.920819	0.99988	31
Glucose transmembrane transport	2	GO:0010829	Negative regulation of glucose transmembrane transp	PEA15INPP5KPRKCBFABP5ENPP1AP	6	3.187	6.50E-04	1.82E-02	0.17399286	3.187087	3.187087	0.99935	22
		GO:0034763	Negative regulation of transmembrane transport	NEDD4PEA15FKBP1ACLIC2MMP9CT	14	3.167	6.80E-04	0.0189	0.17235382	3.167491	3.167491	0.99932	22
Glycosyl process glycoside	2	GO:0016139	Glycoside catabolic process	GLAFUCA1W5PKU3_SHEEPNAGAGU	6	4.169	0.0000677	0.003	0.25228787	4.169411	4.169411	0.99993	23
		GO:1901657	Glycosyl compound metabolic process	FUCA1ADA2APRTHPRT1W5PKU3_SH	14	3.523	0.0003	0.0095	0.20222764	3.522879	3.522879	0.9997	23
Lipid storage maintenance	2	GO:0051235	Maintenance of location	GBAFLNAW5Q6N3_SHEEPPARK7GSI	21	3.638	0.00023	0.0078	0.21079054	3.638272	3.638272	0.99977	19
		GO:0019915	Lipid storage	GM2AGBASTAT5AW5Q6N3_SHEEPST	9	3.004	0.00099	0.0247	0.1607303	3.004365	3.004365	0.99901	19
Modified acid glutathione	2	GO:0006575	Cellular modified amino acid metabolic process	TGBHMTGCLCPARK7VNN2SHMT1CL	28	5.726	0.00000188	0.00016	0.379588	5.725842	5.725842	1	16
		GO:0006749	Glutathione metabolic process	GCLCPARK7CLIC2LOC101116273GSR	13	3.796	0.00016	0.0059	0.2229148	3.79588	3.79588	0.99984	16
Monosaccharide metabolic carbohydrate	2	GO:0005996	Monosaccharide metabolic process	GALK1ALDOARPIAGPIPGK1PKMTPI1	27	5.517	0.00000304	0.00023	0.36382722	5.517126	5.517126	1	30
		GO:0005975	Carbohydrate metabolic process	GALK1CHI3L1ST3GAL4STBD1LANCL1	65	9.955	1.11E-10	2.81E-08	0.75512937	9.954677	9.954677	1	30
Peptidase activity cysteine	2	GO:0052547	Regulation of peptidase activity	FURINPARK7TFPI2A2MNLRP2RARRE	56	6.921	0.00000012	0.0000129	0.48894103	6.920819	6.920819	1	27
		GO:2000116	Regulation of cysteine-type endopeptidase activity	FABP1PYCARDPARK7CAAP1NLRP2AT	26	4.034	0.0000924	0.0037	0.24317983	4.034328	4.034328	0.99991	27
Phosphorus metabolic process	2	GO:0006793	Phosphorus metabolic process	GALK1ACP2KYNUMAPK14W5PK04_	176	9.799	1.59E-10	3.76E-08	0.74248122	9.798603	9.798603	1	21
		GO:0016310	Phosphorylation	GALK1PLXNC1CCNHTP53RKTYK2MA	87	4.145	0.0000716	0.0031	0.25086383	4.145087	4.145087	0.99993	21
Post translational folding	2	GO:0061077	Chaperone-mediated protein folding	ST13HSPA8CCT2HSPA1A-2CD74FKBI	23	8.524	2.99E-09	4.78E-07	0.63205721	8.524329	8.524329	1	18
		GO:0051084	De novo post-translational protein folding	ST13HSPA8SDF2L1HSPH1CCT2HSPA	11	4.033	0.0000927	0.0037	0.24317983	4.03292	4.03292	0.99991	18
Reactive oxidative stress	2	GO:0034614	Cellular response to reactive oxygen species	FABP1W5PVN6_SHEEPANXA1MMP2	14	3.119	0.00076	0.0204	0.16903698	3.119186	3.119186	0.99924	24
		GO:0006979	Response to oxidative stress	FABP1W5PN31_SHEEPGCLCMMP2P	31	3.569	0.00027	0.009	0.20457575	3.568636	3.568636	0.99973	24
Vesicle transport endocytosis	2	GO:0030100	Regulation of endocytosis	ALOX15STAP1RUFY1DAB2TSG101BIN	31	7.175	6.69E-08	0.00000783	0.51062382	7.174574	7.174574	1	25
		GO:0060627	Regulation of vesicle-mediated transport	RAB9APPP3R1PACSIN2CORO1ARAB	66	9.664	2.17E-10	4.98E-08	0.73027707	9.66354	9.66354	1	25

EnrichmentMap analysis of GMØ_M0_ Pathway

Annatotation	Nodes	EnrichmentMap name	EnrichmentMap GS_DESCR	EnrichmentMap Genes	EnrichmentM	EnrichmentM	EnrichmentM	EnrichmentM	EnrichmentM	Enrichmen	Enrichmen	Enrichme	_mdcluster
Cellular responses stress	15	MAP-68882	Mitotic Anaphase	PSMD5PSMB4PSMB3MAD2L1TUBB1	45	6.38299966	4.14E-07	0.0000076	0.51191864	6.383	6.383	1	1
		oas05014	Amyotrophic lateral sclerosis	PSMB4PSMB3ACTR1APSMA3DCTN5	40	5.10568394	0.00000784	0.00011	0.39586073	5.105684	5.105684	0.99999	1
		MAP-4086400	PCP/CE pathway	PSMD5PSMB4PSMB3PSMA3PSMA4	31	11.1972263	6.35E-12	8.34E-10	0.90788339	11.19723	11.19723	1	1
		MAP-8878171	Transcriptional regulation by RUNX1	PSMD5PSMB4PSMB3PSMA3PSMA4	34	6.02410886	9.46E-07	0.0000161	0.47931741	6.024109	6.024109	1	1
		oas05017	Spinocerebellar ataxia	PSMB4PSMB3ATP2A3SPTBN2PSMA3	18	3.26760624	0.00054	0.0031	0.25086383	3.267606	3.267606	0.99946	1
		MAP-9006925	Intracellular signaling by second messengers	PSMD5PSMB4PSMB3RPS6KB2MAPK	41	4.86646109	0.0000136	0.00017	0.37695511	4.866461	4.866461	0.99999	1
		MAP-5688426	Deubiquitination	PSMD5PSMB4PSMB3PSMA3MAP3K	41	3.15490196	0.0007	0.0061	0.22146702	3.154902	3.154902	0.9993	1
		oas05020	Prion disease	PSMB4PSMB3MAPK1HSPA8PSMA3C	35	6.06098022	8.69E-07	0.000016	0.479588	6.06098	6.06098	1	1
		oas05016	Huntington disease	PSMB4PSMB3ACTR1APSMA3DCTN5	31	3.61978876	0.00024	0.0016	0.279588	3.619789	3.619789	0.99976	1
		MAP-5621481	C-type lectin receptors (CLRs)	PSMD5PSMB4PSMB3PSMA3MAP3K	34	10.3242217	4.74E-11	4.5E-09	0.83467875	10.32422	10.32422	1	1
		MAP-8951664	Neddylation	PSMD5PSMB4PSMB3PSMA3GPS1PS	35	2.20760831	0.0062	0.0401	0.13968556	2.207608	2.207608	0.9938	1
		MAP-5683057	MAPK family signaling cascades	SPTAN1PSMB4PSMB3PSMA3PSMA4	56	7.24108811	5.74E-08	0.00000132	0.58794261	7.241088	7.241088	1	1
		MAP-69275	G2/M Transition	PPP1R12APSMD5PSMB4PSMB3TUB	41	7.36754271	4.29E-08	0.00000109	0.59625735	7.367543	7.367543	1	1
		MAP-2262752	Cellular responses to stress	FKBP4PSMB4FABP1HSPA8CAPZA2A	101	9.36051351	4.36E-10	3.72E-08	0.74294571	9.360514	9.360514	1	1
		MAP-9711123	Cellular response to chemical stress	PSMD5PSMB4PSMB3FABP1PSMA3C	41	7.95078198	1.12E-08	3.72E-07	0.64294571	7.950782	7.950782	1	1
Long term potentiation	5	oas04370	VEGF signaling pathway	RAC1MAPK14MAPK1CDC42PXNPLC	12	4.1481304	0.0000711	0.00064	0.319382	4.14813	4.14813	0.99993	2
		oas04662	B cell receptor signaling pathway	RAC1MAPK1PRKCBPPP3R1IGHMCD	14	4.55129368	0.0000281	0.00031	0.35086383	4.551294	4.551294	0.99997	2
		oas04664	Fc epsilon RI signaling pathway	RAC1BTKMAPK14MAPK1PLCG2MAP	9	2.04575749	0.009	0.0301	0.15214335	2.045757	2.045757	0.991	2
		oas04720	Long-term potentiation	PPP1CAMAPK1MAP2K1PRKCBRAP1	9	2.33724217	0.0046	0.0174	0.17594508	2.337242	2.337242	0.9954	2

Supplementary Table 13: Enrichment map analysis of differentially abundant proteins (DAPs) in GMØ compared to M0 (monocytes at 3 hours), presenting significantly enriched Gene Ontology biological process (GO-BP) terms and canonical pathways. Functional enrichment analysis was performed using the StringApp (STRING database) within Cytoscape (version 3.10.3)[1], focusing on Gene Ontology (GO) Biological Process (BP) terms and canonical pathways from the KEGG and Reactome databases[2–6]. Enriched GO terms were clustered as functionally coherent groups using the EnrichmentMap plugin[7]. Statistical significance was determined based on a false discovery rate (FDR) threshold of < 0.05.

		oas04114	Oocyte meiosis	PPP1CAMAPK14YWHAQMAPK1MAI	14	2.45593196	0.0035	0.0144	0.18416375	2.455932	2.455932	0.9965	2
**Glycolysis gluconeogenesis glucose**	4	MAP-70326	Glucose metabolism	PGK2MDH2PKMTPI1W5PK04_SHEEI	16	4.42365865	0.0000377	0.00043	0.33665315	4.423659	4.423659	0.99996	3
		oas00010	Glycolysis / Gluconeogenesis	PGK2PGM1ALDH7A1PKMTPI1GALM	17	6.37263414	4.24E-07	0.00000918	0.50371573	6.372634	6.372634	1	3
		oas01200	Carbon metabolism	PGK2FHSHMT1PGK1GLUD1ENO1ALI	26	7.84466396	1.43E-08	5.95E-07	0.6225483	7.844664	7.844664	1	3
		oas01230	Biosynthesis of amino acids	PGK2SHMT1PKMTPI1IDH1GLULACO	23	9.32882716	4.69E-10	2.6E-08	0.75850267	9.328827	9.328827	1	3
**Cell complex components**	3	MAP-446353	Cell-extracellular matrix interactions	PXNFLNAILKACTN1LIMS1ACTG1RSU	9	3.65757732	0.00022	0.0022	0.26575773	3.657577	3.657577	0.99978	5
		MAP-446388	Regulation of cytoskeletal remodeling and cell spread	PXNACTN1RSU1PARVB	4	2.18045606	0.0066	0.0424	0.13726341	2.180456	2.180456	0.9934	5
		MAP-1500931	Cell-Cell communication	IQGAP1CD2APPXNFLNAILKCDH5GRI	20	2.60205999	0.0025	0.0189	0.17235382	2.60206	2.60206	0.9975	5
**Gtpases rhobtb3 rho**	3	MAP-195258	RHO GTPase Effectors	PPP1R12AYWHAQMAD2L1MYL9TAX	50	5.31336373	0.00000486	0.0000703	0.41530447	5.313364	5.313364	1	6
		MAP-9716542	Signaling by Rho GTPases, Miro GTPases and RHOBTB	DYNC1I2COPS4SPTBN1BTKPICALMN	125	13.7772835	1.67E-14	4.08E-12	1.13893398	13.77728	13.77728	1	6
		MAP-162582	Signal Transduction	W5NPK5_SHEEPMAP3K20W5NRI1_	288	11.3098039	4.9E-12	6.97E-10	0.91567672	11.3098	11.3098	1	6
**Rhog gtpase rhoa**	3	MAP-9013149	RAC1 GTPase cycle	YKT6CYFIP1ARHGAP9RRBP1ARHGAI	26	2.28399666	0.0052	0.0351	0.14546929	2.283997	2.283997	0.9948	4
		MAP-8980692	RHOA GTPase cycle	IQGAP1YKT6ABCD3STX5ERBINHMO	23	3.18708664	0.00065	0.0057	0.22441251	3.187087	3.187087	0.99935	4
		MAP-9013408	RHOG GTPase cycle	W5Q1T4_SHEEPYKT6CDC42CYFIP1S	18	4.43651891	0.0000366	0.00042	0.33767507	4.436519	4.436519	0.99996	4
**GM-CSF signaling**	2	MAP-512988	Interleukin-3, Interleukin-5 and GM-CSF signaling	CBLYWHAZSTAT5AGAB2GRB2VAV1P	10	2.55284197	0.0028	0.0208	0.16819367	2.552842	2.552842	0.9972	14
		MAP-1433557	Signaling by SCF-KIT	CBLMMP9STAT5AGAB2FESGRB2W5I	13	4.19928292	0.0000632	0.00069	0.31611509	4.199283	4.199283	0.99994	14
**Cs ds glycosaminoglycan**	2	oas00531	Glycosaminoglycan degradation	GLB1ARSBGNSHEXBIDSHEXALOC101	10	5.29584948	0.00000506	0.0000764	0.41169066	5.295849	5.295849	0.99999	9
		MAP-2024101	CS/DS degradation	IDUAARSBHEXBIDSHEXALOC101112	7	3.1079054	0.00078	0.0067	0.21739252	3.107905	3.107905	0.99922	9
**Er retrograde traffic**	2	MAP-6811442	Intra-Golgi and retrograde Golgi-to-ER traffic	YKT6TUBB1CAPZA2ACTR1AW5NSS8	37	5.537602	0.0000029	0.0000435	0.43615107	5.537602	5.537602	1	16
		MAP-6811434	COPI-dependent Golgi-to-ER retrograde traffic	KIF2ACOPB2STX18TUBB1W5NSS8_S	17	2.40893539	0.0039	0.0281	0.15512937	2.408935	2.408935	0.9961	16
**Extracellular matrix organization**	2	MAP-1474228	Degradation of the extracellular matrix	CD44MMP9MMP14COL1A1ADAM2	21	2.95860731	0.0011	0.0087	0.20604807	2.958607	2.958607	0.9989	15
		MAP-1474244	Extracellular matrix organization	OGFOD3MMP14PPIBADAM28MMP	43	4.2915791	0.0000511	0.00056	0.3251812	4.291579	4.291579	0.99995	15
**Glutathione metabolism conjugation**	2	oas00480	Glutathione metabolism	PGDGCLCGSRIDH1LOC101116273M	11	3.36653154	0.00043	0.0026	0.25850267	3.366532	3.366532	0.99957	17
		MAP-156590	Glutathione conjugation	AKR1A1CNDP2GCLCLOC101116273I	9	2.79588002	0.0016	0.0124	0.19065783	2.79588	2.79588	0.9984	17
**Inositol phosphate metabolism**	2	oas00562	Inositol phosphate metabolism	MTMR6SYNJ1TPI1MTMR3OCRLPLCI	15	4.75448733	0.0000176	0.00021	0.36777807	4.754487	4.754487	0.99998	13
		MAP-1660499	Synthesis of PIPs at the plasma membrane	MTMR6SYNJ1MTMR3PLEKHA2OCRL	16	4.18243463	0.0000657	0.00071	0.31487417	4.182435	4.182435	0.99993	13
**Iron uptake transport**	2	MAP-917937	Iron uptake and transport	HFEATP6V0D1ATP6V1G1ATP6V1DAC	19	3.55284197	0.00028	0.0027	0.25686362	3.552842	3.552842	0.99972	7
		MAP-1222556	ROS and RNS production in phagocytes	ATP6V0D1ATP6V1G1NCF2ATP6V1DA	12	4.75448733	0.0000176	0.00022	0.36575773	4.754487	4.754487	0.99998	7
**Kaposi sarcoma herpesvirus**	2	oas05170	Human immunodeficiency virus 1 infection	RPS6KB2MAPK1PXNPDIA3PRKCBBST	25	4.05601112	0.0000879	0.00069	0.31611509	4.056011	4.056011	0.99991	11
		oas05167	Kaposi sarcoma-associated herpesvirus infection	RAC1MAPK14MAPK1W5NRI1_SHEE	17	1.98296666	0.0104	0.0334	0.14762535	1.982967	1.982967	0.9896	11
**Metabolic pathways metabolism**	2	oas01100	Metabolic pathways	ATP6V1DCOX2PFASGLUD1W5NRS0_	185	25.5346171	2.92E-26	9.68E-24	2.30141246	25.53462	25.53462	1	8
		MAP-1430728	Metabolism	CHP1SLC2A1FABP1W5NRS0_SHEEPI	282	23.2175274	6.06E-24	2.59E-21	2.05867002	23.21753	23.21753	1	8
**Renal cell carcinoma**	2	oas05211	Renal cell carcinoma	RAC1SLC2A1FHMAPK1VHLCDC42M	10	2.60205999	0.0025	0.0108	0.19665762	2.60206	2.60206	0.9975	12
		oas04722	Neurotrophin signaling pathway	RAC1MAPK14MAPK1CDC42PLCG2M	12	1.91721463	0.0121	0.0369	0.14329736	1.917215	1.917215	0.9879	12
**Trafficking glur2 ampa**	2	MAP-437239	Recycling pathway of L1	SHTN1MAPK1MSNAP2A2-2AP2S1DI	14	7.09963287	7.95E-08	0.00000179	0.5747147	7.099633	7.099633	1	10
		MAP-416993	Trafficking of GluR2-containing AMPA receptors	AP2S1AP2M1AP2A2PRKCBAP2A1AP	7	2.92081875	0.0012	0.0098	0.20087739	2.920819	2.920819	0.9988	10

GMØ, monocyte-derived macrophages differentiated with GM-CSF (Granulocyte-Macrophage Colony-Stimulating Factor) and M0, Monocytes at 3 hours.

Supplementary Table 14: Enrichment map analysis of differentially abundant proteins (DAPs) in MMØ compared to M0 (monocytes at 3 hours), presenting significantly enriched Gene Ontology biological process (GO-BP) terms and canonical pathways. Functional enrichment analysis was performed using the StringApp (STRING database) within Cytoscape (version 3.10.3)[1], focusing on Gene Ontology (GO) Biological Process (BP) terms and canonical pathways from the KEGG and Reactome databases[2–6]. Enriched GO terms were clustered as functionally coherent groups using the EnrichmentMap plugin[7]. Statistical significance was determined based on a false discovery rate (FDR) threshold of < 0.05.

EnrichmentMap analysis of MMØ_M0_ Biological Process

Annotation	Nodes	EnrichmentMap name	EnrichmentMap (	EnrichmentMap Genes	nrichmentMap gs_siz	EnrichmentMap	EnrichmentMap: pv	EnrichmentMap::fd	EnrichmentMap::NE	EnrichmentMap::-log10 (p	EnrichmentMa	EnrichmentMa	_mdcluster
stress communication stimulus	10	GO:0048519	Negative regulati	CHMP6W5NSK4_SHEEPTREM2PIP4	291	5.64975	2.24E-06	2.00E-04	0.369897	5.649751982	5.649751982	0.99999776	1
		GO:0048585	Negative regulati	CHMP6NFKB1INPP5KTREM2PIP4K2	106	5.46344	3.44E-06	2.70E-04	0.356863624	5.463441557	5.463441557	0.99999656	1
		GO:0009987	Cellular process	CHMP6S100A12RALBP1W5NSK4_S	897	13.04721	8.97E-14	1.02E-10	0.999139983	13.04720756	13.04720756	1	1
		GO:0048518	Positive regulatio	S100A12RALBP1TREM2RRM2PIP4K	358	8.29243	5.10E-09	1.48E-06	0.582973828	8.292429824	8.292429824	0.999999995	1
		GO:0006950	Response to stres	CHMP6S100A12W5NSK4_SHEEPTR	197	7.69250	2.03E-08	4.42E-06	0.535457773	7.692503962	7.692503962	0.99999998	1
		GO:0008152	Metabolic proces	CHMP6S100A12NCF2TREM2PIP4K2	499	7.39041	4.07E-08	7.35E-06	0.513371266	7.390405591	7.390405591	0.999999959	1
		GO:1902531	Regulation of intr	S100A12INPP5KRGNTREM2HSPB1K	109	5.49080	3.23E-06	2.60E-04	0.358502665	5.490797478	5.490797478	0.99999677	1
		GO:0048583	Regulation of resp	CHMP6S100A12RGNTREM2PIP4K2/	244	9.69250	2.03E-10	8.52E-08	0.706956041	9.692503962	9.692503962	1	1
		GO:0050896	Response to stim	CHMP6S100A12RALBP1W5NSK4_S	425	4.24795	5.65E-05	2.70E-03	0.256863624	4.247951552	4.247951552	0.9999435	1
		GO:0010647	Positive regulatio	NFKB1S100A12TREM2KANK1UBE2I	112	6.36957	4.27E-07	5.04E-05	0.429756946	6.369572125	6.369572125	0.999999573	1
cellular component biogenesis	9	GO:0030834	Regulation of acti	DSTNPLEKAVILGSNTWF1ADD3ADD	15	6.16368	6.86E-07	7.35E-05	0.413371266	6.163675884	6.163675884	0.999999314	2
		GO:0051130	Positive regulatio	RALBP1PLEKAVILMMP9TREM2PPT1	78	5.59007	2.57E-06	2.20E-04	0.365757732	5.590066877	5.590066877	0.99999743	2
		GO:1902904	Negative regulati	INPP5KAVILSSH1ARHGAP6GSNLOC	22	4.48945	3.24E-05	1.80E-03	0.274472749	4.48945499	4.48945499	0.9999676	2
		GO:0032970	Regulation of acti	INPP5KPLEKAVILRAC1CAPGKANK1S	51	9.98297	1.04E-10	5.39E-08	0.726841123	9.982966661	9.982966661	1	2
		GO:0032271	Regulation of pro	CYFIP1AVILGRB2TBCDTWF1ADD3C/	32	8.02045	9.54E-09	2.46E-06	0.560906489	8.020451625	8.020451625	0.99999999	2
		GO:0090066	Regulation of ana	VAMP2PLEKAVILCAPGKANK1SPTBN	49	7.28819	5.15E-08	8.73E-06	0.505898576	7.288192771	7.288192771	0.999999949	2
		GO:0051129	Negative regulati	INPP5KAVILTREM2TFRCPPP3CACAI	50	3.88606	1.30E-04	5.50E-03	0.225963731	3.886056648	3.886056648	0.99987	2
		GO:0044087	Regulation of cell	CHMP6INPP5KKIF15PLEKMACF1AV	74	5.22841	5.91E-06	4.20E-04	0.337675071	5.228412519	5.228412519	0.99999409	2
		GO:0051128	Regulation of cell	CHMP6INPP5KRALBP1CLASP1TREM	163	9.41229	3.87E-10	1.57E-07	0.680410035	9.412289035	9.412289035	1	2
endocytosis localization establishment	7	GO:0006898	Receptor-mediate	DNAJC13SNX17MSR1AP2A2-2PPT1	26	7.44009	3.63E-08	6.86E-06	0.516367588	7.440093375	7.440093375	0.999999964	3
		GO:0008104	Protein localizatio	JARID2INPP5KATG7DENND10LAMP	158	12.44370	3.60E-13	3.24E-10	0.948945499	12.4436975	12.4436975	1	3
		GO:0006897	Endocytosis	VAMP2SNX17RALBP1EHD3W5NSK4	65	12.95078	1.12E-13	1.15E-10	0.993930216	12.95078198	12.95078198	1	3
		GO:0016192	Vesicle-mediated	CHMP6S100A12RALBP1W5NSK4_S	146	20.63264	2.33E-21	2.65E-17	1.657675413	20.63264408	20.63264408	1	3
		GO:0051179	Localization	CHMP6S100A12RALBP1W5NSK4_S	346	19.67572	2.11E-20	1.20E-16	1.592081875	19.67571754	19.67571754	1	3
		GO:0071702	Organic substanc	TCN1RALBP1ATG7DENND10LAMP2	143	7.65170	2.23E-08	4.45E-06	0.535163999	7.651695137	7.651695137	0.999999978	3
		GO:0051649	Establishment of	CHMP6S100A12DENND10LAMP2AI	137	8.90658	1.24E-09	4.42E-07	0.635457773	8.906578315	8.906578315	0.999999999	3
positive regulation activity	6	GO:0052547	Regulation of pep	BIN1-2CD44THBS1SERPINB8MMP9	43	4.77728	1.67E-05	1.00E-03	0.3	4.777283529	4.777283529	0.9999833	4
		GO:0051336	Regulation of hyd	LOC100101238W5P3V8_SHEEPRAL	91	9.34390	4.53E-10	1.77E-07	0.675202673	9.343901798	9.343901798	1	4
		GO:2000116	Regulation of cyst	PDCD5LGMNCD44THBS1GSNMMP	20	2.92082	1.20E-03	3.22E-02	0.149214413	2.920818754	2.920818754	0.9988	4
		GO:0043085	Positive regulatio	VAC14LOC100101238W5P3V8_SHE	82	5.45346	3.52E-06	2.80E-04	0.355284197	5.453457337	5.453457337	0.99999648	4
		GO:0065009	Regulation of mo	CHMP6RALBP1DENND10NCF2RGN	218	12.46471	3.43E-13	3.24E-10	0.948945499	12.46470588	12.46470588	1	4
		GO:0043087	Regulation of GTI	LOC100101238W5P3V8_SHEEPRAL	41	6.39254	4.05E-07	4.84E-05	0.431515464	6.392544977	6.392544977	0.999999595	4
regulation transport localization	5	GO:0032879	Regulation of loca	INPP5KMS4A1DENND10TREM2PPP	159	13.24489	5.69E-14	7.18E-11	1.014387556	13.24488773	13.24488773	1	5
		GO:0030100	Regulation of end	W5QFU4_SHEEPW5P7F8_SHEEPDA	28	7.31785	4.81E-08	8.27E-06	0.508249449	7.317854924	7.317854924	0.999999952	5
		GO:0051050	Positive regulatio	INPP5KMS4A1TREM2PPT1PPP3CA\	69	7.35458	4.42E-08	7.71E-06	0.511294562	7.354577731	7.354577731	0.999999956	5
		GO:0060341	Regulation of cell	INPP5KDENND10TREM2TFRCVAMP	74	6.74958	1.78E-07	2.52E-05	0.459859946	6.749579998	6.749579998	0.999999822	5
		GO:0060627	Regulation of ves	DENND10TREM2PPT1PPP3CAVAMI	64	12.13194	7.38E-13	5.24E-10	0.928066871	12.13194364	12.13194364	1	5
monovalent cation homeostasis	4	GO:0042592	Homeostatic proc	INPP5KLAMP2LOC101122662RGNT	102	6.95078	1.12E-07	1.72E-05	0.476447155	6.950781977	6.950781977	0.999999888	6
		GO:0050801	Ion homeostasis	CHERPATP6V1G1RGNATP6V1ACCD	44	4.42136	3.79E-05	0.002	0.269897	4.42136079	4.42136079	0.9999621	6
		GO:0065008	Regulation of biol	LOC101110434RGNTREM2STX11ST	245	12.32975	4.68E-13	3.54E-10	0.945099674	12.32975415	12.32975415	1	6
		GO:0055067	Monovalent inorg	ATP6V0D1ATP6V1AATP1B1ATP6V1I	18	3.79588	1.60E-04	0.0064	0.219382003	3.795880017	3.795880017	0.99984	6
phosphate metabolic molecular	4	GO:0019220	Regulation of pho	VAC14CHMP6GPNMBINPP5KRALBI	86	3.38722	4.10E-04	0.0139	0.18569852	3.387216143	3.387216143	0.99959	7
		GO:0044092	Negative regulati	ANXA4CHMP6NFKB1INPP5KTHBS1	81	5.20971	6.17E-06	4.40E-04	0.335654732	5.209714836	5.209714836	0.99999383	7
		GO:0051248	Negative regulati	CHMP6JARID2INPP5KTHBS1SERPIN	71	3.72125	1.90E-04	0.0074	0.213076828	3.721246399	3.721246399	0.99981	7
		GO:0051246	Regulation of pro	CHMP6JARID2GPNMBINPP5KRALBI	164	5.52871	2.96E-06	2.50E-04	0.360205999	5.528708289	5.528708289	0.99999704	7
cellular reactive species	3	GO:0072593	Reactive oxygen s	NQO1CCN2NCF2LOC101122662SH	13	3.29243	5.10E-04	1.65E-02	0.178251606	3.292429824	3.292429824	0.99949	8
		GO:0019430	Removal of super	NQO1MPOPRDX2PRDX1SOD2SOD1	6	3.88606	1.30E-04	0.0054	0.226760624	3.886056648	3.886056648	0.99987	8
		GO:0034614	Cellular response	RIPK1NQO1MMP9PARK7MAPK7SO	15	4.43297	3.69E-05	2.00E-03	0.269897	4.432973634	4.432973634	0.9999631	8
hormone secretion exocytosis	3	GO:0045921	Positive regulatio	UNC13DITGB2RAB3AFGGITGAMLA	11	2.76955	1.70E-03	0.0419	0.137778598	2.769551079	2.769551079	0.9983	12
		GO:0046887	Positive regulatio	PLA2G6SRIFGGSPP1PTPN11VAMP8	12	2.76955	1.70E-03	0.0411	0.138615818	2.769551079	2.769551079	0.9983	12
		GO:0051047	Positive regulatio	UNC13DTREM2PPP3CAVAMP8AAC	25	3.82391	1.50E-04	0.0059	0.222914799	3.823908741	3.823908741	0.99985	12
ph reduction proton	3	GO:0007035	Vacuolar acidifica	ATP6V0D1TCIRG1GRNPPT1ATP6V0/	6	3.02687	9.40E-04	2.63E-02	0.158004425	3.026872146	3.026872146	0.99906	10
		GO:1902600	Proton transmem	ATP6V0D1ATP6V1FATP6V1G1ATP6V	15	2.92082	1.20E-03	3.14E-02	0.150307035	2.920818754	2.920818754	0.9988	10
		GO:0045851	pH reduction	ATP6V0D1TCIRG1ATP6V1AATP6V1E	7	4.77728	1.67E-05	1.00E-03	0.3	4.777283529	4.777283529	0.9999833	10
regulation system activation	3	GO:0002682	Regulation of imr	GPNMBST3GAL4MS4A1TREM2PPP	109	8.07058	8.50E-09	2.30E-06	0.563827216	8.070581074	8.070581074	0.999999992	9
		GO:0030155	Regulation of cell	GPNMBST3GAL4FERMT3MACF1TH	75	10.33819	4.59E-11	2.63E-08	0.758004425	10.33818731	10.33818731	1	9
		GO:0050865	Regulation of cell	GPNMBPLEKTHBS1TREM2CD86TFR	61	7.89279	1.28E-08	3.06E-06	0.551427857	7.89279003	7.89279003	0.999999987	9

Supplementary Table 14: Enrichment map analysis of differentially abundant proteins (DAPs) in MMØ compared to M0 (monocytes at 3 hours), presenting significantly enriched Gene Ontology biological process (GO-BP) terms and canonical pathways. Functional enrichment analysis was performed using the StringApp (STRING database) within Cytoscape (version 3.10.3)[1], focusing on Gene Ontology (GO) Biological Process (BP) terms and canonical pathways from the KEGG and Reactome databases[2–6]. Enriched GO terms were clustered as functionally coherent groups using the EnrichmentMap plugin[7]. Statistical significance was determined based on a false discovery rate (FDR) threshold of < 0.05.

signaling pathway domain	3	GO:2001237	Negative regulati	RIPK1LGALS3BRCA1FGGPARK7ITGA	14	4.27572	5.30E-05	0.0026	0.258502665	4.27572413	4.27572413	0.999947	11
		GO:2001233	Regulation of apo	SLC35F6LGALS3CD44THBS1MMP9T	34	4.41794	3.82E-05	2.00E-03	0.269897	4.417936637	4.417936637	0.9999618	11
		GO:1902041	Regulation of extr	LGALS3BRCA1THBS1FGGFGAFGBPA	8	3.07058	8.50E-04	0.024	0.161978876	3.070581074	3.070581074	0.99915	11
amino acid process	2	GO:0006520	Cellular amino ac	SHMT1SDSAASSDARS2MTHFRMPST	31	4.69897	2.00E-05	1.20E-03	0.292081875	4.698970004	4.698970004	0.99998	14
		GO:0008652	Cellular amino ac	GOT1ALDH1A1SHMT1SDSAASSSEPI	14	4.45223	3.53E-05	0.0019	0.27212464	4.452225295	4.452225295	0.9999647	14
anatomical structure morphogenesis	2	GO:0022603	Regulation of ana	RALBP1W5NSK4_SHEEPTHBS1TFRC	66	5.34294	4.54E-06	3.50E-04	0.345593196	5.342944147	5.342944147	0.99999546	22
		GO:0022604	Regulation of cell	UNC13DCD44LIMS1LOC101105107	32	5.60555	2.48E-06	2.10E-04	0.367778071	5.605548319	5.605548319	0.99999752	22
antigen processing presentation	2	GO:0002478	Antigen processir	W5Q2C4_SHEEPW5Q2V1_SHEEPCL	13	7.09528	8.03E-08	1.28E-05	0.489279003	7.095284455	7.095284455	0.99999992	23
		GO:0002501	Peptide antigen a	W5NYC7_SHEEPW5Q2C4_SHEEPLO	7	3.18709	6.50E-04	2.00E-02	0.169897	3.187086643	3.187086643	0.99935	23
autophagosome organization macroautophagy	2	GO:0016236	Macroautophagy	CHMP6ATG7LAMP2ATG3RAB1APIP	26	6.01592	9.64E-07	9.52E-05	0.402136305	6.015922966	6.015922966	0.999999036	26
		GO:1905037	Autophagosome	GABARAPSTBD1WDR45BATG7TCIR	11	2.72125	1.90E-03	0.0451	0.134582346	2.721246399	2.721246399	0.9981	26
biological process involved	2	GO:0045087	Innate immune re	SARM1LOC100101238UNC13DS100	46	3.18709	6.50E-04	0.02	0.169897	3.187086643	3.187086643	0.99935	32
		GO:0044419	Biological process	CHMP6NFKB1S100A12INPP5KW5N	106	7.48812	3.25E-08	6.26E-06	0.520342567	7.488116639	7.488116639	0.999999968	32
calcium mediated signaling	2	GO:0050850	Positive regulatio	CHERPPLCG2TREM2P2RX4LOC1011	6	2.67778	2.10E-03	0.0489	0.131069114	2.677780705	2.677780705	0.9979	29
		GO:0050848	Regulation of calc	CHERPW5Q3L8_SHEEPPLCG2W5PI	14	4.76700	1.71E-05	1.00E-03	0.3	4.76700389	4.76700389	0.9999829	29
cellular component assembly	2	GO:0016043	Cellular compone	CHMP6ARL3TREM2STX11PIP4K2AR	356	11.64397	2.27E-12	1.51E-09	0.882102305	11.64397414	11.64397414	1	16
		GO:0022607	Cellular compone	CHMP6INPP5KATG7MS4A1ARL3CL	149	4.00087	9.98E-05	4.30E-03	0.236653154	4.000869459	4.000869459	0.9999002	16
chemical response oxygen	2	GO:0070887	Cellular response	JARID2NFKB1S100A12INPP5KTREM	163	8.19246	6.42E-09	1.82E-06	0.573992861	8.192464972	8.192464972	0.999999994	18
		GO:1901701	Cellular response	NFKB1INPP5KMMP9TREM2ESDCD8	71	6.24260	5.72E-07	6.50E-05	0.418708664	6.242603971	6.242603971	0.999999428	18
glycoside catabolic glucuronoside	2	GO:0019391	Glucuronoside ca	GUSBW5PKU3_SHEEPABHD10	3	2.72125	1.90E-03	4.54E-02	0.134294415	2.721246399	2.721246399	0.9981	15
		GO:0016139	Glycoside catabol	FUCA1GUSBFUCA2GLANAGAW5PK	7	5.59688	2.53E-06	2.20E-04	0.365757732	5.596879479	5.596879479	0.99999747	15
lipid storage maintenance	2	GO:0019915	Lipid storage	ENPP1HEXAW5Q6N3_SHEEPSTARD	8	2.92082	0.0012	0.0314	0.150307035	2.920818754	2.920818754	0.9988	13
		GO:0051235	Maintenance of lo	ENPP1HEXASUN1FLNATSPOSYNE1C	19	3.82391	1.50E-04	0.0061	0.221467016	3.823908741	3.823908741	0.99985	13
mononuclear leukocyte migration	2	GO:0050900	Leukocyte migrat	LOC100101238IL16W5P3V8_SHEEP	25	4.82391	1.50E-05	9.20E-04	0.303621217	4.823908741	4.823908741	0.999985	28
		GO:0071674	Mononuclear cell	LOC100101238MYO1GW5P3V8_SH	12	2.72125	0.0019	0.0443	0.135359627	2.721246399	2.721246399	0.9981	28
negative glucose transmembrane	2	GO:0034763	Negative regulati	ENPP1INPP5KFABP5THBS1MMP9N	13	3.43180	3.70E-04	0.0127	0.189619628	3.431798276	3.431798276	0.99963	30
		GO:0010829	Negative regulati	ENPP1APPL2INPP5KFABP5PRKCBPE	6	3.60206	2.50E-04	0.0093	0.203151705	3.602059991	3.602059991	0.99975	30
nuclear envelope organization	2	GO:0006998	Nuclear envelope	TOR1BCHMP6SUN1LMNALMNB2LM	9	2.85387	0.0014	0.0358	0.144611697	2.853871964	2.853871964	0.9986	31
		GO:0007097	Nuclear migratior	DYNC1H1SUN1LMNALMNB2LMNB1	7	3.36653	4.30E-04	0.0142	0.184771166	3.366531544	3.366531544	0.99957	31
nucleobase molecule derivative	2	GO:1901135	Carbohydrate der	FUCA1UCK2HPRT1DCKST3GAL4ALD	82	6.92445	1.19E-07	1.78E-05	0.474958	6.924453039	6.924453039	0.999999881	24
		GO:0055086	Nucleobase-conta	UCK2HPRT1DCKALDOBAK3RRM2SN	52	6.56864	2.70E-07	3.40E-05	0.446852108	6.568636236	6.568636236	0.99999973	24
positive engulfment neuroinflammatory	2	GO:0060099	Regulation of pha	APPL2PLCG2STAP1TREM2W5NRI1_	6	3.46852	3.40E-04	0.0118	0.192811799	3.468521083	3.468521083	0.99966	25
		GO:0150078	Positive regulatio	CTSCPLCG2STAP1TREM2	4	2.72125	0.0019	0.0446	0.135066514	2.721246399	2.721246399	0.9981	25
positive gene expression	2	GO:0010628	Positive regulatio	ACTC1RAB1ATHBS1TREM2TFRCPPF	74	3.72125	1.90E-04	0.0074	0.213076828	3.721246399	3.721246399	0.99981	17
		GO:0001817	Regulation of cyto	ANXA4NFKB1GPNMBRAB1ATHBS1	56	5.08197	8.28E-06	5.60E-04	0.325181197	5.081969663	5.081969663	0.99999172	17
regulation body fluid	2	GO:0050878	Regulation of boc	GP5PLAUINPP5KST3GAL4FERMT3P	34	6.53018	2.95E-07	3.65E-05	0.443770714	6.530177984	6.530177984	0.999999705	19
		GO:0042060	Wound healing	CHMP6GP5FERMT3PLEKMACF1CD4	32	6.99568	1.01E-07	1.60E-05	0.479588002	6.995678626	6.995678626	0.999999899	19
substrate dependent spreading	2	GO:0034446	Substrate adhesic	ITGB3MERTKFERMT3RAB1AFNDC3	11	3.34679	4.50E-04	0.0148	0.182973828	3.346787486	3.346787486	0.99955	27
		GO:0031589	Cell-substrate adl	W5P7F8_SHEEPFERMT3RAB1AITGE	25	5.71220	1.94E-06	1.70E-04	0.376955108	5.71219827	5.71219827	0.99999806	27
supramolecular fiber complex	2	GO:0031334	Positive regulatio	LGALS3PLCG2PLEKTFRCGRB2FCHSD	19	3.08092	0.00083	0.0235	0.162893214	3.080921908	3.080921908	0.99917	20
		GO:1902905	Positive regulatio	CCN2CYFIP1DSTNPLEKGSNGRB2FC	21	4.39362	0.0000404	0.0021	0.267778071	4.393618635	4.393618635	0.9999596	20
zymogen activation fibrinolysis	2	GO:0031638	Zymogen activati	CTSCPLAUCYFIP2LGMNMSR1FGGF	9	2.74473	0.0018	0.0434	0.136251027	2.744727495	2.744727495	0.9982	21
		GO:0042730	Fibrinolysis	PLAUFGGCPB2FGAFGB	5	3.16115	0.00069	0.0207	0.168402965	3.161150909	3.161150909	0.99931	21

EnrichmentMap analysis of MMØ_M0_ Pathway

Annatotation	Nodes	EnrichmentMap name	EnrichmentMap	EnrichmentMap Genes	nrichmentMap gs_siz	EnrichmentMap	EnrichmentMap: pv	EnrichmentMap::fd	EnrichmentMap::NE	EnrichmentMap::-log10 (p	EnrichmentMa	EnrichmentMa	_mdcluster
endocrine salivary secretion	5	oas04919	Thyroid hormone	ITGAVPLCG2MAPK3MAP2K1ATP1B	13	2.657577319	2.20E-03	1.33E-02	0.187614836	2.657577319	2.657577319	0.9978	1
		oas04971	Gastric acid secre	PRKCACA2ACTG1ATP1B1PRKACBIT	8	1.966576245	1.08E-02	4.18E-02	0.137882372	1.966576245	1.966576245	0.9892	1
		oas04970	Salivary secretion	VAMP2PRKG1ATP1B1PRKACBATP1	13	4.061480275	8.68E-05	1.20E-03	0.292081875	4.061480275	4.061480275	0.9999132	1
		oas04972	Pancreatic secreti	ATP2B1PRKCACPB2TPCN2CA2RAP1	12	2.886056648	1.30E-03	9.10E-03	0.204095861	2.886056648	2.886056648	0.9987	1
		oas04961	Endocrine and otl	ATP2B1AP2A2-2PRKCAAP2S1SLC8A	9	3.27572413	5.30E-04	4.80E-03	0.231875876	3.27572413	3.27572413	0.99947	1
pyrimidine nucleotides salvage	4	MAP-8956321	Nucleotide salvag	DCKUCK1PUDPW5NRS0_SHEEPADA	7	3.102372909	7.90E-04	1.96E-02	0.170774393	3.102372909	3.102372909	0.99921	2
		oas00240	Pyrimidine metab	DCKENPP1UCK1W5NRS0_SHEEPRR	7	1.903089987	1.25E-02	4.47E-02	0.134969248	1.903089987	1.903089987	0.9875	2
		oas00230	Purine metabolisı	PAPSS1ENPP1AK2W5NRS0_SHEEPA	14	2.468521083	3.40E-03	1.75E-02	0.175696195	2.468521083	2.468521083	0.9966	2
		MAP-15869	Metabolism of nu	AK2W5NRS0_SHEEPRRM2GSRADSS	17	3.180456064	6.60E-04	1.73E-02	0.17619539	3.180456064	3.180456064	0.99934	2
fc mediated phagocytosis	3	oas04650	Natural killer cell	VAV3PLCG2MAPK3MAP2K1RAC1IG	13	3.113509275	7.70E-04	6.20E-03	0.220760831	3.113509275	3.113509275	0.99923	6
		oas04666	Fc gamma R-med	VAV3PLCG2MAPK3PLA2G6INPPL1N	14	4.452225295	3.53E-05	6.50E-04	0.318708664	4.452225295	4.452225295	0.9999647	6
		oas04662	B cell receptor sig	VAV3PLCG2MAPK3INPPL1NFKB1DA	12	4.161780778	6.89E-05	9.90E-04	0.300436481	4.161780778	4.161780778	0.9999311	6
		oas00511	Other glycan degı	GBANEU1HEXAAGAHEXBFUCA1LO	9	5.575118363	2.66E-06	8.02E-05	0.409582563	5.575118363	5.575118363	0.99999734	5

Supplementary Table 14: Enrichment map analysis of differentially abundant proteins (DAPs) in MMØ compared to M0 (monocytes at 3 hours), presenting significantly enriched Gene Ontology biological process (GO-BP) terms and canonical pathways. Functional enrichment analysis was performed using the StringApp (STRING database) within Cytoscape (version 3.10.3)[1], focusing on Gene Ontology (GO) Biological Process (BP) terms and canonical pathways from the KEGG and Reactome databases[2–6]. Enriched GO terms were clustered as functionally coherent groups using the EnrichmentMap plugin[7]. Statistical significance was determined based on a false discovery rate (FDR) threshold of < 0.05.

globo isoglobo series	3	oas00604	Glycosphingolipid	HEXAST3GAL5HEXBLOC10111216	5	2.677780705	2.10E-03	1.26E-02	0.189962945	2.677780705	2.677780705	0.9979	5
		oas00603	Glycosphingolipid	GLAHEXAHEXBLOC101112162NAGA	5	2.795880017	1.60E-03	1.04E-02	0.198296666	2.795880017	2.795880017	0.9984	5
		MAP-216083	Integrin cell surfa	ITGAVCOL1A2ITGB2ITGAMCD44SPP	14	3.744727495	1.80E-04	6.70E-03	0.21739252	3.744727495	3.744727495	0.99982	4
integrin cell surface	3	MAP-1474244	Extracellular matr	C1QTNF3ITGAVCOL1A2P3H1VCANI	33	2.958607315	1.10E-03	2.67E-02	0.157348874	2.958607315	2.958607315	0.9989	4
		oas04512	ECM-receptor int	ITGAVCOL1A2SPP1GP6ITGB3W5Q6	10	2.37675071	4.20E-03	2.01E-02	0.169680394	2.37675071	2.37675071	0.9958	4
		oas04928	Parathyroid horm	PRKCAMAPK3MAP2K1PRKACBPLD1	10	2.22184875	6.00E-03	2.64E-02	0.157839607	2.22184875	2.22184875	0.994	3
parathyroid hormone synthesis	3	oas04720	Long-term potent	PRKCARPS6KA1MAPK3MAP2K1RAF	9	2.853871964	1.40E-03	0.0095	0.202227639	2.853871964	2.853871964	0.9986	3
		oas04270	Vascular smooth	PRKCAMAPK3PLA2G6PRKG1MAP2K	12	2	1.00E-02	3.92E-02	0.140671393	2	2	0.99	3
		oas00562	Inositol phosphat	PLCG2OCRLIMPA1FIG4INPPL1MTM	13	4.432973634	3.69E-05	6.50E-04	0.318708664	4.432973634	4.432973634	0.9999631	8
pips plasma inositol	3	MAP-1660499	Synthesis of PIPs	OCRLPLEKHA2INPP5KINPPL1SASH3	12	3	1.00E-03	2.47E-02	0.160730305	3	3	0.999	8
		MAP-1483257	Phospholipid met	SLC44A2OCRLPLA2G6INPPL1OSBPL	27	2.638272164	2.30E-03	4.45E-02	0.135163999	2.638272164	2.638272164	0.9977	8
		MAP-9013149	RAC1 GTPase cycl	ABL2VAV3ARHGAP10GIT1RRBP1CH	24	2.698970004	2.00E-03	3.98E-02	0.140011693	2.698970004	2.698970004	0.998	7
rhoa gtpase cycle	3	MAP-9013423	RAC3 GTPase cycl	ABL2GIT1OCRLSTBD1ARAP3ARHGA	15	3.187086643	6.50E-04	1.73E-02	0.17619539	3.187086643	3.187086643	0.99935	7
		MAP-8980692	RHOA GTPase cyc	ARHGAP10VAV3ABCD3STBD1RRBP	20	3.107905397	7.80E-04	1.96E-02	0.170774393	3.107905397	3.107905397	0.99922	7
antifolate resistance metabolism	2	oas01523	Antifolate resista	FOLR3FOLR2ALOX12MTHFRABCC1S	7	2.522878745	3.00E-03	1.56E-02	0.18068754	2.522878745	2.522878745	0.997	11
		MAP-196757	Metabolism of fo	SLC46A3FOLR3MTHFD1LFOLR2MTH	7	3.366531544	4.30E-04	1.28E-02	0.189279003	3.366531544	3.366531544	0.99957	11
apoptotic cleavage cellular	2	MAP-111465	Apoptotic cleavag	CAPGSPTAN1LMNALMNB2ADD1LM	11	3.638272164	2.30E-04	8.20E-03	0.208618615	3.638272164	3.638272164	0.99977	10
		MAP-5357801	Programmed Cell	GSDMDMAPK3LMNB1PDCD6IPCAP	26	4.247183569	5.66E-05	0.0028	0.255284197	4.247183569	4.247183569	0.9999434	10
arginine proline histidine	2	oas00330	Arginine and prol	MAOAGOT1OATALDH7A1P4HA1CN	7	2.036212173	9.20E-03	3.67E-02	0.143533394	2.036212173	2.036212173	0.9908	12
		oas00340	Histidine metabo	MAOAALDH7A1HNMTCNDP2ASPA	6	3.602059991	2.50E-04	2.80E-03	0.255284197	3.602059991	3.602059991	0.99975	12
glycosaminoglycan degradation cs	2	MAP-2024101	CS/DS degradatio	HEXAARSBHEXBIDSLOC101112162V	7	3.568636236	2.70E-04	9.40E-03	0.202687215	3.568636236	3.568636236	0.99973	15
		oas00531	Glycosaminoglyca	SGSHHEXAARSBHEXBIDSLOC10111	10	5.982966661	1.04E-06	3.82E-05	0.441793664	5.982966661	5.982966661	0.99999896	15
ion channel transport	2	MAP-77387	Insulin receptor r	ATP6V0D1ATP6V1FATP6V1B2IDEAT	16	8.882728704	1.31E-09	1.73E-07	0.67619539	8.882728704	8.882728704	0.999999999	14
		MAP-983712	Ion channel trans	ATP6V1FATP6V1C1ATP6V1G1ATP6V	26	3.327902142	4.70E-04	1.39E-02	0.18569852	3.327902142	3.327902142	0.99953	14
metabolic pathways metabolism	2	MAP-1430728	Metabolism	SLC46A3MPC2NAPRTCEMIP2PORP	210	12.79588002	1.60E-13	3.91E-11	1.040782324	12.79588002	12.79588002	1	13
		oas01100	Metabolic pathwa	ENPP1ACO1OCRLKYAT3IMPA1PLA2	149	19.82973828	1.48E-20	2.46E-18	1.760906489	19.82973828	19.82973828	1	13
signaling rho gtpases	2	MAP-162582	Signal Transducti	S100A9W5NRI1_SHEEPRAP1BCPT1	226	7.096367484	8.01E-08	7.60E-06	0.511918641	7.096367484	7.096367484	0.99999992	9
		MAP-9716542	Signaling by Rho	OCRLS100A9PDE5ALBRDYNC1H1HN	108	13.20273246	6.27E-14	2.14E-11	1.066958623	13.20273246	13.20273246	1	9
sphingolipid metabolism glycosphingolipid	2	MAP-1660662	Glycosphingolipid	GBANEU1GM2AARSBHEXBASAH1L	15	5.308034897	4.92E-06	3.80E-04	0.34202164	5.308034897	5.308034897	0.99999508	16
		oas00600	Sphingolipid meta	GLASGPL1GBANEU1PSAPASAH1CE	9	3.48148606	3.30E-04	3.20E-03	0.249485002	3.48148606	3.48148606	0.99967	16
									0.200877392	2.920818754	2.920818754	0.9988	10

MMØ, monocyte-derived macrophages differentiated with M-CSF (Macrophage Colony-Stimulating Factor) and M0, Monocytes at 3 hours.

Supplementary Table 15: Differentially abundant proteins (DAPs) of M1 relative to GMØ and their cellular location.

FoldChange	Location	Protein IDs	Gene Names	P-value	FDR
10	CYTO	M4WG34	IL1b	2.61E-03	0.000
7	CYTO	W5Q9Y6	TCAF2	2.25E-05	0.000
5	CYTO	W5PU22	MCUR1	2.99E-02	0.000
5	CYTO	W5PBW1	SDS	1.03E-02	0.000
5	CYTO	W5PB37	RRAS2	1.89E-05	0.000
4	CYTO	W5NXJ0	CLEC4E	7.82E-03	0.000
4	CYTO	W5NQK9	S100A8	8.39E-05	0.000
4	CYTO	W5QB02	TGM2	4.79E-02	0.000
3	CYTO	W5PMT0	XDH	2.14E-02	0.000
3	CYTO	W5QAJ8	DESI1	1.11E-03	0.000
3	CYTO	W5Q980	ACSL4	2.56E-02	0.000
3	CYTO	W5PYW3	LOC101103238	5.28E-02	0.000
3	CYTO	W5QIR2	C7H15orf48	2.16E-03	0.000
3	CYTO	W5PJV4	ASGR2	2.53E-03	0.000
3	CYTO	P21621	IL1B	1.15E-02	0.000
3	CYTO	W5PJR5	LOC101121216	3.78E-03	0.000
3	CYTO	W5NXM6	PTX3	3.37E-02	0.000
3	CYTO	W5QG63	ZMPSTE24	2.26E-02	0.000
3	CYTO	W5PVJ5	CD274	1.33E-02	0.000
3	CYTO	W5QHZ0	CA12	2.11E-03	0.000
3	CYTO	W5P530	LOC101104705	1.86E-04	0.000
3	CYTO	W5QJ54	ARG2	3.79E-03	0.000
3	CYTO	W5NX57	CHM	2.26E-04	0.000
3	CYTO	W5QGV7		3.15E-03	0.000
2	CYTO	W5PW33		1.19E-02	0.000
2	CYTO	W5QI35	IL1RN	3.44E-02	0.000
2	CYTO	W5QCA6	UBE2F	7.93E-03	0.000
2	CYTO	W5P3Z5	EEPD1	1.01E-02	0.000
2	CYTO	W5QHF4	TRIP12	1.33E-03	0.000
2	CYTO	W5QI48	ENSA	3.65E-02	0.000
2	CYTO	W5PZG8	PECAM1	1.22E-02	0.000
2	CYTO	W5NSB0	HSPA2	5.58E-02	0.000
2	CYTO	W5PL05	MICAL1	6.22E-03	0.000
2	CYTO	W5PGD6	BZW2	2.08E-05	0.000
2	CYTO	W5QCW4	PREB	1.58E-02	0.000
2	CYTO	W5PM41	GSDME	4.73E-02	0.000
2	CYTO	W5PVI4	PLGRKT	4.07E-03	0.000
2	CYTO	W5Q3H9	UPP1	5.49E-02	0.000
2	CYTO	W5NQG2	TBC1D2B	6.68E-03	0.000
2	CYTO	W5Q0A6	NSUN2	2.81E-02	0.000
2	CYTO	W5PTU7	CA2	4.71E-03	0.000
2	CYTO	W5NWF5	RARRES1	3.05E-02	0.000
2	CYTO	W5PM73	UAP1	2.30E-02	0.000
2	CYTO	W5P340	SOD2	1.44E-03	0.000
2	CYTO	W5NTM0		1.13E-02	0.000
2	CYTO	W5Q6G5	STX16	2.11E-02	0.000
2	CYTO	W5P4F9	PRKAR2B	3.45E-03	0.000
2	CYTO	W5PZR1		3.77E-02	0.000
2	CYTO	W5NQP8	NFKB2	2.57E-02	0.000
2	CYTO	W5PM91	SLC1A5	5.54E-02	0.000
2	CYTO	W5QC43	ANTXR2	1.29E-03	0.000
2	CYTO	W5PF68	EMB	3.65E-02	0.000
2	CYTO	W5PSS9	RPL21	3.16E-02	0.000
2	CYTO	W5QCW9	TST	3.00E-02	0.000
2	CYTO	W5Q0D9	CPSF3	1.73E-02	0.000
2	CYTO	W5Q3T6	SAMSN1	2.77E-02	0.000
2	CYTO	W5P7P9	ERBIN	3.91E-02	0.000
2	CYTO	W5QGT0	ATP13A3	3.17E-03	0.000
2	CYTO	W5P1M3		1.93E-02	0.000
2	CYTO	W5NRK5	PPT2	2.38E-02	0.000
2	CYTO	W5NXL1	CASK	1.33E-03	0.000
2	CYTO	W5NVF6	NR3C1	6.01E-03	0.000
2	CYTO	W5PSE3	SH3TC1	1.57E-02	0.000

Supplementary Table 15: Differentially abundant proteins (DAPs) of M1 relative to GMØ and their cellular location.

FoldChange	Location	Protein IDs	Gene Names	P-value	FDR
2	CYTO	W5PQB6	ACSL5	2.12E-02	0.000
2	CYTO	W5P2N3	MON2	7.09E-03	0.000
-2	CYTO	W5Q8P9	IRF2BP2	1.23E-02	0.000
-2	CYTO	W5PYG2	VNN1	3.14E-02	0.000
-2	CYTO	W5PUP6	SPATA13	7.86E-03	0.003
-2	CYTO	W5PHJ3	AHR	1.45E-02	0.000
-2	CYTO	W5P8R0	NDUFA4	2.81E-03	0.000
-2	CYTO	W5NUJ2	PEAK1	4.27E-02	0.000
-2	CYTO	W5PFR9	PEX19	1.06E-02	0.000
-2	CYTO	W5PEE6	ITGA2	7.10E-03	0.006
-2	CYTO	W5QB88	COL11A1	4.16E-02	0.000
-2	CYTO	W5PXG3		1.99E-02	0.002
-2	CYTO	W5PUL5	FCGRT	5.36E-02	0.000
-2	CYTO	W5P6G7	TG	4.77E-02	0.000
-2	CYTO	W5Q9H0	MMP2	3.26E-02	0.000
-2	CYTO	W5Q6K0	HEATR3	4.71E-02	0.002
-2	CYTO	W5NYU6	NT5DC2	1.42E-02	0.000
-2	CYTO	W5PHB4	STAT2	4.26E-02	0.000
-2	CYTO	W5PK31	FCGR3A	1.51E-03	0.000
-2	CYTO	W5QDU0	DENND5B	3.31E-02	0.000
-2	CYTO	W5NUU7	CDH5	1.24E-02	0.000
-2	CYTO	W5NR40	FLT4	5.37E-02	0.000
-2	CYTO	W5NX16		4.25E-02	0.000
-2	CYTO	W5Q4T6		5.56E-02	0.000
-2	CYTO	W5PV80	BANK1	4.59E-02	0.000
-2	CYTO	W5QHV3	FABP1	6.99E-03	0.000
-2	CYTO	W5P949	FILIP1	4.83E-02	0.004
-2	CYTO	W5P6B3	PLSCR1	1.16E-03	0.004
-2	CYTO	W5Q8Q2	XPNPEP3	1.90E-03	0.000
-3	CYTO	W5P684	ACTBL2	5.55E-02	0.004
-3	CYTO	W5PVN6		3.91E-02	0.006
-3	CYTO	W5PBL5	SLC44A1	2.48E-04	0.000
-3	CYTO	W5Q3E9	REEP5	8.13E-03	0.000
-3	CYTO	W5P8N6	GAB2	2.54E-02	0.000
-3	CYTO	W5P026	STAB1	4.91E-02	0.000
-3	CYTO	W5PQA6	CYFIP2	4.16E-02	0.000
-3	CYTO	W5QI12	TLN2	2.46E-02	0.005
-3	CYTO	W5PRY2	ACAP1	3.63E-03	0.000
-3	CYTO	W5PUW3		2.00E-02	0.000
-3	CYTO	W5PR99	DHPS	2.71E-02	0.000
-3	CYTO	W5P481	COL1A1	2.19E-03	0.000
-3	CYTO	W5QAB1	HPX	5.94E-03	0.000
-3	CYTO	W5Q3S9	DSN1	3.77E-02	0.001
-3	CYTO	W5QFA5		2.16E-02	0.000
-3	CYTO	W5PFT7	FBP2	2.15E-02	0.004
-3	CYTO	W5PNZ7	RFC1	1.94E-02	0.000
-4	CYTO	W5Q8D1	WDR74	2.69E-02	0.000
-4	CYTO	W5QBA2	ATRX	2.57E-02	0.000
-4	CYTO	W5PDR0	GUCY1B1	1.53E-02	0.000
-4	CYTO	W5NSN4		1.87E-03	0.000
-4	CYTO	W5NRB6	MIDEAS	3.35E-02	0.000
-4	CYTO	W5PPX2	SENP8	1.01E-02	0.003
-4	CYTO	W5NTJ2	TPX2	3.88E-02	0.006
-4	CYTO	W5NQ68	SZT2	1.54E-02	0.000
-5	CYTO	W5PVR2	LOC101108019	2.58E-02	0.008
-5	CYTO	W5P9Q0	PI4K2B	2.19E-02	0.002
-5	CYTO	W5PYP1	NOP14	2.00E-02	0.000
-5	CYTO	W5PKQ7		2.14E-02	0.007
-5	CYTO	W5Q963	FTO	3.09E-02	0.000
-6	CYTO	W5QGQ3	LOC443320	8.01E-03	0.000
-6	CYTO	W5PEL7	HSPG2	1.02E-02	0.000
-7	CYTO	W5Q6Z3	FGF2	1.58E-02	0.000
7	SEC	W5PYW3	LOC101103238	6.00E-03	0.000

Supplementary Table 15: Differentially abundant proteins (DAPs) of M1 relative to GMØ and their cellular location.

FoldChange	Location	Protein IDs	Gene Names	P-value	FDR
6	SEC	W5PAC2	LOC101105044	1.05E-02	0.000
5	SEC	W5P4V3	MMP3	1.28E-02	0.000
4	SEC	W5PGW9	TNFAIP6	1.68E-02	0.000
4	SEC	W5P5K5	MMP1	2.33E-03	0.000
4	SEC	W5PJR5	LOC101121216	1.78E-02	0.000
4	SEC	W5P2M5	LOC101114535	3.50E-04	0.000
4	SEC	W5P530	LOC101104705	1.85E-04	0.000
4	SEC	W5P4C9	MMP12	3.57E-02	0.000
3	SEC	W5PF04	MAN1A1	1.22E-03	0.000
3	SEC	W5PDH4	MMP9	3.39E-04	0.000
3	SEC	W5PNX6	PREP	5.17E-02	0.000
3	SEC	W5QHS2	MGP	3.12E-02	0.000
2	SEC	W5PFC9	LOC101117129	1.07E-02	0.000
2	SEC	W5QHX9	PLBD1	2.23E-02	0.000
2	SEC	W5PG09	PGK2	5.02E-02	0.000
2	SEC	W5Q691	ISOC1	1.04E-02	0.000
2	SEC	W5P1J8	LOC101113086	4.86E-02	0.000
2	SEC	W5PJC2	PSMB3	3.14E-02	0.000
2	SEC	W5PDR7	C8A	4.93E-02	0.002
2	SEC	W5PEN2	TPD52	4.91E-02	0.000
2	SEC	W5QF71	PLEK	1.91E-02	0.000
2	SEC	W5P3N6	LOC101112162	4.53E-02	0.000
2	SEC	W5PXR1	ENPP1	4.83E-02	0.000
2	SEC	W5NVB9	ARPC5	1.15E-02	0.000
2	SEC	W5PPG3	ALDH9A1	2.48E-02	0.000
2	SEC	W5P359	STIP1	4.39E-02	0.000
-2	SEC	W5NU86	GLA	3.90E-02	0.000
-3	SEC	W5PTZ8	LOC101114959	4.37E-02	0.000
-3	SEC	W5P375	TCP1	1.41E-02	0.000
-4	SEC	W5PTS4	LOC101114275	2.64E-02	0.000

M1, pro-inflammatory macrophages activated with GM-CSF/ LPS/ INF-γ and GMØ, monocyte-derived macrophages differentiated with GM-CSF (Granulocyte-Macrophage Colony-Stimulating Factor). Differentially abundant proteins (DAPs) were identified using a threshold of false discovery rate (FDR, q-value) ≤ 0.05 and absolute fold change ≥ 2. Red-highlighted cells indicate shared differentially abundant proteins (DAPs) between the cell lysate (CYTO) and secretome (SEC) compartments in M1 relative to GMØ, whereas non-highlighted cells represent compartment-specific DAPs unique to either the whole cell lysate or secretome.

Supplementary Table 16: Differentially abundant proteins (DAPs) of M1 relative to Mo and their cellular location.

FoldChange	Location	Protein IDs		P-value	FDR
10	CYTO	W5PEB0	FABP7	1.08E-07	0
8	CYTO	W5NU86	GLA	1.03E-04	0
8	CYTO	W5P530	LOC101104705	8.77E-04	0
8	CYTO	W5PAM4	CTSA	5.94E-07	0
8	CYTO	W5PAC2	LOC101105044	1.79E-04	0
8	CYTO	W5PCH3	SCIN	6.39E-04	0
7	CYTO	W5PF33	GM2A	2.98E-06	0
7	CYTO	W5PU22	MCUR1	2.40E-08	0
7	CYTO	A9YUY8	FABP4	5.31E-04	0
7	CYTO	W5NY01		5.84E-05	0
7	CYTO	W5PRI6	MRC1	5.15E-06	0
7	CYTO	W5P9J8	BLVRB	1.23E-04	0
7	CYTO	W5PFV1	CTSL	2.41E-05	0
7	CYTO	W5PT76	GPNMB	1.68E-05	0
7	CYTO	P51977	ALDH1A1	9.70E-04	0
7	CYTO	W5PIQ6	MSR1	1.35E-06	0
7	CYTO	W5P9G8	PLD3	2.28E-03	0
7	CYTO	W5PQR0	NIBAN2	1.61E-08	0
7	CYTO	W5PJS4	EMILIN2	3.35E-06	0
6	CYTO	W5P4C9	MMP12	3.14E-05	0
6	CYTO	W5PZB2	CD68	2.18E-06	0
6	CYTO	W5Q3H9	UPP1	2.22E-04	0
6	CYTO	W5PKY1	HNMT	4.26E-05	0
6	CYTO	W5Q233	VCAN	6.68E-06	0
6	CYTO	W5NRS0		5.43E-06	0
6	CYTO	W5QHL1	FCGR1A	1.49E-05	0
6	CYTO	W5PBM9	SCPEP1	2.10E-04	0
6	CYTO	W5PFY5	ASAH1	3.43E-04	0
6	CYTO	W5P093	NQO1	1.10E-06	0
6	CYTO	W5QHZ0	CA12	3.68E-05	0
6	CYTO	W5PGH4	TNKS1BP1	2.28E-02	0
6	CYTO	W5P1A5	GBA1	5.85E-06	0
6	CYTO	W5P640	LMNA	2.76E-03	0
6	CYTO	Q9MZS8	CTSD	6.47E-05	0
6	CYTO	W5PCE0	PLBD2	2.63E-04	0
6	CYTO	W5QFU8	CD86	8.39E-05	0
6	CYTO	W5PDH4	MMP9	1.70E-05	0
6	CYTO	W5NVW7	NAGLU	1.97E-04	0
6	CYTO	W5Q263	ICAM1	6.54E-06	0
6	CYTO	W5PUH5	LGMN	2.92E-04	0
6	CYTO	W5P0K8	CLIC2	3.20E-03	0
6	CYTO	W5P1H0	CTSC	3.86E-04	0
6	CYTO	W5P2F1	FOLR2	7.71E-05	0
6	CYTO	W5PI56	DAB2	2.48E-04	0
6	CYTO	W5Q0A3	TLR2	1.58E-05	0
6	CYTO	W5Q2Y1	PLXNC1	5.14E-04	0
6	CYTO	P35623	SHMT1	8.91E-06	0
6	CYTO	W5PTU7	CA2	1.27E-05	0
6	CYTO	W5QBM4	ALCAM	3.10E-05	0
6	CYTO	M4WG34	IL1b	3.13E-02	0
6	CYTO	W5PK56	FCGR2B	6.95E-03	0
5	CYTO	W5PBS4	LRP1	5.24E-05	0
5	CYTO	W5PGC5	GALM	2.73E-04	0
5	CYTO	W5QC89	HEXA	3.39E-06	0
5	CYTO	W5Q940	SHTN1	2.26E-04	0
5	CYTO	W5PAQ4	FUCA1	1.00E-05	0
5	CYTO	W5PRG9	HAL	3.10E-07	0
5	CYTO	W5P8H9	SGPL1	3.56E-05	0

Supplementary Table 16: Differentially abundant proteins (DAPs) of M1 relative to Mo and their cellular location.

FoldChange	Location	Protein IDs		P-value	FDR
5	CYTO	W5PGF4	PLAUR	2.99E-03	0
5	CYTO	W5PYW0	TCIRG1	8.76E-05	0
5	CYTO	W5PE67		5.35E-04	0
5	CYTO	W5PMT0	XDH	1.30E-03	0
5	CYTO	W5Q612	TRPV2	8.23E-07	0
5	CYTO	W5QBZ7	NAGA	3.87E-04	0
5	CYTO	W5NYU9	MPP1	3.83E-06	0
5	CYTO	W5QI70	CTSS	1.85E-04	0
5	CYTO	W5NXM6	PTX3	7.86E-04	0
5	CYTO	W5PV43	LRPAP1	4.68E-07	0
5	CYTO	W5PZ47	CTSH	1.44E-02	0
5	CYTO	W5PA90	AGA	3.08E-06	0
5	CYTO	W5QCD6	IDH1	9.05E-08	0
5	CYTO	W5QI00	LACTB	8.92E-06	0
5	CYTO	W5PXR1	ENPP1	6.35E-03	0
5	CYTO	W5QIW1	LGALS3	4.07E-04	0
5	CYTO	W5PE73	SMPDL3A	1.64E-05	0
5	CYTO	W5QCL8	NPL	3.94E-05	0
5	CYTO	W5QI40	MYO1E	1.76E-04	0
5	CYTO	W5PJV4	ASGR2	5.64E-05	0
5	CYTO	W5P3H8	IGF2R	3.04E-04	0
5	CYTO	W5Q5W2	LOC101110539	3.20E-06	0
5	CYTO	W5Q9Y6	TCAF2	1.12E-05	0
5	CYTO	W5NSH8	NPC2	1.37E-06	0
5	CYTO	W5NUC8	ARMCX3	1.20E-05	0
5	CYTO	W5NTZ3	RENBP	3.14E-05	0
5	CYTO	W5QGG0	TFRC	1.46E-05	0
5	CYTO	P79365	SLC2A1	1.71E-02	0.0051785
5	CYTO	W5PNY4	PDXK	1.17E-03	0
5	CYTO	W5NUI6	SGSH	8.90E-05	0
5	CYTO	W5Q6N3	LOC101115115	2.74E-04	0
5	CYTO	W5NYL0	MAOA	5.75E-05	0
5	CYTO	W5PDH7	NPC1	1.74E-06	0
5	CYTO	W5PM91	SLC1A5	3.61E-02	0
5	CYTO	W5QH35	CAPG	8.07E-06	0
5	CYTO	W5Q7I8	TAX1BP3	6.17E-03	0
5	CYTO	W5PVJ5	CD274	9.14E-06	0
5	CYTO	G3M9U4	ACP5	2.36E-05	0
5	CYTO	W5P150		7.22E-05	0.0002213
5	CYTO	W5P407		1.04E-03	0
5	CYTO	W5PMX5	C5AR1	5.25E-04	0
5	CYTO	W5PY08		5.18E-04	0
5	CYTO	W5Q8J3	RRBP1	1.65E-03	0
5	CYTO	W5QDF4	GSTM3	9.51E-04	0
5	CYTO	W5P5W6	NDRG1	2.01E-03	0
5	CYTO	W5NZJ7	FGR	2.30E-03	0
5	CYTO	W5PXY4	CLEC5A	8.22E-03	0
5	CYTO	W5PWX3	CRYL1	2.53E-05	0
5	CYTO	W5P8M9		1.05E-04	0
5	CYTO	P05028	ATP1B1	3.63E-04	0
5	CYTO	W5PLB8	EPB41L3	1.04E-03	0
4	CYTO	W5NQS7	IFNGR1	4.08E-05	0
4	CYTO	W5PRG8	CREG1	3.31E-04	0
4	CYTO	W5Q678	SEC24D	4.66E-04	0
4	CYTO	W5PII2	ATG4C	1.80E-02	0
4	CYTO	W5PAM2	RHBDF2	3.20E-02	0
4	CYTO	W5NXJ0	CLEC4E	4.31E-03	0
4	CYTO	W5Q9M9	GK	1.63E-03	0

Supplementary Table 16: Differentially abundant proteins (DAPs) of M1 relative to Mo and their cellular location.

FoldChange	Location	Protein IDs		P-value	FDR
4	CYTO	W5NZ62	GNS	5.35E-04	0
4	CYTO	W5P3Q3	LOC100101238	4.59E-03	0
4	CYTO	W5PJR5	LOC101121216	2.72E-05	0.0002036
4	CYTO	P82197	PDXK	1.44E-03	0
4	CYTO	W5NW80	GAA	7.61E-04	0
4	CYTO	W5PIS6	NHLRC3	4.17E-04	0
4	CYTO	W5NZV3	HMOX2	7.47E-03	0
4	CYTO	W5NYE0	ATP6V0D1	5.51E-03	0
4	CYTO	W5PNP1	MFGE8	8.75E-04	0
4	CYTO	W5PTG5	ATP6V0D2	9.66E-03	0
4	CYTO	W5NZK6	PLA2G15	8.65E-05	0
4	CYTO	W5QHG1	EPS8	6.59E-03	0
4	CYTO	W5PUL5	FCGRT	2.40E-03	0
4	CYTO	W5PZG5	OCRL	7.78E-05	0
4	CYTO	W5QEM8	LOC101111528	1.45E-05	0
4	CYTO	W5P2V3	PEPD	6.88E-03	0
4	CYTO	W5QHU8	FNDC3B	2.45E-05	0
4	CYTO	W5QB71	AMDHD2	1.30E-03	0
4	CYTO	W5PQ75	HSPH1	2.68E-03	0
4	CYTO	W5NQG2	TBC1D2B	8.63E-05	0
4	CYTO	W5QA16		8.44E-06	0
4	CYTO	W5P6H9	ACP2	4.12E-03	0
4	CYTO	W5P3L5	RNF13	4.75E-06	0
4	CYTO	W5Q3U3	LOC101102156	9.03E-04	0
4	CYTO	W5P539	TYROBP	8.91E-03	0
4	CYTO	W5Q2R5	SDF2L1	8.72E-03	0
4	CYTO	W5PKU3		5.16E-05	0
4	CYTO	W5QBV7	CD44	1.05E-03	0
4	CYTO	W5QFU4		2.09E-03	0
4	CYTO	W5QI35	IL1RN	2.59E-02	0
4	CYTO	W5P369	AP2A2	1.98E-02	0
4	CYTO	W5Q9F0	LOC101102694	3.40E-03	0.0019059
4	CYTO	W5Q5C8	SOAT1	1.35E-05	0
4	CYTO	W5PYH9		6.86E-03	0
4	CYTO	W5NXH3	LOC101106542	5.98E-05	0
4	CYTO	W5Q0F1	LIPA	3.77E-03	0
4	CYTO	W5Q2U7	PLEC	1.06E-03	0
4	CYTO	W5PUW2	IFI30	1.97E-02	0
4	CYTO	C5IJ93	RAB9A	1.51E-06	0
4	CYTO	W5Q2B3	SUSD5	2.21E-02	0.0085532
4	CYTO	W5P3S0		3.60E-06	0
4	CYTO	W5NUJ5		8.68E-04	0
4	CYTO	W5PWQ4	EPB41L2	4.47E-02	0
4	CYTO	W5QI78	CTSK	1.53E-02	0
4	CYTO	W5PHG1	CDA	4.30E-03	0
4	CYTO	W5PEE9	LAMP1	5.26E-03	0
4	CYTO	W5NU23	FUCA2	1.69E-05	0
4	CYTO	W5P3H9	PICALM	2.43E-02	0
4	CYTO	W5PBC0		1.45E-03	0
4	CYTO	W5NTZ2	LOC101121821	2.46E-02	0
4	CYTO	W5Q284	HAPLN1	7.97E-03	0
4	CYTO	W5Q4U5	CPT1A	3.99E-04	0
4	CYTO	W5PE92	GRN	4.06E-05	0
4	CYTO	W5PBW1	SDS	1.72E-02	0
4	CYTO	W5Q7R3	LOC101118736	3.96E-03	0
4	CYTO	W5PGS4	FABP5	4.70E-05	0
4	CYTO	W5P2W1	NEU1	4.72E-05	0
4	CYTO	W5QHR5	PLEKHO2	6.37E-05	0

Supplementary Table 16: Differentially abundant proteins (DAPs) of M1 relative to Mo and their cellular location.

FoldChange	Location	Protein IDs		P-value	FDR
4	CYTO	W5PKA1	LOC101116157	1.96E-03	0
4	CYTO	W5QHF2	LOC114117536	1.12E-02	0
4	CYTO	W5NZX4	KATNAL2	1.12E-02	0.0035607
4	CYTO	W5PY53	FTH1	1.44E-02	0
4	CYTO	W5Q1W2	SDCBP	3.29E-02	0
4	CYTO	P83205	CTSB	1.34E-03	0
4	CYTO	W5NWX4		3.26E-03	0
4	CYTO	W5Q5T7	ATP6V1C1	6.88E-03	0
4	CYTO	W5PW33		9.37E-04	0
4	CYTO	W5P340	SOD2	1.32E-02	0
4	CYTO	W5PG10	PAPSS1	2.69E-03	0
4	CYTO	W5NVR9	C21H11orf54	1.03E-05	0
4	CYTO	W5QGV7		2.21E-04	0
4	CYTO	W5P4X6	LOC101104287	2.41E-03	0
4	CYTO	W5PBR7	P4HA1	7.81E-04	0
4	CYTO	W5PGG5	CD84	5.65E-03	0
4	CYTO	W5PXS1	RAB3IL1	4.05E-04	0
4	CYTO	W5Q6C5	GGA2	1.34E-02	0
4	CYTO	W5Q0Y4	ZFAND6	1.59E-02	0
4	CYTO	W5QI36	HEBP1	2.95E-03	0
4	CYTO	W5Q1M0	GLB1	1.78E-04	0
4	CYTO	W5PNK3	ACAN	8.08E-05	0
4	CYTO	W5NYK9	CALU	3.67E-02	0
4	CYTO	W5Q3N1	CTSZ	1.21E-06	0
4	CYTO	W5QEH0	TWF1	2.69E-04	0
4	CYTO	W5P703	WFS1	9.28E-05	0
4	CYTO	W5NX56	SPP1	1.16E-03	0
4	CYTO	W5QEH8	CTTNBP2NL	3.38E-05	0
4	CYTO	W5Q4B1	MYCBP2	9.91E-03	0
4	CYTO	W5QBP6	GLUL	3.52E-03	0
4	CYTO	W5Q0U0	P2RX4	4.88E-03	0
4	CYTO	Q6XXL8	DYNLT3	9.29E-03	0
4	CYTO	W5QBG8	PPFIA1	7.13E-04	0
4	CYTO	W5PP04	GNG12	8.83E-03	0
4	CYTO	W5P3N6	LOC101112162	6.21E-04	0
4	CYTO	W5PTE6	CEMIP2	7.24E-04	0
4	CYTO	W5QGT0	ATP13A3	3.85E-03	0
4	CYTO	Q9MZD1	SLC17A5	9.12E-04	0
4	CYTO	W5PGL9		3.27E-05	0
4	CYTO	W5NUG3	GNPDA1	3.34E-02	0
4	CYTO	W5NVV1		1.97E-02	0
3	CYTO	W5Q3V0	IL18	4.41E-05	0
3	CYTO	W5PG41	H6PD	5.41E-04	0
3	CYTO	W5PZG8	PECAM1	4.28E-04	0
3	CYTO	W5NYL7	MTHFD1L	9.97E-06	0
3	CYTO	W5QG92	OSBPL11	7.58E-05	0
3	CYTO	W5QGV5	DOCK10	3.82E-02	0
3	CYTO	W5NUE3	PRDX1	1.42E-03	0
3	CYTO	W5QBA2	ATRX	3.57E-02	0
3	CYTO	W5QBR5	BMP2K	1.01E-02	0
3	CYTO	W5NXT4		1.50E-02	0
3	CYTO	W5Q8K4	SLC3A2	1.62E-02	0
3	CYTO	W5PC32	PGM3	9.80E-04	0
3	CYTO	W5QAU1		5.49E-02	0
3	CYTO	W5P3C6	LOC101111906	2.61E-02	0
3	CYTO	W5PEC0	PKIB	5.62E-03	0
3	CYTO	W5PL89	GSR	5.77E-04	0
3	CYTO	W5Q7X3	GNPNAT1	1.56E-02	0

Supplementary Table 16: Differentially abundant proteins (DAPs) of M1 relative to Mo and their cellular location.

FoldChange	Location	Protein IDs		P-value	FDR
3	CYTO	W5PYW3	LOC101103238	3.76E-02	0
3	CYTO	W5NTE2	PSMG4	1.77E-02	0
3	CYTO	W5P4A8	RNASET2	2.56E-03	0
3	CYTO	W5NZA6	RNF121	2.09E-04	0
3	CYTO	W5NZ70	LGALS3BP	2.86E-02	0
3	CYTO	W5QIC7	YBX3	4.19E-03	0
3	CYTO	W5QCG9	SRXN1	6.25E-03	0
3	CYTO	W5PMA0	AP2S1	1.24E-03	0
3	CYTO	W5Q700	APPL2	1.09E-04	0
3	CYTO	W5NX08	WASHC2C	2.49E-02	0
3	CYTO	W5PF68	EMB	2.25E-04	0.0002152
3	CYTO	W5QCA6	UBE2F	1.91E-06	0
3	CYTO	W5Q777	HS1BP3	7.19E-06	0
3	CYTO	W5P4H0	DNASE1L1	1.81E-02	0
3	CYTO	W5PVE3	LOC101115252	3.39E-02	0
3	CYTO	W5P2F6	FAF1	1.07E-05	0
3	CYTO	W5PGA9	NCSTN	1.34E-03	0
3	CYTO	W5PFP5	PLIN3	9.34E-03	0
3	CYTO	W5QG63	ZMPSTE24	1.28E-04	0
3	CYTO	W5NU94	PSTPIP1	2.51E-03	0
3	CYTO	W5Q7Z6	DIP2B	3.43E-04	0
3	CYTO	W5Q6S3	WASHC4	4.39E-04	0
3	CYTO	W5P536	TRIP10	8.64E-04	0
3	CYTO	P04074	ATP1A1	2.84E-03	0
3	CYTO	W5PFE6	ACOX1	1.19E-04	0
3	CYTO	W5P2D9	SNX9	4.20E-02	0
3	CYTO	W5PC82	ME1	3.55E-02	0
3	CYTO	W5Q9U4	LOC101104050	1.00E-03	0
3	CYTO	W5PC06	SIRPA	1.90E-02	0
3	CYTO	W5NTW3	ITIH1	1.69E-02	0
3	CYTO	W5PIE4	CLPTM1	5.41E-03	0
3	CYTO	W5PEP1	ATP1A2	5.68E-03	0.0002083
3	CYTO	W5QBE4	FGL2	2.71E-02	0
3	CYTO	W5PCK8	LOC100135455	3.80E-02	0
3	CYTO	W5QFD0	RRAGC	5.97E-03	0
3	CYTO	W5PDZ1		5.56E-04	0
3	CYTO	W5P1Q0	AP1B1	1.47E-04	0
3	CYTO	W5PZ94	ACO1	2.06E-02	0
3	CYTO	W5QHX1	EIF5A2	1.28E-02	0
3	CYTO	W5P3W6	OSBPL9	2.72E-04	0
3	CYTO	W5PMU8	GAB1	1.54E-03	0
3	CYTO	O18882	ATP6V0C	4.40E-03	0
3	CYTO	W5Q5N6	BST-2B	5.07E-04	0
3	CYTO	W5Q5P5	GGH	1.94E-03	0
3	CYTO	W5Q1W7	PALLD	5.28E-04	0
3	CYTO	W5PVC8	ERGIC3	8.48E-05	0
3	CYTO	W5NRF7	MAP3K20	4.07E-04	0
3	CYTO	W5PT36	RBM47	3.65E-05	0
3	CYTO	W5NVV6	DNAJC3	7.28E-03	0
3	CYTO	W5PN65	PI4K2A	1.41E-02	0
3	CYTO	W5Q3Y3	SFXN3	2.65E-03	0
3	CYTO	W5Q634	BLVRA	1.96E-02	0
3	CYTO	W5QEE5	TRAPPC3	3.16E-02	0
3	CYTO	W5P743	GLMP	3.47E-04	0
3	CYTO	W5PJN7	HOMER3	4.59E-04	0
3	CYTO	W5Q0G8	IMPA1	2.21E-03	0
3	CYTO	W5PK00	HNRNPH2	3.48E-02	0
3	CYTO	W5QGH9	WDR3	4.89E-02	0.0002094

Supplementary Table 16: Differentially abundant proteins (DAPs) of M1 relative to Mo and their cellular location.

FoldChange	Location	Protein IDs		P-value	FDR
3	CYTO	W5PAX1	GCLC	1.07E-02	0
3	CYTO	W5PB38		1.36E-04	0
3	CYTO	W5QAJ8	DESI1	1.60E-03	0
3	CYTO	W5Q8G9	SPG21	1.92E-04	0
3	CYTO	W5PQV2	LMBRD1	1.03E-02	0
3	CYTO	W5QH13	VPS39	1.42E-04	0
3	CYTO	W5P3I5	CNDP2	1.83E-04	0
3	CYTO	W5Q318	CCNH	1.40E-02	0.0042862
3	CYTO	W5NRW1	FBXO6	1.41E-02	0
3	CYTO	W5PH35	LOC101119706	1.21E-03	0
3	CYTO	W5PJ40	EIF2AK2	4.02E-02	0
3	CYTO	W5PGX8	DNAJC13	1.82E-02	0
3	CYTO	W5PMX7		1.92E-02	0
3	CYTO	W5PCI1	NLRP3	3.39E-04	0
3	CYTO	W5Q7J0	APOB	4.85E-02	0
3	CYTO	W5P3B0	INPPL1	3.92E-03	0
3	CYTO	W5PM73	UAP1	4.13E-03	0
3	CYTO	W5P164	LAMTOR1	1.06E-02	0
3	CYTO	W5QDN8	SLC48A1	2.00E-04	0.0002157
3	CYTO	W5PCD0	FUBP3	8.35E-03	0
3	CYTO	W5NQZ9	GSDMD	1.23E-03	0
3	CYTO	W5PDK4	AIF1	4.58E-03	0
3	CYTO	W5PG74	IRGQ	3.15E-02	0
3	CYTO	W5QEL6	PCYOX1	2.32E-02	0
3	CYTO	W5PIQ4	LOC101120093	2.51E-02	0.0002141
3	CYTO	W5NV06	ATP6V0A1	2.69E-02	0
3	CYTO	W5PCR6	PIK3CD	2.89E-02	0
3	CYTO	Q10994	CSTB	5.09E-06	0
3	CYTO	P29330	FDX1	6.52E-05	0
3	CYTO	W5PEX1	WASHC5	1.98E-02	0
3	CYTO	W5QDJ5	LOC101122319	1.05E-02	0
3	CYTO	W5PAJ2	PSAP	5.27E-03	0
3	CYTO	W5NS94		3.79E-05	0
3	CYTO	W5QCU8	NOP9	3.57E-02	0.0013433
3	CYTO	W5PBE7	ELOA	1.90E-02	0.0002109
3	CYTO	W5PFB1	TOR1B	1.13E-03	0
3	CYTO	W5NVG6	PTPN9	7.80E-05	0
3	CYTO	W5PVC1	SYNJ1	3.66E-03	0
3	CYTO	W5P9K4	NUFIP2	1.53E-02	0
3	CYTO	W5Q8Y5	HDLBP	4.10E-02	0
3	CYTO	W5Q420		3.19E-03	0
3	CYTO	W5PRK6	STAU1	2.29E-03	0
3	CYTO	W5Q686	TPP1	2.59E-03	0
3	CYTO	W5PBY3	OSBPL1A	3.88E-02	0
3	CYTO	W5NYV5	SPAG9	1.54E-02	0
3	CYTO	W5QAP3	TOM1	7.54E-03	0
3	CYTO	W5PS60		4.48E-04	0.0002177
3	CYTO	W5P434	NAGPA	1.01E-03	0
3	CYTO	W5PFH4	MAPKAPK3	2.32E-02	0
3	CYTO	W5Q6T1	ARSB	3.36E-02	0
3	CYTO	W5Q8I7	LOC101122123	1.13E-03	0
3	CYTO	W5PG28	NTMT1	2.44E-03	0
3	CYTO	W5Q0C3	KIF13B	5.39E-03	0
3	CYTO	W5NTM0		1.12E-03	0.0002191
3	CYTO	W5PH85	ITGAX	2.17E-02	0
3	CYTO	W5NW35	MYOF	1.94E-02	0
3	CYTO	W5Q3T6	SAMSN1	8.67E-03	0
3	CYTO	W5PB61		4.17E-03	0.0002163

Supplementary Table 16: Differentially abundant proteins (DAPs) of M1 relative to Mo and their cellular location.

FoldChange	Location	Protein IDs		P-value	FDR
3	CYTO	W5PJT1	PLAC9	1.59E-02	0
3	CYTO	W5NTV0	TMEM165	1.19E-03	0.002641
3	CYTO	W5QE37	SLC11A1	3.52E-02	0
3	CYTO	W5PFE7	ACOX1	7.76E-05	0
3	CYTO	W5P726	PRCP	1.73E-02	0
3	CYTO	W5P3Z5	EEPD1	1.12E-02	0
3	CYTO	W5PGK6	HNRNPLL	2.14E-02	0
3	CYTO	W5Q553	ITGAV	5.02E-04	0
3	CYTO	W5PCS4		9.59E-05	0
3	CYTO	W5QF63	EMC4	8.58E-03	0.0002034
3	CYTO	W5QIJ6	SPPL2A	1.72E-02	0
3	CYTO	W5QC46	ANKFY1	3.52E-02	0
3	CYTO	W5NXU7	HCK	2.72E-02	0
3	CYTO	W5PF85	PEA15	4.79E-05	0
3	CYTO	P21621	IL1B	1.84E-02	0
3	CYTO	W5Q1R9	WASHC3	4.91E-02	0
3	CYTO	Q6XUZ5	IDH1	2.16E-02	0
3	CYTO	W5PT68	FLNB	5.10E-02	0
3	CYTO	W5NRK5	PPT2	1.91E-03	0
3	CYTO	W5QAQ4	NRP2	6.05E-04	0
3	CYTO	W5NYK1	PMVK	1.35E-04	0
3	CYTO	W5QC43	ANTXR2	4.58E-03	0
3	CYTO	W5P5Q2	MVP	2.96E-02	0
3	CYTO	W5P8S0	LY96	2.81E-03	0
3	CYTO	W5NVK6	DAGLB	3.87E-03	0
3	CYTO	W5P6L1	RASGRP4	1.42E-02	0
3	CYTO	Q29524	LPL	2.19E-02	0
3	CYTO	W5PZJ8	ASRGL1	5.92E-03	0
3	CYTO	W5PBJ4	ARHGAP10	3.14E-03	0
3	CYTO	W5NQ85	IDE	1.30E-02	0
3	CYTO	W5NUQ8	GCC1	2.29E-03	0
3	CYTO	W5NX57	CHM	2.28E-04	0.0002047
3	CYTO	W5QA17	ATXN2	1.37E-02	0
3	CYTO	W5NT19	SPAG1	8.37E-03	0
3	CYTO	W5P7F8		2.43E-02	0
3	CYTO	W5PNS2	TLR7	4.85E-03	0
3	CYTO	W5PER8	WDR91	5.63E-03	0
3	CYTO	W5QGR5	WDFY1	3.06E-02	0
3	CYTO	W5PF22	KMO	3.86E-02	0
3	CYTO	W5QBJ0	ANXA3	4.10E-03	0
3	CYTO	W5QGJ2	CHP1	7.46E-03	0
3	CYTO	W5P7B1	SIRT2	3.77E-02	0
3	CYTO	W5Q2V0	YKT6	1.46E-04	0
3	CYTO	W5PN70		1.34E-02	0
3	CYTO	W5P3S7	ATP6AP1	3.59E-02	0
3	CYTO	W5PGC6	DPP9	1.90E-02	0
3	CYTO	W5PVI4	PLGRKT	8.43E-03	0
3	CYTO	W5QHA0	AGFG1	5.41E-02	0
3	CYTO	W5PFR8	FBP1	3.80E-02	0
3	CYTO	W5PMF1	TPMT	1.48E-02	0
3	CYTO	W5PEZ1		1.22E-03	0
3	CYTO	W5PCM4	LUZP1	3.01E-06	0
3	CYTO	W5P5E7		8.87E-03	0
3	CYTO	W5PUJ2	LAMP2	3.50E-02	0
3	CYTO	W5PSG0		1.56E-02	0
3	CYTO	W5P333		9.74E-03	0
3	CYTO	W5PPT8	GLIPR2	2.93E-03	0
3	CYTO	W5P6U2		2.08E-03	0

Supplementary Table 16: Differentially abundant proteins (DAPs) of M1 relative to Mo and their cellular location.

FoldChange	Location	Protein IDs		P-value	FDR
3	CYTO	W5PN60	ABR	1.84E-02	0
2	CYTO	W5PP47		3.65E-02	0
2	CYTO	W5QI12	TLN2	5.08E-02	0.0051718
2	CYTO	W5Q3B2	TYK2	2.46E-03	0
2	CYTO	W5PWR6	KCNAB2	1.16E-02	0
2	CYTO	W5QJ54	ARG2	9.11E-03	0
2	CYTO	W5PWG8	LOC101115420	2.38E-02	0
2	CYTO	W5Q2H2	SLC12A9	2.46E-05	0
2	CYTO	W5Q1K4	DICER1	3.62E-02	0
2	CYTO	W5P2N4	AHCYL2	3.62E-03	0
2	CYTO	W5NSZ7	RAB31	4.12E-02	0
2	CYTO	W5PSD4	GYS1	3.93E-02	0
2	CYTO	W5PU75	GNA11	1.40E-02	0
2	CYTO	W5NPQ5		3.57E-02	0
2	CYTO	W5P2U4	LOC101105644	5.51E-02	0
2	CYTO	W5PES1	IARS1	1.09E-02	0
2	CYTO	W5PUL4	MTMR6	8.36E-03	0
2	CYTO	W5NS07	HM13	2.35E-06	0
2	CYTO	W5NWX7	CLEC4A	6.50E-03	0
2	CYTO	W5NVF6	NR3C1	2.14E-02	0
2	CYTO	W5P374		2.71E-02	0
2	CYTO	W5P8F3	AGPAT5	4.45E-04	0
2	CYTO	W5P363	VAT1	1.76E-02	0
2	CYTO	W5P366	FAM50A	1.25E-02	0
2	CYTO	W5PSD7	RAP2C	5.56E-03	0
2	CYTO	W5PUU2	RRAS	1.10E-02	0
2	CYTO	W5Q4N4	TOR3A	6.29E-03	0
2	CYTO	W5Q411	HSPA13	1.87E-03	0
2	CYTO	W5PVT3	GALNS	1.02E-02	0
2	CYTO	W5PX29	TMEM9B	4.13E-02	0
2	CYTO	W5QCL1	NCF4	4.88E-02	0
2	CYTO	W5PQT3	LOC101105107	1.92E-03	0
2	CYTO	W5NWR6	LXN	2.31E-02	0
2	CYTO	W5PER3	TM9SF3	9.65E-03	0
2	CYTO	W5Q560	OGFOD3	9.90E-03	0
2	CYTO	W5NSS1	PPP4R1	5.21E-03	0
2	CYTO	W5QIE9	BSDC1	1.62E-02	0.0002162
2	CYTO	W5Q922	LOC101105383	2.61E-02	0
2	CYTO	W5PUV6	NCAPD3	2.12E-02	0
2	CYTO	W5PWA8	HSPB1	5.70E-03	0
2	CYTO	W5Q5K0		3.01E-03	0
2	CYTO	W5PPS6		5.89E-05	0
2	CYTO	W5PES0	STX4	2.89E-03	0
2	CYTO	W5PRQ0	RBM3	3.50E-02	0
2	CYTO	W5P1F8	SEC23IP	4.20E-02	0
2	CYTO	W5PD25	CYTIP	9.35E-03	0
2	CYTO	W5Q8J8	VPS41	6.09E-03	0
2	CYTO	W5PMB1	SNX3	1.53E-03	0
2	CYTO	W5PPY5	CD2AP	2.74E-04	0
2	CYTO	W5PYB9	ABCA6	3.22E-02	0
2	CYTO	W5Q0M7	RAP2B	1.51E-02	0
2	CYTO	W5QEU6	ANXA4	2.46E-02	0
2	CYTO	W5P6V4	GLG1	3.99E-02	0
2	CYTO	W5PKV1	DNASE2	1.89E-02	0
2	CYTO	W5Q0N7	IFIT2	5.13E-02	0
2	CYTO	W5NZ80	NIBAN1	5.21E-02	0
2	CYTO	W5PM50	UPF1	4.06E-02	0
2	CYTO	W5NWG5	LOC101118849	1.11E-02	0

Supplementary Table 16: Differentially abundant proteins (DAPs) of M1 relative to Mo and their cellular location.

FoldChange	Location	Protein IDs		P-value	FDR
2	CYTO	W5Q396		2.19E-02	0
2	CYTO	W5PLW1	CTNND1	4.93E-02	0
2	CYTO	W5PXH4	PTPRE	1.51E-04	0
2	CYTO	W5NUE5	SLC38A7	6.66E-03	0
2	CYTO	W5QAS2	STX6	3.01E-02	0
2	CYTO	W5PSX7	FES	4.47E-02	0
2	CYTO	W5PUI3	GOLGA1	4.48E-05	0
2	CYTO	W5NRR6	MGAT2	5.66E-04	0
2	CYTO	W5PA59	LOC101117015	1.94E-04	0
2	CYTO	W5Q2L9		2.49E-02	0
2	CYTO	W5P1U9	OXSM	1.09E-02	0
2	CYTO	W5NUU1	VRK2	6.06E-03	0
2	CYTO	W5PMC5	LOC101110546	3.29E-03	0
2	CYTO	W5NTM7	DENND10	4.89E-02	0
2	CYTO	W5NRA9	ASL	6.51E-03	0
2	CYTO	W5PTW5	RALB	5.46E-02	0
2	CYTO	W5PHQ8	TSTD1	1.74E-02	0
2	CYTO	W5P1S3	SLC25A13	1.60E-02	0
2	CYTO	W5PVM8	UBR7	2.72E-05	0
2	CYTO	W5P813	COL4A1	1.86E-02	0
2	CYTO	W5NXB7		5.41E-02	0
2	CYTO	W5P4I3	TRAF3	7.27E-03	0
2	CYTO	W5NPP2	CPM	1.11E-02	0
2	CYTO	W5P261	PPM1H	4.48E-02	0
2	CYTO	W5PHY4	TYMS	2.30E-02	0
2	CYTO	Q9XT28	ATOX1	6.34E-03	0
2	CYTO	W5PK12	OAT	6.36E-03	0
2	CYTO	W5QJA2	CD14	3.46E-02	0
2	CYTO	W5Q3K9	IL2RG	2.35E-02	0
2	CYTO	W5PU61	SETD7	1.92E-02	0
2	CYTO	W5PA31	RAB20	1.56E-02	0
2	CYTO	W5PRS4	FKBP5	2.35E-02	0
2	CYTO	W5PFM2	AP3M1	3.24E-02	0
2	CYTO	W5NU07		1.77E-02	0
2	CYTO	W5NQ23	WIPF1	1.80E-02	0
2	CYTO	W5PKF9	FIG4	3.43E-02	0
2	CYTO	W5QCF3	SLC35F6	2.06E-03	0
2	CYTO	W5PHJ3	AHR	2.44E-02	0
2	CYTO	W5PVL6	AP2A1	1.67E-02	0
2	CYTO	W5QAF8	ATP7A	1.56E-02	0
2	CYTO	W5QC34	MAN2A1	5.31E-03	0
2	CYTO	W5PTB7	ATG7	4.72E-02	0
2	CYTO	W5NZQ6	CYP1A1	4.91E-02	0
2	CYTO	W5Q497	PGGT1B	2.94E-02	0.000203
2	CYTO	W5PQM4	MOSPD2	2.04E-02	0
2	CYTO	W5QH03	LPP	1.82E-02	0
2	CYTO	W5PUW5	GOLGA5	4.30E-02	0
2	CYTO	W5PSE3	SH3TC1	7.53E-04	0.0002207
2	CYTO	W5P7P6	MYO18A	1.18E-02	0
2	CYTO	W5QDY5	ATP6V1A	3.21E-02	0
2	CYTO	W5PBH0	SDSL	5.82E-03	0
2	CYTO	W5Q5T6	CSNK1D	3.43E-02	0.0002094
2	CYTO	W5QEQ1	TRABD	1.92E-02	0
2	CYTO	W5PMY4	GABARAP	3.85E-02	0
2	CYTO	Q6ECI6	ITGB2	1.90E-02	0
2	CYTO	W5NZD5	POLB	2.87E-03	0.0003989
2	CYTO	W5P848	MLKL	7.56E-03	0
2	CYTO	W5P4T0	VHL	7.59E-05	0.0002039

Supplementary Table 16: Differentially abundant proteins (DAPs) of M1 relative to Mo and their cellular location.

FoldChange	Location	Protein IDs		P-value	FDR
2	CYTO	W5P1T1	ANO10	1.64E-02	0
2	CYTO	W5PI67	IDS	3.56E-03	0
2	CYTO	W5NR63	S100A4	4.75E-02	0
2	CYTO	W5QA59	HGS	2.84E-03	0
2	CYTO	W5NVC9	RAC1	2.65E-02	0
2	CYTO	W5P8A0		4.02E-02	0
2	CYTO	W5PDN7	GPD2	4.50E-02	0
2	CYTO	W5P1U3	LACTB2	2.13E-02	0
2	CYTO	W5NZ67		5.12E-02	0
2	CYTO	W5QBQ8	RAB5C	2.86E-02	0
2	CYTO	W5Q5B5	CSAD	9.61E-03	0
2	CYTO	W5PVC2	CDC37L1	1.69E-02	0
2	CYTO	W5PBK8	SNX11	3.69E-02	0.0002101
2	CYTO	P00349	PGD	2.21E-02	0
2	CYTO	W5P8B4	TRAPPC8	3.55E-03	0
2	CYTO	W5P5P0	SRGAP2	3.37E-02	0
2	CYTO	W5QB00	MCM5	2.70E-02	0
2	CYTO	W5NZ42	SLC2A6	3.79E-03	0
2	CYTO	W5PPM6	PQBP1	3.95E-02	0.0002032
2	CYTO	W5QD46	CYTH4	7.77E-03	0
2	CYTO	W5P316	NAMPT	2.07E-02	0
2	CYTO	W5P6I9	GALNT1	4.29E-02	0
2	CYTO	W5PE91	LOC101105400	4.28E-02	0.0002051
2	CYTO	W5P3U4	COMMD2	3.81E-02	0
2	CYTO	W5Q6E0	CUL2	3.37E-02	0
2	CYTO	W5PTT3	CRTC3	4.40E-02	0
2	CYTO	W5Q175	GNPTAB	5.05E-03	0
2	CYTO	W5QDQ8	MMP14	3.26E-02	0
2	CYTO	W5P983	COL4A2	4.89E-03	0.0002089
2	CYTO	W5PSF8	CRYZ	3.21E-02	0
2	CYTO	W5PEN2	TPD52	3.32E-03	0
2	CYTO	C8BKC7	MYD88	1.39E-02	0
2	CYTO	W5NW41	TBC1D9B	1.01E-02	0
2	CYTO	W5PZ62	ZFYVE16	1.14E-03	0
2	CYTO	W5QH66		1.28E-02	0.0002155
2	CYTO	W5NYA8	SCAMP2	4.74E-02	0
2	CYTO	W5PED5	LOC101116121	4.44E-04	0
2	CYTO	W5PLZ0	ATP6V1B2	1.61E-02	0
2	CYTO	W5Q045	TMED3	2.15E-03	0
2	CYTO	W5PBG1		4.88E-02	0
2	CYTO	W5PVR6	TNFAIP8L2	4.04E-02	0
2	CYTO	W5Q2C9		1.61E-02	0
2	CYTO	W5PFQ8	RRAGA	6.48E-03	0
2	CYTO	W5PIN6	LDHA	2.30E-04	0
2	CYTO	W5NYE8	SEL1L	1.28E-02	0
2	CYTO	W5QCD3	RNASEL	9.11E-03	0
2	CYTO	W5QCM8	EPS15L1	4.22E-02	0
2	CYTO	W5Q3J3	COLGALT1	2.67E-03	0
2	CYTO	W5QAL6	FMNL3	3.49E-04	0.0002182
2	CYTO	W5QG24	PPT1	4.94E-02	0
2	CYTO	W5P3A2		3.41E-02	0
2	CYTO	W5NZJ1	LOC101114075	2.24E-02	0
2	CYTO	W5NPU0	NAPRT	1.92E-02	0
2	CYTO	W5Q6V7	SIPA1	2.53E-02	0
2	CYTO	W5NUX5		2.60E-03	0
2	CYTO	W5Q430	IDUA	1.11E-02	0.0002102
2	CYTO	W5P1U0	TXNL4A	1.10E-03	0
2	CYTO	W5Q1D8	GALNT7	1.30E-03	0.0002181

Supplementary Table 16: Differentially abundant proteins (DAPs) of M1 relative to Mo and their cellular location.

FoldChange	Location	Protein IDs		P-value	FDR
2	CYTO	W5P9Y1	SNX6	5.36E-02	0
2	CYTO	W5PNC8	PADI2	2.82E-02	0
2	CYTO	W5PUT6	CLTC	4.75E-02	0
2	CYTO	W5Q643	LOC101110219	3.56E-02	0.0088881
2	CYTO	W5NSQ3	HFE	4.31E-03	0
2	CYTO	W5NX28	MKNK1	4.24E-02	0
2	CYTO	W5Q971	DPP8	1.89E-05	0
2	CYTO	W5Q6K0	HEATR3	5.10E-02	0.0024645
2	CYTO	W5PPK8		8.51E-03	0
2	CYTO	W5PK41	LPIN2	1.34E-02	0.0001998
2	CYTO	W5NRD1	ERLIN2	3.55E-02	0
2	CYTO	W5NTC4	BMI1	5.37E-02	0
2	CYTO	W5P8I3	RIDA	2.90E-02	0
2	CYTO	W5QB79	GLCE	1.01E-02	0.0002206
2	CYTO	W5P7L0	SUN1	3.20E-03	0.0002168
2	CYTO	W5QHV6	SNX1	2.08E-02	0
2	CYTO	W5QIX6	EXOC5	2.32E-02	0
2	CYTO	W5NPP0		2.40E-02	0
2	CYTO	W5PDX8	FAM120A	1.72E-03	0
2	CYTO	W5PF04	MAN1A1	1.49E-02	0.0002015
2	CYTO	W5PKP3	FAM114A1	2.59E-03	0
2	CYTO	W5QGX7	EHD4	4.55E-02	0
2	CYTO	W5Q2K9	CYFIP1	9.47E-03	0
2	CYTO	W5PI45	ENOSF1	4.77E-02	0
2	CYTO	W5Q3A2		5.62E-02	0
2	CYTO	W5P700	KIF1B	2.04E-02	0
2	CYTO	W5Q867	SLC12A2	4.33E-02	0.0003978
2	CYTO	W5QFZ2	DTX3L	3.22E-02	0
2	CYTO	W5Q8I6	POLDIP2	1.92E-02	0
2	CYTO	W5QHV8	PLD1	4.53E-02	0
2	CYTO	W5PZV5	HS2ST1	1.42E-02	0
2	CYTO	W5P7I3	ARL11	1.28E-02	0
2	CYTO	W5QFY8	PARP9	8.25E-03	0
2	CYTO	W5NPP9	PRKCD	1.32E-02	0
2	CYTO	W5PWI8	EEF1AKMT1	5.56E-03	0
2	CYTO	W5Q1P6	STX2	4.21E-03	0
2	CYTO	W5P381	LOC101116334	1.78E-02	0
2	CYTO	W5Q740	ABCD3	1.72E-02	0
2	CYTO	W5Q0P4	MTMR9	8.01E-03	0
2	CYTO	W5Q4P9	CYRIA	2.80E-02	0
2	CYTO	W5NVT3	SND1	5.02E-02	0
2	CYTO	W5PK31	FCGR3A	3.40E-02	0
2	CYTO	W5NQG1	SETD3	7.69E-04	0
2	CYTO	W5Q4Q8		4.48E-04	0
2	CYTO	W5P5I4	LAMTOR2	4.54E-03	0
2	CYTO	W5Q418	NELFCD	4.64E-03	0
2	CYTO	W5Q928	PCYT2	3.37E-03	0
2	CYTO	W5PJN0	PSPH	2.63E-02	0
2	CYTO	W5Q7X8	CRYBG3	3.45E-02	0
2	CYTO	W5PPJ2	VPS33A	2.38E-02	0
2	CYTO	W5PB20		3.23E-02	0
2	CYTO	W5PC57	MRTFB	1.05E-02	0
2	CYTO	W5QJ49	ATP6V1D	4.39E-02	0
2	CYTO	W5Q9H8	SNX5	1.25E-02	0
2	CYTO	W5QIK3	USP8	4.68E-03	0
2	CYTO	W5P7S0	PIP4K2C	2.91E-02	0
2	CYTO	W5PU63	SYAP1	3.92E-02	0
2	CYTO	W5P4Y1	WDR7	1.75E-02	0

Supplementary Table 16: Differentially abundant proteins (DAPs) of M1 relative to Mo and their cellular location.

FoldChange	Location	Protein IDs		P-value	FDR
2	CYTO	W5QIA8	YARS1	3.84E-02	0
2	CYTO	W5PVU5	PSMB7	4.83E-02	0
2	CYTO	W5QBW4	VAV3	6.85E-03	0
2	CYTO	W5P3H1	LOC101111732	1.24E-02	0
2	CYTO	W5P5A7	VPS16	1.23E-03	0
2	CYTO	W5P6M5	SEC11C	1.57E-05	0.0019084
2	CYTO	W5QHP5	GNB4	3.33E-02	0
2	CYTO	W5QH58	DNAJB11	2.14E-02	0
2	CYTO	W5Q6T6	SKIC3	2.39E-02	0
2	CYTO	W5PKP0	TIGAR	1.29E-02	0.000201
2	CYTO	W5PWF2	ATP6V1H	1.27E-02	0
2	CYTO	W5P6D2	MGLL	8.22E-04	0.0002168
2	CYTO	A2SW69	ANXA2	3.12E-03	0
2	CYTO	C5IWT0	ARF4	4.86E-02	0
2	CYTO	W5PZX0	KRT24	2.72E-02	0
2	CYTO	W5P824	LOC101103096	4.80E-02	0
2	CYTO	W5QCV4	DHRS1	1.85E-02	0
2	CYTO	W5PKN3	PDLIM2	2.86E-02	0
2	CYTO	W5QDU7	RBKS	1.50E-02	0
2	CYTO	W5PZM9	ANXA5	1.36E-03	0
2	CYTO	W5NZZ3	ATP6V1F	1.81E-02	0
2	CYTO	W5PWZ2		1.51E-02	0
2	CYTO	W5PNX6	PREP	3.71E-02	0
2	CYTO	W5QAE8	ALDH7A1	2.86E-03	0
2	CYTO	W5PDE3	SPTLC1	9.36E-03	0
2	CYTO	W5PDU4	NMT2	3.01E-03	0
2	CYTO	W5NSQ8	LOC101103383	8.90E-04	0.0062443
2	CYTO	W5PK60	CLCN5	2.88E-02	0
2	CYTO	W5PCC0	LACC1	3.28E-02	0
2	CYTO	W5Q3L8	LOC101104306	4.78E-03	0
2	CYTO	W5PRP9	UBE2G2	4.53E-02	0
2	CYTO	W5PNW7	VIM	1.13E-04	0
2	CYTO	W5PGC9	MMAA	3.01E-02	0
2	CYTO	W5Q234	RAF1	5.54E-02	0
2	CYTO	W5PVR9	ERMP1	4.85E-02	0
2	CYTO	W5P1M3		7.10E-03	0
2	CYTO	W5NRL0	PLCD1	2.00E-02	0
2	CYTO	W5NUZ1	DYNC1I2	3.85E-02	0
2	CYTO	W5PF26	JAML	7.12E-03	0
2	CYTO	W5NYK8	GALC	4.50E-03	0
2	CYTO	W5PAA0	RBMS1	5.01E-02	0.0002203
2	CYTO	W5NYG8	TSSC4	1.60E-03	0
2	CYTO	W5Q1R8	MAPK1	3.64E-02	0
2	CYTO	W5QB43	CDK6	6.68E-04	0
-2	CYTO	W5PIE9	NIT1	4.75E-02	0
-2	CYTO	W5PTN4	TMPO	4.82E-02	0
-2	CYTO	W5P8R0	NDUFA4	3.42E-02	0
-2	CYTO	W5PAX3	OGFOD2	2.25E-03	0
-2	CYTO	W5NSP0		2.87E-02	0
-2	CYTO	W5PMI8	CUL5	4.61E-03	0
-2	CYTO	W5P8W5		3.62E-02	0
-2	CYTO	W5PDB0	TRIM58	5.02E-02	0
-2	CYTO	W5Q732	PDK3	5.36E-02	0.0003961
-2	CYTO	W5Q804	SPECC1	4.90E-02	0
-2	CYTO	W5Q9R1	RTCA	9.35E-03	0
-2	CYTO	W5QHP6	ARHGDIB	1.61E-02	0
-2	CYTO	W5P1E2	CNST	5.02E-02	0
-2	CYTO	W5QHL5	CAB39	1.94E-02	0

Supplementary Table 16: Differentially abundant proteins (DAPs) of M1 relative to Mo and their cellular location.

FoldChange	Location	Protein IDs		P-value	FDR
-2	CYTO	W5PMC9	ARHGAP6	5.54E-02	0.0071298
-2	CYTO	W5Q8P3	USE1	2.25E-02	0
-2	CYTO	C8BKC5	PRDX2	1.30E-02	0
-2	CYTO	W5PV48	USP11	2.17E-02	0
-2	CYTO	W5P671	DDAH2	3.69E-02	0.0095478
-2	CYTO	W5PTQ0	RNGTT	4.27E-02	0.0002055
-2	CYTO	W5PUR1	CNOT11	4.62E-02	0
-2	CYTO	W5Q9J0	RNPS1	2.41E-02	0
-2	CYTO	W5QBX9	ILKAP	8.07E-03	0
-2	CYTO	W5PJJ5	PGM2L1	3.47E-02	0.0002057
-2	CYTO	W5PYM3	CAAP1	1.02E-02	0.0011547
-2	CYTO	W5Q4D5	BCO2	4.06E-03	0
-2	CYTO	W5NUX2		1.95E-02	0
-2	CYTO	O78750	MT-CO2	5.00E-02	0
-2	CYTO	W5PHL8	NUDT16	2.79E-02	0
-2	CYTO	W5NSI2	RALBP1	2.50E-02	0.0085486
-2	CYTO	W5Q2W6	PPFIBP2	1.87E-02	0.0002217
-2	CYTO	W5PAV5	RFX1	3.65E-02	0
-2	CYTO	W5NQ72	PFAS	8.24E-04	0
-2	CYTO	W5PEA0	FGD3	6.30E-03	0
-2	CYTO	W5NTA4	CGGBP1	1.58E-02	0
-2	CYTO	W5NUT8	PIP4K2A	1.48E-03	0
-2	CYTO	W5PN94	LUC7L	2.59E-02	0
-2	CYTO	W5NW56	IRF2BPL	5.56E-02	0
-2	CYTO	W5NUS2	MED9	5.38E-02	0.000221
-2	CYTO	W5QBV1	FER	1.45E-02	0
-2	CYTO	W5QIY4	ARID4A	3.31E-02	0
-2	CYTO	W5QH43	EIF4A2	2.43E-02	0
-2	CYTO	W5QGL7	RTF1	3.90E-02	0.0044601
-2	CYTO	W5NUE6	PLEKHA2	2.43E-02	0
-2	CYTO	P80190		2.78E-03	0
-2	CYTO	W5NRR9	STAT5A	4.36E-02	0
-2	CYTO	W5QHL6	ITM2C	3.45E-02	0
-2	CYTO	W5Q0P0	GABPA	2.98E-02	0
-2	CYTO	W5P2J9		4.27E-03	0
-2	CYTO	W5PP64	FHL1	2.14E-02	0
-2	CYTO	W5P684	ACTBL2	3.39E-02	0.0042926
-2	CYTO	W5PTB3	INPP5K	5.42E-02	0.0002019
-2	CYTO	C5IS96	LCAT	2.12E-02	0.0002064
-3	CYTO	W5PUI4	RAB3A	3.66E-02	0.0002152
-3	CYTO	W5P173	BMS1	3.39E-02	0
-3	CYTO	W5P795	LRBA	5.07E-02	0
-3	CYTO	W5P0R4	CTIF	6.26E-03	0.0003939
-3	CYTO	W5Q059	SASH3	5.30E-02	0
-3	CYTO	W5PP85	TBC1D10C	5.19E-02	0
-3	CYTO	W5PVX3	PRKG1	3.38E-02	0
-3	CYTO	W5PAV0	STAP1	2.68E-02	0
-3	CYTO	W5NTV6		1.51E-02	0
-3	CYTO	W5PGX7	SSRP1	4.97E-03	0
-3	CYTO	W5Q9H1	ZYX	5.20E-02	0
-3	CYTO	W5PZP8	FIS1	1.22E-03	0
-3	CYTO	W5P9M9	LOC101103862	4.54E-02	0
-3	CYTO	W5NT67	TUBGCP2	4.97E-03	0.0002061
-3	CYTO	W5P8H4	DCTPP1	3.24E-02	0
-3	CYTO	W5P6T0		3.57E-02	0.0011554
-3	CYTO	W5Q7L1	CNN3	1.76E-02	0
-3	CYTO	W5PKA9	F5	9.85E-04	0
-3	CYTO	W5Q6U5	ARMH3	4.33E-02	0

Supplementary Table 16: Differentially abundant proteins (DAPs) of M1 relative to Mo and their cellular location.

FoldChange	Location	Protein IDs		P-value	FDR
-3	CYTO	W5QCH5	WDR47	1.81E-02	0.0002041
-3	CYTO	W5PFJ0	VCL	3.19E-02	0
-3	CYTO	W5PEC3	ANXA6	4.09E-02	0
-3	CYTO	W5PGU9	PLCG2	6.04E-03	0
-3	CYTO	W5QEL7	NDRG2	3.71E-02	0.0078396
-3	CYTO	Q09YJ2	TES	8.52E-03	0
-3	CYTO	W5PAF7	RSL1D1	2.83E-02	0
-3	CYTO	W5NV79	NUP210	3.28E-02	0
-3	CYTO	W5Q3I7	TUBB1	4.42E-02	0
-3	CYTO	P14639	ALB	1.96E-02	0
-3	CYTO	W5PE21	PELP1	1.05E-02	0
-3	CYTO	W5Q5E7	PYCR3	2.30E-02	0
-4	CYTO	W5P433	BRCA1	6.49E-04	0.0037418
-4	CYTO	W5PTS4	LOC101114275	9.33E-04	0
-4	CYTO	W5NQA0	GCC2	4.64E-02	0
-4	CYTO	W5P815	SEPTIN1	4.54E-02	0
-4	CYTO	W5PG02	RASA3	7.90E-03	0
-4	CYTO	W5NRI1		7.09E-03	0.000214
-4	CYTO	W5PX46	LOC101122591	2.35E-02	0
-4	CYTO	W5PP80	FRY	3.43E-02	0.0011579
-4	CYTO	W5P5Y4	RIPOR2	1.74E-02	0
-4	CYTO	W5NTF8	SPNS1	9.09E-03	0
-4	CYTO	W5PZG0	ADD1	9.12E-03	0
-4	CYTO	W5PV80	BANK1	2.60E-02	0
-4	CYTO	W5PD82	CALD1	3.28E-02	0
-4	CYTO	W5PYK8	VNN2	2.34E-04	0
-4	CYTO	W5PLZ3	PACSIN1	4.26E-02	0
-4	CYTO	W5NXW9		4.77E-02	0
-4	CYTO	W5PFI6	RASGRP2	4.99E-02	0
-4	CYTO	W5PHI5	PALM	1.64E-03	0
-4	CYTO	W5PTQ7	TRAF3IP3	1.43E-02	0
-4	CYTO	W5PRY2	ACAP1	2.80E-02	0
-5	CYTO	W5Q9S4	ING3	2.58E-02	0.0011576
-5	CYTO	W5PJ75	SPTAN1	1.95E-03	0
-5	CYTO	W5PRW4	TRIAP1	1.90E-02	0.0002042
-5	CYTO	W5Q2S8	MYL9	4.45E-03	0
-5	CYTO	W5NU34	TREML1	3.45E-02	0
-5	CYTO	W5Q0K9	ABLIM1	2.66E-02	0
-6	CYTO	W5QGQ3	LOC443320	1.23E-02	0.0002067
-6	CYTO	W5P4L3	AVIL	4.41E-02	0
-6	CYTO	W5NT35	LOC443162	2.18E-02	0
-6	CYTO	W5NZX9	SPTBN1	1.55E-03	0
-6	CYTO	W5PF87	ALOX15	2.46E-02	0
-6	CYTO	W5PYD8	PARP4	5.82E-06	0.0092124
-6	CYTO	W5QD99	USP40	5.41E-02	0
-7	CYTO	W5QIK8	SELENBP1	1.01E-02	0
-7	CYTO	W5P733	PDLIM1	3.29E-03	0
9	SEC	W5PYW3		0.000241828	0
8	SEC	W5PDH4		1.30122E-05	0
8	SEC	W5PJR5		0.000147644	0
8	SEC	W5NZA2		0.027985265	0.0080591
8	SEC	W5P3Q3		0.000403342	0
7	SEC	P83205		0.001242925	0
7	SEC	W5P530		1.16023E-05	0
7	SEC	W5PHJ9		0.000618121	0
6	SEC	W5NWF5		0.000794163	0
6	SEC	W5PAC2		0.005095405	0
6	SEC	W5P1H0		0.009103706	0

Supplementary Table 16: Differentially abundant proteins (DAPs) of M1 relative to Mo and their cellular location.

FoldChange	Location	Protein IDs		P-value	FDR
6	SEC	W5QHV3		0.01009832	0
6	SEC	P36925		0.001266035	0.0023566
6	SEC	W5QHL1		0.000126863	0
5	SEC	W5Q754		0.025052063	0.0023529
5	SEC	W5Q233		0.000393588	0
5	SEC	W5NY01		0.007332132	0
5	SEC	W5QB02		0.020445692	0.0023419
5	SEC	W5PEB0		0.001587735	0
4	SEC	W5P2M5		0.002159776	0
4	SEC	W5QBV7		0.008735933	0.0045249
4	SEC	W5Q3X9		0.013034497	0
4	SEC	W5P4V3		0.034837807	0
4	SEC	W5PUH5		0.000100447	0
4	SEC	W5PS94		0.004263573	0
4	SEC	W5PMM5		0.009869208	0
4	SEC	W5PGF4		0.027290988	0
4	SEC	W5PIQ4		0.035503597	0
4	SEC	W5P5K5		0.007691341	0
3	SEC	W5Q1B2		0.035008117	0.0023456
3	SEC	W5Q0F3		0.031375922	0
3	SEC	W5P4C9		0.046293477	0
3	SEC	W5PAL2		0.042051973	0
3	SEC	W5QIR6		0.008991856	0
3	SEC	W5NSH8		0.040228641	0
3	SEC	W5NTD9		0.001770022	0
3	SEC	W5QG01		0.054204275	0
3	SEC	W5PRI6		0.015103721	0
3	SEC	W5P8R4		0.0091572	0
2	SEC	W5PAB5		0.039518032	0.001586
2	SEC	W5PF04		0.017447953	0
2	SEC	W5PDR7		0.024815282	0.0016077
2	SEC	W5PGV0		0.025856627	0
2	SEC	W5QJA2		0.010592242	0
-2	SEC	W5Q1W2		0.045716469	0
-2	SEC	W5P6H2		0.047392996	0.004415
-2	SEC	W5PV50		0.052166384	0.004461
-2	SEC	W5P363		0.043550999	0
-2	SEC	W5PVG1		0.029644571	0
-2	SEC	W5QHL5		0.034276176	0
-2	SEC	W5PVX8		0.026005474	0
-2	SEC	W5NYV5		0.00035139	0.0016038
-2	SEC	W5PYQ9		0.033877306	0
-2	SEC	W5PI22		0.030493743	0
-2	SEC	W5NXX1		0.022279034	0
-3	SEC	W5Q2I7		0.049488089	0
-3	SEC	W5NRL8		0.018039762	0
-3	SEC	W5P8Q0		0.044958451	0
-3	SEC	B7TJ15		0.049361733	0
-3	SEC	W5Q9X5		0.048228902	0
-3	SEC	W5Q0B6		0.029299238	0.0023511
-3	SEC	W5P6M2		0.04335681	0
-3	SEC	W5PS88		0.02876994	0
-3	SEC	W5Q1R8		0.014145781	0.0044412
-3	SEC	W5QEU6		0.039550942	0
-3	SEC	W5P7L2		0.053764547	0
-3	SEC	W5P4J1		0.017783038	0
-3	SEC	W5NX31		0.027450288	0
-3	SEC	W5PF71		0.024531534	0

Supplementary Table 16: Differentially abundant proteins (DAPs) of M1 relative to Mo and their cellular location.

FoldChange	Location	Protein IDs		P-value	FDR
-3	SEC	W5PL67		0.039556819	0
-3	SEC	W5PZ86		0.015889404	0
-3	SEC	W5PMH1		0.019709161	0
-3	SEC	P09670		0.016324793	0.0055905
-3	SEC	W5QFP1		0.055422305	0
-3	SEC	W5PC25		0.043228079	0
-3	SEC	W5Q3N5		0.032537757	0
-3	SEC	W5Q9M6		0.045292743	0
-3	SEC	P80190		0.038611462	0
-3	SEC	W5P538		0.008868299	0
-3	SEC	W5Q5R8		0.03813689	0
-3	SEC	W5P880		0.000970446	0
-3	SEC	W5P359		0.042699452	0
-3	SEC	W5NZ57		0.049361632	0
-3	SEC	W5NPQ6		0.042111725	0
-3	SEC	W5Q701		0.035680115	0
-3	SEC	W5P7J7		0.048140614	0
-3	SEC	W5PJB6		0.006823941	0
-3	SEC	W5P694		0.047836882	0
-3	SEC	W5PIM8		0.027716826	0
-3	SEC	W5P889		0.054261695	0
-3	SEC	Q1ZZU7		0.039103829	0
-4	SEC	W5Q3E3		0.019353434	0
-4	SEC	W5PFV7		0.036966422	0
-4	SEC	W5QBH1		0.012284691	0
-4	SEC	W5PMQ9		0.055229419	0
-4	SEC	W5PNV0		0.022085697	0
-4	SEC	W5Q3A5		0.014024248	0
-4	SEC	W5P9U4		0.054850009	0
-4	SEC	W5PQK3		0.04828543	0
-4	SEC	W5P707		0.026380369	0
-4	SEC	W5P583		0.050635084	0
-4	SEC	W5QD49		0.023404143	0
-4	SEC	W5Q3M9		0.050777701	0
-4	SEC	W5PQI3		0.046308029	0
-4	SEC	W5P4X6		0.050788017	0
-4	SEC	W5PE11		0.042181014	0
-4	SEC	W5PPG3		0.02784853	0
-4	SEC	W5PWG1		0.04984226	0
-4	SEC	W5QFH5		0.003342366	0
-4	SEC	W5NQK6		0.017235241	0
-4	SEC	W5PLS7		0.017829769	0
-4	SEC	W5P6Z6		0.034634738	0
-4	SEC	W5QFN2		0.033909594	0
-4	SEC	W5PIJ6		0.045560855	0
-4	SEC	W5Q731		0.023254682	0
-4	SEC	W5Q0L1		0.043484379	0
-4	SEC	W5NRD9		0.055440436	0.0015898
-4	SEC	W5QD96		0.008083652	0
-4	SEC	W5PVT6		0.052704935	0
-4	SEC	W5NY22		0.013883423	0
-4	SEC	W5NUV1		0.032196239	0
-4	SEC	W5PSM0		0.028211191	0
-4	SEC	W5PK13		0.040076809	0
-4	SEC	W5PMC5		0.054889384	0
-4	SEC	W5PY17		0.022594684	0
-4	SEC	C5IWU0		0.017895559	0
-4	SEC	W5P733		0.024548529	0

Supplementary Table 16: Differentially abundant proteins (DAPs) of M1 relative to Mo and their cellular location.

FoldChange	Location	Protein IDs		P-value	FDR
-4	SEC	W5P4C7		0.034940513	0
-5	SEC	W5PYM5		0.02759104	0
-5	SEC	W5PK85		0.046015246	0
-5	SEC	W5P3N0		0.041471138	0
-5	SEC	Q5MIB5		0.054998151	0
-5	SEC	W5PK95		0.043739695	0
-5	SEC	W5PK66		0.036574128	0
-5	SEC	W5PX84		0.043496543	0.0045662
-5	SEC	Q28554		0.035597607	0
-5	SEC	W5PL19		0.045653805	0
-5	SEC	W5QHQ7		0.01758253	0
-5	SEC	W5PEX2		0.046568315	0
-5	SEC	C5ISA2		0.022555626	0
-5	SEC	W5P3E8		0.042917701	0
-5	SEC	W5PPT6		0.016362845	0
-5	SEC	W5QE14		0.035980455	0
-5	SEC	W5NUT8		0.008768438	0
-5	SEC	W5P765		0.043481971	0
-5	SEC	W5Q2S8		0.041715531	0
-5	SEC	W5PRR5		0.008500307	0
-5	SEC	W5PH15		0.038265232	0
-6	SEC	W5PLD5		0.013887706	0
-6	SEC	W5QAR2		0.045572801	0
-6	SEC	W5QG16		0.04275264	0
-6	SEC	W5PD82		0.045213571	0
-6	SEC	W5PQA8		0.034714864	0
-6	SEC	W5NUI0		0.00560145	0
-6	SEC	W5PE27		0.021896574	0
-6	SEC	W5PFI7		0.037231532	0
-6	SEC	C5IJA0		0.016815047	0
-6	SEC	W5PHW0		0.040993863	0
-6	SEC	W5PK38		0.034074621	0
-6	SEC	W5QIK8		0.05008756	0
-6	SEC	W5PG95		0.022950833	0
-6	SEC	W5Q9H1		0.040425367	0
-6	SEC	W5PFJ0		0.015523535	0
-6	SEC	W5P375		0.000604349	0
-7	SEC	W5QBD7		0.011830852	0
-7	SEC	W5Q3I7		0.027852402	0
-7	SEC	W5P409		0.009831973	0
-8	SEC	W5PQK6		0.020680052	0
-9	SEC	W5QF37		0.032435455	0.0080321

M1, pro-inflammatory macrophages activated with GM-CSF/ LPS/ INF-γ and M0, Monocytes at 3 hours. Differentially abundant proteins (DAPs) were identified using a threshold of false discovery rate (FDR, q-value) ≤ 0.05 and absolute fold change ≥ 2. Red-highlighted cells indicate shared differentially abundant proteins (DAPs) between the cell lysate (CYTO) and secretome (SEC) compartments in M1 relative to M0, whereas non-highlighted cells represent compartment-specific DAPs unique to either the whole cell lysate or secretome.

Supplementary Table 17: Differentially abundant proteins (DAPs) of M2 relative to MMØ and their cellular location.

FoldChange	Location	Protein IDs	Gene Names	P-value	FDR
7	CYTO	W5PFR8	FBP1	1.72E-05	0.000
7	CYTO	W5Q9Y6	TCAF2	9.30E-05	0.000
6	CYTO	W5PZ28	TEX2	1.50E-02	0.002
6	CYTO	W5PYD8	PARP4	5.07E-04	0.009
5	CYTO	W5PES6		1.48E-02	0.000
4	CYTO	W5QI35	IL1RN	2.45E-04	0.000
4	CYTO	W5P0K8	CLIC2	4.24E-02	0.000
4	CYTO	W5PU77	PIP5K1B	9.85E-03	0.004
3	CYTO	O78750	MT-CO2 COII COX2	1.21E-04	0.000
3	CYTO	W5Q6D6	AKAP9	2.36E-05	0.000
3	CYTO	W5PT68	FLNB	1.18E-03	0.000
3	CYTO	W5PZ22	LTN1	1.87E-02	0.000
3	CYTO	W5QGS4	PHGDH	1.18E-02	0.000
3	CYTO	W5NU34	TREML1	2.08E-04	0.000
3	CYTO	W5PVG5	BLTP3B	4.11E-05	0.000
3	CYTO	W5NY85	MSRB3	1.95E-02	0.000
3	CYTO	W5PHG6	SMYD2	1.99E-04	0.000
3	CYTO	W5Q980	ACSL4	3.29E-02	0.000
3	CYTO	W5P060	SYNE1	1.27E-02	0.000
3	CYTO	W5QHZ7	RPS27L	3.55E-02	0.000
3	CYTO	W5PVC3	LOC101117851	1.34E-03	0.000
3	CYTO	W5PCD9	EML4	1.21E-04	0.000
3	CYTO	W5QIR6	GATM	1.51E-02	0.000
3	CYTO	Q29422		1.93E-02	0.000
3	CYTO	W5QDJ9	FGD4	2.09E-02	0.000
3	CYTO	W5PQP3	LIMK1	1.66E-02	0.000
3	CYTO	W5NQ23	WIPF1	1.19E-02	0.000
3	CYTO	W5NVA8	GP6	4.53E-02	0.000
3	CYTO	W5NWF5	RARRES1	3.38E-04	0.000
2	CYTO	W5P094	NID1	3.74E-03	0.000
2	CYTO	W5PBM2	LOC101110634	1.59E-03	0.000
2	CYTO	W5QA98	DNMT3A	4.62E-02	0.000
2	CYTO	W5P173	BMS1	1.53E-04	0.000
2	CYTO	W5NZ32	CANT1	3.99E-02	0.000
2	CYTO	W5PKA1	LOC101116157	1.35E-02	0.000
2	CYTO	W5PM87	MAP2K2	2.73E-02	0.000
2	CYTO	W5PDE5	LOC101120001	5.32E-02	0.000
2	CYTO	W5P0S9	GDPD3	5.25E-04	0.000
2	CYTO	W5Q4G2		1.03E-02	0.000
2	CYTO	W5Q8J0	PIN4	3.79E-02	0.000
2	CYTO	W5QHC0	ST3GAL5	4.11E-04	0.000
2	CYTO	W5Q7Q6	EPHX1	8.89E-03	0.000
2	CYTO	W5PEC0	PKIB	5.32E-02	0.000
2	CYTO	W5Q1Z2		5.46E-02	0.001
2	CYTO	W5P6V7	DDX39A	5.24E-03	0.000
2	CYTO	C8BKD3	ASF1A	4.19E-03	0.000
2	CYTO	W5PI99	HDGFL2	3.79E-03	0.000
2	CYTO	W5PHI1	MRPL3	3.54E-02	0.000
2	CYTO	W5PV01	REPS2	4.93E-02	0.000
2	CYTO	W5NWF2	STXBP5	2.02E-02	0.000
2	CYTO	W5Q000	RPL32	5.18E-02	0.000
2	CYTO	W5PXH4	PTPRE	4.70E-05	0.000
2	CYTO	W5PJY4	RAVER2	3.25E-02	0.000
2	CYTO	W5P9E3		8.59E-03	0.000
2	CYTO	W5PCZ7	TAP1	2.53E-02	0.000
2	CYTO	W5PCT8	ASAP1	1.31E-02	0.000
2	CYTO	W5PRV3	TLE1	9.54E-03	0.000
2	CYTO	W5PNG4	BRCA2	4.73E-02	0.005
2	CYTO	W5NPR6	AATF	1.54E-03	0.000
2	CYTO	W5PBN8	LOC101111409	2.45E-02	0.000
2	CYTO	W5NWL0	CCDC88A	1.32E-02	0.000
2	CYTO	W5PUR1	CNOT11	5.77E-04	0.000
2	CYTO	W5PFQ5	ANKMY2	5.45E-02	0.000

Supplementary Table 17: Differentially abundant proteins (DAPs) of M2 relative to MMØ and their cellular location.

FoldChange	Location	Protein IDs	Gene Names	P-value	FDR
2	CYTO	W5QEL9	ARHGAP31	8.26E-03	0.000
2	CYTO	W5P9W4	INTS4	1.28E-02	0.000
2	CYTO	W5QDG9	CCDC80	5.85E-04	0.000
2	CYTO	W5QB99	TSC22D3	4.11E-04	0.000
2	CYTO	W5P9W1	G6PC3	1.10E-02	0.000
2	CYTO	W5PWK3	TBRG4	6.03E-04	0.001
2	CYTO	W5PJH6		2.97E-03	0.000
2	CYTO	W5PG95	HSPA1A	8.36E-03	0.000
2	CYTO	W5QEG2	MAP7D1	3.87E-03	0.000
2	CYTO	W5PIN6	LDHA	1.99E-04	0.000
2	CYTO	W5P895	CRABP2	1.90E-02	0.000
2	CYTO	W5PGD6	BZW2	5.08E-05	0.000
2	CYTO	W5NS73		4.57E-03	0.003
2	CYTO	W5PQ28	SMYD5	3.57E-02	0.000
2	CYTO	W5PF73	PLAU	5.18E-04	0.000
2	CYTO	W5Q813	NOP58	3.03E-02	0.000
2	CYTO	W5PTM6	CPSF2	1.41E-03	0.000
2	CYTO	W5NQC1	PSEN1	1.94E-02	0.000
2	CYTO	W5NSI2	RALBP1	4.92E-02	0.009
2	CYTO	Q09YJ2	TES	4.80E-03	0.000
2	CYTO	W5QGG8	MAN1A2	2.77E-03	0.000
2	CYTO	W5QDZ8	AAMP	9.89E-05	0.000
2	CYTO	W5P7R2	HECTD1	6.91E-03	0.000
2	CYTO	W5PUM5	KANK1	1.43E-02	0.000
2	CYTO	W5Q6F0	LOC101106791	6.47E-03	0.000
2	CYTO	W5Q527	PAPOLA	4.95E-03	0.000
2	CYTO	W5PMG3	MEMO1	7.83E-04	0.000
-2	CYTO	W5QIU5	GMFB	1.01E-03	0.000
-2	CYTO	W5P916	NOP56	2.69E-02	0.000
-2	CYTO	W5QJ37		1.13E-02	0.000
-2	CYTO	W5PEW2	USP48	2.34E-02	0.000
-2	CYTO	W5P6U2		2.54E-02	0.000
-2	CYTO	W5NUE6	PLEKHA2	4.82E-02	0.000
-2	CYTO	W5NT90	BAG4	3.99E-02	0.000
-2	CYTO	W5Q4U5	CPT1A	1.95E-02	0.000
-2	CYTO	W5Q686	TPP1	4.31E-03	0.000
-2	CYTO	W5QDI5	MREG	4.63E-02	0.000
-2	CYTO	W5P5W7	LOC101110178	5.58E-02	0.001
-2	CYTO	W5Q4P9	CYRIA	2.64E-03	0.000
-2	CYTO	W5P880	PRG4	3.84E-02	0.000
-2	CYTO	W5Q231	FEN1	4.86E-02	0.000
-2	CYTO	W5P506	MSH2	3.85E-02	0.000
-2	CYTO	W5P6L1	RASGRP4	4.14E-02	0.000
-2	CYTO	W5PK79	VWA5A	9.74E-03	0.000
-2	CYTO	W5P848	MLKL	5.27E-02	0.000
-2	CYTO	W5Q234	RAF1	9.70E-03	0.000
-2	CYTO	W5PA83	PRR14	6.30E-04	0.004
-2	CYTO	W5PTS4	LOC101114275	1.07E-03	0.000
-2	CYTO	W5PDD5	STAG1	1.49E-02	0.000
-2	CYTO	W5PZI8		2.28E-02	0.000
-2	CYTO	W5Q0F3	TGFBI	4.11E-02	0.000
-2	CYTO	W5PMW3	POLR2E	4.23E-02	0.000
-2	CYTO	W5P8R4	CSF1R	5.92E-03	0.000
-2	CYTO	W5PNC8	PADI2	3.67E-02	0.000
-2	CYTO	W5P5I4	LAMTOR2	3.73E-02	0.000
-2	CYTO	W5QC43	ANTXR2	1.22E-02	0.000
-2	CYTO	W5P3H8	IGF2R	1.53E-03	0.000
-2	CYTO	W5NZ40	SGF29	5.01E-02	0.000
-2	CYTO	W5NSJ5	OXSR1	3.44E-02	0.000
-2	CYTO	W5PY22		3.84E-02	0.000
-2	CYTO	W5Q5N6	BST-2B	2.63E-02	0.000
-2	CYTO	W5PJS4	EMILIN2	2.66E-02	0.000
-2	CYTO	W5PRB3		3.48E-03	0.000

Supplementary Table 17: Differentially abundant proteins (DAPs) of M2 relative to MMØ and their cellular location.

FoldChange	Location	Protein IDs	Gene Names	P-value	FDR
-2	CYTO	W5PBS5	ABCA1	1.03E-03	0.000
-3	CYTO	W5P2F2		1.55E-02	0.000
-3	CYTO	W5PCX5	CC2D1A	5.61E-03	0.000
-3	CYTO	W5Q7N9	MTX2	1.75E-03	0.000
-3	CYTO	C5IS96	LCAT	1.37E-02	0.000
-3	CYTO	P29701	AHSG FETUA	4.13E-03	0.000
-3	CYTO	W5PFY8	MTSS1	4.89E-03	0.000
-3	CYTO	W5NUK5	HAT1	5.58E-04	0.000
-3	CYTO	W5PYX0		5.18E-02	0.000
-3	CYTO	W5QH68	LRRC57	1.52E-02	0.000
-3	CYTO	W5NWB0		1.85E-03	0.000
-3	CYTO	W5NT23	LOC101119864	3.82E-02	0.007
-3	CYTO	P14639	ALB	7.95E-03	0.000
-3	CYTO	W5NTE2	PSMG4	2.89E-06	0.000
-4	CYTO	W5PNC4	JPT1	1.80E-02	0.000
-4	CYTO	W5P2U3	IVNS1ABP	1.08E-02	0.000
-4	CYTO	W5PVR2	LOC101108019	4.63E-02	0.008
-4	CYTO	W5PGG6	MTMR1	2.58E-02	0.000
-4	CYTO	W5P9M8	UCK1	4.17E-02	0.000
7	SEC	W5P4C9	MMP12	1.41E-07	0.000000
7	SEC	W5NTX3		1.92E-07	0.000000
6	SEC	W5NZX1		6.28E-07	0.000000
6	SEC	W5QDI7	CSF1	2.71E-06	0.000000
5	SEC	W5PDE5	LOC101120001	3.62E-06	0.000000
5	SEC	W5NPK5	LOC443475	3.66E-06	0.000000
5	SEC	W5NRI1		8.07E-06	0.000000
5	SEC	W5PSA3	TCN1	1.22E-05	0.000000
5	SEC	W5QI35	IL1RN	1.42E-05	0.000000
5	SEC	W5PGT0	MYH11	2.77E-05	0.001591
5	SEC	W5Q989	PLOD1	3.15E-05	0.000000
4	SEC	W5P041	ADAM15	7.49E-05	0.000000
4	SEC	W5QDG8	FN1	8.91E-05	0.000000
4	SEC	W5PF04	MAN1A1	1.21E-04	0.000000
4	SEC	W5PTL2	CFP	3.67E-02	0.000000
4	SEC	W5P3Q3	LOC100101238	1.51E-04	0.000000
4	SEC	W5P0W4	SEMA7A	1.82E-04	0.000000
4	SEC	W5PRI6	MRC1	2.78E-04	0.000000
3	SEC	W5PDQ9		3.93E-04	0.000000
3	SEC	W5Q7S8	NRP1	4.52E-04	0.000000
3	SEC	W5PH85	ITGAX	5.98E-04	0.000000
3	SEC	W5QDG9	CCDC80	6.15E-04	0.000000
3	SEC	W5PMR2	MMP19	6.00E-02	0.000000
3	SEC	P50122	TIMP1	6.95E-04	0.000000
3	SEC	W5PNP1	MFGE8	8.79E-04	0.000000
3	SEC	W5NWF5	RARRES1	9.60E-04	0.000000
3	SEC	W5QD11	SORT1	1.15E-03	0.000000
3	SEC	W5PDS4	C1QA	1.41E-03	0.000000
3	SEC	W5PGV0	ITGAM	1.42E-03	0.000000
3	SEC	W5QBE4	FGL2	1.72E-03	0.000000
3	SEC	W5PG72	GOLM1	2.12E-03	0.000000
3	SEC	W5QG77	CD58	2.46E-03	0.000000
3	SEC	W5PFY5	ASAH1	2.66E-03	0.000000
3	SEC	W5PDH4	MMP9	2.91E-03	0.000000
2	SEC	W5QIR6	GATM	3.23E-03	0.000000
2	SEC	W5P5K9	SRGN	3.50E-03	0.000000
2	SEC	Q29524	LPL	4.64E-03	0.000000
2	SEC	W5PF73	PLAU	5.34E-03	0.000000
2	SEC	W5PXR1	ENPP1	5.83E-03	0.000000
2	SEC	W5PTA8	FURIN	6.59E-03	0.004542
2	SEC	W5PGG5	CD84	6.84E-03	0.000808
2	SEC	W5PUH5	LGMN	6.91E-03	0.000000
2	SEC	W5PGF4	PLAUR	7.73E-03	0.000000
2	SEC	W5NPP2	CPM	9.85E-03	0.000000

Supplementary Table 17: Differentially abundant proteins (DAPs) of M2 relative to MMØ and their cellular location.

FoldChange	Location	Protein IDs	Gene Names	P-value	FDR
2	SEC	W5QGG0	TFRC	1.01E-02	0.000000
2	SEC	W5PRU5	MANBA	1.30E-02	0.000000
2	SEC	W5QBV7	CD44	1.31E-02	0.004525
2	SEC	W5PZB0	APLP2	1.38E-02	0.000000
2	SEC	Q6ECI6	ITGB2 CD18	1.40E-02	0.000000
2	SEC	W5NWJ9		1.43E-02	0.000000
2	SEC	W5QF71	PLEK	1.57E-02	0.000000
2	SEC	W5PJS4	EMILIN2	1.77E-02	0.000000
2	SEC	W5QC34	MAN2A1	1.87E-02	0.000000
2	SEC	W5PCC7	LOC101115509	2.00E-02	0.000000
2	SEC	W5P6H2		2.04E-02	0.004415
2	SEC	Q6QAT4	B2M	2.68E-02	0.000000
2	SEC	W5NW80	GAA	2.72E-02	0.001603
2	SEC	W5Q1M0	GLB1	3.02E-02	0.000000
-2	SEC	W5NUU3	TPM3	1.42E+02	0.000000
-2	SEC	W5Q9H1	ZYX	2.03E+02	0.000000
-4	SEC	W5PKR1		1.25E+04	0.004438
-5	SEC	W5Q8Y5	HDLBP	5.26E+04	0.000000

M2, anti-inflammatory macrophages activated with M-CSF/ IL-4 and MMØ, monocyte-derived macrophages differentiated with M-CSF (Macrophage Colony-Stimulating Factor). Differentially abundant proteins (DAPs) were identified using a threshold of false discovery rate (FDR, q-value) ≤ 0.05 and absolute fold change ≥ 2. Red-highlighted cells indicate shared differentially abundant proteins (DAPs) between the cell lysate (CYTO) and secretome (SEC) compartments in M2 relative to MMØ, whereas non-highlighted cells represent compartment-specific DAPs unique to either the whole cell lysate or secretome.

Supplementary Table 18: Differentially abundant proteins (DAPs) of M2 relative to Mo and their cellular location.

FoldChange	Location	Protein IDs		P-value	FDR
10	CYTO	W5PCH3	SCIN	4.63E-05	0.000
10	CYTO	W5PE67		3.02E-05	0.000
9	CYTO	W5NU86	GLA	6.23E-04	0.000
8	CYTO	W5PTU7	CA2	5.63E-06	0.000
8	CYTO	W5PAM4	CTSA	4.54E-05	0.000
8	CYTO	W5PXR1	ENPP1	1.12E-04	0.000
8	CYTO	W5PT76	GPNMB	1.25E-04	0.000
8	CYTO	W5PD43	HTRA1	5.73E-03	0.000
8	CYTO	W5PF33	GM2A	7.80E-05	0.000
8	CYTO	P35623	SHMT1	2.45E-05	0.000
7	CYTO	W5Q6N3	LOC101115115	6.51E-05	0.000
7	CYTO	W5NY01		9.30E-04	0.000
7	CYTO	W5PQR0	NIBAN2	4.04E-06	0.000
7	CYTO	A9YUY8	FABP4	3.61E-04	0.000
7	CYTO	W5PFY5	ASAH1	1.30E-03	0.000
7	CYTO	W5QHG1	EPS8	6.30E-03	0.000
7	CYTO	W5P9J8	BLVRB	1.42E-04	0.000
7	CYTO	W5PIQ6	MSR1	1.26E-04	0.000
7	CYTO	W5QCD6	IDH1	1.49E-05	0.000
7	CYTO	W5PEB0	FABP7	1.76E-03	0.000
7	CYTO	W5Q9Y6	TCAF2	1.32E-05	0.000
7	CYTO	P51977	ALDH1A1 ALDH1	2.45E-03	0.000
6	CYTO	W5Q940	SHTN1	2.07E-03	0.000
6	CYTO	W5Q612	TRPV2	2.37E-05	0.000
6	CYTO	W5PRI6	MRC1	2.42E-04	0.000
6	CYTO	W5PXX3	F13B	2.11E-09	0.007
6	CYTO	W5QIW1	LGALS3	2.67E-03	0.000
6	CYTO	W5PCE0	PLBD2	4.25E-03	0.000
6	CYTO	W5NRS0		1.25E-04	0.000
6	CYTO	W5Q2Y1	PLXNC1	5.79E-03	0.000
6	CYTO	W5PAQ4	FUCA1	2.74E-04	0.000
6	CYTO	W5QC89	HEXA	9.67E-05	0.000
6	CYTO	W5PGC5	GALM	8.92E-04	0.000
6	CYTO	W5Q0Y4	ZFAND6	4.36E-03	0.000
6	CYTO	W5NVW7	NAGLU	2.85E-03	0.000
6	CYTO	W5NPM4	TEX15	5.17E-04	0.003
6	CYTO	W5QCL8	NPL	4.14E-04	0.000
6	CYTO	W5NTZ3	RENBP	3.12E-04	0.000
6	CYTO	W5PBM9	SCPEP1	3.07E-04	0.000
6	CYTO	W5P8H9	SGPL1	5.20E-04	0.000
6	CYTO	P05028	ATP1B1	1.93E-03	0.000
6	CYTO	W5PFR8	FBP1	1.29E-04	0.000
6	CYTO	W5NX56	SPP1	2.95E-04	0.000
6	CYTO	W5QHC0	ST3GAL5	3.04E-04	0.000
6	CYTO	W5PG10	PAPSS1	5.74E-03	0.000
6	CYTO	W5NUI6	SGSH	6.14E-05	0.000
6	CYTO	Q9MZS8	CTSD	1.40E-03	0.000
6	CYTO	W5QBZ7	NAGA	2.26E-03	0.000
6	CYTO	W5QI00	LACTB	4.37E-04	0.000
6	CYTO	W5NZK6	PLA2G15	2.32E-04	0.000
6	CYTO	W5PY08		4.29E-03	0.000
6	CYTO	W5PBS4	LRP1	3.73E-04	0.000
6	CYTO	W5PZG5	OCRL	7.83E-04	0.000
5	CYTO	W5Q678	SEC24D	4.05E-03	0.000
5	CYTO	W5PKY1	HNMT	6.87E-04	0.000
5	CYTO	W5QEM8	LOC101111528	1.15E-04	0.000
5	CYTO	W5QI40	MYO1E	2.87E-03	0.000
5	CYTO	W5PYI8	WWC1	1.40E-04	0.000
5	CYTO	W5PDJ0		4.03E-03	0.000
5	CYTO	W5PPX2	SENP8	2.27E-05	0.003
5	CYTO	W5QBG8	PPFIA1	1.96E-03	0.000
5	CYTO	W5PFD2	HOOK1	2.44E-04	0.009
5	CYTO	O18882	ATP6V0C ATP6C ATP6L	6.55E-04	0.000

Supplementary Table 18: Differentially abundant proteins (DAPs) of M2 relative to Mo and their cellular location.

FoldChange	Location	Protein IDs		P-value	FDR
5	CYTO	W5P5W6	NDRG1	1.34E-02	0.000
5	CYTO	G3M9U4	ACP5	9.07E-04	0.000
5	CYTO	W5QGG0	TFRC	3.53E-05	0.000
5	CYTO	W5NYL7	MTHFD1L	6.44E-05	0.000
5	CYTO	W5P4C9	MMP12	5.10E-02	0.000
5	CYTO	W5QBM4	ALCAM	3.73E-04	0.000
5	CYTO	W5P895	CRABP2	1.51E-03	0.000
5	CYTO	W5Q5T7	ATP6V1C1	6.87E-03	0.000
5	CYTO	W5P640	LMNA	3.25E-03	0.000
5	CYTO	W5P8M9		3.17E-03	0.000
5	CYTO	W5P9G8	PLD3	4.84E-02	0.000
5	CYTO	W5PE91	LOC101105400	1.03E-04	0.000
5	CYTO	W5PWX3	CRYL1	4.63E-04	0.000
5	CYTO	W5NYU9	MPP1	1.59E-05	0.000
5	CYTO	W5PV43	LRPAP1	1.24E-05	0.000
5	CYTO	W5NQS7	IFNGR1	1.75E-04	0.000
5	CYTO	W5NPP2	CPM	5.36E-04	0.000
5	CYTO	W5QFU4		6.81E-03	0.000
5	CYTO	W5NVR9	C21H11orf54	4.03E-06	0.000
5	CYTO	W5Q5C8	SOAT1	2.11E-04	0.000
5	CYTO	W5PGS4	FABP5	5.37E-04	0.000
5	CYTO	W5P026	STAB1	1.14E-04	0.000
5	CYTO	W5PV04	NIFK	2.69E-04	0.000
5	CYTO	W5P0C5	LGALS8	6.13E-05	0.000
5	CYTO	W5PDH7	NPC1	3.21E-04	0.000
5	CYTO	W5P1A5	GBA1	3.84E-03	0.000
5	CYTO	P83205	CTSB	3.05E-03	0.000
5	CYTO	W5NZ62	GNS	4.12E-03	0.000
5	CYTO	W5PI56	DAB2	4.51E-03	0.000
5	CYTO	W5PBC0		3.05E-03	0.000
5	CYTO	Q29524	LPL	3.45E-03	0.000
5	CYTO	W5Q3U3	LOC101102156	6.95E-03	0.000
5	CYTO	W5PE92	GRN	5.97E-04	0.000
5	CYTO	W5PYW0	TCIRG1	3.39E-04	0.000
4	CYTO	W5P1H0	CTSC	5.05E-06	0.000
4	CYTO	W5Q6S3	WASHC4	1.00E-03	0.000
4	CYTO	W5P2V3	PEPD	2.36E-03	0.000
4	CYTO	W5QEH0	TWF1	2.22E-03	0.000
4	CYTO	W5PZB2	CD68	5.65E-03	0.000
4	CYTO	W5P5Q2	MVP	1.11E-02	0.000
4	CYTO	W5Q0F1	LIPA	2.60E-04	0.000
4	CYTO	W5NWP6	DIAPH2	4.57E-03	0.000
4	CYTO	W5PP04	GNG12	6.48E-04	0.000
4	CYTO	W5QH69	RNF181	8.63E-04	0.000
4	CYTO	W5P168	SH3PXD2B	2.33E-05	0.000
4	CYTO	W5P703	WFS1	1.21E-03	0.000
4	CYTO	W5NUC8	ARMCX3	6.68E-04	0.000
4	CYTO	W5Q8J3	RRBP1	6.81E-03	0.000
4	CYTO	W5Q777	HS1BP3	1.92E-04	0.000
4	CYTO	W5PKU3		1.12E-03	0.000
4	CYTO	W5P530	LOC101104705	4.09E-02	0.000
4	CYTO	W5QB71	AMDHD2	1.15E-03	0.000
4	CYTO	W5Q9L2	LOC101109820	2.15E-03	0.000
4	CYTO	W5P3N6	LOC101112162	6.76E-04	0.000
4	CYTO	W5NSH8	NPC2	1.07E-05	0.000
4	CYTO	W5PXS1	RAB3IL1	1.06E-03	0.000
4	CYTO	W5PEE9	LAMP1	1.19E-02	0.000
4	CYTO	W5P078		1.98E-03	0.000
4	CYTO	W5P4K6	FCHO2	2.42E-04	0.000
4	CYTO	W5P7Y8	PALD1	1.48E-04	0.000
4	CYTO	W5PCM4	LUZP1	1.35E-04	0.000
4	CYTO	W5PDZ1		1.80E-03	0.000
4	CYTO	W5QH35	CAPG	4.24E-04	0.000

Supplementary Table 18: Differentially abundant proteins (DAPs) of M2 relative to Mo and their cellular location.

FoldChange	Location	Protein IDs		P-value	FDR
4	CYTO	W5QCM1	ARFGAP3	1.17E-02	0.000
4	CYTO	W5Q263	ICAM1	7.94E-04	0.000
4	CYTO	W5PBJ4	ARHGAP10	3.56E-03	0.000
4	CYTO	W5NX16		9.15E-04	0.000
4	CYTO	W5Q8V2	LIMA1	1.32E-04	0.000
4	CYTO	W5QI78	CTSK	3.92E-02	0.000
4	CYTO	W5Q1M0	GLB1	1.41E-04	0.000
4	CYTO	W5PEZ1		2.72E-04	0.000
4	CYTO	W5NW80	GAA	2.51E-03	0.000
4	CYTO	W5PVH4	TMEM251	5.11E-04	0.000
4	CYTO	W5PVR9	ERMP1	5.48E-03	0.000
4	CYTO	W5PTG5	ATP6V0D2	5.43E-02	0.000
4	CYTO	W5P4X6	LOC101104287	4.04E-03	0.000
4	CYTO	W5PF85	PEA15	9.72E-05	0.000
4	CYTO	W5Q5W2	LOC101110539	5.18E-05	0.000
4	CYTO	P12303	TTR	1.72E-02	0.000
4	CYTO	W5Q700	APPL2	5.28E-05	0.000
4	CYTO	W5NYU6	NT5DC2	1.34E-03	0.000
4	CYTO	W5PZD7		1.18E-04	0.000
4	CYTO	W5P3S0		1.26E-04	0.000
4	CYTO	W5NSS6		1.16E-02	0.000
4	CYTO	W5NU23	FUCA2	5.16E-04	0.000
4	CYTO	W5PUL5	FCGRT	2.29E-03	0.000
4	CYTO	W5PG28	NTMT1	1.25E-03	0.000
4	CYTO	W5PAZ0	RGCC	2.18E-04	0.000
4	CYTO	W5PFB1	TOR1B	3.13E-03	0.000
4	CYTO	C5IJ93	RAB9A	7.67E-04	0.000
4	CYTO	W5PBM2	LOC101110634	1.36E-03	0.000
4	CYTO	W5P7R2	HECTD1	2.68E-04	0.000
4	CYTO	W5NZV3	HMOX2	3.27E-02	0.000
4	CYTO	W5QEH8	CTTNBP2NL	2.45E-03	0.000
4	CYTO	W5NRL0	PLCD1	1.82E-04	0.000
4	CYTO	W5NYK1	PMVK	9.70E-04	0.000
4	CYTO	W5NQZ9	GSDMD	9.35E-04	0.000
4	CYTO	W5Q2J4	DIP2C	1.94E-05	0.000
4	CYTO	W5PT36	RBM47	9.56E-04	0.000
4	CYTO	W5Q2U7	PLEC	1.02E-06	0.000
4	CYTO	W5NPW8	STARD4	1.41E-03	0.000
4	CYTO	W5NWX7	CLEC4A	2.96E-04	0.000
4	CYTO	W5NV37	COMMD10	9.93E-03	0.000
4	CYTO	W5P536	TRIP10	3.84E-04	0.000
4	CYTO	W5QHU8	FNDC3B	7.51E-05	0.000
4	CYTO	W5Q0E7	LIN7C	8.72E-04	0.006
4	CYTO	W5PAC2	LOC101105044	1.43E-02	0.000
4	CYTO	W5PN65	PI4K2A	1.16E-03	0.000
4	CYTO	Q9MZD1	SLC17A5	2.83E-03	0.000
4	CYTO	W5PDU4	NMT2	1.09E-03	0.000
4	CYTO	W5PDH4	MMP9	2.86E-03	0.000
4	CYTO	W5PFE6	ACOX1	1.05E-03	0.000
4	CYTO	W5PKF9	FIG4	7.92E-04	0.000
4	CYTO	W5PI67	IDS	2.95E-03	0.000
4	CYTO	W5NYI4	RIPOR1	4.85E-04	0.000
4	CYTO	W5PUI3	GOLGA1	1.57E-05	0.000
4	CYTO	W5PAJ2	PSAP	6.47E-05	0.000
4	CYTO	W5PUM5	KANK1	9.80E-05	0.000
4	CYTO	W5Q9M9	GK	2.93E-02	0.000
4	CYTO	W5Q000	RPL32	1.15E-04	0.000
4	CYTO	W5QI95	CERS2	3.64E-03	0.000
4	CYTO	W5P4A8	RNASET2	3.62E-04	0.000
4	CYTO	W5Q6C5	GGA2	6.45E-03	0.000
4	CYTO	W5PN70		4.44E-03	0.000
4	CYTO	W5PBG1		9.00E-03	0.000
4	CYTO	W5PIE4	CLPTM1	2.68E-02	0.000

Supplementary Table 18: Differentially abundant proteins (DAPs) of M2 relative to Mo and their cellular location.

FoldChange	Location	Protein IDs		P-value	FDR
4	CYTO	W5Q1W7	PALLD	3.94E-03	0.000
4	CYTO	W5P1Q0	AP1B1	2.78E-05	0.000
4	CYTO	W5NYW8	CEP55	1.23E-04	0.000
4	CYTO	W5P3W6	OSBPL9	3.07E-04	0.000
4	CYTO	W5QA16		1.89E-04	0.000
4	CYTO	W5Q175	GNPTAB	1.07E-03	0.000
4	CYTO	W5PQM4	MOSPD2	3.76E-04	0.000
4	CYTO	W5Q6X2	TNFRSF1B	4.33E-04	0.000
3	CYTO	W5QEE5	TRAPPC3	5.03E-02	0.000
3	CYTO	W5QBJ2	SLC38A10	2.95E-04	0.000
3	CYTO	W5Q0A3	TLR2	4.71E-03	0.000
3	CYTO	W5PIS6	NHLRC3	4.24E-03	0.000
3	CYTO	W5PA90	AGA	7.50E-05	0.000
3	CYTO	W5P3L5	RNF13	4.03E-04	0.000
3	CYTO	W5PX29	TMEM9B	5.17E-02	0.000
3	CYTO	W5PQP3	LIMK1	1.51E-04	0.000
3	CYTO	W5P743	GLMP	2.23E-03	0.000
3	CYTO	W5P2F1	FOLR2	7.50E-06	0.000
3	CYTO	W5NYL0	MAOA	2.43E-02	0.000
3	CYTO	W5QCF3	SLC35F6	4.02E-03	0.000
3	CYTO	W5P093	NQO1	4.42E-04	0.000
3	CYTO	W5Q2K9	CYFIP1	6.37E-05	0.000
3	CYTO	W5QBV7	CD44	4.25E-05	0.000
3	CYTO	W5NS94		7.15E-05	0.000
3	CYTO	W5PJY6	ADAM28	2.40E-03	0.000
3	CYTO	W5PLB8	EPB41L3	1.77E-03	0.000
3	CYTO	W5QIC7	YBX3	2.17E-04	0.000
3	CYTO	W5Q280		1.81E-02	0.000
3	CYTO	W5NQ23	WIPF1	5.80E-03	0.000
3	CYTO	W5Q5Q7	ASPA	1.84E-02	0.000
3	CYTO	W5PTE6	CEMIP2	3.83E-03	0.000
3	CYTO	W5PXK7	RASA4B	3.00E-03	0.000
3	CYTO	W5PEL8	PDXDC1	1.89E-03	0.000
3	CYTO	W5PCD0	FUBP3	2.14E-02	0.000
3	CYTO	W5QBR5	BMP2K	5.17E-03	0.000
3	CYTO	W5NXZ9	RASSF4	5.02E-03	0.000
3	CYTO	W5P3C6	LOC101111906	1.28E-05	0.000
3	CYTO	W5NTW0	CADM1	2.03E-02	0.000
3	CYTO	W5QG92	OSBPL11	2.67E-04	0.000
3	CYTO	W5Q2V0	YKT6	3.66E-05	0.000
3	CYTO	W5Q3N1	CTSZ	5.89E-05	0.000
3	CYTO	W5PGX8	DNAJC13	3.84E-02	0.000
3	CYTO	W5PES0	STX4	7.73E-05	0.000
3	CYTO	W5P434	NAGPA	1.81E-03	0.000
3	CYTO	W5QGF4	VPS18	4.11E-03	0.000
3	CYTO	W5PGC6	DPP9	3.39E-02	0.000
3	CYTO	W5PT68	FLNB	4.88E-02	0.000
3	CYTO	W5PQ47	RAI14	1.51E-02	0.000
3	CYTO	W5PHY4	TYMS	5.36E-03	0.000
3	CYTO	W5PH26		1.95E-03	0.000
3	CYTO	W5Q740	ABCD3	9.50E-03	0.000
3	CYTO	W5QAK7	SCARB1	9.07E-05	0.000
3	CYTO	Q10994	CSTB CST6	5.95E-05	0.000
3	CYTO	W5Q568	TRANK1	6.83E-05	0.006
3	CYTO	W5PGF4	PLAUR	1.64E-02	0.000
3	CYTO	W5PCC0	LACC1	2.71E-03	0.000
3	CYTO	W5PBB5	CTSF	3.55E-03	0.000
3	CYTO	W5P6V4	GLG1	5.23E-02	0.000
3	CYTO	W5PUM8	HIP1	5.88E-04	0.000
3	CYTO	W5QH03	LPP	2.00E-02	0.000
3	CYTO	W5Q8Y5	HDLBP	2.01E-03	0.000
3	CYTO	W5PPY9		1.06E-02	0.000
3	CYTO	W5Q8I7	LOC101122123	2.25E-03	0.000

Supplementary Table 18: Differentially abundant proteins (DAPs) of M2 relative to Mo and their cellular location.

FoldChange	Location	Protein IDs		P-value	FDR
3	CYTO	W5PEX1	WASHC5	9.71E-03	0.000
3	CYTO	W5QCP4	TRAF6	8.42E-04	0.000
3	CYTO	W5P4Q3	MSH6	3.46E-04	0.002
3	CYTO	W5QI36	HEBP1	3.25E-02	0.000
3	CYTO	W5PHG6	SMYD2	1.83E-03	0.000
3	CYTO	W5PVH8	ATP6V1E1	6.77E-05	0.000
3	CYTO	W5Q3C2	EPPK1	5.68E-03	0.000
3	CYTO	W5NS73		2.42E-03	0.003
3	CYTO	W5PE73	SMPDL3A	2.68E-02	0.000
3	CYTO	W5PRG8	CREG1	1.04E-02	0.000
3	CYTO	W5Q5K0		5.06E-03	0.000
3	CYTO	W5Q2R5	SDF2L1	4.70E-02	0.000
3	CYTO	W5NSQ3	HFE	2.45E-03	0.000
3	CYTO	W5PIR3	BLZF1	4.37E-04	0.006
3	CYTO	W5PC32	PGM3	6.11E-03	0.000
3	CYTO	W5Q728	TPCN2	8.93E-04	0.000
3	CYTO	W5PJS4	EMILIN2	1.90E-02	0.000
3	CYTO	W5QHR5	PLEKHO2	1.65E-03	0.000
3	CYTO	W5PSP1	ERC1	2.02E-02	0.000
3	CYTO	W5QEL9	ARHGAP31	5.51E-03	0.000
3	CYTO	W5QAQ4	NRP2	1.20E-03	0.000
3	CYTO	W5PI02	TBC1D13	1.23E-02	0.000
3	CYTO	W5QJ37		2.42E-04	0.000
3	CYTO	W5QAA7	MAPK7	6.49E-04	0.000
3	CYTO	P82197	PDXK PKH	2.14E-02	0.000
3	CYTO	W5NXT6	UBR3	2.76E-03	0.000
3	CYTO	W5PBX4	FAR1	2.21E-03	0.000
3	CYTO	W5P1M3		2.04E-03	0.000
3	CYTO	W5QEL6	PCYOX1	1.53E-02	0.000
3	CYTO	W5PJ58	HPS5	1.40E-03	0.000
3	CYTO	W5QI70	CTSS	1.61E-04	0.000
3	CYTO	W5Q5C7	CTNNA1	3.23E-02	0.000
3	CYTO	W5PQ75	HSPH1	1.08E-02	0.000
3	CYTO	W5Q3B4	TBCD	1.54E-03	0.000
3	CYTO	W5Q3Y3	SFXN3	5.16E-03	0.000
3	CYTO	W5NPP0		6.08E-03	0.000
3	CYTO	W5P5P8	PSMG3	4.36E-02	0.000
3	CYTO	W5Q0C3	KIF13B	3.21E-02	0.000
3	CYTO	W5P3I5	CNDP2	3.36E-04	0.000
3	CYTO	W5QHD4	EIF4G1	5.10E-02	0.000
3	CYTO	W5Q644	VPS29	2.63E-02	0.000
3	CYTO	W5P369	AP2A2	5.25E-06	0.000
3	CYTO	W5PVG5	BLTP3B	2.58E-04	0.000
3	CYTO	W5PPK8		6.25E-03	0.000
3	CYTO	W5Q552	ANAPC7	3.91E-02	0.000
3	CYTO	W5PYE8	MERTK	4.30E-03	0.000
3	CYTO	W5PUL4	MTMR6	1.96E-02	0.000
3	CYTO	W5NUU1	VRK2	5.53E-03	0.000
3	CYTO	W5PQV2	LMBRD1	3.49E-05	0.000
3	CYTO	W5PER8	WDR91	1.89E-02	0.000
3	CYTO	W5Q553	ITGAV	1.60E-03	0.000
3	CYTO	B9VGZ7	SLIRP	2.34E-02	0.000
3	CYTO	W5PVE3	LOC101115252	4.57E-05	0.000
3	CYTO	W5QHL1	FCGR1A	3.40E-02	0.000
3	CYTO	W5QH60	VAMP8	1.12E-02	0.000
3	CYTO	W5QET8	TEP1	4.20E-02	0.000
3	CYTO	W5PYH6	AP3D1	8.18E-03	0.000
3	CYTO	W5PWA8	HSPB1	3.71E-03	0.000
3	CYTO	W5NZ71		2.20E-02	0.000
3	CYTO	W5P3H1	LOC101111732	5.25E-03	0.000
3	CYTO	W5PBX0	ATP11A	4.40E-02	0.000
3	CYTO	W5Q0J1	PLA2G6	5.15E-03	0.005
3	CYTO	W5NR48	KPNA6	3.05E-02	0.000

Supplementary Table 18: Differentially abundant proteins (DAPs) of M2 relative to Mo and their cellular location.

FoldChange	Location	Protein IDs		P-value	FDR
3	CYTO	W5PB38		2.26E-03	0.000
3	CYTO	W5PBR7	P4HA1	1.53E-02	0.000
3	CYTO	W5PYB9	ABCA6	1.02E-03	0.000
3	CYTO	W5PZ62	ZFYVE16	1.34E-02	0.000
3	CYTO	W5PKA1	LOC101116157	4.25E-03	0.000
3	CYTO	W5PNG4	BRCA2	4.32E-02	0.005
3	CYTO	W5PC06	SIRPA	2.45E-04	0.000
3	CYTO	W5Q420		1.35E-02	0.000
3	CYTO	W5NRA9	ASL	1.87E-02	0.000
3	CYTO	W5Q0G8	IMPA1	5.88E-04	0.000
3	CYTO	W5PZ94	ACO1	5.49E-02	0.000
3	CYTO	W5PAX1	GCLC	2.84E-02	0.000
3	CYTO	W5P1U3	LACTB2	3.09E-02	0.000
3	CYTO	W5NPU0	NAPRT	8.23E-03	0.000
3	CYTO	W5PHX8	GLRX2	8.37E-03	0.000
3	CYTO	W5QCG9	SRXN1	4.22E-02	0.000
3	CYTO	W5PF73	PLAU	2.11E-03	0.000
3	CYTO	W5PH35	LOC101119706	2.35E-03	0.000
3	CYTO	W5PQZ5	GDE1	2.75E-03	0.000
3	CYTO	W5Q8T1	CLPX	1.53E-02	0.000
3	CYTO	W5PMA0	AP2S1	1.11E-02	0.000
3	CYTO	W5Q3A2		3.78E-02	0.000
3	CYTO	W5QDC0	SNX17	2.83E-02	0.000
3	CYTO	Q9XT28	ATOX1	8.19E-04	0.000
3	CYTO	W5NQ85	IDE	3.32E-02	0.000
3	CYTO	W5P1T1	ANO10	9.96E-03	0.000
3	CYTO	W5PC57	MRTFB	4.69E-02	0.000
3	CYTO	W5QJ45	GPHN	4.96E-03	0.000
3	CYTO	W5NU07		1.24E-02	0.000
3	CYTO	W5PRM9	BCAT2	5.35E-02	0.000
3	CYTO	W5PN60	ABR	2.26E-02	0.000
3	CYTO	W5P7X7	MAGED2	3.52E-02	0.000
3	CYTO	W5NRI6	PLPBP PROSC	5.60E-03	0.000
3	CYTO	W5P8A0		4.13E-02	0.000
3	CYTO	W5NT95	ATP6AP2	3.02E-04	0.000
3	CYTO	W5PER3	TM9SF3	1.98E-02	0.000
3	CYTO	W5P9L5	RASA2	2.39E-03	0.000
3	CYTO	W5PS50	MPC2	1.70E-02	0.000
3	CYTO	W5QIA5	ETV6	1.38E-02	0.000
3	CYTO	W5Q3R4	CLUH	4.35E-05	0.000
3	CYTO	W5P3H9	PICALM	1.11E-05	0.000
3	CYTO	W5PJH6		1.48E-02	0.000
3	CYTO	W5NVV6	DNAJC3	4.13E-02	0.000
3	CYTO	W5P5E7		3.90E-03	0.000
3	CYTO	W5Q5Z1	YTHDF3	8.28E-03	0.000
3	CYTO	W5PAM5		2.47E-04	0.000
3	CYTO	W5QIU9	S100A10	6.80E-03	0.000
3	CYTO	W5PJN0	PSPH	2.45E-02	0.000
3	CYTO	W5P4B3	NRDC	1.10E-02	0.000
3	CYTO	W5NUQ8	GCC1	2.41E-02	0.000
3	CYTO	W5QH13	VPS39	2.92E-03	0.000
3	CYTO	W5Q3D8	CLASP2	9.11E-03	0.000
3	CYTO	W5PA78	AVL9	1.51E-02	0.000
3	CYTO	W5PJJ3	LARP1	2.60E-02	0.000
3	CYTO	W5PVQ4	TMEM120A	6.69E-03	0.000
3	CYTO	W5QIA8	YARS1	8.49E-04	0.000
3	CYTO	W5Q4N4	TOR3A	7.86E-03	0.000
3	CYTO	W5NV06	ATP6V0A1	3.67E-02	0.000
3	CYTO	W5PCK8	LOC100135455	1.30E-03	0.000
3	CYTO	W5NUJ2	PEAK1	9.20E-03	0.000
3	CYTO	W5NWX4		5.43E-02	0.000
3	CYTO	W5NQG2	TBC1D2B	1.44E-05	0.000
3	CYTO	W5Q8I6	POLDIP2	1.12E-02	0.000

Supplementary Table 18: Differentially abundant proteins (DAPs) of M2 relative to Mo and their cellular location.

FoldChange	Location	Protein IDs		P-value	FDR
3	CYTO	W5PIN6	LDHA	4.07E-04	0.000
3	CYTO	W5P1S3	SLC25A13	4.39E-03	0.000
3	CYTO	W5QEG2	MAP7D1	5.28E-03	0.000
3	CYTO	W5Q8B9	STK25	1.32E-02	0.000
3	CYTO	W5PWR6	KCNAB2	3.55E-03	0.000
3	CYTO	W5PF22	KMO	1.30E-02	0.000
3	CYTO	W5PK12	OAT	5.99E-05	0.000
3	CYTO	W5Q3L8	LOC101104306	1.92E-03	0.000
3	CYTO	W5P560	PIK3C3	5.29E-02	0.000
2	CYTO	W5PUW5	GOLGA5	1.42E-02	0.000
2	CYTO	W5QEL5	MRPS35	5.53E-02	0.000
2	CYTO	W5NSS0	ARAP3	4.95E-03	0.000
2	CYTO	W5QCA6	UBE2F	6.32E-05	0.000
2	CYTO	W5PSG0		6.36E-03	0.000
2	CYTO	W5PXT9	DENND4C	2.80E-03	0.000
2	CYTO	W5PUW2	IFI30	3.57E-03	0.000
2	CYTO	W5QDT8		9.11E-03	0.000
2	CYTO	W5P7L2	ATP6V1G1	8.09E-05	0.000
2	CYTO	W5PHI1	MRPL3	1.98E-02	0.000
2	CYTO	W5QAT6	MTHFR	9.77E-04	0.000
2	CYTO	W5PVA4	ABCF1	2.55E-02	0.000
2	CYTO	W5QIJ6	SPPL2A	4.55E-02	0.000
2	CYTO	W5NY99		6.74E-03	0.000
2	CYTO	W5P3X8	KIF15	5.24E-04	0.000
2	CYTO	W5P4L4	TMX3	4.54E-03	0.000
2	CYTO	W5PGL9		4.40E-03	0.000
2	CYTO	W5PZS4	OSBPL8	1.39E-02	0.000
2	CYTO	W5PNW7	VIM	9.95E-06	0.000
2	CYTO	W5P2N4	AHCYL2	3.29E-02	0.000
2	CYTO	W5QFK2	MACF1	3.13E-02	0.000
2	CYTO	W5PTR6	ACOX3	5.47E-03	0.000
2	CYTO	W5QDF4	GSTM3	6.87E-03	0.000
2	CYTO	W5Q758	DCTN5	3.97E-02	0.000
2	CYTO	P81184	LGALS1	1.83E-04	0.000
2	CYTO	W5Q5B5	CSAD	3.46E-04	0.000
2	CYTO	W5QA59	HGS	1.98E-02	0.000
2	CYTO	W5Q8K4	SLC3A2	6.73E-03	0.000
2	CYTO	W5PLZ0	ATP6V1B2	4.11E-04	0.000
2	CYTO	W5PRS4	FKBP5	2.13E-02	0.000
2	CYTO	W5PUW9	PDCL3	1.89E-02	0.000
2	CYTO	W5QEQ1	TRABD	4.20E-02	0.000
2	CYTO	W5QHV8	PLD1	1.65E-02	0.000
2	CYTO	W5NZ70	LGALS3BP	3.97E-02	0.000
2	CYTO	W5PKS2	SARAF	4.62E-02	0.000
2	CYTO	W5Q501	LOC101115640	1.61E-03	0.000
2	CYTO	W5PWF2	ATP6V1H	9.15E-04	0.000
2	CYTO	W5QC34	MAN2A1	2.94E-03	0.000
2	CYTO	W5PMH1	GSS	3.97E-03	0.000
2	CYTO	W5NSE1	DRG2	2.13E-03	0.000
2	CYTO	W5Q8J8	VPS41	1.79E-03	0.000
2	CYTO	W5P867	MAPKAPK2	3.38E-03	0.000
2	CYTO	W5QAE8	ALDH7A1	8.12E-05	0.000
2	CYTO	W5QA65	ZW10	2.81E-02	0.000
2	CYTO	W5NUE3	PRDX1	6.56E-04	0.000
2	CYTO	W5NWF5	RARRES1	4.66E-03	0.000
2	CYTO	W5PRJ7	CCDC93	5.65E-03	0.000
2	CYTO	W5NWR6	LXN	4.89E-02	0.000
2	CYTO	W5QDF2	ATG3	1.25E-02	0.000
2	CYTO	W5P4G4	RAB7B	2.00E-02	0.000
2	CYTO	W5NYK9	CALU	2.08E-02	0.000
2	CYTO	W5QD46	CYTH4	1.51E-02	0.000
2	CYTO	W5P3A2		2.46E-03	0.000
2	CYTO	W5PWB8	ARHGAP12	1.34E-02	0.000

Supplementary Table 18: Differentially abundant proteins (DAPs) of M2 relative to Mo and their cellular location.

FoldChange	Location	Protein IDs		P-value	FDR
2	CYTO	W5PC18		1.06E-02	0.000
2	CYTO	W5P3H8	IGF2R	1.91E-02	0.000
2	CYTO	W5QJ49	ATP6V1D	5.72E-04	0.000
2	CYTO	W5Q928	PCYT2	1.12E-02	0.000
2	CYTO	W5P1K2	TRAPPC9	9.77E-05	0.000
2	CYTO	P04074	ATP1A1	2.68E-03	0.000
2	CYTO	W5PPR1	UCK2	1.14E-02	0.000
2	CYTO	W5NRF7	MAP3K20	1.37E-03	0.000
2	CYTO	Q6XUZ5	IDH1	2.04E-05	0.000
2	CYTO	Q6XXL8	DYNLT3 TCTE1L	7.89E-03	0.000
2	CYTO	W5P7E8		4.72E-05	0.000
2	CYTO	W5P1T4	PPP4C	1.48E-03	0.000
2	CYTO	W5QIK3	USP8	1.18E-02	0.000
2	CYTO	W5PKV1	DNASE2	2.32E-02	0.000
2	CYTO	W5PEN2	TPD52	6.62E-03	0.000
2	CYTO	W5Q3P7	ITFG1	1.56E-02	0.000
2	CYTO	W5QBW4	VAV3	5.59E-04	0.000
2	CYTO	W5PZN5	KIF3A	4.61E-02	0.000
2	CYTO	W5P7L0	SUN1	6.80E-03	0.000
2	CYTO	W5Q4K4	EVI5	1.10E-02	0.000
2	CYTO	W5Q3C1	NDRG3	1.71E-03	0.000
2	CYTO	W5P4Z8	MRPS27	3.99E-02	0.000
2	CYTO	W5PUJ2	LAMP2	4.93E-03	0.000
2	CYTO	W5PP47		2.24E-04	0.000
2	CYTO	W5PI27	UEVLD	2.19E-03	0.000
2	CYTO	W5Q540	HAGH	7.92E-03	0.000
2	CYTO	W5P8E1	DNPH1 RCL	4.41E-02	0.000
2	CYTO	W5QEU6	ANXA4	3.06E-02	0.000
2	CYTO	W5PG41	H6PD	3.80E-02	0.000
2	CYTO	W5NQQ4	TXLNA	4.37E-02	0.000
2	CYTO	W5PL89	GSR	2.60E-02	0.000
2	CYTO	W5P6P2	LOC101110647	3.31E-02	0.000
2	CYTO	W5PSB2	TBL2	4.04E-03	0.000
2	CYTO	W5Q4Q8		8.81E-04	0.000
2	CYTO	W5PFE7	ACOX1	1.40E-02	0.000
2	CYTO	W5NVF6	NR3C1	4.69E-02	0.000
2	CYTO	W5P6C1	ADSS ADSS2	1.03E-02	0.000
2	CYTO	W5P985	ABHD14B	6.93E-03	0.000
2	CYTO	W5QFN9	UGP2	2.54E-04	0.000
2	CYTO	W5P180		7.80E-03	0.000
2	CYTO	W5P5S5	EMC3	1.92E-02	0.000
2	CYTO	W5PXJ1	MAT2B	4.42E-03	0.000
2	CYTO	W5PFQ5	ANKMY2	3.69E-03	0.000
2	CYTO	W5PGD6	BZW2	1.23E-03	0.000
2	CYTO	W5PJY4	RAVER2	2.13E-02	0.000
2	CYTO	W5NQC1	PSEN1	1.43E-03	0.000
2	CYTO	W5QE35	IPO8	4.86E-03	0.000
2	CYTO	W5PMG3	MEMO1	1.35E-03	0.000
2	CYTO	W5PSF8	CRYZ	3.57E-02	0.000
2	CYTO	W5P5A7	VPS16	1.45E-03	0.000
2	CYTO	W5PDN7	GPD2	4.48E-03	0.000
2	CYTO	W5P612	CRELD1	4.19E-03	0.000
2	CYTO	W5QIX6	EXOC5	5.54E-02	0.000
2	CYTO	W5NUE1	NCF2	9.20E-03	0.000
2	CYTO	W5Q9H8	SNX5	1.87E-02	0.000
2	CYTO	W5PKQ1	SORBS3	3.68E-03	0.000
2	CYTO	W5P7I6	ARAP1	1.01E-03	0.000
2	CYTO	W5PU61	SETD7	4.65E-02	0.000
2	CYTO	W5PXH4	PTPRE	2.87E-03	0.000
2	CYTO	W5PCS4		4.18E-03	0.000
2	CYTO	W5NW41	TBC1D9B	3.20E-02	0.000
2	CYTO	W5PRK6	STAU1	3.76E-02	0.000
2	CYTO	W5PDU8	GARS1	1.53E-04	0.000

Supplementary Table 18: Differentially abundant proteins (DAPs) of M2 relative to Mo and their cellular location.

FoldChange	Location	Protein IDs		P-value	FDR
2	CYTO	W5P1Z5	PMPCA	2.62E-03	0.000
2	CYTO	W5PSX7	FES	1.42E-02	0.000
2	CYTO	W5QHT6	NCEH1	8.31E-04	0.000
2	CYTO	W5NXM4	RPS9	3.94E-02	0.000
2	CYTO	W5P700	KIF1B	3.07E-02	0.000
2	CYTO	W5Q0B4	KYAT3	2.41E-02	0.000
2	CYTO	W5QG87	SNX4	3.32E-02	0.000
2	CYTO	W5P6S8	COBLL1	4.23E-02	0.000
2	CYTO	W5QAL6	FMNL3	3.33E-03	0.000
2	CYTO	W5QHF8	NEMF	1.13E-02	0.000
2	CYTO	W5PZ05		1.16E-04	0.000
2	CYTO	W5QAP3	TOM1	6.43E-03	0.000
2	CYTO	W5PSV2	TRIP11	3.59E-02	0.000
2	CYTO	P68251	YWHAB	1.31E-02	0.000
2	CYTO	W5PS76	UNC45A	1.19E-02	0.000
2	CYTO	W5PUF8	PITRM1	5.86E-03	0.000
2	CYTO	W5QG63	ZMPSTE24	1.30E-02	0.000
2	CYTO	W5QCD2	ADPGK	2.72E-03	0.000
2	CYTO	W5PVT3	GALNS	2.62E-02	0.000
2	CYTO	W5P5H4	AARS AARS1	3.73E-03	0.000
2	CYTO	W5Q3J3	COLGALT1	1.02E-02	0.000
2	CYTO	W5P316	NAMPT	9.63E-03	0.000
2	CYTO	W5QGN5	DLG1	5.03E-05	0.000
2	CYTO	W5NWR7	GPAT4	3.88E-02	0.000
2	CYTO	Q8HY31	UROD	6.40E-03	0.000
2	CYTO	W5QFD2	TRIM33	4.30E-02	0.010
2	CYTO	W5P0D5	PSMD7	1.64E-02	0.000
2	CYTO	W5P3W2	PPP2R5D	1.41E-02	0.000
2	CYTO	W5NYQ9	TIMM44	4.53E-02	0.000
2	CYTO	W5P5K1	NUDT1	3.45E-02	0.000
2	CYTO	W5QEA3		1.70E-03	0.000
2	CYTO	W5Q7T6	AACS	4.21E-02	0.000
2	CYTO	W5PD54	NCK1	1.31E-03	0.000
2	CYTO	W5PXD7	LAMTOR3	5.29E-05	0.000
2	CYTO	W5PP30	ASNA1 GET3	4.15E-05	0.000
2	CYTO	W5NUG3	GNPDA1	4.82E-02	0.000
2	CYTO	W5P1J4	NCAPG	4.63E-02	0.000
2	CYTO	W5PSD7	RAP2C	2.11E-02	0.000
2	CYTO	W5PCT8	ASAP1	8.42E-03	0.000
2	CYTO	W5Q2H2	SLC12A9	1.71E-03	0.000
2	CYTO	W5Q6T1	ARSB	1.14E-02	0.000
2	CYTO	W5QCK4	GRK3	2.65E-02	0.000
2	CYTO	W5Q7J2	AGPS	2.76E-02	0.000
2	CYTO	W5PVC1	SYNJ1	4.45E-03	0.000
2	CYTO	W5P0S9	GDPD3	2.52E-03	0.000
2	CYTO	W5QDJ4	GIGYF2	1.01E-02	0.000
2	CYTO	W5PBQ8	ODR4	2.63E-02	0.000
2	CYTO	W5PHT8	DOCK7	1.75E-04	0.000
2	CYTO	W5P0L9	MAPK3	3.29E-03	0.000
2	CYTO	W5PLQ3	LARS2	1.81E-02	0.000
2	CYTO	W5QDY5	ATP6V1A	9.64E-04	0.000
2	CYTO	W5QFY4	COMMD5	1.63E-03	0.000
2	CYTO	W5NVV3	UBAP2L	5.09E-02	0.000
2	CYTO	W5Q8I0	SPC24	2.44E-02	0.000
2	CYTO	W5PRQ0	RBM3	2.17E-02	0.000
2	CYTO	W5Q465	TRIM36	1.53E-02	0.000
2	CYTO	W5PDX8	FAM120A	7.87E-03	0.000
2	CYTO	W5QHA0	AGFG1	3.95E-04	0.000
2	CYTO	W5PKT0	NAA25	4.80E-02	0.002
2	CYTO	W5Q922	LOC101105383	1.75E-03	0.000
2	CYTO	W5Q7I9	HPS6	7.49E-03	0.000
2	CYTO	W5QGG8	MAN1A2	2.53E-04	0.000
2	CYTO	W5PQ08	B3GLCT	4.88E-02	0.000

Supplementary Table 18: Differentially abundant proteins (DAPs) of M2 relative to Mo and their cellular location.

FoldChange	Location	Protein IDs		P-value	FDR
2	CYTO	W5PWK3	TBRG4	1.53E-02	0.001
2	CYTO	W5NSS1	PPP4R1	4.21E-03	0.000
2	CYTO	W5QFH0	ARSA	8.52E-03	0.000
2	CYTO	W5PA89	ST6GALNAC2	2.51E-02	0.002
2	CYTO	W5QFJ5	STK16	3.21E-02	0.000
2	CYTO	C5HK62	EYA3	1.71E-02	0.000
2	CYTO	W5NWM6	GFM1 EFG1	2.82E-04	0.000
2	CYTO	W5QB79	GLCE	5.35E-02	0.000
2	CYTO	W5P7P6	MYO18A	4.80E-03	0.000
2	CYTO	W5PHS5	RGL2	3.88E-02	0.000
2	CYTO	W5QAS2	STX6	2.12E-02	0.000
2	CYTO	W5QHG2	PTCD3	1.69E-02	0.000
2	CYTO	W5P6G0	PSMD8	1.59E-04	0.000
2	CYTO	W5P5T6	PPP1R14B	5.19E-02	0.000
2	CYTO	W5P488	TTYH3	3.43E-02	0.000
2	CYTO	W5NQG1	SETD3	1.95E-02	0.000
2	CYTO	W5PV12	MIPEP	4.13E-02	0.000
2	CYTO	W5QG24	PPT1	2.26E-03	0.000
2	CYTO	W5QDN8	SLC48A1	2.51E-02	0.000
2	CYTO	W5PSQ0	DOHH	3.32E-02	0.000
2	CYTO	W5P3Z5	EEPD1	1.84E-02	0.000
2	CYTO	W5PYW5	AZI2	8.10E-04	0.000
2	CYTO	W5NYP8	DYNC1H1	4.59E-06	0.000
2	CYTO	W5PRB0	UFL1	4.02E-03	0.000
2	CYTO	W5PJB6	PGM1	7.71E-03	0.000
2	CYTO	W5NZV0	HMOX2	2.13E-03	0.000
2	CYTO	W5Q2L6	EXOC3	5.47E-02	0.000
2	CYTO	W5PME7	PALS2	9.61E-03	0.000
2	CYTO	W5P2N3	MON2	1.19E-02	0.000
2	CYTO	W5QGX6	C1H1orf50	5.39E-02	0.000
2	CYTO	W5Q6V7	SIPA1	4.72E-02	0.000
2	CYTO	W5NXX1	IPO5	3.71E-05	0.000
2	CYTO	W5PD03		1.81E-03	0.000
2	CYTO	W5NZ43		1.43E-02	0.000
2	CYTO	W5QFL4	CSDE1	4.42E-03	0.000
2	CYTO	W5Q1D8	GALNT7	1.45E-02	0.000
2	CYTO	W5Q7Z6	DIP2B	1.86E-02	0.000
2	CYTO	C8BKE1	STAT1 STAT4	2.89E-03	0.000
2	CYTO	W5QB83	SMCR8	1.34E-02	0.000
2	CYTO	W5PGC9	MMAA	6.62E-03	0.000
2	CYTO	W5NV74	CARS1	9.44E-03	0.000
2	CYTO	W5NX86	EIF1B	2.26E-02	0.000
2	CYTO	W5NTM7	DENND10	4.74E-02	0.000
2	CYTO	W5PD41	GIT1	2.55E-03	0.000
2	CYTO	W5P6J4	ZYG11B	3.31E-02	0.000
2	CYTO	W5P7B1	SIRT2	2.02E-02	0.000
2	CYTO	W5NQ97		2.34E-02	0.000
2	CYTO	W5PRW3	GOLPH3	5.56E-03	0.000
2	CYTO	W5P2Q3	RPS6KA1	9.95E-03	0.000
2	CYTO	W5PXJ8	CMBL	2.29E-02	0.000
2	CYTO	W5Q289	ATP2B1	1.99E-03	0.000
2	CYTO	W5PPJ2	VPS33A	9.52E-03	0.000
2	CYTO	W5PXC4	UPB1	1.84E-02	0.000
2	CYTO	W5QCG3	CLCC1	3.99E-02	0.000
2	CYTO	W5PS05	MTHFD2	5.74E-03	0.000
2	CYTO	W5PGJ4	CHN2	1.70E-02	0.000
2	CYTO	A2SW69	ANXA2 ANX2	4.85E-03	0.000
2	CYTO	W5NRK5	PPT2	8.45E-03	0.000
2	CYTO	Q6ECI6	ITGB2 CD18	1.35E-04	0.000
2	CYTO	W5Q7R8	JUP	9.67E-03	0.000
2	CYTO	W5P7H5	ADGRE5	7.22E-04	0.000
2	CYTO	W5Q0M7	RAP2B	1.60E-03	0.000
2	CYTO	W5PWV5	WDR81	1.25E-02	0.000

Supplementary Table 18: Differentially abundant proteins (DAPs) of M2 relative to Mo and their cellular location.

FoldChange	Location	Protein IDs		P-value	FDR
2	CYTO	W5PZ99	KIDINS220	2.39E-02	0.000
2	CYTO	W5QB43	CDK6	7.45E-03	0.000
2	CYTO	W5PTP8	HK2	2.64E-02	0.000
2	CYTO	W5QIM0	GALK2	4.04E-02	0.000
2	CYTO	W5Q0Q2		7.03E-04	0.000
2	CYTO	W5PVC2	CDC37L1	2.76E-03	0.000
2	CYTO	W5Q0P4	MTMR9	2.67E-03	0.000
2	CYTO	W5PD62	CPB2	2.96E-02	0.009
2	CYTO	W5P8N8	IDH3B	1.44E-02	0.000
2	CYTO	W5PZM9	ANXA5	6.45E-04	0.000
2	CYTO	W5Q018	PKN2	6.34E-04	0.000
2	CYTO	W5QGC6	FYTTD1	3.13E-02	0.000
2	CYTO	W5PUU2	RRAS	3.56E-02	0.000
2	CYTO	W5NY29		2.96E-02	0.000
2	CYTO	W5PTI6	ST3GAL4	4.01E-02	0.000
2	CYTO	W5PHV7	SLC43A3	3.55E-02	0.000
2	CYTO	W5P929	MARS1	4.50E-03	0.000
2	CYTO	W5Q4U5	CPT1A	4.96E-02	0.000
2	CYTO	W5NPC7	ACBD3	2.95E-02	0.000
2	CYTO	W5Q6F0	LOC101106791	3.61E-02	0.000
2	CYTO	W5NQ27	ZFYVE1	1.84E-03	0.000
2	CYTO	W5Q165	RRM2	4.04E-02	0.000
2	CYTO	W5NUS6	BLTP2	1.37E-02	0.000
2	CYTO	W5NTC8	DDHD2	2.85E-02	0.000
2	CYTO	W5PIS1	FAM91A1	3.24E-02	0.000
2	CYTO	W5QBG3	APIP	2.78E-02	0.000
2	CYTO	W5PNW2	BLOC1S1	6.41E-03	0.000
2	CYTO	W5PFQ8	RRAGA	1.30E-02	0.000
2	CYTO	W5PL61	WWC3	5.28E-02	0.001
2	CYTO	W5QCC0	RALGAPB	3.09E-02	0.000
2	CYTO	W5P958	IDH3G	4.23E-03	0.000
2	CYTO	W5PMB1	SNX3	2.51E-03	0.000
2	CYTO	W5P3U4	COMMD2	4.50E-02	0.000
2	CYTO	W5NZZ3	ATP6V1F	2.99E-03	0.000
2	CYTO	W5Q738	TMEM173 STING1	5.35E-03	0.000
2	CYTO	W5PNT5	PSMD6	6.20E-03	0.000
2	CYTO	W5PWZ2		1.45E-02	0.000
-2	CYTO	W5QEL2	SNRPG	4.71E-02	0.000
-2	CYTO	W5PIZ3	GRK2	1.81E-02	0.000
-2	CYTO	W5PK31	FCGR3A	2.51E-02	0.000
-2	CYTO	W5PJ14	ESYT2	1.96E-02	0.000
-2	CYTO	W5PFJ5	SMC1A	5.37E-02	0.000
-2	CYTO	W5PV11	IRF3	2.85E-02	0.000
-2	CYTO	W5PKX6	DCTN6	5.62E-02	0.000
-2	CYTO	W5Q3W4	RAB18	2.98E-02	0.000
-2	CYTO	W5PR89	STAMBP	7.81E-03	0.000
-2	CYTO	W5PJD0	PGM2	1.67E-02	0.000
-2	CYTO	W5Q7F2	TIMM29	1.22E-02	0.000
-2	CYTO	C5IWU4	ARL3	2.26E-03	0.000
-2	CYTO	W5PE01	TAGLN2	6.73E-03	0.000
-2	CYTO	W5PLV3	RAB5B	5.86E-03	0.000
-2	CYTO	W5PCG2	TAP2	8.66E-03	0.000
-2	CYTO	W5PRF0	SUB1	6.47E-03	0.000
-2	CYTO	W5P627	GSN	3.53E-02	0.000
-2	CYTO	W5NQ72	PFAS	3.45E-03	0.000
-2	CYTO	W5Q859	STX5	7.04E-04	0.000
-2	CYTO	W5PR48	HPRT1	4.62E-02	0.000
-2	CYTO	W5QGB5	LOC101113369	9.00E-03	0.000
-2	CYTO	W5Q3Q2	ERCC3	1.46E-02	0.004
-2	CYTO	W5PBA6	SPON1	9.50E-03	0.000
-2	CYTO	W5PB27	NDUFS3	7.99E-03	0.000
-2	CYTO	W5PM65	PPP2R5A	4.24E-02	0.000
-2	CYTO	W5PLX5	PRKD2	1.19E-03	0.000

Supplementary Table 18: Differentially abundant proteins (DAPs) of M2 relative to Mo and their cellular location.

FoldChange	Location	Protein IDs		P-value	FDR
-2	CYTO	W5PRJ3	HYCC1	6.68E-03	0.000
-2	CYTO	W5NTA4	CGGBP1	3.25E-02	0.000
-2	CYTO	W5PQ00	DIABLO	3.10E-02	0.000
-2	CYTO	W5QIC3	PRUNE1	9.04E-06	0.000
-2	CYTO	W5Q7H5	HVCN1	4.05E-02	0.000
-2	CYTO	W5Q2I1	MKRN2	1.42E-02	0.003
-2	CYTO	W5PCY9		5.43E-03	0.009
-2	CYTO	W5QF96	INPP5B	3.54E-02	0.000
-2	CYTO	W5QGL7	RTF1	4.99E-02	0.004
-2	CYTO	W5PTU0	CTPS2	2.17E-02	0.000
-2	CYTO	W5QHP6	ARHGDIB	3.63E-05	0.000
-2	CYTO	W5PL67	SUCLG2	7.05E-04	0.000
-2	CYTO	W5PHE6	DDI2	1.30E-03	0.000
-2	CYTO	W5NV31	COPS3	1.50E-03	0.000
-2	CYTO	Q2TCH3	ACLY	5.26E-04	0.000
-2	CYTO	W5PX94	CELF2	1.88E-02	0.000
-2	CYTO	W5PQ04	DEF6	1.76E-02	0.000
-2	CYTO	W5Q3M9	SEPTIN6	3.05E-02	0.000
-2	CYTO	W5Q591	CDC42SE2	4.59E-02	0.000
-2	CYTO	W5PXJ6	ANKRD13A	9.27E-03	0.000
-2	CYTO	W5PIP3	USP47	5.08E-02	0.000
-2	CYTO	W5Q9L9	SART3	3.08E-02	0.000
-2	CYTO	W5Q8W8	H3F3A LOC101108931	3.50E-05	0.000
-2	CYTO	W5QJ62	ACTN1	3.98E-02	0.000
-2	CYTO	W5PWX7	RABGAP1	6.50E-03	0.000
-2	CYTO	W5Q1F2		3.81E-02	0.000
-2	CYTO	W5QDF0	NRBP1	5.41E-02	0.000
-2	CYTO	W5PYM3	CAAP1	8.85E-03	0.001
-2	CYTO	W5PDB0	TRIM58	2.66E-02	0.000
-2	CYTO	W5PJA0	CUTA	5.19E-02	0.000
-2	CYTO	W5PX34	LYPLA1	2.04E-02	0.000
-2	CYTO	W5QD48	TAGLN3	9.40E-03	0.000
-2	CYTO	W5PEE3	FNBP1	7.01E-03	0.000
-2	CYTO	W5QH87	MFF	4.12E-03	0.000
-2	CYTO	W5NY53	MPI	8.31E-03	0.000
-2	CYTO	W5PJJ5	PGM2L1	2.75E-03	0.000
-2	CYTO	W5PM99		1.29E-02	0.000
-2	CYTO	W5Q2Y3	PLEKHB2	2.12E-02	0.000
-2	CYTO	W5PL66	PPA2	1.11E-02	0.000
-2	CYTO	W5P935	APMAP	1.87E-02	0.000
-2	CYTO	W5P098	CORO1A	2.97E-04	0.000
-2	CYTO	W5Q8V5	RBX1	3.99E-02	0.000
-2	CYTO	W5QI77	AK2	3.70E-03	0.000
-2	CYTO	W5PLJ8	ARL6IP1	5.29E-02	0.000
-2	CYTO	W5NZU5		8.17E-03	0.000
-2	CYTO	W5PMY9		3.79E-02	0.000
-2	CYTO	W5PWY1	BAG5	1.24E-02	0.000
-2	CYTO	W5PLD5	CNN2	3.97E-03	0.000
-2	CYTO	W5PCB1	DNAJC9	3.70E-02	0.000
-2	CYTO	W5PEW2	USP48	4.25E-02	0.000
-2	CYTO	W5PTZ8	LOC101114959	3.96E-03	0.000
-2	CYTO	W5NW82	NAP1L4	5.64E-03	0.000
-2	CYTO	W5PQI0	PXN	3.07E-03	0.000
-2	CYTO	W5NPT4	LOC101116298	1.73E-02	0.000
-2	CYTO	W5NUT8	PIP4K2A	1.65E-02	0.000
-2	CYTO	W5QIK2	RFX5	3.80E-02	0.000
-2	CYTO	W5PJF2	EXT1	1.46E-02	0.000
-2	CYTO	C8BKD4	CBX5	2.29E-02	0.000
-2	CYTO	W5QAM4	CAMK2D	2.87E-02	0.000
-2	CYTO	W5Q1S6	EML3	3.11E-02	0.000
-2	CYTO	W5PXS7	DAPP1	4.59E-02	0.000
-2	CYTO	W5NUK5	HAT1	1.38E-02	0.000
-2	CYTO	W5NVS5	CRNKL1	2.69E-02	0.000

Supplementary Table 18: Differentially abundant proteins (DAPs) of M2 relative to Mo and their cellular location.

FoldChange	Location	Protein IDs		P-value	FDR
-2	CYTO	W5PAI1		1.52E-02	0.000
-2	CYTO	W5PNL4	NCBP1	2.36E-02	0.000
-2	CYTO	W5P8H4	DCTPP1	3.84E-02	0.000
-2	CYTO	W5QIY4	ARID4A	1.95E-02	0.000
-2	CYTO	W5QGW0	PPIH	2.68E-02	0.000
-2	CYTO	W5PFY8	MTSS1	5.61E-02	0.000
-2	CYTO	W5Q9C5	LNPEP	1.89E-02	0.000
-2	CYTO	W5NWM7	GSTZ1	5.57E-03	0.000
-2	CYTO	W5QCZ0	MPST	2.93E-04	0.000
-2	CYTO	W5QEP1	POGLUT1	2.08E-02	0.000
-2	CYTO	W5PTM9	UBE2V2	1.50E-05	0.000
-2	CYTO	W5Q4T7		4.18E-02	0.000
-2	CYTO	W5PUS0	CUL4B	2.17E-02	0.000
-2	CYTO	W5PTW9	LRRC32	5.04E-02	0.000
-2	CYTO	W5P631	KLC4	5.39E-04	0.000
-2	CYTO	W5PSX3	DARS2	1.20E-02	0.000
-2	CYTO	W5NRJ3		2.29E-02	0.000
-2	CYTO	W5QCS1	DDX23	3.61E-03	0.000
-2	CYTO	W5PU89	ALDH16A1	3.51E-02	0.000
-2	CYTO	W5P0J5	ZNF22	2.52E-02	0.000
-2	CYTO	W5P4C5	RBM42	4.96E-03	0.000
-2	CYTO	W5PU02	MVD	2.98E-02	0.000
-2	CYTO	W5QH77	USP39	3.75E-02	0.000
-2	CYTO	W5NVL4		3.39E-02	0.000
-2	CYTO	W5QH64	SNAP23	6.69E-03	0.000
-2	CYTO	W5P225	PDCD5	1.68E-02	0.000
-2	CYTO	W5NRI1		1.73E-03	0.000
-2	CYTO	W5P669		1.49E-02	0.000
-2	CYTO	W5P1E2	CNST	1.04E-02	0.000
-2	CYTO	W5Q768	CDS2	2.53E-02	0.000
-2	CYTO	W5PY64	LOC101106288	1.86E-02	0.000
-2	CYTO	W5PAP1	TERF2IP	1.75E-02	0.000
-2	CYTO	W5PLB1	CLIC5	7.63E-03	0.000
-2	CYTO	W5PI68	TBCK	4.35E-02	0.000
-2	CYTO	W5QIH2	TMOD3	1.11E-03	0.000
-2	CYTO	W5PLK6	SHOC2	9.99E-03	0.000
-2	CYTO	B2LU20	SFXN1	2.67E-02	0.000
-2	CYTO	W5PR51	CPNE3	4.87E-02	0.000
-2	CYTO	W5P8W5		2.81E-02	0.000
-2	CYTO	W5PKD2	GCDH	2.67E-02	0.000
-2	CYTO	W5PA22	ACOT8	1.75E-03	0.000
-2	CYTO	W5QD23	LANCL1	1.25E-02	0.000
-2	CYTO	W5NQH2	CHMP6	4.19E-02	0.000
-2	CYTO	W5PWH9	PRKACB	2.11E-04	0.000
-3	CYTO	W5P4E2	PBLD	3.17E-02	0.000
-3	CYTO	W5PSM6	HABP2	2.22E-02	0.000
-3	CYTO	W5P6L3	KAT7	1.90E-02	0.000
-3	CYTO	W5PK38	VASP	3.52E-02	0.000
-3	CYTO	W5Q2W6	PPFIBP2	3.80E-02	0.000
-3	CYTO	W5P668		4.24E-02	0.000
-3	CYTO	W5PVX3	PRKG1	3.54E-02	0.000
-3	CYTO	W5PRR5	LTA4H	1.02E-04	0.000
-3	CYTO	W5PAL2	LOC101105297	4.38E-02	0.000
-3	CYTO	W5PSA9	VPS13A	2.39E-02	0.000
-3	CYTO	W5PHI7	LOC101116892	2.75E-02	0.000
-3	CYTO	W5QFT3		2.64E-02	0.000
-3	CYTO	W5Q6T8	SLAIN2	2.22E-02	0.000
-3	CYTO	W5QHX6	ANP32E	3.24E-02	0.000
-3	CYTO	W5PQ55		2.51E-02	0.000
-3	CYTO	W5QFQ8	LOC101111915	2.34E-03	0.000
-3	CYTO	W5Q9N1	LPCAT2	2.04E-02	0.000
-3	CYTO	W5PZC3	STAMBPL1	1.84E-02	0.000
-3	CYTO	W5PCJ3	LOC101112936	1.86E-02	0.000

Supplementary Table 18: Differentially abundant proteins (DAPs) of M2 relative to Mo and their cellular location.

FoldChange	Location	Protein IDs		P-value	FDR
-3	CYTO	W5QFM2		2.96E-03	0.000
-3	CYTO	W5Q1B3	HMGB2	1.55E-02	0.000
-3	CYTO	W5QB24	ANP32A	7.60E-03	0.000
-3	CYTO	W5P671	DDAH2	1.04E-02	0.010
-3	CYTO	W5P582	ACP1	1.76E-02	0.000
-3	CYTO	W5NXN2		1.10E-02	0.000
-3	CYTO	Q09YJ2	TES	4.63E-03	0.000
-3	CYTO	W5PBZ1	PCIF1	3.65E-02	0.000
-3	CYTO	W5PZP8	FIS1	3.61E-03	0.000
-3	CYTO	W5P0R4	CTIF	8.53E-03	0.000
-3	CYTO	W5P5I4	LAMTOR2	1.46E-02	0.000
-3	CYTO	W5QB58	AASS	4.50E-04	0.000
-3	CYTO	W5PG02	RASA3	1.51E-02	0.000
-3	CYTO	W5PZU8	ASAP2	2.88E-02	0.000
-3	CYTO	W5P2Z8	RGS14	3.66E-02	0.000
-3	CYTO	W5PSQ7		9.88E-03	0.000
-3	CYTO	W5PX55	LSM7	1.29E-02	0.000
-3	CYTO	W5P8I8	PITPNM2	6.54E-03	0.000
-3	CYTO	W5PIW6	PRTN3	5.02E-02	0.000
-3	CYTO	W5PIU9	ARMC6	4.31E-03	0.000
-3	CYTO	W5Q1F6	AIFM1	1.24E-03	0.000
-3	CYTO	W5NQ60	POLR2C	1.00E-02	0.000
-3	CYTO	W5PTQ0	RNGTT	1.83E-02	0.000
-3	CYTO	W5P590	OPTN	1.45E-02	0.000
-3	CYTO	W5Q231	FEN1	5.31E-02	0.000
-3	CYTO	W5P0K3	MBNL1	4.25E-04	0.000
-3	CYTO	W5Q6S0	LBR	7.16E-03	0.000
-3	CYTO	W5P7P8	GCA	1.89E-02	0.000
-3	CYTO	W5PRP1	SLC9A3R1	2.37E-03	0.000
-3	CYTO	W5NTV6		1.03E-02	0.000
-3	CYTO	W5QAQ9	SNU13	5.28E-02	0.000
-3	CYTO	W5PTB3	INPP5K	1.25E-04	0.000
-3	CYTO	W5PIC3		5.53E-02	0.000
-3	CYTO	W5Q297	PRKCA	6.09E-03	0.000
-3	CYTO	W5Q7N9	MTX2	2.35E-03	0.000
-3	CYTO	W5Q876	GPCPD1	1.46E-02	0.000
-3	CYTO	W5PAF9	KIF2A	9.00E-03	0.000
-3	CYTO	W5PYQ9	CPNE1	1.37E-02	0.000
-3	CYTO	W5QCG0		9.98E-03	0.000
-3	CYTO	W5P955	NT5C3A	2.87E-02	0.000
-3	CYTO	W5PZL2		4.57E-02	0.000
-3	CYTO	W5P2F2		2.50E-02	0.000
-3	CYTO	W5PWV6	UBE2B	2.49E-02	0.000
-3	CYTO	W5PGU9	PLCG2	5.28E-04	0.000
-3	CYTO	W5Q8H0	SSH1	3.00E-02	0.000
-3	CYTO	W5PTE9	COL6A1	9.56E-03	0.000
-3	CYTO	W5Q0B6	PPP1R12A	5.61E-04	0.000
-3	CYTO	W5NUX2		6.20E-03	0.000
-3	CYTO	W5NWU0	LOC101122262	2.27E-02	0.000
-3	CYTO	W5P0N1	DHX38	9.11E-03	0.001
-3	CYTO	W5NQH6	S100A9	3.48E-02	0.000
-3	CYTO	W5QBA4	RER1	7.21E-03	0.000
-3	CYTO	W5NWH6		1.86E-03	0.000
-3	CYTO	W5PYK8	VNN2	3.09E-03	0.000
-3	CYTO	W5PA61		2.02E-02	0.000
-3	CYTO	W5PEG1	CETN2	2.98E-02	0.000
-3	CYTO	W5NWX6	APOC3	4.40E-02	0.000
-3	CYTO	W5NS43	ALDH6A1	7.98E-04	0.000
-3	CYTO	W5PID6	CHMP5	4.02E-02	0.000
-3	CYTO	W5Q2C6		2.21E-02	0.000
-3	CYTO	W5Q9U3	CNP	6.52E-03	0.000
-3	CYTO	W5PXR8	POLR2J	1.05E-02	0.000
-3	CYTO	W5Q595	NIF3L1	1.96E-03	0.000

Supplementary Table 18: Differentially abundant proteins (DAPs) of M2 relative to Mo and their cellular location.

FoldChange	Location	Protein IDs		P-value	FDR
-3	CYTO	W5PAV0	STAP1	8.58E-03	0.000
-3	CYTO	W5NTW9	RIPK1	2.93E-02	0.000
-3	CYTO	W5Q9H1	ZYX	3.56E-02	0.000
-3	CYTO	W5QET9	PLA1A	1.17E-03	0.000
-3	CYTO	W5PVY6		5.97E-03	0.000
-3	CYTO	W5Q2W4	RAVER1	3.75E-02	0.000
-3	CYTO	W5NQJ0	LOC101103771	3.74E-02	0.000
-3	CYTO	W5Q3E4	NAAA	3.77E-03	0.000
-3	CYTO	W5P4F9	PRKAR2B	1.86E-02	0.000
-3	CYTO	C5IJ83	RAB8A	1.29E-02	0.000
-3	CYTO	W5PTQ7	TRAF3IP3	1.29E-02	0.000
-3	CYTO	W5PEA0	FGD3	3.61E-03	0.000
-3	CYTO	W5P874	ABHD16A	1.17E-02	0.000
-3	CYTO	W5PW39	LMNB2	2.07E-02	0.000
-3	CYTO	W5PP85	TBC1D10C	1.81E-02	0.000
-3	CYTO	W5PWS1	CD46	1.72E-03	0.000
-3	CYTO	W5Q0P0	GABPA	2.33E-02	0.000
-3	CYTO	W5Q8R0	FRYL	3.70E-02	0.000
-3	CYTO	W5PKS6	DEK	1.62E-02	0.000
-3	CYTO	W5PKA9	F5	7.25E-03	0.000
-3	CYTO	W5NTF8	SPNS1	4.80E-02	0.000
-3	CYTO	W5PQD8	C1QTNF3	1.25E-03	0.000
-3	CYTO	W5QH43	EIF4A2	1.41E-02	0.000
-4	CYTO	W5PI65	MAP4K2	1.24E-03	0.000
-4	CYTO	W5PEL2	OGN	1.92E-02	0.000
-4	CYTO	W5PKY9	MRCL3	2.47E-02	0.000
-4	CYTO	W5QHL6	ITM2C	2.62E-04	0.000
-4	CYTO	W5PGT0	MYH11	2.99E-02	0.000
-4	CYTO	W5P691	CNTRL	2.03E-02	0.000
-4	CYTO	W5PQT2	TPRKB	1.89E-02	0.000
-4	CYTO	C5ISA2	TUBA4A	7.83E-03	0.000
-4	CYTO	W5PZE4	GSTA1-1 GSTA2	1.03E-02	0.000
-4	CYTO	W5PJF3	PPIF	1.28E-02	0.000
-4	CYTO	W5PX46	LOC101122591	3.64E-02	0.000
-4	CYTO	W5Q3J8		6.54E-03	0.000
-4	CYTO	W5PVD4	AK3	1.74E-02	0.000
-4	CYTO	W5P6Q7	MPIG6B	4.33E-02	0.000
-4	CYTO	W5Q2V1		2.44E-03	0.000
-4	CYTO	W5PMJ7	NADK2	4.13E-03	0.000
-4	CYTO	W5P880	PRG4	1.10E-02	0.000
-4	CYTO	W5PEC3	ANXA6	4.82E-02	0.000
-4	CYTO	W5P3V8		1.64E-02	0.000
-4	CYTO	W5PRB3		5.09E-04	0.000
-4	CYTO	W5P6Z6	MAPRE2	5.44E-02	0.000
-4	CYTO	W5P7K2		1.02E-03	0.000
-4	CYTO	W5P795	LRBA	3.22E-02	0.000
-4	CYTO	W5PWL7	NAPSA	5.59E-04	0.000
-4	CYTO	W5Q6F1		2.85E-02	0.000
-4	CYTO	W5P9M9	LOC101103862	1.80E-02	0.000
-4	CYTO	W5NZT7	NMRAL1	4.20E-02	0.000
-4	CYTO	W5PDQ3	SAFB2	4.39E-03	0.000
-4	CYTO	W5QCH5	WDR47	7.79E-04	0.000
-4	CYTO	W5PHN9	F11R	4.86E-02	0.000
-4	CYTO	W5P2J9		2.38E-02	0.000
-4	CYTO	W5PK79	VWA5A	3.17E-03	0.000
-4	CYTO	W5PRC8	IKZF3	4.28E-02	0.000
-4	CYTO	W5PT20	ZC3H18	5.01E-03	0.000
-4	CYTO	W5P432	NIPSNAP1	3.43E-03	0.000
-4	CYTO	W5PDD5	STAG1	1.61E-02	0.000
-4	CYTO	W5PTN4	TMPO	5.12E-02	0.000
-4	CYTO	W5NYC7	DQA	4.04E-02	0.000
-4	CYTO	W5QI61	CDKN1B	4.63E-04	0.000
-4	CYTO	W5NTQ3	H3-4	3.62E-05	0.000

Supplementary Table 18: Differentially abundant proteins (DAPs) of M2 relative to Mo and their cellular location.

FoldChange	Location	Protein IDs		P-value	FDR
-4	CYTO	W5Q7L1	CNN3	5.34E-03	0.000
-4	CYTO	W5PWH2	MYH14	1.42E-02	0.000
-4	CYTO	W5NSJ5	OXSR1	2.36E-02	0.000
-4	CYTO	W5QEK8	EHD3	3.85E-03	0.000
-4	CYTO	W5NXY7	LOC101117960	4.50E-02	0.000
-4	CYTO	W5NSC5		1.40E-02	0.000
-4	CYTO	W5PTS4	LOC101114275	4.47E-03	0.000
-4	CYTO	W5PKQ2	PRXL2A	5.33E-02	0.000
-4	CYTO	W5PXT1	MYO1G	1.61E-02	0.000
-4	CYTO	W5QEL7	NDRG2	1.18E-04	0.008
-4	CYTO	W5QEC3	METTL3	4.26E-03	0.000
-4	CYTO	P29701	AHSG FETUA	1.70E-02	0.000
-4	CYTO	W5NTL4	EVL	2.37E-02	0.000
-4	CYTO	W5PV67	LIMD2	4.78E-03	0.000
-4	CYTO	W5PIN7		2.48E-02	0.000
-4	CYTO	W5QHW2	DAPK2	3.56E-02	0.000
-4	CYTO	W5Q059	SASH3	3.34E-03	0.000
-4	CYTO	W5PKR2	MX1	3.08E-03	0.000
-5	CYTO	W5QCW9	TST	5.35E-02	0.000
-5	CYTO	W5PC20	VPS37B	1.03E-03	0.000
-5	CYTO	W5PMH6	LCN2	4.97E-02	0.000
-5	CYTO	W5PHM1	ALOX12	4.42E-02	0.000
-5	CYTO	W5P7W5	LOC101113516	1.75E-02	0.000
-5	CYTO	W5Q2G2	IL16	2.84E-03	0.000
-5	CYTO	W5PU80	MS4A1	1.03E-02	0.000
-5	CYTO	W5P4U4	GRK6	5.76E-04	0.000
-5	CYTO	W5PRM0	GMPR	2.67E-02	0.000
-5	CYTO	C0IZ95	RAB27A	2.98E-03	0.000
-5	CYTO	W5P340	SOD2	2.03E-03	0.000
-5	CYTO	W5Q5M7	SLC44A2	1.63E-03	0.000
-5	CYTO	W5Q1G8	LOC101120875	3.43E-02	0.000
-5	CYTO	W5PBW1	SDS	1.89E-02	0.000
-5	CYTO	W5P6T0		5.81E-04	0.001
-5	CYTO	W5PJP5	RGS18	2.42E-02	0.000
-5	CYTO	W5PFJ0	VCL	1.57E-02	0.000
-5	CYTO	C5IS96	LCAT	5.11E-03	0.000
-5	CYTO	W5NR20	LOC101109940	2.51E-02	0.010
-5	CYTO	W5PE90	ITPR1	2.15E-02	0.000
-5	CYTO	W5PC51	TGFB1	2.57E-02	0.000
-5	CYTO	W5NV79	NUP210	1.67E-02	0.000
-5	CYTO	P47843	SLC2A3 GLUT3	1.28E-02	0.000
-5	CYTO	W5QFP0	THBS1	1.24E-02	0.000
-5	CYTO	W5NWB0		3.92E-04	0.000
-5	CYTO	W5PNC4	JPT1	2.20E-03	0.000
-5	CYTO	W5PDP2	CD55	3.55E-04	0.000
-5	CYTO	W5P9Q0	PI4K2B	5.92E-04	0.002
-5	CYTO	W5PV80	BANK1	1.71E-02	0.000
-5	CYTO	W5PYX0		7.83E-03	0.000
-5	CYTO	W5PFI6	RASGRP2	1.52E-03	0.000
-6	CYTO	W5PRY2	ACAP1	2.02E-02	0.000
-6	CYTO	W5QAK3	MMRN1	3.02E-02	0.000
-6	CYTO	W5QFP2	LOC101109397	4.25E-03	0.000
-6	CYTO	W5PXV3	CCN2	1.60E-02	0.000
-6	CYTO	W5PFP1	LTF	3.29E-02	0.000
-6	CYTO	W5PFI7	VCL	2.84E-02	0.000
-6	CYTO	W5P7C7	CD180	3.53E-02	0.000
-6	CYTO	W5Q878	PRKCB	7.49E-04	0.000
-6	CYTO	P80190		2.76E-03	0.000
-6	CYTO	P79362	CATHL2 BAC5	1.40E-02	0.000
-6	CYTO	W5PZG0	ADD1	7.36E-03	0.000
-6	CYTO	W5P4F5	RAB27B	5.11E-02	0.000
-6	CYTO	W5PGG6	MTMR1	8.66E-04	0.000
-6	CYTO	W5PY97	SVIL	1.19E-02	0.000

Supplementary Table 18: Differentially abundant proteins (DAPs) of M2 relative to Mo and their cellular location.

FoldChange	Location	Protein IDs		P-value	FDR
-6	CYTO	W5Q9B1	ATP2A3	4.84E-04	0.000
-6	CYTO	W5PM33	SELP	2.92E-02	0.000
-6	CYTO	W5Q3B8		1.10E-02	0.000
-6	CYTO	W5Q9S4	ING3	5.93E-03	0.001
-6	CYTO	W5PLZ3	PACSIN1	6.74E-03	0.000
-6	CYTO	W5PHR3	ADD3	9.46E-03	0.000
-6	CYTO	P54230	CATHL1A	4.38E-03	0.000
-6	CYTO	W5P4L3	AVIL	2.35E-02	0.000
-6	CYTO	W5P815	SEPTIN1	1.57E-02	0.000
-6	CYTO	P14639	ALB	5.62E-03	0.000
-7	CYTO	W5P733	PDLIM1	3.62E-02	0.000
-7	CYTO	W5NUE6	PLEKHA2	1.71E-04	0.000
-7	CYTO	W5QGQ3	LOC443320	4.10E-03	0.000
-7	CYTO	W5Q0K9	ABLIM1	3.26E-03	0.000
-7	CYTO	W5PQI3	ITGB3	8.26E-03	0.000
-7	CYTO	W5P3Y9	CD22	8.06E-03	0.000
-7	CYTO	W5NT35	LOC443162	8.44E-03	0.000
-7	CYTO	W5Q5A6	FGG	1.16E-02	0.000
-7	CYTO	W5PL70	PDCD4	9.33E-04	0.000
-8	CYTO	W5NQ46	FGB	1.54E-02	0.000
-8	CYTO	W5PHQ0	MPO	2.10E-03	0.000
-8	CYTO	W5PP64	FHL1	1.46E-03	0.000
-8	CYTO	W5NXW9		1.56E-04	0.000
-8	CYTO	W5PEX2	GPATCH8	2.60E-03	0.000
-8	CYTO	W5PF87	ALOX15	2.50E-02	0.000
-9	CYTO	W5Q2S8	MYL9	3.12E-03	0.000
-9	CYTO	W5NZX9	SPTBN1	6.73E-03	0.000
-9	CYTO	W5Q3I7	TUBB1	1.57E-03	0.000
-9	CYTO	W5Q5H8	FGA	4.70E-03	0.000
-9	CYTO	W5PJ75	SPTAN1	7.48E-04	0.000
-10	CYTO	W5QIK8	SELENBP1	3.67E-03	0.000
10	SEC	P83205	CTSB	4.22E-03	0.000
9	SEC	W5NWF5	RARRES1	5.43E-04	0.000
9	SEC	W5PDH4	MMP9	4.27E-05	0.000
9	SEC	Q29524	LPL	1.29E-04	0.000
9	SEC	W5PUH5	LGMN	9.00E-06	0.000
8	SEC	W5PF33	GM2A	1.22E-04	0.000
8	SEC	W5QI70	CTSS	2.52E-03	0.000
8	SEC	W5P1H0	CTSC	8.33E-03	0.000
8	SEC	W5P4C9	MMP12	5.77E-04	0.000
8	SEC	W5QGG0	TFRC	9.02E-04	0.000
8	SEC	W5PBM9	SCPEP1	6.78E-05	0.000
7	SEC	W5NSH8	NPC2	2.63E-03	0.000
7	SEC	W5PBS4	LRP1	3.27E-03	0.000
7	SEC	W5PJS4	EMILIN2	3.20E-05	0.000
7	SEC	W5P3Q3	LOC100101238	1.77E-03	0.000
7	SEC	W5QI78	CTSK	6.98E-05	0.000
7	SEC	W5PE92	GRN	6.14E-04	0.000
7	SEC	W5PAJ2	PSAP	6.16E-04	0.000
7	SEC	Q9MZS8	CTSD	7.29E-03	0.000
7	SEC	W5NUZ2	UTRN	3.52E-04	0.002
7	SEC	W5Q0F3	TGFBI	4.76E-04	0.000
7	SEC	W5PGV0	ITGAM	9.60E-04	0.000
6	SEC	W5PRI6	MRC1	2.93E-03	0.000
6	SEC	W5PDE5	LOC101120001	1.48E-02	0.000
6	SEC	W5Q9H0	MMP2	3.39E-04	0.000
6	SEC	W5NTX3		3.13E-04	0.000
6	SEC	W5QC89	HEXA	8.42E-04	0.000
6	SEC	W5Q2U7	PLEC	2.88E-02	0.000
6	SEC	W5QIW1	LGALS3	2.15E-03	0.000
6	SEC	W5QDI7	CSF1	2.15E-04	0.000
6	SEC	W5NTD9	CHI3L1	1.70E-04	0.000
6	SEC	W5NX56	SPP1	1.08E-03	0.000

Supplementary Table 18: Differentially abundant proteins (DAPs) of M2 relative to Mo and their cellular location.

FoldChange	Location	Protein IDs		P-value	FDR
6	SEC	W5PRG8	CREG1	8.56E-03	0.000
6	SEC	Q6ECI6	ITGB2 CD18	8.47E-03	0.000
6	SEC	W5PCH3	SCIN	2.28E-02	0.000
6	SEC	W5NT95	ATP6AP2	3.34E-04	0.000
6	SEC	W5PAM4	CTSA	3.51E-04	0.000
6	SEC	W5NZX1		1.02E-03	0.000
6	SEC	W5Q0F1	LIPA	3.52E-03	0.000
6	SEC	W5PGS4	FABP5	2.26E-02	0.000
6	SEC	W5NY01		1.07E-02	0.000
6	SEC	W5Q989	PLOD1	3.52E-03	0.000
6	SEC	W5PS94	NUCB1	4.77E-03	0.000
6	SEC	A9YUY8	FABP4	2.63E-02	0.000
6	SEC	W5P2V3	PEPD	1.19E-02	0.000
6	SEC	W5QAQ4	NRP2	5.98E-04	0.000
5	SEC	W5NPK5	LOC443475	7.43E-04	0.000
5	SEC	W5P026	STAB1	3.54E-03	0.000
5	SEC	W5P8R4	CSF1R	1.36E-03	0.000
5	SEC	W5P3N6	LOC101112162	2.98E-02	0.000
5	SEC	W5QHL1	FCGR1A	2.22E-03	0.000
5	SEC	W5PMR2	MMP19	4.20E-03	0.000
5	SEC	W5NPP2	CPM	9.50E-04	0.000
5	SEC	W5PT76	GPNMB	1.21E-03	0.000
5	SEC	W5PXR1	ENPP1	2.62E-04	0.000
5	SEC	W5QHV3	FABP1	7.85E-04	0.000
5	SEC	W5PEY4	TCN2	6.42E-04	0.000
5	SEC	W5NUI3	TREM2	1.85E-03	0.000
5	SEC	W5QI35	IL1RN	3.90E-03	0.000
5	SEC	W5QH35	CAPG	4.24E-02	0.000
5	SEC	W5PTL2	CFP	1.15E-02	0.000
5	SEC	W5PZB0	APLP2	1.05E-03	0.000
5	SEC	W5Q754	TTN	5.45E-02	0.002
5	SEC	W5Q689	TPP1	1.08E-04	0.000
5	SEC	W5NTZ3	RENBP	8.61E-03	0.000
5	SEC	W5PG72	GOLM1	6.04E-05	0.000
5	SEC	W5NRI1		5.56E-03	0.000
5	SEC	W5PCC7	LOC101115509	6.02E-04	0.000
5	SEC	W5NTG6	TINAGL1	2.71E-04	0.000
5	SEC	W5QJA2	CD14	1.82E-04	0.000
5	SEC	W5NSK4		1.04E-03	0.000
5	SEC	W5QDG8	FN1	1.30E-04	0.000
5	SEC	W5PGF4	PLAUR	1.15E-02	0.000
5	SEC	W5PCE0	PLBD2	1.45E-02	0.000
5	SEC	W5Q1W2	SDCBP	4.30E-03	0.000
5	SEC	W5P0W4	SEMA7A	2.62E-03	0.000
5	SEC	W5QBV7	CD44	1.35E-02	0.005
4	SEC	W5PF73	PLAU	2.86E-03	0.000
4	SEC	W5P9G8	PLD3	1.88E-02	0.000
4	SEC	W5PS45	MAN2B1	4.75E-02	0.000
4	SEC	W5PF04	MAN1A1	2.50E-03	0.000
4	SEC	W5NU86	GLA	6.95E-04	0.000
4	SEC	W5Q3T9	PDCD6IP	1.24E-02	0.000
4	SEC	W5QBZ7	NAGA	9.68E-03	0.000
4	SEC	W5PGT0	MYH11	1.89E-03	0.002
4	SEC	W5PIC9		1.61E-03	0.000
4	SEC	W5P041	ADAM15	9.58E-05	0.000
4	SEC	W5QFU8	CD86	2.40E-02	0.000
4	SEC	W5PNP1	MFGE8	1.47E-03	0.000
4	SEC	W5NZ76	TIMP2	6.75E-03	0.000
4	SEC	W5P3C6	LOC101111906	2.98E-03	0.000
4	SEC	W5PH85	ITGAX	2.24E-02	0.000
4	SEC	W5PB21	PLA2R1	6.18E-03	0.000
4	SEC	W5Q233	VCAN	3.56E-02	0.000
4	SEC	W5PJR5	LOC101121216	2.66E-02	0.000

Supplementary Table 18: Differentially abundant proteins (DAPs) of M2 relative to Mo and their cellular location.

FoldChange	Location	Protein IDs		P-value	FDR
4	SEC	W5P887	CST3	1.49E-02	0.000
4	SEC	W5PIQ4	LOC101120093	6.79E-03	0.000
4	SEC	W5PFY5	ASAH1	2.43E-06	0.000
4	SEC	W5PSA3	TCN1	1.06E-03	0.000
4	SEC	W5NPM4	TEX15	1.10E-02	0.004
4	SEC	W5Q3U3	LOC101102156	1.35E-02	0.000
4	SEC	W5PKU3		3.99E-03	0.000
4	SEC	W5NUI6	SGSH	1.49E-02	0.000
4	SEC	Q10994	CSTB CST6	2.95E-02	0.000
4	SEC	W5NVW7	NAGLU	3.33E-03	0.000
4	SEC	W5PCM7	LOC101105937	1.09E-02	0.004
4	SEC	W5NW80	GAA	1.11E-02	0.002
4	SEC	W5Q1M0	GLB1	4.56E-03	0.000
4	SEC	W5P1W2	FOLR3	3.91E-03	0.009
3	SEC	W5QIR6	GATM	8.41E-03	0.000
3	SEC	W5P4A8	RNASET2	2.50E-03	0.000
3	SEC	W5Q799	GALNT6	9.27E-03	0.010
3	SEC	W5PCS7	CXCL16	2.39E-03	0.004
3	SEC	W5QFB2	OLFML3	2.75E-02	0.000
3	SEC	Q9MZA9	VIM	2.20E-02	0.000
3	SEC	W5NZ62	GNS	1.08E-02	0.000
3	SEC	W5P5W6	NDRG1	1.36E-02	0.000
3	SEC	W5QDG9	CCDC80	4.30E-02	0.000
3	SEC	W5QD11	SORT1	6.81E-03	0.000
3	SEC	W5QC34	MAN2A1	4.69E-03	0.000
3	SEC	W5PMM5	ERP44	2.07E-02	0.000
3	SEC	W5Q7S8	NRP1	1.27E-02	0.000
3	SEC	W5PC32	PGM3	7.93E-03	0.004
3	SEC	Q7M2U8	APOE	8.61E-03	0.000
3	SEC	W5PEE9	LAMP1	6.02E-03	0.006
3	SEC	W5QI36	HEBP1	5.50E-02	0.000
3	SEC	W5PGG5	CD84	5.06E-02	0.001
2	SEC	W5PWF2	ATP6V1H	5.02E-02	0.000
2	SEC	W5P3V8		4.20E-02	0.000
2	SEC	W5PUJ5	LAMP2	2.03E-02	0.000
2	SEC	W5NZ70	LGALS3BP	7.55E-03	0.000
2	SEC	W5P2K9	LMAN2	4.16E-02	0.000
2	SEC	W5PTS4	LOC101114275	3.76E-02	0.000
2	SEC	W5PV50	ADA2	4.19E-02	0.004
2	SEC	W5PZI1	LOC101113728	5.69E-03	0.000
-2	SEC	W5P8Y7		1.36E-02	0.004
-2	SEC	W5QFH1	ACTC1	2.36E-02	0.000
-2	SEC	P60713	ACTB	2.66E-02	0.000
-2	SEC	W5Q2I7	USO1	3.89E-02	0.000
-2	SEC	W5Q2D9	RAB5A	5.05E-02	0.000
-2	SEC	W5P4J1	VAV1	4.26E-02	0.000
-2	SEC	W5NSM1	ACTR1A	3.02E-02	0.000
-2	SEC	W5NVQ4	CAND1	1.21E-02	0.000
-2	SEC	Q28554	GAPDH G3PDH GAPD	5.40E-02	0.000
-2	SEC	W5Q1I6	IMPDH2 IMPDH	3.23E-02	0.000
-2	SEC	C5IWU0	ARF1 LOC101123118	2.77E-02	0.000
-2	SEC	W5PQ76	SRI	1.49E-02	0.000
-3	SEC	W5QHL5	CAB39	1.79E-02	0.000
-3	SEC	P09670	SOD1	1.52E-02	0.006
-3	SEC	W5PFV5	NPEPPS	2.50E-02	0.000
-3	SEC	W5Q121		3.40E-02	0.000
-3	SEC	W5Q9X5	MAP2K1	2.37E-02	0.000
-3	SEC	W5Q1I8	PPM1F	1.46E-02	0.000
-3	SEC	W5P2J9		3.51E-03	0.000
-3	SEC	Q09YJ2	TES	1.08E-03	0.000
-3	SEC	W5P5F6	TBCB	4.18E-02	0.005
-3	SEC	W5QCX9	TTLL12	4.96E-02	0.000
-3	SEC	W5PTM9	UBE2V2	2.81E-03	0.000

Supplementary Table 18: Differentially abundant proteins (DAPs) of M2 relative to Mo and their cellular location.

FoldChange	Location	Protein IDs		P-value	FDR
-3	SEC	W5Q5A6	FGG	3.20E-02	0.000
-3	SEC	W5NVC9	RAC1	3.90E-02	0.000
-3	SEC	W5PLD5	CNN2	7.81E-03	0.000
-3	SEC	W5PKR1		3.05E-02	0.004
-3	SEC	W5PSZ3	JARID2	5.18E-02	0.005
-3	SEC	W5NSV5	ITGA6	6.99E-03	0.000
-3	SEC	W5QCL5	CSNK2A1	2.06E-02	0.000
-3	SEC	W5PE01	TAGLN2	2.15E-02	0.000
-3	SEC	W5PZ86	SEPTIN8	3.24E-02	0.000
-3	SEC	W5PVT6	UBA1	4.43E-02	0.000
-3	SEC	W5NW82	NAP1L4	2.40E-02	0.000
-3	SEC	W5P8B4	TRAPPC8	2.39E-02	0.006
-4	SEC	W5Q3E3	RAB2A	5.70E-04	0.000
-4	SEC	W5P5A0	FLNA	2.60E-02	0.000
-4	SEC	W5P4F9	PRKAR2B	1.96E-02	0.006
-4	SEC	W5P6X5	STMN1	2.52E-02	0.000
-4	SEC	W5P889	SEPTIN9	2.61E-02	0.000
-4	SEC	W5QI99	NEDD4	2.97E-03	0.000
-4	SEC	W5PQK3	PFKL	1.28E-02	0.000
-4	SEC	W5P5C4	NARS1	4.73E-03	0.000
-4	SEC	C5IJA0	RAN	1.39E-02	0.000
-4	SEC	C5ISA2	TUBA4A	1.72E-02	0.000
-4	SEC	W5P409	FERMT3	2.40E-02	0.000
-4	SEC	W5QHS2	MGP	1.44E-03	0.000
-4	SEC	W5P4C7	SEPTIN7	2.51E-02	0.000
-4	SEC	W5PHU7	UNC13D	5.61E-03	0.000
-4	SEC	W5QJ62	ACTN1	7.07E-03	0.000
-4	SEC	W5NQK6	LIMS1	5.18E-03	0.000
-4	SEC	W5PM33	SELP	4.19E-02	0.000
-4	SEC	W5P538	CD93	8.85E-03	0.000
-4	SEC	W5NRD9		2.88E-03	0.002
-4	SEC	W5P7E2	PPA1	2.53E-02	0.000
-4	SEC	W5QGT4		2.43E-02	0.000
-4	SEC	Q5MIB6	PYGB	1.50E-02	0.000
-4	SEC	W5QF71	PLEK	3.10E-03	0.000
-4	SEC	Q2TCH3	ACLY	3.31E-02	0.000
-4	SEC	W5PMQ9	SAE1	4.29E-03	0.000
-4	SEC	W5P733	PDLIM1	3.55E-02	0.000
-4	SEC	W5PWG1	PFKP	7.77E-03	0.000
-4	SEC	W5PTZ9	LOC101120877	5.44E-03	0.000
-4	SEC	W5PQI3	ITGB3	1.92E-03	0.000
-5	SEC	W5Q6U0	FASN	3.64E-03	0.000
-5	SEC	W5PIJ6	PTPN11	1.99E-02	0.000
-5	SEC	W5QFQ8	LOC101111915	1.95E-02	0.000
-5	SEC	W5P0V5	RAB11B	4.09E-03	0.000
-5	SEC	W5Q3B7	PDE5A	2.82E-02	0.000
-5	SEC	W5QD96	PARVB	5.08E-03	0.000
-5	SEC	W5PY17	STX7	9.37E-03	0.000
-5	SEC	W5PE27	ESD	5.38E-04	0.000
-5	SEC	W5NXW9		4.99E-04	0.000
-5	SEC	W5Q5H8	FGA	3.03E-02	0.000
-5	SEC	W5Q731	ILK	2.66E-02	0.000
-5	SEC	W5PRP1	SLC9A3R1	8.67E-05	0.000
-5	SEC	W5PX84	CCDC171	3.08E-04	0.005
-5	SEC	W5PK85	EML2	1.36E-02	0.000
-6	SEC	W5PRF0	SUB1	4.11E-04	0.000
-6	SEC	W5QEK8	EHD3	1.32E-03	0.000
-6	SEC	W5P6Z6	MAPRE2	4.46E-03	0.000
-6	SEC	W5PRR5	LTA4H	1.24E-03	0.000
-6	SEC	W5PQK6	TLN1	3.63E-02	0.000
-6	SEC	W5PEX2	GPATCH8	1.45E-03	0.000
-6	SEC	W5PNU4	LOC101111106	9.26E-03	0.000
-6	SEC	W5PG95	HSPA1A	4.93E-03	0.000

Supplementary Table 18: Differentially abundant proteins (DAPs) of M2 relative to Mo and their cellular location.

FoldChange	Location	Protein IDs		P-value	FDR
-6	SEC	W5NUT8	PIP4K2A	1.02E-03	0.000
-6	SEC	W5P4L3	AVIL	1.57E-03	0.000
-6	SEC	W5PH15	RSU1	6.54E-03	0.000
-7	SEC	W5PD82	CALD1	1.15E-03	0.000
-7	SEC	W5Q2S8	MYL9	1.96E-03	0.000
-7	SEC	W5QFM1		4.57E-02	0.000
-8	SEC	W5PP64	FHL1	1.88E-03	0.000
-8	SEC	W5PFI7	VCL	4.47E-04	0.000
-8	SEC	W5PFJ0	VCL	1.19E-04	0.000
-8	SEC	W5QIK8	SELENBP1	5.77E-04	0.000
-9	SEC	W5PK38	VASP	1.58E-03	0.000
-9	SEC	W5Q9H1	ZYX	8.16E-03	0.000
-11	SEC	W5Q3I7	TUBB1	3.51E-03	0.000

M2, anti-inflammatory macrophages activated with M-CSF/ IL-4 and M0, Monocytes at 3 hours. Differentially abundant proteins (DAPs) were identified using a threshold of false discovery rate (FDR, q-value) ≤ 0.05 and absolute fold change ≥ 2. Red-highlighted cells indicate shared differentially abundant proteins (DAPs) between the cell lysate and secretome compartments in M2 relative to M0, whereas non-highlighted cells represent compartment-specific DAPs unique to either the whole cell lysate (CYTO) or secretome (SEC).

Supplementary Table 19: Shared and unique differentially abundant proteins (DAPs) between M1 and M2 relative to Mo and their cellular location.

FoldChange	Location	Protein IDs	Gene Names	P-value	FDR	Cell type
10	CYTO	W5PEB0	FABP7	1.08E-07	0	M1_Mo
8	CYTO	W5NU86	GLA	1.03E-04	0	M1_Mo
8	CYTO	W5P530	LOC101104705	8.77E-04	0	M1_Mo
8	CYTO	W5PAM4	CTSA	5.94E-07	0	M1_Mo
8	CYTO	W5PAC2	LOC101105044	1.79E-04	0	M1_Mo
8	CYTO	W5PCH3	SCIN	6.39E-04	0	M1_Mo
7	CYTO	W5PF33	GM2A	2.98E-06	0	M1_Mo
7	CYTO	W5PU22	MCUR1	2.40E-08	0	M1_Mo
7	CYTO	A9YUY8	FABP4	5.31E-04	0	M1_Mo
7	CYTO	W5NY01		5.84E-05	0	M1_Mo
7	CYTO	W5PRI6	MRC1	5.15E-06	0	M1_Mo
7	CYTO	W5P9J8	BLVRB	1.23E-04	0	M1_Mo
7	CYTO	W5PFV1	CTSL	2.41E-05	0	M1_Mo
7	CYTO	W5PT76	GPNMB	1.68E-05	0	M1_Mo
7	CYTO	P51977	ALDH1A1	9.70E-04	0	M1_Mo
7	CYTO	W5PIQ6	MSR1	1.35E-06	0	M1_Mo
7	CYTO	W5P9G8	PLD3	2.28E-03	0	M1_Mo
7	CYTO	W5PQR0	NIBAN2	1.61E-08	0	M1_Mo
7	CYTO	W5PJS4	EMILIN2	3.35E-06	0	M1_Mo
6	CYTO	W5P4C9	MMP12	3.14E-05	0	M1_Mo
6	CYTO	W5PZB2	CD68	2.18E-06	0	M1_Mo
6	CYTO	W5Q3H9	UPP1	2.22E-04	0	M1_Mo
6	CYTO	W5PKY1	HNMT	4.26E-05	0	M1_Mo
6	CYTO	W5Q233	VCAN	6.68E-06	0	M1_Mo
6	CYTO	W5NRS0		5.43E-06	0	M1_Mo
6	CYTO	W5QHL1	FCGR1A	1.49E-05	0	M1_Mo
6	CYTO	W5PBM9	SCPEP1	2.10E-04	0	M1_Mo
6	CYTO	W5PFY5	ASAH1	3.43E-04	0	M1_Mo
6	CYTO	W5P093	NQO1	1.10E-06	0	M1_Mo
6	CYTO	W5QHZ0	CA12	3.68E-05	0	M1_Mo
6	CYTO	W5PGH4	TNKS1BP1	2.28E-02	0	M1_Mo
6	CYTO	W5P1A5	GBA1	5.85E-06	0	M1_Mo
6	CYTO	W5P640	LMNA	2.76E-03	0	M1_Mo
6	CYTO	Q9MZS8	CTSD	6.47E-05	0	M1_Mo
6	CYTO	W5PCE0	PLBD2	2.63E-04	0	M1_Mo
6	CYTO	W5QFU8	CD86	8.39E-05	0	M1_Mo
6	CYTO	W5PDH4	MMP9	1.70E-05	0	M1_Mo
6	CYTO	W5NVW7	NAGLU	1.97E-04	0	M1_Mo
6	CYTO	W5Q263	ICAM1	6.54E-06	0	M1_Mo
6	CYTO	W5PUH5	LGMN	2.92E-04	0	M1_Mo
6	CYTO	W5P0K8	CLIC2	3.20E-03	0	M1_Mo
6	CYTO	W5P1H0	CTSC	3.86E-04	0	M1_Mo
6	CYTO	W5P2F1	FOLR2	7.71E-05	0	M1_Mo
6	CYTO	W5PI56	DAB2	2.48E-04	0	M1_Mo
6	CYTO	W5Q0A3	TLR2	1.58E-05	0	M1_Mo
6	CYTO	W5Q2Y1	PLXNC1	5.14E-04	0	M1_Mo

Supplementary Table 19: Shared and unique differentially abundant proteins (DAPs) between M1 and M2 relative to Mo and their cellular location.

FoldChange	Location	Protein IDs	Gene Names	P-value	FDR	Cell type
6	CYTO	P35623	SHMT1	8.91E-06	0	M1_Mo
6	CYTO	W5PTU7	CA2	1.27E-05	0	M1_Mo
6	CYTO	W5QBM4	ALCAM	3.10E-05	0	M1_Mo
6	CYTO	M4WG34	IL1b	3.13E-02	0	M1_Mo
6	CYTO	W5PK56	FCGR2B	6.95E-03	0	M1_Mo
5	CYTO	W5PBS4	LRP1	5.24E-05	0	M1_Mo
5	CYTO	W5PGC5	GALM	2.73E-04	0	M1_Mo
5	CYTO	W5QC89	HEXA	3.39E-06	0	M1_Mo
5	CYTO	W5Q940	SHTN1	2.26E-04	0	M1_Mo
5	CYTO	W5PAQ4	FUCA1	1.00E-05	0	M1_Mo
5	CYTO	W5PRG9	HAL	3.10E-07	0	M1_Mo
5	CYTO	W5P8H9	SGPL1	3.56E-05	0	M1_Mo
5	CYTO	W5PGF4	PLAUR	2.99E-03	0	M1_Mo
5	CYTO	W5PYW0	TCIRG1	8.76E-05	0	M1_Mo
5	CYTO	W5PE67		5.35E-04	0	M1_Mo
5	CYTO	W5PMT0	XDH	1.30E-03	0	M1_Mo
5	CYTO	W5Q612	TRPV2	8.23E-07	0	M1_Mo
5	CYTO	W5QBZ7	NAGA	3.87E-04	0	M1_Mo
5	CYTO	W5NYU9	MPP1	3.83E-06	0	M1_Mo
5	CYTO	W5QI70	CTSS	1.85E-04	0	M1_Mo
5	CYTO	W5NXM6	PTX3	7.86E-04	0	M1_Mo
5	CYTO	W5PV43	LRPAP1	4.68E-07	0	M1_Mo
5	CYTO	W5PZ47	CTSH	1.44E-02	0	M1_Mo
5	CYTO	W5PA90	AGA	3.08E-06	0	M1_Mo
5	CYTO	W5QCD6	IDH1	9.05E-08	0	M1_Mo
5	CYTO	W5QI00	LACTB	8.92E-06	0	M1_Mo
5	CYTO	W5PXR1	ENPP1	6.35E-03	0	M1_Mo
5	CYTO	W5QIW1	LGALS3	4.07E-04	0	M1_Mo
5	CYTO	W5PE73	SMPDL3A	1.64E-05	0	M1_Mo
5	CYTO	W5QCL8	NPL	3.94E-05	0	M1_Mo
5	CYTO	W5QI40	MYO1E	1.76E-04	0	M1_Mo
5	CYTO	W5PJV4	ASGR2	5.64E-05	0	M1_Mo
5	CYTO	W5P3H8	IGF2R	3.04E-04	0	M1_Mo
5	CYTO	W5Q5W2	LOC101110539	3.20E-06	0	M1_Mo
5	CYTO	W5Q9Y6	TCAF2	1.12E-05	0	M1_Mo
5	CYTO	W5NSH8	NPC2	1.37E-06	0	M1_Mo
5	CYTO	W5NUC8	ARMCX3	1.20E-05	0	M1_Mo
5	CYTO	W5NTZ3	RENBP	3.14E-05	0	M1_Mo
5	CYTO	W5QGG0	TFRC	1.46E-05	0	M1_Mo
5	CYTO	P79365	SLC2A1	1.71E-02	0.005179	M1_Mo
5	CYTO	W5PNY4	PDXK	1.17E-03	0	M1_Mo
5	CYTO	W5NUI6	SGSH	8.90E-05	0	M1_Mo
5	CYTO	W5Q6N3	LOC101115115	2.74E-04	0	M1_Mo
5	CYTO	W5NYL0	MAOA	5.75E-05	0	M1_Mo
5	CYTO	W5PDH7	NPC1	1.74E-06	0	M1_Mo
5	CYTO	W5PM91	SLC1A5	3.61E-02	0	M1_Mo

Supplementary Table 19: Shared and unique differentially abundant proteins (DAPs) between M1 and M2 relative to Mo and their cellular location.

FoldChange	Location	Protein IDs	Gene Names	P-value	FDR	Cell type
5	CYTO	W5QH35	CAPG	8.07E-06	0	M1_Mo
5	CYTO	W5Q7I8	TAX1BP3	6.17E-03	0	M1_Mo
5	CYTO	W5PVJ5	CD274	9.14E-06	0	M1_Mo
5	CYTO	G3M9U4	ACP5	2.36E-05	0	M1_Mo
5	CYTO	W5P150		7.22E-05	0.000221	M1_Mo
5	CYTO	W5P407		1.04E-03	0	M1_Mo
5	CYTO	W5PMX5	C5AR1	5.25E-04	0	M1_Mo
5	CYTO	W5PY08		5.18E-04	0	M1_Mo
5	CYTO	W5Q8J3	RRBP1	1.65E-03	0	M1_Mo
5	CYTO	W5QDF4	GSTM3	9.51E-04	0	M1_Mo
5	CYTO	W5P5W6	NDRG1	2.01E-03	0	M1_Mo
5	CYTO	W5NZJ7	FGR	2.30E-03	0	M1_Mo
5	CYTO	W5PXY4	CLEC5A	8.22E-03	0	M1_Mo
5	CYTO	W5PWX3	CRYL1	2.53E-05	0	M1_Mo
5	CYTO	W5P8M9		1.05E-04	0	M1_Mo
5	CYTO	P05028	ATP1B1	3.63E-04	0	M1_Mo
5	CYTO	W5PLB8	EPB41L3	1.04E-03	0	M1_Mo
4	CYTO	W5NQS7	IFNGR1	4.08E-05	0	M1_Mo
4	CYTO	W5PRG8	CREG1	3.31E-04	0	M1_Mo
4	CYTO	W5Q678	SEC24D	4.66E-04	0	M1_Mo
4	CYTO	W5PII2	ATG4C	1.80E-02	0	M1_Mo
4	CYTO	W5PAM2	RHBDF2	3.20E-02	0	M1_Mo
4	CYTO	W5NXJ0	CLEC4E	4.31E-03	0	M1_Mo
4	CYTO	W5Q9M9	GK	1.63E-03	0	M1_Mo
4	CYTO	W5NZ62	GNS	5.35E-04	0	M1_Mo
4	CYTO	W5P3Q3	LOC100101238	4.59E-03	0	M1_Mo
4	CYTO	W5PJR5	LOC101121216	2.72E-05	0.000204	M1_Mo
4	CYTO	P82197	PDXK	1.44E-03	0	M1_Mo
4	CYTO	W5NW80	GAA	7.61E-04	0	M1_Mo
4	CYTO	W5PIS6	NHLRC3	4.17E-04	0	M1_Mo
4	CYTO	W5NZV3	HMOX2	7.47E-03	0	M1_Mo
4	CYTO	W5NYE0	ATP6V0D1	5.51E-03	0	M1_Mo
4	CYTO	W5PNP1	MFGE8	8.75E-04	0	M1_Mo
4	CYTO	W5PTG5	ATP6V0D2	9.66E-03	0	M1_Mo
4	CYTO	W5NZK6	PLA2G15	8.65E-05	0	M1_Mo
4	CYTO	W5QHG1	EPS8	6.59E-03	0	M1_Mo
4	CYTO	W5PUL5	FCGRT	2.40E-03	0	M1_Mo
4	CYTO	W5PZG5	OCRL	7.78E-05	0	M1_Mo
4	CYTO	W5QEM8	LOC101111528	1.45E-05	0	M1_Mo
4	CYTO	W5P2V3	PEPD	6.88E-03	0	M1_Mo
4	CYTO	W5QHU8	FNDC3B	2.45E-05	0	M1_Mo
4	CYTO	W5QB71	AMDHD2	1.30E-03	0	M1_Mo
4	CYTO	W5PQ75	HSPH1	2.68E-03	0	M1_Mo
4	CYTO	W5NQG2	TBC1D2B	8.63E-05	0	M1_Mo
4	CYTO	W5QA16		8.44E-06	0	M1_Mo
4	CYTO	W5P6H9	ACP2	4.12E-03	0	M1_Mo

Supplementary Table 19: Shared and unique differentially abundant proteins (DAPs) between M1 and M2 relative to Mo and their cellular location.

FoldChange	Location	Protein IDs	Gene Names	P-value	FDR	Cell type
4	CYTO	W5P3L5	RNF13	4.75E-06	0	M1_Mo
4	CYTO	W5Q3U3	LOC101102156	9.03E-04	0	M1_Mo
4	CYTO	W5P539	TYROBP	8.91E-03	0	M1_Mo
4	CYTO	W5Q2R5	SDF2L1	8.72E-03	0	M1_Mo
4	CYTO	W5PKU3		5.16E-05	0	M1_Mo
4	CYTO	W5QBV7	CD44	1.05E-03	0	M1_Mo
4	CYTO	W5QFU4		2.09E-03	0	M1_Mo
4	CYTO	W5QI35	IL1RN	2.59E-02	0	M1_Mo
4	CYTO	W5P369	AP2A2	1.98E-02	0	M1_Mo
4	CYTO	W5Q9F0	LOC101102694	3.40E-03	0.001906	M1_Mo
4	CYTO	W5Q5C8	SOAT1	1.35E-05	0	M1_Mo
4	CYTO	W5PYH9		6.86E-03	0	M1_Mo
4	CYTO	W5NXH3	LOC101106542	5.98E-05	0	M1_Mo
4	CYTO	W5Q0F1	LIPA	3.77E-03	0	M1_Mo
4	CYTO	W5Q2U7	PLEC	1.06E-03	0	M1_Mo
4	CYTO	W5PUW2	IFI30	1.97E-02	0	M1_Mo
4	CYTO	C5IJ93	RAB9A	1.51E-06	0	M1_Mo
4	CYTO	W5Q2B3	SUSD5	2.21E-02	0.008553	M1_Mo
4	CYTO	W5P3S0		3.60E-06	0	M1_Mo
4	CYTO	W5NUJ5		8.68E-04	0	M1_Mo
4	CYTO	W5PWQ4	EPB41L2	4.47E-02	0	M1_Mo
4	CYTO	W5QI78	CTSK	1.53E-02	0	M1_Mo
4	CYTO	W5PHG1	CDA	4.30E-03	0	M1_Mo
4	CYTO	W5PEE9	LAMP1	5.26E-03	0	M1_Mo
4	CYTO	W5NU23	FUCA2	1.69E-05	0	M1_Mo
4	CYTO	W5P3H9	PICALM	2.43E-02	0	M1_Mo
4	CYTO	W5PBC0		1.45E-03	0	M1_Mo
4	CYTO	W5NTZ2	LOC101121821	2.46E-02	0	M1_Mo
4	CYTO	W5Q284	HAPLN1	7.97E-03	0	M1_Mo
4	CYTO	W5Q4U5	CPT1A	3.99E-04	0	M1_Mo
4	CYTO	W5PE92	GRN	4.06E-05	0	M1_Mo
4	CYTO	W5PBW1	SDS	1.72E-02	0	M1_Mo
4	CYTO	W5Q7R3	LOC101118736	3.96E-03	0	M1_Mo
4	CYTO	W5PGS4	FABP5	4.70E-05	0	M1_Mo
4	CYTO	W5P2W1	NEU1	4.72E-05	0	M1_Mo
4	CYTO	W5QHR5	PLEKHO2	6.37E-05	0	M1_Mo
4	CYTO	W5PKA1	LOC101116157	1.96E-03	0	M1_Mo
4	CYTO	W5QHF2	LOC114117536	1.12E-02	0	M1_Mo
4	CYTO	W5NZX4	KATNAL2	1.12E-02	0.003561	M1_Mo
4	CYTO	W5PY53	FTH1	1.44E-02	0	M1_Mo
4	CYTO	W5Q1W2	SDCBP	3.29E-02	0	M1_Mo
4	CYTO	P83205	CTSB	1.34E-03	0	M1_Mo
4	CYTO	W5NWX4		3.26E-03	0	M1_Mo
4	CYTO	W5Q5T7	ATP6V1C1	6.88E-03	0	M1_Mo
4	CYTO	W5PW33		9.37E-04	0	M1_Mo
4	CYTO	W5P340	SOD2	1.32E-02	0	M1_Mo

Supplementary Table 19: Shared and unique differentially abundant proteins (DAPs) between M1 and M2 relative to Mo and their cellular location.

FoldChange	Location	Protein IDs	Gene Names	P-value	FDR	Cell type
4	CYTO	W5PG10	PAPSS1	2.69E-03	0	M1_Mo
4	CYTO	W5NVR9	C21H11orf54	1.03E-05	0	M1_Mo
4	CYTO	W5QGV7		2.21E-04	0	M1_Mo
4	CYTO	W5P4X6	LOC101104287	2.41E-03	0	M1_Mo
4	CYTO	W5PBR7	P4HA1	7.81E-04	0	M1_Mo
4	CYTO	W5PGG5	CD84	5.65E-03	0	M1_Mo
4	CYTO	W5PXS1	RAB3IL1	4.05E-04	0	M1_Mo
4	CYTO	W5Q6C5	GGA2	1.34E-02	0	M1_Mo
4	CYTO	W5Q0Y4	ZFAND6	1.59E-02	0	M1_Mo
4	CYTO	W5QI36	HEBP1	2.95E-03	0	M1_Mo
4	CYTO	W5Q1M0	GLB1	1.78E-04	0	M1_Mo
4	CYTO	W5PNK3	ACAN	8.08E-05	0	M1_Mo
4	CYTO	W5NYK9	CALU	3.67E-02	0	M1_Mo
4	CYTO	W5Q3N1	CTSZ	1.21E-06	0	M1_Mo
4	CYTO	W5QEH0	TWF1	2.69E-04	0	M1_Mo
4	CYTO	W5P703	WFS1	9.28E-05	0	M1_Mo
4	CYTO	W5NX56	SPP1	1.16E-03	0	M1_Mo
4	CYTO	W5QEH8	CTTNBP2NL	3.38E-05	0	M1_Mo
4	CYTO	W5Q4B1	MYCBP2	9.91E-03	0	M1_Mo
4	CYTO	W5QBP6	GLUL	3.52E-03	0	M1_Mo
4	CYTO	W5Q0U0	P2RX4	4.88E-03	0	M1_Mo
4	CYTO	Q6XXL8	DYNLT3	9.29E-03	0	M1_Mo
4	CYTO	W5QBG8	PPFIA1	7.13E-04	0	M1_Mo
4	CYTO	W5PP04	GNG12	8.83E-03	0	M1_Mo
4	CYTO	W5P3N6	LOC101112162	6.21E-04	0	M1_Mo
4	CYTO	W5PTE6	CEMIP2	7.24E-04	0	M1_Mo
4	CYTO	W5QGT0	ATP13A3	3.85E-03	0	M1_Mo
4	CYTO	Q9MZD1	SLC17A5	9.12E-04	0	M1_Mo
4	CYTO	W5PGL9		3.27E-05	0	M1_Mo
4	CYTO	W5NUG3	GNPDA1	3.34E-02	0	M1_Mo
4	CYTO	W5NVV1		1.97E-02	0	M1_Mo
3	CYTO	W5Q3V0	IL18	4.41E-05	0	M1_Mo
3	CYTO	W5PG41	H6PD	5.41E-04	0	M1_Mo
3	CYTO	W5PZG8	PECAM1	4.28E-04	0	M1_Mo
3	CYTO	W5NYL7	MTHFD1L	9.97E-06	0	M1_Mo
3	CYTO	W5QG92	OSBPL11	7.58E-05	0	M1_Mo
3	CYTO	W5QGV5	DOCK10	3.82E-02	0	M1_Mo
3	CYTO	W5NUE3	PRDX1	1.42E-03	0	M1_Mo
3	CYTO	W5QBA2	ATRX	3.57E-02	0	M1_Mo
3	CYTO	W5QBR5	BMP2K	1.01E-02	0	M1_Mo
3	CYTO	W5NXT4		1.50E-02	0	M1_Mo
3	CYTO	W5Q8K4	SLC3A2	1.62E-02	0	M1_Mo
3	CYTO	W5PC32	PGM3	9.80E-04	0	M1_Mo
3	CYTO	W5QAU1		5.49E-02	0	M1_Mo
3	CYTO	W5P3C6	LOC101111906	2.61E-02	0	M1_Mo
3	CYTO	W5PEC0	PKIB	5.62E-03	0	M1_Mo

Supplementary Table 19: Shared and unique differentially abundant proteins (DAPs) between M1 and M2 relative to Mo and their cellular location.

FoldChange	Location	Protein IDs	Gene Names	P-value	FDR	Cell type
3	CYTO	W5PL89	GSR	5.77E-04	0	M1_Mo
3	CYTO	W5Q7X3	GNPNAT1	1.56E-02	0	M1_Mo
3	CYTO	W5PYW3	LOC101103238	3.76E-02	0	M1_Mo
3	CYTO	W5NTE2	PSMG4	1.77E-02	0	M1_Mo
3	CYTO	W5P4A8	RNASET2	2.56E-03	0	M1_Mo
3	CYTO	W5NZA6	RNF121	2.09E-04	0	M1_Mo
3	CYTO	W5NZ70	LGALS3BP	2.86E-02	0	M1_Mo
3	CYTO	W5QIC7	YBX3	4.19E-03	0	M1_Mo
3	CYTO	W5QCG9	SRXN1	6.25E-03	0	M1_Mo
3	CYTO	W5PMA0	AP2S1	1.24E-03	0	M1_Mo
3	CYTO	W5Q700	APPL2	1.09E-04	0	M1_Mo
3	CYTO	W5NX08	WASHC2C	2.49E-02	0	M1_Mo
3	CYTO	W5PF68	EMB	2.25E-04	0.000215	M1_Mo
3	CYTO	W5QCA6	UBE2F	1.91E-06	0	M1_Mo
3	CYTO	W5Q777	HS1BP3	7.19E-06	0	M1_Mo
3	CYTO	W5P4H0	DNASE1L1	1.81E-02	0	M1_Mo
3	CYTO	W5PVE3	LOC101115252	3.39E-02	0	M1_Mo
3	CYTO	W5P2F6	FAF1	1.07E-05	0	M1_Mo
3	CYTO	W5PGA9	NCSTN	1.34E-03	0	M1_Mo
3	CYTO	W5PFP5	PLIN3	9.34E-03	0	M1_Mo
3	CYTO	W5QG63	ZMPSTE24	1.28E-04	0	M1_Mo
3	CYTO	W5NU94	PSTPIP1	2.51E-03	0	M1_Mo
3	CYTO	W5Q7Z6	DIP2B	3.43E-04	0	M1_Mo
3	CYTO	W5Q6S3	WASHC4	4.39E-04	0	M1_Mo
3	CYTO	W5P536	TRIP10	8.64E-04	0	M1_Mo
3	CYTO	P04074	ATP1A1	2.84E-03	0	M1_Mo
3	CYTO	W5PFE6	ACOX1	1.19E-04	0	M1_Mo
3	CYTO	W5P2D9	SNX9	4.20E-02	0	M1_Mo
3	CYTO	W5PC82	ME1	3.55E-02	0	M1_Mo
3	CYTO	W5Q9U4	LOC101104050	1.00E-03	0	M1_Mo
3	CYTO	W5PC06	SIRPA	1.90E-02	0	M1_Mo
3	CYTO	W5NTW3	ITIH1	1.69E-02	0	M1_Mo
3	CYTO	W5PIE4	CLPTM1	5.41E-03	0	M1_Mo
3	CYTO	W5PEP1	ATP1A2	5.68E-03	0.000208	M1_Mo
3	CYTO	W5QBE4	FGL2	2.71E-02	0	M1_Mo
3	CYTO	W5PCK8	LOC100135455	3.80E-02	0	M1_Mo
3	CYTO	W5QFD0	RRAGC	5.97E-03	0	M1_Mo
3	CYTO	W5PDZ1		5.56E-04	0	M1_Mo
3	CYTO	W5P1Q0	AP1B1	1.47E-04	0	M1_Mo
3	CYTO	W5PZ94	ACO1	2.06E-02	0	M1_Mo
3	CYTO	W5QHX1	EIF5A2	1.28E-02	0	M1_Mo
3	CYTO	W5P3W6	OSBPL9	2.72E-04	0	M1_Mo
3	CYTO	W5PMU8	GAB1	1.54E-03	0	M1_Mo
3	CYTO	O18882	ATP6V0C	4.40E-03	0	M1_Mo
3	CYTO	W5Q5N6	BST-2B	5.07E-04	0	M1_Mo
3	CYTO	W5Q5P5	GGH	1.94E-03	0	M1_Mo

Supplementary Table 19: Shared and unique differentially abundant proteins (DAPs) between M1 and M2 relative to Mo and their cellular location.

FoldChange	Location	Protein IDs	Gene Names	P-value	FDR	Cell type
3	CYTO	W5Q1W7	PALLD	5.28E-04	0	M1_Mo
3	CYTO	W5PVC8	ERGIC3	8.48E-05	0	M1_Mo
3	CYTO	W5NRF7	MAP3K20	4.07E-04	0	M1_Mo
3	CYTO	W5PT36	RBM47	3.65E-05	0	M1_Mo
3	CYTO	W5NVV6	DNAJC3	7.28E-03	0	M1_Mo
3	CYTO	W5PN65	PI4K2A	1.41E-02	0	M1_Mo
3	CYTO	W5Q3Y3	SFXN3	2.65E-03	0	M1_Mo
3	CYTO	W5Q634	BLVRA	1.96E-02	0	M1_Mo
3	CYTO	W5QEE5	TRAPPC3	3.16E-02	0	M1_Mo
3	CYTO	W5P743	GLMP	3.47E-04	0	M1_Mo
3	CYTO	W5PJN7	HOMER3	4.59E-04	0	M1_Mo
3	CYTO	W5Q0G8	IMPA1	2.21E-03	0	M1_Mo
3	CYTO	W5PK00	HNRNPH2	3.48E-02	0	M1_Mo
3	CYTO	W5QGH9	WDR3	4.89E-02	0.000209	M1_Mo
3	CYTO	W5PAX1	GCLC	1.07E-02	0	M1_Mo
3	CYTO	W5PB38		1.36E-04	0	M1_Mo
3	CYTO	W5QAJ8	DESI1	1.60E-03	0	M1_Mo
3	CYTO	W5Q8G9	SPG21	1.92E-04	0	M1_Mo
3	CYTO	W5PQV2	LMBRD1	1.03E-02	0	M1_Mo
3	CYTO	W5QH13	VPS39	1.42E-04	0	M1_Mo
3	CYTO	W5P3I5	CNDP2	1.83E-04	0	M1_Mo
3	CYTO	W5Q318	CCNH	1.40E-02	0.004286	M1_Mo
3	CYTO	W5NRW1	FBXO6	1.41E-02	0	M1_Mo
3	CYTO	W5PH35	LOC101119706	1.21E-03	0	M1_Mo
3	CYTO	W5PJ40	EIF2AK2	4.02E-02	0	M1_Mo
3	CYTO	W5PGX8	DNAJC13	1.82E-02	0	M1_Mo
3	CYTO	W5PMX7		1.92E-02	0	M1_Mo
3	CYTO	W5PCI1	NLRP3	3.39E-04	0	M1_Mo
3	CYTO	W5Q7J0	APOB	4.85E-02	0	M1_Mo
3	CYTO	W5P3B0	INPPL1	3.92E-03	0	M1_Mo
3	CYTO	W5PM73	UAP1	4.13E-03	0	M1_Mo
3	CYTO	W5P164	LAMTOR1	1.06E-02	0	M1_Mo
3	CYTO	W5QDN8	SLC48A1	2.00E-04	0.000216	M1_Mo
3	CYTO	W5PCD0	FUBP3	8.35E-03	0	M1_Mo
3	CYTO	W5NQZ9	GSDMD	1.23E-03	0	M1_Mo
3	CYTO	W5PDK4	AIF1	4.58E-03	0	M1_Mo
3	CYTO	W5PG74	IRGQ	3.15E-02	0	M1_Mo
3	CYTO	W5QEL6	PCYOX1	2.32E-02	0	M1_Mo
3	CYTO	W5PIQ4	LOC101120093	2.51E-02	0.000214	M1_Mo
3	CYTO	W5NV06	ATP6V0A1	2.69E-02	0	M1_Mo
3	CYTO	W5PCR6	PIK3CD	2.89E-02	0	M1_Mo
3	CYTO	Q10994	CSTB	5.09E-06	0	M1_Mo
3	CYTO	P29330	FDX1	6.52E-05	0	M1_Mo
3	CYTO	W5PEX1	WASHC5	1.98E-02	0	M1_Mo
3	CYTO	W5QDJ5	LOC101122319	1.05E-02	0	M1_Mo
3	CYTO	W5PAJ2	PSAP	5.27E-03	0	M1_Mo

Supplementary Table 19: Shared and unique differentially abundant proteins (DAPs) between M1 and M2 relative to Mo and their cellular location.

FoldChange	Location	Protein IDs	Gene Names	P-value	FDR	Cell type
3	CYTO	W5NS94		3.79E-05	0	M1_Mo
3	CYTO	W5QCU8	NOP9	3.57E-02	0.001343	M1_Mo
3	CYTO	W5PBE7	ELOA	1.90E-02	0.000211	M1_Mo
3	CYTO	W5PFB1	TOR1B	1.13E-03	0	M1_Mo
3	CYTO	W5NVG6	PTPN9	7.80E-05	0	M1_Mo
3	CYTO	W5PVC1	SYNJ1	3.66E-03	0	M1_Mo
3	CYTO	W5P9K4	NUFIP2	1.53E-02	0	M1_Mo
3	CYTO	W5Q8Y5	HDLBP	4.10E-02	0	M1_Mo
3	CYTO	W5Q420		3.19E-03	0	M1_Mo
3	CYTO	W5PRK6	STAU1	2.29E-03	0	M1_Mo
3	CYTO	W5Q686	TPP1	2.59E-03	0	M1_Mo
3	CYTO	W5PBY3	OSBPL1A	3.88E-02	0	M1_Mo
3	CYTO	W5NYV5	SPAG9	1.54E-02	0	M1_Mo
3	CYTO	W5QAP3	TOM1	7.54E-03	0	M1_Mo
3	CYTO	W5PS60		4.48E-04	0.000218	M1_Mo
3	CYTO	W5P434	NAGPA	1.01E-03	0	M1_Mo
3	CYTO	W5PFH4	MAPKAPK3	2.32E-02	0	M1_Mo
3	CYTO	W5Q6T1	ARSB	3.36E-02	0	M1_Mo
3	CYTO	W5Q8I7	LOC101122123	1.13E-03	0	M1_Mo
3	CYTO	W5PG28	NTMT1	2.44E-03	0	M1_Mo
3	CYTO	W5Q0C3	KIF13B	5.39E-03	0	M1_Mo
3	CYTO	W5NTM0		1.12E-03	0.000219	M1_Mo
3	CYTO	W5PH85	ITGAX	2.17E-02	0	M1_Mo
3	CYTO	W5NW35	MYOF	1.94E-02	0	M1_Mo
3	CYTO	W5Q3T6	SAMSN1	8.67E-03	0	M1_Mo
3	CYTO	W5PB61		4.17E-03	0.000216	M1_Mo
3	CYTO	W5PJT1	PLAC9	1.59E-02	0	M1_Mo
3	CYTO	W5NTV0	TMEM165	1.19E-03	0.002641	M1_Mo
3	CYTO	W5QE37	SLC11A1	3.52E-02	0	M1_Mo
3	CYTO	W5PFE7	ACOX1	7.76E-05	0	M1_Mo
3	CYTO	W5P726	PRCP	1.73E-02	0	M1_Mo
3	CYTO	W5P3Z5	EEPD1	1.12E-02	0	M1_Mo
3	CYTO	W5PGK6	HNRNPLL	2.14E-02	0	M1_Mo
3	CYTO	W5Q553	ITGAV	5.02E-04	0	M1_Mo
3	CYTO	W5PCS4		9.59E-05	0	M1_Mo
3	CYTO	W5QF63	EMC4	8.58E-03	0.000203	M1_Mo
3	CYTO	W5QIJ6	SPPL2A	1.72E-02	0	M1_Mo
3	CYTO	W5QC46	ANKFY1	3.52E-02	0	M1_Mo
3	CYTO	W5NXU7	HCK	2.72E-02	0	M1_Mo
3	CYTO	W5PF85	PEA15	4.79E-05	0	M1_Mo
3	CYTO	P21621	IL1B	1.84E-02	0	M1_Mo
3	CYTO	W5Q1R9	WASHC3	4.91E-02	0	M1_Mo
3	CYTO	Q6XUZ5	IDH1	2.16E-02	0	M1_Mo
3	CYTO	W5PT68	FLNB	5.10E-02	0	M1_Mo
3	CYTO	W5NRK5	PPT2	1.91E-03	0	M1_Mo
3	CYTO	W5QAQ4	NRP2	6.05E-04	0	M1_Mo

Supplementary Table 19: Shared and unique differentially abundant proteins (DAPs) between M1 and M2 relative to Mo and their cellular location.

FoldChange	Location	Protein IDs	Gene Names	P-value	FDR	Cell type
3	CYTO	W5NYK1	PMVK	1.35E-04	0	M1_Mo
3	CYTO	W5QC43	ANTXR2	4.58E-03	0	M1_Mo
3	CYTO	W5P5Q2	MVP	2.96E-02	0	M1_Mo
3	CYTO	W5P8S0	LY96	2.81E-03	0	M1_Mo
3	CYTO	W5NVK6	DAGLB	3.87E-03	0	M1_Mo
3	CYTO	W5P6L1	RASGRP4	1.42E-02	0	M1_Mo
3	CYTO	Q29524	LPL	2.19E-02	0	M1_Mo
3	CYTO	W5PZJ8	ASRGL1	5.92E-03	0	M1_Mo
3	CYTO	W5PBJ4	ARHGAP10	3.14E-03	0	M1_Mo
3	CYTO	W5NQ85	IDE	1.30E-02	0	M1_Mo
3	CYTO	W5NUQ8	GCC1	2.29E-03	0	M1_Mo
3	CYTO	W5NX57	CHM	2.28E-04	0.000205	M1_Mo
3	CYTO	W5QA17	ATXN2	1.37E-02	0	M1_Mo
3	CYTO	W5NT19	SPAG1	8.37E-03	0	M1_Mo
3	CYTO	W5P7F8		2.43E-02	0	M1_Mo
3	CYTO	W5PNS2	TLR7	4.85E-03	0	M1_Mo
3	CYTO	W5PER8	WDR91	5.63E-03	0	M1_Mo
3	CYTO	W5QGR5	WDFY1	3.06E-02	0	M1_Mo
3	CYTO	W5PF22	KMO	3.86E-02	0	M1_Mo
3	CYTO	W5QBJ0	ANXA3	4.10E-03	0	M1_Mo
3	CYTO	W5QGJ2	CHP1	7.46E-03	0	M1_Mo
3	CYTO	W5P7B1	SIRT2	3.77E-02	0	M1_Mo
3	CYTO	W5Q2V0	YKT6	1.46E-04	0	M1_Mo
3	CYTO	W5PN70		1.34E-02	0	M1_Mo
3	CYTO	W5P3S7	ATP6AP1	3.59E-02	0	M1_Mo
3	CYTO	W5PGC6	DPP9	1.90E-02	0	M1_Mo
3	CYTO	W5PVI4	PLGRKT	8.43E-03	0	M1_Mo
3	CYTO	W5QHA0	AGFG1	5.41E-02	0	M1_Mo
3	CYTO	W5PFR8	FBP1	3.80E-02	0	M1_Mo
3	CYTO	W5PMF1	TPMT	1.48E-02	0	M1_Mo
3	CYTO	W5PEZ1		1.22E-03	0	M1_Mo
3	CYTO	W5PCM4	LUZP1	3.01E-06	0	M1_Mo
3	CYTO	W5P5E7		8.87E-03	0	M1_Mo
3	CYTO	W5PUJ2	LAMP2	3.50E-02	0	M1_Mo
3	CYTO	W5PSG0		1.56E-02	0	M1_Mo
3	CYTO	W5P333		9.74E-03	0	M1_Mo
3	CYTO	W5PPT8	GLIPR2	2.93E-03	0	M1_Mo
3	CYTO	W5P6U2		2.08E-03	0	M1_Mo
3	CYTO	W5PN60	ABR	1.84E-02	0	M1_Mo
2	CYTO	W5PP47		3.65E-02	0	M1_Mo
2	CYTO	W5QI12	TLN2	5.08E-02	0.005172	M1_Mo
2	CYTO	W5Q3B2	TYK2	2.46E-03	0	M1_Mo
2	CYTO	W5PWR6	KCNAB2	1.16E-02	0	M1_Mo
2	CYTO	W5QJ54	ARG2	9.11E-03	0	M1_Mo
2	CYTO	W5PWG8	LOC101115420	2.38E-02	0	M1_Mo
2	CYTO	W5Q2H2	SLC12A9	2.46E-05	0	M1_Mo

Supplementary Table 19: Shared and unique differentially abundant proteins (DAPs) between M1 and M2 relative to Mo and their cellular location.

FoldChange	Location	Protein IDs	Gene Names	P-value	FDR	Cell type
2	CYTO	W5Q1K4	DICER1	3.62E-02	0	M1_Mo
2	CYTO	W5P2N4	AHCYL2	3.62E-03	0	M1_Mo
2	CYTO	W5NSZ7	RAB31	4.12E-02	0	M1_Mo
2	CYTO	W5PSD4	GYS1	3.93E-02	0	M1_Mo
2	CYTO	W5PU75	GNA11	1.40E-02	0	M1_Mo
2	CYTO	W5NPQ5		3.57E-02	0	M1_Mo
2	CYTO	W5P2U4	LOC101105644	5.51E-02	0	M1_Mo
2	CYTO	W5PES1	IARS1	1.09E-02	0	M1_Mo
2	CYTO	W5PUL4	MTMR6	8.36E-03	0	M1_Mo
2	CYTO	W5NS07	HM13	2.35E-06	0	M1_Mo
2	CYTO	W5NWX7	CLEC4A	6.50E-03	0	M1_Mo
2	CYTO	W5NVF6	NR3C1	2.14E-02	0	M1_Mo
2	CYTO	W5P374		2.71E-02	0	M1_Mo
2	CYTO	W5P8F3	AGPAT5	4.45E-04	0	M1_Mo
2	CYTO	W5P363	VAT1	1.76E-02	0	M1_Mo
2	CYTO	W5P366	FAM50A	1.25E-02	0	M1_Mo
2	CYTO	W5PSD7	RAP2C	5.56E-03	0	M1_Mo
2	CYTO	W5PUU2	RRAS	1.10E-02	0	M1_Mo
2	CYTO	W5Q4N4	TOR3A	6.29E-03	0	M1_Mo
2	CYTO	W5Q411	HSPA13	1.87E-03	0	M1_Mo
2	CYTO	W5PVT3	GALNS	1.02E-02	0	M1_Mo
2	CYTO	W5PX29	TMEM9B	4.13E-02	0	M1_Mo
2	CYTO	W5QCL1	NCF4	4.88E-02	0	M1_Mo
2	CYTO	W5PQT3	LOC101105107	1.92E-03	0	M1_Mo
2	CYTO	W5NWR6	LXN	2.31E-02	0	M1_Mo
2	CYTO	W5PER3	TM9SF3	9.65E-03	0	M1_Mo
2	CYTO	W5Q560	OGFOD3	9.90E-03	0	M1_Mo
2	CYTO	W5NSS1	PPP4R1	5.21E-03	0	M1_Mo
2	CYTO	W5QIE9	BSDC1	1.62E-02	0.000216	M1_Mo
2	CYTO	W5Q922	LOC101105383	2.61E-02	0	M1_Mo
2	CYTO	W5PUV6	NCAPD3	2.12E-02	0	M1_Mo
2	CYTO	W5PWA8	HSPB1	5.70E-03	0	M1_Mo
2	CYTO	W5Q5K0		3.01E-03	0	M1_Mo
2	CYTO	W5PPS6		5.89E-05	0	M1_Mo
2	CYTO	W5PES0	STX4	2.89E-03	0	M1_Mo
2	CYTO	W5PRQ0	RBM3	3.50E-02	0	M1_Mo
2	CYTO	W5P1F8	SEC23IP	4.20E-02	0	M1_Mo
2	CYTO	W5PD25	CYTIP	9.35E-03	0	M1_Mo
2	CYTO	W5Q8J8	VPS41	6.09E-03	0	M1_Mo
2	CYTO	W5PMB1	SNX3	1.53E-03	0	M1_Mo
2	CYTO	W5PPY5	CD2AP	2.74E-04	0	M1_Mo
2	CYTO	W5PYB9	ABCA6	3.22E-02	0	M1_Mo
2	CYTO	W5Q0M7	RAP2B	1.51E-02	0	M1_Mo
2	CYTO	W5QEU6	ANXA4	2.46E-02	0	M1_Mo
2	CYTO	W5P6V4	GLG1	3.99E-02	0	M1_Mo
2	CYTO	W5PKV1	DNASE2	1.89E-02	0	M1_Mo

Supplementary Table 19: Shared and unique differentially abundant proteins (DAPs) between M1 and M2 relative to Mo and their cellular location.

FoldChange	Location	Protein IDs	Gene Names	P-value	FDR	Cell type
2	CYTO	W5Q0N7	IFIT2	5.13E-02	0	M1_Mo
2	CYTO	W5NZ80	NIBAN1	5.21E-02	0	M1_Mo
2	CYTO	W5PM50	UPF1	4.06E-02	0	M1_Mo
2	CYTO	W5NWG5	LOC101118849	1.11E-02	0	M1_Mo
2	CYTO	W5Q396		2.19E-02	0	M1_Mo
2	CYTO	W5PLW1	CTNND1	4.93E-02	0	M1_Mo
2	CYTO	W5PXH4	PTPRE	1.51E-04	0	M1_Mo
2	CYTO	W5NUE5	SLC38A7	6.66E-03	0	M1_Mo
2	CYTO	W5QAS2	STX6	3.01E-02	0	M1_Mo
2	CYTO	W5PSX7	FES	4.47E-02	0	M1_Mo
2	CYTO	W5PUI3	GOLGA1	4.48E-05	0	M1_Mo
2	CYTO	W5NRR6	MGAT2	5.66E-04	0	M1_Mo
2	CYTO	W5PA59	LOC101117015	1.94E-04	0	M1_Mo
2	CYTO	W5Q2L9		2.49E-02	0	M1_Mo
2	CYTO	W5P1U9	OXSM	1.09E-02	0	M1_Mo
2	CYTO	W5NUU1	VRK2	6.06E-03	0	M1_Mo
2	CYTO	W5PMC5	LOC101110546	3.29E-03	0	M1_Mo
2	CYTO	W5NTM7	DENND10	4.89E-02	0	M1_Mo
2	CYTO	W5NRA9	ASL	6.51E-03	0	M1_Mo
2	CYTO	W5PTW5	RALB	5.46E-02	0	M1_Mo
2	CYTO	W5PHQ8	TSTD1	1.74E-02	0	M1_Mo
2	CYTO	W5P1S3	SLC25A13	1.60E-02	0	M1_Mo
2	CYTO	W5PVM8	UBR7	2.72E-05	0	M1_Mo
2	CYTO	W5P813	COL4A1	1.86E-02	0	M1_Mo
2	CYTO	W5NXB7		5.41E-02	0	M1_Mo
2	CYTO	W5P4I3	TRAF3	7.27E-03	0	M1_Mo
2	CYTO	W5NPP2	CPM	1.11E-02	0	M1_Mo
2	CYTO	W5P261	PPM1H	4.48E-02	0	M1_Mo
2	CYTO	W5PHY4	TYMS	2.30E-02	0	M1_Mo
2	CYTO	Q9XT28	ATOX1	6.34E-03	0	M1_Mo
2	CYTO	W5PK12	OAT	6.36E-03	0	M1_Mo
2	CYTO	W5QJA2	CD14	3.46E-02	0	M1_Mo
2	CYTO	W5Q3K9	IL2RG	2.35E-02	0	M1_Mo
2	CYTO	W5PU61	SETD7	1.92E-02	0	M1_Mo
2	CYTO	W5PA31	RAB20	1.56E-02	0	M1_Mo
2	CYTO	W5PRS4	FKBP5	2.35E-02	0	M1_Mo
2	CYTO	W5PFM2	AP3M1	3.24E-02	0	M1_Mo
2	CYTO	W5NU07		1.77E-02	0	M1_Mo
2	CYTO	W5NQ23	WIPF1	1.80E-02	0	M1_Mo
2	CYTO	W5PKF9	FIG4	3.43E-02	0	M1_Mo
2	CYTO	W5QCF3	SLC35F6	2.06E-03	0	M1_Mo
2	CYTO	W5PHJ3	AHR	2.44E-02	0	M1_Mo
2	CYTO	W5PVL6	AP2A1	1.67E-02	0	M1_Mo
2	CYTO	W5QAF8	ATP7A	1.56E-02	0	M1_Mo
2	CYTO	W5QC34	MAN2A1	5.31E-03	0	M1_Mo
2	CYTO	W5PTB7	ATG7	4.72E-02	0	M1_Mo

Supplementary Table 19: Shared and unique differentially abundant proteins (DAPs) between M1 and M2 relative to Mo and their cellular location.

FoldChange	Location	Protein IDs	Gene Names	P-value	FDR	Cell type
2	CYTO	W5NZQ6	CYP1A1	4.91E-02	0	M1_Mo
2	CYTO	W5Q497	PGGT1B	2.94E-02	0.000203	M1_Mo
2	CYTO	W5PQM4	MOSPD2	2.04E-02	0	M1_Mo
2	CYTO	W5QH03	LPP	1.82E-02	0	M1_Mo
2	CYTO	W5PUW5	GOLGA5	4.30E-02	0	M1_Mo
2	CYTO	W5PSE3	SH3TC1	7.53E-04	0.000221	M1_Mo
2	CYTO	W5P7P6	MYO18A	1.18E-02	0	M1_Mo
2	CYTO	W5QDY5	ATP6V1A	3.21E-02	0	M1_Mo
2	CYTO	W5PBH0	SDSL	5.82E-03	0	M1_Mo
2	CYTO	W5Q5T6	CSNK1D	3.43E-02	0.000209	M1_Mo
2	CYTO	W5QEQ1	TRABD	1.92E-02	0	M1_Mo
2	CYTO	W5PMY4	GABARAP	3.85E-02	0	M1_Mo
2	CYTO	Q6ECI6	ITGB2	1.90E-02	0	M1_Mo
2	CYTO	W5NZD5	POLB	2.87E-03	0.000399	M1_Mo
2	CYTO	W5P848	MLKL	7.56E-03	0	M1_Mo
2	CYTO	W5P4T0	VHL	7.59E-05	0.000204	M1_Mo
2	CYTO	W5P1T1	ANO10	1.64E-02	0	M1_Mo
2	CYTO	W5PI67	IDS	3.56E-03	0	M1_Mo
2	CYTO	W5NR63	S100A4	4.75E-02	0	M1_Mo
2	CYTO	W5QA59	HGS	2.84E-03	0	M1_Mo
2	CYTO	W5NVC9	RAC1	2.65E-02	0	M1_Mo
2	CYTO	W5P8A0		4.02E-02	0	M1_Mo
2	CYTO	W5PDN7	GPD2	4.50E-02	0	M1_Mo
2	CYTO	W5P1U3	LACTB2	2.13E-02	0	M1_Mo
2	CYTO	W5NZ67		5.12E-02	0	M1_Mo
2	CYTO	W5QBQ8	RAB5C	2.86E-02	0	M1_Mo
2	CYTO	W5Q5B5	CSAD	9.61E-03	0	M1_Mo
2	CYTO	W5PVC2	CDC37L1	1.69E-02	0	M1_Mo
2	CYTO	W5PBK8	SNX11	3.69E-02	0.00021	M1_Mo
2	CYTO	P00349	PGD	2.21E-02	0	M1_Mo
2	CYTO	W5P8B4	TRAPPC8	3.55E-03	0	M1_Mo
2	CYTO	W5P5P0	SRGAP2	3.37E-02	0	M1_Mo
2	CYTO	W5QB00	MCM5	2.70E-02	0	M1_Mo
2	CYTO	W5NZ42	SLC2A6	3.79E-03	0	M1_Mo
2	CYTO	W5PPM6	PQBP1	3.95E-02	0.000203	M1_Mo
2	CYTO	W5QD46	CYTH4	7.77E-03	0	M1_Mo
2	CYTO	W5P316	NAMPT	2.07E-02	0	M1_Mo
2	CYTO	W5P6I9	GALNT1	4.29E-02	0	M1_Mo
2	CYTO	W5PE91	LOC101105400	4.28E-02	0.000205	M1_Mo
2	CYTO	W5P3U4	COMMD2	3.81E-02	0	M1_Mo
2	CYTO	W5Q6E0	CUL2	3.37E-02	0	M1_Mo
2	CYTO	W5PTT3	CRTC3	4.40E-02	0	M1_Mo
2	CYTO	W5Q175	GNPTAB	5.05E-03	0	M1_Mo
2	CYTO	W5QDQ8	MMP14	3.26E-02	0	M1_Mo
2	CYTO	W5P983	COL4A2	4.89E-03	0.000209	M1_Mo
2	CYTO	W5PSF8	CRYZ	3.21E-02	0	M1_Mo

Supplementary Table 19: Shared and unique differentially abundant proteins (DAPs) between M1 and M2 relative to Mo and their cellular location.

FoldChange	Location	Protein IDs	Gene Names	P-value	FDR	Cell type
2	CYTO	W5PEN2	TPD52	3.32E-03	0	M1_Mo
2	CYTO	C8BKC7	MYD88	1.39E-02	0	M1_Mo
2	CYTO	W5NW41	TBC1D9B	1.01E-02	0	M1_Mo
2	CYTO	W5PZ62	ZFYVE16	1.14E-03	0	M1_Mo
2	CYTO	W5QH66		1.28E-02	0.000216	M1_Mo
2	CYTO	W5NYA8	SCAMP2	4.74E-02	0	M1_Mo
2	CYTO	W5PED5	LOC101116121	4.44E-04	0	M1_Mo
2	CYTO	W5PLZ0	ATP6V1B2	1.61E-02	0	M1_Mo
2	CYTO	W5Q045	TMED3	2.15E-03	0	M1_Mo
2	CYTO	W5PBG1		4.88E-02	0	M1_Mo
2	CYTO	W5PVR6	TNFAIP8L2	4.04E-02	0	M1_Mo
2	CYTO	W5Q2C9		1.61E-02	0	M1_Mo
2	CYTO	W5PFQ8	RRAGA	6.48E-03	0	M1_Mo
2	CYTO	W5PIN6	LDHA	2.30E-04	0	M1_Mo
2	CYTO	W5NYE8	SEL1L	1.28E-02	0	M1_Mo
2	CYTO	W5QCD3	RNASEL	9.11E-03	0	M1_Mo
2	CYTO	W5QCM8	EPS15L1	4.22E-02	0	M1_Mo
2	CYTO	W5Q3J3	COLGALT1	2.67E-03	0	M1_Mo
2	CYTO	W5QAL6	FMNL3	3.49E-04	0.000218	M1_Mo
2	CYTO	W5QG24	PPT1	4.94E-02	0	M1_Mo
2	CYTO	W5P3A2		3.41E-02	0	M1_Mo
2	CYTO	W5NZJ1	LOC101114075	2.24E-02	0	M1_Mo
2	CYTO	W5NPU0	NAPRT	1.92E-02	0	M1_Mo
2	CYTO	W5Q6V7	SIPA1	2.53E-02	0	M1_Mo
2	CYTO	W5NUX5		2.60E-03	0	M1_Mo
2	CYTO	W5Q430	IDUA	1.11E-02	0.00021	M1_Mo
2	CYTO	W5P1U0	TXNL4A	1.10E-03	0	M1_Mo
2	CYTO	W5Q1D8	GALNT7	1.30E-03	0.000218	M1_Mo
2	CYTO	W5P9Y1	SNX6	5.36E-02	0	M1_Mo
2	CYTO	W5PNC8	PADI2	2.82E-02	0	M1_Mo
2	CYTO	W5PUT6	CLTC	4.75E-02	0	M1_Mo
2	CYTO	W5Q643	LOC101110219	3.56E-02	0.008888	M1_Mo
2	CYTO	W5NSQ3	HFE	4.31E-03	0	M1_Mo
2	CYTO	W5NX28	MKNK1	4.24E-02	0	M1_Mo
2	CYTO	W5Q971	DPP8	1.89E-05	0	M1_Mo
2	CYTO	W5Q6K0	HEATR3	5.10E-02	0.002465	M1_Mo
2	CYTO	W5PPK8		8.51E-03	0	M1_Mo
2	CYTO	W5PK41	LPIN2	1.34E-02	0.0002	M1_Mo
2	CYTO	W5NRD1	ERLIN2	3.55E-02	0	M1_Mo
2	CYTO	W5NTC4	BMI1	5.37E-02	0	M1_Mo
2	CYTO	W5P8I3	RIDA	2.90E-02	0	M1_Mo
2	CYTO	W5QB79	GLCE	1.01E-02	0.000221	M1_Mo
2	CYTO	W5P7L0	SUN1	3.20E-03	0.000217	M1_Mo
2	CYTO	W5QHV6	SNX1	2.08E-02	0	M1_Mo
2	CYTO	W5QIX6	EXOC5	2.32E-02	0	M1_Mo
2	CYTO	W5NPP0		2.40E-02	0	M1_Mo

Supplementary Table 19: Shared and unique differentially abundant proteins (DAPs) between M1 and M2 relative to Mo and their cellular location.

FoldChange	Location	Protein IDs	Gene Names	P-value	FDR	Cell type
2	CYTO	W5PDX8	FAM120A	1.72E-03	0	M1_Mo
2	CYTO	W5PF04	MAN1A1	1.49E-02	0.000202	M1_Mo
2	CYTO	W5PKP3	FAM114A1	2.59E-03	0	M1_Mo
2	CYTO	W5QGX7	EHD4	4.55E-02	0	M1_Mo
2	CYTO	W5Q2K9	CYFIP1	9.47E-03	0	M1_Mo
2	CYTO	W5PI45	ENOSF1	4.77E-02	0	M1_Mo
2	CYTO	W5Q3A2		5.62E-02	0	M1_Mo
2	CYTO	W5P700	KIF1B	2.04E-02	0	M1_Mo
2	CYTO	W5Q867	SLC12A2	4.33E-02	0.000398	M1_Mo
2	CYTO	W5QFZ2	DTX3L	3.22E-02	0	M1_Mo
2	CYTO	W5Q8I6	POLDIP2	1.92E-02	0	M1_Mo
2	CYTO	W5QHV8	PLD1	4.53E-02	0	M1_Mo
2	CYTO	W5PZV5	HS2ST1	1.42E-02	0	M1_Mo
2	CYTO	W5P7I3	ARL11	1.28E-02	0	M1_Mo
2	CYTO	W5QFY8	PARP9	8.25E-03	0	M1_Mo
2	CYTO	W5NPP9	PRKCD	1.32E-02	0	M1_Mo
2	CYTO	W5PWI8	EEF1AKMT1	5.56E-03	0	M1_Mo
2	CYTO	W5Q1P6	STX2	4.21E-03	0	M1_Mo
2	CYTO	W5P381	LOC101116334	1.78E-02	0	M1_Mo
2	CYTO	W5Q740	ABCD3	1.72E-02	0	M1_Mo
2	CYTO	W5Q0P4	MTMR9	8.01E-03	0	M1_Mo
2	CYTO	W5Q4P9	CYRIA	2.80E-02	0	M1_Mo
2	CYTO	W5NVT3	SND1	5.02E-02	0	M1_Mo
2	CYTO	W5PK31	FCGR3A	3.40E-02	0	M1_Mo
2	CYTO	W5NQG1	SETD3	7.69E-04	0	M1_Mo
2	CYTO	W5Q4Q8		4.48E-04	0	M1_Mo
2	CYTO	W5P5I4	LAMTOR2	4.54E-03	0	M1_Mo
2	CYTO	W5Q418	NELFCD	4.64E-03	0	M1_Mo
2	CYTO	W5Q928	PCYT2	3.37E-03	0	M1_Mo
2	CYTO	W5PJN0	PSPH	2.63E-02	0	M1_Mo
2	CYTO	W5Q7X8	CRYBG3	3.45E-02	0	M1_Mo
2	CYTO	W5PPJ2	VPS33A	2.38E-02	0	M1_Mo
2	CYTO	W5PB20		3.23E-02	0	M1_Mo
2	CYTO	W5PC57	MRTFB	1.05E-02	0	M1_Mo
2	CYTO	W5QJ49	ATP6V1D	4.39E-02	0	M1_Mo
2	CYTO	W5Q9H8	SNX5	1.25E-02	0	M1_Mo
2	CYTO	W5QIK3	USP8	4.68E-03	0	M1_Mo
2	CYTO	W5P7S0	PIP4K2C	2.91E-02	0	M1_Mo
2	CYTO	W5PU63	SYAP1	3.92E-02	0	M1_Mo
2	CYTO	W5P4Y1	WDR7	1.75E-02	0	M1_Mo
2	CYTO	W5QIA8	YARS1	3.84E-02	0	M1_Mo
2	CYTO	W5PVU5	PSMB7	4.83E-02	0	M1_Mo
2	CYTO	W5QBW4	VAV3	6.85E-03	0	M1_Mo
2	CYTO	W5P3H1	LOC101111732	1.24E-02	0	M1_Mo
2	CYTO	W5P5A7	VPS16	1.23E-03	0	M1_Mo
2	CYTO	W5P6M5	SEC11C	1.57E-05	0.001908	M1_Mo

Supplementary Table 19: Shared and unique differentially abundant proteins (DAPs) between M1 and M2 relative to Mo and their cellular location.

FoldChange	Location	Protein IDs	Gene Names	P-value	FDR	Cell type
2	CYTO	W5QHP5	GNB4	3.33E-02	0	M1_Mo
2	CYTO	W5QH58	DNAJB11	2.14E-02	0	M1_Mo
2	CYTO	W5Q6T6	SKIC3	2.39E-02	0	M1_Mo
2	CYTO	W5PKP0	TIGAR	1.29E-02	0.000201	M1_Mo
2	CYTO	W5PWF2	ATP6V1H	1.27E-02	0	M1_Mo
2	CYTO	W5P6D2	MGLL	8.22E-04	0.000217	M1_Mo
2	CYTO	A2SW69	ANXA2	3.12E-03	0	M1_Mo
2	CYTO	C5IWT0	ARF4	4.86E-02	0	M1_Mo
2	CYTO	W5PZX0	KRT24	2.72E-02	0	M1_Mo
2	CYTO	W5P824	LOC101103096	4.80E-02	0	M1_Mo
2	CYTO	W5QCV4	DHRS1	1.85E-02	0	M1_Mo
2	CYTO	W5PKN3	PDLIM2	2.86E-02	0	M1_Mo
2	CYTO	W5QDU7	RBKS	1.50E-02	0	M1_Mo
2	CYTO	W5PZM9	ANXA5	1.36E-03	0	M1_Mo
2	CYTO	W5NZZ3	ATP6V1F	1.81E-02	0	M1_Mo
2	CYTO	W5PWZ2		1.51E-02	0	M1_Mo
2	CYTO	W5PNX6	PREP	3.71E-02	0	M1_Mo
2	CYTO	W5QAE8	ALDH7A1	2.86E-03	0	M1_Mo
2	CYTO	W5PDE3	SPTLC1	9.36E-03	0	M1_Mo
2	CYTO	W5PDU4	NMT2	3.01E-03	0	M1_Mo
2	CYTO	W5NSQ8	LOC101103383	8.90E-04	0.006244	M1_Mo
2	CYTO	W5PK60	CLCN5	2.88E-02	0	M1_Mo
2	CYTO	W5PCC0	LACC1	3.28E-02	0	M1_Mo
2	CYTO	W5Q3L8	LOC101104306	4.78E-03	0	M1_Mo
2	CYTO	W5PRP9	UBE2G2	4.53E-02	0	M1_Mo
2	CYTO	W5PNW7	VIM	1.13E-04	0	M1_Mo
2	CYTO	W5PGC9	MMAA	3.01E-02	0	M1_Mo
2	CYTO	W5Q234	RAF1	5.54E-02	0	M1_Mo
2	CYTO	W5PVR9	ERMP1	4.85E-02	0	M1_Mo
2	CYTO	W5P1M3		7.10E-03	0	M1_Mo
2	CYTO	W5NRL0	PLCD1	2.00E-02	0	M1_Mo
2	CYTO	W5NUZ1	DYNC1I2	3.85E-02	0	M1_Mo
2	CYTO	W5PF26	JAML	7.12E-03	0	M1_Mo
2	CYTO	W5NYK8	GALC	4.50E-03	0	M1_Mo
2	CYTO	W5PAA0	RBMS1	5.01E-02	0.00022	M1_Mo
2	CYTO	W5NYG8	TSSC4	1.60E-03	0	M1_Mo
2	CYTO	W5Q1R8	MAPK1	3.64E-02	0	M1_Mo
2	CYTO	W5QB43	CDK6	6.68E-04	0	M1_Mo
-2	CYTO	W5PIE9	NIT1	4.75E-02	0	M1_Mo
-2	CYTO	W5PTN4	TMPO	4.82E-02	0	M1_Mo
-2	CYTO	W5P8R0	NDUFA4	3.42E-02	0	M1_Mo
-2	CYTO	W5PAX3	OGFOD2	2.25E-03	0	M1_Mo
-2	CYTO	W5NSP0		2.87E-02	0	M1_Mo
-2	CYTO	W5PMI8	CUL5	4.61E-03	0	M1_Mo
-2	CYTO	W5P8W5		3.62E-02	0	M1_Mo
-2	CYTO	W5PDB0	TRIM58	5.02E-02	0	M1_Mo

Supplementary Table 19: Shared and unique differentially abundant proteins (DAPs) between M1 and M2 relative to Mo and their cellular location.

FoldChange	Location	Protein IDs	Gene Names	P-value	FDR	Cell type
-2	CYTO	W5Q732	PDK3	5.36E-02	0.000396	M1_Mo
-2	CYTO	W5Q804	SPECC1	4.90E-02	0	M1_Mo
-2	CYTO	W5Q9R1	RTCA	9.35E-03	0	M1_Mo
-2	CYTO	W5QHP6	ARHGDIB	1.61E-02	0	M1_Mo
-2	CYTO	W5P1E2	CNST	5.02E-02	0	M1_Mo
-2	CYTO	W5QHL5	CAB39	1.94E-02	0	M1_Mo
-2	CYTO	W5PMC9	ARHGAP6	5.54E-02	0.00713	M1_Mo
-2	CYTO	W5Q8P3	USE1	2.25E-02	0	M1_Mo
-2	CYTO	C8BKC5	PRDX2	1.30E-02	0	M1_Mo
-2	CYTO	W5PV48	USP11	2.17E-02	0	M1_Mo
-2	CYTO	W5P671	DDAH2	3.69E-02	0.009548	M1_Mo
-2	CYTO	W5PTQ0	RNGTT	4.27E-02	0.000205	M1_Mo
-2	CYTO	W5PUR1	CNOT11	4.62E-02	0	M1_Mo
-2	CYTO	W5Q9J0	RNPS1	2.41E-02	0	M1_Mo
-2	CYTO	W5QBX9	ILKAP	8.07E-03	0	M1_Mo
-2	CYTO	W5PJJ5	PGM2L1	3.47E-02	0.000206	M1_Mo
-2	CYTO	W5PYM3	CAAP1	1.02E-02	0.001155	M1_Mo
-2	CYTO	W5Q4D5	BCO2	4.06E-03	0	M1_Mo
-2	CYTO	W5NUX2		1.95E-02	0	M1_Mo
-2	CYTO	O78750	MT-CO2	5.00E-02	0	M1_Mo
-2	CYTO	W5PHL8	NUDT16	2.79E-02	0	M1_Mo
-2	CYTO	W5NSI2	RALBP1	2.50E-02	0.008549	M1_Mo
-2	CYTO	W5Q2W6	PPFIBP2	1.87E-02	0.000222	M1_Mo
-2	CYTO	W5PAV5	RFX1	3.65E-02	0	M1_Mo
-2	CYTO	W5NQ72	PFAS	8.24E-04	0	M1_Mo
-2	CYTO	W5PEA0	FGD3	6.30E-03	0	M1_Mo
-2	CYTO	W5NTA4	CGGBP1	1.58E-02	0	M1_Mo
-2	CYTO	W5NUT8	PIP4K2A	1.48E-03	0	M1_Mo
-2	CYTO	W5PN94	LUC7L	2.59E-02	0	M1_Mo
-2	CYTO	W5NW56	IRF2BPL	5.56E-02	0	M1_Mo
-2	CYTO	W5NUS2	MED9	5.38E-02	0.000221	M1_Mo
-2	CYTO	W5QBV1	FER	1.45E-02	0	M1_Mo
-2	CYTO	W5QIY4	ARID4A	3.31E-02	0	M1_Mo
-2	CYTO	W5QH43	EIF4A2	2.43E-02	0	M1_Mo
-2	CYTO	W5QGL7	RTF1	3.90E-02	0.00446	M1_Mo
-2	CYTO	W5NUE6	PLEKHA2	2.43E-02	0	M1_Mo
-2	CYTO	P80190		2.78E-03	0	M1_Mo
-2	CYTO	W5NRR9	STAT5A	4.36E-02	0	M1_Mo
-2	CYTO	W5QHL6	ITM2C	3.45E-02	0	M1_Mo
-2	CYTO	W5Q0P0	GABPA	2.98E-02	0	M1_Mo
-2	CYTO	W5P2J9		4.27E-03	0	M1_Mo
-2	CYTO	W5PP64	FHL1	2.14E-02	0	M1_Mo
-2	CYTO	W5P684	ACTBL2	3.39E-02	0.004293	M1_Mo
-2	CYTO	W5PTB3	INPP5K	5.42E-02	0.000202	M1_Mo
-2	CYTO	C5IS96	LCAT	2.12E-02	0.000206	M1_Mo
-3	CYTO	W5PUI4	RAB3A	3.66E-02	0.000215	M1_Mo

Supplementary Table 19: Shared and unique differentially abundant proteins (DAPs) between M1 and M2 relative to Mo and their cellular location.

FoldChange	Location	Protein IDs	Gene Names	P-value	FDR	Cell type
-3	CYTO	W5P173	BMS1	3.39E-02	0	M1_Mo
-3	CYTO	W5P795	LRBA	5.07E-02	0	M1_Mo
-3	CYTO	W5P0R4	CTIF	6.26E-03	0.000394	M1_Mo
-3	CYTO	W5Q059	SASH3	5.30E-02	0	M1_Mo
-3	CYTO	W5PP85	TBC1D10C	5.19E-02	0	M1_Mo
-3	CYTO	W5PVX3	PRKG1	3.38E-02	0	M1_Mo
-3	CYTO	W5PAV0	STAP1	2.68E-02	0	M1_Mo
-3	CYTO	W5NTV6		1.51E-02	0	M1_Mo
-3	CYTO	W5PGX7	SSRP1	4.97E-03	0	M1_Mo
-3	CYTO	W5Q9H1	ZYX	5.20E-02	0	M1_Mo
-3	CYTO	W5PZP8	FIS1	1.22E-03	0	M1_Mo
-3	CYTO	W5P9M9	LOC101103862	4.54E-02	0	M1_Mo
-3	CYTO	W5NT67	TUBGCP2	4.97E-03	0.000206	M1_Mo
-3	CYTO	W5P8H4	DCTPP1	3.24E-02	0	M1_Mo
-3	CYTO	W5P6T0		3.57E-02	0.001155	M1_Mo
-3	CYTO	W5Q7L1	CNN3	1.76E-02	0	M1_Mo
-3	CYTO	W5PKA9	F5	9.85E-04	0	M1_Mo
-3	CYTO	W5Q6U5	ARMH3	4.33E-02	0	M1_Mo
-3	CYTO	W5QCH5	WDR47	1.81E-02	0.000204	M1_Mo
-3	CYTO	W5PFJ0	VCL	3.19E-02	0	M1_Mo
-3	CYTO	W5PEC3	ANXA6	4.09E-02	0	M1_Mo
-3	CYTO	W5PGU9	PLCG2	6.04E-03	0	M1_Mo
-3	CYTO	W5QEL7	NDRG2	3.71E-02	0.00784	M1_Mo
-3	CYTO	Q09YJ2	TES	8.52E-03	0	M1_Mo
-3	CYTO	W5PAF7	RSL1D1	2.83E-02	0	M1_Mo
-3	CYTO	W5NV79	NUP210	3.28E-02	0	M1_Mo
-3	CYTO	W5Q3I7	TUBB1	4.42E-02	0	M1_Mo
-3	CYTO	P14639	ALB	1.96E-02	0	M1_Mo
-3	CYTO	W5PE21	PELP1	1.05E-02	0	M1_Mo
-3	CYTO	W5Q5E7	PYCR3	2.30E-02	0	M1_Mo
-4	CYTO	W5P433	BRCA1	6.49E-04	0.003742	M1_Mo
-4	CYTO	W5PTS4	LOC101114275	9.33E-04	0	M1_Mo
-4	CYTO	W5NQA0	GCC2	4.64E-02	0	M1_Mo
-4	CYTO	W5P815	SEPTIN1	4.54E-02	0	M1_Mo
-4	CYTO	W5PG02	RASA3	7.90E-03	0	M1_Mo
-4	CYTO	W5NRI1		7.09E-03	0.000214	M1_Mo
-4	CYTO	W5PX46	LOC101122591	2.35E-02	0	M1_Mo
-4	CYTO	W5PP80	FRY	3.43E-02	0.001158	M1_Mo
-4	CYTO	W5P5Y4	RIPOR2	1.74E-02	0	M1_Mo
-4	CYTO	W5NTF8	SPNS1	9.09E-03	0	M1_Mo
-4	CYTO	W5PZG0	ADD1	9.12E-03	0	M1_Mo
-4	CYTO	W5PV80	BANK1	2.60E-02	0	M1_Mo
-4	CYTO	W5PD82	CALD1	3.28E-02	0	M1_Mo
-4	CYTO	W5PYK8	VNN2	2.34E-04	0	M1_Mo
-4	CYTO	W5PLZ3	PACSIN1	4.26E-02	0	M1_Mo
-4	CYTO	W5NXW9		4.77E-02	0	M1_Mo

Supplementary Table 19: Shared and unique differentially abundant proteins (DAPs) between M1 and M2 relative to Mo and their cellular location.

FoldChange	Location	Protein IDs	Gene Names	P-value	FDR	Cell type
-4	CYTO	W5PFI6	RASGRP2	4.99E-02	0	M1_Mo
-4	CYTO	W5PHI5	PALM	1.64E-03	0	M1_Mo
-4	CYTO	W5PTQ7	TRAF3IP3	1.43E-02	0	M1_Mo
-4	CYTO	W5PRY2	ACAP1	2.80E-02	0	M1_Mo
-5	CYTO	W5Q9S4	ING3	2.58E-02	0.001158	M1_Mo
-5	CYTO	W5PJ75	SPTAN1	1.95E-03	0	M1_Mo
-5	CYTO	W5PRW4	TRIAP1	1.90E-02	0.000204	M1_Mo
-5	CYTO	W5Q2S8	MYL9	4.45E-03	0	M1_Mo
-5	CYTO	W5NU34	TREML1	3.45E-02	0	M1_Mo
-5	CYTO	W5Q0K9	ABLIM1	2.66E-02	0	M1_Mo
-6	CYTO	W5QGQ3	LOC443320	1.23E-02	0.000207	M1_Mo
-6	CYTO	W5P4L3	AVIL	4.41E-02	0	M1_Mo
-6	CYTO	W5NT35	LOC443162	2.18E-02	0	M1_Mo
-6	CYTO	W5NZX9	SPTBN1	1.55E-03	0	M1_Mo
-6	CYTO	W5PF87	ALOX15	2.46E-02	0	M1_Mo
-6	CYTO	W5PYD8	PARP4	5.82E-06	0.009212	M1_Mo
-6	CYTO	W5QD99	USP40	5.41E-02	0	M1_Mo
-7	CYTO	W5QIK8	SELENBP1	1.01E-02	0	M1_Mo
-7	CYTO	W5P733	PDLIM1	3.29E-03	0	M1_Mo
8	SEC	W5NZA2	FASTKD1	0.027985265	0.008059	M1_Mo
7	SEC	W5PHJ9	LOC101119572	0.000618121	0	M1_Mo
6	SEC	W5NWF5	RARRES1	0.000794163	0	M1_Mo
6	SEC	W5QHV3	FABP1	0.01009832	0	M1_Mo
6	SEC	P36925	CXCL8	0.001266035	0.002357	M1_Mo
5	SEC	W5Q754	TTN	0.025052063	0.002353	M1_Mo
5	SEC	W5QB02	TGM2	0.020445692	0.002342	M1_Mo
4	SEC	W5P2M5	LOC101114535	0.002159776	0	M1_Mo
4	SEC	W5Q3X9	LOC100526782	0.013034497	0	M1_Mo
4	SEC	W5P4V3	MMP3	0.034837807	0	M1_Mo
4	SEC	W5PS94	NUCB1	0.004263573	0	M1_Mo
4	SEC	W5PMM5	ERP44	0.009869208	0	M1_Mo
4	SEC	W5P5K5	MMP1	0.007691341	0	M1_Mo
3	SEC	W5Q1B2	SF3B1	0.035008117	0.002346	M1_Mo
3	SEC	W5Q0F3	TGFBI	0.031375922	0	M1_Mo
3	SEC	W5PAL2	LOC101105297	0.042051973	0	M1_Mo
3	SEC	W5QIR6	GATM	0.008991856	0	M1_Mo
3	SEC	W5NTD9	CHI3L1	0.001770022	0	M1_Mo
3	SEC	W5QG01	DNPEP	0.054204275	0	M1_Mo
3	SEC	W5P8R4	CSF1R	0.0091572	0	M1_Mo
2	SEC	W5PAB5	GPLD1	0.039518032	0.001586	M1_Mo
2	SEC	W5PDR7	C8A	0.024815282	0.001608	M1_Mo
2	SEC	W5PGV0	ITGAM	0.025856627	0	M1_Mo
-2	SEC	W5P6H2		0.047392996	0.004415	M1_Mo
-2	SEC	W5PV50	ADA2	0.052166384	0.004461	M1_Mo
-2	SEC	W5PVG1	CAPN1	0.029644571	0	M1_Mo
-2	SEC	W5PVX8	PPP3CA	0.026005474	0	M1_Mo

Supplementary Table 19: Shared and unique differentially abundant proteins (DAPs) between M1 and M2 relative to Mo and their cellular location.

FoldChange	Location	Protein IDs	Gene Names	P-value	FDR	Cell type
-2	SEC	W5PYQ9	CPNE1	0.033877306	0	M1_Mo
-2	SEC	W5PI22	CNPY2	0.030493743	0	M1_Mo
-2	SEC	W5NXX1	IPO5	0.022279034	0	M1_Mo
-3	SEC	W5Q2I7	USO1	0.049488089	0	M1_Mo
-3	SEC	W5NRL8	EIF3A	0.018039762	0	M1_Mo
-3	SEC	W5P8Q0	AP2B1	0.044958451	0	M1_Mo
-3	SEC	B7TJ15	MAPK14	0.049361733	0	M1_Mo
-3	SEC	W5Q9X5	MAP2K1	0.048228902	0	M1_Mo
-3	SEC	W5Q0B6	PPP1R12A	0.029299238	0.002351	M1_Mo
-3	SEC	W5P6M2	PSMD5	0.04335681	0	M1_Mo
-3	SEC	W5PS88	GOT1	0.02876994	0	M1_Mo
-3	SEC	W5P7L2	ATP6V1G1	0.053764547	0	M1_Mo
-3	SEC	W5P4J1	VAV1	0.017783038	0	M1_Mo
-3	SEC	W5NX31	AHSA1	0.027450288	0	M1_Mo
-3	SEC	W5PF71	KPNB1	0.024531534	0	M1_Mo
-3	SEC	W5PL67	SUCLG2	0.039556819	0	M1_Mo
-3	SEC	W5PZ86	SEPTIN8	0.015889404	0	M1_Mo
-3	SEC	W5PMH1	GSS	0.019709161	0	M1_Mo
-3	SEC	P09670	SOD1	0.016324793	0.005591	M1_Mo
-3	SEC	W5QFP1	PABPC4	0.055422305	0	M1_Mo
-3	SEC	W5PC25	LOC101116273	0.043228079	0	M1_Mo
-3	SEC	W5Q3N5	PRKAR2A	0.032537757	0	M1_Mo
-3	SEC	W5Q9M6		0.045292743	0	M1_Mo
-3	SEC	W5P538	CD93	0.008868299	0	M1_Mo
-3	SEC	W5Q5R8	TXNRD1	0.03813689	0	M1_Mo
-3	SEC	W5P880	PRG4	0.000970446	0	M1_Mo
-3	SEC	W5P359	STIP1	0.042699452	0	M1_Mo
-3	SEC	W5NZ57	PSMB10	0.049361632	0	M1_Mo
-3	SEC	W5NPQ6	CAPZA2	0.042111725	0	M1_Mo
-3	SEC	W5Q701	LOC101112491	0.035680115	0	M1_Mo
-3	SEC	W5P7J7	TWF2	0.048140614	0	M1_Mo
-3	SEC	W5PJB6	PGM1	0.006823941	0	M1_Mo
-3	SEC	W5P694	RPLP2	0.047836882	0	M1_Mo
-3	SEC	W5PIM8	GALK1	0.027716826	0	M1_Mo
-3	SEC	W5P889	SEPTIN9	0.054261695	0	M1_Mo
-3	SEC	Q1ZZU7	MIF	0.039103829	0	M1_Mo
-4	SEC	W5Q3E3	RAB2A	0.019353434	0	M1_Mo
-4	SEC	W5PFV7	COPA	0.036966422	0	M1_Mo
-4	SEC	W5QBH1		0.012284691	0	M1_Mo
-4	SEC	W5PMQ9	SAE1	0.055229419	0	M1_Mo
-4	SEC	W5PNV0	RPL12	0.022085697	0	M1_Mo
-4	SEC	W5Q3A5	PAFAH1B1	0.014024248	0	M1_Mo
-4	SEC	W5P9U4	PSMA1	0.054850009	0	M1_Mo
-4	SEC	W5PQK3	PFKL	0.04828543	0	M1_Mo
-4	SEC	W5P707	ACTN4	0.026380369	0	M1_Mo
-4	SEC	W5P583	ACP1	0.050635084	0	M1_Mo

Supplementary Table 19: Shared and unique differentially abundant proteins (DAPs) between M1 and M2 relative to Mo and their cellular location.

FoldChange	Location	Protein IDs	Gene Names	P-value	FDR	Cell type
-4	SEC	W5QD49	PSME1	0.023404143	0	M1_Mo
-4	SEC	W5Q3M9	SEPTIN6	0.050777701	0	M1_Mo
-4	SEC	W5PQI3	ITGB3	0.046308029	0	M1_Mo
-4	SEC	W5PE11	CDC42	0.042181014	0	M1_Mo
-4	SEC	W5PPG3	ALDH9A1	0.02784853	0	M1_Mo
-4	SEC	W5PWG1	PFKP	0.04984226	0	M1_Mo
-4	SEC	W5QFH5	RAB1A	0.003342366	0	M1_Mo
-4	SEC	W5NQK6	LIMS1	0.017235241	0	M1_Mo
-4	SEC	W5PLS7	GRB2	0.017829769	0	M1_Mo
-4	SEC	W5P6Z6	MAPRE2	0.034634738	0	M1_Mo
-4	SEC	W5QFN2	HCLS1	0.033909594	0	M1_Mo
-4	SEC	W5PIJ6	PTPN11	0.045560855	0	M1_Mo
-4	SEC	W5Q731	ILK	0.023254682	0	M1_Mo
-4	SEC	W5Q0L1	EEF1G	0.043484379	0	M1_Mo
-4	SEC	W5NRD9		0.055440436	0.00159	M1_Mo
-4	SEC	W5QD96	PARVB	0.008083652	0	M1_Mo
-4	SEC	W5PVT6	UBA1	0.052704935	0	M1_Mo
-4	SEC	W5NY22	PCMT1	0.013883423	0	M1_Mo
-4	SEC	W5NUV1	GNB1	0.032196239	0	M1_Mo
-4	SEC	W5PSM0	OSTF1	0.028211191	0	M1_Mo
-4	SEC	W5PK13	CBX3	0.040076809	0	M1_Mo
-4	SEC	W5PY17	STX7	0.022594684	0	M1_Mo
-4	SEC	C5IWU0	ARF1	0.017895559	0	M1_Mo
-4	SEC	W5P4C7	SEPTIN7	0.034940513	0	M1_Mo
-5	SEC	W5PYM5	CCT8	0.02759104	0	M1_Mo
-5	SEC	W5PK85	EML2	0.046015246	0	M1_Mo
-5	SEC	W5P3N0	MAPRE1	0.041471138	0	M1_Mo
-5	SEC	Q5MIB5	PYGL	0.054998151	0	M1_Mo
-5	SEC	W5PK95	HNRNPA2B1	0.043739695	0	M1_Mo
-5	SEC	W5PK66	PARK7	0.036574128	0	M1_Mo
-5	SEC	W5PX84	CCDC171	0.043496543	0.004566	M1_Mo
-5	SEC	Q28554	GAPDH	0.035597607	0	M1_Mo
-5	SEC	W5PL19	LOC101105123	0.045653805	0	M1_Mo
-5	SEC	W5QHQ7	NCL	0.01758253	0	M1_Mo
-5	SEC	W5PEX2	GPATCH8	0.046568315	0	M1_Mo
-5	SEC	C5ISA2	TUBA4A	0.022555626	0	M1_Mo
-5	SEC	W5P3E8	GDI1	0.042917701	0	M1_Mo
-5	SEC	W5PPT6	TUBB	0.016362845	0	M1_Mo
-5	SEC	W5QE14	PCBP1	0.035980455	0	M1_Mo
-5	SEC	W5P765	CCT3	0.043481971	0	M1_Mo
-5	SEC	W5PRR5	LTA4H	0.008500307	0	M1_Mo
-5	SEC	W5PH15	RSU1	0.038265232	0	M1_Mo
-6	SEC	W5PLD5	CNN2	0.013887706	0	M1_Mo
-6	SEC	W5QAR2	F13A1	0.045572801	0	M1_Mo
-6	SEC	W5QG16	CAP1	0.04275264	0	M1_Mo
-6	SEC	W5PQA8	PRDX6	0.034714864	0	M1_Mo

Supplementary Table 19: Shared and unique differentially abundant proteins (DAPs) between M1 and M2 relative to Mo and their cellular location.

FoldChange	Location	Protein IDs	Gene Names	P-value	FDR	Cell type
-6	SEC	W5NUI0		0.00560145	0	M1_Mo
-6	SEC	W5PE27	ESD	0.021896574	0	M1_Mo
-6	SEC	W5PFI7	VCL	0.037231532	0	M1_Mo
-6	SEC	C5IJA0	RAN	0.016815047	0	M1_Mo
-6	SEC	W5PHW0	HSP90AB1	0.040993863	0	M1_Mo
-6	SEC	W5PK38	VASP	0.034074621	0	M1_Mo
-6	SEC	W5PG95	HSPA1A	0.022950833	0	M1_Mo
-6	SEC	W5P375	TCP1	0.000604349	0	M1_Mo
-7	SEC	W5QBD7	YWHAZ	0.011830852	0	M1_Mo
-7	SEC	W5P409	FERMT3	0.009831973	0	M1_Mo
-8	SEC	W5PQK6	TLN1	0.020680052	0	M1_Mo
-9	SEC	W5QF37	LPCAT4	0.032435455	0.008032	M1_Mo
10	CYTO	W5PCH3	SCIN	4.63E-05	0.000	M2_Mo
10	CYTO	W5PE67		3.02E-05	0.000	M2_Mo
9	CYTO	W5NU86	GLA	6.23E-04	0.000	M2_Mo
8	CYTO	W5PTU7	CA2	5.63E-06	0.000	M2_Mo
8	CYTO	W5PAM4	CTSA	4.54E-05	0.000	M2_Mo
8	CYTO	W5PXR1	ENPP1	1.12E-04	0.000	M2_Mo
8	CYTO	W5PT76	GPNMB	1.25E-04	0.000	M2_Mo
8	CYTO	W5PD43	HTRA1	5.73E-03	0.000	M2_Mo
8	CYTO	W5PF33	GM2A	7.80E-05	0.000	M2_Mo
8	CYTO	P35623	SHMT1	2.45E-05	0.000	M2_Mo
7	CYTO	W5Q6N3	LOC101115115	6.51E-05	0.000	M2_Mo
7	CYTO	W5NY01		9.30E-04	0.000	M2_Mo
7	CYTO	W5PQR0	NIBAN2	4.04E-06	0.000	M2_Mo
7	CYTO	A9YUY8	FABP4	3.61E-04	0.000	M2_Mo
7	CYTO	W5PFY5	ASAH1	1.30E-03	0.000	M2_Mo
7	CYTO	W5QHG1	EPS8	6.30E-03	0.000	M2_Mo
7	CYTO	W5P9J8	BLVRB	1.42E-04	0.000	M2_Mo
7	CYTO	W5PIQ6	MSR1	1.26E-04	0.000	M2_Mo
7	CYTO	W5QCD6	IDH1	1.49E-05	0.000	M2_Mo
7	CYTO	W5PEB0	FABP7	1.76E-03	0.000	M2_Mo
7	CYTO	W5Q9Y6	TCAF2	1.32E-05	0.000	M2_Mo
7	CYTO	P51977	ALDH1A1 ALDH1	2.45E-03	0.000	M2_Mo
6	CYTO	W5Q940	SHTN1	2.07E-03	0.000	M2_Mo
6	CYTO	W5Q612	TRPV2	2.37E-05	0.000	M2_Mo
6	CYTO	W5PRI6	MRC1	2.42E-04	0.000	M2_Mo
6	CYTO	W5PXX3	F13B	2.11E-09	0.007	M2_Mo
6	CYTO	W5QIW1	LGALS3	2.67E-03	0.000	M2_Mo
6	CYTO	W5PCE0	PLBD2	4.25E-03	0.000	M2_Mo
6	CYTO	W5NRS0		1.25E-04	0.000	M2_Mo
6	CYTO	W5Q2Y1	PLXNC1	5.79E-03	0.000	M2_Mo
6	CYTO	W5PAQ4	FUCA1	2.74E-04	0.000	M2_Mo
6	CYTO	W5QC89	HEXA	9.67E-05	0.000	M2_Mo
6	CYTO	W5PGC5	GALM	8.92E-04	0.000	M2_Mo
6	CYTO	W5Q0Y4	ZFAND6	4.36E-03	0.000	M2_Mo

Supplementary Table 19: Shared and unique differentially abundant proteins (DAPs) between M1 and M2 relative to Mo and their cellular location.

FoldChange	Location	Protein IDs	Gene Names	P-value	FDR	Cell type
6	CYTO	W5NVW7	NAGLU	2.85E-03	0.000	M2_Mo
6	CYTO	W5NPM4	TEX15	5.17E-04	0.003	M2_Mo
6	CYTO	W5QCL8	NPL	4.14E-04	0.000	M2_Mo
6	CYTO	W5NTZ3	RENBP	3.12E-04	0.000	M2_Mo
6	CYTO	W5PBM9	SCPEP1	3.07E-04	0.000	M2_Mo
6	CYTO	W5P8H9	SGPL1	5.20E-04	0.000	M2_Mo
6	CYTO	P05028	ATP1B1	1.93E-03	0.000	M2_Mo
6	CYTO	W5PFR8	FBP1	1.29E-04	0.000	M2_Mo
6	CYTO	W5NX56	SPP1	2.95E-04	0.000	M2_Mo
6	CYTO	W5QHC0	ST3GAL5	3.04E-04	0.000	M2_Mo
6	CYTO	W5PG10	PAPSS1	5.74E-03	0.000	M2_Mo
6	CYTO	W5NUI6	SGSH	6.14E-05	0.000	M2_Mo
6	CYTO	Q9MZS8	CTSD	1.40E-03	0.000	M2_Mo
6	CYTO	W5QBZ7	NAGA	2.26E-03	0.000	M2_Mo
6	CYTO	W5QI00	LACTB	4.37E-04	0.000	M2_Mo
6	CYTO	W5NZK6	PLA2G15	2.32E-04	0.000	M2_Mo
6	CYTO	W5PY08		4.29E-03	0.000	M2_Mo
6	CYTO	W5PBS4	LRP1	3.73E-04	0.000	M2_Mo
6	CYTO	W5PZG5	OCRL	7.83E-04	0.000	M2_Mo
5	CYTO	W5Q678	SEC24D	4.05E-03	0.000	M2_Mo
5	CYTO	W5PKY1	HNMT	6.87E-04	0.000	M2_Mo
5	CYTO	W5QEM8	LOC101111528	1.15E-04	0.000	M2_Mo
5	CYTO	W5QI40	MYO1E	2.87E-03	0.000	M2_Mo
5	CYTO	W5PYI8	WWC1	1.40E-04	0.000	M2_Mo
5	CYTO	W5PDJ0		4.03E-03	0.000	M2_Mo
5	CYTO	W5PPX2	SENP8	2.27E-05	0.003	M2_Mo
5	CYTO	W5QBG8	PPFIA1	1.96E-03	0.000	M2_Mo
5	CYTO	W5PFD2	HOOK1	2.44E-04	0.009	M2_Mo
5	CYTO	O18882	ATP6V0C ATP6C ATP(	6.55E-04	0.000	M2_Mo
5	CYTO	W5P5W6	NDRG1	1.34E-02	0.000	M2_Mo
5	CYTO	G3M9U4	ACP5	9.07E-04	0.000	M2_Mo
5	CYTO	W5QGG0	TFRC	3.53E-05	0.000	M2_Mo
5	CYTO	W5NYL7	MTHFD1L	6.44E-05	0.000	M2_Mo
5	CYTO	W5P4C9	MMP12	5.10E-02	0.000	M2_Mo
5	CYTO	W5QBM4	ALCAM	3.73E-04	0.000	M2_Mo
5	CYTO	W5P895	CRABP2	1.51E-03	0.000	M2_Mo
5	CYTO	W5Q5T7	ATP6V1C1	6.87E-03	0.000	M2_Mo
5	CYTO	W5P640	LMNA	3.25E-03	0.000	M2_Mo
5	CYTO	W5P8M9		3.17E-03	0.000	M2_Mo
5	CYTO	W5P9G8	PLD3	4.84E-02	0.000	M2_Mo
5	CYTO	W5PE91	LOC101105400	1.03E-04	0.000	M2_Mo
5	CYTO	W5PWX3	CRYL1	4.63E-04	0.000	M2_Mo
5	CYTO	W5NYU9	MPP1	1.59E-05	0.000	M2_Mo
5	CYTO	W5PV43	LRPAP1	1.24E-05	0.000	M2_Mo
5	CYTO	W5NQS7	IFNGR1	1.75E-04	0.000	M2_Mo
5	CYTO	W5NPP2	CPM	5.36E-04	0.000	M2_Mo

Supplementary Table 19: Shared and unique differentially abundant proteins (DAPs) between M1 and M2 relative to Mo and their cellular location.

FoldChange	Location	Protein IDs	Gene Names	P-value	FDR	Cell type
5	CYTO	W5QFU4		6.81E-03	0.000	M2_Mo
5	CYTO	W5NVR9	C21H11orf54	4.03E-06	0.000	M2_Mo
5	CYTO	W5Q5C8	SOAT1	2.11E-04	0.000	M2_Mo
5	CYTO	W5PGS4	FABP5	5.37E-04	0.000	M2_Mo
5	CYTO	W5P026	STAB1	1.14E-04	0.000	M2_Mo
5	CYTO	W5PV04	NIFK	2.69E-04	0.000	M2_Mo
5	CYTO	W5P0C5	LGALS8	6.13E-05	0.000	M2_Mo
5	CYTO	W5PDH7	NPC1	3.21E-04	0.000	M2_Mo
5	CYTO	W5P1A5	GBA1	3.84E-03	0.000	M2_Mo
5	CYTO	P83205	CTSB	3.05E-03	0.000	M2_Mo
5	CYTO	W5NZ62	GNS	4.12E-03	0.000	M2_Mo
5	CYTO	W5PI56	DAB2	4.51E-03	0.000	M2_Mo
5	CYTO	W5PBC0		3.05E-03	0.000	M2_Mo
5	CYTO	Q29524	LPL	3.45E-03	0.000	M2_Mo
5	CYTO	W5Q3U3	LOC101102156	6.95E-03	0.000	M2_Mo
5	CYTO	W5PE92	GRN	5.97E-04	0.000	M2_Mo
5	CYTO	W5PYW0	TCIRG1	3.39E-04	0.000	M2_Mo
4	CYTO	W5P1H0	CTSC	5.05E-06	0.000	M2_Mo
4	CYTO	W5Q6S3	WASHC4	1.00E-03	0.000	M2_Mo
4	CYTO	W5P2V3	PEPD	2.36E-03	0.000	M2_Mo
4	CYTO	W5QEH0	TWF1	2.22E-03	0.000	M2_Mo
4	CYTO	W5PZB2	CD68	5.65E-03	0.000	M2_Mo
4	CYTO	W5P5Q2	MVP	1.11E-02	0.000	M2_Mo
4	CYTO	W5Q0F1	LIPA	2.60E-04	0.000	M2_Mo
4	CYTO	W5NWP6	DIAPH2	4.57E-03	0.000	M2_Mo
4	CYTO	W5PP04	GNG12	6.48E-04	0.000	M2_Mo
4	CYTO	W5QH69	RNF181	8.63E-04	0.000	M2_Mo
4	CYTO	W5P168	SH3PXD2B	2.33E-05	0.000	M2_Mo
4	CYTO	W5P703	WFS1	1.21E-03	0.000	M2_Mo
4	CYTO	W5NUC8	ARMCX3	6.68E-04	0.000	M2_Mo
4	CYTO	W5Q8J3	RRBP1	6.81E-03	0.000	M2_Mo
4	CYTO	W5Q777	HS1BP3	1.92E-04	0.000	M2_Mo
4	CYTO	W5PKU3		1.12E-03	0.000	M2_Mo
4	CYTO	W5P530	LOC101104705	4.09E-02	0.000	M2_Mo
4	CYTO	W5QB71	AMDHD2	1.15E-03	0.000	M2_Mo
4	CYTO	W5Q9L2	LOC101109820	2.15E-03	0.000	M2_Mo
4	CYTO	W5P3N6	LOC101112162	6.76E-04	0.000	M2_Mo
4	CYTO	W5NSH8	NPC2	1.07E-05	0.000	M2_Mo
4	CYTO	W5PXS1	RAB3IL1	1.06E-03	0.000	M2_Mo
4	CYTO	W5PEE9	LAMP1	1.19E-02	0.000	M2_Mo
4	CYTO	W5P078		1.98E-03	0.000	M2_Mo
4	CYTO	W5P4K6	FCHO2	2.42E-04	0.000	M2_Mo
4	CYTO	W5P7Y8	PALD1	1.48E-04	0.000	M2_Mo
4	CYTO	W5PCM4	LUZP1	1.35E-04	0.000	M2_Mo
4	CYTO	W5PDZ1		1.80E-03	0.000	M2_Mo
4	CYTO	W5QH35	CAPG	4.24E-04	0.000	M2_Mo

Supplementary Table 19: Shared and unique differentially abundant proteins (DAPs) between M1 and M2 relative to Mo and their cellular location.

FoldChange	Location	Protein IDs	Gene Names	P-value	FDR	Cell type
4	CYTO	W5QCM1	ARFGAP3	1.17E-02	0.000	M2_Mo
4	CYTO	W5Q263	ICAM1	7.94E-04	0.000	M2_Mo
4	CYTO	W5PBJ4	ARHGAP10	3.56E-03	0.000	M2_Mo
4	CYTO	W5NX16		9.15E-04	0.000	M2_Mo
4	CYTO	W5Q8V2	LIMA1	1.32E-04	0.000	M2_Mo
4	CYTO	W5QI78	CTSK	3.92E-02	0.000	M2_Mo
4	CYTO	W5Q1M0	GLB1	1.41E-04	0.000	M2_Mo
4	CYTO	W5PEZ1		2.72E-04	0.000	M2_Mo
4	CYTO	W5NW80	GAA	2.51E-03	0.000	M2_Mo
4	CYTO	W5PVH4	TMEM251	5.11E-04	0.000	M2_Mo
4	CYTO	W5PVR9	ERMP1	5.48E-03	0.000	M2_Mo
4	CYTO	W5PTG5	ATP6V0D2	5.43E-02	0.000	M2_Mo
4	CYTO	W5P4X6	LOC101104287	4.04E-03	0.000	M2_Mo
4	CYTO	W5PF85	PEA15	9.72E-05	0.000	M2_Mo
4	CYTO	W5Q5W2	LOC101110539	5.18E-05	0.000	M2_Mo
4	CYTO	P12303	TTR	1.72E-02	0.000	M2_Mo
4	CYTO	W5Q700	APPL2	5.28E-05	0.000	M2_Mo
4	CYTO	W5NYU6	NT5DC2	1.34E-03	0.000	M2_Mo
4	CYTO	W5PZD7		1.18E-04	0.000	M2_Mo
4	CYTO	W5P3S0		1.26E-04	0.000	M2_Mo
4	CYTO	W5NSS6		1.16E-02	0.000	M2_Mo
4	CYTO	W5NU23	FUCA2	5.16E-04	0.000	M2_Mo
4	CYTO	W5PUL5	FCGRT	2.29E-03	0.000	M2_Mo
4	CYTO	W5PG28	NTMT1	1.25E-03	0.000	M2_Mo
4	CYTO	W5PAZ0	RGCC	2.18E-04	0.000	M2_Mo
4	CYTO	W5PFB1	TOR1B	3.13E-03	0.000	M2_Mo
4	CYTO	C5IJ93	RAB9A	7.67E-04	0.000	M2_Mo
4	CYTO	W5PBM2	LOC101110634	1.36E-03	0.000	M2_Mo
4	CYTO	W5P7R2	HECTD1	2.68E-04	0.000	M2_Mo
4	CYTO	W5NZV3	HMOX2	3.27E-02	0.000	M2_Mo
4	CYTO	W5QEH8	CTTNBP2NL	2.45E-03	0.000	M2_Mo
4	CYTO	W5NRL0	PLCD1	1.82E-04	0.000	M2_Mo
4	CYTO	W5NYK1	PMVK	9.70E-04	0.000	M2_Mo
4	CYTO	W5NQZ9	GSDMD	9.35E-04	0.000	M2_Mo
4	CYTO	W5Q2J4	DIP2C	1.94E-05	0.000	M2_Mo
4	CYTO	W5PT36	RBM47	9.56E-04	0.000	M2_Mo
4	CYTO	W5Q2U7	PLEC	1.02E-06	0.000	M2_Mo
4	CYTO	W5NPW8	STARD4	1.41E-03	0.000	M2_Mo
4	CYTO	W5NWX7	CLEC4A	2.96E-04	0.000	M2_Mo
4	CYTO	W5NV37	COMMD10	9.93E-03	0.000	M2_Mo
4	CYTO	W5P536	TRIP10	3.84E-04	0.000	M2_Mo
4	CYTO	W5QHU8	FNDC3B	7.51E-05	0.000	M2_Mo
4	CYTO	W5Q0E7	LIN7C	8.72E-04	0.006	M2_Mo
4	CYTO	W5PAC2	LOC101105044	1.43E-02	0.000	M2_Mo
4	CYTO	W5PN65	PI4K2A	1.16E-03	0.000	M2_Mo
4	CYTO	Q9MZD1	SLC17A5	2.83E-03	0.000	M2_Mo

Supplementary Table 19: Shared and unique differentially abundant proteins (DAPs) between M1 and M2 relative to Mo and their cellular location.

FoldChange	Location	Protein IDs	Gene Names	P-value	FDR	Cell type
4	CYTO	W5PDU4	NMT2	1.09E-03	0.000	M2_Mo
4	CYTO	W5PDH4	MMP9	2.86E-03	0.000	M2_Mo
4	CYTO	W5PFE6	ACOX1	1.05E-03	0.000	M2_Mo
4	CYTO	W5PKF9	FIG4	7.92E-04	0.000	M2_Mo
4	CYTO	W5PI67	IDS	2.95E-03	0.000	M2_Mo
4	CYTO	W5NYI4	RIPOR1	4.85E-04	0.000	M2_Mo
4	CYTO	W5PUI3	GOLGA1	1.57E-05	0.000	M2_Mo
4	CYTO	W5PAJ2	PSAP	6.47E-05	0.000	M2_Mo
4	CYTO	W5PUM5	KANK1	9.80E-05	0.000	M2_Mo
4	CYTO	W5Q9M9	GK	2.93E-02	0.000	M2_Mo
4	CYTO	W5Q000	RPL32	1.15E-04	0.000	M2_Mo
4	CYTO	W5QI95	CERS2	3.64E-03	0.000	M2_Mo
4	CYTO	W5P4A8	RNASET2	3.62E-04	0.000	M2_Mo
4	CYTO	W5Q6C5	GGA2	6.45E-03	0.000	M2_Mo
4	CYTO	W5PN70		4.44E-03	0.000	M2_Mo
4	CYTO	W5PBG1		9.00E-03	0.000	M2_Mo
4	CYTO	W5PIE4	CLPTM1	2.68E-02	0.000	M2_Mo
4	CYTO	W5Q1W7	PALLD	3.94E-03	0.000	M2_Mo
4	CYTO	W5P1Q0	AP1B1	2.78E-05	0.000	M2_Mo
4	CYTO	W5NYW8	CEP55	1.23E-04	0.000	M2_Mo
4	CYTO	W5P3W6	OSBPL9	3.07E-04	0.000	M2_Mo
4	CYTO	W5QA16		1.89E-04	0.000	M2_Mo
4	CYTO	W5Q175	GNPTAB	1.07E-03	0.000	M2_Mo
4	CYTO	W5PQM4	MOSPD2	3.76E-04	0.000	M2_Mo
4	CYTO	W5Q6X2	TNFRSF1B	4.33E-04	0.000	M2_Mo
3	CYTO	W5QEE5	TRAPPC3	5.03E-02	0.000	M2_Mo
3	CYTO	W5QBJ2	SLC38A10	2.95E-04	0.000	M2_Mo
3	CYTO	W5Q0A3	TLR2	4.71E-03	0.000	M2_Mo
3	CYTO	W5PIS6	NHLRC3	4.24E-03	0.000	M2_Mo
3	CYTO	W5PA90	AGA	7.50E-05	0.000	M2_Mo
3	CYTO	W5P3L5	RNF13	4.03E-04	0.000	M2_Mo
3	CYTO	W5PX29	TMEM9B	5.17E-02	0.000	M2_Mo
3	CYTO	W5PQP3	LIMK1	1.51E-04	0.000	M2_Mo
3	CYTO	W5P743	GLMP	2.23E-03	0.000	M2_Mo
3	CYTO	W5P2F1	FOLR2	7.50E-06	0.000	M2_Mo
3	CYTO	W5NYL0	MAOA	2.43E-02	0.000	M2_Mo
3	CYTO	W5QCF3	SLC35F6	4.02E-03	0.000	M2_Mo
3	CYTO	W5P093	NQO1	4.42E-04	0.000	M2_Mo
3	CYTO	W5Q2K9	CYFIP1	6.37E-05	0.000	M2_Mo
3	CYTO	W5QBV7	CD44	4.25E-05	0.000	M2_Mo
3	CYTO	W5NS94		7.15E-05	0.000	M2_Mo
3	CYTO	W5PJY6	ADAM28	2.40E-03	0.000	M2_Mo
3	CYTO	W5PLB8	EPB41L3	1.77E-03	0.000	M2_Mo
3	CYTO	W5QIC7	YBX3	2.17E-04	0.000	M2_Mo
3	CYTO	W5Q280		1.81E-02	0.000	M2_Mo
3	CYTO	W5NQ23	WIPF1	5.80E-03	0.000	M2_Mo

Supplementary Table 19: Shared and unique differentially abundant proteins (DAPs) between M1 and M2 relative to Mo and their cellular location.

FoldChange	Location	Protein IDs	Gene Names	P-value	FDR	Cell type
3	CYTO	W5Q5Q7	ASPA	1.84E-02	0.000	M2_Mo
3	CYTO	W5PTE6	CEMIP2	3.83E-03	0.000	M2_Mo
3	CYTO	W5PXK7	RASA4B	3.00E-03	0.000	M2_Mo
3	CYTO	W5PEL8	PDXDC1	1.89E-03	0.000	M2_Mo
3	CYTO	W5PCD0	FUBP3	2.14E-02	0.000	M2_Mo
3	CYTO	W5QBR5	BMP2K	5.17E-03	0.000	M2_Mo
3	CYTO	W5NXZ9	RASSF4	5.02E-03	0.000	M2_Mo
3	CYTO	W5P3C6	LOC101111906	1.28E-05	0.000	M2_Mo
3	CYTO	W5NTW0	CADM1	2.03E-02	0.000	M2_Mo
3	CYTO	W5QG92	OSBPL11	2.67E-04	0.000	M2_Mo
3	CYTO	W5Q2V0	YKT6	3.66E-05	0.000	M2_Mo
3	CYTO	W5Q3N1	CTSZ	5.89E-05	0.000	M2_Mo
3	CYTO	W5PGX8	DNAJC13	3.84E-02	0.000	M2_Mo
3	CYTO	W5PES0	STX4	7.73E-05	0.000	M2_Mo
3	CYTO	W5P434	NAGPA	1.81E-03	0.000	M2_Mo
3	CYTO	W5QGF4	VPS18	4.11E-03	0.000	M2_Mo
3	CYTO	W5PGC6	DPP9	3.39E-02	0.000	M2_Mo
3	CYTO	W5PT68	FLNB	4.88E-02	0.000	M2_Mo
3	CYTO	W5PQ47	RAI14	1.51E-02	0.000	M2_Mo
3	CYTO	W5PHY4	TYMS	5.36E-03	0.000	M2_Mo
3	CYTO	W5PH26		1.95E-03	0.000	M2_Mo
3	CYTO	W5Q740	ABCD3	9.50E-03	0.000	M2_Mo
3	CYTO	W5QAK7	SCARB1	9.07E-05	0.000	M2_Mo
3	CYTO	Q10994	CSTB CST6	5.95E-05	0.000	M2_Mo
3	CYTO	W5Q568	TRANK1	6.83E-05	0.006	M2_Mo
3	CYTO	W5PGF4	PLAUR	1.64E-02	0.000	M2_Mo
3	CYTO	W5PCC0	LACC1	2.71E-03	0.000	M2_Mo
3	CYTO	W5PBB5	CTSF	3.55E-03	0.000	M2_Mo
3	CYTO	W5P6V4	GLG1	5.23E-02	0.000	M2_Mo
3	CYTO	W5PUM8	HIP1	5.88E-04	0.000	M2_Mo
3	CYTO	W5QH03	LPP	2.00E-02	0.000	M2_Mo
3	CYTO	W5Q8Y5	HDLBP	2.01E-03	0.000	M2_Mo
3	CYTO	W5PPY9		1.06E-02	0.000	M2_Mo
3	CYTO	W5Q8I7	LOC101122123	2.25E-03	0.000	M2_Mo
3	CYTO	W5PEX1	WASHC5	9.71E-03	0.000	M2_Mo
3	CYTO	W5QCP4	TRAF6	8.42E-04	0.000	M2_Mo
3	CYTO	W5P4Q3	MSH6	3.46E-04	0.002	M2_Mo
3	CYTO	W5QI36	HEBP1	3.25E-02	0.000	M2_Mo
3	CYTO	W5PHG6	SMYD2	1.83E-03	0.000	M2_Mo
3	CYTO	W5PVH8	ATP6V1E1	6.77E-05	0.000	M2_Mo
3	CYTO	W5Q3C2	EPPK1	5.68E-03	0.000	M2_Mo
3	CYTO	W5NS73		2.42E-03	0.003	M2_Mo
3	CYTO	W5PE73	SMPDL3A	2.68E-02	0.000	M2_Mo
3	CYTO	W5PRG8	CREG1	1.04E-02	0.000	M2_Mo
3	CYTO	W5Q5K0		5.06E-03	0.000	M2_Mo
3	CYTO	W5Q2R5	SDF2L1	4.70E-02	0.000	M2_Mo

Supplementary Table 19: Shared and unique differentially abundant proteins (DAPs) between M1 and M2 relative to Mo and their cellular location.

FoldChange	Location	Protein IDs	Gene Names	P-value	FDR	Cell type
3	CYTO	W5NSQ3	HFE	2.45E-03	0.000	M2_Mo
3	CYTO	W5PIR3	BLZF1	4.37E-04	0.006	M2_Mo
3	CYTO	W5PC32	PGM3	6.11E-03	0.000	M2_Mo
3	CYTO	W5Q728	TPCN2	8.93E-04	0.000	M2_Mo
3	CYTO	W5PJS4	EMILIN2	1.90E-02	0.000	M2_Mo
3	CYTO	W5QHR5	PLEKHO2	1.65E-03	0.000	M2_Mo
3	CYTO	W5PSP1	ERC1	2.02E-02	0.000	M2_Mo
3	CYTO	W5QEL9	ARHGAP31	5.51E-03	0.000	M2_Mo
3	CYTO	W5QAQ4	NRP2	1.20E-03	0.000	M2_Mo
3	CYTO	W5PI02	TBC1D13	1.23E-02	0.000	M2_Mo
3	CYTO	W5QJ37		2.42E-04	0.000	M2_Mo
3	CYTO	W5QAA7	MAPK7	6.49E-04	0.000	M2_Mo
3	CYTO	P82197	PDXK PKH	2.14E-02	0.000	M2_Mo
3	CYTO	W5NXT6	UBR3	2.76E-03	0.000	M2_Mo
3	CYTO	W5PBX4	FAR1	2.21E-03	0.000	M2_Mo
3	CYTO	W5P1M3		2.04E-03	0.000	M2_Mo
3	CYTO	W5QEL6	PCYOX1	1.53E-02	0.000	M2_Mo
3	CYTO	W5PJ58	HPS5	1.40E-03	0.000	M2_Mo
3	CYTO	W5QI70	CTSS	1.61E-04	0.000	M2_Mo
3	CYTO	W5Q5C7	CTNNA1	3.23E-02	0.000	M2_Mo
3	CYTO	W5PQ75	HSPH1	1.08E-02	0.000	M2_Mo
3	CYTO	W5Q3B4	TBCD	1.54E-03	0.000	M2_Mo
3	CYTO	W5Q3Y3	SFXN3	5.16E-03	0.000	M2_Mo
3	CYTO	W5NPP0		6.08E-03	0.000	M2_Mo
3	CYTO	W5P5P8	PSMG3	4.36E-02	0.000	M2_Mo
3	CYTO	W5Q0C3	KIF13B	3.21E-02	0.000	M2_Mo
3	CYTO	W5P3I5	CNDP2	3.36E-04	0.000	M2_Mo
3	CYTO	W5QHD4	EIF4G1	5.10E-02	0.000	M2_Mo
3	CYTO	W5Q644	VPS29	2.63E-02	0.000	M2_Mo
3	CYTO	W5P369	AP2A2	5.25E-06	0.000	M2_Mo
3	CYTO	W5PVG5	BLTP3B	2.58E-04	0.000	M2_Mo
3	CYTO	W5PPK8		6.25E-03	0.000	M2_Mo
3	CYTO	W5Q552	ANAPC7	3.91E-02	0.000	M2_Mo
3	CYTO	W5PYE8	MERTK	4.30E-03	0.000	M2_Mo
3	CYTO	W5PUL4	MTMR6	1.96E-02	0.000	M2_Mo
3	CYTO	W5NUU1	VRK2	5.53E-03	0.000	M2_Mo
3	CYTO	W5PQV2	LMBRD1	3.49E-05	0.000	M2_Mo
3	CYTO	W5PER8	WDR91	1.89E-02	0.000	M2_Mo
3	CYTO	W5Q553	ITGAV	1.60E-03	0.000	M2_Mo
3	CYTO	B9VGZ7	SLIRP	2.34E-02	0.000	M2_Mo
3	CYTO	W5PVE3	LOC101115252	4.57E-05	0.000	M2_Mo
3	CYTO	W5QHL1	FCGR1A	3.40E-02	0.000	M2_Mo
3	CYTO	W5QH60	VAMP8	1.12E-02	0.000	M2_Mo
3	CYTO	W5QET8	TEP1	4.20E-02	0.000	M2_Mo
3	CYTO	W5PYH6	AP3D1	8.18E-03	0.000	M2_Mo
3	CYTO	W5PWA8	HSPB1	3.71E-03	0.000	M2_Mo

Supplementary Table 19: Shared and unique differentially abundant proteins (DAPs) between M1 and M2 relative to Mo and their cellular location.

FoldChange	Location	Protein IDs	Gene Names	P-value	FDR	Cell type
3	CYTO	W5NZ71		2.20E-02	0.000	M2_Mo
3	CYTO	W5P3H1	LOC101111732	5.25E-03	0.000	M2_Mo
3	CYTO	W5PBX0	ATP11A	4.40E-02	0.000	M2_Mo
3	CYTO	W5Q0J1	PLA2G6	5.15E-03	0.005	M2_Mo
3	CYTO	W5NR48	KPNA6	3.05E-02	0.000	M2_Mo
3	CYTO	W5PB38		2.26E-03	0.000	M2_Mo
3	CYTO	W5PBR7	P4HA1	1.53E-02	0.000	M2_Mo
3	CYTO	W5PYB9	ABCA6	1.02E-03	0.000	M2_Mo
3	CYTO	W5PZ62	ZFYVE16	1.34E-02	0.000	M2_Mo
3	CYTO	W5PKA1	LOC101116157	4.25E-03	0.000	M2_Mo
3	CYTO	W5PNG4	BRCA2	4.32E-02	0.005	M2_Mo
3	CYTO	W5PC06	SIRPA	2.45E-04	0.000	M2_Mo
3	CYTO	W5Q420		1.35E-02	0.000	M2_Mo
3	CYTO	W5NRA9	ASL	1.87E-02	0.000	M2_Mo
3	CYTO	W5Q0G8	IMPA1	5.88E-04	0.000	M2_Mo
3	CYTO	W5PZ94	ACO1	5.49E-02	0.000	M2_Mo
3	CYTO	W5PAX1	GCLC	2.84E-02	0.000	M2_Mo
3	CYTO	W5P1U3	LACTB2	3.09E-02	0.000	M2_Mo
3	CYTO	W5NPU0	NAPRT	8.23E-03	0.000	M2_Mo
3	CYTO	W5PHX8	GLRX2	8.37E-03	0.000	M2_Mo
3	CYTO	W5QCG9	SRXN1	4.22E-02	0.000	M2_Mo
3	CYTO	W5PF73	PLAU	2.11E-03	0.000	M2_Mo
3	CYTO	W5PH35	LOC101119706	2.35E-03	0.000	M2_Mo
3	CYTO	W5PQZ5	GDE1	2.75E-03	0.000	M2_Mo
3	CYTO	W5Q8T1	CLPX	1.53E-02	0.000	M2_Mo
3	CYTO	W5PMA0	AP2S1	1.11E-02	0.000	M2_Mo
3	CYTO	W5Q3A2		3.78E-02	0.000	M2_Mo
3	CYTO	W5QDC0	SNX17	2.83E-02	0.000	M2_Mo
3	CYTO	Q9XT28	ATOX1	8.19E-04	0.000	M2_Mo
3	CYTO	W5NQ85	IDE	3.32E-02	0.000	M2_Mo
3	CYTO	W5P1T1	ANO10	9.96E-03	0.000	M2_Mo
3	CYTO	W5PC57	MRTFB	4.69E-02	0.000	M2_Mo
3	CYTO	W5QJ45	GPHN	4.96E-03	0.000	M2_Mo
3	CYTO	W5NU07		1.24E-02	0.000	M2_Mo
3	CYTO	W5PRM9	BCAT2	5.35E-02	0.000	M2_Mo
3	CYTO	W5PN60	ABR	2.26E-02	0.000	M2_Mo
3	CYTO	W5P7X7	MAGED2	3.52E-02	0.000	M2_Mo
3	CYTO	W5NRI6	PLPBP PROSC	5.60E-03	0.000	M2_Mo
3	CYTO	W5P8A0		4.13E-02	0.000	M2_Mo
3	CYTO	W5NT95	ATP6AP2	3.02E-04	0.000	M2_Mo
3	CYTO	W5PER3	TM9SF3	1.98E-02	0.000	M2_Mo
3	CYTO	W5P9L5	RASA2	2.39E-03	0.000	M2_Mo
3	CYTO	W5PS50	MPC2	1.70E-02	0.000	M2_Mo
3	CYTO	W5QIA5	ETV6	1.38E-02	0.000	M2_Mo
3	CYTO	W5Q3R4	CLUH	4.35E-05	0.000	M2_Mo
3	CYTO	W5P3H9	PICALM	1.11E-05	0.000	M2_Mo

Supplementary Table 19: Shared and unique differentially abundant proteins (DAPs) between M1 and M2 relative to Mo and their cellular location.

FoldChange	Location	Protein IDs	Gene Names	P-value	FDR	Cell type
3	CYTO	W5PJH6		1.48E-02	0.000	M2_Mo
3	CYTO	W5NVV6	DNAJC3	4.13E-02	0.000	M2_Mo
3	CYTO	W5P5E7		3.90E-03	0.000	M2_Mo
3	CYTO	W5Q5Z1	YTHDF3	8.28E-03	0.000	M2_Mo
3	CYTO	W5PAM5		2.47E-04	0.000	M2_Mo
3	CYTO	W5QIU9	S100A10	6.80E-03	0.000	M2_Mo
3	CYTO	W5PJN0	PSPH	2.45E-02	0.000	M2_Mo
3	CYTO	W5P4B3	NRDC	1.10E-02	0.000	M2_Mo
3	CYTO	W5NUQ8	GCC1	2.41E-02	0.000	M2_Mo
3	CYTO	W5QH13	VPS39	2.92E-03	0.000	M2_Mo
3	CYTO	W5Q3D8	CLASP2	9.11E-03	0.000	M2_Mo
3	CYTO	W5PA78	AVL9	1.51E-02	0.000	M2_Mo
3	CYTO	W5PJJ3	LARP1	2.60E-02	0.000	M2_Mo
3	CYTO	W5PVQ4	TMEM120A	6.69E-03	0.000	M2_Mo
3	CYTO	W5QIA8	YARS1	8.49E-04	0.000	M2_Mo
3	CYTO	W5Q4N4	TOR3A	7.86E-03	0.000	M2_Mo
3	CYTO	W5NV06	ATP6V0A1	3.67E-02	0.000	M2_Mo
3	CYTO	W5PCK8	LOC100135455	1.30E-03	0.000	M2_Mo
3	CYTO	W5NUJ2	PEAK1	9.20E-03	0.000	M2_Mo
3	CYTO	W5NWX4		5.43E-02	0.000	M2_Mo
3	CYTO	W5NQG2	TBC1D2B	1.44E-05	0.000	M2_Mo
3	CYTO	W5Q8I6	POLDIP2	1.12E-02	0.000	M2_Mo
3	CYTO	W5PIN6	LDHA	4.07E-04	0.000	M2_Mo
3	CYTO	W5P1S3	SLC25A13	4.39E-03	0.000	M2_Mo
3	CYTO	W5QEG2	MAP7D1	5.28E-03	0.000	M2_Mo
3	CYTO	W5Q8B9	STK25	1.32E-02	0.000	M2_Mo
3	CYTO	W5PWR6	KCNAB2	3.55E-03	0.000	M2_Mo
3	CYTO	W5PF22	KMO	1.30E-02	0.000	M2_Mo
3	CYTO	W5PK12	OAT	5.99E-05	0.000	M2_Mo
3	CYTO	W5Q3L8	LOC101104306	1.92E-03	0.000	M2_Mo
3	CYTO	W5P560	PIK3C3	5.29E-02	0.000	M2_Mo
2	CYTO	W5PUW5	GOLGA5	1.42E-02	0.000	M2_Mo
2	CYTO	W5QEL5	MRPS35	5.53E-02	0.000	M2_Mo
2	CYTO	W5NSS0	ARAP3	4.95E-03	0.000	M2_Mo
2	CYTO	W5QCA6	UBE2F	6.32E-05	0.000	M2_Mo
2	CYTO	W5PSG0		6.36E-03	0.000	M2_Mo
2	CYTO	W5PXT9	DENND4C	2.80E-03	0.000	M2_Mo
2	CYTO	W5PUW2	IFI30	3.57E-03	0.000	M2_Mo
2	CYTO	W5QDT8		9.11E-03	0.000	M2_Mo
2	CYTO	W5P7L2	ATP6V1G1	8.09E-05	0.000	M2_Mo
2	CYTO	W5PHI1	MRPL3	1.98E-02	0.000	M2_Mo
2	CYTO	W5QAT6	MTHFR	9.77E-04	0.000	M2_Mo
2	CYTO	W5PVA4	ABCF1	2.55E-02	0.000	M2_Mo
2	CYTO	W5QIJ6	SPPL2A	4.55E-02	0.000	M2_Mo
2	CYTO	W5NY99		6.74E-03	0.000	M2_Mo
2	CYTO	W5P3X8	KIF15	5.24E-04	0.000	M2_Mo

Supplementary Table 19: Shared and unique differentially abundant proteins (DAPs) between M1 and M2 relative to Mo and their cellular location.

FoldChange	Location	Protein IDs	Gene Names	P-value	FDR	Cell type
2	CYTO	W5P4L4	TMX3	4.54E-03	0.000	M2_Mo
2	CYTO	W5PGL9		4.40E-03	0.000	M2_Mo
2	CYTO	W5PZS4	OSBPL8	1.39E-02	0.000	M2_Mo
2	CYTO	W5PNW7	VIM	9.95E-06	0.000	M2_Mo
2	CYTO	W5P2N4	AHCYL2	3.29E-02	0.000	M2_Mo
2	CYTO	W5QFK2	MACF1	3.13E-02	0.000	M2_Mo
2	CYTO	W5PTR6	ACOX3	5.47E-03	0.000	M2_Mo
2	CYTO	W5QDF4	GSTM3	6.87E-03	0.000	M2_Mo
2	CYTO	W5Q758	DCTN5	3.97E-02	0.000	M2_Mo
2	CYTO	P81184	LGALS1	1.83E-04	0.000	M2_Mo
2	CYTO	W5Q5B5	CSAD	3.46E-04	0.000	M2_Mo
2	CYTO	W5QA59	HGS	1.98E-02	0.000	M2_Mo
2	CYTO	W5Q8K4	SLC3A2	6.73E-03	0.000	M2_Mo
2	CYTO	W5PLZ0	ATP6V1B2	4.11E-04	0.000	M2_Mo
2	CYTO	W5PRS4	FKBP5	2.13E-02	0.000	M2_Mo
2	CYTO	W5PUW9	PDCL3	1.89E-02	0.000	M2_Mo
2	CYTO	W5QEQ1	TRABD	4.20E-02	0.000	M2_Mo
2	CYTO	W5QHV8	PLD1	1.65E-02	0.000	M2_Mo
2	CYTO	W5NZ70	LGALS3BP	3.97E-02	0.000	M2_Mo
2	CYTO	W5PKS2	SARAF	4.62E-02	0.000	M2_Mo
2	CYTO	W5Q501	LOC101115640	1.61E-03	0.000	M2_Mo
2	CYTO	W5PWF2	ATP6V1H	9.15E-04	0.000	M2_Mo
2	CYTO	W5QC34	MAN2A1	2.94E-03	0.000	M2_Mo
2	CYTO	W5PMH1	GSS	3.97E-03	0.000	M2_Mo
2	CYTO	W5NSE1	DRG2	2.13E-03	0.000	M2_Mo
2	CYTO	W5Q8J8	VPS41	1.79E-03	0.000	M2_Mo
2	CYTO	W5P867	MAPKAPK2	3.38E-03	0.000	M2_Mo
2	CYTO	W5QAE8	ALDH7A1	8.12E-05	0.000	M2_Mo
2	CYTO	W5QA65	ZW10	2.81E-02	0.000	M2_Mo
2	CYTO	W5NUE3	PRDX1	6.56E-04	0.000	M2_Mo
2	CYTO	W5NWF5	RARRES1	4.66E-03	0.000	M2_Mo
2	CYTO	W5PRJ7	CCDC93	5.65E-03	0.000	M2_Mo
2	CYTO	W5NWR6	LXN	4.89E-02	0.000	M2_Mo
2	CYTO	W5QDF2	ATG3	1.25E-02	0.000	M2_Mo
2	CYTO	W5P4G4	RAB7B	2.00E-02	0.000	M2_Mo
2	CYTO	W5NYK9	CALU	2.08E-02	0.000	M2_Mo
2	CYTO	W5QD46	CYTH4	1.51E-02	0.000	M2_Mo
2	CYTO	W5P3A2		2.46E-03	0.000	M2_Mo
2	CYTO	W5PWB8	ARHGAP12	1.34E-02	0.000	M2_Mo
2	CYTO	W5PC18		1.06E-02	0.000	M2_Mo
2	CYTO	W5P3H8	IGF2R	1.91E-02	0.000	M2_Mo
2	CYTO	W5QJ49	ATP6V1D	5.72E-04	0.000	M2_Mo
2	CYTO	W5Q928	PCYT2	1.12E-02	0.000	M2_Mo
2	CYTO	W5P1K2	TRAPPC9	9.77E-05	0.000	M2_Mo
2	CYTO	P04074	ATP1A1	2.68E-03	0.000	M2_Mo
2	CYTO	W5PPR1	UCK2	1.14E-02	0.000	M2_Mo

Supplementary Table 19: Shared and unique differentially abundant proteins (DAPs) between M1 and M2 relative to Mo and their cellular location.

FoldChange	Location	Protein IDs	Gene Names	P-value	FDR	Cell type
2	CYTO	W5NRF7	MAP3K20	1.37E-03	0.000	M2_Mo
2	CYTO	Q6XUZ5	IDH1	2.04E-05	0.000	M2_Mo
2	CYTO	Q6XXL8	DYNLT3 TCTE1L	7.89E-03	0.000	M2_Mo
2	CYTO	W5P7E8		4.72E-05	0.000	M2_Mo
2	CYTO	W5P1T4	PPP4C	1.48E-03	0.000	M2_Mo
2	CYTO	W5QIK3	USP8	1.18E-02	0.000	M2_Mo
2	CYTO	W5PKV1	DNASE2	2.32E-02	0.000	M2_Mo
2	CYTO	W5PEN2	TPD52	6.62E-03	0.000	M2_Mo
2	CYTO	W5Q3P7	ITFG1	1.56E-02	0.000	M2_Mo
2	CYTO	W5QBW4	VAV3	5.59E-04	0.000	M2_Mo
2	CYTO	W5PZN5	KIF3A	4.61E-02	0.000	M2_Mo
2	CYTO	W5P7L0	SUN1	6.80E-03	0.000	M2_Mo
2	CYTO	W5Q4K4	EVI5	1.10E-02	0.000	M2_Mo
2	CYTO	W5Q3C1	NDRG3	1.71E-03	0.000	M2_Mo
2	CYTO	W5P4Z8	MRPS27	3.99E-02	0.000	M2_Mo
2	CYTO	W5PUJ2	LAMP2	4.93E-03	0.000	M2_Mo
2	CYTO	W5PP47		2.24E-04	0.000	M2_Mo
2	CYTO	W5PI27	UEVLD	2.19E-03	0.000	M2_Mo
2	CYTO	W5Q540	HAGH	7.92E-03	0.000	M2_Mo
2	CYTO	W5P8E1	DNPH1 RCL	4.41E-02	0.000	M2_Mo
2	CYTO	W5QEU6	ANXA4	3.06E-02	0.000	M2_Mo
2	CYTO	W5PG41	H6PD	3.80E-02	0.000	M2_Mo
2	CYTO	W5NQQ4	TXLNA	4.37E-02	0.000	M2_Mo
2	CYTO	W5PL89	GSR	2.60E-02	0.000	M2_Mo
2	CYTO	W5P6P2	LOC101110647	3.31E-02	0.000	M2_Mo
2	CYTO	W5PSB2	TBL2	4.04E-03	0.000	M2_Mo
2	CYTO	W5Q4Q8		8.81E-04	0.000	M2_Mo
2	CYTO	W5PFE7	ACOX1	1.40E-02	0.000	M2_Mo
2	CYTO	W5NVF6	NR3C1	4.69E-02	0.000	M2_Mo
2	CYTO	W5P6C1	ADSS ADSS2	1.03E-02	0.000	M2_Mo
2	CYTO	W5P985	ABHD14B	6.93E-03	0.000	M2_Mo
2	CYTO	W5QFN9	UGP2	2.54E-04	0.000	M2_Mo
2	CYTO	W5P180		7.80E-03	0.000	M2_Mo
2	CYTO	W5P5S5	EMC3	1.92E-02	0.000	M2_Mo
2	CYTO	W5PXJ1	MAT2B	4.42E-03	0.000	M2_Mo
2	CYTO	W5PFQ5	ANKMY2	3.69E-03	0.000	M2_Mo
2	CYTO	W5PGD6	BZW2	1.23E-03	0.000	M2_Mo
2	CYTO	W5PJY4	RAVER2	2.13E-02	0.000	M2_Mo
2	CYTO	W5NQC1	PSEN1	1.43E-03	0.000	M2_Mo
2	CYTO	W5QE35	IPO8	4.86E-03	0.000	M2_Mo
2	CYTO	W5PMG3	MEMO1	1.35E-03	0.000	M2_Mo
2	CYTO	W5PSF8	CRYZ	3.57E-02	0.000	M2_Mo
2	CYTO	W5P5A7	VPS16	1.45E-03	0.000	M2_Mo
2	CYTO	W5PDN7	GPD2	4.48E-03	0.000	M2_Mo
2	CYTO	W5P612	CRELD1	4.19E-03	0.000	M2_Mo
2	CYTO	W5QIX6	EXOC5	5.54E-02	0.000	M2_Mo

Supplementary Table 19: Shared and unique differentially abundant proteins (DAPs) between M1 and M2 relative to Mo and their cellular location.

FoldChange	Location	Protein IDs	Gene Names	P-value	FDR	Cell type
2	CYTO	W5NUE1	NCF2	9.20E-03	0.000	M2_Mo
2	CYTO	W5Q9H8	SNX5	1.87E-02	0.000	M2_Mo
2	CYTO	W5PKQ1	SORBS3	3.68E-03	0.000	M2_Mo
2	CYTO	W5P7I6	ARAP1	1.01E-03	0.000	M2_Mo
2	CYTO	W5PU61	SETD7	4.65E-02	0.000	M2_Mo
2	CYTO	W5PXH4	PTPRE	2.87E-03	0.000	M2_Mo
2	CYTO	W5PCS4		4.18E-03	0.000	M2_Mo
2	CYTO	W5NW41	TBC1D9B	3.20E-02	0.000	M2_Mo
2	CYTO	W5PRK6	STAU1	3.76E-02	0.000	M2_Mo
2	CYTO	W5PDU8	GARS1	1.53E-04	0.000	M2_Mo
2	CYTO	W5P1Z5	PMPCA	2.62E-03	0.000	M2_Mo
2	CYTO	W5PSX7	FES	1.42E-02	0.000	M2_Mo
2	CYTO	W5QHT6	NCEH1	8.31E-04	0.000	M2_Mo
2	CYTO	W5NXM4	RPS9	3.94E-02	0.000	M2_Mo
2	CYTO	W5P700	KIF1B	3.07E-02	0.000	M2_Mo
2	CYTO	W5Q0B4	KYAT3	2.41E-02	0.000	M2_Mo
2	CYTO	W5QG87	SNX4	3.32E-02	0.000	M2_Mo
2	CYTO	W5P6S8	COBLL1	4.23E-02	0.000	M2_Mo
2	CYTO	W5QAL6	FMNL3	3.33E-03	0.000	M2_Mo
2	CYTO	W5QHF8	NEMF	1.13E-02	0.000	M2_Mo
2	CYTO	W5PZ05		1.16E-04	0.000	M2_Mo
2	CYTO	W5QAP3	TOM1	6.43E-03	0.000	M2_Mo
2	CYTO	W5PSV2	TRIP11	3.59E-02	0.000	M2_Mo
2	CYTO	P68251	YWHAB	1.31E-02	0.000	M2_Mo
2	CYTO	W5PS76	UNC45A	1.19E-02	0.000	M2_Mo
2	CYTO	W5PUF8	PITRM1	5.86E-03	0.000	M2_Mo
2	CYTO	W5QG63	ZMPSTE24	1.30E-02	0.000	M2_Mo
2	CYTO	W5QCD2	ADPGK	2.72E-03	0.000	M2_Mo
2	CYTO	W5PVT3	GALNS	2.62E-02	0.000	M2_Mo
2	CYTO	W5P5H4	AARS AARS1	3.73E-03	0.000	M2_Mo
2	CYTO	W5Q3J3	COLGALT1	1.02E-02	0.000	M2_Mo
2	CYTO	W5P316	NAMPT	9.63E-03	0.000	M2_Mo
2	CYTO	W5QGN5	DLG1	5.03E-05	0.000	M2_Mo
2	CYTO	W5NWR7	GPAT4	3.88E-02	0.000	M2_Mo
2	CYTO	Q8HY31	UROD	6.40E-03	0.000	M2_Mo
2	CYTO	W5QFD2	TRIM33	4.30E-02	0.010	M2_Mo
2	CYTO	W5P0D5	PSMD7	1.64E-02	0.000	M2_Mo
2	CYTO	W5P3W2	PPP2R5D	1.41E-02	0.000	M2_Mo
2	CYTO	W5NYQ9	TIMM44	4.53E-02	0.000	M2_Mo
2	CYTO	W5P5K1	NUDT1	3.45E-02	0.000	M2_Mo
2	CYTO	W5QEA3		1.70E-03	0.000	M2_Mo
2	CYTO	W5Q7T6	AACS	4.21E-02	0.000	M2_Mo
2	CYTO	W5PD54	NCK1	1.31E-03	0.000	M2_Mo
2	CYTO	W5PXD7	LAMTOR3	5.29E-05	0.000	M2_Mo
2	CYTO	W5PP30	ASNA1 GET3	4.15E-05	0.000	M2_Mo
2	CYTO	W5NUG3	GNPDA1	4.82E-02	0.000	M2_Mo

Supplementary Table 19: Shared and unique differentially abundant proteins (DAPs) between M1 and M2 relative to Mo and their cellular location.

FoldChange	Location	Protein IDs	Gene Names	P-value	FDR	Cell type
2	CYTO	W5P1J4	NCAPG	4.63E-02	0.000	M2_Mo
2	CYTO	W5PSD7	RAP2C	2.11E-02	0.000	M2_Mo
2	CYTO	W5PCT8	ASAP1	8.42E-03	0.000	M2_Mo
2	CYTO	W5Q2H2	SLC12A9	1.71E-03	0.000	M2_Mo
2	CYTO	W5Q6T1	ARSB	1.14E-02	0.000	M2_Mo
2	CYTO	W5QCK4	GRK3	2.65E-02	0.000	M2_Mo
2	CYTO	W5Q7J2	AGPS	2.76E-02	0.000	M2_Mo
2	CYTO	W5PVC1	SYNJ1	4.45E-03	0.000	M2_Mo
2	CYTO	W5P0S9	GDPD3	2.52E-03	0.000	M2_Mo
2	CYTO	W5QDJ4	GIGYF2	1.01E-02	0.000	M2_Mo
2	CYTO	W5PBQ8	ODR4	2.63E-02	0.000	M2_Mo
2	CYTO	W5PHT8	DOCK7	1.75E-04	0.000	M2_Mo
2	CYTO	W5P0L9	MAPK3	3.29E-03	0.000	M2_Mo
2	CYTO	W5PLQ3	LARS2	1.81E-02	0.000	M2_Mo
2	CYTO	W5QDY5	ATP6V1A	9.64E-04	0.000	M2_Mo
2	CYTO	W5QFY4	COMMD5	1.63E-03	0.000	M2_Mo
2	CYTO	W5NVV3	UBAP2L	5.09E-02	0.000	M2_Mo
2	CYTO	W5Q8I0	SPC24	2.44E-02	0.000	M2_Mo
2	CYTO	W5PRQ0	RBM3	2.17E-02	0.000	M2_Mo
2	CYTO	W5Q465	TRIM36	1.53E-02	0.000	M2_Mo
2	CYTO	W5PDX8	FAM120A	7.87E-03	0.000	M2_Mo
2	CYTO	W5QHA0	AGFG1	3.95E-04	0.000	M2_Mo
2	CYTO	W5PKT0	NAA25	4.80E-02	0.002	M2_Mo
2	CYTO	W5Q922	LOC101105383	1.75E-03	0.000	M2_Mo
2	CYTO	W5Q7I9	HPS6	7.49E-03	0.000	M2_Mo
2	CYTO	W5QGG8	MAN1A2	2.53E-04	0.000	M2_Mo
2	CYTO	W5PQ08	B3GLCT	4.88E-02	0.000	M2_Mo
2	CYTO	W5PWK3	TBRG4	1.53E-02	0.001	M2_Mo
2	CYTO	W5NSS1	PPP4R1	4.21E-03	0.000	M2_Mo
2	CYTO	W5QFH0	ARSA	8.52E-03	0.000	M2_Mo
2	CYTO	W5PA89	ST6GALNAC2	2.51E-02	0.002	M2_Mo
2	CYTO	W5QFJ5	STK16	3.21E-02	0.000	M2_Mo
2	CYTO	C5HK62	EYA3	1.71E-02	0.000	M2_Mo
2	CYTO	W5NWM6	GFM1 EFG1	2.82E-04	0.000	M2_Mo
2	CYTO	W5QB79	GLCE	5.35E-02	0.000	M2_Mo
2	CYTO	W5P7P6	MYO18A	4.80E-03	0.000	M2_Mo
2	CYTO	W5PHS5	RGL2	3.88E-02	0.000	M2_Mo
2	CYTO	W5QAS2	STX6	2.12E-02	0.000	M2_Mo
2	CYTO	W5QHG2	PTCD3	1.69E-02	0.000	M2_Mo
2	CYTO	W5P6G0	PSMD8	1.59E-04	0.000	M2_Mo
2	CYTO	W5P5T6	PPP1R14B	5.19E-02	0.000	M2_Mo
2	CYTO	W5P488	TTYH3	3.43E-02	0.000	M2_Mo
2	CYTO	W5NQG1	SETD3	1.95E-02	0.000	M2_Mo
2	CYTO	W5PV12	MIPEP	4.13E-02	0.000	M2_Mo
2	CYTO	W5QG24	PPT1	2.26E-03	0.000	M2_Mo
2	CYTO	W5QDN8	SLC48A1	2.51E-02	0.000	M2_Mo

Supplementary Table 19: Shared and unique differentially abundant proteins (DAPs) between M1 and M2 relative to Mo and their cellular location.

FoldChange	Location	Protein IDs	Gene Names	P-value	FDR	Cell type
2	CYTO	W5PSQ0	DOHH	3.32E-02	0.000	M2_Mo
2	CYTO	W5P3Z5	EEPD1	1.84E-02	0.000	M2_Mo
2	CYTO	W5PYW5	AZI2	8.10E-04	0.000	M2_Mo
2	CYTO	W5NYP8	DYNC1H1	4.59E-06	0.000	M2_Mo
2	CYTO	W5PRB0	UFL1	4.02E-03	0.000	M2_Mo
2	CYTO	W5PJB6	PGM1	7.71E-03	0.000	M2_Mo
2	CYTO	W5NZV0	HMOX2	2.13E-03	0.000	M2_Mo
2	CYTO	W5Q2L6	EXOC3	5.47E-02	0.000	M2_Mo
2	CYTO	W5PME7	PALS2	9.61E-03	0.000	M2_Mo
2	CYTO	W5P2N3	MON2	1.19E-02	0.000	M2_Mo
2	CYTO	W5QGX6	C1H1orf50	5.39E-02	0.000	M2_Mo
2	CYTO	W5Q6V7	SIPA1	4.72E-02	0.000	M2_Mo
2	CYTO	W5NXX1	IPO5	3.71E-05	0.000	M2_Mo
2	CYTO	W5PD03		1.81E-03	0.000	M2_Mo
2	CYTO	W5NZ43		1.43E-02	0.000	M2_Mo
2	CYTO	W5QFL4	CSDE1	4.42E-03	0.000	M2_Mo
2	CYTO	W5Q1D8	GALNT7	1.45E-02	0.000	M2_Mo
2	CYTO	W5Q7Z6	DIP2B	1.86E-02	0.000	M2_Mo
2	CYTO	C8BKE1	STAT1 STAT4	2.89E-03	0.000	M2_Mo
2	CYTO	W5QB83	SMCR8	1.34E-02	0.000	M2_Mo
2	CYTO	W5PGC9	MMAA	6.62E-03	0.000	M2_Mo
2	CYTO	W5NV74	CARS1	9.44E-03	0.000	M2_Mo
2	CYTO	W5NX86	EIF1B	2.26E-02	0.000	M2_Mo
2	CYTO	W5NTM7	DENND10	4.74E-02	0.000	M2_Mo
2	CYTO	W5PD41	GIT1	2.55E-03	0.000	M2_Mo
2	CYTO	W5P6J4	ZYG11B	3.31E-02	0.000	M2_Mo
2	CYTO	W5P7B1	SIRT2	2.02E-02	0.000	M2_Mo
2	CYTO	W5NQ97		2.34E-02	0.000	M2_Mo
2	CYTO	W5PRW3	GOLPH3	5.56E-03	0.000	M2_Mo
2	CYTO	W5P2Q3	RPS6KA1	9.95E-03	0.000	M2_Mo
2	CYTO	W5PXJ8	CMBL	2.29E-02	0.000	M2_Mo
2	CYTO	W5Q289	ATP2B1	1.99E-03	0.000	M2_Mo
2	CYTO	W5PPJ2	VPS33A	9.52E-03	0.000	M2_Mo
2	CYTO	W5PXC4	UPB1	1.84E-02	0.000	M2_Mo
2	CYTO	W5QCG3	CLCC1	3.99E-02	0.000	M2_Mo
2	CYTO	W5PS05	MTHFD2	5.74E-03	0.000	M2_Mo
2	CYTO	W5PGJ4	CHN2	1.70E-02	0.000	M2_Mo
2	CYTO	A2SW69	ANXA2 ANX2	4.85E-03	0.000	M2_Mo
2	CYTO	W5NRK5	PPT2	8.45E-03	0.000	M2_Mo
2	CYTO	Q6ECI6	ITGB2 CD18	1.35E-04	0.000	M2_Mo
2	CYTO	W5Q7R8	JUP	9.67E-03	0.000	M2_Mo
2	CYTO	W5P7H5	ADGRE5	7.22E-04	0.000	M2_Mo
2	CYTO	W5Q0M7	RAP2B	1.60E-03	0.000	M2_Mo
2	CYTO	W5PWV5	WDR81	1.25E-02	0.000	M2_Mo
2	CYTO	W5PZ99	KIDINS220	2.39E-02	0.000	M2_Mo
2	CYTO	W5QB43	CDK6	7.45E-03	0.000	M2_Mo

Supplementary Table 19: Shared and unique differentially abundant proteins (DAPs) between M1 and M2 relative to Mo and their cellular location.

FoldChange	Location	Protein IDs	Gene Names	P-value	FDR	Cell type
2	CYTO	W5PTP8	HK2	2.64E-02	0.000	M2_Mo
2	CYTO	W5QIM0	GALK2	4.04E-02	0.000	M2_Mo
2	CYTO	W5Q0Q2		7.03E-04	0.000	M2_Mo
2	CYTO	W5PVC2	CDC37L1	2.76E-03	0.000	M2_Mo
2	CYTO	W5Q0P4	MTMR9	2.67E-03	0.000	M2_Mo
2	CYTO	W5PD62	CPB2	2.96E-02	0.009	M2_Mo
2	CYTO	W5P8N8	IDH3B	1.44E-02	0.000	M2_Mo
2	CYTO	W5PZM9	ANXA5	6.45E-04	0.000	M2_Mo
2	CYTO	W5Q018	PKN2	6.34E-04	0.000	M2_Mo
2	CYTO	W5QGC6	FYTTD1	3.13E-02	0.000	M2_Mo
2	CYTO	W5PUU2	RRAS	3.56E-02	0.000	M2_Mo
2	CYTO	W5NY29		2.96E-02	0.000	M2_Mo
2	CYTO	W5PTI6	ST3GAL4	4.01E-02	0.000	M2_Mo
2	CYTO	W5PHV7	SLC43A3	3.55E-02	0.000	M2_Mo
2	CYTO	W5P929	MARS1	4.50E-03	0.000	M2_Mo
2	CYTO	W5Q4U5	CPT1A	4.96E-02	0.000	M2_Mo
2	CYTO	W5NPC7	ACBD3	2.95E-02	0.000	M2_Mo
2	CYTO	W5Q6F0	LOC101106791	3.61E-02	0.000	M2_Mo
2	CYTO	W5NQ27	ZFYVE1	1.84E-03	0.000	M2_Mo
2	CYTO	W5Q165	RRM2	4.04E-02	0.000	M2_Mo
2	CYTO	W5NUS6	BLTP2	1.37E-02	0.000	M2_Mo
2	CYTO	W5NTC8	DDHD2	2.85E-02	0.000	M2_Mo
2	CYTO	W5PIS1	FAM91A1	3.24E-02	0.000	M2_Mo
2	CYTO	W5QBG3	APIP	2.78E-02	0.000	M2_Mo
2	CYTO	W5PNW2	BLOC1S1	6.41E-03	0.000	M2_Mo
2	CYTO	W5PFQ8	RRAGA	1.30E-02	0.000	M2_Mo
2	CYTO	W5PL61	WWC3	5.28E-02	0.001	M2_Mo
2	CYTO	W5QCC0	RALGAPB	3.09E-02	0.000	M2_Mo
2	CYTO	W5P958	IDH3G	4.23E-03	0.000	M2_Mo
2	CYTO	W5PMB1	SNX3	2.51E-03	0.000	M2_Mo
2	CYTO	W5P3U4	COMMD2	4.50E-02	0.000	M2_Mo
2	CYTO	W5NZZ3	ATP6V1F	2.99E-03	0.000	M2_Mo
2	CYTO	W5Q738	TMEM173 STING1	5.35E-03	0.000	M2_Mo
2	CYTO	W5PNT5	PSMD6	6.20E-03	0.000	M2_Mo
2	CYTO	W5PWZ2		1.45E-02	0.000	M2_Mo
-2	CYTO	W5QEL2	SNRPG	4.71E-02	0.000	M2_Mo
-2	CYTO	W5PIZ3	GRK2	1.81E-02	0.000	M2_Mo
-2	CYTO	W5PK31	FCGR3A	2.51E-02	0.000	M2_Mo
-2	CYTO	W5PJ14	ESYT2	1.96E-02	0.000	M2_Mo
-2	CYTO	W5PFJ5	SMC1A	5.37E-02	0.000	M2_Mo
-2	CYTO	W5PV11	IRF3	2.85E-02	0.000	M2_Mo
-2	CYTO	W5PKX6	DCTN6	5.62E-02	0.000	M2_Mo
-2	CYTO	W5Q3W4	RAB18	2.98E-02	0.000	M2_Mo
-2	CYTO	W5PR89	STAMBP	7.81E-03	0.000	M2_Mo
-2	CYTO	W5PJD0	PGM2	1.67E-02	0.000	M2_Mo
-2	CYTO	W5Q7F2	TIMM29	1.22E-02	0.000	M2_Mo

Supplementary Table 19: Shared and unique differentially abundant proteins (DAPs) between M1 and M2 relative to Mo and their cellular location.

FoldChange	Location	Protein IDs	Gene Names	P-value	FDR	Cell type
-2	CYTO	C5IWU4	ARL3	2.26E-03	0.000	M2_Mo
-2	CYTO	W5PE01	TAGLN2	6.73E-03	0.000	M2_Mo
-2	CYTO	W5PLV3	RAB5B	5.86E-03	0.000	M2_Mo
-2	CYTO	W5PCG2	TAP2	8.66E-03	0.000	M2_Mo
-2	CYTO	W5PRF0	SUB1	6.47E-03	0.000	M2_Mo
-2	CYTO	W5P627	GSN	3.53E-02	0.000	M2_Mo
-2	CYTO	W5NQ72	PFAS	3.45E-03	0.000	M2_Mo
-2	CYTO	W5Q859	STX5	7.04E-04	0.000	M2_Mo
-2	CYTO	W5PR48	HPRT1	4.62E-02	0.000	M2_Mo
-2	CYTO	W5QGB5	LOC101113369	9.00E-03	0.000	M2_Mo
-2	CYTO	W5Q3Q2	ERCC3	1.46E-02	0.004	M2_Mo
-2	CYTO	W5PBA6	SPON1	9.50E-03	0.000	M2_Mo
-2	CYTO	W5PB27	NDUFS3	7.99E-03	0.000	M2_Mo
-2	CYTO	W5PM65	PPP2R5A	4.24E-02	0.000	M2_Mo
-2	CYTO	W5PLX5	PRKD2	1.19E-03	0.000	M2_Mo
-2	CYTO	W5PRJ3	HYCC1	6.68E-03	0.000	M2_Mo
-2	CYTO	W5NTA4	CGGBP1	3.25E-02	0.000	M2_Mo
-2	CYTO	W5PQ00	DIABLO	3.10E-02	0.000	M2_Mo
-2	CYTO	W5QIC3	PRUNE1	9.04E-06	0.000	M2_Mo
-2	CYTO	W5Q7H5	HVCN1	4.05E-02	0.000	M2_Mo
-2	CYTO	W5Q2I1	MKRN2	1.42E-02	0.003	M2_Mo
-2	CYTO	W5PCY9		5.43E-03	0.009	M2_Mo
-2	CYTO	W5QF96	INPP5B	3.54E-02	0.000	M2_Mo
-2	CYTO	W5QGL7	RTF1	4.99E-02	0.004	M2_Mo
-2	CYTO	W5PTU0	CTPS2	2.17E-02	0.000	M2_Mo
-2	CYTO	W5QHP6	ARHGDIB	3.63E-05	0.000	M2_Mo
-2	CYTO	W5PL67	SUCLG2	7.05E-04	0.000	M2_Mo
-2	CYTO	W5PHE6	DDI2	1.30E-03	0.000	M2_Mo
-2	CYTO	W5NV31	COPS3	1.50E-03	0.000	M2_Mo
-2	CYTO	Q2TCH3	ACLY	5.26E-04	0.000	M2_Mo
-2	CYTO	W5PX94	CELF2	1.88E-02	0.000	M2_Mo
-2	CYTO	W5PQ04	DEF6	1.76E-02	0.000	M2_Mo
-2	CYTO	W5Q3M9	SEPTIN6	3.05E-02	0.000	M2_Mo
-2	CYTO	W5Q591	CDC42SE2	4.59E-02	0.000	M2_Mo
-2	CYTO	W5PXJ6	ANKRD13A	9.27E-03	0.000	M2_Mo
-2	CYTO	W5PIP3	USP47	5.08E-02	0.000	M2_Mo
-2	CYTO	W5Q9L9	SART3	3.08E-02	0.000	M2_Mo
-2	CYTO	W5Q8W8	H3F3A LOC10110893	3.50E-05	0.000	M2_Mo
-2	CYTO	W5QJ62	ACTN1	3.98E-02	0.000	M2_Mo
-2	CYTO	W5PWX7	RABGAP1	6.50E-03	0.000	M2_Mo
-2	CYTO	W5Q1F2		3.81E-02	0.000	M2_Mo
-2	CYTO	W5QDF0	NRBP1	5.41E-02	0.000	M2_Mo
-2	CYTO	W5PYM3	CAAP1	8.85E-03	0.001	M2_Mo
-2	CYTO	W5PDB0	TRIM58	2.66E-02	0.000	M2_Mo
-2	CYTO	W5PJA0	CUTA	5.19E-02	0.000	M2_Mo
-2	CYTO	W5PX34	LYPLA1	2.04E-02	0.000	M2_Mo

Supplementary Table 19: Shared and unique differentially abundant proteins (DAPs) between M1 and M2 relative to Mo and their cellular location.

FoldChange	Location	Protein IDs	Gene Names	P-value	FDR	Cell type
-2	CYTO	W5QD48	TAGLN3	9.40E-03	0.000	M2_Mo
-2	CYTO	W5PEE3	FNBP1	7.01E-03	0.000	M2_Mo
-2	CYTO	W5QH87	MFF	4.12E-03	0.000	M2_Mo
-2	CYTO	W5NY53	MPI	8.31E-03	0.000	M2_Mo
-2	CYTO	W5PJJ5	PGM2L1	2.75E-03	0.000	M2_Mo
-2	CYTO	W5PM99		1.29E-02	0.000	M2_Mo
-2	CYTO	W5Q2Y3	PLEKHB2	2.12E-02	0.000	M2_Mo
-2	CYTO	W5PL66	PPA2	1.11E-02	0.000	M2_Mo
-2	CYTO	W5P935	APMAP	1.87E-02	0.000	M2_Mo
-2	CYTO	W5P098	CORO1A	2.97E-04	0.000	M2_Mo
-2	CYTO	W5Q8V5	RBX1	3.99E-02	0.000	M2_Mo
-2	CYTO	W5QI77	AK2	3.70E-03	0.000	M2_Mo
-2	CYTO	W5PLJ8	ARL6IP1	5.29E-02	0.000	M2_Mo
-2	CYTO	W5NZU5		8.17E-03	0.000	M2_Mo
-2	CYTO	W5PMY9		3.79E-02	0.000	M2_Mo
-2	CYTO	W5PWY1	BAG5	1.24E-02	0.000	M2_Mo
-2	CYTO	W5PLD5	CNN2	3.97E-03	0.000	M2_Mo
-2	CYTO	W5PCB1	DNAJC9	3.70E-02	0.000	M2_Mo
-2	CYTO	W5PEW2	USP48	4.25E-02	0.000	M2_Mo
-2	CYTO	W5PTZ8	LOC101114959	3.96E-03	0.000	M2_Mo
-2	CYTO	W5NW82	NAP1L4	5.64E-03	0.000	M2_Mo
-2	CYTO	W5PQI0	PXN	3.07E-03	0.000	M2_Mo
-2	CYTO	W5NPT4	LOC101116298	1.73E-02	0.000	M2_Mo
-2	CYTO	W5NUT8	PIP4K2A	1.65E-02	0.000	M2_Mo
-2	CYTO	W5QIK2	RFX5	3.80E-02	0.000	M2_Mo
-2	CYTO	W5PJF2	EXT1	1.46E-02	0.000	M2_Mo
-2	CYTO	C8BKD4	CBX5	2.29E-02	0.000	M2_Mo
-2	CYTO	W5QAM4	CAMK2D	2.87E-02	0.000	M2_Mo
-2	CYTO	W5Q1S6	EML3	3.11E-02	0.000	M2_Mo
-2	CYTO	W5PXS7	DAPP1	4.59E-02	0.000	M2_Mo
-2	CYTO	W5NUK5	HAT1	1.38E-02	0.000	M2_Mo
-2	CYTO	W5NVS5	CRNKL1	2.69E-02	0.000	M2_Mo
-2	CYTO	W5PAI1		1.52E-02	0.000	M2_Mo
-2	CYTO	W5PNL4	NCBP1	2.36E-02	0.000	M2_Mo
-2	CYTO	W5P8H4	DCTPP1	3.84E-02	0.000	M2_Mo
-2	CYTO	W5QIY4	ARID4A	1.95E-02	0.000	M2_Mo
-2	CYTO	W5QGW0	PPIH	2.68E-02	0.000	M2_Mo
-2	CYTO	W5PFY8	MTSS1	5.61E-02	0.000	M2_Mo
-2	CYTO	W5Q9C5	LNPEP	1.89E-02	0.000	M2_Mo
-2	CYTO	W5NWM7	GSTZ1	5.57E-03	0.000	M2_Mo
-2	CYTO	W5QCZ0	MPST	2.93E-04	0.000	M2_Mo
-2	CYTO	W5QEP1	POGLUT1	2.08E-02	0.000	M2_Mo
-2	CYTO	W5PTM9	UBE2V2	1.50E-05	0.000	M2_Mo
-2	CYTO	W5Q4T7		4.18E-02	0.000	M2_Mo
-2	CYTO	W5PUS0	CUL4B	2.17E-02	0.000	M2_Mo
-2	CYTO	W5PTW9	LRRC32	5.04E-02	0.000	M2_Mo

Supplementary Table 19: Shared and unique differentially abundant proteins (DAPs) between M1 and M2 relative to Mo and their cellular location.

FoldChange	Location	Protein IDs	Gene Names	P-value	FDR	Cell type
-2	CYTO	W5P631	KLC4	5.39E-04	0.000	M2_Mo
-2	CYTO	W5PSX3	DARS2	1.20E-02	0.000	M2_Mo
-2	CYTO	W5NRJ3		2.29E-02	0.000	M2_Mo
-2	CYTO	W5QCS1	DDX23	3.61E-03	0.000	M2_Mo
-2	CYTO	W5PU89	ALDH16A1	3.51E-02	0.000	M2_Mo
-2	CYTO	W5P0J5	ZNF22	2.52E-02	0.000	M2_Mo
-2	CYTO	W5P4C5	RBM42	4.96E-03	0.000	M2_Mo
-2	CYTO	W5PU02	MVD	2.98E-02	0.000	M2_Mo
-2	CYTO	W5QH77	USP39	3.75E-02	0.000	M2_Mo
-2	CYTO	W5NVL4		3.39E-02	0.000	M2_Mo
-2	CYTO	W5QH64	SNAP23	6.69E-03	0.000	M2_Mo
-2	CYTO	W5P225	PDCD5	1.68E-02	0.000	M2_Mo
-2	CYTO	W5NRI1		1.73E-03	0.000	M2_Mo
-2	CYTO	W5P669		1.49E-02	0.000	M2_Mo
-2	CYTO	W5P1E2	CNST	1.04E-02	0.000	M2_Mo
-2	CYTO	W5Q768	CDS2	2.53E-02	0.000	M2_Mo
-2	CYTO	W5PY64	LOC101106288	1.86E-02	0.000	M2_Mo
-2	CYTO	W5PAP1	TERF2IP	1.75E-02	0.000	M2_Mo
-2	CYTO	W5PLB1	CLIC5	7.63E-03	0.000	M2_Mo
-2	CYTO	W5PI68	TBCK	4.35E-02	0.000	M2_Mo
-2	CYTO	W5QIH2	TMOD3	1.11E-03	0.000	M2_Mo
-2	CYTO	W5PLK6	SHOC2	9.99E-03	0.000	M2_Mo
-2	CYTO	B2LU20	SFXN1	2.67E-02	0.000	M2_Mo
-2	CYTO	W5PR51	CPNE3	4.87E-02	0.000	M2_Mo
-2	CYTO	W5P8W5		2.81E-02	0.000	M2_Mo
-2	CYTO	W5PKD2	GCDH	2.67E-02	0.000	M2_Mo
-2	CYTO	W5PA22	ACOT8	1.75E-03	0.000	M2_Mo
-2	CYTO	W5QD23	LANCL1	1.25E-02	0.000	M2_Mo
-2	CYTO	W5NQH2	CHMP6	4.19E-02	0.000	M2_Mo
-2	CYTO	W5PWH9	PRKACB	2.11E-04	0.000	M2_Mo
-3	CYTO	W5P4E2	PBLD	3.17E-02	0.000	M2_Mo
-3	CYTO	W5PSM6	HABP2	2.22E-02	0.000	M2_Mo
-3	CYTO	W5P6L3	KAT7	1.90E-02	0.000	M2_Mo
-3	CYTO	W5PK38	VASP	3.52E-02	0.000	M2_Mo
-3	CYTO	W5Q2W6	PPFIBP2	3.80E-02	0.000	M2_Mo
-3	CYTO	W5P668		4.24E-02	0.000	M2_Mo
-3	CYTO	W5PVX3	PRKG1	3.54E-02	0.000	M2_Mo
-3	CYTO	W5PRR5	LTA4H	1.02E-04	0.000	M2_Mo
-3	CYTO	W5PAL2	LOC101105297	4.38E-02	0.000	M2_Mo
-3	CYTO	W5PSA9	VPS13A	2.39E-02	0.000	M2_Mo
-3	CYTO	W5PHI7	LOC101116892	2.75E-02	0.000	M2_Mo
-3	CYTO	W5QFT3		2.64E-02	0.000	M2_Mo
-3	CYTO	W5Q6T8	SLAIN2	2.22E-02	0.000	M2_Mo
-3	CYTO	W5QHX6	ANP32E	3.24E-02	0.000	M2_Mo
-3	CYTO	W5PQ55		2.51E-02	0.000	M2_Mo
-3	CYTO	W5QFQ8	LOC101111915	2.34E-03	0.000	M2_Mo

Supplementary Table 19: Shared and unique differentially abundant proteins (DAPs) between M1 and M2 relative to Mo and their cellular location.

FoldChange	Location	Protein IDs	Gene Names	P-value	FDR	Cell type
-3	CYTO	W5Q9N1	LPCAT2	2.04E-02	0.000	M2_Mo
-3	CYTO	W5PZC3	STAMBPL1	1.84E-02	0.000	M2_Mo
-3	CYTO	W5PCJ3	LOC101112936	1.86E-02	0.000	M2_Mo
-3	CYTO	W5QFM2		2.96E-03	0.000	M2_Mo
-3	CYTO	W5Q1B3	HMGB2	1.55E-02	0.000	M2_Mo
-3	CYTO	W5QB24	ANP32A	7.60E-03	0.000	M2_Mo
-3	CYTO	W5P671	DDAH2	1.04E-02	0.010	M2_Mo
-3	CYTO	W5P582	ACP1	1.76E-02	0.000	M2_Mo
-3	CYTO	W5NXN2		1.10E-02	0.000	M2_Mo
-3	CYTO	Q09YJ2	TES	4.63E-03	0.000	M2_Mo
-3	CYTO	W5PBZ1	PCIF1	3.65E-02	0.000	M2_Mo
-3	CYTO	W5PZP8	FIS1	3.61E-03	0.000	M2_Mo
-3	CYTO	W5P0R4	CTIF	8.53E-03	0.000	M2_Mo
-3	CYTO	W5P5I4	LAMTOR2	1.46E-02	0.000	M2_Mo
-3	CYTO	W5QB58	AASS	4.50E-04	0.000	M2_Mo
-3	CYTO	W5PG02	RASA3	1.51E-02	0.000	M2_Mo
-3	CYTO	W5PZU8	ASAP2	2.88E-02	0.000	M2_Mo
-3	CYTO	W5P2Z8	RGS14	3.66E-02	0.000	M2_Mo
-3	CYTO	W5PSQ7		9.88E-03	0.000	M2_Mo
-3	CYTO	W5PX55	LSM7	1.29E-02	0.000	M2_Mo
-3	CYTO	W5P8I8	PITPNM2	6.54E-03	0.000	M2_Mo
-3	CYTO	W5PIW6	PRTN3	5.02E-02	0.000	M2_Mo
-3	CYTO	W5PIU9	ARMC6	4.31E-03	0.000	M2_Mo
-3	CYTO	W5Q1F6	AIFM1	1.24E-03	0.000	M2_Mo
-3	CYTO	W5NQ60	POLR2C	1.00E-02	0.000	M2_Mo
-3	CYTO	W5PTQ0	RNGTT	1.83E-02	0.000	M2_Mo
-3	CYTO	W5P590	OPTN	1.45E-02	0.000	M2_Mo
-3	CYTO	W5Q231	FEN1	5.31E-02	0.000	M2_Mo
-3	CYTO	W5P0K3	MBNL1	4.25E-04	0.000	M2_Mo
-3	CYTO	W5Q6S0	LBR	7.16E-03	0.000	M2_Mo
-3	CYTO	W5P7P8	GCA	1.89E-02	0.000	M2_Mo
-3	CYTO	W5PRP1	SLC9A3R1	2.37E-03	0.000	M2_Mo
-3	CYTO	W5NTV6		1.03E-02	0.000	M2_Mo
-3	CYTO	W5QAQ9	SNU13	5.28E-02	0.000	M2_Mo
-3	CYTO	W5PTB3	INPP5K	1.25E-04	0.000	M2_Mo
-3	CYTO	W5PIC3		5.53E-02	0.000	M2_Mo
-3	CYTO	W5Q297	PRKCA	6.09E-03	0.000	M2_Mo
-3	CYTO	W5Q7N9	MTX2	2.35E-03	0.000	M2_Mo
-3	CYTO	W5Q876	GPCPD1	1.46E-02	0.000	M2_Mo
-3	CYTO	W5PAF9	KIF2A	9.00E-03	0.000	M2_Mo
-3	CYTO	W5PYQ9	CPNE1	1.37E-02	0.000	M2_Mo
-3	CYTO	W5QCG0		9.98E-03	0.000	M2_Mo
-3	CYTO	W5P955	NT5C3A	2.87E-02	0.000	M2_Mo
-3	CYTO	W5PZL2		4.57E-02	0.000	M2_Mo
-3	CYTO	W5P2F2		2.50E-02	0.000	M2_Mo
-3	CYTO	W5PWV6	UBE2B	2.49E-02	0.000	M2_Mo

Supplementary Table 19: Shared and unique differentially abundant proteins (DAPs) between M1 and M2 relative to Mo and their cellular location.

FoldChange	Location	Protein IDs	Gene Names	P-value	FDR	Cell type
-3	CYTO	W5PGU9	PLCG2	5.28E-04	0.000	M2_Mo
-3	CYTO	W5Q8H0	SSH1	3.00E-02	0.000	M2_Mo
-3	CYTO	W5PTE9	COL6A1	9.56E-03	0.000	M2_Mo
-3	CYTO	W5Q0B6	PPP1R12A	5.61E-04	0.000	M2_Mo
-3	CYTO	W5NUX2		6.20E-03	0.000	M2_Mo
-3	CYTO	W5NWU0	LOC101122262	2.27E-02	0.000	M2_Mo
-3	CYTO	W5P0N1	DHX38	9.11E-03	0.001	M2_Mo
-3	CYTO	W5NQH6	S100A9	3.48E-02	0.000	M2_Mo
-3	CYTO	W5QBA4	RER1	7.21E-03	0.000	M2_Mo
-3	CYTO	W5NWH6		1.86E-03	0.000	M2_Mo
-3	CYTO	W5PYK8	VNN2	3.09E-03	0.000	M2_Mo
-3	CYTO	W5PA61		2.02E-02	0.000	M2_Mo
-3	CYTO	W5PEG1	CETN2	2.98E-02	0.000	M2_Mo
-3	CYTO	W5NWX6	APOC3	4.40E-02	0.000	M2_Mo
-3	CYTO	W5NS43	ALDH6A1	7.98E-04	0.000	M2_Mo
-3	CYTO	W5PID6	CHMP5	4.02E-02	0.000	M2_Mo
-3	CYTO	W5Q2C6		2.21E-02	0.000	M2_Mo
-3	CYTO	W5Q9U3	CNP	6.52E-03	0.000	M2_Mo
-3	CYTO	W5PXR8	POLR2J	1.05E-02	0.000	M2_Mo
-3	CYTO	W5Q595	NIF3L1	1.96E-03	0.000	M2_Mo
-3	CYTO	W5PAV0	STAP1	8.58E-03	0.000	M2_Mo
-3	CYTO	W5NTW9	RIPK1	2.93E-02	0.000	M2_Mo
-3	CYTO	W5Q9H1	ZYX	3.56E-02	0.000	M2_Mo
-3	CYTO	W5QET9	PLA1A	1.17E-03	0.000	M2_Mo
-3	CYTO	W5PVY6		5.97E-03	0.000	M2_Mo
-3	CYTO	W5Q2W4	RAVER1	3.75E-02	0.000	M2_Mo
-3	CYTO	W5NQJ0	LOC101103771	3.74E-02	0.000	M2_Mo
-3	CYTO	W5Q3E4	NAAA	3.77E-03	0.000	M2_Mo
-3	CYTO	W5P4F9	PRKAR2B	1.86E-02	0.000	M2_Mo
-3	CYTO	C5IJ83	RAB8A	1.29E-02	0.000	M2_Mo
-3	CYTO	W5PTQ7	TRAF3IP3	1.29E-02	0.000	M2_Mo
-3	CYTO	W5PEA0	FGD3	3.61E-03	0.000	M2_Mo
-3	CYTO	W5P874	ABHD16A	1.17E-02	0.000	M2_Mo
-3	CYTO	W5PW39	LMNB2	2.07E-02	0.000	M2_Mo
-3	CYTO	W5PP85	TBC1D10C	1.81E-02	0.000	M2_Mo
-3	CYTO	W5PWS1	CD46	1.72E-03	0.000	M2_Mo
-3	CYTO	W5Q0P0	GABPA	2.33E-02	0.000	M2_Mo
-3	CYTO	W5Q8R0	FRYL	3.70E-02	0.000	M2_Mo
-3	CYTO	W5PKS6	DEK	1.62E-02	0.000	M2_Mo
-3	CYTO	W5PKA9	F5	7.25E-03	0.000	M2_Mo
-3	CYTO	W5NTF8	SPNS1	4.80E-02	0.000	M2_Mo
-3	CYTO	W5PQD8	C1QTNF3	1.25E-03	0.000	M2_Mo
-3	CYTO	W5QH43	EIF4A2	1.41E-02	0.000	M2_Mo
-4	CYTO	W5PI65	MAP4K2	1.24E-03	0.000	M2_Mo
-4	CYTO	W5PEL2	OGN	1.92E-02	0.000	M2_Mo
-4	CYTO	W5PKY9	MRCL3	2.47E-02	0.000	M2_Mo

Supplementary Table 19: Shared and unique differentially abundant proteins (DAPs) between M1 and M2 relative to Mo and their cellular location.

FoldChange	Location	Protein IDs	Gene Names	P-value	FDR	Cell type
-4	CYTO	W5QHL6	ITM2C	2.62E-04	0.000	M2_Mo
-4	CYTO	W5PGT0	MYH11	2.99E-02	0.000	M2_Mo
-4	CYTO	W5P691	CNTRL	2.03E-02	0.000	M2_Mo
-4	CYTO	W5PQT2	TPRKB	1.89E-02	0.000	M2_Mo
-4	CYTO	C5ISA2	TUBA4A	7.83E-03	0.000	M2_Mo
-4	CYTO	W5PZE4	GSTA1-1 GSTA2	1.03E-02	0.000	M2_Mo
-4	CYTO	W5PJF3	PPIF	1.28E-02	0.000	M2_Mo
-4	CYTO	W5PX46	LOC101122591	3.64E-02	0.000	M2_Mo
-4	CYTO	W5Q3J8		6.54E-03	0.000	M2_Mo
-4	CYTO	W5PVD4	AK3	1.74E-02	0.000	M2_Mo
-4	CYTO	W5P6Q7	MPIG6B	4.33E-02	0.000	M2_Mo
-4	CYTO	W5Q2V1		2.44E-03	0.000	M2_Mo
-4	CYTO	W5PMJ7	NADK2	4.13E-03	0.000	M2_Mo
-4	CYTO	W5P880	PRG4	1.10E-02	0.000	M2_Mo
-4	CYTO	W5PEC3	ANXA6	4.82E-02	0.000	M2_Mo
-4	CYTO	W5P3V8		1.64E-02	0.000	M2_Mo
-4	CYTO	W5PRB3		5.09E-04	0.000	M2_Mo
-4	CYTO	W5P6Z6	MAPRE2	5.44E-02	0.000	M2_Mo
-4	CYTO	W5P7K2		1.02E-03	0.000	M2_Mo
-4	CYTO	W5P795	LRBA	3.22E-02	0.000	M2_Mo
-4	CYTO	W5PWL7	NAPSA	5.59E-04	0.000	M2_Mo
-4	CYTO	W5Q6F1		2.85E-02	0.000	M2_Mo
-4	CYTO	W5P9M9	LOC101103862	1.80E-02	0.000	M2_Mo
-4	CYTO	W5NZT7	NMRAL1	4.20E-02	0.000	M2_Mo
-4	CYTO	W5PDQ3	SAFB2	4.39E-03	0.000	M2_Mo
-4	CYTO	W5QCH5	WDR47	7.79E-04	0.000	M2_Mo
-4	CYTO	W5PHN9	F11R	4.86E-02	0.000	M2_Mo
-4	CYTO	W5P2J9		2.38E-02	0.000	M2_Mo
-4	CYTO	W5PK79	VWA5A	3.17E-03	0.000	M2_Mo
-4	CYTO	W5PRC8	IKZF3	4.28E-02	0.000	M2_Mo
-4	CYTO	W5PT20	ZC3H18	5.01E-03	0.000	M2_Mo
-4	CYTO	W5P432	NIPSNAP1	3.43E-03	0.000	M2_Mo
-4	CYTO	W5PDD5	STAG1	1.61E-02	0.000	M2_Mo
-4	CYTO	W5PTN4	TMPO	5.12E-02	0.000	M2_Mo
-4	CYTO	W5NYC7	DQA	4.04E-02	0.000	M2_Mo
-4	CYTO	W5QI61	CDKN1B	4.63E-04	0.000	M2_Mo
-4	CYTO	W5NTQ3	H3-4	3.62E-05	0.000	M2_Mo
-4	CYTO	W5Q7L1	CNN3	5.34E-03	0.000	M2_Mo
-4	CYTO	W5PWH2	MYH14	1.42E-02	0.000	M2_Mo
-4	CYTO	W5NSJ5	OXSR1	2.36E-02	0.000	M2_Mo
-4	CYTO	W5QEK8	EHD3	3.85E-03	0.000	M2_Mo
-4	CYTO	W5NXY7	LOC101117960	4.50E-02	0.000	M2_Mo
-4	CYTO	W5NSC5		1.40E-02	0.000	M2_Mo
-4	CYTO	W5PTS4	LOC101114275	4.47E-03	0.000	M2_Mo
-4	CYTO	W5PKQ2	PRXL2A	5.33E-02	0.000	M2_Mo
-4	CYTO	W5PXT1	MYO1G	1.61E-02	0.000	M2_Mo

Supplementary Table 19: Shared and unique differentially abundant proteins (DAPs) between M1 and M2 relative to Mo and their cellular location.

FoldChange	Location	Protein IDs	Gene Names	P-value	FDR	Cell type
-4	CYTO	W5QEL7	NDRG2	1.18E-04	0.008	M2_Mo
-4	CYTO	W5QEC3	METTL3	4.26E-03	0.000	M2_Mo
-4	CYTO	P29701	AHSG FETUA	1.70E-02	0.000	M2_Mo
-4	CYTO	W5NTL4	EVL	2.37E-02	0.000	M2_Mo
-4	CYTO	W5PV67	LIMD2	4.78E-03	0.000	M2_Mo
-4	CYTO	W5PIN7		2.48E-02	0.000	M2_Mo
-4	CYTO	W5QHW2	DAPK2	3.56E-02	0.000	M2_Mo
-4	CYTO	W5Q059	SASH3	3.34E-03	0.000	M2_Mo
-4	CYTO	W5PKR2	MX1	3.08E-03	0.000	M2_Mo
-5	CYTO	W5QCW9	TST	5.35E-02	0.000	M2_Mo
-5	CYTO	W5PC20	VPS37B	1.03E-03	0.000	M2_Mo
-5	CYTO	W5PMH6	LCN2	4.97E-02	0.000	M2_Mo
-5	CYTO	W5PHM1	ALOX12	4.42E-02	0.000	M2_Mo
-5	CYTO	W5P7W5	LOC101113516	1.75E-02	0.000	M2_Mo
-5	CYTO	W5Q2G2	IL16	2.84E-03	0.000	M2_Mo
-5	CYTO	W5PU80	MS4A1	1.03E-02	0.000	M2_Mo
-5	CYTO	W5P4U4	GRK6	5.76E-04	0.000	M2_Mo
-5	CYTO	W5PRM0	GMPR	2.67E-02	0.000	M2_Mo
-5	CYTO	C0IZ95	RAB27A	2.98E-03	0.000	M2_Mo
-5	CYTO	W5P340	SOD2	2.03E-03	0.000	M2_Mo
-5	CYTO	W5Q5M7	SLC44A2	1.63E-03	0.000	M2_Mo
-5	CYTO	W5Q1G8	LOC101120875	3.43E-02	0.000	M2_Mo
-5	CYTO	W5PBW1	SDS	1.89E-02	0.000	M2_Mo
-5	CYTO	W5P6T0		5.81E-04	0.001	M2_Mo
-5	CYTO	W5PJP5	RGS18	2.42E-02	0.000	M2_Mo
-5	CYTO	W5PFJ0	VCL	1.57E-02	0.000	M2_Mo
-5	CYTO	C5IS96	LCAT	5.11E-03	0.000	M2_Mo
-5	CYTO	W5NR20	LOC101109940	2.51E-02	0.010	M2_Mo
-5	CYTO	W5PE90	ITPR1	2.15E-02	0.000	M2_Mo
-5	CYTO	W5PC51	TGFB1	2.57E-02	0.000	M2_Mo
-5	CYTO	W5NV79	NUP210	1.67E-02	0.000	M2_Mo
-5	CYTO	P47843	SLC2A3 GLUT3	1.28E-02	0.000	M2_Mo
-5	CYTO	W5QFP0	THBS1	1.24E-02	0.000	M2_Mo
-5	CYTO	W5NWB0		3.92E-04	0.000	M2_Mo
-5	CYTO	W5PNC4	JPT1	2.20E-03	0.000	M2_Mo
-5	CYTO	W5PDP2	CD55	3.55E-04	0.000	M2_Mo
-5	CYTO	W5P9Q0	PI4K2B	5.92E-04	0.002	M2_Mo
-5	CYTO	W5PV80	BANK1	1.71E-02	0.000	M2_Mo
-5	CYTO	W5PYX0		7.83E-03	0.000	M2_Mo
-5	CYTO	W5PFI6	RASGRP2	1.52E-03	0.000	M2_Mo
-6	CYTO	W5PRY2	ACAP1	2.02E-02	0.000	M2_Mo
-6	CYTO	W5QAK3	MMRN1	3.02E-02	0.000	M2_Mo
-6	CYTO	W5QFP2	LOC101109397	4.25E-03	0.000	M2_Mo
-6	CYTO	W5PXV3	CCN2	1.60E-02	0.000	M2_Mo
-6	CYTO	W5PFP1	LTF	3.29E-02	0.000	M2_Mo
-6	CYTO	W5PFI7	VCL	2.84E-02	0.000	M2_Mo

Supplementary Table 19: Shared and unique differentially abundant proteins (DAPs) between M1 and M2 relative to Mo and their cellular location.

FoldChange	Location	Protein IDs	Gene Names	P-value	FDR	Cell type
-6	CYTO	W5P7C7	CD180	3.53E-02	0.000	M2_Mo
-6	CYTO	W5Q878	PRKCB	7.49E-04	0.000	M2_Mo
-6	CYTO	P80190		2.76E-03	0.000	M2_Mo
-6	CYTO	P79362	CATHL2 BAC5	1.40E-02	0.000	M2_Mo
-6	CYTO	W5PZG0	ADD1	7.36E-03	0.000	M2_Mo
-6	CYTO	W5P4F5	RAB27B	5.11E-02	0.000	M2_Mo
-6	CYTO	W5PGG6	MTMR1	8.66E-04	0.000	M2_Mo
-6	CYTO	W5PY97	SVIL	1.19E-02	0.000	M2_Mo
-6	CYTO	W5Q9B1	ATP2A3	4.84E-04	0.000	M2_Mo
-6	CYTO	W5PM33	SELP	2.92E-02	0.000	M2_Mo
-6	CYTO	W5Q3B8		1.10E-02	0.000	M2_Mo
-6	CYTO	W5Q9S4	ING3	5.93E-03	0.001	M2_Mo
-6	CYTO	W5PLZ3	PACSIN1	6.74E-03	0.000	M2_Mo
-6	CYTO	W5PHR3	ADD3	9.46E-03	0.000	M2_Mo
-6	CYTO	P54230	CATHL1A	4.38E-03	0.000	M2_Mo
-6	CYTO	W5P4L3	AVIL	2.35E-02	0.000	M2_Mo
-6	CYTO	W5P815	SEPTIN1	1.57E-02	0.000	M2_Mo
-6	CYTO	P14639	ALB	5.62E-03	0.000	M2_Mo
-7	CYTO	W5P733	PDLIM1	3.62E-02	0.000	M2_Mo
-7	CYTO	W5NUE6	PLEKHA2	1.71E-04	0.000	M2_Mo
-7	CYTO	W5QGQ3	LOC443320	4.10E-03	0.000	M2_Mo
-7	CYTO	W5Q0K9	ABLIM1	3.26E-03	0.000	M2_Mo
-7	CYTO	W5PQI3	ITGB3	8.26E-03	0.000	M2_Mo
-7	CYTO	W5P3Y9	CD22	8.06E-03	0.000	M2_Mo
-7	CYTO	W5NT35	LOC443162	8.44E-03	0.000	M2_Mo
-7	CYTO	W5Q5A6	FGG	1.16E-02	0.000	M2_Mo
-7	CYTO	W5PL70	PDCD4	9.33E-04	0.000	M2_Mo
-8	CYTO	W5NQ46	FGB	1.54E-02	0.000	M2_Mo
-8	CYTO	W5PHQ0	MPO	2.10E-03	0.000	M2_Mo
-8	CYTO	W5PP64	FHL1	1.46E-03	0.000	M2_Mo
-8	CYTO	W5NXW9		1.56E-04	0.000	M2_Mo
-8	CYTO	W5PEX2	GPATCH8	2.60E-03	0.000	M2_Mo
-8	CYTO	W5PF87	ALOX15	2.50E-02	0.000	M2_Mo
-9	CYTO	W5Q2S8	MYL9	3.12E-03	0.000	M2_Mo
-9	CYTO	W5NZX9	SPTBN1	6.73E-03	0.000	M2_Mo
-9	CYTO	W5Q3I7	TUBB1	1.57E-03	0.000	M2_Mo
-9	CYTO	W5Q5H8	FGA	4.70E-03	0.000	M2_Mo
-9	CYTO	W5PJ75	SPTAN1	7.48E-04	0.000	M2_Mo
-10	CYTO	W5QIK8	SELENBP1	3.67E-03	0.000	M2_Mo
9	SEC	W5PUH5	LGMN	9.00E-06	0.000	M2_Mo
7	SEC	W5P3Q3	LOC100101238	1.77E-03	0.000	M2_Mo
7	SEC	W5NUZ2	UTRN	3.52E-04	0.002	M2_Mo
7	SEC	W5Q0F3	TGFBI	4.76E-04	0.000	M2_Mo
7	SEC	W5PGV0	ITGAM	9.60E-04	0.000	M2_Mo
6	SEC	W5PDE5	LOC101120001	1.48E-02	0.000	M2_Mo
6	SEC	W5Q9H0	MMP2	3.39E-04	0.000	M2_Mo

Supplementary Table 19: Shared and unique differentially abundant proteins (DAPs) between M1 and M2 relative to Mo and their cellular location.

FoldChange	Location	Protein IDs	Gene Names	P-value	FDR	Cell type
6	SEC	W5NTX3		3.13E-04	0.000	M2_Mo
6	SEC	W5QDI7	CSF1	2.15E-04	0.000	M2_Mo
6	SEC	W5NTD9	CHI3L1	1.70E-04	0.000	M2_Mo
6	SEC	W5NZX1		1.02E-03	0.000	M2_Mo
6	SEC	W5Q989	PLOD1	3.52E-03	0.000	M2_Mo
6	SEC	W5PS94	NUCB1	4.77E-03	0.000	M2_Mo
5	SEC	W5NPK5	LOC443475	7.43E-04	0.000	M2_Mo
5	SEC	W5P8R4	CSF1R	1.36E-03	0.000	M2_Mo
5	SEC	W5PMR2	MMP19	4.20E-03	0.000	M2_Mo
5	SEC	W5QHV3	FABP1	7.85E-04	0.000	M2_Mo
5	SEC	W5PEY4	TCN2	6.42E-04	0.000	M2_Mo
5	SEC	W5NUI3	TREM2	1.85E-03	0.000	M2_Mo
5	SEC	W5QI35	IL1RN	3.90E-03	0.000	M2_Mo
5	SEC	W5PTL2	CFP	1.15E-02	0.000	M2_Mo
5	SEC	W5PZB0	APLP2	1.05E-03	0.000	M2_Mo
5	SEC	W5Q754	TTN	5.45E-02	0.002	M2_Mo
5	SEC	W5Q689	TPP1	1.08E-04	0.000	M2_Mo
5	SEC	W5PG72	GOLM1	6.04E-05	0.000	M2_Mo
5	SEC	W5PCC7	LOC101115509	6.02E-04	0.000	M2_Mo
5	SEC	W5NTG6	TINAGL1	2.71E-04	0.000	M2_Mo
5	SEC	W5QJA2	CD14	1.82E-04	0.000	M2_Mo
5	SEC	W5NSK4		1.04E-03	0.000	M2_Mo
5	SEC	W5QDG8	FN1	1.30E-04	0.000	M2_Mo
5	SEC	W5Q1W2	SDCBP	4.30E-03	0.000	M2_Mo
5	SEC	W5P0W4	SEMA7A	2.62E-03	0.000	M2_Mo
4	SEC	W5PS45	MAN2B1	4.75E-02	0.000	M2_Mo
4	SEC	W5PF04	MAN1A1	2.50E-03	0.000	M2_Mo
4	SEC	W5Q3T9	PDCD6IP	1.24E-02	0.000	M2_Mo
4	SEC	W5PIC9		1.61E-03	0.000	M2_Mo
4	SEC	W5P041	ADAM15	9.58E-05	0.000	M2_Mo
4	SEC	W5QFU8	CD86	2.40E-02	0.000	M2_Mo
4	SEC	W5PNP1	MFGE8	1.47E-03	0.000	M2_Mo
4	SEC	W5NZ76	TIMP2	6.75E-03	0.000	M2_Mo
4	SEC	W5PH85	ITGAX	2.24E-02	0.000	M2_Mo
4	SEC	W5PB21	PLA2R1	6.18E-03	0.000	M2_Mo
4	SEC	W5Q233	VCAN	3.56E-02	0.000	M2_Mo
4	SEC	W5PJR5	LOC101121216	2.66E-02	0.000	M2_Mo
4	SEC	W5P887	CST3	1.49E-02	0.000	M2_Mo
4	SEC	W5PIQ4	LOC101120093	6.79E-03	0.000	M2_Mo
4	SEC	W5PSA3	TCN1	1.06E-03	0.000	M2_Mo
4	SEC	W5PCM7	LOC101105937	1.09E-02	0.004	M2_Mo
4	SEC	W5P1W2	FOLR3	3.91E-03	0.009	M2_Mo
3	SEC	W5QIR6	GATM	8.41E-03	0.000	M2_Mo
3	SEC	W5Q799	GALNT6	9.27E-03	0.010	M2_Mo
3	SEC	W5PCS7	CXCL16	2.39E-03	0.004	M2_Mo
3	SEC	W5QFB2	OLFML3	2.75E-02	0.000	M2_Mo

Supplementary Table 19: Shared and unique differentially abundant proteins (DAPs) between M1 and M2 relative to Mo and their cellular location.

FoldChange	Location	Protein IDs	Gene Names	P-value	FDR	Cell type
3	SEC	Q9MZA9	VIM	2.20E-02	0.000	M2_Mo
3	SEC	W5QDG9	CCDC80	4.30E-02	0.000	M2_Mo
3	SEC	W5QD11	SORT1	6.81E-03	0.000	M2_Mo
3	SEC	W5PMM5	ERP44	2.07E-02	0.000	M2_Mo
3	SEC	W5Q7S8	NRP1	1.27E-02	0.000	M2_Mo
3	SEC	Q7M2U8	APOE	8.61E-03	0.000	M2_Mo
3	SEC	W5PGG5	CD84	5.06E-02	0.001	M2_Mo
2	SEC	W5PUJ5	LAMP2	2.03E-02	0.000	M2_Mo
2	SEC	W5P2K9	LMAN2	4.16E-02	0.000	M2_Mo
2	SEC	W5PV50	ADA2	4.19E-02	0.004	M2_Mo
2	SEC	W5PZI1	LOC101113728	5.69E-03	0.000	M2_Mo
-2	SEC	W5P8Y7		1.36E-02	0.004	M2_Mo
-2	SEC	W5QFH1	ACTC1	2.36E-02	0.000	M2_Mo
-2	SEC	P60713	ACTB	2.66E-02	0.000	M2_Mo
-2	SEC	W5Q2I7	USO1	3.89E-02	0.000	M2_Mo
-2	SEC	W5Q2D9	RAB5A	5.05E-02	0.000	M2_Mo
-2	SEC	W5P4J1	VAV1	4.26E-02	0.000	M2_Mo
-2	SEC	W5NSM1	ACTR1A	3.02E-02	0.000	M2_Mo
-2	SEC	W5NVQ4	CAND1	1.21E-02	0.000	M2_Mo
-2	SEC	Q28554	GAPDH G3PDH GAPD	5.40E-02	0.000	M2_Mo
-2	SEC	W5Q1I6	IMPDH2 IMPDH	3.23E-02	0.000	M2_Mo
-2	SEC	C5IWU0	ARF1 LOC101123118	2.77E-02	0.000	M2_Mo
-2	SEC	W5PQ76	SRI	1.49E-02	0.000	M2_Mo
-3	SEC	W5QHL5	CAB39	1.79E-02	0.000	M2_Mo
-3	SEC	P09670	SOD1	1.52E-02	0.006	M2_Mo
-3	SEC	W5PFV5	NPEPPS	2.50E-02	0.000	M2_Mo
-3	SEC	W5Q121		3.40E-02	0.000	M2_Mo
-3	SEC	W5Q9X5	MAP2K1	2.37E-02	0.000	M2_Mo
-3	SEC	W5Q1I8	PPM1F	1.46E-02	0.000	M2_Mo
-3	SEC	W5P5F6	TBCB	4.18E-02	0.005	M2_Mo
-3	SEC	W5QCX9	TTLL12	4.96E-02	0.000	M2_Mo
-3	SEC	W5NVC9	RAC1	3.90E-02	0.000	M2_Mo
-3	SEC	W5PKR1		3.05E-02	0.004	M2_Mo
-3	SEC	W5PSZ3	JARID2	5.18E-02	0.005	M2_Mo
-3	SEC	W5NSV5	ITGA6	6.99E-03	0.000	M2_Mo
-3	SEC	W5QCL5	CSNK2A1	2.06E-02	0.000	M2_Mo
-3	SEC	W5PZ86	SEPTIN8	3.24E-02	0.000	M2_Mo
-3	SEC	W5PVT6	UBA1	4.43E-02	0.000	M2_Mo
-3	SEC	W5P8B4	TRAPPC8	2.39E-02	0.006	M2_Mo
-4	SEC	W5Q3E3	RAB2A	5.70E-04	0.000	M2_Mo
-4	SEC	W5P5A0	FLNA	2.60E-02	0.000	M2_Mo
-4	SEC	W5P6X5	STMN1	2.52E-02	0.000	M2_Mo
-4	SEC	W5P889	SEPTIN9	2.61E-02	0.000	M2_Mo
-4	SEC	W5QI99	NEDD4	2.97E-03	0.000	M2_Mo
-4	SEC	W5PQK3	PFKL	1.28E-02	0.000	M2_Mo
-4	SEC	W5P5C4	NARS1	4.73E-03	0.000	M2_Mo

Supplementary Table 19: Shared and unique differentially abundant proteins (DAPs) between M1 and M2 relative to Mo and their cellular location.

FoldChange	Location	Protein IDs	Gene Names	P-value	FDR	Cell type
-4	SEC	C5IJA0	RAN	1.39E-02	0.000	M2_Mo
-4	SEC	W5P409	FERMT3	2.40E-02	0.000	M2_Mo
-4	SEC	W5QHS2	MGP	1.44E-03	0.000	M2_Mo
-4	SEC	W5P4C7	SEPTIN7	2.51E-02	0.000	M2_Mo
-4	SEC	W5PHU7	UNC13D	5.61E-03	0.000	M2_Mo
-4	SEC	W5NQK6	LIMS1	5.18E-03	0.000	M2_Mo
-4	SEC	W5P538	CD93	8.85E-03	0.000	M2_Mo
-4	SEC	W5NRD9		2.88E-03	0.002	M2_Mo
-4	SEC	W5P7E2	PPA1	2.53E-02	0.000	M2_Mo
-4	SEC	W5QGT4		2.43E-02	0.000	M2_Mo
-4	SEC	Q5MIB6	PYGB	1.50E-02	0.000	M2_Mo
-4	SEC	W5QF71	PLEK	3.10E-03	0.000	M2_Mo
-4	SEC	W5PMQ9	SAE1	4.29E-03	0.000	M2_Mo
-4	SEC	W5PWG1	PFKP	7.77E-03	0.000	M2_Mo
-4	SEC	W5PTZ9	LOC101120877	5.44E-03	0.000	M2_Mo
-5	SEC	W5Q6U0	FASN	3.64E-03	0.000	M2_Mo
-5	SEC	W5PIJ6	PTPN11	1.99E-02	0.000	M2_Mo
-5	SEC	W5P0V5	RAB11B	4.09E-03	0.000	M2_Mo
-5	SEC	W5Q3B7	PDE5A	2.82E-02	0.000	M2_Mo
-5	SEC	W5QD96	PARVB	5.08E-03	0.000	M2_Mo
-5	SEC	W5PY17	STX7	9.37E-03	0.000	M2_Mo
-5	SEC	W5PE27	ESD	5.38E-04	0.000	M2_Mo
-5	SEC	W5Q731	ILK	2.66E-02	0.000	M2_Mo
-5	SEC	W5PX84	CCDC171	3.08E-04	0.005	M2_Mo
-5	SEC	W5PK85	EML2	1.36E-02	0.000	M2_Mo
-6	SEC	W5PQK6	TLN1	3.63E-02	0.000	M2_Mo
-6	SEC	W5PNU4	LOC101111106	9.26E-03	0.000	M2_Mo
-6	SEC	W5PG95	HSPA1A	4.93E-03	0.000	M2_Mo
-6	SEC	W5PH15	RSU1	6.54E-03	0.000	M2_Mo
-7	SEC	W5PD82	CALD1	1.15E-03	0.000	M2_Mo
-7	SEC	W5QFM1		4.57E-02	0.000	M2_Mo

M1, pro-inflammatory macrophages activated with GM-CSF/ LPS/ INF-γ; M2, anti-inflammatory macrophages activated with M-CSF/ IL-4 and M0, Monocytes at 3 hours. Differentially abundant proteins (DAPs) were identified using a threshold of false discovery rate (FDR, q-value) ≤ 0.05 and absolute fold change ≥ 2. Red-highlighted cells indicate shared differentially abundant proteins (DAPs) between M1 and M2 relative to M0, whereas non-highlighted cells denote DAPs that are unique to either the M1 or M2 subtype. “CYTO” denotes proteins identified in whole-cell lysates, and “SEC” refers to those detected in the secretome.

Supplementary Table 20: Differentially abundant proteins (DAPs) of M1 relative to M2 and their cellular location.

FoldChange	Location	Protein IDs	Gene Names	P-value	FDR
8	CYTO	W5Q5H8	FGA	8.59E-04	0.000
8	CYTO	W5Q5A6	FGG	1.11E-03	0.000
7	CYTO	W5NQ46	FGB	2.47E-03	0.000
7	CYTO	W5PBW1	SDS	3.98E-03	0.000
7	CYTO	W5P340	SOD2	9.32E-04	0.000
6	CYTO	W5PYX0		2.65E-04	0.000
6	CYTO	M4WG34	IL1b IL1B	2.08E-02	0.000
6	CYTO	W5PK56	FCGR2B	5.05E-03	0.000
5	CYTO	W5PU22	MCUR1	2.22E-02	0.000
5	CYTO	W5NQW9	LOC101104482	2.14E-05	0.000
5	CYTO	W5PNC4	JPT1	1.30E-03	0.000
5	CYTO	W5PFV1	CTSL	7.90E-03	0.000
5	CYTO	W5PMT0	XDH	1.28E-03	0.000
5	CYTO	W5NXM6	PTX3	5.08E-04	0.000
5	CYTO	W5NRX0	PPFIA4	7.09E-03	0.001
5	CYTO	W5QAK3	MMRN1	5.16E-03	0.000
5	CYTO	W5PP64	FHL1	1.01E-04	0.000
5	CYTO	W5Q3I7	TUBB1	1.96E-03	0.000
5	CYTO	W5PKR2	MX1	9.63E-05	0.000
5	CYTO	W5PQI3	ITGB3	1.12E-02	0.000
5	CYTO	W5PEX2	GPATCH8	4.08E-02	0.000
5	CYTO	W5QFP0	THBS1	2.16E-04	0.000
5	CYTO	W5P880	PRG4	6.59E-06	0.000
5	CYTO	W5QFU8	CD86	5.08E-04	0.000
5	CYTO	P79365	SLC2A1 GLUT1	1.82E-02	0.005
5	CYTO	W5NUJ7	LOC101123419	3.44E-04	0.000
5	CYTO	P54230	CATHL1A BAC1A DOD	8.63E-06	0.000
5	CYTO	W5PHQ0	MPO	5.00E-02	0.000
5	CYTO	W5PVJ5	CD274	1.30E-04	0.000
5	CYTO	W5PRG9	HAL	8.90E-05	0.000
5	CYTO	W5NQK9	S100A8	1.08E-04	0.000
5	CYTO	W5PWL7	NAPSA	3.69E-06	0.000
5	CYTO	W5NZ40	SGF29	9.26E-04	0.000
4	CYTO	W5PWH2	MYH14	4.75E-04	0.000
4	CYTO	P29701	AHSG FETUA	1.86E-03	0.000
4	CYTO	W5PC51	TGFB1	5.26E-05	0.000
4	CYTO	W5P2P7	SYPL1	2.85E-07	0.000
4	CYTO	W5PWS1	CD46	1.24E-04	0.000
4	CYTO	W5Q233	VCAN	2.48E-02	0.000
4	CYTO	W5Q3H9	UPP1	1.68E-02	0.000
4	CYTO	W5PE90	ITPR1	7.20E-03	0.000
4	CYTO	W5PNY4	PDXK	1.84E-02	0.000
4	CYTO	W5NUE6	PLEKHA2	5.01E-05	0.000
4	CYTO	W5Q9K1	QSOX1	5.72E-04	0.000
4	CYTO	W5PM91	SLC1A5	4.79E-04	0.000
4	CYTO	W5Q284	HAPLN1	1.91E-04	0.000

Supplementary Table 20: Differentially abundant proteins (DAPs) of M1 relative to M2 and their cellular location.

FoldChange	Location	Protein IDs	Gene Names	P-value	FDR
4	CYTO	W5PHG1	CDA	2.34E-03	0.000
4	CYTO	W5Q3E4	NAAA	8.36E-05	0.000
4	CYTO	W5NZX4	KATNAL2	8.91E-06	0.004
4	CYTO	W5P407		5.15E-03	0.000
4	CYTO	P20757	AGT SERPINA8	5.78E-06	0.000
4	CYTO	W5PH81	C7	4.38E-05	0.000
4	CYTO	W5PAM2	RHBDF2	5.74E-03	0.000
4	CYTO	W5PXV3	CCN2	8.82E-04	0.000
4	CYTO	W5PID6	CHMP5	5.92E-03	0.000
4	CYTO	C5IJ83	RAB8A	7.22E-05	0.000
4	CYTO	W5NWB0		4.81E-05	0.000
4	CYTO	W5P5I4	LAMTOR2	1.34E-03	0.000
4	CYTO	W5NXJ0	CLEC4E	1.02E-02	0.000
4	CYTO	W5PJ75	SPTAN1	2.14E-03	0.000
4	CYTO	W5PXY4	CLEC5A	1.48E-02	0.000
4	CYTO	W5NTW3	ITIH1	4.17E-04	0.000
4	CYTO	W5QDG8	FN1	1.32E-03	0.000
4	CYTO	W5PAC2	LOC101105044	1.72E-02	0.000
4	CYTO	W5PWI4	RGN	8.46E-06	0.000
4	CYTO	W5PZE4	GSTA1-1 GSTA2	2.09E-04	0.000
4	CYTO	W5PII2	ATG4C	3.78E-02	0.000
4	CYTO	W5PGG6	MTMR1	2.65E-02	0.000
4	CYTO	W5PMX5	C5AR1	3.09E-03	0.000
4	CYTO	W5P7G4	TNC	2.94E-04	0.000
4	CYTO	W5Q9B1	ATP2A3	9.08E-03	0.000
4	CYTO	W5NSA6	LOC101122940	5.91E-05	0.000
4	CYTO	W5PMX7		3.78E-02	0.000
4	CYTO	W5P150		9.19E-03	0.000
4	CYTO	W5PLT8		4.29E-02	0.000
4	CYTO	W5PEL2	OGN	1.35E-04	0.000
4	CYTO	W5PYW3	LOC101103238	3.45E-02	0.000
4	CYTO	W5Q2B3	SUSD5	3.55E-02	0.009
4	CYTO	W5PTE9	COL6A1	2.97E-05	0.000
4	CYTO	P47843	SLC2A3 GLUT3	3.98E-03	0.000
4	CYTO	Q29400	IGFBP2	7.88E-04	0.000
3	CYTO	W5PYH9		3.96E-03	0.000
3	CYTO	W5PAJ9	APOM	3.15E-03	0.000
3	CYTO	W5QC43	ANTXR2	1.86E-04	0.000
3	CYTO	W5QJ54	ARG2	6.38E-04	0.000
3	CYTO	W5NXY7	LOC101117960	1.06E-05	0.000
3	CYTO	W5Q723	BHMT	1.09E-03	0.000
3	CYTO	W5PUG0	RNF114	2.14E-03	0.000
3	CYTO	W5Q9F0	LOC101102694	9.15E-03	0.002
3	CYTO	W5P7W5	LOC101113516	7.24E-04	0.000
3	CYTO	W5P949	FILIP1	1.27E-02	0.004
3	CYTO	W5P3H8	IGF2R	8.55E-05	0.000

Supplementary Table 20: Differentially abundant proteins (DAPs) of M1 relative to M2 and their cellular location.

FoldChange	Location	Protein IDs	Gene Names	P-value	FDR
3	CYTO	W5Q3T6	SAMSN1	7.24E-03	0.000
3	CYTO	W5Q824	GSTA1	1.09E-03	0.000
3	CYTO	W5PEU9	STAM2	1.87E-04	0.000
3	CYTO	W5PEQ9	SRSF1	7.01E-03	0.000
3	CYTO	W5QHZ0	CA12	1.44E-02	0.000
3	CYTO	W5QFB2	OLFML3	1.19E-02	0.000
3	CYTO	P14639	ALB	3.15E-03	0.000
3	CYTO	W5P0K3	MBNL1	2.13E-03	0.000
3	CYTO	W5QF73	NDUFB4	2.02E-02	0.000
3	CYTO	W5PEB0	FABP7	4.08E-03	0.000
3	CYTO	W5NSC5		3.30E-02	0.000
3	CYTO	W5Q2S8	MYL9	5.83E-03	0.000
3	CYTO	W5NUK5	HAT1	5.93E-05	0.000
3	CYTO	W5PQD8	C1QTNF3	4.36E-04	0.000
3	CYTO	W5PJR5	LOC101121216	3.26E-03	0.000
3	CYTO	W5PAL2	LOC101105297	7.25E-03	0.000
3	CYTO	W5Q7N9	MTX2	4.70E-04	0.000
3	CYTO	W5QGV7		2.61E-03	0.000
3	CYTO	W5PJV4	ASGR2	1.68E-02	0.000
3	CYTO	W5NZH3		1.08E-03	0.000
3	CYTO	W5PDP2	CD55	4.23E-03	0.000
3	CYTO	W5Q1X3	LRRC8C	2.22E-02	0.000
3	CYTO	W5Q4B1	MYCBP2	2.40E-02	0.000
3	CYTO	W5PJS4	EMILIN2	1.12E-02	0.000
3	CYTO	W5PEI4	KLKB1	6.63E-04	0.000
3	CYTO	W5PTI0	CD37	4.29E-02	0.000
3	CYTO	W5PBE1	ISG15	4.73E-04	0.000
3	CYTO	W5PFP1	LTF	2.01E-02	0.000
3	CYTO	W5NZ47	RBP4	4.75E-02	0.000
3	CYTO	W5P2F6	FAF1	2.97E-05	0.000
3	CYTO	W5NTE2	PSMG4	3.27E-03	0.000
3	CYTO	W5QIR2	C7H15orf48	5.30E-02	0.000
3	CYTO	W5P4E2	PBLD	1.71E-03	0.000
3	CYTO	W5P530	LOC101104705	3.15E-04	0.000
3	CYTO	W5QHV3	FABP1	1.36E-03	0.000
3	CYTO	W5PUH5	LGMN	4.83E-04	0.000
3	CYTO	W5PYQ9	CPNE1	1.65E-03	0.000
3	CYTO	W5P935	APMAP	3.89E-03	0.000
3	CYTO	W5PU89	ALDH16A1	5.82E-05	0.000
3	CYTO	W5P9K4	NUFIP2	3.09E-03	0.000
3	CYTO	W5NZJ7	FGR	9.59E-03	0.000
3	CYTO	W5Q7X3	GNPNAT1	1.37E-02	0.000
3	CYTO	Q28893	IGFBP4	9.94E-04	0.000
3	CYTO	W5QJA2	CD14	4.46E-02	0.000
3	CYTO	W5PJF2	EXT1	1.28E-03	0.000
3	CYTO	W5QI29	ECM1	2.61E-02	0.000

Supplementary Table 20: Differentially abundant proteins (DAPs) of M1 relative to M2 and their cellular location.

FoldChange	Location	Protein IDs	Gene Names	P-value	FDR
3	CYTO	W5PCA0	ALDOB	2.02E-04	0.000
3	CYTO	W5PDS4	C1QA	1.13E-02	0.000
3	CYTO	W5PRB3		1.47E-04	0.000
3	CYTO	W5QHL1	FCGR1A	1.85E-02	0.000
3	CYTO	W5PEY2	CTSV	1.05E-02	0.000
3	CYTO	W5Q4Z3		4.87E-02	0.000
3	CYTO	W5PWV6	UBE2B	7.98E-04	0.000
3	CYTO	W5NXT4		6.42E-04	0.000
3	CYTO	W5QGR5	WDFY1	1.08E-04	0.000
3	CYTO	W5PY97	SVIL	3.98E-02	0.000
3	CYTO	W5P874	ABHD16A	2.18E-04	0.000
3	CYTO	W5QCG0		3.84E-04	0.000
3	CYTO	P80190		2.31E-02	0.000
3	CYTO	W5QCT2		3.87E-03	0.000
3	CYTO	W5NQH6	S100A9	6.23E-03	0.000
3	CYTO	W5PBS5	ABCA1	9.42E-04	0.000
3	CYTO	W5PHN9	F11R	1.16E-02	0.000
3	CYTO	W5Q5N6	BST-2B	1.28E-02	0.000
3	CYTO	W5PPS6		8.61E-04	0.000
3	CYTO	W5QET9	PLA1A	7.47E-05	0.000
3	CYTO	W5PF68	EMB	1.09E-03	0.000
3	CYTO	W5NVG6	PTPN9	6.64E-04	0.000
3	CYTO	W5PH99	FYCO1	4.19E-04	0.000
3	CYTO	W5P5T4	LOC101108131	3.55E-04	0.000
3	CYTO	W5Q761	UBE2D2	5.32E-03	0.000
3	CYTO	W5PNK3	ACAN	8.05E-04	0.000
3	CYTO	W5PQE5	SERPINH1	1.21E-02	0.000
3	CYTO	W5Q3K9	IL2RG	1.56E-04	0.000
3	CYTO	W5PSM6	HABP2	1.31E-03	0.000
3	CYTO	W5QCY7	SPP2	4.71E-04	0.000
3	CYTO	W5QAX4	DHRS11	7.78E-03	0.000
3	CYTO	W5PDP6	C1QC	1.80E-04	0.000
3	CYTO	W5PGA9	NCSTN	5.29E-03	0.000
3	CYTO	W5P631	KLC4	6.88E-06	0.000
3	CYTO	W5PDQ0		6.58E-03	0.000
3	CYTO	W5NWX6	APOC3	2.21E-02	0.000
3	CYTO	W5PCL0	LOC101103343	5.33E-02	0.000
3	CYTO	W5Q686	TPP1	1.09E-04	0.000
3	CYTO	W5NRD1	ERLIN2	2.43E-02	0.000
3	CYTO	W5Q7T8	THBS4	5.92E-03	0.000
3	CYTO	W5QH64	SNAP23	7.48E-04	0.000
3	CYTO	W5PXT1	MYO1G	1.37E-02	0.000
3	CYTO	W5PW33		2.07E-02	0.000
3	CYTO	W5NYA8	SCAMP2	5.88E-03	0.000
3	CYTO	W5QGP4	APOD	4.35E-05	0.000
3	CYTO	W5PRS6	CRK	3.95E-02	0.000

Supplementary Table 20: Differentially abundant proteins (DAPs) of M1 relative to M2 and their cellular location.

FoldChange	Location	Protein IDs	Gene Names	P-value	FDR
3	CYTO	W5Q3B2	TYK2	5.02E-03	0.000
3	CYTO	W5PGT0	MYH11	3.32E-03	0.000
2	CYTO	W5PD71	LOC101115495	1.38E-03	0.001
2	CYTO	W5PMU8	GAB1	9.44E-03	0.000
2	CYTO	W5Q4U5	CPT1A	1.03E-03	0.000
2	CYTO	W5QA17	ATXN2	1.26E-02	0.000
2	CYTO	W5Q1G8	LOC101120875	2.88E-02	0.000
2	CYTO	W5NUJ5		2.91E-03	0.000
2	CYTO	W5Q9U4	LOC101104050	1.45E-02	0.000
2	CYTO	W5PR51	CPNE3	1.80E-02	0.000
2	CYTO	W5PBE7	ELOA	3.93E-02	0.000
2	CYTO	W5PCI1	NLRP3	4.89E-03	0.000
2	CYTO	W5PD25	CYTIP	1.16E-03	0.000
2	CYTO	W5Q878	PRKCB	1.27E-02	0.000
2	CYTO	W5PY22		8.73E-03	0.000
2	CYTO	W5PE73	SMPDL3A	1.88E-02	0.000
2	CYTO	W5PFI7	VCL	2.71E-02	0.000
2	CYTO	W5PC20	VPS37B	1.25E-03	0.000
2	CYTO	P21621	IL1B	2.62E-02	0.000
2	CYTO	W5PNC9	CRYBG1	6.54E-04	0.000
2	CYTO	W5QGT0	ATP13A3	1.30E-03	0.000
2	CYTO	W5Q0F3	TGFBI	3.13E-02	0.000
2	CYTO	W5PM73	UAP1	3.09E-02	0.000
2	CYTO	W5NTZ8	RCN2	5.31E-02	0.000
2	CYTO	W5PC47	LOC101115694	5.26E-02	0.000
2	CYTO	W5Q1F6	AIFM1	3.99E-04	0.000
2	CYTO	W5PL47	BMP1	4.07E-03	0.000
2	CYTO	W5Q7J4	LOC101107035	7.54E-03	0.000
2	CYTO	W5NTM0		1.22E-02	0.000
2	CYTO	W5NWM7	GSTZ1	1.53E-02	0.000
2	CYTO	W5QF96	INPP5B	5.89E-04	0.000
2	CYTO	W5Q337	GLRX5	1.29E-02	0.000
2	CYTO	W5PJF3	PPIF	3.31E-02	0.000
2	CYTO	P29330	FDX1	2.23E-03	0.000
2	CYTO	W5PIL2	LOC101105132	2.70E-03	0.000
2	CYTO	W5PZI8		3.29E-02	0.000
2	CYTO	W5P601	STOM	7.83E-03	0.000
2	CYTO	W5Q3Z5	STEEP1	2.24E-02	0.000
2	CYTO	W5PU46	TMEM256	7.98E-03	0.000
2	CYTO	W5PIU9	ARMC6	3.11E-03	0.000
2	CYTO	W5PB20		3.59E-03	0.000
2	CYTO	W5QG77	CD58	1.01E-03	0.000
2	CYTO	W5PK31	FCGR3A	1.58E-02	0.000
2	CYTO	W5P5V0	COLEC11	1.33E-04	0.000
2	CYTO	W5QAJ8	DESI1	1.46E-02	0.000
2	CYTO	W5PW30	RBIS	5.21E-02	0.000

Supplementary Table 20: Differentially abundant proteins (DAPs) of M1 relative to M2 and their cellular location.

FoldChange	Location	Protein IDs	Gene Names	P-value	FDR
2	CYTO	W5NWW4	CREBBP	5.06E-03	0.000
2	CYTO	C5IS96	LCAT	1.35E-03	0.000
2	CYTO	W5PNP1	MFGE8	2.04E-02	0.000
2	CYTO	W5PN18	NCF1	7.30E-05	0.000
2	CYTO	W5QJ55	VTI1B	3.56E-02	0.000
2	CYTO	W5NSJ5	OXSR1	2.54E-02	0.000
2	CYTO	W5P848	MLKL	4.25E-02	0.000
2	CYTO	W5P669		1.04E-03	0.000
2	CYTO	W5PDQ3	SAFB2	2.83E-02	0.000
2	CYTO	W5NYT0	APOBR	1.09E-02	0.000
2	CYTO	B3VSB9	ITM2B	3.09E-02	0.000
2	CYTO	W5PVC8	ERGIC3	1.76E-02	0.000
2	CYTO	W5Q0A3	TLR2	1.12E-05	0.000
2	CYTO	W5NZ42	SLC2A6	1.90E-03	0.000
2	CYTO	W5Q1S6	EML3	7.61E-04	0.000
2	CYTO	W5Q7J0	APOB	7.46E-05	0.000
2	CYTO	W5NUE5	SLC38A7	1.32E-02	0.000
2	CYTO	W5Q0Q5	ATP5PF	4.00E-02	0.000
2	CYTO	W5NVV1		1.10E-02	0.000
2	CYTO	W5PJP5	RGS18	8.51E-03	0.000
2	CYTO	W5PIL5		3.45E-02	0.000
2	CYTO	W5P4U4	GRK6	1.48E-03	0.000
2	CYTO	W5P2F1	FOLR2	1.06E-02	0.000
2	CYTO	W5PBK8	SNX11	3.19E-02	0.000
2	CYTO	W5NXN2		1.52E-03	0.000
2	CYTO	W5NX28	MKNK1	7.76E-03	0.000
2	CYTO	W5Q2C9		1.30E-04	0.000
2	CYTO	W5PAR2		8.57E-04	0.000
2	CYTO	W5PZB7	RIGI	4.65E-02	0.000
2	CYTO	W5QIE9	BSDC1	2.69E-03	0.000
2	CYTO	W5P9X0		5.16E-02	0.000
2	CYTO	W5NTT7	COL1A2	2.42E-02	0.000
2	CYTO	W5P6U2		2.40E-04	0.000
2	CYTO	W5P093	NQO1	2.78E-03	0.000
2	CYTO	W5P5F6	TBCB	3.49E-02	0.000
2	CYTO	W5PDH4	MMP9	8.69E-03	0.000
2	CYTO	C5ISA2	TUBA4A	2.81E-04	0.000
2	CYTO	W5NTA0		1.52E-03	0.000
2	CYTO	W5P6U5	LMAN1	6.99E-03	0.000
2	CYTO	W5P2F2		3.89E-02	0.000
2	CYTO	W5NUX5		4.85E-03	0.000
2	CYTO	W5NXU7	HCK	2.10E-03	0.000
2	CYTO	W5PFY8	MTSS1	2.33E-02	0.000
2	CYTO	W5QCD3	RNASEL	6.04E-04	0.000
2	CYTO	W5P590	OPTN	3.75E-05	0.000
2	CYTO	W5Q7R3	LOC101118736	2.16E-02	0.000

Supplementary Table 20: Differentially abundant proteins (DAPs) of M1 relative to M2 and their cellular location.

FoldChange	Location	Protein IDs	Gene Names	P-value	FDR
2	CYTO	W5Q234	RAF1	1.18E-02	0.000
2	CYTO	W5QGV5	DOCK10	2.57E-05	0.000
2	CYTO	W5P9Z9	CSNK2B	1.20E-02	0.000
2	CYTO	W5Q2G2	IL16	2.47E-02	0.000
2	CYTO	W5P773		1.86E-02	0.000
2	CYTO	W5NYC4		1.61E-02	0.000
2	CYTO	W5Q5P5	GGH	3.41E-02	0.000
2	CYTO	W5Q1F2		8.09E-03	0.000
2	CYTO	W5P8S0	LY96	2.06E-02	0.000
2	CYTO	W5PVB4	PTPN1	2.84E-02	0.000
2	CYTO	W5Q634	BLVRA	2.15E-02	0.000
2	CYTO	W5P7I1	ACTR3B	3.14E-02	0.000
2	CYTO	W5P582	ACP1	1.23E-02	0.000
2	CYTO	W5PIT5	PPOX	4.68E-02	0.000
2	CYTO	W5PKQ2	PRXL2A	3.73E-02	0.000
2	CYTO	W5QIQ6	SQOR	1.24E-03	0.000
2	CYTO	W5P6M5	SEC11C	6.67E-06	0.002
2	CYTO	W5PAP1	TERF2IP	4.22E-02	0.000
2	CYTO	W5PUV6	NCAPD3	1.41E-03	0.000
2	CYTO	W5Q4P9	CYRIA FAM49A	3.91E-04	0.000
2	CYTO	W5PR43		5.30E-02	0.000
2	CYTO	W5PMH4	RAB39A	5.36E-04	0.000
2	CYTO	W5NXF0	SQSTM1	3.37E-03	0.000
2	CYTO	W5PB46	PLTP	3.11E-02	0.000
2	CYTO	W5NVK6	DAGLB	2.69E-03	0.000
2	CYTO	W5QCL1	NCF4	7.65E-04	0.000
2	CYTO	W5PI45	ENOSF1	1.91E-03	0.000
2	CYTO	W5NZM0	VDAC3	1.65E-04	0.000
2	CYTO	W5QHF2	LOC114117536	9.45E-03	0.000
2	CYTO	W5PXI2		5.48E-03	0.000
2	CYTO	W5PVI4	PLGRKT	1.66E-02	0.000
2	CYTO	W5PHM1	ALOX12	5.55E-02	0.000
2	CYTO	W5QB58	AASS	4.80E-05	0.000
2	CYTO	W5QEK8	EHD3	1.03E-02	0.000
2	CYTO	W5NQ60	POLR2C	3.39E-03	0.000
2	CYTO	W5PXI9	MRRF	7.82E-03	0.000
2	CYTO	W5PND9		1.07E-03	0.000
2	CYTO	W5PSL9	HPD	2.15E-02	0.000
2	CYTO	W5PZD2	ALDH8A1	4.93E-02	0.000
2	CYTO	W5QHX9	PLBD1	9.49E-04	0.000
2	CYTO	W5PKX6	DCTN6	1.47E-02	0.000
2	CYTO	W5P6Q7	MPIG6B	4.68E-02	0.000
2	CYTO	W5PRM0	GMPR	4.14E-02	0.000
2	CYTO	W5P8F3	AGPAT5	8.19E-03	0.000
2	CYTO	W5PZ10	CMPK2	1.78E-02	0.000
2	CYTO	W5NYG8	TSSC4	1.55E-02	0.000

Supplementary Table 20: Differentially abundant proteins (DAPs) of M1 relative to M2 and their cellular location.

FoldChange	Location	Protein IDs	Gene Names	P-value	FDR
2	CYTO	W5NZU5		2.29E-03	0.000
2	CYTO	W5PKY9	MRCL3	1.86E-03	0.000
2	CYTO	W5NXX6	PSTPIP2	2.41E-02	0.000
2	CYTO	W5PZR1		2.06E-02	0.000
2	CYTO	W5QFD0	RRAGC	1.44E-04	0.000
2	CYTO	W5PX94	CELF2	2.39E-03	0.000
2	CYTO	W5NUU7	CDH5	2.59E-02	0.000
2	CYTO	W5Q411	HSPA13	8.64E-04	0.000
2	CYTO	W5NQG2	TBC1D2B	1.17E-02	0.000
2	CYTO	W5NS07	HM13	3.76E-02	0.000
2	CYTO	W5P2Z8	RGS14	5.57E-02	0.000
2	CYTO	W5PMF1	TPMT	2.69E-05	0.000
2	CYTO	W5PMR2	MMP19	2.72E-02	0.000
2	CYTO	W5P4I3	TRAF3	2.33E-02	0.000
2	CYTO	W5QD80	SAMM50	1.62E-03	0.000
2	CYTO	W5QAQ9	SNU13	3.94E-02	0.000
2	CYTO	W5NUW8	TREM1	9.46E-04	0.000
2	CYTO	W5PZJ8	ASRGL1	1.02E-03	0.000
2	CYTO	W5PFR7	ETHE1	1.45E-03	0.000
2	CYTO	W5PED5	LOC101116121	1.91E-02	0.000
2	CYTO	W5NZD5	POLB	1.19E-02	0.000
2	CYTO	W5NX57	CHM	1.29E-02	0.000
2	CYTO	W5Q0Q6	EXOSC4	2.89E-02	0.000
2	CYTO	W5PNL4	NCBP1	3.51E-03	0.000
2	CYTO	W5PK60	CLCN5	3.15E-02	0.000
2	CYTO	W5QFQ8	LOC101111915	4.05E-04	0.000
2	CYTO	W5P1B9	SEC16A	1.92E-02	0.000
2	CYTO	W5PNS4		1.76E-03	0.009
2	CYTO	B2LYK6	RAB7A	1.49E-05	0.000
2	CYTO	C0IZ95	RAB27A	4.61E-02	0.000
2	CYTO	W5PDR1	RAB6A	1.54E-04	0.000
2	CYTO	W5PL70	PDCD4	4.85E-02	0.000
2	CYTO	W5PFL8	CD9	2.09E-02	0.000
2	CYTO	W5QAN4	UBASH3B	1.89E-02	0.000
2	CYTO	W5Q418	NELFCD	1.40E-03	0.000
2	CYTO	W5Q9U3	CNP	1.57E-02	0.000
2	CYTO	W5P583	ACP1	4.64E-02	0.000
2	CYTO	W5PT49	NFKB1	9.96E-05	0.000
2	CYTO	W5Q2V2	PAH	2.88E-02	0.000
2	CYTO	W5P745		3.15E-02	0.000
2	CYTO	W5PAF8	CELF1	9.19E-03	0.000
2	CYTO	W5P6H9	ACP2	2.27E-05	0.000
-2	CYTO	W5PME7	PALS2	3.28E-02	0.000
-2	CYTO	W5QHT8	TRIP4	2.49E-02	0.000
-2	CYTO	W5P7K1	TBC1D10B	3.29E-03	0.000
-2	CYTO	W5PND1		5.58E-05	0.000

Supplementary Table 20: Differentially abundant proteins (DAPs) of M1 relative to M2 and their cellular location.

FoldChange	Location	Protein IDs	Gene Names	P-value	FDR
-2	CYTO	W5PHX1	AHCY	2.68E-04	0.000
-2	CYTO	W5PEZ1		7.39E-03	0.000
-2	CYTO	W5P2W9	LOC100037668 LOC1	2.21E-04	0.000
-2	CYTO	W5P5T8	RPS7	3.46E-05	0.000
-2	CYTO	O18882	ATP6V0C ATP6C ATP(	3.03E-06	0.000
-2	CYTO	W5QC01	GHDC	4.26E-02	0.000
-2	CYTO	W5PSM4	LSM4	2.72E-04	0.000
-2	CYTO	W5PRM1	SNRPB2	3.51E-03	0.000
-2	CYTO	W5QFF9		1.80E-04	0.000
-2	CYTO	W5QIW1	LGALS3	6.81E-05	0.000
-2	CYTO	W5PNH4	ATG5	5.47E-02	0.000
-2	CYTO	W5PC55	CASP3	1.62E-02	0.000
-2	CYTO	W5P2D7	ARID1A	5.46E-04	0.000
-2	CYTO	W5PS05	MTHFD2	3.59E-02	0.000
-2	CYTO	W5NSE1	DRG2	1.37E-02	0.000
-2	CYTO	W5PIY3		2.44E-03	0.000
-2	CYTO	W5PTD1	VPS11	2.93E-02	0.000
-2	CYTO	W5QJ45	GPHN	3.41E-02	0.000
-2	CYTO	W5NQX0	ARHGEF18	1.68E-02	0.000
-2	CYTO	W5PX73	SPG7	2.35E-02	0.000
-2	CYTO	W5QDA7	AP1G2	1.17E-02	0.000
-2	CYTO	W5PY18	LOC101118100	6.41E-04	0.000
-2	CYTO	E7ECV8	NUDT9	2.11E-03	0.000
-2	CYTO	W5P4G4	RAB7B	1.40E-02	0.000
-2	CYTO	W5P4Z0	FAM120B	4.56E-02	0.000
-2	CYTO	W5PTU8	MARK1	5.28E-02	0.009
-2	CYTO	W5PHY4	TYMS	1.86E-02	0.000
-2	CYTO	W5PS90	DYNLL1	8.56E-04	0.000
-2	CYTO	W5QI72	BORCS5	6.52E-04	0.000
-2	CYTO	W5Q728	TPCN2	1.46E-02	0.000
-2	CYTO	W5P797	U2SURP	2.32E-02	0.000
-2	CYTO	W5QGQ8	SUCLG1	4.57E-03	0.000
-2	CYTO	W5NQ27	ZFYVE1	1.25E-03	0.000
-2	CYTO	W5PPE2	MGST3	1.37E-02	0.000
-2	CYTO	W5QDT3	DAD1	1.59E-03	0.000
-2	CYTO	W5Q2G7	NSMAF	3.85E-02	0.000
-2	CYTO	W5PK71	SUMF2	2.17E-04	0.000
-2	CYTO	W5NXF6	TUBG1	4.32E-03	0.000
-2	CYTO	W5PPR1	UCK2	1.01E-02	0.000
-2	CYTO	W5PR04	IDH2	2.15E-06	0.000
-2	CYTO	W5PQL2	SRSF11	2.06E-02	0.000
-2	CYTO	W5P1M3		3.06E-03	0.000
-2	CYTO	W5P2K4	CLYBL	4.05E-04	0.000
-2	CYTO	W5PUS4	SMARCB1	2.97E-03	0.000
-2	CYTO	W5PN75	TBC1D2	3.23E-03	0.000
-2	CYTO	W5QFV9	PPIE	1.87E-02	0.000

Supplementary Table 20: Differentially abundant proteins (DAPs) of M1 relative to M2 and their cellular location.

FoldChange	Location	Protein IDs	Gene Names	P-value	FDR
-2	CYTO	W5P1U7	PTPN13	1.42E-02	0.009
-2	CYTO	W5PR38	DDX18	8.80E-03	0.000
-2	CYTO	W5Q3E5	METAP2	1.20E-03	0.000
-2	CYTO	W5NSS0	ARAP3	1.25E-02	0.000
-2	CYTO	W5QEM1	TIA1	1.49E-05	0.000
-2	CYTO	W5QCD6	IDH1	3.56E-06	0.000
-2	CYTO	W5NWL0	CCDC88A	4.27E-03	0.000
-2	CYTO	W5QJ40	FNTB	2.83E-03	0.000
-2	CYTO	W5Q644	VPS29	6.81E-04	0.000
-2	CYTO	W5Q6J7	TBCA	2.81E-03	0.000
-2	CYTO	W5PG83	PTPA	3.38E-04	0.000
-2	CYTO	W5PA68	TRMT112	1.30E-04	0.000
-2	CYTO	W5NWX7	CLEC4A	2.85E-02	0.000
-2	CYTO	W5PMW6		3.22E-02	0.000
-2	CYTO	W5PFB4	DENR	5.31E-04	0.000
-2	CYTO	W5QGK9	NDUFAF1	6.05E-03	0.000
-2	CYTO	W5Q2R9		3.99E-06	0.000
-2	CYTO	W5Q4D0	GAK	1.95E-05	0.000
-2	CYTO	W5P0D5	PSMD7	4.62E-04	0.000
-2	CYTO	W5NZ14	SHC1	4.40E-02	0.000
-2	CYTO	W5NWZ2	CIAO2B	1.64E-02	0.000
-2	CYTO	C8BKD3	ASF1A	4.89E-02	0.000
-2	CYTO	W5Q2L6	EXOC3	5.56E-04	0.000
-2	CYTO	W5Q000	RPL32	2.60E-02	0.000
-2	CYTO	W5PPK3	SNX15	3.90E-02	0.002
-2	CYTO	W5PPQ3	TRAPPC10	2.64E-03	0.000
-2	CYTO	W5PIE3	PPME1	4.49E-04	0.000
-2	CYTO	W5Q740	ABCD3	1.41E-03	0.000
-2	CYTO	W5PJU6	FARSA	3.31E-05	0.000
-2	CYTO	W5P1K4	TNPO3	1.50E-02	0.000
-2	CYTO	W5QGG8	MAN1A2	8.11E-03	0.000
-2	CYTO	W5Q527	PAPOLA	5.58E-03	0.000
-2	CYTO	W5P3L4	ASNS	6.79E-03	0.002
-2	CYTO	W5NZ21	LOC101105090	4.08E-04	0.000
-2	CYTO	W5P994	LONP1	1.12E-04	0.000
-2	CYTO	W5QGX6	C1H1orf50	4.66E-05	0.000
-2	CYTO	W5Q9W6	RTF2	2.37E-03	0.000
-2	CYTO	W5Q766	TSC2	3.70E-02	0.000
-2	CYTO	W5QF41	ARHGAP25	1.51E-05	0.000
-2	CYTO	W5PQ37	TOR2A	6.61E-04	0.000
-2	CYTO	W5NT95	ATP6AP2	3.39E-03	0.000
-2	CYTO	W5Q3Y0	GLMN	4.86E-03	0.000
-2	CYTO	W5PI67	IDS	7.15E-03	0.000
-2	CYTO	W5PYH5	EFL1	2.02E-03	0.000
-2	CYTO	W5P0T8	PSME4	6.54E-03	0.000
-2	CYTO	W5PBX4	FAR1	9.64E-03	0.000

Supplementary Table 20: Differentially abundant proteins (DAPs) of M1 relative to M2 and their cellular location.

FoldChange	Location	Protein IDs	Gene Names	P-value	FDR
-2	CYTO	W5PZN5	KIF3A	3.20E-02	0.000
-2	CYTO	W5QIM8	OLR1	3.26E-02	0.000
-2	CYTO	W5QFC2	FHL3	4.13E-03	0.000
-2	CYTO	W5P684	ACTBL2	4.28E-02	0.004
-2	CYTO	P81184	LGALS1	2.96E-05	0.000
-2	CYTO	W5PUF8	PITRM1	1.28E-02	0.000
-2	CYTO	W5NWF5	RARRES1	2.66E-02	0.000
-2	CYTO	W5PCM4	LUZP1	3.25E-04	0.000
-2	CYTO	W5QB36	CAT	1.52E-05	0.000
-2	CYTO	W5P8H4	DCTPP1	2.10E-02	0.000
-2	CYTO	W5QDC0	SNX17	3.53E-03	0.000
-2	CYTO	W5PFH7	DCAF8	8.40E-04	0.000
-2	CYTO	W5PPG7	NUP153	4.20E-02	0.000
-2	CYTO	W5PMY2	USP10	1.42E-02	0.000
-2	CYTO	W5PTW0	GNL1	4.57E-02	0.000
-2	CYTO	W5PB87	VWA8	4.90E-02	0.000
-2	CYTO	W5QA79	XPR1	2.09E-02	0.000
-2	CYTO	W5P4K6	FCHO2	9.34E-06	0.000
-2	CYTO	W5P995	LRPPRC	4.65E-05	0.000
-2	CYTO	W5PCZ2	PPP3CB	3.46E-02	0.000
-2	CYTO	W5NX76	MAP2K3	2.06E-03	0.000
-2	CYTO	W5PZS4	OSBPL8	2.15E-05	0.000
-2	CYTO	W5PPU8		4.98E-03	0.000
-2	CYTO	W5PIK1	LZTFL1	1.32E-02	0.000
-2	CYTO	W5PC19	STX18	4.36E-05	0.000
-2	CYTO	W5PPE7		8.55E-05	0.000
-2	CYTO	W5Q175	GNPTAB	1.37E-04	0.000
-2	CYTO	W5NUX4	WDR48	1.29E-04	0.000
-2	CYTO	W5QBG8	PPFIA1	7.38E-03	0.000
-2	CYTO	W5NXT6	UBR3	3.38E-03	0.000
-2	CYTO	W5NUJ2	PEAK1	3.66E-02	0.000
-2	CYTO	W5P0K1	BECN1	2.03E-03	0.000
-2	CYTO	W5NWM6	GFM1 EFG1	2.30E-03	0.000
-2	CYTO	W5QHF9	EIF2B5	1.84E-03	0.000
-2	CYTO	W5PCH3	SCIN	9.57E-04	0.000
-2	CYTO	W5PHI1	MRPL3	4.62E-02	0.000
-2	CYTO	W5NYS1	SRSF6	3.60E-02	0.000
-2	CYTO	W5PC18		1.77E-05	0.000
-2	CYTO	W5QGS1	MRPL44	8.81E-03	0.000
-2	CYTO	W5PI02	TBC1D13	1.46E-05	0.000
-2	CYTO	W5Q501	LOC101115640	1.00E-03	0.000
-2	CYTO	W5Q8M9	PYCR2	3.19E-02	0.000
-2	CYTO	W5Q518	MRPL17	4.24E-02	0.000
-2	CYTO	W5NX56	SPP1	1.40E-02	0.000
-2	CYTO	W5Q5Z1	YTHDF3	3.59E-05	0.000
-2	CYTO	W5QEL5	MRPS35	6.57E-04	0.000

Supplementary Table 20: Differentially abundant proteins (DAPs) of M1 relative to M2 and their cellular location.

FoldChange	Location	Protein IDs	Gene Names	P-value	FDR
-2	CYTO	W5PP30	ASNA1 GET3	8.69E-06	0.000
-2	CYTO	W5QEH3	RNF25	4.71E-06	0.000
-2	CYTO	W5P867	MAPKAPK2	3.74E-02	0.000
-2	CYTO	W5PJL3		1.34E-03	0.000
-2	CYTO	W5PMG3	MEMO1	1.73E-04	0.000
-2	CYTO	W5P612	CRELD1	1.47E-02	0.000
-2	CYTO	W5PU58	UBLCP1	1.59E-04	0.000
-2	CYTO	W5P929	MARS1	9.90E-04	0.000
-2	CYTO	W5PV12	MIPEP	1.30E-03	0.000
-2	CYTO	W5PXJ1	MAT2B	8.83E-05	0.000
-2	CYTO	B0LRP7	SRP14	2.16E-04	0.000
-2	CYTO	W5P5Q2	MVP	3.26E-02	0.000
-2	CYTO	W5P023		5.48E-05	0.000
-2	CYTO	W5PQD7	GID8	3.22E-04	0.000
-2	CYTO	W5NV88	KPNA4	2.16E-03	0.000
-2	CYTO	W5Q6N3	LOC101115115	2.90E-02	0.000
-2	CYTO	W5PSY5	RAB3GAP2	3.51E-02	0.000
-2	CYTO	W5NYJ4	DKC1	7.16E-05	0.000
-2	CYTO	W5QEJ7	ARHGEF40	1.56E-03	0.000
-2	CYTO	W5PJQ5	HEATR5B	3.40E-05	0.000
-2	CYTO	W5P4Z8	MRPS27	1.02E-04	0.000
-2	CYTO	W5PUW9	PDCL3	3.07E-03	0.000
-2	CYTO	W5QIA5	ETV6	1.68E-02	0.000
-2	CYTO	W5Q088	CBL	5.06E-02	0.000
-2	CYTO	W5Q8K0	RBBP6	5.93E-04	0.000
-2	CYTO	W5PC93	CCDC97	2.06E-02	0.000
-2	CYTO	W5QGB2	RMDN3	1.37E-02	0.000
-2	CYTO	W5PNN1	AMPD3	4.31E-02	0.006
-2	CYTO	W5Q5T7	ATP6V1C1	2.14E-04	0.000
-2	CYTO	W5QA63	FRAS1	1.58E-02	0.008
-2	CYTO	W5PGB0	SIRT3	5.72E-04	0.000
-2	CYTO	W5P3N2	FLOT2	9.94E-03	0.000
-2	CYTO	W5Q2Y8	EIF4E	1.07E-05	0.000
-2	CYTO	W5PXK7	RASA4B	8.95E-03	0.000
-2	CYTO	W5PTQ7	TRAF3IP3	2.22E-02	0.000
-2	CYTO	W5PJJ3	LARP1	1.85E-04	0.000
-2	CYTO	W5QHG2	PTCD3	1.19E-04	0.000
-2	CYTO	W5NQC8	RPL27A	3.36E-04	0.000
-2	CYTO	W5Q758	DCTN5	1.51E-02	0.000
-2	CYTO	W5PIA7	PSMG1	6.96E-03	0.000
-2	CYTO	W5PLQ3	LARS2	5.14E-04	0.000
-2	CYTO	W5P5C2	SPAG7	1.02E-04	0.000
-2	CYTO	W5PJ58	HPS5	4.36E-02	0.000
-2	CYTO	W5Q5K4	LOC101106166	1.50E-04	0.000
-2	CYTO	W5PMK9	MRPS11	1.30E-03	0.000
-2	CYTO	W5NSD9	REXO2	3.50E-05	0.000

Supplementary Table 20: Differentially abundant proteins (DAPs) of M1 relative to M2 and their cellular location.

FoldChange	Location	Protein IDs	Gene Names	P-value	FDR
-2	CYTO	W5PXV9	SLC4A7	6.76E-03	0.000
-2	CYTO	W5PFP6	TRNT1	3.52E-06	0.000
-2	CYTO	W5PCC0	LACC1	1.98E-02	0.000
-2	CYTO	W5PX15	SLC30A9	7.82E-03	0.000
-2	CYTO	W5PQ79		7.23E-06	0.000
-2	CYTO	W5NWE0	MECR	7.47E-03	0.000
-2	CYTO	W5PFL3	PAIP1	8.01E-04	0.000
-2	CYTO	W5PWZ8	SNRNP200	3.79E-04	0.000
-2	CYTO	W5QGC6	FYTTD1	2.02E-03	0.000
-2	CYTO	W5NQR1		1.44E-04	0.000
-2	CYTO	W5PDU4	NMT2	8.18E-04	0.000
-2	CYTO	W5PNG4	BRCA2	3.09E-02	0.005
-2	CYTO	P68251	YWHAB	1.60E-02	0.000
-2	CYTO	W5NUR8		9.83E-03	0.000
-2	CYTO	W5PRN2	BUD23	3.19E-03	0.000
-2	CYTO	W5P7B0	DDX31	2.54E-03	0.000
-2	CYTO	W5PGI5	INTS6	1.68E-04	0.000
-2	CYTO	W5NZ71		2.49E-04	0.000
-2	CYTO	W5Q562	ZC3H15	1.15E-02	0.000
-2	CYTO	W5Q3C1	NDRG3	7.73E-05	0.000
-2	CYTO	W5Q3R4	CLUH	1.68E-02	0.000
-2	CYTO	W5NXV1	PACS1	9.66E-06	0.000
-2	CYTO	W5PJ85	SMCHD1	2.98E-02	0.000
-2	CYTO	W5P6R0	COPG2	5.16E-02	0.000
-2	CYTO	W5PX32	EPM2AIP1	2.15E-02	0.000
-2	CYTO	W5QEL9	ARHGAP31	1.04E-02	0.000
-2	CYTO	W5PRB0	UFL1	4.10E-04	0.000
-2	CYTO	W5PPX0	LYPLAL1	1.04E-03	0.000
-2	CYTO	W5PBA2	BAG6	2.19E-02	0.000
-2	CYTO	W5Q2P3		8.89E-05	0.000
-2	CYTO	W5QEA6	WDR77	1.19E-05	0.000
-2	CYTO	W5PVR9	ERMP1	3.48E-02	0.000
-2	CYTO	W5Q4B7	RAP1GDS1	3.25E-02	0.000
-2	CYTO	W5NT32	CRIP2	1.64E-03	0.000
-2	CYTO	W5NZD0	EXOC2	2.01E-03	0.000
-2	CYTO	W5NS73		1.17E-02	0.003
-2	CYTO	W5P7A8	TRMT61A	2.45E-03	0.000
-2	CYTO	W5NX16		4.92E-02	0.000
-2	CYTO	W5QDZ8	AAMP	1.12E-03	0.000
-2	CYTO	W5Q4G2		1.60E-02	0.000
-2	CYTO	W5PHV7	SLC43A3	2.95E-02	0.000
-2	CYTO	W5PHL8	NUDT16	1.12E-03	0.000
-2	CYTO	W5NY25	EIF3C EIF3S8 LOC101	7.43E-04	0.000
-2	CYTO	B9VH02	EIF1AX	1.68E-04	0.000
-2	CYTO	W5P9E3		2.92E-02	0.000
-2	CYTO	W5Q4U9	NUBP2	3.11E-02	0.000

Supplementary Table 20: Differentially abundant proteins (DAPs) of M1 relative to M2 and their cellular location.

FoldChange	Location	Protein IDs	Gene Names	P-value	FDR
-2	CYTO	W5Q413	SMS	1.68E-03	0.000
-2	CYTO	W5QET8	TEP1	3.33E-02	0.000
-2	CYTO	W5Q336	MCEE	2.00E-02	0.000
-2	CYTO	W5PU49	SDHAF2 PGL2 SDH5	7.46E-03	0.000
-2	CYTO	W5PQM4	MOSPD2	7.78E-04	0.000
-2	CYTO	W5PIB9	NFX1	1.38E-02	0.000
-2	CYTO	W5PLP3	SMARCA5	3.70E-04	0.000
-2	CYTO	W5NTH2	EIF2B3	1.79E-03	0.000
-2	CYTO	W5PNT2	EXOSC7	7.20E-03	0.000
-2	CYTO	W5Q804	SPECC1	9.30E-04	0.000
-2	CYTO	W5NV74	CARS1	1.63E-02	0.000
-2	CYTO	W5QBJ2	SLC38A10	1.42E-04	0.000
-2	CYTO	W5PF71	KPNB1	3.42E-04	0.000
-2	CYTO	W5QCU3	CSTF2T	4.47E-02	0.000
-2	CYTO	W5QA09		2.31E-07	0.000
-2	CYTO	W5QG87	SNX4	1.95E-02	0.000
-2	CYTO	W5Q3P7	ITFG1	3.92E-04	0.000
-2	CYTO	W5PG10	PAPSS1	2.48E-05	0.000
-2	CYTO	W5QH60	VAMP8	7.73E-03	0.000
-2	CYTO	Q29422		4.61E-02	0.000
-2	CYTO	W5PJR8	UBE2L6	4.37E-02	0.000
-2	CYTO	W5NRL0	PLCD1	6.92E-03	0.000
-2	CYTO	W5NU48	CRTC2	1.19E-03	0.002
-2	CYTO	W5PD74	UFSP2	7.54E-04	0.000
-2	CYTO	W5QHD4	EIF4G1	2.85E-05	0.000
-2	CYTO	W5PQ47	RAI14	2.18E-02	0.000
-2	CYTO	W5PT24	MRPL53	3.96E-03	0.000
-2	CYTO	W5PSW9	RPL8	8.33E-06	0.000
-2	CYTO	W5PIX4	RRP12	1.73E-02	0.000
-2	CYTO	W5NVU7	PNPT1	2.15E-03	0.000
-2	CYTO	W5PB34	ELP1	2.64E-03	0.000
-2	CYTO	W5QDG9	CCDC80	2.28E-03	0.000
-2	CYTO	W5NTQ4	GATAD2B	1.04E-03	0.000
-2	CYTO	W5PD56	HUWE1	2.22E-02	0.000
-2	CYTO	W5QDK7	XRCC5	3.43E-03	0.000
-2	CYTO	W5NYT2	PBXIP1	1.10E-02	0.000
-2	CYTO	W5QCW3	RABGGTA	1.67E-03	0.000
-2	CYTO	W5Q6B0	DNTTIP2	1.11E-02	0.000
-2	CYTO	W5P0D4	EIF3J EIF3S1	2.71E-03	0.000
-2	CYTO	W5Q1I6	IMPDH2 IMPDH	1.37E-05	0.000
-2	CYTO	W5P1R7	NUB1	9.26E-03	0.000
-2	CYTO	W5P4L4	TMX3	1.13E-05	0.000
-2	CYTO	W5NWT7	GNL3	1.83E-03	0.000
-2	CYTO	W5P945	ATP1B3	5.04E-02	0.000
-2	CYTO	W5PUM8	HIP1	9.06E-04	0.000
-2	CYTO	W5PIE9	NIT1	6.90E-04	0.000

Supplementary Table 20: Differentially abundant proteins (DAPs) of M1 relative to M2 and their cellular location.

FoldChange	Location	Protein IDs	Gene Names	P-value	FDR
-2	CYTO	W5QGF4	VPS18	3.26E-02	0.000
-2	CYTO	W5PH26		1.13E-03	0.000
-2	CYTO	W5QCZ6	LOC101115461	4.47E-04	0.000
-2	CYTO	W5PEL8	PDXDC1	3.39E-02	0.000
-2	CYTO	W5NPR6	AATF	1.11E-04	0.000
-2	CYTO	W5NPP2	CPM	2.35E-02	0.000
-2	CYTO	W5P7E8		1.20E-03	0.000
-2	CYTO	W5Q5Q7	ASPA	3.09E-02	0.000
-2	CYTO	W5PHX8	GLRX2	7.76E-04	0.000
-2	CYTO	W5PF73	PLAU	7.64E-03	0.000
-2	CYTO	W5PPF1	AGPAT3	4.27E-03	0.000
-2	CYTO	W5P5P8	PSMG3	8.87E-04	0.000
-2	CYTO	W5Q9T2	RANGAP1	9.89E-04	0.000
-2	CYTO	W5NSK3		8.41E-03	0.000
-2	CYTO	W5Q018	PKN2	2.46E-03	0.000
-2	CYTO	W5P9P2	MYO6	1.10E-03	0.000
-2	CYTO	W5Q4K4	EVI5	6.96E-03	0.000
-2	CYTO	W5PVH8	ATP6V1E1	5.18E-05	0.000
-2	CYTO	W5NSI2	RALBP1	3.13E-03	0.009
-2	CYTO	W5Q969	PWP1	4.70E-05	0.000
-2	CYTO	W5NV37	COMMD10	5.33E-03	0.000
-2	CYTO	W5Q1C4	DNMT1	4.58E-02	0.001
-2	CYTO	W5NSS6		4.29E-03	0.000
-2	CYTO	W5QAJ6	GRAP	4.49E-03	0.000
-2	CYTO	W5P180		5.36E-03	0.000
-2	CYTO	W5PZ99	KIDINS220	2.84E-02	0.000
-2	CYTO	W5Q552	ANAPC7	1.51E-02	0.000
-2	CYTO	W5QCM1	ARFGAP3	1.41E-03	0.000
-2	CYTO	W5Q675	RPS2	4.65E-05	0.000
-2	CYTO	W5PCT8	ASAP1	7.31E-05	0.000
-2	CYTO	W5PY12	LOC101119765	3.12E-04	0.003
-2	CYTO	W5P2J8		1.70E-03	0.000
-2	CYTO	W5Q9J0	RNPS1	4.79E-05	0.000
-2	CYTO	W5PNV5	GCN1	4.42E-04	0.000
-2	CYTO	W5NT67	TUBGCP2	7.43E-03	0.000
-2	CYTO	W5PI99	HDGFL2	6.17E-03	0.000
-2	CYTO	W5PZD7		3.16E-02	0.000
-2	CYTO	W5PHB4	STAT2	1.49E-02	0.000
-2	CYTO	W5QHT6	NCEH1	2.05E-05	0.000
-2	CYTO	W5PHT7	FKBP3	2.38E-04	0.000
-2	CYTO	W5P5S5	EMC3	1.22E-03	0.000
-2	CYTO	W5QJ37		1.65E-03	0.000
-2	CYTO	W5NRI6	PLPBP PROSC	8.46E-05	0.000
-2	CYTO	W5Q9Z8	PHAX	2.89E-04	0.000
-2	CYTO	W5Q6R6	SHPK	3.02E-05	0.000
-2	CYTO	W5PQR5	RPL18	4.28E-02	0.000

Supplementary Table 20: Differentially abundant proteins (DAPs) of M1 relative to M2 and their cellular location.

FoldChange	Location	Protein IDs	Gene Names	P-value	FDR
-2	CYTO	W5P7R2	HECTD1	1.98E-02	0.000
-2	CYTO	W5QI95	CERS2	6.71E-04	0.000
-2	CYTO	W5P0S9	GDPD3	1.94E-03	0.000
-2	CYTO	W5Q600	TSC22D4	2.01E-02	0.000
-2	CYTO	W5PQ88	RPS6KB2	6.42E-04	0.000
-2	CYTO	W5PA78	AVL9	8.24E-04	0.000
-2	CYTO	W5QCS0	RBCK1	1.11E-02	0.000
-2	CYTO	W5Q3B4	TBCD	9.14E-05	0.000
-2	CYTO	W5PBM2	LOC101110634	6.18E-04	0.000
-2	CYTO	W5Q1Z2		3.24E-02	0.001
-2	CYTO	W5QHZ7	RPS27L	4.67E-05	0.000
-2	CYTO	W5P6P2	LOC101110647	6.54E-05	0.000
-2	CYTO	W5NTJ6	RPL28	3.34E-03	0.000
-2	CYTO	W5PWT9	DOP1B	2.69E-04	0.000
-2	CYTO	W5PSQ0	DOHH	4.30E-04	0.000
-2	CYTO	W5PVA4	ABCF1	8.95E-03	0.000
-2	CYTO	W5Q7M1	RRP8	7.31E-05	0.000
-2	CYTO	W5PC02	CHD4	1.22E-04	0.000
-2	CYTO	W5NR48	KPNA6	4.00E-05	0.000
-2	CYTO	W5PHG6	SMYD2	2.91E-04	0.000
-2	CYTO	W5NZW5	WDFY3	2.99E-03	0.000
-2	CYTO	W5PYH6	AP3D1	5.24E-04	0.000
-3	CYTO	W5QDT8		1.66E-02	0.000
-3	CYTO	W5QCI3	LOC101106419	5.12E-04	0.000
-3	CYTO	W5NWP6	DIAPH2	5.56E-03	0.000
-3	CYTO	W5NTW0	CADM1	7.95E-03	0.000
-3	CYTO	W5Q9L2	LOC101109820	1.06E-02	0.000
-3	CYTO	W5PSW3	BAZ1B	5.38E-04	0.000
-3	CYTO	W5PM03	RFC3	1.24E-05	0.000
-3	CYTO	W5NZC5	DNAJA3	2.39E-03	0.000
-3	CYTO	W5Q0J1	PLA2G6	5.37E-04	0.005
-3	CYTO	W5PGD6	BZW2	6.85E-07	0.000
-3	CYTO	W5QB99	TSC22D3	3.59E-04	0.000
-3	CYTO	W5QDR4	ATXN10	3.99E-04	0.000
-3	CYTO	W5PTU7	CA2	3.16E-04	0.000
-3	CYTO	W5QFY4	COMMD5	6.62E-05	0.000
-3	CYTO	W5PMJ1	PTGES2	5.51E-04	0.000
-3	CYTO	W5QI06	PRPF3	8.97E-03	0.000
-3	CYTO	W5Q0E7	LIN7C	1.68E-03	0.006
-3	CYTO	W5NRR3	PARP1	7.43E-04	0.000
-3	CYTO	W5QDB8		1.32E-02	0.000
-3	CYTO	W5Q3X2		1.48E-05	0.000
-3	CYTO	W5NXM4	RPS9	3.87E-05	0.000
-3	CYTO	W5P272	CKAP5	1.07E-04	0.000
-3	CYTO	W5Q2N7	METAP1	1.15E-03	0.000
-3	CYTO	W5P468	UTP15	3.96E-04	0.000

Supplementary Table 20: Differentially abundant proteins (DAPs) of M1 relative to M2 and their cellular location.

FoldChange	Location	Protein IDs	Gene Names	P-value	FDR
-3	CYTO	B9VGZ7	SLIRP	4.14E-03	0.000
-3	CYTO	W5PRM6	PDS5A	1.26E-02	0.000
-3	CYTO	W5PSP1	ERC1	3.83E-03	0.000
-3	CYTO	W5NY92	DOCK4	2.82E-02	0.000
-3	CYTO	W5NXN8	MTOR	1.43E-03	0.000
-3	CYTO	W5NPI1	BCLAF1	1.77E-06	0.000
-3	CYTO	W5PQ74	GRWD1	2.33E-04	0.000
-3	CYTO	W5PJH6		1.04E-03	0.000
-3	CYTO	W5PNW2	BLOC1S1	4.62E-04	0.000
-3	CYTO	W5PFR8	FBP1	6.89E-03	0.000
-3	CYTO	W5PWK3	TBRG4	2.62E-04	0.001
-3	CYTO	W5PUD6	NUP58	7.27E-04	0.000
-3	CYTO	W5NX86	EIF1B	4.33E-03	0.000
-3	CYTO	W5PVZ7	MRPL57	4.37E-03	0.000
-3	CYTO	W5P3W2	PPP2R5D	6.23E-05	0.000
-3	CYTO	W5NSF7		3.10E-03	0.000
-3	CYTO	W5NTT1	ARHGAP4	1.76E-04	0.000
-3	CYTO	W5PJY6	ADAM28	2.15E-03	0.000
-3	CYTO	W5Q2H3	ELAC2	1.48E-04	0.000
-3	CYTO	W5QIF1	MYO5A	5.93E-04	0.000
-3	CYTO	W5NYQ9	TIMM44	5.13E-04	0.000
-3	CYTO	W5PVH4	TMEM251	2.08E-04	0.000
-3	CYTO	W5P0C5	LGALS8	4.76E-04	0.000
-3	CYTO	W5P1Z2	SRM	2.70E-02	0.000
-3	CYTO	W5QA65	ZW10	7.72E-05	0.000
-3	CYTO	W5P3J0	RPL6	1.66E-06	0.000
-3	CYTO	W5PE91	LOC101105400	9.20E-03	0.000
-3	CYTO	W5QCC0	RALGAPB	9.00E-04	0.000
-3	CYTO	W5PG93	HP1BP3	6.51E-04	0.000
-3	CYTO	W5P026	STAB1	4.38E-02	0.000
-3	CYTO	W5PU00	MRPS9	1.05E-04	0.000
-3	CYTO	W5Q8T1	CLPX	1.38E-04	0.000
-3	CYTO	W5Q8V2	LIMA1	1.77E-02	0.000
-3	CYTO	W5PSG9	DTNBP1	1.10E-05	0.000
-3	CYTO	W5PFQ5	ANKMY2	3.12E-04	0.000
-3	CYTO	W5NVT8	NOP16	3.39E-02	0.000
-3	CYTO	W5NXZ9	RASSF4	2.76E-04	0.000
-3	CYTO	W5Q5E7	PYCR3	1.31E-02	0.000
-3	CYTO	W5Q5C7	CTNNA1	3.92E-03	0.000
-3	CYTO	W5P5T6	PPP1R14B	3.15E-04	0.000
-3	CYTO	W5NTI0	TOP1	8.31E-04	0.000
-3	CYTO	W5Q3E9	REEP5	2.72E-02	0.000
-3	CYTO	W5P1Z5	PMPCA	2.50E-04	0.000
-3	CYTO	W5Q655		4.31E-02	0.000
-3	CYTO	W5QAK7	SCARB1	4.13E-04	0.000
-3	CYTO	W5QJ24	PPP2R5E	2.73E-03	0.000

Supplementary Table 20: Differentially abundant proteins (DAPs) of M1 relative to M2 and their cellular location.

FoldChange	Location	Protein IDs	Gene Names	P-value	FDR
-3	CYTO	W5Q540	HAGH	3.82E-04	0.000
-3	CYTO	W5PQP3	LIMK1	3.30E-05	0.000
-3	CYTO	W5Q2J4	DIP2C	6.59E-03	0.000
-3	CYTO	W5Q992	MYO9B	3.75E-03	0.000
-3	CYTO	W5PYE8	MERTK	3.14E-03	0.000
-3	CYTO	W5PKT0	NAA25	1.70E-05	0.002
-3	CYTO	W5PV01	REPS2	8.36E-03	0.000
-3	CYTO	W5PZ22	LTN1	2.13E-02	0.000
-3	CYTO	W5QEG2	MAP7D1	1.45E-04	0.000
-3	CYTO	W5NPW8	STARD4	2.19E-05	0.000
-3	CYTO	W5PSV2	TRIP11	3.61E-03	0.000
-3	CYTO	W5PN94	LUC7L	3.28E-04	0.000
-3	CYTO	W5Q6D6	AKAP9	2.56E-06	0.000
-3	CYTO	W5P078		1.09E-02	0.000
-3	CYTO	W5Q4L7	AP3S1	7.49E-04	0.000
-3	CYTO	W5PF54	CDK3	4.88E-02	0.000
-3	CYTO	W5PXR1	ENPP1	3.26E-02	0.000
-3	CYTO	W5P173	BMS1	4.64E-04	0.000
-3	CYTO	W5Q6X2	TNFRSF1B	4.09E-06	0.000
-3	CYTO	W5QH69	RNF181	9.96E-03	0.000
-3	CYTO	W5Q8J0	PIN4	1.24E-03	0.000
-3	CYTO	W5Q3D8	CLASP2	7.81E-06	0.000
-3	CYTO	W5PV90	EIF5B	7.94E-05	0.000
-3	CYTO	W5NYI4	RIPOR1	2.63E-05	0.000
-3	CYTO	W5PUM5	KANK1	4.19E-06	0.000
-3	CYTO	W5P8E1	DNPH1 RCL	1.05E-03	0.000
-3	CYTO	W5PFD2	HOOK1	8.58E-03	0.009
-3	CYTO	W5PU77	PIP5K1B	8.40E-03	0.004
-4	CYTO	O78750	COX2	1.50E-05	0.000
-4	CYTO	W5P0V0	LOC114110488	1.73E-03	0.000
-4	CYTO	W5PE67		1.05E-03	0.000
-4	CYTO	W5QA91	NAT10	2.73E-05	0.000
-4	CYTO	W5Q9Z9	RPL4	2.51E-05	0.000
-4	CYTO	W5PS76	UNC45A	7.57E-06	0.000
-4	CYTO	W5PVG5	BLTP3B	1.78E-04	0.000
-4	CYTO	W5Q6F0	LOC101106791	4.32E-03	0.000
-4	CYTO	W5PXX3	F13B	4.98E-02	0.007
-4	CYTO	W5Q8Q2	XPNPEP3	1.96E-05	0.000
-4	CYTO	W5PDJ0		1.03E-03	0.000
-4	CYTO	W5NYU6	NT5DC2	1.35E-04	0.000
-4	CYTO	W5NYW8	CEP55	1.89E-04	0.000
-4	CYTO	W5Q568	TRANK1	3.20E-07	0.006
-4	CYTO	W5PES6		3.04E-02	0.000
-4	CYTO	W5PHB8	GALT	3.01E-04	0.000
-4	CYTO	W5PD43	HTRA1	2.10E-02	0.000
-4	CYTO	W5PZ28	TEX2	4.05E-02	0.002

Supplementary Table 20: Differentially abundant proteins (DAPs) of M1 relative to M2 and their cellular location.

FoldChange	Location	Protein IDs	Gene Names	P-value	FDR
-4	CYTO	W5PYI8	WWC1	2.13E-03	0.000
-4	CYTO	W5PV04	NIFK	7.39E-04	0.000
-4	CYTO	W5P895	CRABP2	9.37E-04	0.000
-5	CYTO	W5PPX2	SENP8	3.47E-03	0.003
-5	CYTO	W5NY85	MSRB3	3.92E-04	0.000
-5	CYTO	W5Q458		3.47E-03	0.003
-5	CYTO	W5PZ31	NFS1	1.65E-02	0.000
-5	CYTO	W5P1J4	NCAPG	7.76E-03	0.000
-5	CYTO	W5QHC0	ST3GAL5	5.16E-06	0.000
-6	CYTO	W5NPM4	TEX15	6.70E-04	0.003
-6	CYTO	W5QG29	DES	5.18E-03	0.002
-6	CYTO	W5PYD8	PARP4	5.36E-04	0.009
8	SEC	W5PYW3	LOC101103238	4.49E-04	0.000
6	SEC	W5PAC2	LOC101105044	1.12E-02	0.000
6	SEC	W5Q0L2	SERPINA5	1.94E-02	0.000
5	SEC	W5NZA2	FASTKD1	3.14E-02	0.008
5	SEC	W5P4V3	MMP3	1.75E-02	0.000
5	SEC	W5QJA1		2.99E-02	0.000
4	SEC	W5PHJ9	LOC101119572	4.61E-02	0.000
4	SEC	W5P530	LOC101104705	2.06E-02	0.000
3	SEC	W5P2M5	LOC101114535	5.96E-04	0.000
3	SEC	W5PJR5	LOC101121216	5.37E-03	0.000
3	SEC	W5PSL9	HPD	5.80E-03	0.004
3	SEC	W5QB02	TGM2	5.27E-02	0.002
3	SEC	W5PEB0	FABP7	2.79E-02	0.000
3	SEC	W5PNX6	PREP	1.54E-02	0.000
2	SEC	W5PDR7	C8A	2.12E-02	0.002
2	SEC	W5QG01	DNPEP	7.85E-03	0.000
2	SEC	W5P5K5	MMP1	4.71E-02	0.000
2	SEC	W5NUJ7	LOC101123419	2.98E-02	0.000
2	SEC	W5PLT8		8.33E-03	0.004
-2	SEC	W5P5K9	SRGN	3.15E-03	0.000
-2	SEC	W5P8Z3	PSMC4	1.43E-02	0.000
-2	SEC	W5Q2I7	USO1	1.69E-02	0.000
-2	SEC	W5Q1B3	HMGB2	3.24E-02	0.000
-2	SEC	W5Q3C2	EPPK1	3.67E-02	0.000
-2	SEC	W5NY01		5.27E-03	0.000
-2	SEC	W5PGF4	PLAUR	1.31E-03	0.000
-2	SEC	W5PHP7		8.38E-03	0.004
-2	SEC	W5NVC9	RAC1	3.89E-02	0.000
-2	SEC	W5PDD2	TLL2	2.35E-02	0.007
-2	SEC	W5PJ13		5.33E-03	0.000
-2	SEC	W5PQQ6	NECAP2	7.13E-03	0.005
-2	SEC	W5NRD9		1.40E-02	0.002
-2	SEC	W5PJN6	KYNU	2.47E-02	0.006
-2	SEC	W5NX31	AHSA1	4.32E-02	0.000

Supplementary Table 20: Differentially abundant proteins (DAPs) of M1 relative to M2 and their cellular location.

FoldChange	Location	Protein IDs	Gene Names	P-value	FDR
-2	SEC	W5Q0B6	PPP1R12A	2.71E-02	0.002
-2	SEC	W5PFM8	ADK	4.61E-02	0.000
-2	SEC	W5QBH1		4.37E-02	0.000
-2	SEC	W5PSG0		1.93E-02	0.005
-2	SEC	W5P6M2	PSMD5	2.78E-02	0.000
-2	SEC	W5P5W9	TPI1	1.98E-02	0.000
-2	SEC	W5PIM8	GALK1	1.16E-02	0.000
-2	SEC	W5NSP2	RPS8	3.17E-02	0.000
-2	SEC	W5QI29	ECM1	1.88E-02	0.000
-2	SEC	W5PXC6		6.33E-04	0.000
-2	SEC	W5PFV5	NPEPPS	9.23E-04	0.000
-2	SEC	W5PTR5	HSPA5	8.04E-03	0.000
-2	SEC	W5PS94	NUCB1	2.01E-07	0.000
-2	SEC	W5PTW4	PITPNA	2.86E-02	0.000
-2	SEC	W5P2W1	NEU1	1.72E-03	0.008
-2	SEC	W5PPG3	ALDH9A1	2.22E-02	0.000
-2	SEC	W5PVG1	CAPN1	3.10E-02	0.000
-2	SEC	W5P5B6	VSIG4	6.66E-03	0.000
-2	SEC	W5PF12	HSD17B10	9.49E-03	0.000
-2	SEC	A7UHZ2	PSMD4	1.97E-02	0.000
-2	SEC	W5QCX9	TTLL12	1.44E-02	0.000
-2	SEC	W5Q8J3	RRBP1	3.90E-02	0.000
-2	SEC	A2SW69	ANXA2 ANX2	2.88E-02	0.000
-2	SEC	W5NZR9	PSMC1	1.99E-03	0.000
-2	SEC	W5P8I3	RIDA	5.07E-02	0.000
-2	SEC	W5P5A0	FLNA	2.13E-02	0.000
-2	SEC	W5P2D9	SNX9	1.28E-02	0.000
-2	SEC	W5PVJ7	GON7	1.94E-02	0.004
-2	SEC	W5PUD2	RPS21	4.19E-02	0.000
-2	SEC	W5QDF4	GSTM3	5.32E-02	0.000
-2	SEC	W5NSM1	ACTR1A	1.85E-03	0.000
-2	SEC	W5NZ70	LGALS3BP	3.61E-02	0.000
-2	SEC	W5NSD9	REXO2	1.87E-02	0.009
-2	SEC	W5NVD7	COPS4	3.70E-02	0.008
-2	SEC	W5QFL1	LOC101116286	1.79E-03	0.000
-2	SEC	W5PPH6	RPS3	2.61E-02	0.000
-2	SEC	W5PDU8	GARS1	4.74E-02	0.000
-2	SEC	W5QDM2	PSMB5	3.58E-02	0.000
-2	SEC	W5NYV5	SPAG9	4.45E-04	0.002
-2	SEC	W5Q3N5	PRKAR2A	1.23E-02	0.000
-2	SEC	W5P9K6	PSMC3	5.11E-03	0.000
-2	SEC	W5PRH3	EIF6 ITGB4BP	4.74E-02	0.000
-2	SEC	Q1ZZU7	MIF	3.73E-02	0.000
-2	SEC	W5P4C7	SEPTIN7	3.59E-02	0.000
-2	SEC	W5PJ95	PPP2R2A	1.17E-02	0.000
-2	SEC	W5PJY3	RPL23	5.33E-02	0.000

Supplementary Table 20: Differentially abundant proteins (DAPs) of M1 relative to M2 and their cellular location.

FoldChange	Location	Protein IDs	Gene Names	P-value	FDR
-2	SEC	W5PR48	HPRT1	4.74E-02	0.000
-2	SEC	W5PVT6	UBA1	5.31E-02	0.000
-2	SEC	W5PVX8	PPP3CA	1.92E-02	0.000
-2	SEC	W5NWJ9		1.26E-02	0.000
-2	SEC	W5PMM7	PDIA3	4.31E-02	0.000
-2	SEC	W5Q9T3	LMNB1	2.06E-02	0.000
-2	SEC	W5PJ98		1.23E-02	0.000
-2	SEC	W5QIU9	S100A10	4.98E-03	0.000
-2	SEC	W5PFD0	CORO6	5.41E-02	0.009
-3	SEC	W5QHD8	PSMD2	3.07E-02	0.000
-3	SEC	W5QC35	PDIA4	1.15E-02	0.000
-3	SEC	W5Q3A5	PAFAH1B1 LIS1	5.31E-02	0.000
-3	SEC	W5PMP1	COTL1	2.10E-02	0.000
-3	SEC	W5PIG7	ENO1	1.01E-02	0.000
-3	SEC	W5P659	HK1	2.43E-02	0.000
-3	SEC	W5PAQ4	FUCA1	2.42E-02	0.000
-3	SEC	P50413	TXN	2.43E-02	0.000
-3	SEC	W5PMA0	AP2S1	4.25E-02	0.002
-3	SEC	W5QB13	NSFL1C	1.52E-02	0.000
-3	SEC	W5PZM9	ANXA5	2.86E-02	0.000
-3	SEC	W5NZK7	PLA2G15	2.46E-02	0.000
-3	SEC	W5P7P6	MYO18A	3.93E-02	0.000
-3	SEC	W5PLV2	ALDH2	3.07E-02	0.000
-3	SEC	W5P2K9	LMAN2	1.92E-03	0.000
-3	SEC	W5QH35	CAPG	1.91E-02	0.000
-3	SEC	W5P363	VAT1	9.83E-03	0.000
-3	SEC	W5P4R1	MSN	2.23E-02	0.000
-3	SEC	W5PK66	PARK7	2.17E-02	0.000
-3	SEC	W5QF90		2.66E-02	0.000
-3	SEC	W5QC41	PKM	4.60E-03	0.000
-3	SEC	W5Q563	GFUS	1.02E-02	0.003
-3	SEC	C5ISA2	TUBA4A	5.99E-03	0.000
-3	SEC	W5PI22	CNPY2	3.91E-02	0.000
-3	SEC	W5QIL2	PSMB4	1.70E-02	0.000
-3	SEC	B0FZL9	SRSF3	3.60E-02	0.000
-3	SEC	W5P223	OTUB1	4.70E-02	0.000
-3	SEC	W5PKV1	DNASE2	2.99E-02	0.000
-3	SEC	Q6QAT4	B2M	8.34E-04	0.000
-3	SEC	W5Q5J5	LOC101110855	3.03E-03	0.000
-3	SEC	W5NYH2	LOC101102072	4.09E-03	0.000
-3	SEC	W5NVY0		5.00E-03	0.000
-3	SEC	W5Q4R2	APBB1IP	3.02E-02	0.004
-3	SEC	W5QF71	PLEK	3.62E-04	0.000
-3	SEC	P83205	CTSB	6.82E-04	0.000
-3	SEC	W5Q701	LOC101112491	4.36E-02	0.000
-3	SEC	W5PQA6	CYFIP2	1.46E-02	0.000

Supplementary Table 20: Differentially abundant proteins (DAPs) of M1 relative to M2 and their cellular location.

FoldChange	Location	Protein IDs	Gene Names	P-value	FDR
-3	SEC	W5PX22	SNRPD3	4.51E-02	0.000
-3	SEC	W5P508	EIF3B EIF3S9	1.05E-02	0.000
-3	SEC	W5PF04	MAN1A1	4.66E-05	0.000
-3	SEC	W5P985	ABHD14B	1.82E-02	0.000
-3	SEC	W5PJ66	CFD	1.19E-02	0.000
-3	SEC	W5NZP8	WASF2	3.27E-02	0.000
-3	SEC	W5P3V8		9.92E-03	0.000
-3	SEC	W5PLE9	ERP29	5.45E-03	0.000
-3	SEC	Q9XT28	ATOX1	1.69E-02	0.000
-3	SEC	W5Q0N0	LOC101119757	7.66E-03	0.000
-3	SEC	W5PUH7		1.57E-02	0.000
-3	SEC	W5PMY0	PLA2G7	2.10E-02	0.006
-3	SEC	W5Q1T4		1.55E-02	0.000
-3	SEC	W5NPN4	HSPA8	1.35E-02	0.000
-3	SEC	W5P5M7	VPS26A	2.73E-02	0.000
-3	SEC	W5PE73	SMPDL3A	9.58E-03	0.000
-3	SEC	W5PK04	PGAM1	4.63E-02	0.000
-3	SEC	W5PJC2	PSMB3	2.76E-02	0.000
-3	SEC	W5P1G7		4.61E-02	0.000
-3	SEC	C5IWV1	FH	1.99E-02	0.000
-3	SEC	W5P8R3	HDGF	2.03E-02	0.000
-3	SEC	W5NUZ1	DYNC1I2	3.86E-02	0.000
-3	SEC	W5NZ57	PSMB10	4.05E-02	0.000
-3	SEC	W5PQR0	NIBAN2	3.71E-02	0.000
-3	SEC	W5PE27	ESD	1.28E-03	0.000
-3	SEC	W5PRU5	MANBA	2.84E-04	0.000
-3	SEC	W5NY22	PCMT1	4.36E-02	0.000
-3	SEC	W5QD49	PSME1	5.24E-02	0.000
-3	SEC	W5NZQ2	LAP3	3.30E-02	0.000
-3	SEC	W5PGG5	CD84	5.44E-03	0.001
-3	SEC	W5P1H0	CTSC	3.09E-05	0.000
-3	SEC	W5NS93	PSMA4	4.67E-03	0.000
-3	SEC	W5PS88	GOT1	1.28E-02	0.000
-3	SEC	W5PDQ9		1.98E-02	0.000
-3	SEC	W5PQK7	EIF5A	8.77E-03	0.000
-3	SEC	W5PL19	LOC101105123	3.29E-02	0.000
-3	SEC	W5PDN2		3.38E-02	0.000
-3	SEC	W5P369	AP2A2	1.74E-02	0.000
-3	SEC	W5P824	LOC101103096	2.75E-02	0.000
-3	SEC	W5P8Q0	AP2B1	2.87E-02	0.000
-3	SEC	W5PG09	PGK2	1.98E-02	0.000
-3	SEC	W5NXX1	IPO5	6.04E-03	0.000
-3	SEC	W5PEE9	LAMP1	1.72E-02	0.006
-3	SEC	W5QCL8	NPL	1.76E-02	0.000
-3	SEC	W5QJA2	CD14	2.26E-04	0.000
-3	SEC	W5QCP7	PACSIN2	1.25E-02	0.000

Supplementary Table 20: Differentially abundant proteins (DAPs) of M1 relative to M2 and their cellular location.

FoldChange	Location	Protein IDs	Gene Names	P-value	FDR
-3	SEC	W5PWF2	ATP6V1H	2.14E-02	0.000
-3	SEC	W5PE11	CDC42	3.61E-03	0.000
-3	SEC	W5PNE1		2.60E-02	0.007
-3	SEC	W5QJ99	TNFRSF1A	2.60E-03	0.000
-3	SEC	W5PB07	PSMA8	5.54E-02	0.000
-3	SEC	W5P726	PRCP	9.30E-03	0.000
-3	SEC	W5PVL6	AP2A1	1.59E-03	0.000
-3	SEC	W5P6F9	DPP3	5.38E-02	0.000
-3	SEC	W5PL67	SUCLG2	4.66E-03	0.000
-3	SEC	W5NPL9		4.91E-02	0.000
-3	SEC	W5Q8S5	ERAP1	1.96E-02	0.000
-3	SEC	W5NVT3	SND1	2.89E-02	0.000
-3	SEC	W5PI38	CS	1.49E-02	0.000
-3	SEC	W5QFG8	ACTR2	4.25E-02	0.000
-3	SEC	W5NW80	GAA	1.03E-04	0.002
-3	SEC	W5QAZ0	PRPSAP2	1.62E-02	0.006
-3	SEC	W5PSZ5	ANXA1	2.90E-02	0.000
-3	SEC	W5Q3N1	CTSZ	1.43E-04	0.000
-3	SEC	W5P8R4	CSF1R	8.05E-05	0.000
-3	SEC	W5Q5R8	TXNRD1	4.56E-02	0.000
-3	SEC	W5PDC7	PSMB9	1.32E-02	0.000
-3	SEC	W5PK13	CBX3	2.33E-02	0.000
-3	SEC	W5QBV3	PGK1	1.23E-02	0.000
-3	SEC	W5P583	ACP1	2.36E-02	0.000
-3	SEC	W5PDD0	LCP1	2.77E-02	0.000
-3	SEC	W5PBY5		1.37E-02	0.000
-3	SEC	W5PEH2	HADH	2.65E-02	0.000
-3	SEC	W5QHU9	PPIB	2.64E-02	0.000
-3	SEC	W5PTA8	FURIN	1.02E-03	0.005
-3	SEC	W5PLB9	PSMD11	4.10E-02	0.000
-3	SEC	W5P1W2	FOLR3	2.16E-02	0.009
-3	SEC	W5PVU5	PSMB7	3.91E-02	0.000
-3	SEC	W5PHX1	AHCY	6.66E-03	0.000
-3	SEC	W5P814	KIF5A	7.12E-03	0.000
-3	SEC	W5QBE4	FGL2	3.67E-03	0.000
-3	SEC	W5NUG0	GOT2	1.62E-02	0.000
-3	SEC	W5QF93	BCL2L15	1.11E-02	0.000
-3	SEC	W5Q5R5	SAMHD1	7.16E-03	0.000
-3	SEC	W5PC32	PGM3	2.28E-04	0.004
-3	SEC	W5Q038	AHNAK	3.68E-02	0.000
-3	SEC	W5Q9P7	CFL1	4.12E-02	0.000
-3	SEC	W5PW97	PSMC5	3.21E-02	0.000
-3	SEC	W5NTD9	CHI3L1	6.06E-04	0.000
-3	SEC	W5PE22	GDI2	2.70E-03	0.000
-3	SEC	W5QFH5	RAB1A	4.83E-03	0.000
-3	SEC	W5PLS7	GRB2	1.63E-03	0.000

Supplementary Table 20: Differentially abundant proteins (DAPs) of M1 relative to M2 and their cellular location.

FoldChange	Location	Protein IDs	Gene Names	P-value	FDR
-3	SEC	W5Q5K9	ARPC3	5.18E-02	0.000
-3	SEC	W5QGL6	PAK2	2.89E-02	0.000
-3	SEC	W5PGC5	GALM	4.85E-02	0.000
-3	SEC	W5QJ62	ACTN1	8.30E-03	0.000
-3	SEC	W5P9G8	PLD3	1.42E-02	0.000
-3	SEC	W5P4X6	LOC101104287	3.08E-02	0.000
-3	SEC	W5PR81	TARS1	4.10E-02	0.000
-3	SEC	W5QBQ8	RAB5C	1.76E-05	0.000
-3	SEC	W5QG77	CD58	1.12E-03	0.000
-3	SEC	W5Q4E1	HSP90B1	2.60E-02	0.000
-3	SEC	W5P500	PSMB1	4.91E-03	0.000
-3	SEC	W5Q6E7	EIF2S3	1.70E-02	0.000
-4	SEC	W5PUJ4	SYNCRIP	3.32E-04	0.000
-4	SEC	W5QFQ0	MDH1	1.66E-02	0.000
-4	SEC	W5Q951	CORO1C	4.03E-02	0.000
-4	SEC	W5NYS1	SRSF6	9.91E-03	0.002
-4	SEC	C5IWU0	ARF1 LOC101123118	5.94E-04	0.000
-4	SEC	W5QFN9	UGP2	4.32E-02	0.000
-4	SEC	W5NZJ3	HNRNPH1	7.20E-03	0.000
-4	SEC	W5Q305	GANAB	4.38E-03	0.000
-4	SEC	W5P694	RPLP2	2.37E-02	0.000
-4	SEC	W5QGD1	LDHB	7.10E-03	0.000
-4	SEC	W5PJJ7		6.38E-03	0.000
-4	SEC	W5PXX7	CCT5	2.74E-02	0.000
-4	SEC	W5P6H2		1.03E-03	0.004
-4	SEC	W5PCL8	PSMB8	4.91E-02	0.000
-4	SEC	W5Q4L0		1.55E-02	0.000
-4	SEC	W5PE01	TAGLN2	1.97E-02	0.000
-4	SEC	W5QD41	PSME2	1.04E-02	0.000
-4	SEC	W5P2V0	EZR	1.02E-02	0.000
-4	SEC	W5P323	GPI	5.30E-03	0.000
-4	SEC	W5Q6T1	ARSB	3.68E-06	0.000
-4	SEC	W5NPQ6	CAPZA2	3.69E-03	0.000
-4	SEC	W5QAA9	ACO2	2.44E-03	0.000
-4	SEC	W5Q694		1.29E-02	0.000
-4	SEC	W5QIQ4	LOC101113241	1.11E-02	0.000
-4	SEC	W5P6C1	ADSS ADSS2	3.82E-02	0.000
-4	SEC	W5Q1R1	VPS35	3.14E-03	0.000
-4	SEC	W5PQ07	LOXL4	5.28E-02	0.000
-4	SEC	W5Q805	PSMC6	1.74E-03	0.000
-4	SEC	W5P3N0	MAPRE1	2.34E-02	0.000
-4	SEC	W5Q3D2		7.53E-03	0.005
-4	SEC	C5IJA0	RAN	8.09E-03	0.000
-4	SEC	W5PEM1		7.11E-03	0.000
-4	SEC	W5P3N6	LOC101112162	6.77E-05	0.000
-4	SEC	W5PSM0	OSTF1	4.56E-02	0.000

Supplementary Table 20: Differentially abundant proteins (DAPs) of M1 relative to M2 and their cellular location.

FoldChange	Location	Protein IDs	Gene Names	P-value	FDR
-4	SEC	W5QC89	HEXA	3.34E-02	0.000
-4	SEC	W5NRA9	ASL	5.83E-03	0.000
-4	SEC	W5P303	YWHAB	4.75E-02	0.000
-4	SEC	W5PQ98	ACTR3	9.61E-03	0.000
-4	SEC	W5PLD5	CNN2	1.83E-04	0.000
-4	SEC	W5PN69	LOC101109111	1.20E-02	0.000
-4	SEC	W5PGJ7	LOC101105208	2.28E-02	0.000
-4	SEC	W5PJB6	PGM1	2.49E-03	0.000
-4	SEC	W5Q7C7	PSMA2	2.54E-02	0.000
-4	SEC	W5PMH1	GSS	1.98E-02	0.000
-4	SEC	W5PK33	EFHD2	1.51E-02	0.000
-4	SEC	W5Q0L1	EEF1G	1.35E-03	0.000
-4	SEC	W5P9U4	PSMA1	2.02E-02	0.000
-4	SEC	W5P4C9	MMP12	3.72E-02	0.000
-4	SEC	W5Q5G8	TKT	6.29E-03	0.000
-4	SEC	W5QBL7	ARPC1B	2.64E-02	0.000
-4	SEC	W5NWF5	RARRES1	5.30E-04	0.000
-4	SEC	W5QJ49	ATP6V1D	1.12E-02	0.000
-4	SEC	W5NSD5	RAP1B	1.32E-04	0.000
-4	SEC	W5Q2F4	LOC101112223	2.84E-02	0.000
-4	SEC	W5Q7S8	NRP1	6.74E-03	0.000
-4	SEC	W5PC09	PSMA6	4.26E-02	0.000
-4	SEC	Q5MIB5	PYGL	4.67E-02	0.000
-4	SEC	W5Q0F3	TGFBI	8.88E-03	0.000
-4	SEC	W5QB71	AMDHD2	1.61E-02	0.000
-4	SEC	A8DR93	HSPCA HSP90AA1	2.21E-03	0.000
-4	SEC	W5QFZ3	CCT4	1.75E-02	0.000
-4	SEC	W5NUN8	AKR1A1	9.89E-03	0.000
-4	SEC	W5QD11	SORT1	1.81E-04	0.000
-4	SEC	W5Q282	PDIA6	2.42E-02	0.000
-4	SEC	W5P3I0	PICALM	2.35E-04	0.000
-4	SEC	W5QHQ7	NCL	1.66E-02	0.000
-4	SEC	W5PIH2	B4GALT1	9.15E-03	0.000
-4	SEC	W5PW05	MDH2	6.91E-04	0.000
-4	SEC	W5PVX4	DBNL	1.32E-03	0.000
-4	SEC	W5QIV1		1.42E-02	0.000
-4	SEC	W5PWZ2		3.61E-04	0.000
-4	SEC	W5PR73	CORO1B	1.36E-03	0.000
-4	SEC	W5P610	CLIC1	1.22E-04	0.000
-4	SEC	W5PD15	EEF1A1	9.09E-03	0.000
-4	SEC	W5QC34	MAN2A1	3.75E-05	0.000
-4	SEC	W5Q8I4	ST13	5.21E-03	0.000
-4	SEC	W5PFR8	FBP1	3.72E-02	0.000
-4	SEC	W5PV50	ADA2	5.94E-06	0.004
-4	SEC	W5Q799	GALNT6	2.97E-05	0.010
-4	SEC	W5PRI6	MRC1	2.85E-03	0.000

Supplementary Table 20: Differentially abundant proteins (DAPs) of M1 relative to M2 and their cellular location.

FoldChange	Location	Protein IDs	Gene Names	P-value	FDR
-4	SEC	W5P689	USP5	1.11E-02	0.000
-4	SEC	W5Q9H2		9.11E-03	0.000
-4	SEC	Q9MZA9	VIM	1.27E-02	0.000
-4	SEC	W5PUH5	LGMN	1.83E-04	0.000
-4	SEC	W5NZ62	GNS	4.77E-03	0.000
-4	SEC	W5P5W6	NDRG1	4.65E-04	0.000
-4	SEC	W5QDZ3	ARPC2	7.56E-03	0.000
-4	SEC	W5QDG8	FN1	2.13E-06	0.000
-4	SEC	W5PSA3	TCN1	2.74E-03	0.000
-4	SEC	W5PGS4	FABP5	2.01E-03	0.000
-4	SEC	W5PJG7	CALR	2.93E-02	0.000
-4	SEC	W5P7X3		6.34E-03	0.000
-4	SEC	W5PZ65	PSMA7	3.44E-03	0.000
-4	SEC	Q28554	GAPDH G3PDH GAPD	5.16E-04	0.000
-4	SEC	W5PPT6	TUBB	3.90E-04	0.000
-4	SEC	W5P285	G6PD	2.23E-02	0.000
-4	SEC	W5P4A8	RNASET2	7.68E-06	0.000
-4	SEC	Q6XUZ5	IDH1	1.18E-02	0.000
-4	SEC	W5QI36	HEBP1	2.20E-02	0.000
-4	SEC	W5P716		2.57E-03	0.000
-4	SEC	W5QFN2	HCLS1	7.72E-05	0.000
-4	SEC	W5QE19	PSMB2	1.41E-02	0.000
-4	SEC	W5PNP1	MFGE8	2.77E-04	0.000
-4	SEC	A9YUY8	FABP4	3.63E-02	0.000
-4	SEC	W5QGG6	CCT2	1.03E-02	0.000
-4	SEC	W5QFU8	CD86	1.33E-03	0.000
-5	SEC	W5QFB2	OLFML3	5.00E-04	0.000
-5	SEC	Q9MZS8	CTSD	5.86E-04	0.000
-5	SEC	W5NXE5	LOC101104501	2.18E-03	0.000
-5	SEC	W5QEU6	ANXA4	1.54E-03	0.000
-5	SEC	W5PTZ8	LOC101114959	2.43E-03	0.000
-5	SEC	W5PK95	HNRNPA2B1	1.09E-02	0.000
-5	SEC	W5PYQ7	RPS12	9.18E-03	0.000
-5	SEC	W5Q5V0	GBE1	6.01E-04	0.000
-5	SEC	W5QI35	IL1RN	3.53E-03	0.000
-5	SEC	W5P0W4	SEMA7A	3.07E-04	0.000
-5	SEC	W5PNW7	VIM	1.17E-03	0.000
-5	SEC	W5PUW2	IFI30	8.86E-03	0.000
-5	SEC	W5P887	CST3	1.47E-02	0.000
-5	SEC	C8BKE1	STAT1 STAT4	6.65E-05	0.000
-5	SEC	W5QCS4	SARS1	5.56E-04	0.000
-5	SEC	W5P707	ACTN4	1.00E-02	0.000
-5	SEC	W5PCC7	LOC101115509	3.33E-05	0.000
-5	SEC	W5NUI6	SGSH	3.43E-05	0.000
-5	SEC	W5NUG3	GNPDA1	2.75E-03	0.000
-5	SEC	W5QDG9	CCDC80	1.67E-03	0.000

Supplementary Table 20: Differentially abundant proteins (DAPs) of M1 relative to M2 and their cellular location.

FoldChange	Location	Protein IDs	Gene Names	P-value	FDR
-5	SEC	W5P7L2	ATP6V1G1	1.06E-03	0.000
-5	SEC	W5PB21	PLA2R1	3.43E-05	0.000
-5	SEC	W5NSH8	NPC2	1.17E-03	0.000
-5	SEC	W5PMB1	SNX3	4.50E-03	0.000
-5	SEC	W5NYA7	LOC101114319	1.35E-02	0.000
-5	SEC	W5Q349		8.74E-03	0.000
-5	SEC	W5PLZ0	ATP6V1B2	1.10E-02	0.000
-5	SEC	W5PD48	CYRIB	3.72E-03	0.000
-5	SEC	W5PVP5	APRT	2.10E-03	0.000
-5	SEC	W5PUJ5	LAMP2	6.31E-03	0.000
-5	SEC	W5P3C6	LOC101111906	1.82E-04	0.000
-5	SEC	P00349	PGD	1.69E-02	0.000
-5	SEC	G3M9U4	ACP5	1.92E-02	0.000
-5	SEC	W5PIC9		3.16E-05	0.000
-5	SEC	W5P375	TCP1	2.32E-03	0.000
-5	SEC	W5PU66	IQGAP1	3.70E-03	0.000
-5	SEC	W5PTU7	CA2	2.19E-02	0.000
-5	SEC	W5QBD7	YWHAZ	2.56E-03	0.000
-5	SEC	W5NTG6	TINAGL1	9.15E-05	0.000
-5	SEC	W5P041	ADAM15	3.83E-05	0.000
-5	SEC	W5NX56	SPP1	4.18E-03	0.000
-5	SEC	W5PCN2	ANXA7	9.22E-03	0.000
-5	SEC	W5NU86	GLA	1.40E-03	0.000
-5	SEC	W5PFY5	ASAH1	6.66E-06	0.000
-5	SEC	W5PKD3	GNAI2	3.31E-04	0.000
-5	SEC	W5QG24	PPT1	3.19E-06	0.000
-5	SEC	W5PWA8	HSPB1	2.68E-03	0.000
-5	SEC	W5PH85	ITGAX	1.13E-03	0.000
-5	SEC	W5P3I5	CNDP2	1.83E-02	0.000
-5	SEC	W5PBS4	LRP1	2.43E-04	0.000
-5	SEC	W5PCM7	LOC101105937	2.66E-04	0.004
-5	SEC	W5QIW1	LGALS3	2.05E-03	0.000
-5	SEC	W5PZB0	APLP2	3.05E-04	0.000
-5	SEC	W5P9L9		2.10E-03	0.000
-5	SEC	W5PCS7	CXCL16	9.12E-08	0.004
-5	SEC	W5PKU3		9.09E-06	0.000
-5	SEC	W5P409	FERMT3	7.87E-04	0.000
-5	SEC	W5QG16	CAP1	2.79E-03	0.000
-5	SEC	W5PXR1	ENPP1	2.62E-04	0.000
-5	SEC	W5PQK6	TLN1	8.76E-03	0.000
-5	SEC	W5NUV1	GNB1	3.90E-06	0.000
-5	SEC	W5QDY5	ATP6V1A	1.90E-02	0.000
-5	SEC	W5PIN6	LDHA	1.45E-02	0.000
-5	SEC	W5PGV0	ITGAM	9.55E-05	0.000
-5	SEC	W5NX91	RPS27A	7.31E-03	0.000
-5	SEC	W5PT76	GPNMB	2.17E-03	0.000

Supplementary Table 20: Differentially abundant proteins (DAPs) of M1 relative to M2 and their cellular location.

FoldChange	Location	Protein IDs	Gene Names	P-value	FDR
-5	SEC	W5PUT6	CLTC	1.12E-02	0.000
-5	SEC	W5Q3U3	LOC101102156	6.49E-05	0.000
-5	SEC	W5PQA8	PRDX6	1.13E-03	0.000
-5	SEC	W5QB61	FKBP1A	6.95E-04	0.000
-5	SEC	W5PTS4	LOC101114275	5.74E-03	0.000
-5	SEC	W5NTZ3	RENBP	9.51E-04	0.000
-5	SEC	W5Q0F1	LIPA	7.57E-03	0.000
-6	SEC	W5NPM4	TEX15	6.16E-05	0.004
-6	SEC	W5PG72	GOLM1	1.55E-04	0.000
-6	SEC	W5Q9H0	MMP2	3.41E-03	0.000
-6	SEC	W5Q1M0	GLB1	1.06E-06	0.000
-6	SEC	W5Q989	PLOD1	7.40E-04	0.000
-6	SEC	W5Q3T9	PDCD6IP	2.41E-04	0.000
-6	SEC	W5NZX1		3.78E-03	0.000
-6	SEC	W5NSK4		2.59E-05	0.000
-6	SEC	W5PE92	GRN	1.82E-02	0.000
-6	SEC	W5PF33	GM2A	5.67E-03	0.000
-6	SEC	W5NZ76	TIMP2	3.34E-09	0.000
-6	SEC	W5QGG0	TFRC	2.68E-04	0.000
-6	SEC	W5PS45	MAN2B1	2.73E-04	0.000
-6	SEC	W5NUI3	TREM2	1.44E-05	0.000
-6	SEC	W5Q689	TPP1	2.64E-05	0.000
-6	SEC	W5QBZ7	NAGA	4.45E-04	0.000
-6	SEC	W5NVW7	NAGLU	3.93E-05	0.000
-6	SEC	W5QAQ4	NRP2	3.22E-06	0.000
-6	SEC	W5NRI1		3.25E-05	0.000
-6	SEC	P50122	TIMP1	7.87E-06	0.000
-6	SEC	W5PGT0	MYH11	1.60E-06	0.002
-6	SEC	W5PF73	PLAU	3.70E-07	0.000
-6	SEC	W5PJ54	DPYSL2	6.48E-03	0.000
-6	SEC	W5P2V3	PEPD	9.35E-03	0.000
-6	SEC	W5Q1W2	SDCBP	2.98E-05	0.000
-6	SEC	W5PAM4	CTSA	4.89E-05	0.000
-6	SEC	W5PCE0	PLBD2	4.32E-05	0.000
-6	SEC	W5QI70	CTSS	2.13E-05	0.000
-6	SEC	W5PDE5	LOC101120001	6.59E-03	0.000
-6	SEC	W5NPP2	CPM	4.10E-05	0.000
-6	SEC	W5PN84	EEF2	4.85E-04	0.000
-6	SEC	W5PZ47	CTSH	7.65E-07	0.000
-6	SEC	W5PCH3	SCIN	3.07E-03	0.000
-6	SEC	W5PMR2	MMP19	8.34E-05	0.000
-6	SEC	W5Q2U7	PLEC	2.56E-03	0.000
-6	SEC	W5NPK5	LOC443475	2.20E-05	0.000
-7	SEC	W5QDI7	CSF1	2.74E-06	0.000
-7	SEC	Q6ECI6	ITGB2 CD18	2.78E-05	0.000
-7	SEC	W5PBM9	SCPEP1	1.44E-05	0.000

Supplementary Table 20: Differentially abundant proteins (DAPs) of M1 relative to M2 and their cellular location.

FoldChange	Location	Protein IDs	Gene Names	P-value	FDR
-7	SEC	W5NUI0		2.36E-03	0.000
-7	SEC	W5NT95	ATP6AP2	2.34E-06	0.000
-7	SEC	W5PHW0	HSP90AB1	5.06E-04	0.000
-7	SEC	W5PRG8	CREG1	1.11E-03	0.000
-7	SEC	W5PTL2	CFP	8.94E-05	0.000
-7	SEC	W5PAJ2	PSAP	6.67E-05	0.000
-7	SEC	W5P026	STAB1	7.13E-04	0.000
-7	SEC	W5PEY4	TCN2	1.24E-05	0.000
-7	SEC	W5NUZ2	UTRN	1.61E-06	0.002
-7	SEC	W5PJS4	EMILIN2	8.10E-06	0.000
-7	SEC	P81184	LGALS1	2.82E-06	0.000
-8	SEC	W5NTX3		4.05E-08	0.000
-8	SEC	W5QI78	CTSK	8.73E-07	0.000
-9	SEC	Q29524	LPL	8.65E-06	0.000

M1, pro-inflammatory macrophages activated with GM-CSF/ LPS/ INF-γ and M2, anti-inflammatory macrophages activated with M-CSF/ IL-4. Differentially abundant proteins (DAPs) were identified using a threshold of false discovery rate (FDR, q-value) ≤ 0.05 and absolute fold change ≥ 2. Red-highlighted cells indicate shared differentially abundant proteins (DAPs) between the cell lysate (CYTO) and secretome (SEC)compartments in M1 relative to M2, whereas non-highlighted cells represent compartment-specific DAPs unique to either the whole cell lysate or secretome.

Supplementary Table 21: List of 20 M1-specific and 57 M2-specific differentially abundant proteins (DAPs) that uniquely distinguish M1 and M2 macrophages, respectively, from M0 (monocytes at 3 hours), GMØ (macrophages differentiated with GM-CSF), MMØ (macrophages differentiated with M-CSF), and the opposing polarized subtype. M1, pro-inflammatory macrophages activated with GM-CSF/ LPS/ INF-γ; M2, anti-inflammatory macrophages activated with M-CSF/ IL-4. All proteins listed were identified as significantly differentially abundant with a false discovery rate (FDR, q-value) < 0.001.

Protein ID	Entry Name	Protein names	Gene Names	Marker
W5NTZ2	W5NTZ2_SHEEP	Pyrin	MEFV	**M1**
W5NXJ0	W5NXJ0_SHEEP	C-type lectin domain-containing protein	CLEC4E	**M1**
W5PJV4	W5PJV4_SHEEP	C-type lectin domain-containing protein	ASGR2	**M1**
W5PMT0	W5PMT0_SHEEP	Xanthine dehydrogenase/oxidase (EC 1.17.1.4) (EC 1.17.3.2)	XDH	**M1**
W5QGV7	W5QGV7_SHEEP	Uncharacterized protein	MB21D2	**M1**
P21621	IL1B_SHEEP	Interleukin-1 beta (IL-1 beta)	IL1B	**M1**
W5PYW3	W5PYW3_SHEEP	C-X-C motif chemokine 3	CXCL6	**M1**
W5P530	W5P530_SHEEP	Serpin domain-containing protein	SERPINB2	**M1**
W5PAC2	W5PAC2_SHEEP	Serpin domain-containing protein	SERPINB2	**M1**
W5Q0A3	W5Q0A3_SHEEP	Toll-like receptor 2	TLR2	**M1**
W5QJ54	W5QJ54_SHEEP	Arginase (EC 3.5.3.1)	ARG2	**M1**
M4WG34	M4WG34_SHEEP	Multifunctional fusion protein [Includes: Interleukin-1; Interleukin-1 beta]	IL1B	**M1**
W5P2M5	W5P2M5_SHEEP	C-C motif chemokine	CCL3	**M1**
W5P4V3	W5P4V3_SHEEP	Stromelysin-1 (EC 3.4.24.17) (Matrix metalloproteinase-3)	MMP3	**M1**
W5NZD5	W5NZD5_SHEEP	DNA polymerase (EC 2.7.7.7)	POLB	**M1**
W5PNK3	W5PNK3_SHEEP	Aggrecan core protein (Cartilage-specific proteoglycan core protein)	ACAN	**M1**
W5PRG9	W5PRG9_SHEEP	Histidine ammonia-lyase (EC 4.3.1.3)	HAL	**M1**
W5PW33	W5PW33_SHEEP	Amine oxidase domain-containing protein	AOC3	**M1**
W5PJR5	W5PJR5_SHEEP	Serum amyloid A protein	SAA1	**M1**
W5PVJ5	W5PVJ5_SHEEP	Ig-like domain-containing protein (Programmed cell death 1 ligand 1)	CD274	**M1**

Protein ID	Entry Name	Protein names	Gene Names	Marker
W5P4C9	W5P4C9_SHEEP	Peptidase metallopeptidase domain-containing protein (Matrix metalloproteinase-12 (MMP-12)	MMP12	**M2**
W5PES6	W5PES6_SHEEP	Actin-related protein 2/3 complex subunit	ARPC1A	**M2**
W5PF73	W5PF73_SHEEP	Urokinase-type plasminogen activator	PLAU	**M2**
W5PH85	W5PH85_SHEEP	VWFA domain-containing protein	ITGAX	**M2**
W5PNG4	W5PNG4_SHEEP	Tower domain-containing protein	BRCA2	**M2**
W5PNP1	W5PNP1_SHEEP	Lactadherin	MFGE8	**M2**

Supplementary Table 21: List of 20 M1-specific and 57 M2-specific differentially abundant proteins (DAPs) that uniquely distinguish M1 and M2 macrophages, respectively, from M0 (monocytes at 3 hours), GMØ (macrophages differentiated with GM-CSF), MMØ (macrophages differentiated with M-CSF), and the opposing polarized subtype. M1, pro-inflammatory macrophages activated with GM-CSF/ LPS/ INF-γ; M2, anti-inflammatory macrophages activated with M-CSF/ IL-4. All proteins listed were identified as significantly differentially abundant with a false discovery rate (FDR, q-value) < 0.001.

Protein ID	Entry Name	Protein names	Gene Names	Marker
W5PQP3	W5PQP3_SHEEP	LIM domain kinase 1 (EC 2.7.11.1)	LIMK1	**M2**
W5Q655	W5Q655_SHEEP	ADP-ribosylation factor 1	ARF1	**M2**
W5QEL9	W5QEL9_SHEEP	Rho-GAP domain-containing protein	ARHGAP31	**M2**
Q29422	CD1B2_SHEEP	T-cell surface glycoprotein CD1b-2 (Antigen IAH-CC14) (sCD1B-42) (CD antigen CD1b-2)	CD1B2	**M2**
W5PHG6	W5PHG6_SHEEP	[histone H3]-lysine(4) N-trimethyltransferase (EC 2.1.1.354)	SMYD2	**M2**
W5PHX8	A0A6P3TIL4_SHEEP	Glutaredoxin-2, mitochondrial	GLRX2	**M2**
W5Q6D6	W5Q6D6_SHEEP	ELK domain-containing protein	AKAP9	**M2**
W5Q989	W5Q989_SHEEP	Procollagen-lysine,2-oxoglutarate 5-dioxygenase 1 (EC 1.14.11.4) (Lysyl hydroxylase 1)	PLOD1	**M2**
W5NPP2	W5NPP2_SHEEP	Peptidase M14 carboxypeptidase A domain-containing protein	CPM	**M2**
W5NS73	W5NS73_SHEEP	Septin	SEPTIN10	**M2**
W5PIN6	A0A6P7DAB4_SHEEP	L-lactate dehydrogenase (EC 1.1.1.27)	LDHA	**M2**
W5PRI6	W5PRI6_SHEEP	Mannose receptor C-type 1	MRC1	**M2**
W5QHC0	W5QHC0_SHEEP	Lactosylceramide alpha-2,3-sialyltransferase (EC 2.4.3.9) (CMP-NeuAc:lactosylceramide alpha-2,3-sialyltra	ST3GAL5	**M2**
W5PFR8	W5PFR8_SHEEP	Fructose-1,6-bisphosphatase 1 (EC 3.1.3.11) (D-fructose-1,6-bisphosphate 1-phosphohydrolase 1) (Liver FB	FBP1	**M2**
W5PHI1	W5PHI1_SHEEP	Large ribosomal subunit protein uL3m (39S ribosomal protein L3, mitochondrial)	MRPL3	**M2**
W5PJH6	W5PJH6_SHEEP	Zinc finger E-box-binding homeobox 2 (C2H2-type domain-containing protein)	ZEB2	**M2**
W5PVG5	W5PVG5_SHEEP	Chorein N-terminal domain-containing protein	BLTP3B	**M2**
W5PWK3	W5PWK3_SHEEP	FAST kinase domain-containing protein 4 (Protein TBRG4) (Transforming growth factor beta regulator 4)	TBRG4	**M2**
W5Q568	W5Q568_SHEEP	UvrD-like helicase ATP-binding domain-containing protein	TRANK1	**M2**
W5QC34	W5QC34_SHEEP	Alpha-mannosidase (EC 3.2.1.-)	MAN2A1	**M2**
W5QDG9	W5QDG9_SHEEP	Coiled-coil domain-containing protein 80	CCDC80	**M2**
W5QEG2	W5QEG2_SHEEP	MAP7 domain containing 1	MAP7D1	**M2**
W5NWF5	W5NWF5_SHEEP	Cystatin LXN-type domain-containing protein	RARRES1	**M2**
W5PCM4	W5PCM4_SHEEP	Leucine zipper protein 1	LUZP1	**M2**
W5PCT8	W5PCT8_SHEEP	ArfGAP with SH3 domain, ankyrin repeat and PH domain 1	ASAP1	**M2**
W5PFY5	W5PFY5_SHEEP	Acid ceramidase (EC 3.5.1.23)	ASAH1	**M2**
W5Q8B9	W5Q8B9_SHEEP	non-specific serine/threonine protein kinase (EC 2.7.11.1)	STK25	**M2**
W5QAQ4	W5QAQ4_SHEEP	Neuropilin	NRP2	**M2**

Supplementary Table 21: List of 20 M1-specific and 57 M2-specific differentially abundant proteins (DAPs) that uniquely distinguish M1 and M2 macrophages, respectively, from M0 (monocytes at 3 hours), GMØ (macrophages differentiated with GM-CSF), MMØ (macrophages differentiated with M-CSF), and the opposing polarized subtype. M1, pro-inflammatory macrophages activated with GM-CSF/ LPS/ INF-γ; M2, anti-inflammatory macrophages activated with M-CSF/ IL-4. All proteins listed were identified as significantly differentially abundant with a false discovery rate (FDR, q-value) < 0.001.

Protein ID	Entry Name	Protein names	Gene Names	Marker
W5PDE5	W5PDE5_SHEEP	Sushi domain-containing protein	C4BPA	**M2**
W5P0W4	W5P0W4_SHEEP	Semaphorin 7A (John Milton Hagen blood group)	SEMA7A	**M2**
W5NZX1	W5NZX1_SHEEP	Ig-like domain-containing protein	IL18BP	**M2**
W5P041	W5P041_SHEEP	Disintegrin and metalloproteinase domain-containing protein 15	ADAM15	**M2**
W5PGT0	W5PGT0_SHEEP	Myosin-11 (Myosin heavy chain 11)	MYH11	**M2**
W5PGV0	W5PGV0_SHEEP	Integrin subunit alpha M	ITGAM	**M2**
W5Q7S8	W5Q7S8_SHEEP	Neuropilin-1	NRP1	**M2**
W5QDG8	W5QDG8_SHEEP	Fibronectin	FN1	**M2**
W5QDI7	W5QDI7_SHEEP	Macrophage colony-stimulating factor 1 (CSF-1) (MCSF)	CSF1	**M2**
W5QI35	W5QI35_SHEEP	Interleukin-1 receptor antagonist protein	IL1RN	**M2**
W5NPK5	W5NPK5_SHEEP	Alpha-macroglobulin receptor-binding domain-containing protein	LOC443475	**M2**
W5NRI1	W5NRI1_SHEEP	Alpha-2-macroglobulin bait region domain-containing protein	A2M	**M2**
W5PSA3	W5PSA3_SHEEP	Transcobalamin-like C-terminal domain-containing protein	TCN1	**M2**
W5PTL2	W5PTL2_SHEEP	Properdin	CFP	**M2**
W5QD11	W5QD11_SHEEP	VPS10 domain-containing protein	SORT1	**M2**
W5NTX3	W5NTX3_SHEEP	Platelet-derived growth factor (PDGF) family profile domain-containing protein	PDGFA	**M2**
W5PMR2	W5PMR2_SHEEP	Matrix metalloproteinase-19 (Peptidase metallopeptidase domain-containing protein)	MMP19	**M2**
W5PF04	W5PF04_SHEEP	alpha-1,2-Mannosidase (EC 3.2.1.-)	MAN1A1	**M2**
W5PG72	W5PG72_SHEEP	Golgi membrane protein 1	GOLM1	**M2**
W5PBM2	W5PBM2_SHEEP	T-cell surface glycoprotein CD1e, membrane-associated (Ig-like domain-containing protein)	CD1E	**M2**
W5P0S9	W5P0S9_SHEEP	GP-PDE domain-containing protein	GDPD3	**M2**
W5PBX4	W5PBX4_SHEEP	Fatty acyl-CoA reductase (EC 1.2.1.84)	FAR1	**M2**
W5Q2Y8	W5Q2Y8_SHEEP	Eukaryotic translation initiation factor 4E	EIF4E	**M2**

Supplementary Table 22: Scores of potential hub protein analysis for M1- and M2-specific differentially abundant proteins (DAPs) generated using CytoHubba in Cytoscape. [8]. Hub proteins were ranked based on five topological scoring algorithms: Maximal Clique Centrality (MCC), Degree of Connectivity, Edge Percolated Component (EPC), Closeness, and Radiality. M1, pro-inflammatory macrophages activated with GM-CSF/ LPS/ INF-γ; M2, anti-inflammatory macrophages activated with M-CSF/ IL-4.

A. Hub proteins in M1 MAcrophages							
Protein symbol	Protein name	MCC	DC	EPC	Closeness	Radiality	Subtype
IL1B	Interleukin-1 beta	65.00	13.00	6.91	13.50	2.68	M1
CCL3	C-X-C motif chemokine 3	42.00	7.00	6.06	10.50	2.32	M1
MMP3	Stromelysin-1 (EC 3.4.24.17) (Matrix metalloproteinase-3)	38.00	7.00	6.01	10.33	2.26	M1
CD274	Programmed cell death 1 ligand 1	14.00	5.00	5.21	9.50	2.20	M1
**B. Hub proteins in M2 MAcrophages**							
Protein symbol	Protein name	MCC	DC	EPC	Closeness	Radiality	Subtype
ITGAM	Integrin subunit alpha M	99.00	10.00	11.46	16.50	2.10	M2
MMP12	Matrix metalloproteinase-12	93.00	10.00	11.31	16.50	2.10	M2
CSF1	Macrophage colony-stimulating factor 1 (MCSF)	85.00	8.00	11.02	15.33	2.04	M2
ITGAX	VWFA domain-containing protein (Integrin alpha-X)	66.00	7.00	10.82	14.83	2.03	M2
FN1	Fibronectin	57.00	12.00	11.66	17.83	2.18	M2
MRC1	Mannose receptor C-type 1	56.00	7.00	10.73	14.08	1.93	M2
IL1RN	Interleukin-1 receptor antagonist protein	31.00	6.00	9.92	13.58	1.91	M2
PLAU	Urokinase-type plasminogen activator	18.00	5.00	9.49	14.00	2.01	M2
NRP1	Neuropilin-1	15.00	7.00	9.77	14.50	1.99	M2
CD1E	T-cell surface glycoprotein CD1e, membrane-associated	12.00	4.00	9.14	12.50	1.87	M2
A2M	Alpha-2-macroglobulin bait region domain-containing protein	11.00	7.00	9.01	14.67	2.01	M2
PDGFA	Platelet-derived growth factor (PDGF) family profile domain-containing protein	8.00	4.00	9.36	13.00	1.93	M2
SEMA7A	Semaphorin-7A	3.00	3.00	6.73	11.25	1.74	M2
CFP	Properdin	2.00	2.00	6.08	11.42	1.81	M2
ZEB2	Zinc finger E-box-binding homeobox 2	2.00	2.00	4.90	11.08	1.77	M2
MMP19	Matrix metalloproteinase-19	2.00	2.00	5.80	10.92	1.74	M2

Supplementary Table 23: Comparative functional enrichment analysis of M1 and M2 realtaive to monocyte. Functional enrichment analysis was performed using the StringApp (STRING database) within Cytoscape (version 3.10.3)[1], focusing on Gene Ontology (GO) Biological Process (BP) terms[2,3].

Functional enrichment analysis of M1_Mo

category	term name_M1_M	description_M1_Mo	FDR value	# genes	ackground ge	Colu
GO Biological Process	GO:0009056	Catabolic process	3.94E-20	177	1731	
GO Biological Process	GO:0016192	Vesicle-mediated transport	1.52E-17	134	1197	
GO Biological Process	GO:1901575	Organic substance catabolic process	3.83E-16	151	1509	
GO Biological Process	GO:0044248	Cellular catabolic process	1.90E-12	135	1423	
GO Biological Process	GO:0006810	Transport	2.84E-11	255	3596	
GO Biological Process	GO:0051179	Localization	2.84E-11	291	4284	
GO Biological Process	GO:0033036	Macromolecule localization	8.71E-11	171	2117	
GO Biological Process	GO:0009987	Cellular process	1.09E-10	797	15933	
GO Biological Process	GO:0051234	Establishment of localization	1.59E-10	259	3748	
GO Biological Process	GO:0044281	Small molecule metabolic process	2.73E-10	132	1497	
GO Biological Process	GO:0070887	Cellular response to chemical stimulus	5.61E-10	163	2039	
GO Biological Process	GO:0008104	Protein localization	5.83E-10	143	1701	
GO Biological Process	GO:0051641	Cellular localization	6.09E-10	184	2416	
GO Biological Process	GO:1901136	Carbohydrate derivative catabolic process	6.20E-10	33	146	
GO Biological Process	GO:0045184	Establishment of protein localization	2.10E-09	102	1071	
GO Biological Process	GO:0006897	Endocytosis	2.36E-09	57	429	
GO Biological Process	GO:0015031	Protein transport	4.01E-09	96	993	
GO Biological Process	GO:0046907	Intracellular transport	5.56E-09	111	1237	
GO Biological Process	GO:0010033	Response to organic substance	1.59E-08	154	1990	
GO Biological Process	GO:0002376	Immune system process	3.90E-08	142	1807	
GO Biological Process	GO:1901135	Carbohydrate derivative metabolic process	5.70E-08	84	864	
GO Biological Process	GO:1901565	Organonitrogen compound catabolic process	5.70E-08	93	1003	
GO Biological Process	GO:0008152	Metabolic process	6.39E-08	460	8199	
GO Biological Process	GO:0042592	Homeostatic process	9.66E-08	102	1161	
GO Biological Process	GO:0005975	Carbohydrate metabolic process	1.38E-07	52	420	
GO Biological Process	GO:0032879	Regulation of localization	1.38E-07	132	1675	
GO Biological Process	GO:0065009	Regulation of molecular function	2.81E-07	184	2635	
GO Biological Process	GO:0006898	Receptor-mediated endocytosis	3.04E-07	27	133	
GO Biological Process	GO:0051649	Establishment of localization in cell	3.10E-07	125	1580	
GO Biological Process	GO:0044237	Cellular metabolic process	3.16E-07	380	6572	
GO Biological Process	GO:0044282	Small molecule catabolic process	3.16E-07	43	316	
GO Biological Process	GO:0019725	Cellular homeostasis	6.51E-07	56	500	
GO Biological Process	GO:0071310	Cellular response to organic substance	9.17E-07	122	1562	
GO Biological Process	GO:0002682	Regulation of immune system process	1.53E-06	100	1202	
GO Biological Process	GO:0071705	Nitrogen compound transport	1.68E-06	111	1391	
GO Biological Process	GO:0006950	Response to stress	1.91E-06	180	2640	
GO Biological Process	GO:0065008	Regulation of biological quality	2.26E-06	203	3085	
GO Biological Process	GO:0060627	Regulation of vesicle-mediated transport	2.35E-06	50	438	
GO Biological Process	GO:0071702	Organic substance transport	2.61E-06	131	1756	
GO Biological Process	GO:0044419	Biological process involved in interspecies interaction between	3.03E-06	98	1190	
GO Biological Process	GO:1901701	Cellular response to oxygen-containing compound	3.15E-06	70	739	
GO Biological Process	GO:0046466	Membrane lipid catabolic process	3.22E-06	14	36	
GO Biological Process	GO:0016042	Lipid catabolic process	3.85E-06	36	261	
GO Biological Process	GO:0016197	Endosomal transport	4.30E-06	31	202	
GO Biological Process	GO:0055067	Monovalent inorganic cation homeostasis	4.30E-06	24	125	
GO Biological Process	GO:0001817	Regulation of cytokine production	4.48E-06	59	581	
GO Biological Process	GO:0009605	Response to external stimulus	4.54E-06	135	1851	
GO Biological Process	GO:0030004	Cellular monovalent inorganic cation homeostasis	4.72E-06	20	87	
GO Biological Process	GO:0050790	Regulation of catalytic activity	4.72E-06	144	2016	
GO Biological Process	GO:0048878	Chemical homeostasis	7.23E-06	70	760	
GO Biological Process	GO:0071704	Organic substance metabolic process	8.00E-06	423	7705	
GO Biological Process	GO:0006886	Intracellular protein transport	1.06E-05	60	614	
GO Biological Process	GO:0019377	Glycolipid catabolic process	1.06E-05	10	16	
GO Biological Process	GO:1901700	Response to oxygen-containing compound	1.46E-05	82	971	

Supplementary Table 23: Comparative functional enrichment analysis of M1 and M2 realtaive to monocyte. Functional enrichment analysis was performed using the StringApp (STRING database) within Cytoscape (version 3.10.3)[1], focusing on Gene Ontology (GO) Biological Process (BP) terms[2,3].

GO Biological Process	GO:0098771	Inorganic ion homeostasis	1.52E-05	47	430	
GO Biological Process	GO:0050764	Regulation of phagocytosis	1.57E-05	19	86	
GO Biological Process	GO:0006873	Cellular ion homeostasis	1.62E-05	41	348	
GO Biological Process	GO:0030003	Cellular cation homeostasis	1.64E-05	40	335	
GO Biological Process	GO:0051239	Regulation of multicellular organismal process	1.64E-05	149	2159	
GO Biological Process	GO:0071345	Cellular response to cytokine stimulus	1.78E-05	56	566	
GO Biological Process	GO:0051707	Response to other organism	2.05E-05	86	1049	
GO Biological Process	GO:0006885	Regulation of pH	2.09E-05	18	79	
GO Biological Process	GO:0009607	Response to biotic stimulus	2.23E-05	89	1103	
GO Biological Process	GO:1901564	Organonitrogen compound metabolic process	2.23E-05	294	5039	
GO Biological Process	GO:0030149	Sphingolipid catabolic process	2.39E-05	12	31	
GO Biological Process	GO:0044093	Positive regulation of molecular function	2.67E-05	99	1281	
GO Biological Process	GO:0034097	Response to cytokine	2.78E-05	59	623	
GO Biological Process	GO:0044242	Cellular lipid catabolic process	3.08E-05	27	179	
GO Biological Process	GO:0007033	Vacuole organization	3.17E-05	24	145	
GO Biological Process	GO:0046479	Glycosphingolipid catabolic process	3.17E-05	9	14	
GO Biological Process	GO:0051049	Regulation of transport	3.17E-05	102	1340	
GO Biological Process	GO:0030036	Actin cytoskeleton organization	3.24E-05	51	506	
GO Biological Process	GO:0055080	Cation homeostasis	3.55E-05	45	420	
GO Biological Process	GO:0046514	Ceramide catabolic process	3.61E-05	10	20	
GO Biological Process	GO:0006793	Phosphorus metabolic process	3.64E-05	125	1757	
GO Biological Process	GO:0030641	Regulation of cellular pH	4.08E-05	16	66	
GO Biological Process	GO:0030029	Actin filament-based process	5.74E-05	53	548	
GO Biological Process	GO:0030334	Regulation of cell migration	7.47E-05	65	742	
GO Biological Process	GO:0050776	Regulation of immune response	7.47E-05	62	694	
GO Biological Process	GO:0006575	Cellular modified amino acid metabolic process	9.04E-05	24	156	
GO Biological Process	GO:0051336	Regulation of hydrolase activity	9.04E-05	73	878	
GO Biological Process	GO:0050865	Regulation of cell activation	1.00E-04	51	530	
GO Biological Process	GO:0030155	Regulation of cell adhesion	1.10E-04	57	625	
GO Biological Process	GO:0043436	Oxoacid metabolic process	1.10E-04	66	768	
GO Biological Process	GO:0006909	Phagocytosis	1.20E-04	23	148	
GO Biological Process	GO:0007010	Cytoskeleton organization	1.20E-04	89	1160	
GO Biological Process	GO:0032101	Regulation of response to external stimulus	1.20E-04	63	723	
GO Biological Process	GO:1901361	Organic cyclic compound catabolic process	1.50E-04	35	304	
GO Biological Process	GO:0002694	Regulation of leukocyte activation	1.70E-04	48	496	
GO Biological Process	GO:1901615	Organic hydroxy compound metabolic process	1.70E-04	41	392	
GO Biological Process	GO:0006796	Phosphate-containing compound metabolic process	2.10E-04	120	1736	
GO Biological Process	GO:0019752	Carboxylic acid metabolic process	2.10E-04	64	754	
GO Biological Process	GO:0040012	Regulation of locomotion	2.10E-04	68	821	
GO Biological Process	GO:0055082	Cellular chemical homeostasis	2.10E-04	42	410	
GO Biological Process	GO:2000145	Regulation of cell motility	2.10E-04	66	788	
GO Biological Process	GO:0009617	Response to bacterium	2.20E-04	49	517	
GO Biological Process	GO:0051050	Positive regulation of transport	2.20E-04	58	659	
GO Biological Process	GO:0009636	Response to toxic substance	2.40E-04	22	144	
GO Biological Process	GO:0043254	Regulation of protein-containing complex assembly	2.60E-04	38	357	
GO Biological Process	GO:0051249	Regulation of lymphocyte activation	2.60E-04	40	386	
GO Biological Process	GO:0002237	Response to molecule of bacterial origin	2.70E-04	26	195	
GO Biological Process	GO:0006952	Defense response	2.70E-04	80	1034	
GO Biological Process	GO:0007040	Lysosome organization	2.70E-04	15	70	
GO Biological Process	GO:2000377	Regulation of reactive oxygen species metabolic process	2.70E-04	17	90	
GO Biological Process	GO:0010035	Response to inorganic substance	3.50E-04	35	320	
GO Biological Process	GO:0044262	Cellular carbohydrate metabolic process	3.60E-04	22	149	
GO Biological Process	GO:0048583	Regulation of response to stimulus	3.60E-04	198	3268	
GO Biological Process	GO:0016052	Carbohydrate catabolic process	3.70E-04	17	93	
GO Biological Process	GO:0060341	Regulation of cellular localization	3.80E-04	63	757	
GO Biological Process	GO:0044270	Cellular nitrogen compound catabolic process	4.00E-04	31	267	

Supplementary Table 23: Comparative functional enrichment analysis of M1 and M2 realtaive to monocyte. Functional enrichment analysis was performed using the StringApp (STRING database) within Cytoscape (version 3.10.3)[1], focusing on Gene Ontology (GO) Biological Process (BP) terms[2,3].

GO Biological Process	GO:0006629	Lipid metabolic process	4.40E-04	86	1155	
GO Biological Process	GO:0042147	Retrograde transport, endosome to Golgi	4.40E-04	15	74	
GO Biological Process	GO:0044255	Cellular lipid metabolic process	4.40E-04	70	879	
GO Biological Process	GO:0019439	Aromatic compound catabolic process	5.10E-04	32	285	
GO Biological Process	GO:0019637	Organophosphate metabolic process	5.10E-04	65	799	
GO Biological Process	GO:0098754	Detoxification	5.10E-04	18	107	
GO Biological Process	GO:0001819	Positive regulation of cytokine production	5.30E-04	38	372	
GO Biological Process	GO:1901658	Glycosyl compound catabolic process	5.50E-04	10	31	
GO Biological Process	GO:0030100	Regulation of endocytosis	5.60E-04	22	155	
GO Biological Process	GO:0097242	Amyloid-beta clearance	5.90E-04	9	24	
GO Biological Process	GO:0071219	Cellular response to molecule of bacterial origin	6.10E-04	20	132	
GO Biological Process	GO:0032880	Regulation of protein localization	6.40E-04	57	674	
GO Biological Process	GO:0008154	Actin polymerization or depolymerization	6.80E-04	13	58	
GO Biological Process	GO:0030593	Neutrophil chemotaxis	6.80E-04	13	58	
GO Biological Process	GO:0046700	Heterocycle catabolic process	7.90E-04	30	265	
GO Biological Process	GO:0030574	Collagen catabolic process	8.10E-04	10	33	
GO Biological Process	GO:0032103	Positive regulation of response to external stimulus	8.10E-04	35	337	
GO Biological Process	GO:1990266	Neutrophil migration	8.10E-04	14	69	
GO Biological Process	GO:0042221	Response to chemical	8.50E-04	200	3365	
GO Biological Process	GO:0051453	Regulation of intracellular pH	8.90E-04	13	60	
GO Biological Process	GO:0002684	Positive regulation of immune system process	9.00E-04	60	734	
GO Biological Process	GO:0007035	Vacuolar acidification	9.00E-04	8	19	
GO Biological Process	GO:0031347	Regulation of defense response	9.90E-04	43	462	
GO Biological Process	GO:0016139	Glycoside catabolic process	0.001	6	8	
GO Biological Process	GO:0030833	Regulation of actin filament polymerization	0.001	20	138	
GO Biological Process	GO:0032956	Regulation of actin cytoskeleton organization	0.0011	33	315	
GO Biological Process	GO:0051016	Barbed-end actin filament capping	0.0011	8	20	
GO Biological Process	GO:0051246	Regulation of protein metabolic process	0.0011	144	2276	
GO Biological Process	GO:0071346	Cellular response to interferon-gamma	0.0011	15	82	
GO Biological Process	GO:0016050	Vesicle organization	0.0013	27	233	
GO Biological Process	GO:0050896	Response to stimulus	0.0013	383	7243	
GO Biological Process	GO:0050900	Leukocyte migration	0.0013	23	179	
GO Biological Process	GO:0006954	Inflammatory response	0.0014	34	335	
GO Biological Process	GO:0007041	Lysosomal transport	0.0014	17	107	
GO Biological Process	GO:0007264	Small GTPase mediated signal transduction	0.0014	26	221	
GO Biological Process	GO:0048584	Positive regulation of response to stimulus	0.0014	116	1758	
GO Biological Process	GO:0051452	Intracellular pH reduction	0.0014	9	28	
GO Biological Process	GO:0071216	Cellular response to biotic stimulus	0.0014	21	155	
GO Biological Process	GO:0016043	Cellular component organization	0.0015	280	5055	
GO Biological Process	GO:0032271	Regulation of protein polymerization	0.0015	23	182	
GO Biological Process	GO:0042325	Regulation of phosphorylation	0.0015	77	1046	
GO Biological Process	GO:0019216	Regulation of lipid metabolic process	0.0016	31	294	
GO Biological Process	GO:0036230	Granulocyte activation	0.0016	9	29	
GO Biological Process	GO:0051693	Actin filament capping	0.0016	10	37	
GO Biological Process	GO:0098542	Defense response to other organism	0.0016	61	771	
GO Biological Process	GO:0016482	Cytosolic transport	0.0017	18	121	
GO Biological Process	GO:0046486	Glycerolipid metabolic process	0.0017	34	340	
GO Biological Process	GO:0050866	Negative regulation of cell activation	0.0017	22	171	
GO Biological Process	GO:0061024	Membrane organization	0.0017	54	657	
GO Biological Process	GO:0097529	Myeloid leukocyte migration	0.0017	16	98	
GO Biological Process	GO:1901698	Response to nitrogen compound	0.0017	58	723	
GO Biological Process	GO:1990748	Cellular detoxification	0.0017	16	98	
GO Biological Process	GO:0043087	Regulation of GTPase activity	0.0018	33	326	
GO Biological Process	GO:0010256	Endomembrane system organization	0.0019	41	449	
GO Biological Process	GO:0006066	Alcohol metabolic process	0.002	29	270	
GO Biological Process	GO:0019220	Regulation of phosphate metabolic process	0.002	83	1166	

Supplementary Table 23: Comparative functional enrichment analysis of M1 and M2 realtaive to monocyte. Functional enrichment analysis was performed using the StringApp (STRING database) within Cytoscape (version 3.10.3)[1], focusing on Gene Ontology (GO) Biological Process (BP) terms[2,3].

GO Biological Process	GO:0034341	Response to interferon-gamma	0.002	16	100	
GO Biological Process	GO:0022603	Regulation of anatomical structure morphogenesis	0.0021	57	713	
GO Biological Process	GO:0032970	Regulation of actin filament-based process	0.0021	34	344	
GO Biological Process	GO:0071222	Cellular response to lipopolysaccharide	0.0021	18	124	
GO Biological Process	GO:0051128	Regulation of cellular component organization	0.0022	125	1955	
GO Biological Process	GO:0044283	Small molecule biosynthetic process	0.0023	36	377	
GO Biological Process	GO:0010628	Positive regulation of gene expression	0.0024	70	942	
GO Biological Process	GO:0007229	Integrin-mediated signaling pathway	0.0025	14	80	
GO Biological Process	GO:0070663	Regulation of leukocyte proliferation	0.0025	24	204	
GO Biological Process	GO:0032496	Response to lipopolysaccharide	0.0026	22	178	
GO Biological Process	GO:0048518	Positive regulation of biological process	0.0026	295	5423	
GO Biological Process	GO:0006875	Cellular metal ion homeostasis	0.0027	29	276	
GO Biological Process	GO:0007034	Vacuolar transport	0.0027	19	140	
GO Biological Process	GO:0043085	Positive regulation of catalytic activity	0.0027	70	947	
GO Biological Process	GO:0051130	Positive regulation of cellular component organization	0.0027	66	878	
GO Biological Process	GO:0055086	Nucleobase-containing small molecule metabolic process	0.0029	41	461	
GO Biological Process	GO:0002695	Negative regulation of leukocyte activation	0.003	20	154	
GO Biological Process	GO:0016053	Organic acid biosynthetic process	0.003	25	221	
GO Biological Process	GO:0061025	Membrane fusion	0.003	18	129	
GO Biological Process	GO:0010243	Response to organonitrogen compound	0.0031	52	643	
GO Biological Process	GO:0031663	Lipopolysaccharide-mediated signaling pathway	0.0031	9	33	
GO Biological Process	GO:0042119	Neutrophil activation	0.0031	8	25	
GO Biological Process	GO:0050766	Positive regulation of phagocytosis	0.0031	12	61	
GO Biological Process	GO:0030335	Positive regulation of cell migration	0.0032	38	417	
GO Biological Process	GO:0071356	Cellular response to tumor necrosis factor	0.0032	18	130	
GO Biological Process	GO:0071840	Cellular component organization or biogenesis	0.0034	288	5301	
GO Biological Process	GO:0009057	Macromolecule catabolic process	0.0035	67	906	
GO Biological Process	GO:0002831	Regulation of response to biotic stimulus	0.0037	27	254	
GO Biological Process	GO:0044238	Primary metabolic process	0.0037	381	7312	
GO Biological Process	GO:0045851	pH reduction	0.0037	6	12	
GO Biological Process	GO:0051345	Positive regulation of hydrolase activity	0.0037	41	468	
GO Biological Process	GO:0033993	Response to lipid	0.0039	38	422	
GO Biological Process	GO:0006689	Ganglioside catabolic process	0.0045	5	7	
GO Biological Process	GO:0071396	Cellular response to lipid	0.0048	30	303	
GO Biological Process	GO:0055065	Metal ion homeostasis	0.0049	33	349	
GO Biological Process	GO:0002683	Negative regulation of immune system process	0.005	31	319	
GO Biological Process	GO:0006040	Amino sugar metabolic process	0.005	9	36	
GO Biological Process	GO:0010038	Response to metal ion	0.005	22	189	
GO Biological Process	GO:0009064	Glutamine family amino acid metabolic process	0.0053	12	66	
GO Biological Process	GO:0046394	Carboxylic acid biosynthetic process	0.0054	24	218	
GO Biological Process	GO:0042063	Gliogenesis	0.0057	22	191	
GO Biological Process	GO:0019915	Lipid storage	0.0058	9	37	
GO Biological Process	GO:0034599	Cellular response to oxidative stress	0.0058	19	151	
GO Biological Process	GO:1902600	Proton transmembrane transport	0.0058	16	113	
GO Biological Process	GO:0045807	Positive regulation of endocytosis	0.0059	13	78	
GO Biological Process	GO:0006672	Ceramide metabolic process	0.0062	14	90	
GO Biological Process	GO:0040017	Positive regulation of locomotion	0.0062	39	450	
GO Biological Process	GO:0062197	Cellular response to chemical stress	0.0062	21	179	
GO Biological Process	GO:0001775	Cell activation	0.0063	43	516	
GO Biological Process	GO:0016477	Cell migration	0.0063	57	753	
GO Biological Process	GO:0071496	Cellular response to external stimulus	0.0063	26	250	
GO Biological Process	GO:0034614	Cellular response to reactive oxygen species	0.0064	13	79	
GO Biological Process	GO:0001932	Regulation of protein phosphorylation	0.0067	66	914	
GO Biological Process	GO:0019751	Polyol metabolic process	0.0067	14	91	
GO Biological Process	GO:0030595	Leukocyte chemotaxis	0.0067	15	103	
GO Biological Process	GO:0051250	Negative regulation of lymphocyte activation	0.0074	17	129	

Supplementary Table 23: Comparative functional enrichment analysis of M1 and M2 realtaive to monocyte. Functional enrichment analysis was performed using the StringApp (STRING database) within Cytoscape (version 3.10.3)[1], focusing on Gene Ontology (GO) Biological Process (BP) terms[2,3].

GO Biological Process	GO:0072583	Clathrin-dependent endocytosis	0.0076	8	30	
GO Biological Process	GO:0002699	Positive regulation of immune effector process	0.0078	23	211	
GO Biological Process	GO:0045087	Innate immune response	0.0078	44	539	
GO Biological Process	GO:0050830	Defense response to Gram-positive bacterium	0.0078	13	81	
GO Biological Process	GO:0051716	Cellular response to stimulus	0.0078	310	5846	
GO Biological Process	GO:0002697	Regulation of immune effector process	0.0079	29	300	
GO Biological Process	GO:0032272	Negative regulation of protein polymerization	0.0079	12	70	
GO Biological Process	GO:0032642	Regulation of chemokine production	0.0079	12	70	
GO Biological Process	GO:0050727	Regulation of inflammatory response	0.0083	26	256	
GO Biological Process	GO:0098869	Cellular oxidant detoxification	0.0084	13	82	
GO Biological Process	GO:0051240	Positive regulation of multicellular organismal process	0.0087	77	1125	
GO Biological Process	GO:0006048	UDP-N-acetylglucosamine biosynthetic process	0.009	5	9	
GO Biological Process	GO:0050670	Regulation of lymphocyte proliferation	0.0091	21	186	
GO Biological Process	GO:0006807	Nitrogen compound metabolic process	0.0092	348	6684	
GO Biological Process	GO:0071674	Mononuclear cell migration	0.0092	13	83	
GO Biological Process	GO:0009967	Positive regulation of signal transduction	0.0096	82	1222	
GO Biological Process	GO:0010941	Regulation of cell death	0.0098	82	1223	
GO Biological Process	GO:0009719	Response to endogenous stimulus	0.0099	66	931	
GO Biological Process	GO:0032680	Regulation of tumor necrosis factor production	0.0099	15	108	
GO Biological Process	GO:0051493	Regulation of cytoskeleton organization	0.01	40	481	
GO Biological Process	GO:0030837	Negative regulation of actin filament polymerization	0.0105	11	62	
GO Biological Process	GO:0006979	Response to oxidative stress	0.0109	25	247	
GO Biological Process	GO:0031349	Positive regulation of defense response	0.0109	23	218	
GO Biological Process	GO:0033554	Cellular response to stress	0.0109	87	1322	
GO Biological Process	GO:0032535	Regulation of cellular component size	0.0111	29	308	
GO Biological Process	GO:0032722	Positive regulation of chemokine production	0.0111	10	52	
GO Biological Process	GO:0050863	Regulation of T cell activation	0.0111	28	293	
GO Biological Process	GO:0110053	Regulation of actin filament organization	0.0111	25	248	
GO Biological Process	GO:1902743	Regulation of lamellipodium organization	0.0111	9	42	
GO Biological Process	GO:0032760	Positive regulation of tumor necrosis factor production	0.0114	11	63	
GO Biological Process	GO:0051235	Maintenance of location	0.0114	17	136	
GO Biological Process	GO:1901616	Organic hydroxy compound catabolic process	0.0114	11	63	
GO Biological Process	GO:0007265	Ras protein signal transduction	0.0115	19	163	
GO Biological Process	GO:0030203	Glycosaminoglycan metabolic process	0.0115	13	86	
GO Biological Process	GO:0045454	Cell redox homeostasis	0.0115	8	33	
GO Biological Process	GO:0002886	Regulation of myeloid leukocyte mediated immunity	0.0121	10	53	
GO Biological Process	GO:0031333	Negative regulation of protein-containing complex assembly	0.0121	16	124	
GO Biological Process	GO:0005996	Monosaccharide metabolic process	0.0125	18	151	
GO Biological Process	GO:0009966	Regulation of signal transduction	0.0125	144	2439	
GO Biological Process	GO:0045321	Leukocyte activation	0.0125	38	456	
GO Biological Process	GO:0048522	Positive regulation of cellular process	0.0125	260	4831	
GO Biological Process	GO:0055072	Iron ion homeostasis	0.0133	14	100	
GO Biological Process	GO:0006650	Glycerophospholipid metabolic process	0.0136	26	268	
GO Biological Process	GO:0042167	Heme catabolic process	0.0136	4	5	
GO Biological Process	GO:0002274	Myeloid leukocyte activation	0.0148	15	114	
GO Biological Process	GO:0070665	Positive regulation of leukocyte proliferation	0.0148	15	114	
GO Biological Process	GO:0090066	Regulation of anatomical structure size	0.0156	34	397	
GO Biological Process	GO:0051051	Negative regulation of transport	0.0161	27	287	
GO Biological Process	GO:0071241	Cellular response to inorganic substance	0.0165	17	142	
GO Biological Process	GO:0006955	Immune response	0.0166	70	1029	
GO Biological Process	GO:0031623	Receptor internalization	0.0166	10	56	
GO Biological Process	GO:0050729	Positive regulation of inflammatory response	0.0166	14	103	
GO Biological Process	GO:0010876	Lipid localization	0.0167	31	351	
GO Biological Process	GO:0045785	Positive regulation of cell adhesion	0.0167	32	367	
GO Biological Process	GO:0046488	Phosphatidylinositol metabolic process	0.0167	18	156	
GO Biological Process	GO:0055076	Transition metal ion homeostasis	0.0167	16	129	

Supplementary Table 23: Comparative functional enrichment analysis of M1 and M2 realtaive to monocyte. Functional enrichment analysis was performed using the StringApp (STRING database) within Cytoscape (version 3.10.3)[1], focusing on Gene Ontology (GO) Biological Process (BP) terms[2,3].

GO Biological Process	GO:0035556	Intracellular signal transduction	0.017	82	1254	
GO Biological Process	GO:0010634	Positive regulation of epithelial cell migration	0.0178	15	117	
GO Biological Process	GO:0045937	Positive regulation of phosphate metabolic process	0.0178	54	743	
GO Biological Process	GO:0043067	Regulation of programmed cell death	0.0184	75	1127	
GO Biological Process	GO:0010647	Positive regulation of cell communication	0.0185	87	1353	
GO Biological Process	GO:0043547	Positive regulation of GTPase activity	0.0185	24	245	
GO Biological Process	GO:0032787	Monocarboxylic acid metabolic process	0.0187	38	469	
GO Biological Process	GO:0048534	Hematopoietic or lymphoid organ development	0.0189	44	571	
GO Biological Process	GO:0007015	Actin filament organization	0.0191	25	261	
GO Biological Process	GO:1901699	Cellular response to nitrogen compound	0.0192	38	470	
GO Biological Process	GO:0008652	Cellular amino acid biosynthetic process	0.0194	11	69	
GO Biological Process	GO:2000379	Positive regulation of reactive oxygen species metabolic proces	0.0194	8	37	
GO Biological Process	GO:0006665	Sphingolipid metabolic process	0.0196	16	132	
GO Biological Process	GO:0006749	Glutathione metabolic process	0.0196	10	58	
GO Biological Process	GO:0006879	Cellular iron ion homeostasis	0.0196	12	81	
GO Biological Process	GO:0019430	Removal of superoxide radicals	0.0196	5	12	
GO Biological Process	GO:0150093	Amyloid-beta clearance by transcytosis	0.0196	4	6	
GO Biological Process	GO:2001268	Negative regulation of cysteine-type endopeptidase activity inv	0.0196	4	6	
GO Biological Process	GO:0043244	Regulation of protein-containing complex disassembly	0.0197	15	119	
GO Biological Process	GO:0042129	Regulation of T cell proliferation	0.0199	17	146	
GO Biological Process	GO:0031668	Cellular response to extracellular stimulus	0.0202	21	203	
GO Biological Process	GO:0046395	Carboxylic acid catabolic process	0.0202	21	203	
GO Biological Process	GO:0002685	Regulation of leukocyte migration	0.0211	19	175	
GO Biological Process	GO:0016236	Macroautophagy	0.0211	18	161	
GO Biological Process	GO:0023056	Positive regulation of signaling	0.0211	87	1362	
GO Biological Process	GO:0046916	Cellular transition metal ion homeostasis	0.0211	14	107	
GO Biological Process	GO:0043277	Apoptotic cell clearance	0.0214	8	38	
GO Biological Process	GO:0002696	Positive regulation of leukocyte activation	0.0215	26	280	
GO Biological Process	GO:0010743	Regulation of macrophage derived foam cell differentiation	0.0215	6	20	
GO Biological Process	GO:0042981	Regulation of apoptotic process	0.0215	73	1100	
GO Biological Process	GO:0044403	Biological process involved in symbiotic interaction	0.0215	20	190	
GO Biological Process	GO:1901657	Glycosyl compound metabolic process	0.0224	11	71	
GO Biological Process	GO:0048259	Regulation of receptor-mediated endocytosis	0.0225	12	83	
GO Biological Process	GO:0031647	Regulation of protein stability	0.0227	25	266	
GO Biological Process	GO:0002703	Regulation of leukocyte mediated immunity	0.023	21	206	
GO Biological Process	GO:1901607	Alpha-amino acid biosynthetic process	0.0232	10	60	
GO Biological Process	GO:0071675	Regulation of mononuclear cell migration	0.0234	13	96	
GO Biological Process	GO:0048284	Organelle fusion	0.0237	14	109	
GO Biological Process	GO:0006906	Vesicle fusion	0.024	12	84	
GO Biological Process	GO:0019318	Hexose metabolic process	0.024	16	136	
GO Biological Process	GO:0035987	Endodermal cell differentiation	0.024	8	39	
GO Biological Process	GO:0048245	Eosinophil chemotaxis	0.024	5	13	
GO Biological Process	GO:0061462	Protein localization to lysosome	0.024	8	39	
GO Biological Process	GO:1901605	Alpha-amino acid metabolic process	0.024	19	178	
GO Biological Process	GO:0034655	Nucleobase-containing compound catabolic process	0.0247	23	238	
GO Biological Process	GO:0060326	Cell chemotaxis	0.0254	18	165	
GO Biological Process	GO:0010632	Regulation of epithelial cell migration	0.0255	20	194	
GO Biological Process	GO:0022407	Regulation of cell-cell adhesion	0.0261	32	382	
GO Biological Process	GO:0006643	Membrane lipid metabolic process	0.0262	19	180	
GO Biological Process	GO:0046477	Glycosylceramide catabolic process	0.0262	4	7	
GO Biological Process	GO:0048549	Positive regulation of pinocytosis	0.0262	4	7	
GO Biological Process	GO:0030162	Regulation of proteolysis	0.0266	49	674	
GO Biological Process	GO:0022604	Regulation of cell morphogenesis	0.0267	23	240	
GO Biological Process	GO:0006520	Cellular amino acid metabolic process	0.0275	24	256	
GO Biological Process	GO:0044092	Negative regulation of molecular function	0.0275	64	947	
GO Biological Process	GO:0002520	Immune system development	0.0283	45	606	

Supplementary Table 23: Comparative functional enrichment analysis of M1 and M2 realtaive to monocyte. Functional enrichment analysis was performed using the StringApp (STRING database) within Cytoscape (version 3.10.3)[1], focusing on Gene Ontology (GO) Biological Process (BP) terms[2,3].

GO Biological Process	GO:1903037	Regulation of leukocyte cell-cell adhesion	0.0283	26	288	
GO Biological Process	GO:0032386	Regulation of intracellular transport	0.029	23	242	
GO Biological Process	GO:0010591	Regulation of lamellipodium assembly	0.0292	7	31	
GO Biological Process	GO:0010829	Negative regulation of glucose transmembrane transport	0.0292	5	14	
GO Biological Process	GO:0031952	Regulation of protein autophosphorylation	0.0292	7	31	
GO Biological Process	GO:0045056	Transcytosis	0.0292	5	14	
GO Biological Process	GO:0050821	Protein stabilization	0.0292	18	168	
GO Biological Process	GO:0051702	Biological process involved in interaction with symbiont	0.0292	12	87	
GO Biological Process	GO:2000641	Regulation of early endosome to late endosome transport	0.0292	5	14	
GO Biological Process	GO:2001234	Negative regulation of apoptotic signaling pathway	0.0298	19	183	
GO Biological Process	GO:0042327	Positive regulation of phosphorylation	0.0302	49	680	
GO Biological Process	GO:0009628	Response to abiotic stimulus	0.0304	52	734	
GO Biological Process	GO:0071248	Cellular response to metal ion	0.0306	15	127	
GO Biological Process	GO:1902903	Regulation of supramolecular fiber organization	0.0311	29	339	
GO Biological Process	GO:0050671	Positive regulation of lymphocyte proliferation	0.0318	13	101	
GO Biological Process	GO:0048870	Cell motility	0.0328	61	901	
GO Biological Process	GO:0040011	Locomotion	0.0329	35	440	
GO Biological Process	GO:1900076	Regulation of cellular response to insulin stimulus	0.0333	9	53	
GO Biological Process	GO:0071417	Cellular response to organonitrogen compound	0.0338	34	424	
GO Biological Process	GO:0002888	Positive regulation of myeloid leukocyte mediated immunity	0.034	6	23	
GO Biological Process	GO:0006622	Protein targeting to lysosome	0.034	6	23	
GO Biological Process	GO:0009063	Cellular amino acid catabolic process	0.034	13	102	
GO Biological Process	GO:0009065	Glutamine family amino acid catabolic process	0.034	6	23	
GO Biological Process	GO:0009141	Nucleoside triphosphate metabolic process	0.0341	17	157	
GO Biological Process	GO:0019262	N-acetylneuraminate catabolic process	0.0346	4	8	
GO Biological Process	GO:0042102	Positive regulation of T cell proliferation	0.0346	11	77	
GO Biological Process	GO:0006525	Arginine metabolic process	0.0351	5	15	
GO Biological Process	GO:0006644	Phospholipid metabolic process	0.0351	29	343	
GO Biological Process	GO:0046348	Amino sugar catabolic process	0.0351	5	15	
GO Biological Process	GO:0060099	Regulation of phagocytosis, engulfment	0.0351	5	15	
GO Biological Process	GO:0019882	Antigen processing and presentation	0.0355	12	90	
GO Biological Process	GO:1905475	Regulation of protein localization to membrane	0.0355	17	158	
GO Biological Process	GO:0008360	Regulation of cell shape	0.0357	15	130	
GO Biological Process	GO:0044275	Cellular carbohydrate catabolic process	0.0366	7	33	
GO Biological Process	GO:0046856	Phosphatidylinositol dephosphorylation	0.0366	7	33	
GO Biological Process	GO:0043069	Negative regulation of programmed cell death	0.0374	49	690	
GO Biological Process	GO:0006914	Autophagy	0.0381	22	234	
GO Biological Process	GO:0045719	Negative regulation of glycogen biosynthetic process	0.0381	3	3	
GO Biological Process	GO:0052547	Regulation of peptidase activity	0.0381	33	412	
GO Biological Process	GO:0051247	Positive regulation of protein metabolic process	0.0384	78	1230	
GO Biological Process	GO:1905476	Negative regulation of protein localization to membrane	0.0386	6	24	
GO Biological Process	GO:0048193	Golgi vesicle transport	0.0391	24	266	
GO Biological Process	GO:0071407	Cellular response to organic cyclic compound	0.0398	29	347	
GO Biological Process	GO:0009894	Regulation of catabolic process	0.0431	55	805	
GO Biological Process	GO:0002221	Pattern recognition receptor signaling pathway	0.0442	10	68	
GO Biological Process	GO:0046164	Alcohol catabolic process	0.0442	8	45	
GO Biological Process	GO:1903828	Negative regulation of protein localization	0.0444	16	148	
GO Biological Process	GO:0006527	Arginine catabolic process	0.045	4	9	
GO Biological Process	GO:0046365	Monosaccharide catabolic process	0.045	6	25	
GO Biological Process	GO:0000902	Cell morphogenesis	0.0454	44	609	
GO Biological Process	GO:0009725	Response to hormone	0.0455	34	435	
GO Biological Process	GO:0009991	Response to extracellular stimulus	0.0455	26	302	
GO Biological Process	GO:0072593	Reactive oxygen species metabolic process	0.0459	11	81	
GO Biological Process	GO:0009620	Response to fungus	0.0464	7	35	
GO Biological Process	GO:0043300	Regulation of leukocyte degranulation	0.0464	7	35	
GO Biological Process	GO:0061640	Cytoskeleton-dependent cytokinesis	0.0464	12	94	

Supplementary Table 23: Comparative functional enrichment analysis of M1 and M2 realtaive to monocyte. Functional enrichment analysis was performed using the StringApp (STRING database) within Cytoscape (version 3.10.3)[1], focusing on Gene Ontology (GO) Biological Process (BP) terms[2,3].

GO Biological Process	GO:0006935	Chemotaxis	0.0465	33	419	
GO Biological Process	GO:0046434	Organophosphate catabolic process	0.0468	15	135	
GO Biological Process	GO:0032757	Positive regulation of interleukin-8 production	0.0483	8	46	
GO Biological Process	GO:0046173	Polyol biosynthetic process	0.0483	8	46	
GO Biological Process	GO:0050864	Regulation of B cell activation	0.0484	13	108	
GO Biological Process	GO:0071453	Cellular response to oxygen levels	0.0491	11	82	
GO Biological Process	GO:0006006	Glucose metabolic process	0.0493	12	95	

Functional enrichment analysis of M2_Mo

category	term name_M2_M	description_M2_Mo	FDR value	# genes	ackground ge	gene
GO Biological Process	GO:0016192	Vesicle-mediated transport	8.33E-20	163	1197	
GO Biological Process	GO:0009056	Catabolic process	2.15E-15	193	1731	
GO Biological Process	GO:0044281	Small molecule metabolic process	1.27E-14	172	1497	
GO Biological Process	GO:0051179	Localization	2.47E-14	365	4284	
GO Biological Process	GO:0051641	Cellular localization	2.47E-14	239	2416	
GO Biological Process	GO:0033036	Macromolecule localization	4.04E-12	209	2117	
GO Biological Process	GO:0051234	Establishment of localization	1.10E-11	318	3748	
GO Biological Process	GO:0006810	Transport	4.73E-11	305	3596	
GO Biological Process	GO:1901575	Organic substance catabolic process	4.75E-11	160	1509	
GO Biological Process	GO:0060627	Regulation of vesicle-mediated transport	5.53E-11	71	438	
GO Biological Process	GO:0008104	Protein localization	8.99E-11	173	1701	
GO Biological Process	GO:0009987	Cellular process	7.08E-10	984	15933	
GO Biological Process	GO:0006897	Endocytosis	2.41E-09	66	429	
GO Biological Process	GO:0008152	Metabolic process	2.41E-09	574	8199	
GO Biological Process	GO:0044248	Cellular catabolic process	2.41E-09	147	1423	
GO Biological Process	GO:0032879	Regulation of localization	4.94E-09	164	1675	
GO Biological Process	GO:0051649	Establishment of localization in cell	5.01E-09	157	1580	
GO Biological Process	GO:0046907	Intracellular transport	4.87E-08	128	1237	
GO Biological Process	GO:0006793	Phosphorus metabolic process	6.96E-08	165	1757	
GO Biological Process	GO:0051049	Regulation of transport	6.96E-08	135	1340	
GO Biological Process	GO:0016043	Cellular component organization	1.43E-07	377	5055	
GO Biological Process	GO:0030155	Regulation of cell adhesion	1.67E-07	78	625	
GO Biological Process	GO:0065009	Regulation of molecular function	1.69E-07	223	2635	
GO Biological Process	GO:0071704	Organic substance metabolic process	1.78E-07	533	7705	
GO Biological Process	GO:1901135	Carbohydrate derivative metabolic process	1.81E-07	97	864	
GO Biological Process	GO:1901564	Organonitrogen compound metabolic process	1.81E-07	375	5039	
GO Biological Process	GO:0051336	Regulation of hydrolase activity	1.86E-07	98	878	
GO Biological Process	GO:0006796	Phosphate-containing compound metabolic process	2.01E-07	161	1736	
GO Biological Process	GO:0019637	Organophosphate metabolic process	3.02E-07	91	799	
GO Biological Process	GO:0051493	Regulation of cytoskeleton organization	5.30E-07	64	481	
GO Biological Process	GO:0006898	Receptor-mediated endocytosis	6.02E-07	30	133	
GO Biological Process	GO:0055086	Nucleobase-containing small molecule metabolic process	6.23E-07	62	461	
GO Biological Process	GO:1902903	Regulation of supramolecular fiber organization	6.24E-07	51	339	
GO Biological Process	GO:0051050	Positive regulation of transport	9.59E-07	78	659	
GO Biological Process	GO:0050790	Regulation of catalytic activity	1.57E-06	176	2016	
GO Biological Process	GO:0006996	Organelle organization	1.63E-06	258	3265	
GO Biological Process	GO:0007041	Lysosomal transport	1.64E-06	26	107	
GO Biological Process	GO:0002376	Immune system process	2.02E-06	161	1807	
GO Biological Process	GO:0044237	Cellular metabolic process	2.02E-06	459	6572	
GO Biological Process	GO:0043436	Oxoacid metabolic process	2.74E-06	85	768	
GO Biological Process	GO:0007229	Integrin-mediated signaling pathway	3.33E-06	22	80	
GO Biological Process	GO:0051128	Regulation of cellular component organization	3.33E-06	170	1955	
GO Biological Process	GO:0007034	Vacuolar transport	4.24E-06	29	140	
GO Biological Process	GO:0071840	Cellular component organization or biogenesis	4.24E-06	381	5301	
GO Biological Process	GO:0070887	Cellular response to chemical stimulus	4.27E-06	175	2039	
GO Biological Process	GO:0032970	Regulation of actin filament-based process	4.42E-06	49	344	

Supplementary Table 23: Comparative functional enrichment analysis of M1 and M2 realtaive to monocyte. Functional enrichment analysis was performed using the StringApp (STRING database) within Cytoscape (version 3.10.3)[1], focusing on Gene Ontology (GO) Biological Process (BP) terms[2,3].

GO Biological Process	GO:0045184	Establishment of protein localization	4.42E-06	107	1071	
GO Biological Process	GO:0061024	Membrane organization	5.52E-06	75	657	
GO Biological Process	GO:0032956	Regulation of actin cytoskeleton organization	5.89E-06	46	315	
GO Biological Process	GO:0007033	Vacuole organization	7.15E-06	29	145	
GO Biological Process	GO:0005975	Carbohydrate metabolic process	7.40E-06	55	420	
GO Biological Process	GO:1901136	Carbohydrate derivative catabolic process	7.84E-06	29	146	
GO Biological Process	GO:0006950	Response to stress	8.13E-06	213	2640	
GO Biological Process	GO:0030036	Actin cytoskeleton organization	8.61E-06	62	506	
GO Biological Process	GO:0010256	Endomembrane system organization	1.01E-05	57	449	
GO Biological Process	GO:1901565	Organonitrogen compound catabolic process	1.18E-05	100	1003	
GO Biological Process	GO:0016042	Lipid catabolic process	1.28E-05	40	261	
GO Biological Process	GO:0019752	Carboxylic acid metabolic process	1.35E-05	81	754	
GO Biological Process	GO:0016050	Vesicle organization	1.73E-05	37	233	
GO Biological Process	GO:0030100	Regulation of endocytosis	2.07E-05	29	155	
GO Biological Process	GO:0043254	Regulation of protein-containing complex assembly	2.25E-05	48	357	
GO Biological Process	GO:0015031	Protein transport	2.28E-05	98	993	
GO Biological Process	GO:0002682	Regulation of immune system process	2.40E-05	113	1202	
GO Biological Process	GO:0048583	Regulation of response to stimulus	2.45E-05	250	3268	
GO Biological Process	GO:0065008	Regulation of biological quality	2.83E-05	238	3085	
GO Biological Process	GO:0016197	Endosomal transport	4.29E-05	33	202	
GO Biological Process	GO:0045785	Positive regulation of cell adhesion	4.29E-05	48	367	
GO Biological Process	GO:0071702	Organic substance transport	4.52E-05	150	1756	
GO Biological Process	GO:0009611	Response to wounding	5.06E-05	38	257	
GO Biological Process	GO:0010810	Regulation of cell-substrate adhesion	5.06E-05	29	164	
GO Biological Process	GO:0110053	Regulation of actin filament organization	5.76E-05	37	248	
GO Biological Process	GO:0006753	Nucleoside phosphate metabolic process	5.92E-05	51	408	
GO Biological Process	GO:0032101	Regulation of response to external stimulus	6.02E-05	76	723	
GO Biological Process	GO:0090066	Regulation of anatomical structure size	6.12E-05	50	397	
GO Biological Process	GO:0044238	Primary metabolic process	6.74E-05	489	7312	
GO Biological Process	GO:0044242	Cellular lipid catabolic process	7.95E-05	30	179	
GO Biological Process	GO:0050764	Regulation of phagocytosis	8.03E-05	20	86	
GO Biological Process	GO:0044087	Regulation of cellular component biogenesis	8.67E-05	84	839	
GO Biological Process	GO:0007010	Cytoskeleton organization	9.65E-05	107	1160	
GO Biological Process	GO:0048518	Positive regulation of biological process	9.65E-05	377	5423	
GO Biological Process	GO:2000145	Regulation of cell motility	9.65E-05	80	788	
GO Biological Process	GO:0044419	Biological process involved in interspecies interaction between	9.91E-05	109	1190	
GO Biological Process	GO:0032271	Regulation of protein polymerization	9.94E-05	30	182	
GO Biological Process	GO:0033043	Regulation of organelle organization	1.00E-04	98	1037	
GO Biological Process	GO:0042060	Wound healing	1.10E-04	32	204	
GO Biological Process	GO:0043087	Regulation of GTPase activity	1.10E-04	43	326	
GO Biological Process	GO:0051246	Regulation of protein metabolic process	1.10E-04	182	2276	
GO Biological Process	GO:0030334	Regulation of cell migration	1.20E-04	76	742	
GO Biological Process	GO:0044282	Small molecule catabolic process	1.20E-04	42	316	
GO Biological Process	GO:0001952	Regulation of cell-matrix adhesion	1.40E-04	19	82	
GO Biological Process	GO:0009117	Nucleotide metabolic process	1.40E-04	49	401	
GO Biological Process	GO:1903532	Positive regulation of secretion by cell	1.40E-04	30	186	
GO Biological Process	GO:0010035	Response to inorganic substance	1.60E-04	42	320	
GO Biological Process	GO:0051047	Positive regulation of secretion	1.60E-04	32	209	
GO Biological Process	GO:0006909	Phagocytosis	1.70E-04	26	148	
GO Biological Process	GO:0050865	Regulation of cell activation	1.80E-04	59	530	
GO Biological Process	GO:0006629	Lipid metabolic process	1.90E-04	105	1155	
GO Biological Process	GO:0040012	Regulation of locomotion	1.90E-04	81	821	
GO Biological Process	GO:0051345	Positive regulation of hydrolase activity	1.90E-04	54	468	
GO Biological Process	GO:0007596	Blood coagulation	2.00E-04	20	94	
GO Biological Process	GO:0016482	Cytosolic transport	2.00E-04	23	121	
GO Biological Process	GO:0044255	Cellular lipid metabolic process	2.00E-04	85	879	

Supplementary Table 23: Comparative functional enrichment analysis of M1 and M2 realtaive to monocyte. Functional enrichment analysis was performed using the StringApp (STRING database) within Cytoscape (version 3.10.3)[1], focusing on Gene Ontology (GO) Biological Process (BP) terms[2,3].

GO Biological Process	GO:0051668	Localization within membrane	2.00E-04	53	458	
GO Biological Process	GO:0051239	Regulation of multicellular organismal process	2.20E-04	172	2159	
GO Biological Process	GO:0007040	Lysosome organization	2.50E-04	17	70	
GO Biological Process	GO:0032272	Negative regulation of protein polymerization	2.50E-04	17	70	
GO Biological Process	GO:1902531	Regulation of intracellular signal transduction	2.50E-04	119	1368	
GO Biological Process	GO:1902905	Positive regulation of supramolecular fiber organization	2.70E-04	25	144	
GO Biological Process	GO:0051247	Positive regulation of protein metabolic process	3.00E-04	109	1230	
GO Biological Process	GO:0051130	Positive regulation of cellular component organization	3.20E-04	84	878	
GO Biological Process	GO:0051235	Maintenance of location	3.30E-04	24	136	
GO Biological Process	GO:0010941	Regulation of cell death	3.80E-04	108	1223	
GO Biological Process	GO:0030835	Negative regulation of actin filament depolymerization	3.80E-04	13	42	
GO Biological Process	GO:0032103	Positive regulation of response to external stimulus	4.10E-04	42	337	
GO Biological Process	GO:0006914	Autophagy	4.30E-04	33	234	
GO Biological Process	GO:0010033	Response to organic substance	4.40E-04	159	1990	
GO Biological Process	GO:0016139	Glycoside catabolic process	4.40E-04	7	8	
GO Biological Process	GO:0046466	Membrane lipid catabolic process	4.50E-04	12	36	
GO Biological Process	GO:0071705	Nitrogen compound transport	4.50E-04	119	1391	
GO Biological Process	GO:1901701	Cellular response to oxygen-containing compound	4.70E-04	73	739	
GO Biological Process	GO:0048522	Positive regulation of cellular process	4.80E-04	335	4831	
GO Biological Process	GO:0016236	Macroautophagy	4.90E-04	26	161	
GO Biological Process	GO:0030162	Regulation of proteolysis	5.10E-04	68	674	
GO Biological Process	GO:0044093	Positive regulation of molecular function	5.50E-04	111	1281	
GO Biological Process	GO:0050878	Regulation of body fluid levels	5.50E-04	33	238	
GO Biological Process	GO:0051693	Actin filament capping	5.50E-04	12	37	
GO Biological Process	GO:0097242	Amyloid-beta clearance	5.50E-04	10	24	
GO Biological Process	GO:1901615	Organic hydroxy compound metabolic process	5.50E-04	46	392	
GO Biological Process	GO:0032535	Regulation of cellular component size	5.60E-04	39	308	
GO Biological Process	GO:0009966	Regulation of signal transduction	5.80E-04	187	2439	
GO Biological Process	GO:1902904	Negative regulation of supramolecular fiber organization	5.80E-04	25	153	
GO Biological Process	GO:0016477	Cell migration	7.80E-04	73	753	
GO Biological Process	GO:0030834	Regulation of actin filament depolymerization	7.80E-04	14	54	
GO Biological Process	GO:0022603	Regulation of anatomical structure morphogenesis	8.00E-04	70	713	
GO Biological Process	GO:0052547	Regulation of peptidase activity	8.00E-04	47	412	
GO Biological Process	GO:0071310	Cellular response to organic substance	8.00E-04	129	1562	
GO Biological Process	GO:0043547	Positive regulation of GTPase activity	8.60E-04	33	245	
GO Biological Process	GO:0046486	Glycerolipid metabolic process	8.80E-04	41	340	
GO Biological Process	GO:0051016	Barbed-end actin filament capping	8.80E-04	9	20	
GO Biological Process	GO:0051640	Organelle localization	8.80E-04	49	439	
GO Biological Process	GO:0060341	Regulation of cellular localization	8.80E-04	73	757	
GO Biological Process	GO:1901616	Organic hydroxy compound catabolic process	8.80E-04	15	63	
GO Biological Process	GO:0009617	Response to bacterium	8.90E-04	55	517	
GO Biological Process	GO:0048284	Organelle fusion	9.80E-04	20	109	
GO Biological Process	GO:0007015	Actin filament organization	0.0011	34	261	
GO Biological Process	GO:0019377	Glycolipid catabolic process	0.0014	8	16	
GO Biological Process	GO:0090407	Organophosphate biosynthetic process	0.0014	48	436	
GO Biological Process	GO:0006807	Nitrogen compound metabolic process	0.0015	439	6684	
GO Biological Process	GO:0061640	Cytoskeleton-dependent cytokinesis	0.0015	18	94	
GO Biological Process	GO:0006520	Cellular amino acid metabolic process	0.0017	33	256	
GO Biological Process	GO:0042592	Homeostatic process	0.0017	100	1161	
GO Biological Process	GO:0006650	Glycerophospholipid metabolic process	0.0018	34	268	
GO Biological Process	GO:0007155	Cell adhesion	0.0018	71	749	
GO Biological Process	GO:0080134	Regulation of response to stress	0.0018	93	1062	
GO Biological Process	GO:0070527	Platelet aggregation	0.0019	9	23	
GO Biological Process	GO:0031589	Cell-substrate adhesion	0.002	24	157	
GO Biological Process	GO:0050766	Positive regulation of phagocytosis	0.0021	14	61	
GO Biological Process	GO:0072665	Protein localization to vacuole	0.0021	14	61	

Supplementary Table 23: Comparative functional enrichment analysis of M1 and M2 realtaive to monocyte. Functional enrichment analysis was performed using the StringApp (STRING database) within Cytoscape (version 3.10.3)[1], focusing on Gene Ontology (GO) Biological Process (BP) terms[2,3].

GO Biological Process	GO:1905475	Regulation of protein localization to membrane	0.0021	24	158	
GO Biological Process	GO:0008333	Endosome to lysosome transport	0.0022	13	53	
GO Biological Process	GO:0043067	Regulation of programmed cell death	0.0022	97	1127	
GO Biological Process	GO:0046164	Alcohol catabolic process	0.0022	12	45	
GO Biological Process	GO:0010647	Positive regulation of cell communication	0.0023	112	1353	
GO Biological Process	GO:0030837	Negative regulation of actin filament polymerization	0.0023	14	62	
GO Biological Process	GO:0032880	Regulation of protein localization	0.0023	65	674	
GO Biological Process	GO:0042981	Regulation of apoptotic process	0.0023	95	1100	
GO Biological Process	GO:0044262	Cellular carbohydrate metabolic process	0.0023	23	149	
GO Biological Process	GO:0051495	Positive regulation of cytoskeleton organization	0.0023	25	170	
GO Biological Process	GO:1901700	Response to oxygen-containing compound	0.0023	86	971	
GO Biological Process	GO:0001817	Regulation of cytokine production	0.0024	58	581	
GO Biological Process	GO:0002478	Antigen processing and presentation of exogenous peptide ant	0.0024	10	31	
GO Biological Process	GO:0030149	Sphingolipid catabolic process	0.0024	10	31	
GO Biological Process	GO:0045921	Positive regulation of exocytosis	0.0024	15	71	
GO Biological Process	GO:1901658	Glycosyl compound catabolic process	0.0024	10	31	
GO Biological Process	GO:0051051	Negative regulation of transport	0.0025	35	287	
GO Biological Process	GO:0002703	Regulation of leukocyte mediated immunity	0.0026	28	206	
GO Biological Process	GO:0010811	Positive regulation of cell-substrate adhesion	0.0026	18	100	
GO Biological Process	GO:0019882	Antigen processing and presentation	0.0026	17	90	
GO Biological Process	GO:0051707	Response to other organism	0.0026	91	1049	
GO Biological Process	GO:0006623	Protein targeting to vacuole	0.0027	11	39	
GO Biological Process	GO:0010646	Regulation of cell communication	0.0027	201	2747	
GO Biological Process	GO:0023051	Regulation of signaling	0.0027	202	2763	
GO Biological Process	GO:0023056	Positive regulation of signaling	0.0027	112	1362	
GO Biological Process	GO:0050818	Regulation of coagulation	0.0027	13	55	
GO Biological Process	GO:0061462	Protein localization to lysosome	0.0027	11	39	
GO Biological Process	GO:0048584	Positive regulation of response to stimulus	0.0028	138	1758	
GO Biological Process	GO:0019884	Antigen processing and presentation of exogenous antigen	0.0032	11	40	
GO Biological Process	GO:0048259	Regulation of receptor-mediated endocytosis	0.0032	16	83	
GO Biological Process	GO:0050727	Regulation of inflammatory response	0.0032	32	256	
GO Biological Process	GO:1903530	Regulation of secretion by cell	0.0032	43	392	
GO Biological Process	GO:0006644	Phospholipid metabolic process	0.0033	39	343	
GO Biological Process	GO:0009607	Response to biotic stimulus	0.0033	94	1103	
GO Biological Process	GO:0015918	Sterol transport	0.0033	15	74	
GO Biological Process	GO:0016052	Carbohydrate catabolic process	0.0033	17	93	
GO Biological Process	GO:0006066	Alcohol metabolic process	0.0035	33	270	
GO Biological Process	GO:0006906	Vesicle fusion	0.0035	16	84	
GO Biological Process	GO:0031333	Negative regulation of protein-containing complex assembly	0.0036	20	124	
GO Biological Process	GO:0046479	Glycosphingolipid catabolic process	0.0036	7	14	
GO Biological Process	GO:0006575	Cellular modified amino acid metabolic process	0.0037	23	156	
GO Biological Process	GO:0008064	Regulation of actin polymerization or depolymerization	0.0037	23	156	
GO Biological Process	GO:0043085	Positive regulation of catalytic activity	0.0037	83	947	
GO Biological Process	GO:0046514	Ceramide catabolic process	0.0037	8	20	
GO Biological Process	GO:0010466	Negative regulation of peptidase activity	0.004	30	237	
GO Biological Process	GO:0098876	Vesicle-mediated transport to the plasma membrane	0.004	19	115	
GO Biological Process	GO:0048534	Hematopoietic or lymphoid organ development	0.0041	56	571	
GO Biological Process	GO:0072521	Purine-containing compound metabolic process	0.0041	38	335	
GO Biological Process	GO:0051046	Regulation of secretion	0.0042	46	439	
GO Biological Process	GO:0051129	Negative regulation of cellular component organization	0.0042	55	558	
GO Biological Process	GO:0051248	Negative regulation of protein metabolic process	0.0042	79	894	
GO Biological Process	GO:0051494	Negative regulation of cytoskeleton organization	0.0042	22	147	
GO Biological Process	GO:0052548	Regulation of endopeptidase activity	0.0042	41	374	
GO Biological Process	GO:0048519	Negative regulation of biological process	0.0044	306	4510	
GO Biological Process	GO:0051241	Negative regulation of multicellular organismal process	0.0044	69	754	
GO Biological Process	GO:0009967	Positive regulation of signal transduction	0.0046	101	1222	

Supplementary Table 23: Comparative functional enrichment analysis of M1 and M2 realtaive to monocyte. Functional enrichment analysis was performed using the StringApp (STRING database) within Cytoscape (version 3.10.3)[1], focusing on Gene Ontology (GO) Biological Process (BP) terms[2,3].

GO Biological Process	GO:0010628	Positive regulation of gene expression	0.0046	82	942	
GO Biological Process	GO:0010876	Lipid localization	0.0046	39	351	
GO Biological Process	GO:0030833	Regulation of actin filament polymerization	0.0046	21	138	
GO Biological Process	GO:0060099	Regulation of phagocytosis, engulfment	0.0046	7	15	
GO Biological Process	GO:0048870	Cell motility	0.0049	79	901	
GO Biological Process	GO:2001234	Negative regulation of apoptotic signaling pathway	0.0049	25	183	
GO Biological Process	GO:0061025	Membrane fusion	0.0052	20	129	
GO Biological Process	GO:0007032	Endosome organization	0.0053	12	52	
GO Biological Process	GO:0010951	Negative regulation of endopeptidase activity	0.0054	29	231	
GO Biological Process	GO:0002684	Positive regulation of immune system process	0.0056	67	734	
GO Biological Process	GO:0007160	Cell-matrix adhesion	0.0056	17	99	
GO Biological Process	GO:0045861	Negative regulation of proteolysis	0.0056	36	317	
GO Biological Process	GO:0019725	Cellular homeostasis	0.0057	50	500	
GO Biological Process	GO:2001233	Regulation of apoptotic signaling pathway	0.0057	35	305	
GO Biological Process	GO:0022407	Regulation of cell-cell adhesion	0.0058	41	382	
GO Biological Process	GO:0051651	Maintenance of location in cell	0.0058	15	80	
GO Biological Process	GO:0010638	Positive regulation of organelle organization	0.0059	47	461	
GO Biological Process	GO:0030097	Hemopoiesis	0.0059	52	528	
GO Biological Process	GO:0050776	Regulation of immune response	0.0059	64	694	
GO Biological Process	GO:1901657	Glycosyl compound metabolic process	0.006	14	71	
GO Biological Process	GO:0001775	Cell activation	0.0062	51	516	
GO Biological Process	GO:0019915	Lipid storage	0.0062	10	37	
GO Biological Process	GO:0002699	Positive regulation of immune effector process	0.0065	27	211	
GO Biological Process	GO:0071219	Cellular response to molecule of bacterial origin	0.0065	20	132	
GO Biological Process	GO:0006979	Response to oxidative stress	0.0066	30	247	
GO Biological Process	GO:0010038	Response to metal ion	0.0071	25	189	
GO Biological Process	GO:0070372	Regulation of ERK1 and ERK2 cascade	0.0071	29	236	
GO Biological Process	GO:0071216	Cellular response to biotic stimulus	0.0071	22	155	
GO Biological Process	GO:0043277	Apoptotic cell clearance	0.0072	10	38	
GO Biological Process	GO:0030301	Cholesterol transport	0.0076	12	55	
GO Biological Process	GO:0033365	Protein localization to organelle	0.0076	59	632	
GO Biological Process	GO:0050900	Leukocyte migration	0.0077	24	179	
GO Biological Process	GO:0048878	Chemical homeostasis	0.0078	68	760	
GO Biological Process	GO:1903076	Regulation of protein localization to plasma membrane	0.0078	17	103	
GO Biological Process	GO:0002694	Regulation of leukocyte activation	0.0079	49	496	
GO Biological Process	GO:0002697	Regulation of immune effector process	0.0079	34	300	
GO Biological Process	GO:0002520	Immune system development	0.008	57	606	
GO Biological Process	GO:0046434	Organophosphate catabolic process	0.008	20	135	
GO Biological Process	GO:1903034	Regulation of response to wounding	0.0082	18	114	
GO Biological Process	GO:0019318	Hexose metabolic process	0.0086	20	136	
GO Biological Process	GO:0006790	Sulfur compound metabolic process	0.0087	32	277	
GO Biological Process	GO:0022406	Membrane docking	0.0091	14	75	
GO Biological Process	GO:0034250	Positive regulation of cellular amide metabolic process	0.0092	19	126	
GO Biological Process	GO:0045851	pH reduction	0.0093	6	12	
GO Biological Process	GO:0048193	Golgi vesicle transport	0.0093	31	266	
GO Biological Process	GO:0030168	Platelet activation	0.0096	10	40	
GO Biological Process	GO:0034109	Homotypic cell-cell adhesion	0.0096	10	40	
GO Biological Process	GO:0022607	Cellular component assembly	0.0102	160	2176	
GO Biological Process	GO:0008154	Actin polymerization or depolymerization	0.0108	12	58	
GO Biological Process	GO:0017157	Regulation of exocytosis	0.0108	23	173	
GO Biological Process	GO:0044092	Negative regulation of molecular function	0.011	80	947	
GO Biological Process	GO:0001954	Positive regulation of cell-matrix adhesion	0.0111	10	41	
GO Biological Process	GO:0019693	Ribose phosphate metabolic process	0.0111	36	333	
GO Biological Process	GO:0032231	Regulation of actin filament bundle assembly	0.0111	15	87	
GO Biological Process	GO:0044275	Cellular carbohydrate catabolic process	0.0111	9	33	
GO Biological Process	GO:0009605	Response to external stimulus	0.0115	139	1851	

Supplementary Table 23: Comparative functional enrichment analysis of M1 and M2 realtaive to monocyte. Functional enrichment analysis was performed using the StringApp (STRING database) within Cytoscape (version 3.10.3)[1], focusing on Gene Ontology (GO) Biological Process (BP) terms[2,3].

GO Biological Process	GO:0045727	Positive regulation of translation	0.0116	17	108	
GO Biological Process	GO:0097435	Supramolecular fiber organization	0.0116	49	507	
GO Biological Process	GO:0030193	Regulation of blood coagulation	0.0118	11	50	
GO Biological Process	GO:0060548	Negative regulation of cell death	0.0118	67	761	
GO Biological Process	GO:0045807	Positive regulation of endocytosis	0.012	14	78	
GO Biological Process	GO:0008360	Regulation of cell shape	0.0121	19	130	
GO Biological Process	GO:0060100	Positive regulation of phagocytosis, engulfment	0.0122	6	13	
GO Biological Process	GO:0034614	Cellular response to reactive oxygen species	0.0133	14	79	
GO Biological Process	GO:0043086	Negative regulation of catalytic activity	0.0138	60	666	
GO Biological Process	GO:0019220	Regulation of phosphate metabolic process	0.0139	94	1166	
GO Biological Process	GO:0042325	Regulation of phosphorylation	0.0139	86	1046	
GO Biological Process	GO:1901605	Alpha-amino acid metabolic process	0.0146	23	178	
GO Biological Process	GO:0010543	Regulation of platelet activation	0.015	9	35	
GO Biological Process	GO:0042063	Gliogenesis	0.0157	24	191	
GO Biological Process	GO:0006886	Intracellular protein transport	0.0159	56	614	
GO Biological Process	GO:0010829	Negative regulation of glucose transmembrane transport	0.0159	6	14	
GO Biological Process	GO:0061041	Regulation of wound healing	0.0159	15	91	
GO Biological Process	GO:2000641	Regulation of early endosome to late endosome transport	0.0159	6	14	
GO Biological Process	GO:0022604	Regulation of cell morphogenesis	0.016	28	240	
GO Biological Process	GO:0031347	Regulation of defense response	0.016	45	462	
GO Biological Process	GO:1901361	Organic cyclic compound catabolic process	0.0168	33	304	
GO Biological Process	GO:0002886	Regulation of myeloid leukocyte mediated immunity	0.0169	11	53	
GO Biological Process	GO:1900076	Regulation of cellular response to insulin stimulus	0.0169	11	53	
GO Biological Process	GO:0048523	Negative regulation of cellular process	0.0174	265	3935	
GO Biological Process	GO:0051056	Regulation of small GTPase mediated signal transduction	0.0174	25	205	
GO Biological Process	GO:0050729	Positive regulation of inflammatory response	0.018	16	103	
GO Biological Process	GO:0002683	Negative regulation of immune system process	0.0183	34	319	
GO Biological Process	GO:0055067	Monovalent inorganic cation homeostasis	0.0185	18	125	
GO Biological Process	GO:0050896	Response to stimulus	0.0196	457	7243	
GO Biological Process	GO:0002705	Positive regulation of leukocyte mediated immunity	0.02	18	126	
GO Biological Process	GO:0006869	Lipid transport	0.02	34	321	
GO Biological Process	GO:0007030	Golgi organization	0.02	18	126	
GO Biological Process	GO:0050819	Negative regulation of coagulation	0.02	9	37	
GO Biological Process	GO:0071345	Cellular response to cytokine stimulus	0.0202	52	566	
GO Biological Process	GO:0034097	Response to cytokine	0.0204	56	623	
GO Biological Process	GO:0048585	Negative regulation of response to stimulus	0.0204	103	1322	
GO Biological Process	GO:0009259	Ribonucleotide metabolic process	0.0205	34	322	
GO Biological Process	GO:0050801	Ion homeostasis	0.0205	43	442	
GO Biological Process	GO:0072657	Protein localization to membrane	0.0205	39	388	
GO Biological Process	GO:0098771	Inorganic ion homeostasis	0.0217	42	430	
GO Biological Process	GO:0030335	Positive regulation of cell migration	0.0222	41	417	
GO Biological Process	GO:0032456	Endocytic recycling	0.0222	12	65	
GO Biological Process	GO:0050714	Positive regulation of protein secretion	0.0225	14	85	
GO Biological Process	GO:0034599	Cellular response to oxidative stress	0.0231	20	151	
GO Biological Process	GO:0010883	Regulation of lipid storage	0.0236	10	47	
GO Biological Process	GO:0051346	Negative regulation of hydrolase activity	0.0243	33	313	
GO Biological Process	GO:0098754	Detoxification	0.0243	16	107	
GO Biological Process	GO:0065007	Biological regulation	0.0245	732	12208	
GO Biological Process	GO:0001818	Negative regulation of cytokine production	0.0252	24	200	
GO Biological Process	GO:0006622	Protein targeting to lysosome	0.0254	7	23	
GO Biological Process	GO:0072378	Blood coagulation, fibrin clot formation	0.0254	5	10	
GO Biological Process	GO:0072594	Establishment of protein localization to organelle	0.0254	32	301	
GO Biological Process	GO:0061518	Microglial cell proliferation	0.0255	4	5	
GO Biological Process	GO:1905952	Regulation of lipid localization	0.0255	18	130	
GO Biological Process	GO:2000147	Positive regulation of cell motility	0.0255	42	435	
GO Biological Process	GO:0043244	Regulation of protein-containing complex disassembly	0.0257	17	119	

Supplementary Table 23: Comparative functional enrichment analysis of M1 and M2 realtaive to monocyte. Functional enrichment analysis was performed using the StringApp (STRING database) within Cytoscape (version 3.10.3)[1], focusing on Gene Ontology (GO) Biological Process (BP) terms[2,3].

GO Biological Process	GO:0071363	Cellular response to growth factor stimulus	0.0257	35	341	
GO Biological Process	GO:0051222	Positive regulation of protein transport	0.0261	24	201	
GO Biological Process	GO:0061045	Negative regulation of wound healing	0.0261	10	48	
GO Biological Process	GO:0070848	Response to growth factor	0.0263	36	355	
GO Biological Process	GO:0040017	Positive regulation of locomotion	0.0264	43	450	
GO Biological Process	GO:0140056	Organelle localization by membrane tethering	0.0264	12	67	
GO Biological Process	GO:0006163	Purine nucleotide metabolic process	0.0268	33	316	
GO Biological Process	GO:1990748	Cellular detoxification	0.0269	15	98	
GO Biological Process	GO:1902533	Positive regulation of intracellular signal transduction	0.0278	66	780	
GO Biological Process	GO:0006446	Regulation of translational initiation	0.0281	11	58	
GO Biological Process	GO:0007159	Leukocyte cell-cell adhesion	0.0287	9	40	
GO Biological Process	GO:0031113	Regulation of microtubule polymerization	0.0287	9	40	
GO Biological Process	GO:0097006	Regulation of plasma lipoprotein particle levels	0.0287	9	40	
GO Biological Process	GO:0034763	Negative regulation of transmembrane transport	0.0288	13	78	
GO Biological Process	GO:0062197	Cellular response to chemical stress	0.0289	22	179	
GO Biological Process	GO:0010720	Positive regulation of cell development	0.0294	25	216	
GO Biological Process	GO:0006887	Exocytosis	0.03	24	204	
GO Biological Process	GO:0070374	Positive regulation of ERK1 and ERK2 cascade	0.03	20	156	
GO Biological Process	GO:1905953	Negative regulation of lipid localization	0.03	8	32	
GO Biological Process	GO:1905477	Positive regulation of protein localization to membrane	0.0301	14	89	
GO Biological Process	GO:0051099	Positive regulation of binding	0.0302	18	133	
GO Biological Process	GO:0034446	Substrate adhesion-dependent cell spreading	0.0307	11	59	
GO Biological Process	GO:0000302	Response to reactive oxygen species	0.0309	15	100	
GO Biological Process	GO:0006885	Regulation of pH	0.031	13	79	
GO Biological Process	GO:0008652	Cellular amino acid biosynthetic process	0.0312	12	69	
GO Biological Process	GO:0009141	Nucleoside triphosphate metabolic process	0.0316	20	157	
GO Biological Process	GO:0043066	Negative regulation of apoptotic process	0.0316	58	669	
GO Biological Process	GO:0031349	Positive regulation of defense response	0.032	25	218	
GO Biological Process	GO:0042730	Fibrinolysis	0.032	5	11	
GO Biological Process	GO:0051014	Actin filament severing	0.032	5	11	
GO Biological Process	GO:0006873	Cellular ion homeostasis	0.0324	35	348	
GO Biological Process	GO:0006954	Inflammatory response	0.0329	34	335	
GO Biological Process	GO:0051656	Establishment of organelle localization	0.0332	33	322	
GO Biological Process	GO:0019886	Antigen processing and presentation of exogenous peptide ant	0.034	7	25	
GO Biological Process	GO:0030195	Negative regulation of blood coagulation	0.034	8	33	
GO Biological Process	GO:0030574	Collagen catabolic process	0.034	8	33	
GO Biological Process	GO:0031663	Lipopolysaccharide-mediated signaling pathway	0.034	8	33	
GO Biological Process	GO:0046856	Phosphatidylinositol dephosphorylation	0.034	8	33	
GO Biological Process	GO:0002237	Response to molecule of bacterial origin	0.0346	23	195	
GO Biological Process	GO:0044283	Small molecule biosynthetic process	0.0346	37	377	
GO Biological Process	GO:0071222	Cellular response to lipopolysaccharide	0.0346	17	124	
GO Biological Process	GO:0032233	Positive regulation of actin filament bundle assembly	0.0349	10	51	
GO Biological Process	GO:0007039	Protein catabolic process in the vacuole	0.0357	4	6	
GO Biological Process	GO:0048261	Negative regulation of receptor-mediated endocytosis	0.0357	6	18	
GO Biological Process	GO:0150093	Amyloid-beta clearance by transcytosis	0.0357	4	6	
GO Biological Process	GO:1903972	Regulation of cellular response to macrophage colony-stimulati	0.0357	4	6	
GO Biological Process	GO:0009968	Negative regulation of signal transduction	0.0364	83	1048	
GO Biological Process	GO:0043069	Negative regulation of programmed cell death	0.0366	59	690	
GO Biological Process	GO:0055080	Cation homeostasis	0.0368	40	420	
GO Biological Process	GO:0009165	Nucleotide biosynthetic process	0.0369	24	209	
GO Biological Process	GO:1902652	Secondary alcohol metabolic process	0.0369	14	92	
GO Biological Process	GO:0045806	Negative regulation of endocytosis	0.0382	8	34	
GO Biological Process	GO:0071825	Protein-lipid complex subunit organization	0.0382	8	34	
GO Biological Process	GO:0046887	Positive regulation of hormone secretion	0.0386	13	82	
GO Biological Process	GO:0071346	Cellular response to interferon-gamma	0.0386	13	82	
GO Biological Process	GO:0098927	Vesicle-mediated transport between endosomal compartments	0.0387	7	26	

Supplementary Table 23: Comparative functional enrichment analysis of M1 and M2 realtaive to monocyte. Functional enrichment analysis was performed using the StringApp (STRING database) within Cytoscape (version 3.10.3)[1], focusing on Gene Ontology (GO) Biological Process (BP) terms[2,3].

GO Biological Process	GO:0150116	Regulation of cell-substrate junction organization	0.0391	9	43	
GO Biological Process	GO:1903035	Negative regulation of response to wounding	0.0394	11	62	
GO Biological Process	GO:2001237	Negative regulation of extrinsic apoptotic signaling pathway	0.0394	12	72	
GO Biological Process	GO:0019430	Removal of superoxide radicals	0.0397	5	12	
GO Biological Process	GO:0010942	Positive regulation of cell death	0.0399	40	423	
GO Biological Process	GO:0035556	Intracellular signal transduction	0.0404	96	1254	
GO Biological Process	GO:0055082	Cellular chemical homeostasis	0.041	39	410	
GO Biological Process	GO:0009150	Purine ribonucleotide metabolic process	0.0412	31	302	
GO Biological Process	GO:0007035	Vacuolar acidification	0.042	6	19	
GO Biological Process	GO:0010761	Fibroblast migration	0.042	6	19	
GO Biological Process	GO:0032369	Negative regulation of lipid transport	0.042	6	19	
GO Biological Process	GO:0043300	Regulation of leukocyte degranulation	0.0427	8	35	
GO Biological Process	GO:0032760	Positive regulation of tumor necrosis factor production	0.0429	11	63	
GO Biological Process	GO:0048002	Antigen processing and presentation of peptide antigen	0.0429	11	63	
GO Biological Process	GO:0009395	Phospholipid catabolic process	0.0433	9	44	
GO Biological Process	GO:0030032	Lamellipodium assembly	0.0446	7	27	
GO Biological Process	GO:0046174	Polyol catabolic process	0.0446	7	27	
GO Biological Process	GO:0042147	Retrograde transport, endosome to Golgi	0.0462	12	74	
GO Biological Process	GO:2000116	Regulation of cysteine-type endopeptidase activity	0.0467	21	177	
GO Biological Process	GO:0031032	Actomyosin structure organization	0.0485	15	107	
GO Biological Process	GO:0044403	Biological process involved in symbiotic interaction	0.0485	22	190	
GO Biological Process	GO:0006689	Ganglioside catabolic process	0.0486	4	7	
GO Biological Process	GO:0006952	Defense response	0.0499	81	1034	

Red-highlighted cells indicate Gene Ontology Biological Process terms shared between M1 and M2 compared to M0, whereas non-highlighted cells represent subtype-specific enrichment. M1, pro-inflammatory macrophages activated with GM-CSF/ LPS/ INF-γ; M2, anti-inflammatory macrophages activated with M-CSF/ IL-4, and M0, Monocytes at 3 hours.

Supplementary Table 24: Comparative functional enrichment analysis of M1 and M2 relative to respective MØ (monocyte derived macrophage). Functional enrichment analysis was performed using the StringApp (STRING database) within Cytoscape (version 3.10.3)[1], focusing on Gene Ontology (GO) Biological Process (BP) terms[2,3].

Functional enrichment analysis of M1_GMØ

category	term name_M1_G	description_M1_GMØ	FDR value	# genes	ackground ger	genes
GO Biological Process	GO:0009607	Response to biotic stimulus	0.0066	25	1103	[S100A8, STAB1, CLEC4E, PTX3, W5P026_SHEEP, LOC101114535, MMP12, ERBIN, C8A, LOC101117129, STAT2, GSDME,
GO Biological Process	GO:0009617	Response to bacterium	0.0066	16	517	[S100A8, STAB1, CLEC4E, W5P026_SHEEP, LOC101114535, ERBIN, LOC101117129, RNASE4, W5PTZ8_SHEEP, BANK1,
GO Biological Process	GO:0032963	Collagen metabolic process	0.0066	6	52	[COL1A1, MMP12, MMP3, MMP1, MMP9, MMP2]
GO Biological Process	GO:0044419	Biological process involved in interspe	0.0066	25	1190	[S100A8, STAB1, CLEC4E, PTX3, W5P026_SHEEP, LOC101114535, TCP1, MMP12, ERBIN, C8A, LOC101117129, STAT2,
GO Biological Process	GO:0051707	Response to other organism	0.0066	24	1049	[S100A8, STAB1, CLEC4E, PTX3, W5P026_SHEEP, LOC101114535, MMP12, ERBIN, C8A, LOC101117129, STAT2, GSDME,
GO Biological Process	GO:0065009	Regulation of molecular function	0.0066	41	2635	[SZT2, TBC1D2B, S100A8, FLT4, HSPA2, W5NSN4_SHEEP, TPX2, RARRES1, CHM, PTX3, LOC101113086, LOC101114535, TCP1, PRKAR2B, LOC101104705, ERBIN, NDUFA4, LOC101105044, MMP9, LOC101117129, PEX19, MICAL1, XDH, CYFIP2,
GO Biological Process	GO:0030574	Collagen catabolic process	0.0111	5	33	[MMP12, MMP3, MMP1, MMP9, MMP2]
GO Biological Process	GO:0050790	Regulation of catalytic activity	0.0117	33	2016	[SZT2, TBC1D2B, S100A8, FLT4, W5NSN4_SHEEP, TPX2, RARRES1, CHM, PTX3, LOC101113086, LOC101114535, TCP1, PRKAR2B, LOC101104705, NDUFA4, LOC101105044, MMP9, LOC101117129, MICAL1, XDH, CYFIP2, ACAP1, RNASE4,
GO Biological Process	GO:0098542	Defense response to other organism	0.0199	18	771	[S100A8, STAB1, CLEC4E, PTX3, W5P026_SHEEP, LOC101114535, C8A, LOC101117129, STAT2, RNASE4, W5PTZ8_SHEEP,
GO Biological Process	GO:0002683	Negative regulation of immune systen	0.0314	11	319	[LOC101114535, MMP12, TNFAIP6, STAT2, AHR, BANK1, CD274, W5PVN6_SHEEP, IL4I1, SAMSN1, ARG2]
GO Biological Process	GO:0009605	Response to external stimulus	0.0314	30	1851	[SZT2, S100A8, STAB1, CLEC4E, PTX3, W5P026_SHEEP, LOC101114535, COL1A1, MMP12, ERBIN, C8A, EMB,
GO Biological Process	GO:0032879	Regulation of localization	0.0314	28	1675	[S100A8, HSPA2, CASK, PTX3, UBAC2, LOC101114535, TCP1, EEPD1, NDUFA4, MMP9, TNFAIP6, MICAL1, ACSL5, SH3TC1,
GO Biological Process	GO:0050777	Negative regulation of immune respor	0.0314	7	115	[MMP12, STAT2, AHR, W5PVN6_SHEEP, IL4I1, SAMSN1, ARG2]
GO Biological Process	GO:0051246	Regulation of protein metabolic proce	0.0314	34	2276	[S100A8, FLT4, HSPA2, TPX2, CDH5, RARRES1, PTX3, LOC101114535, PRKAR2B, LOC101104705, LOC101105044, MMP9,
GO Biological Process	GO:1901700	Response to oxygen-containing compo	0.0314	20	971	[S100A8, NR3C1, LOC101114535, SOD2, COL1A1, ERBIN, MMP9, GUCY1B1, MAN1A1, STAT2, AHR, CA2, CD274,
GO Biological Process	GO:0051049	Regulation of transport	0.0322	24	1340	[S100A8, HSPA2, CASK, PTX3, UBAC2, LOC101114535, EEPD1, NDUFA4, MMP9, MICAL1, ACSL5, SH3TC1, RNASE4, CA2,

Functional enrichment analysis of M2_MMØ

category	term name M2_M	description_M2_MMØ	FDR value	# genes	ackground ger	genes
GO Biological Process	GO:0007229	Integrin-mediated signaling pathway	0.0018	9	80	[ADAM15, SEMA7A, ITGAM, ITGAX, ITGB2, NRP1, ZYX, FN1, PLEK]
GO Biological Process	GO:0001934	Positive regulation of protein phospho	0.0031	21	607	[W5NPK5_SHEEP, PSEN1, W5NRI1_SHEEP, RALBP1, BAG4, PGF, CCDC88A, SEMA7A, DIPK2A, CSF1R, PDGFC, MMP9, PLAUR, MAP2K2, RNASE4, RAF1, AKAP9, NRP1, CD44, CSF1, TFRC]
GO Biological Process	GO:0065009	Regulation of molecular function	0.005	50	2635	[W5NPK5_SHEEP, PSEN1, W5NRI1_SHEEP, RALBP1, OXSR1, BAG4, STXBP5, RARRES1, CCDC88A, ADAM15, CLIC2, B2M, LOC100101238, MSH2, LAMTOR2, RASGRP4, DIPK2A, CSF1R, PDGFC, ASAP1, MMP9, PKIB, PLAU, PLAUR, MAP2K2, LIMK1, FURIN, RNASE4, TIMP1, LGMN, PARP4, APLP2, CAMK1D, RAF1, BST-2B, AKAP9, NRP1, W5Q9Y6_SHEEP, FGL2, CD44,
GO Biological Process	GO:0001932	Regulation of protein phosphorylation	0.0077	25	914	[W5NPK5_SHEEP, PSEN1, W5NRI1_SHEEP, RALBP1, BAG4, PGF, CCDC88A, SEMA7A, DIPK2A, CSF1R, PDGFC, MMP9, PKIB,
GO Biological Process	GO:0002684	Positive regulation of immune system	0.0077	21	734	[W5NPK5_SHEEP, PSEN1, W5NRI1_SHEEP, PGF, W5NWJ9_SHEEP, B2M, SEMA7A, MMP12, MSH2, TNFSF13B, CSF1R, CD1E,
GO Biological Process	GO:0002697	Regulation of immune effector proces	0.0077	13	300	[W5NPK5_SHEEP, W5NRI1_SHEEP, B2M, SEMA7A, MSH2, CD1E, LOC101111409, LOC101120001, CD84, ITGAM, ITGB2,
GO Biological Process	GO:0002699	Positive regulation of immune effecto	0.0077	11	211	[W5NPK5_SHEEP, W5NRI1_SHEEP, B2M, SEMA7A, MSH2, CD1E, LOC101111409, CD84, ITGAM, ITGB2, TFRC]
GO Biological Process	GO:0002703	Regulation of leukocyte mediated imn	0.0077	11	206	[W5NPK5_SHEEP, W5NRI1_SHEEP, B2M, MSH2, CD1E, LOC101111409, LOC101120001, CD84, ITGAM, ITGB2, TFRC]
GO Biological Process	GO:0002705	Positive regulation of leukocyte media	0.0077	9	126	[W5NPK5_SHEEP, W5NRI1_SHEEP, B2M, MSH2, CD1E, LOC101111409, ITGAM, ITGB2, TFRC]
GO Biological Process	GO:0002822	Regulation of adaptive immune respor	0.0077	9	145	[W5NPK5_SHEEP, W5NRI1_SHEEP, B2M, MSH2, TNFSF13B, CD1E, LOC101111409, LOC101120001, TFRC]
GO Biological Process	GO:0002824	Positive regulation of adaptive immun	0.0077	8	92	[W5NPK5_SHEEP, W5NRI1_SHEEP, B2M, MSH2, TNFSF13B, CD1E, LOC101111409, TFRC]
GO Biological Process	GO:0010810	Regulation of cell-substrate adhesion	0.0077	10	164	[PLEKHA2, ADAM15, NID1, MMP12, PLAU, KANK1, NRP1, FN1, CCDC80, CSF1]
GO Biological Process	GO:0010951	Negative regulation of endopeptidase	0.0077	11	231	[W5NPK5_SHEEP, W5NRI1_SHEEP, RARRES1, MMP9, PLAUR, FURIN, TIMP1, APLP2, BST-2B, CD44, AHSG]
GO Biological Process	GO:0030155	Regulation of cell adhesion	0.0077	20	625	[BAG4, PLEKHA2, W5NWJ9_SHEEP, ADAM15, NID1, MMP12, TNFSF13B, PLAU, PLAUR, EMILIN2, ITGB2, KANK1, NRP1,
GO Biological Process	GO:0032233	Positive regulation of actin filament bu	0.0077	6	51	[BAG4, CCDC88A, MTSS1, LIMK1, NRP1, PLEK]
GO Biological Process	GO:0042325	Regulation of phosphorylation	0.0077	26	1046	[W5NPK5_SHEEP, PSEN1, W5NRI1_SHEEP, RALBP1, BAG4, PGF, CCDC88A, SEMA7A, DIPK2A, CSF1R, PDGFC, MMP9, PKIB,
GO Biological Process	GO:0045937	Positive regulation of phosphate meta	0.0077	22	743	[W5NPK5_SHEEP, PSEN1, W5NRI1_SHEEP, RALBP1, BAG4, PGF, CCDC88A, SEMA7A, DIPK2A, CSF1R, PDGFC, MMP9,
GO Biological Process	GO:0048583	Regulation of response to stimulus	0.0077	57	3268	[W5NPK5_SHEEP, AATF, PSEN1, W5NRI1_SHEEP, BAG4, PGF, W5NWJ9_SHEEP, CCDC88A, B2M, SEMA7A, IVNS1ABP, LOC100101238, MMP12, MSH2, LAMTOR2, TNFSF13B, RASGRP4, DIPK2A, CSF1R, PDGFC, CD1E, LOC101111409, ABCA1,
GO Biological Process	GO:0048584	Positive regulation of response to stim	0.0077	36	1758	[W5NPK5_SHEEP, PSEN1, W5NRI1_SHEEP, BAG4, PGF, W5NWJ9_SHEEP, CCDC88A, B2M, SEMA7A, LOC100101238, MMP12, MSH2, LAMTOR2, TNFSF13B, RASGRP4, CSF1R, PDGFC, CD1E, LOC101111409, MMP9, C1QC, C1QA, PLAUR,
GO Biological Process	GO:0050790	Regulation of catalytic activity	0.0077	40	2016	[W5NPK5_SHEEP, PSEN1, W5NRI1_SHEEP, RALBP1, BAG4, STXBP5, RARRES1, CCDC88A, LOC100101238, MSH2, LAMTOR2, RASGRP4, DIPK2A, CSF1R, PDGFC, ASAP1, MMP9, PKIB, PLAUR, MAP2K2, LIMK1, FURIN, RNASE4, TIMP1, LGMN, PARP4,
GO Biological Process	GO:0051128	Regulation of cellular component orga	0.0077	39	1955	[W5NPK5_SHEEP, PSEN1, W5NRI1_SHEEP, RALBP1, BAG4, STXBP5, W5NWJ9_SHEEP, CCDC88A, LCAT, ADAM15, B2M, SEMA7A, LAMTOR2, W5P5W7_SHEEP, CRABP2, CSF1R, ASAP1, MMP9, PKIB, ARPC1A, FBP1, MTSS1, HSPA1A-2, PLAUR,
GO Biological Process	GO:0051336	Regulation of hydrolase activity	0.0077	23	878	[W5NPK5_SHEEP, W5NRI1_SHEEP, RALBP1, BAG4, RARRES1, LOC100101238, RASGRP4, ASAP1, MMP9, PLAUR, FURIN,
GO Biological Process	GO:0051346	Negative regulation of hydrolase activ	0.0077	13	313	[W5NPK5_SHEEP, W5NRI1_SHEEP, BAG4, RARRES1, MMP9, PLAUR, FURIN, TIMP1, APLP2, BST-2B, CD44, SORT1, AHSG]
GO Biological Process	GO:0052547	Regulation of peptidase activity	0.0077	15	412	[W5NPK5_SHEEP, W5NRI1_SHEEP, RARRES1, MMP9, PLAUR, FURIN, TIMP1, LGMN, APLP2, BST-2B, FGL2, CD44, FN1,

Supplementary Table 24: Comparative functional enrichment analysis of M1 and M2 relative to respective MØ (monocyte derived macrophage). Functional enrichment analysis was performed using the StringApp (STRING database) within Cytoscape (version 3.10.3)[1], focusing on Gene Ontology (GO) Biological Process (BP) terms[2,3].

GO Biological Process	GO:0098883	Synapse pruning	0.0077	4	12	[W5NPK5_SHEEP, W5NRI1_SHEEP, C1QC, C1QA]
GO Biological Process	GO:1902905	Positive regulation of supramolecular f	0.0077	9	144	[PSEN1, BAG4, CCDC88A, MTSS1, HSPA1A-2, LIMK1, AKAP9, NRP1, PLEK]
GO Biological Process	GO:0019220	Regulation of phosphate metabolic pro	0.0079	27	1166	[W5NPK5_SHEEP, PSEN1, W5NRI1_SHEEP, RALBP1, BAG4, PGF, CCDC88A, SEMA7A, DIPK2A, CSF1R, PDGFC, MMP9, PKIB,
GO Biological Process	GO:0051246	Regulation of protein metabolic proce	0.0079	42	2276	[W5NPK5_SHEEP, AATF, PSEN1, W5NRI1_SHEEP, RALBP1, BAG4, PGF, RARRES1, CCDC88A, SGF29, SEMA7A, IVNS1ABP, DIPK2A, CSF1R, PDGFC, MMP9, PKIB, HSPA1A-2, BZW2, PLAUR, CD84, MAP2K2, LIMK1, ITGB2, FURIN, RNASE4, TIMP1,
GO Biological Process	GO:1902903	Regulation of supramolecular fiber org	0.0099	13	339	[PSEN1, BAG4, CCDC88A, ARPC1A, MTSS1, HSPA1A-2, LIMK1, KANK1, FAM49A, AKAP9, NRP1, PLEK, GMFB]
GO Biological Process	GO:0030334	Regulation of cell migration	0.0113	20	742	[BAG4, PGF, W5NWJ9_SHEEP, ADAM15, SEMA7A, CSF1R, PDGFC, MMP9, PLAU, EMILIN2, PADI2, TIMP1, LGMN, KANK1,
GO Biological Process	GO:0044093	Positive regulation of molecular functi	0.0123	28	1281	[PSEN1, RALBP1, CCDC88A, CLIC2, B2M, LOC100101238, MSH2, RASGRP4, DIPK2A, CSF1R, PDGFC, ASAP1, MMP9, PKIB,
GO Biological Process	GO:0051130	Positive regulation of cellular compone	0.0126	22	878	[W5NPK5_SHEEP, PSEN1, W5NRI1_SHEEP, RALBP1, BAG4, CCDC88A, B2M, SEMA7A, CRABP2, ASAP1, MMP9, PKIB, MTSS1,
GO Biological Process	GO:0002460	Adaptive immune response based on s	0.0129	8	128	[W5NPK5_SHEEP, W5NRI1_SHEEP, IL18BP, B2M, MSH2, TNFSF13B, C1QC, C1QA]
GO Biological Process	GO:0002376	Immune system process	0.0135	35	1807	[W5NPK5_SHEEP, PSEN1, W5NRI1_SHEEP, W5NWJ9_SHEEP, CD1B2_SHEEP, IL18BP, ADAM15, B2M, LOC100101238, MSH2, TNFSF13B, HECTD1, PRG4, CSF1R, CD1E, LOC101111409, TAP1, MMP9, C1QC, C1QA, CD84, BRCA2, MRC1, ITGB2, ENPP1, RAF1, BST-2B, ZYX, FGL2, CD44, CSF1, PLEK, CD58, TFRC, IL1RN]
GO Biological Process	GO:0031399	Regulation of protein modification pro	0.0139	28	1298	[W5NPK5_SHEEP, PSEN1, W5NRI1_SHEEP, RALBP1, BAG4, PGF, CCDC88A, SGF29, SEMA7A, IVNS1ABP, DIPK2A, CSF1R, PDGFC, MMP9, PKIB, PLAUR, MAP2K2, LIMK1, ITGB2, RNASE4, ENPP1, RAF1, AKAP9, NRP1, CD44, FN1, CSF1, TFRC]
GO Biological Process	GO:0002688	Regulation of leukocyte chemotaxis	0.0141	7	96	[PGF, W5NWJ9_SHEEP, CSF1R, PADI2, LGMN, CAMK1D, CSF1]
GO Biological Process	GO:0045785	Positive regulation of cell adhesion	0.0173	13	367	[BAG4, PLEKHA2, NID1, TNFSF13B, PLAUR, EMILIN2, ITGB2, NRP1, CD44, FN1, CCDC80, CSF1, TFRC]
GO Biological Process	GO:0045861	Negative regulation of proteolysis	0.0173	12	317	[W5NPK5_SHEEP, PSEN1, W5NRI1_SHEEP, RARRES1, MMP9, PLAUR, FURIN, TIMP1, APLP2, BST-2B, CD44, AHSG]
GO Biological Process	GO:0050778	Positive regulation of immune respons	0.0173	14	421	[W5NPK5_SHEEP, PSEN1, W5NRI1_SHEEP, B2M, MMP12, MSH2, TNFSF13B, CD1E, LOC101111409, C1QC, C1QA, ITGAM, ITGB2, TFRC]
GO Biological Process	GO:0051239	Regulation of multicellular organismal	0.0179	39	2159	[W5NPK5_SHEEP, PSEN1, W5NRI1_SHEEP, PGF, GAA, W5NWJ9_SHEEP, SGF29, LCAT, B2M, SEMA7A, MMP12, MSH2, SRGN, CRABP2, CSF1R, PDGFC, MMP9, C1QC, PLAU, CD84, EMILIN2, LPL, BRCA2, LIMK1, FURIN, TIMP1, LGMN, ENPP1, AKAP9, NRP1, FGL2, MAN2A1, FN1, CSF1, AAMP, CD58, TFRC, AHSG, GATM]
GO Biological Process	GO:0052548	Regulation of endopeptidase activity	0.0191	13	374	[W5NPK5_SHEEP, W5NRI1_SHEEP, RARRES1, MMP9, PLAUR, FURIN, TIMP1, LGMN, APLP2, BST-2B, CD44, AHSG, RPS27L]
GO Biological Process	GO:0071840	Cellular component organization or bi	0.0196	75	5301	[W5NPK5_SHEEP, AATF, WIPF1, PSEN1, W5NRI1_SHEEP, BAG4, PSMG4, HAT1, TPM3, GAA, W5NWJ9_SHEEP, CCDC88A, LCAT, TOMM20, SYNE1, NID1, B2M, SEMA7A, BMS1, MMP12, MSH2, SRGN, W5P5W7_SHEEP, MLKL, CRABP2, CSF1R, NOP56, PDGFC, ABCA1, EML4, ASAP1, STAG1, MMP9, C1QC, C1QA, ARPC1A, ASF1A, MTSS1, GOLM1, MYH11, SMYD2, LPL, PADI2, BRCA2, MFGE8, SMYD5, LIMK1, CISD2, FLNB, LGMN, KANK1, APLP2, TGFBI, AAR2, RAF1, CLPTM1L, FAM49A, TPP1, AKAP9, MTX2, NRP1, NOP58, PIN4, ZYX, DNMT3A, MAN2A1, SORT1, FN1, CCDC80, MREG, FGD4, MAP7D1, PLEK, PHGDH, RPS27L]
GO Biological Process	GO:0002682	Regulation of immune system process	0.0205	26	1202	[W5NPK5_SHEEP, PSEN1, W5NRI1_SHEEP, PGF, W5NWJ9_SHEEP, B2M, SEMA7A, MMP12, MSH2, TNFSF13B, CSF1R, CD1E, LOC101111409, LOC101120001, C1QC, C1QA, CD84, ITGAM, PADI2, ITGB2, LGMN, CAMK1D, FGL2, FN1, CSF1, TFRC]
GO Biological Process	GO:0150064	Vertebrate eye-specific patterning	0.0205	3	7	[W5NPK5_SHEEP, W5NRI1_SHEEP, C1QA]
GO Biological Process	GO:0002708	Positive regulation of lymphocyte med	0.0219	7	107	[W5NPK5_SHEEP, W5NRI1_SHEEP, B2M, MSH2, CD1E, LOC101111409, TFRC]
GO Biological Process	GO:0002888	Positive regulation of myeloid leukocy	0.0234	4	23	[W5NPK5_SHEEP, W5NRI1_SHEEP, ITGAM, ITGB2]
GO Biological Process	GO:0002250	Adaptive immune response	0.0242	10	236	[W5NPK5_SHEEP, W5NRI1_SHEEP, CD1B2_SHEEP, IL18BP, B2M, MSH2, TNFSF13B, TAP1, C1QC, C1QA]
GO Biological Process	GO:0150062	Complement-mediated synapse pruni	0.0256	3	8	[W5NPK5_SHEEP, W5NRI1_SHEEP, C1QA]
GO Biological Process	GO:0051247	Positive regulation of protein metabol	0.0264	26	1230	[W5NPK5_SHEEP, PSEN1, W5NRI1_SHEEP, RALBP1, BAG4, PGF, CCDC88A, SEMA7A, DIPK2A, CSF1R, PDGFC, MMP9, HSPA1A-2, PLAUR, MAP2K2, RNASE4, LGMN, RAF1, AKAP9, NRP1, FGL2, CD44, FN1, CSF1, TFRC, RPS27L]
GO Biological Process	GO:0010033	Response to organic substance	0.0276	36	1990	[PSEN1, BAG4, PGF, W5NWJ9_SHEEP, ADAM15, B2M, LOC100101238, LAMTOR2, TNFSF13B, CSF1R, PDGFC, CSF2RA, MMP9, MAN1A1, ASAH1, HSPA1A-2, MYH11, LPL, PADI2, MRC1, ITGB2, RNASE4, LGMN, ENPP1, IFIT3, RAF1, AKAP9, NRP1, ZYX, DNMT3A, CD44, SORT1, CSF1, CD58, TFRC, IL1RN]
GO Biological Process	GO:0032970	Regulation of actin filament-based pro	0.0279	12	344	[BAG4, CCDC88A, CSF1R, ARPC1A, MTSS1, LIMK1, KANK1, FAM49A, AKAP9, NRP1, PLEK, GMFB]
GO Biological Process	GO:0035295	Tube development	0.0287	18	698	[W5NPK5_SHEEP, PSEN1, W5NRI1_SHEEP, PGF, MMP12, HECTD1, PDGFC, MTSS1, MFGE8, RNASE4, TGFBI, NRP1, MAN2A1, FN1, CSF1, AAMP, MAN1A2, PHGDH]
GO Biological Process	GO:0051651	Maintenance of location in cell	0.0294	6	80	[PSEN1, GAA, CCDC88A, SYNE1, ALB, AKAP9]
GO Biological Process	GO:0002706	Regulation of lymphocyte mediated im	0.0298	8	156	[W5NPK5_SHEEP, W5NRI1_SHEEP, B2M, MSH2, CD1E, LOC101111409, LOC101120001, TFRC]
GO Biological Process	GO:0010638	Positive regulation of organelle organi	0.0304	14	461	[RALBP1, BAG4, CCDC88A, ASAP1, MMP9, PKIB, MTSS1, HSPA1A-2, PLAUR, LIMK1, FEN1, AKAP9, NRP1, PLEK]
GO Biological Process	GO:0110053	Regulation of actin filament organizati	0.0304	10	248	[BAG4, CCDC88A, ARPC1A, MTSS1, LIMK1, KANK1, FAM49A, NRP1, PLEK, GMFB]
GO Biological Process	GO:0001952	Regulation of cell-matrix adhesion	0.0314	6	82	[PLEKHA2, ADAM15, MMP12, PLAU, NRP1, CSF1]

Supplementary Table 24: Comparative functional enrichment analysis of M1 and M2 relative to respective MØ (monocyte derived macrophage). Functional enrichment analysis was performed using the StringApp (STRING database) within Cytoscape (version 3.10.3)[1], focusing on Gene Ontology (GO) Biological Process (BP) terms[2,3].

GO Biological Process	GO:0048518	Positive regulation of biological proces	0.0314	75	5423	[W5NPK5_SHEEP, AATF, PSEN1, W5NRI1_SHEEP, RALBP1, OXSR1, BAG4, PGF, PLEKHA2, W5NWB0_SHEEP, STXBP5, W5NWJ9_SHEEP, CCDC88A, NID1, CLIC2, B2M, SEMA7A, LOC100101238, MMP12, MSH2, LAMTOR2, W5P5W7_SHEEP, TNFSF13B, RASGRP4, DIPK2A, CRABP2, CSF1R, PDGFC, CD1E, LOC101111409, ASAP1, MMP9, C1QC, C1QA, PKIB, PLAU, MTSS1, HSPA1A-2, PLAUR, CD84, ITGAM, HDGFL2, EMILIN2, LPL, MAP2K2, PADI2, BRCA2, MFGE8, LIMK1, TLE1, ITGB2, FURIN, RNASE4, TIMP1, LGMN, KANK1, CAMK1D, FEN1, RAF1, AKAP9, NRP1, W5Q9Y6_SHEEP, FGL2, CD44, MAN2A1, FN1, CCDC80, CSF1, AAMP, PLEK, CD58, TFRC, AHSG, RPS27L, GATM]
GO Biological Process	GO:0022603	Regulation of anatomical structure mo	0.0332	18	713	[W5NPK5_SHEEP, PSEN1, W5NRI1_SHEEP, RALBP1, PGF, SEMA7A, TNFSF13B, CRABP2, CSF1R, EMILIN2, LIMK1, KANK1, NRP1, CD44, FN1, CSF1, AAMP, TFRC]
GO Biological Process	GO:0043254	Regulation of protein-containing comp	0.0339	12	357	[BAG4, STXBP5, LCAT, ASAP1, ARPC1A, HSPA1A-2, KANK1, RAF1, FAM49A, AKAP9, PLEK, TFRC]
GO Biological Process	GO:0002886	Regulation of myeloid leukocyte media	0.0345	5	53	[W5NPK5_SHEEP, W5NRI1_SHEEP, CD84, ITGAM, ITGB2]
GO Biological Process	GO:0030335	Positive regulation of cell migration	0.0367	13	417	[BAG4, PGF, W5NWJ9_SHEEP, SEMA7A, CSF1R, PDGFC, MMP9, PLAU, LGMN, NRP1, FN1, CSF1, AAMP]
GO Biological Process	GO:0032956	Regulation of actin cytoskeleton orgar	0.043	11	315	[BAG4, CCDC88A, CSF1R, ARPC1A, MTSS1, LIMK1, KANK1, FAM49A, NRP1, PLEK, GMFB]
GO Biological Process	GO:0051495	Positive regulation of cytoskeleton org	0.0444	8	170	[BAG4, CCDC88A, MTSS1, HSPA1A-2, LIMK1, AKAP9, NRP1, PLEK]
GO Biological Process	GO:0006950	Response to stress	0.045	43	2640	[W5NPK5_SHEEP, AATF, PSEN1, W5NRI1_SHEEP, PGF, TREML1, GP6, MSRB3, ADAM15, LOC100101238, MMP12, MSH2, MLKL, CSF1R, ABCA1, TAP1, MMP9, C1QA, MAN1A1, PLAU, ASF1A, MTSS1, HSPA1A-2, BRCA2, MRC1, RNASE4, TIMP1, ALB, PARP4, IFIT3, FEN1, BST-2B, NRP1, PLOD1, ZYX, CD44, FN1, CSF1, PLEK, CD58, AHSG, RPS27L, IL1RN]
GO Biological Process	GO:0030162	Regulation of proteolysis	0.0453	17	674	[W5NPK5_SHEEP, PSEN1, W5NRI1_SHEEP, RARRES1, MMP9, HSPA1A-2, PLAUR, FURIN, TIMP1, LGMN, APLP2, BST-2B, FGL2, CD44, FN1, AHSG, RPS27L]
GO Biological Process	GO:0010628	Positive regulation of gene expression	0.0455	21	942	[W5NPK5_SHEEP, PSEN1, W5NRI1_SHEEP, W5NWB0_SHEEP, B2M, SEMA7A, MMP12, MSH2, W5P5W7_SHEEP, CSF1R, CD84, LPL, MAP2K2, PADI2, TLE1, FURIN, FN1, CSF1, CD58, TFRC, RPS27L]
GO Biological Process	GO:0016043	Cellular component organization	0.0493	70	5055	[W5NPK5_SHEEP, WIPF1, PSEN1, W5NRI1_SHEEP, BAG4, PSMG4, HAT1, TPM3, GAA, W5NWJ9_SHEEP, CCDC88A, LCAT, TOMM20, SYNE1, NID1, B2M, SEMA7A, MMP12, MSH2, SRGN, W5P5W7_SHEEP, MLKL, CRABP2, CSF1R, PDGFC, ABCA1, EML4, ASAP1, STAG1, MMP9, C1QC, C1QA, ARPC1A, ASF1A, MTSS1, GOLM1, MYH11, SMYD2, LPL, PADI2, BRCA2, MFGE8, SMYD5, LIMK1, CISD2, FLNB, LGMN, KANK1, APLP2, TGFBI, AAR2, RAF1, CLPTM1L, FAM49A, TPP1, AKAP9, MTX2, NRP1, ZYX, DNMT3A, MAN2A1, SORT1, FN1, CCDC80, MREG, FGD4, MAP7D1, PLEK, PHGDH, RPS27L]

Red-highlighted cells indicate Gene Ontology Biological Process terms shared between M1 and M2 compared to MØ, whereas non-highlighted cells represent subtype-specific enrichment. M1, pro-inflammatory macrophages activated with GM-CSF/ LPS/ INF-γ and M2, anti-inflammatory macrophages activated with M-CSF/ IL-4.

Supplementary Table 25: Enrichment map analysis of differentially abundant proteins (DAPs) in M1 compared to GMØ, presenting significantly enriched Gene Ontology biological process (GO-BP) terms and canonical pathways. unctional enrichment analysis was performed using the StringApp (STRING database) within Cytoscape (version 3.10.3) [1], focusing on Gene Ontology (GO) Biological Process (BP) terms and canonical pathways from the KEGG and Reactome databases [2–6]. Enriched GO terms were clustered as functionally coherent groups using the EnrichmentMap plugin [7]. . Statistical significance was determined based on a false discovery rate (FDR) threshold of < 0.05. M1, pro-inflammatory macrophages activated with GM-CSF/ LPS/ INF-γ and GMØ, monocyte-derived macrophages differentiated with GM-CSF (Granulocyte-Macrophage Colony-Stimulating Factor).

EnrichmentMap analysis of M1_GMØ_ Biological Process

Annotation	Nodes
Defense response external	6
Collagen process catabolic	2
Molecular function catalytic	2
Negative regulation immune	2
Regulation transport localization	2

name	EnrichmentMap GS_DESCR	EnrichmentMap Genes	EnrichmentMap gs_size	EnrichmentMap -log10 (pvalue	EnrichmentMap: pvalue (Data set 1)	EnrichmentMap::fdr_qval	EnrichmentMap::NES (Data Set	EnrichmentMap::-log10 (pvalue	EnrichmentMap::-lo	EnrichmentMap cc	_mdcluster
GO:0098542	Defense response to other organism	S100A8STAB1CLEC4EPTX3W5P026_SHE	18	4.754487332	1.76E-05	1.99E-02	0.355896525	4.754487332	4.754487332	0.9999824	1
GO:0009617	Response to bacterium	S100A8STAB1CLEC4EW5P026_SHEEPLC	16	5.754487332	1.76E-06	6.60E-03	0.456172672	5.754487332	5.754487332	0.99999824	1
GO:0009607	Response to biotic stimulus	S100A8STAB1CLEC4EPTX3W5P026_SHE	25	6.235823868	5.81E-07	6.60E-03	0.456172672	6.235823868	6.235823868	0.999999419	1
GO:0051707	Response to other organism	S100A8STAB1CLEC4EPTX3W5P026_SHE	24	6.067019178	8.57E-07	6.60E-03	0.456172672	6.067019178	6.067019178	0.999999143	1
GO:0009605	Response to external stimulus	S100A8STAB1EMBCLEC4ECOL11A1PTX3	30	4.505845406	3.12E-05	3.14E-02	0.314456975	4.505845406	4.505845406	0.9999688	1
GO:0044419	Biological process involved in interspe	S100A8STAB1CLEC4EPTX3W5P026_SHE	25	5.651695137	2.23E-06	6.60E-03	0.456172672	5.651695137	5.651695137	0.99999777	1
GO:0030574	Collagen catabolic process	MMP12MMP3MMP1MMP9MMP2	5	5.106793247	7.82E-06	1.11E-02	0.408937494	5.106793247	5.106793247	0.99999218	2
GO:0032963	Collagen metabolic process	MMP12COL1A1MMP3MMP1MMP9MN	6	5.40560745	3.93E-06	6.60E-03	0.456172672	5.40560745	5.40560745	0.99999607	2
GO:0065009	Regulation of molecular function	S100A8PTX3LOC101114535ERBINLOC1	41	5.705533774	1.97E-06	0.0066	0.456172672	5.705533774	5.705533774	0.99999803	4
GO:0050790	Regulation of catalytic activity	S100A8PTX3LOC101114535LOC101117	33	5.031050319	9.31E-06	1.17E-02	0.404154356	5.031050319	5.031050319	0.99999069	4
GO:0050777	Negative regulation of immune respo	IL4I1W5PVN6_SHEEPSAMSN1MMP12A	7	4.510041521	3.09E-05	3.14E-02	0.314456975	4.510041521	4.510041521	0.9999691	3
GO:0002683	Negative regulation of immune syster	CD274IL4I1W5PVN6_SHEEPSAMSN1LO	11	4.517126416	3.04E-05	3.14E-02	0.314456975	4.517126416	4.517126416	0.9999696	3
GO:0051049	Regulation of transport	S100A8HSPA2CASKPTX3UBAC2LOC101	24	4.316952962	4.82E-05	3.22E-02	0.312171103	4.316952962	4.316952962	0.9999518	5
GO:0032879	Regulation of localization	S100A8CASKPTX3UBAC2LOC10111453	28	4.454692884	3.51E-05	3.14E-02	0.314456975	4.454692884	4.454692884	0.9999649	5

Pathway analysis of M1_GMØ

KEGG pathways

term description	#term ID	observed gene count	background gene count	strength	signal	false discovery rate	matching proteins in your network (IDs)	matching proteins in your
**IL-17 signaling pathway**	oas04657	5	73	1.21	0.62	0.0063	9940.ENSOARP00000000449,9940.ENSOARP00000005	S100A8,MMP3,MMP1,MMP9,IL1B
**Metabolic pathways**	oas01100	15	1263	0.45	0.31	0.0417	9940.ENSOARP00000004297,9940.ENSOARP00000005	LOC101113086,LOC101112162,SDS,MAN1A1,PGK2,UAP1,XDH,ACSL5,CA2,ENPP1,UPP1,ACSL4,TST,CA12,ARG2
**Peroxisome**	oas04146	4	69	1.14	0.4	0.0417	9940.ENSOARP00000004840,9940.ENSOARP00000011	SOD2,XDH,ACSL5,ACSL4
**Ferroptosis**	oas04216	3	38	1.27	0.4	0.0417	9940.ENSOARP00000009144,9940.ENSOARP00000012	LOC101117129,ACSL5,ACSL4
**C-type lectin receptor signaling pathway**	oas04625	4	79	1.08	0.39	0.0417	9940.ENSOARP00000000488,9940.ENSOARP00000002	NFKB2,CLEC4E,RRAS2,IL1B
**TNF signaling pathway**	oas04668	4	90	1.02	0.39	0.0417	9940.ENSOARP00000005456,9940.ENSOARP00000008	MMP3,MMP9,FAS,IL1B
**Rheumatoid arthritis**	oas05323	4	87	1.04	0.39	0.0417	9940.ENSOARP00000004675,9940.ENSOARP00000005	LOC101114535,MMP3,MMP1,IL1B

Supplementary Table 26: Enrichment map analysis of differentially abundant proteins (DAPs) in M2 compared to MMØ, presenting significantly enriched Gene Ontology biological process (GO-BP) terms and canonical pathways. Functional enrichment analysis was performed using the StringApp (STRING database) within Cytoscape (version 3.10.3) [1], focusing on Gene Ontology (GO) Biological Process (BP) terms and canonical pathways from the KEGG and Reactome databases [2–6]. Enriched GO terms were clustered as functionally coherent groups using the EnrichmentMap plugin [7]. Statistical significance was determined based on a false discovery rate (FDR) threshold of < 0.05. M2, anti-inflammatory macrophages activated with M-CSF/ IL-4 and MMØ, monocyte-derived macrophages differentiated with M-CSF (Macrophage Colony-Stimulating Factor).

EnrichmentMap analysis of M2_MMØ_ Biological Process

Annotation	Nodes	EnrichmentMap name	EnrichmentMap GS_DESCR	EnrichmentMap Genes	EnrichmentMap gs_size	EnrichmentMap -log1	EnrichmentMap: pvalue (Data set 1	EnrichmentMap::fdr_qvalue (D	EnrichmentMap::NES (Data S	EnrichmentMap::-log10 (pval	EnrichmentMap::-log10 (pval	EnrichmentMap colouring (Data Set 1)	_mdcluster
protein process metabolic	13	GO:0044093	Positive regulation of molecular function	FN1PLEKPSEN1RALBP1CCD	28	4.385102784	4.12E-05	1.23E-02	0.216818026	4.385102784	4.385102784	0.9999588	1
		GO:0051246	Regulation of protein metabolic process	CD84SEMA7AITGB2NRP1FN	42	4.640164518	2.29E-05	7.90E-03	0.238643821	4.640164518	4.640164518	0.9999771	1
		GO:0001934	Positive regulation of protein phosphorylation	SEMA7ANRP1W5NPK5_SHE	21	6.259637311	5.50E-07	3.10E-03	0.284759678	6.259637311	6.259637311	0.99999945	1
		GO:0042325	Regulation of phosphorylation	SEMA7AITGB2NRP1FN1FBP	26	5.023191663	9.48E-06	7.70E-03	0.239907929	5.023191663	5.023191663	0.99999052	1
		GO:0045937	Positive regulation of phosphate metabolic process	SEMA7ANRP1PLEKW5NPK5	22	5.455931956	3.50E-06	7.70E-03	0.239907929	5.455931956	5.455931956	0.9999965	1
		GO:0001932	Regulation of protein phosphorylation	SEMA7AITGB2NRP1FN1W5	25	5.543633967	2.86E-06	7.70E-03	0.239907929	5.543633967	5.543633967	0.99999714	1
		GO:0065009	Regulation of molecular function	ADAM15NRP1FN1PLEKW5	50	5.876148359	1.33E-06	5.00E-03	0.261193715	5.876148359	5.876148359	0.99999867	1
		GO:0051128	Regulation of cellular component organization	ADAM15SEMA7AKANK1NR	39	5.073143291	8.45E-06	7.70E-03	0.239907929	5.073143291	5.073143291	0.99999155	1
		GO:0019220	Regulation of phosphate metabolic process	SEMA7AITGB2NRP1FN1FBP	27	4.665546249	2.16E-05	7.90E-03	0.238643821	4.665546249	4.665546249	0.9999784	1
		GO:0051130	Positive regulation of cellular component organization	B2MSEMA7AASAP1NRP1PK	22	4.364516253	4.32E-05	1.26E-02	0.215630077	4.364516253	4.364516253	0.9999568	1
		GO:0050790	Regulation of catalytic activity	NRP1FN1PLEKW5NPK5_SHI	40	5.145086978	7.16E-06	7.70E-03	0.239907929	5.145086978	5.145086978	0.99999284	1
		GO:0051247	Positive regulation of protein metabolic process	SEMA7ANRP1FN1W5NPK5	26	3.886056648	1.30E-04	2.64E-02	0.179166345	3.886056648	3.886056648	0.99987	1
		GO:0031399	Regulation of protein modification process	SEMA7AITGB2NRP1FN1W5	28	4.287350298	5.16E-05	1.39E-02	0.210789457	4.287350298	4.287350298	0.9999484	1
regulation of immune system process	12	GO:0002376	Immune system process	C1QAADAM15CD84ITGB2Z	35	4.313363731	4.86E-05	1.35E-02	0.212228902	4.313363731	4.313363731	0.9999514	2
		GO:0002705	Positive regulation of leukocyte mediated immunity	B2MMSH2ITGAMITGB2W5	9	5.260427656	5.49E-06	7.70E-03	0.239907929	5.260427656	5.260427656	0.99999451	2
		GO:0002822	Regulation of adaptive immune response based on somatic recombination	B2MLOC101120001MSH2V	9	4.798602876	1.59E-05	7.70E-03	0.239907929	4.798602876	4.798602876	0.9999841	2
		GO:0002708	Positive regulation of lymphocyte mediated immunity	B2MMSH2W5NPK5_SHEEP	7	4	1.00E-04	2.19E-02	0.18837893	4	4	0.9999	2
		GO:0002824	Positive regulation of adaptive immune response based on somatic recomb	B2MMSH2W5NPK5_SHEEP	8	5.319664487	4.79E-06	7.70E-03	0.239907929	5.319664487	5.319664487	0.99999521	2
		GO:0002706	Regulation of lymphocyte mediated immunity	B2MLOC101120001MSH2V	8	3.769551079	1.70E-04	2.98E-02	0.173194232	3.769551079	3.769551079	0.99983	2
		GO:0002682	Regulation of immune system process	C1QACD84SEMA7ALOC101	26	4.027334408	9.39E-05	2.05E-02	0.191635608	4.027334408	4.027334408	0.9999061	2
		GO:0050778	Positive regulation of immune response	C1QAB2MMSH2ITGAMITGE	14	4.15864053	6.94E-05	1.73E-02	0.200002297	4.15864053	4.15864053	0.9999306	2
		GO:0002684	Positive regulation of immune system process	C1QAB2MCD84SEMA7AMS	21	5.007888512	9.82E-06	7.70E-03	0.239907929	5.007888512	5.007888512	0.99999018	2
		GO:0002699	Positive regulation of immune effector process	B2MCD84SEMA7AMSH2ITG	11	5.055517328	8.80E-06	7.70E-03	0.239907929	5.055517328	5.055517328	0.9999912	2
		GO:0002703	Regulation of leukocyte mediated immunity	B2MCD84LOC101120001M	11	5.149353765	7.09E-06	7.70E-03	0.239907929	5.149353765	5.149353765	0.99999291	2
		GO:0002697	Regulation of immune effector process	B2MCD84SEMA7ALOC1011	13	5.040005162	9.12E-06	7.70E-03	0.239907929	5.040005162	5.040005162	0.99999088	2
organization actin cytoskeleton	8	GO:0010638	Positive regulation of organelle organization	ASAP1NRP1PKIBMTSS1LIM	14	3.769551079	1.70E-04	3.04E-02	0.172211525	3.769551079	3.769551079	0.99983	3
		GO:0032956	Regulation of actin cytoskeleton organization	CCDC88ACSF1RARPC1AKAN	11	3.552841969	2.80E-04	4.30E-02	0.155117253	3.552841969	3.552841969	0.99972	3
		GO:0032233	Positive regulation of actin filament bundle assembly	CCDC88ANRP1MTSS1LIMK	6	4.801342913	1.58E-05	7.70E-03	0.239907929	4.801342913	4.801342913	0.9999842	3
		GO:0051495	Positive regulation of cytoskeleton organization	CCDC88AHSPA1A-2NRP1M	8	3.537602002	2.90E-04	4.44E-02	0.153537793	3.537602002	3.537602002	0.99971	3
		GO:0110053	Regulation of actin filament organization	CCDC88AARPC1AKANK1NR	10	3.744727495	1.80E-04	3.04E-02	0.172211525	3.744727495	3.744727495	0.99982	3
		GO:1902903	Regulation of supramolecular fiber organization	KANK1NRP1MTSS1LIMK1P	13	4.503070352	3.14E-05	9.90E-03	0.22751876	4.503070352	4.503070352	0.9999686	3
		GO:0032970	Regulation of actin filament-based process	CCDC88ACSF1RKANK1ARP	12	3.823908741	1.50E-04	2.79E-02	0.176442034	3.823908741	3.823908741	0.99985	3
		GO:1902905	Positive regulation of supramolecular fiber organization	CCDC88AHSPA1A-2NRP1M	9	4.821023053	1.51E-05	7.70E-03	0.239907929	4.821023053	4.821023053	0.9999849	3
negative activity proteolysis	7	GO:0051346	Negative regulation of hydrolase activity	W5NPK5_SHEEPFURINTIM	13	4.850780887	1.41E-05	7.70E-03	0.239907929	4.850780887	4.850780887	0.9999859	4
		GO:0010951	Negative regulation of endopeptidase activity	BST-2BMMP9PLAURAHSGW	11	4.705533774	1.97E-05	7.70E-03	0.239907929	4.705533774	4.705533774	0.9999803	4
		GO:0052547	Regulation of peptidase activity	FN1W5NPK5_SHEEPFURIN	15	4.860120914	1.38E-05	7.70E-03	0.239907929	4.860120914	4.860120914	0.9999862	4
		GO:0051336	Regulation of hydrolase activity	LOC100101238RASGRP4AS	23	4.838631998	1.45E-05	7.70E-03	0.239907929	4.838631998	4.838631998	0.9999855	4
		GO:0030162	Regulation of proteolysis	FN1W5NPK5_SHEEPFURIN	17	3.508638306	3.10E-04	4.53E-02	0.152548513	3.508638306	3.508638306	0.99969	4
		GO:0052548	Regulation of endopeptidase activity	W5NPK5_SHEEPFURINTIM	13	4.084072788	8.24E-05	0.0191	0.195122742	4.084072788	4.084072788	0.9999176	4
		GO:0045861	Negative regulation of proteolysis	BST-2BMMP9PLAURAHSGW	12	4.146301788	7.14E-05	1.73E-02	0.200002297	4.146301788	4.146301788	0.9999286	4
synapse pruning myeloid	5	GO:0150064	Vertebrate eye-specific patterning	C1QAW5NPK5_SHEEPW5N	3	4.025949097	9.42E-05	0.0205	0.191635608	4.025949097	4.025949097	0.9999058	5
		GO:0150062	Complement-mediated synapse pruning	C1QAW5NPK5_SHEEPW5N	3	3.886056648	1.30E-04	0.0256	0.180683311	3.886056648	3.886056648	0.99987	5
		GO:0098883	Synapse pruning	C1QAW5NPK5_SHEEPW5N	4	4.896196279	1.27E-05	7.70E-03	0.239907929	4.896196279	4.896196279	0.9999873	5
		GO:0002888	Positive regulation of myeloid leukocyte mediated immunity	ITGAMITGB2W5NPK5_SHE	4	3.958607315	1.10E-04	0.0234	0.185113002	3.958607315	3.958607315	0.99989	5
		GO:0002886	Regulation of myeloid leukocyte mediated immunity	CD84ITGAMITGB2W5NPK5	5	3.657577319	2.20E-04	3.45E-02	0.16597457	3.657577319	3.657577319	0.99978	5
multicellular organismal stimulus	4	GO:0051239	Regulation of multicellular organismal process	CD84SEMA7ANRP1EMILIN	39	4.122053048	7.55E-05	1.79E-02	0.198321538	4.122053048	4.122053048	0.9999245	6
		GO:0048583	Regulation of response to stimulus	SEMA7AITGAMITGB2NRP1	57	5.565431096	2.72E-06	0.0077	0.239907929	5.565431096	5.565431096	0.99999728	6
		GO:0048584	Positive regulation of response to stimulus	C1QASEMA7AITGAMITGB2	36	4.93930216	1.15E-05	7.70E-03	0.239907929	4.93930216	4.93930216	0.9999885	6
		GO:0048518	Positive regulation of biological process	SEMA7AITGAMITGB2NRP1	75	3.721246399	1.90E-04	0.0314	0.170615998	3.721246399	3.721246399	0.99981	6
cellular component biogenesis	2	GO:0071840	Cellular component organization or biogenesis	MTX2NOP58SEMA7APIN4	75	4.055517328	8.80E-05	0.0196	0.193848834	4.055517328	4.055517328	0.999912	10
		GO:0016043	Cellular component organization	MTX2SEMA7ADNMT3AMR	70	3.468521083	3.40E-04	0.0493	0.148377105	3.468521083	3.468521083	0.99966	10
positive cell adhesion	2	GO:0030155	Regulation of cell adhesion	ADAM15PLEKHA2NID1ITG	20	5.484126156	3.28E-06	7.70E-03	0.239907929	5.484126156	5.484126156	0.99999672	7
		GO:0045785	Positive regulation of cell adhesion	PLEKHA2NID1ITGB2NRP1C	13	4.163675884	6.86E-05	1.73E-02	0.200002297	4.163675884	4.163675884	0.9999314	7
positive cell migration	2	GO:0030335	Positive regulation of cell migration	SEMA7ANRP1PLAUFN1LGN	13	3.638272164	2.30E-04	3.67E-02	0.162927126	3.638272164	3.638272164	0.99977	9
		GO:0030334	Regulation of cell migration	ADAM15SEMA7AKANK1NR	20	4.434152181	3.68E-05	1.13E-02	0.220998282	4.434152181	4.434152181	0.9999632	9
somatic immune adaptive	2	GO:0002460	Adaptive immune response based on somatic recombination of immune re	C1QAB2MMSH2W5NPK5_S	8	4.342944147	4.54E-05	1.29E-02	0.214470083	4.342944147	4.342944147	0.9999546	8
		GO:0002250	Adaptive immune response	C1QAB2MTAP1MSH2W5NP	10	3.920818754	1.20E-04	2.42E-02	0.183455787	3.920818754	3.920818754	0.99988	8
substrate adhesion matrix	2	GO:0001952	Regulation of cell-matrix adhesion	ADAM15PLEKHA2NRP1PLA	6	3.721246399	1.90E-04	0.0314	0.170615998	3.721246399	3.721246399	0.99981	11
		GO:0010810	Regulation of cell-substrate adhesion	ADAM15PLEKHA2NID1KAN	10	5.2069084	6.21E-06	7.70E-03	0.239907929	5.2069084	5.2069084	0.99999379	11

EnrichmentMap analysis of M2_MMØ_ pathways

KEGG pathways

term description	#term ID	observed gene count	background gene count	strength	signal	false discovery rate	matching proteins in your network (
Hematopoietic cell lineage	oas04640	9	85	1.05	0.97	0.0000829	CD1B2_SHEEP.CSF1R.CD1E.LOC101111409.CSF2RA.ITGAM.CD44.CSF1.TFRC
Lysosome	oas04142	8	111	0.88	0.61	0.0028	GAA.IGF2R.ASAH1.MANBA.LGMN.GLB1.TPP1.SORT1
Regulation of actin cytoskeleton	oas04810	8	157	0.73	0.42	0.0188	PDGFC.ARPC1A.ITGAM.MAP2K2.LIMK1.PIP5K1B.RAF1.FN1
Proteoglycans in cancer	oas05205	8	160	0.72	0.42	0.0188	MMP9.PLAU.PLAUR.MAP2K2.FLNB.RAF1.CD44.FN1
MicroRNAs in cancer	oas05206	7	131	0.75	0.41	0.0219	MMP9.PLAU.ZEB2.MAP2K2.RAF1.DNMT3A.CD44
Complement and coagulation cascades	oas04610	5	63	0.92	0.41	0.0246	W5NRI1_SHEEP.C1QA.PLAU.PLAUR.ITGAM
Rap1 signaling pathway	oas04015	7	150	0.69	0.37	0.0296	PGF.CSF1R.PDGFC.ITGAM.MAP2K2.RAF1.CSF1
Pathways in cancer	oas05200	12	407	0.49	0.34	0.0296	RALBP1.PGF.TPM3.MSH2.CSF1R.CSF2RA.EML4.MMP9.MAP2K2.BRCA2.RAF1.FN1
Cholesterol metabolism	oas04979	4	42	1	0.39	0.0326	LCAT.ABCA1.LPL.SORT1
Prostate cancer	oas05215	5	74	0.85	0.38	0.0326	PDGFC.MMP9.PLAU.MAP2K2.RAF1
MAPK signaling pathway	oas04010	8	223	0.58	0.32	0.047	PGF.CSF1R.PDGFC.HSPA1A-2.MAP2K2.FLNB.RAF1.CSF1
Ras signaling pathway	oas04014	7	179	0.62	0.32	0.0487	RALBP1.PGF.CSF1R.PDGFC.MAP2K2.RAF1.CSF1
Amoebiasis	oas05146	5	89	0.77	0.33	0.0487	CD1B2_SHEEP.CD1E.LOC101111409.ITGAM.FN1
Acute myeloid leukemia	oas05221	4	51	0.92	0.34	0.0487	CSF1R.ITGAM.MAP2K2.RAF1

Reactome Pathways

term description	#term ID	observed gene count	background gene count	strength	signal	false discovery rate	matching proteins in your network (
Neutrophil degranulation	MAP-6798695	29	847	0.56	0.85	0.00000589	W5NPK5_SHEEP.PSEN1.W5NRI1_SHEEP.GAA.W5NWJ9_SHEEP.CANT1.B2M.W5P2F2_SHEEP.IGF2R.LAMTOR2.MMP9.PLAU.ASAH1.HSPA1A-2.PLAUR.ITGAM.ITGAX.PADI2.MANBA.ITGB2.TCN1.W5PYX0_SHEEP.GLB1.BST-2B.FGL2.CD44.CD58.AHSG.GMFB
Innate Immune System	MAP-168249	38	1588	0.4	0.62	0.00011	W5NPK5_SHEEP.WIPF1.PSEN1.W5NRI1_SHEEP.GAA.W5NWJ9_SHEEP.CANT1.B2M.W5P2F2_SHEEP.IGF2R.LAMTOR2.LOC101120001.MMP9.C1QC.C1QA.ARPC1A.PLAU.ASAH1.HSPA1A-2.PLAUR.ITGAM.ITGAX.PADI2.LIMK1.MANBA.ITGB2.TCN1.LGMN.W5PYX0_SHEEP.GLB1.RAF1.BST-2B.FGL2.CD44.AAMP.CD58.AHSG.GMFB
Immune System	MAP-168256	55	2924	0.3	0.53	0.0002	W5NPK5_SHEEP.WIPF1.PSEN1.W5NRI1_SHEEP.TREML1.GAA.W5NWJ9_SHEEP.CANT1.IL18BP.B2M.W5P2F2_SHEEP.IVNS1ABP.IGF2R.LAMTOR2.TNFSF13B.HECTD1.CSF1R.CD1E.LOC101111409.CSF2RA.TAP1.LOC101120001.MMP9.C1QC.C1QA.ARPC1A.PLAU.ASAH1.HSPA1A-2.PLAUR.ITGAM.ITGAX.ZEB2.PADI2.LIMK1.MRC1.MANBA.ITGB2.TCN1.FLNB.LGMN.W5PYX0_SHEEP.LTN1.IFIT3.GLB1.RAF1.BST-2B.FGL2.CD44.CSF1.AAMP.CD58.AHSG.IL1RN.GMFB
Post-translational protein phosphorylation	MAP-8957275	11	157	0.87	0.81	0.00024	W5NPK5_SHEEP.W5NRI1_SHEEP.W5NWJ9_SHEEP.GOLM1.MFGE8.TIMP1.ALB.APLP2.FN1.CSF1.AHSG
Regulation of Insulin-like Growth Factor (IGF) transport and uptake by Insulin-like Grow	MAP-381426	11	164	0.85	0.79	0.00029	W5NPK5_SHEEP.W5NRI1_SHEEP.W5NWJ9_SHEEP.GOLM1.MFGE8.TIMP1.ALB.APLP2.FN1.CSF1.AHSG
Extracellular matrix organization	MAP-1474244	15	358	0.65	0.65	0.00073	W5NWJ9_SHEEP.ADAM15.NID1.MMP12.PRG4.MMP9.ITGAM.ITGAX.EMILIN2.ITGB2.FURIN.TIMP1.PLOD1.CD44.FN1
Degradation of the extracellular matrix	MAP-1474228	10	155	0.83	0.7	0.0009	W5NWJ9_SHEEP.ADAM15.NID1.MMP12.PRG4.MMP9.FURIN.TIMP1.CD44.FN1
Cell surface interactions at the vascular wall	MAP-202733	8	155	0.74	0.37	0.0332	GP6.CD84.ITGAM.ITGAX.ITGB2.CD44.FN1.CD58

Supplementary Table 27: Enrichment map analysis of M1-specific differentially abundant proteins (DAPs; n = 20), highlighting significantly enriched Gene Ontology Biological Process (GO-BP) terms and canonical pathways. Functional enrichment analysis was performed using the StringApp (STRING database) within Cytoscape (version 3.10.3) [1], focusing on Gene Ontology (GO) Biological Process (BP) terms and canonical pathways from the KEGG and Reactome databases [2–6]. Enriched GO terms were clustered as functionally coherent groups using the EnrichmentMap plugin [7]. Statistical significance was assessed using a false discovery rate (FDR) threshold of < 0.05. M1 refers to pro-inflammatory macrophages stimulated with GM-CSF, LPS, and IFN-γ.

EnrichmentMap analysis of M1 markers_ Biological Process

Annotation	Nodes	name	EnrichmentMap GS_DESCR	EnrichmentMap Genes	nentMap	EnrichmentMap -log10 (p	EnrichmentMap: pvalue (	EnrichmentMap::	EnrichmentMap::NES (Data S	EnrichmentMap::-log10 (pval	EnrichmentMap::-log10 (pval	EnrichmentMap colouring (Data Set 1)	_mdcluster
		GO:0009617	Response to bacterium	CLEC4ELOC101114535CX	6	5.314258261	4.85E-06	5.00E-03	0.679327772	5.314258261	5.314258261	0.99999515	1
		GO:0098542	Defense response to other organism	MEFVCLEC4ELOC101114	7	5.48945499	3.24E-06	3.70E-03	0.717934189	5.48945499	5.48945499	0.99999676	1
		GO:0001817	Regulation of cytokine production	CLEC4ELOC101114535TL	5	3.853871964	1.40E-04	4.52E-02	0.397040374	3.853871964	3.853871964	0.99986	1
		GO:0071222	Cellular response to lipopolysaccharide	LOC101114535CXCL6IL1B	4	5.317854924	4.81E-06	5.00E-03	0.679327772	5.317854924	5.317854924	0.99999519	1
		GO:0071219	Cellular response to molecule of bacterial origin	LOC101114535CXCL6TLR	5	6.920818754	1.20E-07	1.20E-03	0.862306576	6.920818754	6.920818754	0.99999988	1
		GO:0050729	Positive regulation of inflammatory response	LOC101114535TLR2IL1B	3	3.920818754	1.20E-04	4.26E-02	0.404636239	3.920818754	3.920818754	0.99988	1
**Defense responses**	13	GO:0051707	Response to other organism	MEFVCLEC4ELOC101114	8	5.725842151	1.88E-06	3.10E-03	0.740619494	5.725842151	5.725842151	0.99999812	1
		GO:0006952	Defense response	MEFVCLEC4EPOLBLOC10	9	6.970616222	1.07E-07	1.20E-03	0.862306576	6.970616222	6.970616222	0.999999893	1
		GO:0045087	Innate immune response	MEFVCLEC4ELOC101114	5	4.000434512	9.99E-05	4.05E-02	0.411117838	4.000434512	4.000434512	0.9999001	1
		GO:0006954	Inflammatory response	POLBLOC101114535LOC1	6	6.392544977	4.05E-07	1.20E-03	0.862306576	6.392544977	6.392544977	0.999999595	1
		GO:0006955	Immune response	MEFVCLEC4ELOC101114	8	5.787812396	1.63E-06	3.10E-03	0.740619494	5.787812396	5.787812396	0.99999837	1
		GO:0002682	Regulation of immune system process	CLEC4ELOC101114535TL	7	4.241845378	5.73E-05	2.60E-02	0.467943758	4.241845378	4.241845378	0.9999427	1
		GO:0002376	Immune system process	MEFVCLEC4EPOLBLOC10	9	4.950781977	1.12E-05	8.50E-03	0.611292869	4.950781977	4.950781977	0.9999888	1
		GO:0008285	Negative regulation of cell population proliferation	IL1BARG2CD274IL4I1XDH	5	4.085128182	8.22E-05	3.46E-02	0.43130519	4.085128182	4.085128182	0.9999178	2
		GO:0042129	Regulation of T cell proliferation	IL1BARG2CD274IL4I1	4	5.044793462	9.02E-06	7.30E-03	0.630806258	5.044793462	5.044793462	0.99999098	2
**Negative regulation of cell proliferation**	6	GO:0032642	Regulation of chemokine production	TLR2IL1BARG2	3	4.4225082	3.78E-05	2.05E-02	0.498417009	4.4225082	4.4225082	0.9999622	2
		GO:0009063	Cellular amino acid catabolic process	ARG2IL4I1HAL	3	3.958607315	1.10E-04	4.26E-02	0.404636239	3.958607315	3.958607315	0.99989	2
		GO:1903037	Regulation of leukocyte cell-cell adhesion	IL1BARG2CD274IL4I1	4	3.920818754	1.20E-04	4.26E-02	0.404636239	3.920818754	3.920818754	0.99988	2
		GO:0042130	Negative regulation of T cell proliferation	ARG2CD274IL4I1	3	4.614393726	2.43E-05	1.53E-02	0.535929357	4.614393726	4.614393726	0.9999757	2

Pathway analysis of M1 markers

KEGG pathways

term description	#term ID	observed gene count	background gene count	strength	signal	false discovery rate	g proteins in your network
Rheumatoid arthritis	oas05323	4	87	1.7	1.37	0.00041	C101114535,MMP3,TLR2,IL1B
Toll-like receptor signaling pathway	oas04620	3	81	1.61	0.85	0.0096	LOC101114535,TLR2,IL1B
Chagas disease	oas05142	3	87	1.58	0.84	0.0096	LOC101114535,TLR2,IL1B
Amoebiasis	oas05146	3	89	1.57	0.84	0.0096	TLR2,IL1B,ARG2
Tuberculosis	oas05152	3	143	1.36	0.68	0.0197	CLEC4E,TLR2,IL1B
Malaria	oas05144	2	42	1.72	0.62	0.0399	TLR2,IL1B

Supplementary Table 28: Enrichment map analysis of M2-specific differentially abundant proteins (DAPs; n = 57), highlighting significantly enriched Gene Ontology Biological Process (GO-BP) terms and canonical pathways. Functional enrichment analysis was performed using the StringApp (STRING database) within Cytoscape (version 3.10.3) [1], focusing on Gene Ontology (GO) Biological Process (BP) terms and canonical pathways from the KEGG and Reactome databases [2–6]. Enriched GO terms were clustered as functionally coherent groups using the EnrichmentMap plugin [7]. Statistical significance was evaluated using a false discovery rate (FDR) threshold of < 0.05. M2 denotes anti-inflammatory macrophages stimulated with M-CSF and IL-4.

EnrichmentMap analysis of M2 markers_ Biological Process

Annotation	Nodes	EnrichmentMap name	EnrichmentMap GS_DESCR	Enrichmentl	nentMap	EnrichmentMap -log10 (pv	EnrichmentMap: pvalue (I	EnrichmentMap::fdr_qvalue	EnrichmentMap::NES (Data Set 1)	EnrichmentMap::-log10 (p	EnrichmentMap::-log10 (pval	EnrichmentMap colouring (Da	_mdcluster
Vascular endothelial growth factor signaling pathway	4	GO:0001755	Neural crest cell migration	NRP2SEMA7	4	4.821023053	1.51E-05	3.42E-02	0.196015639	4.821023053	4.821023053	0.9999849	2
		GO:0038084	Vascular endothelial growth fa	NRP2NRP1P	3	4.612610174	2.44E-05	3.42E-02	0.196015639	4.612610174	4.612610174	0.9999756	2
		GO:0007229	Integrin-mediated signaling pa	ADAM15SEN	6	6.913640169	1.22E-07	1.40E-03	0.381591744	6.913640169	6.913640169	0.999999878	2
		GO:0048762	Mesenchymal cell differentiat	NRP2ADAM1	5	4.335358024	4.62E-05	4.03E-02	0.186484918	4.335358024	4.335358024	0.9999538	2
Positive regulation neurogenesis	4	GO:0050772	Positive regulation of axonoge	SEMA7ALIM	4	4.730487056	1.86E-05	3.42E-02	0.196015639	4.730487056	4.730487056	0.9999814	1
		GO:0050769	Positive regulation of neuroge	MAN2A1SEN	5	4.106793247	7.82E-05	4.93E-02	0.174779678	4.106793247	4.106793247	0.9999218	1
		GO:0045773	Positive regulation of axon ext	SEMA7ALIM	3	4.612610174	2.44E-05	3.42E-02	0.196015639	4.612610174	4.612610174	0.9999756	1
		GO:0010720	Positive regulation of cell deve	MAN2A1SEN	6	4.517126416	3.04E-05	3.42E-02	0.196015639	4.517126416	4.517126416	0.9999696	1
Complement activation pathway	3	GO:0006957	Complement activation, altern	CFPW5NPK5	3	4.747146969	1.79E-05	3.42E-02	0.196015639	4.747146969	4.747146969	0.9999821	3
		GO:0030449	Regulation of complement act	LOC1011200	3	4.179798541	6.61E-05	4.41E-02	0.181252365	4.179798541	4.179798541	0.9999339	3
		GO:0002888	Positive regulation of myeloid	ITGAMW5NF	3	4.325138859	4.73E-05	4.03E-02	0.186484918	4.325138859	4.325138859	0.9999527	3
Cell substrate adhesion	2	GO:0010810	Regulation of cell-substrate ad	ADAM15NRF	7	6.401209493	3.97E-07	2.30E-03	0.352763855	6.401209493	6.401209493	0.999999603	4
		GO:0001952	Regulation of cell-matrix adhe	ADAM15NRF	5	5.413412695	3.86E-06	1.10E-02	0.261885744	5.413412695	5.413412695	0.99999614	4
Tube development tissue	2	GO:0035295	Tube development	NRP2MAN2A	11	5.565431096	2.72E-06	1.03E-02	0.2657039	5.565431096	5.565431096	0.99999728	5
		GO:0009888	Tissue development	ADAM15SEN	14	4.742321425	1.81E-05	3.42E-02	0.196015639	4.742321425	4.742321425	0.9999819	5

Pathway analysis of M2 markers

KEGG pathways

term description	#term ID	observed gene count	background gene count	strength	signal	false discovery rate	matching proteins in your	ing proteins in your network (	Column1
Hematopoietic cell lineage	oas04640	4	85	1.24	0.46	0.0334	9940.ENSOARP000000026	D1B2_SHEEP,CD1E,ITGAM,CSF1	
Amoebiasis	oas05146	4	89	1.22	0.46	0.0334	9940.ENSOARP000000026	D1B2_SHEEP,CD1E,ITGAM,FN1	

Reactome pathways

term description	#term ID	observed gene count	background gene count	strength	signal	false discovery rate	matching proteins in your	ing proteins in your network (	Column1
Alternative complement activation	MAP-173736	3	11	2	0.69	0.009	9940.ENSOARP000000000	NPK5_SHEEP,W5NRI1_SHEEP,CFP	
Activation of C3 and C5	MAP-174577	3	15	1.87	0.69	0.009	9940.ENSOARP000000000	NPK5_SHEEP,W5NRI1_SHEEP,CFP	
Regulation of Insulin-like Growth Factor (IGF) transp	MAP-381426	6	164	1.13	0.6	0.009	9940.ENSOARP000000000	EP,W5NRI1_SHEEP,GOLM1,MFGE8,FN1,CSF1	
Post-translational protein phosphorylation	MAP-8957275	6	157	1.15	0.61	0.009	9940.ENSOARP000000000	EP,W5NRI1_SHEEP,GOLM1,MFGE8,FN1,CSF1	
Extracellular matrix organization	MAP-1474244	7	358	0.86	0.46	0.0195	9940.ENSOARP000000037	MP12,ITGAM,ITGAX,MMP19,PLOD1,FN1	
Neurophilin interactions with VEGF and VEGFR	MAP-194306	2	4	2.26	0.52	0.0307	9940.ENSOARP000000187	NRP1,NRP2	
Regulation of Complement cascade	MAP-977606	4	99	1.17	0.43	0.0432	9940.ENSOARP000000000	HEEP,W5NRI1_SHEEP,LOC101120001,CFP	
